# The association of tobacco use on gastrointestinal cancers: A secondary dataset analysis of the Global Burden of Disease Study 2021 and Mendelian randomization

**DOI:** 10.18332/tid/215178

**Published:** 2026-01-22

**Authors:** Yuan Liu, Changming Liu, Xiaowu Li, Juan He, Quan Zhou, Yi Chen, Jinfeng Tang

**Affiliations:** 1 The Affiliated Changsha Hospital of Xiangya School of Medicine, Central South University, Department of Hepatology, Changsha, China

**Keywords:** gastrointestinal cancers, disease burden, mortality rate, disability-adjusted life years, Mendelian randomization

## Abstract

**INTRODUCTION:**

Gastrointestinal cancers remain a major global health issue, with tobacco use as a key factor. Understanding the impact of tobacco use on these cancers and its regional trends is essential for effective prevention strategies.

**METHODS:**

Using data from the Global Burden of Disease (GBD) study, we analyzed mortality and disability-adjusted life years (DALYs) related to tobacco from 1990 to 2021. Joinpoint regression estimated average annual percent change (AAPC), and ARIMA predicted disease burden up to 2036. Two-sample Mendelian randomization (MR) analysis with GWAS data, applied methods such as inverse variance weighting (IVW) and MR-Egger for causal inference.

**RESULTS:**

Esophageal cancer had the highest burden in 2021, with a mortality rate of 2.54 deaths per 100000 population and a DALY rate of 58.49 DALYs per 100000 population. Stomach cancer showed the most significant decrease, with mortality dropping from 2.81 to 1.25 deaths per 100000 population (AAPC= -2.58; 95% uncertainty interval, UI: -2.61– -2.55) and DALY rates decreasing from 71.71 to 29.01 DALYs per 100000 population (AAPC= -2.87; 95% UI: -2.90 – -2.84). The disease burden was higher in older males. ARIMA analysis showed a general decline in disease burden, though some regions had an increasing trend. MR analysis did not provide genetic evidence supporting an association between tobacco use and these cancers.

**CONCLUSIONS:**

From 1990 to 2021, the global burden of gastrointestinal cancers linked to tobacco use showed a declining trend. However, mortality and DALY rates remain high, with significant regional, age, and gender differences, highlighting the need for continued tobacco control efforts.

## INTRODUCTION

Gastrointestinal cancers, encompassing esophageal, stomach, liver, pancreatic, and colorectal cancers, represent a significant global health challenge. Annually, these cancers account for approximately 4.8 million new cases (23.9% of all cancer cases) and 3.2 million cancer-related deaths (33.2% of all cancer deaths) worldwide^1^. Given the combined effects of population growth and aging, these figures are projected to rise further, imposing even greater pressure on already overstretched healthcare systems and resulting in substantial economic burdens^2^. While each of these cancers exhibits distinct characteristics, they also share common risk factors, including poor diet, obesity, and chronic inflammation^3^. Tobacco use has been definitively established as a major risk factor for several gastrointestinal cancers^4-8^. However, the epidemiological patterns and trends of tobacco-related gastrointestinal cancers remain poorly understood at the national, regional, and global levels. Therefore, gaining a comprehensive understanding of the impact and trends of tobacco use on the burden of gastrointestinal cancers is essential for evaluating the effectiveness of past preventive measures, shaping future public health policies, and optimizing the allocation of healthcare resources.

The Global Burden of Disease (GBD) 2021 framework for disease, injury, and risk factors provides a standardized approach to assessing the burden of cancer across various locations and time periods, with a focus on cancer incidence, mortality, and disability-adjusted life years (DALYs). The GBD study categorizes age into 5-year intervals and evaluates the long-term impact of specific diseases in countries and regions worldwide. The GBD 2021 framework also highlights the proportion of cancer burden attributable to modifiable risk factors, as well as the temporal trends in these proportions^9^. In this study, we utilized data from the GBD 2021 report to analyze the spatial and temporal progression of five common gastrointestinal cancers linked to tobacco use. Additionally, Mendelian randomization (MR) provides a method for assessing causal relationships between various risk factors and diseases through genome-wide association studies (GWAS). Unlike observational studies, MR leverages the random distribution of genetic variations to mitigate the influence of confounding factors^10^.

## METHODS

### Study design

This study employed a two-part design combining a secondary analysis of the GBD 2021 dataset and a two-sample MR approach. The GBD 2021 study provides comprehensive, comparable estimates of disease burden across 204 countries and territories, covering the period from 1990 to 2021. In this study, we extracted data on gastrointestinal cancers and tobacco exposure from the 2021 GBD release to assess global, regional, and temporal patterns. For the MR analysis, we used publicly available GWAS summary statistics from large-scale consortia. Genetic variants associated with tobacco use traits were used as instrumental variables to explore the potential causal relationship between tobacco exposure and gastrointestinal cancer outcomes.

### Eligibility criteria

In the GBD study, the population attributable fraction (PAF) incorporates current and past smoking prevalence, the continuous exposure distribution, relative risks, and the theoretical minimum-risk exposure level. It combines global dose–response risk curves with country–age–sex–specific continuous exposure distributions to capture cross-country differences in risk attributable to smoking. The tobacco use-related deaths and DALYs from cancer were calculated for each country, age, sex, year, by multiplying the PAFs by the total number of deaths or DALYs estimated in GBD 2021 for each country, age, sex, year, and type of cancer. PAFs were also used to calculate years of life lost (YLL), and years lived with disability (YLDs)^11^.

This study employed a two-sample MR approach to investigate the causal relationship between tobacco use and common gastrointestinal cancers. The genetic instruments used for MR analysis must meet the following three criteria: 1) single nucleotide polymorphisms (SNPs) should be associated with the tobacco use under study; 2) SNPs should not be correlated with confounding factors; and 3) SNPs must affect the outcome through an exposure that is not directly linked to it^12^ (Supplementary file Figure 1). Therefore, we selected SNPs significantly associated with tobacco use (p<10-6) from large-scale GWAS studies and ensured that these SNPs were not in linkage disequilibrium (r^2^<0.001), excluding alleles containing palindromic SNPs to align exposure and outcome. The F-statistics for all selected SNPs were >10, indicating sufficient strength of the instrumental variables^13^. R^2^ values were calculated using the formula:

R^2^ = [2 × EAF × (1 - EAF) × β^2^] / [2 × EAF × (1 - EAF) × β^2^ + SE^2^ × N]

The F-statistic was calculated from:

F = R^2^ × (N - 2) / (1 - R^2^)

where β and SE refer to the SNP–exposure association, EAF denotes effect allele frequency, and N is the GWAS sample size for the exposure. An F-statistic >10 indicates sufficient instrument strength.

### Data sources and measures

*GBD data sources*

The data source selected for tobacco use and gastrointestinal cancers is the GBD database, managed by the Institute for Health Metrics and Evaluation (IHME) at the University of Washington^14^. This collaborative initiative involves contributions from over 9000 researchers worldwide and encompasses data on 371 diseases and injuries across 204 countries from 1990 to 2021, stratified by age and gender^15^. The sociodemographic index (SDI), which ranges from 0 to 1, is used to measure socioeconomic development, with higher SDI values indicating better development. Based on the SDI, regions are categorized into five groups: low (<0.46), low-middle (0.46–0.60), middle (0.61–0.69), high-middle (0.70–0.81), and high (>0.81). Since the data utilized in this study are derived from this publicly accessible database, no specific ethical approval was required.

In the GBD 2021 study, tobacco use encompasses various modes, including current or past use of any smoked tobacco products, current use of any chewed tobacco products, and average daily exposure to airborne particulate matter from secondhand smoke^14^. For our analysis, we selected data sets on five common gastrointestinal cancers from the GBD database: esophageal cancer, stomach cancer, liver cancer, pancreatic cancer, and colorectal cancer^16^. The definitions of the diseases are described in detail in Supplementary file Table 1.

*GWAS data sources*

Genetic data on tobacco use were sourced from the UK Biobank, which has collected data from 500000 participants and is widely regarded as the most comprehensive and extensively utilized database of its kind^17^. Data on gastrointestinal cancers were obtained from the 12th release (R12) of the FinnGen consortium. The latest data from FinnGen includes 282064 women and 218284 men. The FinnGen study is a large-scale genomic initiative that has analyzed over 500000 Finnish biobank samples, linking genetic variations with health data to explore disease mechanisms and predispositions. This project is a collaboration between Finnish research organizations, biobanks, and international industry partners^18^.

### GBD data statistical analysis

In this study, we obtained data on mortality rates, DALYs, and age-standardized rates (ASR) for esophageal cancer, gastric cancer, liver cancer, pancreatic cancer, and colorectal cancer, categorized by gender (male and female) and age (19 age groups, ranging from <5 years to ≥95 years in 5-year intervals) for the global population, various income regions, and different countries from the GBD 2021 study. The ASR per 100000 population was calculated based on GBD 2021 population estimates, enabling consistent comparisons across different populations and time periods. All estimates were presented with 95% uncertainty intervals (95% UIs), determined by the 2.5th and 97.5th percentiles of 1000 draws from the uncertainty distribution. Through descriptive analysis, we gained insights into the temporal trends in the burden of tobacco-induced gastrointestinal cancers. Additionally, we analyzed summary statistics for various age groups and genders.

We employed Joinpoint software to calculate the annual percentage change (APC), average annual percentage change (AAPC), and the corresponding 95% UI for five common gastrointestinal cancers globally, across different regions, and within individual countries from 1990 to 2021. The best-fitting model was selected for comparison, and trends in disease burden were assessed. If the 95% UI of the AAPC estimate was >0, it indicated an increasing trend; if <0, a decreasing trend; and 0, no trend^19^.

To gain a deeper understanding of the future burden of tobacco-related gastrointestinal cancers, we employed an autoregressive integrated moving average (ARIMA) model to predict trends in age-standardized mortality rates (ASMR) and age-standardized disability-adjusted life years rates (ASDR) globally and for individual countries from 2022 to 2036. For forecasting purposes, ARIMA models are commonly used for non-stationary data, and the original time series are preprocessed prior to modeling. Given that the variance changes with the level of the series or exhibits multiplicative growth, a log (or Box–Cox) transformation was applied to stabilize the variance. The series was then stationarized following the Box–Jenkins approach, with (non-seasonal/seasonal) differencing used to determine the differencing order (d) [/(D)] until the series became approximately stationary^20^.

Three steps were undertaken to fit the optimal ARIMA model: 1) stationarity testing, 2) model identification and order determination, and 3) model diagnostics. All possible parameter values were obtained using the autocorrelation function (ACF) and partial autocorrelation function (PACF). Finally, all datasets were fitted and validated through simultaneous automatic model fitting, and the best-performing model was selected based on the Akaike information criterion (AIC) and the coefficient of determination (R^2^)^21^.

Statistical analysis and data visualization for this study were conducted using R statistical software (version 4.3.1, https://www.R-project.org/) and Joinpoint software (version 5.1.0, https://github.com/DanChaltiel/nih.joinpoint). Statistical significance was determined using a p<0.05.

### MR analysis

The inverse variance-weighted (IVW) method was used as the primary analysis, supplemented by alternative methods including the weighted median method, MR-Egger method, weighted mode method, and simple mode method^22^. Sensitivity analyses were performed using the MR-Egger and weighted median methods^23^. The Cochran Q statistic from the IVW method was used to assess heterogeneity in individual causal effects, with p<0.05 indicating significant heterogeneity^24^. When there is heterogeneity, we use the IVW random effects model; otherwise, we use the fixed effects model. Furthermore, we validated the robustness of the results through a leave-one-out analysis. The scatter plots are useful for visualizing the strength and direction of the relationships and for detecting potential outliers or inconsistencies in the data. This Mendelian randomization analysis was conducted and reported in accordance with the STROBE-MR guidelines.

## RESULTS

### Overview of the global burden and different SDI regions

Table 1 provides detailed information on mortality rates, DALYs, ASMR, and ASDR for common gastrointestinal cancers caused by tobacco use from 1990 to 2021, both globally and across different SDI regions (Supplementary file Figure 2). According to the 2021 global disease burden analysis, esophageal cancer has the highest disease burden, with a mortality rate of 2.54 deaths per 100000 people (95% UI: 1.99–3.14) and a DALY rate of 58.49 DALYs per 100000 people (95% UI: 46.03–72.28). Following esophageal cancer are stomach cancer, pancreatic cancer, liver cancer [mortality rate: 0.61 deaths per 100000 people (95% UI: 0.21–1.01), DALY rate: 16.90 DALYs per 100000 people (95% UI: 5.76–28.26)], and colorectal cancer [mortality rate: 0.55 deaths per 100000 people (95% UI: 0.34–0.77), DALY rate: 14.12 DALYs per 100000 people (95% UI: 8.84–19.50)].

**Table 1:** Global burden estimates of tobacco-attributable gastrointestinal cancers by cancer type: age-standardized mortality rate (ASMR) age-standardized DALY rate (ASDR) and number of deaths, GBD 1990–2021

*Disease*	*Location*	*1990*		*2021*		*1990–2021 APC*	*1990*		*2021*		*1990–2021 APC*
		*Deaths* *(95% UI)*	*Death ASR* *per 100000* *(95%UI)*	*Deaths* *(95% UI)*	*Death ASR* *per 100000* *(95% UI)*	*Net drift (%/* *year)* *(95% UI)*	*DALYs* *(95% UI)*	*DALYs ASR* *per 100000* *(95% UI)*	*DALYs* *(95% UI)*	*DALYs ASR* *per 100000* *(95% UI)*	*Net drift (%/* *year)* *(95% UI)*
**Esophageal cancer**	Global	143333 (117012–170295)	3.63 (2.97–4.31)	219185 (172166–270731)	2.54 (1.99–3.14)	-1.17 (-1.20 – -1.14)	3844096 (3139094–4585376)	93.31 (76.19–111.32)	5136277 (4040644–6350151)	58.49 (46.03–72.28)	-1.19 (-1.39 – -0.98)
	Low SDI	2774 (2253–3320)	1.27 (1.03–1.52)	4524 (3601–5574)	0.93 (0.74–1.15)	-0.73 (-0.80 – -0.66)	79011 (64043–94591)	32.36 (26.24–38.73)	126444 (100440–155507)	23.19 (18.46–28.49)	-0.73 (-0.97 – -0.48)
	Low-middle SDI	9479 (7884–11286)	1.60 (1.33–1.90)	16581 (13542–19829)	1.18 (0.96–1.42)	-1.31 (-1.34 – -1.28)	267895 (224058–319293)	40.86 (34.04–48.70)	443711 (362461–531665)	29.36 (24.01–35.11)	-1.31 (-1.45 – -1.17)
	Middle SDI	56459 (44978–69933)	5.54 (4.39–6.83)	86733 (65048–112366)	3.3 (2.47–4.28)	-0.92 (-0.96 – -0.88)	1556511 (1242518–1932994)	140.70 (112.53–174.49)	2037140 (1540952–2630988)	73.43 (55.44–94.89)	-0.92 (-1.03 – -0.81)
	High-middle SDI	46618 (37144–56996)	4.66 (3.72–5.70)	75223 (56618–98194)	3.74 (2.81–4.88)	-0.97 (-1.01 – -0.94)	1262909 (1002475–1542666)	122.56 (97.48–149.80)	1763634 (1324867–2308908)	87.61 (65.79–114.5)	-0.97 (-1.09 – -0.85)
	High SDI	27942 (22891–32511)	2.53 (2.07–2.94)	36045 (28280–43801)	1.68 (1.33–2.04)	-1.70 (-1.75 – -1.66)	676154 (558731–787083)	62.73 (51.89–72.99)	763422 (608707–920290)	38.5 (30.88–46.29)	-1.70 (-1.91 – -1.49)
**Stomach cancer**	Global	109818 (89441–131513)	2.81 (2.29–3.36)	107926 (84603–138448)	1.25 (0.98–1.61)	-2.58 (-2.61 – -2.55)	2929437 (2380225–3509272)	71.17 (58.04–85.11)	2537998 (1991161–3270229)	29.01 (22.75–37.32)	-2.87 (-2.90 – -2.84)
	Low SDI	1287 (916–1623)	0.59 (0.42–0.74)	1710 (1127–2129)	0.36 (0.23–0.44)	-2.27 (-2.32 – -2.22)	629078 (530058–735218)	57.90 (48.80–67.70)	317863 (260975–381945)	15.84 (12.99–18.91)	-2.60 (-2.66 – -2.55)
	Low-middle SDI	5875 (4621–7463)	0.99 (0.78–1.26)	7784 (5978–9586)	0.56 (0.43–0.69)	-3.79 (-3.82 – -3.76)	166305 (130651–210207)	25.32 (19.87–32.04)	205166 (157311–253378)	13.64 (10.45–16.82)	-4.11 (-4.15 – -4.09)
	Middle SDI	35854 (27881–45182)	3.50 (2.72–4.45)	43073 (32395–57603)	1.63 (1.23–2.18)	-1.84 (-1.88 – -1.81)	1084808 (874067–1299890)	105.19 (84.78–126.1)	925650 (712993–1220612)	46.47 (35.85–61.16)	-1.96 (-2.00 – -1.93)
	High-middle SDI	39723 (32105–47502)	3.98 (3.22–4.75)	39047 (30050–51057)	1.95 (1.50–2.55)	-1.58 (-1.63 – -1.53)	1010299 (774014–1270169)	90.29 (69.96–113.53)	1041308 (777836–1396036)	37.48 (28.13–50.23)	-1.75 (-1.80 – -1.71)
	High SDI	26991 (22570–31941)	2.42 (2.02–2.86)	16255 (13205–19907)	0.73 (0.60–0.89)	-2.44 (-2.49 – -2.39)	36731 (26058–46343)	14.99 (10.67–18.91)	46646 (31155–58286)	8.65 (5.72–10.79)	-2.81 (-2.85 – -2.77)
**Liver cancer**	Global	31744 (11068–51200)	0.77 (0.27–1.24)	53054 (18268–88111)	0.61 (0.21–1.01)	-0.75 (-0.81 – -0.70)	991836 (348072–1595195)	23.19 (8.13–37.31)	1482896 (505000–2478906)	16.90 (5.76–28.26)	-1.00 (-1.05 – -0.96)
	Low SDI	875 (282–1566)	0.38 (0.12–0.68)	1387 (421–2586)	0.27 (0.08–0.51)	-0.64 (-0.69 – -0.59)	26273 (8452–47293)	10.35 (3.33–18.56)	40997 (12409–76755)	7.18 (2.18–13.42)	-0.85 (-0.90 – -0.81)
	Low-middle SDI	2476 (853–4210)	0.39 (0.14–0.67)	5559 (1863–9309)	0.38 (0.13–0.63)	-0.99 (-1.06 – -0.92)	74635 (25535–127821)	10.92 (3.75–18.61)	158781 (52990266505)	10.22 (3.42–17.13)	-1.47 (-1.54 – -1.41)
	Middle SDI	11299 (401018336)	1.01 (0.361.63)	20495 (6999–35022)	0.74 (0.25–1.25)	-0.14 (-0.17 – -0.11)	372736 (132796–604729)	30.70 (10.92–49.75)	610098 (2099221039304)	21.21 (7.3–36.15)	-0.24 (-0.31 – -0.19)
	High-middle SDI	8817 (3047–14584)	0.86 (0.301.42)	13971 (4700–23203)	0.71 (0.24–1.18)	-1.09 (-1.12 – -1.07)	282739 (97869–467347)	27.13 (9.40–44.87)	404123 (134606–679376)	21.03 (6.99–35.45)	-1.18 (-1.20 – -1.15)
	High SDI	8259 (2975–13552)	0.77 (0.281.26)	11612 (3626–20166)	0.57 (0.18–0.97)	-1.00 (-1.04 – -0.95)	234908 (85232–382989)	22.5 (8.16–36.72)	268085 (86207–458316)	14.27 (4.66–24.26)	-1.14 (-1.19 – -1.10)
**Pancreatic cancer**	Global	38431 (34758–42214)	0.98 (0.89–1.08)	72170 (62853–82937)	0.83 (0.73–0.96)	-0.53 (-0.55 – -0.50)	1030312 (935957–1124872)	24.98 (22.66–27.32)	1789503 (1567221–2042057)	20.38 (17.84–23.25)	-0.66 (-0.69 – -0.63)
	Low SDI	1431 (1146–1724)	0.24 (0.19–0.29)	4085 (3610–4606)	0.29 (0.25–0.32)	-0.06 (-0.11 – -0.03)	40537 (32495–48720)	6.16 (4.94–7.40)	110333 (97373–124389)	7.26 (6.41–8.18)	-0.24 (-0.28 – -0.20)
	Low-middle SDI	302 (224–375)	0.14 (0.10–0.17)	644 (518–798)	0.13 (0.11–0.16)	-0.72 (-0.75 – -0.70)	8486 (6283–10533)	3.49 (2.59–4.32)	17693 (14285–22096)	3.27 (2.63–4.06)	-0.87 (-0.91 – -0.84)
	Middle SDI	6385 (5551–7324)	0.62 (0.54–0.71)	16366 (13663–19625)	0.60 (0.50–0.72)	0.59 (0.5–70.62)	185818 (160779–214639)	16.35 (14.18–18.78)	432976 (358675–520051)	15.27 (12.7–18.32)	0.53 (0.51–0.56)
	High-middle SDI	12599 (11434–13843)	1.25 (1.13–1.37)	24348 (20924–28483)	1.22 (1.05–1.42)	-0.10 (-0.14 – -0.07)	359802 (327229–395588)	34.64 (31.52–38.11)	630580 (538232–737073)	31.89 (27.26–37.23)	-0.20 (-0.22 – -0.18)
	High SDI	17658 (15910–19454)	1.60 (1.44–1.76)	26650 (22763–30844)	1.28 (1.11–1.47)	-0.08 (-0.11 – -0.05)	434178 (395058–473717)	40.38 (36.8–43.96)	595958 (522741–672271)	31.07 (27.49–34.78)	-0.22 (-0.25 – -0.19)
**Colon and rectum cancer**	Global	31090 (19792–42082)	0.80 (0.51–1.09)	47613 (29670–66040)	0.55 (0.34–0.77)	-1.19 (-1.20 – -1.17)	850250 (541714–1141631)	20.64 (13.14–27.74)	1235667 (7736061–708237)	14.12 (8.84–19.50)	-1.21 (-1.23 – -1.19)
	Low SDI	400 (246–575)	0.18 (0.11–0.26)	692 (421–980)	0.15 (0.09–0.21)	-0.57 (-0.61 – -0.54)	268894 (171305–362492)	16.43 (10.44–22.40)	390958 (244023–549549)	13.88 (8.65–19.50)	-0.66 (-0.70 – -0.62)
	Low-middle SDI	1595 (997–2228)	0.27 (0.16–0.37)	3482 (2162–4923)	0.25 (0.15–0.35)	-2.01 (-2.04 – -1.98)	11302 (7054–16190)	4.66 (2.89–6.68)	19096 (11714–26945)	3.54 (2.16–5.00)	-1.99 (-2.02 – -1.96)
	Middle SDI	6316 (4021–8602)	0.62 (0.39–0.84)	14441 (8977–20447)	0.54 (0.33–0.76)	-0.21 (-0.24 – -0.17)	187638 (119051–255845)	26.03 (16.55–35.10)	413021 (259115–568504)	21.02 (13.17–28.94)	-0.32 (-0.35 – -0.29)
	High-middle SDI	9480 (6009–12803)	0.95 (0.60–1.28)	15719 (9899–21835)	0.79 (0.50–1.10)	-0.74 (-0.76 – -0.71)	46066 (28785–64202)	6.95 (4.35–9.70)	95591 (59545–135417)	6.28 (3.91–8.88)	-0.87 (-0.89 – -0.85)
	High SDI	13255 (8278–18169)	1.20 (0.75–1.64)	13220 (8023–18488)	0.64 (0.39–0.89)	-0.43 (-0.46 – -0.41)	335149 (214286–458439)	31.21 (20.00–42.65)	315506 (195787–437063)	16.85 (10.56–23.23)	-0.55 (-0.57 – -0.52)

SDI: sustainable development index. UI: uncertainty interval. APC: annual percentage change. ASR: age-standardized rate.

Notably, the global disease burden of common gastrointestinal cancers is decreasing. The most significant decrease is seen in stomach cancer, with mortality rates dropping from 2.81 deaths per 100000 people (95% UI: 2.29–3.36) to 1.25 deaths per 100000 people (95% UI: 0.98–1.61), reflecting an average annual percentage change (AAPC) of -2.58 (95% UI: -2.61 – -2.55). The DALY rate for gastric cancer decreased from 71.71 DALYs per 100000 people (95% UI: 58.04–85.11) to 29.01 DALYs per 100000 people (95% UI: 22.75–37.32), with an AAPC of -2.87 (95% UI: -2.90 – -2.84). The slowest decline was observed in pancreatic cancer, where the mortality rate dropped from 0.98 deaths per 100000 people (95% UI: 0.89–1.08) to 0.83 deaths per 100000 people (95% UI: 0.73–0.96), corresponding to an AAPC of -0.53 (95% UI: -0.55 – -0.50). The DALY rate decreased from 24.98 DALYs per 100000 people (95% UI: 22.66–27.32) to 20.38 DALYs per 100000 people (95% UI: 17.84–23.25), with an AAPC of -0.66 (95% UI: -0.69 – -0.63).

Moreover, this downward trend is not only observed in the global disease burden but also in analyses of different SDI regions, where the burden of tobacco-related gastrointestinal cancers has generally declined over time. However, it is important to note that, from 1990 to 2021, the number of deaths and DALYs due to tobacco-related gastrointestinal cancers has continued to increase globally and within various SDI regions. In addition, we studied the varying disease burden results due to tobacco use between different countries, and the trends also differ (Supplementary file Table 2).

### Burden and trends of diseases caused by tobacco use across different genders and age groups

Supplementary file Figure 3 illustrates the disease burden of stomach cancer caused by tobacco across different SDI regions from 1990 to 2021. From 1990 to 2021, both the ASMR and ASDR for stomach cancer decreased in both men and women across all regions: global, low SDI, low-middle SDI, middle SDI, middle-high SDI, and high SDI. This downward trend was also observed in colorectal cancer. However, for pancreatic cancer, the disease burden trends in both sexes in low-middle and low SDI regions appeared to stabilize over time. In contrast, the overall trend for esophageal cancer showed a decline in both sexes, although an increasing trend was observed in the middle-high SDI regions between 1998 and 2004. Regarding liver cancer, a notable upward trend was observed, particularly in high SDI regions, where both the ASMR and ASDR for women increased (Supplementary file Figure 3).

Among the five most common gastrointestinal cancers, esophageal cancer accounts for the highest disease burden attributable to tobacco use across various age groups. From 1990 to 2021, the number of esophageal cancer-related deaths and DALYs attributable to tobacco use was consistently lower in women compared to men. The number of deaths increased with age, peaking among men in the 65–69 years age group in 1990 and the 70–74 years age group in 2021. In women, the highest number of deaths was observed in the 70–74 years age group in both 1990 and 2021.

From 1990 to 2019, the observed number of deaths and DALYs among women was consistently lower than that among men. The number of deaths attributable to alcohol-related esophageal cancer increased with age, peaking among men aged 65–69 years. In women, the highest number of deaths occurred in the 70–74 years age group. Similarly, the number of DALYs increased with age, reaching a peak among men aged 60–64 years. For women, age-specific DALYs peaked in the 65–69 years age group. Both mortality rates and DALYs showed an upward trend across age groups, from 20–24 years to 70–74 years, with a decline observed among men and a relatively stable trend among women . The number of deaths and DALYs from colorectal cancer, gastric cancer, liver cancer, and pancreatic cancer in both men and women are primarily concentrated in the 60–64, 65–69, and 70–74 age groups. The peak mortality rate is observed in the 90–94 age group, while the highest DALY rates are predominantly found in the 70–74 age group. Detailed analysis results for other cancers are provided in the Supplementary file Figure 3.

### National burden of disease attributable to tobacco use

In 2021, tobacco use contributed to 140514 deaths from esophageal cancer (95% UI: 103905–183437), 65604 deaths from gastric cancer (95% UI: 48081–90844), 23714 deaths from liver cancer (95% UI: 7911–41777), 23303 deaths from pancreatic cancer (95% UI: 17697–29826), and 17277 deaths from colorectal cancer (95% UI: 10520–25641), with these being the leading causes of tobacco-related cancer mortality. In certain countries, no tobacco-related cancer deaths were reported annually. Among the remaining countries, the highest incidence rates for various cancers are as follows: esophageal cancer (India, United States, Japan, and the United Kingdom), gastric cancer (Japan, India, Russia, and the United States), liver cancer (United States, India, Japan, and Vietnam), pancreatic cancer (United States, Japan, Russia, and Germany), and colorectal cancer (United States, Japan, Russia, and India).

China is projected to have the highest number of DALYs attributable to tobacco use in 2021: esophageal cancer 3238100 (95% UI: 2353463–4262163), gastric cancer 1550245 (95% UI: 1135747–2166216), liver cancer 712311 (95% UI: 239926–1256109), pancreatic cancer 601155 (95% UI: 450656–771764), and colorectal cancer 459250 (95% UI: 276317–684962). In some countries, no DALYs related to tobacco use are reported annually. Among the remaining countries, the highest incidence rates for various cancers are as follows: esophageal cancer (India, United States, Japan, and Brazil), gastric cancer (India, Japan, Russia, and the United States), liver cancer (India, United States, Vietnam, and Japan), pancreatic cancer (United States, Russia, Japan, and Germany), and colorectal cancer (United States, Russia, Japan, and India).

**Figure 1:**
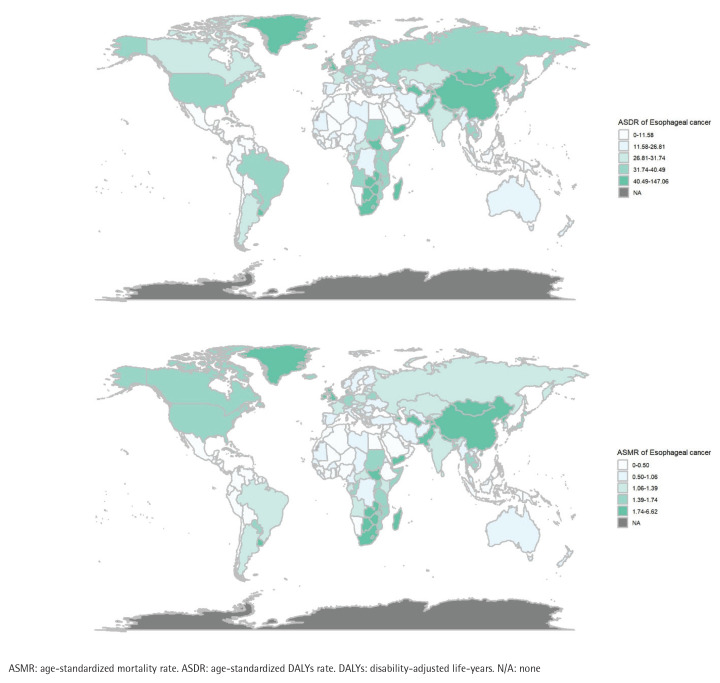
Global distribution of tobacco-attributable esophageal cancer burden in 2021: A) age-standardized mortality rate (ASMR) per 100000 population; B) age-standardized DALY rate (ASDR) per 100000 population

In 2021, the top five countries with the highest ASMR for different gastrointestinal cancers were as follows: esophageal cancer (China, Lesotho, Greenland, Malawi, and Zimbabwe), gastric cancer (Mongolia, China, Kiribati, Yemen, and Kyrgyzstan), liver cancer (Mongolia, Tonga, Gambia, Egypt, and Vietnam), pancreatic cancer (Greenland, Greece, Montenegro, Armenia, and Monaco), and colorectal cancer (Greenland, Croatia, Uruguay, Bulgaria, and Hungary). Additionally, the top five countries with the highest ASDR were: esophageal cancer (China, Lesotho, Greenland, Malawi, and Zimbabwe), gastric cancer (Mongolia, China, Kiribati, North Korea, and Kyrgyzstan), liver cancer (Mongolia, Tonga, Gambia, Vietnam, and Egypt), pancreatic cancer (Greenland, Montenegro, Bulgaria, Greece, and Armenia), and colorectal cancer (Greenland, Bulgaria, Hungary, Croatia, and Uruguay) (Figure 1).

### Prediction of the burden of tobacco-related gastrointestinal tumors by 2036

**Figure 2:**
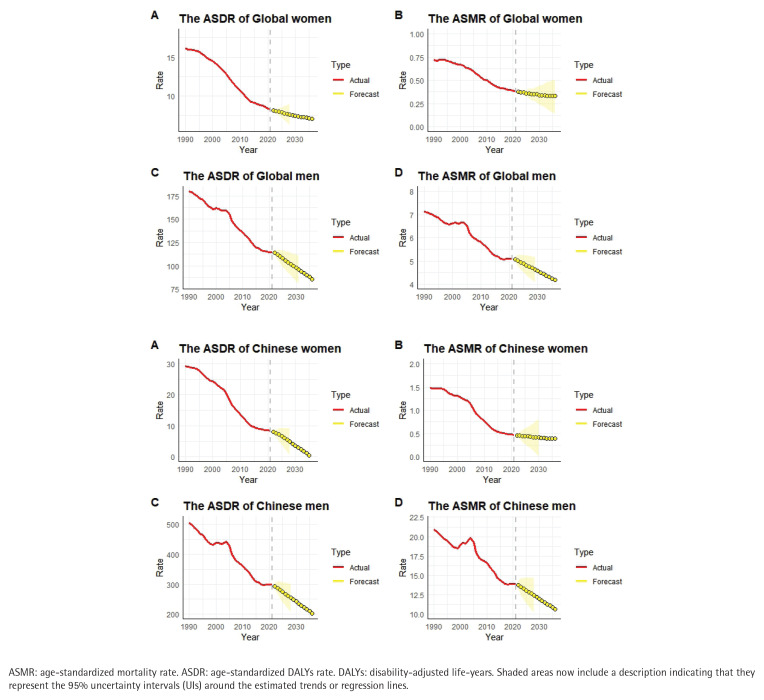
Trends and projections (1990–2036) of tobacco-attributable esophageal cancer burden: age-standardized mortality rate (ASMR) and age-standardized DALY rate (ASDR) per 100000 population globally (A) and in China (B), with gender-specific predictions for 2022–2036

The ARIMA model was employed to forecast the number of DALYs attributable to tobacco use, along with their ASR from 2022 to 2036. The predictions suggest that, globally, the ASMR and ASDR for esophageal cancer, gastric cancer, liver cancer, pancreatic cancer, and colorectal cancer due to tobacco use will decline for both men and women during this period. However, the total number of deaths and DALYs is projected to increase, with the exception of gastric cancer, where both the number of deaths and DALYs are expected to decrease. A similar analysis applied to China-specific data indicates that, from 2022 to 2036, the ASMR and ASDR for tobacco-related esophageal (Figure 2) and gastric cancers in both men and women are projected to decline, while liver cancer, pancreatic cancer, and colorectal cancer will remain relatively stable. In contrast, the number of deaths and DALYs associated with these cancers is expected to rise, although the DALY count for gastric cancer is anticipated to remain relatively stable.

Similarly, we conducted a predictive analysis to assess the future burden of tobacco-related cancers across different cancer types from 2022 to 2036 (Supplementary file Figure 4). The results indicate that the number of tobacco-related cancer deaths and DALYs is projected to increase in most countries; however, notable regional disparities remain across nations (Supplementary file Tables 3–7).

### MR analysis

Supplementary file Table 8 presents the summary statistics for the GWAS datasets used in this study. We screened tobacco-related SNPs and excluded variants that were potentially confounded by alcohol use. Ultimately, six SNPs were retained as instrumental variables for subsequent MR analyses. Using the IVW method as the primary analytical approach, we did not observe genetic evidence supporting a causal effect of tobacco use on esophageal cancer, gastric cancer, liver cancer, pancreatic cancer, or colorectal cancer.

Moreover, the results from the weighted median, MR-Egger, weighted mode, and simple mode methods were broadly consistent with the IVW findings, providing additional support for the stability of the estimates. Cochran’s Q test based on the IVW method indicated no detectable heterogeneity among the instrumental variables, and MR-Egger regression did not suggest the presence of directional horizontal pleiotropy. The leave-one-out analysis showed that the overall estimates were not driven by any single SNP (Supplement file Figure 5). The scatter plot presents SNP-specific estimates obtained from several MR methods evaluating the association between tobacco use and gastrointestinal cancer outcomes (Supplementary file Figure 6).

Overall, our Mendelian randomization analyses do not provide evidence supporting a genetically predicted effect of tobacco use on the risk of common gastrointestinal cancers (Supplementary file Table 9). We did not encounter substantial missing data that required further imputation beyond what was already addressed in the original GWAS sources.

## DISCUSSION

This study utilizes the most recent GBD 2021 data to analyze the disease burden of tobacco-related gastrointestinal cancers from global, regional, national, age, and gender perspectives, providing a comprehensive evaluation of the differences in disease burden across these various dimensions. Moreover, our analysis extends beyond previous GBD studies that have primarily identified tobacco as a risk factor by employing MR to investigate the causal relationship between tobacco use and gastrointestinal cancers. The findings of this study reveal significant spatial variations in the disease burden of tobacco-related gastrointestinal cancers across different countries and regions, aligning with previous research findings^25,26^. Between 1990 and 2021, both the ASMR and ASDR for tobacco-related esophageal cancer, gastric cancer, liver cancer, pancreatic cancer, and colorectal cancer showed a global decline across most SDI regions. Rather than directly indicating improvements in prevention, control, or treatment, these downward trends in age-standardized rates should be interpreted cautiously, as they may also reflect demographic changes, shifts in competing risks, or variations in exposure patterns over time. Notably, despite the decrease in age-standardized rates, the absolute numbers of deaths and DALYs continued to rise in many regions, likely driven by population growth and aging. These demographic dynamics highlight the need to consider both standardized and absolute measures when evaluating the burden of tobacco-related gastrointestinal cancers.

From 1990 to 2019, the increase in the number of deaths and DALYs attributable to tobacco use was primarily driven by global population growth and aging. However, during this period, both the ASMR and ASDR showed a downward trend. Subsequent predictions using the ARIMA model suggest that, through 2036, both the ASMR and ASDR will continue to decline overall. Two potential factors may contribute to this decline. First, it can be attributed to the growing awareness of the harmful effects of smoking, coupled with the continued implementation of smoking-related policies and regulations^27^. These efforts include increasing healthcare professionals’ awareness of the dangers of tobacco use, as well as the enforcement of smoking bans and regulations by countries participating in the World Health Organization (WHO) Framework Convention on Tobacco Control^28^. However, significant disparities remain in the tobacco control efforts across different countries and regions^29^. In some nations, the disease burden remains high due to economic limitations, a lack of effective intervention measures, and unhealthy lifestyles that increase tobacco exposure, leading to limited access to healthcare and public health resources. Additionally, this may be partly due to demographic changes and advancements in medical treatment, which have contributed to improved health outcomes^25^.

Our results indicate that the disease burden of tobacco-related gastrointestinal cancers, measured in terms of the number of deaths, DALYs, ASMR, and ASDR, is higher in men than in women. This disparity is primarily attributed to sociocultural and environmental factors. Men are more susceptible to cultural influences, which typically result in higher rates of smoking and alcohol consumption. Additionally, physiological differences, particularly in hormone levels, play distinct roles in the development of gastrointestinal cancers between men and women^30^. These factors may help explain the observed gender disparity in disease burden. Differences in disease burden across countries and regions are also evident in terms of age. The study found that both ASMR and ASDR are higher among the elderly, a trend consistently observed across different SDI regions. Older individuals generally have had longer exposure to tobacco, while their physiological and cognitive functions decline with age^31^. Consequently, as individuals age, the health impacts of tobacco accumulate, intensifying the disease burden over time. It is also noteworthy that the peak in the number of cases and standardized rates often occurs in different age groups, which can be attributed to the varying proportions of age groups within the total population.

MR analysis did not provide genetic evidence supporting a causal relationship between tobacco use and the incidence of gastrointestinal cancers. This absence of a detected association may reflect the complex and potentially differing impacts of firsthand and secondhand smoke on gastrointestinal cancer risk, which are not fully captured by the aggregate genetic instruments used in our analysis. Population-specific differences may also contribute to these findings. Despite the lack of support from our MR results, numerous experimental and epidemiological studies have explored the biological pathways through which tobacco constituents may influence gastrointestinal health. Evidence indicates that carcinogenic components of tobacco – such as free radicals and mutagenic compounds – can impair the epithelial barrier of the gastrointestinal tract, promote inflammation and cellular injury, reduce mucus secretion, diminish mucosal blood flow, and interfere with prostaglandin synthesis^32^. These effects further compromise the gastric mucosal defense system, thereby increasing the risk of malignancy^33^.

### Limitations

This study has several limitations. First, the GBD 2021 data used in this study are based on extensive modeling, which, despite efforts to adjust for various factors, may still introduce bias, particularly in low- and middle-income regions with limited data. The accuracy of these estimates may not fully reflect actual conditions in such areas, and we recommend enhanced international collaboration to improve disease monitoring and data quality. Second, GBD data lacks detailed personal information, limiting our ability to analyze additional risk factors and covariates comprehensively. Additionally, the MR analysis was based solely on European populations, limiting the generalizability of the findings to other regions. Moreover, several inherent limitations affect both the GBD and MR analyses. For GBD, risk-attribution assumptions, inherent model limitations, and unpredictable changes (e.g. in screening guidelines and treatment efficacy) must be considered. The risk of Type I error is increased due to multiple comparisons. Emerging trends in e-cigarette use may also impact our findings, but such data are not fully captured. For the MR analysis, unobserved pleiotropy, vertical pleiotropy, and residual population stratification are potential sources of bias. While causal inference can be postulated, it cannot be definitively proven. In conclusion, while we aim to mitigate these limitations, our results should be interpreted with caution, particularly regarding generalizability and causal relationships.

## CONCLUSIONS

Although the age-standardized burden of tobacco-related gastrointestinal cancers has declined since 1990, tobacco remains an important associated risk factor, particularly among older men. Our findings highlight substantial increases in the absolute numbers of deaths and DALYs, which are likely influenced by demographic changes such as population growth and aging. While this study cannot assess the direct impact of specific tobacco control policies, the persistent disease burden underscores the continued public health relevance of tobacco exposure. Strengthening surveillance, monitoring trends, and further evaluating the effects of tobacco-related risk reduction strategies remain important for guiding future cancer prevention efforts.

## Supplementary Material



## Data Availability

The original contributions presented in the study are included in the article and the Supplementary file. Further inquiries can be directed to the corresponding author.
