# Supplementary material for: The association of tobacco use on gastrointestinal cancers: A secondary dataset analysis of the Global Burden of Disease Study 2021 and Mendelian randomization
Source: Tob Induc Dis. 2026 Jan 22;24:10.18332/tid/215178. doi: 10.18332/tid/215178 (PMC12825413; doi:10.18332/tid/215178)
Supplement: Supplementary file 1 [file TID-24-07-s1.pdf]

Supplementary Material

Supplementary Tables

Supplement table.1 List of International Classification of Diseases (ICD) codes mapped to gastrointestinal cancers in GBD 2021

**Cause**	**ICD10**
Esophageal cancer	C15.0-C15.9, D00.1, D13.0
Gastic cancer	C16-16.9, D00.2, D13.1, D37.1
Liver cancer	C22–C22.4, C22.7-C22.8
Pancreatic cancer	C25.0-C25.9
Colon and rectum cancer	C18-C21, D01, D12, D37.3-D37.5, Z12.1-Z12.13, Z85.03-Z85.048, Z86.010

Supplement table.2 Disease burden caused by tobacco use in different countries in 1990 and 2021.

	1990		2021		1990–2021 APC	1990		2021		1990–2021 APC
**Location**	**Death number**	**Death ASR, per 100,000**	**Death number**	**Death ASR, per 100,000**	**Net drift (%/year)**	**DALYs number**	**DALYs ASR, per 100,000**	**DALYs number**	**DALYs ASR, per 100,000**	**Net drift (%/year)**
Afghanistan	76 (45,121)	1.13 (0.66,1.79)	98 (61,148)	1.06 (0.67,1.58)	-0.20 (-0.22,-0.18)	2031 (1161,3192)	27.84 (16.22,43.76)	2902 (1772,4523)	25.54 (15.64,38.6)	-0.27 (-0.29,-0.26)
Albania	19 (14,25)	0.97 (0.71,1.25)	34 (23,48)	0.78 (0.53,1.07)	-0.79 (-0.96,-0.66)	483 (357,629)	22.79 (16.85,29.43)	777 (522,1075)	17.88 (12.07,24.69)	-0.88 (-1.02,-0.76)
Algeria	33 (25,42)	0.32 (0.24,0.41)	88 (60,121)	0.29 (0.20,0.39)	-0.35 (-0.38,-0.33)	793 (593,1028)	6.68 (5.03,8.61)	2035 (1380,2812)	5.81 (3.97,8.01)	-0.46 (-0.49,-0.44)
American Sa	0 (0,0)	0.36 (0.25,0.52)	0 (0,0)	0.43 (0.30,0.58)	0.64 (0.51,0.78)	2 (2,3)	9.17 (6.45,12.63)	6 (4,8)	11.05 (7.73,14.91)	0.71 (0.59,0.85)
Andorra	1 (0,1)	0.97 (0.59,1.48)	1 (0,1)	0.47 (0.25,0.75)	-2.43 (-2.63,-2.28)	14 (8,21)	23.73 (14.39,36.41)	17 (9,27)	11.15 (5.94,17.53)	-2.55 (-2.76,-2.38)
Angola	84 (54,121)	2.14 (1.39,3.04)	160 (111,225)	1.37 (0.95,1.91)	-1.40 (-1.47,-1.33)	2512 (1604,3670)	56.76 (36.54,82.10)	4763 (3265,6762)	35.31 (24.56,49.73)	-1.49 (-1.57,-1.40)
Antigua and I	0 (0,1)	0.69 (0.48,0.92)	1 (0,1)	0.55 (0.39,0.73)	-0.92 (-1.14,-0.74)	8 (6,11)	16.33 (11.68,21.63)	14 (10,18)	12.50 (8.88,16.45)	-1.04 (-1.28,-0.87)
Argentina	795 (605,991)	2.44 (1.86,3.04)	664 (488,852)	1.18 (0.87,1.51)	-2.22 (-2.31,-2.12)	20331 (15718,2468	61.90 (47.97,75.39)	15533 (11638,19551	28.37 (21.29,35.53)	-2.41 (-2.50,-2.31)
Armenia	36 (31,41)	1.34 (1.13,1.54)	22 (18,27)	0.49 (0.40,0.59)	-3.30 (-3.68,-2.98)	956 (806,1097)	32.95 (27.79,37.74)	525 (427,630)	11.59 (9.44,13.88)	-3.39 (-3.71,-3.09)
Australia	317 (243,396)	1.59 (1.22,1.99)	395 (278,531)	0.85 (0.60,1.13)	-2.06 (-2.21,-1.95)	7347 (5725,9100)	37.42 (29.15,46.33)	8207 (5932,10692)	19.19 (14.12,24.83)	-2.21 (-2.31,-2.12)
Austria	125 (97,153)	1.09 (0.85,1.33)	174 (137,219)	0.98 (0.77,1.22)	-0.31 (-0.42,-0.22)	3076 (2419,3752)	28.55 (22.41,34.83)	3885 (3086,4787)	23.57 (18.84,29.06)	-0.63 (-0.79,-0.48)
Azerbaijan	147 (107,188)	2.95 (2.15,3.81)	222 (153,299)	2.30 (1.60,3.09)	-0.80 (-0.90,-0.71)	4006 (2920,5123)	75.34 (54.74,96.25)	5715 (3966,7699)	52.94 (36.56,71.48)	-1.15 (-1.25,-1.06)
Bahamas	2 (1,3)	1.44 (0.91,2.13)	5 (3,6)	1.11 (0.74,1.57)	-0.75 (-0.89,-0.57)	60 (39,86)	37.74 (24.49,54.67)	121 (81,167)	27.75 (18.42,38.46)	-0.94 (-1.05,-0.80)
Bahrain	2 (2,3)	1.76 (1.23,2.33)	5 (4,8)	0.91 (0.63,1.26)	-2.08 (-2.19,-1.98)	59 (40,81)	36.41 (25.16,49.10)	140 (91,208)	17.10 (11.73,24.47)	-2.41 (-2.50,-2.33)
Bangladesh	1309 (886,1826)	2.86 (1.93,3.97)	2284 (1522,3241)	1.71 (1.14,2.41)	-1.52 (-1.63,-1.42)	36570 (25042,5177	74.15 (50.17,104.11)	58118 (38558,83419	40.91 (27.24,58.46)	-1.82 (-1.91,-1.75)
Barbados	4 (3,5)	1.29 (0.93,1.72)	5 (3,6)	0.86 (0.56,1.21)	-1.13 (-1.31,-0.94)	81 (59,107)	28.58 (20.94,37.60)	93 (60,130)	18.05 (11.79,25.31)	-1.33 (-1.49,-1.15)
Belarus	186 (150,224)	1.41 (1.14,1.69)	229 (167,292)	1.40 (1.03,1.79)	-0.41 (-0.60,-0.22)	5214 (4203,6314)	39.25 (31.79,47.54)	6259 (4525,8054)	39.19 (28.42,50.18)	-0.46 (-0.66,-0.25)
Belgium	315 (250,377)	2.06 (1.65,2.46)	407 (314,510)	1.74 (1.34,2.16)	-0.54 (-0.69,-0.39)	7303 (5875,8666)	50.22 (40.71,59.51)	8634 (6638,10705)	40.38 (31.17,49.77)	-0.76 (-0.93,-0.59)
Belize	0 (0,1)	0.43 (0.32,0.56)	1 (1,2)	0.43 (0.31,0.56)	0.03 (-0.14,0.18)	10 (8,13)	10.89 (8.34,13.90)	34 (25,44)	10.62 (7.78,13.87)	-0.01 (-0.20,0.16)
Benin	8 (6,10)	0.39 (0.28,0.54)	23 (16,32)	0.47 (0.32,0.65)	0.59 (0.54,0.64)	199 (145,273)	9.87 (7.22,13.54)	624 (428,861)	11.56 (7.96,15.81)	0.51 (0.47,0.55)
Bermuda	1 (1,1)	1.56 (1.05,2.23)	1 (1,2)	0.88 (0.61,1.21)	-1.96 (-2.09,-1.81)	24 (17,34)	38.27 (26.45,52.97)	27 (19,36)	20.95 (14.53,28.14)	-2.08 (-2.22,-1.90)
Bhutan	5 (3,7)	2.05 (1.34,2.89)	8 (6,12)	1.46 (1.00,2.06)	-1.05 (-1.08,-1.02)	131 (86,185)	50.25 (32.91,71.16)	198 (134,285)	32.46 (21.99,46.61)	-1.37 (-1.40,-1.33)
Bolivia (Pluri	14 (9,20)	0.46 (0.31,0.67)	28 (17,41)	0.32 (0.21,0.48)	-1.10 (-1.14,-1.06)	347 (240,504)	10.69 (7.34,15.55)	656 (418,989)	7.11 (4.50,10.67)	-1.30 (-1.34,-1.26)
Bosnia and H	45 (34,58)	1.09 (0.82,1.41)	68 (47,93)	1.09 (0.74,1.47)	0.07 (-0.04,0.20)	1257 (955,1617)	27.86 (21.08,35.66)	1670 (1144,2281)	27.52 (18.84,37.57)	0.07 (-0.08,0.24)
Botswana	20 (13,28)	3.62 (2.46,5.18)	34 (24,48)	2.44 (1.69,3.42)	-1.25 (-1.33,-1.19)	556 (374,798)	92.32 (62,131.30)	935 (648,1310)	59.23 (41.35,82.84)	-1.42 (-1.50,-1.36)
Brazil	2868 (2288,3430)	3.34 (2.65,4.02)	3493 (2667,4419)	1.39 (1.06,1.76)	-2.75 (-2.80,-2.69)	77032 (62248,9159	82.54 (66.38,98.56)	86634 (65959,10900	33.68 (25.68,42.37)	-2.82 (-2.86,-2.77)
Brunei Darus	1 (1,2)	1.64 (1.15,2.19)	3 (2,4)	0.81 (0.57,1.11)	-2.32 (-2.46,-2.21)	38 (27,50)	38.06 (27.18,50.60)	67 (47,93)	18.34 (12.77,25.1)	-2.36 (-2.48,-2.25)
Bulgaria	151 (119,184)	1.20 (0.95,1.46)	134 (100,170)	1.02 (0.77,1.29)	-0.53 (-0.68,-0.34)	4325 (3460,5207)	34.29 (27.66,41.19)	3601 (2745,4529)	29.29 (22.51,36.66)	-0.50 (-0.64,-0.31)
Burkina Faso	16 (11,22)	0.36 (0.26,0.49)	57 (38,81)	0.61 (0.40,0.86)	1.73 (1.68,1.76)	466 (333,633)	9.86 (7.05,13.56)	1685 (1108,2396)	16.51 (10.85,23.54)	1.68 (1.64,1.72)
Burundi	75 (53,101)	3.23 (2.27,4.32)	61 (42,84)	1.22 (0.85,1.67)	-3.10 (-3.12,-3.07)	2209 (1533,2970)	89.58 (62.14,120.35)	1846 (1275,2549)	32.90 (22.87,44.96)	-3.19 (-3.22,-3.16)
Cabo Verde	3 (2,4)	1.26 (0.94,1.64)	9 (6,12)	1.98 (1.39,2.71)	1.52 (1.22,1.82)	71 (53,93)	32.99 (24.73,43.47)	246 (170,334)	52.24 (36.66,71.73)	1.56 (1.32,1.81)
Cambodia	97 (73,124)	2.28 (1.74,2.97)	175 (126,229)	1.52 (1.10,1.97)	-1.31 (-1.33,-1.29)	2641 (1954,3366)	55.97 (41.95,71.71)	4503 (3164,6012)	34.91 (24.95,46.1)	-1.52 (-1.54,-1.50)
Cameroon	21 (14,29)	0.46 (0.32,0.64)	95 (59,142)	0.75 (0.47,1.10)	1.58 (1.55,1.61)	607 (402,851)	12.28 (8.32,17.06)	2821 (1743,4277)	19.84 (12.37,29.72)	1.57 (1.54,1.60)
Canada	671 (538,802)	2.05 (1.64,2.44)	1087 (822,1384)	1.45 (1.10,1.84)	-1.19 (-1.33,-1.07)	15297 (12350,1825	47.45 (38.42,56.60)	22312 (16982,28103	31.74 (24.37,40.07)	-1.36 (-1.50,-1.24)
Central Afric	21 (14,30)	1.76 (1.21,2.46)	25 (17,37)	1.09 (0.74,1.54)	-1.56 (-1.58,-1.53)	644 (426,911)	48.46 (32.77,68.16)	801 (522,1181)	29.65 (19.85,42.71)	-1.59 (-1.62,-1.56)
Chad	10 (7,13)	0.36 (0.25,0.47)	40 (27,60)	0.73 (0.49,1.06)	2.40 (2.36,2.43)	253 (177,340)	8.80 (6.16,11.78)	1112 (715,1688)	18.08 (11.92,26.87)	2.40 (2.36,2.44)
Chile	161 (120,206)	1.63 (1.20,2.10)	115 (86,150)	0.44 (0.33,0.58)	-4.14 (-4.22,-4.06)	4176 (3188,5234)	40.58 (30.85,50.84)	2719 (2075,3450)	10.71 (8.20,13.57)	-4.25 (-4.33,-4.18)
China	85093 (65881,106	10.47 (8.13,13.04)	140514 (103905,1	6.62 (4.88,8.66)	-1.50 (-1.54,-1.45)	2324652 (1803869,	260.83 (202.20,326.75)	3238100 (2353463,4	147.06 (107.28,192.84)	-1.87 (-1.92,-1.82)
Colombia	144 (111,181)	0.86 (0.66,1.11)	113 (80,153)	0.20 (0.14,0.28)	-4.65 (-4.87,-4.47)	3695 (2888,4554)	20.46 (15.83,25.5)	2511 (1794,3373)	4.52 (3.23,6.07)	-4.89 (-5.11,-4.70)
Comoros	8 (6,12)	4.22 (2.95,5.91)	13 (9,19)	2.76 (1.90,4.03)	-1.41 (-1.49,-1.33)	235 (161,339)	109.70 (75.49,156.70)	362 (244,533)	69.16 (47.01,101.75)	-1.53 (-1.62,-1.44)
Congo	21 (14,31)	1.98 (1.32,2.96)	39 (25,56)	1.50 (1.00,2.13)	-0.86 (-0.89,-0.83)	604 (395,903)	51.91 (34.31,77.25)	1116 (729,1672)	37.16 (24.39,53.19)	-1.06 (-1.10,-1.02)
Cook Islands	0 (0,0)	0.86 (0.56,1.23)	0 (0,0)	0.61 (0.42,0.87)	-1.03 (-1.11,-0.94)	3 (2,4)	20.86 (13.89,29.25)	4 (3,5)	15.10 (10.52,21.38)	-0.97 (-1.04,-0.88)
Costa Rica	14 (11,18)	0.85 (0.63,1.08)	19 (14,26)	0.35 (0.25,0.47)	-2.85 (-3.02,-2.65)	314 (237,394)	18.25 (13.76,22.95)	410 (295,536)	7.42 (5.36,9.70)	-3.01 (-3.17,-2.80)
Croatia	131 (105,155)	2.11 (1.69,2.50)	110 (83,137)	1.26 (0.96,1.55)	-0.07 (-0.09,-0.05)	3550 (2864,4206)	54.90 (44.24,65.02)	2548 (1946,3167)	31.54 (24.2,39.33)	-0.11 (-0.14,-0.08)
Cuba	165 (128,203)	1.64 (1.27,2.01)	355 (263,453)	1.79 (1.33,2.28)	-1.71 (-1.85,-1.54)	3866 (3089,4670)	38.07 (30.45,46)	9033 (6752,11412)	46.37 (34.8,58.53)	-1.83 (-1.99,-1.61)
Cyprus	5 (4,7)	0.68 (0.48,0.91)	13 (9,17)	0.61 (0.43,0.82)	0.38 (0.23,0.49)	113 (79,150)	14.34 (10.12,19.07)	280 (194,382)	13.67 (9.51,18.59)	0.72 (0.57,0.83)
Czechia	182 (142,225)	1.32 (1.03,1.63)	261 (190,334)	1.24 (0.91,1.58)	-0.39 (-0.48,-0.32)	4771 (3783,5843)	35.65 (28.31,43.73)	6209 (4591,7924)	31.69 (23.46,40.47)	-0.15 (-0.23,-0.09)
C么te d'Ivoir	7 (5,9)	0.17 (0.12,0.24)	19 (12,27)	0.17 (0.12,0.24)	-0.14 (-0.25,-0.03)	203 (142,276)	4.48 (3.16,6.13)	546 (363,779)	4.34 (2.90,6.20)	-0.37 (-0.52,-0.25)
Democratic P	621 (408,881)	3.76 (2.50,5.28)	1083 (736,1570)	3.19 (2.16,4.62)	-0.53 (-0.54,-0.52)	18218 (11903,2579	100.58 (66.06,142.09)	30361 (20515,44463	86.77 (58.85,126.40)	-0.47 (-0.49,-0.46)
Democratic R	164 (108,228)	1.03 (0.69,1.43)	265 (163,384)	0.71 (0.43,1.02)	-1.22 (-1.26,-1.18)	4864 (3163,6771)	27.52 (18.08,38.3)	7957 (4915,11564)	18.79 (11.58,27.19)	-1.25 (-1.31,-1.19)
Denmark	229 (189,268)	2.83 (2.34,3.32)	267 (204,330)	2.15 (1.66,2.65)	-0.91 (-1.02,-0.81)	5082 (4186,5952)	67.22 (55.2,78.61)	5272 (4103,6454)	46.10 (35.95,56.24)	-1.21 (-1.33,-1.12)
Djibouti	4 (3,6)	3.26 (2.08,4.88)	16 (9,24)	2.60 (1.61,3.89)	-0.73 (-0.77,-0.69)	128 (81,199)	82.66 (51.69,124.75)	451 (269,701)	63.12 (38.96,96.05)	-0.88 (-0.94,-0.83)

Dominica	0 (0,1)	0.80 (0.57,1.08)	1 (0,1)	0.70 (0.48,1.03)	-0.38 (-0.42,-0.35)	11 (8,15)	19.42 (13.99,25.99)	16 (10,23)	17.82 (11.94,26.05)	-0.26 (-0.29,-0.23)
Dominican R	25 (17,34)	0.77 (0.53,1.05)	69 (47,99)	0.71 (0.48,1.02)	-0.22 (-0.43,0.00)	600 (412,818)	16.42 (11.24,22.44)	1601 (1076,2305)	15.94 (10.65,22.88)	-0.01 (-0.13,0.09)
Ecuador	23 (18,30)	0.48 (0.36,0.61)	28 (19,38)	0.18 (0.12,0.24)	-3.24 (-3.47,-3.00)	540 (419,674)	10.38 (8.06,13.05)	583 (406,797)	3.58 (2.49,4.87)	-3.50 (-3.72,-3.28)
Egypt	97 (73,126)	0.41 (0.30,0.53)	225 (165,302)	0.42 (0.31,0.57)	0.17 (0.07,0.27)	2754 (2032,3592)	9.57 (7.13,12.41)	5884 (4278,7939)	9.17 (6.73,12.3)	-0.10 (-0.20,0.02)
El Salvador	6 (4,9)	0.22 (0.15,0.32)	14 (9,20)	0.23 (0.15,0.33)	0.13 (-0.08,0.32)	164 (118,228)	5.45 (3.89,7.58)	343 (229,490)	5.67 (3.79,8.10)	0.20 (-0.04,0.41)
Equatorial Gu	3 (2,5)	1.54 (1.03,2.23)	6 (3,8)	1.12 (0.72,1.62)	-0.98 (-1.05,-0.92)	95 (62,141)	42.81 (28.33,63.50)	165 (98,243)	28.89 (17.71,42.04)	-1.22 (-1.30,-1.16)
Eritrea	27 (16,39)	1.95 (1.20,2.83)	32 (19,50)	1.05 (0.65,1.61)	-2.00 (-2.05,-1.95)	909 (533,1330)	59.04 (35.28,85.90)	1063 (609,1653)	30.16 (17.91,46.36)	-2.17 (-2.23,-2.12)
Estonia	28 (22,34)	1.35 (1.05,1.66)	26 (19,33)	1.04 (0.77,1.33)	-0.74 (-0.99,-0.45)	787 (618,966)	38.27 (30.06,46.80)	636 (473,819)	27.08 (20.35,34.72)	-0.99 (-1.23,-0.70)
Eswatini	6 (4,9)	2.34 (1.60,3.21)	10 (6,14)	1.77 (1.16,2.52)	-0.87 (-0.93,-0.82)	182 (123,255)	59.34 (40.81,82.96)	285 (180,413)	46.12 (29.88,65.79)	-0.80 (-0.86,-0.73)
Ethiopia	119 (81,176)	0.59 (0.40,0.88)	109 (78,156)	0.27 (0.19,0.38)	-2.51 (-2.53,-2.49)	3493 (2370,5210)	15.80 (10.73,23.44)	2932 (2057,4086)	6.50 (4.61,9.18)	-2.82 (-2.84,-2.80)
Fiji	2 (1,3)	0.60 (0.42,0.82)	4 (3,6)	0.50 (0.34,0.69)	-0.76 (-0.92,-0.66)	63 (44,86)	15.75 (11,21.56)	111 (76,158)	12.99 (8.98,18.43)	-0.76 (-0.90,-0.66)
Finland	72 (53,94)	1.02 (0.76,1.33)	89 (64,117)	0.72 (0.53,0.93)	-1.00 (-1.12,-0.88)	1767 (1347,2250)	26.00 (19.98,32.96)	1945 (1438,2518)	17.66 (13.38,22.46)	-1.27 (-1.43,-1.16)
France	2835 (2199,3406)	3.55 (2.76,4.25)	1628 (1222,2066)	1.17 (0.90,1.46)	-3.49 (-3.58,-3.42)	70191 (54849,8374	92.95 (73.38,110.69)	34626 (27025,43072	28.19 (22.16,34.82)	-3.73 (-3.82,-3.67)
Gabon	7 (5,10)	1.24 (0.85,1.73)	13 (9,18)	1.21 (0.83,1.65)	-0.04 (-0.08,0.01)	199 (138,280)	33.16 (23.01,46.80)	376 (251,534)	31.74 (21.57,44.24)	-0.13 (-0.17,-0.08)
Gambia	1 (1,1)	0.26 (0.18,0.36)	2 (2,3)	0.25 (0.17,0.35)	-0.11 (-0.23,-0.00)	26 (18,36)	6.96 (4.79,9.58)	69 (46,96)	6.62 (4.43,9.14)	-0.16 (-0.29,-0.04)
Georgia	57 (44,70)	0.89 (0.69,1.09)	29 (22,36)	0.48 (0.37,0.60)	-2.00 (-2.25,-1.78)	1491 (1153,1833)	22.78 (17.66,27.90)	683 (526,848)	11.80 (9.07,14.63)	-2.12 (-2.37,-1.91)
Germany	1983 (1554,2398)	1.61 (1.26,1.95)	2801 (2125,3483)	1.48 (1.14,1.82)	-0.18 (-0.30,-0.08)	51249 (40369,6193	43.64 (34.37,52.61)	61206 (47334,74917	35.79 (27.89,43.65)	-0.56 (-0.71,-0.42)
Ghana	12 (8,16)	0.21 (0.14,0.27)	44 (30,60)	0.28 (0.20,0.38)	1.04 (1.00,1.07)	331 (227,444)	5.03 (3.49,6.73)	1187 (821,1606)	6.73 (4.62,9.10)	0.95 (0.91,0.98)
Greece	188 (156,221)	1.21 (1.01,1.43)	208 (167,251)	0.87 (0.71,1.04)	-1.11 (-1.27,-0.96)	4118 (3413,4842)	26.98 (22.4,31.74)	4283 (3495,5069)	20.94 (17.18,24.70)	-0.76 (-0.87,-0.66)
Greenland	3 (2,4)	9.15 (6.63,12.01)	3 (2,5)	4.71 (3.20,6.79)	-2.03 (-2.10,-1.96)	88 (65,117)	233.54 (170.09,309.00)	89 (63,122)	114.60 (80.54,160.18)	-2.19 (-2.25,-2.13)
Grenada	1 (1,1)	1.20 (0.86,1.64)	1 (1,1)	0.72 (0.52,0.93)	-1.42 (-1.91,-1.13)	21 (15,28)	32.46 (23.49,43.83)	24 (18,31)	19.00 (14.00,24.55)	-1.61 (-2.01,-1.22)
Guam	0 (0,1)	0.55 (0.40,0.75)	1 (1,1)	0.45 (0.33,0.59)	-0.62 (-0.78,-0.44)	13 (10,17)	14.53 (10.79,19.31)	28 (21,37)	13.64 (10.04,17.75)	-0.28 (-0.44,-0.13)
Guatemala	10 (7,13)	0.35 (0.25,0.47)	21 (15,28)	0.20 (0.14,0.27)	-1.86 (-2.10,-1.54)	255 (193,330)	7.41 (5.51,9.78)	480 (339,644)	4.34 (3.06,5.86)	-1.80 (-2.07,-1.47)
Guinea	5 (3,7)	0.15 (0.10,0.20)	9 (6,14)	0.17 (0.11,0.25)	0.47 (0.43,0.49)	128 (91,178)	3.72 (2.66,5.15)	254 (161,379)	4.28 (2.74,6.37)	0.45 (0.41,0.48)
Guinea-Bissa	1 (1,2)	0.35 (0.23,0.48)	5 (3,7)	0.68 (0.47,0.95)	2.25 (2.22,2.29)	41 (27,59)	9.47 (6.23,13.45)	159 (106,226)	18.81 (12.66,26.36)	2.28 (2.24,2.32)
Guyana	1 (1,2)	0.36 (0.26,0.47)	2 (1,3)	0.31 (0.20,0.45)	-0.25 (-0.47,0.03)	39 (28,50)	9.60 (7.07,12.54)	62 (41,91)	8.70 (5.70,12.69)	-0.27 (-0.50,-0.08)
Haiti	19 (13,28)	0.57 (0.39,0.83)	23 (15,35)	0.31 (0.20,0.48)	-1.87 (-1.91,-1.84)	592 (397,877)	16.25 (10.93,24.01)	688 (433,1043)	8.39 (5.31,12.77)	-2.07 (-2.11,-2.04)
Honduras	5 (3,7)	0.26 (0.18,0.35)	21 (14,29)	0.36 (0.24,0.50)	1.08 (1.01,1.15)	123 (86,169)	5.97 (4.18,8.18)	486 (332,682)	7.71 (5.25,10.78)	0.91 (0.85,0.96)
Hungary	304 (241,362)	2.11 (1.68,2.52)	249 (191,317)	1.36 (1.05,1.72)	-1.45 (-1.58,-1.33)	8970 (7186,10703)	63.85 (51.33,76.44)	6408 (4955,8109)	37.30 (28.97,47.01)	-1.72 (-1.86,-1.60)
Iceland	7 (6,9)	2.57 (2.03,3.09)	10 (7,13)	1.64 (1.20,2.12)	-1.42 (-1.55,-1.32)	166 (132,198)	60.30 (47.87,71.84)	207 (154,265)	37.34 (27.69,47.34)	-1.54 (-1.65,-1.46)
India	6816 (5532,8305)	1.49 (1.20,1.82)	13223 (10368,163	1.13 (0.89,1.40)	-0.84 (-0.91,-0.78)	201502 (164016,24	39.12 (31.82,47.80)	355613 (276612,444	28.40 (22.11,35.31)	-1.00 (-1.05,-0.95)
Indonesia	401 (303,502)	0.44 (0.33,0.55)	1015 (755,1317)	0.45 (0.34,0.58)	0.09 (0.06,0.11)	11056 (8429,13870)	10.66 (8.07,13.37)	27159 (19975,35259	10.56 (7.83,13.69)	-0.05 (-0.08,-0.02)
Iran (Islamic	317 (228,416)	1.33 (0.95,1.74)	757 (561,956)	1.06 (0.77,1.33)	-0.74 (-0.81,-0.69)	8513 (6179,11179)	30.90 (22.32,40.50)	17655 (13351,22164	22.58 (17.06,28.47)	-1.01 (-1.09,-0.95)
Iraq	40 (29,55)	0.53 (0.38,0.74)	107 (69,154)	0.50 (0.33,0.72)	-0.13 (-0.20,-0.05)	1042 (741,1419)	13.09 (9.32,17.86)	2722 (1720,3918)	11.25 (7.17,16.10)	-0.46 (-0.54,-0.38)
Ireland	176 (141,212)	4.25 (3.41,5.14)	155 (114,202)	1.89 (1.40,2.46)	-2.62 (-2.77,-2.53)	3690 (2955,4438)	90.65 (72.78,109.16)	3076 (2301,3987)	39.10 (29.28,50.57)	-2.71 (-2.82,-2.62)
Israel	42 (33,53)	0.87 (0.67,1.09)	60 (44,78)	0.47 (0.35,0.61)	-1.92 (-2.00,-1.84)	910 (722,1130)	19.10 (15.20,23.74)	1238 (946,1575)	10.48 (7.97,13.27)	-1.83 (-1.96,-1.71)
Italy	1365 (1077,1663)	1.54 (1.21,1.87)	841 (638,1070)	0.57 (0.44,0.72)	-3.14 (-3.24,-3.05)	32246 (25741,3890	37.60 (30.12,45.30)	16960 (13113,21328	13.03 (10.24,16.22)	-3.38 (-3.49,-3.30)
Jamaica	15 (11,19)	0.82 (0.61,1.06)	22 (15,32)	0.71 (0.47,1.03)	0.06 (-0.54,0.91)	337 (253,426)	19.51 (14.70,24.52)	517 (346,763)	16.81 (11.26,24.79)	0.08 (-0.57,1.00)
Japan	4629 (3852,5356)	2.69 (2.24,3.12)	5935 (4586,7310)	1.55 (1.22,1.89)	-1.80 (-1.87,-1.75)	111070 (92743,128	63.69 (53.17,73.50)	108771 (85520,1332	33.54 (26.74,40.51)	-2.10 (-2.17,-2.04)
Jordan	6 (5,8)	0.53 (0.39,0.67)	28 (20,36)	0.42 (0.31,0.54)	-0.75 (-0.81,-0.68)	173 (130,227)	12.39 (9.26,16.12)	716 (514,953)	9.18 (6.62,12.09)	-0.95 (-0.99,-0.91)
Kazakhstan	667 (493,862)	5.23 (3.85,6.82)	208 (159,267)	1.15 (0.87,1.48)	-4.65 (-4.85,-4.48)	18472 (13875,2342	138.02 (103.07,175.44)	5565 (4292,7086)	28.81 (22.05,36.74)	-4.84 (-5.07,-4.67)
Kenya	100 (71,147)	1.28 (0.90,1.92)	295 (209,425)	1.34 (0.94,1.92)	0.14 (0.09,0.19)	2729 (1944,3956)	31.77 (22.56,46.16)	8239 (5836,11986)	33.12 (23.47,47.87)	0.14 (0.10,0.17)
Kiribati	1 (1,1)	2.44 (1.73,3.24)	2 (1,2)	2.24 (1.56,3.10)	-0.26 (-0.30,-0.23)	25 (18,33)	62.89 (45.21,83.29)	47 (32,66)	57.65 (39.66,80.42)	-0.27 (-0.30,-0.25)
Kuwait	3 (2,4)	0.62 (0.44,0.80)	9 (6,12)	0.39 (0.27,0.50)	-1.63 (-2.11,-0.88)	97 (70,127)	14.91 (10.68,19.46)	212 (151,280)	7.52 (5.30,9.87)	-1.91 (-2.54,-1.19)
Kyrgyzstan	104 (83,129)	3.47 (2.76,4.25)	58 (45,74)	1.26 (0.99,1.59)	-2.91 (-3.10,-2.72)	2888 (2286,3557)	92.50 (73.50,113.57)	1526 (1161,1969)	30.10 (23.12,38.72)	-3.42 (-3.60,-3.23)
Lao People's	36 (24,50)	1.76 (1.21,2.45)	42 (31,59)	1.00 (0.72,1.39)	-1.80 (-1.82,-1.78)	980 (655,1391)	44.50 (30,62.71)	1114 (804,1572)	23.20 (16.75,32.36)	-2.07 (-2.09,-2.06)
Latvia	47 (38,57)	1.29 (1.06,1.57)	41 (30,51)	1.12 (0.85,1.39)	-0.37 (-0.67,-0.04)	1322 (1092,1603)	36.83 (30.38,44.54)	1056 (797,1304)	31.21 (23.5,38.18)	-0.48 (-0.80,-0.15)
Lebanon	13 (9,18)	0.62 (0.44,0.86)	30 (22,40)	0.49 (0.36,0.66)	-0.69 (-0.75,-0.63)	336 (239,463)	14.76 (10.60,20.45)	638 (467,855)	10.81 (7.94,14.54)	-0.92 (-1.01,-0.85)
Lesotho	27 (17,40)	3.31 (2.09,4.93)	54 (38,74)	5.12 (3.66,7.05)	1.56 (1.47,1.64)	664 (436,969)	77.40 (50.51,113.06)	1513 (1045,2104)	131.64 (91.42,181.96)	1.83 (1.75,1.92)
Liberia	3 (3,5)	0.30 (0.22,0.40)	11 (7,17)	0.52 (0.34,0.76)	1.71 (1.64,1.76)	95 (69,128)	8.01 (5.77,10.79)	343 (223,509)	13.99 (9.05,20.71)	1.77 (1.71,1.83)
Libya	12 (8,18)	0.67 (0.43,1.00)	40 (27,57)	0.81 (0.54,1.14)	0.60 (0.50,0.70)	319 (206,479)	16.37 (10.61,24.56)	1096 (715,1575)	19.48 (12.81,27.49)	0.56 (0.49,0.64)
Lithuania	56 (43,70)	1.23 (0.95,1.53)	69 (53,86)	1.30 (1,1.6)	0.03 (-0.18,0.19)	1541 (1202,1887)	33.94 (26.48,41.52)	1780 (1378,2204)	35.39 (27.46,43.70)	-0.03 (-0.27,0.18)
Luxembourg	12 (8,16)	2.18 (1.56,2.89)	14 (9,19)	1.27 (0.88,1.76)	-1.77 (-1.92,-1.62)	291 (209,386)	54.95 (39.57,73.01)	302 (211,413)	29.45 (20.6,40.09)	-2.10 (-2.27,-1.94)
Madagascar	168 (117,234)	3.24 (2.25,4.47)	225 (142,334)	1.79 (1.14,2.63)	-1.89 (-1.94,-1.83)	5109 (3553,7092)	90.68 (63.34,124.94)	7395 (4647,11010)	51.18 (32.52,75.60)	-1.81 (-1.86,-1.75)
Malawi	124 (91,161)	3.47 (2.58,4.48)	321 (227,435)	4.50 (3.20,6.02)	0.91 (0.86,0.95)	3398 (2522,4402)	84.64 (62.25,109.23)	9001 (6283,12315)	113.16 (79.97,153.38)	1.00 (0.96,1.04)

Malaysia	79 (60,100)	0.90 (0.69,1.15)	216 (164,277)	0.79 (0.60,1.02)	-0.39 (-0.60,-0.14)	2006 (1521,2561)	21.48 (16.38,27.44)	5398 (4068,6957)	18.42 (13.92,23.64)	-0.45 (-0.61,-0.24)
Maldives	1 (1,1)	1.31 (0.92,1.79)	2 (1,2)	0.53 (0.40,0.68)	-2.97 (-3.08,-2.82)	31 (21,43)	31.93 (22.21,43.48)	40 (29,52)	11.40 (8.41,14.79)	-3.38 (-3.51,-3.22)
Mali	10 (7,13)	0.26 (0.19,0.34)	29 (19,41)	0.34 (0.23,0.48)	0.92 (0.89,0.96)	284 (207,373)	6.63 (4.87,8.66)	799 (538,1153)	8.48 (5.67,12.15)	0.83 (0.79,0.86)
Malta	6 (5,7)	1.38 (1.05,1.72)	8 (6,10)	0.83 (0.63,1.08)	-1.56 (-1.69,-1.43)	144 (111,179)	33.49 (25.95,41.42)	176 (131,226)	20.87 (15.84,26.77)	-1.39 (-1.52,-1.25)
Marshall Islar	0 (0,0)	0.65 (0.39,1.06)	0 (0,0)	0.60 (0.36,0.93)	-0.26 (-0.36,-0.12)	3 (2,5)	16.94 (10.01,27.26)	6 (4,10)	15.36 (9.44,23.32)	-0.29 (-0.38,-0.18)
Mauritania	5 (3,7)	0.46 (0.31,0.65)	14 (9,21)	0.65 (0.40,0.97)	1.11 (1.05,1.16)	132 (87,188)	12.55 (8.30,17.78)	389 (232,597)	16.79 (10.15,25.75)	0.94 (0.88,0.99)
Mauritius	7 (6,9)	1.05 (0.84,1.25)	19 (15,23)	1.01 (0.82,1.20)	-0.03 (-0.35,0.30)	198 (161,236)	26.07 (21.21,31.02)	494 (400,588)	25.79 (20.96,30.66)	0.06 (-0.26,0.37)
Mexico	232 (179,287)	0.63 (0.48,0.79)	263 (195,335)	0.21 (0.16,0.27)	-3.44 (-3.57,-3.29)	5336 (4200,6464)	12.86 (10.03,15.73)	6177 (4605,7804)	4.80 (3.58,6.08)	-3.13 (-3.24,-3.00)
Micronesia (F	0 (0,1)	0.93 (0.65,1.28)	1 (0,1)	0.78 (0.52,1.10)	-0.58 (-0.60,-0.56)	13 (9,19)	25.64 (17.72,35.34)	19 (12,27)	21.53 (14.22,30.48)	-0.57 (-0.58,-0.56)
Monaco	1 (1,2)	2.05 (1.30,2.90)	2 (1,3)	1.89 (1.20,2.89)	-0.27 (-0.29,-0.25)	30 (19,43)	49.35 (31.79,69.47)	38 (24,58)	44.47 (28.85,67.62)	-0.35 (-0.37,-0.33)
Mongolia	48 (35,64)	4.78 (3.46,6.40)	83 (58,111)	3.96 (2.75,5.22)	-0.55 (-0.76,-0.41)	1186 (865,1611)	112.81 (82.16,152.37)	2227 (1554,3051)	92.86 (64.89,124.03)	-0.66 (-0.83,-0.49)
Montenegro	7 (5,10)	1.11 (0.80,1.49)	12 (8,16)	1.19 (0.82,1.63)	0.51 (0.36,0.65)	203 (146,270)	30.71 (22.13,40.82)	302 (210,411)	30.46 (21.16,41.38)	0.04 (-0.11,0.17)
Morocco	24 (16,33)	0.17 (0.12,0.24)	51 (33,71)	0.15 (0.10,0.21)	-0.45 (-0.50,-0.41)	619 (433,835)	4.23 (2.96,5.74)	1308 (852,1837)	3.61 (2.34,5.02)	-0.51 (-0.56,-0.47)
Mozambique	73 (52,96)	1.35 (0.98,1.77)	146 (102,195)	1.42 (1.01,1.88)	0.21 (0.18,0.24)	1882 (1363,2512)	31.01 (22.44,41.05)	4006 (2794,5413)	34.29 (24.07,45.89)	0.33 (0.30,0.37)
Myanmar	423 (303,572)	1.85 (1.33,2.49)	380 (270,530)	0.79 (0.56,1.11)	-2.71 (-2.73,-2.69)	11975 (8529,16261)	48.13 (34.46,65.27)	10225 (7303,14306)	19.58 (13.94,27.43)	-2.87 (-2.89,-2.85)
Namibia	3 (2,4)	0.51 (0.38,0.67)	5 (4,7)	0.42 (0.30,0.54)	-0.63 (-0.68,-0.59)	81 (59,109)	12.08 (8.89,16.17)	142 (99,194)	9.85 (7.05,13.13)	-0.64 (-0.69,-0.58)
Nauru	0 (0,0)	1.12 (0.71,1.62)	0 (0,0)	0.81 (0.53,1.12)	-1.04 (-1.06,-1.03)	2 (1,2)	29.71 (18.57,42.51)	1 (1,2)	22.15 (14.44,31.19)	-0.94 (-0.96,-0.92)
Nepal	225 (163,303)	2.53 (1.84,3.38)	393 (271,550)	1.78 (1.23,2.51)	-1.11 (-1.14,-1.09)	6352 (4573,8629)	63.16 (45.61,85.16)	10002 (6807,14419)	41.73 (28.59,59.45)	-1.32 (-1.35,-1.30)
Netherlands	539 (428,644)	2.69 (2.14,3.21)	887 (661,1114)	2.38 (1.78,2.97)	-0.40 (-0.54,-0.28)	12040 (9597,14249)	62.44 (49.83,73.71)	17412 (13205,21652	49.81 (38.12,61.63)	-0.73 (-0.83,-0.65)
New Zealand	70 (53,87)	1.75 (1.33,2.19)	75 (56,98)	0.85 (0.63,1.10)	-2.31 (-2.52,-2.10)	1529 (1171,1870)	38.88 (29.83,47.40)	1459 (1108,1870)	17.37 (13.28,22.17)	-2.60 (-2.80,-2.40)
Nicaragua	3 (2,4)	0.23 (0.17,0.30)	8 (5,11)	0.16 (0.11,0.23)	-1.06 (-1.29,-0.89)	78 (57,101)	5.06 (3.70,6.59)	181 (124,255)	3.66 (2.50,5.16)	-1.07 (-1.28,-0.90)
Niger	7 (5,10)	0.28 (0.19,0.38)	31 (21,46)	0.40 (0.27,0.58)	1.27 (1.21,1.32)	213 (141,302)	7.02 (4.68,9.81)	885 (574,1328)	9.93 (6.62,14.66)	1.17 (1.10,1.23)
Nigeria	78 (54,113)	0.18 (0.13,0.26)	203 (137,286)	0.22 (0.15,0.31)	0.67 (0.63,0.70)	2138 (1451,3158)	4.59 (3.16,6.72)	5812 (3835,8330)	5.70 (3.83,8.05)	0.69 (0.65,0.73)
Niue	0 (0,0)	0.62 (0.40,0.93)	0 (0,0)	0.62 (0.41,0.94)	-0.03 (-0.04,-0.01)	0 (0,0)	15.96 (10.46,23.76)	0 (0,1)	15.31 (10.18,23.02)	-0.14 (-0.15,-0.12)
North Maced	14 (10,18)	0.74 (0.53,0.95)	23 (16,31)	0.71 (0.49,0.94)	-0.17 (-0.33,-0.04)	382 (277,490)	19.17 (13.91,24.56)	596 (409,819)	17.41 (11.92,23.89)	-0.26 (-0.44,-0.11)
Northern Mar	0 (0,0)	0.38 (0.27,0.56)	0 (0,0)	0.63 (0.45,0.87)	1.56 (1.42,1.70)	2 (1,3)	9.71 (6.90,13.88)	9 (7,12)	15.91 (11.71,21.27)	1.57 (1.43,1.71)
Norway	73 (59,89)	1.08 (0.86,1.30)	80 (58,103)	0.78 (0.58,1.00)	-0.97 (-1.12,-0.83)	1594 (1290,1915)	25.49 (20.64,30.37)	1664 (1254,2099)	17.54 (13.36,21.92)	-1.15 (-1.29,-1.02)
Oman	3 (2,4)	0.43 (0.27,0.63)	5 (4,8)	0.32 (0.22,0.46)	-0.96 (-1.05,-0.87)	74 (46,109)	10.49 (6.61,15.43)	151 (98,218)	7.12 (4.75,10.39)	-1.22 (-1.27,-1.15)
Pakistan	1304 (1018,1583)	2.42 (1.89,2.95)	2311 (1735,3052)	2.03 (1.53,2.68)	-0.57 (-0.60,-0.55)	33700 (26553,4119	58.53 (46.02,71.32)	63042 (46968,83795	48.69 (36.56,64.53)	-0.59 (-0.62,-0.57)
Palau	0 (0,0)	0.87 (0.61,1.22)	0 (0,0)	0.73 (0.54,1.00)	-0.63 (-0.67,-0.58)	2 (2,3)	23.00 (16.05,31.83)	5 (4,7)	19.78 (14.42,27.44)	-0.52 (-0.56,-0.49)
Palestine	5 (3,7)	0.61 (0.42,0.86)	8 (6,11)	0.39 (0.27,0.53)	-1.51 (-1.61,-1.43)	107 (70,155)	12.64 (8.43,18.25)	200 (137,279)	8.01 (5.48,11.16)	-1.49 (-1.57,-1.41)
Panama	7 (5,9)	0.49 (0.37,0.64)	10 (7,14)	0.22 (0.15,0.31)	-2.71 (-2.89,-2.54)	149 (113,191)	10.22 (7.75,13.14)	199 (132,279)	4.50 (3.00,6.33)	-2.73 (-2.93,-2.56)
Papua New G	7 (4,11)	0.39 (0.24,0.61)	15 (10,23)	0.28 (0.18,0.43)	-1.05 (-1.09,-1.01)	231 (139,365)	10.80 (6.58,16.83)	485 (321,732)	7.76 (5.11,11.91)	-1.05 (-1.10,-1.00)
Paraguay	33 (24,42)	1.54 (1.12,1.99)	80 (54,115)	1.41 (0.95,2.05)	-0.12 (-0.24,-0.00)	785 (580,1020)	35.28 (26.00,45.56)	1932 (1289,2828)	32.66 (21.75,47.42)	-0.15 (-0.27,-0.04)
Peru	22 (15,31)	0.20 (0.14,0.28)	47 (31,71)	0.14 (0.09,0.21)	-0.90 (-1.14,-0.70)	507 (355,696)	4.28 (2.99,5.91)	1003 (667,1514)	3.00 (1.99,4.54)	-1.00 (-1.28,-0.79)
Philippines	165 (130,209)	0.59 (0.46,0.74)	373 (280,476)	0.46 (0.35,0.58)	-0.78 (-0.85,-0.71)	4744 (3714,6009)	14.77 (11.66,18.75)	10284 (7749,13153)	11.58 (8.71,14.83)	-0.81 (-0.89,-0.74)
Poland	770 (631,910)	1.76 (1.44,2.08)	831 (647,1028)	1.18 (0.92,1.46)	-1.29 (-1.42,-1.19)	20743 (17203,2431	47.48 (39.44,55.69)	20595 (16206,25338	30.88 (24.43,37.91)	-1.39 (-1.52,-1.28)
Portugal	237 (177,302)	1.69 (1.27,2.15)	204 (153,260)	0.94 (0.71,1.17)	-1.91 (-2.02,-1.82)	5912 (4497,7440)	43.16 (32.92,54.08)	5086 (3885,6359)	25.86 (19.93,32.19)	-1.63 (-1.77,-1.48)
Puerto Rico	60 (39,90)	1.68 (1.08,2.50)	38 (24,59)	0.53 (0.34,0.82)	-3.78 (-4.05,-3.50)	1376 (922,2007)	38.11 (25.55,55.51)	805 (521,1243)	12.65 (8.29,19.04)	-3.61 (-3.89,-3.32)
Qatar	1 (1,2)	1.76 (1.10,2.51)	5 (3,7)	0.73 (0.42,1.12)	-2.83 (-3.12,-2.54)	38 (23,57)	37.07 (23.03,53.19)	141 (83,210)	14.68 (8.36,22.67)	-3.00 (-3.24,-2.75)
Republic of K	1019 (766,1292)	3.49 (2.65,4.41)	1138 (810,1522)	1.19 (0.85,1.59)	-3.48 (-3.55,-3.41)	28056 (20966,3552	87.17 (65.46,110.54)	23348 (16494,31424	24.25 (17.08,32.69)	-4.11 (-4.17,-4.05)
Republic of M	55 (41,69)	1.19 (0.89,1.50)	61 (47,75)	0.99 (0.78,1.23)	-0.69 (-1.03,-0.34)	1571 (1163,1998)	33.41 (24.84,42.23)	1652 (1285,2046)	27.46 (21.37,33.94)	-0.72 (-1.05,-0.37)
Romania	163 (127,202)	0.57 (0.44,0.72)	318 (245,403)	0.93 (0.73,1.18)	1.60 (1.49,1.72)	4774 (3752,5874)	16.59 (13.10,20.37)	8736 (6748,10937)	27.14 (21.15,33.78)	1.60 (1.49,1.73)
Russian Fede	3449 (2833,4027)	1.84 (1.51,2.16)	3147 (2526,3810)	1.30 (1.04,1.57)	-1.08 (-1.21,-0.86)	99270 (82162,1153	52.70 (43.59,61.20)	85156 (68287,10269	36.01 (28.87,43.34)	-1.10 (-1.36,-0.85)
Rwanda	129 (84,177)	4.75 (3.14,6.49)	165 (113,232)	2.93 (2.00,4.18)	-1.55 (-1.60,-1.51)	3590 (2296,4924)	119.61 (78.09,163.36)	4294 (2898,6057)	66.28 (45.26,93.46)	-1.90 (-1.95,-1.84)
Saint Kitts an	0 (0,0)	0.76 (0.51,1.09)	0 (0,1)	0.57 (0.38,0.79)	-0.87 (-0.99,-0.74)	7 (4,9)	18.65 (12.90,25.95)	10 (7,14)	13.43 (9.20,18.45)	-0.99 (-1.11,-0.85)
Saint Lucia	1 (1,1)	1.26 (0.90,1.64)	2 (1,3)	0.84 (0.58,1.14)	-1.08 (-1.27,-0.86)	27 (20,34)	31.13 (23.09,39.74)	52 (36,71)	20.95 (14.48,28.53)	-1.14 (-1.32,-0.94)
Saint Vincent	0 (0,0)	0.39 (0.28,0.51)	1 (0,1)	0.41 (0.30,0.56)	0.12 (0.01,0.23)	7 (5,9)	10.27 (7.64,12.97)	16 (12,21)	10.73 (7.87,14.20)	0.09 (-0.02,0.17)
Samoa	0 (0,0)	0.41 (0.29,0.53)	0 (0,1)	0.32 (0.23,0.44)	-0.75 (-0.77,-0.74)	9 (7,12)	10.24 (7.23,13.28)	12 (9,17)	8.10 (5.87,10.89)	-0.76 (-0.78,-0.75)
San Marino	0 (0,0)	0.79 (0.53,1.10)	0 (0,0)	0.35 (0.20,0.55)	-2.95 (-3.39,-2.72)	6 (4,9)	18.55 (12.59,26.09)	5 (3,9)	8.06 (4.41,13.16)	-2.95 (-3.34,-2.75)
Sao Tome and	0 (0,0)	0.16 (0.12,0.23)	0 (0,1)	0.41 (0.28,0.57)	3.00 (2.92,3.08)	3 (2,4)	4.40 (3.12,6.07)	14 (9,19)	10.90 (7.28,15.37)	2.97 (2.89,3.04)
Saudi Arabia	28 (17,43)	0.51 (0.31,0.79)	79 (49,113)	0.47 (0.29,0.68)	-0.27 (-0.32,-0.21)	743 (449,1179)	12.40 (7.43,19.51)	2315 (1457,3366)	10.86 (6.74,15.74)	-0.42 (-0.47,-0.37)
Senegal	14 (10,19)	0.41 (0.29,0.57)	44 (30,64)	0.55 (0.37,0.80)	0.96 (0.86,1.04)	404 (286,558)	11.56 (8.21,15.98)	1270 (859,1869)	14.95 (10.13,21.72)	0.83 (0.73,0.92)
Serbia	113 (76,162)	0.98 (0.67,1.41)	145 (95,212)	0.90 (0.59,1.32)	-0.33 (-0.45,-0.20)	3163 (2099,4531)	25.87 (17.38,36.94)	3637 (2362,5363)	23.89 (15.45,35.28)	-0.18 (-0.29,-0.06)
Seychelles	1 (1,2)	2.19 (1.59,2.91)	2 (2,3)	1.79 (1.37,2.33)	-0.72 (-0.91,-0.56)	32 (23,42)	56.90 (41.34,75.09)	55 (41,72)	43.87 (33,57.43)	-0.84 (-0.96,-0.74)

Sierra Leone	9 (6,12)	0.45 (0.32,0.61)	24 (17,34)	0.63 (0.45,0.88)	1.16 (1.12,1.19)	249 (175,342)	11.87 (8.37,16.28)	695 (486,992)	16.91 (11.85,23.90)	1.18 (1.14,1.22)
Singapore	35 (25,45)	1.64 (1.18,2.15)	37 (27,48)	0.43 (0.31,0.56)	-4.29 (-4.69,-3.92)	876 (642,1138)	38.97 (28.19,50.76)	831 (613,1063)	9.36 (6.92,11.95)	-4.49 (-4.88,-4.10)
Slovakia	101 (69,139)	1.69 (1.17,2.32)	105 (72,149)	1.12 (0.77,1.58)	-1.33 (-1.43,-1.23)	2883 (1971,4016)	49.35 (33.96,68.36)	2780 (1865,3981)	30.56 (20.43,44.06)	-1.57 (-1.69,-1.46)
Slovenia	40 (31,50)	1.61 (1.23,1.99)	40 (29,52)	0.95 (0.69,1.23)	-1.98 (-2.24,-1.81)	1139 (881,1403)	45.61 (35.32,56.12)	985 (715,1253)	24.95 (18.05,31.8)	-2.03 (-2.28,-1.82)
Solomon Isla	1 (1,2)	0.87 (0.58,1.26)	3 (2,4)	0.73 (0.48,1.09)	-0.56 (-0.64,-0.51)	35 (21,51)	22.82 (14.44,33.25)	78 (49,118)	19.77 (12.76,29.44)	-0.47 (-0.57,-0.41)
Somalia	63 (38,94)	2.59 (1.62,3.82)	93 (61,140)	1.52 (1.01,2.24)	-1.69 (-1.71,-1.68)	1971 (1173,3010)	70.15 (42.43,105.8)	2841 (1800,4373)	40.44 (26.60,60.73)	-1.73 (-1.75,-1.71)
South Africa	741 (577,928)	3.66 (2.84,4.59)	949 (746,1174)	2.06 (1.62,2.52)	-1.90 (-2.00,-1.79)	21305 (16822,2660	96.47 (75.90,120.72)	26759 (20794,33463	53.75 (41.96,66.93)	-1.97 (-2.08,-1.85)
South Sudan	67 (45,98)	2.63 (1.76,3.82)	68 (43,101)	1.79 (1.16,2.63)	-1.24 (-1.27,-1.21)	1817 (1191,2677)	68.20 (44.62,100.58)	2000 (1235,2932)	45.68 (29.09,67.50)	-1.26 (-1.29,-1.23)
Spain	1209 (991,1432)	2.25 (1.84,2.66)	943 (715,1150)	1.00 (0.77,1.21)	-2.59 (-2.65,-2.53)	30743 (25167,3635	59.66 (49.15,70.32)	21148 (16267,25599	24.56 (19.02,29.64)	-2.88 (-2.96,-2.81)
Sri Lanka	198 (152,253)	1.97 (1.52,2.51)	317 (188,450)	1.17 (0.70,1.67)	-1.57 (-1.74,-1.42)	5232 (3995,6803)	46.69 (35.90,60.43)	8016 (4784,11643)	28.68 (17.19,41.38)	-1.48 (-1.65,-1.34)
Sudan	122 (80,174)	1.38 (0.91,1.97)	254 (155,384)	1.46 (0.89,2.22)	0.17 (0.16,0.18)	3151 (2036,4563)	33.11 (21.46,47.94)	6356 (3822,9683)	32.37 (19.83,49.45)	-0.07 (-0.09,-0.06)
Suriname	1 (1,2)	0.53 (0.39,0.72)	3 (2,4)	0.42 (0.28,0.61)	-0.60 (-0.81,-0.39)	37 (27,50)	13.77 (10.25,18.56)	77 (52,111)	11.41 (7.63,16.32)	-0.52 (-0.72,-0.33)
Sweden	185 (146,229)	1.18 (0.93,1.45)	242 (176,315)	1.06 (0.77,1.36)	-0.42 (-0.59,-0.27)	3751 (2984,4559)	26.02 (20.80,31.44)	4579 (3369,5886)	22.26 (16.42,28.5)	-0.57 (-0.73,-0.42)
Switzerland	236 (186,286)	2.28 (1.81,2.76)	229 (176,287)	1.19 (0.91,1.49)	-2.11 (-2.28,-1.96)	5374 (4297,6490)	55.38 (44.67,67.07)	4369 (3353,5431)	25.22 (19.37,31.16)	-2.57 (-2.78,-2.42)
Syrian Arab R	16 (12,22)	0.34 (0.25,0.46)	38 (25,54)	0.34 (0.23,0.47)	-0.04 (-0.12,0.03)	414 (292,566)	7.77 (5.51,10.54)	939 (603,1345)	7.04 (4.61,9.94)	-0.35 (-0.44,-0.27)
Taiwan (Prov	775 (631,917)	4.90 (3.99,5.76)	1384 (1096,1662)	3.29 (2.61,3.95)	-1.40 (-1.50,-1.31)	21231 (17163,2510	125.40 (102.22,148.14)	37800 (29999,45812	92.9 (73.53,112.54)	-0.98 (-1.10,-0.88)
Tajikistan	129 (92,169)	4.84 (3.45,6.33)	105 (64,165)	2.12 (1.26,3.30)	-2.69 (-2.84,-2.55)	3355 (2360,4473)	118.67 (83.51,156.41)	2683 (1615,4211)	46.22 (27.79,72.15)	-3.15 (-3.30,-3.02)
Thailand	743 (530,1060)	2.17 (1.55,3.09)	1554 (1131,2183)	1.40 (1.02,1.97)	-1.54 (-1.64,-1.43)	20339 (14442,2946	53.75 (38.23,77.17)	40833 (29374,56970	36.98 (26.68,51.57)	-1.19 (-1.33,-0.99)
Timor-Leste	2 (1,3)	0.75 (0.49,1.09)	5 (3,7)	0.59 (0.39,0.83)	-0.79 (-0.84,-0.74)	56 (34,83)	18.32 (11.47,27.12)	123 (80,175)	14.10 (9.22,20.13)	-0.85 (-0.88,-0.80)
Togo	8 (6,13)	0.73 (0.48,1.09)	43 (28,63)	1.15 (0.76,1.66)	1.52 (1.48,1.57)	232 (152,337)	17.99 (11.87,26.44)	1247 (809,1838)	29.25 (19.03,42.74)	1.59 (1.55,1.65)
Tokelau	0 (0,0)	0.61 (0.36,0.93)	0 (0,0)	0.46 (0.30,0.67)	-0.90 (-0.92,-0.87)	0 (0,0)	15.06 (8.96,22.77)	0 (0,0)	11.58 (7.65,16.31)	-0.85 (-0.87,-0.83)
Tonga	0 (0,1)	0.84 (0.58,1.14)	1 (0,1)	0.75 (0.52,1.07)	-0.42 (-0.52,-0.33)	11 (8,15)	19.66 (13.69,26.62)	14 (10,20)	17.65 (12.28,25.13)	-0.42 (-0.51,-0.33)
Trinidad and	5 (4,6)	0.62 (0.47,0.79)	8 (5,11)	0.41 (0.27,0.58)	-1.07 (-1.35,-0.80)	133 (103,164)	15.76 (12.19,19.56)	212 (141,298)	10.78 (7.19,15.19)	-0.95 (-1.24,-0.66)
Tunisia	15 (11,19)	0.31 (0.22,0.41)	39 (24,57)	0.30 (0.19,0.43)	-0.16 (-0.24,-0.11)	351 (252,471)	6.87 (4.96,9.14)	893 (546,1327)	6.55 (4.03,9.66)	-0.15 (-0.19,-0.10)
Turkey	340 (245,449)	1.01 (0.74,1.32)	570 (389,765)	0.62 (0.42,0.83)	-1.60 (-1.64,-1.55)	9303 (6740,12490)	25.22 (18.37,33.62)	13809 (9371,18683)	14.31 (9.72,19.33)	-1.85 (-1.89,-1.81)
Turkmenistan	198 (162,234)	10.45 (8.56,12.34)	90 (62,124)	2.34 (1.63,3.22)	-4.60 (-4.88,-4.36)	5422 (4408,6456)	268.42 (219.29,316.68)	2389 (1631,3290)	56.49 (38.83,77.82)	-4.85 (-5.05,-4.65)
Tuvalu	0 (0,0)	0.73 (0.50,1.03)	0 (0,0)	0.58 (0.40,0.83)	-0.73 (-0.74,-0.72)	1 (1,2)	19.36 (13.40,26.88)	2 (1,2)	15.32 (10.74,21.61)	-0.75 (-0.76,-0.74)
Uganda	98 (70,131)	1.59 (1.13,2.10)	204 (146,281)	1.44 (1.03,1.98)	-0.33 (-0.37,-0.28)	2698 (1913,3631)	39.90 (28.24,53.52)	5791 (4121,7976)	36.20 (25.84,49.85)	-0.33 (-0.38,-0.28)
Ukraine	1073 (873,1278)	1.47 (1.19,1.75)	685 (431,1013)	0.91 (0.57,1.34)	-1.46 (-1.69,-1.20)	31189 (25266,3727	43.18 (34.98,51.57)	19526 (12183,29208	26.81 (16.76,40.03)	-1.45 (-1.70,-1.18)
United Arab E	2 (2,4)	0.69 (0.44,1.05)	12 (8,17)	0.56 (0.37,0.80)	-0.82 (-1.17,-0.51)	73 (48,114)	15.54 (9.96,24.35)	376 (251,536)	10.02 (6.55,14.11)	-1.63 (-2.06,-1.30)
United Kingd	3512 (2772,4143)	3.77 (2.99,4.45)	3773 (2809,4721)	2.72 (2.04,3.39)	-1.00 (-1.07,-0.94)	73984 (59094,8681	84.54 (67.71,98.99)	71016 (53586,88111	55.99 (42.65,69.37)	-1.28 (-1.35,-1.21)
United Repub	298 (217,403)	2.83 (2.09,3.80)	371 (254,526)	1.51 (1.04,2.11)	-2.01 (-2.04,-1.98)	8285 (5989,11183)	71.85 (52.27,98.05)	10313 (7030,14814)	37.50 (25.64,53.78)	-2.08 (-2.10,-2.05)
United States	6956 (5663,8150)	2.22 (1.81,2.59)	10453 (8063,1280	1.74 (1.35,2.13)	-0.77 (-0.83,-0.72)	168946 (138654,19	56.49 (46.34,65.63)	231871 (180692,279	40.49 (31.69,48.76)	-1.09 (-1.14,-1.04)
United States	1 (0,1)	0.83 (0.47,1.32)	1 (0,1)	0.48 (0.27,0.78)	-1.85 (-2.01,-1.72)	18 (10,28)	20.03 (11.50,31.57)	20 (11,33)	11.36 (6.44,18.47)	-1.91 (-2.07,-1.78)
Uruguay	117 (90,146)	2.97 (2.27,3.70)	96 (72,122)	1.75 (1.33,2.19)	-1.78 (-1.99,-1.54)	2882 (2257,3547)	74.78 (58.58,91.87)	2142 (1654,2645)	41.94 (32.4,51.6)	-1.93 (-2.13,-1.71)
Uzbekistan	252 (187,323)	2.18 (1.61,2.80)	176 (131,228)	0.70 (0.52,0.91)	-3.58 (-3.90,-3.36)	7127 (5412,8978)	59.30 (44.96,74.86)	4834 (3622,6233)	17.17 (12.78,22.32)	-3.69 (-3.96,-3.46)
Vanuatu	0 (0,1)	0.57 (0.39,0.82)	1 (0,1)	0.40 (0.28,0.56)	-1.18 (-1.21,-1.13)	10 (7,15)	14.89 (10.25,21.89)	21 (15,29)	10.60 (7.41,14.66)	-1.14 (-1.18,-1.09)
Venezuela (B	62 (48,78)	0.69 (0.53,0.87)	89 (54,125)	0.30 (0.18,0.42)	-2.58 (-2.77,-2.37)	1523 (1194,1912)	15.73 (12.24,19.82)	2165 (1315,3080)	7.01 (4.25,9.98)	-2.50 (-2.76,-2.25)
Viet Nam	349 (245,480)	0.87 (0.62,1.19)	945 (667,1259)	0.93 (0.67,1.22)	0.21 (0.19,0.25)	9148 (6324,12857)	22.13 (15.40,30.85)	26254 (18083,35587	24.10 (16.84,32.36)	0.29 (0.26,0.32)
Yemen	108 (72,153)	2.27 (1.53,3.21)	226 (142,322)	1.76 (1.11,2.50)	-0.81 (-0.84,-0.77)	3035 (2020,4345)	57.58 (38.76,81.75)	5945 (3666,8615)	40.68 (25.67,58.15)	-1.09 (-1.14,-1.05)
Zambia	73 (52,99)	2.74 (1.94,3.72)	126 (85,177)	1.97 (1.35,2.81)	-1.04 (-1.08,-1.01)	1994 (1416,2720)	67.37 (48.03,91.10)	3519 (2319,4978)	47.92 (32.39,67.50)	-1.10 (-1.13,-1.06)
Zimbabwe	154 (105,210)	4.02 (2.73,5.50)	260 (181,346)	4.01 (2.86,5.31)	0.02 (-0.04,0.09)	4037 (2782,5508)	96.13 (66.18,131.45)	7297 (4945,9878)	98.25 (68.02,129.87)	0.13 (0.07,0.21)
Afghanistan	83 (37,132)	1.26 (0.56,1.99)	114 (48,166)	1.24 (0.48,1.83)	0.37 (0.36,0.39)	2145 (993,3411)	29.92 (13.79,47.19)	3338 (1485,4985)	29.47 (12.34,43.15)	-0.03 (-0.05,-0.00)
Albania	63 (45,88)	3.33 (2.4,4.63)	93 (65,131)	2.07 (1.47,2.93)	-0.28 (-0.44,-0.16)	1452 (1013,2045)	71.12 (50.38,100.39)	1883 (1320,2616)	42.55 (29.86,58.76)	-1.57 (-1.71,-1.44)
Algeria	85 (56,113)	0.89 (0.59,1.17)	126 (85,177)	0.44 (0.29,0.61)	-0.97 (-1.00,-0.95)	1973 (1292,2636)	17.14 (11.40,22.75)	2778 (1832,3830)	8.23 (5.49,11.43)	-2.25 (-2.29,-2.22)
American Sar	0 (0,1)	1.68 (1.25,2.25)	1 (0,1)	1.18 (0.87,1.58)	0.39 (0.26,0.51)	12 (9,16)	45.72 (34.33,61.20)	17 (13,23)	32.89 (24.02,44.01)	-1.10 (-1.16,-1.03)
Andorra	1 (1,1)	1.73 (1.04,2.67)	1 (1,2)	0.63 (0.38,0.97)	-2.38 (-2.58,-2.23)	23 (14,34)	38.81 (23.87,58.95)	21 (13,32)	13.89 (8.33,21.26)	-3.35 (-3.56,-3.21)
Angola	29 (18,40)	0.73 (0.45,1.01)	46 (30,63)	0.39 (0.26,0.53)	-0.42 (-0.45,-0.38)	878 (561,1245)	19.47 (12.39,27.29)	1423 (925,1989)	10.21 (6.67,13.96)	-1.98 (-2.04,-1.90)
Antigua and I	1 (0,1)	1.02 (0.77,1.34)	1 (0,1)	0.52 (0.39,0.67)	-0.17 (-0.41,0.13)	12 (9,16)	23.73 (18.35,30.38)	13 (10,16)	11.48 (8.85,14.64)	-2.42 (-2.79,-2.01)
Argentina	453 (361,552)	1.39 (1.10,1.69)	385 (307,475)	0.69 (0.55,0.85)	-2.10 (-2.20,-2.02)	12075 (9715,14512)	36.80 (29.65,44.21)	9826 (7919,11983)	18.23 (14.70,22.23)	-2.16 (-2.23,-2.08)
Armenia	104 (85,124)	3.77 (3.06,4.49)	78 (63,95)	1.78 (1.43,2.16)	0.18 (-0.05,0.36)	2949 (2422,3514)	100.43 (82.20,119.25)	1819 (1473,2209)	41.75 (33.89,50.55)	-2.60 (-2.86,-2.40)
Australia	148 (117,182)	0.75 (0.59,0.91)	98 (76,127)	0.22 (0.17,0.28)	-3.16 (-3.23,-3.10)	3446 (2771,4130)	17.62 (14.16,21.07)	2091 (1663,2620)	5.12 (4.09,6.27)	-3.95 (-4.03,-3.88)
Austria	208 (166,255)	1.72 (1.38,2.09)	89 (69,112)	0.49 (0.38,0.61)	-1.41 (-1.55,-1.29)	4510 (3609,5386)	39.96 (32.18,47.46)	1896 (1501,2346)	11.57 (9.20,14.33)	-3.96 (-4.03,-3.88)
Azerbaijan	157 (106,214)	3.09 (2.09,4.26)	195 (138,289)	2.02 (1.44,3.02)	-0.12 (-0.21,-0.03)	4544 (3155,6141)	84.31 (57.56,114.51)	5187 (3652,7746)	47.87 (33.71,71.83)	-1.38 (-1.46,-1.32)
Bahamas	1 (1,1)	0.71 (0.50,0.99)	2 (1,2)	0.41 (0.29,0.56)	0.06 (-0.08,0.18)	29 (22,39)	17.92 (13.25,24.78)	42 (30,56)	9.83 (6.94,13.18)	-1.70 (-1.88,-1.47)

Bahrain	2 (2,3)	1.75 (1.17,2.50)	4 (3,6)	0.71 (0.48,1.02)	-1.88 (-1.99,-1.76)	60 (40,84)	36.37 (24.46,51.78)	111 (76,164)	13.46 (9.27,19.40)	-2.96 (-3.07,-2.82)
Bangladesh	560 (349,759)	1.23 (0.78,1.66)	733 (453,1023)	0.55 (0.34,0.76)	-1.16 (-1.31,-1.04)	15514 (9451,21219)	31.76 (19.54,43.16)	18333 (11382,25840	12.98 (8.02,18.22)	-2.43 (-2.55,-2.31)
Barbados	3 (2,4)	0.90 (0.67,1.17)	2 (1,3)	0.37 (0.25,0.51)	-0.44 (-0.68,-0.20)	57 (43,74)	19.47 (14.95,24.81)	39 (26,53)	7.65 (5.18,10.48)	-2.70 (-2.90,-2.51)
Belarus	534 (416,680)	4.10 (3.20,5.21)	257 (186,338)	1.60 (1.16,2.10)	-0.96 (-1.15,-0.77)	14617 (11441,1832	112.69 (88.27,141.24)	6592 (4785,8609)	42.27 (30.73,55.20)	-3.05 (-3.28,-2.81)
Belgium	292 (232,362)	1.84 (1.46,2.27)	123 (93,156)	0.50 (0.39,0.63)	-2.62 (-2.88,-2.41)	5859 (4720,7066)	38.6 (31.38,46.35)	2394 (1849,2968)	11.07 (8.68,13.62)	-4.13 (-4.22,-4.03)
Belize	1 (1,1)	0.74 (0.59,0.92)	1 (1,2)	0.48 (0.37,0.62)	0.32 (0.16,0.46)	17 (14,20)	18.1 (14.56,22.04)	37 (29,48)	11.68 (9.09,14.92)	-1.32 (-1.53,-1.12)
Benin	7 (5,10)	0.37 (0.25,0.51)	8 (5,11)	0.17 (0.10,0.23)	-2.30 (-2.35,-2.26)	192 (126,269)	9.37 (6.17,13.04)	228 (139,306)	4.17 (2.55,5.66)	-2.52 (-2.56,-2.49)
Bermuda	1 (0,1)	0.82 (0.60,1.08)	0 (0,1)	0.30 (0.22,0.40)	-1.39 (-1.52,-1.26)	12 (9,16)	19.35 (14.62,24.84)	9 (6,12)	6.80 (5.03,8.92)	-3.35 (-3.49,-3.17)
Bhutan	1 (1,2)	0.42 (0.23,0.70)	1 (1,2)	0.26 (0.16,0.41)	-0.36 (-0.40,-0.33)	27 (15,43)	10.34 (5.79,16.62)	34 (20,54)	5.60 (3.36,8.91)	-1.53 (-1.57,-1.50)
Bolivia (Pluri	70 (52,94)	2.28 (1.71,3.03)	109 (75,160)	1.23 (0.85,1.82)	-0.91 (-0.95,-0.89)	1881 (1408,2535)	56.43 (42.23,75.79)	2730 (1848,4066)	28.95 (19.61,42.84)	-1.93 (-1.96,-1.90)
Bosnia and H	79 (59,104)	2.01 (1.49,2.67)	86 (60,120)	1.35 (0.95,1.87)	1.00 (0.89,1.09)	2082 (1585,2713)	48.42 (36.13,63.02)	1907 (1336,2645)	30.99 (21.77,42.80)	-1.21 (-1.33,-1.06)
Botswana	4 (3,6)	0.79 (0.50,1.10)	5 (3,7)	0.37 (0.23,0.51)	-0.85 (-0.91,-0.81)	121 (73,167)	19.98 (12.29,27.58)	145 (90,205)	9.03 (5.68,12.71)	-2.38 (-2.43,-2.33)
Brazil	2434 (1961,2947)	2.89 (2.32,3.54)	1845 (1465,2297)	0.74 (0.59,0.92)	-1.97 (-2.05,-1.90)	63095 (51337,7520	68.20 (55.37,82.02)	44111 (35566,54125	17.27 (13.92,21.2)	-4.27 (-4.32,-4.22)
Brunei Darus	3 (2,4)	3.60 (2.29,4.77)	3 (2,4)	0.95 (0.67,1.29)	-2.26 (-2.40,-2.14)	86 (56,114)	81.87 (53.12,108.33)	77 (55,105)	21.00 (15.03,28.49)	-4.32 (-4.50,-4.17)
Bulgaria	378 (292,484)	2.98 (2.32,3.78)	188 (141,245)	1.35 (1.02,1.76)	0.39 (0.29,0.49)	10195 (8025,13064)	80.71 (63.76,102.22)	4713 (3570,6126)	36.89 (28.19,47.95)	-2.45 (-2.57,-2.35)
Burkina Faso	13 (8,18)	0.29 (0.19,0.40)	20 (12,28)	0.22 (0.13,0.30)	-0.74 (-0.78,-0.71)	384 (243,519)	8.08 (5.16,10.93)	596 (356,804)	5.78 (3.44,7.84)	-0.98 (-1.02,-0.95)
Burundi	14 (8,19)	0.60 (0.37,0.83)	11 (7,16)	0.23 (0.15,0.33)	-2.56 (-2.59,-2.53)	394 (239,559)	15.96 (9.71,22.62)	341 (212,481)	6.14 (3.78,8.65)	-2.96 (-2.99,-2.94)
Cabo Verde	2 (1,3)	0.92 (0.65,1.24)	2 (2,3)	0.55 (0.38,0.77)	6.14 (5.55,6.68)	53 (38,71)	24.36 (17.34,33.23)	66 (45,96)	13.97 (9.60,20.02)	-1.65 (-1.90,-1.34)
Cambodia	100 (67,141)	2.41 (1.61,3.41)	152 (105,217)	1.35 (0.94,1.89)	-0.23 (-0.25,-0.21)	2702 (1782,3838)	57.63 (38.37,81.35)	3886 (2638,5589)	30.43 (20.83,43.63)	-1.85 (-1.87,-1.83)
Cameroon	16 (11,22)	0.36 (0.24,0.48)	29 (17,42)	0.23 (0.13,0.32)	-1.03 (-1.05,-1.00)	500 (324,671)	9.82 (6.40,13.10)	884 (514,1288)	6.06 (3.53,8.78)	-1.47 (-1.50,-1.43)
Canada	456 (353,554)	1.38 (1.07,1.68)	314 (240,401)	0.41 (0.32,0.52)	-2.73 (-2.85,-2.58)	9942 (7846,12045)	30.52 (24.16,36.92)	6022 (4726,7589)	8.63 (6.85,10.83)	-3.80 (-3.93,-3.68)
Central Afric	8 (5,12)	0.67 (0.41,0.93)	9 (6,13)	0.38 (0.23,0.52)	-1.26 (-1.29,-1.23)	253 (153,361)	18.75 (11.34,26.71)	299 (184,437)	10.65 (6.61,15.16)	-1.86 (-1.89,-1.83)
Chad	12 (8,16)	0.42 (0.28,0.56)	21 (13,30)	0.38 (0.25,0.54)	-0.30 (-0.33,-0.27)	299 (202,403)	10.32 (6.94,13.89)	576 (362,836)	9.30 (5.94,13.39)	-0.31 (-0.35,-0.26)
Chile	247 (198,301)	2.42 (1.94,2.97)	213 (167,266)	0.84 (0.65,1.04)	-1.86 (-1.99,-1.73)	7050 (5759,8544)	66.85 (54.49,81.26)	5712 (4524,6965)	22.9 (18.14,27.97)	-3.40 (-3.46,-3.34)
China	51502 (38094,659	6.23 (4.66,8.03)	65604 (48081,908	3.10 (2.27,4.30)	-1.23 (-1.33,-1.14)	1448966 (1070928,1	160.20 (118.18,204.68)	1550245 (1135747,2	71.07 (52.06,99.29)	-2.25 (-2.32,-2.20)
Colombia	326 (263,398)	1.88 (1.51,2.30)	259 (193,345)	0.47 (0.35,0.62)	-2.98 (-3.20,-2.80)	9097 (7427,11050)	48.26 (39.14,58.78)	6758 (5036,8963)	12.22 (9.10,16.2)	-4.44 (-4.67,-4.33)
Comoros	1 (1,1)	0.54 (0.32,0.77)	1 (1,2)	0.29 (0.18,0.42)	-0.76 (-0.80,-0.72)	27 (15,41)	13.12 (7.31,19.13)	34 (22,50)	6.63 (4.25,9.65)	-2.03 (-2.09,-1.97)
Congo	5 (3,7)	0.49 (0.31,0.66)	8 (5,11)	0.31 (0.19,0.41)	0.17 (0.15,0.20)	153 (98,211)	13.02 (8.29,17.77)	240 (151,335)	7.75 (4.88,10.50)	-1.48 (-1.53,-1.42)
Cook Islands	0 (0,0)	0.87 (0.64,1.21)	0 (0,0)	0.39 (0.28,0.53)	-0.75 (-0.81,-0.71)	3 (2,4)	22.61 (16.55,31.05)	3 (2,3)	10.30 (7.19,13.8)	-2.56 (-2.60,-2.53)
Costa Rica	58 (45,72)	3.44 (2.69,4.30)	54 (42,69)	0.99 (0.76,1.25)	-1.62 (-1.80,-1.44)	1352 (1093,1657)	76.95 (61.94,94.82)	1264 (972,1575)	22.93 (17.65,28.56)	-3.98 (-4.13,-3.81)
Croatia	245 (190,307)	4.17 (3.24,5.21)	115 (88,146)	1.23 (0.93,1.56)	-0.46 (-0.49,-0.43)	5791 (4534,7201)	93.64 (73.72,116.38)	2306 (1750,2931)	26.90 (20.45,34.07)	-1.40 (-1.43,-1.37)
Cuba	97 (76,123)	0.96 (0.75,1.20)	102 (78,131)	0.52 (0.40,0.66)	-0.50 (-0.66,-0.37)	2299 (1847,2838)	22.49 (18.07,27.71)	2415 (1861,3077)	12.53 (9.66,15.91)	-3.93 (-4.07,-3.81)
Cyprus	11 (8,16)	1.54 (1.12,2.25)	14 (10,20)	0.69 (0.47,0.94)	-0.32 (-0.48,-0.14)	237 (174,341)	30.82 (22.72,44.31)	297 (205,404)	14.37 (9.90,19.38)	-1.89 (-2.03,-1.77)
Czechia	312 (245,400)	2.21 (1.73,2.81)	127 (96,161)	0.58 (0.44,0.73)	-1.99 (-2.11,-1.88)	7356 (5803,9326)	53.32 (42.09,67.38)	2794 (2124,3553)	13.77 (10.49,17.52)	-2.54 (-2.63,-2.45)
C么te d'Ivoir	6 (4,7)	0.14 (0.09,0.19)	10 (6,14)	0.09 (0.06,0.12)	-1.05 (-1.11,-0.99)	169 (114,228)	3.63 (2.45,4.84)	302 (189,422)	2.32 (1.47,3.17)	-4.18 (-4.32,-4.03)
Democratic P	478 (322,682)	2.83 (1.90,4.06)	727 (502,989)	2.13 (1.48,2.89)	-0.64 (-0.65,-0.62)	14566 (9688,20999)	79.05 (53.18,113.81)	21219 (14373,29299	60.68 (41.48,83.61)	-0.91 (-0.92,-0.90)
Democratic R	51 (32,69)	0.32 (0.20,0.43)	78 (49,109)	0.20 (0.13,0.29)	-0.88 (-0.91,-0.85)	1498 (945,2057)	8.47 (5.42,11.57)	2407 (1516,3402)	5.54 (3.48,7.75)	-1.46 (-1.50,-1.42)
Denmark	162 (131,198)	1.92 (1.55,2.34)	81 (62,102)	0.64 (0.49,0.80)	-1.59 (-1.83,-1.31)	3257 (2658,3942)	41.52 (34.03,49.97)	1560 (1201,1931)	13.47 (10.45,16.61)	-3.47 (-3.58,-3.37)
Djibouti	1 (1,1)	0.64 (0.40,0.95)	3 (2,4)	0.51 (0.32,0.73)	0.10 (0.08,0.12)	25 (15,38)	15.90 (9.84,24.07)	86 (54,124)	12.14 (7.62,17.18)	-0.71 (-0.74,-0.68)
Dominica	1 (1,1)	1.28 (0.96,1.67)	1 (0,1)	0.78 (0.56,1.07)	-0.44 (-0.47,-0.41)	18 (13,23)	30.70 (23.09,39.26)	16 (12,23)	19.06 (13.78,26.11)	-1.61 (-1.64,-1.58)
Dominican R	30 (22,42)	0.93 (0.66,1.31)	60 (41,82)	0.61 (0.42,0.84)	-0.29 (-0.57,-0.12)	702 (520,965)	19.22 (14.17,26.77)	1341 (935,1837)	13.36 (9.30,18.35)	-1.31 (-1.47,-1.13)
Ecuador	96 (78,117)	1.90 (1.54,2.33)	118 (85,159)	0.73 (0.53,0.99)	-1.09 (-1.53,-0.79)	2438 (1994,2921)	45.19 (36.93,54.51)	2852 (2035,3842)	17.21 (12.32,23.16)	-3.21 (-3.45,-2.97)
Egypt	120 (87,195)	0.47 (0.33,0.86)	432 (255,597)	0.80 (0.49,1.09)	-1.56 (-1.67,-1.45)	3515 (2634,5207)	11.74 (8.55,18.84)	11616 (6589,16078)	17.71 (10.39,24.46)	1.72 (1.60,1.85)
El Salvador	18 (14,25)	0.62 (0.47,0.85)	37 (26,50)	0.60 (0.42,0.82)	0.34 (0.20,0.48)	512 (396,690)	16.55 (12.75,22.32)	985 (704,1330)	16.33 (11.68,22.07)	-0.11 (-0.38,0.12)
Equatorial Gu	1 (1,2)	0.55 (0.34,0.80)	1 (1,2)	0.21 (0.13,0.30)	0.12 (0.06,0.17)	34 (21,51)	15.41 (9.35,22.71)	33 (19,48)	5.51 (3.30,7.84)	-3.07 (-3.13,-3.01)
Eritrea	5 (3,7)	0.34 (0.19,0.51)	6 (3,8)	0.17 (0.11,0.25)	-0.71 (-0.75,-0.68)	169 (92,252)	10.64 (5.91,15.65)	194 (114,293)	5.17 (3.13,7.68)	-2.21 (-2.28,-2.14)
Estonia	54 (43,68)	2.64 (2.10,3.32)	23 (18,30)	0.91 (0.70,1.17)	-0.55 (-0.76,-0.29)	1516 (1217,1881)	74.98 (60.31,92.83)	553 (423,701)	23.61 (18.11,29.72)	-3.58 (-3.74,-3.43)
Eswatini	1 (1,2)	0.46 (0.30,0.63)	1 (1,2)	0.27 (0.18,0.37)	-1.00 (-1.07,-0.93)	34 (22,47)	11.32 (7.29,15.63)	42 (27,60)	6.86 (4.43,9.58)	-1.68 (-1.74,-1.63)
Ethiopia	62 (37,88)	0.29 (0.18,0.42)	41 (27,57)	0.10 (0.06,0.14)	-1.22 (-1.25,-1.20)	1876 (1120,2614)	8.19 (4.93,11.52)	1142 (749,1599)	2.42 (1.59,3.41)	-3.54 (-3.57,-3.51)
Fiji	3 (1,4)	0.79 (0.38,1.08)	3 (1,5)	0.43 (0.18,0.63)	-0.53 (-0.62,-0.45)	93 (45,129)	21.79 (10.44,29.93)	101 (42,151)	11.61 (4.76,17.23)	-1.94 (-2.03,-1.81)
Finland	81 (62,104)	1.14 (0.88,1.45)	34 (25,45)	0.27 (0.20,0.35)	-2.87 (-2.99,-2.76)	1963 (1539,2463)	28.58 (22.59,35.61)	709 (529,923)	6.52 (4.95,8.30)	-4.53 (-4.63,-4.46)
France	1121 (884,1389)	1.31 (1.04,1.61)	567 (432,735)	0.40 (0.31,0.51)	-1.83 (-1.92,-1.75)	23031 (18449,2827	28.78 (23.27,35.04)	11760 (9121,14828)	9.84 (7.75,12.29)	-3.68 (-3.76,-3.63)
Gabon	2 (1,3)	0.37 (0.22,0.52)	3 (2,4)	0.25 (0.15,0.33)	-0.01 (-0.04,0.02)	59 (36,85)	9.90 (6.00,14.16)	78 (49,108)	6.54 (4.07,8.88)	-1.27 (-1.31,-1.21)
Gambia	1 (0,1)	0.22 (0.14,0.31)	1 (1,2)	0.11 (0.07,0.15)	-1.05 (-1.15,-0.94)	23 (14,33)	5.92 (3.68,8.33)	32 (21,45)	3.00 (1.95,4.12)	-2.03 (-2.14,-1.91)
Georgia	132 (104,165)	2.07 (1.64,2.58)	94 (75,116)	1.58 (1.27,1.92)	1.55 (1.19,1.84)	3741 (2963,4656)	58.21 (46.66,72.19)	2284 (1839,2772)	39.94 (32.36,48.33)	-1.02 (-1.49,-0.59)

Germany	2651 (2010,3329)	2.02 (1.54,2.52)	1211 (918,1556)	0.61 (0.48,0.77)	-2.16 (-2.25,-2.07)	55916 (42951,6926	45.13 (34.93,55.75)	24619 (19170,30744	14.38 (11.2,17.74)	-3.74 (-3.79,-3.67)
Ghana	12 (7,16)	0.22 (0.14,0.30)	21 (14,29)	0.14 (0.10,0.19)	-0.40 (-0.44,-0.36)	313 (205,438)	4.91 (3.14,6.77)	551 (373,766)	3.19 (2.18,4.42)	-1.36 (-1.40,-1.32)
Greece	464 (375,558)	3.02 (2.44,3.64)	317 (253,394)	1.24 (1.01,1.53)	-1.28 (-1.39,-1.20)	9918 (8064,11804)	65.50 (53.42,77.69)	5940 (4822,7265)	27.37 (22.21,33.22)	-2.86 (-2.96,-2.77)
Greenland	1 (1,2)	3.52 (2.43,5.02)	1 (1,1)	1.11 (0.78,1.58)	-1.89 (-2.02,-1.77)	35 (25,49)	91.20 (63.06,127.97)	21 (15,29)	28.23 (20.22,39.15)	-3.55 (-3.62,-3.47)
Grenada	0 (0,1)	0.65 (0.50,0.83)	0 (0,0)	0.31 (0.23,0.39)	-0.16 (-0.30,-0.02)	11 (9,14)	17.20 (13.37,21.75)	10 (7,12)	7.83 (5.89,9.95)	-2.17 (-2.45,-2.00)
Guam	0 (0,1)	0.48 (0.37,0.64)	1 (0,1)	0.31 (0.23,0.40)	-0.30 (-0.56,-0.08)	12 (10,17)	13.40 (10.28,17.84)	20 (15,25)	9.95 (7.34,12.62)	-1.47 (-1.64,-1.32)
Guatemala	36 (28,44)	1.16 (0.91,1.45)	81 (61,106)	0.75 (0.56,0.98)	-1.89 (-2.10,-1.61)	959 (757,1192)	26.46 (20.98,33.13)	2082 (1555,2712)	18.29 (13.69,23.85)	-1.61 (-1.86,-1.40)
Guinea	12 (8,17)	0.35 (0.24,0.51)	16 (10,22)	0.29 (0.19,0.41)	0.32 (0.30,0.33)	309 (207,448)	8.98 (6.05,13.00)	444 (288,623)	7.34 (4.79,10.34)	-0.63 (-0.66,-0.61)
Guinea-Bissa	1 (1,2)	0.34 (0.22,0.47)	2 (1,3)	0.30 (0.19,0.39)	0.09 (0.06,0.11)	41 (27,59)	9.28 (6.00,13.43)	71 (46,97)	8.13 (5.33,10.95)	-0.41 (-0.44,-0.38)
Guyana	3 (2,3)	0.69 (0.54,0.89)	2 (1,3)	0.33 (0.22,0.46)	-0.56 (-0.89,-0.30)	73 (58,93)	18.05 (14.16,22.99)	62 (42,90)	8.86 (6.03,12.70)	-2.28 (-2.44,-2.10)
Haiti	34 (22,51)	1.04 (0.67,1.52)	34 (21,50)	0.49 (0.31,0.71)	-1.03 (-1.06,-1.00)	1035 (649,1526)	28.62 (18.09,42.35)	997 (608,1442)	12.44 (7.66,17.91)	-2.34 (-2.38,-2.30)
Honduras	21 (15,27)	1.05 (0.75,1.39)	61 (43,84)	1.01 (0.70,1.39)	0.86 (0.81,0.91)	565 (410,740)	26.11 (18.75,34.33)	1520 (1090,2074)	23.34 (16.55,31.96)	-0.05 (-0.10,0.00)
Hungary	411 (312,529)	2.75 (2.10,3.51)	146 (111,187)	0.76 (0.58,0.97)	-0.58 (-0.72,-0.46)	10174 (7866,12976)	69.46 (54.41,88.07)	3466 (2628,4398)	19.44 (14.79,24.57)	-4.16 (-4.27,-4.05)
Iceland	7 (6,9)	2.47 (1.91,3.04)	3 (2,4)	0.50 (0.37,0.65)	-2.70 (-2.80,-2.60)	152 (118,187)	54.15 (41.82,66.53)	59 (44,76)	10.43 (7.84,13.36)	-5.11 (-5.19,-5.03)
India	3141 (2356,4239)	0.68 (0.51,0.93)	4159 (3107,5508)	0.36 (0.27,0.47)	-0.83 (-0.92,-0.72)	92666 (69882,1237	17.98 (13.50,24.22)	107766 (80807,1443	8.68 (6.52,11.60)	-2.05 (-2.15,-1.94)
Indonesia	738 (519,976)	0.77 (0.53,1.02)	1501 (1078,2073)	0.65 (0.48,0.90)	0.41 (0.38,0.44)	21757 (15260,2874	19.93 (13.97,26.30)	41855 (29889,57965	15.90 (11.43,21.93)	-0.52 (-0.54,-0.50)
Iran (Islamic	431 (266,564)	1.76 (1.04,2.31)	711 (410,903)	0.97 (0.55,1.25)	-0.16 (-0.24,-0.10)	11814 (7547,15375)	42.18 (26.20,55.14)	17401 (10584,21821	21.77 (12.85,27.37)	-1.91 (-1.98,-1.85)
Iraq	77 (54,113)	1.02 (0.71,1.49)	144 (94,196)	0.70 (0.46,0.94)	0.34 (0.26,0.43)	1993 (1405,2931)	24.66 (17.30,36.25)	3597 (2382,4922)	14.99 (9.82,20.35)	-1.17 (-1.25,-1.08)
Ireland	102 (81,125)	2.45 (1.95,3.00)	37 (28,47)	0.45 (0.34,0.56)	-2.86 (-3.04,-2.72)	2049 (1650,2484)	49.38 (39.93,59.67)	701 (559,862)	8.87 (7.12,10.82)	-5.42 (-5.54,-5.31)
Israel	66 (52,83)	1.37 (1.08,1.70)	59 (45,75)	0.46 (0.36,0.59)	-1.72 (-2.01,-1.42)	1441 (1156,1781)	29.91 (24.04,36.59)	1212 (958,1510)	10.18 (8.07,12.64)	-3.36 (-3.54,-3.17)
Italy	2385 (1847,3005)	2.64 (2.05,3.31)	1060 (790,1330)	0.69 (0.53,0.85)	-2.54 (-2.60,-2.48)	51597 (41162,6401	59.28 (47.63,73.25)	19994 (15520,24559	14.94 (11.86,18.23)	-4.29 (-4.35,-4.24)
Jamaica	22 (17,27)	1.19 (0.94,1.50)	17 (11,24)	0.55 (0.37,0.78)	-0.40 (-0.83,-0.00)	488 (389,605)	28.07 (22.47,34.81)	401 (266,575)	13.07 (8.66,18.72)	-2.72 (-3.19,-2.25)
Japan	8973 (7283,10840	5.30 (4.28,6.42)	5440 (4225,6938)	1.31 (1.04,1.62)	-1.60 (-1.66,-1.53)	210098 (172745,251	122.18 (100.6,146.07)	91630 (73016,11332	27.02 (21.99,32.52)	-4.45 (-4.50,-4.42)
Jordan	14 (10,19)	1.19 (0.87,1.64)	37 (26,50)	0.57 (0.39,0.76)	-0.93 (-0.98,-0.88)	384 (282,524)	27.52 (20.23,37.81)	964 (668,1301)	12.38 (8.56,16.62)	-2.32 (-2.38,-2.26)
Kazakhstan	405 (308,533)	3.05 (2.30,4.07)	160 (125,200)	0.84 (0.66,1.06)	-1.12 (-1.29,-0.92)	12390 (9630,15869)	89.88 (69.60,116.42)	4702 (3745,5793)	23.63 (18.79,29.24)	-3.95 (-4.09,-3.82)
Kenya	30 (20,44)	0.38 (0.25,0.55)	60 (44,81)	0.27 (0.20,0.37)	-0.33 (-0.37,-0.27)	815 (537,1177)	9.40 (6.23,13.71)	1699 (1243,2306)	6.74 (4.90,9.10)	-1.13 (-1.17,-1.09)
Kiribati	1 (1,1)	2.95 (2.17,3.76)	2 (1,3)	2.53 (1.78,3.34)	0.35 (0.32,0.37)	34 (25,44)	81.39 (60.61,105.28)	60 (41,80)	69.91 (48.83,92.78)	-0.50 (-0.52,-0.49)
Kuwait	2 (2,3)	0.44 (0.30,0.61)	7 (5,10)	0.30 (0.22,0.41)	0.25 (-0.25,0.78)	66 (48,91)	10.11 (7.07,14.02)	178 (129,243)	6.08 (4.33,8.19)	-1.04 (-1.67,-0.22)
Kyrgyzstan	119 (95,145)	3.93 (3.15,4.82)	104 (79,134)	2.15 (1.63,2.76)	-0.46 (-0.72,-0.23)	3503 (2808,4299)	112.22 (90.54,136.76)	3006 (2292,3859)	56.10 (42.60,72.16)	-2.04 (-2.34,-1.82)
Lao People's	41 (26,58)	2.00 (1.29,2.86)	40 (28,57)	0.95 (0.66,1.36)	-0.56 (-0.58,-0.55)	1148 (736,1641)	51.41 (33.05,73.35)	1087 (750,1547)	22.20 (15.55,31.49)	-2.37 (-2.39,-2.36)
Latvia	92 (73,114)	2.56 (2.04,3.19)	37 (28,48)	1.02 (0.77,1.31)	0.34 (0.06,0.60)	2594 (2081,3195)	73.80 (59.14,90.66)	948 (716,1208)	28.58 (21.59,36.25)	-3.02 (-3.20,-2.78)
Lebanon	29 (20,40)	1.43 (1.00,1.93)	54 (39,74)	0.87 (0.61,1.18)	-0.41 (-0.47,-0.35)	757 (523,1040)	33.78 (23.43,46.07)	1119 (804,1507)	18.76 (13.50,25.25)	-1.54 (-1.62,-1.47)
Lesotho	6 (4,9)	0.73 (0.44,1.06)	11 (7,15)	1.01 (0.63,1.39)	1.28 (1.15,1.38)	146 (86,212)	16.95 (10.13,24.36)	312 (187,430)	26.51 (16.02,36.59)	1.17 (1.09,1.25)
Liberia	3 (2,5)	0.30 (0.21,0.41)	5 (3,7)	0.21 (0.13,0.31)	-1.32 (-1.37,-1.27)	93 (65,129)	7.82 (5.45,10.83)	139 (84,205)	5.67 (3.4,8.22)	-1.04 (-1.13,-0.97)
Libya	15 (9,21)	0.82 (0.52,1.17)	30 (20,42)	0.63 (0.40,0.89)	0.38 (0.31,0.45)	371 (238,541)	19.11 (12.22,27.59)	807 (530,1128)	14.42 (9.39,20.26)	-0.82 (-0.90,-0.75)
Lithuania	108 (86,134)	2.40 (1.91,2.97)	52 (39,66)	0.94 (0.71,1.19)	-0.82 (-1.44,-0.34)	2907 (2347,3509)	65.33 (52.94,78.96)	1235 (936,1559)	25.09 (19.03,31.67)	-3.14 (-3.32,-3.03)
Luxembourg	9 (6,13)	1.70 (1.17,2.42)	5 (3,7)	0.45 (0.31,0.63)	-2.29 (-2.42,-2.21)	212 (150,298)	39.74 (28.49,55.29)	98 (70,135)	9.42 (6.73,13.05)	-4.25 (-4.34,-4.17)
Madagascar	18 (12,24)	0.37 (0.24,0.50)	15 (9,22)	0.14 (0.09,0.21)	-2.37 (-2.42,-2.32)	494 (320,679)	9.17 (6.12,62)	465 (283,682)	3.56 (2.22,5.22)	-2.94 (-2.99,-2.88)
Malawi	8 (6,10)	0.23 (0.17,0.31)	13 (8,17)	0.18 (0.12,0.25)	0.31 (0.26,0.34)	209 (151,273)	5.34 (3.83,7.02)	344 (227,479)	4.36 (2.93,6.01)	-0.78 (-0.82,-0.74)
Malaysia	75 (56,98)	0.86 (0.64,1.13)	121 (92,159)	0.45 (0.34,0.60)	-0.79 (-1.11,-0.47)	1911 (1462,2474)	20.03 (15.21,26.09)	3035 (2355,4064)	10.42 (8.09,13.89)	-2.26 (-2.47,-2.04)
Maldives	1 (1,1)	1.32 (0.87,1.95)	1 (1,2)	0.38 (0.27,0.53)	-1.74 (-1.82,-1.61)	27 (18,38)	29.52 (19.93,43.33)	26 (18,35)	7.49 (5.34,10.34)	-3.97 (-4.07,-3.82)
Mali	21 (15,27)	0.55 (0.41,0.75)	46 (32,64)	0.57 (0.40,0.78)	1.07 (1.04,1.11)	576 (419,748)	13.58 (9.99,17.79)	1230 (847,1727)	13.25 (9.27,18.48)	0.10 (0.06,0.13)
Malta	8 (6,10)	1.81 (1.36,2.32)	5 (4,6)	0.47 (0.36,0.61)	-2.69 (-2.94,-2.45)	179 (137,226)	41.44 (31.70,52.35)	98 (75,127)	10.91 (8.44,14.03)	-4.24 (-4.42,-4.02)
Marshall Isla	0 (0,0)	1.39 (0.93,2.00)	0 (0,1)	1.02 (0.65,1.44)	0.68 (0.59,0.78)	7 (5,10)	38.92 (25.99,55.03)	13 (8,18)	28.63 (18.69,40.50)	-0.95 (-1.01,-0.85)
Mauritania	4 (3,6)	0.41 (0.28,0.56)	5 (3,7)	0.23 (0.14,0.32)	-1.20 (-1.26,-1.16)	118 (83,162)	11.05 (7.74,15.33)	133 (85,183)	5.70 (3.62,7.95)	-1.88 (-1.95,-1.81)
Mauritius	9 (8,11)	1.27 (1.03,1.54)	14 (12,18)	0.78 (0.64,0.95)	-2.46 (-2.90,-2.04)	256 (211,307)	32.65 (26.93,39.59)	373 (304,452)	19.87 (16.20,23.92)	-1.30 (-1.58,-0.96)
Mexico	488 (399,594)	1.28 (1.03,1.57)	470 (363,591)	0.38 (0.29,0.48)	-2.61 (-2.70,-2.49)	11858 (9814,14101)	27.66 (22.81,33.44)	11680 (9127,14617)	8.97 (6.98,11.22)	-3.88 (-3.96,-3.80)
Micronesia (F	1 (1,1)	2.08 (1.50,2.83)	1 (1,2)	1.43 (0.98,2.02)	0.09 (0.08,0.10)	33 (23,45)	60.53 (43.19,83.13)	37 (25,52)	41.94 (28.09,58.74)	-1.20 (-1.22,-1.19)
Monaco	1 (1,2)	1.62 (1.01,2.37)	1 (1,1)	0.76 (0.50,1.12)	-0.63 (-0.64,-0.61)	24 (15,35)	37.13 (24.06,53.86)	15 (10,22)	16.97 (11.08,25.42)	-2.42 (-2.44,-2.40)
Mongolia	45 (33,63)	4.27 (3.11,5.96)	82 (59,113)	3.42 (2.44,4.49)	-1.11 (-1.18,-1.03)	1292 (926,1787)	116.95 (84.13,162.28)	2509 (1776,3455)	92.89 (66.29,126.46)	-0.77 (-0.89,-0.64)
Montenegro	10 (7,13)	1.53 (1.05,2.09)	12 (8,16)	1.16 (0.82,1.57)	0.49 (0.40,0.59)	249 (171,337)	38.79 (26.88,52.50)	271 (194,366)	27.15 (19.45,36.66)	-0.76 (-0.88,-0.63)
Morocco	30 (20,41)	0.22 (0.14,0.3.00)	40 (25,54)	0.12 (0.07,0.16)	-0.73 (-0.76,-0.70)	822 (540,1119)	5.51 (3.60,7.53)	1050 (674,1450)	2.88 (1.84,3.97)	-2.00 (-2.03,-1.97)
Mozambique	19 (12,25)	0.36 (0.24,0.48)	30 (20,42)	0.29 (0.19,0.40)	-0.14 (-0.17,-0.10)	492 (332,659)	8.12 (5.37,10.90)	837 (567,1173)	7.08 (4.64,9.75)	-0.60 (-0.65,-0.56)
Myanmar	463 (320,631)	2.08 (1.44,2.84)	267 (192,369)	0.58 (0.42,0.79)	-2.18 (-2.20,-2.16)	12874 (8893,17568)	52.43 (36.30,71.65)	6819 (4891,9459)	13.44 (9.65,18.51)	-4.06 (-4.08,-4.03)
Namibia	1 (1,2)	0.25 (0.18,0.34)	2 (1,3)	0.15 (0.11,0.20)	-0.43 (-0.47,-0.40)	37 (27,50)	5.69 (4.13,7.65)	48 (34,66)	3.45 (2.45,4.61)	-1.55 (-1.60,-1.49)

Nauru	0 (0,0)	2.32 (1.57,3.24)	0 (0,0)	1.51 (1.03,2.03)	-0.53 (-0.54,-0.52)	4 (3,5)	66.38 (45.15,92.28)	3 (2,4)	45.03 (30.78,61.49)	-1.38 (-1.40,-1.36)
Nepal	93 (62,128)	1.05 (0.70,1.44)	108 (72,151)	0.50 (0.33,0.69)	-1.13 (-1.16,-1.11)	2601 (1686,3595)	25.95 (17.14,35.67)	2604 (1753,3691)	11.03 (7.43,15.44)	-2.37 (-2.40,-2.35)
Netherlands	509 (403,639)	2.48 (1.97,3.11)	192 (147,243)	0.51 (0.40,0.64)	-2.34 (-2.45,-2.24)	10446 (8302,12995)	52.77 (42.12,65.35)	3552 (2778,4371)	10.29 (8.11,12.63)	-5.02 (-5.09,-4.95)
New Zealand	42 (34,52)	1.07 (0.85,1.31)	31 (24,39)	0.36 (0.29,0.45)	-1.84 (-1.96,-1.73)	980 (787,1184)	25.30 (20.36,30.41)	664 (534,821)	8.44 (6.84,10.39)	-3.53 (-3.69,-3.39)
Nicaragua	11 (8,14)	0.75 (0.57,0.97)	22 (16,30)	0.47 (0.34,0.64)	-0.51 (-0.70,-0.35)	305 (236,382)	18.57 (14.32,23.31)	596 (435,801)	11.55 (8.33,15.65)	-1.52 (-1.77,-1.32)
Niger	6 (4,8)	0.24 (0.16,0.34)	13 (8,18)	0.19 (0.12,0.26)	-0.72 (-0.75,-0.69)	168 (108,237)	5.66 (3.67,7.84)	338 (200,479)	4.02 (2.44,5.59)	-0.79 (-0.83,-0.75)
Nigeria	28 (19,44)	0.06 (0.04,0.10)	25 (17,37)	0.03 (0.02,0.04)	-1.49 (-1.51,-1.47)	761 (506,1203)	1.63 (1.09,2.58)	717 (484,1051)	0.70 (0.47,1.04)	-2.70 (-2.73,-2.67)
Niue	0 (0,0)	0.96 (0.66,1.40)	0 (0,0)	0.67 (0.47,0.94)	0.36 (0.34,0.37)	1 (0,1)	26.27 (17.98,37.95)	0 (0,1)	17.82 (12.62,24.85)	-1.14 (-1.16,-1.12)
North Maced	64 (45,89)	3.47 (2.45,4.89)	64 (44,87)	1.95 (1.36,2.66)	-0.12 (-0.28,0.01)	1660 (1202,2293)	85.03 (61.66,118.21)	1523 (1048,2061)	44.71 (31.05,60.37)	-1.77 (-1.90,-1.60)
Northern Mar	0 (0,0)	1.21 (0.86,1.70)	0 (0,1)	0.77 (0.58,1.00)	1.05 (0.99,1.12)	8 (5,11)	32.57 (23.06,45.57)	12 (9,16)	20.53 (15.67,26.66)	-1.45 (-1.51,-1.41)
Norway	93 (76,114)	1.33 (1.09,1.62)	23 (18,30)	0.23 (0.18,0.28)	-3.30 (-3.41,-3.22)	1954 (1611,2351)	30.72 (25.55,36.69)	475 (378,580)	5.10 (4.08,6.16)	-5.66 (-5.75,-5.57)
Oman	4 (3,7)	0.68 (0.43,1.01)	4 (3,6)	0.25 (0.17,0.34)	-1.10 (-1.25,-0.93)	126 (79,188)	16.82 (10.66,24.99)	120 (83,163)	5.41 (3.66,7.34)	-3.23 (-3.35,-3.12)
Pakistan	325 (224,446)	0.61 (0.42,0.83)	369 (256,518)	0.32 (0.22,0.45)	-0.68 (-0.70,-0.66)	8185 (5670,11436)	14.26 (9.85,19.82)	9999 (6831,14128)	7.75 (5.35,10.90)	-2.00 (-2.03,-1.96)
Palau	0 (0,0)	1.56 (1.11,2.24)	0 (0,0)	0.96 (0.67,1.33)	-0.82 (-0.86,-0.77)	4 (3,6)	40.93 (29.05,58.33)	7 (5,9)	26.18 (18.25,35.94)	-1.57 (-1.61,-1.53)
Palestine	12 (8,18)	1.57 (0.99,2.23)	16 (11,22)	0.72 (0.47,1.01)	-0.90 (-0.98,-0.83)	284 (179,407)	33.25 (20.97,47.61)	392 (269,533)	15.38 (10.38,21.10)	-2.49 (-2.58,-2.41)
Panama	13 (10,16)	0.92 (0.73,1.16)	18 (13,24)	0.41 (0.30,0.55)	-1.25 (-1.43,-1.07)	306 (250,375)	20.44 (16.66,25.09)	406 (294,547)	9.21 (6.67,12.41)	-2.45 (-2.60,-2.24)
Papua New G	21 (13,31)	1.08 (0.67,1.57)	38 (25,57)	0.69 (0.45,0.99)	-0.23 (-0.29,-0.17)	691 (406,1013)	31.22 (18.65,45.63)	1293 (827,1936)	19.77 (12.81,29.11)	-1.47 (-1.52,-1.42)
Paraguay	31 (23,41)	1.50 (1.10,2.00)	46 (31,64)	0.82 (0.55,1.16)	-0.21 (-0.36,-0.07)	717 (541,939)	32.34 (24.34,42.48)	1051 (715,1469)	17.93 (12.10,25.04)	-1.76 (-1.88,-1.65)
Peru	110 (82,148)	0.96 (0.71,1.29)	212 (144,291)	0.64 (0.43,0.88)	-0.67 (-0.88,-0.51)	2813 (2052,3761)	22.86 (16.73,30.58)	5091 (3410,7045)	15.00 (10.07,20.72)	-1.21 (-1.48,-1.02)
Philippines	200 (152,260)	0.73 (0.55,0.94)	324 (239,458)	0.40 (0.30,0.57)	-0.75 (-0.82,-0.67)	5969 (4564,7676)	18.12 (13.73,23.48)	9154 (6776,12928)	10.20 (7.56,14.41)	-1.88 (-1.95,-1.82)
Poland	1340 (1096,1614)	3.05 (2.49,3.66)	647 (516,804)	0.90 (0.72,1.11)	-0.25 (-0.34,-0.16)	34380 (28525,4077	78.04 (64.81,92.29)	15052 (12111,18422	22.28 (18.06,27.10)	-3.98 (-4.08,-3.87)
Portugal	365 (279,472)	2.61 (2.00,3.35)	163 (127,204)	0.71 (0.56,0.87)	-1.52 (-1.65,-1.41)	8845 (6887,11146)	64.86 (50.78,81.10)	3765 (3021,4610)	18.69 (15.15,22.64)	-4.10 (-4.19,-3.98)
Puerto Rico	31 (22,44)	0.85 (0.59,1.21)	18 (12,27)	0.24 (0.17,0.36)	-1.33 (-1.52,-1.14)	673 (489,932)	18.51 (13.41,25.59)	364 (254,535)	5.76 (4.05,8.34)	-3.95 (-4.10,-3.78)
Qatar	1 (1,2)	1.48 (0.94,2.26)	3 (2,4)	0.37 (0.23,0.55)	-2.26 (-2.58,-1.94)	35 (23,54)	32.24 (20.43,50.13)	85 (55,122)	7.76 (4.80,11.48)	-4.37 (-4.68,-4.05)
Republic of K	2767 (1930,3553)	9.77 (7.04,12.57)	1668 (1220,2261)	1.78 (1.30,2.40)	-1.87 (-1.93,-1.82)	76289 (51415,9767	237.53 (164.91,305.41)	32423 (24279,43153	34.86 (26.25,46.05)	-5.36 (-5.42,-5.32)
Republic of M	121 (82,168)	2.65 (1.80,3.64)	74 (54,98)	1.21 (0.90,1.60)	0.06 (-0.18,0.29)	3486 (2386,4829)	74.31 (51.08,102.33)	1959 (1448,2578)	32.86 (24.39,42.91)	-2.57 (-2.81,-2.32)
Romania	476 (374,587)	1.67 (1.31,2.07)	355 (269,454)	1.00 (0.77,1.27)	0.36 (0.23,0.50)	13501 (10824,1639	46.98 (37.88,57.07)	9037 (6998,11317)	27.35 (21.18,33.96)	-1.59 (-1.73,-1.44)
Russian Fede	5927 (4956,6950)	3.20 (2.67,3.75)	3225 (2622,3921)	1.34 (1.08,1.61)	-0.56 (-0.66,-0.45)	173786 (146355,20	93.74 (78.78,108.71)	84086 (68441,10165	35.89 (29.32,43.07)	-2.68 (-2.81,-2.53)
Rwanda	25 (16,34)	0.94 (0.60,1.25)	30 (20,41)	0.53 (0.36,0.73)	-0.56 (-0.59,-0.54)	685 (436,928)	23.01 (14.79,30.81)	768 (521,1054)	11.88 (8.13,16.24)	-1.81 (-1.85,-1.78)
Saint Kitts an	0 (0,0)	0.71 (0.52,0.95)	0 (0,0)	0.32 (0.23,0.43)	-0.78 (-0.86,-0.69)	6 (5,8)	16.83 (12.74,21.62)	5 (4,7)	7.21 (5.06,9.66)	-2.51 (-2.61,-2.38)
Saint Lucia	1 (1,1)	1.28 (1.00,1.62)	1 (1,2)	0.52 (0.38,0.69)	-1.52 (-1.73,-1.34)	27 (22,33)	31.24 (25.03,38.58)	30 (23,41)	12.44 (9.23,16.57)	-2.81 (-2.91,-2.72)
Saint Vincent	1 (0,1)	0.78 (0.61,0.98)	1 (0,1)	0.44 (0.33,0.57)	-0.10 (-0.29,0.03)	14 (11,17)	20.22 (16.09,24.98)	16 (12,21)	11.22 (8.51,14.41)	-1.90 (-2.02,-1.79)
Samoa	1 (1,1)	1.32 (0.80,1.78)	1 (1,2)	0.89 (0.54,1.18)	-0.55 (-0.56,-0.53)	30 (18,40)	33.23 (20.46,44.66)	34 (21,45)	22.50 (13.93,29.54)	-1.26 (-1.28,-1.25)
San Marino	1 (1,2)	3.59 (2.30,5.23)	1 (0,1)	0.98 (0.56,1.63)	-3.49 (-3.97,-3.17)	27 (18,39)	76.75 (52.07,110.95)	15 (8,24)	20.92 (11.88,34.65)	-4.50 (-4.94,-4.27)
Sao Tome an	0 (0,0)	0.31 (0.22,0.41)	0 (0,0)	0.29 (0.20,0.40)	1.22 (1.15,1.30)	5 (4,7)	7.93 (5.66,10.73)	9 (6,12)	7.45 (5.29,10.13)	-0.10 (-0.14,-0.06)
Saudi Arabia	23 (14,38)	0.42 (0.25,0.67)	43 (28,76)	0.24 (0.16,0.39)	-0.19 (-0.22,-0.17)	644 (385,1038)	10.29 (6.15,16.74)	1362 (873,2466)	5.72 (3.76,9.97)	-1.87 (-1.89,-1.85)
Senegal	15 (9,20)	0.44 (0.28,0.60)	19 (12,27)	0.24 (0.15,0.33)	-1.45 (-1.56,-1.34)	447 (288,607)	12.55 (8.05,17.17)	556 (355,780)	6.45 (4.07,9.07)	-2.13 (-2.26,-2.00)
Serbia	168 (123,215)	1.55 (1.11,2.02)	145 (104,197)	0.88 (0.64,1.19)	-0.36 (-0.46,-0.25)	4561 (3360,5803)	39.10 (28.99,49.97)	3464 (2477,4672)	22.43 (16.23,30.39)	-1.71 (-1.84,-1.56)
Seychelles	1 (0,1)	1.19 (0.83,1.62)	1 (0,1)	0.59 (0.44,0.80)	-1.32 (-1.61,-1.14)	17 (12,23)	30.34 (21.66,40.75)	17 (13,23)	14.23 (10.51,18.96)	-2.28 (-2.45,-2.11)
Sierra Leone	10 (7,14)	0.51 (0.35,0.69)	12 (8,16)	0.31 (0.20,0.43)	-1.65 (-1.68,-1.62)	287 (191,390)	13.54 (9.02,18.32)	343 (222,477)	8.26 (5.35,11.52)	-1.58 (-1.62,-1.55)
Singapore	31 (23,41)	1.47 (1.07,1.93)	19 (14,24)	0.22 (0.17,0.29)	-3.03 (-3.41,-2.76)	792 (595,1035)	34.58 (25.64,45.20)	409 (319,511)	4.70 (3.66,5.91)	-5.85 (-5.98,-5.72)
Slovakia	117 (83,158)	1.95 (1.39,2.61)	69 (48,94)	0.72 (0.50,0.97)	-1.11 (-1.19,-1.05)	3022 (2221,3990)	50.87 (37.51,67.42)	1667 (1168,2226)	17.98 (12.72,23.88)	-3.18 (-3.26,-3.11)
Slovenia	57 (45,72)	2.30 (1.82,2.89)	33 (24,44)	0.74 (0.55,0.98)	-0.53 (-0.74,-0.37)	1501 (1186,1870)	60.74 (48.19,75.25)	722 (541,954)	17.64 (13.25,23.18)	-3.65 (-3.79,-3.54)
Solomon Isla	3 (2,5)	2.35 (1.45,3.25)	6 (4,9)	1.69 (1.17,2.36)	0.06 (0.01,0.09)	105 (60,149)	65.40 (39.26,91.39)	206 (142,292)	48.69 (33.65,68.67)	-1.09 (-1.15,-1.04)
Somalia	14 (9,21)	0.60 (0.36,0.87)	24 (14,36)	0.39 (0.23,0.56)	-1.06 (-1.09,-1.04)	447 (265,662)	15.85 (9.59,23.30)	770 (442,1131)	10.45 (6.04,15.29)	-1.38 (-1.40,-1.36)
South Africa	144 (103,180)	0.69 (0.49,0.87)	154 (118,190)	0.32 (0.24,0.40)	-1.56 (-1.66,-1.47)	4344 (3115,5333)	19.04 (13.57,23.60)	4603 (3554,5648)	9.00 (6.92,11.06)	-2.45 (-2.61,-2.31)
South Sudan	13 (7,20)	0.51 (0.29,0.80)	13 (7,19)	0.34 (0.20,0.50)	-0.77 (-0.80,-0.74)	349 (196,550)	13.04 (7.30,20.58)	379 (205,561)	8.66 (4.79,12.74)	-1.28 (-1.31,-1.26)
Spain	1440 (1145,1788)	2.60 (2.07,3.22)	664 (515,845)	0.66 (0.52,0.83)	-2.22 (-2.28,-2.17)	32176 (25697,3987	60.30 (48.35,74.13)	13584 (10816,16732	15.33 (12.3,18.64)	-4.28 (-4.32,-4.25)
Sri Lanka	68 (50,89)	0.68 (0.49,0.89)	49 (28,74)	0.18 (0.11,0.27)	-1.99 (-2.20,-1.80)	1826 (1315,2358)	16.13 (11.68,20.84)	1241 (708,1826)	4.45 (2.57,6.54)	-4.23 (-4.51,-4.02)
Sudan	150 (69,231)	1.70 (0.77,2.59)	205 (74,329)	1.18 (0.41,1.86)	-0.71 (-0.73,-0.70)	3918 (1853,5978)	40.60 (19.01,62.03)	5110 (1910,8210)	25.85 (9.42,41.43)	-1.18 (-1.19,-1.17)
Suriname	2 (2,3)	0.94 (0.68,1.26)	3 (2,5)	0.47 (0.31,0.71)	-0.75 (-0.92,-0.57)	66 (47,87)	24.45 (17.65,32.20)	84 (56,125)	12.58 (8.35,18.77)	-1.87 (-2.02,-1.68)
Sweden	191 (147,240)	1.18 (0.92,1.47)	83 (61,106)	0.35 (0.26,0.44)	-1.35 (-1.46,-1.26)	3715 (2910,4600)	25.24 (20.05,31.09)	1483 (1124,1887)	7.04 (5.38,8.97)	-3.88 (-4.00,-3.80)
Switzerland	179 (139,227)	1.66 (1.29,2.09)	87 (66,113)	0.44 (0.34,0.57)	-0.25 (-0.47,-0.07)	3689 (2899,4625)	36.57 (28.98,45.59)	1590 (1245,2031)	9.13 (7.22,11.63)	-4.26 (-4.37,-4.18)
Syrian Arab	47 (33,66)	0.97 (0.67,1.37)	66 (45,93)	0.58 (0.39,0.80)	-0.38 (-0.48,-0.29)	1248 (873,1729)	22.89 (15.96,31.88)	1669 (1113,2332)	12.30 (8.27,17.23)	-1.76 (-1.84,-1.67)
Taiwan (Prov	468 (347,603)	3.14 (2.36,4.03)	447 (344,567)	1.03 (0.79,1.30)	-1.18 (-1.37,-0.98)	11732 (8797,15017)	71.44 (53.57,91.83)	8745 (6815,10943)	20.82 (16.26,26.05)	-3.52 (-3.66,-3.38)

Tajikistan	112 (75,157)	4.07 (2.75,5.64)	65 (43,95)	1.19 (0.79,1.76)	-2.12 (-2.26,-1.97)	3128 (2034,4426)	108.13 (70.95,151.99)	1804 (1181,2666)	28.60 (19.20,42.42)	-4.01 (-4.14,-3.89)
Thailand	489 (292,666)	1.45 (0.88,1.99)	655 (320,962)	0.60 (0.29,0.88)	-1.80 (-1.90,-1.69)	13422 (7896,18418)	35.39 (21.02,48.23)	16967 (8088,25084)	15.79 (7.48,23.33)	-2.79 (-3.06,-2.58)
Timor-Leste	2 (1,4)	0.92 (0.52,1.40)	5 (3,8)	0.62 (0.41,0.93)	-0.24 (-0.28,-0.20)	75 (40,112)	23.09 (12.88,35.28)	132 (86,203)	15.09 (9.89,23.05)	-1.30 (-1.37,-1.23)
Togo	8 (5,11)	0.69 (0.46,1.02)	16 (10,24)	0.44 (0.27,0.64)	-1.57 (-1.59,-1.54)	216 (149,313)	16.70 (11.28,24.36)	472 (283,683)	11.11 (6.73,16.06)	-1.42 (-1.45,-1.38)
Tokelau	0 (0,0)	1.23 (0.83,1.76)	0 (0,0)	0.64 (0.47,0.88)	-0.54 (-0.56,-0.51)	0 (0,1)	32.14 (21.71,45.36)	0 (0,0)	17.28 (12.48,24.42)	-2.06 (-2.08,-2.04)
Tonga	1 (1,2)	2.54 (1.81,3.68)	1 (1,2)	1.65 (1.18,2.31)	-0.07 (-0.14,0.01)	34 (25,49)	60.14 (43.22,85.67)	32 (23,45)	39.48 (27.82,55.38)	-1.40 (-1.50,-1.31)
Trinidad and	6 (5,8)	0.77 (0.61,0.97)	5 (4,7)	0.28 (0.19,0.38)	-1.01 (-1.27,-0.73)	164 (132,201)	19.16 (15.43,23.58)	143 (97,197)	7.36 (5.04,10.19)	-2.99 (-3.22,-2.73)
Tunisia	44 (30,61)	0.98 (0.68,1.37)	70 (44,98)	0.55 (0.35,0.77)	-0.45 (-0.50,-0.40)	1039 (705,1427)	20.76 (14.14,28.68)	1569 (975,2211)	11.67 (7.30,16.37)	-1.89 (-1.93,-1.83)
Turkey	1360 (941,1782)	4.04 (2.79,5.34)	1297 (904,1804)	1.40 (0.98,1.93)	-1.32 (-1.37,-1.26)	37650 (25510,4943	101.90 (69.92,134.18)	31910 (21761,44614	33.06 (22.68,45.92)	-3.35 (-3.40,-3.29)
Turkmenistan	54 (44,67)	2.78 (2.24,3.42)	33 (24,46)	0.82 (0.58,1.13)	-1.46 (-1.66,-1.26)	1587 (1285,1943)	76.32 (61.71,93.61)	953 (672,1325)	21.6 (15.28,29.74)	-3.89 (-4.08,-3.67)
Tuvalu	0 (0,0)	1.73 (1.24,2.39)	0 (0,0)	1.06 (0.78,1.43)	0.16 (0.15,0.16)	4 (3,5)	48.51 (34.43,67.54)	3 (2,4)	29.76 (22.05,39.91)	-1.57 (-1.58,-1.56)
Uganda	19 (13,26)	0.30 (0.21,0.41)	24 (17,34)	0.17 (0.12,0.24)	-0.67 (-0.71,-0.63)	517 (361,702)	7.59 (5.32,10.45)	699 (496,984)	4.29 (3.06,6.03)	-1.86 (-1.91,-1.81)
Ukraine	2363 (1899,2937)	3.27 (2.65,4.06)	751 (481,1092)	0.99 (0.64,1.44)	-0.51 (-0.75,-0.24)	66889 (54666,8216	94.40 (77.40,115.72)	20497 (13074,29942	28.39 (18.02,41.41)	-3.87 (-4.18,-3.66)
United Arab I	4 (3,6)	1.07 (0.70,1.55)	10 (7,14)	0.43 (0.29,0.61)	-1.22 (-1.66,-0.76)	128 (81,189)	24.68 (15.96,36.19)	334 (227,468)	7.91 (5.5,11.21)	-3.08 (-3.43,-2.79)
United Kingd	2056 (1641,2498)	2.15 (1.73,2.61)	721 (550,920)	0.51 (0.39,0.64)	-2.64 (-2.69,-2.59)	41005 (33249,4936	45.53 (36.99,54.70)	12810 (10044,15699	10.01 (7.92,12.20)	-4.58 (-4.62,-4.54)
United Repub	66 (43,88)	0.62 (0.41,0.84)	74 (48,103)	0.29 (0.19,0.41)	-1.56 (-1.59,-1.54)	1832 (1188,2481)	15.78 (10.30,21.37)	2110 (1357,3025)	7.46 (4.81,10.54)	-2.39 (-2.41,-2.37)
United States	2974 (2384,3577)	0.92 (0.74,1.10)	2065 (1598,2578)	0.35 (0.27,0.43)	-0.97 (-1.02,-0.94)	68153 (55168,8123	22.18 (17.95,26.38)	44971 (35992,55115	8.09 (6.52,9.85)	-3.13 (-3.21,-3.08)
United States	1 (0,1)	0.75 (0.44,1.17)	1 (0,1)	0.29 (0.17,0.43)	-1.84 (-1.97,-1.73)	16 (10,23)	17.48 (10.84,26.95)	12 (7,17)	6.60 (4.05,9.67)	-3.09 (-3.23,-2.96)
Uruguay	64 (51,78)	1.64 (1.31,2.00)	53 (41,66)	1.00 (0.78,1.23)	-0.90 (-1.01,-0.78)	1617 (1311,1928)	42.79 (34.76,50.75)	1261 (996,1554)	25.53 (20.32,30.94)	-1.63 (-1.73,-1.51)
Uzbekistan	104 (80,131)	0.88 (0.68,1.11)	121 (89,157)	0.44 (0.32,0.57)	1.29 (1.14,1.44)	3246 (2565,4094)	26.54 (20.84,33.37)	3743 (2776,4795)	12.26 (9.05,15.84)	-2.26 (-2.43,-2.11)
Vanuatu	1 (1,1)	1.43 (0.95,2.03)	1 (1,2)	0.81 (0.57,1.13)	-0.75 (-0.78,-0.71)	28 (18,40)	38.62 (25.78,55.06)	45 (31,65)	22.19 (15.41,31.50)	-1.82 (-1.86,-1.77)
Venezuela (B	136 (110,164)	1.46 (1.17,1.76)	158 (109,219)	0.53 (0.37,0.74)	-1.40 (-1.57,-1.23)	3610 (2945,4311)	35.84 (29.08,42.84)	4090 (2796,5586)	13.15 (9.04,18.00)	-3.27 (-3.46,-3.09)
Viet Nam	707 (451,938)	1.76 (1.14,2.33)	827 (595,1171)	0.82 (0.59,1.18)	0.18 (0.16,0.20)	18845 (11738,2520	45.44 (28.54,60.87)	23250 (16725,32373	21.42 (15.36,29.92)	-2.43 (-2.46,-2.41)
Yemen	136 (60,209)	2.84 (1.25,4.36)	286 (105,423)	2.22 (0.81,3.29)	0.12 (0.08,0.17)	3901 (1735,5940)	72.85 (32.18,111.89)	7520 (2767,11162)	51.04 (18.69,75.41)	-0.76 (-0.79,-0.73)
Zambia	12 (8,16)	0.46 (0.31,0.59)	18 (12,24)	0.27 (0.18,0.38)	-0.05 (-0.09,-0.02)	328 (219,434)	11.10 (7.45,14.67)	502 (323,692)	6.69 (4.38,9.29)	-1.62 (-1.66,-1.59)
Zimbabwe	33 (24,46)	0.89 (0.64,1.22)	53 (39,71)	0.80 (0.58,1.06)	0.05 (-0.02,0.11)	877 (621,1214)	20.87 (14.88,28.75)	1552 (1115,2121)	20.16 (14.50,26.98)	-0.26 (-0.31,-0.20)
Afghanistan	15 (4,30)	0.21 (0.06,0.43)	25 (7,48)	0.25 (0.07,0.46)	0.49 (0.45,0.52)	410 (118,815)	5.52 (1.60,11.08)	818 (244,1575)	6.75 (2.00,13.07)	0.66 (0.62,0.69)
Albania	30 (9,51)	1.49 (0.45,2.54)	51 (17,96)	1.15 (0.39,2.14)	-0.89 (-1.04,-0.77)	765 (230,1311)	35.58 (10.70,60.93)	1197 (398,2263)	27.50 (9.16,51.82)	-0.91 (-1.09,-0.77)
Algeria	16 (5,28)	0.14 (0.04,0.24)	60 (19,106)	0.17 (0.05,0.31)	0.76 (0.73,0.80)	451 (146,777)	3.43 (1.11,5.99)	1621 (515,2892)	4.23 (1.35,7.49)	0.66 (0.63,0.71)
American Sar	0 (0,0)	0.47 (0.15,0.86)	0 (0,1)	0.67 (0.21,1.18)	1.14 (1.02,1.25)	4 (1,7)	13.96 (4.47,25.61)	11 (4,19)	19.86 (6.44,35.27)	1.19 (1.08,1.30)
Andorra	1 (0,1)	1.24 (0.41,2.26)	1 (0,3)	0.96 (0.30,1.75)	-0.81 (-0.86,-0.76)	20 (7,36)	32.91 (11.09,60.31)	37 (11,69)	24.75 (7.57,45.58)	-0.92 (-0.96,-0.88)
Angola	49 (5,155)	1.18 (0.10,3.85)	77 (12,203)	0.60 (0.09,1.61)	-2.15 (-2.21,-2.07)	1538 (144,4855)	33.02 (3.03,105.48)	2443 (387,6460)	16.79 (2.65,44.54)	-2.14 (-2.19,-2.09)
Antigua and I	0 (0,0)	0.23 (0.07,0.42)	0 (0,0)	0.20 (0.06,0.37)	-0.38 (-0.68,-0.10)	3 (1,5)	5.93 (1.80,10.57)	6 (2,10)	4.93 (1.52,8.81)	-0.59 (-0.90,-0.31)
Argentina	24 (8,40)	0.07 (0.02,0.12)	71 (22,123)	0.13 (0.04,0.22)	1.89 (1.74,2.03)	674 (225,1139)	2.05 (0.69,3.46)	1881 (601,3220)	3.52 (1.12,6.00)	1.78 (1.62,1.93)
Armenia	23 (8,40)	0.84 (0.29,1.44)	34 (12,59)	0.78 (0.28,1.33)	-0.17 (-0.48,0.21)	660 (225,1126)	22.37 (7.67,38.29)	895 (327,1524)	20.63 (7.55,34.99)	-0.21 (-0.51,0.15)
Australia	41 (13,70)	0.21 (0.07,0.36)	164 (52,295)	0.39 (0.13,0.70)	2.03 (1.95,2.10)	1139 (381,1927)	5.99 (2.01,10.12)	4171 (1374,7342)	10.75 (3.57,18.83)	1.95 (1.86,2.04)
Austria	36 (11,62)	0.31 (0.10,0.54)	92 (28,159)	0.54 (0.17,0.92)	1.66 (1.52,1.78)	920 (299,1577)	8.65 (2.83,14.66)	2203 (689,3745)	13.86 (4.39,23.36)	1.42 (1.29,1.53)
Azerbaijan	31 (10,59)	0.59 (0.18,1.1)	95 (31,197)	0.89 (0.29,1.83)	1.31 (1.20,1.40)	947 (298,1825)	17.07 (5.36,32.89)	2763 (911,5759)	23.51 (7.70,48.76)	1.07 (0.98,1.15)
Bahamas	0 (0,1)	0.26 (0.08,0.49)	1 (0,2)	0.22 (0.07,0.40)	-0.51 (-0.71,-0.33)	12 (4,21)	7.09 (2.17,12.86)	25 (7,47)	5.70 (1.69,10.53)	-0.57 (-0.75,-0.33)
Bahrain	1 (0,2)	0.78 (0.25,1.38)	3 (1,6)	0.43 (0.14,0.77)	-1.93 (-2.07,-1.81)	35 (11,61)	18.27 (5.82,32.37)	99 (31,182)	9.61 (3.13,16.99)	-2.01 (-2.13,-1.91)
Bangladesh	168 (55,290)	0.36 (0.12,0.62)	441 (152,781)	0.32 (0.11,0.56)	-0.29 (-0.39,-0.19)	4812 (1560,8305)	9.66 (3.12,16.77)	11606 (3907,21153)	8.06 (2.73,14.55)	-0.49 (-0.58,-0.41)
Barbados	0 (0,1)	0.12 (0.04,0.23)	1 (0,1)	0.12 (0.04,0.23)	-0.08 (-0.24,0.09)	8 (2,16)	3.14 (0.90,5.79)	14 (4,27)	2.83 (0.86,5.39)	-0.33 (-0.47,-0.18)
Belarus	40 (14,66)	0.31 (0.11,0.50)	60 (20,103)	0.37 (0.13,0.64)	0.64 (0.49,0.82)	1184 (420,1952)	9.03 (3.2,14.83)	1660 (564,2822)	10.65 (3.63,18.18)	0.49 (0.30,0.71)
Belgium	55 (18,93)	0.35 (0.12,0.60)	90 (26,159)	0.40 (0.12,0.71)	0.41 (0.26,0.61)	1295 (435,2173)	8.84 (2.98,14.85)	2070 (625,3627)	10.19 (3.12,17.61)	0.52 (0.39,0.65)
Belize	0 (0,0)	0.15 (0.05,0.28)	1 (0,1)	0.18 (0.06,0.33)	0.55 (0.34,0.73)	4 (1,6)	3.91 (1.23,6.91)	15 (5,26)	4.66 (1.51,8.24)	0.52 (0.35,0.68)
Benin	21 (6,40)	1.01 (0.29,2.00)	19 (5,36)	0.36 (0.10,0.66)	-3.28 (-3.35,-3.22)	605 (172,1192)	28.36 (8.08,55.6)	583 (154,1091)	9.73 (2.66,18.16)	-3.38 (-3.46,-3.32)
Bermuda	0 (0,0)	0.28 (0.09,0.50)	0 (0,0)	0.14 (0.04,0.25)	-2.47 (-2.66,-2.32)	4 (1,8)	6.97 (2.24,12.45)	4 (1,8)	3.37 (1.05,6.03)	-2.54 (-2.75,-2.37)
Bhutan	1 (0,1)	0.25 (0.07,0.51)	2 (0,3)	0.27 (0.08,0.51)	0.27 (0.24,0.31)	19 (5,40)	6.69 (1.76,14.11)	42 (12,81)	6.70 (1.92,12.91)	0.01 (-0.02,0.04)
Bolivia (Pluri	7 (2,13)	0.21 (0.06,0.40)	19 (6,37)	0.21 (0.07,0.42)	0.10 (0.03,0.16)	181 (56,344)	5.36 (1.65,10.24)	486 (154,935)	5.12 (1.6,9.92)	-0.14 (-0.20,-0.08)
Bosnia and H	33 (11,55)	0.78 (0.25,1.31)	48 (15,85)	0.76 (0.24,1.33)	-0.13 (-0.25,-0.02)	969 (312,1590)	21.27 (6.86,35.1)	1185 (378,2055)	19.51 (6.27,33.76)	-0.33 (-0.46,-0.20)
Botswana	3 (1,6)	0.53 (0.13,1.11)	8 (2,24)	0.55 (0.13,1.48)	0.15 (0.05,0.21)	89 (20,195)	14.13 (3.32,30.37)	260 (57,763)	14.84 (3.34,42.25)	0.11 (0.03,0.18)
Brazil	218 (75,357)	0.24 (0.08,0.40)	468 (146,818)	0.18 (0.06,0.32)	-0.89 (-1.03,-0.79)	6279 (2174,10204)	6.45 (2.23,10.54)	11989 (3779,20565)	4.65 (1.46,7.99)	-1.05 (-1.17,-0.94)
Brunei Darus	2 (1,3)	1.86 (0.58,3.29)	3 (1,6)	0.84 (0.27,1.55)	-2.59 (-2.69,-2.52)	59 (18,105)	50.50 (15.53,88.6)	94 (31,169)	22.54 (7.51,40.72)	-2.63 (-2.74,-2.56)
Bulgaria	115 (37,198)	0.91 (0.29,1.55)	51 (15,92)	0.40 (0.12,0.72)	-2.71 (-2.94,-2.46)	3374 (1094,5748)	26.94 (8.76,45.84)	1428 (450,2561)	12.14 (3.90,21.64)	-2.55 (-2.83,-2.24)
Burkina Faso	60 (15,138)	1.31 (0.33,2.99)	69 (17,145)	0.68 (0.17,1.43)	-2.09 (-2.14,-2.05)	1918 (489,4414)	39.15 (9.98,90.00)	2268 (567,4840)	20.20 (5.03,42.30)	-2.11 (-2.16,-2.07)

Burundi	6 (1,19)	0.25 (0.06,0.79)	5 (1,15)	0.10 (0.02,0.29)	-3.10 (-3.13,-3.06)	177 (44,554)	7.03 (1.75,22.18)	150 (30,438)	2.61 (0.54,7.74)	-3.17 (-3.21,-3.12)
Cabo Verde	1 (0,2)	0.41 (0.12,0.82)	2 (1,4)	0.44 (0.12,0.86)	0.19 (0.05,0.36)	25 (7,49)	12.31 (3.61,23.59)	61 (17,118)	12.48 (3.51,24.09)	-0.03 (-0.14,0.10)
Cambodia	49 (14,138)	1.08 (0.29,3.03)	96 (23,249)	0.76 (0.18,1.99)	-1.11 (-1.14,-1.09)	1485 (404,4330)	29.44 (8.03,83.55)	2773 (639,7099)	20.14 (4.71,51.55)	-1.22 (-1.25,-1.20)
Cameroon	49 (14,102)	0.99 (0.28,2.02)	67 (18,158)	0.47 (0.13,1.12)	-2.37 (-2.42,-2.32)	1656 (476,3569)	30.67 (8.74,64.49)	2289 (603,5313)	14.14 (3.76,32.85)	-2.47 (-2.52,-2.42)
Canada	85 (29,146)	0.26 (0.09,0.45)	318 (96,556)	0.44 (0.13,0.77)	1.76 (1.63,1.93)	2241 (762,3798)	7.06 (2.40,11.97)	7347 (2235,12733)	10.94 (3.38,18.8)	1.54 (1.41,1.65)
Central Afric	10 (2,22)	0.84 (0.20,1.84)	9 (2,20)	0.36 (0.09,0.84)	-2.83 (-2.90,-2.76)	323 (75,669)	23.78 (5.52,51.00)	288 (73,658)	10.16 (2.53,23.51)	-2.80 (-2.87,-2.73)
Chad	30 (8,70)	1.05 (0.27,2.43)	38 (11,80)	0.63 (0.18,1.33)	-1.68 (-1.77,-1.59)	879 (233,2034)	29.19 (7.65,67.62)	1161 (326,2493)	17.02 (4.96,36.43)	-1.75 (-1.84,-1.66)
Chile	18 (6,32)	0.18 (0.06,0.31)	74 (23,132)	0.29 (0.09,0.51)	1.77 (1.59,1.90)	520 (168,906)	4.95 (1.59,8.65)	1888 (606,3255)	7.41 (2.38,12.75)	1.53 (1.34,1.67)
China	14380 (5025,2364	1.57 (0.54,2.57)	23714 (7911,4177	1.11 (0.37,1.96)	-1.08 (-1.15,-1.02)	488411 (171930,80	49.96 (17.56,82.51)	712311 (239926,125	33.75 (11.37,59.41)	-1.23 (-1.29,-1.18)
Colombia	37 (12,65)	0.21 (0.07,0.37)	64 (21,117)	0.12 (0.04,0.21)	-2.19 (-2.43,-1.87)	1056 (352,1836)	5.56 (1.84,9.71)	1503 (487,2734)	2.72 (0.88,4.94)	-2.58 (-2.82,-2.24)
Comoros	1 (0,2)	0.38 (0.10,0.87)	1 (0,2)	0.23 (0.06,0.51)	-1.61 (-1.69,-1.52)	20 (5,48)	9.46 (2.33,22.03)	31 (8,69)	5.85 (1.46,12.93)	-1.61 (-1.71,-1.51)
Congo	8 (2,17)	0.71 (0.20,1.54)	10 (3,23)	0.37 (0.09,0.82)	-2.06 (-2.11,-2.01)	233 (67,497)	19.51 (5.54,41.59)	321 (77,711)	9.90 (2.46,22.06)	-2.20 (-2.28,-2.12)
Cook Islands	0 (0,0)	1.10 (0.35,1.88)	0 (0,1)	1.09 (0.34,1.97)	-0.06 (-0.15,0.01)	4 (1,8)	31.87 (9.97,54.20)	8 (2,15)	31.56 (9.95,58.13)	-0.06 (-0.16,0.02)
Costa Rica	9 (3,15)	0.51 (0.16,0.88)	26 (8,48)	0.48 (0.14,0.88)	-0.14 (-0.41,0.11)	217 (72,372)	12.19 (4.02,20.91)	607 (178,1098)	10.96 (3.23,19.83)	-0.19 (-0.44,0.10)
Croatia	20 (7,35)	0.33 (0.11,0.57)	33 (11,58)	0.38 (0.13,0.65)	-2.59 (-2.64,-2.55)	573 (181,981)	9.08 (2.90,15.54)	778 (261,1336)	9.50 (3.21,16.30)	-2.61 (-2.68,-2.56)
Cuba	27 (9,45)	0.26 (0.09,0.44)	31 (11,54)	0.16 (0.05,0.27)	0.36 (0.16,0.58)	713 (252,1198)	6.98 (2.46,11.70)	825 (281,1408)	4.33 (1.50,7.47)	0.10 (-0.12,0.32)
Cyprus	3 (1,6)	0.43 (0.13,0.77)	8 (2,14)	0.38 (0.11,0.67)	-1.58 (-1.73,-1.42)	84 (27,146)	10.35 (3.36,18.09)	183 (55,319)	9.03 (2.71,15.59)	-1.47 (-1.63,-1.31)
Czechia	89 (29,150)	0.64 (0.21,1.08)	64 (21,115)	0.30 (0.10,0.53)	-0.38 (-0.44,-0.30)	2399 (811,3994)	17.78 (6.03,29.56)	1513 (494,2669)	7.65 (2.53,13.44)	-0.46 (-0.50,-0.40)
C么te d'Ivoir	18 (5,35)	0.41 (0.11,0.78)	23 (5,47)	0.18 (0.04,0.38)	-2.25 (-2.40,-2.08)	618 (168,1179)	12.01 (3.23,22.76)	761 (175,1525)	5.30 (1.24,10.77)	-2.54 (-2.68,-2.37)
Democratic P	233 (67,466)	1.23 (0.35,2.44)	253 (80,479)	0.72 (0.23,1.38)	-1.73 (-1.76,-1.70)	8223 (2323,16411)	41.44 (11.80,82.68)	8538 (2728,16393)	24.16 (7.71,46.07)	-1.74 (-1.77,-1.71)
Democratic R	34 (7,104)	0.21 (0.04,0.63)	46 (9,132)	0.12 (0.02,0.34)	-1.86 (-1.89,-1.82)	1017 (216,3148)	5.71 (1.21,17.38)	1424 (270,4051)	3.27 (0.61,9.32)	-1.81 (-1.85,-1.77)
Denmark	21 (7,35)	0.26 (0.09,0.43)	46 (14,81)	0.38 (0.12,0.65)	1.14 (0.94,1.34)	493 (168,809)	6.70 (2.29,10.94)	972 (308,1684)	8.61 (2.77,14.85)	0.86 (0.69,1.05)
Djibouti	0 (0,1)	0.31 (0.08,0.73)	2 (1,5)	0.37 (0.09,0.76)	0.65 (0.60,0.69)	14 (3,34)	8.28 (2.01,19.69)	71 (18,153)	9.40 (2.36,19.74)	0.40 (0.36,0.45)
Dominica	0 (0,0)	0.21 (0.06,0.39)	0 (0,0)	0.26 (0.07,0.50)	0.80 (0.69,0.91)	3 (1,6)	5.13 (1.49,9.57)	5 (1,11)	6.33 (1.73,12.29)	0.71 (0.61,0.81)
Dominican R	6 (2,10)	0.16 (0.05,0.28)	19 (6,34)	0.19 (0.06,0.35)	0.77 (0.68,0.85)	147 (46,270)	3.81 (1.19,6.91)	486 (145,871)	4.79 (1.43,8.56)	0.82 (0.75,0.89)
Ecuador	16 (5,29)	0.32 (0.10,0.56)	28 (9,52)	0.17 (0.05,0.32)	-2.16 (-2.48,-1.86)	431 (143,759)	7.82 (2.61,13.74)	650 (206,1180)	3.93 (1.24,7.13)	-2.40 (-2.73,-2.09)
Egypt	376 (125,700)	1.41 (0.47,2.63)	1383 (415,2453)	2.20 (0.65,3.84)	1.43 (1.24,1.57)	10883 (3597,20406)	36.15 (12.12,67.51)	39496 (11845,69857	55.11 (16.5,97.1)	1.43 (1.28,1.55)
El Salvador	3 (1,5)	0.09 (0.03,0.17)	5 (2,9)	0.09 (0.03,0.16)	-0.24 (-0.39,-0.10)	75 (23,134)	2.46 (0.77,4.43)	138 (40,251)	2.30 (0.67,4.18)	-0.16 (-0.32,-0.02)
Equatorial Gu	0 (0,1)	0.16 (0.03,0.69)	2 (0,4)	0.31 (0.08,0.72)	2.04 (1.97,2.11)	10 (2,43)	4.54 (0.83,19.26)	50 (13,110)	8.22 (2.25,19.01)	1.89 (1.81,1.98)
Eritrea	2 (0,4)	0.11 (0.03,0.28)	2 (1,6)	0.07 (0.02,0.17)	-1.52 (-1.59,-1.46)	59 (14,142)	3.60 (0.83,8.84)	85 (18,212)	2.18 (0.48,5.44)	-1.57 (-1.64,-1.50)
Estonia	8 (3,13)	0.37 (0.13,0.62)	10 (3,18)	0.42 (0.13,0.73)	0.58 (0.18,0.95)	225 (78,377)	11.21 (3.86,18.74)	259 (79,447)	11.51 (3.55,19.84)	0.39 (0.11,0.73)
Eswatini	2 (0,4)	0.57 (0.12,1.27)	6 (1,15)	0.97 (0.18,2.56)	1.75 (1.64,1.86)	46 (8,108)	14.41 (2.69,33.32)	172 (31,476)	26.11 (4.83,70.8)	1.94 (1.82,2.06)
Ethiopia	14 (4,32)	0.07 (0.02,0.15)	17 (5,37)	0.04 (0.01,0.09)	-1.67 (-1.69,-1.64)	425 (113,973)	1.86 (0.50,4.22)	501 (131,1084)	1.05 (0.27,2.24)	-1.85 (-1.88,-1.83)
Fiji	2 (1,4)	0.52 (0.17,0.96)	4 (1,7)	0.46 (0.15,0.88)	-0.43 (-0.54,-0.34)	67 (22,121)	15.40 (4.94,27.85)	120 (39,222)	13.38 (4.39,25.00)	-0.50 (-0.61,-0.40)
Finland	20 (6,35)	0.29 (0.09,0.50)	44 (13,83)	0.36 (0.11,0.67)	0.63 (0.53,0.71)	532 (175,906)	7.84 (2.60,13.26)	974 (292,1820)	8.85 (2.71,16.26)	0.29 (0.16,0.38)
France	536 (179,910)	0.67 (0.23,1.14)	890 (273,1585)	0.69 (0.22,1.22)	0.05 (-0.06,0.14)	13459 (4583,22751)	17.68 (6.05,29.91)	20739 (6652,36792)	17.70 (5.83,31.70)	-0.03 (-0.13,0.06)
Gabon	3 (1,8)	0.55 (0.14,1.40)	6 (2,12)	0.54 (0.14,1.10)	-0.04 (-0.08,-0.01)	94 (23,237)	15.49 (3.86,39.07)	181 (44,382)	14.84 (3.71,30.87)	-0.14 (-0.18,-0.09)
Gambia	11 (3,19)	2.73 (0.84,4.91)	23 (7,45)	2.07 (0.64,4.10)	-0.83 (-0.97,-0.66)	366 (112,671)	85.16 (26.39,154.48)	757 (231,1535)	63.43 (19.25,127.52)	-0.89 (-1.07,-0.67)
Georgia	35 (12,58)	0.53 (0.19,0.90)	32 (12,55)	0.57 (0.20,0.96)	0.42 (-0.09,0.87)	1044 (377,1737)	15.94 (5.75,26.73)	911 (323,1535)	16.59 (5.87,27.89)	0.29 (-0.22,0.74)
Germany	432 (141,744)	0.34 (0.11,0.58)	844 (257,1506)	0.46 (0.14,0.80)	0.94 (0.87,1.00)	10449 (3457,17576)	8.71 (2.90,14.64)	18929 (5904,32700)	11.32 (3.6,19.28)	0.88 (0.80,0.95)
Ghana	28 (8,55)	0.48 (0.14,0.93)	52 (15,110)	0.34 (0.10,0.72)	-1.12 (-1.18,-1.07)	849 (241,1631)	12.14 (3.46,23.14)	1470 (398,3105)	7.96 (2.22,16.9)	-1.31 (-1.37,-1.26)
Greece	39 (13,65)	0.26 (0.09,0.43)	125 (42,210)	0.57 (0.20,0.95)	2.54 (2.33,2.73)	994 (336,1631)	6.89 (2.34,11.26)	2883 (984,4775)	15.27 (5.21,25.28)	2.58 (2.38,2.80)
Greenland	0 (0,1)	0.90 (0.28,1.58)	1 (0,1)	0.87 (0.29,1.58)	-0.08 (-0.16,-0.01)	11 (3,19)	25.89 (8.07,44.62)	18 (6,33)	22.75 (7.75,40.88)	-0.37 (-0.45,-0.30)
Grenada	0 (0,0)	0.16 (0.05,0.30)	0 (0,1)	0.28 (0.08,0.51)	2.14 (1.81,2.50)	3 (1,5)	4.28 (1.28,8.10)	8 (3,14)	6.65 (2.09,11.76)	1.47 (1.19,1.79)
Guam	0 (0,0)	0.32 (0.11,0.55)	1 (0,2)	0.59 (0.20,1.04)	1.91 (1.79,2.02)	9 (3,15)	9.57 (3.14,16.08)	39 (13,67)	18.71 (6.36,32.33)	2.15 (2.05,2.25)
Guatemala	16 (5,28)	0.49 (0.15,0.86)	31 (10,55)	0.29 (0.09,0.52)	-1.46 (-1.99,-0.80)	421 (136,740)	11.65 (3.71,20.34)	763 (244,1369)	6.79 (2.18,12.21)	-1.87 (-2.41,-1.36)
Guinea	45 (14,82)	1.33 (0.41,2.43)	54 (15,112)	0.91 (0.26,1.87)	-1.21 (-1.26,-1.16)	1354 (426,2450)	38.22 (11.85,69.55)	1671 (484,3417)	25.93 (7.46,53.41)	-1.25 (-1.30,-1.20)
Guinea-Bissa	5 (1,12)	1.29 (0.29,2.79)	6 (2,11)	0.71 (0.19,1.38)	-1.97 (-2.09,-1.86)	175 (41,379)	38.06 (8.75,82.68)	203 (57,402)	21.10 (5.87,41.05)	-1.96 (-2.07,-1.85)
Guyana	1 (0,1)	0.20 (0.06,0.37)	1 (0,2)	0.16 (0.05,0.29)	-0.70 (-0.90,-0.52)	23 (7,41)	5.51 (1.70,9.95)	31 (10,56)	4.34 (1.37,7.99)	-0.65 (-0.87,-0.44)
Haiti	3 (1,7)	0.10 (0.02,0.24)	5 (1,11)	0.07 (0.01,0.17)	-1.16 (-1.20,-1.13)	91 (24,210)	2.56 (0.66,6.04)	126 (29,313)	1.64 (0.37,4.08)	-1.43 (-1.48,-1.40)
Honduras	3 (1,6)	0.14 (0.04,0.31)	15 (4,29)	0.24 (0.07,0.47)	1.70 (1.64,1.77)	76 (22,161)	3.55 (1.01,7.53)	372 (108,738)	5.68 (1.63,11.2)	1.54 (1.48,1.61)
Hungary	50 (17,82)	0.34 (0.12,0.56)	35 (11,63)	0.19 (0.06,0.35)	-1.88 (-2.11,-1.68)	1415 (488,2347)	10.05 (3.45,16.67)	899 (290,1627)	5.25 (1.68,9.45)	-2.15 (-2.40,-1.94)
Iceland	1 (0,1)	0.30 (0.10,0.50)	2 (1,4)	0.39 (0.12,0.70)	0.84 (0.70,0.99)	21 (7,35)	7.73 (2.52,12.81)	51 (16,91)	9.21 (2.89,16.43)	0.68 (0.56,0.82)
India	1054 (360,1803)	0.22 (0.07,0.37)	2531 (786,4356)	0.21 (0.07,0.36)	-0.12 (-0.21,-0.05)	31796 (10908,53939	6.03 (2.06,10.29)	69092 (21539,11879	5.45 (1.70,9.38)	-0.30 (-0.38,-0.23)
Indonesia	374 (118,803)	0.36 (0.11,0.78)	1165 (404,2599)	0.46 (0.16,1.03)	0.79 (0.76,0.82)	11752 (3721,25145)	10.23 (3.23,21.92)	35488 (12297,78711	12.63 (4.37,28.13)	0.68 (0.66,0.71)

Iran (Islamic	36 (11,66)	0.13 (0.04,0.25)	145 (47,247)	0.19 (0.06,0.32)	1.23 (1.06,1.39)	1068 (325,1934)	3.58 (1.08,6.48)	4016 (1304,6876)	4.75 (1.53,8.08)	1.06 (0.91,1.21)
Iraq	40 (12,70)	0.51 (0.15,0.91)	122 (38,224)	0.54 (0.17,1.00)	0.14 (0.06,0.22)	1139 (332,2000)	13.68 (3.97,24.06)	3321 (1000,6071)	12.97 (4,23.71)	-0.20 (-0.27,-0.12)
Ireland	11 (4,19)	0.27 (0.09,0.45)	25 (8,45)	0.32 (0.09,0.56)	0.35 (0.20,0.48)	256 (83,434)	6.36 (2.09,10.76)	562 (171,997)	7.32 (2.24,12.97)	0.32 (0.15,0.47)
Israel	12 (4,21)	0.25 (0.08,0.42)	26 (8,46)	0.21 (0.06,0.38)	-0.63 (-0.77,-0.50)	292 (98,491)	6.13 (2.04,10.36)	610 (183,1062)	5.31 (1.59,9.17)	-0.60 (-0.74,-0.47)
Italy	790 (263,1302)	0.89 (0.30,1.46)	596 (188,1023)	0.44 (0.14,0.74)	-2.23 (-2.48,-1.93)	19879 (6692,32625)	23.21 (7.83,38.08)	13329 (4259,22412)	10.89 (3.53,18.24)	-2.36 (-2.75,-1.97)
Jamaica	2 (1,3)	0.10 (0.03,0.18)	4 (1,7)	0.12 (0.04,0.23)	0.63 (0.15,1.09)	43 (13,80)	2.55 (0.80,4.69)	97 (29,182)	3.17 (0.94,5.92)	0.57 (0.05,1.09)
Japan	3315 (1189,5328)	1.90 (0.68,3.05)	2525 (804,4455)	0.68 (0.22,1.17)	-3.23 (-3.38,-3.09)	93599 (33794,1495	53.50 (19.32,85.53)	46873 (14970,80539	15.39 (5.00,25.87)	-3.93 (-4.08,-3.80)
Jordan	3 (1,7)	0.26 (0.08,0.48)	14 (4,24)	0.18 (0.05,0.32)	-1.11 (-1.18,-1.05)	103 (33,195)	6.79 (2.12,12.93)	393 (115,684)	4.56 (1.30,8.01)	-1.25 (-1.33,-1.18)
Kazakhstan	151 (52,253)	1.10 (0.38,1.85)	74 (25,126)	0.38 (0.13,0.66)	-3.45 (-3.61,-3.29)	4883 (1697,8108)	34.54 (11.95,57.69)	2232 (761,3747)	11.07 (3.74,18.69)	-3.67 (-3.83,-3.49)
Kenya	13 (3,25)	0.16 (0.04,0.31)	47 (13,87)	0.20 (0.06,0.37)	0.72 (0.68,0.77)	371 (98,728)	4.14 (1.08,8.14)	1403 (399,2564)	5.31 (1.52,9.74)	0.79 (0.76,0.83)
Kiribati	0 (0,1)	0.98 (0.35,1.68)	1 (0,1)	0.86 (0.29,1.51)	-0.43 (-0.47,-0.39)	13 (4,22)	29.48 (10.40,50.26)	23 (8,41)	26.05 (8.72,46.03)	-0.42 (-0.46,-0.38)
Kuwait	3 (1,5)	0.42 (0.13,0.71)	3 (1,5)	0.10 (0.03,0.17)	-4.89 (-5.75,-4.03)	93 (30,157)	11.56 (3.72,19.33)	76 (26,136)	2.20 (0.71,3.98)	-5.44 (-6.27,-4.61)
Kyrgyzstan	19 (6,32)	0.60 (0.20,1.03)	20 (7,34)	0.39 (0.14,0.66)	-1.48 (-2.07,-0.88)	609 (210,1037)	19.16 (6.59,32.70)	602 (216,1017)	10.88 (3.93,18.28)	-1.87 (-2.43,-1.32)
Lao People's	31 (10,56)	1.44 (0.47,2.55)	46 (14,83)	0.96 (0.29,1.71)	-1.30 (-1.33,-1.27)	965 (314,1755)	41.20 (13.45,74.77)	1381 (407,2490)	25.99 (7.68,46.82)	-1.48 (-1.51,-1.46)
Latvia	11 (4,19)	0.32 (0.11,0.54)	13 (4,22)	0.36 (0.12,0.61)	0.50 (0.12,0.85)	345 (120,566)	9.87 (3.44,16.20)	343 (117,568)	10.44 (3.61,17.27)	0.26 (-0.11,0.57)
Lebanon	9 (3,16)	0.38 (0.12,0.72)	18 (6,32)	0.31 (0.10,0.54)	-0.69 (-0.75,-0.64)	246 (76,462)	10.37 (3.21,19.69)	480 (151,849)	8.40(2.65,14.89)	-0.68 (-0.78,-0.62)
Lesotho	4 (1,9)	0.47 (0.09,1.07)	19 (3,65)	1.67 (0.28,5.52)	4.21 (4.12,4.29)	116 (20,267)	12.81 (2.24,29.42)	641 (101,2161)	50.49 (8.21,168.76)	4.56 (4.46,4.64)
Liberia	16 (4,32)	1.37 (0.40,2.76)	19 (5,37)	0.79 (0.20,1.53)	-1.79 (-1.86,-1.72)	478 (131,943)	39.09 (10.77,76.48)	618 (179,1213)	22.36 (6.02,43.74)	-1.81 (-1.89,-1.75)
Libya	8 (2,15)	0.40 (0.13,0.75)	33 (9,61)	0.58 (0.16,1.10)	1.22 (1.14,1.28)	240 (74,460)	11.29 (3.51,21.35)	1038 (295,1878)	16.09 (4.55,29.75)	1.15 (1.07,1.23)
Lithuania	10 (3,16)	0.22 (0.07,0.37)	20 (7,34)	0.38 (0.13,0.65)	1.97 (1.71,2.27)	288 (97,476)	6.51 (2.19,10.75)	524 (176,895)	10.84 (3.65,18.42)	1.69 (1.46,1.95)
Luxembourg	2 (1,4)	0.42 (0.14,0.71)	4 (1,8)	0.43 (0.13,0.74)	0.07 (-0.03,0.18)	58 (19,97)	10.96 (3.65,18.25)	104 (32,179)	10.24 (3.17,17.66)	-0.22 (-0.33,-0.11)
Madagascar	8 (2,18)	0.16 (0.04,0.35)	9 (2,21)	0.08 (0.02,0.18)	-2.31 (-2.39,-2.25)	253 (67,536)	4.48 (1.20,9.51)	283 (67,670)	2.07 (0.49,4.83)	-2.51 (-2.57,-2.45)
Malawi	11 (3,22)	0.29 (0.08,0.58)	29 (9,57)	0.39 (0.12,0.76)	0.99 (0.90,1.07)	303 (80,609)	7.30 (1.96,14.61)	867 (247,1670)	10.38 (3.03,19.96)	1.08 (0.99,1.16)
Malaysia	50 (16,87)	0.53 (0.17,0.92)	165 (49,287)	0.57 (0.17,1.00)	0.43 (0.25,0.61)	1467 (476,2600)	14.28 (4.64,25.3)	4797 (1399,8442)	15.65 (4.55,27.38)	0.30 (0.13,0.48)
Maldives	1 (0,2)	1.25 (0.46,2.18)	2 (1,4)	0.78 (0.25,1.38)	-1.54 (-1.62,-1.48)	30 (11,54)	30.87 (11.02,54.49)	61 (19,110)	17.13 (5.32,31.17)	-1.89 (-1.93,-1.85)
Mali	53 (15,97)	1.32 (0.38,2.38)	143 (41,264)	1.66 (0.48,3.07)	0.73 (0.68,0.77)	1604 (461,2880)	35.85 (10.32,64.36)	4262 (1202,7888)	42.86 (12.28,79.53)	0.57 (0.52,0.61)
Malta	1 (0,2)	0.22 (0.07,0.38)	2 (1,4)	0.26 (0.08,0.47)	0.53 (0.39,0.66)	25 (8,42)	5.71 (1.90,9.66)	58 (18,102)	6.72 (2.05,11.79)	0.55 (0.39,0.68)
Marshall Isla	0 (0,0)	0.25 (0.07,0.62)	0 (0,0)	0.31 (0.08,0.73)	0.64 (0.55,0.73)	1 (0,3)	7.49 (2.00,18.25)	4 (1,10)	9.20 (2.49,22.20)	0.64 (0.57,0.70)
Mauritania	29 (4,70)	2.72 (0.38,6.64)	23 (6,48)	1.02 (0.26,2.11)	-3.11 (-3.17,-3.06)	932 (125,2250)	83.4 (11.44,199.67)	715 (175,1502)	28.94 (7.16,59.89)	-3.39 (-3.44,-3.33)
Mauritius	3 (1,5)	0.43 (0.15,0.72)	1 (0,2)	0.07 (0.02,0.12)	-4.81 (-5.89,-3.10)	95 (33,157)	11.9 (4.09,19.71)	38 (13,63)	1.94 (0.68,3.28)	-4.82 (-5.91,-3.06)
Mexico	89 (30,148)	0.22 (0.07,0.37)	250 (85,426)	0.20 (0.07,0.35)	-0.29 (-0.39,-0.17)	2234 (762,3705)	5.14 (1.75,8.58)	5974 (1991,10109)	4.63 (1.55,7.83)	-0.32 (-0.43,-0.20)
Micronesia (F	0 (0,1)	0.67 (0.21,1.30)	1 (0,1)	0.66 (0.20,1.35)	-0.08 (-0.12,-0.02)	11 (3,22)	20.93 (6.44,40.10)	19 (5,40)	20.57 (5.98,42.35)	-0.08 (-0.13,-0.03)
Monaco	0 (0,1)	0.47 (0.14,0.86)	1 (0,1)	0.85 (0.24,1.54)	1.91 (1.87,1.95)	7 (2,13)	12.39 (3.78,22.33)	18 (5,32)	21.93 (6.20,40.56)	1.86 (1.82,1.90)
Mongolia	68 (21,126)	6.30 (1.94,11.65)	193 (59,335)	7.67 (2.35,13.4)	0.61 (0.51,0.71)	2027 (650,3698)	181.40 (57.74,330.46)	5961 (1829,10398)	214.7 (65.67,373.23)	0.53 (0.44,0.62)
Montenegro	5 (2,8)	0.76 (0.25,1.32)	8 (3,16)	0.83 (0.27,1.54)	0.29 (0.18,0.38)	141 (48,246)	21.52 (7.21,37.38)	219 (73,410)	22.15 (7.39,41.45)	0.13 (0.01,0.21)
Morocco	5 (2,9)	0.03 (0.01,0.06)	13 (4,25)	0.04 (0.01,0.07)	0.12 (0.08,0.16)	158 (49,286)	1.02 (0.32,1.87)	395 (112,742)	1.04 (0.29,1.94)	0.03 (0.01,0.06)
Mozambique	66 (19,133)	1.16 (0.34,2.36)	146 (32,389)	1.36 (0.31,3.65)	0.51 (0.44,0.56)	1806 (524,3641)	28.38 (8.32,57.28)	4216 (924,11540)	34.29 (7.53,91.80)	0.60 (0.55,0.64)
Myanmar	139 (37,330)	0.59 (0.16,1.39)	170 (49,446)	0.35 (0.10,0.90)	-1.74 (-1.77,-1.71)	4069 (1107,9800)	16.07 (4.35,38.20)	4699 (1360,12535)	8.92 (2.57,23.59)	-1.91 (-1.94,-1.87)
Namibia	1 (0,3)	0.22 (0.07,0.44)	3 (1,6)	0.25 (0.08,0.46)	0.43 (0.36,0.53)	32 (9,69)	4.97 (1.41,10.28)	84 (26,156)	5.85 (1.77,10.87)	0.55 (0.47,0.65)
Nauru	0 (0,0)	0.91 (0.29,1.58)	0 (0,0)	0.67 (0.21,1.26)	-1.01 (-1.06,-0.96)	2 (1,3)	29.22 (9.47,51.86)	2 (1,3)	22.93 (7.05,42.91)	-0.83 (-0.88,-0.79)
Nepal	24 (8,44)	0.26 (0.09,0.48)	81 (24,151)	0.35 (0.11,0.66)	0.96 (0.94,0.99)	680 (221,1250)	6.71 (2.18,12.22)	2054 (609,3792)	8.47 (2.51,15.63)	0.77 (0.74,0.79)
Netherlands	39 (13,64)	0.20 (0.07,0.33)	114 (35,203)	0.32 (0.10,0.58)	1.51 (1.39,1.61)	982 (330,1606)	5.19 (1.75,8.44)	2516 (788,4474)	7.68 (2.41,13.53)	1.25 (1.13,1.34)
New Zealand	11 (4,19)	0.29 (0.1,0.49)	37 (12,66)	0.45 (0.14,0.81)	1.47 (1.31,1.66)	313 (105,524)	8.39 (2.84,14.04)	937 (306,1633)	12.35 (4.07,21.39)	1.29 (1.13,1.50)
Nicaragua	3 (1,6)	0.22 (0.07,0.41)	10 (3,18)	0.20 (0.06,0.36)	-0.57 (-0.84,-0.36)	91 (31,170)	5.63 (1.87,10.39)	254 (82,466)	4.96 (1.59,9.13)	-0.66 (-0.93,-0.45)
Niger	19 (5,43)	0.67 (0.18,1.48)	20 (5,41)	0.25 (0.07,0.52)	-3.24 (-3.32,-3.16)	619 (172,1395)	18.47 (5.13,40.78)	603 (159,1282)	6.34 (1.69,13.22)	-3.45 (-3.52,-3.38)
Nigeria	55 (15,124)	0.12 (0.03,0.28)	64 (17,121)	0.07 (0.02,0.13)	-2.00 (-2.04,-1.95)	1631 (426,3593)	3.38 (0.89,7.46)	1931 (500,3624)	1.79 (0.47,3.39)	-2.06 (-2.10,-2.01)
Niue	0 (0,0)	0.42 (0.13,0.82)	0 (0,0)	0.48 (0.14,0.99)	0.38 (0.34,0.43)	0 (0,0)	12.58 (3.88,23.99)	0 (0,1)	14.02 (4.23,29.18)	0.31 (0.26,0.36)
North Maced	24 (8,40)	1.23 (0.41,2.08)	34 (11,62)	1.04 (0.33,1.85)	-0.53 (-0.62,-0.43)	680 (240,1129)	33.65 (11.73,56.02)	904 (290,1619)	26.48 (8.52,47.41)	-0.77 (-0.86,-0.64)
Northern Mar	0 (0,0)	0.57 (0.18,1.02)	0 (0,1)	0.72 (0.23,1.29)	0.67 (0.54,0.83)	4 (1,8)	16.82 (5.36,30.06)	13 (4,23)	20.07 (6.66,36.69)	0.53 (0.40,0.69)
Norway	13 (4,22)	0.21 (0.07,0.35)	24 (7,42)	0.26 (0.08,0.44)	0.65 (0.48,0.79)	326 (111,546)	5.66 (1.94,9.37)	594 (188,1023)	6.94 (2.21,11.90)	0.66 (0.48,0.80)
Oman	2 (0,3)	0.21 (0.07,0.44)	5 (1,9)	0.21 (0.06,0.41)	-0.06 (-0.14,0.02)	50 (16,108)	6.13 (1.95,12.97)	155 (45,294)	5.71 (1.58,11.08)	-0.30 (-0.41,-0.18)
Pakistan	194 (64,352)	0.34 (0.11,0.62)	359 (116,649)	0.29 (0.09,0.52)	-0.57 (-0.61,-0.54)	5450 (1796,9793)	9.14 (3.01,16.34)	10601 (3384,19026)	7.70 (2.48,13.86)	-0.54 (-0.57,-0.51)
Palau	0 (0,0)	0.88 (0.26,1.65)	0 (0,0)	0.83 (0.26,1.54)	-0.30 (-0.38,-0.23)	3 (1,5)	25.96 (7.52,49.50)	7 (2,13)	25.27 (7.79,47.16)	-0.11 (-0.18,-0.04)
Palestine	6 (2,10)	0.68 (0.21,1.24)	13 (4,23)	0.54 (0.17,0.91)	-0.74 (-0.82,-0.64)	144 (46,269)	16.24 (5.16,30.04)	370 (120,640)	13.15 (4.21,22.58)	-0.64 (-0.71,-0.56)
Panama	4 (1,7)	0.29 (0.09,0.50)	8 (2,15)	0.17 (0.05,0.33)	-1.66 (-1.79,-1.50)	98 (30,171)	6.59 (2.03,11.51)	173 (53,323)	3.92 (1.21,7.33)	-1.69 (-1.80,-1.56)

Papua New G	7 (1,22)	0.35 (0.07,1.06)	12 (3,37)	0.20 (0.05,0.60)	-1.77 (-1.81,-1.74)	237 (49,728)	10.56 (2.18,32.34)	415 (91,1293)	6.21 (1.37,18.92)	-1.74 (-1.77,-1.70)
Paraguay	7 (2,12)	0.31 (0.10,0.54)	22 (7,40)	0.38 (0.11,0.70)	0.74 (0.66,0.84)	167 (54,296)	7.41 (2.40,13.18)	547 (163,1001)	9.16 (2.72,16.73)	0.74 (0.65,0.84)
Peru	11 (3,21)	0.10 (0.03,0.18)	42 (12,81)	0.13 (0.04,0.25)	0.96 (0.76,1.12)	304 (88,562)	2.44 (0.71,4.51)	1008 (284,1994)	2.99 (0.84,5.89)	0.74 (0.53,0.90)
Philippines	373 (120,650)	1.15 (0.37,1.99)	672 (232,1140)	0.77 (0.27,1.32)	-1.24 (-1.28,-1.19)	12717 (4136,21518)	34.83 (11.25,60.31)	20745 (7153,34998)	22.05 (7.62,37.45)	-1.43 (-1.48,-1.38)
Poland	30 (10,50)	0.07 (0.02,0.11)	145 (48,248)	0.21 (0.07,0.36)	3.54 (3.31,3.71)	829 (291,1363)	1.90 (0.66,3.11)	3733 (1258,6381)	5.69 (1.94,9.71)	3.46 (3.24,3.62)
Portugal	27 (9,47)	0.20 (0.07,0.34)	96 (29,171)	0.46 (0.14,0.79)	2.72 (2.48,2.95)	748 (253,1275)	5.60 (1.90,9.52)	2547 (799,4337)	13.29 (4.24,22.49)	2.93 (2.69,3.16)
Puerto Rico	9 (3,16)	0.24 (0.08,0.44)	17 (6,30)	0.25 (0.08,0.46)	0.12 (-0.19,0.41)	229 (74,407)	6.35 (2.05,11.23)	400 (131,722)	6.63 (2.16,11.99)	0.16 (-0.15,0.45)
Qatar	1 (0,2)	0.76 (0.23,1.44)	8 (2,15)	0.96 (0.27,1.86)	0.69 (0.46,0.93)	29 (9,56)	19.26 (5.65,36.22)	265 (78,500)	22.41 (6.24,43.27)	0.46 (0.26,0.65)
Republic of K	1729 (592,2947)	5.32 (1.80,9.06)	1603 (488,2737)	1.71 (0.53,2.91)	-3.60 (-3.67,-3.55)	55861 (19338,9676	156.34 (53.8,267.64)	41202 (13254,70048	44.9 (14.50,76.33)	-3.95 (-4.03,-3.89)
Republic of M	13 (5,23)	0.29 (0.10,0.50)	17 (6,30)	0.29 (0.10,0.50)	-0.02 (-0.56,0.67)	405 (138,678)	8.62 (2.96,14.39)	495 (165,836)	8.65 (2.90,14.58)	-0.02 (-0.54,0.61)
Romania	45 (15,79)	0.16 (0.05,0.27)	118 (38,206)	0.34 (0.11,0.59)	2.63 (2.44,2.86)	1310 (453,2242)	4.53 (1.57,7.71)	3181 (1049,5498)	9.83 (3.28,16.94)	2.63 (2.43,2.88)
Russian Fede	340 (118,549)	0.18 (0.06,0.29)	702 (244,1156)	0.30 (0.10,0.49)	1.56 (1.27,1.85)	10290 (3629,16619)	5.54 (1.96,8.94)	19993 (7009,32682)	8.78 (3.09,14.33)	1.61 (1.32,1.90)
Rwanda	16 (4,33)	0.60 (0.17,1.21)	25 (7,48)	0.42 (0.13,0.81)	-1.34 (-1.51,-1.15)	452 (121,933)	14.96 (4.16,30.88)	654 (192,1289)	9.81 (2.90,19.33)	-1.60 (-1.80,-1.39)
Saint Kitts an	0 (0,0)	0.21 (0.06,0.39)	0 (0,0)	0.21 (0.07,0.40)	0.12 (-0.25,0.54)	2 (1,4)	5.51 (1.58,10.07)	4 (1,7)	5.06 (1.62,9.66)	-0.21 (-0.56,0.20)
Saint Lucia	0 (0,0)	0.19 (0.06,0.34)	0 (0,1)	0.12 (0.04,0.22)	-1.50 (-1.63,-1.35)	4 (1,8)	4.96 (1.57,8.94)	8 (2,14)	3.17 (0.99,5.67)	-1.41 (-1.55,-1.26)
Saint Vincent	0 (0,0)	0.26 (0.08,0.45)	0 (0,1)	0.22 (0.07,0.39)	-0.60 (-0.80,-0.41)	5 (1,8)	6.73 (2.01,11.81)	8 (3,15)	5.69 (1.78,10.12)	-0.60 (-0.79,-0.42)
Samoa	1 (0,1)	0.63 (0.19,1.14)	1 (0,1)	0.49 (0.15,0.91)	-0.89 (-0.98,-0.80)	17 (5,31)	18.01 (5.39,32.77)	23 (7,42)	14.14 (4.27,26.14)	-0.82 (-0.91,-0.74)
San Marino	0 (0,0)	0.25 (0.08,0.44)	0 (0,0)	0.23 (0.07,0.48)	-0.27 (-0.30,-0.24)	2 (1,4)	6.37 (2.04,11.01)	4 (1,8)	5.72 (1.75,11.81)	-0.34 (-0.37,-0.32)
Sao Tome an	0 (0,0)	0.12 (0.03,0.22)	0 (0,0)	0.10 (0.03,0.24)	-0.73 (-0.87,-0.64)	2 (1,4)	3.26 (0.97,6.23)	4 (1,9)	2.67 (0.76,6.64)	-0.74 (-0.89,-0.65)
Saudi Arabia	22 (6,45)	0.35 (0.10,0.72)	68 (20,128)	0.36 (0.11,0.68)	0.06 (0.02,0.10)	702 (208,1397)	9.98 (2.91,20.34)	2117 (625,3914)	9.03 (2.62,17.07)	-0.32 (-0.38,-0.27)
Senegal	35 (9,69)	0.98 (0.27,1.96)	32 (9,61)	0.37 (0.10,0.73)	-3.12 (-3.28,-2.94)	1155 (305,2294)	30.94 (8.19,61.57)	1037 (273,2010)	11.29 (3.01,21.55)	-3.27 (-3.43,-3.09)
Serbia	76 (24,137)	0.64 (0.20,1.15)	100 (30,181)	0.62 (0.18,1.10)	-0.08 (-0.16,-0.01)	2194 (697,3980)	17.66 (5.57,32.06)	2542 (752,4517)	16.70 (4.97,29.13)	-0.18 (-0.27,-0.09)
Seychelles	1 (0,1)	1.09 (0.37,1.87)	1 (0,1)	0.53 (0.17,0.94)	-2.25 (-2.32,-2.17)	17 (6,29)	30.63 (10.22,52.89)	19 (6,33)	14.49 (4.63,25.47)	-2.35 (-2.40,-2.30)
Sierra Leone	32 (7,68)	1.55 (0.34,3.25)	19 (6,36)	0.47 (0.14,0.88)	-3.79 (-3.84,-3.73)	990 (229,2065)	45.05 (10.26,93.83)	606 (174,1154)	13.47 (3.97,25.66)	-3.80 (-3.86,-3.75)
Singapore	18 (6,30)	0.76 (0.24,1.32)	32 (10,57)	0.36 (0.11,0.64)	-2.46 (-2.67,-2.28)	534 (168,908)	21.33 (6.75,36.34)	817 (252,1463)	9.12 (2.82,16.25)	-2.79 (-3.00,-2.59)
Slovakia	38 (12,69)	0.64 (0.2,1.15)	41 (13,79)	0.43 (0.14,0.82)	-1.33 (-1.42,-1.24)	1100 (345,1988)	18.67 (5.92,33.81)	1062 (347,2039)	11.51 (3.77,21.95)	-1.58 (-1.68,-1.48)
Slovenia	11 (3,19)	0.44 (0.14,0.77)	19 (6,35)	0.44 (0.13,0.81)	0.19 (-0.20,0.50)	323 (102,560)	13.18 (4.20,22.71)	454 (138,817)	11.34 (3.46,20.27)	-0.30 (-0.73,0.08)
Solomon Isla	1 (0,4)	0.9 (0.19,2.47)	3 (1,5)	0.64 (0.19,1.25)	-1.20 (-1.25,-1.14)	46 (9,123)	27.25 (5.64,73.92)	88 (26,174)	19.94 (5.88,39.15)	-1.08 (-1.13,-1.01)
Somalia	14 (3,32)	0.55 (0.11,1.28)	24 (5,60)	0.38 (0.08,0.90)	-1.21 (-1.26,-1.17)	451 (90,1048)	15.29 (3.18,34.61)	778 (170,1963)	10.22 (2.28,25.02)	-1.32 (-1.36,-1.28)
South Africa	100 (26,211)	0.45 (0.12,0.96)	230 (76,410)	0.47 (0.15,0.84)	0.06 (-0.06,0.17)	3307 (833,6858)	13.77 (3.49,28.96)	7305 (2452,12943)	13.89 (4.63,24.67)	0.03 (-0.10,0.15)
South Sudan	8 (2,17)	0.30 (0.07,0.64)	13 (3,26)	0.32 (0.08,0.67)	0.16 (0.09,0.22)	229 (52,487)	8.36 (1.89,17.82)	381 (100,797)	8.41 (2.13,17.6)	0.00 (-0.06,0.06)
Spain	293 (99,492)	0.54 (0.18,0.90)	521 (157,905)	0.58 (0.18,1.00)	0.12 (0.04,0.20)	7548 (2621,12474)	14.48 (5.07,23.78)	12644 (3946,22114)	15.22 (4.8,26.34)	0.06 (-0.03,0.15)
Sri Lanka	26 (9,44)	0.25 (0.09,0.42)	35 (10,68)	0.13 (0.04,0.24)	-2.12 (-2.24,-1.98)	713 (253,1234)	6.19 (2.22,10.59)	908 (265,1738)	3.22 (0.95,6.14)	-2.08 (-2.21,-1.93)
Sudan	20 (5,44)	0.22 (0.06,0.48)	40 (12,81)	0.21 (0.06,0.42)	-0.14 (-0.17,-0.12)	593 (159,1239)	5.89 (1.58,12.52)	1156 (357,2325)	5.30 (1.65,10.65)	-0.36 (-0.39,-0.34)
Suriname	1 (0,1)	0.24 (0.07,0.43)	2 (1,3)	0.26 (0.08,0.49)	0.27 (0.19,0.37)	18 (6,32)	6.67 (1.99,11.6)	49 (15,89)	7.22 (2.23,13.15)	0.31 (0.22,0.40)
Sweden	46 (14,77)	0.31 (0.10,0.52)	63 (19,115)	0.30 (0.09,0.55)	0.06 (-0.30,0.39)	1044 (330,1751)	7.84 (2.50,13.11)	1354 (420,2478)	7.25 (2.27,13.13)	-0.11 (-0.48,0.23)
Switzerland	56 (18,95)	0.55 (0.18,0.93)	78 (24,136)	0.44 (0.14,0.76)	-0.69 (-0.92,-0.52)	1357 (437,2265)	14.25 (4.60,23.71)	1710 (537,2938)	10.40 (3.28,17.86)	-1.04 (-1.23,-0.89)
Syrian Arab F	38 (11,68)	0.72 (0.22,1.33)	62 (18,114)	0.47 (0.13,0.85)	-1.48 (-1.63,-1.37)	1094 (329,1952)	19.06 (5.70,34.30)	1730 (500,3173)	11.65 (3.35,21.34)	-1.61 (-1.74,-1.51)
Taiwan (Prov	175 (62,289)	1.02 (0.36,1.68)	388 (121,652)	0.94 (0.29,1.57)	-0.77 (-1.49,-0.26)	5926 (2131,9744)	32.79 (11.77,53.96)	10716 (3330,17880)	26.82 (8.32,44.59)	-1.09 (-1.77,-0.60)
Tajikistan	11 (3,21)	0.38 (0.11,0.75)	13 (4,27)	0.23 (0.06,0.46)	-1.59 (-1.69,-1.49)	317 (102,610)	10.74 (3.39,20.65)	407 (106,817)	6.01 (1.57,12.04)	-1.88 (-1.96,-1.79)
Thailand	866 (302,1438)	2.33 (0.81,3.85)	1456 (459,2682)	1.33 (0.42,2.43)	-1.91 (-2.10,-1.77)	26576 (9302,44803)	65.33 (22.72,109.31)	40398 (12568,74729	37.26 (11.52,68.25)	-1.91 (-2.10,-1.76)
Timor-Leste	1 (0,2)	0.37 (0.11,0.76)	2 (1,6)	0.29 (0.08,0.68)	-0.83 (-0.87,-0.80)	36 (11,74)	10.14 (3.02,20.98)	71 (19,170)	7.86 (2.14,18.78)	-0.82 (-0.86,-0.79)
Togo	9 (2,18)	0.64 (0.18,1.35)	15 (4,31)	0.34 (0.09,0.71)	-2.06 (-2.12,-1.99)	277 (79,602)	18.66 (5.29,40.00)	487 (124,1061)	9.95 (2.49,21.25)	-2.03 (-2.08,-1.97)
Tokelau	0 (0,0)	0.37 (0.10,0.83)	0 (0,0)	0.49 (0.14,1.00)	0.92 (0.84,1.02)	0 (0,0)	10.48 (2.81,23.19)	0 (0,0)	14.64 (4.16,29.51)	1.06 (0.98,1.16)
Tonga	2 (1,4)	3.29 (1.02,6.08)	2 (1,4)	2.86 (0.87,5.24)	-0.42 (-0.53,-0.32)	57 (18,102)	93.42 (29.46,169.91)	69 (21,128)	81.96 (25.26,152.29)	-0.48 (-0.59,-0.39)
Trinidad and	2 (1,3)	0.20 (0.06,0.36)	3 (1,6)	0.17 (0.05,0.31)	-0.60 (-0.79,-0.36)	47 (15,83)	5.47 (1.75,9.55)	90 (28,165)	4.56 (1.43,8.38)	-0.54 (-0.73,-0.32)
Tunisia	10 (3,17)	0.19 (0.06,0.35)	31 (10,56)	0.23 (0.08,0.42)	0.54 (0.49,0.58)	262 (87,464)	4.89 (1.62,8.65)	818 (265,1490)	5.84 (1.90,10.67)	0.60 (0.56,0.65)
Turkey	148 (48,257)	0.41 (0.13,0.73)	311 (91,561)	0.32 (0.09,0.58)	-0.80 (-0.85,-0.75)	4396 (1462,7505)	11.44 (3.77,19.64)	8490 (2480,15350)	8.62 (2.52,15.54)	-0.91 (-0.95,-0.86)
Turkmenistan	11 (4,19)	0.53 (0.18,0.93)	15 (4,28)	0.34 (0.09,0.63)	-1.74 (-2.00,-1.49)	340 (117,606)	15.85 (5.44,28.15)	473 (133,872)	9.97 (2.80,18.41)	-1.72 (-2.00,-1.44)
Tuvalu	0 (0,0)	0.50 (0.15,0.98)	0 (0,0)	0.53 (0.16,1.07)	0.18 (0.12,0.22)	1 (0,2)	14.82 (4.38,29.12)	2 (1,4)	15.95 (4.63,32.88)	0.20 (0.14,0.25)
Uganda	28 (9,52)	0.43 (0.14,0.80)	45 (13,89)	0.30 (0.09,0.58)	-1.17 (-1.21,-1.13)	820 (261,1527)	11.65 (3.75,21.39)	1359 (385,2764)	7.98 (2.29,15.79)	-1.23 (-1.29,-1.19)
Ukraine	145 (51,242)	0.20 (0.07,0.33)	110 (35,199)	0.15 (0.05,0.27)	-0.94 (-1.34,-0.46)	4261 (1528,7050)	5.97 (2.15,9.88)	3232 (1043,5814)	4.60 (1.50,8.26)	-0.87 (-1.27,-0.41)
United Arab	13 (1,6)	0.62 (0.19,1.24)	33 (10,64)	0.79 (0.24,1.55)	0.75 (0.66,0.84)	100 (32,193)	16.61 (5.31,32.68)	1130 (337,2136)	18.68 (5.88,36.48)	0.34 (0.27,0.40)
United Kingd	270 (90,447)	0.30 (0.10,0.50)	780 (245,1398)	0.60 (0.19,1.06)	2.17 (2.06,2.28)	6277 (2127,10338)	7.53 (2.57,12.35)	16627 (5358,29298)	14.08 (4.57,24.44)	1.98 (1.87,2.09)
United Repul	67 (20,125)	0.62 (0.18,1.13)	98 (26,197)	0.38 (0.10,0.78)	-1.57 (-1.61,-1.53)	1967 (587,3669)	16.50 (4.96,30.90)	2890 (759,6019)	9.97 (2.61,20.27)	-1.64 (-1.67,-1.61)

United States	775 (249,1323)	0.25 (0.08,0.42)	2606 (808,4612)	0.45 (0.14,0.79)	1.89 (1.83,1.94)	20258 (6660,34271)	6.86 (2.26,11.56)	63755 (20011,11092	11.57 (3.65,20.04)	1.65 (1.56,1.70)
United States	0 (0,0)	0.12 (0.04,0.23)	0 (0,1)	0.17 (0.05,0.34)	1.11 (1.00,1.21)	3 (1,6)	3.18 (0.90,5.97)	8 (2,15)	4.51 (1.28,8.68)	1.16 (1.05,1.27)
Uruguay	5 (2,9)	0.13 (0.04,0.22)	16 (5,29)	0.31 (0.10,0.55)	2.71 (2.54,2.87)	142 (46,235)	3.74 (1.22,6.15)	402 (129,709)	8.21 (2.66,14.34)	2.52 (2.34,2.69)
Uzbekistan	21 (6,40)	0.17 (0.05,0.34)	87 (27,157)	0.30 (0.09,0.54)	1.75 (1.65,1.88)	656 (195,1274)	5.33 (1.59,10.24)	2693 (844,4872)	8.61 (2.69,15.70)	1.56 (1.46,1.69)
Vanuatu	0 (0,1)	0.49 (0.14,1.05)	1 (0,1)	0.36 (0.10,0.78)	-1.05 (-1.10,-0.99)	10 (3,21)	13.63 (4.03,28.97)	20 (5,43)	9.91 (2.58,21.15)	-1.03 (-1.11,-0.97)
Venezuela (B	52 (17,89)	0.55 (0.18,0.95)	54 (17,99)	0.18 (0.05,0.33)	-3.74 (-4.19,-3.23)	1387 (466,2363)	13.88 (4.66,23.71)	1374 (424,2517)	4.41 (1.36,8.09)	-3.87 (-4.32,-3.35)
Viet Nam	980 (351,1731)	2.37 (0.86,4.18)	2047 (672,3758)	1.88 (0.62,3.42)	-0.76 (-0.80,-0.72)	29348 (10144,5180	69.64 (24.47,123.05)	63453 (20749,11672	55.80 (18.35,102.26)	-0.72 (-0.76,-0.69)
Yemen	16 (4,35)	0.30 (0.08,0.69)	28 (8,57)	0.48 (0.28,0.71)	-1.39 (-1.42,-1.37)	488 (123,1057)	8.61 (2.18,18.90)	825 (229,1655)	5.14 (1.41,10.34)	-1.64 (-1.69,-1.61)
Zambia	17 (5,31)	0.62 (0.18,1.13)	16 (4,48)	0.12 (0.08,0.18)	-2.81 (-2.90,-2.73)	493 (151,890)	16.03 (4.78,28.95)	456 (101,1385)	6.12 (1.42,18.07)	-3.11 (-3.22,-3.02)
Zimbabwe	49 (13,100)	1.19 (0.32,2.40)	71 (20,140)	0.55 (0.38,0.75)	-0.43 (-0.52,-0.35)	1400 (371,2896)	31.40 (8.47,64.72)	2157 (607,4319)	27.06 (7.70,53.05)	-0.55 (-0.64,-0.46)
Afghanistan	27 (14,50)	0.43 (0.23,0.77)	38 (22,58)	0.37 (0.28,0.48)	0.37 (0.36,0.39)	660 (348,1211)	9.45 (5.04,17.23)	995 (544,1528)	10.48 (5.9,15.88)	0.34 (0.33,0.36)
Albania	2 (2,3)	0.13 (0.10,0.18)	5 (4,8)	1.14 (0.71,1.65)	-0.28 (-0.44,-0.16)	51 (39,68)	2.63 (1.99,3.53)	105 (72,150)	2.33 (1.59,3.33)	-0.44 (-0.59,-0.33)
Algeria	60 (40,80)	0.74 (0.48,0.98)	140 (99,194)	0.37 (0.26,0.53)	-0.97 (-1.00,-0.95)	1301 (884,1715)	12.70 (8.62,16.63)	2896 (2047,3959)	9.38 (6.60,12.92)	-0.98 (-1.00,-0.96)
American Sa	0 (0,0)	0.33 (0.24,0.46)	0 (0,0)	0.35 (0.27,0.44)	0.39 (0.26,0.51)	2 (1,2)	7.93 (5.76,10.77)	5 (3,6)	9.07 (6.89,11.79)	0.50 (0.38,0.62)
Andorra	1 (1,2)	2.3 (1.52,3.32)	2 (1,3)	0.64 (0.51,0.78)	-2.38 (-2.58,-2.23)	28 (18,42)	48.64 (31.76,71.37)	37 (23,55)	23.98 (14.91,35.41)	-2.39 (-2.58,-2.24)
Angola	14 (9,20)	0.43 (0.30,0.63)	36 (25,50)	2.3 (1.85,2.83)	-0.42 (-0.45,-0.38)	360 (242,534)	9.57 (6.53,14.07)	947 (675,1334)	8.19 (5.84,11.45)	-0.47 (-0.51,-0.42)
Antigua and I	0 (0,0)	0.37 (0.29,0.47)	0 (0,0)	0.36 (0.28,0.48)	-0.17 (-0.41,0.13)	4 (3,5)	7.96 (6.23,10.07)	8 (6,10)	7.15 (5.48,9.00)	-0.33 (-0.62,0.01)
Argentina	407 (332,478)	1.24 (1.01,1.47)	365 (287,447)	0.81 (0.65,1.00)	-2.10 (-2.20,-2.02)	9835 (8249,11556)	29.67 (24.80,34.83)	8426 (6807,10155)	15.18 (12.3,18.15)	-2.09 (-2.23,-1.99)
Armenia	56 (46,68)	2.16 (1.77,2.61)	101 (82,125)	0.69 (0.48,0.96)	0.18 (-0.05,0.36)	1442 (1173,1747)	51.13 (41.54,61.58)	2238 (1807,2710)	50.89 (41.12,61.61)	0.03 (-0.17,0.22)
Australia	195 (162,233)	0.97 (0.81,1.16)	177 (133,235)	0.25 (0.18,0.32)	-3.16 (-3.23,-3.10)	4402 (3710,5164)	22.07 (18.60,25.86)	3468 (2750,4446)	7.75 (6.22,9.76)	-3.36 (-3.45,-3.27)
Austria	159 (133,188)	1.28 (1.08,1.50)	159 (126,199)	1.38 (0.93,1.96)	-1.41 (-1.55,-1.29)	3450 (2936,4032)	29.51 (25.22,34.39)	3232 (2648,3888)	18.23 (15.07,21.75)	-1.50 (-1.64,-1.38)
Azerbaijan	35 (26,49)	0.72 (0.53,0.99)	65 (45,92)	0.35 (0.21,0.73)	-0.12 (-0.21,-0.03)	965 (701,1350)	18.25 (13.41,25.73)	1712 (1140,2454)	16.06 (10.83,22.64)	-0.43 (-0.51,-0.36)
Bahamas	0 (0,0)	0.24 (0.18,0.32)	1 (1,1)	0.31 (0.21,0.42)	0.06 (-0.08,0.18)	9 (6,11)	5.67 (4.23,7.31)	22 (15,28)	5.37 (3.81,6.90)	-0.04 (-0.23,0.10)
Bahrain	3 (2,4)	2.47 (1.80,3.32)	8 (5,12)	0.98 (0.77,1.20)	-1.88 (-1.99,-1.76)	79 (59,104)	51.02 (37.27,66.68)	216 (139,326)	27.39 (18.03,39.44)	-2.00 (-2.07,-1.93)
Bangladesh	210 (126,337)	0.52 (0.31,0.84)	422 (239,865)	1.05 (0.86,1.27)	-1.16 (-1.31,-1.04)	4611 (2802,7304)	10.51 (6.33,16.70)	8479 (4757,17457)	6.49 (3.67,13.34)	-1.42 (-1.54,-1.33)
Barbados	1 (1,1)	0.37 (0.29,0.45)	2 (1,2)	0.29 (0.21,0.36)	-0.44 (-0.68,-0.20)	22 (17,27)	7.22 (5.74,8.78)	30 (21,41)	5.64 (3.89,7.71)	-0.62 (-0.89,-0.37)
Belarus	172 (148,196)	1.31 (1.13,1.50)	162 (127,198)	0.10 (0.07,0.15)	-0.96 (-1.15,-0.77)	4121 (3566,4704)	31.11 (26.85,35.38)	3808 (2967,4662)	23.17 (17.95,28.40)	-0.88 (-1.07,-0.70)
Belgium	374 (315,436)	2.33 (1.96,2.72)	267 (214,328)	0.78 (0.58,1.02)	-2.62 (-2.88,-2.41)	7723 (6556,8886)	49.81 (42.53,57.28)	5179 (4339,6200)	22.52 (19.05,26.78)	-2.64 (-2.87,-2.45)
Belize	0 (0,0)	0.28 (0.22,0.34)	1 (1,1)	0.19 (0.11,0.44)	0.32 (0.16,0.46)	5 (4,6)	5.78 (4.68,7.02)	17 (13,22)	5.98 (4.55,7.52)	0.27 (0.09,0.44)
Benin	4 (3,6)	0.21 (0.15,0.31)	5 (3,7)	0.25 (0.16,0.37)	-2.30 (-2.35,-2.26)	90 (64,132)	4.59 (3.25,6.74)	114 (78,158)	2.25 (1.55,3.18)	-2.24 (-2.28,-2.20)
Bermuda	1 (1,1)	1.18 (0.89,1.53)	1 (1,2)	1.55 (1.11,2.06)	-1.39 (-1.52,-1.26)	16 (12,20)	25.18 (19.16,32.36)	23 (17,30)	16.34 (12.16,21.35)	-1.46 (-1.59,-1.35)
Bhutan	0 (0,1)	0.21 (0.11,0.38)	1 (1,2)	0.42 (0.31,0.58)	-0.36 (-0.40,-0.33)	9 (4,16)	4.16 (2.13,7.31)	19 (11,44)	3.37 (1.91,7.72)	-0.63 (-0.66,-0.60)
Bolivia (Pluri	9 (6,14)	0.34 (0.22,0.49)	21 (13,30)	0.50 (0.41,0.62)	-0.91 (-0.95,-0.89)	216 (145,309)	7.04 (4.73,10.01)	446 (297,654)	5.05 (3.33,7.45)	-1.07 (-1.10,-1.04)
Bosnia and H	43 (35,54)	1.15 (0.93,1.40)	101 (73,135)	0.53 (0.39,0.69)	1.00 (0.89,1.09)	1081 (873,1343)	25.9 (21.07,31.93)	2157 (1552,2864)	33.61 (24.1,44.64)	0.94 (0.84,1.04)
Botswana	3 (2,3)	0.56 (0.40,0.76)	5 (4,7)	1.41 (1.13,1.79)	-0.85 (-0.91,-0.81)	64 (44,87)	11.76 (8.25,15.9)	124 (87,180)	8.85 (6.28,12.43)	-0.89 (-0.94,-0.85)
Brazil	755 (654,861)	0.96 (0.82,1.09)	1235 (999,1524)	0.08 (0.06,0.12)	-1.97 (-2.05,-1.90)	17496 (15308,1984	20.15 (17.53,22.97)	26059 (21410,31667	10.37 (8.51,12.63)	-2.09 (-2.19,-2.00)
Brunei Darus	1 (1,1)	1.06 (0.80,1.42)	1 (1,2)	0.17 (0.11,0.25)	-2.26 (-2.40,-2.14)	21 (16,28)	21.99 (16.64,29.34)	35 (25,45)	10.59 (7.78,13.63)	-2.38 (-2.51,-2.27)
Bulgaria	159 (133,190)	1.26 (1.05,1.49)	207 (164,261)	0.16 (0.08,0.24)	0.39 (0.29,0.49)	4150 (3473,4913)	31.95 (26.86,37.67)	5024 (3973,6342)	36.70 (28.81,46.68)	0.50 (0.38,0.58)
Burkina Faso	4 (3,6)	0.11 (0.07,0.15)	7 (5,10)	0.54 (0.37,0.83)	-0.74 (-0.78,-0.71)	110 (73,158)	2.48 (1.67,3.55)	191 (134,269)	1.97 (1.39,2.79)	-0.75 (-0.78,-0.72)
Burundi	8 (5,12)	0.38 (0.23,0.58)	7 (4,10)	0.16 (0.11,0.23)	-2.56 (-2.59,-2.53)	186 (117,288)	8.23 (5.15,12.7)	169 (107,256)	3.61 (2.36,5.36)	-2.63 (-2.65,-2.60)
Cabo Verde	0 (0,0)	0.03 (0.01,0.08)	1 (0,1)	0.76 (0.59,0.94)	6.14 (5.55,6.68)	1 (1,4)	0.59 (0.33,1.69)	16 (8,24)	3.65 (1.81,5.31)	6.07 (5.61,6.52)
Cambodia	22 (16,32)	0.59 (0.43,0.86)	56 (38,86)	0.23 (0.15,0.32)	-0.23 (-0.25,-0.21)	530 (380,783)	12.38 (8.94,18.37)	1315 (897,1967)	11.06 (7.54,16.76)	-0.34 (-0.36,-0.33)
Cameroon	9 (6,12)	0.22 (0.16,0.29)	18 (12,26)	0.19 (0.12,0.29)	-1.03 (-1.05,-1.00)	230 (169,312)	5.03 (3.70,6.71)	510 (336,722)	3.79 (2.50,5.41)	-0.92 (-0.95,-0.89)
Canada	603 (511,696)	1.83 (1.55,2.11)	596 (459,751)	0.31 (0.24,0.39)	-2.73 (-2.85,-2.58)	12958 (11092,1492	39.66 (33.91,45.60)	10915 (8736,13458)	14.78 (11.93,18.12)	-3.05 (-3.18,-2.89)
Central Afric	3 (2,5)	0.33 (0.21,0.49)	4 (3,6)	0.87 (0.66,1.18)	-1.26 (-1.29,-1.23)	89 (54,135)	7.73 (4.94,11.52)	120 (78,175)	5.28 (3.60,7.63)	-1.24 (-1.26,-1.21)
Chad	5 (3,8)	0.21 (0.13,0.30)	9 (6,14)	0.14 (0.10,0.19)	-0.30 (-0.33,-0.27)	119 (74,167)	4.31 (2.68,6.01)	222 (135,327)	3.99 (2.44,5.90)	-0.23 (-0.26,-0.19)
Chile	55 (43,67)	0.56 (0.44,0.69)	80 (62,100)	0.28 (0.15,0.49)	-1.86 (-1.99,-1.73)	1413 (1148,1702)	13.82 (11.17,16.68)	1922 (1561,2337)	7.48 (6.09,9.08)	-1.96 (-2.09,-1.86)
China	8405 (5640,10517	1.24 (0.84,1.53)	17178 (12936,234	0.41 (0.28,0.58)	-1.23 (-1.33,-1.14)	202646 (131999,25	25.29 (16.79,31.73)	359985 (266681,490	17.09 (12.74,23.35)	-1.26 (-1.33,-1.19)
Colombia	56 (47,68)	0.35 (0.29,0.43)	77 (58,103)	0.48 (0.35,0.65)	-2.98 (-3.20,-2.80)	1326 (1097,1571)	7.69 (6.40,9.18)	1608 (1216,2139)	2.92 (2.21,3.88)	-3.04 (-3.21,-2.90)
Comoros	1 (0,1)	0.36 (0.22,0.56)	1 (1,2)	0.35 (0.27,0.45)	-0.76 (-0.80,-0.72)	13 (8,21)	7.16 (4.38,11.37)	24 (12,40)	5.27 (2.75,9.15)	-1.02 (-1.07,-0.97)
Congo	4 (2,5)	0.39 (0.25,0.57)	8 (6,12)	1.81 (1.43,2.23)	0.17 (0.15,0.20)	88 (53,130)	8.48 (5.22,12.42)	206 (139,299)	8.32 (5.68,12.14)	-0.05 (-0.07,-0.01)
Cook Islands	0 (0,0)	0.61 (0.44,0.80)	0 (0,0)	1.04 (0.81,1.28)	-0.75 (-0.81,-0.71)	2 (1,2)	14.52 (10.59,19.04)	3 (2,4)	11.63 (8.47,16.10)	-0.71 (-0.77,-0.67)
Costa Rica	10 (8,12)	0.61 (0.5,0.73)	19 (15,25)	1.28 (0.92,1.78)	-1.62 (-1.80,-1.44)	206 (174,242)	12.19 (10.25,14.36)	391 (306,487)	7.14 (5.58,8.90)	-1.67 (-1.86,-1.49)
Croatia	118 (98,141)	2.08 (1.72,2.50)	20 (13,29)	1.31 (1.01,1.65)	-0.46 (-0.49,-0.43)	2574 (2178,3028)	42.59 (35.69,50.17)	504 (322,766)	38.24 (30.53,47.09)	-0.47 (-0.50,-0.44)
Cuba	120 (100,141)	1.19 (0.98,1.39)	176 (139,218)	0.22 (0.15,0.32)	-0.50 (-0.66,-0.37)	2547 (2173,2944)	24.88 (21.31,28.87)	3469 (2765,4280)	22.44 (17.79,27.18)	-0.41 (-0.69,-0.15)

Cyprus	16 (12,21)	2.41 (1.74,3.28)	211 (164,259)	0.48 (0.34,0.71)	-0.32 (-0.48,-0.14)	318 (242,429)	42.84 (32.05,57.33)	4428 (3489,5376)	26.45 (19.15,36.25)	-0.21 (-0.46,0.01)
Czechia	259 (216,309)	1.82 (1.51,2.16)	27 (20,38)	0.15 (0.10,0.22)	-1.99 (-2.11,-1.88)	6080 (5094,7195)	43.29 (36.38,51.31)	561 (405,771)	29.89 (23.32,37.75)	-1.58 (-1.72,-1.44)
C么te d'Ivoir	8 (5,11)	0.26 (0.18,0.36)	301 (234,381)	1.48 (1.23,1.77)	-1.05 (-1.11,-0.99)	203 (136,282)	5.40 (3.62,7.34)	6504 (5092,8275)	4.70 (3.10,6.82)	-1.19 (-1.27,-1.12)
Democratic P	84 (57,115)	0.58 (0.4,0.79)	155 (111,230)	0.53 (0.32,0.87)	-0.64 (-0.65,-0.62)	2258 (1561,3098)	13.60 (9.36,18.66)	4037 (2834,6009)	11.92 (8.35,17.65)	-0.43 (-0.44,-0.41)
Democratic R	26 (17,36)	0.19 (0.13,0.28)	47 (31,69)	0.36 (0.25,0.52)	-0.88 (-0.91,-0.85)	671 (448,950)	4.32 (2.86,6.17)	1289 (855,1879)	3.43 (2.25,5.02)	-0.75 (-0.78,-0.71)
Denmark	209 (168,266)	2.44 (1.98,3.08)	195 (160,233)	0.30 (0.20,0.42)	-1.59 (-1.83,-1.31)	4197 (3411,5296)	52.15 (42.65,65.02)	3631 (3056,4314)	29.57 (24.82,34.93)	-1.78 (-2.03,-1.48)
Djibouti	0 (0,1)	0.51 (0.33,0.79)	2 (1,4)	0.17 (0.12,0.23)	0.10 (0.08,0.12)	12 (7,20)	10.32 (6.48,15.94)	56 (32,95)	10.13 (5.92,16.72)	-0.07 (-0.11,-0.04)
Dominica	0 (0,0)	0.42 (0.30,0.57)	0 (0,0)	1.60 (1.03,2.94)	-0.44 (-0.47,-0.41)	5 (4,7)	8.73 (6.39,11.77)	7 (5,9)	7.73 (5.36,11.03)	-0.39 (-0.43,-0.36)
Dominican R	10 (8,13)	0.34 (0.25,0.44)	28 (20,41)	0.09 (0.06,0.12)	-0.29 (-0.57,-0.12)	213 (160,273)	6.24 (4.70,7.98)	568 (386,822)	5.79 (3.95,8.35)	-0.24 (-0.36,-0.14)
Ecuador	11 (9,14)	0.24 (0.19,0.30)	27 (19,36)	0.28 (0.17,0.46)	-1.09 (-1.53,-0.79)	226 (182,268)	4.53 (3.65,5.43)	520 (365,695)	3.23 (2.28,4.33)	-1.13 (-1.58,-0.81)
Egypt	634 (394,804)	2.62 (1.90,3.38)	803 (531,1428)	0.14 (0.09,0.22)	-1.56 (-1.67,-1.45)	18473 (9849,23646)	63.69 (39.16,80.87)	20753 (13988,36281	34.21 (22.78,61.01)	-1.96 (-2.11,-1.86)
El Salvador	2 (2,3)	0.08 (0.06,0.11)	5 (4,7)	0.89 (0.72,1.09)	0.34 (0.20,0.48)	53 (39,71)	1.78 (1.30,2.40)	123 (88,171)	2.03 (1.45,2.81)	0.47 (0.30,0.62)
Equatorial Gu	0 (0,1)	0.27 (0.18,0.40)	1 (1,2)	0.22 (0.14,0.32)	0.12 (0.06,0.17)	12 (7,19)	6.27 (4.01,9.38)	30 (18,47)	6.02 (3.64,9.76)	-0.12 (-0.19,-0.06)
Eritrea	2 (1,3)	0.18 (0.10,0.27)	3 (2,5)	0.10 (0.06,0.16)	-0.71 (-0.75,-0.68)	55 (32,84)	4.35 (2.57,6.65)	96 (58,151)	3.25 (2.06,5.03)	-0.96 (-1.01,-0.91)
Estonia	22 (18,26)	1.05 (0.88,1.25)	25 (20,31)	0.25 (0.14,0.35)	-0.55 (-0.76,-0.29)	552 (468,649)	26.65 (22.57,31.29)	543 (438,658)	21.02 (17.04,25.40)	-0.75 (-0.98,-0.47)
Eswatini	1 (0,1)	0.31 (0.22,0.43)	1 (1,2)	0.40 (0.31,0.50)	-1.00 (-1.07,-0.93)	17 (12,23)	6.38 (4.48,8.81)	26 (17,39)	4.84 (3.10,7.18)	-0.87 (-0.93,-0.83)
Ethiopia	26 (14,41)	0.14 (0.08,0.23)	36 (21,60)	1.09 (0.87,1.32)	-1.22 (-1.25,-1.20)	656 (365,1048)	3.27 (1.81,5.23)	805 (477,1375)	1.98 (1.18,3.35)	-1.59 (-1.61,-1.57)
Fiji	1 (1,1)	0.29 (0.20,0.38)	2 (1,3)	0.32 (0.22,0.48)	-0.53 (-0.62,-0.45)	27 (18,35)	7.25 (4.92,9.42)	51 (28,73)	6.09 (3.44,8.77)	-0.55 (-0.66,-0.45)
Finland	67 (55,80)	0.92 (0.75,1.09)	55 (43,70)	0.14 (0.10,0.21)	-2.87 (-2.99,-2.76)	1509 (1245,1778)	21.24 (17.69,24.94)	1114 (875,1357)	9.03 (7.25,10.97)	-2.95 (-3.06,-2.85)
France	1699 (1421,1982)	1.97 (1.65,2.28)	1669 (1311,2071)	1.96 (1.58,2.37)	-1.83 (-1.92,-1.75)	35566 (30431,4088	43.71 (37.67,50.16)	32202 (26129,38715	23.73 (19.17,28.34)	-1.83 (-1.88,-1.78)
Gabon	2 (1,2)	0.32 (0.21,0.45)	3 (2,4)	0.89 (0.71,1.08)	-0.01 (-0.04,0.02)	41 (27,59)	7.17 (4.69,10.18)	75 (51,110)	7.12 (4.99,10.47)	-0.01 (-0.05,0.02)
Gambia	1 (0,1)	0.20 (0.12,0.27)	1 (1,2)	0.19 (0.12,0.28)	-1.05 (-1.15,-0.94)	15 (10,20)	4.33 (2.77,5.91)	30 (20,42)	3.11 (2.12,4.48)	-1.00 (-1.12,-0.87)
Georgia	76 (58,96)	1.20 (0.92,1.52)	119 (97,143)	2.21 (1.86,2.61)	1.55 (1.19,1.84)	2026 (1579,2573)	31.33 (24.44,39.66)	2707 (2209,3269)	46.02 (37.52,55.66)	1.41 (1.11,1.69)
Germany	2339 (1958,2686)	1.75 (1.47,1.99)	1903 (1492,2333)	1.07 (0.72,1.47)	-2.16 (-2.25,-2.07)	48215 (41102,5490	37.64 (32.26,42.75)	36350 (29465,44147	19.16 (15.97,23.11)	-2.13 (-2.21,-2.04)
Ghana	9 (6,13)	0.21 (0.15,0.30)	23 (15,35)	0.30 (0.23,0.39)	-0.40 (-0.44,-0.36)	220 (149,301)	3.94 (2.72,5.36)	529 (360,784)	3.45 (2.32,5.21)	-0.42 (-0.46,-0.39)
Greece	507 (439,572)	3.27 (2.83,3.68)	598 (495,712)	0.23 (0.17,0.29)	-1.28 (-1.39,-1.20)	10576 (9279,11859)	67.93 (59.7,76.09)	10950 (9282,12899)	46.88 (40.34,54.59)	-1.24 (-1.34,-1.14)
Greenland	1 (0,1)	1.98 (1.30,2.49)	1 (0,1)	0.07 (0.05,0.09)	-1.89 (-2.02,-1.77)	15 (10,19)	44.74 (29.21,57.35)	17 (11,24)	23.56 (15.97,32.81)	-1.93 (-2.02,-1.84)
Grenada	0 (0,0)	0.33 (0.25,0.43)	0 (0,0)	0.39 (0.25,0.57)	-0.16 (-0.30,-0.02)	5 (4,7)	7.62 (5.72,9.56)	8 (6,10)	6.75 (5.04,8.54)	-0.22 (-0.35,-0.11)
Guam	0 (0,0)	0.25 (0.18,0.33)	0 (0,1)	0.16 (0.10,0.24)	-0.30 (-0.56,-0.08)	5 (4,7)	6.38 (4.72,8.51)	14 (10,18)	6.58 (4.91,8.40)	0.09 (-0.10,0.24)
Guatemala	3 (3,4)	0.13 (0.10,0.17)	7 (6,10)	0.21 (0.15,0.29)	-1.89 (-2.10,-1.61)	79 (62,99)	2.51 (1.96,3.16)	159 (120,202)	1.48 (1.11,1.88)	-1.70 (-1.91,-1.46)
Guinea	10 (7,14)	0.35 (0.23,0.49)	19 (12,28)	0.19 (0.11,0.29)	0.32 (0.30,0.33)	228 (156,323)	7.07 (4.81,9.95)	435 (283,651)	8.05 (5.25,11.98)	0.42 (0.40,0.44)
Guinea-Bissa	1 (0,1)	0.15 (0.09,0.23)	1 (1,2)	0.13 (0.08,0.20)	0.09 (0.06,0.11)	14 (8,22)	3.56 (1.99,5.48)	29 (19,44)	3.76 (2.47,5.66)	0.18 (0.16,0.21)
Guyana	1 (1,1)	0.26 (0.20,0.32)	1 (1,2)	1.42 (1.14,1.78)	-0.56 (-0.89,-0.30)	21 (17,27)	5.75 (4.53,7.29)	32 (22,44)	4.81 (3.3,6.66)	-0.39 (-0.57,-0.24)
Haiti	8 (5,12)	0.27 (0.17,0.41)	11 (7,17)	0.72 (0.56,0.91)	-1.03 (-1.06,-1.00)	202 (128,310)	6.16 (3.93,9.38)	283 (168,413)	4.07 (2.45,6.00)	-1.29 (-1.32,-1.26)
Honduras	2 (1,3)	0.10 (0.07,0.14)	7 (5,11)	0.24 (0.19,0.30)	0.86 (0.81,0.91)	43 (31,61)	2.18 (1.52,3.04)	165 (104,253)	2.69 (1.68,4.16)	0.75 (0.71,0.79)
Hungary	260 (216,306)	1.72 (1.44,2.03)	282 (225,354)	0.35 (0.21,0.63)	-0.58 (-0.72,-0.46)	6286 (5273,7322)	42.11 (35.48,48.91)	6760 (5429,8447)	36.36 (29.27,44.73)	-0.38 (-0.53,-0.24)
Iceland	5 (4,6)	1.66 (1.39,1.94)	4 (3,6)	0.48 (0.35,0.60)	-2.70 (-2.80,-2.60)	102 (86,119)	35.86 (30.35,41.60)	88 (70,113)	15.09 (12.04,19.24)	-2.80 (-2.91,-2.70)
India	1162 (792,1513)	0.31 (0.21,0.41)	2494 (1952,3204)	1.84 (1.22,2.63)	-0.83 (-0.92,-0.72)	28136 (19209,3637	6.51 (4.43,8.46)	53665 (41655,69142	4.70 (3.65,6.05)	-1.04 (-1.11,-0.93)
Indonesia	243 (184,330)	0.30 (0.23,0.42)	668 (419,1193)	0.71 (0.56,0.89)	0.41 (0.38,0.44)	6033 (4616,7958)	6.43 (4.89,8.64)	16260 (10245,28369	7.05 (4.44,12.55)	0.29 (0.27,0.31)
Iran (Islamic	115 (82,152)	0.50 (0.36,0.67)	340 (252,423)	0.86 (0.67,1.07)	-0.16 (-0.24,-0.10)	3110 (2231,4105)	11.62 (8.32,15.30)	8311 (6262,10275)	10.77 (8.03,13.39)	-0.24 (-0.33,-0.17)
Iraq	122 (84,171)	1.69 (1.16,2.36)	347 (227,502)	1.18 (0.96,1.41)	0.34 (0.26,0.43)	2806 (1909,3945)	36.74 (24.95,51.54)	8248 (5397,12180)	37.44 (24.61,54.54)	0.10 (0.03,0.18)
Ireland	75 (64,86)	1.78 (1.52,2.06)	60 (47,75)	0.40 (0.28,0.56)	-2.86 (-3.04,-2.72)	1500 (1293,1724)	35.77 (30.79,41.02)	1135 (894,1396)	13.88 (10.98,17.02)	-2.98 (-3.11,-2.86)
Israel	73 (60,86)	1.49 (1.23,1.79)	112 (87,141)	0.52 (0.42,0.62)	-1.72 (-2.01,-1.42)	1529 (1292,1794)	31.23 (26.46,36.71)	2192 (1742,2678)	17.74 (14.21,21.61)	-1.65 (-1.96,-1.35)
Italy	2382 (2026,2719)	2.58 (2.19,2.94)	1943 (1541,2357)	1.01 (0.71,1.41)	-2.54 (-2.60,-2.48)	52909 (45288,5984	58.51 (50.22,66.09)	37094 (30037,44360	25.58 (21.17,30.13)	-2.69 (-2.76,-2.62)
Jamaica	9 (7,11)	0.47 (0.38,0.58)	12 (9,17)	0.50 (0.39,0.62)	-0.40 (-0.83,-0.00)	177 (145,216)	9.83 (8.09,11.98)	268 (187,371)	8.73 (6.09,12.13)	-0.33 (-0.79,0.09)
Japan	1410 (1237,1588)	0.86 (0.75,0.97)	2376 (1846,2937)	0.11 (0.08,0.15)	-1.60 (-1.66,-1.53)	29685 (26362,3312	17.43 (15.40,19.47)	39094 (31528,46856	10.59 (8.87,12.46)	-1.54 (-1.61,-1.46)
Jordan	15 (11,19)	1.36 (0.99,1.79)	60 (42,85)	0.15 (0.11,0.19)	-0.93 (-0.98,-0.88)	367 (268,488)	28.95 (21.18,38.36)	1545 (1058,2190)	21.37 (14.96,30.18)	-0.96 (-0.99,-0.93)
Kazakhstan	90 (66,124)	0.72 (0.52,0.98)	88 (69,109)	0.8 (0.60,1.03)	-1.12 (-1.29,-0.92)	2477 (1791,3456)	18.68 (13.52,25.95)	2390 (1876,2931)	12.55 (9.82,15.48)	-1.18 (-1.31,-1.04)
Kenya	8 (5,12)	0.12 (0.08,0.18)	21 (15,27)	0.52 (0.40,0.67)	-0.33 (-0.37,-0.27)	178 (114,266)	2.39 (1.52,3.56)	487 (356,641)	2.28 (1.65,2.96)	-0.15 (-0.19,-0.09)
Kiribati	0 (0,0)	0.14 (0.10,0.17)	0 (0,0)	0.47 (0.30,0.73)	0.35 (0.32,0.37)	1 (1,2)	3.33 (2.57,4.13)	3 (2,4)	3.66 (2.70,4.74)	0.31 (0.28,0.33)
Kuwait	3 (3,4)	0.72 (0.57,0.88)	18 (14,23)	1.32 (1.05,1.62)	0.25 (-0.25,0.78)	89 (71,107)	15.82 (12.48,19.33)	461 (349,590)	16.66 (12.42,21.50)	-0.01 (-0.54,0.56)
Kyrgyzstan	17 (13,21)	0.60 (0.47,0.74)	24 (18,30)	2.94 (2.12,4.01)	-0.46 (-0.72,-0.23)	449 (350,544)	14.85 (11.54,18.00)	637 (493,815)	12.61 (9.78,16.09)	-0.53 (-0.69,-0.32)
Lao People's	10 (6,16)	0.56 (0.33,0.87)	18 (11,27)	0.56 (0.34,0.83)	-0.56 (-0.58,-0.55)	251 (149,396)	12.29 (7.38,19.30)	409 (263,620)	9.42 (6.05,14.44)	-0.85 (-0.87,-0.83)
Latvia	43 (37,51)	1.20 (1.02,1.39)	54 (43,66)	0.12 (0.07,0.17)	0.34 (0.06,0.60)	1070 (928,1235)	29.66 (25.72,34.04)	1189 (945,1462)	31.45 (25.30,38.60)	0.23 (-0.06,0.50)
Lebanon	65 (42,91)	3.38 (2.20,4.76)	189 (136,259)	1.97 (1.36,2.89)	-0.41 (-0.47,-0.35)	1521 (948,2171)	71.75 (45.70,101.00)	3592 (2622,4812)	58.87 (43.13,78.57)	-0.60 (-0.67,-0.54)

Lesotho	3 (2,4)	0.39 (0.27,0.56)	5 (3,8)	1.14 (0.93,1.38)	1.28 (1.15,1.38)	65 (45,96)	7.94 (5.48,11.63)	139 (78,212)	12.73 (7.40,19.20)	1.63 (1.55,1.69)
Liberia	2 (1,3)	0.17 (0.12,0.25)	2 (1,3)	0.93 (0.73,1.17)	-1.32 (-1.37,-1.27)	43 (28,64)	3.76 (2.52,5.55)	59 (38,90)	2.64 (1.71,3.87)	-1.14 (-1.19,-1.09)
Libya	29 (19,42)	1.76 (1.11,2.56)	86 (59,124)	0.12 (0.08,0.18)	0.38 (0.31,0.45)	659 (441,962)	36.49 (24.35,53.18)	2045 (1413,2934)	41.00 (28.28,58.79)	0.40 (0.32,0.47)
Lithuania	62 (51,72)	1.36 (1.13,1.59)	71 (57,86)	1.21 (0.85,1.66)	-0.82 (-1.44,-0.34)	1377 (1146,1595)	30.39 (25.33,35.01)	1412 (1158,1689)	24.79 (20.3,29.62)	-0.99 (-1.56,-0.53)
Luxembourg	10 (9,12)	1.85 (1.51,2.19)	10 (8,13)	0.72 (0.47,0.98)	-2.29 (-2.42,-2.21)	227 (188,267)	41.15 (33.95,48.57)	208 (163,257)	19.71 (15.53,24.26)	-2.44 (-2.67,-2.30)
Madagascar	10 (7,14)	0.26 (0.19,0.36)	9 (6,13)	0.46 (0.34,0.60)	-2.37 (-2.42,-2.32)	234 (168,314)	5.08 (3.64,6.88)	231 (148,324)	2.34 (1.54,3.37)	-2.46 (-2.51,-2.40)
Malawi	32 (24,41)	1.11 (0.83,1.45)	72 (49,98)	1.04 (0.71,1.49)	0.31 (0.26,0.34)	719 (538,935)	21.20 (15.95,27.27)	1671 (1104,2340)	24.50 (16.71,33.56)	0.48 (0.44,0.51)
Malaysia	67 (38,97)	0.84 (0.46,1.22)	175 (116,239)	0.79 (0.62,0.99)	-0.79 (-1.11,-0.47)	1443 (848,2007)	16.70 (9.69,23.37)	3698 (2511,4942)	13.73 (9.18,18.46)	-0.70 (-0.91,-0.39)
Maldives	0 (0,1)	0.78 (0.50,1.14)	1 (1,2)	0.37 (0.23,0.53)	-1.74 (-1.82,-1.61)	10 (7,15)	14.06 (9.17,20.65)	22 (16,30)	7.70 (5.65,10.15)	-2.01 (-2.11,-1.88)
Mali	22 (16,29)	0.75 (0.54,1.01)	67 (46,95)	0.16 (0.09,0.26)	1.07 (1.04,1.11)	498 (356,658)	14.15 (10.22,18.79)	1460 (1017,2109)	18.99 (13.12,27.13)	0.98 (0.95,1.01)
Malta	8 (7,10)	1.87 (1.53,2.23)	8 (6,10)	0.57 (0.48,0.67)	-2.69 (-2.94,-2.45)	181 (152,212)	41.62 (35.04,48.90)	174 (140,218)	18.81 (15.26,23.45)	-2.55 (-2.75,-2.36)
Marshall Isla	0 (0,0)	0.30 (0.20,0.45)	0 (0,0)	0.19 (0.15,0.24)	0.68 (0.59,0.78)	1 (1,2)	7.39 (4.84,11.13)	3 (2,5)	8.78 (5.55,12.80)	0.59 (0.52,0.68)
Mauritania	2 (1,3)	0.23 (0.16,0.33)	3 (2,5)	0.44 (0.29,0.62)	-1.20 (-1.26,-1.16)	51 (36,74)	5.17 (3.62,7.51)	73 (42,119)	3.38 (1.94,5.52)	-1.38 (-1.46,-1.31)
Mauritius	8 (7,9)	1.22 (1.04,1.42)	10 (9,12)	1.37 (0.61,3.39)	-2.46 (-2.90,-2.04)	185 (160,212)	25.97 (22.34,29.83)	224 (193,260)	11.99 (10.30,13.94)	-2.57 (-3.05,-2.10)
Mexico	154 (129,181)	0.44 (0.36,0.52)	225 (175,278)	0.30 (0.21,0.43)	-2.61 (-2.70,-2.49)	3223 (2734,3773)	8.16 (6.90,9.57)	4642 (3686,5753)	3.74 (2.96,4.64)	-2.48 (-2.58,-2.35)
Micronesia (F	0 (0,0)	0.43 (0.29,0.61)	0 (0,0)	1.49 (1.07,2.03)	0.09 (0.08,0.10)	5 (4,8)	10.76 (7.33,15.22)	10 (6,13)	11.38 (7.45,15.89)	0.18 (0.18,0.19)
Monaco	1 (1,2)	1.66 (1.00,2.43)	1 (1,4)	0.25 (0.17,0.36)	-0.63 (-0.64,-0.61)	25 (15,37)	36.47 (21.83,54.26)	28 (13,70)	29.09 (12.98,73.52)	-0.75 (-0.77,-0.74)
Mongolia	4 (3,6)	0.42 (0.26,0.61)	6 (5,9)	0.27 (0.19,0.38)	-1.11 (-1.18,-1.03)	109 (67,157)	10.32 (6.37,14.89)	178 (126,256)	7.30 (5.19,10.47)	-1.14 (-1.20,-1.06)
Montenegro	8 (6,11)	1.35 (0.99,1.80)	15 (11,20)	0.29 (0.20,0.49)	0.49 (0.40,0.59)	206 (154,268)	32.36 (24.11,42.25)	347 (256,468)	33.94 (25.02,45.59)	0.34 (0.22,0.47)
Morocco	41 (27,55)	0.31 (0.2,0.42)	81 (55,115)	0.27 (0.21,0.36)	-0.73 (-0.76,-0.70)	927 (645,1258)	6.68 (4.56,9.03)	1943 (1313,2739)	5.62 (3.84,8.01)	-0.56 (-0.58,-0.53)
Mozambique	13 (9,19)	0.28 (0.19,0.40)	24 (17,34)	0.48 (0.29,0.70)	-0.14 (-0.17,-0.10)	301 (204,438)	5.53 (3.72,7.93)	584 (422,828)	5.58 (4.03,7.93)	0.04 (0.01,0.07)
Myanmar	113 (83,156)	0.58 (0.43,0.80)	122 (83,199)	0.29 (0.19,0.57)	-2.18 (-2.20,-2.16)	2709 (1968,3741)	12.22 (8.99,16.83)	2654 (1818,4181)	5.73 (3.93,9.20)	-2.42 (-2.45,-2.40)
Namibia	1 (1,2)	0.31 (0.23,0.41)	3 (2,4)	1.16 (0.95,1.42)	-0.43 (-0.47,-0.40)	33 (24,43)	5.80 (4.32,7.52)	61 (45,80)	5.04 (3.82,6.62)	-0.43 (-0.48,-0.39)
Nauru	0 (0,0)	0.57 (0.35,0.81)	0 (0,0)	0.64 (0.49,0.81)	-0.53 (-0.54,-0.52)	1 (0,1)	13.49 (8.20,19.43)	1 (0,1)	12.35 (7.25,18.15)	-0.29 (-0.30,-0.28)
Nepal	29 (18,46)	0.42 (0.25,0.66)	56 (36,110)	0.09 (0.06,0.12)	-1.13 (-1.16,-1.11)	653 (413,1027)	7.93 (4.92,12.47)	1105 (703,2221)	5.18 (3.33,10.34)	-1.34 (-1.36,-1.31)
Netherlands	499 (423,569)	2.41 (2.04,2.74)	449 (368,552)	0.08 (0.05,0.15)	-2.34 (-2.45,-2.24)	10026 (8633,11313)	49.93 (43.07,56.16)	8096 (6725,9891)	22.14 (18.47,26.84)	-2.60 (-2.71,-2.49)
New Zealand	46 (38,55)	1.13 (0.94,1.35)	58 (45,74)	0.02 (0.01,0.04)	-1.84 (-1.96,-1.73)	978 (816,1148)	24.39 (20.41,28.58)	1064 (838,1320)	12.27 (9.77,15.16)	-2.20 (-2.34,-2.08)
Nicaragua	1 (1,2)	0.1 (0.07,0.13)	4 (3,5)	0.33 (0.22,0.47)	-0.51 (-0.70,-0.35)	30 (22,38)	2.04 (1.49,2.62)	85 (62,116)	1.78 (1.30,2.42)	-0.46 (-0.66,-0.28)
Niger	2 (1,3)	0.11 (0.06,0.17)	4 (3,8)	1.75 (1.28,2.29)	-0.72 (-0.75,-0.69)	47 (31,76)	1.95 (1.24,3.13)	96 (58,166)	1.41 (0.83,2.46)	-1.04 (-1.06,-1.02)
Nigeria	13 (9,19)	0.03 (0.02,0.05)	17 (11,27)	0.49 (0.38,0.64)	-1.49 (-1.51,-1.47)	279 (187,429)	0.68 (0.46,1.03)	398 (268,625)	0.45 (0.30,0.73)	-1.27 (-1.30,-1.25)
Niue	0 (0,0)	0.30 (0.20,0.40)	0 (0,0)	0.66 (0.50,0.82)	0.36 (0.34,0.37)	0 (0,0)	7.57 (5.02,10.21)	0 (0,0)	8.27 (5.38,11.75)	0.27 (0.26,0.29)
North Maced	32 (25,40)	1.80 (1.40,2.27)	56 (40,74)	0.22 (0.15,0.30)	-0.12 (-0.28,0.01)	763 (603,951)	40.33 (31.74,50.36)	1302 (936,1709)	37.94 (27.47,49.84)	-0.17 (-0.31,-0.06)
Northern Mar	0 (0,0)	0.35 (0.24,0.59)	0 (0,0)	1.25 (0.87,1.84)	1.05 (0.99,1.12)	2 (1,3)	8.51 (5.89,14.12)	7 (5,9)	11.87 (9.14,15.31)	1.10 (0.99,1.19)
Norway	137 (117,160)	1.85 (1.59,2.15)	73 (55,92)	0.12 (0.09,0.16)	-3.30 (-3.41,-3.22)	2678 (2330,3071)	38.83 (34.08,44.33)	1331 (1071,1617)	13.00 (10.54,15.71)	-3.46 (-3.55,-3.39)
Oman	2 (1,3)	0.31 (0.20,0.45)	3 (2,4)	1.11 (0.83,1.53)	-1.10 (-1.25,-0.93)	45 (29,67)	7.00 (4.46,10.24)	83 (58,112)	4.56 (3.13,6.22)	-1.35 (-1.42,-1.26)
Pakistan	758 (559,997)	1.55 (1.13,2.06)	1217 (858,1782)	0.14 (0.10,0.19)	-0.68 (-0.70,-0.66)	16379 (12332,2136	30.72 (22.9,39.94)	27682 (19440,40572	24.89 (17.55,36.4)	-0.68 (-0.70,-0.65)
Palau	0 (0,0)	0.15 (0.11,0.21)	0 (0,0)	0.16 (0.09,0.26)	-0.82 (-0.86,-0.77)	0 (0,1)	3.64 (2.58,4.97)	1 (1,1)	2.97 (2.09,4.12)	-0.66 (-0.71,-0.62)
Palestine	11 (8,16)	1.47 (1.00,2.04)	23 (17,31)	0.43 (0.30,0.60)	-0.90 (-0.98,-0.83)	248 (168,351)	29.98 (20.37,42.31)	580 (426,761)	23.78 (17.7,31.82)	-0.72 (-0.84,-0.64)
Panama	3 (2,4)	0.22 (0.18,0.26)	6 (5,9)	0.12 (0.08,0.19)	-1.25 (-1.43,-1.07)	62 (52,75)	4.33 (3.58,5.22)	127 (92,168)	2.88 (2.09,3.83)	-1.27 (-1.46,-1.07)
Papua New G	3 (2,5)	0.18 (0.10,0.28)	8 (4,12)	0.25 (0.19,0.37)	-0.23 (-0.29,-0.17)	86 (43,134)	4.39 (2.33,6.84)	238 (127,390)	4.17 (2.27,6.60)	-0.16 (-0.22,-0.10)
Paraguay	9 (7,12)	0.47 (0.36,0.60)	23 (16,32)	1.85 (1.52,2.22)	-0.21 (-0.36,-0.07)	182 (140,238)	8.70 (6.64,11.34)	455 (310,638)	8.16 (5.60,11.41)	-0.09 (-0.16,-0.00)
Peru	17 (11,23)	0.16 (0.11,0.22)	40 (25,61)	0.74 (0.59,0.90)	-0.67 (-0.88,-0.51)	340 (241,464)	3.00 (2.10,4.12)	779 (495,1189)	2.36 (1.49,3.61)	-0.65 (-0.87,-0.49)
Philippines	76 (59,98)	0.32 (0.25,0.42)	189 (144,271)	0.35 (0.26,0.47)	-0.75 (-0.82,-0.67)	1958 (1529,2512)	6.76 (5.22,8.71)	4814 (3606,6702)	5.77 (4.37,8.21)	-0.47 (-0.52,-0.43)
Poland	876 (766,981)	1.97 (1.72,2.21)	1398 (1142,1681)	0.55 (0.35,0.86)	-0.25 (-0.34,-0.16)	21753 (19191,2412	48.67 (42.93,54.1)	30413 (25065,36248	41.86 (34.61,49.81)	-0.46 (-0.57,-0.34)
Portugal	169 (141,200)	1.19 (1.00,1.42)	189 (147,235)	0.69 (0.48,0.90)	-1.52 (-1.65,-1.41)	3750 (3153,4402)	26.37 (22.25,30.84)	4089 (3261,4973)	17.96 (14.60,21.7)	-1.30 (-1.45,-1.20)
Puerto Rico	19 (14,24)	0.53 (0.40,0.68)	28 (20,37)	1.00 (0.82,1.17)	-1.33 (-1.52,-1.14)	387 (297,496)	10.65 (8.18,13.59)	541 (392,726)	7.75 (5.66,10.40)	-1.04 (-1.29,-0.82)
Qatar	1 (1,1)	1.12 (0.80,1.57)	3 (2,5)	1.31 (1.05,1.59)	-2.26 (-2.58,-1.94)	25 (17,36)	24.71 (17.42,34.66)	113 (68,174)	12.10 (7.49,18.82)	-2.40 (-2.64,-2.18)
Republic of K	292 (220,358)	1.22 (0.92,1.51)	644 (448,851)	0.89 (0.74,1.05)	-1.87 (-1.93,-1.82)	6909 (5284,8442)	24.72 (18.84,30.36)	11638 (8309,15106)	12.28 (8.78,15.93)	-2.25 (-2.30,-2.20)
Republic of M	42 (34,49)	0.95 (0.77,1.12)	61 (51,72)	0.57 (0.38,0.88)	0.06 (-0.18,0.29)	1094 (888,1278)	23.81 (19.23,27.86)	1565 (1299,1832)	25.57 (21.27,29.76)	0.17 (-0.17,0.52)
Romania	331 (279,388)	1.17 (0.98,1.37)	500 (397,612)	0.30 (0.22,0.42)	0.36 (0.23,0.50)	8658 (7299,10012)	29.63 (25.11,34.14)	11539 (9337,13962)	32.10 (26.09,38.68)	0.25 (0.10,0.40)
Russian Fede	1974 (1741,2214)	1.07 (0.94,1.20)	2197 (1816,2588)	0.40 (0.29,0.53)	-0.56 (-0.66,-0.45)	51541 (45654,5751	27.41 (24.25,30.60)	53476 (44416,62662	21.89 (18.23,25.60)	-0.71 (-0.82,-0.59)
Rwanda	15 (11,21)	0.68 (0.49,0.96)	27 (18,42)	0.33 (0.25,0.42)	-0.56 (-0.59,-0.54)	352 (251,489)	13.65 (9.71,18.91)	587 (377,930)	10.64 (7.04,16.54)	-0.79 (-0.84,-0.76)
Saint Kitts an	0 (0,0)	0.38 (0.29,0.50)	0 (0,0)	0.38 (0.26,0.53)	-0.78 (-0.86,-0.69)	3 (2,4)	8.11 (6.29,10.34)	4 (3,6)	6.02 (4.31,8.20)	-0.94 (-1.05,-0.80)
Saint Lucia	1 (0,1)	0.63 (0.49,0.81)	1 (1,1)	0.91 (0.57,1.36)	-1.52 (-1.73,-1.34)	11 (9,14)	13.02 (10.38,16.10)	20 (15,26)	8.24 (6.10,10.90)	-1.45 (-1.58,-1.32)
Saint Vincent	0 (0,0)	0.33 (0.26,0.41)	0 (0,1)	0.27 (0.17,0.42)	-0.10 (-0.29,0.03)	5 (4,6)	7.04 (5.61,8.66)	10 (8,13)	6.91 (5.38,8.77)	-0.05 (-0.19,0.06)

Samoa	0 (0,0)	0.45 (0.33,0.63)	1 (0,1)	0.27 (0.17,0.49)	-0.55 (-0.56,-0.53)	9 (6,13)	10.08 (7.36,14.55)	12 (8,18)	8.44 (5.73,12.01)	-0.58 (-0.59,-0.57)
San Marino	1 (1,1)	2.39 (1.74,3.15)	1 (0,1)	0.16 (0.10,0.24)	-3.49 (-3.97,-3.17)	18 (13,23)	49.41 (36.59,65.46)	14 (9,21)	19.44 (11.51,30.06)	-3.33 (-3.74,-3.08)
Sao Tome an	0 (0,0)	0.19 (0.13,0.26)	0 (0,0)	1.44 (1.03,1.93)	1.22 (1.15,1.30)	3 (2,4)	4.39 (3.13,6.01)	7 (4,11)	6.17 (3.88,9.36)	1.11 (1.04,1.17)
Saudi Arabia	15 (9,23)	0.29 (0.18,0.45)	45 (28,83)	1.07 (0.84,1.39)	-0.19 (-0.22,-0.17)	398 (241,621)	6.84 (4.22,10.63)	1430 (873,2719)	6.75 (4.20,12.57)	-0.05 (-0.07,-0.03)
Senegal	8 (5,10)	0.25 (0.17,0.33)	12 (8,17)	0.16 (0.11,0.23)	-1.45 (-1.56,-1.34)	202 (138,277)	6.02 (4.11,8.27)	309 (206,451)	3.81 (2.50,5.67)	-1.47 (-1.61,-1.32)
Serbia	169 (127,221)	1.66 (1.25,2.18)	248 (176,336)	0.20 (0.16,0.26)	-0.36 (-0.46,-0.25)	4146 (3146,5422)	36.59 (27.74,47.81)	5596 (3983,7549)	33.82 (24.38,45.25)	-0.22 (-0.32,-0.11)
Seychelles	1 (1,1)	1.51 (1.14,1.92)	1 (1,1)	1.08 (0.78,1.47)	-1.32 (-1.61,-1.14)	18 (14,23)	31.67 (24.47,40.34)	24 (19,32)	21.47 (16.84,28.10)	-1.43 (-1.65,-1.28)
Sierra Leone	5 (3,7)	0.26 (0.19,0.36)	5 (4,8)	0.90 (0.70,1.15)	-1.65 (-1.68,-1.62)	116 (80,156)	5.71 (3.96,7.68)	134 (93,197)	3.49 (2.46,5.06)	-1.56 (-1.58,-1.54)
Singapore	10 (8,12)	0.52 (0.41,0.62)	17 (13,22)	0.42 (0.24,0.63)	-3.03 (-3.41,-2.76)	248 (202,297)	11.50 (9.4,13.78)	367 (291,452)	4.21 (3.34,5.19)	-3.34 (-3.69,-3.09)
Slovakia	93 (72,121)	1.54 (1.20,2.00)	107 (77,147)	0.19 (0.11,0.31)	-1.11 (-1.19,-1.05)	2201 (1731,2843)	36.35 (28.56,46.99)	2416 (1753,3305)	24.76 (18.08,33.52)	-1.23 (-1.30,-1.16)
Slovenia	26 (21,31)	1.04 (0.86,1.25)	41 (31,53)	0.34 (0.27,0.42)	-0.53 (-0.74,-0.37)	659 (545,773)	26.56 (22.06,31.22)	912 (711,1159)	21.58 (16.81,27.11)	-0.77 (-1.00,-0.59)
Solomon Isla	0 (0,1)	0.41 (0.21,0.63)	1 (1,2)	0.24 (0.15,0.36)	0.06 (0.01,0.09)	14 (7,22)	9.80 (4.74,15.15)	39 (22,60)	10.50 (5.89,16.05)	0.21 (0.17,0.26)
Somalia	5 (3,8)	0.27 (0.16,0.46)	9 (5,15)	1.37 (1.09,1.66)	-1.06 (-1.09,-1.04)	125 (72,213)	5.72 (3.43,9.79)	244 (133,411)	4.18 (2.33,6.99)	-0.99 (-1.02,-0.97)
South Africa	103 (74,145)	0.55 (0.39,0.78)	148 (116,180)	0.17 (0.10,0.24)	-1.56 (-1.66,-1.47)	2513 (1833,3430)	12.11 (8.76,16.73)	3769 (2982,4565)	7.95 (6.29,9.66)	-1.39 (-1.51,-1.28)
South Sudan	7 (4,10)	0.30 (0.19,0.44)	7 (4,11)	0.55 (0.37,0.80)	-0.77 (-0.80,-0.74)	156 (101,238)	6.28 (4.10,9.52)	170 (105,271)	4.79 (2.97,7.38)	-0.85 (-0.88,-0.83)
Spain	1552 (1323,1783)	2.74 (2.35,3.15)	1456 (1159,1804)	0.32 (0.21,0.45)	-2.22 (-2.28,-2.17)	34254 (29354,3882	61.71 (52.95,69.76)	28693 (23198,35050	30.21 (24.65,36.34)	-2.32 (-2.39,-2.26)
Sri Lanka	26 (20,35)	0.31 (0.23,0.42)	42 (24,61)	0.77 (0.59,0.95)	-1.99 (-2.20,-1.80)	570 (430,760)	5.83 (4.39,7.76)	879 (484,1283)	3.27 (1.83,4.76)	-1.86 (-2.09,-1.68)
Sudan	57 (32,100)	0.68 (0.38,1.19)	91 (61,133)	0.87 (0.70,1.06)	-0.71 (-0.73,-0.70)	1395 (773,2458)	15.17 (8.54,26.71)	2226 (1476,3246)	11.77 (7.89,17.08)	-0.81 (-0.82,-0.79)
Suriname	1 (1,1)	0.41 (0.30,0.55)	2 (1,3)	0.76 (0.50,1.10)	-0.75 (-0.92,-0.57)	23 (18,30)	9.23 (6.91,12.08)	47 (31,66)	7.28 (4.81,10.26)	-0.67 (-0.83,-0.52)
Sweden	195 (162,232)	1.18 (0.99,1.40)	192 (145,240)	0.87 (0.71,1.02)	-1.35 (-1.46,-1.26)	3807 (3201,4430)	24.91 (21.11,28.93)	3423 (2687,4223)	15.22 (12.10,18.71)	-1.56 (-1.69,-1.47)
Switzerland	102 (85,120)	0.93 (0.77,1.09)	182 (144,223)	0.16 (0.11,0.24)	-0.25 (-0.47,-0.07)	2090 (1752,2435)	20.22 (16.93,23.47)	3234 (2620,3928)	17.18 (13.93,20.79)	-0.62 (-0.84,-0.45)
Syrian Arab R	39 (28,53)	0.85 (0.60,1.18)	83 (55,119)	0.59 (0.42,0.81)	-0.38 (-0.48,-0.29)	957 (684,1302)	18.68 (13.45,25.36)	2084 (1366,3010)	15.93 (10.52,22.77)	-0.50 (-0.59,-0.43)
Taiwan (Prov	178 (153,205)	1.28 (1.08,1.47)	382 (313,451)	0.26 (0.17,0.43)	-1.18 (-1.37,-0.98)	4352 (3725,4948)	27.66 (23.64,31.53)	7420 (6167,8646)	17.33 (14.43,20.11)	-1.49 (-1.69,-1.28)
Tajikistan	8 (5,13)	0.31 (0.20,0.52)	8 (5,12)	0.28 (0.19,0.40)	-2.12 (-2.26,-1.97)	200 (130,303)	7.22 (4.71,11.03)	218 (135,330)	3.66 (2.32,5.51)	-2.27 (-2.40,-2.13)
Thailand	300 (212,381)	1.03 (0.73,1.33)	639 (456,890)	0.30 (0.19,0.43)	-1.80 (-1.90,-1.69)	6752 (4822,8691)	20.47 (14.47,26.24)	13031 (9172,18369)	11.87 (8.39,16.75)	-1.78 (-1.88,-1.68)
Timor-Leste	1 (0,1)	0.28 (0.17,0.44)	2 (1,3)	0.44 (0.27,0.70)	-0.24 (-0.28,-0.20)	14 (8,23)	5.73 (3.40,9.10)	43 (28,68)	5.23 (3.36,8.23)	-0.28 (-0.31,-0.25)
Togo	5 (3,6)	0.46 (0.33,0.61)	9 (6,13)	0.30 (0.20,0.40)	-1.57 (-1.59,-1.54)	109 (77,148)	9.42 (6.74,12.72)	240 (164,347)	6.25 (4.27,8.89)	-1.32 (-1.35,-1.29)
Tokelau	0 (0,0)	0.36 (0.23,0.52)	0 (0,0)	1.35 (0.84,1.91)	-0.54 (-0.56,-0.51)	0 (0,0)	8.22 (5.17,11.98)	0 (0,0)	7.21 (4.50,10.26)	-0.44 (-0.47,-0.41)
Tonga	0 (0,0)	0.44 (0.27,0.73)	0 (0,1)	1.29 (0.94,1.76)	-0.07 (-0.14,0.01)	5 (3,8)	9.53 (5.85,15.52)	8 (5,12)	9.56 (5.67,15.53)	-0.01 (-0.08,0.06)
Trinidad and	3 (3,4)	0.43 (0.36,0.51)	6 (4,8)	0.26 (0.19,0.36)	-1.01 (-1.27,-0.73)	78 (64,91)	9.33 (7.72,10.88)	134 (94,179)	6.70 (4.67,8.94)	-0.76 (-0.99,-0.54)
Tunisia	65 (45,90)	1.53 (1.06,2.10)	165 (103,235)	0.36 (0.24,0.52)	-0.45 (-0.50,-0.40)	1402 (988,1952)	29.70 (20.91,40.97)	3535 (2210,5182)	27.03 (16.89,39.48)	-0.35 (-0.39,-0.31)
Turkey	616 (437,840)	1.94 (1.39,2.65)	1172 (852,1599)	0.20 (0.14,0.27)	-1.32 (-1.37,-1.26)	15554 (10965,2130	44.74 (31.78,61.1)	28109 (20166,3820	29.46 (21.19,40.03)	-1.32 (-1.36,-1.28)
Turkmenistan	8 (6,9)	0.41 (0.34,0.49)	10 (7,14)	0.87 (0.57,1.22)	-1.46 (-1.66,-1.26)	212 (179,251)	10.57 (8.92,12.64)	296 (210,407)	6.85 (4.88,9.40)	-1.34 (-1.94,-1.07)
Tuvalu	0 (0,0)	0.35 (0.24,0.48)	0 (0,0)	0.83 (0.58,1.16)	0.16 (0.15,0.16)	1 (0,1)	8.61 (5.79,11.76)	1 (1,1)	8.97 (6.01,12.41)	0.13 (0.13,0.14)
Uganda	13 (9,17)	0.24 (0.17,0.32)	23 (17,32)	1.12 (0.89,1.37)	-0.67 (-0.71,-0.63)	291 (200,401)	4.83 (3.35,6.58)	549 (397,789)	3.98 (2.90,5.58)	-0.65 (-0.69,-0.61)
Ukraine	765 (595,1017)	1.04 (0.82,1.37)	697 (454,972)	0.26 (0.17,0.38)	-0.51 (-0.75,-0.24)	18661 (14408,2504	25.13 (19.34,33.67)	16935 (10969,2387	21.80 (14.06,30.89)	-0.39 (-0.67,-0.08)
United Arab	4 (2,8)	1.16 (0.62,2.25)	17 (12,25)	0.80 (0.64,1.00)	-1.22 (-1.66,-0.76)	125 (67,241)	27.07 (14.13,52.66)	580 (405,815)	16.13 (11.23,22.67)	-1.68 (-2.13,-1.27)
United Kingd	2495 (2155,2844)	2.59 (2.24,2.95)	1633 (1288,2023)	0.16 (0.10,0.23)	-2.64 (-2.69,-2.59)	49982 (43517,5661	54.67 (47.62,61.77)	28486 (23272,3431	21.28 (17.62,25.31)	-2.97 (-3.02,-2.91)
United Repul	37 (26,53)	0.42 (0.30,0.59)	55 (37,80)	1.26 (1.02,1.52)	-1.56 (-1.59,-1.54)	873 (620,1257)	8.50 (6.07,12.22)	1277 (852,1871)	5.24 (3.53,7.58)	-1.56 (-1.58,-1.54)
United States	3587 (3037,4217)	1.08 (0.92,1.27)	4957 (3948,6196)	0.24 (0.18,0.33)	-0.97 (-1.02,-0.94)	82180 (69941,9451	25.98 (22.30,29.82)	107083 (87699,1296	18.13 (14.89,21.81)	-1.15 (-1.19,-1.12)
United States	0 (0,0)	0.27 (0.19,0.39)	0 (0,0)	0.23 (0.14,0.34)	-1.84 (-1.97,-1.73)	5 (4,7)	6.03 (4.21,8.54)	6 (4,10)	3.38 (2.16,4.98)	-1.93 (-2.07,-1.82)
Uruguay	66 (54,77)	1.63 (1.36,1.92)	73 (58,89)	0.26 (0.18,0.36)	-0.90 (-1.01,-0.78)	1533 (1294,1783)	38.88 (32.91,45.08)	1519 (1240,1819)	28.43 (23.21,33.76)	-1.10 (-1.22,-0.99)
Uzbekistan	18 (12,25)	0.16 (0.10,0.23)	59 (43,79)	0.39 (0.30,0.50)	1.29 (1.14,1.44)	480 (329,663)	4.08 (2.79,5.59)	1601 (1147,2116)	5.86 (4.24,7.78)	1.20 (1.04,1.36)
Vanuatu	0 (0,0)	0.29 (0.17,0.43)	0 (0,1)	0.85 (0.58,1.18)	-0.75 (-0.78,-0.71)	4 (2,7)	6.67 (3.90,10.05)	10 (6,15)	5.43 (3.29,8.28)	-0.70 (-0.73,-0.67)
Venezuela (B	34 (28,40)	0.39 (0.32,0.47)	74 (52,101)	0.32 (0.19,0.55)	-1.40 (-1.57,-1.23)	786 (646,922)	8.41 (6.92,9.88)	1605 (1131,2225)	5.37 (3.80,7.38)	-1.50 (-1.71,-1.27)
Viet Nam	138 (94,183)	0.37 (0.25,0.49)	351 (266,447)	1.53 (1.13,2.04)	0.18 (0.16,0.20)	3038 (2082,4114)	7.69 (5.30,10.3)	8296 (6134,10720)	8.40 (6.30,10.8)	0.29 (0.28,0.31)
Yemen	36 (21,57)	0.81 (0.47,1.27)	102 (69,141)	0.57(0.31,1.26)	0.12 (0.08,0.17)	978 (563,1551)	19.48 (11.24,30.82)	2582 (1701,3611)	18.70 (12.50,25.95)	-0.15 (-0.20,-0.10)
Zambia	7 (6,10)	0.33 (0.25,0.44)	17 (10,32)	0.82 (0.62,1.01)	-0.05 (-0.09,-0.02)	174 (130,234)	6.71 (5.12,8.96)	424 (234,836)	6.64 (3.78,12.16)	-0.02 (-0.05,0.01)
Zimbabwe	51 (38,67)	1.54 (1.13,2.04)	84 (62,114)	0.36 (0.16,0.46)	0.05 (-0.02,0.11)	1171 (854,1547)	30.82 (22.78,40.54)	2109 (1547,2893)	32.09 (23.78,43.23)	0.16 (0.11,0.21)
Afghanistan	6 (3,12)	0.09 (0.04,0.17)	16 (9,27)	0.16 (0.09,0.27)	1.89 (1.87,1.91)	234 (168,314)	2.27 (1.13,4.42)	506 (274,884)	4.16 (2.37,7.25)	1.98 (1.96,2.00)
Albania	17 (13,22)	0.87 (0.68,1.10)	52 (36,73)	1.17 (0.81,1.64)	0.90 (0.79,1.00)	719 (538,935)	20.5 (16.03,26.45)	1165 (800,1659)	26.94 (18.52,38.22)	0.81 (0.69,0.91)
Algeria	24 (19,31)	0.23 (0.18,0.29)	83 (59,111)	0.26 (0.19,0.35)	0.42 (0.40,0.45)	1443 (848,2007)	4.96 (3.86,6.31)	2022 (1461,2693)	5.61 (4.02,7.51)	0.39 (0.36,0.41)
American Sa	0 (0,0)	0.62 (0.48,0.77)	0 (0,1)	0.80 (0.63,1.00)	0.88 (0.77,0.98)	10 (7,15)	16.73 (13.23,20.78)	12 (9,15)	21.99 (17.31,27.96)	0.90 (0.80,1.00)
Andorra	2 (1,2)	2.69 (1.85,3.79)	2 (2,3)	1.59 (1.02,2.23)	-1.83 (-2.04,-1.67)	498 (356,658)	68.30 (46.44,96.70)	60 (38,85)	39.6 (25.07,56.06)	-1.89 (-2.09,-1.72)
Angola	9 (7,13)	0.24 (0.17,0.33)	30 (20,43)	0.24 (0.16,0.35)	0.14 (0.08,0.20)	181 (152,212)	6.43 (4.54,8.96)	932 (597,1329)	6.61 (4.31,9.43)	0.10 (0.04,0.16)

Antigua and I	0 (0,0)	0.37 (0.30,0.45)	0 (0,1)	0.46 (0.38,0.56)	0.68 (0.53,0.86)	1 (1,2)	9.43 (7.69,11.10)	13 (11,15)	11.17 (9.35,13.12)	0.55 (0.41,0.71)
Argentina	533 (465,611)	1.62 (1.42,1.86)	734 (630,851)	1.33 (1.14,1.54)	-0.60 (-0.69,-0.53)	51 (36,74)	44.42 (39.20,50.45)	19006 (16493,21773	35.33 (30.72,40.37)	-0.71 (-0.80,-0.63)
Armenia	37 (31,45)	1.31 (1.10,1.60)	84 (68,101)	1.90 (1.55,2.29)	1.22 (1.06,1.38)	185 (160,212)	36.49 (30.26,44.32)	2137 (1737,2564)	49.42 (40.23,59.25)	1.10 (0.94,1.27)
Australia	216 (192,244)	1.09 (0.97,1.24)	306 (250,374)	0.69 (0.57,0.84)	-1.45 (-1.58,-1.36)	3223 (2734,3773)	27.84 (24.95,31.10)	7091 (5993,8357)	17.52 (14.88,20.68)	-1.49 (-1.58,-1.42)
Austria	157 (137,177)	1.38 (1.22,1.55)	245 (212,280)	1.42 (1.23,1.61)	0.11 (-0.00,0.21)	5 (4,8)	36.77 (32.89,40.79)	5757 (5028,6503)	35.99 (31.63,40.63)	-0.08 (-0.18,0.02)
Azerbaijan	23 (17,33)	0.44 (0.32,0.63)	77 (52,110)	0.73 (0.50,1.01)	1.61 (1.52,1.70)	25 (15,37)	12.96 (9.37,18.45)	2237 (1482,3281)	19.24 (12.92,27.89)	1.27 (1.14,1.39)
Bahamas	1 (0,1)	0.32 (0.26,0.40)	1 (1,2)	0.34 (0.25,0.44)	0.25 (0.14,0.34)	109 (67,157)	8.83 (7.05,10.88)	40 (29,51)	8.97 (6.66,11.46)	0.13 (-0.00,0.24)
Bahrain	1 (1,2)	0.95 (0.76,1.16)	7 (5,10)	0.87 (0.64,1.20)	-0.25 (-0.33,-0.18)	206 (154,268)	21.93 (17.41,26.78)	192 (137,284)	18.91 (13.88,26.94)	-0.45 (-0.51,-0.41)
Bangladesh	107 (60,150)	0.24 (0.13,0.33)	302 (201,443)	0.22 (0.15,0.33)	-0.02 (-0.11,0.05)	927 (645,1258)	6.00 (3.34,8.45)	7563 (5007,11370)	5.33 (3.53,7.92)	-0.28 (-0.34,-0.22)
Barbados	1 (1,2)	0.49 (0.40,0.58)	2 (2,3)	0.43 (0.31,0.57)	-0.29 (-0.40,-0.18)	301 (204,438)	12.19 (10.13,14.37)	51 (37,67)	10.10 (7.32,13.39)	-0.42 (-0.52,-0.32)
Belarus	136 (121,153)	1.04 (0.92,1.16)	209 (165,259)	1.31 (1.04,1.62)	0.69 (0.48,0.89)	2709 (1968,3741)	29.28 (26.11,32.58)	5830 (4601,7284)	37.65 (29.77,47.07)	0.83 (0.55,1.12)
Belgium	255 (225,288)	1.66 (1.46,1.86)	281 (242,331)	1.25 (1.08,1.45)	-1.02 (-1.18,-0.90)	33 (24,43)	40.40 (36.18,45.09)	6314 (5500,7257)	30.88 (27.09,35.18)	-0.94 (-1.09,-0.80)
Belize	0 (0,0)	0.32 (0.25,0.44)	1 (1,2)	0.45 (0.37,0.55)	1.19 (1.01,1.36)	1 (0,1)	8.34 (6.65,11.27)	38 (31,45)	11.68 (9.54,14.05)	1.14 (0.98,1.32)
Benin	2 (2,3)	0.11 (0.08,0.14)	5 (4,7)	0.10 (0.08,0.14)	-0.10 (-0.14,-0.07)	653 (413,1027)	2.75 (2.11,3.49)	147 (105,202)	2.66 (1.91,3.61)	-0.10 (-0.14,-0.07)
Bermuda	1 (0,1)	1.07 (0.76,1.37)	1 (1,1)	0.84 (0.65,1.08)	-0.95 (-1.08,-0.82)	10026 (8633,11313)	26.29 (18.89,32.93)	26 (20,33)	20.43 (15.82,25.91)	-0.98 (-1.16,-0.84)
Bhutan	0 (0,0)	0.08 (0.04,0.14)	1 (0,1)	0.12 (0.07,0.18)	1.10 (1.06,1.14)	978 (816,1148)	2.09 (0.92,3.50)	16 (9,25)	2.67 (1.50,4.08)	0.79 (0.75,0.83)
Bolivia (Pluri	15 (10,20)	0.48 (0.32,0.63)	47 (31,68)	0.52 (0.34,0.75)	0.28 (0.23,0.33)	30 (22,38)	12.24 (8.34,16.30)	1222 (797,1767)	12.80 (8.36,18.50)	0.16 (0.11,0.20)
Bosnia and H	52 (44,62)	1.25 (1.04,1.48)	103 (79,129)	1.64 (1.26,2.07)	0.99 (0.90,1.08)	47 (31,76)	33.29 (27.78,39.61)	2512 (1929,3207)	41.87 (32.23,53.81)	0.86 (0.76,0.96)
Botswana	3 (2,4)	0.54 (0.40,0.74)	9 (6,13)	0.63 (0.44,0.85)	0.46 (0.41,0.51)	279 (187,429)	13.96 (10.22,19.34)	251 (165,371)	15.55 (10.57,22.17)	0.35 (0.30,0.42)
Brazil	930 (835,1030)	1.06 (0.95,1.19)	1880 (1594,2193)	0.74 (0.63,0.87)	-1.09 (-1.14,-1.04)	0 (0,0)	26.87 (24.26,29.56)	47631 (41215,54386	18.48 (15.96,21.14)	-1.17 (-1.22,-1.13)
Brunei Darus	1 (1,1)	0.93 (0.72,1.17)	2 (2,3)	0.62 (0.48,0.78)	-1.36 (-1.48,-1.26)	763 (603,951)	22.98 (17.85,28.71)	61 (47,76)	15.26 (11.89,18.98)	-1.35 (-1.45,-1.27)
Bulgaria	171 (148,198)	1.37 (1.19,1.58)	219 (174,268)	1.69 (1.34,2.06)	0.74 (0.61,0.85)	2 (1,3)	41.91 (36.29,48.01)	6036 (4793,7380)	50.17 (40.18,61.24)	0.61 (0.47,0.73)
Burkina Faso	3 (2,4)	0.06 (0.05,0.09)	8 (6,11)	0.09 (0.06,0.12)	1.03 (1.00,1.06)	2678 (2330,3071)	1.77 (1.24,2.46)	250 (169,344)	2.40 (1.64,3.30)	1.02 (0.99,1.04)
Burundi	5 (4,6)	0.21 (0.16,0.27)	5 (3,8)	0.11 (0.07,0.17)	-2.14 (-2.17,-2.12)	45 (29,67)	5.66 (4.11,7.38)	156 (98,255)	2.84 (1.82,4.53)	-2.18 (-2.22,-2.16)
Cabo Verde	0 (0,0)	0.04 (0.03,0.05)	2 (1,2)	0.34 (0.22,0.46)	7.22 (6.93,7.44)	16379 (12332,2136	1.06 (0.84,1.31)	43 (30,58)	8.9 (6.07,12.13)	7.13 (6.89,7.33)
Cambodia	22 (15,31)	0.49 (0.34,0.69)	72 (52,94)	0.59 (0.43,0.76)	0.63 (0.61,0.64)	0 (0,1)	12.67 (8.74,18.11)	1977 (1400,2646)	14.74 (10.58,19.38)	0.51 (0.49,0.53)
Cameroon	7 (5,9)	0.15 (0.11,0.19)	24 (16,36)	0.18 (0.12,0.27)	0.74 (0.72,0.77)	248 (168,351)	4.06 (3.03,5.42)	751 (508,1106)	5.05 (3.43,7.51)	0.70 (0.67,0.72)
Canada	557 (496,628)	1.71 (1.52,1.92)	739 (607,878)	1.01 (0.84,1.19)	-1.78 (-1.91,-1.70)	62 (52,75)	41.74 (37.43,46.69)	16013 (13477,18601	23.57 (20.04,27.21)	-1.86 (-1.99,-1.77)
Central Afric	2 (2,3)	0.19 (0.13,0.25)	3 (2,5)	0.14 (0.10,0.20)	-1.00 (-1.03,-0.98)	86 (43,134)	5.33 (3.57,7.24)	109 (72,160)	3.94 (2.67,5.63)	-0.97 (-1.00,-0.94)
Chad	2 (2,3)	0.08 (0.06,0.11)	7 (5,9)	0.12 (0.08,0.17)	1.35 (1.32,1.38)	182 (140,238)	2.00 (1.39,2.67)	192 (132,266)	3.05 (2.12,4.18)	1.40 (1.36,1.43)
Chile	74 (64,86)	0.73 (0.62,0.84)	174 (144,205)	0.68 (0.57,0.80)	-0.29 (-0.46,-0.12)	340 (241,464)	20.03 (17.28,23.17)	4783 (4066,5476)	19.06 (16.26,21.81)	-0.11 (-0.27,0.03)
China	8331 (6830,10121	0.99 (0.82,1.20)	23303 (17697,2982	1.09 (0.83,1.38)	0.28 (0.21,0.34)	1958 (1529,2512)	26.63 (21.68,32.39)	601155 (450656,771	27.67 (20.76,35.36)	0.10 (0.04,0.15)
Colombia	105 (92,121)	0.59 (0.52,0.68)	170 (133,216)	0.31 (0.24,0.39)	-2.20 (-2.35,-2.06)	21753 (19191,2412	15.93 (13.99,18.33)	4400 (3475,5555)	7.91 (6.25,9.99)	-2.32 (-2.52,-2.14)
Comoros	1 (0,1)	0.28 (0.19,0.38)	1 (1,2)	0.24 (0.17,0.36)	-0.58 (-0.65,-0.50)	3750 (3153,4402)	6.95 (4.67,9.48)	29 (19,45)	5.66 (3.79,8.73)	-0.70 (-0.79,-0.61)
Congo	3 (2,4)	0.25 (0.18,0.35)	8 (5,11)	0.30 (0.19,0.41)	0.54 (0.51,0.59)	387 (297,496)	6.72 (4.48,9.45)	238 (150,335)	7.62 (4.85,10.70)	0.43 (0.40,0.47)
Cook Islands	0 (0,0)	0.61 (0.49,0.74)	0 (0,0)	0.57 (0.44,0.71)	-0.23 (-0.29,-0.18)	25 (17,36)	15.99 (12.54,19.28)	4 (3,5)	14.94 (11.47,19.08)	-0.20 (-0.25,-0.14)
Costa Rica	10 (8,11)	0.56 (0.47,0.64)	29 (23,35)	0.53 (0.43,0.64)	-0.15 (-0.47,0.16)	6909 (5284,8442)	13.64 (11.64,15.61)	705 (573,849)	12.71 (10.34,15.32)	-0.30 (-0.59,0.01)
Côte d'Ivoire	105 (92,121)	1.73 (1.51,1.99)	141 (117,169)	1.58 (1.30,1.88)	0.29 (0.24,0.33)	1094 (888,1278)	44.15 (38.48,49.89)	3142 (2572,3725)	38.40 (31.53,45.39)	0.30 (0.25,0.33)
Croatia	110 (95,126)	1.07 (0.92,1.22)	153 (124,185)	0.78 (0.64,0.94)	-0.26 (-0.51,0.00)	8658 (7299,10012)	25.94 (22.71,29.22)	3939 (3234,4722)	20.62 (16.93,24.67)	-0.25 (-0.49,-0.07)
Cuba	8 (6,10)	1.06 (0.80,1.34)	23 (17,29)	1.08 (0.81,1.37)	-1.10 (-1.23,-0.89)	51541 (45654,5751	24.02 (18.44,30.16)	529 (395,667)	26.16 (19.66,32.96)	-0.70 (-0.84,-0.55)
Cyprus	314 (274,357)	2.27 (1.98,2.57)	379 (311,459)	1.81 (1.49,2.18)	-0.02 (-0.10,0.05)	352 (251,489)	61.59 (54.21,69.51)	9021 (7481,10845)	46.36 (38.48,55.16)	0.24 (0.16,0.30)
Czechia	4 (3,5)	0.09 (0.06,0.11)	11 (7,16)	0.10 (0.07,0.14)	-0.72 (-0.82,-0.63)	3 (2,4)	2.33 (1.70,3.08)	340 (224,510)	2.57 (1.73,3.76)	-0.89 (-1.01,-0.79)
Democratic P	97 (68,137)	0.55 (0.40,0.78)	192 (128,269)	0.56 (0.37,0.78)	0.02 (0.01,0.03)	11 (9,14)	16.28 (11.49,23.06)	5867 (3889,8437)	16.7 (11.22,23.79)	0.08 (0.07,0.09)
Democratic R	23 (16,31)	0.14 (0.1,0.19)	45 (27,68)	0.12 (0.07,0.18)	-0.67 (-0.71,-0.64)	5 (4,6)	3.81 (2.70,5.08)	1371 (839,2072)	3.16 (1.93,4.80)	-0.59 (-0.64,-0.54)
Denmark	153 (134,177)	1.89 (1.66,2.17)	225 (192,263)	1.82 (1.57,2.11)	-0.03 (-0.22,0.19)	9 (6,13)	45.33 (40.17,51.17)	4569 (3950,5273)	40.22 (34.69,46.01)	-0.39 (-0.61,-0.19)
Djibouti	0 (0,1)	0.30 (0.18,0.46)	2 (1,4)	0.39 (0.24,0.59)	0.77 (0.74,0.79)	18 (13,23)	7.68 (4.48,11.83)	67 (40,109)	9.36 (5.77,14.77)	0.64 (0.61,0.67)
Dominica	0 (0,0)	0.48 (0.37,0.61)	1 (0,1)	0.65 (0.48,0.87)	0.97 (0.94,1.01)	3 (2,4)	12.13 (9.63,15.18)	14 (11,20)	16.62 (12.34,22.54)	1.04 (1.01,1.07)
Dominican R	19 (14,24)	0.54 (0.42,0.69)	78 (60,105)	0.80 (0.61,1.06)	1.23 (1.10,1.35)	398 (241,621)	12.50 (9.58,15.79)	1893 (1458,2526)	18.73 (14.45,25.02)	1.40 (1.29,1.47)
Ecuador	20 (17,23)	0.39 (0.34,0.45)	58 (42,76)	0.35 (0.26,0.47)	-0.57 (-0.81,-0.39)	202 (138,277)	9.59 (8.32,10.89)	1418 (1021,1882)	8.55 (6.15,11.36)	-0.64 (-0.92,-0.44)
Egypt	96 (81,114)	0.35 (0.30,0.42)	559 (445,715)	0.90 (0.72,1.13)	3.06 (2.96,3.15)	4146 (3146,5422)	9.34 (7.87,11.12)	16039 (12631,20472	22.44 (17.90,28.45)	2.84 (2.74,2.93)
El Salvador	4 (3,5)	0.14 (0.11,0.17)	17 (12,22)	0.28 (0.21,0.37)	2.42 (2.23,2.63)	18 (14,23)	3.95 (3.24,4.72)	463 (344,610)	7.72 (5.75,10.19)	2.43 (2.25,2.65)
Equatorial Gu	0 (0,1)	0.19 (0.13,0.26)	2 (1,3)	0.33 (0.21,0.48)	1.84 (1.78,1.89)	116 (80,156)	5.30 (3.48,7.34)	52 (31,78)	8.82 (5.33,12.89)	1.69 (1.62,1.74)
Eritrea	1 (1,2)	0.1 (0.07,0.13)	2 (1,4)	0.08 (0.04,0.12)	-0.77 (-0.81,-0.73)	248 (202,297)	2.95 (2.11,3.90)	84 (46,137)	2.28 (1.30,3.64)	-0.87 (-0.92,-0.82)
Estonia	27 (24,31)	1.31 (1.16,1.5)	35 (28,41)	1.40 (1.14,1.64)	0.25 (-0.24,0.64)	2201 (1731,2843)	37.83 (33.27,42.97)	862 (698,1008)	37.68 (30.63,44.05)	0.06 (-0.36,0.42)
Eswatini	1 (1,2)	0.45 (0.28,0.66)	3 (2,4)	0.50 (0.29,0.78)	0.36 (0.31,0.41)	659 (545,773)	10.96 (6.93,16.66)	75 (43,123)	12.39 (7.28,19.95)	0.44 (0.40,0.48)

Ethiopia	9 (4,15)	0.04 (0.02,0.07)	16 (9,25)	0.04 (0.02,0.06)	-0.55 (-0.59,-0.52)	14 (7,22)	1.18 (0.58,1.99)	427 (253,689)	0.93 (0.55,1.48)	-0.78 (-0.81,-0.76)
Fiji	2 (1,2)	0.42 (0.32,0.53)	3 (2,5)	0.42 (0.29,0.58)	-0.02 (-0.11,0.07)	125 (72,213)	11.46 (8.87,14.30)	99 (68,137)	11.33 (7.87,15.61)	-0.04 (-0.14,0.06)
Finland	93 (79,107)	1.32 (1.14,1.52)	137 (113,165)	1.15 (0.96,1.36)	-0.46 (-0.57,-0.38)	2513 (1833,3430)	35.68 (31.39,40.44)	3115 (2614,3670)	29.27 (24.86,33.92)	-0.66 (-0.77,-0.56)
France	1002 (879,1141)	1.25 (1.10,1.4)	1722 (1445,2036)	1.33 (1.13,1.54)	0.21 (0.15,0.26)	156 (101,238)	32.35 (28.70,36.09)	40002 (34116,46100	34.47 (29.84,39.40)	0.24 (0.17,0.31)
Gabon	1 (1,2)	0.23 (0.15,0.35)	4 (3,5)	0.35 (0.23,0.47)	1.33 (1.30,1.36)	34254 (29354,3882	6.27 (4.19,9.54)	112 (75,155)	9.36 (6.26,12.80)	1.31 (1.27,1.35)
Gambia	1 (0,1)	0.14 (0.10,0.19)	1 (1,2)	0.14 (0.10,0.19)	0.07 (-0.04,0.19)	570 (430,760)	3.91 (2.84,5.18)	41 (28,56)	3.84 (2.69,5.13)	0.04 (-0.08,0.18)
Georgia	23 (18,31)	0.35 (0.27,0.46)	66 (56,78)	1.15 (0.97,1.35)	4.21 (3.86,4.54)	1395 (773,2458)	10.44 (8.20,13.82)	1794 (1520,2101)	32.72 (27.76,38.22)	3.89 (3.54,4.22)
Germany	1864 (1608,2107)	1.48 (1.28,1.67)	2764 (2370,3235)	1.50 (1.30,1.74)	0.03 (-0.05,0.10)	23 (18,30)	37.31 (33,41.79)	61876 (53432,71023	37.19 (32.26,42.05)	-0.01 (-0.09,0.06)
Ghana	5 (3,7)	0.08 (0.05,0.12)	28 (19,43)	0.18 (0.12,0.27)	2.67 (2.63,2.70)	3807 (3201,4430)	1.94 (1.28,2.83)	767 (511,1176)	4.30 (2.89,6.57)	2.62 (2.59,2.65)
Greece	298 (269,330)	1.94 (1.76,2.16)	454 (395,519)	2.01 (1.78,2.28)	0.06 (-0.04,0.15)	2090 (1752,2435)	47.56 (43.32,52.18)	9895 (8796,11149)	50.02 (44.85,56)	0.11 (0.01,0.18)
Greenland	2 (1,2)	4.55 (3.68,5.45)	2 (2,3)	3.15 (2.46,4.00)	-1.06 (-1.15,-0.97)	957 (684,1302)	121.06 (99.10,143.98)	66 (52,81)	83.20 (66.05,102.38)	-1.15 (-1.23,-1.08)
Grenada	0 (0,0)	0.58 (0.47,0.71)	1 (1,1)	0.75 (0.59,0.93)	0.89 (0.75,1.02)	4352 (3725,4948)	15.80 (12.95,19.13)	24 (19,30)	19.73 (15.62,24.18)	0.79 (0.66,0.91)
Guam	0 (0,0)	0.44 (0.36,0.53)	1 (1,1)	0.52 (0.42,0.64)	0.50 (0.32,0.64)	200 (130,303)	12.05 (10.07,14.47)	33 (27,41)	15.81 (12.74,19.25)	0.83 (0.69,0.94)
Guatemala	5 (4,6)	0.16 (0.14,0.19)	25 (19,31)	0.23 (0.18,0.28)	0.82 (0.59,1.04)	6752 (4822,8691)	3.91 (3.38,4.56)	636 (506,793)	5.62 (4.44,7.01)	0.84 (0.59,1.07)
Guinea	3 (2,4)	0.09 (0.06,0.12)	7 (5,10)	0.13 (0.09,0.18)	1.29 (1.26,1.31)	14 (8,23)	2.20 (1.59,2.97)	199 (139,288)	3.29 (2.31,4.74)	1.32 (1.29,1.35)
Guinea-Bissa	0 (0,0)	0.08 (0.04,0.11)	1 (1,1)	0.12 (0.08,0.17)	1.49 (1.46,1.52)	109 (77,148)	2.10 (1.20,3.11)	30 (19,42)	3.39 (2.2,4.76)	1.59 (1.55,1.62)
Guyana	1 (1,1)	0.31 (0.25,0.38)	3 (2,4)	0.40 (0.30,0.54)	1.03 (0.82,1.20)	0 (0,0)	8.59 (7.07,10.51)	81 (59,109)	11.32 (8.32,15.25)	1.14 (0.90,1.33)
Haiti	10 (7,13)	0.29 (0.20,0.40)	15 (10,22)	0.21 (0.14,0.31)	-0.92 (-0.95,-0.89)	5 (3,8)	8.08 (5.40,11.20)	446 (300,629)	5.52 (3.72,7.90)	-1.16 (-1.20,-1.13)
Honduras	6 (5,7)	0.29 (0.23,0.36)	30 (22,41)	0.47 (0.35,0.66)	1.63 (1.59,1.68)	78 (64,91)	7.62 (6.08,9.56)	785 (570,1090)	11.79 (8.61,16.34)	1.49 (1.44,1.53)
Hungary	257 (226,294)	1.76 (1.55,2.01)	291 (238,348)	1.60 (1.31,1.91)	-0.36 (-0.48,-0.28)	1402 (988,1952)	51.33 (45.13,58.22)	7558 (6190,9009)	44.40 (36.66,52.75)	-0.48 (-0.67,-0.35)
Iceland	5 (4,6)	1.75 (1.51,1.99)	7 (5,8)	1.14 (0.94,1.38)	-1.39 (-1.54,-1.29)	15554 (10965,2130	41.74 (36.36,47.18)	147 (124,176)	26.94 (22.72,31.94)	-1.35 (-1.44,-1.26)
India	777 (594,974)	0.17 (0.13,0.21)	1901 (1572,2252)	0.16 (0.13,0.19)	-0.10 (-0.16,-0.01)	212 (179,251)	4.41 (3.37,5.50)	49334 (40578,58296	3.96 (3.26,4.69)	-0.34 (-0.40,-0.28)
Indonesia	322 (267,395)	0.33 (0.27,0.40)	1392 (1006,1835)	0.58 (0.41,0.76)	1.85 (1.83,1.87)	1 (0,1)	8.70 (7.22,10.63)	39919 (28803,53236	14.73 (10.69,19.53)	1.71 (1.70,1.73)
Iran (Islamic	58 (46,72)	0.22 (0.18,0.27)	304 (254,355)	0.39 (0.32,0.46)	1.86 (1.78,1.92)	291 (200,401)	5.80 (4.64,7.14)	8395 (7084,9775)	9.96 (8.40,11.64)	1.72 (1.62,1.80)
Iraq	47 (32,65)	0.61 (0.41,0.84)	189 (133,246)	0.83 (0.59,1.09)	1.04 (0.99,1.09)	18661 (14408,2504	15.89 (10.77,21.86)	5213 (3646,6863)	20.09 (14.06,26.06)	0.77 (0.73,0.81)
Ireland	92 (83,102)	2.22 (2.00,2.47)	83 (68,98)	1.03 (0.86,1.21)	-2.43 (-2.56,-2.36)	125 (67,241)	50.55 (45.96,55.81)	1795 (1506,2091)	23.46 (19.71,27.13)	-2.47 (-2.62,-2.38)
Israel	74 (63,84)	1.52 (1.32,1.74)	137 (115,160)	1.12 (0.95,1.30)	-0.98 (-1.09,-0.86)	49982 (43517,5661	37.23 (32.99,41.69)	3120 (2699,3583)	26.98 (23.5,30.78)	-0.98 (-1.12,-0.85)
Italy	1448 (1298,1604)	1.64 (1.48,1.81)	1640 (1370,1923)	1.18 (1.01,1.36)	-1.08 (-1.17,-1.02)	873 (620,1257)	42.21 (38.39,46.03)	35769 (30901,40872	28.93 (25.53,32.70)	-1.26 (-1.37,-1.19)
Jamaica	7 (6,8)	0.39 (0.32,0.47)	13 (9,18)	0.42 (0.31,0.58)	0.55 (0.09,1.22)	82180 (69941,9451	10.04 (8.31,12.29)	327 (236,453)	10.63 (7.69,14.72)	0.53 (0.03,1.25)
Japan	2962 (2706,3243)	1.73 (1.58,1.89)	4369 (3642,5115)	1.27 (1.09,1.43)	-1.05 (-1.10,-1.00)	5 (4,7)	42.55 (39.14,46.18)	86876 (74807,98471	30.38 (26.84,33.80)	-1.11 (-1.15,-1.06)
Jordan	6 (5,8)	0.47 (0.37,0.59)	46 (34,62)	0.63 (0.46,0.83)	0.92 (0.83,1.01)	1533 (1294,1783)	11.97 (9.42,14.85)	1316 (959,1791)	15.44 (11.26,20.67)	0.83 (0.76,0.89)
Kazakhstan	85 (64,119)	0.62 (0.47,0.87)	132 (108,157)	0.67 (0.55,0.80)	0.25 (0.12,0.41)	480 (329,663)	19.27 (14.38,27.43)	4067 (3298,4854)	20.05 (16.31,23.8)	0.15 (0.00,0.33)
Kenya	11 (7,16)	0.14 (0.09,0.21)	44 (33,57)	0.20 (0.15,0.25)	1.04 (0.99,1.09)	4 (2,7)	3.50 (2.29,5.07)	1247 (934,1633)	4.94 (3.70,6.40)	1.14 (1.09,1.19)
Kiribati	0 (0,0)	0.20 (0.16,0.24)	0 (0,0)	0.24 (0.17,0.33)	0.66 (0.63,0.69)	786 (646,922)	5.23 (4.18,6.41)	5 (4,7)	6.33 (4.42,8.82)	0.63 (0.61,0.66)
Kuwait	3 (2,3)	0.43 (0.35,0.51)	17 (13,21)	0.59 (0.46,0.77)	1.01 (0.52,1.76)	3038 (2082,4114)	10.96 (9.09,12.79)	483 (367,612)	13.73 (10.53,17.54)	0.70 (0.18,1.52)
Kyrgyzstan	18 (15,22)	0.59 (0.48,0.71)	38 (30,48)	0.74 (0.59,0.93)	0.49 (0.04,0.87)	978 (563,1551)	17.30 (13.90,20.89)	1183 (936,1495)	21.11 (16.63,26.51)	0.37 (-0.09,0.74)
Lao People's	9 (5,14)	0.43 (0.26,0.67)	24 (17,32)	0.52 (0.37,0.69)	0.64 (0.62,0.66)	174 (130,234)	11.51 (6.81,17.97)	671 (465,897)	13.21 (9.26,17.74)	0.46 (0.44,0.48)
Latvia	49 (43,56)	1.36 (1.18,1.56)	55 (45,67)	1.57 (1.29,1.89)	0.42 (-0.05,0.80)	1171 (854,1547)	38.98 (34.05,44.28)	1481 (1215,1777)	45.85 (37.70,54.88)	0.45 (0.02,0.79)
Lebanon	14 (9,19)	0.65 (0.42,0.87)	55 (39,76)	0.92 (0.65,1.26)	1.11 (1.04,1.18)	1277 (918,1734)	16.47 (10.50,22.09)	1277 (918,1734)	21.95 (15.80,29.77)	0.96 (0.87,1.03)
Lesotho	3 (2,4)	0.32 (0.24,0.44)	9 (6,12)	0.80 (0.53,1.12)	2.99 (2.92,3.05)	253 (163,353)	8.01 (5.78,10.78)	253 (163,353)	21.44 (13.97,30.04)	3.28 (3.22,3.34)
Liberia	1 (1,2)	0.10 (0.07,0.14)	3 (2,4)	0.13 (0.07,0.20)	0.59 (0.52,0.64)	87 (47,138)	2.77 (1.84,3.88)	87 (47,138)	3.47 (1.92,5.42)	0.68 (0.59,0.73)
Libya	14 (10,19)	0.75 (0.52,1.02)	58 (40,79)	1.11 (0.76,1.49)	1.26 (1.19,1.32)	1702 (1141,2300)	19.28 (12.99,26.83)	1702 (1141,2300)	28.19 (18.99,38.30)	1.22 (1.16,1.28)
Lithuania	59 (51,66)	1.31 (1.14,1.46)	73 (61,85)	1.4 (1.17,1.63)	0.21 (0.04,0.40)	1917 (1605,2224)	37.29 (32.30,41.59)	1917 (1605,2224)	39.98 (33.56,46.16)	0.20 (0.01,0.42)
Luxembourg	9 (8,10)	1.65 (1.43,1.88)	13 (10,15)	1.20 (0.99,1.45)	-1.07 (-1.21,-0.96)	291 (243,346)	43.16 (37.86,48.51)	291 (243,346)	28.69 (23.95,33.92)	-1.35 (-1.50,-1.25)
Madagascar	8 (5,11)	0.16 (0.11,0.22)	9 (5,14)	0.08 (0.05,0.13)	-2.08 (-2.13,-2.04)	261 (159,420)	3.91 (2.72,5.52)	261 (159,420)	2.02 (1.28,3.22)	-2.12 (-2.16,-2.08)
Malawi	2 (2,3)	0.07 (0.05,0.09)	6 (4,9)	0.09 (0.06,0.12)	0.79 (0.75,0.83)	171 (114,245)	1.65 (1.26,2.14)	171 (114,245)	2.16 (1.46,3.06)	0.90 (0.86,0.94)
Malaysia	27 (22,33)	0.30 (0.25,0.37)	102 (84,122)	0.37 (0.30,0.44)	0.61 (0.43,0.81)	2677 (2224,3225)	7.53 (6.14,9.13)	2677 (2224,3225)	9.01 (7.46,10.87)	0.70 (0.48,0.91)
Maldives	0 (0,0)	0.42 (0.27,0.60)	1 (1,1)	0.33 (0.25,0.43)	-0.79 (-0.90,-0.64)	26 (19,34)	9.71 (6.28,14.33)	26 (19,34)	7.21 (5.41,9.32)	-1.06 (-1.19,-0.90)
Mali	5 (4,7)	0.15 (0.11,0.18)	20 (14,27)	0.24 (0.18,0.33)	1.71 (1.67,1.74)	522 (374,717)	3.52 (2.67,4.47)	522 (374,717)	5.68 (4.08,7.71)	1.58 (1.54,1.61)
Malta	6 (6,7)	1.49 (1.28,1.72)	10 (8,12)	1.15 (0.96,1.37)	-0.79 (-0.93,-0.68)	247 (208,297)	37.86 (32.87,42.87)	247 (208,297)	30.81 (26.23,36.56)	-0.62 (-0.76,-0.51)
Marshall Isla	0 (0,0)	0.27 (0.21,0.35)	0 (0,0)	0.41 (0.28,0.59)	1.43 (1.36,1.50)	5 (3,7)	7.48 (5.85,9.62)	5 (3,7)	11.45 (7.66,16.53)	1.43 (1.38,1.48)
Mauritania	2 (1,2)	0.15 (0.11,0.20)	4 (3,6)	0.19 (0.13,0.26)	0.76 (0.71,0.80)	118 (79,170)	4.10 (3.09,5.42)	118 (79,170)	5.01 (3.36,7.05)	0.66 (0.58,0.73)
Mauritius	5 (4,5)	0.66 (0.59,0.74)	14 (12,15)	0.72 (0.64,0.80)	0.19 (-0.01,0.42)	362 (319,403)	17.59 (15.77,19.5)	362 (319,403)	19.00 (16.81,21.08)	0.20 (0.01,0.41)
Mexico	352 (314,394)	0.88 (0.78,0.99)	555 (459,658)	0.44 (0.37,0.52)	-2.20 (-2.28,-2.12)	13989 (11580,1663	20.66 (18.51,23.01)	13989 (11580,16631	10.70 (8.84,12.75)	-2.11 (-2.22,-2.03)
Micronesia (F	0 (0,0)	0.55 (0.41,0.73)	1 (0,1)	0.76 (0.54,1.02)	1.04 (1.03,1.05)	19 (13,26)	15.42 (11.35,20.41)	19 (13,26)	21.44 (14.91,29.2)	1.07 (1.06,1.08)

Monaco	1 (1,2)	2.03 (1.42,2.78)	2 (1,3)	1.86 (1.25,2.72)	-0.29 (-0.30,-0.27)	38 (26,56)	52.15 (37.11,72.21)	38 (26,56)	46.50 (30.22,68.53)	-0.38 (-0.39,-0.37)
Mongolia	2 (2,3)	0.21 (0.16,0.29)	31 (22,41)	1.24 (0.88,1.66)	5.74 (5.60,5.86)	964 (687,1296)	6.09 (4.48,8.09)	964 (687,1296)	34.90 (24.90,46.48)	5.74 (5.60,5.85)
Montenegro	11 (9,13)	1.68 (1.33,2.11)	19 (15,25)	1.93 (1.49,2.46)	0.70 (0.59,0.80)	505 (394,642)	47.05 (37.52,59.06)	505 (394,642)	51.13 (39.76,64.94)	0.33 (0.20,0.44)
Morocco	17 (13,22)	0.12 (0.09,0.16)	56 (41,75)	0.16 (0.11,0.21)	0.82 (0.77,0.86)	1566 (1136,2047)	3.29 (2.51,4.19)	1566 (1136,2047)	4.19 (3.05,5.52)	0.78 (0.73,0.83)
Mozambique	3 (2,3)	0.05 (0.04,0.06)	6 (5,8)	0.06 (0.05,0.08)	0.89 (0.85,0.92)	174 (130,225)	1.10 (0.84,1.40)	174 (130,225)	1.50 (1.14,1.94)	1.04 (1.01,1.07)
Myanmar	134 (88,202)	0.59 (0.40,0.88)	218 (155,296)	0.46 (0.33,0.61)	-0.81 (-0.82,-0.79)	5670 (4017,7735)	15.06 (9.76,22.84)	5670 (4017,7735)	11.03 (7.87,14.88)	-1.00 (-1.02,-0.99)
Namibia	1 (1,1)	0.18 (0.14,0.23)	2 (2,3)	0.20 (0.15,0.25)	0.36 (0.29,0.42)	64 (45,84)	4.13 (3.19,5.23)	64 (45,84)	4.51 (3.24,5.84)	0.30 (0.24,0.36)
Nauru	0 (0,0)	0.82 (0.51,1.26)	0 (0,0)	0.88 (0.53,1.26)	0.21 (0.19,0.24)	2 (1,2)	22.78 (13.84,35.28)	2 (1,2)	25.05 (14.89,36.23)	0.31 (0.28,0.33)
Nepal	19 (10,28)	0.22 (0.11,0.32)	52 (30,78)	0.23 (0.14,0.36)	0.18 (0.15,0.21)	1225 (708,1843)	5.28 (2.75,7.70)	1225 (708,1843)	5.19 (3.00,7.84)	-0.05 (-0.08,-0.03)
Netherlands	405 (360,455)	2.03 (1.81,2.28)	499 (418,588)	1.39 (1.18,1.63)	-1.23 (-1.31,-1.16)	10645 (9155,12351)	49.35 (44.58,54.63)	10645 (9155,12351)	32.10 (27.76,36.80)	-1.41 (-1.52,-1.33)
New Zealand	49 (42,56)	1.23 (1.06,1.42)	79 (65,95)	0.93 (0.78,1.11)	-0.95 (-1.07,-0.86)	1786 (1515,2087)	30.38 (26.65,34.31)	1786 (1515,2087)	22.33 (19.12,25.96)	-1.05 (-1.18,-0.95)
Nicaragua	3 (2,3)	0.18 (0.14,0.22)	12 (9,16)	0.25 (0.19,0.33)	1.20 (1.00,1.36)	328 (253,436)	4.61 (3.67,5.68)	328 (253,436)	6.35 (4.88,8.46)	1.14 (0.92,1.35)
Niger	1 (1,2)	0.05 (0.03,0.06)	4 (2,6)	0.05 (0.03,0.08)	0.54 (0.51,0.57)	106 (61,161)	1.11 (0.74,1.51)	106 (61,161)	1.23 (0.73,1.84)	0.34 (0.29,0.38)
Nigeria	9 (6,12)	0.02 (0.02,0.03)	17 (12,22)	0.02 (0.01,0.03)	-0.42 (-0.45,-0.39)	476 (337,642)	0.51 (0.36,0.66)	476 (337,642)	0.47 (0.34,0.62)	-0.26 (-0.29,-0.24)
Niue	0 (0,0)	0.38 (0.27,0.51)	0 (0,0)	0.58 (0.44,0.79)	1.40 (1.39,1.42)	0 (0,0)	10.45 (7.36,14.35)	0 (0,0)	15.75 (11.44,21.39)	1.32 (1.31,1.33)
North Maced	27 (22,34)	1.40 (1.15,1.76)	55 (42,72)	1.65 (1.25,2.13)	0.56 (0.41,0.67)	1451 (1081,1924)	37.82 (30.64,46.92)	1451 (1081,1924)	42.31 (31.66,55.68)	0.36 (0.19,0.49)
Northern Mar	0 (0,0)	0.60 (0.47,0.77)	0 (0,1)	0.88 (0.72,1.07)	1.28 (1.17,1.37)	14 (12,18)	16.06 (12.43,21)	14 (12,18)	23.4 (19.18,28.48)	1.23 (1.15,1.31)
Norway	114 (100,130)	1.70 (1.51,1.92)	91 (76,105)	0.93 (0.78,1.06)	-1.87 (-2.03,-1.73)	2047 (1763,2308)	42.07 (38.17,46.48)	2047 (1763,2308)	22.57 (19.75,25.37)	-1.94 (-2.07,-1.82)
Oman	1 (1,1)	0.13 (0.09,0.19)	3 (2,4)	0.15 (0.11,0.21)	0.57 (0.50,0.64)	94 (64,132)	3.41 (2.33,4.92)	94 (64,132)	3.81 (2.65,5.24)	0.37 (0.30,0.45)
Pakistan	99 (78,121)	0.18 (0.14,0.22)	217 (153,314)	0.19 (0.13,0.27)	0.10 (0.06,0.13)	5936 (4128,8610)	4.41 (3.49,5.4)	5936 (4128,8610)	4.56 (3.20,6.62)	0.11 (0.07,0.14)
Palau	0 (0,0)	0.53 (0.39,0.71)	0 (0,0)	0.53 (0.4,0.72)	-0.01 (-0.04,0.02)	3 (2,4)	13.50 (9.93,18.07)	3 (2,4)	12.99 (9.59,17.52)	-0.14 (-0.16,-0.11)
Palestine	6 (4,8)	0.74 (0.53,1.01)	20 (16,25)	0.82 (0.65,1.00)	0.25 (0.17,0.35)	553 (442,687)	17.58 (12.15,24.04)	553 (442,687)	19.78 (15.8,24.4)	0.34 (0.26,0.45)
Panama	3 (3,4)	0.23 (0.20,0.27)	12 (9,15)	0.26 (0.20,0.34)	0.44 (0.10,0.75)	273 (203,349)	5.64 (4.87,6.38)	273 (203,349)	6.20 (4.60,7.93)	0.10 (-0.44,0.52)
Papua New G	4 (2,6)	0.19 (0.12,0.29)	11 (7,17)	0.21 (0.14,0.31)	0.30 (0.26,0.34)	357 (231,555)	5.25 (3.39,8.04)	357 (231,555)	5.76 (3.80,8.82)	0.32 (0.27,0.36)
Paraguay	11 (9,13)	0.52 (0.42,0.63)	49 (36,66)	0.88 (0.63,1.17)	1.85 (1.75,1.95)	1174 (862,1588)	11.93 (9.73,14.38)	1174 (862,1588)	19.91 (14.6,26.84)	1.79 (1.71,1.87)
Peru	36 (28,48)	0.31 (0.24,0.41)	113 (78,160)	0.34 (0.24,0.48)	0.42 (0.22,0.58)	2765 (1913,3915)	7.63 (5.83,10.09)	2765 (1913,3915)	8.19 (5.65,11.54)	0.35 (0.13,0.52)
Philippines	147 (121,178)	0.51 (0.42,0.62)	446 (359,551)	0.54 (0.44,0.66)	0.26 (0.21,0.31)	12787 (10164,1592	13.22 (10.87,15.99)	12787 (10164,15921	14.11 (11.34,17.47)	0.21 (0.17,0.27)
Poland	793 (723,869)	1.80 (1.64,1.97)	980 (848,1124)	1.40 (1.21,1.60)	-0.89 (-0.95,-0.84)	24868 (21627,2821	51.73 (47.76,56.37)	24868 (21627,28215	37.56 (32.74,42.51)	-1.09 (-1.15,-1.04)
Portugal	123 (108,138)	0.88 (0.77,0.99)	161 (137,187)	0.76 (0.65,0.87)	-0.52 (-0.72,-0.40)	4136 (3589,4729)	23.84 (21.08,26.44)	4136 (3589,4729)	21.39 (18.51,24.30)	-0.37 (-0.60,-0.20)
Puerto Rico	17 (14,20)	0.45 (0.38,0.54)	41 (31,53)	0.60 (0.47,0.77)	0.97 (0.78,1.15)	940 (732,1207)	10.88 (9.12,12.81)	940 (732,1207)	15.69 (12.28,19.99)	1.21 (1.01,1.41)
Qatar	1 (1,1)	0.67 (0.49,0.87)	5 (3,8)	0.53 (0.34,0.80)	-0.94 (-1.24,-0.67)	177 (118,262)	16.22 (11.92,21.26)	177 (118,262)	12.56 (8.17,19.48)	-0.85 (-1.06,-0.66)
Republic of K	496 (410,599)	1.69 (1.39,2.04)	1043 (802,1329)	1.10 (0.85,1.40)	-1.42 (-1.47,-1.37)	22546 (17517,2818	43.05 (35.52,51.82)	22546 (17517,28181	24.08 (18.74,30.09)	-1.90 (-1.94,-1.86)
Republic of M	49 (42,57)	1.07 (0.9,1.24)	73 (61,85)	1.23 (1.04,1.42)	0.12 (-0.08,0.32)	2117 (1791,2434)	31.80 (27.40,36.71)	2117 (1791,2434)	36.59 (31.13,41.92)	0.39 (0.12,0.68)
Romania	321 (281,363)	1.12 (0.97,1.26)	504 (413,590)	1.47 (1.21,1.73)	0.90 (0.80,1.06)	13760 (11336,1614	33.25 (29.21,37.5)	13760 (11336,16144	42.96 (35.39,50.41)	0.96 (0.85,1.12)
Russian Fede	2371 (2183,2567)	1.28 (1.18,1.39)	3111 (2685,3558)	1.31 (1.13,1.50)	0.28 (0.15,0.41)	87999 (75917,1004	39.93 (36.82,43.13)	87999 (75917,10042	38.4 (33.18,43.67)	0.09 (-0.05,0.23)
Rwanda	12 (9,16)	0.46 (0.35,0.58)	24 (16,36)	0.42 (0.29,0.63)	-0.21 (-0.24,-0.18)	607 (405,920)	10.96 (8.42,14.13)	607 (405,920)	9.47 (6.44,14.21)	-0.45 (-0.49,-0.41)
Saint Kitts an	0 (0,0)	0.41 (0.31,0.52)	0 (0,0)	0.43 (0.32,0.56)	0.09 (-0.04,0.24)	8 (6,10)	10.48 (8.01,12.95)	8 (6,10)	10.28 (7.71,13.45)	0.05 (-0.12,0.21)
Saint Lucia	0 (0,1)	0.52 (0.42,0.63)	1 (1,2)	0.50 (0.39,0.64)	-0.07 (-0.18,0.03)	32 (25,41)	13.50 (11.36,16.02)	32 (25,41)	13.07 (10.08,16.55)	-0.06 (-0.17,0.04)
Saint Vincent	0 (0,0)	0.42 (0.35,0.51)	1 (1,1)	0.51 (0.41,0.62)	0.56 (0.46,0.65)	20 (16,24)	11.22 (9.31,13.26)	20 (16,24)	13.47 (10.96,16.42)	0.58 (0.48,0.67)
Samoa	0 (0,1)	0.54 (0.43,0.66)	1 (1,1)	0.51 (0.39,0.67)	-0.20 (-0.22,-0.19)	21 (16,28)	14.28 (11.14,17.61)	21 (16,28)	13.62 (10.39,17.87)	-0.16 (-0.18,-0.14)
San Marino	1 (0,1)	1.51 (1.19,1.91)	1 (0,1)	0.73 (0.46,1.07)	-2.61 (-3.04,-2.35)	12 (7,17)	36.83 (29.38,45.85)	12 (7,17)	18.04 (10.81,26.86)	-2.49 (-2.84,-2.26)
Sao Tome and	0 (0,0)	0.03 (0.02,0.03)	0 (0,0)	0.04 (0.03,0.06)	1.86 (1.81,1.90)	1 (1,2)	0.67 (0.50,0.85)	1 (1,2)	1.17 (0.84,1.62)	1.83 (1.78,1.87)
Saudi Arabia	7 (5,9)	0.12 (0.08,0.16)	58 (43,76)	0.27 (0.20,0.35)	2.62 (2.58,2.65)	1940 (1406,2540)	3.21 (2.25,4.25)	1940 (1406,2540)	7.15 (5.28,9.32)	2.61 (2.58,2.64)
Senegal	4 (3,5)	0.12 (0.09,0.16)	11 (7,15)	0.13 (0.09,0.18)	0.10 (-0.08,0.23)	321 (218,458)	3.50 (2.59,4.56)	321 (218,458)	3.65 (2.50,5.17)	-0.05 (-0.18,0.06)
Serbia	157 (124,206)	1.36 (1.08,1.79)	245 (185,313)	1.53 (1.15,1.96)	0.42 (0.32,0.53)	6265 (4727,8089)	37.26 (29.55,48.84)	6265 (4727,8089)	41.55 (31.14,53.84)	0.36 (0.24,0.48)
Seychelles	1 (0,1)	0.94 (0.77,1.13)	1 (1,1)	0.88 (0.71,1.06)	-0.30 (-0.46,-0.14)	28 (23,34)	25.15 (20.43,30.28)	28 (23,34)	22.46 (18.26,26.7)	-0.38 (-0.47,-0.30)
Sierra Leone	3 (2,3)	0.13 (0.10,0.17)	5 (4,8)	0.14 (0.10,0.19)	0.34 (0.31,0.37)	163 (112,228)	3.40 (2.52,4.42)	163 (112,228)	3.87 (2.71,5.34)	0.42 (0.38,0.46)
Singapore	14 (12,16)	0.62 (0.53,0.71)	34 (29,40)	0.39 (0.33,0.46)	-1.46 (-1.84,-1.20)	828 (706,970)	16.02 (14.06,18.54)	828 (706,970)	9.28 (7.94,10.85)	-1.64 (-2.38,-0.79)
Slovakia	94 (75,117)	1.57 (1.26,1.96)	131 (103,169)	1.38 (1.08,1.79)	-0.41 (-0.51,-0.32)	3449 (2643,4514)	45.69 (36.35,57.69)	3449 (2643,4514)	37.92 (29.06,49.66)	-0.61 (-0.73,-0.51)
Slovenia	28 (24,32)	1.13 (0.96,1.31)	44 (35,55)	1.05 (0.83,1.30)	-0.34 (-0.57,-0.15)	1086 (859,1331)	32.04 (27.39,36.76)	1086 (859,1331)	27.75 (22.08,34.01)	-0.69 (-0.96,-0.44)
Solomon Isla	1 (0,1)	0.36 (0.22,0.51)	2 (1,3)	0.50 (0.36,0.69)	1.03 (0.97,1.09)	57 (40,80)	9.87 (5.82,14.08)	57 (40,80)	14.00 (9.89,19.58)	1.14 (1.07,1.20)
Somalia	4 (2,6)	0.15 (0.09,0.25)	6 (3,10)	0.10 (0.05,0.16)	-1.38 (-1.40,-1.37)	187 (96,307)	4.02 (2.31,6.56)	187 (96,307)	2.63 (1.38,4.27)	-1.36 (-1.38,-1.33)
South Africa	145 (118,196)	0.70 (0.56,0.95)	297 (248,348)	0.62 (0.52,0.73)	-0.47 (-0.60,-0.31)	8719 (7307,10178)	19.19 (15.69,25.58)	8719 (7307,10178)	17.17 (14.44,20.08)	-0.47 (-0.62,-0.25)
South Sudan	6 (4,9)	0.24 (0.15,0.36)	7 (4,11)	0.19 (0.11,0.28)	-0.73 (-0.76,-0.70)	204 (118,312)	5.98 (3.62,9.29)	204 (118,312)	4.73 (2.77,7.22)	-0.76 (-0.80,-0.72)
Spain	671 (596,748)	1.25 (1.11,1.39)	908 (782,1060)	1.01 (0.88,1.16)	-0.70 (-0.82,-0.62)	22190 (19361,2542	33.19 (29.79,36.58)	22190 (19361,25422	26.89 (23.59,30.72)	-0.71 (-0.85,-0.62)

Sri Lanka	22 (18,26)	0.21 (0.17,0.26)	39 (24,58)	0.14 (0.09,0.21)	-1.25 (-1.54,-1.06)	1009 (599,1502)	5.23 (4.26,6.33)	1009 (599,1502)	3.60 (2.15,5.36)	-1.13 (-1.46,-0.88)
Sudan	13 (8,20)	0.14 (0.09,0.22)	44 (27,69)	0.23 (0.14,0.36)	1.62 (1.61,1.64)	1248 (760,1976)	3.73 (2.38,5.77)	1248 (760,1976)	5.80 (3.53,9.12)	1.45 (1.44,1.46)
Suriname	2 (1,2)	0.72 (0.57,0.87)	5 (3,6)	0.73 (0.51,0.98)	0.13 (-0.06,0.29)	137 (97,186)	19.68 (15.65,23.62)	137 (97,186)	20.33 (14.41,27.38)	0.19 (0.03,0.32)
Sweden	254 (217,291)	1.68 (1.45,1.92)	283 (226,349)	1.28 (1.03,1.55)	-0.90 (-1.09,-0.78)	5624 (4586,6781)	40.39 (35.39,45.57)	5624 (4586,6781)	28.35 (23.34,33.75)	-1.15 (-1.35,-1.02)
Switzerland	111 (95,131)	1.08 (0.94,1.26)	223 (188,262)	1.21 (1.03,1.41)	0.27 (0.07,0.44)	4570 (3931,5312)	27.00 (23.58,30.96)	4570 (3931,5312)	27.24 (23.48,31.44)	-0.10 (-0.31,0.08)
Syrian Arab F	21 (15,28)	0.41 (0.29,0.54)	71 (47,99)	0.54 (0.36,0.73)	0.79 (0.72,0.88)	1968 (1320,2762)	10.78 (7.74,13.94)	1968 (1320,2762)	13.31 (8.94,18.65)	0.65 (0.57,0.74)
Taiwan (Prov	130 (116,142)	0.80 (0.72,0.88)	417 (355,481)	0.99 (0.84,1.14)	0.85 (0.64,1.03)	10541 (8959,12148)	21.60 (19.47,23.69)	10541 (8959,12148)	25.76 (21.94,29.39)	0.72 (0.58,0.89)
Tajikistan	6 (4,9)	0.21 (0.14,0.32)	7 (4,10)	0.12 (0.07,0.17)	-1.93 (-2.03,-1.84)	209 (131,310)	5.74 (3.82,8.42)	209 (131,310)	3.07 (1.93,4.53)	-2.08 (-2.20,-1.96)
Thailand	260 (206,325)	0.75 (0.60,0.92)	763 (564,1044)	0.69 (0.52,0.94)	-0.22 (-0.33,-0.12)	19946 (14617,2752(	18.99 (14.94,23.76)	19946 (14617,27526	18.30 (13.40,25.14)	-0.09 (-0.20,0.02)
Timor-Leste	1 (0,1)	0.25 (0.15,0.35)	3 (2,3)	0.30 (0.22,0.40)	0.64 (0.59,0.68)	70 (50,94)	6.38 (3.82,9.22)	70 (50,94)	7.82 (5.61,10.53)	0.66 (0.61,0.70)
Togo	2 (1,2)	0.15 (0.11,0.20)	7 (5,10)	0.18 (0.12,0.24)	0.63 (0.59,0.66)	219 (149,303)	3.87 (2.79,5.12)	219 (149,303)	4.88 (3.34,6.63)	0.77 (0.73,0.82)
Tokelau	0 (0,0)	0.35 (0.23,0.51)	0 (0,0)	0.46 (0.30,0.66)	0.89 (0.87,0.91)	0 (0,0)	9.32 (6.04,13.51)	0 (0,0)	12.55 (8.08,17.91)	0.97 (0.95,0.99)
Tonga	1 (0,1)	0.97 (0.73,1.32)	1 (1,1)	1.12 (0.83,1.48)	0.47 (0.39,0.55)	23 (16,30)	23.54 (17.97,31.81)	23 (16,30)	27.7 (20.09,36.93)	0.51 (0.45,0.59)
Trinidad and	5 (4,6)	0.62 (0.53,0.72)	11 (8,14)	0.56 (0.40,0.73)	-0.32 (-0.54,-0.15)	294 (209,391)	16.55 (14.38,19.16)	294 (209,391)	15 (10.66,19.97)	-0.18 (-0.39,-0.00)
Tunisia	19 (15,24)	0.40 (0.31,0.50)	66 (46,93)	0.50 (0.36,0.70)	0.70 (0.63,0.75)	1650 (1140,2313)	9.38 (7.24,11.87)	1650 (1140,2313)	11.95 (8.30,16.70)	0.77 (0.72,0.82)
Turkey	470 (351,616)	1.33 (1.00,1.73)	1177 (884,1483)	1.23 (0.92,1.55)	-0.25 (-0.30,-0.20)	31667 (23910,4016	36.13 (26.96,47.66)	31667 (23910,40168	32.17 (24.39,40.88)	-0.39 (-0.44,-0.33)
Turkmenistan	0 (0,1)	0.02 (0.02,0.03)	11 (8,16)	0.27 (0.19,0.36)	8.32 (7.63,8.93)	360 (255,493)	0.70 (0.60,0.82)	360 (255,493)	7.73 (5.53,10.58)	8.03 (7.38,8.55)
Tuvalu	0 (0,0)	0.36 (0.28,0.44)	0 (0,0)	0.53 (0.40,0.70)	1.32 (1.31,1.34)	2 (1,2)	9.84 (7.67,12.37)	2 (1,2)	14.76 (10.99,19.62)	1.32 (1.31,1.34)
Uganda	12 (9,16)	0.20 (0.15,0.26)	34 (24,49)	0.24 (0.17,0.34)	0.68 (0.64,0.72)	966 (655,1422)	4.88 (3.65,6.61)	966 (655,1422)	6.06 (4.17,8.65)	0.66 (0.62,0.71)
Ukraine	640 (499,863)	0.89 (0.69,1.20)	728 (497,1011)	0.99 (0.68,1.39)	0.43 (0.20,0.78)	21749 (14848,3053	27.56 (21.29,38.3)	21749 (14848,30535	31.19 (21.21,43.98)	0.42 (0.16,0.81)
United Arab I	4 (2,7)	0.83 (0.52,1.38)	31 (23,40)	0.85 (0.61,1.14)	0.15 (-0.30,0.54)	1071 (785,1390)	21.70 (13.18,36.15)	1071 (785,1390)	18.52 (13.66,24.52)	-0.55 (-0.96,-0.23)
United Kingd	1733 (1537,1945)	1.90 (1.69,2.12)	1640 (1381,1975)	1.23 (1.05,1.46)	-1.41 (-1.49,-1.35)	33655 (28901,3945	45.23 (40.79,49.52)	33655 (28901,39458	27.89 (24.3,32.32)	-1.57 (-1.67,-1.50)
United Repub	32 (23,44)	0.31 (0.22,0.41)	60 (40,95)	0.24 (0.16,0.38)	-0.74 (-0.76,-0.73)	1681 (1074,2700)	7.68 (5.56,10.71)	1681 (1074,2700)	6.07 (3.97,9.57)	-0.76 (-0.77,-0.74)
United States	5096 (4526,5757)	1.61 (1.44,1.81)	7903 (6669,9412)	1.34 (1.14,1.58)	-0.62 (-0.66,-0.59)	180575 (155796,20	41.06 (37.03,45.46)	180575 (155796,209	32.26 (27.99,37.16)	-0.80 (-0.84,-0.77)
United States	0 (0,0)	0.35 (0.27,0.46)	1 (0,1)	0.36 (0.25,0.51)	0.04 (-0.10,0.14)	16 (11,22)	9.21 (7.09,11.95)	16 (11,22)	9.48 (6.59,13.28)	0.04 (-0.09,0.14)
Uruguay	62 (53,71)	1.59 (1.37,1.82)	94 (78,110)	1.80 (1.51,2.09)	0.42 (0.31,0.53)	2339 (1980,2689)	42.96 (37.56,48.6)	2339 (1980,2689)	48.02 (40.96,54.96)	0.38 (0.21,0.53)
Uzbekistan	13 (9,16)	0.11 (0.08,0.14)	63 (49,80)	0.22 (0.17,0.28)	2.46 (2.32,2.59)	1972 (1512,2533)	3.18 (2.31,4.09)	1972 (1512,2533)	6.38 (4.92,8.17)	2.29 (2.15,2.44)
Vanuatu	0 (0,0)	0.28 (0.21,0.36)	1 (0,1)	0.30 (0.23,0.38)	0.22 (0.19,0.26)	16 (12,20)	7.36 (5.57,9.67)	16 (12,20)	7.95 (6.08,10.11)	0.23 (0.19,0.27)
Venezuela (B	18 (16,20)	0.19 (0.16,0.21)	136 (95,184)	0.45 (0.31,0.60)	2.59 (2.16,3.03)	3565 (2455,4854)	4.92 (4.30,5.55)	3565 (2455,4854)	11.32 (7.81,15.38)	2.45 (2.00,2.90)
Viet Nam	116 (87,152)	0.29 (0.22,0.38)	384 (288,488)	0.38 (0.28,0.47)	0.87 (0.85,0.89)	10799 (7946,14122)	7.36 (5.48,9.67)	10799 (7946,14122)	9.90 (7.36,12.78)	0.99 (0.97,1.01)
Yemen	10 (6,15)	0.19 (0.11,0.30)	39 (25,53)	0.28 (0.18,0.38)	1.16 (1.12,1.20)	1123 (719,1589)	5.35 (3.15,8.42)	1123 (719,1589)	7.11 (4.56,9.83)	0.93 (0.88,0.97)
Zambia	8 (6,10)	0.29 (0.23,0.36)	18 (12,25)	0.28 (0.19,0.38)	-0.09 (-0.12,-0.06)	490 (337,680)	6.92 (5.33,8.73)	490 (337,680)	6.69 (4.63,9.28)	-0.10 (-0.13,-0.08)
Zimbabwe	26 (20,33)	0.69 (0.54,0.87)	56 (42,72)	0.83 (0.64,1.06)	0.73 (0.64,0.85)	1628 (1233,2137)	16.45 (12.73,20.59)	1628 (1233,2137)	21.23 (16.12,27.59)	0.90 (0.82,1.01)
Afghanistan	11 (4,21)	0.16 (0.06,0.30)	20 (9,35)	0.19 (0.09,0.33)	0.54 (0.52,0.56)	316 (101,593)	4.26 (1.37,7.92)	663 (291,1174)	5.17 (2.37,8.99)	0.63 (0.62,0.65)
Albania	8 (5,12)	0.43 (0.26,0.64)	20 (12,30)	0.46 (0.27,0.69)	0.13 (-0.06,0.27)	202 (123,296)	9.69 (5.94,14.21)	442 (262,670)	10.17 (6.03,15.49)	0.07 (-0.11,0.23)
Algeria	24 (15,35)	0.24 (0.14,0.36)	60 (34,91)	0.19 (0.11,0.29)	-0.70 (-0.73,-0.67)	628 (384,924)	5.20 (3.16,7.66)	1504 (849,2296)	4.18 (2.36,6.38)	-0.70 (-0.72,-0.67)
American Sar	0 (0,0)	0.71 (0.42,1.04)	0 (0,0)	0.65 (0.39,0.97)	-0.15 (-0.24,-0.04)	5 (3,7)	19.18 (11.48,27.83)	9 (6,14)	17.81 (10.96,26.62)	-0.18 (-0.26,-0.08)
Andorra	1 (0,1)	1.55 (0.84,2.41)	1 (1,2)	0.80 (0.41,1.24)	-2.27 (-2.48,-2.12)	23 (13,37)	39.26 (21.72,62.29)	31 (16,47)	20.18 (10.66,31.26)	-2.27 (-2.48,-2.11)
Angola	9 (5,15)	0.24 (0.14,0.39)	27 (15,42)	0.22 (0.13,0.35)	-0.26 (-0.31,-0.20)	288 (160,470)	6.48 (3.69,10.52)	821 (470,1317)	5.91 (3.41,9.37)	-0.28 (-0.33,-0.21)
Antigua and I	0 (0,0)	0.31 (0.18,0.44)	0 (0,1)	0.36 (0.21,0.52)	0.49 (0.29,0.72)	4 (2,6)	7.71 (4.55,11.05)	10 (6,14)	8.55 (5.04,12.35)	0.38 (0.18,0.59)
Argentina	364 (222,509)	1.11 (0.68,1.56)	498 (302,714)	0.90 (0.55,1.29)	-0.63 (-0.70,-0.56)	9972 (6134,13647)	30.37 (18.69,41.52)	13207 (8141,18933)	24.59 (15.19,35.28)	-0.65 (-0.73,-0.58)
Armenia	20 (13,27)	0.70 (0.45,0.94)	30 (19,42)	0.68 (0.42,0.94)	-0.12 (-0.35,0.13)	606 (389,823)	20.23 (13.06,27.45)	757 (472,1038)	17.49 (10.92,23.98)	-0.60 (-0.78,-0.39)
Australia	217 (135,304)	1.12 (0.70,1.55)	176 (106,266)	0.41 (0.25,0.61)	-3.21 (-3.30,-3.13)	5872 (3685,8133)	30.82 (19.34,42.73)	4513 (2775,6702)	11.6 (7.13,17.09)	-3.13 (-3.21,-3.03)
Austria	123 (76,168)	1.07 (0.66,1.47)	90 (51,129)	0.52 (0.30,0.73)	-2.33 (-2.38,-2.27)	3119 (1941,4233)	29.10 (18.15,39.60)	2212 (1278,3126)	14 (8.15,19.84)	-2.33 (-2.40,-2.26)
Azerbaijan	20 (12,29)	0.38 (0.23,0.55)	36 (20,55)	0.34 (0.19,0.52)	-0.38 (-0.45,-0.32)	656 (400,929)	11.74 (7.15,16.74)	1090 (614,1672)	9.37 (5.27,14.29)	-0.76 (-0.85,-0.69)
Bahamas	1 (0,1)	0.43 (0.25,0.61)	2 (1,3)	0.48 (0.27,0.72)	0.50 (0.36,0.60)	20 (12,28)	11.84 (7.19,16.95)	54 (30,81)	12.4 (6.94,18.52)	0.20 (0.06,0.43)
Bahrain	1 (0,1)	0.51 (0.31,0.73)	3 (2,5)	0.39 (0.23,0.61)	-0.85 (-0.93,-0.76)	23 (14,33)	11.89 (7.24,16.90)	91 (53,146)	8.79 (5.24,13.88)	-1.02 (-1.06,-0.97)
Bangladesh	144 (79,222)	0.32 (0.18,0.49)	294 (172,457)	0.22 (0.13,0.34)	-1.03 (-1.13,-0.94)	3912 (2145,6080)	8.09 (4.40,12.50)	7396 (4338,11701)	5.24 (3.07,8.20)	-1.25 (-1.33,-1.18)
Barbados	1 (1,2)	0.39 (0.22,0.55)	2 (1,3)	0.41 (0.23,0.64)	0.21 (0.06,0.35)	27 (15,37)	9.75 (5.70,13.69)	48 (27,75)	9.62 (5.36,14.77)	0.15 (0.03,0.29)
Belarus	107 (66,151)	0.82 (0.51,1.15)	149 (87,220)	0.92 (0.54,1.36)	0.14 (-0.04,0.37)	3060 (1885,4280)	23.56 (14.50,32.81)	3934 (2286,5820)	24.86 (14.46,36.74)	-0.08 (-0.28,0.16)
Belgium	208 (128,289)	1.35 (0.83,1.86)	132 (76,189)	0.57 (0.34,0.82)	-2.79 (-2.91,-2.65)	4881 (3037,6697)	33.40 (20.80,45.55)	3040 (1789,4368)	14.85 (8.86,21.37)	-2.74 (-2.89,-2.63)
Belize	0 (0,0)	0.22 (0.13,0.30)	1 (1,1)	0.29 (0.17,0.44)	0.98 (0.79,1.13)	5 (3,7)	5.42 (3.29,7.63)	23 (14,35)	7.36 (4.42,10.95)	0.94 (0.75,1.10)
Benin	2 (1,3)	0.09 (0.05,0.13)	3 (2,4)	0.06 (0.03,0.09)	-1.30 (-1.33,-1.26)	44 (26,67)	2.19 (1.27,3.29)	77 (42,117)	1.43 (0.79,2.22)	-1.35 (-1.38,-1.32)
Bermuda	1 (0,1)	0.96 (0.57,1.38)	1 (0,1)	0.62 (0.36,0.96)	-1.48 (-1.57,-1.39)	15 (9,21)	23.48 (14.2,33.43)	20 (11,31)	15.25 (8.93,23.78)	-1.40 (-1.53,-1.27)
Bhutan	0 (0,0)	0.11 (0.05,0.19)	1 (0,1)	0.11 (0.06,0.17)	-0.22 (-0.27,-0.18)	8 (4,13)	2.94 (1.29,4.91)	16 (8,25)	2.51 (1.34,3.98)	-0.48 (-0.51,-0.45)

Bolivia (Pluri	11 (6,17)	0.36 (0.19,0.55)	31 (16,52)	0.35 (0.18,0.58)	-0.11 (-0.14,-0.09)	300 (162,459)	8.99 (4.83,13.71)	777 (415,1304)	8.24 (4.36,13.82)	-0.27 (-0.30,-0.25)
Bosnia and H	29 (19,41)	0.71 (0.45,1.00)	70 (42,104)	1.11 (0.66,1.65)	1.49 (1.38,1.59)	852 (548,1192)	19.04 (12.25,26.58)	1701 (1010,2547)	28.21 (16.58,42.28)	1.31 (1.19,1.42)
Botswana	2 (1,3)	0.43 (0.24,0.65)	6 (3,8)	0.40 (0.23,0.60)	-0.22 (-0.27,-0.18)	63 (35,98)	10.57 (6.04,16.23)	152 (85,239)	9.60 (5.39,14.75)	-0.29 (-0.33,-0.24)
Brazil	539 (338,740)	0.61 (0.38,0.85)	1207 (733,1725)	0.48 (0.29,0.68)	-0.75 (-0.80,-0.70)	15330 (9643,20967)	15.90 (9.99,21.78)	32255 (19917,45804	12.50 (7.71,17.77)	-0.75 (-0.79,-0.70)
Brunei Darus	2 (1,2)	1.49 (0.91,2.20)	2 (1,4)	0.69 (0.41,1.02)	-2.49 (-2.60,-2.40)	44 (27,65)	38.25 (23.49,56.55)	75 (44,110)	18.13 (10.74,26.74)	-2.38 (-2.44,-2.32)
Bulgaria	148 (93,208)	1.18 (0.74,1.65)	166 (102,250)	1.25 (0.77,1.85)	0.27 (0.16,0.39)	4441 (2800,6187)	36.12 (22.8,49.95)	4538 (2801,6718)	37.03 (22.94,54.98)	0.18 (0.06,0.30)
Burkina Faso	3 (2,4)	0.07 (0.04,0.10)	6 (3,10)	0.07 (0.04,0.11)	0.22 (0.19,0.24)	81 (45,123)	1.74 (0.97,2.64)	181 (95,273)	1.81 (0.96,2.70)	0.12 (0.10,0.14)
Burundi	6 (4,9)	0.27 (0.16,0.41)	8 (4,13)	0.17 (0.09,0.26)	-1.54 (-1.58,-1.51)	173 (99,263)	7.11 (4.03,10.69)	227 (124,366)	4.22 (2.32,6.78)	-1.66 (-1.69,-1.62)
Cabo Verde	0 (0,0)	0.06 (0.03,0.09)	0 (0,1)	0.11 (0.06,0.17)	2.25 (1.99,2.54)	3 (2,5)	1.51 (0.90,2.26)	13 (7,19)	2.78 (1.48,4.24)	2.07 (1.84,2.31)
Cambodia	34 (19,52)	0.79 (0.44,1.20)	101 (59,151)	0.85 (0.51,1.28)	0.26 (0.25,0.28)	975 (532,1476)	20.14 (11.01,30.53)	2753 (1551,4127)	20.79 (12.03,31.09)	0.12 (0.11,0.14)
Cameroon	5 (3,8)	0.12 (0.07,0.17)	13 (6,20)	0.10 (0.05,0.16)	-0.34 (-0.36,-0.32)	152 (90,226)	3.06 (1.80,4.57)	382 (183,604)	2.69 (1.28,4.23)	-0.43 (-0.45,-0.41)
Canada	383 (239,534)	1.18 (0.74,1.65)	400 (246,580)	0.56 (0.35,0.81)	-2.36 (-2.47,-2.26)	9785 (6193,13510)	30.74 (19.42,42.63)	9500 (5837,13644)	14.72 (9.07,21.22)	-2.33 (-2.39,-2.28)
Central Afric	3 (1,4)	0.22 (0.11,0.36)	4 (2,6)	0.16 (0.08,0.27)	-1.01 (-1.04,-0.97)	79 (39,131)	5.99 (3.00,9.87)	122 (59,212)	4.46 (2.22,7.59)	-0.93 (-0.98,-0.89)
Chad	2 (1,4)	0.09 (0.05,0.14)	6 (3,9)	0.11 (0.06,0.17)	0.60 (0.57,0.64)	62 (36,94)	2.16 (1.25,3.27)	156 (83,251)	2.59 (1.37,4.12)	0.61 (0.58,0.64)
Chile	53 (32,74)	0.52 (0.31,0.73)	121 (71,180)	0.47 (0.28,0.70)	-0.27 (-0.37,-0.14)	1559 (956,2176)	14.69 (8.96,20.55)	3377 (2058,4909)	13.58 (8.28,19.66)	-0.23 (-0.44,-0.11)
China	7775 (4961,10887	0.95 (0.60,1.33)	17277 (10520,256	0.82 (0.50,1.21)	-0.47 (-0.51,-0.43)	232204 (149570,32	25.32 (16.29,35.44)	459250 (276317,684	21.44 (12.95,31.98)	-0.54 (-0.58,-0.50)
Colombia	54 (34,76)	0.31 (0.19,0.43)	131 (77,198)	0.24 (0.14,0.36)	-0.86 (-0.98,-0.76)	1543 (969,2183)	8.09 (5.11,11.45)	3491 (2089,5236)	6.31 (3.77,9.47)	-0.83 (-0.95,-0.72)
Comoros	1 (0,1)	0.31 (0.18,0.51)	1 (1,2)	0.30 (0.17,0.47)	-0.21 (-0.27,-0.14)	16 (8,26)	7.54 (4.24,12.35)	33 (18,52)	6.58 (3.56,10.39)	-0.46 (-0.53,-0.36)
Congo	2 (1,4)	0.23 (0.12,0.36)	6 (4,9)	0.24 (0.14,0.35)	0.18 (0.15,0.22)	69 (37,109)	5.91 (3.21,9.36)	180 (105,272)	5.91 (3.52,9.02)	0.02 (-0.01,0.07)
Cook Islands	0 (0,0)	0.32 (0.18,0.46)	0 (0,0)	0.20 (0.12,0.29)	-1.44 (-1.48,-1.39)	1 (1,2)	8.42 (4.85,12.29)	1 (1,2)	5.37 (3.24,7.92)	-1.42 (-1.46,-1.37)
Costa Rica	7 (4,10)	0.39 (0.24,0.56)	28 (16,41)	0.50 (0.29,0.74)	1.06 (0.79,1.34)	171 (103,244)	9.54 (5.82,13.64)	709 (425,1029)	12.84 (7.66,18.64)	1.15 (0.87,1.44)
Côte d'Ivoire	87 (56,121)	1.46 (0.94,2.03)	126 (76,182)	1.39 (0.85,2.00)	-0.54 (-0.58,-0.52)	2253 (1473,3153)	36.18 (23.61,50.37)	2813 (1741,3996)	34.16 (21.12,48.26)	-0.55 (-0.58,-0.52)
Croatia	82 (51,112)	0.81 (0.50,1.10)	139 (84,199)	0.70 (0.42,1.01)	-0.23 (-0.39,-0.09)	2159 (1359,2964)	21.19 (13.34,29.04)	3501 (2141,4939)	18.28 (11.21,25.82)	-0.20 (-0.36,-0.06)
Cuba	7 (4,9)	0.91 (0.56,1.32)	12 (7,17)	0.56 (0.34,0.83)	-0.39 (-0.51,-0.28)	165 (102,238)	20.85 (13.00,29.86)	279 (172,408)	13.74 (8.41,20.02)	-0.41 (-0.53,-0.31)
Cyprus	292 (175,411)	2.09 (1.26,2.95)	219 (129,327)	1.03 (0.61,1.53)	-1.62 (-1.70,-1.55)	7569 (4572,10619)	55.75 (33.90,77.89)	5250 (3125,7826)	26.73 (15.92,39.92)	-1.36 (-1.45,-1.31)
Czechia	3 (2,4)	0.07 (0.04,0.11)	7 (4,11)	0.06 (0.03,0.10)	-2.32 (-2.36,-2.28)	89 (52,136)	1.92 (1.09,2.88)	213 (112,348)	1.63 (0.87,2.58)	-2.42 (-2.47,-2.37)
Democratic P	89 (51,135)	0.52 (0.30,0.77)	158 (85,260)	0.46 (0.25,0.75)	-0.36 (-0.37,-0.35)	2864 (1643,4394)	15.25 (8.82,23.30)	4911 (2644,8079)	14.04 (7.59,22.96)	-0.27 (-0.28,-0.26)
Democratic R	19 (11,28)	0.12 (0.07,0.19)	36 (19,60)	0.10 (0.05,0.17)	-0.72 (-0.76,-0.68)	544 (316,830)	3.16 (1.80,4.77)	1092 (568,1830)	2.59 (1.35,4.29)	-0.63 (-0.68,-0.58)
Denmark	170 (103,236)	2.09 (1.27,2.90)	132 (75,195)	1.06 (0.60,1.56)	-2.14 (-2.23,-2.03)	3809 (2348,5272)	50.42 (31.08,69.57)	2721 (1558,3986)	23.99 (13.85,34.91)	-2.27 (-2.37,-2.16)
Djibouti	1 (0,1)	0.41 (0.23,0.66)	3 (2,5)	0.58 (0.32,0.92)	1.17 (1.14,1.20)	16 (8,26)	10.11 (5.61,16.55)	92 (49,148)	13.35 (7.27,21.08)	0.92 (0.88,0.95)
Dominica	0 (0,0)	0.34 (0.20,0.50)	0 (0,0)	0.39 (0.22,0.59)	0.45 (0.42,0.47)	5 (3,7)	8.2 (4.95,11.75)	8 (5,12)	9.58 (5.65,14.34)	0.50 (0.47,0.52)
Dominican R	13 (8,19)	0.39 (0.23,0.56)	40 (24,61)	0.41 (0.24,0.62)	0.16 (0.02,0.31)	320 (197,461)	8.57 (5.28,12.44)	960 (575,1496)	9.51 (5.69,14.82)	0.40 (0.31,0.47)
Ecuador	12 (8,17)	0.25 (0.15,0.35)	39 (21,59)	0.24 (0.13,0.37)	-0.05 (-0.20,0.10)	310 (195,433)	5.76 (3.61,7.99)	960 (540,1442)	5.79 (3.27,8.71)	-0.01 (-0.24,0.17)
Egypt	67 (43,95)	0.24 (0.15,0.35)	238 (143,352)	0.39 (0.23,0.56)	1.62 (1.53,1.70)	2158 (1408,3064)	6.64 (4.28,9.45)	7243 (4359,10807)	9.96 (5.98,14.69)	1.35 (1.28,1.42)
El Salvador	3 (2,4)	0.10 (0.06,0.15)	11 (6,16)	0.17 (0.10,0.26)	1.76 (1.55,1.93)	89 (54,126)	2.84 (1.70,4.02)	307 (181,463)	5.11 (3.02,7.71)	1.91 (1.70,2.07)
Equatorial Gu	0 (0,1)	0.19 (0.11,0.30)	1 (1,2)	0.24 (0.13,0.39)	0.68 (0.62,0.74)	11 (6,18)	5.21 (2.76,8.20)	36 (18,58)	6.10 (3.20,9.94)	0.53 (0.45,0.60)
Eritrea	2 (1,3)	0.14 (0.08,0.21)	4 (2,7)	0.13 (0.07,0.21)	-0.19 (-0.23,-0.15)	65 (36,101)	4.14 (2.33,6.34)	137 (72,239)	3.73 (2.00,6.30)	-0.36 (-0.41,-0.32)
Estonia	17 (11,24)	0.84 (0.54,1.17)	19 (12,29)	0.75 (0.47,1.08)	-0.39 (-0.58,-0.20)	494 (318,692)	24.38 (15.6,34.23)	467 (292,678)	19.80 (12.32,28.63)	-0.76 (-0.92,-0.58)
Eswatini	1 (0,1)	0.31 (0.17,0.49)	2 (1,2)	0.29 (0.15,0.46)	-0.13 (-0.19,-0.09)	21 (11,34)	7.22 (3.96,11.44)	43 (22,67)	7.14 (3.64,11.09)	-0.03 (-0.07,0.01)
Ethiopia	60 (28,95)	0.31 (0.15,0.48)	72 (42,109)	0.18 (0.11,0.28)	-1.64 (-1.71,-1.59)	1685 (792,2667)	7.84 (3.78,12.39)	1821 (1057,2780)	4.16 (2.42,6.29)	-2.00 (-2.04,-1.95)
Fiji	2 (1,2)	0.44 (0.24,0.66)	3 (1,5)	0.36 (0.19,0.58)	-0.63 (-0.71,-0.54)	50 (27,73)	12.14 (6.61,17.76)	83 (43,131)	9.62 (5.01,15.16)	-0.74 (-0.82,-0.66)
Finland	40 (24,56)	0.58 (0.35,0.81)	45 (25,67)	0.38 (0.21,0.55)	-1.38 (-1.43,-1.32)	1084 (661,1518)	16.17 (9.91,22.70)	1079 (611,1567)	10.21 (5.81,14.69)	-1.47 (-1.53,-1.41)
France	980 (606,1368)	1.18 (0.73,1.64)	890 (525,1292)	0.66 (0.40,0.96)	-1.84 (-1.89,-1.80)	22937 (14486,3139	29.56 (18.73,40.43)	20788 (12492,30214	17.66 (10.63,25.65)	-1.63 (-1.68,-1.58)
Gabon	2 (1,3)	0.27 (0.12,0.47)	3 (2,5)	0.29 (0.15,0.47)	0.18 (0.14,0.21)	43 (19,77)	7.26 (3.22,12.80)	90 (47,140)	7.58 (3.99,11.93)	0.15 (0.11,0.19)
Gambia	0 (0,0)	0.07 (0.04,0.10)	0 (0,1)	0.05 (0.03,0.08)	-0.95 (-1.06,-0.84)	7 (4,11)	1.84 (1.09,2.80)	14 (8,22)	1.32 (0.73,2.06)	-0.99 (-1.11,-0.85)
Georgia	32 (20,43)	0.49 (0.31,0.68)	43 (26,61)	0.75 (0.45,1.05)	1.36 (1.02,1.67)	981 (612,1347)	15.41 (9.60,21.1)	1153 (708,1626)	20.80 (12.90,29.38)	0.99 (0.69,1.28)
Germany	1670 (1005,2406)	1.31 (0.79,1.88)	1214 (698,1747)	0.65 (0.38,0.93)	-2.19 (-2.29,-2.08)	40254 (24493,5712	33.51 (20.50,47.31)	28440 (16496,40538	17.38 (10.06,24.52)	-2.05 (-2.14,-1.96)
Ghana	4 (2,6)	0.07 (0.04,0.11)	13 (8,20)	0.09 (0.05,0.14)	0.84 (0.81,0.86)	98 (57,149)	1.58 (0.91,2.40)	338 (200,516)	2.01 (1.19,3.02)	0.79 (0.77,0.81)
Greece	137 (87,188)	0.90 (0.57,1.24)	201 (120,289)	0.84 (0.51,1.19)	-0.31 (-0.45,-0.21)	3248 (2047,4444)	21.81 (13.79,29.83)	4302 (2621,6051)	21 (12.88,29.4)	-0.20 (-0.33,-0.10)
Greenland	1 (1,1)	2.90 (1.78,4.08)	1 (1,2)	1.55 (0.93,2.24)	-1.96 (-2.03,-1.87)	33 (20,46)	80.41 (49.2,112.19)	33 (20,47)	42.29 (25.46,60.57)	-2.00 (-2.07,-1.92)
Grenada	0 (0,0)	0.36 (0.22,0.54)	1 (0,1)	0.43 (0.25,0.65)	0.68 (0.56,0.81)	6 (4,9)	9.70 (5.84,13.94)	13 (8,20)	10.94 (6.37,16.15)	0.40 (0.27,0.54)
Guam	0 (0,1)	0.59 (0.36,0.85)	1 (1,1)	0.45 (0.27,0.65)	-0.91 (-1.09,-0.78)	15 (9,21)	16.26 (9.86,23.14)	29 (18,41)	13.81 (8.43,19.93)	-0.58 (-0.71,-0.48)
Guatemala	4 (2,5)	0.12 (0.07,0.17)	17 (10,25)	0.15 (0.09,0.23)	1.06 (0.83,1.40)	102 (63,146)	2.76 (1.68,3.97)	447 (258,655)	3.89 (2.24,5.68)	1.24 (1.04,1.53)
Guinea	3 (2,4)	0.09 (0.05,0.13)	5 (3,8)	0.09 (0.05,0.15)	0.24 (0.21,0.26)	73 (44,110)	2.14 (1.27,3.27)	138 (74,218)	2.32 (1.26,3.71)	0.28 (0.24,0.30)
Guinea-Bissa	0 (0,1)	0.09 (0.04,0.14)	1 (0,1)	0.10 (0.05,0.15)	0.35 (0.32,0.37)	10 (5,16)	2.29 (1.11,3.66)	22 (12,34)	2.57 (1.35,3.99)	0.38 (0.35,0.41)

Guyana	1 (1,2)	0.35 (0.21,0.51)	2 (1,4)	0.37 (0.20,0.58)	0.38 (0.19,0.57)	37 (23,54)	9.16 (5.64,13.18)	70 (38,112)	10.00 (5.38,15.81)	0.41 (0.19,0.65)
Haiti	11 (6,17)	0.34 (0.18,0.54)	17 (8,28)	0.26 (0.12,0.42)	-0.90 (-0.93,-0.87)	319 (167,497)	9.04 (4.72,14.09)	485 (245,781)	6.25 (3.08,10.19)	-1.14 (-1.17,-1.12)
Honduras	3 (2,4)	0.13 (0.08,0.20)	11 (7,17)	0.18 (0.11,0.27)	1.02 (0.95,1.08)	81 (48,119)	3.62 (2.14,5.34)	304 (180,447)	4.53 (2.67,6.70)	0.80 (0.75,0.84)
Hungary	226 (140,324)	1.54 (0.95,2.21)	225 (131,333)	1.23 (0.72,1.81)	-0.70 (-0.77,-0.62)	6199 (3876,8912)	43.32 (27.17,62.40)	5934 (3519,8669)	34.84 (20.76,50.51)	-0.70 (-0.77,-0.62)
Iceland	2 (1,3)	0.85 (0.53,1.18)	2 (1,3)	0.40 (0.24,0.58)	-2.43 (-2.55,-2.30)	59 (37,80)	21.47 (13.61,29.36)	54 (32,79)	10.02 (5.96,14.54)	-2.38 (-2.53,-2.25)
India	842 (523,1206)	0.18 (0.11,0.26)	1620 (958,2384)	0.14 (0.08,0.20)	-0.86 (-0.96,-0.76)	24876 (15534,3583	4.84 (3.02,6.95)	43481 (25450,63613	3.47 (2.03,5.07)	-1.00 (-1.05,-0.94)
Indonesia	406 (245,590)	0.43 (0.26,0.62)	1499 (840,2359)	0.65 (0.36,1.02)	1.36 (1.34,1.37)	11992 (7267,17414)	11.02 (6.67,16.02)	42519 (23555,67164	16.05 (8.94,25.30)	1.23 (1.21,1.25)
Iran (Islamic	57 (34,84)	0.21 (0.12,0.31)	175 (107,249)	0.22 (0.14,0.32)	0.15 (0.08,0.22)	1766 (1059,2599)	5.88 (3.49,8.67)	5097 (3177,7301)	5.94 (3.66,8.49)	0.05 (-0.04,0.12)
Iraq	26 (15,39)	0.33 (0.19,0.49)	78 (44,117)	0.34 (0.20,0.51)	0.19 (0.14,0.25)	749 (437,1131)	8.82 (5.16,13.28)	2227 (1252,3370)	8.44 (4.82,12.78)	-0.13 (-0.17,-0.09)
Ireland	71 (44,98)	1.74 (1.09,2.40)	47 (29,70)	0.59 (0.36,0.87)	-3.44 (-3.52,-3.37)	1642 (1041,2248)	41.25 (26.26,56.56)	1093 (678,1583)	14.36 (8.86,20.70)	-3.37 (-3.45,-3.31)
Israel	49 (30,69)	1.02 (0.62,1.44)	57 (34,84)	0.46 (0.28,0.68)	-2.53 (-2.61,-2.45)	1193 (735,1653)	25.18 (15.50,34.86)	1324 (799,1920)	11.48 (6.93,16.62)	-2.53 (-2.61,-2.45)
Italy	985 (619,1339)	1.11 (0.70,1.51)	821 (500,1170)	0.58 (0.36,0.81)	-2.13 (-2.18,-2.09)	24540 (15621,3332	29.11 (18.58,39.36)	18519 (11725,25797	14.94 (9.57,20.69)	-2.14 (-2.20,-2.09)
Jamaica	7 (4,10)	0.39 (0.23,0.56)	15 (8,23)	0.48 (0.26,0.75)	1.19 (0.67,1.90)	168 (103,243)	9.75 (5.99,14.08)	374 (207,588)	12.19 (6.77,19.16)	1.27 (0.72,2.03)
Japan	1923 (1229,2586)	1.13 (0.72,1.52)	2120 (1296,2984)	0.62 (0.39,0.86)	-1.94 (-1.98,-1.90)	52180 (33926,6975	30.31 (19.71,40.52)	45692 (28780,63207	16.53 (10.47,22.64)	-1.96 (-2.00,-1.93)
Jordan	8 (5,12)	0.63 (0.38,0.92)	37 (21,57)	0.50 (0.30,0.77)	-0.71 (-0.77,-0.66)	254 (156,371)	16.48 (10.11,24.13)	1119 (650,1736)	12.85 (7.45,19.96)	-0.79 (-0.84,-0.75)
Kazakhstan	76 (49,104)	0.56 (0.36,0.77)	68 (42,95)	0.35 (0.22,0.49)	-1.42 (-1.59,-1.22)	2442 (1575,3338)	17.47 (11.16,23.85)	2067 (1278,2882)	10.33 (6.41,14.40)	-1.66 (-1.85,-1.38)
Kenya	10 (6,14)	0.13 (0.07,0.18)	32 (19,47)	0.14 (0.08,0.21)	0.37 (0.33,0.41)	274 (158,398)	3.16 (1.83,4.62)	900 (531,1361)	3.58 (2.13,5.36)	0.41 (0.36,0.46)
Kiribati	0 (0,0)	0.66 (0.40,0.95)	0 (0,1)	0.64 (0.39,0.96)	-0.09 (-0.11,-0.07)	7 (4,11)	18.07 (10.78,25.60)	15 (9,23)	17.52 (10.51,26.63)	-0.09 (-0.11,-0.07)
Kuwait	1 (1,2)	0.24 (0.14,0.33)	10 (6,14)	0.33 (0.20,0.51)	1.91 (1.30,2.57)	46 (28,64)	6.05 (3.65,8.44)	296 (178,445)	8.06 (4.82,12.04)	1.60 (0.97,2.29)
Kyrgyzstan	12 (8,17)	0.40 (0.25,0.55)	16 (10,23)	0.31 (0.19,0.47)	-0.70 (-0.82,-0.59)	388 (250,533)	12.23 (7.85,16.80)	504 (317,723)	9.03 (5.62,13.05)	-0.96 (-1.08,-0.84)
Lao People's	15 (7,25)	0.73 (0.35,1.22)	34 (20,52)	0.77 (0.45,1.15)	0.18 (0.17,0.20)	440 (205,737)	19.41 (9.13,32.58)	966 (552,1477)	19.17 (11.02,28.98)	-0.03 (-0.04,-0.01)
Latvia	30 (19,42)	0.84 (0.53,1.17)	28 (17,40)	0.76 (0.48,1.09)	-0.23 (-0.37,-0.05)	852 (538,1186)	24.11 (15.20,33.56)	718 (457,1023)	21.27 (13.65,30.37)	-0.37 (-0.51,-0.19)
Lebanon	16 (9,24)	0.74 (0.43,1.12)	43 (25,63)	0.69 (0.40,1.02)	-0.17 (-0.22,-0.12)	425 (233,642)	18.56 (10.44,28.17)	973 (576,1417)	16.46 (9.78,23.88)	-0.35 (-0.40,-0.30)
Lesotho	2 (1,3)	0.21 (0.12,0.33)	5 (3,8)	0.50 (0.27,0.78)	2.93 (2.85,3.00)	44 (25,69)	5.11 (2.87,7.91)	158 (85,250)	13.36 (7.26,20.75)	3.20 (3.13,3.27)
Liberia	1 (1,2)	0.09 (0.05,0.14)	2 (1,3)	0.08 (0.03,0.13)	-0.57 (-0.64,-0.52)	27 (16,41)	2.3 (1.32,3.44)	47 (21,83)	1.98 (0.89,3.46)	-0.51 (-0.56,-0.46)
Libya	9 (5,14)	0.49 (0.28,0.77)	27 (15,42)	0.52 (0.29,0.79)	0.19 (0.15,0.24)	264 (156,411)	12.87 (7.51,20.04)	824 (467,1257)	13.45 (7.44,20.62)	0.18 (0.13,0.23)
Lithuania	33 (20,45)	0.72 (0.44,1.00)	41 (25,59)	0.73 (0.45,1.05)	-0.02 (-0.16,0.12)	892 (550,1216)	19.91 (12.28,27.12)	980 (605,1408)	19.36 (11.94,27.68)	-0.15 (-0.31,-0.01)
Luxembourg	7 (5,10)	1.37 (0.84,1.92)	6 (4,9)	0.60 (0.35,0.88)	-2.60 (-2.71,-2.49)	189 (118,263)	35.97 (22.62,49.82)	152 (90,220)	14.95 (8.92,21.53)	-2.81 (-2.91,-2.72)
Madagascar	10 (6,15)	0.21 (0.12,0.31)	13 (7,20)	0.13 (0.07,0.20)	-1.48 (-1.52,-1.44)	268 (161,402)	5.06 (3.02,7.57)	376 (194,592)	3.04 (1.58,4.77)	-1.61 (-1.66,-1.56)
Malawi	5 (3,7)	0.14 (0.08,0.20)	10 (6,16)	0.15 (0.09,0.24)	0.36 (0.33,0.40)	120 (73,174)	3.10 (1.90,4.53)	279 (158,453)	3.62 (2.07,5.76)	0.51 (0.47,0.55)
Malaysia	69 (43,99)	0.78 (0.48,1.14)	189 (116,272)	0.69 (0.42,1.00)	-0.40 (-0.60,-0.17)	1859 (1151,2640)	19.07 (11.77,27.17)	4962 (2990,7129)	16.80 (10.27,23.95)	-0.37 (-0.54,-0.18)
Maldives	0 (0,1)	0.48 (0.26,0.74)	1 (0,1)	0.27 (0.16,0.40)	-1.84 (-1.95,-1.70)	10 (5,16)	11.06 (6.03,16.83)	21 (12,31)	5.79 (3.38,8.61)	-2.12 (-2.25,-1.98)
Mali	4 (3,7)	0.13 (0.07,0.19)	13 (8,22)	0.17 (0.1,0.28)	1.06 (1.03,1.10)	119 (70,179)	2.92 (1.72,4.43)	347 (201,557)	3.88 (2.26,6.21)	0.95 (0.92,0.99)
Malta	4 (2,5)	0.84 (0.51,1.20)	4 (2,6)	0.46 (0.26,0.67)	-1.85 (-1.97,-1.69)	96 (58,135)	22.39 (13.55,31.45)	102 (60,148)	12.36 (7.31,17.76)	-1.75 (-1.95,-1.56)
Marshall Isla	0 (0,0)	0.36 (0.21,0.55)	0 (0,0)	0.39 (0.22,0.60)	0.21 (0.15,0.29)	2 (1,3)	10.06 (5.96,15.31)	5 (2,7)	10.68 (6.03,16.73)	0.21 (0.16,0.27)
Mauritania	1 (1,2)	0.13 (0.07,0.20)	2 (1,4)	0.11 (0.06,0.17)	-0.51 (-0.56,-0.46)	36 (21,55)	3.41 (1.96,5.18)	63 (34,97)	2.74 (1.49,4.21)	-0.72 (-0.78,-0.67)
Mauritius	2 (2,3)	0.34 (0.21,0.47)	12 (8,17)	0.64 (0.42,0.90)	2.01 (1.78,2.26)	72 (45,97)	9.11 (5.74,12.39)	317 (202,443)	16.77 (10.77,23.37)	2.04 (1.81,2.30)
Mexico	122 (77,166)	0.31 (0.19,0.42)	338 (207,474)	0.27 (0.16,0.38)	-0.35 (-0.43,-0.26)	3142 (2015,4265)	7.14 (4.56,9.72)	8949 (5521,12581)	6.82 (4.19,9.59)	-0.04 (-0.12,0.05)
Micronesia (F	0 (0,0)	0.68 (0.40,0.98)	1 (0,1)	0.64 (0.35,0.99)	-0.18 (-0.19,-0.17)	10 (6,15)	19.14 (11.4,28.18)	16 (9,25)	18.22 (9.95,28.2)	-0.16 (-0.17,-0.16)
Monaco	1 (0,1)	1.29 (0.74,1.86)	1 (1,2)	1.11 (0.63,1.69)	-0.51 (-0.52,-0.49)	20 (11,29)	34.07 (19.47,50.35)	24 (14,36)	29.19 (16.52,45.02)	-0.51 (-0.52,-0.49)
Mongolia	3 (2,5)	0.31 (0.18,0.49)	9 (5,14)	0.37 (0.22,0.57)	0.49 (0.40,0.57)	101 (59,158)	9.00 (5.26,14.07)	290 (165,441)	10.55 (6.03,16.02)	0.44 (0.36,0.52)
Montenegro	6 (4,8)	0.93 (0.56,1.29)	10 (6,15)	0.99 (0.62,1.47)	0.49 (0.34,0.62)	169 (103,235)	25.99 (15.82,36.03)	259 (161,383)	26.24 (16.31,38.68)	0.16 (0.02,0.26)
Morocco	22 (12,34)	0.15 (0.09,0.23)	60 (32,91)	0.17 (0.09,0.26)	0.31 (0.29,0.34)	670 (363,1014)	4.34 (2.35,6.55)	1727 (931,2621)	4.63 (2.49,7.02)	0.21 (0.19,0.24)
Mozambique	4 (2,6)	0.08 (0.05,0.12)	9 (5,13)	0.09 (0.05,0.14)	0.36 (0.33,0.40)	101 (61,148)	1.74 (1.06,2.55)	230 (135,342)	2.04 (1.20,3.02)	0.55 (0.52,0.58)
Myanmar	199 (109,306)	0.89 (0.50,1.34)	263 (152,395)	0.56 (0.33,0.83)	-1.49 (-1.50,-1.48)	5558 (2962,8754)	22.64 (12.24,35.23)	6821 (4002,10232)	13.38 (7.75,19.99)	-1.70 (-1.71,-1.68)
Namibia	1 (1,2)	0.27 (0.16,0.40)	3 (2,4)	0.24 (0.15,0.34)	-0.37 (-0.43,-0.32)	38 (23,56)	5.96 (3.63,8.73)	73 (44,104)	5.27 (3.20,7.50)	-0.36 (-0.42,-0.30)
Nauru	0 (0,0)	1.03 (0.49,1.74)	0 (0,0)	0.80 (0.41,1.25)	-0.80 (-0.81,-0.78)	2 (1,3)	28.91 (13.57,48.28)	2 (1,3)	23.38 (11.85,35.75)	-0.68 (-0.69,-0.67)
Nepal	27 (14,41)	0.31 (0.16,0.47)	45 (26,69)	0.21 (0.12,0.32)	-1.25 (-1.27,-1.23)	733 (383,1155)	7.44 (3.92,11.61)	1098 (635,1685)	4.65 (2.70,7.17)	-1.50 (-1.52,-1.48)
Netherlands	328 (206,451)	1.64 (1.03,2.26)	378 (226,555)	1.06 (0.64,1.54)	-1.39 (-1.55,-1.28)	8106 (5071,11108)	42.32 (26.54,58.07)	8667 (5276,12431)	26.28 (16.17,37.54)	-1.54 (-1.67,-1.43)
New Zealand	68 (41,95)	1.75 (1.07,2.44)	67 (41,101)	0.78 (0.48,1.18)	-2.62 (-2.69,-2.55)	1757 (1098,2454)	46.22 (28.97,64.52)	1514 (923,2265)	18.93 (11.6,28.28)	-2.86 (-3.02,-2.73)
Nicaragua	2 (1,3)	0.13 (0.08,0.19)	8 (5,12)	0.16 (0.09,0.24)	0.60 (0.39,0.76)	59 (37,86)	3.47 (2.16,5.05)	222 (131,327)	4.23 (2.49,6.24)	0.60 (0.33,0.82)
Niger	1 (1,2)	0.05 (0.03,0.08)	3 (2,5)	0.05 (0.02,0.08)	-0.15 (-0.18,-0.11)	34 (18,54)	1.18 (0.62,1.87)	84 (42,138)	1.03 (0.52,1.65)	-0.43 (-0.47,-0.40)
Nigeria	25 (14,40)	0.06 (0.03,0.09)	36 (20,54)	0.04 (0.02,0.06)	-1.20 (-1.22,-1.18)	664 (372,1071)	1.45 (0.81,2.32)	1008 (559,1504)	1.01 (0.57,1.50)	-1.17 (-1.19,-1.15)
Niue	0 (0,0)	0.40 (0.23,0.6)	0 (0,0)	0.41 (0.24,0.60)	0.09 (0.08,0.11)	0 (0,0)	11.05 (6.49,16.99)	0 (0,0)	11.07 (6.55,16.51)	0.00 (-0.01,0.02)
North Maced	18 (11,26)	0.96 (0.61,1.35)	34 (20,49)	1.01 (0.60,1.48)	0.18 (0.04,0.29)	520 (329,721)	25.99 (16.41,36.08)	886 (532,1312)	25.91 (15.52,38.24)	-0.05 (-0.18,0.05)

Northern Mar	0 (0,0)	0.76 (0.46,1.13)	0 (0,1)	0.65 (0.40,0.91)	-0.45 (-0.51,-0.38)	5 (3,7)	20.29 (12.32,29.85)	10 (6,15)	17.18 (10.68,24.62)	-0.45 (-0.51,-0.38)
Norway	96 (61,131)	1.45 (0.92,1.97)	53 (32,76)	0.53 (0.32,0.76)	-3.24 (-3.33,-3.16)	2239 (1416,3055)	37.22 (23.86,50.93)	1217 (759,1702)	13.54 (8.56,18.88)	-3.25 (-3.32,-3.18)
Oman	1 (0,1)	0.12 (0.07,0.19)	2 (1,3)	0.09 (0.05,0.13)	-1.04 (-1.11,-0.96)	25 (14,39)	3.18 (1.76,5.03)	52 (30,84)	2.11 (1.20,3.30)	-1.31 (-1.37,-1.25)
Pakistan	141 (90,198)	0.26 (0.16,0.37)	254 (149,380)	0.22 (0.13,0.33)	-0.56 (-0.58,-0.54)	3733 (2364,5235)	6.42 (4.05,9.02)	7049 (4030,10581)	5.39 (3.13,8.08)	-0.57 (-0.59,-0.54)
Palau	0 (0,0)	0.31 (0.17,0.48)	0 (0,0)	0.25 (0.14,0.36)	-0.80 (-0.84,-0.76)	1 (0,1)	8.28 (4.72,12.89)	2 (1,2)	6.27 (3.63,9.2)	-0.89 (-0.93,-0.87)
Palestine	6 (3,9)	0.76 (0.42,1.13)	15 (9,21)	0.61 (0.36,0.86)	-0.76 (-0.81,-0.70)	164 (92,246)	18.35 (10.16,27.51)	425 (251,597)	15.06 (8.81,21.26)	-0.69 (-0.74,-0.64)
Panama	4 (2,6)	0.28 (0.17,0.40)	11 (6,17)	0.25 (0.14,0.38)	-0.27 (-0.37,-0.16)	102 (62,143)	6.73 (4.13,9.46)	279 (163,417)	6.33 (3.69,9.47)	-0.14 (-0.24,-0.03)
Papua New G	3 (2,4)	0.14 (0.08,0.22)	6 (4,9)	0.11 (0.06,0.16)	-0.76 (-0.81,-0.71)	90 (49,141)	4.06 (2.24,6.31)	207 (121,306)	3.18 (1.86,4.74)	-0.77 (-0.81,-0.73)
Paraguay	9 (6,13)	0.43 (0.28,0.62)	31 (18,48)	0.55 (0.32,0.86)	1.04 (0.95,1.14)	224 (141,319)	9.94 (6.26,14.27)	760 (438,1154)	12.84 (7.39,19.48)	0.99 (0.90,1.07)
Peru	22 (13,32)	0.19 (0.11,0.28)	68 (37,108)	0.21 (0.11,0.33)	0.37 (0.13,0.54)	545 (326,804)	4.46 (2.69,6.60)	1642 (913,2581)	4.86 (2.69,7.63)	0.36 (0.11,0.54)
Philippines	208 (131,292)	0.72 (0.45,1.01)	591 (360,832)	0.71 (0.43,1.01)	0.02 (-0.03,0.07)	6412 (4059,8963)	18.93 (11.93,26.58)	17388 (10655,24218	19.06 (11.64,26.59)	0.04 (-0.03,0.09)
Poland	564 (367,776)	1.28 (0.83,1.76)	839 (518,1175)	1.18 (0.73,1.66)	-0.35 (-0.40,-0.29)	15740 (10217,2145	35.86 (23.29,48.93)	20554 (12866,28732	30.59 (19.23,42.71)	-0.53 (-0.60,-0.48)
Portugal	114 (68,164)	0.82 (0.49,1.17)	122 (74,175)	0.56 (0.34,0.79)	-1.12 (-1.21,-1.04)	3028 (1820,4350)	22.46 (13.55,32.27)	3170 (1957,4505)	16.27 (10.11,23.10)	-0.98 (-1.07,-0.82)
Puerto Rico	18 (11,27)	0.51 (0.31,0.74)	31 (17,48)	0.45 (0.26,0.70)	-0.43 (-0.59,-0.28)	474 (286,684)	13.15 (7.96,18.97)	748 (427,1143)	12.42 (7.09,18.94)	-0.10 (-0.25,0.06)
Qatar	0 (0,1)	0.31 (0.19,0.48)	2 (1,4)	0.23 (0.13,0.37)	-1.03 (-1.27,-0.78)	12 (7,18)	7.73 (4.62,11.74)	86 (49,142)	5.60 (3.14,9.11)	-1.10 (-1.28,-0.95)
Republic of K	222 (140,307)	0.77 (0.48,1.06)	523 (313,758)	0.56 (0.33,0.81)	-1.08 (-1.13,-1.03)	6628 (4102,9326)	19.8 (12.43,27.48)	12021 (7355,17528)	13.03 (7.91,18.96)	-1.41 (-1.46,-1.36)
Republic of M	31 (20,44)	0.68 (0.43,0.96)	51 (31,74)	0.85 (0.52,1.23)	0.73 (0.54,0.92)	938 (591,1318)	20.15 (12.69,28.36)	1425 (872,2041)	24.23 (14.82,34.58)	0.64 (0.39,0.94)
Romania	195 (124,275)	0.68 (0.44,0.97)	368 (216,526)	1.04 (0.62,1.48)	1.61 (1.48,1.76)	5816 (3744,8102)	20.25 (12.94,28.31)	9715 (5770,13856)	29.52 (17.57,41.85)	1.44 (1.31,1.60)
Russian Fede	1367 (878,1844)	0.74 (0.47,1.00)	1948 (1258,2620)	0.81 (0.52,1.09)	0.41 (0.25,0.63)	40006 (25947,5380	21.59 (13.99,29)	52956 (34615,71247	22.70 (14.85,30.43)	0.29 (0.15,0.47)
Rwanda	13 (8,19)	0.50 (0.30,0.74)	26 (15,41)	0.48 (0.28,0.77)	-0.06 (-0.09,-0.03)	343 (200,507)	11.77 (6.92,17.33)	657 (374,1051)	10.42 (5.94,16.61)	-0.37 (-0.41,-0.34)
Saint Kitts an	0 (0,0)	0.36 (0.21,0.53)	0 (0,0)	0.36 (0.20,0.54)	0.05 (-0.08,0.18)	3 (2,5)	8.92 (5.32,13.03)	6 (4,9)	8.57 (4.79,12.70)	-0.14 (-0.29,0.02)
Saint Lucia	0 (0,0)	0.35 (0.20,0.51)	1 (0,1)	0.29 (0.16,0.45)	-0.54 (-0.65,-0.44)	8 (5,11)	8.94 (5.25,12.66)	18 (10,27)	7.33 (4.14,11.13)	-0.54 (-0.63,-0.45)
Saint Vincent	0 (0,0)	0.29 (0.17,0.42)	1 (0,1)	0.35 (0.21,0.51)	0.62 (0.52,0.72)	5 (3,7)	7.32 (4.38,10.38)	13 (8,19)	8.83 (5.28,12.77)	0.57 (0.45,0.66)
Samoa	0 (0,0)	0.37 (0.22,0.54)	0 (0,1)	0.31 (0.19,0.46)	-0.60 (-0.61,-0.58)	9 (5,13)	9.98 (5.88,14.49)	13 (8,19)	8.44 (5.13,12.26)	-0.55 (-0.57,-0.54)
San Marino	0 (0,1)	1.15 (0.69,1.67)	0 (0,1)	0.43 (0.22,0.71)	-3.39 (-3.73,-3.17)	10 (6,14)	28.52 (17.00,40.50)	7 (4,12)	10.89 (5.63,18.07)	-3.29 (-3.60,-3.09)
Sao Tome an	0 (0,0)	0.13 (0.08,0.20)	0 (0,0)	0.18 (0.10,0.29)	1.08 (1.02,1.12)	2 (1,3)	3.35 (1.89,5.02)	6 (3,9)	4.60 (2.53,7.08)	1.03 (0.98,1.07)
Saudi Arabia	7 (4,11)	0.11 (0.06,0.17)	45 (27,68)	0.19 (0.11,0.28)	1.78 (1.76,1.81)	242 (130,383)	3.17 (1.72,5.03)	1660 (978,2515)	5.47 (3.27,8.19)	1.76 (1.73,1.79)
Senegal	4 (2,6)	0.11 (0.07,0.17)	7 (4,11)	0.09 (0.05,0.14)	-0.78 (-0.96,-0.65)	113 (65,168)	3.19 (1.84,4.73)	210 (110,330)	2.46 (1.28,3.83)	-1.02 (-1.16,-0.88)
Serbia	147 (89,217)	1.32 (0.80,1.92)	190 (112,278)	1.18 (0.70,1.72)	-0.34 (-0.46,-0.23)	4200 (2567,6216)	35.28 (21.59,52.02)	4871 (2951,7113)	32.14 (19.51,46.56)	-0.33 (-0.46,-0.20)
Seychelles	0 (0,1)	0.79 (0.47,1.12)	1 (1,1)	0.89 (0.56,1.27)	0.37 (0.21,0.51)	12 (7,16)	20.81 (12.32,29.11)	28 (18,39)	22.55 (14.22,31.79)	0.24 (0.12,0.35)
Sierra Leone	2 (1,4)	0.12 (0.07,0.18)	4 (2,6)	0.10 (0.06,0.15)	-0.73 (-0.76,-0.70)	66 (38,99)	3.16 (1.81,4.75)	103 (59,163)	2.53 (1.49,3.98)	-0.70 (-0.74,-0.67)
Singapore	16 (10,22)	0.70 (0.43,0.98)	24 (15,34)	0.27 (0.17,0.39)	-2.89 (-3.00,-2.78)	452 (275,637)	18.57 (11.27,26.3)	635 (396,892)	7.13 (4.45,10.05)	-2.99 (-3.10,-2.90)
Slovakia	88 (55,127)	1.47 (0.91,2.10)	115 (70,171)	1.19 (0.73,1.76)	-0.67 (-0.74,-0.60)	2456 (1523,3483)	41.46 (25.67,58.7)	2954 (1814,4320)	31.74 (19.29,46.11)	-0.88 (-0.97,-0.80)
Slovenia	21 (13,31)	0.86 (0.52,1.26)	25 (14,37)	0.58 (0.34,0.87)	-1.45 (-1.60,-1.31)	613 (378,882)	24.95 (15.39,35.87)	608 (354,900)	15.68 (9.16,23.10)	-1.70 (-1.86,-1.55)
Solomon Isla	1 (0,1)	0.50 (0.25,0.77)	2 (1,3)	0.48 (0.26,0.74)	-0.15 (-0.21,-0.11)	22 (10,34)	13.71 (6.48,21.25)	56 (30,85)	13.60 (7.27,20.79)	-0.05 (-0.11,-0.00)
Somalia	5 (2,10)	0.23 (0.11,0.42)	11 (5,21)	0.19 (0.09,0.34)	-0.58 (-0.59,-0.56)	164 (77,297)	5.99 (2.81,10.87)	351 (156,640)	4.98 (2.24,9.08)	-0.59 (-0.60,-0.57)
South Africa	85 (54,126)	0.41 (0.26,0.62)	152 (95,215)	0.32 (0.20,0.46)	-0.84 (-0.94,-0.75)	2522 (1585,3602)	11.16 (7.01,16.21)	4516 (2845,6385)	8.87 (5.59,12.52)	-0.77 (-0.89,-0.66)
South Sudan	7 (4,12)	0.29 (0.15,0.50)	10 (5,16)	0.28 (0.13,0.44)	-0.15 (-0.17,-0.13)	190 (97,333)	7.17 (3.72,12.53)	280 (137,458)	6.64 (3.23,10.76)	-0.25 (-0.28,-0.23)
Spain	589 (362,826)	1.09 (0.67,1.53)	695 (422,997)	0.73 (0.45,1.05)	-1.28 (-1.34,-1.23)	14853 (9191,20765)	28.89 (17.99,40.35)	16541 (10253,23397	19.42 (12.06,27.36)	-1.30 (-1.37,-1.22)
Sri Lanka	24 (15,34)	0.25 (0.15,0.35)	39 (20,63)	0.15 (0.07,0.23)	-1.70 (-1.86,-1.55)	636 (389,885)	5.71 (3.47,7.99)	987 (493,1628)	3.55 (1.78,5.84)	-1.53 (-1.72,-1.37)
Sudan	15 (8,24)	0.16 (0.09,0.25)	30 (15,51)	0.15 (0.08,0.26)	-0.09 (-0.09,-0.08)	446 (241,718)	4.36 (2.34,7.00)	905 (460,1549)	4.01 (2.05,6.86)	-0.27 (-0.28,-0.27)
Suriname	1 (1,2)	0.56 (0.34,0.80)	3 (2,5)	0.53 (0.30,0.83)	-0.11 (-0.30,0.06)	41 (25,58)	15.04 (9.21,21.25)	96 (56,147)	14.42 (8.29,22.20)	-0.07 (-0.20,0.05)
Sweden	149 (91,213)	0.99 (0.61,1.41)	132 (77,193)	0.59 (0.35,0.87)	-1.66 (-1.74,-1.60)	3350 (2082,4780)	24.61 (15.48,34.95)	2737 (1616,4029)	14.06 (8.20,20.60)	-1.85 (-1.95,-1.77)
Switzerland	81 (52,113)	0.79 (0.50,1.09)	90 (56,133)	0.49 (0.30,0.72)	-1.60 (-1.72,-1.49)	1960 (1249,2708)	20.38 (12.91,28.18)	1953 (1215,2917)	11.75 (7.31,17.54)	-1.88 (-2.04,-1.76)
Syrian Arab F	17 (10,24)	0.31 (0.18,0.46)	32 (16,50)	0.25 (0.13,0.38)	-0.79 (-0.91,-0.68)	511 (300,749)	8.65 (5.12,12.70)	922 (474,1430)	6.30 (3.26,9.73)	-1.03 (-1.09,-0.96)
Taiwan (Prov	138 (88,190)	0.87 (0.55,1.20)	382 (240,531)	0.90 (0.57,1.26)	0.17 (0.04,0.32)	4075 (2587,5585)	23.57 (14.96,32.3)	9459 (5983,12994)	23.24 (14.87,31.92)	-0.09 (-0.25,0.07)
Tajikistan	8 (5,12)	0.30 (0.18,0.43)	7 (4,11)	0.12 (0.07,0.18)	-3.05 (-3.14,-2.96)	269 (168,383)	8.95 (5.54,12.73)	232 (132,382)	3.26 (1.90,5.09)	-3.28 (-3.39,-3.18)
Thailand	294 (183,422)	0.87 (0.53,1.25)	834 (459,1338)	0.76 (0.42,1.22)	-0.40 (-0.52,-0.28)	8161 (5137,11655)	21.46 (13.49,30.56)	21639 (11842,34947	19.97 (10.9,32.12)	-0.20 (-0.38,-0.05)
Timor-Leste	1 (1,2)	0.39 (0.21,0.59)	3 (2,5)	0.42 (0.24,0.61)	0.24 (0.20,0.28)	31 (17,48)	9.63 (5.26,14.73)	92 (53,135)	10.43 (6.02,15.28)	0.26 (0.22,0.31)
Togo	1 (1,2)	0.13 (0.07,0.19)	4 (2,7)	0.11 (0.06,0.18)	-0.40 (-0.42,-0.36)	42 (24,66)	3.18 (1.85,4.91)	126 (66,205)	2.90 (1.56,4.66)	-0.28 (-0.31,-0.24)
Tokelau	0 (0,0)	0.42 (0.25,0.62)	0 (0,0)	0.36 (0.20,0.55)	-0.56 (-0.57,-0.54)	0 (0,0)	11.29 (6.60,16.75)	0 (0,0)	9.67 (5.46,15.39)	-0.50 (-0.51,-0.48)
Tonga	0 (0,0)	0.35 (0.21,0.50)	0 (0,0)	0.31 (0.17,0.46)	-0.40 (-0.46,-0.33)	5 (3,7)	8.44 (5.14,12.33)	6 (4,9)	7.51 (4.34,11.27)	-0.39 (-0.45,-0.33)
Trinidad and	5 (3,7)	0.57 (0.35,0.81)	10 (5,15)	0.49 (0.28,0.77)	-0.41 (-0.58,-0.25)	127 (79,182)	14.74 (9.15,21.19)	263 (150,410)	13.42 (7.63,20.87)	-0.08 (-0.25,0.14)
Tunisia	20 (12,30)	0.42 (0.26,0.64)	50 (28,78)	0.38 (0.21,0.60)	-0.34 (-0.38,-0.29)	504 (315,752)	9.76 (6.10,14.62)	1253 (726,1983)	9.12 (5.24,14.44)	-0.24 (-0.28,-0.20)
Turkey	335 (206,483)	0.94 (0.58,1.36)	566 (331,836)	0.60 (0.35,0.88)	-1.44 (-1.48,-1.39)	10264 (6229,14626)	26.44 (16.18,37.77)	15811 (9327,23466)	16.17 (9.53,23.97)	-1.56 (-1.60,-1.53)

Turkmenistan	5 (3,7)	0.23 (0.15,0.32)	6 (3,9)	0.13 (0.08,0.20)	-1.72 (-1.95,-1.36)	159 (105,222)	7.29 (4.82,10.14)	190 (108,287)	4.05 (2.33,6.16)	-1.88 (-2.13,-1.65)
Tuvalu	0 (0,0)	0.48 (0.28,0.72)	0 (0,0)	0.46 (0.28,0.67)	-0.17 (-0.18,-0.16)	1 (1,2)	13.54 (7.88,20.20)	1 (1,2)	12.72 (7.63,18.48)	-0.20 (-0.21,-0.19)
Uganda	13 (7,19)	0.22 (0.13,0.32)	26 (16,41)	0.19 (0.11,0.29)	-0.41 (-0.45,-0.37)	344 (202,508)	5.16 (3.03,7.63)	743 (442,1149)	4.66 (2.81,7.18)	-0.37 (-0.43,-0.32)
Ukraine	707 (456,963)	0.98 (0.63,1.33)	507 (275,832)	0.66 (0.36,1.09)	-1.38 (-1.56,-1.21)	20627 (13458,2807[illegible]	29.18 (18.95,39.74)	14053 (7639,22928)	19.29 (10.48,31.47)	-1.46 (-1.63,-1.27)
United Arab [illegible]	12 (1,4)	0.46 (0.22,0.76)	9 (5,16)	0.27 (0.15,0.46)	-1.85 (-2.20,-1.55)	85 (42,147)	12.24 (5.86,20.41)	342 (187,593)	5.85 (3.14,9.97)	-2.57 (-2.99,-2.21)
United Kingd	1604 (998,2225)	1.76 (1.10,2.43)	960 (575,1379)	0.72 (0.44,1.03)	-2.87 (-2.96,-2.81)	36378 (22949,4998[illegible]	42.87 (27.22,58.69)	20681 (12673,29285[illegible]	17.48 (10.79,24.52)	-2.89 (-2.97,-2.82)
United Repub	40 (24,59)	0.39 (0.23,0.58)	85 (49,133)	0.35 (0.20,0.54)	-0.35 (-0.38,-0.33)	1104 (659,1648)	9.67 (5.75,14.35)	2374 (1346,3656)	8.58 (4.92,13.35)	-0.39 (-0.41,-0.37)
United States	3362 (2084,4706)	1.07 (0.66,1.49)	3115 (1853,4474)	0.55 (0.33,0.78)	-2.15 (-2.24,-2.07)	85789 (53665,1188[illegible]	28.62 (17.96,39.61)	81046 (49062,11263[illegible]	15.55 (9.5,21.59)	-1.97 (-2.05,-1.89)
United States	0 (0,1)	0.53 (0.31,0.81)	1 (0,1)	0.32 (0.17,0.50)	-1.75 (-1.93,-1.62)	13 (7,19)	13.58 (7.96,20.28)	14 (8,22)	8.26 (4.64,12.81)	-1.72 (-1.89,-1.60)
Uruguay	50 (30,70)	1.28 (0.78,1.8)	68 (40,100)	1.26 (0.75,1.84)	0.01 (-0.13,0.14)	1319 (813,1840)	35.13 (21.74,48.88)	1638 (977,2356)	33.25 (19.91,47.18)	-0.09 (-0.24,0.05)
Uzbekistan	16 (9,23)	0.13 (0.08,0.19)	41 (24,60)	0.14 (0.08,0.21)	0.23 (0.10,0.34)	517 (309,730)	4.18 (2.48,5.96)	1327 (790,1942)	4.23 (2.49,6.19)	0.08 (-0.04,0.21)
Vanuatu	0 (0,0)	0.41 (0.22,0.61)	1 (0,1)	0.30 (0.17,0.46)	-0.99 (-1.02,-0.95)	7 (4,12)	10.60 (5.62,16.27)	15 (9,23)	7.90 (4.45,12.06)	-0.99 (-1.03,-0.95)
Venezuela (B	35 (22,48)	0.37 (0.23,0.50)	92 (52,140)	0.30 (0.17,0.47)	-0.47 (-0.60,-0.32)	996 (619,1383)	9.62 (5.97,13.28)	2492 (1395,3766)	7.97 (4.47,12.06)	-0.49 (-0.64,-0.31)
Viet Nam	221 (130,320)	0.55 (0.33,0.80)	769 (472,1095)	0.76 (0.47,1.07)	1.05 (1.02,1.08)	5967 (3443,8704)	14.41 (8.39,21.09)	21943 (13292,32031[illegible]	20.21 (12.33,29.25)	1.11 (1.09,1.14)
Yemen	14 (7,22)	0.27 (0.14,0.43)	38 (20,60)	0.27 (0.14,0.42)	0.02 (-0.03,0.05)	435 (219,707)	7.60 (3.86,12.39)	1170 (607,1849)	7.10 (3.68,11.13)	-0.21 (-0.28,-0.16)
Zambia	7 (4,11)	0.29 (0.17,0.42)	21 (10,45)	0.34 (0.17,0.67)	0.57 (0.52,0.61)	197 (117,292)	6.80 (4.06,10.18)	600 (285,1339)	8.15 (4,17.35)	0.57 (0.53,0.60)
Zimbabwe	17 (10,25)	0.46 (0.27,0.67)	32 (20,48)	0.49 (0.30,0.71)	0.26 (0.23,0.30)	454 (273,673)	10.81 (6.44,15.85)	940 (558,1449)	12.32 (7.49,18.51)	0.45 (0.40,0.49)

Supplement table.3 Disease burden prediction for esophageal cancer death and disability-adjusted life years numbers and age-standardized rate to 2036 in different countries.

Country	Year	Death		DALY	
		(N)	ASR(per100,000)	(N)	ASR(per100,000)
Afghanistan	2022	97 (96–99)	0 (0–0)	2922 (2880–2964)	25 (25–26)
Afghanistan	2023	96 (93–100)	0 (0–0)	2940 (2851–3028)	25 (23–26)
Afghanistan	2024	96 (90–101)	0 (0–0)	2955 (2814–3095)	24 (22–26)
Afghanistan	2025	95 (87–103)	0 (0–0)	2968 (2772–3163)	23 (21–26)
Afghanistan	2026	94 (83–105)	0 (0–0)	2979 (2726–3232)	23 (19–27)
Afghanistan	2027	93 (79–107)	0 (0–0)	2988 (2678–3299)	22 (18–27)
Afghanistan	2028	92 (75–109)	0 (0–0)	2997 (2629–3365)	22 (16–28)
Afghanistan	2029	91 (70–112)	0 (0–0)	3004 (2579–3429)	21 (14–28)
Afghanistan	2030	90 (66–115)	0 (0–0)	3010 (2529–3492)	21 (13–29)
Afghanistan	2031	89 (61–118)	0 (0–0)	3016 (2479–3552)	20 (11–30)
Afghanistan	2032	89 (56–122)	0 (0–0)	3020 (2430–3611)	20 (9–31)
Afghanistan	2033	88 (50–125)	0 (0–0)	3025 (2381–3668)	19 (7–32)
Afghanistan	2034	87 (45–129)	0 (0–0)	3028 (2333–3723)	19 (5–33)
Afghanistan	2035	86 (39–133)	0 (0–0)	3031 (2285–3777)	18 (3–34)
Afghanistan	2036	85 (33–137)	0 (0–0)	3034 (2239–3828)	18 (1–35)
Albania	2022	35 (33–36)	1 (1–1)	777 (738–815)	18 (17–19)
Albania	2023	35 (32–38)	1 (1–1)	776 (706–846)	18 (16–20)
Albania	2024	36 (32–39)	1 (1–1)	776 (677–875)	18 (15–21)
Albania	2025	36 (31–41)	1 (1–1)	776 (652–900)	18 (15–22)
Albania	2026	36 (31–42)	1 (1–1)	776 (630–922)	18 (14–22)
Albania	2027	37 (31–43)	1 (1–1)	776 (610–942)	18 (14–22)
Albania	2028	37 (31–44)	1 (1–1)	776 (592–960)	18 (13–23)
Albania	2029	38 (31–45)	1 (1–1)	776 (575–976)	18 (13–23)
Albania	2030	38 (31–46)	1 (1–1)	776 (560–992)	18 (13–24)
Albania	2031	39 (31–47)	1 (1–1)	776 (545–1006)	18 (12–24)
Albania	2032	39 (31–48)	1 (1–1)	776 (532–1020)	18 (12–24)
Albania	2033	40 (31–49)	1 (1–2)	776 (519–1033)	18 (12–24)
Albania	2034	40 (31–50)	1 (1–2)	776 (507–1045)	18 (11–25)
Albania	2035	41 (31–51)	1 (1–2)	776 (495–1057)	18 (11–25)
Albania	2036	41 (31–52)	1 (1–2)	776 (484–1068)	18 (11–25)

Algeria	2022	90 (89–91)	0 (0–0)	2090 (2070–2110)	6 (6–6)
Algeria	2023	92 (90–94)	0 (0–0)	2144 (2099–2189)	6 (6–6)
Algeria	2024	94 (91–97)	0 (0–0)	2198 (2123–2273)	6 (6–6)
Algeria	2025	96 (91–100)	0 (0–0)	2253 (2143–2362)	6 (6–6)
Algeria	2026	98 (92–104)	0 (0–0)	2307 (2158–2455)	6 (6–6)
Algeria	2027	100 (92–108)	0 (0–0)	2361 (2170–2552)	6 (5–6)
Algeria	2028	102 (92–112)	0 (0–0)	2415 (2178–2652)	6 (5–6)
Algeria	2029	104 (92–116)	0 (0–0)	2470 (2184–2756)	6 (5–6)
Algeria	2030	106 (92–120)	0 (0–0)	2524 (2186–2862)	6 (5–7)
Algeria	2031	108 (92–124)	0 (0–0)	2578 (2185–2971)	6 (5–7)
Algeria	2032	110 (91–128)	0 (0–0)	2633 (2182–3083)	6 (5–7)
Algeria	2033	112 (91–133)	0 (0–0)	2687 (2176–3198)	6 (5–7)
Algeria	2034	114 (90–137)	0 (0–0)	2741 (2168–3314)	6 (5–7)
Algeria	2035	116 (89–142)	0 (0–0)	2795 (2157–3434)	6 (5–7)
Algeria	2036	117 (88–147)	0 (0–0)	2850 (2144–3555)	6 (5–7)
American Samoa	2022	0 (0–0)	1 (1–1)	6 (6–6)	11 (10–12)
American Samoa	2023	0 (0–0)	1 (1–1)	6 (5–6)	11 (10–12)
American Samoa	2024	0 (0–0)	1 (1–1)	6 (5–7)	11 (10–12)
American Samoa	2025	0 (0–0)	1 (1–1)	6 (6–7)	11 (9–13)
American Samoa	2026	0 (0–0)	1 (1–1)	6 (6–7)	11 (9–13)
American Samoa	2027	0 (0–0)	1 (1–1)	6 (6–7)	11 (9–13)
American Samoa	2028	0 (0–0)	1 (1–1)	6 (6–7)	11 (9–13)
American Samoa	2029	0 (0–0)	1 (1–1)	7 (6–8)	11 (9–13)
American Samoa	2030	0 (0–0)	1 (1–1)	7 (6–8)	11 (9–13)
American Samoa	2031	0 (0–0)	1 (1–1)	7 (6–8)	11 (9–14)
American Samoa	2032	0 (0–0)	1 (1–1)	7 (6–8)	11 (8–14)
American Samoa	2033	0 (0–0)	1 (1–1)	7 (6–8)	11 (8–14)
American Samoa	2034	0 (0–0)	1 (1–1)	7 (6–8)	11 (8–14)
American Samoa	2035	0 (0–0)	1 (1–1)	7 (6–8)	11 (8–14)
American Samoa	2036	0 (0–0)	1 (1–1)	7 (6–9)	11 (8–14)
Andorra	2022	1 (1–1)	2 (1–2)	17 (15–19)	11 (10–12)
Andorra	2023	1 (1–1)	2 (1–2)	17 (15–20)	10 (9–12)
Andorra	2024	1 (1–1)	1 (1–2)	17 (14–20)	10 (8–12)

Andorra	2025	1 (1–1)	1 (1–2)	17 (14–21)	10 (7–12)
Andorra	2026	1 (1–1)	1 (1–2)	17 (13–21)	9 (6–12)
Andorra	2027	1 (1–1)	1 (1–2)	17 (13–21)	9 (6–12)
Andorra	2028	1 (1–1)	1 (1–2)	17 (12–22)	8 (5–12)
Andorra	2029	1 (1–1)	1 (1–2)	17 (12–22)	8 (4–11)
Andorra	2030	1 (1–1)	1 (1–2)	17 (12–22)	8 (4–11)
Andorra	2031	1 (1–1)	1 (1–2)	17 (12–23)	7 (3–11)
Andorra	2032	1 (0–1)	1 (1–2)	17 (11–23)	7 (3–11)
Andorra	2033	1 (0–1)	1 (1–2)	17 (11–23)	6 (2–11)
Andorra	2034	1 (0–1)	1 (1–2)	17 (11–23)	6 (1–10)
Andorra	2035	1 (0–1)	1 (1–2)	17 (11–24)	5 (1–10)
Andorra	2036	1 (0–1)	1 (0–2)	17 (10–24)	5 (0–10)
Angola	2022	166 (163–169)	0 (0–0)	4942 (4846–5037)	35 (34–37)
Angola	2023	172 (166–177)	0 (0–0)	5120 (4941–5299)	36 (33–38)
Angola	2024	177 (169–186)	0 (0–0)	5298 (5026–5571)	36 (32–39)
Angola	2025	183 (171–196)	0 (0–0)	5477 (5099–5854)	36 (31–40)
Angola	2026	189 (173–205)	0 (0–0)	5655 (5163–6147)	36 (30–42)
Angola	2027	195 (175–215)	0 (0–0)	5833 (5217–6450)	36 (29–43)
Angola	2028	201 (176–225)	0 (0–0)	6012 (5262–6761)	36 (28–45)
Angola	2029	206 (177–236)	0 (0–0)	6190 (5299–7080)	36 (26–46)
Angola	2030	212 (178–247)	0 (0–0)	6368 (5329–7408)	36 (25–48)
Angola	2031	218 (178–258)	0 (0–0)	6547 (5351–7742)	36 (23–50)
Angola	2032	224 (179–269)	0 (0–0)	6725 (5366–8084)	37 (22–51)
Angola	2033	230 (179–281)	0 (0–0)	6903 (5374–8433)	37 (20–53)
Angola	2034	236 (179–292)	0 (0–0)	7082 (5376–8788)	37 (18–55)
Angola	2035	241 (178–304)	0 (0–0)	7260 (5371–9149)	37 (16–57)
Angola	2036	247 (178–316)	0 (0–0)	7438 (5360–9516)	37 (15–59)
Antigua and Barbuda	2022	1 (1–1)	0 (0–1)	14 (13–15)	13 (11–14)
Antigua and Barbuda	2023	1 (1–1)	0 (0–1)	14 (12–16)	13 (10–15)
Antigua and Barbuda	2024	1 (1–1)	0 (0–1)	14 (12–17)	13 (10–15)
Antigua and Barbuda	2025	1 (1–1)	0 (0–1)	14 (11–17)	13 (10–15)
Antigua and Barbuda	2026	1 (1–1)	0 (0–1)	14 (11–17)	13 (9–16)
Antigua and Barbuda	2027	1 (1–1)	0 (0–1)	14 (11–18)	13 (9–16)

Antigua and Barbuda	2028	1 (1–1)	0 (0–1)	14 (10–18)	13 (9–16)
Antigua and Barbuda	2029	1 (1–1)	0 (0–1)	14 (10–18)	13 (8–17)
Antigua and Barbuda	2030	1 (1–1)	0 (0–1)	14 (10–19)	13 (8–17)
Antigua and Barbuda	2031	1 (1–1)	0 (0–1)	14 (10–19)	13 (8–17)
Antigua and Barbuda	2032	1 (0–1)	0 (0–1)	14 (9–19)	13 (8–17)
Antigua and Barbuda	2033	1 (0–1)	0 (0–1)	14 (9–19)	13 (7–18)
Antigua and Barbuda	2034	1 (0–1)	0 (0–1)	14 (9–20)	13 (7–18)
Antigua and Barbuda	2035	1 (1–1)	0 (0–1)	14 (9–20)	13 (7–18)
Antigua and Barbuda	2036	1 (1–1)	0 (0–1)	14 (9–20)	13 (7–18)
Argentina	2022	633 (591–674)	1 (1–1)	14819 (13840–15798)	27 (24–29)
Argentina	2023	615 (540–689)	1 (1–1)	14413 (12664–16161)	25 (21–30)
Argentina	2024	597 (494–700)	1 (1–1)	14006 (11618–16393)	24 (19–30)
Argentina	2025	579 (449–709)	1 (1–1)	13599 (10608–16590)	23 (17–29)
Argentina	2026	561 (404–718)	1 (0–1)	13192 (9606–16779)	22 (15–29)
Argentina	2027	544 (359–728)	1 (0–1)	12786 (8601–16970)	21 (13–29)
Argentina	2028	526 (313–738)	1 (-0–1)	12379 (7589–17169)	20 (12–28)
Argentina	2029	508 (267–749)	0 (-0–1)	11972 (6565–17379)	19 (10–28)
Argentina	2030	490 (220–761)	0 (-1–1)	11565 (5529–17602)	18 (8–27)
Argentina	2031	472 (172–773)	0 (-1–1)	11158 (4478–17839)	17 (7–27)
Argentina	2032	455 (123–786)	0 (-1–1)	10752 (3413–18090)	16 (5–26)
Argentina	2033	437 (74–800)	0 (-1–1)	10345 (2333–18357)	14 (3–26)
Argentina	2034	419 (23–815)	-0 (-2–1)	9938 (1238–18638)	13 (2–25)
Argentina	2035	401 (-28–830)	-0 (-2–2)	9531 (129–18934)	12 (0–24)
Argentina	2036	383 (-80–846)	-0 (-2–2)	9125 (-996–19245)	11 (-1–24)
Armenia	2022	22 (20–25)	2 (2–2)	529 (464–594)	12 (10–13)
Armenia	2023	22 (18–27)	2 (2–2)	533 (432–634)	12 (8–15)
Armenia	2024	22 (17–28)	2 (2–2)	537 (402–672)	11 (7–16)
Armenia	2025	22 (16–29)	2 (2–2)	541 (372–710)	11 (6–17)
Armenia	2026	22 (15–30)	2 (2–2)	545 (341–749)	11 (4–18)
Armenia	2027	22 (14–31)	2 (2–2)	549 (308–790)	11 (3–20)
Armenia	2028	22 (13–31)	2 (2–2)	553 (275–831)	11 (1–21)
Armenia	2029	22 (13–32)	2 (2–2)	557 (240–874)	11 (-0–23)
Armenia	2030	22 (12–33)	2 (2–2)	561 (204–919)	11 (-2–25)

Armenia	2031	22 (12–33)	2 (2–2)	565 (166–964)	11 (-4–26)
Armenia	2032	22 (11–34)	2 (2–3)	569 (128–1011)	11 (-6–28)
Armenia	2033	22 (10–34)	2 (2–3)	573 (88–1059)	11 (-8–30)
Armenia	2034	22 (10–35)	2 (2–3)	577 (46–1108)	11 (-10–32)
Armenia	2035	22 (9–35)	2 (2–3)	581 (4–1159)	11 (-12–34)
Armenia	2036	22 (9–36)	2 (2–3)	585 (-40–1210)	11 (-14–36)
Australia	2022	393 (378–408)	1 (1–1)	8093 (7778–8407)	18 (17–19)
Australia	2023	395 (377–414)	1 (1–1)	8131 (7754–8508)	18 (17–19)
Australia	2024	398 (377–418)	1 (1–1)	8118 (7669–8567)	17 (16–18)
Australia	2025	400 (377–423)	1 (1–1)	8123 (7617–8628)	17 (15–18)
Australia	2026	402 (377–428)	1 (1–1)	8121 (7563–8679)	16 (14–17)
Australia	2027	405 (378–432)	1 (1–1)	8122 (7516–8727)	15 (14–17)
Australia	2028	407 (378–436)	1 (1–1)	8121 (7472–8771)	15 (13–16)
Australia	2029	409 (379–440)	1 (1–1)	8122 (7431–8812)	14 (12–16)
Australia	2030	412 (380–444)	1 (0–1)	8121 (7392–8851)	14 (12–15)
Australia	2031	414 (381–448)	1 (0–1)	8121 (7355–8888)	13 (11–15)
Australia	2032	416 (381–452)	1 (0–1)	8121 (7320–8923)	12 (10–14)
Australia	2033	419 (382–455)	1 (0–1)	8121 (7286–8957)	12 (10–14)
Australia	2034	421 (383–459)	1 (0–1)	8121 (7254–8989)	11 (9–13)
Australia	2035	423 (384–463)	1 (0–1)	8121 (7222–9021)	11 (8–13)
Australia	2036	426 (385–466)	1 (0–1)	8121 (7192–9051)	10 (8–12)
Austria	2022	176 (168–184)	1 (1–1)	3885 (3684–4087)	24 (22–25)
Austria	2023	177 (166–189)	1 (1–1)	3885 (3600–4170)	24 (21–26)
Austria	2024	179 (165–193)	1 (1–1)	3885 (3536–4234)	24 (21–26)
Austria	2025	181 (164–197)	1 (1–1)	3885 (3482–4288)	24 (21–27)
Austria	2026	182 (164–201)	1 (1–2)	3885 (3434–4336)	24 (20–27)
Austria	2027	184 (164–204)	1 (1–2)	3885 (3391–4379)	24 (20–27)
Austria	2028	185 (164–207)	1 (1–2)	3885 (3352–4418)	24 (20–27)
Austria	2029	187 (164–211)	1 (1–2)	3885 (3315–4455)	24 (19–28)
Austria	2030	189 (164–214)	1 (1–2)	3885 (3281–4490)	24 (19–28)
Austria	2031	190 (164–217)	1 (1–2)	3885 (3248–4522)	24 (19–28)
Austria	2032	192 (164–219)	1 (1–2)	3885 (3217–4553)	24 (19–28)
Austria	2033	193 (165–222)	1 (0–2)	3885 (3187–4583)	24 (18–29)

Austria	2034	195 (165–225)	1 (0–2)	3885 (3159–4612)	24 (18–29)
Austria	2035	197 (166–228)	1 (0–2)	3885 (3131–4639)	24 (18–29)
Austria	2036	198 (166–230)	1 (0–2)	3885 (3105–4666)	24 (18–29)
Azerbaijan	2022	223 (215–231)	1 (1–1)	5699 (5492–5907)	52 (49–55)
Azerbaijan	2023	225 (210–239)	1 (1–1)	5706 (5347–6065)	51 (46–57)
Azerbaijan	2024	227 (207–246)	1 (1–1)	5704 (5174–6234)	51 (43–59)
Azerbaijan	2025	229 (205–253)	1 (1–1)	5705 (5006–6405)	51 (41–61)
Azerbaijan	2026	231 (203–259)	1 (0–1)	5705 (4839–6572)	50 (39–62)
Azerbaijan	2027	234 (202–265)	1 (0–1)	5706 (4676–6735)	50 (37–64)
Azerbaijan	2028	236 (201–271)	1 (0–1)	5706 (4520–6892)	50 (35–66)
Azerbaijan	2029	238 (201–276)	1 (0–1)	5706 (4369–7043)	50 (33–67)
Azerbaijan	2030	241 (200–281)	1 (0–1)	5706 (4225–7187)	50 (32–69)
Azerbaijan	2031	243 (200–286)	1 (0–1)	5706 (4087–7325)	50 (31–70)
Azerbaijan	2032	245 (200–291)	1 (0–1)	5706 (3955–7458)	50 (29–71)
Azerbaijan	2033	248 (200–295)	1 (0–1)	5706 (3828–7585)	50 (28–72)
Azerbaijan	2034	250 (200–300)	1 (0–1)	5706 (3706–7707)	50 (27–73)
Azerbaijan	2035	253 (201–304)	1 (0–1)	5706 (3590–7823)	50 (26–74)
Azerbaijan	2036	255 (201–309)	1 (0–1)	5706 (3477–7936)	50 (25–75)
Bahamas	2022	5 (4–5)	0 (0–0)	123 (118–128)	28 (25–30)
Bahamas	2023	5 (4–5)	0 (0–0)	125 (116–133)	27 (24–31)
Bahamas	2024	5 (4–5)	0 (0–0)	127 (113–140)	27 (22–32)
Bahamas	2025	5 (4–6)	0 (0–0)	128 (109–147)	27 (20–33)
Bahamas	2026	5 (4–6)	0 (0–0)	130 (105–155)	27 (19–35)
Bahamas	2027	5 (4–6)	0 (0–0)	131 (100–163)	26 (17–36)
Bahamas	2028	5 (3–6)	0 (0–0)	133 (94–171)	26 (15–38)
Bahamas	2029	5 (3–7)	0 (0–0)	135 (89–181)	26 (13–39)
Bahamas	2030	5 (3–7)	0 (0–0)	136 (82–190)	26 (11–41)
Bahamas	2031	5 (3–8)	0 (0–0)	138 (75–200)	25 (8–42)
Bahamas	2032	5 (2–8)	0 (0–0)	139 (68–211)	25 (6–44)
Bahamas	2033	5 (2–8)	0 (0–0)	141 (61–221)	25 (4–46)
Bahamas	2034	5 (2–9)	0 (0–0)	143 (53–233)	25 (1–48)
Bahamas	2035	5 (1–9)	0 (0–0)	144 (44–244)	25 (-1–50)
Bahamas	2036	5 (1–10)	0 (0–0)	146 (36–256)	24 (-4–52)

Bahrain	2022	6 (6–6)	1 (1–1)	152 (149–155)	17 (16–19)
Bahrain	2023	6 (6–7)	1 (1–1)	164 (157–171)	18 (15–20)
Bahrain	2024	7 (6–7)	1 (1–1)	176 (164–188)	18 (14–22)
Bahrain	2025	7 (7–8)	1 (1–1)	189 (171–206)	18 (13–23)
Bahrain	2026	8 (7–9)	1 (1–1)	201 (177–224)	18 (11–25)
Bahrain	2027	8 (7–10)	1 (1–1)	213 (182–243)	18 (10–26)
Bahrain	2028	9 (7–11)	1 (1–1)	225 (187–263)	18 (9–27)
Bahrain	2029	9 (7–11)	1 (1–1)	237 (192–283)	18 (8–29)
Bahrain	2030	10 (7–12)	1 (1–1)	249 (196–303)	18 (7–30)
Bahrain	2031	10 (7–13)	1 (1–1)	261 (199–324)	18 (6–31)
Bahrain	2032	11 (7–14)	1 (1–1)	274 (202–345)	18 (5–32)
Bahrain	2033	11 (8–15)	1 (1–1)	286 (205–367)	18 (4–33)
Bahrain	2034	12 (8–16)	1 (1–1)	298 (207–389)	18 (3–34)
Bahrain	2035	12 (8–17)	1 (1–1)	310 (209–412)	18 (2–35)
Bahrain	2036	13 (8–18)	1 (1–1)	322 (210–434)	18 (1–35)
Bangladesh	2022	2341 (2274–2408)	0 (0–0)	59373 (57933–60812)	40 (38–42)
Bangladesh	2023	2398 (2248–2547)	0 (0–0)	60627 (57409–63846)	39 (35–43)
Bangladesh	2024	2454 (2204–2705)	0 (0–0)	61882 (56497–67267)	38 (33–43)
Bangladesh	2025	2511 (2144–2878)	0 (0–0)	63137 (55253–71020)	37 (30–44)
Bangladesh	2026	2568 (2071–3064)	0 (0–0)	64391 (53717–75066)	36 (28–43)
Bangladesh	2027	2624 (1985–3263)	0 (0–0)	65646 (51916–79376)	35 (26–43)
Bangladesh	2028	2681 (1888–3474)	0 (0–0)	66901 (49870–83931)	33 (24–43)
Bangladesh	2029	2737 (1781–3694)	0 (0–0)	68155 (47598–88713)	32 (22–43)
Bangladesh	2030	2794 (1663–3925)	0 (0–0)	69410 (45111–93708)	31 (20–42)
Bangladesh	2031	2851 (1536–4165)	0 (0–0)	70664 (42423–98906)	30 (18–42)
Bangladesh	2032	2907 (1400–4415)	0 (0–0)	71919 (39542–104296)	29 (16–41)
Bangladesh	2033	2964 (1256–4672)	0 (0–0)	73174 (36478–109869)	28 (14–41)
Bangladesh	2034	3021 (1103–4938)	0 (0–0)	74428 (33238–115619)	26 (13–40)
Bangladesh	2035	3077 (943–5212)	0 (0–0)	75683 (29828–121538)	25 (11–40)
Bangladesh	2036	3134 (775–5493)	0 (0–0)	76938 (26254–127621)	24 (9–39)
Barbados	2022	4 (4–5)	0 (0–0)	93 (86–100)	18 (16–20)
Barbados	2023	4 (4–5)	0 (0–0)	93 (84–102)	17 (15–20)
Barbados	2024	4 (4–5)	0 (0–0)	93 (82–104)	17 (14–20)

Barbados	2025	4 (4–5)	0 (0–0)	93 (80–106)	17 (13–21)
Barbados	2026	4 (4–5)	0 (0–0)	93 (78–108)	16 (12–21)
Barbados	2027	4 (4–5)	0 (0–1)	93 (77–109)	16 (11–21)
Barbados	2028	4 (4–5)	0 (0–1)	93 (75–111)	16 (11–21)
Barbados	2029	4 (4–5)	0 (0–1)	93 (74–112)	15 (10–21)
Barbados	2030	4 (4–5)	0 (0–1)	93 (73–113)	15 (9–21)
Barbados	2031	4 (4–5)	0 (0–1)	93 (72–114)	15 (9–21)
Barbados	2032	4 (4–5)	0 (0–1)	93 (71–115)	14 (8–21)
Barbados	2033	4 (4–5)	0 (0–1)	93 (70–116)	14 (7–21)
Barbados	2034	4 (4–5)	0 (0–1)	93 (69–117)	14 (7–21)
Barbados	2035	4 (4–5)	0 (0–1)	93 (68–118)	13 (6–21)
Barbados	2036	4 (4–5)	0 (0–1)	93 (67–119)	13 (5–20)
Belarus	2022	221 (202–239)	1 (1–1)	5943 (5433–6452)	39 (35–43)
Belarus	2023	216 (195–238)	1 (1–1)	5804 (5232–6375)	39 (33–45)
Belarus	2024	213 (191–236)	1 (1–1)	5804 (5232–6375)	39 (32–46)
Belarus	2025	212 (189–234)	1 (1–1)	5804 (5232–6375)	39 (31–47)
Belarus	2026	211 (188–234)	1 (1–1)	5804 (5232–6375)	39 (30–48)
Belarus	2027	210 (188–233)	1 (1–1)	5804 (5232–6375)	39 (29–49)
Belarus	2028	210 (187–233)	1 (1–2)	5804 (5232–6375)	39 (28–50)
Belarus	2029	210 (187–233)	1 (1–2)	5804 (5232–6375)	39 (28–51)
Belarus	2030	210 (187–233)	1 (1–2)	5804 (5232–6375)	39 (27–51)
Belarus	2031	210 (187–233)	1 (1–2)	5804 (5232–6375)	39 (26–52)
Belarus	2032	210 (187–233)	1 (1–2)	5804 (5232–6375)	39 (26–53)
Belarus	2033	210 (187–233)	1 (1–2)	5804 (5232–6375)	39 (25–53)
Belarus	2034	210 (187–233)	1 (1–2)	5804 (5232–6375)	39 (25–54)
Belarus	2035	210 (187–233)	1 (1–2)	5804 (5232–6375)	39 (24–54)
Belarus	2036	210 (187–233)	1 (1–2)	5804 (5232–6375)	39 (23–55)
Belgium	2022	410 (390–430)	1 (1–1)	8562 (8051–9074)	39 (36–42)
Belgium	2023	413 (385–441)	1 (1–1)	8491 (7711–9270)	38 (34–43)
Belgium	2024	416 (381–450)	1 (1–1)	8419 (7395–9443)	37 (31–43)
Belgium	2025	419 (379–459)	1 (1–1)	8347 (7084–9611)	36 (28–44)
Belgium	2026	422 (377–466)	1 (1–1)	8276 (6772–9780)	35 (26–44)
Belgium	2027	425 (376–473)	1 (1–1)	8204 (6455–9953)	34 (23–45)

Belgium	2028	428 (375–480)	1 (1–1)	8132 (6133–10131)	33 (21–46)
Belgium	2029	431 (374–487)	1 (1–1)	8061 (5805–10316)	32 (18–46)
Belgium	2030	434 (374–493)	1 (1–1)	7989 (5470–10507)	31 (15–47)
Belgium	2031	437 (374–500)	1 (1–1)	7917 (5129–10705)	30 (12–48)
Belgium	2032	439 (373–506)	1 (1–1)	7846 (4781–10911)	29 (9–48)
Belgium	2033	442 (373–511)	1 (1–1)	7774 (4425–11122)	28 (6–49)
Belgium	2034	445 (374–517)	1 (1–1)	7702 (4063–11341)	27 (3–50)
Belgium	2035	448 (374–523)	1 (1–1)	7630 (3694–11567)	26 (0–51)
Belgium	2036	451 (374–528)	1 (1–1)	7559 (3318–11800)	25 (-3–52)
Belize	2022	1 (1–1)	0 (0–0)	35 (34–37)	11 (10–12)
Belize	2023	1 (1–1)	0 (0–1)	36 (33–39)	11 (10–13)
Belize	2024	1 (1–2)	0 (0–1)	37 (33–41)	12 (10–14)
Belize	2025	1 (1–2)	0 (0–1)	38 (33–42)	12 (10–15)
Belize	2026	1 (1–2)	0 (0–1)	39 (34–44)	13 (10–15)
Belize	2027	1 (1–2)	0 (0–1)	39 (34–45)	13 (10–16)
Belize	2028	1 (1–2)	0 (0–1)	40 (34–46)	13 (10–16)
Belize	2029	2 (1–2)	0 (0–1)	41 (34–47)	13 (10–16)
Belize	2030	2 (1–2)	0 (0–1)	42 (35–49)	13 (10–16)
Belize	2031	2 (1–2)	0 (0–1)	42 (35–50)	13 (10–15)
Belize	2032	2 (1–2)	0 (0–1)	43 (36–51)	12 (10–15)
Belize	2033	2 (1–2)	0 (0–1)	44 (36–52)	12 (9–15)
Belize	2034	2 (1–2)	0 (0–1)	45 (36–53)	12 (9–15)
Belize	2035	2 (1–2)	0 (0–1)	46 (37–54)	12 (9–15)
Belize	2036	2 (1–2)	0 (0–1)	46 (37–55)	12 (9–15)
Benin	2022	24 (23–24)	0 (0–0)	646 (636–656)	12 (11–12)
Benin	2023	25 (24–25)	0 (0–0)	667 (649–685)	11 (11–12)
Benin	2024	25 (24–26)	0 (0–0)	688 (662–714)	11 (11–12)
Benin	2025	26 (25–27)	0 (0–0)	710 (675–745)	11 (11–12)
Benin	2026	27 (25–29)	0 (0–0)	731 (686–776)	11 (10–13)
Benin	2027	28 (26–30)	0 (0–0)	753 (697–808)	11 (10–13)
Benin	2028	28 (26–31)	0 (0–0)	774 (707–841)	11 (10–13)
Benin	2029	29 (26–32)	0 (0–0)	795 (716–874)	11 (10–13)
Benin	2030	30 (26–33)	0 (0–0)	817 (725–908)	11 (10–13)

Benin	2031	31 (27–35)	0 (0–0)	838 (733–943)	11 (10–13)
Benin	2032	31 (27–36)	0 (0–0)	859 (741–978)	11 (9–13)
Benin	2033	32 (27–37)	0 (0–0)	881 (748–1014)	11 (9–14)
Benin	2034	33 (27–38)	0 (0–0)	902 (754–1050)	11 (9–14)
Benin	2035	34 (28–40)	0 (0–0)	924 (760–1087)	11 (9–14)
Benin	2036	34 (28–41)	0 (0–0)	945 (765–1125)	11 (9–14)
Bermuda	2022	1 (1–1)	1 (1–1)	27 (26–29)	21 (19–23)
Bermuda	2023	1 (1–1)	1 (1–1)	28 (25–30)	21 (18–24)
Bermuda	2024	1 (1–1)	1 (1–1)	28 (24–32)	21 (16–25)
Bermuda	2025	1 (1–2)	1 (1–1)	28 (24–33)	21 (15–26)
Bermuda	2026	1 (1–2)	1 (1–1)	29 (23–34)	20 (14–27)
Bermuda	2027	1 (1–2)	1 (1–1)	29 (22–36)	20 (12–29)
Bermuda	2028	1 (1–2)	1 (1–1)	29 (22–37)	20 (11–30)
Bermuda	2029	1 (1–2)	1 (1–1)	30 (21–38)	20 (9–31)
Bermuda	2030	1 (1–2)	1 (1–1)	30 (20–40)	20 (7–33)
Bermuda	2031	1 (1–2)	1 (1–1)	30 (19–41)	20 (6–34)
Bermuda	2032	1 (1–2)	1 (1–1)	31 (18–43)	20 (4–36)
Bermuda	2033	2 (1–2)	1 (1–1)	31 (17–45)	20 (2–37)
Bermuda	2034	2 (1–2)	1 (1–1)	31 (17–46)	20 (0–39)
Bermuda	2035	2 (1–2)	1 (1–1)	32 (16–48)	20 (-1–41)
Bermuda	2036	2 (1–2)	1 (1–1)	32 (15–50)	20 (-3–43)
Bhutan	2022	9 (9–9)	0 (0–0)	203 (201–205)	32 (32–33)
Bhutan	2023	9 (9–9)	0 (0–0)	207 (204–210)	32 (31–33)
Bhutan	2024	9 (9–9)	0 (0–0)	211 (206–216)	32 (31–34)
Bhutan	2025	9 (9–10)	0 (0–0)	216 (208–223)	32 (30–34)
Bhutan	2026	9 (9–10)	0 (0–0)	220 (210–230)	32 (29–35)
Bhutan	2027	10 (9–10)	0 (0–0)	224 (211–237)	32 (29–35)
Bhutan	2028	10 (9–10)	0 (0–0)	228 (212–244)	32 (28–36)
Bhutan	2029	10 (9–11)	0 (0–0)	232 (213–252)	32 (27–36)
Bhutan	2030	10 (10–11)	0 (0–0)	237 (214–259)	32 (26–37)
Bhutan	2031	10 (10–11)	0 (0–0)	241 (214–267)	31 (25–38)
Bhutan	2032	11 (10–11)	0 (0–0)	245 (215–275)	31 (24–38)
Bhutan	2033	11 (10–12)	0 (0–0)	249 (215–284)	31 (23–39)

Bhutan	2034	11 (10–12)	0 (0–0)	253 (215–292)	31 (22–40)
Bhutan	2035	11 (10–12)	0 (0–0)	258 (215–301)	31 (21–41)
Bhutan	2036	11 (10–13)	0 (0–0)	262 (214–309)	31 (20–41)
Bolivia (Plurinational State of)	2022	27 (27–28)	1 (1–1)	650 (641–659)	7 (7–7)
Bolivia (Plurinational State of)	2023	27 (26–28)	1 (0–1)	644 (623–664)	7 (7–7)
Bolivia (Plurinational State of)	2024	26 (24–28)	1 (0–1)	638 (604–672)	7 (6–7)
Bolivia (Plurinational State of)	2025	25 (23–28)	1 (0–1)	632 (582–682)	6 (6–7)
Bolivia (Plurinational State of)	2026	25 (22–28)	0 (0–1)	626 (559–694)	6 (5–7)
Bolivia (Plurinational State of)	2027	24 (20–28)	0 (0–1)	620 (533–707)	6 (5–7)
Bolivia (Plurinational State of)	2028	24 (19–29)	0 (0–1)	614 (507–722)	6 (4–8)
Bolivia (Plurinational State of)	2029	23 (17–29)	0 (0–1)	609 (478–739)	6 (4–8)
Bolivia (Plurinational State of)	2030	23 (15–30)	0 (0–1)	603 (449–756)	6 (3–8)
Bolivia (Plurinational State of)	2031	22 (14–31)	0 (0–1)	597 (418–776)	5 (3–8)
Bolivia (Plurinational State of)	2032	21 (12–31)	0 (0–1)	591 (386–796)	5 (2–8)
Bolivia (Plurinational State of)	2033	21 (10–32)	0 (0–1)	585 (353–817)	5 (1–9)
Bolivia (Plurinational State of)	2034	20 (8–33)	0 (0–1)	579 (319–840)	5 (1–9)
Bolivia (Plurinational State of)	2035	20 (6–34)	0 (0–1)	573 (283–863)	5 (0–9)
Bolivia (Plurinational State of)	2036	19 (4–34)	0 (0–1)	567 (247–888)	5 (-0–9)
Bosnia and Herzegovina	2022	66 (62–71)	2 (2–2)	1670 (1544–1795)	28 (26–30)
Bosnia and Herzegovina	2023	62 (54–70)	2 (1–2)	1670 (1492–1847)	28 (26–31)
Bosnia and Herzegovina	2024	59 (47–72)	2 (1–2)	1670 (1452–1887)	29 (26–32)
Bosnia and Herzegovina	2025	55 (38–73)	1 (1–2)	1670 (1419–1921)	29 (26–32)
Bosnia and Herzegovina	2026	52 (30–75)	1 (1–2)	1670 (1389–1951)	29 (26–32)
Bosnia and Herzegovina	2027	49 (20–78)	1 (1–2)	1670 (1362–1977)	29 (26–32)
Bosnia and Herzegovina	2028	46 (10–81)	1 (1–2)	1670 (1337–2002)	29 (26–33)
Bosnia and Herzegovina	2029	42 (-0–84)	1 (1–2)	1670 (1314–2025)	29 (26–33)
Bosnia and Herzegovina	2030	39 (-11–88)	1 (1–2)	1670 (1293–2047)	29 (26–33)
Bosnia and Herzegovina	2031	35 (-22–93)	1 (1–2)	1670 (1273–2067)	30 (26–33)
Bosnia and Herzegovina	2032	32 (-34–97)	1 (1–2)	1670 (1253–2086)	30 (26–33)
Bosnia and Herzegovina	2033	29 (-45–102)	1 (0–2)	1670 (1235–2105)	30 (26–33)
Bosnia and Herzegovina	2034	25 (-58–108)	1 (0–2)	1670 (1217–2123)	30 (26–33)
Bosnia and Herzegovina	2035	22 (-70–113)	1 (0–2)	1670 (1200–2140)	30 (26–33)
Bosnia and Herzegovina	2036	18 (-83–119)	1 (0–2)	1670 (1183–2156)	30 (26–33)

Botswana	2022	34 (33–35)	1 (1–1)	943 (918–969)	58 (56–61)
Botswana	2023	34 (32–36)	1 (1–1)	945 (884–1006)	57 (51–64)
Botswana	2024	34 (31–38)	1 (1–1)	944 (844–1044)	56 (44–69)
Botswana	2025	35 (31–39)	1 (1–1)	942 (804–1080)	55 (37–74)
Botswana	2026	35 (30–40)	1 (1–1)	940 (768–1113)	54 (28–80)
Botswana	2027	36 (30–41)	1 (1–1)	939 (736–1142)	53 (19–87)
Botswana	2028	36 (30–42)	1 (1–1)	938 (708–1168)	52 (9–95)
Botswana	2029	36 (30–43)	1 (1–1)	938 (684–1191)	51 (-1–104)
Botswana	2030	37 (30–44)	1 (1–1)	937 (663–1212)	50 (-12–113)
Botswana	2031	37 (30–45)	1 (1–1)	937 (644–1231)	49 (-24–123)
Botswana	2032	38 (30–46)	1 (1–1)	938 (626–1249)	48 (-36–133)
Botswana	2033	38 (30–47)	1 (1–1)	938 (609–1266)	47 (-49–144)
Botswana	2034	39 (30–48)	1 (1–1)	938 (593–1282)	46 (-63–155)
Botswana	2035	39 (30–48)	1 (1–1)	938 (578–1297)	45 (-76–167)
Botswana	2036	40 (30–49)	1 (1–1)	938 (563–1312)	44 (-91–179)
Brazil	2022	3513 (3437–3590)	1 (1–1)	86634 (84341–88926)	32 (31–34)
Brazil	2023	3534 (3425–3642)	1 (1–1)	86634 (83392–89876)	31 (28–33)
Brazil	2024	3554 (3421–3687)	1 (1–1)	86634 (82663–90604)	29 (26–32)
Brazil	2025	3574 (3421–3727)	1 (1–1)	86634 (82049–91218)	27 (24–30)
Brazil	2026	3594 (3423–3766)	1 (1–1)	86634 (81507–91760)	26 (22–29)
Brazil	2027	3614 (3426–3802)	1 (1–1)	86634 (81018–92249)	24 (21–28)
Brazil	2028	3634 (3432–3837)	1 (1–1)	86634 (80568–92699)	23 (19–27)
Brazil	2029	3655 (3438–3872)	1 (1–1)	86634 (80150–93118)	21 (17–25)
Brazil	2030	3675 (3445–3905)	1 (1–1)	86634 (79756–93511)	19 (15–24)
Brazil	2031	3695 (3452–3937)	1 (1–1)	86634 (79384–93883)	18 (13–23)
Brazil	2032	3715 (3461–3969)	1 (1–1)	86634 (79030–94237)	16 (11–21)
Brazil	2033	3735 (3470–4001)	1 (1–1)	86634 (78692–94575)	15 (10–20)
Brazil	2034	3755 (3479–4032)	1 (1–1)	86634 (78368–94899)	13 (8–19)
Brazil	2035	3776 (3489–4063)	1 (0–1)	86634 (78056–95211)	12 (6–17)
Brazil	2036	3796 (3499–4093)	1 (0–1)	86634 (77755–95512)	10 (4–16)
Brunei Darussalam	2022	3 (3–3)	1 (1–1)	69 (67–71)	18 (17–19)
Brunei Darussalam	2023	3 (3–3)	1 (1–1)	70 (66–75)	18 (15–20)
Brunei Darussalam	2024	3 (2–3)	1 (0–1)	72 (65–79)	17 (13–22)

Brunei Darussalam	2025	3 (2–3)	1 (0–1)	74 (64–83)	17 (11–23)
Brunei Darussalam	2026	3 (2–4)	1 (0–1)	75 (62–88)	17 (8–25)
Brunei Darussalam	2027	3 (2–4)	1 (0–1)	77 (59–94)	17 (6–27)
Brunei Darussalam	2028	3 (2–4)	1 (0–1)	78 (57–100)	16 (3–30)
Brunei Darussalam	2029	3 (2–4)	1 (0–1)	80 (54–105)	16 (-0–32)
Brunei Darussalam	2030	3 (2–5)	1 (0–1)	81 (50–112)	16 (-4–35)
Brunei Darussalam	2031	3 (2–5)	1 (-0–1)	83 (47–118)	15 (-7–38)
Brunei Darussalam	2032	3 (2–5)	1 (-0–1)	84 (43–125)	15 (-11–41)
Brunei Darussalam	2033	4 (1–6)	0 (-0–1)	86 (39–132)	15 (-14–44)
Brunei Darussalam	2034	4 (1–6)	0 (-0–1)	87 (35–139)	14 (-18–47)
Brunei Darussalam	2035	4 (1–6)	0 (-0–1)	89 (31–146)	14 (-22–50)
Brunei Darussalam	2036	4 (1–7)	0 (-1–1)	90 (26–154)	14 (-26–54)
Bulgaria	2022	135 (124–147)	2 (2–2)	3672 (3346–3998)	30 (27–32)
Bulgaria	2023	133 (118–148)	2 (2–2)	3586 (3111–4061)	29 (25–33)
Bulgaria	2024	136 (115–157)	2 (1–2)	3668 (2987–4349)	30 (24–35)
Bulgaria	2025	134 (111–157)	2 (1–2)	3589 (2822–4356)	29 (23–35)
Bulgaria	2026	137 (110–163)	2 (1–2)	3664 (2758–4570)	30 (23–37)
Bulgaria	2027	134 (107–162)	2 (1–2)	3593 (2619–4567)	29 (22–37)
Bulgaria	2028	136 (106–167)	2 (1–2)	3661 (2577–4746)	30 (21–38)
Bulgaria	2029	134 (103–166)	2 (1–2)	3596 (2452–4739)	29 (20–38)
Bulgaria	2030	136 (103–170)	2 (1–2)	3658 (2421–4896)	30 (20–39)
Bulgaria	2031	134 (99–169)	2 (1–2)	3598 (2308–4889)	29 (19–39)
Bulgaria	2032	136 (99–173)	2 (1–2)	3656 (2283–5028)	30 (19–40)
Bulgaria	2033	134 (96–172)	2 (1–2)	3601 (2179–5023)	29 (18–40)
Bulgaria	2034	136 (96–176)	2 (1–2)	3653 (2157–5149)	30 (18–41)
Bulgaria	2035	134 (94–175)	2 (1–2)	3603 (2061–5146)	29 (17–41)
Bulgaria	2036	136 (94–179)	2 (1–2)	3651 (2042–5260)	30 (17–42)
Burkina Faso	2022	59 (58–60)	0 (0–0)	1731 (1707–1756)	17 (16–17)
Burkina Faso	2023	60 (59–62)	0 (0–0)	1777 (1722–1832)	17 (16–17)
Burkina Faso	2024	62 (59–65)	0 (0–0)	1823 (1731–1916)	17 (16–18)
Burkina Faso	2025	63 (59–68)	0 (0–0)	1870 (1735–2005)	17 (15–18)
Burkina Faso	2026	65 (59–71)	0 (0–0)	1916 (1733–2098)	17 (14–19)
Burkina Faso	2027	66 (59–74)	0 (0–0)	1962 (1727–2197)	17 (14–19)

Burkina Faso	2028	68 (58–77)	0 (0–0)	2008 (1716–2300)	17 (13–20)
Burkina Faso	2029	69 (58–81)	0 (0–0)	2054 (1702–2406)	17 (13–21)
Burkina Faso	2030	71 (57–84)	0 (0–0)	2100 (1684–2516)	17 (12–22)
Burkina Faso	2031	72 (57–88)	0 (0–0)	2146 (1663–2630)	17 (11–22)
Burkina Faso	2032	74 (56–92)	0 (0–0)	2192 (1638–2747)	17 (10–23)
Burkina Faso	2033	75 (55–96)	0 (0–0)	2238 (1610–2867)	17 (9–24)
Burkina Faso	2034	77 (54–100)	0 (0–0)	2285 (1579–2990)	17 (9–25)
Burkina Faso	2035	78 (53–104)	0 (0–0)	2331 (1545–3116)	17 (8–26)
Burkina Faso	2036	80 (52–108)	0 (0–0)	2377 (1509–3245)	17 (7–27)
Burundi	2022	62 (61–64)	0 (0–0)	1889 (1849–1929)	33 (32–34)
Burundi	2023	64 (61–66)	0 (0–0)	1931 (1841–2022)	32 (30–35)
Burundi	2024	65 (61–70)	0 (0–0)	1974 (1823–2125)	32 (28–36)
Burundi	2025	67 (60–73)	0 (0–0)	2017 (1796–2237)	32 (26–37)
Burundi	2026	68 (59–77)	0 (0–0)	2059 (1760–2358)	31 (24–39)
Burundi	2027	69 (58–81)	0 (0–0)	2102 (1717–2486)	31 (21–41)
Burundi	2028	71 (56–85)	0 (0–0)	2144 (1667–2621)	31 (18–43)
Burundi	2029	72 (55–89)	0 (0–0)	2187 (1611–2763)	31 (15–46)
Burundi	2030	73 (53–94)	0 (0–0)	2229 (1548–2910)	30 (12–48)
Burundi	2031	75 (51–99)	0 (0–0)	2272 (1481–3063)	30 (9–51)
Burundi	2032	76 (49–104)	0 (0–0)	2314 (1407–3222)	30 (6–53)
Burundi	2033	78 (47–109)	0 (0–0)	2357 (1329–3385)	29 (3–56)
Burundi	2034	79 (44–114)	0 (0–0)	2399 (1245–3554)	29 (-1–59)
Burundi	2035	80 (42–119)	0 (0–0)	2442 (1157–3727)	29 (-5–62)
Burundi	2036	82 (39–125)	0 (0–0)	2485 (1064–3905)	28 (-9–65)
Cabo Verde	2022	9 (8–10)	0 (0–0)	252 (233–271)	52 (47–57)
Cabo Verde	2023	9 (8–10)	0 (0–0)	257 (230–285)	52 (45–59)
Cabo Verde	2024	9 (8–11)	0 (0–0)	263 (230–296)	52 (43–61)
Cabo Verde	2025	10 (8–11)	0 (0–0)	269 (230–307)	52 (42–62)
Cabo Verde	2026	10 (8–12)	0 (0–0)	274 (231–318)	52 (41–64)
Cabo Verde	2027	10 (8–12)	0 (0–0)	280 (233–327)	52 (40–65)
Cabo Verde	2028	10 (8–12)	0 (0–1)	286 (235–337)	52 (39–66)
Cabo Verde	2029	10 (8–13)	0 (0–1)	291 (237–346)	52 (38–67)
Cabo Verde	2030	11 (8–13)	0 (0–1)	297 (239–355)	52 (37–67)

Cabo Verde	2031	11 (8–13)	0 (0–1)	303 (242–364)	52 (36–68)
Cabo Verde	2032	11 (8–14)	0 (0–1)	308 (244–372)	52 (35–69)
Cabo Verde	2033	11 (8–14)	0 (0–1)	314 (247–381)	52 (35–70)
Cabo Verde	2034	11 (8–14)	0 (0–1)	320 (250–389)	52 (34–70)
Cabo Verde	2035	12 (8–15)	0 (0–1)	325 (253–397)	52 (33–71)
Cabo Verde	2036	12 (9–15)	0 (0–1)	331 (256–406)	52 (33–72)
Cambodia	2022	180 (179–181)	1 (1–1)	4627 (4596–4659)	35 (34–35)
Cambodia	2023	185 (182–187)	1 (1–1)	4752 (4682–4822)	35 (34–36)
Cambodia	2024	190 (185–194)	1 (1–1)	4877 (4760–4994)	35 (33–36)
Cambodia	2025	195 (189–201)	1 (1–1)	5002 (4831–5172)	34 (32–37)
Cambodia	2026	200 (191–208)	1 (1–1)	5126 (4895–5358)	34 (31–37)
Cambodia	2027	205 (194–215)	1 (1–1)	5251 (4953–5549)	34 (30–38)
Cambodia	2028	210 (196–223)	1 (1–1)	5376 (5007–5745)	34 (29–39)
Cambodia	2029	215 (199–231)	1 (1–1)	5501 (5055–5946)	34 (28–40)
Cambodia	2030	220 (201–238)	1 (1–1)	5625 (5098–6152)	34 (27–40)
Cambodia	2031	224 (203–246)	1 (1–1)	5750 (5138–6362)	34 (26–41)
Cambodia	2032	229 (204–255)	1 (1–1)	5875 (5173–6577)	34 (25–42)
Cambodia	2033	234 (206–263)	1 (1–1)	5999 (5204–6795)	34 (24–43)
Cambodia	2034	239 (207–271)	1 (1–1)	6124 (5231–7017)	34 (23–44)
Cambodia	2035	244 (209–280)	1 (1–1)	6249 (5255–7243)	34 (22–45)
Cambodia	2036	249 (210–289)	1 (1–1)	6374 (5275–7472)	34 (21–46)
Cameroon	2022	97 (97–98)	0 (0–0)	2897 (2876–2917)	20 (19–20)
Cameroon	2023	99 (98–101)	0 (0–0)	2973 (2927–3018)	20 (19–20)
Cameroon	2024	102 (99–104)	0 (0–0)	3049 (2973–3125)	19 (18–20)
Cameroon	2025	104 (100–107)	0 (0–0)	3125 (3014–3236)	19 (18–21)
Cameroon	2026	106 (101–111)	0 (0–0)	3201 (3050–3352)	19 (17–21)
Cameroon	2027	108 (102–114)	0 (0–0)	3277 (3083–3471)	19 (16–21)
Cameroon	2028	110 (102–118)	0 (0–0)	3353 (3113–3593)	19 (16–22)
Cameroon	2029	112 (103–122)	0 (0–0)	3429 (3139–3719)	19 (15–22)
Cameroon	2030	115 (103–126)	0 (0–0)	3505 (3162–3848)	18 (14–23)
Cameroon	2031	117 (104–130)	0 (0–0)	3581 (3183–3980)	18 (13–23)
Cameroon	2032	119 (104–134)	0 (0–0)	3657 (3200–4114)	18 (12–24)
Cameroon	2033	121 (104–138)	0 (0–0)	3733 (3215–4251)	18 (12–24)

Cameroon	2034	123 (104–142)	0 (0–0)	3809 (3228–4391)	18 (11–25)
Cameroon	2035	125 (104–147)	0 (0–0)	3885 (3238–4533)	18 (10–26)
Cameroon	2036	128 (104–151)	0 (0–0)	3962 (3246–4677)	18 (9–26)
Canada	2022	1100 (1044–1156)	1 (1–1)	22538 (21326–23751)	31 (29–34)
Canada	2023	1114 (1034–1193)	1 (1–1)	22765 (21050–24479)	31 (27–34)
Canada	2024	1127 (1030–1224)	1 (1–1)	22991 (20891–25090)	30 (26–34)
Canada	2025	1141 (1029–1253)	1 (1–1)	23217 (20793–25642)	30 (25–34)
Canada	2026	1154 (1029–1279)	1 (1–1)	23444 (20733–26154)	29 (24–35)
Canada	2027	1167 (1030–1305)	1 (1–1)	23670 (20701–26639)	29 (23–35)
Canada	2028	1181 (1033–1329)	1 (1–1)	23896 (20689–27103)	28 (22–34)
Canada	2029	1194 (1036–1353)	1 (1–1)	24122 (20694–27551)	28 (21–34)
Canada	2030	1208 (1040–1376)	1 (1–1)	24349 (20712–27985)	27 (20–34)
Canada	2031	1221 (1044–1398)	1 (1–1)	24575 (20742–28408)	27 (19–34)
Canada	2032	1234 (1049–1420)	1 (1–1)	24801 (20781–28822)	26 (18–34)
Canada	2033	1248 (1054–1442)	1 (1–1)	25028 (20829–29227)	26 (17–34)
Canada	2034	1261 (1059–1463)	1 (1–1)	25254 (20883–29624)	25 (17–34)
Canada	2035	1275 (1065–1484)	1 (1–1)	25480 (20945–30016)	25 (16–34)
Canada	2036	1288 (1071–1505)	1 (0–1)	25707 (21012–30401)	24 (15–33)
Central African Republic	2022	25 (25–26)	0 (0–0)	807 (794–821)	29 (28–30)
Central African Republic	2023	26 (25–26)	0 (0–0)	814 (787–840)	29 (27–30)
Central African Republic	2024	26 (24–27)	0 (0–0)	820 (779–861)	28 (26–30)
Central African Republic	2025	26 (23–28)	0 (0–0)	827 (769–885)	28 (25–31)
Central African Republic	2026	26 (23–29)	0 (0–0)	833 (757–910)	27 (23–31)
Central African Republic	2027	26 (22–30)	0 (0–0)	840 (743–937)	27 (22–31)
Central African Republic	2028	26 (21–31)	0 (0–0)	846 (728–965)	26 (20–32)
Central African Republic	2029	26 (20–32)	0 (0–0)	853 (711–994)	26 (19–33)
Central African Republic	2030	26 (19–33)	0 (0–0)	859 (694–1025)	25 (17–33)
Central African Republic	2031	26 (18–34)	0 (0–0)	866 (675–1057)	25 (15–34)
Central African Republic	2032	26 (17–35)	0 (0–0)	872 (654–1091)	24 (13–35)
Central African Republic	2033	26 (16–37)	0 (0–0)	879 (633–1125)	24 (11–36)
Central African Republic	2034	26 (15–38)	0 (0–0)	886 (610–1161)	23 (9–37)
Central African Republic	2035	26 (13–39)	0 (0–0)	892 (587–1197)	23 (7–38)
Central African Republic	2036	26 (12–41)	0 (0–0)	899 (562–1235)	22 (5–39)

Chad	2022	42 (41–42)	0 (0–0)	1151 (1141–1161)	18 (18–18)
Chad	2023	43 (42–44)	0 (0–0)	1190 (1168–1213)	18 (18–19)
Chad	2024	44 (42–45)	0 (0–0)	1229 (1191–1267)	18 (18–19)
Chad	2025	45 (42–47)	0 (0–0)	1268 (1213–1324)	19 (17–20)
Chad	2026	46 (43–49)	0 (0–0)	1307 (1232–1383)	19 (17–20)
Chad	2027	47 (43–51)	0 (0–0)	1346 (1249–1443)	19 (17–21)
Chad	2028	48 (43–53)	0 (0–0)	1385 (1265–1506)	19 (16–21)
Chad	2029	49 (43–56)	0 (0–0)	1425 (1279–1570)	19 (16–22)
Chad	2030	50 (43–58)	0 (0–0)	1464 (1292–1635)	19 (16–22)
Chad	2031	51 (42–60)	0 (0–0)	1503 (1303–1702)	19 (15–23)
Chad	2032	52 (42–63)	0 (0–0)	1542 (1313–1771)	19 (15–23)
Chad	2033	54 (42–65)	0 (0–0)	1581 (1321–1840)	19 (15–24)
Chad	2034	55 (42–68)	0 (0–0)	1620 (1329–1911)	19 (14–24)
Chad	2035	56 (41–70)	0 (0–0)	1659 (1335–1983)	19 (14–25)
Chad	2036	57 (41–73)	0 (0–0)	1698 (1340–2056)	19 (14–25)
Chile	2022	113 (108–119)	1 (1–1)	2681 (2541–2821)	10 (9–11)
Chile	2023	112 (104–119)	1 (1–1)	2658 (2456–2860)	9 (7–10)
Chile	2024	110 (99–121)	1 (1–1)	2635 (2349–2921)	8 (6–10)
Chile	2025	109 (93–124)	1 (1–1)	2612 (2226–2998)	7 (5–9)
Chile	2026	107 (87–127)	1 (1–1)	2589 (2091–3087)	6 (4–8)
Chile	2027	106 (80–132)	1 (1–1)	2566 (1945–3187)	5 (2–7)
Chile	2028	104 (72–136)	1 (1–1)	2543 (1788–3298)	4 (1–7)
Chile	2029	103 (64–141)	1 (1–1)	2520 (1623–3417)	3 (0–6)
Chile	2030	101 (55–147)	1 (1–1)	2497 (1450–3545)	2 (-1–5)
Chile	2031	99 (46–153)	1 (1–1)	2474 (1268–3680)	1 (-2–4)
Chile	2032	98 (36–159)	1 (1–1)	2451 (1079–3823)	0 (-3–3)
Chile	2033	96 (27–166)	1 (1–1)	2428 (883–3973)	-1 (-4–3)
Chile	2034	95 (16–173)	1 (1–1)	2405 (680–4130)	-2 (-5–2)
Chile	2035	93 (6–181)	1 (1–1)	2382 (471–4294)	-3 (-7–1)
Chile	2036	92 (-5–189)	1 (1–1)	2359 (255–4464)	-4 (-8–0)
China	2022	3350 (140784–14591 [illegible]	1 (1–1)	[illegible] 79463 (3221302–333762 [illegible]	145 (139–150)
China	2023	5195 (139594–15079 [illegible]	1 (1–1)	[illegible] 09811 (3182901–343672 [illegible]	141 (130–151)
China	2024	7040 (139546–15453 [illegible]	1 (1–1)	[illegible] 40160 (3170367–350995 [illegible]	136 (121–151)

China	2025	8885 (139888–15788	1 (1–1)	70508 (3166663–357435	132 (114–150)
China	2026	0729 (140447–16101	1 (1–1)	00857 (3167884–363383	129 (108–149)
China	2027	2574 (141150–16399	1 (1–1)	31205 (3172362–369004	125 (103–147)
China	2028	4419 (141958–16688	1 (1–1)	61554 (3179201–374390	122 (98–145)
China	2029	6264 (142845–16968	1 (1–1)	91902 (3187852–379595	118 (93–143)
China	2030	8108 (143796–17242	1 (1–1)	22251 (3197952–384655	114 (88–141)
China	2031	9953 (144799–17510	1 (1–1)	52599 (3209243–389595	111 (83–139)
China	2032	1798 (145847–17774	1 (1–1)	82948 (3221538–394435	107 (78–136)
China	2033	3642 (146933–18035	1 (1–1)	13297 (3234693–399190	103 (73–134)
China	2034	5487 (148052–18292	1 (1–1)	43645 (3248596–403869	100 (68–132)
China	2035	7332 (149200–18546	1 (1–1)	73994 (3263156–408483	96 (63–129)
China	2036	9177 (150374–18797	1 (1–1)	04342 (3278302–413038	92 (58–127)
Colombia	2022	113 (103–123)	0 (0–0)	2473 (2227–2720)	5 (4–5)
Colombia	2023	113 (99–127)	0 (0–0)	2435 (2086–2784)	5 (4–6)
Colombia	2024	113 (95–130)	0 (0–0)	2397 (1970–2824)	5 (4–6)
Colombia	2025	113 (93–132)	0 (0–0)	2359 (1866–2852)	5 (3–7)
Colombia	2026	113 (90–135)	0 (0–0)	2321 (1769–2872)	5 (3–7)
Colombia	2027	113 (88–137)	0 (0–0)	2282 (1678–2886)	5 (3–8)
Colombia	2028	113 (86–139)	0 (0–0)	2244 (1592–2896)	6 (2–9)
Colombia	2029	113 (84–141)	0 (0–0)	2206 (1509–2903)	6 (2–9)
Colombia	2030	113 (83–142)	0 (0–0)	2168 (1428–2907)	6 (2–10)
Colombia	2031	113 (81–144)	0 (0–0)	2130 (1350–2909)	6 (1–11)
Colombia	2032	113 (80–146)	0 (0–0)	2091 (1274–2909)	6 (0–12)
Colombia	2033	113 (78–147)	0 (0–0)	2053 (1199–2907)	6 (-0–13)
Colombia	2034	113 (77–148)	0 (0–0)	2015 (1126–2904)	6 (-1–14)
Colombia	2035	113 (75–150)	0 (0–1)	1977 (1054–2899)	7 (-1–15)
Colombia	2036	113 (74–151)	0 (0–1)	1939 (984–2893)	7 (-2–16)
Comoros	2022	13 (13–14)	0 (0–0)	366 (352–380)	68 (64–72)
Comoros	2023	14 (13–14)	0 (0–0)	370 (350–390)	67 (61–72)
Comoros	2024	14 (13–14)	0 (0–0)	374 (349–398)	65 (59–72)
Comoros	2025	14 (13–15)	0 (0–0)	378 (350–406)	64 (56–72)
Comoros	2026	14 (13–15)	0 (0–0)	382 (350–414)	63 (54–71)
Comoros	2027	14 (13–15)	0 (0–0)	386 (351–421)	61 (52–71)

Comoros	2028	14 (13–15)	0 (0–0)	390 (353–428)	60 (50–70)
Comoros	2029	14 (13–16)	0 (0–0)	394 (354–434)	59 (48–69)
Comoros	2030	15 (13–16)	0 (0–0)	398 (356–441)	57 (46–69)
Comoros	2031	15 (13–16)	0 (0–0)	402 (358–447)	56 (44–68)
Comoros	2032	15 (14–16)	0 (0–0)	406 (359–454)	55 (42–67)
Comoros	2033	15 (14–17)	0 (0–0)	411 (361–460)	53 (40–67)
Comoros	2034	15 (14–17)	0 (0–0)	415 (364–466)	52 (38–66)
Comoros	2035	15 (14–17)	0 (0–0)	419 (366–472)	51 (37–65)
Comoros	2036	16 (14–17)	0 (0–0)	423 (368–478)	50 (35–64)
Congo	2022	40 (39–40)	0 (0–0)	1156 (1136–1176)	37 (36–38)
Congo	2023	41 (40–42)	0 (0–0)	1194 (1155–1234)	37 (35–39)
Congo	2024	42 (40–44)	0 (0–0)	1233 (1170–1297)	37 (33–41)
Congo	2025	43 (40–46)	0 (0–0)	1272 (1181–1363)	37 (32–42)
Congo	2026	44 (40–49)	0 (0–0)	1311 (1189–1433)	37 (30–44)
Congo	2027	45 (40–51)	0 (0–0)	1350 (1195–1505)	37 (28–46)
Congo	2028	46 (39–54)	0 (0–0)	1389 (1198–1580)	37 (26–48)
Congo	2029	47 (39–56)	0 (0–0)	1428 (1198–1657)	37 (24–50)
Congo	2030	49 (38–59)	0 (0–0)	1466 (1197–1736)	37 (21–52)
Congo	2031	50 (38–62)	0 (0–0)	1505 (1193–1818)	37 (19–54)
Congo	2032	51 (37–64)	0 (0–0)	1544 (1187–1902)	36 (16–57)
Congo	2033	52 (36–67)	0 (0–0)	1583 (1179–1987)	36 (13–60)
Congo	2034	53 (36–70)	0 (0–0)	1622 (1169–2075)	36 (10–62)
Congo	2035	54 (35–73)	0 (0–0)	1661 (1158–2164)	36 (7–65)
Congo	2036	55 (34–76)	0 (0–0)	1700 (1144–2255)	36 (4–68)
Cook Islands	2022	0 (0–0)	1 (1–1)	4 (4–4)	15 (14–16)
Cook Islands	2023	0 (0–0)	1 (1–1)	4 (4–4)	15 (14–16)
Cook Islands	2024	0 (0–0)	1 (1–1)	4 (4–4)	15 (13–16)
Cook Islands	2025	0 (0–0)	1 (0–1)	4 (4–4)	14 (13–16)
Cook Islands	2026	0 (0–0)	1 (0–1)	4 (4–4)	14 (13–16)
Cook Islands	2027	0 (0–0)	1 (0–1)	4 (4–4)	14 (12–16)
Cook Islands	2028	0 (0–0)	1 (0–1)	4 (4–5)	14 (12–16)
Cook Islands	2029	0 (0–0)	1 (0–1)	4 (4–5)	14 (12–15)
Cook Islands	2030	0 (0–0)	1 (0–1)	4 (4–5)	13 (11–15)

Cook Islands	2031	0 (0–0)	1 (0–1)	4 (4–5)	13 (11–15)
Cook Islands	2032	0 (0–0)	1 (0–1)	4 (4–5)	13 (11–15)
Cook Islands	2033	0 (0–0)	1 (0–1)	4 (4–5)	13 (11–15)
Cook Islands	2034	0 (0–0)	1 (0–1)	4 (4–5)	13 (10–15)
Cook Islands	2035	0 (0–0)	1 (0–1)	4 (4–5)	12 (10–15)
Cook Islands	2036	0 (0–0)	1 (0–1)	4 (4–5)	12 (10–15)
Costa Rica	2022	19 (18–21)	1 (0–1)	401 (366–436)	7 (6–8)
Costa Rica	2023	19 (18–21)	1 (0–1)	405 (365–445)	6 (5–8)
Costa Rica	2024	20 (17–22)	1 (0–1)	403 (355–451)	6 (5–7)
Costa Rica	2025	20 (17–22)	1 (0–1)	404 (351–457)	6 (4–7)
Costa Rica	2026	20 (17–23)	1 (0–1)	404 (345–462)	5 (4–7)
Costa Rica	2027	20 (17–23)	1 (0–1)	404 (341–467)	5 (3–7)
Costa Rica	2028	20 (17–23)	1 (0–1)	404 (336–472)	5 (3–6)
Costa Rica	2029	20 (17–24)	1 (0–1)	404 (332–476)	4 (2–6)
Costa Rica	2030	21 (17–24)	1 (0–1)	404 (328–480)	4 (2–6)
Costa Rica	2031	21 (17–24)	1 (0–1)	404 (324–484)	4 (1–6)
Costa Rica	2032	21 (17–25)	1 (0–1)	404 (320–487)	3 (1–5)
Costa Rica	2033	21 (17–25)	1 (0–1)	404 (317–491)	3 (1–5)
Costa Rica	2034	21 (17–25)	1 (0–1)	404 (314–494)	2 (0–5)
Costa Rica	2035	21 (17–26)	1 (0–1)	404 (311–497)	2 (-0–5)
Costa Rica	2036	22 (17–26)	1 (0–1)	404 (307–500)	2 (-1–4)
Croatia	2022	19 (19–19)	0 (0–0)	554 (545–563)	4 (4–4)
Croatia	2023	19 (18–20)	0 (0–0)	562 (542–582)	4 (4–4)
Croatia	2024	19 (18–20)	0 (0–0)	570 (536–603)	4 (4–4)
Croatia	2025	20 (18–21)	0 (0–0)	578 (529–627)	4 (3–5)
Croatia	2026	20 (18–22)	0 (0–0)	586 (519–652)	4 (3–5)
Croatia	2027	20 (17–23)	0 (0–0)	594 (508–679)	4 (2–5)
Croatia	2028	20 (17–23)	0 (0–0)	601 (496–707)	4 (2–5)
Croatia	2029	20 (17–24)	0 (0–0)	609 (482–737)	3 (1–5)
Croatia	2030	21 (16–25)	0 (0–0)	617 (467–768)	3 (1–6)
Croatia	2031	21 (16–26)	0 (0–0)	625 (450–800)	3 (0–6)
Croatia	2032	21 (15–27)	0 (0–0)	633 (432–834)	3 (-0–6)
Croatia	2033	21 (15–28)	0 (0–0)	641 (413–868)	3 (-1–6)

Croatia	2034	22 (14–29)	0 (0–0)	649 (393–904)	3 (-1–7)
Croatia	2035	22 (13–31)	0 (0–0)	657 (372–941)	3 (-2–7)
Croatia	2036	22 (13–32)	0 (0–0)	664 (350–979)	3 (-2–8)
Cuba	2022	110 (100–120)	2 (1–2)	2548 (2268–2828)	31 (27–35)
Cuba	2023	110 (96–125)	2 (1–2)	2548 (2153–2944)	30 (24–36)
Cuba	2024	110 (92–128)	2 (1–2)	2548 (2064–3033)	29 (22–36)
Cuba	2025	110 (90–131)	2 (1–2)	2548 (1989–3108)	29 (21–36)
Cuba	2026	110 (87–133)	2 (1–2)	2548 (1922–3174)	28 (19–37)
Cuba	2027	110 (85–135)	2 (1–2)	2548 (1863–3234)	27 (17–37)
Cuba	2028	110 (83–137)	2 (1–2)	2548 (1808–3289)	26 (16–37)
Cuba	2029	110 (81–139)	2 (1–2)	2548 (1757–3340)	26 (14–37)
Cuba	2030	110 (79–141)	2 (1–2)	2548 (1709–3388)	25 (13–37)
Cuba	2031	110 (78–143)	2 (1–2)	2548 (1663–3434)	24 (11–37)
Cuba	2032	110 (76–144)	2 (1–2)	2548 (1620–3477)	23 (10–36)
Cuba	2033	110 (74–146)	2 (1–2)	2548 (1579–3518)	22 (9–36)
Cuba	2034	110 (73–147)	2 (1–2)	2548 (1539–3558)	22 (7–36)
Cuba	2035	110 (72–149)	2 (1–2)	2548 (1501–3596)	21 (6–36)
Cuba	2036	110 (70–150)	2 (1–2)	2548 (1464–3632)	20 (5–36)
Cyprus	2022	362 (347–376)	1 (1–1)	9200 (8820–9579)	46 (44–49)
Cyprus	2023	368 (347–388)	1 (1–1)	9366 (8830–9903)	46 (43–50)
Cyprus	2024	374 (349–399)	1 (1–1)	9533 (8876–10190)	46 (42–50)
Cyprus	2025	380 (351–409)	1 (1–1)	9700 (8941–10459)	46 (42–51)
Cyprus	2026	386 (354–419)	1 (1–1)	9866 (9018–10715)	46 (41–52)
Cyprus	2027	392 (357–428)	1 (1–1)	10033 (9103–10963)	46 (41–52)
Cyprus	2028	398 (360–437)	1 (0–1)	10200 (9196–11204)	46 (40–53)
Cyprus	2029	404 (363–446)	1 (0–1)	10366 (9293–11440)	46 (40–53)
Cyprus	2030	411 (367–454)	1 (0–1)	10533 (9395–11672)	46 (39–53)
Cyprus	2031	417 (371–463)	1 (0–1)	10700 (9500–11900)	46 (39–54)
Cyprus	2032	423 (375–471)	1 (0–1)	10866 (9608–12125)	46 (39–54)
Cyprus	2033	429 (379–479)	1 (0–1)	11033 (9718–12348)	46 (38–55)
Cyprus	2034	435 (383–487)	1 (0–1)	11200 (9831–12568)	46 (38–55)
Cyprus	2035	441 (387–496)	1 (0–1)	11366 (9946–12786)	46 (38–55)
Cyprus	2036	447 (391–504)	1 (0–1)	11533 (10063–13003)	46 (37–55)

Czechia	2022	13 (13–14)	1 (1–1)	280 (269–291)	13 (13–14)
Czechia	2023	13 (13–14)	1 (1–1)	283 (264–302)	13 (12–14)
Czechia	2024	14 (13–14)	1 (1–1)	287 (261–313)	13 (11–15)
Czechia	2025	14 (13–15)	1 (1–1)	291 (259–324)	13 (11–15)
Czechia	2026	14 (13–15)	1 (1–1)	296 (258–334)	13 (10–15)
Czechia	2027	14 (13–16)	1 (0–1)	301 (258–344)	13 (10–16)
Czechia	2028	15 (13–16)	1 (0–1)	306 (259–353)	13 (10–16)
Czechia	2029	15 (13–16)	1 (0–1)	311 (260–362)	13 (9–16)
Czechia	2030	15 (14–17)	1 (0–1)	316 (261–371)	13 (9–16)
Czechia	2031	15 (14–17)	0 (-0–1)	321 (262–379)	13 (9–17)
Czechia	2032	16 (14–17)	0 (-0–1)	326 (264–388)	13 (8–17)
Czechia	2033	16 (14–18)	0 (-0–1)	331 (266–396)	13 (8–17)
Czechia	2034	16 (14–18)	0 (-1–1)	336 (268–404)	13 (8–17)
Czechia	2035	16 (14–18)	0 (-1–1)	341 (270–412)	13 (8–18)
Czechia	2036	17 (15–18)	0 (-1–1)	346 (272–420)	13 (8–18)
C么te d'Ivoire	2022	263 (254–273)	2 (2–2)	6255 (5979–6531)	32 (30–34)
C么te d'Ivoire	2023	266 (252–279)	2 (2–2)	6301 (5911–6692)	32 (29–34)
C么te d'Ivoire	2024	268 (252–285)	2 (2–2)	6348 (5870–6826)	32 (28–35)
C么te d'Ivoire	2025	271 (252–290)	2 (2–2)	6394 (5842–6946)	32 (28–35)
C么te d'Ivoire	2026	273 (252–295)	2 (2–2)	6441 (5823–7058)	32 (28–36)
C么te d'Ivoire	2027	276 (252–300)	2 (2–2)	6487 (5811–7163)	32 (27–36)
C么te d'Ivoire	2028	278 (253–304)	2 (2–2)	6533 (5803–7263)	32 (27–37)
C么te d'Ivoire	2029	281 (254–308)	2 (2–2)	6580 (5799–7360)	32 (26–37)
C么te d'Ivoire	2030	284 (254–313)	2 (2–2)	6626 (5798–7454)	32 (26–37)
C么te d'Ivoire	2031	286 (255–317)	2 (2–2)	6672 (5800–7545)	32 (26–38)
C么te d'Ivoire	2032	289 (257–321)	2 (2–2)	6719 (5803–7634)	32 (26–38)
C么te d'Ivoire	2033	291 (258–325)	2 (1–2)	6765 (5809–7721)	32 (25–38)
C么te d'Ivoire	2034	294 (259–329)	2 (1–2)	6812 (5816–7807)	32 (25–38)
C么te d'Ivoire	2035	296 (260–332)	2 (1–2)	6858 (5825–7891)	32 (25–39)
C么te d'Ivoire	2036	299 (261–336)	2 (1–2)	6904 (5835–7973)	32 (24–39)
Democratic People's Republic of Korea	2022	1098 (1092–1104)	1 (1–1)	30763 (30591–30935)	86 (85–87)
Democratic People's Republic of Korea	2023	1113 (1099–1127)	1 (1–1)	31165 (30780–31549)	85 (84–87)
Democratic People's Republic of Korea	2024	1128 (1104–1152)	1 (1–1)	31567 (30923–32210)	84 (82–87)

Democratic People's Republic of Korea	2025	1143 (1109–1178)	1 (1–1)	31968 (31026–32911)	84 (80–87)
Democratic People's Republic of Korea	2026	1158 (1111–1205)	1 (1–1)	32370 (31094–33646)	83 (78–87)
Democratic People's Republic of Korea	2027	1173 (1113–1234)	1 (1–1)	32772 (31131–34413)	82 (76–88)
Democratic People's Republic of Korea	2028	1189 (1114–1263)	1 (1–1)	33174 (31138–35210)	81 (74–88)
Democratic People's Republic of Korea	2029	1204 (1113–1294)	1 (1–1)	33576 (31119–36033)	80 (72–89)
Democratic People's Republic of Korea	2030	1219 (1112–1326)	1 (0–1)	33978 (31073–36882)	80 (69–90)
Democratic People's Republic of Korea	2031	1234 (1110–1358)	1 (0–1)	34380 (31004–37755)	79 (67–91)
Democratic People's Republic of Korea	2032	1249 (1107–1391)	1 (0–1)	34781 (30912–38651)	78 (64–92)
Democratic People's Republic of Korea	2033	1264 (1103–1425)	1 (0–1)	35183 (30797–39569)	77 (62–93)
Democratic People's Republic of Korea	2034	1279 (1098–1460)	1 (0–1)	35585 (30662–40509)	76 (59–94)
Democratic People's Republic of Korea	2035	1294 (1093–1496)	1 (0–1)	35987 (30506–41468)	76 (56–95)
Democratic People's Republic of Korea	2036	1310 (1087–1532)	1 (0–1)	36389 (30331–42447)	75 (54–96)
Democratic Republic of the Congo	2022	273 (268–278)	0 (0–0)	8190 (8042–8337)	19 (18–19)
Democratic Republic of the Congo	2023	281 (272–289)	0 (0–0)	8422 (8164–8681)	19 (18–20)
Democratic Republic of the Congo	2024	288 (276–301)	0 (0–0)	8655 (8276–9033)	19 (17–20)
Democratic Republic of the Congo	2025	296 (279–313)	0 (0–0)	8887 (8378–9397)	19 (17–20)
Democratic Republic of the Congo	2026	304 (282–325)	0 (0–0)	9120 (8469–9771)	19 (16–21)
Democratic Republic of the Congo	2027	311 (285–338)	0 (0–0)	9353 (8550–10156)	19 (16–21)
Democratic Republic of the Congo	2028	319 (287–351)	0 (0–0)	9585 (8621–10550)	18 (15–22)
Democratic Republic of the Congo	2029	327 (289–365)	0 (0–0)	9818 (8683–10953)	18 (15–22)
Democratic Republic of the Congo	2030	335 (290–379)	0 (0–0)	10051 (8736–11365)	18 (14–23)
Democratic Republic of the Congo	2031	342 (292–393)	0 (0–0)	10283 (8781–11785)	18 (14–23)
Democratic Republic of the Congo	2032	350 (293–407)	0 (0–0)	10516 (8818–12214)	18 (13–24)
Democratic Republic of the Congo	2033	358 (294–422)	0 (0–0)	10749 (8847–12650)	18 (12–24)
Democratic Republic of the Congo	2034	365 (294–437)	0 (0–0)	10981 (8869–13094)	18 (12–25)
Democratic Republic of the Congo	2035	373 (295–452)	0 (0–0)	11214 (8883–13545)	18 (11–25)
Democratic Republic of the Congo	2036	381 (295–467)	0 (0–0)	11446 (8890–14003)	18 (10–26)
Denmark	2022	269 (257–280)	2 (2–2)	5301 (5067–5536)	45 (42–48)
Denmark	2023	262 (241–283)	2 (2–2)	5269 (4906–5633)	45 (40–49)
Denmark	2024	258 (234–282)	2 (1–2)	5253 (4863–5643)	44 (39–49)
Denmark	2025	255 (229–281)	2 (1–2)	5245 (4848–5641)	43 (37–49)
Denmark	2026	253 (226–279)	2 (1–2)	5240 (4842–5639)	43 (36–49)
Denmark	2027	251 (224–278)	2 (1–2)	5238 (4839–5637)	42 (35–49)

Denmark	2028	250 (223–277)	2 (1–2)	5237 (4838–5636)	41 (33–49)
Denmark	2029	249 (222–276)	2 (1–3)	5237 (4838–5636)	41 (32–49)
Denmark	2030	248 (221–276)	2 (1–3)	5236 (4837–5635)	40 (31–49)
Denmark	2031	248 (221–275)	2 (1–3)	5236 (4837–5635)	39 (30–49)
Denmark	2032	248 (220–275)	2 (1–3)	5236 (4837–5635)	39 (29–49)
Denmark	2033	247 (220–275)	2 (1–3)	5236 (4837–5635)	38 (27–48)
Denmark	2034	247 (220–275)	2 (1–3)	5236 (4837–5635)	37 (26–48)
Denmark	2035	247 (220–275)	2 (1–3)	5236 (4837–5635)	37 (25–48)
Denmark	2036	247 (220–275)	2 (1–3)	5236 (4837–5635)	36 (24–48)
Djibouti	2022	16 (16–16)	0 (0–0)	463 (457–469)	62 (61–64)
Djibouti	2023	17 (16–17)	0 (0–0)	476 (468–483)	61 (59–63)
Djibouti	2024	17 (17–17)	0 (0–0)	488 (478–498)	60 (57–63)
Djibouti	2025	17 (17–18)	0 (0–0)	500 (488–512)	59 (55–63)
Djibouti	2026	18 (17–18)	0 (0–0)	512 (497–527)	58 (53–63)
Djibouti	2027	18 (18–19)	0 (0–0)	525 (507–542)	57 (51–64)
Djibouti	2028	19 (18–20)	0 (0–0)	537 (516–558)	56 (49–64)
Djibouti	2029	19 (18–20)	0 (0–0)	549 (525–573)	55 (47–64)
Djibouti	2030	20 (19–21)	0 (0–0)	561 (534–589)	55 (44–65)
Djibouti	2031	20 (19–21)	0 (0–0)	574 (543–604)	54 (42–66)
Djibouti	2032	21 (19–22)	0 (0–0)	586 (552–620)	53 (39–66)
Djibouti	2033	21 (19–23)	0 (0–0)	598 (561–636)	52 (36–67)
Djibouti	2034	22 (20–23)	0 (0–0)	611 (569–652)	51 (34–68)
Djibouti	2035	22 (20–24)	0 (0–0)	623 (577–668)	50 (31–68)
Djibouti	2036	22 (20–25)	0 (0–0)	635 (586–685)	49 (28–69)
Dominica	2022	1 (1–1)	1 (1–1)	16 (16–16)	18 (17–18)
Dominica	2023	1 (1–1)	1 (1–1)	16 (15–16)	18 (17–18)
Dominica	2024	1 (1–1)	1 (1–1)	16 (15–17)	17 (17–18)
Dominica	2025	1 (1–1)	1 (1–1)	16 (15–17)	17 (16–18)
Dominica	2026	1 (1–1)	1 (1–1)	16 (15–18)	17 (16–19)
Dominica	2027	1 (1–1)	1 (1–1)	16 (15–18)	17 (16–19)
Dominica	2028	1 (1–1)	1 (1–1)	17 (14–19)	17 (15–19)
Dominica	2029	1 (1–1)	1 (1–1)	17 (14–19)	17 (15–19)
Dominica	2030	1 (1–1)	1 (1–1)	17 (14–20)	17 (15–19)

Dominica	2031	1 (1–1)	1 (1–1)	17 (13–21)	17 (15–19)
Dominica	2032	1 (1–1)	1 (1–1)	17 (13–21)	17 (15–19)
Dominica	2033	1 (1–1)	1 (1–1)	17 (13–22)	17 (15–20)
Dominica	2034	1 (0–1)	1 (1–1)	17 (12–23)	17 (15–20)
Dominica	2035	1 (0–1)	1 (1–1)	18 (12–23)	17 (15–20)
Dominica	2036	1 (0–1)	1 (1–1)	18 (11–24)	17 (15–20)
Dominican Republic	2022	71 (67–74)	1 (1–1)	1633 (1550–1717)	16 (15–17)
Dominican Republic	2023	72 (67–77)	1 (1–1)	1666 (1548–1784)	16 (14–17)
Dominican Republic	2024	74 (67–80)	1 (1–1)	1698 (1553–1842)	16 (14–18)
Dominican Republic	2025	75 (68–82)	1 (1–1)	1730 (1563–1897)	16 (14–18)
Dominican Republic	2026	76 (68–85)	1 (1–1)	1763 (1576–1949)	16 (14–18)
Dominican Republic	2027	78 (69–87)	1 (1–1)	1795 (1590–1999)	16 (13–19)
Dominican Republic	2028	79 (70–89)	1 (1–1)	1827 (1606–2048)	16 (13–19)
Dominican Republic	2029	81 (70–91)	1 (1–1)	1859 (1623–2095)	16 (13–19)
Dominican Republic	2030	82 (71–93)	1 (1–1)	1892 (1641–2142)	16 (13–19)
Dominican Republic	2031	84 (72–95)	1 (1–1)	1924 (1660–2188)	16 (13–19)
Dominican Republic	2032	85 (73–97)	1 (1–1)	1956 (1680–2233)	16 (12–19)
Dominican Republic	2033	86 (74–99)	1 (1–1)	1989 (1700–2278)	16 (12–20)
Dominican Republic	2034	88 (75–101)	1 (1–1)	2021 (1720–2322)	16 (12–20)
Dominican Republic	2035	89 (75–103)	1 (1–1)	2053 (1741–2365)	16 (12–20)
Dominican Republic	2036	91 (76–105)	1 (1–1)	2086 (1762–2409)	16 (12–20)
Ecuador	2022	28 (25–31)	0 (0–0)	583 (525–641)	3 (3–4)
Ecuador	2023	28 (24–32)	0 (0–0)	583 (501–666)	3 (2–4)
Ecuador	2024	28 (23–33)	0 (0–0)	583 (482–684)	3 (2–4)
Ecuador	2025	28 (22–33)	0 (0–0)	583 (467–700)	3 (1–4)
Ecuador	2026	28 (22–34)	0 (0–1)	583 (453–714)	2 (1–4)
Ecuador	2027	28 (21–34)	0 (0–1)	583 (440–726)	2 (0–4)
Ecuador	2028	28 (21–35)	0 (0–1)	583 (429–737)	2 (-0–4)
Ecuador	2029	28 (20–36)	0 (0–1)	583 (418–748)	2 (-0–4)
Ecuador	2030	28 (20–36)	0 (0–1)	583 (408–758)	2 (-1–4)
Ecuador	2031	28 (19–36)	0 (0–1)	583 (399–768)	1 (-1–4)
Ecuador	2032	28 (19–37)	0 (0–1)	583 (390–777)	1 (-2–4)
Ecuador	2033	28 (18–37)	0 (0–1)	583 (381–785)	1 (-2–4)

Ecuador	2034	28 (18–38)	0 (0–1)	583 (373–793)	1 (-2–4)
Ecuador	2035	28 (18–38)	0 (0–1)	583 (365–801)	1 (-3–4)
Ecuador	2036	28 (17–38)	0 (0–1)	583 (357–809)	0 (-3–3)
Egypt	2022	230 (223–237)	1 (1–1)	5985 (5782–6188)	9 (9–10)
Egypt	2023	234 (224–244)	1 (1–1)	6086 (5799–6373)	9 (9–10)
Egypt	2024	238 (226–250)	1 (1–1)	6187 (5836–6538)	9 (8–10)
Egypt	2025	242 (228–256)	1 (1–1)	6288 (5882–6693)	9 (8–10)
Egypt	2026	246 (230–262)	1 (1–1)	6389 (5936–6842)	9 (8–10)
Egypt	2027	250 (233–267)	1 (1–1)	6490 (5993–6986)	9 (8–10)
Egypt	2028	254 (236–273)	1 (1–1)	6591 (6054–7127)	9 (8–10)
Egypt	2029	258 (239–278)	1 (1–1)	6692 (6118–7265)	9 (8–10)
Egypt	2030	263 (242–284)	1 (1–1)	6793 (6185–7401)	9 (8–11)
Egypt	2031	267 (245–289)	1 (1–1)	6894 (6253–7535)	9 (8–11)
Egypt	2032	271 (248–294)	1 (1–1)	6995 (6322–7667)	9 (8–11)
Egypt	2033	275 (251–299)	1 (1–1)	7096 (6393–7798)	9 (8–11)
Egypt	2034	279 (254–304)	1 (1–1)	7197 (6466–7928)	9 (8–11)
Egypt	2035	283 (257–309)	1 (1–1)	7298 (6539–8056)	9 (7–11)
Egypt	2036	287 (260–314)	1 (1–1)	7399 (6613–8184)	9 (7–11)
El Salvador	2022	14 (13–15)	0 (0–0)	349 (322–376)	6 (5–6)
El Salvador	2023	14 (13–16)	0 (0–0)	354 (317–392)	6 (5–6)
El Salvador	2024	15 (13–17)	0 (0–0)	360 (314–407)	6 (5–6)
El Salvador	2025	15 (13–17)	0 (0–0)	366 (312–420)	6 (5–6)
El Salvador	2026	15 (13–18)	0 (0–0)	372 (312–432)	6 (5–6)
El Salvador	2027	15 (13–18)	0 (0–0)	378 (312–443)	6 (5–6)
El Salvador	2028	16 (13–18)	0 (0–0)	383 (312–454)	6 (5–6)
El Salvador	2029	16 (13–19)	0 (0–0)	389 (313–465)	6 (5–6)
El Salvador	2030	16 (13–19)	0 (0–0)	395 (314–475)	6 (5–6)
El Salvador	2031	16 (13–20)	0 (0–0)	401 (316–485)	6 (5–6)
El Salvador	2032	17 (13–20)	0 (0–0)	406 (317–495)	6 (5–6)
El Salvador	2033	17 (13–21)	0 (0–0)	412 (319–505)	6 (5–6)
El Salvador	2034	17 (13–21)	0 (0–0)	418 (321–515)	6 (5–6)
El Salvador	2035	17 (14–21)	0 (0–0)	424 (323–524)	6 (5–6)
El Salvador	2036	18 (14–22)	0 (0–0)	429 (325–533)	6 (5–6)

Equatorial Guinea	2022	6 (6–6)	0 (0–0)	170 (166–174)	29 (27–30)
Equatorial Guinea	2023	6 (6–6)	0 (0–0)	175 (166–185)	29 (26–31)
Equatorial Guinea	2024	6 (5–6)	0 (0–0)	180 (164–196)	28 (24–33)
Equatorial Guinea	2025	6 (5–7)	0 (0–0)	185 (162–209)	28 (21–35)
Equatorial Guinea	2026	6 (5–7)	0 (0–0)	191 (159–223)	28 (19–38)
Equatorial Guinea	2027	6 (5–8)	0 (0–0)	196 (155–237)	28 (16–40)
Equatorial Guinea	2028	6 (5–8)	0 (0–0)	201 (150–252)	28 (13–43)
Equatorial Guinea	2029	7 (5–9)	0 (0–0)	206 (144–267)	28 (9–46)
Equatorial Guinea	2030	7 (4–9)	0 (0–0)	211 (138–284)	27 (6–49)
Equatorial Guinea	2031	7 (4–10)	0 (0–0)	216 (131–301)	27 (2–52)
Equatorial Guinea	2032	7 (4–10)	0 (0–0)	221 (124–318)	27 (-2–56)
Equatorial Guinea	2033	7 (4–11)	0 (0–0)	226 (116–336)	27 (-6–59)
Equatorial Guinea	2034	7 (3–11)	0 (0–1)	231 (108–355)	27 (-10–63)
Equatorial Guinea	2035	7 (3–12)	0 (0–1)	236 (99–374)	27 (-14–67)
Equatorial Guinea	2036	8 (3–12)	0 (0–1)	242 (90–393)	26 (-19–71)
Eritrea	2022	33 (32–34)	0 (0–0)	1066 (1026–1106)	30 (28–31)
Eritrea	2023	33 (31–35)	0 (0–0)	1044 (963–1124)	29 (26–32)
Eritrea	2024	33 (30–36)	0 (0–0)	1017 (909–1124)	28 (24–33)
Eritrea	2025	33 (29–37)	0 (0–0)	999 (879–1119)	27 (22–33)
Eritrea	2026	33 (29–38)	0 (0–0)	993 (868–1117)	27 (20–34)
Eritrea	2027	34 (29–38)	0 (0–0)	992 (865–1119)	26 (18–34)
Eritrea	2028	34 (29–39)	0 (0–0)	990 (861–1119)	25 (16–34)
Eritrea	2029	34 (29–39)	0 (0–0)	985 (853–1117)	24 (14–34)
Eritrea	2030	34 (29–40)	0 (0–0)	978 (843–1113)	23 (13–34)
Eritrea	2031	35 (29–40)	0 (0–0)	972 (835–1109)	22 (11–34)
Eritrea	2032	35 (29–41)	0 (0–0)	967 (828–1106)	21 (10–33)
Eritrea	2033	35 (29–42)	0 (0–0)	964 (824–1103)	21 (8–33)
Eritrea	2034	35 (29–42)	0 (0–0)	961 (820–1102)	20 (7–33)
Eritrea	2035	36 (29–42)	0 (0–0)	958 (817–1100)	19 (5–33)
Eritrea	2036	36 (29–43)	0 (0–0)	956 (814–1098)	18 (4–32)
Estonia	2022	26 (23–29)	1 (1–2)	636 (560–712)	27 (24–31)
Estonia	2023	26 (22–30)	1 (1–2)	636 (528–743)	27 (22–32)
Estonia	2024	26 (21–31)	1 (1–2)	636 (504–767)	27 (21–33)

Estonia	2025	26 (21–31)	1 (1–2)	636 (484–788)	27 (20–34)
Estonia	2026	26 (20–32)	1 (1–2)	636 (466–806)	27 (19–35)
Estonia	2027	26 (19–33)	1 (1–2)	636 (450–822)	27 (18–36)
Estonia	2028	26 (19–33)	1 (1–2)	636 (435–837)	27 (18–37)
Estonia	2029	26 (18–34)	2 (1–2)	636 (421–851)	27 (17–37)
Estonia	2030	26 (18–34)	2 (1–2)	636 (408–864)	27 (16–38)
Estonia	2031	26 (18–35)	2 (1–2)	636 (395–876)	27 (16–38)
Estonia	2032	26 (17–35)	2 (1–2)	636 (384–888)	27 (15–39)
Estonia	2033	26 (17–35)	2 (1–2)	636 (373–899)	27 (15–39)
Estonia	2034	26 (16–36)	2 (1–2)	636 (362–910)	27 (14–40)
Estonia	2035	26 (16–36)	2 (1–2)	636 (351–920)	27 (14–40)
Estonia	2036	26 (16–37)	2 (1–2)	636 (341–930)	27 (13–41)
Eswatini	2022	9 (9–10)	0 (0–0)	275 (265–285)	44 (42–46)
Eswatini	2023	9 (8–10)	0 (0–0)	265 (240–291)	42 (36–47)
Eswatini	2024	8 (7–10)	0 (0–0)	255 (209–301)	39 (30–48)
Eswatini	2025	8 (5–10)	0 (0–0)	245 (174–316)	37 (23–51)
Eswatini	2026	7 (4–10)	0 (0–1)	235 (136–334)	35 (16–54)
Eswatini	2027	7 (2–11)	0 (0–1)	225 (95–355)	32 (8–57)
Eswatini	2028	6 (1–12)	0 (0–1)	215 (50–379)	30 (-1–61)
Eswatini	2029	6 (-1–12)	0 (0–1)	204 (4–405)	28 (-10–66)
Eswatini	2030	5 (-3–13)	0 (0–1)	194 (-45–434)	26 (-20–71)
Eswatini	2031	5 (-5–14)	0 (0–1)	184 (-97–465)	23 (-29–76)
Eswatini	2032	4 (-7–15)	0 (0–1)	174 (-150–498)	21 (-40–82)
Eswatini	2033	4 (-9–16)	0 (0–1)	164 (-206–534)	19 (-50–88)
Eswatini	2034	3 (-11–17)	0 (0–1)	154 (-263–571)	17 (-62–95)
Eswatini	2035	3 (-13–18)	0 (0–1)	143 (-323–610)	14 (-73–101)
Eswatini	2036	2 (-15–19)	0 (0–1)	133 (-384–651)	12 (-85–109)
Ethiopia	2022	111 (110–112)	0 (0–0)	3011 (2981–3041)	6 (6–7)
Ethiopia	2023	113 (111–116)	0 (0–0)	3090 (3023–3157)	6 (6–7)
Ethiopia	2024	115 (111–120)	0 (0–0)	3169 (3056–3281)	6 (6–7)
Ethiopia	2025	118 (111–124)	0 (0–0)	3247 (3083–3412)	6 (6–7)
Ethiopia	2026	120 (111–128)	0 (0–0)	3326 (3104–3549)	6 (6–7)
Ethiopia	2027	122 (111–133)	0 (0–0)	3405 (3119–3691)	6 (5–8)

Ethiopia	2028	124 (111–137)	0 (0–0)	3484 (3129–3839)	6 (5–8)
Ethiopia	2029	126 (110–142)	0 (0–0)	3563 (3134–3991)	6 (5–8)
Ethiopia	2030	128 (109–147)	0 (0–0)	3641 (3135–4148)	6 (4–8)
Ethiopia	2031	130 (108–152)	0 (0–0)	3720 (3132–4309)	6 (4–9)
Ethiopia	2032	132 (107–158)	0 (0–0)	3799 (3124–4474)	6 (4–9)
Ethiopia	2033	135 (106–163)	0 (0–0)	3878 (3113–4642)	6 (3–10)
Ethiopia	2034	137 (105–169)	0 (0–0)	3956 (3098–4815)	6 (3–10)
Ethiopia	2035	139 (103–175)	0 (0–0)	4035 (3080–4991)	6 (2–10)
Ethiopia	2036	141 (101–180)	0 (0–0)	4114 (3058–5170)	6 (2–11)
Fiji	2022	4 (4–4)	0 (0–0)	113 (108–117)	13 (12–14)
Fiji	2023	4 (4–4)	0 (0–0)	114 (108–121)	13 (12–14)
Fiji	2024	4 (4–4)	0 (0–0)	116 (108–124)	13 (12–14)
Fiji	2025	4 (4–4)	0 (0–0)	118 (109–126)	13 (12–14)
Fiji	2026	4 (4–5)	0 (0–0)	119 (109–129)	13 (11–15)
Fiji	2027	4 (4–5)	0 (0–0)	121 (110–132)	13 (11–15)
Fiji	2028	4 (4–5)	0 (0–0)	122 (110–134)	13 (11–15)
Fiji	2029	4 (4–5)	0 (0–0)	124 (111–136)	13 (11–15)
Fiji	2030	4 (4–5)	0 (0–0)	125 (112–139)	13 (11–15)
Fiji	2031	4 (4–5)	0 (0–0)	127 (113–141)	13 (11–15)
Fiji	2032	5 (4–5)	0 (0–0)	128 (114–143)	13 (11–15)
Fiji	2033	5 (4–5)	0 (0–0)	130 (115–145)	13 (10–16)
Fiji	2034	5 (4–5)	0 (0–0)	132 (116–148)	13 (10–16)
Fiji	2035	5 (4–5)	0 (0–0)	133 (117–150)	13 (10–16)
Fiji	2036	5 (4–5)	0 (0–0)	135 (118–152)	13 (10–16)
Finland	2022	91 (86–95)	1 (1–1)	1945 (1849–2042)	17 (16–18)
Finland	2023	91 (86–96)	1 (1–1)	1945 (1837–2053)	17 (16–18)
Finland	2024	92 (87–97)	1 (1–1)	1945 (1815–2075)	17 (15–18)
Finland	2025	92 (87–98)	1 (1–1)	1945 (1802–2089)	17 (15–18)
Finland	2026	93 (87–99)	1 (1–1)	1945 (1787–2103)	16 (15–18)
Finland	2027	93 (87–100)	1 (1–1)	1945 (1775–2115)	16 (14–18)
Finland	2028	94 (88–100)	1 (1–1)	1945 (1763–2127)	16 (14–18)
Finland	2029	95 (88–101)	1 (1–1)	1945 (1752–2138)	15 (14–17)
Finland	2030	95 (88–102)	1 (1–1)	1945 (1741–2149)	15 (13–17)

Finland	2031	96 (88–103)	1 (1–1)	1945 (1731–2159)	15 (13–17)
Finland	2032	96 (89–104)	1 (1–1)	1945 (1722–2168)	15 (12–17)
Finland	2033	97 (89–105)	1 (1–1)	1945 (1713–2177)	14 (12–17)
Finland	2034	97 (89–106)	1 (1–1)	1945 (1704–2186)	14 (12–17)
Finland	2035	98 (90–106)	1 (1–1)	1945 (1696–2195)	14 (11–16)
Finland	2036	99 (90–107)	1 (1–1)	1945 (1687–2203)	14 (11–16)
France	2022	1589 (1496–1681)	1 (1–1)	33479 (31248–35709)	27 (24–29)
France	2023	1550 (1419–1680)	1 (1–1)	32331 (29177–35486)	26 (22–30)
France	2024	1511 (1351–1671)	1 (1–1)	31184 (27320–35047)	25 (19–30)
France	2025	1472 (1287–1656)	1 (1–1)	30037 (25576–34498)	23 (16–31)
France	2026	1433 (1226–1639)	1 (1–1)	28889 (23902–33877)	22 (13–31)
France	2027	1394 (1168–1620)	1 (1–1)	27742 (22278–33206)	21 (10–32)
France	2028	1355 (1111–1599)	1 (1–1)	26595 (20693–32496)	20 (7–33)
France	2029	1316 (1055–1577)	1 (1–1)	25448 (19139–31757)	19 (3–34)
France	2030	1277 (1000–1554)	1 (1–1)	24300 (17609–30992)	17 (-0–35)
France	2031	1238 (946–1530)	1 (1–1)	23153 (16099–30207)	16 (-4–36)
France	2032	1199 (893–1505)	1 (1–1)	22006 (14608–29404)	15 (-7–37)
France	2033	1160 (840–1480)	1 (1–1)	20858 (13132–28585)	14 (-11–38)
France	2034	1121 (788–1454)	1 (1–1)	19711 (11669–27754)	13 (-15–40)
France	2035	1082 (737–1427)	1 (1–1)	18564 (10218–26910)	11 (-19–41)
France	2036	1043 (686–1401)	1 (1–2)	17417 (8778–26056)	10 (-23–43)
Gabon	2022	13 (13–13)	0 (0–0)	379 (373–385)	32 (31–32)
Gabon	2023	13 (13–14)	0 (0–0)	382 (368–395)	32 (30–33)
Gabon	2024	13 (13–14)	0 (0–0)	384 (362–407)	32 (30–33)
Gabon	2025	13 (12–14)	0 (0–0)	387 (354–420)	32 (30–34)
Gabon	2026	13 (12–15)	0 (0–0)	390 (345–435)	32 (29–34)
Gabon	2027	14 (12–15)	0 (0–0)	393 (336–451)	32 (29–34)
Gabon	2028	14 (11–16)	0 (0–0)	396 (325–467)	32 (29–34)
Gabon	2029	14 (11–16)	0 (0–0)	399 (313–485)	32 (29–35)
Gabon	2030	14 (11–17)	0 (0–0)	402 (300–504)	32 (28–35)
Gabon	2031	14 (10–18)	0 (0–0)	405 (286–523)	32 (28–35)
Gabon	2032	14 (10–18)	0 (0–0)	408 (272–543)	32 (28–35)
Gabon	2033	14 (9–19)	0 (0–0)	410 (256–564)	32 (28–35)

Gabon	2034	14 (9–20)	0 (0–0)	413 (241–586)	32 (28–36)
Gabon	2035	14 (8–20)	0 (0–0)	416 (224–609)	32 (27–36)
Gabon	2036	14 (8–21)	0 (0–0)	419 (206–632)	32 (27–36)
Gambia	2022	2 (2–3)	0 (0–0)	70 (67–74)	7 (6–7)
Gambia	2023	3 (2–3)	0 (0–0)	72 (67–76)	7 (6–7)
Gambia	2024	3 (2–3)	0 (0–0)	73 (68–79)	7 (6–7)
Gambia	2025	3 (2–3)	0 (0–0)	75 (68–81)	7 (6–7)
Gambia	2026	3 (2–3)	0 (0–0)	76 (69–83)	7 (6–7)
Gambia	2027	3 (2–3)	0 (0–0)	77 (70–85)	7 (6–8)
Gambia	2028	3 (3–3)	0 (0–0)	79 (71–87)	7 (6–8)
Gambia	2029	3 (3–3)	0 (0–0)	80 (71–89)	7 (6–8)
Gambia	2030	3 (3–3)	0 (0–0)	82 (72–91)	7 (5–8)
Gambia	2031	3 (3–3)	0 (0–0)	83 (73–93)	7 (5–8)
Gambia	2032	3 (3–3)	0 (0–0)	84 (74–95)	7 (5–8)
Gambia	2033	3 (3–3)	0 (0–0)	86 (75–96)	7 (5–8)
Gambia	2034	3 (3–3)	0 (0–0)	87 (76–98)	7 (5–8)
Gambia	2035	3 (3–3)	0 (0–0)	88 (77–100)	7 (5–8)
Gambia	2036	3 (3–4)	0 (0–0)	90 (78–102)	7 (5–8)
Georgia	2022	28 (23–32)	1 (1–1)	662 (542–783)	11 (10–13)
Georgia	2023	27 (19–36)	1 (1–1)	651 (430–872)	11 (8–15)
Georgia	2024	27 (15–39)	1 (1–1)	645 (334–956)	11 (6–16)
Georgia	2025	27 (12–42)	1 (1–1)	642 (250–1034)	11 (5–17)
Georgia	2026	27 (9–45)	1 (1–2)	640 (176–1104)	11 (4–18)
Georgia	2027	27 (6–47)	1 (1–2)	639 (110–1168)	11 (3–19)
Georgia	2028	27 (4–49)	1 (1–2)	638 (51–1226)	11 (2–20)
Georgia	2029	27 (2–52)	1 (1–2)	638 (-4–1280)	11 (1–21)
Georgia	2030	27 (-0–53)	1 (1–2)	638 (-54–1330)	11 (0–22)
Georgia	2031	27 (-2–55)	1 (1–2)	638 (-101–1377)	11 (-0–23)
Georgia	2032	27 (-4–57)	1 (1–2)	638 (-145–1421)	11 (-1–23)
Georgia	2033	27 (-5–59)	1 (1–2)	638 (-187–1463)	11 (-2–24)
Georgia	2034	27 (-7–60)	1 (1–2)	638 (-227–1503)	11 (-2–25)
Georgia	2035	27 (-8–62)	2 (1–2)	638 (-266–1541)	11 (-3–25)
Georgia	2036	27 (-10–63)	2 (1–2)	638 (-302–1578)	11 (-3–26)

Germany	2022	2827 (2657–2997)	1 (1–2)	61206 (56998–65415)	36 (33–39)
Germany	2023	2854 (2613–3094)	1 (1–2)	61206 (55254–67159)	36 (32–40)
Germany	2024	2880 (2586–3174)	1 (1–2)	61206 (53916–68497)	36 (31–41)
Germany	2025	2906 (2567–3246)	1 (1–2)	61206 (52789–69624)	36 (30–42)
Germany	2026	2933 (2553–3313)	1 (1–2)	61206 (51795–70618)	36 (29–42)
Germany	2027	2959 (2543–3375)	1 (1–2)	61206 (50897–71516)	36 (29–43)
Germany	2028	2986 (2536–3435)	1 (1–2)	61206 (50071–72342)	36 (28–43)
Germany	2029	3012 (2531–3492)	1 (1–2)	61206 (49302–73111)	36 (28–44)
Germany	2030	3038 (2529–3548)	1 (1–2)	61206 (48580–73833)	36 (27–45)
Germany	2031	3065 (2528–3602)	1 (1–2)	61206 (47897–74516)	36 (27–45)
Germany	2032	3091 (2528–3654)	1 (1–2)	61206 (47247–75166)	36 (26–45)
Germany	2033	3117 (2529–3706)	1 (1–2)	61206 (46626–75787)	36 (26–46)
Germany	2034	3144 (2531–3756)	1 (1–2)	61206 (46031–76382)	36 (25–46)
Germany	2035	3170 (2535–3806)	1 (1–2)	61206 (45458–76955)	36 (25–47)
Germany	2036	3197 (2539–3854)	1 (1–2)	61206 (44905–77508)	36 (24–47)
Ghana	2022	45 (44–45)	0 (0–0)	1202 (1191–1213)	7 (7–7)
Ghana	2023	45 (44–46)	0 (0–0)	1217 (1193–1242)	7 (6–7)
Ghana	2024	45 (44–47)	0 (0–0)	1233 (1192–1273)	6 (6–7)
Ghana	2025	46 (44–48)	0 (0–0)	1248 (1189–1307)	6 (6–7)
Ghana	2026	46 (43–49)	0 (0–0)	1263 (1183–1343)	6 (6–7)
Ghana	2027	47 (43–50)	0 (0–0)	1279 (1175–1382)	6 (6–7)
Ghana	2028	47 (43–52)	0 (0–0)	1294 (1166–1422)	6 (5–7)
Ghana	2029	48 (42–53)	0 (0–0)	1309 (1155–1463)	6 (5–7)
Ghana	2030	48 (42–55)	0 (0–0)	1324 (1142–1507)	6 (5–7)
Ghana	2031	49 (41–56)	0 (0–0)	1340 (1128–1552)	6 (5–7)
Ghana	2032	49 (40–58)	0 (0–0)	1355 (1112–1598)	6 (5–8)
Ghana	2033	49 (40–59)	0 (0–0)	1370 (1095–1646)	6 (4–8)
Ghana	2034	50 (39–61)	0 (0–0)	1385 (1076–1695)	6 (4–8)
Ghana	2035	50 (38–63)	0 (0–0)	1401 (1056–1745)	6 (4–8)
Ghana	2036	51 (37–64)	0 (0–0)	1416 (1035–1797)	6 (4–8)
Greece	2022	206 (196–216)	2 (2–2)	4264 (4062–4465)	21 (20–22)
Greece	2023	204 (191–217)	2 (2–2)	4248 (3991–4505)	21 (19–22)
Greece	2024	203 (188–218)	2 (2–2)	4235 (3949–4522)	20 (18–22)

Greece	2025	202 (186–218)	2 (2–2)	4225 (3922–4529)	20 (18–22)
Greece	2026	201 (184–218)	2 (2–2)	4218 (3903–4532)	20 (18–22)
Greece	2027	200 (183–217)	2 (2–2)	4211 (3891–4532)	20 (17–22)
Greece	2028	199 (181–217)	2 (2–2)	4206 (3882–4531)	20 (17–22)
Greece	2029	199 (181–217)	2 (2–2)	4202 (3875–4529)	19 (16–22)
Greece	2030	198 (180–216)	2 (2–2)	4199 (3871–4528)	19 (16–22)
Greece	2031	198 (179–216)	2 (2–2)	4197 (3867–4526)	19 (16–22)
Greece	2032	197 (179–216)	2 (2–2)	4195 (3865–4525)	19 (15–22)
Greece	2033	197 (178–216)	2 (2–2)	4193 (3863–4524)	19 (15–22)
Greece	2034	197 (178–215)	2 (2–2)	4192 (3861–4523)	18 (14–22)
Greece	2035	197 (178–215)	2 (2–2)	4191 (3860–4522)	18 (14–22)
Greece	2036	196 (178–215)	2 (2–2)	4190 (3859–4521)	18 (14–22)
Greenland	2022	4 (3–4)	3 (3–3)	92 (89–95)	114 (110–118)
Greenland	2023	3 (3–4)	3 (3–3)	91 (88–95)	110 (106–115)
Greenland	2024	4 (3–4)	3 (3–3)	90 (86–94)	107 (101–112)
Greenland	2025	3 (3–4)	3 (3–3)	89 (84–94)	103 (97–108)
Greenland	2026	4 (3–4)	3 (3–3)	88 (82–94)	99 (93–105)
Greenland	2027	4 (3–4)	3 (3–3)	87 (80–95)	95 (89–102)
Greenland	2028	4 (3–4)	3 (2–3)	87 (78–95)	92 (85–98)
Greenland	2029	4 (3–4)	3 (2–3)	86 (76–95)	88 (81–95)
Greenland	2030	4 (3–4)	3 (2–3)	85 (74–96)	84 (77–91)
Greenland	2031	4 (3–4)	3 (2–3)	84 (71–96)	80 (73–88)
Greenland	2032	4 (3–4)	3 (2–3)	83 (69–97)	77 (69–85)
Greenland	2033	4 (3–4)	3 (2–3)	82 (66–98)	73 (65–81)
Greenland	2034	4 (3–4)	3 (2–3)	81 (64–99)	69 (61–78)
Greenland	2035	4 (3–4)	3 (2–3)	80 (61–99)	65 (56–74)
Greenland	2036	4 (3–4)	2 (2–3)	79 (58–100)	62 (52–71)
Grenada	2022	1 (1–1)	1 (1–1)	24 (20–27)	19 (15–22)
Grenada	2023	1 (1–1)	1 (1–1)	24 (19–29)	19 (13–24)
Grenada	2024	1 (1–1)	1 (1–1)	24 (18–30)	19 (11–26)
Grenada	2025	1 (1–1)	1 (1–1)	24 (17–31)	19 (10–28)
Grenada	2026	1 (1–1)	1 (1–1)	24 (16–32)	19 (8–29)
Grenada	2027	1 (1–1)	1 (1–1)	24 (15–32)	19 (7–31)

Grenada	2028	1 (1–1)	1 (1–1)	24 (15–33)	19 (6–32)
Grenada	2029	1 (1–1)	1 (1–1)	24 (14–34)	19 (5–33)
Grenada	2030	1 (0–1)	1 (1–1)	24 (13–34)	19 (4–34)
Grenada	2031	1 (0–1)	1 (1–1)	24 (13–35)	19 (3–35)
Grenada	2032	1 (0–1)	1 (0–1)	24 (12–35)	19 (2–36)
Grenada	2033	1 (0–1)	1 (0–1)	24 (12–36)	19 (1–36)
Grenada	2034	1 (0–1)	1 (0–1)	24 (11–36)	19 (0–37)
Grenada	2035	1 (0–1)	1 (0–1)	24 (11–37)	19 (-0–38)
Grenada	2036	1 (0–1)	1 (0–1)	24 (10–37)	19 (-1–39)
Guam	2022	1 (1–1)	1 (0–1)	29 (27–31)	14 (12–15)
Guam	2023	1 (1–1)	1 (0–1)	29 (26–32)	14 (12–15)
Guam	2024	1 (1–1)	1 (0–1)	30 (26–33)	14 (12–16)
Guam	2025	1 (1–1)	1 (0–1)	30 (26–34)	14 (11–16)
Guam	2026	1 (1–1)	1 (0–1)	31 (26–35)	14 (11–16)
Guam	2027	1 (1–1)	1 (0–1)	31 (26–36)	14 (11–17)
Guam	2028	1 (1–1)	1 (0–1)	32 (26–37)	14 (11–17)
Guam	2029	1 (1–1)	1 (0–1)	32 (27–38)	14 (10–17)
Guam	2030	1 (1–1)	1 (0–1)	33 (27–39)	14 (10–17)
Guam	2031	1 (1–1)	1 (0–1)	33 (27–39)	14 (10–17)
Guam	2032	1 (1–1)	1 (0–1)	34 (27–40)	14 (10–18)
Guam	2033	1 (1–1)	1 (0–1)	34 (27–41)	14 (10–18)
Guam	2034	1 (1–1)	1 (0–1)	35 (27–42)	14 (9–18)
Guam	2035	1 (1–2)	1 (0–1)	35 (28–43)	14 (9–18)
Guam	2036	1 (1–2)	1 (0–1)	36 (28–43)	14 (9–18)
Guatemala	2022	21 (19–23)	0 (0–0)	487 (443–530)	4 (3–5)
Guatemala	2023	21 (19–24)	0 (0–0)	494 (433–556)	4 (3–6)
Guatemala	2024	22 (19–25)	0 (0–0)	501 (426–577)	4 (3–6)
Guatemala	2025	22 (18–26)	0 (0–0)	509 (422–596)	4 (3–6)
Guatemala	2026	22 (18–26)	0 (0–0)	516 (418–613)	4 (2–6)
Guatemala	2027	23 (18–27)	0 (0–0)	523 (416–630)	4 (2–6)
Guatemala	2028	23 (18–28)	0 (0–0)	530 (415–645)	4 (2–7)
Guatemala	2029	23 (18–29)	0 (0–0)	538 (414–661)	4 (2–7)
Guatemala	2030	24 (18–29)	0 (0–0)	545 (414–675)	4 (2–7)

Guatemala	2031	24 (18–30)	0 (0–0)	552 (414–690)	4 (2–7)
Guatemala	2032	25 (18–31)	0 (0–0)	559 (415–704)	4 (1–7)
Guatemala	2033	25 (19–31)	0 (0–0)	566 (416–717)	4 (1–7)
Guatemala	2034	25 (19–32)	0 (0–0)	574 (417–731)	4 (1–7)
Guatemala	2035	26 (19–32)	0 (0–0)	581 (418–744)	4 (1–8)
Guatemala	2036	26 (19–33)	0 (0–0)	588 (420–757)	4 (1–8)
Guinea	2022	9 (9–9)	0 (0–0)	254 (251–257)	4 (4–4)
Guinea	2023	9 (9–9)	0 (0–0)	254 (247–261)	4 (4–4)
Guinea	2024	9 (9–10)	0 (0–0)	254 (242–265)	4 (4–4)
Guinea	2025	9 (8–10)	0 (0–0)	254 (236–271)	4 (4–4)
Guinea	2026	9 (8–10)	0 (0–0)	253 (230–277)	4 (3–4)
Guinea	2027	9 (8–10)	0 (0–0)	253 (224–283)	4 (3–5)
Guinea	2028	9 (8–10)	0 (0–0)	253 (216–290)	4 (3–5)
Guinea	2029	9 (7–10)	0 (0–0)	253 (209–298)	4 (3–5)
Guinea	2030	9 (7–11)	0 (0–0)	253 (200–306)	4 (3–5)
Guinea	2031	9 (7–11)	0 (0–0)	253 (192–315)	4 (2–5)
Guinea	2032	9 (6–11)	0 (0–0)	253 (183–324)	4 (2–5)
Guinea	2033	9 (6–11)	0 (0–0)	253 (173–333)	3 (2–5)
Guinea	2034	9 (6–12)	0 (0–0)	253 (163–343)	3 (2–5)
Guinea	2035	9 (5–12)	0 (0–0)	253 (153–353)	3 (1–5)
Guinea	2036	9 (5–12)	0 (0–0)	253 (142–363)	3 (1–6)
Guinea-Bissau	2022	5 (5–5)	0 (0–0)	162 (160–163)	19 (19–19)
Guinea-Bissau	2023	5 (5–5)	0 (0–0)	164 (160–168)	19 (18–19)
Guinea-Bissau	2024	5 (5–6)	0 (0–0)	167 (160–173)	18 (18–19)
Guinea-Bissau	2025	5 (5–6)	0 (0–0)	169 (158–179)	18 (17–20)
Guinea-Bissau	2026	5 (5–6)	0 (0–0)	171 (156–186)	18 (16–20)
Guinea-Bissau	2027	5 (5–6)	0 (0–0)	173 (154–193)	18 (15–21)
Guinea-Bissau	2028	5 (5–6)	0 (0–0)	175 (151–200)	18 (15–21)
Guinea-Bissau	2029	6 (4–7)	0 (0–0)	178 (147–208)	18 (14–22)
Guinea-Bissau	2030	6 (4–7)	0 (0–0)	180 (143–216)	18 (13–23)
Guinea-Bissau	2031	6 (4–7)	0 (0–0)	182 (139–225)	18 (12–23)
Guinea-Bissau	2032	6 (4–7)	0 (0–0)	184 (135–234)	18 (11–24)
Guinea-Bissau	2033	6 (4–8)	0 (0–0)	187 (130–243)	17 (10–25)

Guinea-Bissau	2034	6 (3–8)	0 (0–0)	189 (125–252)	17 (9–26)
Guinea-Bissau	2035	6 (3–8)	0 (0–0)	191 (120–262)	17 (8–26)
Guinea-Bissau	2036	6 (3–9)	0 (0–0)	193 (114–272)	17 (7–27)
Guyana	2022	2 (2–2)	0 (0–0)	63 (59–67)	9 (8–9)
Guyana	2023	2 (2–2)	0 (0–0)	64 (58–70)	9 (8–10)
Guyana	2024	2 (2–2)	0 (0–0)	65 (58–72)	9 (8–10)
Guyana	2025	2 (2–2)	0 (0–0)	66 (58–73)	9 (8–10)
Guyana	2026	2 (2–3)	0 (0–0)	66 (57–75)	9 (8–10)
Guyana	2027	2 (2–3)	0 (0–0)	67 (57–77)	9 (8–10)
Guyana	2028	2 (2–3)	0 (0–0)	68 (57–78)	9 (8–10)
Guyana	2029	2 (2–3)	0 (0–1)	69 (57–80)	9 (8–10)
Guyana	2030	2 (2–3)	0 (0–1)	69 (57–81)	9 (8–10)
Guyana	2031	2 (2–3)	0 (0–1)	70 (58–83)	9 (8–10)
Guyana	2032	2 (2–3)	0 (0–1)	71 (58–84)	9 (8–10)
Guyana	2033	2 (2–3)	0 (0–1)	72 (58–85)	9 (8–10)
Guyana	2034	2 (2–3)	0 (0–1)	72 (58–87)	9 (8–10)
Guyana	2035	2 (2–3)	0 (0–1)	73 (58–88)	9 (8–10)
Guyana	2036	2 (2–3)	0 (0–1)	74 (58–89)	9 (8–10)
Haiti	2022	23 (23–24)	0 (0–0)	701 (688–714)	8 (8–9)
Haiti	2023	24 (23–24)	0 (0–0)	712 (692–732)	8 (8–9)
Haiti	2024	24 (23–25)	0 (0–0)	725 (692–758)	8 (7–9)
Haiti	2025	24 (23–26)	0 (0–0)	736 (691–781)	8 (7–9)
Haiti	2026	25 (23–27)	0 (0–0)	749 (689–809)	8 (7–9)
Haiti	2027	25 (23–27)	0 (0–0)	760 (685–836)	8 (6–9)
Haiti	2028	25 (22–28)	0 (0–0)	773 (680–865)	8 (6–10)
Haiti	2029	26 (22–29)	0 (0–0)	784 (674–895)	8 (5–10)
Haiti	2030	26 (22–30)	0 (0–0)	796 (667–926)	8 (5–10)
Haiti	2031	26 (21–31)	0 (0–0)	808 (659–958)	7 (4–11)
Haiti	2032	27 (21–32)	0 (0–0)	820 (650–991)	7 (4–11)
Haiti	2033	27 (21–33)	0 (0–0)	832 (640–1025)	7 (3–11)
Haiti	2034	27 (20–34)	0 (0–0)	844 (629–1060)	7 (3–12)
Haiti	2035	28 (20–35)	0 (0–0)	856 (617–1095)	7 (2–12)
Haiti	2036	28 (19–36)	0 (0–0)	868 (605–1131)	7 (1–13)

High SDI	2022	36244 (35747–36741	1 (1–1)	763234 (751709–774759	38 (37–39)
High SDI	2023	36442 (35641–37244	1 (1–1)	763047 (744053–782040	37 (36–38)
High SDI	2024	36641 (35536–37746	1 (1–1)	762859 (736226–789492	36 (34–38)
High SDI	2025	36839 (35419–38259	1 (1–1)	762672 (727973–797370	36 (33–38)
High SDI	2026	37038 (35287–38788	1 (1–1)	762484 (719237–805731	35 (31–38)
High SDI	2027	37236 (35139–39334	1 (1–1)	762297 (710011–814583	34 (30–39)
High SDI	2028	37435 (34974–39896	1 (1–1)	762109 (700303–823916	34 (28–39)
High SDI	2029	37633 (34793–40474	1 (1–1)	761922 (690127–833716	33 (27–39)
High SDI	2030	37832 (34596–41068	1 (1–1)	761734 (679499–843969	32 (25–39)
High SDI	2031	38030 (34383–41677	1 (1–1)	761547 (668434–854659	31 (23–40)
High SDI	2032	38229 (34156–42302	1 (1–1)	761359 (656946–865773	31 (22–40)
High SDI	2033	38427 (33913–42941	1 (1–1)	761172 (645048–877295	30 (20–40)
High SDI	2034	38626 (33657–43594	1 (1–1)	760984 (632754–889214	29 (18–41)
High SDI	2035	38824 (33387–44261	1 (1–1)	760797 (620075–901518	29 (16–41)
High SDI	2036	39023 (33103–44942	1 (1–1)	760609 (607022–914196	28 (14–42)
High-middle SDI	2022	76594 (75279–77909	1 (1–1)	84131 (1753763–181449	87 (85–90)
High-middle SDI	2023	77549 (74610–80487	1 (1–1)	00369 (1734344–186639	86 (81–91)
High-middle SDI	2024	78488 (74515–82462	1 (1–1)	16607 (1728310–190490	85 (78–92)
High-middle SDI	2025	79428 (74637–84218	1 (1–1)	32845 (1726858–193883	84 (75–92)
High-middle SDI	2026	80367 (74880–85854	1 (1–1)	49083 (1727962–197020	83 (73–92)
High-middle SDI	2027	81306 (75201–87411	1 (1–1)	65321 (1730757–199988	81 (71–92)
High-middle SDI	2028	82245 (75579–88912	1 (1–1)	81559 (1734779–202834	80 (69–92)
High-middle SDI	2029	83185 (76001–90368	1 (1–1)	97797 (1739741–205585	79 (67–92)
High-middle SDI	2030	84124 (76458–91790	1 (1–1)	14035 (1745457–208261	78 (65–91)
High-middle SDI	2031	85063 (76944–93183	1 (1–1)	30273 (1751791–210875	77 (63–91)
High-middle SDI	2032	86002 (77453–94552	1 (1–1)	46511 (1758647–213437	76 (61–90)
High-middle SDI	2033	86942 (77983–95900	1 (1–1)	62749 (1765950–215954	75 (60–90)
High-middle SDI	2034	87881 (78532–97231	1 (1–1)	78987 (1773641–218433	74 (58–90)
High-middle SDI	2035	88820 (79095–98545	1 (1–1)	95225 (1781674–220877	73 (56–89)
High-middle SDI	2036	89760 (79673–99846	1 (1–1)	11463 (1790011–223291	71 (54–89)
Honduras	2022	21 (20–22)	0 (0–0)	493 (476–510)	8 (7–8)
Honduras	2023	21 (20–23)	0 (0–1)	500 (467–532)	8 (7–8)
Honduras	2024	22 (20–24)	0 (0–1)	506 (456–557)	8 (7–8)

Honduras	2025	22 (20–25)	0 (0–1)	513 (442–583)	8 (6–9)
Honduras	2026	23 (20–26)	0 (0–1)	520 (427–612)	8 (6–9)
Honduras	2027	23 (20–27)	0 (0–1)	526 (409–643)	8 (6–9)
Honduras	2028	24 (20–27)	1 (0–1)	533 (390–676)	8 (6–9)
Honduras	2029	24 (20–28)	1 (0–1)	540 (369–710)	8 (6–9)
Honduras	2030	25 (20–29)	1 (0–1)	546 (347–746)	8 (6–9)
Honduras	2031	25 (20–30)	1 (0–1)	553 (323–783)	8 (6–10)
Honduras	2032	26 (20–31)	1 (0–1)	560 (297–822)	8 (5–10)
Honduras	2033	26 (21–31)	1 (0–1)	566 (271–862)	8 (5–10)
Honduras	2034	27 (21–32)	1 (0–1)	573 (243–903)	8 (5–10)
Honduras	2035	27 (21–33)	1 (0–1)	580 (213–946)	8 (5–10)
Honduras	2036	28 (21–34)	1 (0–1)	586 (183–990)	8 (5–10)
Hungary	2022	242 (222–262)	2 (2–2)	6199 (5619–6779)	36 (32–40)
Hungary	2023	235 (204–267)	2 (2–2)	5990 (5052–6928)	35 (28–41)
Hungary	2024	229 (186–272)	2 (2–2)	5781 (4485–7077)	34 (25–43)
Hungary	2025	222 (168–277)	2 (2–2)	5572 (3904–7241)	32 (21–44)
Hungary	2026	216 (149–282)	2 (2–2)	5363 (3303–7423)	31 (16–46)
Hungary	2027	209 (130–288)	2 (2–2)	5154 (2683–7625)	30 (12–48)
Hungary	2028	202 (110–294)	2 (2–2)	4945 (2043–7847)	29 (8–50)
Hungary	2029	196 (90–301)	2 (2–2)	4736 (1384–8089)	28 (3–52)
Hungary	2030	189 (70–308)	2 (2–2)	4527 (705–8350)	26 (-2–54)
Hungary	2031	182 (49–316)	2 (2–2)	4318 (8–8629)	25 (-7–57)
Hungary	2032	176 (27–325)	2 (2–2)	4109 (-707–8926)	24 (-12–60)
Hungary	2033	169 (5–333)	2 (2–2)	3901 (-1440–9241)	23 (-17–62)
Hungary	2034	163 (-18–343)	2 (2–2)	3692 (-2190–9573)	21 (-22–65)
Hungary	2035	156 (-41–353)	2 (2–2)	3483 (-2956–9921)	20 (-28–68)
Hungary	2036	149 (-64–363)	2 (2–2)	3274 (-3739–10286)	19 (-33–71)
Iceland	2022	10 (9–10)	1 (1–1)	206 (196–215)	36 (34–39)
Iceland	2023	10 (9–11)	1 (1–1)	205 (189–222)	35 (31–39)
Iceland	2024	10 (9–11)	1 (1–1)	205 (183–227)	35 (29–40)
Iceland	2025	10 (8–11)	1 (1–1)	205 (178–232)	34 (28–40)
Iceland	2026	10 (8–11)	1 (1–1)	205 (174–236)	33 (26–40)
Iceland	2027	10 (8–11)	1 (1–1)	205 (170–240)	32 (25–40)

Iceland	2028	10 (8–12)	1 (1–1)	205 (167–243)	32 (23–40)
Iceland	2029	10 (8–12)	1 (1–1)	205 (163–246)	31 (22–40)
Iceland	2030	10 (8–12)	1 (1–1)	205 (161–249)	30 (21–40)
Iceland	2031	10 (7–12)	1 (1–1)	205 (158–252)	29 (19–39)
Iceland	2032	10 (7–12)	1 (1–1)	205 (155–254)	29 (18–39)
Iceland	2033	10 (7–12)	1 (1–1)	205 (153–257)	28 (17–39)
Iceland	2034	10 (7–12)	1 (1–1)	205 (150–259)	27 (16–39)
Iceland	2035	10 (7–12)	1 (1–1)	205 (148–261)	26 (14–38)
Iceland	2036	10 (7–13)	1 (1–1)	205 (146–263)	26 (13–38)
India	2022	13429 (13152–13707	0 (0–0)	360241 (353454–367027	28 (27–29)
India	2023	13636 (13243–14029	0 (0–0)	365103 (353742–376464	28 (26–29)
India	2024	13843 (13362–14324	0 (0–0)	370047 (354972–385122	27 (25–29)
India	2025	14049 (13494–14605	0 (0–0)	375018 (356827–393209	27 (25–29)
India	2026	14256 (13635–14877	0 (0–0)	379998 (359106–400891	27 (24–29)
India	2027	14463 (13783–15143	0 (0–0)	384982 (361686–408278	26 (23–29)
India	2028	14669 (13935–15404	0 (0–0)	389967 (364488–415446	26 (23–29)
India	2029	14876 (14091–15662	0 (0–0)	394952 (367462–422443	26 (22–29)
India	2030	15083 (14250–15916	0 (0–0)	399938 (370573–429302	25 (21–29)
India	2031	15290 (14411–16168	0 (0–0)	404923 (373796–436050	25 (21–29)
India	2032	15496 (14575–16417	0 (0–0)	409908 (377114–442702	25 (20–29)
India	2033	15703 (14741–16665	0 (0–0)	414894 (380513–449274	24 (20–29)
India	2034	15910 (14908–16911	0 (0–0)	419879 (383982–455776	24 (19–29)
India	2035	16116 (15077–17155	0 (0–0)	424865 (387512–462217	24 (19–29)
India	2036	16323 (15247–17398	0 (0–0)	429850 (391097–468603	23 (18–28)
Indonesia	2022	1049 (1037–1061)	1 (1–1)	28015 (27700–28330)	11 (10–11)
Indonesia	2023	1082 (1060–1103)	1 (1–1)	28871 (28311–29430)	11 (10–11)
Indonesia	2024	1115 (1081–1149)	1 (1–1)	29726 (28900–30553)	11 (10–11)
Indonesia	2025	1148 (1100–1197)	1 (1–1)	30582 (29462–31701)	11 (10–11)
Indonesia	2026	1181 (1117–1246)	1 (1–1)	31437 (30000–32875)	11 (10–11)
Indonesia	2027	1215 (1133–1296)	1 (1–1)	32293 (30513–34073)	11 (10–11)
Indonesia	2028	1248 (1148–1348)	1 (1–1)	33149 (31005–35293)	11 (10–11)
Indonesia	2029	1281 (1161–1401)	1 (1–1)	34004 (31475–36534)	11 (10–11)
Indonesia	2030	1314 (1173–1455)	1 (1–1)	34860 (31925–37795)	11 (10–12)

Indonesia	2031	1348 (1184–1511)	1 (1–1)	35716 (32356–39075)	11 (10–12)
Indonesia	2032	1381 (1194–1567)	1 (1–1)	36571 (32768–40374)	11 (10–12)
Indonesia	2033	1414 (1204–1624)	1 (0–1)	37427 (33162–41691)	11 (10–12)
Indonesia	2034	1447 (1212–1683)	1 (0–1)	38282 (33540–43025)	11 (10–12)
Indonesia	2035	1480 (1219–1742)	1 (0–1)	39138 (33901–44376)	11 (10–12)
Indonesia	2036	1514 (1225–1802)	1 (0–1)	39994 (34245–45742)	11 (10–12)
Iran (Islamic Republic of)	2022	769 (745–794)	0 (0–0)	17950 (17328–18573)	22 (21–23)
Iran (Islamic Republic of)	2023	783 (743–823)	0 (0–0)	18245 (17365–19126)	22 (20–24)
Iran (Islamic Republic of)	2024	797 (744–850)	0 (0–0)	18540 (17461–19619)	22 (20–24)
Iran (Islamic Republic of)	2025	811 (748–874)	0 (0–0)	18835 (17590–20080)	22 (19–24)
Iran (Islamic Republic of)	2026	825 (753–897)	0 (0–0)	19130 (17737–20522)	21 (18–25)
Iran (Islamic Republic of)	2027	839 (759–919)	0 (0–0)	19425 (17899–20950)	21 (17–25)
Iran (Islamic Republic of)	2028	853 (766–940)	0 (0–0)	19720 (18072–21367)	21 (17–25)
Iran (Islamic Republic of)	2029	867 (773–961)	0 (0–0)	20015 (18253–21776)	20 (16–25)
Iran (Islamic Republic of)	2030	881 (781–981)	0 (0–1)	20310 (18441–22178)	20 (15–25)
Iran (Islamic Republic of)	2031	895 (789–1001)	0 (0–1)	20604 (18635–22574)	20 (15–25)
Iran (Islamic Republic of)	2032	909 (798–1020)	0 (0–1)	20899 (18834–22965)	20 (14–25)
Iran (Islamic Republic of)	2033	923 (807–1039)	0 (0–1)	21194 (19037–23351)	19 (14–25)
Iran (Islamic Republic of)	2034	937 (816–1059)	0 (0–1)	21489 (19244–23734)	19 (13–25)
Iran (Islamic Republic of)	2035	951 (825–1077)	0 (0–1)	21784 (19454–24114)	19 (13–25)
Iran (Islamic Republic of)	2036	965 (834–1096)	0 (0–1)	22079 (19667–24491)	19 (12–25)
Iraq	2022	116 (113–120)	1 (1–1)	2910 (2840–2980)	12 (11–12)
Iraq	2023	121 (115–127)	1 (1–1)	3036 (2917–3156)	12 (11–12)
Iraq	2024	128 (119–138)	1 (1–1)	3200 (3008–3392)	12 (11–13)
Iraq	2025	134 (121–147)	1 (1–1)	3341 (3072–3610)	12 (11–14)
Iraq	2026	141 (124–158)	1 (1–1)	3496 (3138–3853)	13 (11–14)
Iraq	2027	147 (126–168)	1 (1–1)	3642 (3190–4093)	13 (11–15)
Iraq	2028	154 (128–180)	1 (1–1)	3793 (3239–4347)	13 (10–16)
Iraq	2029	160 (129–191)	1 (1–1)	3941 (3279–4604)	13 (10–17)
Iraq	2030	166 (130–203)	1 (1–1)	4092 (3314–4870)	14 (10–17)
Iraq	2031	173 (131–215)	1 (1–1)	4241 (3342–5140)	14 (10–18)
Iraq	2032	179 (131–227)	1 (1–1)	4390 (3364–5416)	14 (10–19)
Iraq	2033	186 (131–240)	1 (1–1)	4540 (3381–5698)	15 (9–20)

Iraq	2034	192 (131–253)	1 (1–1)	4689 (3393–5985)	15 (9–21)
Iraq	2035	199 (131–266)	1 (1–1)	4839 (3400–6277)	15 (9–22)
Iraq	2036	205 (131–279)	1 (1–2)	4988 (3402–6574)	15 (8–23)
Ireland	2022	155 (148–162)	1 (1–1)	3057 (2914–3199)	37 (35–40)
Ireland	2023	155 (145–165)	1 (1–1)	3037 (2836–3238)	36 (32–39)
Ireland	2024	155 (143–167)	1 (1–1)	3017 (2771–3263)	34 (30–38)
Ireland	2025	155 (141–169)	1 (1–1)	2997 (2713–3282)	32 (28–37)
Ireland	2026	155 (140–170)	1 (1–1)	2977 (2659–3296)	31 (25–36)
Ireland	2027	155 (138–172)	1 (1–1)	2958 (2609–3306)	29 (23–35)
Ireland	2028	155 (137–173)	1 (1–1)	2938 (2561–3314)	27 (21–34)
Ireland	2029	155 (136–174)	1 (0–1)	2918 (2516–3320)	26 (19–33)
Ireland	2030	155 (135–176)	1 (0–1)	2898 (2471–3325)	24 (17–31)
Ireland	2031	155 (134–177)	1 (0–1)	2878 (2428–3328)	22 (15–30)
Ireland	2032	155 (132–178)	1 (0–1)	2859 (2387–3330)	21 (13–29)
Ireland	2033	155 (131–179)	1 (0–1)	2839 (2346–3332)	19 (11–28)
Ireland	2034	155 (130–180)	1 (0–1)	2819 (2306–3332)	17 (9–26)
Ireland	2035	155 (130–181)	0 (0–1)	2799 (2267–3332)	16 (7–25)
Ireland	2036	155 (129–182)	0 (0–1)	2779 (2228–3330)	14 (5–24)
Israel	2022	60 (57–63)	1 (1–1)	1239 (1172–1306)	10 (9–11)
Israel	2023	61 (56–65)	1 (1–1)	1239 (1122–1355)	10 (8–12)
Israel	2024	61 (55–67)	1 (1–1)	1239 (1088–1389)	10 (8–12)
Israel	2025	62 (54–69)	1 (1–1)	1239 (1060–1417)	9 (7–12)
Israel	2026	62 (54–71)	1 (1–1)	1239 (1037–1441)	9 (6–12)
Israel	2027	63 (54–72)	1 (1–1)	1239 (1015–1462)	9 (6–12)
Israel	2028	64 (54–74)	1 (1–1)	1239 (996–1482)	9 (5–12)
Israel	2029	64 (54–75)	1 (1–1)	1239 (978–1500)	8 (5–12)
Israel	2030	65 (53–76)	1 (1–1)	1239 (961–1517)	8 (4–12)
Israel	2031	65 (53–77)	1 (1–1)	1239 (945–1533)	8 (4–12)
Israel	2032	66 (53–79)	1 (1–1)	1239 (930–1548)	7 (3–12)
Israel	2033	67 (53–80)	1 (1–1)	1239 (915–1562)	7 (3–12)
Israel	2034	67 (53–81)	1 (1–1)	1239 (902–1576)	7 (2–11)
Israel	2035	68 (53–82)	1 (1–1)	1239 (888–1589)	7 (2–11)
Israel	2036	68 (54–83)	1 (1–1)	1239 (876–1602)	6 (2–11)

Italy	2022	824 (794–854)	1 (1–1)	16467 (15734–17201)	13 (12–13)
Italy	2023	807 (765–849)	1 (1–1)	15974 (14937–17012)	12 (11–13)
Italy	2024	790 (739–841)	1 (1–1)	15481 (14210–16752)	12 (10–13)
Italy	2025	773 (714–832)	1 (1–1)	14988 (13521–16455)	11 (9–13)
Italy	2026	756 (690–823)	1 (1–1)	14495 (12854–16135)	11 (8–13)
Italy	2027	739 (667–812)	1 (1–1)	14002 (12205–15799)	10 (7–13)
Italy	2028	722 (644–801)	1 (1–1)	13509 (11568–15450)	10 (6–14)
Italy	2029	706 (622–789)	1 (1–1)	13016 (10941–15091)	9 (5–14)
Italy	2030	689 (600–778)	1 (1–1)	12523 (10322–14723)	9 (4–14)
Italy	2031	672 (578–766)	1 (1–1)	12029 (9710–14349)	8 (3–14)
Italy	2032	655 (557–753)	1 (1–1)	11536 (9103–13969)	8 (2–14)
Italy	2033	638 (535–741)	1 (1–1)	11043 (8502–13585)	7 (1–14)
Italy	2034	621 (514–728)	1 (1–1)	10550 (7905–13195)	7 (-0–14)
Italy	2035	604 (493–715)	1 (1–1)	10057 (7312–12802)	6 (-2–14)
Italy	2036	587 (472–702)	1 (1–1)	9564 (6723–12405)	6 (-3–15)
Jamaica	2022	21 (17–26)	0 (0–0)	506 (387–625)	17 (12–22)
Jamaica	2023	22 (17–27)	0 (0–0)	513 (384–641)	17 (11–22)
Jamaica	2024	21 (15–28)	0 (0–1)	509 (352–666)	17 (10–23)
Jamaica	2025	22 (15–28)	0 (0–1)	511 (341–681)	17 (9–24)
Jamaica	2026	21 (14–29)	0 (0–1)	510 (322–697)	17 (9–25)
Jamaica	2027	22 (14–29)	0 (0–1)	510 (310–711)	17 (8–25)
Jamaica	2028	21 (13–30)	0 (0–1)	510 (295–724)	17 (7–26)
Jamaica	2029	22 (13–30)	0 (0–1)	510 (284–737)	17 (7–27)
Jamaica	2030	22 (12–31)	0 (0–1)	510 (271–749)	17 (6–27)
Jamaica	2031	22 (12–31)	0 (0–1)	510 (260–760)	17 (6–28)
Jamaica	2032	22 (11–32)	0 (0–1)	510 (249–771)	17 (5–28)
Jamaica	2033	22 (11–32)	0 (0–1)	510 (239–781)	17 (5–28)
Jamaica	2034	22 (10–33)	0 (0–1)	510 (229–791)	17 (5–29)
Jamaica	2035	22 (10–33)	0 (0–1)	510 (220–801)	17 (4–29)
Jamaica	2036	22 (10–33)	0 (0–1)	510 (210–810)	17 (4–30)
Japan	2022	5982 (5850–6113)	1 (1–1)	.08686 (105502–111870	33 (32–34)
Japan	2023	6028 (5798–6258)	1 (1–1)	.08601 (102800–114403	33 (30–35)
Japan	2024	6074 (5738–6411)	1 (1–1)	108516 (99792–117240)	32 (29–36)

Japan	2025	6121 (5668–6574)	1 (1–1)	108431 (96471–120391)	32 (27–36)
Japan	2026	6167 (5589–6746)	1 (1–1)	108347 (92855–123839)	31 (25–37)
Japan	2027	6214 (5500–6927)	1 (1–1)	108262 (88960–127563)	31 (23–38)
Japan	2028	6260 (5403–7117)	1 (1–1)	108177 (84806–131548)	30 (21–39)
Japan	2029	6306 (5298–7315)	1 (1–1)	108092 (80405–135779)	30 (19–40)
Japan	2030	6353 (5185–7521)	1 (1–1)	108007 (75772–140242)	29 (17–41)
Japan	2031	6399 (5064–7734)	1 (1–1)	107922 (70916–144928)	29 (15–43)
Japan	2032	6445 (4937–7954)	1 (1–1)	107837 (65848–149827)	28 (12–44)
Japan	2033	6492 (4802–8182)	1 (1–1)	107752 (60576–154929)	28 (10–45)
Japan	2034	6538 (4661–8415)	1 (1–1)	107668 (55107–160228)	27 (8–47)
Japan	2035	6585 (4513–8656)	1 (1–1)	107583 (49449–165716)	27 (5–48)
Japan	2036	6631 (4360–8902)	1 (1–1)	107498 (43607–171388)	26 (3–50)
Jordan	2022	29 (29–30)	1 (1–1)	767 (759–776)	9 (9–10)
Jordan	2023	31 (30–32)	1 (1–1)	819 (796–841)	9 (9–10)
Jordan	2024	33 (32–35)	1 (1–1)	870 (831–910)	9 (8–10)
Jordan	2025	35 (33–38)	1 (1–1)	922 (863–981)	9 (8–11)
Jordan	2026	37 (34–40)	1 (1–1)	973 (892–1055)	9 (7–12)
Jordan	2027	39 (35–43)	1 (1–1)	1025 (919–1131)	10 (7–12)
Jordan	2028	41 (36–46)	1 (1–1)	1076 (944–1209)	10 (6–13)
Jordan	2029	43 (36–49)	1 (0–1)	1128 (967–1289)	10 (6–14)
Jordan	2030	45 (37–52)	1 (0–1)	1179 (988–1370)	10 (5–14)
Jordan	2031	47 (38–55)	1 (0–1)	1231 (1008–1454)	10 (4–15)
Jordan	2032	49 (38–59)	1 (0–1)	1282 (1026–1538)	10 (4–16)
Jordan	2033	50 (39–62)	1 (0–1)	1334 (1043–1625)	10 (3–17)
Jordan	2034	52 (39–65)	1 (0–1)	1385 (1058–1713)	10 (2–18)
Jordan	2035	54 (40–69)	1 (0–1)	1437 (1071–1802)	10 (1–19)
Jordan	2036	56 (40–72)	1 (0–1)	1488 (1084–1893)	10 (0–20)
Kazakhstan	2022	194 (163–224)	1 (1–1)	5350 (4498–6202)	25 (19–31)
Kazakhstan	2023	179 (135–222)	1 (1–1)	5134 (3831–6438)	22 (14–30)
Kazakhstan	2024	164 (111–217)	1 (1–1)	4919 (3200–6637)	18 (8–28)
Kazakhstan	2025	149 (88–211)	1 (1–1)	4703 (2575–6831)	15 (3–26)
Kazakhstan	2026	134 (66–203)	1 (1–1)	4488 (1947–7029)	11 (-2–24)
Kazakhstan	2027	120 (44–195)	1 (0–1)	4272 (1310–7235)	8 (-7–22)

Kazakhstan	2028	105 (24–186)	1 (0–1)	4057 (662–7451)	4 (-11–20)
Kazakhstan	2029	90 (3–177)	1 (0–1)	3841 (3–7679)	1 (-16–17)
Kazakhstan	2030	75 (-17–168)	1 (0–1)	3626 (-668–7919)	-3 (-20–15)
Kazakhstan	2031	61 (-37–158)	1 (0–1)	3410 (-1352–8172)	-6 (-25–12)
Kazakhstan	2032	46 (-56–148)	1 (0–1)	3195 (-2048–8438)	-10 (-29–9)
Kazakhstan	2033	31 (-76–137)	1 (0–1)	2979 (-2757–8716)	-13 (-34–7)
Kazakhstan	2034	16 (-95–127)	1 (0–1)	2764 (-3479–9006)	-17 (-38–4)
Kazakhstan	2035	1 (-114–116)	1 (0–1)	2548 (-4213–9309)	-21 (-42–1)
Kazakhstan	2036	-13 (-132–106)	1 (0–1)	2333 (-4959–9624)	-24 (-47–-1)
Kenya	2022	301 (297–304)	0 (0–0)	8408 (8317–8499)	33 (32–34)
Kenya	2023	306 (300–313)	0 (0–0)	8578 (8398–8758)	33 (32–34)
Kenya	2024	312 (302–322)	0 (0–0)	8750 (8480–9020)	33 (31–35)
Kenya	2025	318 (305–331)	0 (0–0)	8922 (8565–9279)	33 (30–35)
Kenya	2026	324 (308–340)	0 (0–0)	9095 (8655–9534)	33 (30–36)
Kenya	2027	330 (311–349)	0 (0–0)	9267 (8750–9785)	33 (29–37)
Kenya	2028	336 (315–357)	0 (0–0)	9441 (8850–10032)	33 (29–37)
Kenya	2029	342 (318–366)	0 (0–0)	9614 (8954–10274)	33 (29–38)
Kenya	2030	348 (322–374)	0 (0–0)	9788 (9063–10512)	33 (29–38)
Kenya	2031	354 (326–382)	0 (0–0)	9961 (9176–10746)	34 (29–38)
Kenya	2032	360 (330–390)	0 (0–0)	10135 (9292–10977)	34 (29–39)
Kenya	2033	366 (334–398)	0 (0–0)	10309 (9411–11206)	34 (29–39)
Kenya	2034	372 (339–406)	0 (0–0)	10482 (9533–11431)	34 (29–39)
Kenya	2035	379 (343–414)	0 (0–0)	10656 (9657–11655)	34 (29–39)
Kenya	2036	385 (347–422)	0 (0–0)	10830 (9784–11876)	34 (29–39)
Kiribati	2022	2 (2–2)	0 (0–0)	48 (47–48)	57 (56–58)
Kiribati	2023	2 (2–2)	0 (0–0)	48 (48–49)	57 (55–59)
Kiribati	2024	2 (2–2)	0 (0–0)	49 (48–50)	57 (54–60)
Kiribati	2025	2 (2–2)	0 (0–0)	50 (49–51)	57 (53–61)
Kiribati	2026	2 (2–2)	0 (0–0)	51 (49–52)	56 (51–62)
Kiribati	2027	2 (2–2)	0 (0–0)	51 (50–53)	56 (49–63)
Kiribati	2028	2 (2–2)	0 (0–0)	52 (50–54)	56 (48–64)
Kiribati	2029	2 (2–2)	0 (0–0)	53 (51–55)	56 (46–65)
Kiribati	2030	2 (2–2)	0 (0–0)	53 (51–55)	55 (44–67)

Kiribati	2031	2 (2–2)	0 (0–0)	54 (52–56)	55 (42–68)
Kiribati	2032	2 (2–2)	0 (0–0)	55 (52–57)	55 (40–70)
Kiribati	2033	2 (2–2)	0 (0–0)	55 (53–58)	54 (38–71)
Kiribati	2034	2 (2–2)	0 (0–0)	56 (54–59)	54 (35–73)
Kiribati	2035	2 (2–2)	0 (0–0)	57 (54–60)	54 (33–75)
Kiribati	2036	2 (2–2)	0 (0–0)	58 (55–60)	54 (31–77)
Kuwait	2022	9 (7–11)	1 (0–1)	212 (174–251)	8 (4–11)
Kuwait	2023	9 (7–11)	1 (0–1)	212 (159–266)	8 (2–13)
Kuwait	2024	9 (6–12)	1 (0–1)	212 (146–279)	8 (1–14)
Kuwait	2025	9 (6–12)	1 (0–1)	212 (136–289)	8 (-0–15)
Kuwait	2026	9 (5–13)	1 (0–1)	212 (127–298)	8 (-1–16)
Kuwait	2027	9 (5–13)	1 (0–1)	212 (119–306)	8 (-2–17)
Kuwait	2028	9 (5–13)	1 (0–1)	212 (112–313)	8 (-3–18)
Kuwait	2029	9 (5–13)	1 (0–1)	212 (105–320)	8 (-3–19)
Kuwait	2030	9 (4–14)	1 (0–1)	212 (98–327)	8 (-4–19)
Kuwait	2031	9 (4–14)	1 (0–1)	212 (92–333)	8 (-5–20)
Kuwait	2032	9 (4–14)	1 (0–1)	212 (86–339)	8 (-5–20)
Kuwait	2033	9 (4–14)	1 (0–1)	212 (80–345)	8 (-6–21)
Kuwait	2034	9 (3–15)	1 (0–1)	212 (75–350)	8 (-7–22)
Kuwait	2035	9 (3–15)	1 (0–1)	212 (70–355)	8 (-7–22)
Kuwait	2036	9 (3–15)	1 (0–1)	212 (65–360)	8 (-8–23)
Kyrgyzstan	2022	56 (50–63)	1 (1–1)	1534 (1368–1700)	28 (23–33)
Kyrgyzstan	2023	55 (46–64)	1 (1–1)	1547 (1332–1761)	26 (19–33)
Kyrgyzstan	2024	53 (42–64)	1 (1–1)	1558 (1317–1799)	24 (15–33)
Kyrgyzstan	2025	52 (39–65)	1 (0–1)	1569 (1289–1849)	22 (12–32)
Kyrgyzstan	2026	50 (36–65)	1 (0–1)	1581 (1251–1910)	20 (9–31)
Kyrgyzstan	2027	49 (33–64)	1 (0–1)	1592 (1204–1980)	18 (6–30)
Kyrgyzstan	2028	47 (30–64)	1 (0–1)	1604 (1149–2059)	16 (3–29)
Kyrgyzstan	2029	46 (28–64)	1 (0–1)	1615 (1087–2143)	14 (-0–28)
Kyrgyzstan	2030	44 (25–63)	1 (0–1)	1626 (1018–2234)	12 (-3–27)
Kyrgyzstan	2031	43 (23–63)	1 (0–1)	1638 (945–2330)	10 (-6–26)
Kyrgyzstan	2032	41 (20–62)	1 (0–1)	1649 (867–2432)	8 (-9–25)
Kyrgyzstan	2033	40 (18–62)	1 (0–1)	1660 (784–2537)	6 (-12–24)

Kyrgyzstan	2034	38 (15–61)	1 (0–1)	1672 (696–2647)	4 (-14–22)
Kyrgyzstan	2035	37 (13–61)	1 (0–1)	1683 (605–2761)	2 (-17–21)
Kyrgyzstan	2036	35 (10–60)	1 (0–1)	1695 (510–2879)	-0 (-20–20)
Lao People's Democratic Republic	2022	43 (43–44)	1 (1–1)	1134 (1122–1147)	23 (22–23)
Lao People's Democratic Republic	2023	44 (43–45)	1 (1–1)	1154 (1131–1178)	23 (22–23)
Lao People's Democratic Republic	2024	45 (43–46)	1 (1–1)	1174 (1137–1212)	22 (21–23)
Lao People's Democratic Republic	2025	45 (43–47)	1 (1–1)	1194 (1141–1248)	22 (20–23)
Lao People's Democratic Republic	2026	46 (44–49)	1 (1–1)	1214 (1143–1286)	22 (20–24)
Lao People's Democratic Republic	2027	47 (44–50)	1 (1–1)	1234 (1143–1325)	21 (19–24)
Lao People's Democratic Republic	2028	48 (44–52)	1 (1–1)	1254 (1142–1366)	21 (18–24)
Lao People's Democratic Republic	2029	48 (43–53)	1 (1–1)	1274 (1139–1409)	21 (17–24)
Lao People's Democratic Republic	2030	49 (43–55)	1 (0–1)	1294 (1136–1452)	20 (16–24)
Lao People's Democratic Republic	2031	50 (43–57)	1 (0–1)	1314 (1131–1497)	20 (15–25)
Lao People's Democratic Republic	2032	51 (43–58)	1 (0–1)	1334 (1124–1544)	20 (14–25)
Lao People's Democratic Republic	2033	51 (43–60)	1 (0–1)	1354 (1117–1591)	19 (13–25)
Lao People's Democratic Republic	2034	52 (42–62)	1 (0–1)	1374 (1109–1639)	19 (12–26)
Lao People's Democratic Republic	2035	53 (42–64)	1 (0–1)	1394 (1099–1689)	19 (11–26)
Lao People's Democratic Republic	2036	54 (42–66)	1 (0–1)	1414 (1089–1739)	18 (10–27)
Latvia	2022	41 (36–45)	2 (1–2)	1056 (926–1186)	31 (27–35)
Latvia	2023	41 (34–47)	2 (1–2)	1056 (872–1240)	31 (26–37)
Latvia	2024	41 (33–48)	2 (1–2)	1056 (831–1282)	31 (25–38)
Latvia	2025	41 (32–50)	2 (1–2)	1056 (796–1317)	31 (24–39)
Latvia	2026	41 (31–51)	2 (1–2)	1056 (765–1347)	31 (23–40)
Latvia	2027	41 (30–52)	2 (1–2)	1056 (737–1375)	31 (22–40)
Latvia	2028	41 (29–53)	2 (1–2)	1056 (712–1401)	31 (21–41)
Latvia	2029	41 (28–53)	2 (1–2)	1056 (688–1424)	31 (21–42)
Latvia	2030	41 (27–54)	2 (1–2)	1056 (665–1447)	31 (20–42)
Latvia	2031	41 (26–55)	2 (1–2)	1056 (644–1468)	31 (19–43)
Latvia	2032	41 (26–56)	2 (1–2)	1056 (624–1488)	31 (19–44)
Latvia	2033	41 (25–56)	2 (1–2)	1056 (605–1507)	31 (18–44)
Latvia	2034	41 (24–57)	2 (1–2)	1056 (587–1526)	31 (18–45)
Latvia	2035	41 (24–58)	2 (1–2)	1056 (569–1543)	31 (17–45)
Latvia	2036	41 (23–58)	2 (1–2)	1056 (552–1560)	31 (17–46)

Lebanon	2022	31 (30–31)	1 (1–1)	648 (633–662)	11 (10–11)
Lebanon	2023	31 (30–32)	1 (1–1)	657 (628–686)	11 (10–11)
Lebanon	2024	31 (30–33)	1 (1–1)	666 (624–708)	11 (9–12)
Lebanon	2025	32 (29–34)	1 (1–1)	675 (621–730)	10 (9–12)
Lebanon	2026	32 (29–35)	1 (1–1)	684 (618–751)	10 (9–12)
Lebanon	2027	33 (29–36)	1 (1–1)	693 (616–771)	10 (8–12)
Lebanon	2028	33 (29–37)	1 (1–1)	702 (615–790)	10 (8–12)
Lebanon	2029	34 (29–38)	1 (1–1)	711 (614–808)	10 (8–12)
Lebanon	2030	34 (29–39)	1 (1–1)	720 (614–826)	10 (8–12)
Lebanon	2031	35 (29–40)	1 (1–1)	729 (615–843)	10 (7–12)
Lebanon	2032	35 (29–41)	1 (1–1)	737 (616–859)	9 (7–12)
Lebanon	2033	36 (29–42)	1 (1–1)	746 (617–875)	9 (7–12)
Lebanon	2034	36 (29–43)	1 (1–1)	755 (619–891)	9 (7–12)
Lebanon	2035	37 (29–44)	1 (1–1)	764 (621–907)	9 (6–12)
Lebanon	2036	37 (29–45)	1 (1–1)	773 (623–922)	9 (6–12)
Lesotho	2022	51 (49–52)	1 (1–1)	1451 (1408–1494)	127 (123–130)
Lesotho	2023	47 (43–51)	1 (1–1)	1389 (1277–1501)	121 (112–131)
Lesotho	2024	44 (37–51)	1 (1–1)	1327 (1128–1525)	116 (100–132)
Lesotho	2025	41 (31–52)	1 (1–1)	1264 (965–1564)	111 (87–136)
Lesotho	2026	38 (23–53)	1 (1–1)	1202 (789–1615)	106 (72–140)
Lesotho	2027	35 (16–54)	1 (1–1)	1140 (602–1678)	101 (57–145)
Lesotho	2028	32 (8–56)	1 (1–1)	1078 (404–1752)	96 (41–150)
Lesotho	2029	29 (-0–58)	1 (1–1)	1016 (197–1835)	91 (24–157)
Lesotho	2030	25 (-9–60)	1 (1–1)	954 (-20–1927)	86 (7–164)
Lesotho	2031	22 (-18–63)	1 (1–1)	891 (-245–2028)	80 (-12–173)
Lesotho	2032	19 (-27–66)	1 (1–1)	829 (-478–2137)	75 (-31–181)
Lesotho	2033	16 (-37–69)	1 (1–1)	767 (-719–2253)	70 (-50–191)
Lesotho	2034	13 (-46–72)	1 (1–1)	705 (-967–2377)	65 (-71–201)
Lesotho	2035	10 (-56–76)	1 (1–1)	643 (-1223–2509)	60 (-91–211)
Lesotho	2036	7 (-67–80)	1 (1–1)	581 (-1486–2647)	55 (-113–222)
Liberia	2022	12 (12–12)	0 (0–0)	361 (356–366)	14 (14–14)
Liberia	2023	12 (12–13)	0 (0–0)	378 (367–390)	14 (14–15)
Liberia	2024	13 (12–13)	0 (0–0)	396 (376–416)	14 (13–15)

Liberia	2025	13 (12–14)	0 (0–0)	413 (384–442)	14 (13–16)
Liberia	2026	14 (13–15)	0 (0–0)	431 (392–470)	15 (13–16)
Liberia	2027	14 (13–16)	0 (0–0)	448 (398–499)	15 (12–17)
Liberia	2028	15 (13–17)	0 (0–0)	466 (403–528)	15 (12–17)
Liberia	2029	15 (13–18)	0 (0–0)	483 (408–559)	15 (12–18)
Liberia	2030	16 (13–19)	0 (0–0)	501 (411–590)	15 (11–18)
Liberia	2031	16 (13–20)	0 (0–0)	518 (414–622)	15 (11–19)
Liberia	2032	17 (13–21)	0 (0–0)	536 (417–655)	15 (10–19)
Liberia	2033	17 (13–22)	0 (0–0)	553 (418–688)	15 (10–19)
Liberia	2034	18 (13–23)	0 (0–0)	571 (419–722)	15 (10–20)
Liberia	2035	18 (13–24)	0 (0–0)	588 (420–757)	15 (9–20)
Liberia	2036	19 (13–25)	0 (0–0)	606 (419–792)	15 (9–21)
Libya	2022	41 (40–42)	1 (1–1)	1126 (1101–1150)	19 (19–20)
Libya	2023	42 (40–45)	1 (1–1)	1155 (1101–1209)	19 (18–21)
Libya	2024	43 (39–47)	1 (1–1)	1184 (1093–1275)	19 (17–21)
Libya	2025	44 (38–50)	1 (1–1)	1214 (1080–1347)	19 (17–22)
Libya	2026	45 (36–53)	1 (1–1)	1243 (1062–1423)	19 (16–22)
Libya	2027	45 (35–56)	1 (1–1)	1272 (1040–1504)	19 (16–22)
Libya	2028	46 (33–60)	1 (1–1)	1301 (1014–1589)	19 (16–23)
Libya	2029	47 (31–63)	1 (1–1)	1331 (983–1678)	19 (15–23)
Libya	2030	48 (29–67)	1 (1–1)	1360 (949–1771)	19 (15–23)
Libya	2031	49 (27–71)	1 (1–1)	1389 (912–1867)	19 (15–24)
Libya	2032	50 (24–75)	1 (1–1)	1419 (871–1966)	19 (14–24)
Libya	2033	51 (22–79)	1 (1–1)	1448 (828–2068)	19 (14–24)
Libya	2034	52 (19–84)	1 (1–1)	1477 (781–2174)	19 (14–24)
Libya	2035	52 (16–88)	1 (1–1)	1507 (731–2282)	19 (14–25)
Libya	2036	53 (13–93)	1 (1–1)	1536 (679–2393)	19 (14–25)
Lithuania	2022	69 (64–75)	1 (1–1)	1780 (1616–1943)	36 (32–39)
Lithuania	2023	69 (61–78)	1 (1–1)	1780 (1548–2011)	36 (32–40)
Lithuania	2024	69 (59–79)	1 (1–2)	1780 (1496–2063)	36 (32–41)
Lithuania	2025	69 (58–81)	1 (1–2)	1780 (1452–2107)	36 (32–41)
Lithuania	2026	69 (56–82)	1 (1–2)	1780 (1413–2146)	36 (32–41)
Lithuania	2027	69 (55–84)	1 (1–2)	1780 (1378–2181)	36 (32–41)

Lithuania	2028	69 (54–85)	1 (1–2)	1780 (1346–2213)	36 (32–41)
Lithuania	2029	69 (53–86)	1 (1–2)	1780 (1316–2243)	36 (32–41)
Lithuania	2030	69 (52–87)	1 (1–2)	1780 (1288–2271)	36 (32–41)
Lithuania	2031	69 (51–88)	1 (1–2)	1780 (1262–2297)	37 (32–41)
Lithuania	2032	69 (50–89)	1 (1–2)	1780 (1236–2323)	37 (32–41)
Lithuania	2033	69 (49–90)	1 (1–2)	1780 (1212–2347)	37 (32–41)
Lithuania	2034	69 (48–90)	1 (1–2)	1780 (1189–2370)	37 (32–41)
Lithuania	2035	69 (48–91)	1 (1–2)	1780 (1167–2392)	37 (32–41)
Lithuania	2036	69 (47–92)	1 (1–2)	1780 (1145–2414)	37 (32–41)
Low SDI	2022	4607 (4558–4657)	0 (0–0)	128726 (127427–130025	23 (23–23)
Low SDI	2023	4690 (4600–4781)	0 (0–0)	131008 (128104–133912	23 (22–23)
Low SDI	2024	4774 (4636–4911)	0 (0–0)	133289 (128430–138149	22 (21–24)
Low SDI	2025	4857 (4668–5046)	0 (0–0)	135571 (128458–142684	22 (21–24)
Low SDI	2026	4940 (4695–5185)	0 (0–0)	137853 (128222–147484	22 (20–24)
Low SDI	2027	5023 (4718–5329)	0 (0–0)	140135 (127746–152523	22 (20–24)
Low SDI	2028	5107 (4736–5477)	0 (0–0)	142416 (127051–157782	21 (19–24)
Low SDI	2029	5190 (4751–5629)	0 (0–0)	144698 (126150–163246	21 (18–24)
Low SDI	2030	5273 (4761–5785)	0 (0–0)	146980 (125056–168903	21 (18–24)
Low SDI	2031	5356 (4768–5944)	0 (0–0)	149261 (123780–174743	20 (17–23)
Low SDI	2032	5440 (4772–6107)	0 (0–0)	151543 (122331–180755	20 (17–23)
Low SDI	2033	5523 (4772–6273)	0 (0–0)	153825 (120716–186934	20 (17–23)
Low SDI	2034	5606 (4770–6442)	0 (0–0)	156107 (118942–193271	20 (16–23)
Low SDI	2035	5689 (4764–6615)	0 (0–0)	158388 (117015–199762	19 (16–23)
Low SDI	2036	5773 (4755–6790)	0 (0–0)	160670 (114940–206400	19 (15–23)
Low-middle SDI	2022	16776 (16570–16982	0 (0–0)	448877 (443874–453879	29 (28–30)
Low-middle SDI	2023	16990 (16636–17343	0 (0–0)	454317 (445751–462883	29 (28–29)
Low-middle SDI	2024	17211 (16735–17686	0 (0–0)	459865 (448336–471393	28 (27–29)
Low-middle SDI	2025	17435 (16856–18014	0 (0–0)	465454 (451415–479493	28 (26–29)
Low-middle SDI	2026	17660 (16991–18329	0 (0–0)	471060 (454837–487283	27 (26–29)
Low-middle SDI	2027	17886 (17136–18635	0 (0–0)	476672 (458507–494837	27 (25–29)
Low-middle SDI	2028	18111 (17289–18934	0 (0–0)	482287 (462360–502214	27 (25–29)
Low-middle SDI	2029	18337 (17448–19226	0 (0–0)	487902 (466355–509450	26 (24–28)
Low-middle SDI	2030	18563 (17612–19515	0 (0–0)	493518 (470463–516573	26 (24–28)

Low-middle SDI	2031	[illegible]18789 (17779–19799[illegible]	0 (0–0)	[illegible]99134 (474664–523605[illegible]	26 (23–28)
Low-middle SDI	2032	[illegible]19015 (17950–20080[illegible]	0 (0–0)	[illegible]04751 (478942–530559[illegible]	25 (23–28)
Low-middle SDI	2033	[illegible]19241 (18124–20359[illegible]	0 (0–0)	[illegible]10367 (483286–537448[illegible]	25 (22–28)
Low-middle SDI	2034	[illegible]19467 (18299–20635[illegible]	0 (0–0)	[illegible]15983 (487687–544279[illegible]	25 (22–27)
Low-middle SDI	2035	[illegible]19693 (18477–20909[illegible]	0 (0–0)	[illegible]21599 (492139–551060[illegible]	24 (21–27)
Low-middle SDI	2036	[illegible]19919 (18657–21181[illegible]	0 (0–0)	[illegible]27216 (496634–557797[illegible]	24 (21–27)
Luxembourg	2022	13 (13–14)	1 (1–1)	304 (287–321)	28 (25–31)
Luxembourg	2023	13 (13–14)	1 (1–1)	305 (286–323)	27 (24–31)
Luxembourg	2024	13 (12–15)	1 (1–1)	305 (286–324)	27 (22–31)
Luxembourg	2025	13 (12–15)	1 (1–1)	305 (286–324)	26 (21–31)
Luxembourg	2026	13 (12–15)	1 (1–1)	305 (286–324)	25 (19–30)
Luxembourg	2027	13 (12–15)	1 (1–1)	305 (286–324)	24 (18–30)
Luxembourg	2028	13 (12–15)	1 (1–1)	305 (286–324)	23 (17–30)
Luxembourg	2029	13 (12–15)	1 (1–1)	305 (286–324)	22 (16–29)
Luxembourg	2030	13 (12–15)	1 (0–1)	305 (286–324)	21 (14–29)
Luxembourg	2031	13 (12–15)	1 (0–1)	305 (286–324)	21 (13–28)
Luxembourg	2032	13 (12–15)	1 (0–1)	305 (286–324)	20 (12–28)
Luxembourg	2033	13 (12–15)	1 (0–1)	305 (286–324)	19 (11–27)
Luxembourg	2034	13 (11–15)	1 (0–1)	305 (286–324)	18 (10–27)
Luxembourg	2035	13 (11–16)	1 (0–1)	305 (286–324)	17 (8–26)
Luxembourg	2036	13 (11–16)	1 (0–1)	305 (286–324)	16 (7–26)
Madagascar	2022	230 (224–235)	0 (0–0)	7535 (7359–7711)	50 (48–52)
Madagascar	2023	234 (225–243)	0 (0–0)	7675 (7393–7956)	49 (45–52)
Madagascar	2024	239 (226–251)	0 (0–0)	7815 (7429–8200)	47 (43–51)
Madagascar	2025	243 (227–259)	0 (0–0)	7955 (7462–8447)	46 (41–51)
Madagascar	2026	248 (228–267)	0 (0–0)	8095 (7490–8699)	45 (40–50)
Madagascar	2027	252 (228–276)	0 (0–0)	8235 (7513–8956)	44 (38–49)
Madagascar	2028	257 (229–284)	0 (0–0)	8375 (7530–9219)	42 (36–48)
Madagascar	2029	261 (229–293)	0 (0–0)	8514 (7542–9487)	41 (34–48)
Madagascar	2030	265 (229–302)	0 (0–0)	8654 (7549–9760)	40 (33–47)
Madagascar	2031	270 (229–311)	0 (0–0)	8794 (7551–10038)	38 (31–46)
Madagascar	2032	274 (228–321)	0 (0–0)	8934 (7548–10321)	37 (29–45)
Madagascar	2033	279 (228–330)	0 (0–0)	9074 (7540–10608)	36 (28–44)

Madagascar	2034	283 (227–340)	0 (0–0)	9214 (7528–10901)	35 (26–43)
Madagascar	2035	288 (226–350)	0 (0–0)	9354 (7511–11198)	33 (25–42)
Madagascar	2036	292 (225–360)	0 (0–0)	9494 (7490–11499)	32 (23–41)
Malawi	2022	323 (318–328)	0 (0–0)	9111 (8974–9247)	112 (109–115)
Malawi	2023	326 (314–337)	0 (0–0)	9233 (8923–9543)	110 (104–117)
Malawi	2024	329 (311–348)	0 (0–0)	9375 (8885–9865)	109 (98–120)
Malawi	2025	334 (310–359)	0 (0–0)	9533 (8876–10189)	107 (91–124)
Malawi	2026	340 (310–370)	0 (0–0)	9700 (8897–10503)	106 (84–128)
Malawi	2027	346 (311–381)	0 (0–0)	9872 (8942–10801)	105 (76–133)
Malawi	2028	352 (313–391)	0 (0–0)	10045 (9005–11086)	103 (67–139)
Malawi	2029	358 (316–401)	0 (0–0)	10219 (9080–11358)	102 (59–145)
Malawi	2030	364 (318–410)	0 (0–0)	10392 (9164–11621)	100 (49–151)
Malawi	2031	370 (321–419)	0 (0–0)	10565 (9254–11876)	99 (40–158)
Malawi	2032	376 (324–428)	0 (0–0)	10738 (9349–12126)	97 (30–165)
Malawi	2033	382 (328–437)	0 (0–0)	10910 (9448–12371)	96 (19–173)
Malawi	2034	388 (331–445)	0 (0–0)	11082 (9551–12613)	94 (8–181)
Malawi	2035	394 (334–454)	0 (0–0)	11254 (9657–12851)	93 (-3–189)
Malawi	2036	400 (338–462)	0 (0–0)	11426 (9765–13087)	92 (-14–198)
Malaysia	2022	212 (199–224)	0 (0–0)	5508 (5235–5781)	18 (17–20)
Malaysia	2023	219 (205–234)	0 (0–0)	5617 (5231–6003)	18 (16–21)
Malaysia	2024	222 (205–240)	0 (0–0)	5727 (5254–6199)	18 (16–21)
Malaysia	2025	227 (207–247)	0 (0–0)	5836 (5290–6382)	18 (15–21)
Malaysia	2026	231 (209–253)	0 (0–0)	5946 (5335–6556)	18 (15–22)
Malaysia	2027	236 (212–259)	0 (0–0)	6055 (5386–6724)	18 (15–22)
Malaysia	2028	240 (215–265)	0 (0–0)	6164 (5442–6887)	18 (14–22)
Malaysia	2029	244 (217–271)	0 (0–0)	6274 (5502–7046)	18 (14–23)
Malaysia	2030	248 (220–277)	0 (0–0)	6383 (5564–7202)	18 (14–23)
Malaysia	2031	253 (223–282)	0 (0–0)	6493 (5630–7356)	18 (14–23)
Malaysia	2032	257 (226–288)	0 (0–0)	6602 (5697–7507)	18 (13–23)
Malaysia	2033	261 (229–293)	0 (0–0)	6712 (5766–7657)	18 (13–24)
Malaysia	2034	265 (232–299)	0 (0–0)	6821 (5837–7805)	18 (13–24)
Malaysia	2035	270 (235–304)	0 (0–0)	6930 (5909–7952)	18 (13–24)
Malaysia	2036	274 (238–310)	0 (0–0)	7040 (5983–8097)	18 (13–24)

Maldives	2022	2 (2–2)	0 (0–0)	31 (24–37)	11 (10–12)
Maldives	2023	2 (2–2)	0 (0–0)	31 (24–37)	11 (9–13)
Maldives	2024	2 (2–2)	0 (0–0)	31 (24–37)	11 (8–14)
Maldives	2025	2 (2–2)	0 (0–0)	31 (24–37)	11 (7–15)
Maldives	2026	2 (2–2)	0 (0–0)	31 (24–37)	11 (6–16)
Maldives	2027	2 (2–2)	0 (0–0)	31 (24–37)	11 (5–17)
Maldives	2028	2 (2–2)	0 (0–0)	31 (24–37)	11 (4–18)
Maldives	2029	2 (2–3)	0 (0–0)	31 (24–37)	11 (2–20)
Maldives	2030	2 (2–3)	0 (0–0)	31 (24–37)	11 (1–21)
Maldives	2031	2 (2–3)	0 (0–0)	31 (24–37)	11 (-1–22)
Maldives	2032	2 (2–3)	0 (0–0)	31 (24–37)	11 (-2–24)
Maldives	2033	2 (2–3)	0 (0–0)	31 (24–37)	11 (-4–25)
Maldives	2034	2 (2–3)	0 (0–0)	31 (24–37)	11 (-5–27)
Maldives	2035	2 (2–3)	0 (0–0)	31 (24–37)	11 (-7–28)
Maldives	2036	2 (2–3)	0 (0–0)	31 (24–37)	11 (-9–30)
Mali	2022	29 (29–30)	0 (0–0)	811 (804–819)	8 (8–9)
Mali	2023	29 (29–30)	0 (0–0)	824 (808–840)	8 (8–9)
Mali	2024	30 (29–31)	0 (0–0)	837 (810–864)	8 (8–9)
Mali	2025	30 (28–32)	0 (0–0)	849 (810–889)	8 (8–9)
Mali	2026	30 (28–32)	0 (0–0)	862 (808–916)	8 (8–9)
Mali	2027	30 (28–33)	0 (0–0)	875 (805–944)	8 (7–9)
Mali	2028	31 (27–34)	0 (0–0)	887 (801–973)	8 (7–9)
Mali	2029	31 (27–35)	0 (0–0)	900 (796–1003)	8 (7–9)
Mali	2030	31 (26–36)	0 (0–0)	912 (790–1035)	8 (7–10)
Mali	2031	31 (26–37)	0 (0–0)	925 (783–1067)	8 (7–10)
Mali	2032	32 (25–38)	0 (0–0)	938 (775–1101)	8 (6–10)
Mali	2033	32 (24–39)	0 (0–0)	950 (765–1135)	8 (6–10)
Mali	2034	32 (24–40)	0 (0–0)	963 (755–1170)	8 (6–10)
Mali	2035	32 (23–41)	0 (0–0)	975 (744–1206)	8 (6–10)
Mali	2036	32 (22–43)	0 (0–0)	988 (733–1243)	8 (6–10)
Malta	2022	8 (8–9)	1 (1–1)	176 (164–188)	20 (19–22)
Malta	2023	8 (8–9)	1 (1–1)	176 (160–193)	20 (17–23)
Malta	2024	8 (8–9)	1 (1–1)	176 (156–196)	20 (16–23)

Malta	2025	8 (8–9)	1 (1–1)	176 (153–200)	19 (16–23)
Malta	2026	8 (8–9)	1 (1–1)	176 (150–202)	19 (15–23)
Malta	2027	8 (8–9)	1 (1–1)	176 (147–205)	18 (14–23)
Malta	2028	9 (8–9)	1 (1–1)	176 (145–207)	18 (13–23)
Malta	2029	9 (8–9)	1 (1–1)	176 (143–209)	18 (12–23)
Malta	2030	9 (8–9)	1 (1–1)	176 (141–211)	17 (12–23)
Malta	2031	9 (8–9)	1 (1–1)	176 (139–213)	17 (11–23)
Malta	2032	9 (8–9)	1 (1–1)	176 (137–215)	16 (10–22)
Malta	2033	9 (8–9)	1 (1–1)	176 (136–217)	16 (10–22)
Malta	2034	9 (8–10)	1 (1–1)	176 (134–218)	16 (9–22)
Malta	2035	9 (8–10)	1 (1–1)	176 (132–220)	15 (8–22)
Malta	2036	9 (8–10)	1 (1–1)	176 (131–222)	15 (8–22)
Marshall Islands	2022	0 (0–0)	0 (0–0)	6 (6–6)	15 (15–16)
Marshall Islands	2023	0 (0–0)	0 (0–0)	6 (6–7)	16 (15–16)
Marshall Islands	2024	0 (0–0)	0 (0–0)	7 (6–7)	16 (15–16)
Marshall Islands	2025	0 (0–0)	0 (0–0)	7 (6–7)	16 (15–17)
Marshall Islands	2026	0 (0–0)	0 (0–0)	7 (6–7)	16 (15–17)
Marshall Islands	2027	0 (0–0)	0 (0–0)	7 (6–7)	16 (15–17)
Marshall Islands	2028	0 (0–0)	0 (0–0)	7 (6–8)	16 (15–17)
Marshall Islands	2029	0 (0–0)	0 (0–0)	7 (6–8)	16 (15–17)
Marshall Islands	2030	0 (0–0)	0 (0–0)	7 (6–8)	16 (15–17)
Marshall Islands	2031	0 (0–0)	0 (0–0)	7 (6–8)	16 (15–17)
Marshall Islands	2032	0 (0–0)	0 (0–1)	7 (6–8)	16 (15–17)
Marshall Islands	2033	0 (0–0)	0 (0–1)	8 (6–9)	16 (15–17)
Marshall Islands	2034	0 (0–0)	0 (0–1)	8 (6–9)	16 (15–17)
Marshall Islands	2035	0 (0–0)	0 (0–1)	8 (6–9)	16 (15–17)
Marshall Islands	2036	0 (0–0)	0 (0–1)	8 (6–9)	16 (15–17)
Mauritania	2022	15 (14–15)	0 (0–0)	410 (401–418)	17 (17–18)
Mauritania	2023	15 (15–16)	0 (0–0)	432 (416–448)	18 (17–19)
Mauritania	2024	16 (15–17)	0 (0–0)	454 (428–479)	18 (17–20)
Mauritania	2025	17 (16–18)	0 (0–0)	476 (440–511)	19 (17–21)
Mauritania	2026	18 (16–19)	0 (0–0)	497 (450–544)	19 (17–22)
Mauritania	2027	19 (17–21)	0 (0–0)	519 (460–579)	20 (17–23)

Mauritania	2028	19 (17–22)	0 (0–0)	541 (468–614)	21 (16–25)
Mauritania	2029	20 (17–23)	0 (0–0)	563 (475–650)	21 (16–26)
Mauritania	2030	21 (17–24)	0 (0–0)	584 (482–687)	22 (16–28)
Mauritania	2031	22 (18–26)	0 (0–0)	606 (487–725)	22 (15–29)
Mauritania	2032	22 (18–27)	0 (0–0)	628 (492–764)	23 (15–31)
Mauritania	2033	23 (18–28)	0 (0–0)	650 (496–803)	23 (14–32)
Mauritania	2034	24 (18–30)	0 (0–0)	672 (500–843)	24 (14–34)
Mauritania	2035	25 (18–31)	0 (0–0)	693 (503–884)	24 (13–35)
Mauritania	2036	25 (18–32)	0 (0–0)	715 (505–925)	25 (13–37)
Mauritius	2022	20 (19–22)	1 (1–1)	518 (478–558)	27 (24–30)
Mauritius	2023	21 (18–23)	1 (1–1)	528 (455–602)	27 (20–34)
Mauritius	2024	21 (17–25)	1 (1–1)	538 (442–635)	27 (18–36)
Mauritius	2025	21 (17–26)	1 (1–1)	549 (434–663)	27 (16–38)
Mauritius	2026	22 (17–27)	1 (1–1)	559 (429–689)	27 (15–39)
Mauritius	2027	22 (17–28)	1 (1–1)	569 (425–713)	27 (13–41)
Mauritius	2028	23 (17–28)	1 (1–1)	579 (422–736)	27 (12–42)
Mauritius	2029	23 (17–29)	1 (1–1)	589 (421–758)	27 (11–43)
Mauritius	2030	23 (17–30)	1 (1–1)	600 (420–779)	27 (10–44)
Mauritius	2031	24 (17–31)	1 (1–1)	610 (420–800)	27 (9–45)
Mauritius	2032	24 (17–32)	1 (0–1)	620 (420–820)	27 (8–46)
Mauritius	2033	25 (17–33)	1 (0–1)	630 (421–839)	27 (7–47)
Mauritius	2034	25 (17–33)	1 (0–1)	640 (422–858)	27 (6–48)
Mauritius	2035	25 (17–34)	1 (0–1)	650 (424–877)	27 (6–48)
Mauritius	2036	26 (17–35)	1 (0–1)	661 (426–896)	27 (5–49)
Mexico	2022	263 (251–274)	0 (0–0)	6177 (5917–6436)	5 (4–5)
Mexico	2023	263 (246–279)	0 (0–0)	6177 (5810–6543)	4 (4–5)
Mexico	2024	263 (243–282)	0 (0–0)	6177 (5727–6626)	4 (3–5)
Mexico	2025	263 (240–285)	0 (0–0)	6177 (5658–6695)	4 (3–5)
Mexico	2026	263 (237–288)	0 (0–0)	6177 (5597–6756)	4 (3–5)
Mexico	2027	263 (235–291)	0 (0–0)	6177 (5541–6812)	4 (2–5)
Mexico	2028	263 (232–293)	0 (0–0)	6177 (5490–6863)	4 (2–5)
Mexico	2029	263 (230–295)	0 (0–0)	6177 (5443–6910)	3 (2–5)
Mexico	2030	263 (228–297)	0 (0–0)	6177 (5399–6954)	3 (1–5)

Mexico	2031	263 (226–299)	0 (0–0)	6177 (5357–6996)	3 (1–5)
Mexico	2032	263 (225–300)	0 (0–0)	6177 (5316–7037)	3 (0–6)
Mexico	2033	263 (223–302)	0 (0–0)	6177 (5278–7075)	3 (0–6)
Mexico	2034	263 (221–304)	0 (0–0)	6177 (5242–7111)	3 (-0–6)
Mexico	2035	263 (220–305)	0 (0–0)	6177 (5206–7147)	2 (-1–6)
Mexico	2036	263 (218–307)	0 (0–0)	6177 (5172–7181)	2 (-1–6)
Micronesia (Federated States of)	2022	1 (1–1)	1 (1–1)	19 (19–19)	21 (21–22)
Micronesia (Federated States of)	2023	1 (1–1)	1 (1–1)	19 (19–20)	21 (21–22)
Micronesia (Federated States of)	2024	1 (1–1)	1 (1–1)	20 (19–20)	21 (21–22)
Micronesia (Federated States of)	2025	1 (1–1)	1 (1–1)	20 (20–21)	21 (21–22)
Micronesia (Federated States of)	2026	1 (1–1)	1 (1–1)	20 (20–21)	21 (21–22)
Micronesia (Federated States of)	2027	1 (1–1)	1 (1–1)	21 (20–22)	21 (20–22)
Micronesia (Federated States of)	2028	1 (1–1)	1 (1–1)	21 (20–22)	21 (20–21)
Micronesia (Federated States of)	2029	1 (1–1)	1 (1–1)	21 (20–23)	21 (20–21)
Micronesia (Federated States of)	2030	1 (1–1)	1 (1–1)	22 (20–23)	21 (20–21)
Micronesia (Federated States of)	2031	1 (1–1)	1 (1–1)	22 (21–24)	20 (20–21)
Micronesia (Federated States of)	2032	1 (1–1)	1 (1–1)	22 (21–24)	20 (19–21)
Micronesia (Federated States of)	2033	1 (1–1)	1 (1–1)	23 (21–25)	20 (19–21)
Micronesia (Federated States of)	2034	1 (1–1)	1 (1–1)	23 (21–25)	20 (19–21)
Micronesia (Federated States of)	2035	1 (1–1)	1 (1–1)	23 (21–26)	20 (19–21)
Micronesia (Federated States of)	2036	1 (1–1)	1 (1–1)	24 (21–26)	20 (19–21)
Middle SDI	2022	38338 (86879–89797	1 (1–1)	60923 (2026837–20950	72 (70–74)
Middle SDI	2023	39444 (86097–92791	1 (1–1)	77156 (2006029–21482	70 (65–75)
Middle SDI	2024	90464 (85689–95239	1 (1–1)	93390 (1998752–21880	68 (61–74)
Middle SDI	2025	91484 (85619–97349	1 (1–1)	09623 (1996250–22229	66 (58–74)
Middle SDI	2026	92504 (85722–99285	1 (1–1)	25857 (1996432–22552	64 (54–73)
Middle SDI	2027	3524 (85935–10111	1 (1–1)	42090 (1998396–22857	62 (51–72)
Middle SDI	2028	4543 (86226–10286	1 (1–1)	58324 (2001654–23149	59 (48–71)
Middle SDI	2029	5563 (86576–10455	1 (1–1)	74557 (2005908–23432	57 (45–69)
Middle SDI	2030	6583 (86972–10619	1 (1–1)	90791 (2010958–23706	55 (42–68)
Middle SDI	2031	7603 (87407–10779	1 (1–1)	07024 (2016664–23973	53 (39–67)
Middle SDI	2032	8623 (87873–10937	1 (1–1)	23258 (2022922–24235	51 (37–65)
Middle SDI	2033	9643 (88366–11091	1 (1–1)	39491 (2029654–24493	49 (34–64)

Middle SDI	2034	)0663 (88883–11244	1 (1–1)	55725 (2036798–247465	47 (31–63)
Middle SDI	2035	)1682 (89421–11394	1 (1–1)	71958 (2044304–249961	45 (28–61)
Middle SDI	2036	)2702 (89977–11542	1 (1–1)	88192 (2052133–252425	43 (26–60)
Monaco	2022	2 (2–2)	2 (2–2)	38 (38–39)	45 (44–45)
Monaco	2023	2 (2–2)	2 (2–2)	39 (38–40)	45 (43–46)
Monaco	2024	2 (2–2)	2 (2–2)	39 (37–41)	45 (42–47)
Monaco	2025	2 (2–2)	2 (2–2)	40 (37–42)	45 (41–49)
Monaco	2026	2 (2–2)	2 (2–2)	40 (36–43)	45 (39–50)
Monaco	2027	2 (2–2)	2 (2–2)	40 (36–45)	45 (38–52)
Monaco	2028	2 (2–2)	2 (2–2)	41 (35–46)	45 (36–54)
Monaco	2029	2 (2–2)	2 (2–2)	41 (34–48)	45 (34–56)
Monaco	2030	2 (2–2)	2 (2–2)	41 (33–50)	45 (32–58)
Monaco	2031	2 (2–3)	2 (2–2)	42 (32–51)	45 (30–60)
Monaco	2032	2 (2–3)	2 (2–2)	42 (31–53)	45 (28–62)
Monaco	2033	2 (1–3)	2 (2–2)	42 (29–55)	45 (26–64)
Monaco	2034	2 (1–3)	2 (1–2)	43 (28–57)	45 (23–67)
Monaco	2035	2 (1–3)	2 (1–2)	43 (27–59)	45 (21–70)
Monaco	2036	2 (1–3)	2 (1–2)	43 (25–61)	45 (18–72)
Mongolia	2022	84 (82–87)	1 (1–1)	2260 (2188–2332)	91 (86–97)
Mongolia	2023	86 (82–89)	1 (1–1)	2294 (2192–2396)	89 (81–98)
Mongolia	2024	87 (82–91)	1 (1–1)	2327 (2203–2452)	88 (77–99)
Mongolia	2025	88 (82–93)	1 (1–1)	2361 (2217–2505)	86 (72–99)
Mongolia	2026	89 (83–95)	1 (1–1)	2395 (2233–2556)	84 (68–100)
Mongolia	2027	90 (83–97)	1 (1–1)	2428 (2252–2605)	82 (64–101)
Mongolia	2028	91 (84–98)	1 (1–1)	2462 (2271–2652)	81 (59–102)
Mongolia	2029	92 (85–100)	1 (1–2)	2495 (2291–2699)	79 (55–103)
Mongolia	2030	94 (85–102)	1 (1–2)	2529 (2313–2745)	77 (50–104)
Mongolia	2031	95 (86–103)	1 (0–2)	2562 (2334–2790)	75 (45–105)
Mongolia	2032	96 (87–105)	1 (0–2)	2596 (2357–2835)	74 (40–107)
Mongolia	2033	97 (88–106)	1 (0–2)	2629 (2380–2879)	72 (36–108)
Mongolia	2034	98 (88–108)	1 (0–2)	2663 (2403–2923)	70 (31–109)
Mongolia	2035	99 (89–109)	1 (0–2)	2697 (2427–2966)	68 (26–111)
Mongolia	2036	100 (90–111)	1 (0–2)	2730 (2451–3009)	67 (21–113)

Montenegro	2022	12 (11–13)	2 (2–2)	279 (263–294)	29 (27–31)
Montenegro	2023	12 (11–13)	2 (2–2)	279 (247–310)	30 (27–33)
Montenegro	2024	12 (11–13)	2 (2–2)	279 (237–320)	31 (27–34)
Montenegro	2025	12 (11–14)	2 (2–2)	279 (229–328)	31 (28–34)
Montenegro	2026	13 (11–14)	2 (2–2)	279 (222–335)	31 (28–35)
Montenegro	2027	13 (11–14)	2 (2–2)	279 (215–342)	32 (28–35)
Montenegro	2028	13 (11–15)	2 (2–2)	279 (210–347)	32 (28–35)
Montenegro	2029	13 (11–15)	2 (2–2)	279 (205–352)	32 (28–36)
Montenegro	2030	13 (11–15)	2 (2–2)	279 (200–357)	32 (28–36)
Montenegro	2031	13 (11–15)	2 (2–2)	279 (195–362)	32 (29–36)
Montenegro	2032	14 (11–16)	2 (2–2)	279 (191–366)	32 (29–36)
Montenegro	2033	14 (11–16)	2 (2–2)	279 (187–370)	32 (29–36)
Montenegro	2034	14 (11–16)	2 (2–2)	279 (183–374)	32 (29–36)
Montenegro	2035	14 (11–16)	2 (2–2)	279 (179–378)	32 (29–36)
Montenegro	2036	14 (12–17)	2 (2–2)	279 (175–382)	32 (29–36)
Morocco	2022	52 (51–53)	0 (0–0)	1334 (1317–1351)	4 (4–4)
Morocco	2023	53 (51–54)	0 (0–0)	1359 (1321–1397)	4 (3–4)
Morocco	2024	54 (51–56)	0 (0–0)	1384 (1320–1448)	4 (3–4)
Morocco	2025	55 (51–58)	0 (0–0)	1409 (1316–1503)	4 (3–4)
Morocco	2026	56 (51–60)	0 (0–0)	1435 (1308–1561)	4 (3–4)
Morocco	2027	57 (50–63)	0 (0–0)	1460 (1297–1623)	4 (3–4)
Morocco	2028	58 (50–65)	0 (0–0)	1485 (1283–1687)	3 (3–4)
Morocco	2029	59 (49–68)	0 (0–0)	1510 (1267–1754)	3 (3–4)
Morocco	2030	59 (49–70)	0 (0–0)	1536 (1247–1824)	3 (3–4)
Morocco	2031	60 (48–73)	0 (0–0)	1561 (1226–1896)	3 (2–5)
Morocco	2032	61 (47–76)	0 (0–0)	1586 (1202–1970)	3 (2–5)
Morocco	2033	62 (46–79)	0 (0–0)	1611 (1176–2047)	3 (2–5)
Morocco	2034	63 (45–82)	0 (0–0)	1637 (1148–2125)	3 (2–5)
Morocco	2035	64 (44–85)	0 (0–0)	1662 (1118–2206)	3 (2–5)
Morocco	2036	65 (42–88)	0 (0–0)	1687 (1086–2288)	3 (1–5)
Mozambique	2022	145 (143–147)	0 (0–0)	3989 (3945–4034)	33 (33–34)
Mozambique	2023	144 (140–148)	0 (0–0)	3973 (3876–4070)	32 (31–34)
Mozambique	2024	143 (136–149)	0 (0–0)	3958 (3798–4117)	31 (29–34)

Mozambique	2025	142 (133–151)	0 (0–0)	3943 (3714–4172)	31 (27–34)
Mozambique	2026	141 (129–153)	0 (0–0)	3929 (3625–4233)	30 (25–34)
Mozambique	2027	140 (125–155)	0 (0–0)	3916 (3533–4300)	29 (23–34)
Mozambique	2028	139 (121–158)	0 (0–0)	3904 (3437–4371)	28 (21–35)
Mozambique	2029	138 (116–160)	0 (0–0)	3892 (3339–4445)	27 (18–35)
Mozambique	2030	138 (112–163)	0 (0–0)	3881 (3239–4523)	26 (16–36)
Mozambique	2031	137 (108–165)	0 (0–0)	3871 (3138–4603)	25 (13–36)
Mozambique	2032	136 (104–168)	0 (0–0)	3861 (3036–4685)	24 (11–37)
Mozambique	2033	136 (100–171)	0 (0–0)	3851 (2933–4769)	23 (8–38)
Mozambique	2034	135 (96–174)	0 (0–0)	3842 (2830–4854)	22 (5–39)
Mozambique	2035	135 (92–177)	0 (0–0)	3834 (2727–4941)	21 (3–40)
Mozambique	2036	134 (88–180)	0 (0–0)	3826 (2623–5028)	20 (-0–41)
Myanmar	2022	385 (380–390)	0 (0–0)	10363 (10234–10492)	19 (19–20)
Myanmar	2023	390 (379–400)	0 (0–0)	10496 (10248–10744)	19 (18–20)
Myanmar	2024	395 (377–412)	0 (0–0)	10631 (10234–11029)	19 (17–20)
Myanmar	2025	399 (374–425)	0 (0–0)	10766 (10199–11333)	19 (17–21)
Myanmar	2026	404 (370–439)	0 (0–0)	10901 (10144–11657)	18 (16–21)
Myanmar	2027	409 (365–454)	0 (0–0)	11035 (10073–11998)	18 (15–22)
Myanmar	2028	414 (359–469)	0 (0–0)	11170 (9986–12354)	18 (14–22)
Myanmar	2029	419 (352–485)	0 (0–0)	11305 (9884–12725)	18 (12–23)
Myanmar	2030	424 (345–502)	0 (0–1)	11439 (9768–13110)	17 (11–24)
Myanmar	2031	428 (337–520)	0 (0–1)	11574 (9639–13508)	17 (10–24)
Myanmar	2032	433 (328–538)	0 (0–1)	11708 (9498–13919)	17 (9–25)
Myanmar	2033	438 (319–557)	0 (0–1)	11843 (9344–14342)	17 (7–26)
Myanmar	2034	443 (309–576)	0 (0–1)	11978 (9179–14776)	16 (6–27)
Myanmar	2035	448 (299–596)	0 (0–1)	12112 (9003–15221)	16 (5–28)
Myanmar	2036	453 (288–617)	0 (0–1)	12247 (8817–15677)	16 (3–29)
Namibia	2022	5 (5–5)	0 (0–0)	142 (139–146)	10 (9–10)
Namibia	2023	5 (5–6)	0 (0–0)	142 (135–150)	9 (9–10)
Namibia	2024	5 (5–6)	0 (0–0)	143 (131–155)	9 (8–11)
Namibia	2025	5 (5–6)	0 (0–0)	143 (126–160)	9 (7–11)
Namibia	2026	5 (4–6)	0 (0–0)	143 (122–165)	9 (6–12)
Namibia	2027	5 (4–6)	0 (0–0)	143 (117–170)	9 (5–12)

Namibia	2028	5 (4–6)	0 (0–0)	143 (112–175)	9 (4–13)
Namibia	2029	5 (4–6)	0 (0–0)	144 (108–180)	8 (3–14)
Namibia	2030	5 (4–7)	0 (0–0)	144 (103–184)	8 (2–15)
Namibia	2031	5 (4–7)	0 (0–0)	144 (98–189)	8 (1–15)
Namibia	2032	5 (3–7)	0 (0–0)	144 (94–194)	8 (-1–16)
Namibia	2033	5 (3–7)	0 (0–0)	144 (90–198)	8 (-2–17)
Namibia	2034	5 (3–7)	0 (0–0)	144 (85–203)	7 (-3–18)
Namibia	2035	5 (3–7)	0 (0–0)	144 (81–207)	7 (-5–19)
Namibia	2036	5 (3–8)	0 (0–0)	144 (77–211)	7 (-6–20)
Nauru	2022	0 (0–0)	1 (1–1)	1 (1–2)	22 (22–22)
Nauru	2023	0 (0–0)	1 (1–1)	2 (1–2)	22 (21–22)
Nauru	2024	0 (0–0)	1 (1–1)	2 (1–2)	22 (21–23)
Nauru	2025	0 (0–0)	1 (1–1)	2 (1–2)	22 (20–23)
Nauru	2026	0 (0–0)	1 (1–1)	2 (1–2)	22 (20–23)
Nauru	2027	0 (0–0)	1 (1–1)	2 (1–2)	21 (19–24)
Nauru	2028	0 (0–0)	1 (1–1)	2 (1–2)	21 (18–24)
Nauru	2029	0 (0–0)	1 (1–1)	2 (1–2)	21 (17–25)
Nauru	2030	0 (0–0)	1 (1–1)	2 (1–2)	21 (17–25)
Nauru	2031	0 (0–0)	1 (1–1)	2 (1–2)	21 (16–26)
Nauru	2032	0 (0–0)	1 (1–1)	2 (1–2)	21 (15–27)
Nauru	2033	0 (0–0)	1 (1–1)	2 (1–2)	21 (14–27)
Nauru	2034	0 (0–0)	1 (1–1)	2 (1–2)	20 (13–28)
Nauru	2035	0 (0–0)	1 (1–1)	2 (1–2)	20 (12–29)
Nauru	2036	0 (0–0)	1 (1–1)	2 (1–2)	20 (11–29)
Nepal	2022	398 (393–402)	0 (0–0)	10147 (10038–10255)	42 (41–42)
Nepal	2023	402 (393–412)	0 (0–0)	10245 (10023–10468)	41 (40–43)
Nepal	2024	407 (390–423)	0 (0–0)	10363 (9967–10758)	41 (38–44)
Nepal	2025	411 (387–435)	0 (0–0)	10458 (9866–11051)	41 (37–45)
Nepal	2026	416 (383–449)	0 (0–0)	10566 (9738–11394)	40 (35–46)
Nepal	2027	420 (378–462)	0 (0–0)	10662 (9576–11748)	40 (33–47)
Nepal	2028	425 (373–477)	0 (0–0)	10766 (9393–12138)	40 (31–49)
Nepal	2029	429 (367–492)	0 (0–0)	10863 (9183–12543)	40 (29–50)
Nepal	2030	434 (360–508)	0 (0–0)	10965 (8955–12974)	39 (27–52)

Nepal	2031	439 (352–525)	0 (0–0)	11063 (8705–13421)	39 (24–54)
Nepal	2032	443 (344–542)	0 (0–0)	11163 (8437–13890)	39 (22–56)
Nepal	2033	448 (335–560)	0 (0–0)	11262 (8151–14374)	38 (19–58)
Nepal	2034	452 (326–578)	0 (0–0)	11362 (7848–14877)	38 (16–60)
Nepal	2035	457 (316–597)	0 (0–0)	11462 (7529–15395)	38 (13–62)
Nepal	2036	461 (306–616)	0 (0–0)	11562 (7194–15929)	38 (10–65)
Netherlands	2022	889 (859–920)	1 (1–1)	17256 (16593–17919)	48 (46–51)
Netherlands	2023	891 (845–938)	1 (1–1)	17101 (16040–18162)	47 (42–51)
Netherlands	2024	893 (831–956)	1 (1–1)	16945 (15492–18398)	45 (39–51)
Netherlands	2025	896 (818–973)	1 (1–1)	16789 (14931–18648)	43 (36–51)
Netherlands	2026	898 (804–991)	1 (1–1)	16634 (14353–18915)	42 (32–51)
Netherlands	2027	900 (791–1009)	1 (1–1)	16478 (13755–19202)	40 (29–51)
Netherlands	2028	902 (776–1027)	1 (1–1)	16323 (13137–19509)	38 (25–51)
Netherlands	2029	904 (761–1047)	1 (1–1)	16167 (12499–19835)	37 (21–52)
Netherlands	2030	906 (746–1066)	1 (1–1)	16011 (11842–20181)	35 (18–52)
Netherlands	2031	908 (730–1086)	1 (1–1)	15856 (11165–20546)	33 (14–53)
Netherlands	2032	910 (713–1107)	1 (1–1)	15700 (10470–20930)	32 (10–54)
Netherlands	2033	912 (696–1128)	1 (1–1)	15545 (9758–21332)	30 (6–55)
Netherlands	2034	914 (679–1150)	1 (1–1)	15389 (9027–21751)	28 (1–56)
Netherlands	2035	917 (661–1172)	1 (1–1)	15233 (8279–22188)	27 (-3–56)
Netherlands	2036	919 (642–1195)	1 (1–1)	15078 (7515–22641)	25 (-7–58)
New Zealand	2022	73 (67–80)	1 (1–1)	1459 (1324–1595)	17 (14–19)
New Zealand	2023	72 (64–79)	1 (1–1)	1459 (1268–1651)	16 (12–20)
New Zealand	2024	71 (63–79)	1 (1–1)	1459 (1225–1694)	15 (11–20)
New Zealand	2025	71 (63–79)	1 (1–1)	1459 (1188–1730)	15 (10–20)
New Zealand	2026	71 (63–78)	1 (1–1)	1459 (1156–1762)	14 (8–20)
New Zealand	2027	71 (63–78)	1 (1–1)	1459 (1127–1791)	13 (7–19)
New Zealand	2028	70 (63–78)	1 (1–1)	1459 (1101–1818)	13 (6–19)
New Zealand	2029	70 (63–78)	1 (1–1)	1459 (1076–1842)	12 (5–19)
New Zealand	2030	70 (63–78)	1 (1–1)	1459 (1053–1866)	11 (4–19)
New Zealand	2031	70 (63–78)	1 (1–1)	1459 (1031–1888)	10 (2–18)
New Zealand	2032	70 (63–78)	1 (1–1)	1459 (1010–1908)	10 (1–18)
New Zealand	2033	70 (63–78)	1 (1–1)	1459 (990–1928)	9 (0–18)

New Zealand	2034	70 (63–78)	1 (1–1)	1459 (971–1948)	8 (-1–17)
New Zealand	2035	70 (63–78)	1 (1–1)	1459 (952–1966)	8 (-2–17)
New Zealand	2036	70 (63–78)	1 (1–1)	1459 (934–1984)	7 (-3–17)
Nicaragua	2022	8 (7–8)	0 (0–0)	187 (176–198)	4 (3–4)
Nicaragua	2023	8 (8–9)	0 (0–0)	190 (178–202)	4 (3–4)
Nicaragua	2024	8 (8–9)	0 (0–0)	193 (180–207)	4 (3–4)
Nicaragua	2025	8 (8–9)	0 (0–0)	197 (183–211)	4 (3–4)
Nicaragua	2026	8 (8–9)	0 (0–0)	200 (185–215)	3 (3–4)
Nicaragua	2027	9 (8–9)	0 (0–0)	204 (188–219)	3 (3–4)
Nicaragua	2028	9 (8–10)	0 (0–0)	207 (190–223)	3 (3–4)
Nicaragua	2029	9 (8–10)	0 (0–0)	210 (193–228)	3 (3–4)
Nicaragua	2030	9 (8–10)	0 (0–0)	214 (195–232)	3 (3–4)
Nicaragua	2031	9 (8–10)	0 (0–0)	217 (198–236)	3 (2–4)
Nicaragua	2032	9 (8–10)	0 (0–0)	220 (201–240)	3 (2–4)
Nicaragua	2033	9 (8–10)	0 (0–0)	224 (203–244)	3 (2–4)
Nicaragua	2034	10 (9–11)	0 (0–0)	227 (206–248)	3 (2–4)
Nicaragua	2035	10 (9–11)	0 (0–0)	230 (209–252)	3 (2–4)
Nicaragua	2036	10 (9–11)	0 (0–0)	234 (212–256)	3 (2–4)
Niger	2022	32 (32–33)	0 (0–0)	910 (896–923)	10 (10–10)
Niger	2023	33 (32–34)	0 (0–0)	931 (908–953)	10 (10–10)
Niger	2024	34 (33–35)	0 (0–0)	954 (918–991)	10 (9–10)
Niger	2025	35 (33–37)	0 (0–0)	976 (925–1027)	10 (9–11)
Niger	2026	36 (33–38)	0 (0–0)	999 (932–1067)	10 (9–11)
Niger	2027	37 (34–39)	0 (0–0)	1022 (936–1107)	10 (9–11)
Niger	2028	37 (34–41)	0 (0–0)	1044 (940–1149)	10 (8–11)
Niger	2029	38 (34–42)	0 (0–0)	1067 (942–1192)	10 (8–11)
Niger	2030	39 (34–44)	0 (0–0)	1089 (943–1236)	10 (8–12)
Niger	2031	40 (34–46)	0 (0–0)	1112 (942–1282)	10 (8–12)
Niger	2032	41 (34–47)	0 (0–0)	1135 (941–1328)	10 (7–12)
Niger	2033	42 (34–49)	0 (0–0)	1157 (939–1376)	10 (7–12)
Niger	2034	42 (34–51)	0 (0–0)	1180 (935–1424)	10 (7–13)
Niger	2035	43 (34–53)	0 (0–0)	1202 (931–1474)	10 (7–13)
Niger	2036	44 (34–54)	0 (0–0)	1225 (926–1524)	10 (6–13)

Nigeria	2022	209 (207–212)	0 (0–0)	6014 (5941–6087)	6 (6–6)
Nigeria	2023	216 (210–221)	0 (0–0)	6217 (6054–6380)	6 (6–6)
Nigeria	2024	222 (213–230)	0 (0–0)	6419 (6147–6692)	6 (5–6)
Nigeria	2025	228 (216–241)	0 (0–0)	6622 (6223–7021)	6 (5–6)
Nigeria	2026	234 (217–251)	0 (0–0)	6825 (6285–7365)	6 (5–6)
Nigeria	2027	241 (219–263)	0 (0–0)	7027 (6333–7722)	6 (5–6)
Nigeria	2028	247 (220–274)	0 (0–0)	7230 (6368–8091)	6 (5–7)
Nigeria	2029	253 (221–286)	0 (0–0)	7432 (6393–8472)	6 (5–7)
Nigeria	2030	260 (221–298)	0 (0–0)	7635 (6406–8864)	6 (5–7)
Nigeria	2031	266 (221–311)	0 (0–0)	7838 (6409–9266)	6 (5–7)
Nigeria	2032	272 (221–324)	0 (0–0)	8040 (6403–9678)	6 (4–7)
Nigeria	2033	279 (220–337)	0 (0–0)	8243 (6387–10099)	6 (4–7)
Nigeria	2034	285 (219–351)	0 (0–0)	8446 (6362–10529)	6 (4–7)
Nigeria	2035	291 (218–364)	0 (0–0)	8648 (6329–10968)	6 (4–7)
Nigeria	2036	298 (217–378)	0 (0–0)	8851 (6287–11415)	6 (4–7)
Niue	2022	0 (0–0)	1 (1–1)	0 (0–0)	15 (15–16)
Niue	2023	0 (0–0)	1 (1–1)	0 (0–0)	15 (15–16)
Niue	2024	0 (0–0)	1 (1–1)	0 (0–0)	15 (15–16)
Niue	2025	0 (0–0)	1 (1–1)	0 (0–0)	15 (15–16)
Niue	2026	0 (0–0)	1 (1–1)	0 (0–0)	16 (14–17)
Niue	2027	0 (0–0)	1 (1–1)	0 (0–0)	16 (14–17)
Niue	2028	0 (0–0)	1 (1–1)	0 (0–0)	16 (14–18)
Niue	2029	0 (0–0)	1 (1–1)	0 (0–0)	16 (13–18)
Niue	2030	0 (0–0)	1 (1–1)	0 (0–0)	16 (13–19)
Niue	2031	0 (0–0)	1 (1–1)	0 (0–0)	16 (12–19)
Niue	2032	0 (0–0)	1 (1–1)	0 (0–0)	16 (12–20)
Niue	2033	0 (0–0)	1 (1–1)	0 (0–0)	16 (12–20)
Niue	2034	0 (0–0)	1 (1–1)	0 (0–0)	16 (11–21)
Niue	2035	0 (0–0)	1 (1–1)	0 (0–0)	16 (11–21)
Niue	2036	0 (0–0)	1 (1–1)	0 (0–0)	16 (10–22)
North Macedonia	2022	23 (22–24)	2 (2–2)	585 (558–613)	17 (16–18)
North Macedonia	2023	23 (21–24)	2 (1–2)	570 (530–610)	16 (15–18)
North Macedonia	2024	22 (20–24)	2 (1–2)	554 (506–603)	16 (14–18)

North Macedonia	2025	21 (19–24)	2 (1–2)	542 (478–607)	15 (13–18)
North Macedonia	2026	21 (18–24)	1 (1–2)	530 (443–617)	15 (12–18)
North Macedonia	2027	20 (16–25)	1 (1–2)	516 (407–624)	14 (11–18)
North Macedonia	2028	20 (15–25)	1 (1–2)	502 (372–631)	14 (10–18)
North Macedonia	2029	19 (13–25)	1 (1–2)	489 (335–642)	13 (8–18)
North Macedonia	2030	19 (12–26)	1 (1–2)	476 (295–656)	13 (7–18)
North Macedonia	2031	18 (10–26)	1 (1–2)	462 (254–670)	12 (6–18)
North Macedonia	2032	18 (9–27)	1 (1–2)	448 (212–684)	12 (5–18)
North Macedonia	2033	17 (7–27)	1 (1–2)	435 (169–700)	11 (3–19)
North Macedonia	2034	17 (5–28)	1 (0–2)	421 (125–718)	10 (2–19)
North Macedonia	2035	16 (4–29)	1 (0–2)	408 (79–737)	10 (1–19)
North Macedonia	2036	16 (2–30)	1 (0–2)	394 (32–756)	9 (-1–20)
Northern Mariana Islands	2022	0 (0–0)	1 (1–1)	9 (9–10)	15 (14–17)
Northern Mariana Islands	2023	0 (0–0)	1 (1–1)	9 (8–10)	15 (13–17)
Northern Mariana Islands	2024	0 (0–0)	1 (1–1)	10 (8–11)	15 (12–18)
Northern Mariana Islands	2025	0 (0–0)	1 (1–1)	10 (8–11)	15 (11–19)
Northern Mariana Islands	2026	0 (0–0)	1 (1–1)	10 (8–12)	15 (10–20)
Northern Mariana Islands	2027	0 (0–0)	1 (1–1)	10 (8–12)	15 (10–20)
Northern Mariana Islands	2028	0 (0–0)	1 (1–1)	11 (8–13)	15 (9–21)
Northern Mariana Islands	2029	0 (0–0)	1 (1–1)	11 (8–13)	15 (8–22)
Northern Mariana Islands	2030	0 (0–0)	1 (0–1)	11 (8–14)	15 (8–22)
Northern Mariana Islands	2031	0 (0–0)	1 (0–1)	11 (9–14)	15 (7–23)
Northern Mariana Islands	2032	0 (0–0)	1 (0–1)	11 (9–14)	15 (7–23)
Northern Mariana Islands	2033	0 (0–0)	1 (0–1)	12 (9–15)	15 (6–23)
Northern Mariana Islands	2034	0 (0–1)	1 (0–1)	12 (9–15)	15 (6–24)
Northern Mariana Islands	2035	0 (0–1)	1 (0–1)	12 (9–15)	15 (6–24)
Northern Mariana Islands	2036	0 (0–1)	1 (0–1)	12 (9–16)	15 (5–25)
Norway	2022	80 (75–85)	1 (1–1)	1652 (1550–1755)	17 (16–19)
Norway	2023	79 (72–86)	1 (1–1)	1641 (1503–1779)	17 (15–19)
Norway	2024	79 (71–87)	1 (1–1)	1631 (1470–1792)	17 (14–19)
Norway	2025	78 (69–87)	1 (1–1)	1622 (1444–1800)	17 (14–19)
Norway	2026	78 (68–87)	1 (1–1)	1614 (1423–1804)	16 (13–19)
Norway	2027	77 (67–88)	1 (1–1)	1607 (1406–1807)	16 (13–19)

Norway	2028	77 (66–88)	1 (1–1)	1600 (1392–1808)	16 (12–19)
Norway	2029	77 (66–88)	1 (1–1)	1594 (1381–1807)	15 (12–19)
Norway	2030	76 (65–87)	1 (1–1)	1589 (1371–1807)	15 (11–19)
Norway	2031	76 (64–87)	1 (0–1)	1584 (1362–1806)	15 (11–19)
Norway	2032	76 (64–87)	1 (0–1)	1579 (1355–1804)	15 (10–19)
Norway	2033	75 (64–87)	1 (0–1)	1575 (1348–1803)	14 (10–19)
Norway	2034	75 (63–87)	1 (0–1)	1572 (1343–1801)	14 (9–19)
Norway	2035	75 (63–87)	1 (0–1)	1569 (1338–1799)	14 (9–19)
Norway	2036	75 (63–87)	1 (0–1)	1566 (1334–1798)	14 (9–19)
Oman	2022	6 (5–6)	0 (0–0)	154 (148–160)	7 (7–7)
Oman	2023	6 (5–6)	0 (0–0)	158 (148–167)	7 (6–8)
Oman	2024	6 (5–6)	0 (0–0)	161 (148–174)	7 (6–8)
Oman	2025	6 (5–7)	0 (0–0)	165 (148–181)	7 (6–8)
Oman	2026	6 (5–7)	0 (0–0)	168 (148–188)	7 (5–8)
Oman	2027	6 (5–7)	0 (0–0)	171 (148–195)	7 (5–8)
Oman	2028	6 (5–7)	0 (0–0)	175 (147–203)	6 (5–8)
Oman	2029	6 (5–8)	0 (0–0)	178 (146–210)	6 (5–8)
Oman	2030	6 (5–8)	0 (0–0)	182 (146–218)	6 (4–8)
Oman	2031	7 (5–8)	0 (0–0)	185 (145–225)	6 (4–8)
Oman	2032	7 (5–9)	0 (0–0)	188 (143–233)	6 (4–8)
Oman	2033	7 (5–9)	0 (0–0)	192 (142–242)	6 (4–8)
Oman	2034	7 (5–9)	0 (0–0)	195 (141–250)	6 (4–8)
Oman	2035	7 (5–9)	0 (0–0)	199 (139–258)	6 (3–8)
Oman	2036	7 (4–10)	0 (0–0)	202 (137–267)	6 (3–8)
Pakistan	2022	2339 (2313–2365)	0 (0–0)	64057 (63343–64771)	48 (47–49)
Pakistan	2023	2367 (2308–2426)	0 (0–0)	65071 (63475–66668)	47 (45–50)
Pakistan	2024	2394 (2296–2493)	0 (0–0)	66086 (63414–68758)	47 (43–50)
Pakistan	2025	2422 (2278–2566)	0 (0–0)	67100 (63189–71012)	46 (41–51)
Pakistan	2026	2450 (2254–2645)	0 (0–0)	68115 (62819–73411)	46 (39–53)
Pakistan	2027	2477 (2226–2729)	0 (0–0)	69130 (62317–75942)	45 (36–54)
Pakistan	2028	2505 (2193–2817)	0 (0–0)	70144 (61695–78593)	44 (33–55)
Pakistan	2029	2533 (2156–2909)	0 (0–0)	71159 (60959–81358)	44 (30–57)
Pakistan	2030	2560 (2115–3006)	0 (0–0)	72173 (60118–84229)	43 (27–59)

Pakistan	2031	2588 (2071–3105)	0 (0–0)	73188 (59176–87199)	43 (24–61)
Pakistan	2032	2616 (2023–3209)	0 (0–0)	74202 (58139–90265)	42 (21–63)
Pakistan	2033	2644 (1972–3316)	0 (0–0)	75217 (57011–93423)	41 (18–65)
Pakistan	2034	2671 (1917–3426)	0 (0–0)	76231 (55795–96667)	41 (14–67)
Pakistan	2035	2699 (1859–3539)	0 (0–0)	77246 (54495–99996)	40 (10–70)
Pakistan	2036	2727 (1798–3655)	0 (0–0)	78260 (53114–103406)	39 (7–72)
Palau	2022	0 (0–0)	1 (1–1)	5 (5–5)	20 (19–20)
Palau	2023	0 (0–0)	1 (1–1)	5 (5–5)	20 (19–20)
Palau	2024	0 (0–0)	1 (1–1)	5 (5–6)	20 (19–20)
Palau	2025	0 (0–0)	1 (1–1)	5 (5–6)	19 (19–20)
Palau	2026	0 (0–0)	1 (1–1)	6 (5–6)	19 (18–20)
Palau	2027	0 (0–0)	1 (1–1)	6 (5–6)	19 (18–20)
Palau	2028	0 (0–0)	1 (1–1)	6 (5–6)	19 (18–20)
Palau	2029	0 (0–0)	1 (1–1)	6 (5–6)	19 (18–20)
Palau	2030	0 (0–0)	1 (1–1)	6 (5–7)	19 (17–20)
Palau	2031	0 (0–0)	1 (1–1)	6 (5–7)	19 (17–20)
Palau	2032	0 (0–0)	1 (1–1)	6 (5–7)	19 (17–20)
Palau	2033	0 (0–0)	1 (1–1)	6 (5–7)	19 (17–20)
Palau	2034	0 (0–0)	1 (1–1)	6 (5–7)	19 (17–20)
Palau	2035	0 (0–0)	1 (1–1)	6 (5–7)	18 (17–20)
Palau	2036	0 (0–0)	1 (1–1)	6 (6–7)	18 (16–20)
Palestine	2022	9 (8–9)	1 (1–1)	208 (202–214)	8 (8–8)
Palestine	2023	9 (8–9)	1 (1–1)	214 (202–225)	8 (7–9)
Palestine	2024	9 (8–10)	1 (1–1)	219 (202–235)	8 (7–9)
Palestine	2025	9 (8–10)	1 (1–1)	223 (202–243)	8 (7–9)
Palestine	2026	9 (8–10)	1 (1–1)	227 (202–251)	8 (6–9)
Palestine	2027	9 (8–11)	1 (1–1)	230 (202–258)	7 (6–9)
Palestine	2028	10 (8–11)	1 (1–1)	234 (203–264)	7 (6–9)
Palestine	2029	10 (8–11)	1 (1–1)	237 (203–271)	7 (6–9)
Palestine	2030	10 (8–12)	1 (1–1)	240 (204–277)	7 (5–9)
Palestine	2031	10 (8–12)	1 (1–1)	244 (205–283)	7 (5–9)
Palestine	2032	10 (8–12)	1 (1–1)	247 (206–288)	7 (5–8)
Palestine	2033	10 (8–12)	1 (1–1)	250 (207–294)	7 (5–8)

Palestine	2034	10 (8–12)	1 (1–1)	254 (208–299)	6 (4–8)
Palestine	2035	11 (8–13)	1 (1–1)	257 (209–305)	6 (4–8)
Palestine	2036	11 (8–13)	1 (1–1)	260 (211–310)	6 (4–8)
Panama	2022	10 (9–10)	0 (0–0)	200 (189–211)	4 (4–5)
Panama	2023	10 (9–10)	0 (0–0)	202 (186–217)	4 (3–5)
Panama	2024	10 (9–11)	0 (0–0)	203 (184–222)	4 (3–5)
Panama	2025	10 (9–11)	0 (0–0)	205 (183–227)	4 (3–5)
Panama	2026	10 (9–11)	0 (0–0)	206 (182–231)	4 (3–5)
Panama	2027	10 (9–11)	0 (0–0)	208 (181–235)	3 (2–5)
Panama	2028	10 (9–12)	0 (0–0)	210 (181–238)	3 (2–4)
Panama	2029	10 (9–12)	0 (0–0)	211 (181–242)	3 (2–4)
Panama	2030	10 (9–12)	0 (0–0)	213 (180–245)	3 (1–4)
Panama	2031	11 (9–12)	0 (0–0)	214 (180–249)	3 (1–4)
Panama	2032	11 (9–12)	0 (0–0)	216 (180–252)	2 (1–4)
Panama	2033	11 (9–12)	0 (0–0)	218 (180–255)	2 (1–4)
Panama	2034	11 (9–13)	0 (0–0)	219 (180–258)	2 (0–4)
Panama	2035	11 (9–13)	0 (0–0)	221 (180–262)	2 (0–4)
Panama	2036	11 (9–13)	0 (0–0)	222 (180–265)	2 (-0–4)
Papua New Guinea	2022	16 (15–16)	0 (0–0)	501 (491–511)	8 (7–8)
Papua New Guinea	2023	16 (16–17)	0 (0–0)	517 (500–534)	8 (7–8)
Papua New Guinea	2024	17 (16–17)	0 (0–0)	534 (509–558)	7 (7–8)
Papua New Guinea	2025	17 (16–18)	0 (0–0)	550 (516–583)	7 (7–8)
Papua New Guinea	2026	17 (16–19)	0 (0–0)	566 (523–609)	7 (7–8)
Papua New Guinea	2027	18 (16–20)	0 (0–0)	583 (530–635)	7 (6–8)
Papua New Guinea	2028	18 (16–21)	0 (0–0)	599 (535–662)	7 (6–8)
Papua New Guinea	2029	19 (16–21)	0 (0–0)	615 (541–690)	7 (6–8)
Papua New Guinea	2030	19 (17–22)	0 (0–0)	631 (545–718)	7 (6–8)
Papua New Guinea	2031	20 (17–23)	0 (0–0)	648 (549–747)	7 (6–8)
Papua New Guinea	2032	20 (17–24)	0 (0–0)	664 (553–776)	7 (6–8)
Papua New Guinea	2033	21 (17–25)	0 (0–0)	680 (555–805)	7 (5–8)
Papua New Guinea	2034	21 (17–26)	0 (0–0)	697 (558–836)	7 (5–8)
Papua New Guinea	2035	22 (17–27)	0 (0–0)	713 (560–866)	6 (5–8)
Papua New Guinea	2036	22 (17–28)	0 (0–0)	729 (561–897)	6 (5–8)

Paraguay	2022	81 (78–84)	1 (1–1)	1969 (1901–2037)	33 (31–35)
Paraguay	2023	83 (79–87)	1 (1–1)	2006 (1910–2102)	33 (30–35)
Paraguay	2024	84 (79–90)	1 (1–1)	2043 (1926–2161)	33 (29–36)
Paraguay	2025	86 (80–92)	1 (1–1)	2080 (1945–2216)	33 (29–36)
Paraguay	2026	88 (81–94)	1 (1–1)	2117 (1966–2269)	33 (28–37)
Paraguay	2027	89 (82–96)	1 (1–1)	2154 (1988–2321)	33 (28–37)
Paraguay	2028	91 (83–98)	1 (1–1)	2191 (2012–2371)	33 (28–38)
Paraguay	2029	92 (84–100)	1 (1–1)	2228 (2036–2420)	33 (27–38)
Paraguay	2030	94 (85–102)	1 (0–1)	2265 (2062–2469)	33 (27–38)
Paraguay	2031	95 (86–104)	1 (0–1)	2302 (2088–2517)	33 (27–39)
Paraguay	2032	97 (87–106)	1 (0–1)	2339 (2114–2564)	33 (26–39)
Paraguay	2033	98 (88–108)	1 (0–1)	2376 (2141–2611)	33 (26–39)
Paraguay	2034	100 (89–110)	1 (0–1)	2413 (2169–2658)	33 (26–39)
Paraguay	2035	101 (90–112)	1 (0–1)	2450 (2196–2704)	33 (26–40)
Paraguay	2036	103 (91–114)	1 (0–1)	2487 (2225–2750)	33 (25–40)
Peru	2022	46 (43–50)	0 (0–0)	997 (925–1069)	3 (3–3)
Peru	2023	46 (40–52)	0 (0–0)	994 (869–1119)	3 (3–3)
Peru	2024	46 (38–54)	0 (0–0)	993 (822–1164)	3 (2–4)
Peru	2025	46 (36–56)	0 (0–0)	992 (783–1202)	3 (2–4)
Peru	2026	46 (34–57)	0 (0–0)	992 (749–1236)	3 (2–4)
Peru	2027	46 (33–59)	0 (0–0)	992 (718–1266)	3 (2–4)
Peru	2028	46 (32–60)	0 (0–0)	992 (691–1293)	3 (2–4)
Peru	2029	46 (30–61)	0 (0–0)	992 (666–1318)	3 (2–4)
Peru	2030	46 (29–63)	0 (0–0)	992 (642–1342)	3 (2–4)
Peru	2031	46 (28–64)	0 (0–0)	992 (620–1364)	3 (2–4)
Peru	2032	46 (27–65)	0 (0–0)	992 (600–1385)	3 (2–4)
Peru	2033	46 (26–66)	0 (0–0)	992 (580–1404)	3 (2–4)
Peru	2034	46 (25–66)	0 (0–0)	992 (561–1423)	3 (2–4)
Peru	2035	46 (25–67)	0 (0–0)	992 (543–1441)	3 (2–4)
Peru	2036	46 (24–68)	0 (0–0)	992 (526–1458)	3 (2–4)
Philippines	2022	379 (373–386)	1 (1–1)	10463 (10270–10656)	11 (11–12)
Philippines	2023	386 (377–395)	1 (1–1)	10642 (10369–10914)	11 (11–12)
Philippines	2024	393 (382–404)	1 (1–1)	10820 (10486–11155)	11 (10–12)

Philippines	2025	400 (387–412)	1 (1–1)	10999 (10613–11385)	11 (10–12)
Philippines	2026	406 (392–421)	1 (1–1)	11178 (10746–11609)	11 (9–12)
Philippines	2027	413 (397–429)	1 (1–1)	11356 (10884–11829)	11 (9–12)
Philippines	2028	420 (403–437)	1 (1–1)	11535 (11025–12046)	10 (9–12)
Philippines	2029	426 (408–444)	1 (1–1)	11714 (11168–12260)	10 (8–12)
Philippines	2030	433 (414–452)	1 (0–1)	11893 (11314–12471)	10 (8–13)
Philippines	2031	440 (420–460)	1 (0–1)	12071 (11461–12682)	10 (7–13)
Philippines	2032	447 (425–468)	1 (0–1)	12250 (11610–12890)	10 (7–13)
Philippines	2033	453 (431–475)	1 (1–1)	12429 (11760–13097)	10 (6–13)
Philippines	2034	460 (437–483)	1 (0–1)	12607 (11912–13303)	10 (6–13)
Philippines	2035	467 (443–491)	1 (0–1)	12786 (12064–13508)	9 (5–13)
Philippines	2036	473 (449–498)	1 (0–1)	12965 (12218–13712)	9 (5–14)
Poland	2022	831 (794–867)	1 (1–1)	20720 (19698–21742)	30 (28–32)
Poland	2023	831 (779–882)	1 (1–1)	20829 (19467–22191)	30 (27–33)
Poland	2024	831 (767–894)	1 (1–1)	20925 (19350–22501)	29 (26–33)
Poland	2025	831 (758–904)	1 (1–1)	21010 (19287–22734)	29 (25–33)
Poland	2026	831 (749–912)	1 (1–1)	21085 (19255–22916)	28 (24–33)
Poland	2027	831 (741–920)	1 (1–1)	21151 (19242–23060)	28 (23–32)
Poland	2028	831 (734–927)	1 (1–1)	21209 (19241–23177)	27 (22–32)
Poland	2029	831 (727–934)	1 (1–1)	21260 (19248–23273)	27 (21–32)
Poland	2030	831 (721–940)	1 (1–1)	21305 (19259–23352)	26 (20–32)
Poland	2031	831 (715–946)	1 (1–1)	21345 (19273–23417)	26 (19–32)
Poland	2032	831 (709–952)	1 (1–1)	21380 (19288–23472)	25 (18–32)
Poland	2033	831 (704–957)	1 (1–1)	21411 (19304–23518)	24 (18–31)
Poland	2034	831 (699–962)	1 (1–2)	21438 (19319–23557)	24 (17–31)
Poland	2035	831 (694–967)	1 (1–2)	21462 (19334–23590)	23 (16–31)
Poland	2036	831 (689–972)	1 (0–2)	21483 (19348–23618)	23 (15–30)
Portugal	2022	204 (194–215)	1 (1–1)	5086 (4769–5402)	25 (23–27)
Portugal	2023	204 (190–219)	1 (1–1)	5086 (4638–5533)	25 (22–28)
Portugal	2024	204 (186–222)	1 (1–1)	5086 (4538–5634)	24 (21–28)
Portugal	2025	204 (183–225)	1 (1–1)	5086 (4453–5718)	24 (20–28)
Portugal	2026	204 (181–228)	1 (1–1)	5086 (4378–5793)	23 (19–28)
Portugal	2027	204 (179–230)	1 (1–1)	5086 (4311–5861)	23 (18–28)

Portugal	2028	204 (177–232)	1 (1–1)	5086 (4249–5923)	22 (17–27)
Portugal	2029	204 (175–234)	1 (1–1)	5086 (4191–5980)	21 (16–27)
Portugal	2030	204 (173–236)	1 (1–1)	5086 (4137–6035)	21 (15–27)
Portugal	2031	204 (171–237)	1 (1–1)	5086 (4085–6086)	20 (14–27)
Portugal	2032	204 (170–239)	1 (1–1)	5086 (4037–6135)	20 (13–26)
Portugal	2033	204 (168–240)	1 (1–1)	5086 (3990–6182)	19 (12–26)
Portugal	2034	204 (167–242)	1 (1–1)	5086 (3945–6226)	19 (11–26)
Portugal	2035	204 (165–243)	1 (1–1)	5086 (3902–6269)	18 (10–26)
Portugal	2036	204 (164–245)	1 (1–1)	5086 (3861–6311)	17 (10–25)
Puerto Rico	2022	37 (32–42)	1 (1–1)	779 (662–896)	11 (9–14)
Puerto Rico	2023	36 (30–42)	1 (1–1)	760 (618–902)	11 (8–13)
Puerto Rico	2024	35 (28–43)	1 (1–1)	741 (579–904)	10 (7–13)
Puerto Rico	2025	35 (27–43)	1 (1–1)	723 (541–904)	9 (6–12)
Puerto Rico	2026	34 (25–43)	1 (0–1)	704 (506–902)	8 (5–12)
Puerto Rico	2027	33 (24–43)	1 (0–1)	685 (472–899)	7 (3–11)
Puerto Rico	2028	32 (22–42)	1 (0–1)	667 (439–895)	7 (2–11)
Puerto Rico	2029	32 (21–42)	1 (0–1)	648 (406–890)	6 (1–10)
Puerto Rico	2030	31 (20–42)	1 (0–1)	629 (375–884)	5 (0–9)
Puerto Rico	2031	30 (18–42)	1 (0–1)	611 (344–877)	4 (-1–9)
Puerto Rico	2032	29 (17–42)	1 (0–1)	592 (314–870)	3 (-2–8)
Puerto Rico	2033	29 (16–41)	1 (0–1)	573 (284–863)	2 (-3–8)
Puerto Rico	2034	28 (15–41)	1 (0–1)	555 (254–855)	2 (-4–7)
Puerto Rico	2035	27 (14–41)	1 (0–1)	536 (225–847)	1 (-5–6)
Puerto Rico	2036	27 (12–41)	1 (0–1)	517 (196–838)	-0 (-6–6)
Qatar	2022	5 (5–5)	1 (0–1)	148 (143–153)	14 (10–18)
Qatar	2023	5 (5–5)	1 (0–1)	151 (143–160)	14 (6–22)
Qatar	2024	5 (5–6)	1 (0–1)	155 (143–166)	14 (3–24)
Qatar	2025	5 (5–6)	1 (0–1)	158 (145–172)	14 (1–26)
Qatar	2026	5 (5–6)	1 (0–1)	162 (146–177)	14 (-1–28)
Qatar	2027	5 (5–6)	1 (0–1)	165 (148–182)	14 (-2–30)
Qatar	2028	6 (5–6)	1 (0–1)	169 (150–187)	14 (-4–31)
Qatar	2029	6 (5–6)	1 (0–1)	172 (152–192)	14 (-5–33)
Qatar	2030	6 (5–7)	1 (0–1)	175 (154–197)	14 (-6–34)

Qatar	2031	6 (5–7)	1 (0–1)	179 (156–201)	14 (-8–35)
Qatar	2032	6 (5–7)	1 (0–1)	182 (158–206)	14 (-9–36)
Qatar	2033	6 (5–7)	1 (0–1)	186 (161–211)	14 (-10–37)
Qatar	2034	6 (5–7)	1 (0–1)	189 (163–215)	14 (-11–38)
Qatar	2035	6 (5–7)	1 (0–1)	193 (166–220)	14 (-12–39)
Qatar	2036	6 (5–7)	1 (0–1)	196 (168–224)	14 (-13–40)
Republic of Korea	2022	1169 (1139–1198)	1 (1–1)	23803 (23065–24542)	24 (22–26)
Republic of Korea	2023	1197 (1145–1250)	1 (1–1)	24208 (22892–25523)	24 (20–27)
Republic of Korea	2024	1224 (1146–1301)	1 (1–1)	24566 (22646–26487)	23 (19–28)
Republic of Korea	2025	1248 (1144–1351)	1 (1–1)	24884 (22336–27433)	23 (16–30)
Republic of Korea	2026	1270 (1139–1401)	1 (1–1)	25167 (21976–28357)	23 (14–32)
Republic of Korea	2027	1290 (1132–1449)	1 (1–1)	25417 (21577–29258)	23 (12–34)
Republic of Korea	2028	1309 (1122–1497)	1 (1–1)	25640 (21148–30131)	22 (9–36)
Republic of Korea	2029	1327 (1110–1543)	1 (1–1)	25837 (20697–30978)	22 (7–38)
Republic of Korea	2030	1343 (1097–1588)	1 (1–1)	26013 (20228–31797)	22 (4–40)
Republic of Korea	2031	1357 (1082–1632)	1 (1–1)	26168 (19747–32589)	22 (1–43)
Republic of Korea	2032	1371 (1066–1675)	1 (1–1)	26306 (19257–33355)	21 (-2–45)
Republic of Korea	2033	1383 (1049–1717)	1 (1–1)	26429 (18763–34094)	21 (-6–48)
Republic of Korea	2034	1394 (1032–1757)	1 (1–2)	26537 (18266–34809)	21 (-9–51)
Republic of Korea	2035	1405 (1013–1797)	1 (1–2)	26634 (17768–35499)	21 (-12–53)
Republic of Korea	2036	1415 (994–1835)	1 (1–2)	26719 (17272–36167)	20 (-16–56)
Republic of Moldova	2022	61 (55–66)	1 (1–1)	1652 (1485–1818)	27 (24–31)
Republic of Moldova	2023	61 (53–68)	1 (1–1)	1652 (1416–1887)	27 (23–32)
Republic of Moldova	2024	61 (51–70)	1 (1–1)	1652 (1363–1940)	27 (22–33)
Republic of Moldova	2025	61 (50–72)	1 (1–1)	1652 (1318–1985)	27 (21–34)
Republic of Moldova	2026	61 (49–73)	1 (1–1)	1652 (1279–2024)	27 (20–35)
Republic of Moldova	2027	61 (47–74)	1 (1–1)	1652 (1243–2060)	27 (20–35)
Republic of Moldova	2028	61 (46–75)	1 (1–2)	1652 (1211–2092)	27 (19–36)
Republic of Moldova	2029	61 (45–76)	1 (1–2)	1652 (1180–2123)	27 (18–36)
Republic of Moldova	2030	61 (44–77)	1 (1–2)	1652 (1152–2151)	27 (18–37)
Republic of Moldova	2031	61 (43–78)	1 (1–2)	1652 (1125–2178)	27 (17–38)
Republic of Moldova	2032	61 (43–79)	1 (1–2)	1652 (1099–2204)	27 (17–38)
Republic of Moldova	2033	61 (42–80)	1 (1–2)	1652 (1074–2229)	27 (16–38)

Republic of Moldova	2034	61 (41–80)	1 (1–2)	1652 (1051–2252)	27 (16–39)
Republic of Moldova	2035	61 (40–81)	1 (1–2)	1652 (1028–2275)	27 (16–39)
Republic of Moldova	2036	61 (40–82)	1 (1–2)	1652 (1006–2297)	27 (15–40)
Romania	2022	320 (300–340)	1 (1–2)	8741 (8155–9327)	27 (25–29)
Romania	2023	321 (290–352)	1 (1–2)	8746 (7838–9653)	27 (24–30)
Romania	2024	322 (281–363)	1 (1–2)	8750 (7540–9961)	27 (23–31)
Romania	2025	324 (273–374)	1 (1–2)	8755 (7242–10268)	27 (22–32)
Romania	2026	325 (264–386)	1 (1–2)	8760 (6937–10583)	27 (21–33)
Romania	2027	326 (255–398)	1 (1–2)	8765 (6623–10906)	27 (20–34)
Romania	2028	327 (245–409)	1 (1–2)	8769 (6299–11239)	27 (18–35)
Romania	2029	329 (236–422)	1 (1–2)	8774 (5964–11584)	27 (17–36)
Romania	2030	330 (225–434)	1 (1–2)	8779 (5618–11939)	27 (16–37)
Romania	2031	331 (215–447)	1 (1–2)	8783 (5262–12305)	27 (15–39)
Romania	2032	333 (204–461)	1 (1–2)	8788 (4894–12683)	27 (13–40)
Romania	2033	334 (193–474)	1 (1–2)	8793 (4515–13070)	27 (12–41)
Romania	2034	335 (182–488)	1 (1–2)	8798 (4126–13469)	26 (10–43)
Romania	2035	336 (170–503)	1 (1–2)	8802 (3727–13878)	26 (9–44)
Romania	2036	338 (158–517)	1 (1–2)	8807 (3317–14297)	26 (7–45)
Russian Federation	2022	3147 (2910–3385)	1 (1–1)	85156 (78062–92250)	36 (32–39)
Russian Federation	2023	3147 (2811–3483)	1 (1–1)	85156 (75123–95188)	36 (30–41)
Russian Federation	2024	3147 (2736–3559)	1 (1–1)	85156 (72869–97443)	36 (28–43)
Russian Federation	2025	3147 (2672–3622)	1 (1–1)	85156 (70968–99344)	36 (27–44)
Russian Federation	2026	3147 (2616–3678)	1 (1–1)	85156 (69293–101018)	36 (26–46)
Russian Federation	2027	3147 (2565–3729)	1 (1–1)	85156 (67779–102532)	36 (25–47)
Russian Federation	2028	3147 (2519–3776)	1 (1–1)	85156 (66387–103925)	36 (24–48)
Russian Federation	2029	3147 (2475–3819)	1 (1–1)	85156 (65091–105220)	36 (23–49)
Russian Federation	2030	3147 (2435–3860)	1 (1–1)	85156 (63874–106438)	36 (22–49)
Russian Federation	2031	3147 (2396–3898)	1 (1–1)	85156 (62723–107589)	36 (21–50)
Russian Federation	2032	3147 (2359–3935)	1 (1–1)	85156 (61628–108684)	36 (20–51)
Russian Federation	2033	3147 (2324–3970)	1 (1–1)	85156 (60582–109730)	36 (20–52)
Russian Federation	2034	3147 (2291–4004)	1 (1–1)	85156 (59578–110733)	36 (19–52)
Russian Federation	2035	3147 (2258–4036)	1 (1–1)	85156 (58613–111699)	36 (18–53)
Russian Federation	2036	3147 (2227–4067)	1 (1–1)	85156 (57681–112630)	36 (18–54)

Rwanda	2022	166 (158–174)	0 (0–0)	4294 (4055–4534)	66 (64–69)
Rwanda	2023	162 (143–181)	0 (0–0)	4177 (3616–4737)	66 (60–73)
Rwanda	2024	158 (130–185)	0 (0–0)	4030 (3254–4807)	66 (55–78)
Rwanda	2025	153 (120–186)	0 (0–0)	3894 (2983–4804)	66 (49–83)
Rwanda	2026	148 (112–185)	0 (0–1)	3779 (2791–4767)	66 (43–88)
Rwanda	2027	145 (105–184)	0 (0–1)	3690 (2659–4721)	66 (38–94)
Rwanda	2028	141 (100–182)	0 (0–1)	3623 (2568–4677)	66 (32–99)
Rwanda	2029	139 (97–181)	0 (0–1)	3573 (2506–4640)	66 (27–105)
Rwanda	2030	137 (94–179)	0 (0–1)	3537 (2464–4610)	66 (21–110)
Rwanda	2031	135 (92–178)	0 (0–1)	3512 (2436–4588)	66 (16–115)
Rwanda	2032	134 (90–177)	0 (0–1)	3494 (2416–4571)	66 (12–120)
Rwanda	2033	133 (89–176)	0 (0–1)	3481 (2403–4560)	66 (7–124)
Rwanda	2034	132 (88–175)	0 (0–1)	3473 (2394–4551)	66 (3–128)
Rwanda	2035	131 (87–175)	1 (0–1)	3467 (2388–4545)	66 (-1–132)
Rwanda	2036	130 (87–174)	1 (0–1)	3462 (2383–4541)	66 (-5–136)
Saint Kitts and Nevis	2022	0 (0–0)	0 (0–0)	10 (10–11)	13 (12–14)
Saint Kitts and Nevis	2023	0 (0–0)	0 (0–0)	11 (10–11)	13 (12–14)
Saint Kitts and Nevis	2024	0 (0–0)	0 (0–0)	11 (10–12)	13 (11–14)
Saint Kitts and Nevis	2025	0 (0–0)	0 (0–0)	11 (10–12)	13 (11–14)
Saint Kitts and Nevis	2026	0 (0–0)	0 (0–0)	12 (10–13)	13 (11–14)
Saint Kitts and Nevis	2027	0 (0–1)	0 (0–0)	12 (10–14)	12 (10–14)
Saint Kitts and Nevis	2028	0 (0–1)	0 (0–0)	12 (10–14)	12 (10–14)
Saint Kitts and Nevis	2029	0 (0–1)	0 (0–0)	12 (10–15)	12 (10–14)
Saint Kitts and Nevis	2030	0 (0–1)	0 (0–1)	13 (10–15)	12 (9–14)
Saint Kitts and Nevis	2031	0 (0–1)	0 (0–1)	13 (10–16)	12 (9–14)
Saint Kitts and Nevis	2032	1 (0–1)	0 (0–1)	13 (10–17)	12 (9–14)
Saint Kitts and Nevis	2033	1 (0–1)	0 (0–1)	14 (10–17)	11 (8–14)
Saint Kitts and Nevis	2034	1 (0–1)	0 (0–1)	14 (9–18)	11 (8–14)
Saint Kitts and Nevis	2035	1 (0–1)	0 (0–1)	14 (9–19)	11 (8–14)
Saint Kitts and Nevis	2036	1 (0–1)	0 (0–1)	14 (9–20)	11 (8–14)
Saint Lucia	2022	2 (2–2)	1 (0–1)	53 (50–55)	21 (19–22)
Saint Lucia	2023	2 (2–2)	1 (0–1)	54 (50–57)	20 (18–23)
Saint Lucia	2024	2 (2–2)	1 (0–1)	54 (50–59)	20 (17–23)

Saint Lucia	2025	2 (2–2)	1 (0–1)	55 (50–60)	20 (16–23)
Saint Lucia	2026	2 (2–2)	1 (0–1)	56 (51–61)	19 (15–23)
Saint Lucia	2027	2 (2–2)	1 (0–1)	57 (51–63)	19 (15–23)
Saint Lucia	2028	2 (2–3)	1 (0–1)	58 (51–64)	19 (14–23)
Saint Lucia	2029	2 (2–3)	1 (0–1)	58 (52–65)	18 (13–23)
Saint Lucia	2030	2 (2–3)	1 (0–1)	59 (52–66)	18 (13–23)
Saint Lucia	2031	2 (2–3)	1 (0–1)	60 (52–68)	18 (12–23)
Saint Lucia	2032	2 (2–3)	1 (0–1)	61 (53–69)	17 (11–23)
Saint Lucia	2033	2 (2–3)	1 (0–1)	62 (53–70)	17 (11–23)
Saint Lucia	2034	2 (2–3)	1 (0–1)	62 (54–71)	17 (10–23)
Saint Lucia	2035	3 (2–3)	1 (0–1)	63 (54–72)	16 (10–23)
Saint Lucia	2036	3 (2–3)	1 (0–1)	64 (55–73)	16 (9–23)
Saint Vincent and the Grenadines	2022	1 (1–1)	1 (0–1)	16 (15–17)	11 (10–11)
Saint Vincent and the Grenadines	2023	1 (1–1)	1 (0–1)	16 (15–18)	11 (10–12)
Saint Vincent and the Grenadines	2024	1 (1–1)	1 (0–1)	17 (15–18)	10 (9–12)
Saint Vincent and the Grenadines	2025	1 (1–1)	1 (0–1)	17 (14–19)	10 (9–12)
Saint Vincent and the Grenadines	2026	1 (1–1)	1 (0–1)	17 (14–20)	10 (9–12)
Saint Vincent and the Grenadines	2027	1 (1–1)	1 (0–1)	17 (13–22)	10 (9–12)
Saint Vincent and the Grenadines	2028	1 (1–1)	1 (0–1)	18 (13–23)	10 (9–12)
Saint Vincent and the Grenadines	2029	1 (0–1)	1 (0–1)	18 (12–24)	10 (9–12)
Saint Vincent and the Grenadines	2030	1 (0–1)	1 (0–1)	18 (12–25)	10 (9–12)
Saint Vincent and the Grenadines	2031	1 (0–1)	1 (0–1)	19 (11–26)	10 (9–12)
Saint Vincent and the Grenadines	2032	1 (0–1)	1 (0–1)	19 (10–28)	10 (9–12)
Saint Vincent and the Grenadines	2033	1 (0–1)	1 (0–1)	19 (9–29)	10 (9–12)
Saint Vincent and the Grenadines	2034	1 (0–1)	1 (0–1)	19 (9–30)	10 (9–12)
Saint Vincent and the Grenadines	2035	1 (0–1)	1 (0–1)	20 (8–32)	10 (9–12)
Saint Vincent and the Grenadines	2036	1 (0–1)	1 (0–1)	20 (7–33)	10 (9–12)
Samoa	2022	0 (0–0)	1 (1–1)	13 (12–13)	8 (8–8)
Samoa	2023	0 (0–0)	1 (1–1)	13 (13–13)	8 (8–8)
Samoa	2024	0 (0–0)	1 (0–1)	13 (13–13)	8 (8–8)
Samoa	2025	0 (0–1)	1 (0–1)	13 (13–14)	8 (8–8)
Samoa	2026	0 (0–1)	1 (0–1)	13 (13–14)	8 (8–9)
Samoa	2027	1 (0–1)	1 (0–1)	14 (13–15)	8 (7–9)

Samoa	2028	1 (0–1)	1 (0–1)	14 (13–15)	8 (7–9)
Samoa	2029	1 (0–1)	1 (0–1)	14 (13–16)	8 (7–9)
Samoa	2030	1 (0–1)	1 (0–1)	14 (13–16)	8 (7–9)
Samoa	2031	1 (0–1)	1 (0–1)	15 (13–17)	8 (7–9)
Samoa	2032	1 (0–1)	1 (0–1)	15 (13–17)	8 (7–9)
Samoa	2033	1 (0–1)	1 (0–1)	15 (13–18)	8 (7–9)
Samoa	2034	1 (0–1)	1 (0–1)	15 (13–18)	8 (7–10)
Samoa	2035	1 (0–1)	1 (0–1)	16 (12–19)	8 (6–10)
Samoa	2036	1 (0–1)	1 (0–1)	16 (12–19)	8 (6–10)
San Marino	2022	0 (0–0)	1 (1–1)	5 (4–6)	8 (6–9)
San Marino	2023	0 (0–0)	1 (1–1)	5 (4–7)	7 (5–9)
San Marino	2024	0 (0–0)	1 (0–1)	5 (4–7)	7 (5–9)
San Marino	2025	0 (0–0)	1 (0–1)	5 (3–7)	7 (4–9)
San Marino	2026	0 (0–0)	1 (0–1)	5 (3–8)	6 (3–9)
San Marino	2027	0 (0–0)	1 (0–1)	5 (3–8)	6 (3–9)
San Marino	2028	0 (0–0)	1 (0–1)	5 (3–8)	6 (2–9)
San Marino	2029	0 (0–0)	1 (0–1)	5 (3–8)	5 (1–9)
San Marino	2030	0 (0–0)	1 (0–1)	5 (2–8)	5 (1–9)
San Marino	2031	0 (0–0)	0 (0–1)	5 (2–8)	5 (0–9)
San Marino	2032	0 (0–0)	0 (0–1)	5 (2–9)	4 (-0–9)
San Marino	2033	0 (0–0)	0 (-0–1)	5 (2–9)	4 (-1–9)
San Marino	2034	0 (0–0)	0 (-0–1)	5 (2–9)	4 (-1–9)
San Marino	2035	0 (0–0)	0 (-0–1)	5 (2–9)	3 (-2–8)
San Marino	2036	0 (0–0)	0 (-0–1)	5 (2–9)	3 (-2–8)
Sao Tome and Principe	2022	0 (0–0)	0 (0–0)	14 (14–14)	11 (11–11)
Sao Tome and Principe	2023	1 (0–1)	0 (0–0)	15 (14–15)	11 (11–11)
Sao Tome and Principe	2024	1 (1–1)	0 (0–0)	15 (15–16)	11 (11–12)
Sao Tome and Principe	2025	1 (1–1)	0 (0–0)	16 (15–17)	11 (11–12)
Sao Tome and Principe	2026	1 (1–1)	0 (0–0)	17 (15–18)	12 (11–12)
Sao Tome and Principe	2027	1 (1–1)	0 (0–0)	17 (16–19)	12 (11–13)
Sao Tome and Principe	2028	1 (1–1)	0 (0–0)	18 (16–20)	12 (11–13)
Sao Tome and Principe	2029	1 (1–1)	0 (0–0)	18 (16–21)	12 (11–13)
Sao Tome and Principe	2030	1 (1–1)	0 (0–0)	19 (16–22)	12 (11–14)

Sao Tome and Principe	2031	1 (1–1)	0 (0–0)	19 (16–23)	12 (11–14)
Sao Tome and Principe	2032	1 (1–1)	0 (0–0)	20 (16–24)	13 (11–14)
Sao Tome and Principe	2033	1 (1–1)	0 (0–0)	21 (17–25)	13 (11–15)
Sao Tome and Principe	2034	1 (1–1)	0 (0–0)	21 (17–26)	13 (11–15)
Sao Tome and Principe	2035	1 (1–1)	0 (0–0)	22 (17–27)	13 (11–15)
Sao Tome and Principe	2036	1 (1–1)	0 (0–0)	22 (17–28)	13 (11–16)
Saudi Arabia	2022	82 (81–83)	0 (0–0)	2407 (2374–2440)	11 (10–11)
Saudi Arabia	2023	85 (82–87)	0 (0–0)	2500 (2426–2573)	11 (10–11)
Saudi Arabia	2024	88 (84–92)	0 (0–0)	2592 (2470–2715)	11 (10–11)
Saudi Arabia	2025	91 (85–97)	0 (0–0)	2684 (2505–2864)	10 (9–12)
Saudi Arabia	2026	94 (86–102)	0 (0–0)	2777 (2534–3020)	10 (9–12)
Saudi Arabia	2027	97 (87–107)	0 (0–0)	2869 (2557–3182)	10 (9–12)
Saudi Arabia	2028	100 (88–113)	0 (0–0)	2962 (2574–3349)	10 (8–13)
Saudi Arabia	2029	104 (88–119)	0 (0–0)	3054 (2587–3522)	10 (8–13)
Saudi Arabia	2030	107 (89–125)	0 (0–0)	3147 (2594–3699)	10 (7–13)
Saudi Arabia	2031	110 (89–131)	0 (0–0)	3239 (2597–3882)	10 (7–13)
Saudi Arabia	2032	113 (89–137)	0 (0–0)	3332 (2595–4068)	10 (7–14)
Saudi Arabia	2033	116 (89–143)	0 (0–0)	3424 (2589–4259)	10 (7–14)
Saudi Arabia	2034	119 (89–150)	0 (0–0)	3516 (2580–4453)	10 (6–14)
Saudi Arabia	2035	122 (88–156)	0 (0–0)	3609 (2566–4652)	10 (6–14)
Saudi Arabia	2036	125 (88–163)	0 (0–0)	3701 (2549–4854)	10 (6–15)
Senegal	2022	46 (44–47)	0 (0–0)	1315 (1273–1357)	15 (14–16)
Senegal	2023	47 (45–49)	0 (0–0)	1360 (1292–1427)	15 (14–17)
Senegal	2024	49 (46–52)	0 (0–0)	1404 (1312–1497)	16 (14–17)
Senegal	2025	50 (46–54)	0 (0–0)	1449 (1331–1567)	16 (14–18)
Senegal	2026	52 (47–57)	0 (0–0)	1494 (1348–1639)	16 (14–18)
Senegal	2027	53 (48–59)	0 (0–0)	1538 (1365–1712)	16 (13–19)
Senegal	2028	55 (48–62)	0 (0–0)	1583 (1380–1786)	16 (13–20)
Senegal	2029	56 (49–64)	0 (0–0)	1628 (1394–1862)	17 (13–20)
Senegal	2030	58 (49–67)	0 (0–0)	1672 (1406–1939)	17 (13–21)
Senegal	2031	60 (49–70)	0 (0–0)	1717 (1417–2017)	17 (12–22)
Senegal	2032	61 (50–72)	0 (0–0)	1762 (1427–2096)	17 (12–22)
Senegal	2033	63 (50–75)	0 (0–0)	1806 (1436–2177)	17 (12–23)

Senegal	2034	64 (50–78)	0 (0–0)	1851 (1444–2258)	18 (11–24)
Senegal	2035	66 (51–81)	0 (0–0)	1896 (1451–2341)	18 (11–25)
Senegal	2036	67 (51–84)	0 (0–0)	1941 (1456–2425)	18 (11–25)
Serbia	2022	144 (134–153)	2 (1–2)	3595 (3334–3857)	24 (22–25)
Serbia	2023	143 (128–157)	2 (1–2)	3554 (3155–3953)	23 (20–26)
Serbia	2024	142 (123–160)	2 (1–2)	3512 (2988–4037)	23 (19–26)
Serbia	2025	141 (117–164)	2 (1–2)	3471 (2823–4118)	23 (18–27)
Serbia	2026	140 (112–167)	2 (1–2)	3429 (2658–4201)	22 (17–27)
Serbia	2027	139 (107–170)	2 (1–2)	3388 (2490–4285)	22 (16–28)
Serbia	2028	138 (101–174)	2 (1–2)	3346 (2320–4372)	21 (15–28)
Serbia	2029	137 (95–178)	2 (1–2)	3304 (2146–4463)	21 (14–29)
Serbia	2030	136 (90–181)	2 (1–2)	3263 (1969–4556)	21 (12–29)
Serbia	2031	135 (84–185)	2 (1–2)	3221 (1788–4654)	20 (11–30)
Serbia	2032	134 (78–189)	2 (1–2)	3180 (1604–4755)	20 (10–30)
Serbia	2033	133 (72–194)	2 (1–2)	3138 (1417–4859)	20 (9–31)
Serbia	2034	132 (65–198)	2 (1–2)	3096 (1225–4968)	19 (8–31)
Serbia	2035	131 (59–202)	2 (1–2)	3055 (1030–5079)	19 (6–32)
Serbia	2036	130 (52–207)	2 (1–2)	3013 (832–5195)	19 (5–32)
Seychelles	2022	2 (2–2)	1 (1–1)	56 (53–58)	44 (41–47)
Seychelles	2023	2 (2–2)	1 (1–1)	56 (51–61)	44 (39–50)
Seychelles	2024	2 (2–2)	1 (1–1)	57 (51–63)	44 (37–52)
Seychelles	2025	2 (2–2)	1 (1–1)	58 (50–65)	44 (36–53)
Seychelles	2026	2 (2–2)	1 (1–1)	58 (50–67)	44 (35–54)
Seychelles	2027	2 (2–3)	1 (1–1)	59 (50–69)	44 (33–55)
Seychelles	2028	2 (2–3)	1 (1–1)	60 (49–71)	44 (33–56)
Seychelles	2029	2 (2–3)	1 (1–1)	61 (49–72)	44 (32–57)
Seychelles	2030	2 (2–3)	1 (1–1)	61 (49–74)	44 (31–58)
Seychelles	2031	2 (2–3)	1 (1–1)	62 (49–75)	44 (30–59)
Seychelles	2032	2 (2–3)	1 (1–1)	63 (49–76)	44 (29–59)
Seychelles	2033	2 (2–3)	1 (1–1)	64 (50–78)	44 (29–60)
Seychelles	2034	2 (2–3)	1 (1–1)	64 (50–79)	44 (28–61)
Seychelles	2035	2 (2–3)	1 (1–1)	65 (50–80)	44 (27–61)
Seychelles	2036	2 (2–3)	1 (1–1)	66 (50–82)	44 (27–62)

Sierra Leone	2022	25 (24–25)	0 (0–0)	719 (709–728)	17 (17–17)
Sierra Leone	2023	25 (25–26)	0 (0–0)	742 (721–763)	17 (16–18)
Sierra Leone	2024	26 (25–27)	0 (0–0)	766 (731–801)	17 (16–18)
Sierra Leone	2025	27 (25–29)	0 (0–0)	789 (738–840)	17 (15–19)
Sierra Leone	2026	28 (26–30)	0 (0–0)	812 (743–882)	17 (15–19)
Sierra Leone	2027	29 (26–31)	0 (0–0)	836 (746–925)	17 (14–19)
Sierra Leone	2028	29 (26–33)	0 (0–0)	859 (748–970)	17 (14–20)
Sierra Leone	2029	30 (26–34)	0 (0–0)	883 (749–1017)	17 (14–20)
Sierra Leone	2030	31 (26–36)	0 (0–0)	906 (748–1065)	17 (13–21)
Sierra Leone	2031	32 (26–37)	0 (0–0)	930 (746–1114)	17 (13–21)
Sierra Leone	2032	32 (26–39)	0 (0–0)	953 (742–1164)	17 (12–22)
Sierra Leone	2033	33 (26–41)	0 (0–0)	976 (737–1216)	17 (12–22)
Sierra Leone	2034	34 (25–42)	0 (0–0)	1000 (731–1268)	17 (11–23)
Sierra Leone	2035	35 (25–44)	0 (0–0)	1023 (725–1322)	17 (11–23)
Sierra Leone	2036	35 (25–46)	0 (0–0)	1047 (716–1377)	17 (10–24)
Singapore	2022	36 (30–41)	0 (0–0)	826 (696–956)	8 (4–12)
Singapore	2023	35 (29–42)	0 (0–0)	823 (670–977)	7 (3–12)
Singapore	2024	35 (28–42)	0 (0–0)	822 (660–983)	6 (1–12)
Singapore	2025	35 (27–42)	0 (0–0)	821 (656–985)	5 (-0–11)
Singapore	2026	34 (27–42)	0 (0–0)	820 (654–985)	4 (-2–11)
Singapore	2027	34 (27–42)	0 (0–0)	819 (653–985)	3 (-3–10)
Singapore	2028	34 (27–42)	0 (0–0)	819 (653–985)	3 (-5–10)
Singapore	2029	34 (26–41)	0 (0–0)	819 (653–985)	2 (-6–9)
Singapore	2030	34 (26–41)	0 (0–0)	819 (652–985)	1 (-8–9)
Singapore	2031	34 (26–41)	0 (0–0)	819 (652–985)	-0 (-9–8)
Singapore	2032	34 (26–41)	0 (0–0)	819 (652–985)	-1 (-10–8)
Singapore	2033	34 (26–41)	0 (0–0)	819 (652–985)	-2 (-12–7)
Singapore	2034	34 (26–41)	0 (0–0)	819 (652–985)	-3 (-13–6)
Singapore	2035	34 (26–41)	0 (0–0)	819 (652–985)	-4 (-14–6)
Singapore	2036	34 (26–41)	0 (0–0)	819 (652–985)	-5 (-16–5)
Slovakia	2022	105 (101–110)	1 (1–1)	2814 (2683–2944)	30 (28–32)
Slovakia	2023	105 (99–112)	1 (1–1)	2836 (2680–2991)	29 (27–32)
Slovakia	2024	105 (98–113)	1 (1–1)	2850 (2686–3014)	29 (25–32)

Slovakia	2025	105 (96–114)	1 (1–1)	2859 (2691–3026)	28 (24–32)
Slovakia	2026	105 (95–115)	1 (1–2)	2864 (2695–3033)	28 (23–32)
Slovakia	2027	105 (94–116)	1 (1–2)	2868 (2698–3037)	27 (22–32)
Slovakia	2028	105 (94–117)	1 (1–2)	2870 (2700–3040)	26 (21–32)
Slovakia	2029	105 (93–118)	1 (1–2)	2872 (2702–3042)	26 (20–31)
Slovakia	2030	105 (92–119)	1 (1–2)	2873 (2703–3043)	25 (19–31)
Slovakia	2031	105 (91–120)	1 (1–2)	2873 (2703–3043)	24 (18–31)
Slovakia	2032	105 (91–120)	1 (1–2)	2874 (2704–3044)	24 (17–31)
Slovakia	2033	105 (90–121)	1 (1–2)	2874 (2704–3044)	23 (16–30)
Slovakia	2034	105 (89–122)	1 (1–2)	2874 (2704–3044)	23 (15–30)
Slovakia	2035	105 (89–122)	1 (1–2)	2874 (2704–3044)	22 (15–30)
Slovakia	2036	105 (88–123)	1 (1–2)	2874 (2704–3044)	21 (14–29)
Slovenia	2022	40 (37–44)	1 (1–1)	985 (893–1078)	24 (21–27)
Slovenia	2023	40 (36–45)	1 (1–1)	985 (855–1116)	24 (20–27)
Slovenia	2024	40 (34–46)	1 (1–1)	985 (825–1145)	23 (19–27)
Slovenia	2025	40 (34–47)	1 (1–1)	985 (800–1170)	22 (18–27)
Slovenia	2026	40 (33–48)	1 (1–1)	985 (779–1192)	21 (16–26)
Slovenia	2027	40 (32–49)	1 (1–1)	985 (759–1212)	21 (15–26)
Slovenia	2028	40 (31–49)	1 (1–1)	985 (741–1230)	20 (14–26)
Slovenia	2029	40 (31–50)	1 (1–1)	985 (724–1247)	19 (13–25)
Slovenia	2030	40 (30–51)	1 (1–1)	985 (708–1263)	19 (12–25)
Slovenia	2031	40 (30–51)	1 (1–1)	985 (693–1278)	18 (11–24)
Slovenia	2032	40 (29–52)	1 (1–1)	985 (679–1292)	17 (10–24)
Slovenia	2033	40 (29–52)	1 (1–1)	985 (665–1306)	16 (9–23)
Slovenia	2034	40 (28–53)	1 (1–1)	985 (652–1319)	16 (8–23)
Slovenia	2035	40 (28–53)	1 (1–1)	985 (639–1331)	15 (7–23)
Slovenia	2036	40 (27–54)	1 (1–1)	985 (627–1343)	14 (6–22)
Solomon Islands	2022	3 (3–3)	1 (0–1)	80 (79–82)	20 (19–20)
Solomon Islands	2023	3 (3–3)	1 (0–1)	82 (80–85)	20 (19–20)
Solomon Islands	2024	3 (3–3)	1 (0–1)	84 (81–88)	19 (18–20)
Solomon Islands	2025	3 (3–3)	1 (0–1)	86 (82–91)	19 (18–21)
Solomon Islands	2026	3 (3–3)	1 (0–1)	88 (83–94)	19 (18–21)
Solomon Islands	2027	3 (3–3)	1 (0–1)	90 (84–97)	19 (18–21)

Solomon Islands	2028	3 (3–3)	1 (0–1)	92 (85–100)	19 (18–21)
Solomon Islands	2029	3 (3–3)	1 (0–1)	94 (85–104)	19 (17–21)
Solomon Islands	2030	3 (3–3)	1 (0–1)	96 (86–107)	19 (17–21)
Solomon Islands	2031	3 (3–4)	1 (1–1)	98 (87–110)	19 (17–21)
Solomon Islands	2032	3 (3–4)	1 (1–1)	100 (88–113)	19 (17–21)
Solomon Islands	2033	3 (3–4)	1 (1–1)	103 (88–117)	19 (17–21)
Solomon Islands	2034	3 (3–4)	1 (1–1)	105 (89–120)	18 (16–21)
Solomon Islands	2035	3 (3–4)	1 (1–1)	107 (90–124)	18 (16–21)
Solomon Islands	2036	3 (3–4)	1 (1–1)	109 (90–127)	18 (16–21)
Somalia	2022	92 (92–93)	0 (0–0)	2854 (2823–2885)	40 (39–40)
Somalia	2023	93 (91–95)	0 (0–0)	2872 (2795–2949)	39 (38–40)
Somalia	2024	94 (90–98)	0 (0–0)	2893 (2768–3017)	38 (36–40)
Somalia	2025	95 (90–100)	0 (0–0)	2916 (2746–3085)	37 (34–40)
Somalia	2026	96 (90–101)	0 (0–0)	2940 (2728–3152)	36 (32–40)
Somalia	2027	97 (91–103)	0 (0–0)	2966 (2714–3217)	35 (31–40)
Somalia	2028	98 (91–104)	0 (0–0)	2992 (2703–3280)	34 (29–39)
Somalia	2029	99 (91–106)	0 (0–0)	3018 (2695–3341)	33 (27–39)
Somalia	2030	100 (92–107)	0 (0–0)	3045 (2690–3399)	32 (26–39)
Somalia	2031	100 (92–109)	0 (0–0)	3071 (2687–3456)	31 (24–39)
Somalia	2032	101 (93–110)	0 (0–0)	3098 (2686–3511)	31 (23–39)
Somalia	2033	102 (93–111)	0 (0–0)	3125 (2686–3564)	30 (21–38)
Somalia	2034	103 (94–113)	0 (0–0)	3153 (2688–3617)	29 (19–38)
Somalia	2035	104 (94–114)	0 (0–0)	3180 (2692–3668)	28 (18–38)
Somalia	2036	105 (95–115)	0 (0–0)	3207 (2696–3718)	27 (16–37)
South Africa	2022	950 (877–1024)	1 (1–1)	26901 (24741–29062)	53 (46–61)
South Africa	2023	953 (820–1085)	1 (1–1)	27003 (23215–30790)	53 (40–67)
South Africa	2024	955 (773–1137)	1 (1–1)	27077 (21999–32154)	53 (34–72)
South Africa	2025	958 (737–1178)	1 (1–1)	27131 (21084–33179)	53 (29–77)
South Africa	2026	960 (711–1209)	1 (1–1)	27173 (20416–33929)	53 (25–82)
South Africa	2027	962 (693–1231)	1 (1–1)	27204 (19941–34467)	53 (21–85)
South Africa	2028	964 (681–1248)	1 (1–1)	27229 (19609–34849)	53 (17–89)
South Africa	2029	966 (673–1259)	1 (1–1)	27248 (19379–35117)	53 (14–92)
South Africa	2030	967 (668–1267)	1 (1–1)	27263 (19222–35305)	53 (11–95)

South Africa	2031	968 (665–1272)	1 (1–1)	27275 (19116–35435)	53 (8–98)
South Africa	2032	969 (663–1275)	1 (1–1)	27285 (19044–35526)	53 (5–101)
South Africa	2033	970 (662–1278)	1 (0–1)	27293 (18997–35588)	53 (3–103)
South Africa	2034	970 (662–1279)	1 (0–1)	27299 (18966–35632)	53 (0–106)
South Africa	2035	971 (662–1280)	1 (0–1)	27304 (18946–35662)	53 (-2–108)
South Africa	2036	971 (662–1281)	1 (0–1)	27308 (18933–35684)	53 (-4–110)
South Sudan	2022	69 (68–70)	0 (0–0)	2043 (2006–2080)	46 (45–47)
South Sudan	2023	70 (68–73)	0 (0–0)	2082 (2012–2152)	46 (43–48)
South Sudan	2024	71 (67–76)	0 (0–0)	2118 (2012–2224)	46 (42–49)
South Sudan	2025	72 (66–79)	0 (0–0)	2151 (2006–2296)	45 (41–50)
South Sudan	2026	73 (64–82)	0 (0–0)	2181 (1995–2367)	45 (40–51)
South Sudan	2027	74 (63–85)	0 (0–0)	2208 (1980–2436)	45 (38–53)
South Sudan	2028	75 (61–89)	0 (0–0)	2233 (1962–2503)	45 (36–54)
South Sudan	2029	76 (59–93)	0 (0–0)	2254 (1941–2568)	45 (35–56)
South Sudan	2030	77 (57–97)	0 (0–0)	2274 (1918–2630)	45 (33–58)
South Sudan	2031	78 (55–101)	0 (0–0)	2290 (1892–2688)	45 (31–60)
South Sudan	2032	79 (53–106)	0 (0–0)	2304 (1865–2744)	45 (29–62)
South Sudan	2033	80 (50–110)	0 (0–0)	2316 (1837–2796)	45 (27–64)
South Sudan	2034	81 (48–115)	0 (0–0)	2326 (1807–2845)	45 (24–66)
South Sudan	2035	82 (45–120)	0 (0–0)	2334 (1777–2891)	45 (22–68)
South Sudan	2036	83 (42–125)	0 (0–0)	2340 (1746–2934)	45 (20–70)
Spain	2022	927 (891–964)	1 (1–1)	20838 (19902–21774)	23 (22–25)
Spain	2023	912 (857–967)	1 (1–1)	20529 (19205–21852)	22 (20–24)
Spain	2024	897 (825–968)	1 (1–1)	20219 (18598–21840)	21 (19–24)
Spain	2025	881 (793–969)	1 (1–1)	19910 (18038–21782)	20 (17–23)
Spain	2026	866 (761–970)	1 (1–1)	19600 (17507–21693)	19 (16–22)
Spain	2027	850 (729–971)	1 (1–1)	19291 (16998–21583)	18 (14–21)
Spain	2028	835 (697–973)	1 (1–1)	18981 (16505–21458)	17 (13–20)
Spain	2029	819 (664–975)	1 (1–1)	18672 (16024–21319)	16 (12–19)
Spain	2030	804 (631–977)	1 (1–1)	18362 (15554–21170)	14 (10–19)
Spain	2031	788 (597–980)	1 (1–1)	18053 (15093–21013)	13 (9–18)
Spain	2032	773 (563–983)	1 (1–1)	17743 (14639–20848)	12 (8–17)
Spain	2033	758 (529–986)	1 (1–1)	17434 (14191–20676)	11 (6–16)

Spain	2034	742 (494–990)	1 (1–1)	17124 (13749–20499)	10 (5–15)
Spain	2035	727 (459–995)	1 (1–1)	16815 (13312–20317)	9 (4–14)
Spain	2036	711 (423–999)	1 (1–1)	16505 (12880–20130)	8 (2–13)
Sri Lanka	2022	321 (303–340)	0 (0–0)	8106 (7603–8609)	28 (25–31)
Sri Lanka	2023	325 (299–351)	0 (0–0)	8196 (7485–8908)	28 (24–31)
Sri Lanka	2024	329 (297–361)	0 (0–0)	8286 (7414–9157)	27 (22–32)
Sri Lanka	2025	333 (296–370)	0 (0–0)	8376 (7369–9382)	26 (21–32)
Sri Lanka	2026	337 (296–378)	0 (0–0)	8466 (7341–9591)	26 (20–32)
Sri Lanka	2027	341 (295–386)	0 (0–0)	8555 (7323–9788)	25 (19–32)
Sri Lanka	2028	344 (296–393)	0 (0–0)	8645 (7314–9976)	25 (17–32)
Sri Lanka	2029	348 (296–400)	0 (0–0)	8735 (7312–10158)	24 (16–32)
Sri Lanka	2030	352 (297–407)	0 (0–0)	8825 (7315–10334)	23 (15–32)
Sri Lanka	2031	356 (298–414)	0 (0–0)	8915 (7324–10506)	23 (14–31)
Sri Lanka	2032	360 (299–421)	0 (0–0)	9005 (7336–10673)	22 (13–31)
Sri Lanka	2033	364 (300–428)	0 (0–0)	9094 (7351–10837)	22 (12–31)
Sri Lanka	2034	368 (301–434)	0 (0–0)	9184 (7370–10998)	21 (11–31)
Sri Lanka	2035	372 (303–440)	0 (0–0)	9274 (7391–11156)	21 (10–31)
Sri Lanka	2036	375 (304–447)	0 (0–0)	9364 (7415–11312)	20 (9–30)
Sudan	2022	258 (257–260)	0 (0–0)	6495 (6458–6533)	32 (32–33)
Sudan	2023	263 (259–267)	0 (0–0)	6634 (6537–6731)	32 (32–33)
Sudan	2024	267 (260–274)	0 (0–0)	6773 (6602–6944)	32 (32–33)
Sudan	2025	271 (260–282)	0 (0–0)	6912 (6654–7170)	32 (32–33)
Sudan	2026	276 (261–291)	0 (0–0)	7051 (6695–7406)	32 (31–33)
Sudan	2027	280 (260–299)	0 (0–0)	7190 (6727–7653)	32 (31–34)
Sudan	2028	284 (260–309)	0 (0–0)	7329 (6750–7908)	32 (31–34)
Sudan	2029	288 (259–318)	0 (0–0)	7468 (6764–8172)	32 (31–34)
Sudan	2030	293 (257–328)	0 (0–0)	7607 (6770–8443)	32 (31–34)
Sudan	2031	297 (256–338)	0 (0–0)	7746 (6769–8722)	32 (30–34)
Sudan	2032	301 (254–349)	0 (0–0)	7884 (6761–9007)	32 (30–34)
Sudan	2033	305 (251–359)	0 (0–0)	8023 (6747–9300)	32 (30–35)
Sudan	2034	310 (249–371)	0 (0–0)	8162 (6726–9599)	32 (30–35)
Sudan	2035	314 (246–382)	0 (0–0)	8301 (6698–9904)	32 (30–35)
Sudan	2036	318 (243–393)	0 (0–0)	8440 (6665–10215)	32 (30–35)

Suriname	2022	3 (2–3)	1 (1–1)	74 (70–78)	11 (10–12)
Suriname	2023	3 (2–3)	1 (1–1)	76 (67–84)	11 (9–13)
Suriname	2024	3 (2–3)	1 (1–1)	80 (68–91)	11 (9–14)
Suriname	2025	3 (2–3)	1 (1–1)	82 (70–95)	12 (9–15)
Suriname	2026	3 (2–3)	1 (1–1)	83 (70–97)	11 (8–15)
Suriname	2027	3 (2–4)	1 (1–1)	84 (69–98)	11 (8–15)
Suriname	2028	3 (2–4)	1 (1–1)	85 (69–100)	11 (8–15)
Suriname	2029	3 (2–4)	1 (1–1)	86 (70–102)	11 (8–15)
Suriname	2030	3 (2–4)	1 (1–1)	87 (70–105)	11 (7–15)
Suriname	2031	3 (2–4)	1 (1–1)	89 (71–107)	11 (7–16)
Suriname	2032	3 (2–4)	1 (1–1)	90 (71–109)	11 (7–16)
Suriname	2033	3 (2–4)	1 (1–1)	91 (71–111)	11 (7–16)
Suriname	2034	3 (2–4)	1 (1–1)	92 (72–113)	11 (7–16)
Suriname	2035	3 (2–4)	1 (1–1)	94 (72–115)	11 (6–16)
Suriname	2036	3 (2–4)	1 (1–1)	95 (73–117)	11 (6–17)
Sweden	2022	239 (227–251)	1 (1–1)	4532 (4288–4776)	22 (21–24)
Sweden	2023	239 (218–260)	1 (1–1)	4532 (4109–4956)	22 (20–24)
Sweden	2024	239 (212–267)	1 (1–1)	4532 (3985–5079)	22 (20–25)
Sweden	2025	239 (207–272)	1 (1–2)	4532 (3885–5179)	22 (19–25)
Sweden	2026	239 (202–276)	1 (1–2)	4532 (3799–5266)	22 (19–26)
Sweden	2027	239 (198–280)	1 (1–2)	4532 (3721–5343)	22 (18–26)
Sweden	2028	239 (195–284)	1 (1–2)	4532 (3650–5414)	22 (18–26)
Sweden	2029	239 (191–287)	1 (1–2)	4532 (3585–5480)	22 (18–27)
Sweden	2030	239 (188–290)	1 (1–2)	4532 (3524–5541)	22 (18–27)
Sweden	2031	239 (185–293)	1 (1–2)	4532 (3466–5598)	22 (17–27)
Sweden	2032	239 (183–296)	1 (1–2)	4532 (3411–5653)	22 (17–27)
Sweden	2033	239 (180–299)	1 (1–2)	4532 (3359–5705)	22 (17–28)
Sweden	2034	239 (177–301)	1 (1–2)	4532 (3309–5755)	22 (17–28)
Sweden	2035	239 (175–304)	1 (1–2)	4532 (3261–5803)	22 (16–28)
Sweden	2036	239 (173–306)	1 (1–2)	4532 (3215–5849)	22 (16–28)
Switzerland	2022	229 (217–241)	1 (1–1)	4369 (4081–4657)	24 (22–27)
Switzerland	2023	229 (212–247)	1 (1–1)	4369 (3962–4777)	23 (20–27)
Switzerland	2024	229 (208–250)	1 (1–1)	4369 (3871–4868)	22 (18–27)

Switzerland	2025	229 (204–254)	1 (1–1)	4369 (3793–4945)	21 (16–26)
Switzerland	2026	229 (202–257)	1 (1–2)	4369 (3725–5013)	20 (15–26)
Switzerland	2027	229 (199–259)	1 (1–2)	4369 (3664–5075)	19 (13–25)
Switzerland	2028	229 (196–262)	1 (1–2)	4369 (3607–5131)	18 (12–25)
Switzerland	2029	229 (194–264)	1 (1–2)	4369 (3555–5184)	17 (10–24)
Switzerland	2030	229 (192–266)	1 (1–2)	4369 (3505–5233)	16 (9–24)
Switzerland	2031	229 (190–268)	1 (1–2)	4369 (3459–5280)	15 (8–23)
Switzerland	2032	229 (188–270)	1 (1–2)	4369 (3414–5324)	15 (6–23)
Switzerland	2033	229 (186–272)	1 (1–2)	4369 (3372–5367)	14 (5–22)
Switzerland	2034	229 (185–274)	1 (1–2)	4369 (3331–5408)	13 (4–22)
Switzerland	2035	229 (183–275)	1 (1–2)	4369 (3292–5447)	12 (2–21)
Switzerland	2036	229 (181–277)	1 (1–2)	4369 (3254–5485)	11 (1–20)
Syrian Arab Republic	2022	39 (38–40)	1 (1–1)	957 (929–984)	7 (7–7)
Syrian Arab Republic	2023	40 (38–42)	1 (0–1)	974 (928–1019)	7 (6–8)
Syrian Arab Republic	2024	41 (38–43)	1 (0–1)	991 (931–1051)	7 (6–8)
Syrian Arab Republic	2025	41 (38–44)	1 (0–1)	1008 (936–1080)	7 (6–8)
Syrian Arab Republic	2026	42 (39–45)	1 (0–1)	1025 (943–1108)	7 (6–8)
Syrian Arab Republic	2027	43 (39–47)	1 (0–1)	1042 (950–1134)	7 (6–8)
Syrian Arab Republic	2028	44 (39–48)	1 (0–1)	1059 (959–1160)	7 (6–8)
Syrian Arab Republic	2029	44 (40–49)	1 (0–1)	1076 (968–1185)	7 (6–8)
Syrian Arab Republic	2030	45 (40–50)	1 (0–1)	1093 (978–1209)	7 (6–8)
Syrian Arab Republic	2031	46 (41–51)	1 (0–1)	1111 (988–1233)	7 (6–8)
Syrian Arab Republic	2032	46 (41–52)	1 (0–1)	1128 (999–1257)	7 (6–8)
Syrian Arab Republic	2033	47 (42–53)	1 (0–1)	1145 (1010–1280)	7 (6–8)
Syrian Arab Republic	2034	48 (42–54)	1 (0–1)	1162 (1021–1303)	7 (6–9)
Syrian Arab Republic	2035	49 (43–55)	1 (0–1)	1179 (1032–1326)	7 (6–9)
Syrian Arab Republic	2036	49 (43–55)	1 (0–1)	1196 (1044–1348)	7 (5–9)
Taiwan (Province of China)	2022	1384 (1318–1449)	1 (1–1)	37547 (35596–39498)	90 (83–97)
Taiwan (Province of China)	2023	1384 (1282–1486)	1 (1–1)	37293 (34121–40465)	87 (76–99)
Taiwan (Province of China)	2024	1384 (1247–1522)	1 (1–1)	37040 (32638–41441)	85 (69–100)
Taiwan (Province of China)	2025	1385 (1212–1558)	1 (1–1)	36786 (31099–42473)	82 (63–101)
Taiwan (Province of China)	2026	1385 (1175–1595)	1 (1–1)	36533 (29492–43573)	79 (56–102)
Taiwan (Province of China)	2027	1385 (1138–1633)	1 (1–1)	36279 (27813–44745)	76 (49–104)

Taiwan (Province of China)	2028	1386 (1099–1672)	1 (1–1)	36026 (26064–45988)	73 (41–105)
Taiwan (Province of China)	2029	1386 (1059–1713)	1 (1–1)	35772 (24244–47300)	71 (34–107)
Taiwan (Province of China)	2030	1386 (1017–1756)	1 (1–1)	35519 (22357–48680)	68 (26–109)
Taiwan (Province of China)	2031	1387 (974–1800)	1 (1–1)	35265 (20404–50126)	65 (19–111)
Taiwan (Province of China)	2032	1387 (929–1845)	1 (1–1)	35011 (18387–51636)	62 (11–114)
Taiwan (Province of China)	2033	1387 (883–1892)	1 (1–1)	34758 (16308–53207)	59 (3–116)
Taiwan (Province of China)	2034	1388 (836–1939)	1 (1–1)	34504 (14170–54839)	57 (-6–119)
Taiwan (Province of China)	2035	1388 (787–1989)	1 (1–1)	34251 (11973–56529)	54 (-14–122)
Taiwan (Province of China)	2036	1388 (737–2039)	1 (1–1)	33997 (9718–58276)	51 (-22–125)
Tajikistan	2022	106 (99–112)	0 (0–0)	2741 (2575–2906)	44 (39–49)
Tajikistan	2023	106 (95–117)	0 (0–0)	2798 (2515–3082)	42 (34–49)
Tajikistan	2024	106 (91–121)	0 (0–0)	2856 (2446–3266)	39 (30–48)
Tajikistan	2025	106 (88–124)	0 (0–0)	2914 (2367–3461)	37 (26–47)
Tajikistan	2026	106 (85–127)	0 (0–0)	2972 (2278–3665)	35 (23–46)
Tajikistan	2027	106 (83–130)	0 (0–0)	3029 (2179–3880)	32 (19–45)
Tajikistan	2028	106 (81–132)	0 (0–0)	3087 (2070–4104)	30 (16–44)
Tajikistan	2029	106 (79–134)	0 (0–0)	3145 (1952–4337)	28 (13–42)
Tajikistan	2030	106 (77–136)	0 (0–0)	3203 (1826–4579)	25 (9–41)
Tajikistan	2031	106 (75–138)	0 (0–0)	3260 (1691–4830)	23 (6–40)
Tajikistan	2032	106 (73–140)	0 (0–0)	3318 (1548–5088)	21 (3–38)
Tajikistan	2033	106 (71–141)	0 (0–0)	3376 (1397–5355)	18 (-0–36)
Tajikistan	2034	106 (70–143)	0 (0–0)	3433 (1238–5629)	16 (-3–35)
Tajikistan	2035	106 (68–144)	0 (0–0)	3491 (1073–5910)	14 (-6–33)
Tajikistan	2036	106 (67–146)	0 (0–0)	3549 (900–6198)	11 (-9–32)
Thailand	2022	1581 (1532–1630)	1 (1–1)	41494 (40205–42783)	36 (34–39)
Thailand	2023	1607 (1538–1676)	1 (1–1)	42155 (40332–43978)	36 (33–39)
Thailand	2024	1633 (1548–1718)	1 (1–1)	42816 (40583–45048)	35 (31–39)
Thailand	2025	1659 (1561–1757)	1 (1–1)	43477 (40899–46055)	35 (30–39)
Thailand	2026	1685 (1576–1795)	1 (1–1)	44138 (41256–47020)	34 (29–39)
Thailand	2027	1712 (1592–1831)	1 (1–1)	44799 (41642–47956)	34 (28–39)
Thailand	2028	1738 (1608–1867)	1 (1–1)	45460 (42050–48871)	33 (27–39)
Thailand	2029	1764 (1625–1902)	1 (1–1)	46121 (42475–49767)	33 (26–39)
Thailand	2030	1790 (1643–1937)	1 (1–1)	46782 (42915–50649)	32 (25–39)

Thailand	2031	1816 (1661–1971)	1 (1–1)	47443 (43367–51520)	32 (24–39)
Thailand	2032	1842 (1680–2005)	1 (1–1)	48104 (43829–52380)	31 (24–39)
Thailand	2033	1869 (1699–2038)	1 (1–1)	48765 (44300–53231)	30 (23–38)
Thailand	2034	1895 (1718–2071)	1 (1–1)	49427 (44779–54074)	30 (22–38)
Thailand	2035	1921 (1738–2104)	1 (1–1)	50088 (45265–54911)	29 (21–38)
Thailand	2036	1947 (1757–2137)	1 (1–1)	50749 (45756–55741)	29 (20–38)
Timor-Leste	2022	5 (5–5)	0 (0–0)	125 (123–128)	14 (14–14)
Timor-Leste	2023	5 (5–5)	0 (0–0)	128 (124–132)	14 (13–15)
Timor-Leste	2024	5 (5–5)	0 (0–0)	130 (123–136)	14 (13–15)
Timor-Leste	2025	5 (5–5)	0 (0–0)	132 (123–141)	14 (12–16)
Timor-Leste	2026	5 (5–6)	0 (0–0)	134 (122–147)	14 (12–16)
Timor-Leste	2027	5 (5–6)	0 (0–0)	136 (121–152)	14 (11–17)
Timor-Leste	2028	5 (5–6)	0 (0–0)	139 (119–158)	14 (11–17)
Timor-Leste	2029	5 (5–6)	0 (0–0)	141 (118–164)	14 (10–18)
Timor-Leste	2030	6 (5–6)	0 (0–0)	143 (116–170)	14 (9–18)
Timor-Leste	2031	6 (4–7)	0 (0–0)	145 (114–177)	14 (8–19)
Timor-Leste	2032	6 (4–7)	0 (0–0)	147 (112–183)	14 (8–20)
Timor-Leste	2033	6 (4–7)	0 (0–0)	150 (109–190)	14 (7–20)
Timor-Leste	2034	6 (4–7)	0 (0–0)	152 (106–197)	14 (6–21)
Timor-Leste	2035	6 (4–8)	0 (0–0)	154 (104–204)	14 (5–22)
Timor-Leste	2036	6 (4–8)	0 (0–0)	156 (101–212)	13 (4–23)
Togo	2022	45 (45–46)	0 (0–0)	1302 (1289–1315)	29 (29–30)
Togo	2023	47 (46–48)	0 (0–0)	1357 (1328–1386)	30 (29–31)
Togo	2024	49 (47–51)	0 (0–0)	1412 (1363–1461)	30 (28–31)
Togo	2025	51 (49–53)	0 (0–0)	1467 (1395–1539)	30 (28–32)
Togo	2026	53 (50–56)	0 (0–0)	1522 (1425–1619)	30 (27–33)
Togo	2027	55 (51–59)	0 (0–0)	1577 (1452–1702)	30 (26–34)
Togo	2028	57 (52–62)	0 (0–0)	1632 (1477–1787)	30 (26–35)
Togo	2029	58 (52–65)	0 (0–0)	1687 (1500–1875)	30 (25–35)
Togo	2030	60 (53–68)	0 (0–0)	1742 (1521–1964)	30 (25–36)
Togo	2031	62 (54–71)	0 (0–0)	1797 (1540–2055)	30 (24–37)
Togo	2032	64 (55–74)	0 (0–0)	1852 (1557–2147)	30 (23–38)
Togo	2033	66 (55–77)	0 (0–0)	1907 (1573–2242)	30 (23–38)

Togo	2034	68 (56–80)	0 (0–0)	1962 (1587–2338)	30 (22–39)
Togo	2035	70 (56–83)	0 (0–0)	2017 (1599–2435)	31 (22–39)
Togo	2036	72 (57–87)	0 (0–0)	2072 (1610–2534)	31 (21–40)
Tokelau	2022	0 (0–0)	0 (0–0)	0 (0–0)	11 (11–12)
Tokelau	2023	0 (0–0)	0 (0–0)	0 (0–0)	11 (11–12)
Tokelau	2024	0 (0–0)	0 (0–0)	0 (0–0)	11 (11–12)
Tokelau	2025	0 (0–0)	0 (0–0)	0 (0–0)	11 (10–12)
Tokelau	2026	0 (0–0)	0 (0–0)	0 (0–0)	11 (10–12)
Tokelau	2027	0 (0–0)	0 (0–0)	0 (0–0)	11 (10–12)
Tokelau	2028	0 (0–0)	0 (0–1)	0 (0–0)	11 (10–12)
Tokelau	2029	0 (0–0)	0 (0–1)	0 (0–0)	11 (10–12)
Tokelau	2030	0 (0–0)	0 (0–1)	0 (0–0)	10 (9–11)
Tokelau	2031	0 (0–0)	0 (0–1)	0 (0–0)	10 (9–11)
Tokelau	2032	0 (0–0)	0 (0–1)	0 (0–0)	10 (9–11)
Tokelau	2033	0 (0–0)	0 (0–1)	0 (0–0)	10 (9–11)
Tokelau	2034	0 (0–0)	0 (0–1)	0 (0–0)	10 (9–11)
Tokelau	2035	0 (0–0)	1 (0–1)	0 (0–0)	10 (9–11)
Tokelau	2036	0 (0–0)	1 (0–1)	0 (0–0)	10 (8–11)
Tonga	2022	1 (1–1)	1 (1–1)	14 (14–15)	18 (17–19)
Tonga	2023	1 (1–1)	1 (1–1)	14 (13–16)	18 (16–19)
Tonga	2024	1 (1–1)	1 (1–1)	14 (13–16)	18 (15–20)
Tonga	2025	1 (1–1)	1 (1–1)	14 (12–16)	18 (15–20)
Tonga	2026	1 (0–1)	1 (1–1)	14 (12–17)	18 (14–21)
Tonga	2027	1 (0–1)	1 (1–1)	14 (12–17)	18 (14–21)
Tonga	2028	1 (0–1)	1 (1–1)	14 (12–17)	18 (14–21)
Tonga	2029	1 (0–1)	1 (1–1)	14 (12–17)	18 (14–22)
Tonga	2030	1 (0–1)	1 (1–1)	14 (11–17)	18 (13–22)
Tonga	2031	1 (0–1)	1 (1–1)	14 (11–18)	18 (13–22)
Tonga	2032	1 (0–1)	1 (1–1)	14 (11–18)	18 (13–22)
Tonga	2033	1 (0–1)	1 (1–1)	14 (11–18)	18 (13–23)
Tonga	2034	1 (0–1)	1 (1–1)	14 (11–18)	18 (12–23)
Tonga	2035	1 (0–1)	1 (1–1)	14 (11–18)	18 (12–23)
Tonga	2036	1 (0–1)	1 (1–1)	14 (10–18)	18 (12–23)

Trinidad and Tobago	2022	8 (8–9)	1 (1–1)	214 (201–228)	11 (10–12)
Trinidad and Tobago	2023	8 (8–9)	1 (1–1)	217 (198–236)	11 (9–12)
Trinidad and Tobago	2024	8 (7–9)	1 (0–1)	219 (196–243)	11 (9–13)
Trinidad and Tobago	2025	8 (7–9)	1 (0–1)	222 (195–249)	11 (8–13)
Trinidad and Tobago	2026	9 (7–10)	1 (0–1)	224 (194–254)	11 (8–13)
Trinidad and Tobago	2027	9 (7–10)	1 (0–1)	227 (194–260)	11 (8–14)
Trinidad and Tobago	2028	9 (7–10)	1 (0–1)	229 (194–265)	11 (8–14)
Trinidad and Tobago	2029	9 (7–10)	1 (0–1)	232 (194–270)	11 (7–14)
Trinidad and Tobago	2030	9 (7–10)	1 (0–1)	234 (194–275)	11 (7–14)
Trinidad and Tobago	2031	9 (7–11)	1 (0–1)	237 (194–280)	11 (7–15)
Trinidad and Tobago	2032	9 (7–11)	1 (0–1)	239 (195–284)	11 (7–15)
Trinidad and Tobago	2033	9 (7–11)	1 (0–1)	242 (195–289)	11 (7–15)
Trinidad and Tobago	2034	9 (7–11)	1 (0–1)	245 (196–293)	11 (6–15)
Trinidad and Tobago	2035	9 (7–11)	1 (0–1)	247 (197–298)	11 (6–15)
Trinidad and Tobago	2036	9 (8–11)	1 (0–1)	250 (197–302)	11 (6–15)
Tunisia	2022	39 (39–40)	1 (0–1)	914 (904–923)	6 (6–7)
Tunisia	2023	40 (40–41)	0 (0–1)	937 (920–953)	6 (6–7)
Tunisia	2024	41 (40–42)	0 (0–1)	958 (932–984)	6 (6–7)
Tunisia	2025	42 (41–43)	0 (0–1)	980 (944–1017)	6 (6–7)
Tunisia	2026	43 (41–45)	0 (0–1)	1002 (953–1051)	6 (6–7)
Tunisia	2027	44 (41–46)	0 (0–1)	1024 (963–1086)	6 (5–7)
Tunisia	2028	44 (42–47)	0 (0–1)	1046 (971–1122)	6 (5–7)
Tunisia	2029	45 (42–49)	0 (0–1)	1068 (978–1159)	6 (5–8)
Tunisia	2030	46 (42–50)	0 (0–1)	1090 (984–1197)	6 (5–8)
Tunisia	2031	47 (42–52)	0 (0–1)	1112 (989–1235)	6 (4–8)
Tunisia	2032	48 (43–53)	0 (0–1)	1134 (994–1274)	6 (4–8)
Tunisia	2033	49 (43–55)	0 (0–1)	1156 (998–1315)	6 (4–8)
Tunisia	2034	50 (43–56)	0 (0–1)	1178 (1001–1355)	6 (4–9)
Tunisia	2035	50 (43–58)	0 (0–1)	1200 (1004–1397)	6 (3–9)
Tunisia	2036	51 (43–60)	0 (0–1)	1222 (1006–1439)	6 (3–9)
Turkey	2022	576 (563–589)	1 (1–1)	13930 (13618–14242)	14 (14–15)
Turkey	2023	582 (557–607)	1 (1–1)	14029 (13383–14674)	14 (13–15)
Turkey	2024	589 (553–625)	1 (1–1)	14108 (13104–15113)	14 (12–15)

Turkey	2025	596 (549–642)	1 (1–1)	14173 (12800–15546)	14 (11–16)
Turkey	2026	603 (547–659)	1 (1–1)	14225 (12484–15966)	14 (10–17)
Turkey	2027	610 (545–676)	1 (1–1)	14268 (12165–16371)	13 (10–17)
Turkey	2028	618 (544–692)	1 (1–1)	14302 (11846–16758)	13 (9–18)
Turkey	2029	625 (544–707)	1 (1–1)	14330 (11532–17128)	13 (8–18)
Turkey	2030	633 (545–721)	1 (1–1)	14353 (11225–17480)	13 (7–19)
Turkey	2031	641 (546–736)	1 (1–2)	14371 (10925–17816)	13 (7–20)
Turkey	2032	648 (547–750)	1 (1–2)	14386 (10634–18137)	13 (6–20)
Turkey	2033	656 (549–763)	1 (1–2)	14398 (10352–18443)	13 (5–21)
Turkey	2034	663 (551–776)	1 (1–2)	14408 (10079–18736)	13 (5–21)
Turkey	2035	671 (553–789)	1 (1–2)	14415 (9814–19017)	13 (4–22)
Turkey	2036	679 (555–802)	1 (1–2)	14422 (9558–19286)	13 (3–23)
Turkmenistan	2022	92 (79–105)	0 (0–0)	2433 (2073–2792)	56 (40–72)
Turkmenistan	2023	94 (73–114)	0 (0–0)	2477 (1906–3047)	55 (29–81)
Turkmenistan	2024	96 (68–123)	0 (0–0)	2521 (1745–3296)	55 (19–90)
Turkmenistan	2025	98 (62–133)	0 (0–0)	2565 (1579–3550)	54 (8–100)
Turkmenistan	2026	99 (56–143)	0 (0–0)	2609 (1405–3812)	53 (-3–110)
Turkmenistan	2027	101 (50–153)	0 (0–0)	2653 (1222–4083)	53 (-15–120)
Turkmenistan	2028	103 (43–164)	0 (0–0)	2697 (1030–4363)	52 (-27–131)
Turkmenistan	2029	105 (35–175)	0 (0–0)	2741 (828–4654)	51 (-40–142)
Turkmenistan	2030	107 (28–186)	0 (0–0)	2785 (616–4953)	51 (-53–154)
Turkmenistan	2031	109 (20–198)	0 (0–0)	2829 (395–5262)	50 (-66–166)
Turkmenistan	2032	111 (12–210)	0 (0–1)	2873 (165–5580)	49 (-80–179)
Turkmenistan	2033	113 (3–223)	0 (0–1)	2917 (-74–5907)	49 (-95–192)
Turkmenistan	2034	115 (-6–236)	0 (0–1)	2961 (-321–6242)	48 (-110–206)
Turkmenistan	2035	117 (-15–249)	0 (0–1)	3005 (-577–6586)	47 (-125–220)
Turkmenistan	2036	119 (-25–262)	0 (0–1)	3049 (-841–6939)	47 (-141–235)
Tuvalu	2022	0 (0–0)	1 (1–1)	2 (2–2)	15 (15–15)
Tuvalu	2023	0 (0–0)	1 (1–1)	2 (2–2)	15 (15–16)
Tuvalu	2024	0 (0–0)	1 (1–1)	2 (2–2)	15 (15–16)
Tuvalu	2025	0 (0–0)	1 (1–1)	2 (2–2)	15 (14–16)
Tuvalu	2026	0 (0–0)	1 (1–1)	2 (2–2)	15 (14–16)
Tuvalu	2027	0 (0–0)	1 (1–1)	2 (2–2)	15 (14–16)

Tuvalu	2028	0 (0–0)	1 (1–1)	2 (2–2)	15 (14–16)
Tuvalu	2029	0 (0–0)	1 (1–1)	2 (2–2)	15 (13–17)
Tuvalu	2030	0 (0–0)	1 (1–1)	2 (2–2)	15 (13–17)
Tuvalu	2031	0 (0–0)	1 (1–1)	2 (2–2)	15 (12–17)
Tuvalu	2032	0 (0–0)	1 (1–1)	2 (2–2)	15 (12–18)
Tuvalu	2033	0 (0–0)	1 (1–1)	2 (2–2)	15 (12–18)
Tuvalu	2034	0 (0–0)	1 (0–1)	2 (2–2)	15 (11–18)
Tuvalu	2035	0 (0–0)	1 (0–1)	2 (2–2)	15 (11–19)
Tuvalu	2036	0 (0–0)	1 (0–1)	2 (2–2)	15 (10–19)
Uganda	2022	210 (206–213)	0 (0–0)	5957 (5858–6055)	36 (35–37)
Uganda	2023	214 (208–221)	0 (0–0)	6109 (5926–6292)	36 (34–38)
Uganda	2024	219 (209–229)	0 (0–0)	6267 (5974–6559)	36 (32–39)
Uganda	2025	224 (209–238)	0 (0–0)	6422 (6007–6837)	35 (31–40)
Uganda	2026	229 (210–248)	0 (0–0)	6579 (6026–7131)	35 (29–41)
Uganda	2027	234 (209–258)	0 (0–0)	6735 (6033–7437)	35 (27–43)
Uganda	2028	239 (209–268)	0 (0–0)	6891 (6028–7754)	35 (25–45)
Uganda	2029	243 (207–279)	0 (0–0)	7047 (6013–8081)	34 (23–46)
Uganda	2030	248 (206–290)	0 (0–0)	7203 (5988–8419)	34 (20–48)
Uganda	2031	253 (204–302)	0 (0–0)	7360 (5954–8766)	34 (18–50)
Uganda	2032	258 (202–314)	0 (0–0)	7516 (5910–9122)	34 (15–52)
Uganda	2033	263 (200–326)	0 (0–0)	7672 (5857–9487)	33 (13–54)
Uganda	2034	268 (197–338)	0 (0–0)	7828 (5797–9860)	33 (10–57)
Uganda	2035	272 (194–351)	0 (0–0)	7985 (5728–10241)	33 (7–59)
Uganda	2036	277 (191–364)	0 (0–0)	8141 (5652–10630)	33 (4–61)
Ukraine	2022	673 (605–740)	1 (1–1)	19150 (17184–21115)	26 (24–29)
Ukraine	2023	660 (565–756)	1 (1–1)	18773 (15994–21553)	26 (22–29)
Ukraine	2024	648 (531–765)	1 (1–1)	18397 (14993–21801)	25 (21–30)
Ukraine	2025	635 (500–770)	1 (1–1)	18021 (14090–21952)	25 (19–30)
Ukraine	2026	623 (471–774)	1 (1–1)	17645 (13250–22039)	24 (18–30)
Ukraine	2027	610 (444–776)	1 (1–1)	17268 (12454–22083)	24 (17–30)
Ukraine	2028	597 (419–776)	1 (1–1)	16892 (11692–22092)	23 (16–30)
Ukraine	2029	585 (394–776)	1 (1–1)	16516 (10957–22075)	23 (15–30)
Ukraine	2030	572 (370–775)	1 (1–1)	16140 (10244–22036)	22 (14–30)

Ukraine	2031	560 (346–774)	1 (1–1)	15763 (9548–21979)	22 (13–30)
Ukraine	2032	547 (323–772)	1 (1–1)	15387 (8869–21906)	21 (12–30)
Ukraine	2033	535 (301–769)	1 (1–1)	15011 (8203–21819)	20 (11–30)
Ukraine	2034	522 (279–766)	1 (1–1)	14635 (7548–21721)	20 (10–29)
Ukraine	2035	510 (257–763)	1 (1–1)	14259 (6905–21612)	19 (10–29)
Ukraine	2036	497 (236–759)	1 (1–1)	13882 (6270–21494)	19 (9–29)
United Arab Emirates	2022	12 (11–13)	1 (1–1)	384 (355–413)	11 (9–13)
United Arab Emirates	2023	12 (11–13)	1 (1–1)	394 (363–425)	11 (8–15)
United Arab Emirates	2024	13 (12–14)	1 (1–1)	404 (372–437)	12 (8–16)
United Arab Emirates	2025	13 (12–14)	1 (1–1)	414 (380–448)	13 (8–17)
United Arab Emirates	2026	13 (12–15)	1 (0–1)	424 (388–460)	13 (8–18)
United Arab Emirates	2027	14 (12–15)	1 (0–1)	434 (397–471)	14 (8–19)
United Arab Emirates	2028	14 (13–15)	1 (0–1)	444 (405–483)	14 (9–19)
United Arab Emirates	2029	14 (13–16)	1 (0–1)	454 (414–494)	14 (9–20)
United Arab Emirates	2030	15 (13–16)	1 (0–1)	464 (422–505)	15 (9–20)
United Arab Emirates	2031	15 (13–16)	1 (0–1)	474 (431–516)	15 (9–20)
United Arab Emirates	2032	15 (14–17)	1 (0–1)	484 (440–527)	15 (9–21)
United Arab Emirates	2033	16 (14–17)	1 (0–1)	494 (449–539)	15 (9–21)
United Arab Emirates	2034	16 (14–17)	1 (0–1)	503 (457–550)	15 (9–21)
United Arab Emirates	2035	16 (15–18)	1 (0–1)	513 (466–561)	15 (10–21)
United Arab Emirates	2036	16 (15–18)	1 (0–1)	523 (475–572)	15 (10–21)
United Kingdom	2022	3897 (3791–4003)	1 (1–1)	70137 (67732–72541)	54 (52–57)
United Kingdom	2023	3892 (3674–4109)	1 (1–1)	69257 (65453–73061)	52 (48–57)
United Kingdom	2024	3888 (3616–4159)	1 (1–1)	68377 (63215–73540)	51 (45–57)
United Kingdom	2025	3884 (3578–4190)	1 (1–1)	67498 (60946–74049)	49 (41–57)
United Kingdom	2026	3881 (3552–4209)	1 (1–1)	66618 (58627–74609)	47 (38–57)
United Kingdom	2027	3878 (3534–4222)	1 (1–1)	65738 (56248–75228)	45 (34–57)
United Kingdom	2028	3876 (3520–4231)	1 (1–1)	64859 (53808–75909)	44 (30–57)
United Kingdom	2029	3874 (3510–4237)	1 (1–1)	63979 (51305–76653)	42 (26–58)
United Kingdom	2030	3872 (3503–4241)	1 (1–1)	63099 (48741–77458)	40 (22–58)
United Kingdom	2031	3870 (3497–4244)	1 (1–1)	62220 (46116–78323)	38 (18–59)
United Kingdom	2032	3869 (3493–4245)	1 (1–1)	61340 (43432–79248)	37 (14–60)
United Kingdom	2033	3868 (3490–4247)	1 (1–1)	60460 (40690–80230)	35 (9–61)

United Kingdom	2034	3867 (3487–4247)	1 (1–1)	59581 (37891–81270)	33 (5–62)
United Kingdom	2035	3866 (3485–4248)	1 (1–1)	58701 (35037–82364)	31 (0–63)
United Kingdom	2036	3866 (3483–4248)	1 (1–1)	57821 (32129–83513)	30 (-4–64)
United Republic of Tanzania	2022	375 (372–378)	0 (0–0)	10475 (10383–10567)	37 (37–38)
United Republic of Tanzania	2023	376 (369–383)	0 (0–0)	10553 (10354–10751)	37 (36–39)
United Republic of Tanzania	2024	374 (363–384)	0 (0–0)	10542 (10226–10858)	37 (34–39)
United Republic of Tanzania	2025	369 (354–383)	0 (0–0)	10446 (10012–10880)	37 (33–40)
United Republic of Tanzania	2026	361 (343–380)	0 (0–0)	10273 (9730–10817)	36 (32–41)
United Republic of Tanzania	2027	352 (331–374)	0 (0–0)	10037 (9398–10675)	36 (30–42)
United Republic of Tanzania	2028	342 (318–366)	0 (0–0)	9754 (9040–10468)	36 (29–43)
United Republic of Tanzania	2029	331 (306–357)	0 (0–0)	9445 (8678–10213)	36 (27–45)
United Republic of Tanzania	2030	321 (295–348)	0 (0–0)	9132 (8332–9933)	36 (26–46)
United Republic of Tanzania	2031	312 (285–339)	0 (0–0)	8837 (8021–9652)	36 (24–47)
United Republic of Tanzania	2032	305 (278–332)	0 (0–0)	8578 (7759–9397)	35 (22–49)
United Republic of Tanzania	2033	300 (273–327)	0 (0–0)	8373 (7554–9193)	35 (21–50)
United Republic of Tanzania	2034	298 (270–325)	0 (0–0)	8236 (7412–9059)	35 (19–51)
United Republic of Tanzania	2035	298 (270–326)	0 (0–0)	8173 (7334–9012)	35 (17–53)
United Republic of Tanzania	2036	301 (272–330)	0 (0–0)	8187 (7320–9054)	35 (16–54)
United States of America	2022	10634 (10467–10801	1 (1–1)	234834 (230592–239077	40 (39–41)
United States of America	2023	10749 (10460–11039	1 (1–1)	236397 (228519–244275	39 (37–41)
United States of America	2024	10865 (10491–11239	1 (1–1)	237960 (227292–248628	38 (35–41)
United States of America	2025	10980 (10538–11423	1 (1–1)	239522 (226339–252706	38 (34–42)
United States of America	2026	11096 (10594–11598	1 (1–1)	241085 (225506–256664	37 (32–42)
United States of America	2027	11211 (10657–11766	1 (1–1)	242648 (224729–260566	36 (31–42)
United States of America	2028	11327 (10724–11930	1 (1–1)	244210 (223973–264447	36 (29–42)
United States of America	2029	11443 (10795–12090	1 (1–1)	245773 (223218–268327	35 (27–43)
United States of America	2030	11558 (10868–12248	1 (1–1)	247336 (222452–272219	34 (26–43)
United States of America	2031	11674 (10944–12403	1 (1–1)	248898 (221666–276130	34 (24–44)
United States of America	2032	11789 (11022–12556	1 (1–1)	250461 (220855–280067	33 (22–44)
United States of America	2033	11905 (11102–12707	1 (1–1)	252024 (220014–284033	32 (20–45)
United States of America	2034	12020 (11184–12857	1 (1–1)	253586 (219142–288031	32 (18–45)
United States of America	2035	12136 (11266–13005	1 (1–1)	255149 (218236–292062	31 (16–46)
United States of America	2036	12251 (11350–13152	1 (1–1)	256712 (217294–296129	30 (14–46)

United States Virgin Islands	2022	1 (1–1)	0 (0–0)	19 (18–21)	11 (10–12)
United States Virgin Islands	2023	1 (1–1)	0 (0–0)	19 (16–21)	11 (10–12)
United States Virgin Islands	2024	1 (1–1)	0 (0–0)	18 (15–22)	11 (9–12)
United States Virgin Islands	2025	1 (1–1)	0 (0–1)	17 (13–22)	10 (8–12)
United States Virgin Islands	2026	1 (1–1)	0 (0–1)	17 (11–22)	10 (8–12)
United States Virgin Islands	2027	1 (1–1)	0 (0–1)	16 (10–22)	10 (7–12)
United States Virgin Islands	2028	1 (1–1)	0 (0–1)	15 (8–23)	9 (7–12)
United States Virgin Islands	2029	1 (0–1)	0 (0–1)	15 (6–23)	9 (7–12)
United States Virgin Islands	2030	1 (0–1)	0 (0–1)	14 (4–24)	9 (6–12)
United States Virgin Islands	2031	1 (0–1)	0 (0–1)	13 (2–24)	9 (6–11)
United States Virgin Islands	2032	1 (0–1)	0 (0–1)	13 (0–25)	8 (5–11)
United States Virgin Islands	2033	1 (0–1)	0 (0–1)	12 (-2–26)	8 (5–11)
United States Virgin Islands	2034	1 (0–1)	0 (0–1)	11 (-4–26)	8 (4–11)
United States Virgin Islands	2035	1 (0–1)	0 (0–1)	11 (-6–27)	7 (4–11)
United States Virgin Islands	2036	1 (0–1)	0 (0–1)	10 (-8–28)	7 (4–11)
Uruguay	2022	96 (87–105)	2 (2–2)	2142 (1938–2346)	41 (36–46)
Uruguay	2023	96 (84–109)	2 (2–2)	2142 (1854–2431)	40 (33–47)
Uruguay	2024	96 (81–112)	2 (2–2)	2142 (1789–2495)	39 (31–47)
Uruguay	2025	96 (79–114)	2 (2–2)	2142 (1735–2550)	38 (28–47)
Uruguay	2026	96 (77–116)	2 (2–2)	2142 (1686–2598)	37 (26–47)
Uruguay	2027	96 (75–118)	2 (2–2)	2142 (1643–2642)	36 (24–47)
Uruguay	2028	96 (73–120)	2 (2–2)	2142 (1603–2682)	35 (22–47)
Uruguay	2029	96 (71–121)	2 (2–2)	2142 (1566–2719)	33 (20–47)
Uruguay	2030	96 (70–123)	2 (2–2)	2142 (1531–2754)	32 (18–47)
Uruguay	2031	96 (68–124)	2 (2–2)	2142 (1498–2787)	31 (16–46)
Uruguay	2032	96 (67–125)	2 (2–2)	2142 (1466–2818)	30 (15–46)
Uruguay	2033	96 (66–127)	2 (2–2)	2142 (1436–2848)	29 (13–46)
Uruguay	2034	96 (64–128)	2 (2–2)	2142 (1407–2877)	28 (11–45)
Uruguay	2035	96 (63–129)	2 (1–2)	2142 (1380–2905)	27 (9–45)
Uruguay	2036	96 (62–130)	2 (1–2)	2142 (1353–2932)	26 (8–44)
Uzbekistan	2022	177 (151–203)	0 (0–0)	4859 (4108–5611)	16 (11–21)
Uzbekistan	2023	177 (127–227)	0 (0–0)	4859 (3479–6240)	15 (6–24)
Uzbekistan	2024	177 (111–242)	0 (0–0)	4859 (3058–6661)	14 (2–25)

Uzbekistan	2025	177 (99–255)	0 (0–0)	4859 (2718–7001)	12 (-2–26)
Uzbekistan	2026	177 (88–266)	0 (0–0)	4859 (2425–7294)	11 (-5–27)
Uzbekistan	2027	177 (78–275)	0 (0–0)	4859 (2164–7555)	10 (-8–27)
Uzbekistan	2028	177 (70–284)	0 (0–0)	4859 (1926–7793)	8 (-11–28)
Uzbekistan	2029	177 (61–292)	0 (0–0)	4859 (1705–8014)	7 (-14–28)
Uzbekistan	2030	177 (54–300)	0 (0–0)	4859 (1500–8219)	6 (-16–28)
Uzbekistan	2031	177 (47–307)	0 (0–0)	4859 (1306–8413)	4 (-19–28)
Uzbekistan	2032	177 (40–314)	0 (0–0)	4859 (1122–8597)	3 (-21–28)
Uzbekistan	2033	177 (33–320)	0 (0–0)	4859 (947–8772)	2 (-24–28)
Uzbekistan	2034	177 (27–327)	0 (0–0)	4859 (779–8940)	1 (-26–27)
Uzbekistan	2035	177 (21–332)	0 (0–0)	4859 (618–9101)	-1 (-29–27)
Uzbekistan	2036	177 (16–338)	0 (0–0)	4859 (463–9256)	-2 (-31–27)
Vanuatu	2022	1 (1–1)	0 (0–0)	21 (21–21)	10 (10–11)
Vanuatu	2023	1 (1–1)	0 (0–0)	21 (21–22)	10 (10–11)
Vanuatu	2024	1 (1–1)	0 (0–0)	22 (21–22)	10 (10–11)
Vanuatu	2025	1 (1–1)	0 (0–0)	22 (21–23)	10 (9–11)
Vanuatu	2026	1 (1–1)	0 (0–0)	22 (22–23)	10 (9–11)
Vanuatu	2027	1 (1–1)	0 (0–0)	23 (22–24)	10 (9–11)
Vanuatu	2028	1 (1–1)	0 (0–0)	23 (22–24)	10 (9–11)
Vanuatu	2029	1 (1–1)	0 (0–0)	23 (22–25)	9 (8–11)
Vanuatu	2030	1 (1–1)	0 (0–0)	24 (23–25)	9 (8–10)
Vanuatu	2031	1 (1–1)	0 (0–0)	24 (23–25)	9 (8–10)
Vanuatu	2032	1 (1–1)	0 (0–0)	24 (23–26)	9 (8–10)
Vanuatu	2033	1 (1–1)	0 (0–0)	25 (24–26)	9 (8–10)
Vanuatu	2034	1 (1–1)	0 (0–0)	25 (24–26)	9 (7–10)
Vanuatu	2035	1 (1–1)	0 (0–0)	26 (24–27)	9 (7–10)
Vanuatu	2036	1 (1–1)	0 (0–0)	26 (24–27)	9 (7–10)
Venezuela (Bolivarian Republic of)	2022	90 (84–95)	0 (0–1)	2182 (2054–2311)	7 (6–7)
Venezuela (Bolivarian Republic of)	2023	91 (85–97)	0 (0–1)	2203 (2071–2336)	6 (5–7)
Venezuela (Bolivarian Republic of)	2024	92 (85–98)	0 (0–1)	2224 (2087–2361)	6 (5–7)
Venezuela (Bolivarian Republic of)	2025	93 (86–99)	0 (0–1)	2245 (2104–2387)	6 (5–7)
Venezuela (Bolivarian Republic of)	2026	94 (86–101)	0 (0–1)	2266 (2121–2412)	5 (4–7)
Venezuela (Bolivarian Republic of)	2027	94 (87–102)	0 (0–1)	2287 (2138–2437)	5 (4–6)

Venezuela (Bolivarian Republic of)	2028	95 (88–103)	0 (0–1)	2308 (2155–2461)	5 (4–6)
Venezuela (Bolivarian Republic of)	2029	96 (88–104)	0 (0–1)	2329 (2172–2486)	5 (3–6)
Venezuela (Bolivarian Republic of)	2030	97 (89–106)	0 (0–1)	2350 (2190–2511)	4 (3–6)
Venezuela (Bolivarian Republic of)	2031	98 (89–107)	0 (0–1)	2371 (2207–2535)	4 (2–6)
Venezuela (Bolivarian Republic of)	2032	99 (90–108)	0 (0–1)	2392 (2224–2560)	4 (2–5)
Venezuela (Bolivarian Republic of)	2033	100 (91–109)	0 (0–1)	2413 (2242–2584)	3 (2–5)
Venezuela (Bolivarian Republic of)	2034	101 (91–110)	0 (0–1)	2434 (2259–2609)	3 (1–5)
Venezuela (Bolivarian Republic of)	2035	102 (92–111)	0 (0–1)	2455 (2277–2633)	3 (1–5)
Venezuela (Bolivarian Republic of)	2036	103 (92–113)	0 (0–1)	2476 (2295–2657)	3 (1–4)
Viet Nam	2022	973 (964–982)	0 (0–0)	26921 (26663–27179)	24 (24–24)
Viet Nam	2023	1000 (983–1017)	0 (0–0)	27588 (27084–28092)	24 (23–25)
Viet Nam	2024	1027 (1001–1053)	0 (0–0)	28255 (27465–29044)	24 (23–25)
Viet Nam	2025	1055 (1018–1091)	0 (0–0)	28922 (27810–30033)	24 (23–25)
Viet Nam	2026	1082 (1035–1129)	0 (0–0)	29588 (28121–31056)	24 (23–25)
Viet Nam	2027	1109 (1050–1168)	0 (0–0)	30255 (28401–32109)	24 (22–25)
Viet Nam	2028	1136 (1065–1208)	0 (0–0)	30922 (28653–33191)	24 (22–26)
Viet Nam	2029	1164 (1078–1249)	0 (0–0)	31589 (28878–34299)	24 (22–26)
Viet Nam	2030	1191 (1091–1290)	0 (0–0)	32255 (29078–35433)	24 (22–26)
Viet Nam	2031	1218 (1104–1333)	0 (0–0)	32922 (29254–36591)	24 (22–26)
Viet Nam	2032	1245 (1115–1375)	0 (0–0)	33589 (29407–37771)	24 (22–26)
Viet Nam	2033	1273 (1126–1419)	0 (0–0)	34256 (29538–38973)	24 (22–26)
Viet Nam	2034	1300 (1137–1463)	0 (0–0)	34923 (29649–40197)	24 (22–26)
Viet Nam	2035	1327 (1147–1508)	0 (0–0)	35589 (29738–41440)	24 (22–26)
Viet Nam	2036	1354 (1156–1553)	0 (0–0)	36256 (29809–42703)	24 (21–26)
Yemen	2022	230 (226–233)	0 (0–0)	6071 (5966–6176)	40 (40–41)
Yemen	2023	234 (227–241)	0 (0–0)	6186 (5986–6387)	40 (38–42)
Yemen	2024	238 (228–248)	0 (0–0)	6294 (6002–6585)	40 (37–43)
Yemen	2025	242 (229–254)	0 (0–0)	6397 (6022–6772)	40 (36–44)
Yemen	2026	246 (231–261)	0 (0–0)	6497 (6045–6949)	40 (35–45)
Yemen	2027	249 (232–267)	0 (0–0)	6595 (6073–7118)	40 (34–46)
Yemen	2028	253 (234–273)	0 (0–0)	6693 (6106–7280)	40 (33–47)
Yemen	2029	257 (236–278)	0 (0–0)	6790 (6142–7437)	40 (32–48)
Yemen	2030	261 (238–284)	0 (0–0)	6886 (6183–7589)	40 (30–49)

Yemen	2031	265 (240–290)	0 (0–0)	6982 (6227–7737)	40 (29–50)
Yemen	2032	269 (242–295)	0 (0–0)	7078 (6274–7882)	40 (28–51)
Yemen	2033	272 (244–300)	0 (0–0)	7174 (6323–8024)	40 (28–52)
Yemen	2034	276 (247–306)	0 (0–0)	7269 (6375–8164)	40 (27–53)
Yemen	2035	280 (249–311)	0 (0–0)	7365 (6429–8302)	40 (26–53)
Yemen	2036	284 (252–316)	0 (0–0)	7461 (6484–8438)	40 (25–54)
Zambia	2022	126 (124–128)	0 (0–0)	3555 (3507–3603)	47 (46–48)
Zambia	2023	126 (122–131)	0 (0–0)	3590 (3484–3696)	46 (43–48)
Zambia	2024	127 (120–133)	0 (0–0)	3623 (3448–3798)	45 (40–49)
Zambia	2025	127 (117–137)	0 (0–0)	3655 (3403–3907)	44 (37–50)
Zambia	2026	127 (114–140)	0 (0–0)	3686 (3349–4022)	43 (34–51)
Zambia	2027	127 (111–143)	0 (0–0)	3715 (3289–4141)	42 (31–52)
Zambia	2028	128 (108–147)	0 (0–0)	3743 (3222–4264)	40 (27–54)
Zambia	2029	128 (104–151)	0 (0–0)	3770 (3151–4389)	39 (23–56)
Zambia	2030	128 (101–155)	0 (0–0)	3796 (3074–4517)	38 (19–57)
Zambia	2031	128 (97–159)	0 (0–0)	3821 (2994–4647)	37 (15–59)
Zambia	2032	128 (94–163)	0 (0–0)	3844 (2911–4778)	36 (11–62)
Zambia	2033	128 (90–167)	0 (0–0)	3867 (2824–4911)	35 (6–64)
Zambia	2034	129 (86–171)	0 (0–0)	3889 (2734–5044)	34 (2–66)
Zambia	2035	129 (82–175)	0 (0–0)	3910 (2643–5178)	33 (-3–69)
Zambia	2036	129 (78–179)	0 (0–0)	3930 (2549–5312)	32 (-8–72)
Zimbabwe	2022	257 (251–264)	1 (1–1)	7320 (7147–7492)	97 (94–100)
Zimbabwe	2023	255 (240–270)	1 (1–1)	7338 (6979–7698)	97 (91–103)
Zimbabwe	2024	252 (227–277)	1 (1–1)	7354 (6790–7917)	97 (88–107)
Zimbabwe	2025	249 (213–286)	1 (1–1)	7366 (6591–8142)	98 (86–111)
Zimbabwe	2026	247 (198–296)	1 (1–1)	7377 (6388–8366)	100 (85–114)
Zimbabwe	2027	244 (181–308)	1 (1–1)	7386 (6185–8587)	101 (85–118)
Zimbabwe	2028	242 (163–320)	1 (1–1)	7393 (5984–8802)	103 (85–121)
Zimbabwe	2029	239 (144–334)	1 (1–1)	7399 (5787–9011)	105 (86–123)
Zimbabwe	2030	236 (124–349)	1 (1–1)	7404 (5595–9214)	106 (86–125)
Zimbabwe	2031	234 (104–364)	1 (1–1)	7408 (5408–9409)	107 (87–127)
Zimbabwe	2032	231 (82–381)	1 (1–1)	7412 (5226–9597)	108 (88–128)
Zimbabwe	2033	229 (59–398)	1 (1–1)	7415 (5050–9779)	109 (89–129)

Zimbabwe	2034	226 (36–416)	1 (1–1)	7417 (4880–9954)	110 (90–130)
Zimbabwe	2035	223 (12–435)	1 (1–1)	7419 (4715–10123)	110 (90–130)
Zimbabwe	2036	221 (-13–455)	1 (1–1)	7420 (4556–10285)	110 (90–130)

Supplement table.4 Disease burden prediction for stomach cancer death and disability-adjusted life years numbers and age-standardized rate to 2036 in different countries.

Country	Year	Death		DALY	
		(N)	ASR(per100,000)	(N)	ASR(per100,000)
Afghanistan	2022	112 (110–113)	1 (1–1)	2922 (2880–2964)	29 (28–29)
Afghanistan	2023	110 (106–113)	1 (1–1)	2940 (2851–3028)	28 (27–30)
Afghanistan	2024	108 (102–114)	1 (1–1)	2955 (2814–3095)	28 (27–30)
Afghanistan	2025	106 (97–115)	1 (1–1)	2968 (2772–3163)	28 (26–31)
Afghanistan	2026	104 (92–116)	1 (1–1)	2979 (2726–3232)	29 (26–32)
Afghanistan	2027	102 (86–118)	1 (1–1)	2988 (2678–3299)	29 (25–33)
Afghanistan	2028	100 (81–119)	1 (1–1)	2997 (2629–3365)	30 (25–34)
Afghanistan	2029	98 (75–121)	1 (1–1)	3004 (2579–3429)	30 (25–35)
Afghanistan	2030	96 (69–124)	1 (1–1)	3010 (2529–3492)	31 (26–37)
Afghanistan	2031	94 (62–126)	1 (1–2)	3016 (2479–3552)	32 (26–38)
Afghanistan	2032	92 (55–129)	1 (1–2)	3020 (2430–3611)	33 (27–39)
Afghanistan	2033	90 (49–132)	1 (1–2)	3025 (2381–3668)	34 (28–41)
Afghanistan	2034	88 (42–135)	1 (1–2)	3028 (2333–3723)	35 (28–42)
Afghanistan	2035	86 (34–139)	1 (1–2)	3031 (2285–3777)	36 (29–43)
Afghanistan	2036	84 (27–142)	2 (1–2)	3034 (2239–3828)	37 (30–44)
Albania	2022	94 (90–98)	2 (2–2)	777 (738–815)	42 (39–46)
Albania	2023	95 (87–103)	2 (2–2)	776 (706–846)	41 (35–48)
Albania	2024	96 (86–106)	2 (2–2)	776 (677–875)	40 (32–49)
Albania	2025	97 (85–109)	2 (1–2)	776 (652–900)	40 (30–50)
Albania	2026	98 (84–111)	2 (1–2)	776 (630–922)	39 (27–50)
Albania	2027	99 (84–114)	2 (1–2)	776 (610–942)	38 (25–51)
Albania	2028	100 (84–116)	2 (1–2)	776 (592–960)	37 (23–51)
Albania	2029	101 (84–118)	2 (1–2)	776 (575–976)	36 (21–51)
Albania	2030	102 (83–120)	2 (1–2)	776 (560–992)	35 (19–51)
Albania	2031	103 (83–123)	2 (1–2)	776 (545–1006)	34 (18–51)
Albania	2032	104 (83–125)	2 (1–2)	776 (532–1020)	34 (16–51)
Albania	2033	105 (83–127)	2 (1–2)	776 (519–1033)	33 (14–51)
Albania	2034	106 (83–128)	2 (1–2)	776 (507–1045)	32 (13–51)
Albania	2035	107 (84–130)	2 (1–2)	776 (495–1057)	31 (11–51)
Albania	2036	108 (84–132)	2 (1–2)	776 (484–1068)	30 (9–51)
Algeria	2022	128 (126–130)	0 (0–0)	2090 (2070–2110)	8 (8–8)

Algeria	2023	129 (125–133)	0 (0–0)	2144 (2099–2189)	8 (8–8)
Algeria	2024	130 (124–137)	0 (0–0)	2198 (2123–2273)	8 (7–9)
Algeria	2025	131 (122–141)	0 (0–0)	2253 (2143–2362)	8 (7–9)
Algeria	2026	133 (119–146)	0 (0–0)	2307 (2158–2455)	8 (7–9)
Algeria	2027	134 (117–151)	0 (0–0)	2361 (2170–2552)	8 (6–9)
Algeria	2028	135 (114–156)	0 (0–0)	2415 (2178–2652)	8 (6–9)
Algeria	2029	136 (111–162)	0 (0–0)	2470 (2184–2756)	7 (5–10)
Algeria	2030	138 (107–168)	0 (0–0)	2524 (2186–2862)	7 (5–10)
Algeria	2031	139 (104–174)	0 (0–1)	2578 (2185–2971)	7 (4–10)
Algeria	2032	140 (100–180)	0 (0–1)	2633 (2182–3083)	7 (4–11)
Algeria	2033	141 (96–187)	0 (0–1)	2687 (2176–3198)	7 (3–11)
Algeria	2034	143 (91–194)	0 (0–1)	2741 (2168–3314)	7 (3–11)
Algeria	2035	144 (87–201)	0 (0–1)	2795 (2157–3434)	7 (2–12)
Algeria	2036	145 (82–208)	0 (0–1)	2850 (2144–3555)	7 (2–12)
American Samoa	2022	1 (1–1)	1 (1–1)	6 (6–6)	32 (31–34)
American Samoa	2023	1 (1–1)	1 (1–1)	6 (5–6)	32 (30–34)
American Samoa	2024	1 (1–1)	1 (1–1)	6 (5–7)	32 (29–34)
American Samoa	2025	1 (1–1)	1 (1–1)	6 (6–7)	31 (28–34)
American Samoa	2026	1 (1–1)	1 (1–1)	6 (6–7)	31 (27–34)
American Samoa	2027	1 (1–1)	1 (1–1)	6 (6–7)	30 (27–34)
American Samoa	2028	1 (1–1)	1 (1–1)	6 (6–7)	30 (26–34)
American Samoa	2029	1 (1–1)	1 (1–1)	7 (6–8)	30 (25–34)
American Samoa	2030	1 (1–1)	1 (1–1)	7 (6–8)	29 (24–34)
American Samoa	2031	1 (1–1)	1 (1–1)	7 (6–8)	29 (24–34)
American Samoa	2032	1 (1–1)	1 (1–1)	7 (6–8)	28 (23–34)
American Samoa	2033	1 (1–1)	1 (1–1)	7 (6–8)	28 (22–33)
American Samoa	2034	1 (1–1)	1 (1–1)	7 (6–8)	28 (22–33)
American Samoa	2035	1 (1–1)	1 (1–1)	7 (6–8)	27 (21–33)
American Samoa	2036	1 (1–1)	1 (1–1)	7 (6–9)	27 (21–33)
Andorra	2022	1 (1–1)	1 (1–1)	17 (15–19)	13 (12–15)
Andorra	2023	1 (1–1)	1 (0–1)	17 (15–20)	13 (10–15)
Andorra	2024	1 (1–1)	1 (0–1)	17 (14–20)	12 (8–15)
Andorra	2025	1 (1–1)	1 (0–1)	17 (14–21)	11 (7–15)

Andorra	2026	1 (1–1)	0 (0–1)	17 (13–21)	10 (5–15)
Andorra	2027	1 (1–1)	0 (0–1)	17 (13–21)	9 (4–15)
Andorra	2028	1 (1–1)	0 (0–1)	17 (12–22)	9 (3–14)
Andorra	2029	1 (1–1)	0 (0–1)	17 (12–22)	8 (2–14)
Andorra	2030	1 (1–1)	0 (0–1)	17 (12–22)	7 (0–14)
Andorra	2031	1 (1–1)	0 (-0–1)	17 (12–23)	6 (-1–13)
Andorra	2032	1 (1–1)	0 (-0–1)	17 (11–23)	5 (-2–13)
Andorra	2033	1 (1–1)	0 (-0–1)	17 (11–23)	5 (-3–12)
Andorra	2034	1 (1–1)	0 (-0–1)	17 (11–23)	4 (-4–12)
Andorra	2035	1 (1–1)	0 (-0–1)	17 (11–24)	3 (-5–11)
Andorra	2036	1 (1–1)	0 (-0–1)	17 (10–24)	2 (-6–11)
Angola	2022	48 (47–49)	0 (0–0)	4942 (4846–5037)	10 (10–11)
Angola	2023	49 (47–51)	0 (0–0)	5120 (4941–5299)	10 (9–11)
Angola	2024	50 (48–53)	0 (0–0)	5298 (5026–5571)	10 (9–11)
Angola	2025	52 (48–55)	0 (0–0)	5477 (5099–5854)	10 (9–11)
Angola	2026	53 (49–58)	0 (0–0)	5655 (5163–6147)	10 (8–12)
Angola	2027	55 (49–60)	0 (0–0)	5833 (5217–6450)	10 (8–12)
Angola	2028	56 (49–63)	0 (0–0)	6012 (5262–6761)	10 (7–12)
Angola	2029	57 (49–66)	0 (0–0)	6190 (5299–7080)	10 (7–13)
Angola	2030	59 (49–68)	0 (0–0)	6368 (5329–7408)	10 (6–13)
Angola	2031	60 (49–71)	0 (0–1)	6547 (5351–7742)	10 (6–14)
Angola	2032	61 (49–74)	0 (0–1)	6725 (5366–8084)	10 (5–14)
Angola	2033	63 (49–77)	0 (0–1)	6903 (5374–8433)	10 (4–15)
Angola	2034	64 (49–80)	0 (0–1)	7082 (5376–8788)	9 (4–15)
Angola	2035	66 (49–83)	0 (0–1)	7260 (5371–9149)	9 (3–16)
Angola	2036	67 (48–86)	0 (0–1)	7438 (5360–9516)	9 (2–16)
Antigua and Barbuda	2022	1 (0–1)	1 (0–1)	14 (13–15)	11 (10–13)
Antigua and Barbuda	2023	1 (0–1)	0 (0–1)	14 (12–16)	11 (8–13)
Antigua and Barbuda	2024	1 (0–1)	0 (0–1)	14 (12–17)	10 (8–13)
Antigua and Barbuda	2025	1 (0–1)	0 (0–1)	14 (11–17)	10 (7–13)
Antigua and Barbuda	2026	1 (0–1)	0 (0–1)	14 (11–17)	10 (6–13)
Antigua and Barbuda	2027	1 (0–1)	0 (0–1)	14 (11–18)	9 (5–13)
Antigua and Barbuda	2028	1 (0–1)	0 (0–1)	14 (10–18)	9 (5–13)

Antigua and Barbuda	2029	1 (0–1)	0 (0–1)	14 (10–18)	8 (4–13)
Antigua and Barbuda	2030	1 (0–1)	0 (0–1)	14 (10–19)	8 (3–13)
Antigua and Barbuda	2031	1 (0–1)	0 (0–1)	14 (10–19)	8 (3–13)
Antigua and Barbuda	2032	1 (0–1)	0 (0–1)	14 (9–19)	7 (2–12)
Antigua and Barbuda	2033	1 (0–1)	0 (0–1)	14 (9–19)	7 (1–12)
Antigua and Barbuda	2034	1 (0–1)	0 (0–1)	14 (9–20)	6 (1–12)
Antigua and Barbuda	2035	1 (0–1)	0 (0–1)	14 (9–20)	6 (0–12)
Antigua and Barbuda	2036	1 (0–1)	0 (-0–1)	14 (9–20)	6 (-1–12)
Argentina	2022	369 (350–387)	1 (1–1)	14819 (13840–15798)	17 (16–18)
Argentina	2023	361 (328–394)	1 (1–1)	14413 (12664–16161)	16 (14–18)
Argentina	2024	357 (311–403)	1 (0–1)	14006 (11618–16393)	16 (13–18)
Argentina	2025	355 (297–413)	1 (0–1)	13599 (10608–16590)	15 (11–18)
Argentina	2026	354 (286–422)	1 (0–1)	13192 (9606–16779)	14 (10–18)
Argentina	2027	353 (276–430)	1 (0–1)	12786 (8601–16970)	14 (9–18)
Argentina	2028	353 (268–438)	0 (0–1)	12379 (7589–17169)	13 (8–18)
Argentina	2029	353 (260–446)	0 (0–1)	11972 (6565–17379)	12 (7–18)
Argentina	2030	353 (253–453)	0 (0–1)	11565 (5529–17602)	12 (6–18)
Argentina	2031	353 (246–459)	0 (0–1)	11158 (4478–17839)	11 (5–17)
Argentina	2032	353 (240–465)	0 (0–1)	10752 (3413–18090)	10 (4–17)
Argentina	2033	353 (234–471)	0 (0–1)	10345 (2333–18357)	10 (3–17)
Argentina	2034	353 (229–477)	0 (0–1)	9938 (1238–18638)	9 (2–16)
Argentina	2035	353 (223–482)	0 (0–1)	9531 (129–18934)	9 (1–16)
Argentina	2036	353 (218–487)	0 (0–1)	9125 (-996–19245)	8 (0–16)
Armenia	2022	78 (71–85)	2 (2–2)	529 (464–594)	40 (34–45)
Armenia	2023	78 (68–88)	2 (1–2)	533 (432–634)	38 (30–46)
Armenia	2024	78 (66–90)	2 (1–2)	537 (402–672)	36 (27–45)
Armenia	2025	78 (64–92)	2 (1–2)	541 (372–710)	34 (23–45)
Armenia	2026	78 (63–94)	1 (1–2)	545 (341–749)	32 (20–44)
Armenia	2027	78 (61–95)	1 (1–2)	549 (308–790)	30 (17–44)
Armenia	2028	78 (60–97)	1 (1–2)	553 (275–831)	28 (14–43)
Armenia	2029	78 (58–98)	1 (1–2)	557 (240–874)	27 (11–42)
Armenia	2030	78 (57–99)	1 (1–2)	561 (204–919)	25 (9–41)
Armenia	2031	78 (56–100)	1 (0–2)	565 (166–964)	23 (6–40)

Armenia	2032	78 (55–101)	1 (0–2)	569 (128–1011)	21 (3–39)
Armenia	2033	78 (54–102)	1 (0–2)	573 (88–1059)	19 (0–38)
Armenia	2034	78 (53–103)	1 (0–2)	577 (46–1108)	17 (-2–37)
Armenia	2035	78 (52–104)	1 (0–2)	581 (4–1159)	15 (-5–35)
Armenia	2036	78 (51–105)	1 (0–2)	585 (-40–1210)	13 (-8–34)
Australia	2022	97 (93–100)	0 (0–0)	8093 (7778–8407)	5 (5–5)
Australia	2023	97 (93–100)	0 (0–0)	8131 (7754–8508)	5 (4–5)
Australia	2024	97 (92–101)	0 (0–0)	8118 (7669–8567)	5 (4–5)
Australia	2025	97 (91–102)	0 (0–0)	8123 (7617–8628)	4 (3–5)
Australia	2026	97 (90–104)	0 (0–0)	8121 (7563–8679)	4 (3–6)
Australia	2027	97 (88–105)	0 (0–0)	8122 (7516–8727)	4 (2–6)
Australia	2028	97 (87–107)	0 (0–0)	8121 (7472–8771)	4 (2–6)
Australia	2029	97 (85–108)	0 (0–0)	8122 (7431–8812)	4 (1–6)
Australia	2030	97 (83–110)	0 (0–0)	8121 (7392–8851)	4 (0–7)
Australia	2031	97 (81–112)	0 (-0–0)	8121 (7355–8888)	3 (-0–7)
Australia	2032	97 (79–114)	0 (-0–0)	8121 (7320–8923)	3 (-1–7)
Australia	2033	97 (77–116)	0 (-0–0)	8121 (7286–8957)	3 (-2–8)
Australia	2034	97 (75–119)	0 (-0–0)	8121 (7254–8989)	3 (-2–8)
Australia	2035	97 (73–121)	0 (-0–0)	8121 (7222–9021)	3 (-3–8)
Australia	2036	97 (70–124)	0 (-0–0)	8121 (7192–9051)	3 (-4–9)
Austria	2022	85 (80–90)	0 (0–0)	3885 (3684–4087)	11 (10–12)
Austria	2023	82 (73–91)	0 (0–0)	3885 (3600–4170)	10 (9–12)
Austria	2024	78 (66–91)	0 (0–1)	3885 (3536–4234)	10 (7–12)
Austria	2025	75 (58–91)	0 (0–1)	3885 (3482–4288)	9 (6–12)
Austria	2026	72 (51–92)	0 (0–1)	3885 (3434–4336)	8 (5–12)
Austria	2027	68 (43–93)	0 (0–1)	3885 (3391–4379)	8 (3–12)
Austria	2028	65 (34–95)	0 (0–1)	3885 (3352–4418)	7 (2–13)
Austria	2029	61 (26–96)	0 (0–1)	3885 (3315–4455)	6 (0–13)
Austria	2030	58 (17–98)	0 (-0–1)	3885 (3281–4490)	6 (-1–13)
Austria	2031	54 (8–100)	0 (-0–1)	3885 (3248–4522)	5 (-3–13)
Austria	2032	51 (-1–103)	0 (-0–1)	3885 (3217–4553)	5 (-5–14)
Austria	2033	48 (-10–105)	0 (-0–1)	3885 (3187–4583)	4 (-7–14)
Austria	2034	44 (-20–108)	0 (-0–1)	3885 (3159–4612)	3 (-8–15)

Austria	2035	41 (-30–111)	0 (-0–1)	3885 (3131–4639)	3 (-10–15)
Austria	2036	37 (-40–114)	0 (-1–1)	3885 (3105–4666)	2 (-12–16)
Azerbaijan	2022	195 (187–202)	2 (2–2)	5699 (5492–5907)	47 (44–50)
Azerbaijan	2023	194 (180–208)	2 (2–2)	5706 (5347–6065)	46 (40–52)
Azerbaijan	2024	194 (175–214)	2 (2–2)	5704 (5174–6234)	45 (36–55)
Azerbaijan	2025	194 (170–218)	2 (1–2)	5705 (5006–6405)	45 (33–57)
Azerbaijan	2026	194 (166–222)	2 (1–2)	5705 (4839–6572)	45 (30–60)
Azerbaijan	2027	194 (162–226)	2 (1–2)	5706 (4676–6735)	44 (27–62)
Azerbaijan	2028	194 (159–229)	2 (1–3)	5706 (4520–6892)	44 (24–64)
Azerbaijan	2029	194 (156–233)	2 (1–3)	5706 (4369–7043)	44 (22–67)
Azerbaijan	2030	194 (153–235)	2 (1–3)	5706 (4225–7187)	44 (20–69)
Azerbaijan	2031	194 (150–238)	2 (1–3)	5706 (4087–7325)	44 (18–71)
Azerbaijan	2032	194 (148–241)	2 (1–3)	5706 (3955–7458)	44 (16–73)
Azerbaijan	2033	194 (145–243)	2 (1–3)	5706 (3828–7585)	44 (14–74)
Azerbaijan	2034	194 (143–245)	2 (1–3)	5706 (3706–7707)	44 (12–76)
Azerbaijan	2035	194 (141–248)	2 (1–3)	5706 (3590–7823)	44 (11–78)
Azerbaijan	2036	194 (139–250)	2 (1–3)	5706 (3477–7936)	44 (9–79)
Bahamas	2022	2 (2–2)	0 (0–0)	123 (118–128)	10 (9–10)
Bahamas	2023	2 (2–2)	0 (0–0)	125 (116–133)	9 (8–10)
Bahamas	2024	2 (2–2)	0 (0–0)	127 (113–140)	9 (8–10)
Bahamas	2025	2 (2–2)	0 (0–0)	128 (109–147)	9 (7–10)
Bahamas	2026	2 (2–2)	0 (0–0)	130 (105–155)	9 (7–10)
Bahamas	2027	2 (2–2)	0 (0–0)	131 (100–163)	8 (7–10)
Bahamas	2028	2 (2–2)	0 (0–0)	133 (94–171)	8 (6–10)
Bahamas	2029	2 (2–2)	0 (0–0)	135 (89–181)	8 (6–10)
Bahamas	2030	2 (2–2)	0 (0–0)	136 (82–190)	8 (5–10)
Bahamas	2031	2 (2–2)	0 (0–0)	138 (75–200)	7 (5–10)
Bahamas	2032	2 (2–2)	0 (0–0)	139 (68–211)	7 (5–9)
Bahamas	2033	2 (2–2)	0 (0–0)	141 (61–221)	7 (4–9)
Bahamas	2034	2 (2–2)	0 (0–0)	143 (53–233)	7 (4–9)
Bahamas	2035	2 (2–2)	0 (0–0)	144 (44–244)	6 (4–9)
Bahamas	2036	2 (2–2)	0 (0–0)	146 (36–256)	6 (3–9)
Bahrain	2022	5 (5–5)	1 (1–1)	152 (149–155)	13 (12–14)

Bahrain	2023	5 (5–5)	1 (1–1)	164 (157–171)	13 (11–15)
Bahrain	2024	5 (5–6)	1 (0–1)	176 (164–188)	12 (9–15)
Bahrain	2025	6 (5–6)	1 (0–1)	189 (171–206)	12 (8–15)
Bahrain	2026	6 (5–7)	1 (0–1)	201 (177–224)	11 (7–15)
Bahrain	2027	6 (5–8)	1 (0–1)	213 (182–243)	10 (6–15)
Bahrain	2028	7 (5–8)	0 (0–1)	225 (187–263)	10 (4–15)
Bahrain	2029	7 (5–9)	0 (0–1)	237 (192–283)	9 (3–15)
Bahrain	2030	7 (5–9)	0 (0–1)	249 (196–303)	8 (2–14)
Bahrain	2031	8 (5–10)	0 (0–1)	261 (199–324)	8 (1–14)
Bahrain	2032	8 (5–11)	0 (0–1)	274 (202–345)	7 (-0–14)
Bahrain	2033	8 (5–12)	0 (-0–1)	286 (205–367)	6 (-1–14)
Bahrain	2034	9 (5–12)	0 (-0–1)	298 (207–389)	5 (-2–13)
Bahrain	2035	9 (5–13)	0 (-0–1)	310 (209–412)	5 (-3–13)
Bahrain	2036	9 (5–14)	0 (-0–1)	322 (210–434)	4 (-4–13)
Bangladesh	2022	742 (716–767)	1 (0–1)	59373 (57933–60812)	13 (12–13)
Bangladesh	2023	742 (687–796)	1 (0–1)	60627 (57409–63846)	12 (10–14)
Bangladesh	2024	742 (669–814)	0 (0–1)	61882 (56497–67267)	11 (9–14)
Bangladesh	2025	742 (654–829)	0 (0–1)	63137 (55253–71020)	11 (8–14)
Bangladesh	2026	742 (642–841)	0 (0–1)	64391 (53717–75066)	10 (6–13)
Bangladesh	2027	742 (631–852)	0 (0–1)	65646 (51916–79376)	9 (5–13)
Bangladesh	2028	742 (621–862)	0 (0–1)	66901 (49870–83931)	9 (4–13)
Bangladesh	2029	742 (612–872)	0 (0–1)	68155 (47598–88713)	8 (3–13)
Bangladesh	2030	742 (603–880)	0 (0–1)	69410 (45111–93708)	7 (2–12)
Bangladesh	2031	742 (595–888)	0 (0–1)	70664 (42423–98906)	7 (2–12)
Bangladesh	2032	742 (587–896)	0 (0–1)	71919 (39542–104296)	6 (1–11)
Bangladesh	2033	742 (580–904)	0 (0–1)	73174 (36478–109869)	5 (-0–11)
Bangladesh	2034	742 (573–911)	0 (-0–1)	74428 (33238–115619)	5 (-1–11)
Bangladesh	2035	742 (566–917)	0 (-0–1)	75683 (29828–121538)	4 (-2–10)
Bangladesh	2036	742 (559–924)	0 (-0–0)	76938 (26254–127621)	3 (-3–10)
Barbados	2022	2 (2–2)	0 (0–0)	93 (86–100)	7 (6–8)
Barbados	2023	2 (2–2)	0 (0–0)	93 (84–102)	7 (5–8)
Barbados	2024	2 (1–2)	0 (0–0)	93 (82–104)	7 (5–8)
Barbados	2025	2 (1–2)	0 (0–0)	93 (80–106)	6 (4–8)

Barbados	2026	2 (1–2)	0 (0–0)	93 (78–108)	6 (3–8)
Barbados	2027	2 (1–2)	0 (0–0)	93 (77–109)	5 (3–8)
Barbados	2028	2 (1–2)	0 (0–0)	93 (75–111)	5 (2–8)
Barbados	2029	2 (1–2)	0 (0–0)	93 (74–112)	5 (2–8)
Barbados	2030	2 (1–2)	0 (0–0)	93 (73–113)	4 (1–7)
Barbados	2031	2 (1–2)	0 (0–0)	93 (72–114)	4 (1–7)
Barbados	2032	2 (1–2)	0 (-0–0)	93 (71–115)	3 (0–7)
Barbados	2033	2 (1–2)	0 (-0–0)	93 (70–116)	3 (-1–7)
Barbados	2034	2 (1–2)	0 (-0–0)	93 (69–117)	3 (-1–6)
Barbados	2035	1 (1–2)	0 (-0–0)	93 (68–118)	2 (-2–6)
Barbados	2036	1 (1–2)	0 (-0–0)	93 (67–119)	2 (-2–6)
Belarus	2022	248 (217–278)	2 (1–2)	5943 (5433–6452)	40 (34–46)
Belarus	2023	239 (195–282)	1 (1–2)	5804 (5232–6375)	38 (30–46)
Belarus	2024	230 (176–283)	1 (1–2)	5804 (5232–6375)	35 (25–45)
Belarus	2025	221 (159–282)	1 (1–2)	5804 (5232–6375)	33 (22–45)
Belarus	2026	212 (143–281)	1 (1–2)	5804 (5232–6375)	31 (18–44)
Belarus	2027	203 (127–278)	1 (1–2)	5804 (5232–6375)	29 (14–43)
Belarus	2028	194 (112–276)	1 (0–2)	5804 (5232–6375)	26 (11–42)
Belarus	2029	185 (98–272)	1 (0–2)	5804 (5232–6375)	24 (8–40)
Belarus	2030	176 (83–269)	1 (0–1)	5804 (5232–6375)	22 (4–39)
Belarus	2031	167 (69–265)	1 (0–1)	5804 (5232–6375)	20 (1–38)
Belarus	2032	158 (56–260)	1 (0–1)	5804 (5232–6375)	17 (-2–37)
Belarus	2033	149 (42–256)	1 (-0–1)	5804 (5232–6375)	15 (-5–35)
Belarus	2034	140 (29–251)	1 (-0–1)	5804 (5232–6375)	13 (-8–34)
Belarus	2035	131 (16–247)	0 (-0–1)	5804 (5232–6375)	10 (-11–32)
Belarus	2036	122 (3–242)	0 (-0–1)	5804 (5232–6375)	8 (-14–31)
Belgium	2022	119 (111–127)	0 (0–1)	8562 (8051–9074)	11 (10–12)
Belgium	2023	115 (102–128)	0 (0–1)	8491 (7711–9270)	10 (9–12)
Belgium	2024	111 (92–129)	0 (0–1)	8419 (7395–9443)	10 (8–12)
Belgium	2025	107 (83–131)	0 (0–1)	8347 (7084–9611)	9 (6–12)
Belgium	2026	103 (73–132)	0 (0–1)	8276 (6772–9780)	9 (5–12)
Belgium	2027	99 (62–135)	0 (0–1)	8204 (6455–9953)	8 (4–13)
Belgium	2028	94 (52–137)	0 (0–1)	8132 (6133–10131)	8 (3–13)

Belgium	2029	90 (41–140)	0 (0–1)	8061 (5805–10316)	7 (1–13)
Belgium	2030	86 (30–143)	0 (-0–1)	7989 (5470–10507)	7 (0–14)
Belgium	2031	82 (18–146)	0 (-0–1)	7917 (5129–10705)	7 (-1–14)
Belgium	2032	78 (6–150)	0 (-0–1)	7846 (4781–10911)	6 (-3–15)
Belgium	2033	74 (-6–154)	0 (-0–1)	7774 (4425–11122)	6 (-4–15)
Belgium	2034	70 (-18–158)	0 (-0–1)	7702 (4063–11341)	5 (-6–16)
Belgium	2035	66 (-31–163)	0 (-0–1)	7630 (3694–11567)	5 (-7–17)
Belgium	2036	62 (-44–168)	0 (-1–1)	7559 (3318–11800)	4 (-9–17)
Belize	2022	1 (1–2)	0 (0–1)	35 (34–37)	12 (10–14)
Belize	2023	1 (1–2)	0 (0–1)	36 (33–39)	12 (8–16)
Belize	2024	1 (1–2)	1 (0–1)	37 (33–41)	12 (7–18)
Belize	2025	1 (1–2)	1 (0–1)	38 (33–42)	12 (6–19)
Belize	2026	1 (1–2)	1 (0–1)	39 (34–44)	12 (4–20)
Belize	2027	1 (1–2)	1 (0–1)	39 (34–45)	12 (4–21)
Belize	2028	1 (1–2)	1 (0–1)	40 (34–46)	12 (3–22)
Belize	2029	1 (1–2)	1 (0–1)	41 (34–47)	12 (2–22)
Belize	2030	1 (1–2)	1 (0–1)	42 (35–49)	12 (1–23)
Belize	2031	1 (1–2)	1 (0–1)	42 (35–50)	12 (1–24)
Belize	2032	1 (1–2)	1 (0–1)	43 (36–51)	12 (0–24)
Belize	2033	1 (1–2)	1 (-0–1)	44 (36–52)	12 (-0–25)
Belize	2034	1 (1–2)	1 (-0–1)	45 (36–53)	12 (-1–25)
Belize	2035	1 (1–2)	1 (-0–1)	46 (37–54)	12 (-1–26)
Belize	2036	1 (1–2)	1 (-0–1)	46 (37–55)	12 (-2–26)
Benin	2022	8 (8–9)	0 (0–0)	646 (636–656)	4 (4–4)
Benin	2023	8 (8–9)	0 (0–0)	667 (649–685)	4 (4–4)
Benin	2024	8 (8–9)	0 (0–0)	688 (662–714)	4 (3–4)
Benin	2025	8 (8–9)	0 (0–0)	710 (675–745)	3 (3–4)
Benin	2026	9 (8–9)	0 (0–0)	731 (686–776)	3 (3–4)
Benin	2027	9 (8–9)	0 (0–0)	753 (697–808)	3 (3–3)
Benin	2028	9 (8–9)	0 (0–0)	774 (707–841)	3 (3–3)
Benin	2029	9 (8–9)	0 (0–0)	795 (716–874)	3 (3–3)
Benin	2030	9 (8–9)	0 (0–0)	817 (725–908)	3 (2–3)
Benin	2031	9 (8–9)	0 (0–0)	838 (733–943)	2 (2–3)

Benin	2032	9 (8–9)	0 (0–0)	859 (741–978)	2 (2–3)
Benin	2033	9 (8–9)	0 (0–0)	881 (748–1014)	2 (2–3)
Benin	2034	9 (8–9)	0 (0–0)	902 (754–1050)	2 (2–2)
Benin	2035	9 (8–9)	0 (0–0)	924 (760–1087)	2 (1–2)
Benin	2036	9 (8–9)	0 (0–0)	945 (765–1125)	2 (1–2)
Bermuda	2022	0 (0–0)	0 (0–0)	27 (26–29)	7 (6–8)
Bermuda	2023	0 (0–0)	0 (0–0)	28 (25–30)	7 (5–8)
Bermuda	2024	0 (0–1)	0 (0–0)	28 (24–32)	7 (5–9)
Bermuda	2025	0 (0–1)	0 (0–0)	28 (24–33)	7 (4–10)
Bermuda	2026	0 (0–1)	0 (0–0)	29 (23–34)	7 (3–10)
Bermuda	2027	0 (0–1)	0 (0–0)	29 (22–36)	7 (2–11)
Bermuda	2028	0 (0–1)	0 (0–1)	29 (22–37)	7 (2–12)
Bermuda	2029	0 (0–1)	0 (0–1)	30 (21–38)	7 (1–13)
Bermuda	2030	0 (0–1)	0 (-0–1)	30 (20–40)	7 (-0–14)
Bermuda	2031	0 (0–1)	0 (-0–1)	30 (19–41)	7 (-1–15)
Bermuda	2032	1 (0–1)	0 (-0–1)	31 (18–43)	7 (-2–16)
Bermuda	2033	1 (0–1)	0 (-0–1)	31 (17–45)	7 (-3–17)
Bermuda	2034	1 (0–1)	0 (-0–1)	31 (17–46)	7 (-5–18)
Bermuda	2035	1 (0–1)	0 (-0–1)	32 (16–48)	7 (-6–19)
Bermuda	2036	1 (0–1)	0 (-0–1)	32 (15–50)	7 (-7–20)
Bhutan	2022	2 (2–2)	0 (0–0)	203 (201–205)	6 (5–6)
Bhutan	2023	2 (2–2)	0 (0–0)	207 (204–210)	5 (5–6)
Bhutan	2024	2 (2–2)	0 (0–0)	211 (206–216)	5 (5–6)
Bhutan	2025	2 (2–2)	0 (0–0)	216 (208–223)	5 (5–6)
Bhutan	2026	2 (2–2)	0 (0–0)	220 (210–230)	5 (5–6)
Bhutan	2027	2 (2–2)	0 (0–0)	224 (211–237)	5 (5–6)
Bhutan	2028	2 (2–2)	0 (0–0)	228 (212–244)	5 (4–6)
Bhutan	2029	2 (2–2)	0 (0–0)	232 (213–252)	5 (4–6)
Bhutan	2030	2 (2–2)	0 (0–0)	237 (214–259)	5 (4–6)
Bhutan	2031	2 (2–2)	0 (0–0)	241 (214–267)	5 (4–6)
Bhutan	2032	2 (2–2)	0 (0–0)	245 (215–275)	5 (4–6)
Bhutan	2033	2 (2–2)	0 (0–0)	249 (215–284)	5 (3–6)
Bhutan	2034	2 (2–2)	0 (0–0)	253 (215–292)	5 (3–6)

Bhutan	2035	2 (2–2)	0 (0–0)	258 (215–301)	5 (3–6)
Bhutan	2036	2 (2–2)	0 (0–0)	262 (214–309)	5 (3–6)
Bolivia (Plurinational State of)	2022	107 (106–109)	1 (1–1)	650 (641–659)	28 (28–29)
Bolivia (Plurinational State of)	2023	105 (102–109)	1 (1–1)	644 (623–664)	28 (27–29)
Bolivia (Plurinational State of)	2024	103 (97–109)	1 (1–1)	638 (604–672)	27 (25–29)
Bolivia (Plurinational State of)	2025	101 (92–110)	1 (1–1)	632 (582–682)	26 (23–30)
Bolivia (Plurinational State of)	2026	99 (87–111)	1 (1–1)	626 (559–694)	26 (22–30)
Bolivia (Plurinational State of)	2027	97 (82–112)	1 (1–1)	620 (533–707)	25 (20–31)
Bolivia (Plurinational State of)	2028	95 (76–114)	1 (1–1)	614 (507–722)	25 (18–32)
Bolivia (Plurinational State of)	2029	93 (71–115)	1 (1–1)	609 (478–739)	24 (16–32)
Bolivia (Plurinational State of)	2030	91 (64–118)	1 (0–1)	603 (449–756)	23 (13–33)
Bolivia (Plurinational State of)	2031	89 (58–120)	1 (0–1)	597 (418–776)	23 (11–34)
Bolivia (Plurinational State of)	2032	87 (52–122)	1 (0–1)	591 (386–796)	22 (9–36)
Bolivia (Plurinational State of)	2033	85 (45–125)	1 (0–1)	585 (353–817)	21 (6–37)
Bolivia (Plurinational State of)	2034	83 (38–128)	1 (-0–1)	579 (319–840)	21 (4–38)
Bolivia (Plurinational State of)	2035	81 (31–131)	1 (-0–2)	573 (283–863)	20 (1–39)
Bolivia (Plurinational State of)	2036	79 (24–134)	1 (-0–2)	567 (247–888)	20 (-2–41)
Bosnia and Herzegovina	2022	83 (76–90)	1 (1–1)	1670 (1544–1795)	30 (27–34)
Bosnia and Herzegovina	2023	77 (66–88)	1 (1–1)	1670 (1492–1847)	30 (25–34)
Bosnia and Herzegovina	2024	73 (55–91)	1 (1–1)	1670 (1452–1887)	29 (24–35)
Bosnia and Herzegovina	2025	67 (42–92)	1 (1–2)	1670 (1419–1921)	29 (22–35)
Bosnia and Herzegovina	2026	62 (29–95)	1 (1–2)	1670 (1389–1951)	28 (21–35)
Bosnia and Herzegovina	2027	57 (15–98)	1 (1–2)	1670 (1362–1977)	28 (20–35)
Bosnia and Herzegovina	2028	52 (1–103)	1 (1–2)	1670 (1337–2002)	27 (19–35)
Bosnia and Herzegovina	2029	46 (-15–107)	1 (1–2)	1670 (1314–2025)	26 (18–35)
Bosnia and Herzegovina	2030	41 (-30–113)	1 (1–2)	1670 (1293–2047)	26 (16–35)
Bosnia and Herzegovina	2031	36 (-47–119)	1 (1–2)	1670 (1273–2067)	25 (15–35)
Bosnia and Herzegovina	2032	31 (-63–125)	1 (1–2)	1670 (1253–2086)	25 (14–35)
Bosnia and Herzegovina	2033	26 (-81–132)	1 (1–2)	1670 (1235–2105)	24 (13–35)
Bosnia and Herzegovina	2034	21 (-98–140)	1 (1–2)	1670 (1217–2123)	24 (12–35)
Bosnia and Herzegovina	2035	16 (-117–148)	1 (1–2)	1670 (1200–2140)	23 (11–35)
Bosnia and Herzegovina	2036	10 (-135–156)	1 (1–2)	1670 (1183–2156)	23 (10–35)
Botswana	2022	5 (5–5)	0 (0–0)	943 (918–969)	9 (8–9)

Botswana	2023	5 (5–6)	0 (0–0)	945 (884–1006)	9 (8–10)
Botswana	2024	5 (5–6)	0 (0–0)	944 (844–1044)	9 (7–10)
Botswana	2025	5 (5–6)	0 (0–0)	942 (804–1080)	9 (7–11)
Botswana	2026	5 (5–6)	0 (0–0)	940 (768–1113)	9 (6–11)
Botswana	2027	5 (5–6)	0 (0–0)	939 (736–1142)	9 (5–12)
Botswana	2028	5 (4–6)	0 (0–0)	938 (708–1168)	8 (5–12)
Botswana	2029	5 (4–6)	0 (0–0)	938 (684–1191)	8 (4–13)
Botswana	2030	5 (4–6)	0 (0–0)	937 (663–1212)	8 (3–14)
Botswana	2031	5 (4–6)	0 (0–0)	937 (644–1231)	8 (2–14)
Botswana	2032	5 (4–6)	0 (0–0)	938 (626–1249)	8 (2–15)
Botswana	2033	5 (4–6)	0 (0–0)	938 (609–1266)	8 (1–15)
Botswana	2034	5 (4–6)	0 (0–0)	938 (593–1282)	8 (1–16)
Botswana	2035	5 (4–6)	0 (0–0)	938 (578–1297)	8 (-0–17)
Botswana	2036	5 (4–6)	0 (-0–0)	938 (563–1312)	8 (-1–17)
Brazil	2022	1817 (1762–1872)	1 (1–1)	86634 (84341–88926)	17 (16–18)
Brazil	2023	1798 (1705–1890)	1 (1–1)	86634 (83392–89876)	16 (14–18)
Brazil	2024	1778 (1660–1897)	1 (1–1)	86634 (82663–90604)	16 (13–18)
Brazil	2025	1759 (1619–1899)	1 (0–1)	86634 (82049–91218)	15 (12–19)
Brazil	2026	1740 (1582–1898)	1 (0–1)	86634 (81507–91760)	15 (10–19)
Brazil	2027	1720 (1546–1895)	1 (0–1)	86634 (81018–92249)	14 (9–20)
Brazil	2028	1701 (1511–1891)	1 (0–1)	86634 (80568–92699)	14 (7–20)
Brazil	2029	1682 (1478–1885)	1 (0–1)	86634 (80150–93118)	13 (6–21)
Brazil	2030	1662 (1446–1879)	1 (0–1)	86634 (79756–93511)	13 (4–22)
Brazil	2031	1643 (1414–1872)	0 (0–1)	86634 (79384–93883)	12 (2–23)
Brazil	2032	1624 (1383–1865)	0 (-0–1)	86634 (79030–94237)	12 (0–23)
Brazil	2033	1604 (1352–1856)	0 (-0–1)	86634 (78692–94575)	11 (-1–24)
Brazil	2034	1585 (1322–1848)	0 (-0–1)	86634 (78368–94899)	11 (-3–25)
Brazil	2035	1566 (1293–1839)	0 (-0–1)	86634 (78056–95211)	10 (-5–26)
Brazil	2036	1546 (1263–1829)	0 (-0–1)	86634 (77755–95512)	10 (-7–27)
Brunei Darussalam	2022	3 (3–3)	1 (1–1)	69 (67–71)	21 (19–22)
Brunei Darussalam	2023	3 (3–3)	1 (1–1)	70 (66–75)	20 (16–24)
Brunei Darussalam	2024	3 (3–4)	1 (1–1)	72 (65–79)	20 (13–26)
Brunei Darussalam	2025	3 (3–4)	1 (0–1)	74 (64–83)	19 (10–28)

Brunei Darussalam	2026	3 (2–4)	1 (0–2)	75 (62–88)	19 (6–31)
Brunei Darussalam	2027	3 (2–5)	1 (-0–2)	77 (59–94)	18 (2–34)
Brunei Darussalam	2028	3 (2–5)	1 (-0–2)	78 (57–100)	18 (-2–38)
Brunei Darussalam	2029	4 (2–5)	1 (-1–2)	80 (54–105)	17 (-7–42)
Brunei Darussalam	2030	4 (2–6)	1 (-1–2)	81 (50–112)	17 (-12–46)
Brunei Darussalam	2031	4 (1–6)	1 (-1–3)	83 (47–118)	16 (-17–50)
Brunei Darussalam	2032	4 (1–6)	1 (-1–3)	84 (43–125)	16 (-22–54)
Brunei Darussalam	2033	4 (1–7)	1 (-2–3)	86 (39–132)	15 (-28–59)
Brunei Darussalam	2034	4 (0–7)	1 (-2–4)	87 (35–139)	15 (-34–64)
Brunei Darussalam	2035	4 (0–8)	1 (-2–4)	89 (31–146)	15 (-40–69)
Brunei Darussalam	2036	4 (-0–8)	1 (-3–4)	90 (26–154)	14 (-46–74)
Bulgaria	2022	181 (165–198)	1 (1–1)	3672 (3346–3998)	35 (32–39)
Bulgaria	2023	175 (152–198)	1 (1–1)	3586 (3111–4061)	34 (29–39)
Bulgaria	2024	169 (141–197)	1 (1–1)	3668 (2987–4349)	33 (27–39)
Bulgaria	2025	163 (131–195)	1 (1–1)	3589 (2822–4356)	31 (24–38)
Bulgaria	2026	157 (121–193)	1 (1–1)	3664 (2758–4570)	30 (22–38)
Bulgaria	2027	151 (111–190)	1 (1–1)	3593 (2619–4567)	28 (20–37)
Bulgaria	2028	145 (102–188)	1 (1–1)	3661 (2577–4746)	27 (18–36)
Bulgaria	2029	138 (93–184)	1 (1–1)	3596 (2452–4739)	26 (16–36)
Bulgaria	2030	132 (84–181)	1 (0–1)	3658 (2421–4896)	24 (14–35)
Bulgaria	2031	126 (75–178)	1 (0–1)	3598 (2308–4889)	23 (12–34)
Bulgaria	2032	120 (66–174)	1 (0–1)	3656 (2283–5028)	21 (10–33)
Bulgaria	2033	114 (58–170)	1 (0–1)	3601 (2179–5023)	20 (8–32)
Bulgaria	2034	108 (49–166)	1 (0–1)	3653 (2157–5149)	19 (6–31)
Bulgaria	2035	102 (41–162)	1 (0–1)	3603 (2061–5146)	17 (4–30)
Bulgaria	2036	96 (33–158)	1 (0–1)	3651 (2042–5260)	16 (2–29)
Burkina Faso	2022	21 (20–21)	0 (0–0)	1731 (1707–1756)	6 (6–6)
Burkina Faso	2023	21 (20–21)	0 (0–0)	1777 (1722–1832)	6 (5–6)
Burkina Faso	2024	21 (20–22)	0 (0–0)	1823 (1731–1916)	6 (5–6)
Burkina Faso	2025	21 (19–23)	0 (0–0)	1870 (1735–2005)	5 (5–6)
Burkina Faso	2026	21 (19–24)	0 (0–0)	1916 (1733–2098)	5 (4–6)
Burkina Faso	2027	22 (19–25)	0 (0–0)	1962 (1727–2197)	5 (4–6)
Burkina Faso	2028	22 (18–25)	0 (0–0)	2008 (1716–2300)	5 (4–7)

Burkina Faso	2029	22 (17–26)	0 (0–0)	2054 (1702–2406)	5 (3–7)
Burkina Faso	2030	22 (17–27)	0 (0–0)	2100 (1684–2516)	5 (3–7)
Burkina Faso	2031	22 (16–29)	0 (0–0)	2146 (1663–2630)	5 (3–7)
Burkina Faso	2032	23 (15–30)	0 (0–0)	2192 (1638–2747)	5 (2–8)
Burkina Faso	2033	23 (15–31)	0 (0–0)	2238 (1610–2867)	5 (2–8)
Burkina Faso	2034	23 (14–32)	0 (0–0)	2285 (1579–2990)	5 (1–8)
Burkina Faso	2035	23 (13–33)	0 (0–0)	2331 (1545–3116)	5 (1–9)
Burkina Faso	2036	23 (12–34)	0 (0–0)	2377 (1509–3245)	5 (0–9)
Burundi	2022	12 (12–12)	0 (0–0)	1889 (1849–1929)	6 (6–6)
Burundi	2023	12 (12–13)	0 (0–0)	1931 (1841–2022)	6 (6–6)
Burundi	2024	12 (12–13)	0 (0–0)	1974 (1823–2125)	6 (5–7)
Burundi	2025	13 (12–14)	0 (0–0)	2017 (1796–2237)	6 (5–7)
Burundi	2026	13 (11–14)	0 (0–0)	2059 (1760–2358)	6 (5–7)
Burundi	2027	13 (11–15)	0 (0–0)	2102 (1717–2486)	6 (4–8)
Burundi	2028	14 (11–16)	0 (0–0)	2144 (1667–2621)	6 (4–8)
Burundi	2029	14 (11–17)	0 (0–0)	2187 (1611–2763)	6 (3–8)
Burundi	2030	14 (11–17)	0 (0–0)	2229 (1548–2910)	6 (3–9)
Burundi	2031	14 (11–18)	0 (0–0)	2272 (1481–3063)	6 (2–9)
Burundi	2032	15 (10–19)	0 (0–0)	2314 (1407–3222)	6 (2–10)
Burundi	2033	15 (10–20)	0 (0–0)	2357 (1329–3385)	6 (1–10)
Burundi	2034	15 (10–21)	0 (0–0)	2399 (1245–3554)	6 (1–10)
Burundi	2035	16 (9–22)	0 (0–0)	2442 (1157–3727)	6 (0–11)
Burundi	2036	16 (9–23)	0 (0–0)	2485 (1064–3905)	6 (-0–11)
Cabo Verde	2022	2 (2–3)	1 (0–1)	252 (233–271)	14 (13–16)
Cabo Verde	2023	2 (2–3)	1 (0–1)	257 (230–285)	14 (12–17)
Cabo Verde	2024	2 (2–3)	1 (0–1)	263 (230–296)	15 (11–18)
Cabo Verde	2025	2 (2–3)	1 (0–1)	269 (230–307)	15 (11–19)
Cabo Verde	2026	2 (2–3)	1 (0–1)	274 (231–318)	15 (10–20)
Cabo Verde	2027	2 (2–3)	1 (0–1)	280 (233–327)	15 (9–21)
Cabo Verde	2028	2 (2–3)	1 (0–1)	286 (235–337)	15 (9–22)
Cabo Verde	2029	2 (2–3)	1 (0–1)	291 (237–346)	16 (8–23)
Cabo Verde	2030	2 (2–3)	1 (0–1)	297 (239–355)	16 (7–25)
Cabo Verde	2031	2 (2–3)	1 (0–1)	303 (242–364)	16 (6–26)

Cabo Verde	2032	2 (2–3)	1 (0–1)	308 (244–372)	16 (5–27)
Cabo Verde	2033	2 (2–3)	1 (0–1)	314 (247–381)	16 (4–29)
Cabo Verde	2034	2 (2–3)	1 (0–1)	320 (250–389)	17 (3–30)
Cabo Verde	2035	2 (1–3)	1 (0–1)	325 (253–397)	17 (2–31)
Cabo Verde	2036	2 (1–3)	1 (0–1)	331 (256–406)	17 (1–33)
Cambodia	2022	155 (154–156)	1 (1–1)	4627 (4596–4659)	30 (30–31)
Cambodia	2023	158 (156–161)	1 (1–1)	4752 (4682–4822)	30 (29–31)
Cambodia	2024	162 (158–166)	1 (1–1)	4877 (4760–4994)	30 (28–31)
Cambodia	2025	165 (159–172)	1 (1–1)	5002 (4831–5172)	29 (27–32)
Cambodia	2026	169 (160–177)	1 (1–1)	5126 (4895–5358)	29 (26–33)
Cambodia	2027	172 (161–183)	1 (1–1)	5251 (4953–5549)	29 (25–33)
Cambodia	2028	176 (162–189)	1 (1–1)	5376 (5007–5745)	29 (24–34)
Cambodia	2029	179 (163–196)	1 (1–1)	5501 (5055–5946)	29 (22–35)
Cambodia	2030	183 (163–202)	1 (1–1)	5625 (5098–6152)	28 (21–36)
Cambodia	2031	186 (163–209)	1 (1–1)	5750 (5138–6362)	28 (20–37)
Cambodia	2032	190 (163–216)	1 (1–1)	5875 (5173–6577)	28 (19–37)
Cambodia	2033	193 (163–223)	1 (1–1)	5999 (5204–6795)	28 (17–38)
Cambodia	2034	196 (163–230)	1 (1–1)	6124 (5231–7017)	28 (16–39)
Cambodia	2035	200 (163–237)	1 (1–1)	6249 (5255–7243)	28 (15–40)
Cambodia	2036	203 (162–244)	1 (1–1)	6374 (5275–7472)	27 (14–41)
Cameroon	2022	29 (29–29)	0 (0–0)	2897 (2876–2917)	6 (6–6)
Cameroon	2023	29 (28–30)	0 (0–0)	2973 (2927–3018)	6 (5–6)
Cameroon	2024	29 (28–30)	0 (0–0)	3049 (2973–3125)	5 (5–6)
Cameroon	2025	29 (27–31)	0 (0–0)	3125 (3014–3236)	5 (5–6)
Cameroon	2026	29 (27–32)	0 (0–0)	3201 (3050–3352)	5 (4–6)
Cameroon	2027	29 (26–32)	0 (0–0)	3277 (3083–3471)	5 (4–6)
Cameroon	2028	29 (25–33)	0 (0–0)	3353 (3113–3593)	5 (3–6)
Cameroon	2029	29 (24–34)	0 (0–0)	3429 (3139–3719)	4 (3–6)
Cameroon	2030	29 (24–35)	0 (0–0)	3505 (3162–3848)	4 (3–6)
Cameroon	2031	29 (23–36)	0 (0–0)	3581 (3183–3980)	4 (2–6)
Cameroon	2032	29 (22–37)	0 (0–0)	3657 (3200–4114)	4 (2–6)
Cameroon	2033	29 (21–38)	0 (0–0)	3733 (3215–4251)	4 (1–6)
Cameroon	2034	29 (20–39)	0 (0–0)	3809 (3228–4391)	4 (1–6)

Cameroon	2035	29 (19–40)	0 (0–0)	3885 (3238–4533)	3 (0–6)
Cameroon	2036	29 (18–41)	0 (0–0)	3962 (3246–4677)	3 (-0–7)
Canada	2022	309 (287–332)	0 (0–0)	22538 (21326–23751)	8 (7–9)
Canada	2023	305 (280–330)	0 (0–0)	22765 (21050–24479)	8 (6–9)
Canada	2024	300 (272–328)	0 (0–0)	22991 (20891–25090)	7 (5–9)
Canada	2025	296 (265–326)	0 (0–0)	23217 (20793–25642)	7 (4–10)
Canada	2026	291 (259–324)	0 (0–0)	23444 (20733–26154)	6 (3–10)
Canada	2027	287 (252–321)	0 (0–0)	23670 (20701–26639)	6 (2–10)
Canada	2028	282 (246–319)	0 (0–0)	23896 (20689–27103)	5 (1–10)
Canada	2029	278 (239–316)	0 (0–0)	24122 (20694–27551)	5 (0–10)
Canada	2030	273 (233–314)	0 (-0–0)	24349 (20712–27985)	4 (-1–10)
Canada	2031	269 (227–311)	0 (-0–0)	24575 (20742–28408)	4 (-2–10)
Canada	2032	264 (221–308)	0 (-0–0)	24801 (20781–28822)	4 (-3–10)
Canada	2033	260 (214–305)	0 (-0–0)	25028 (20829–29227)	3 (-4–10)
Canada	2034	255 (208–302)	0 (-0–0)	25254 (20883–29624)	3 (-5–10)
Canada	2035	251 (202–299)	0 (-0–0)	25480 (20945–30016)	2 (-6–11)
Canada	2036	246 (196–296)	0 (-0–0)	25707 (21012–30401)	2 (-8–11)
Central African Republic	2022	9 (9–9)	0 (0–0)	807 (794–821)	10 (10–11)
Central African Republic	2023	9 (9–10)	0 (0–0)	814 (787–840)	10 (10–11)
Central African Republic	2024	9 (9–10)	0 (0–0)	820 (779–861)	10 (9–11)
Central African Republic	2025	9 (9–10)	0 (0–0)	827 (769–885)	10 (9–11)
Central African Republic	2026	9 (8–10)	0 (0–0)	833 (757–910)	10 (8–11)
Central African Republic	2027	9 (8–10)	0 (0–0)	840 (743–937)	9 (8–11)
Central African Republic	2028	9 (8–11)	0 (0–0)	846 (728–965)	9 (7–11)
Central African Republic	2029	9 (8–11)	0 (0–0)	853 (711–994)	9 (6–11)
Central African Republic	2030	9 (7–11)	0 (0–0)	859 (694–1025)	9 (6–12)
Central African Republic	2031	9 (7–12)	0 (0–0)	866 (675–1057)	8 (5–12)
Central African Republic	2032	9 (7–12)	0 (0–0)	872 (654–1091)	8 (4–12)
Central African Republic	2033	9 (7–12)	0 (0–0)	879 (633–1125)	8 (3–12)
Central African Republic	2034	9 (6–13)	0 (0–0)	886 (610–1161)	8 (3–13)
Central African Republic	2035	9 (6–13)	0 (0–0)	892 (587–1197)	7 (2–13)
Central African Republic	2036	9 (6–13)	0 (0–0)	899 (562–1235)	7 (1–13)
Chad	2022	21 (21–21)	0 (0–0)	1151 (1141–1161)	9 (9–9)

Chad	2023	21 (21–22)	0 (0–0)	1190 (1168–1213)	9 (9–10)
Chad	2024	22 (21–22)	0 (0–0)	1229 (1191–1267)	9 (8–10)
Chad	2025	22 (21–23)	0 (0–0)	1268 (1213–1324)	9 (8–10)
Chad	2026	22 (21–23)	0 (0–0)	1307 (1232–1383)	9 (7–11)
Chad	2027	23 (21–24)	0 (0–0)	1346 (1249–1443)	9 (7–11)
Chad	2028	23 (21–24)	0 (0–0)	1385 (1265–1506)	9 (6–12)
Chad	2029	23 (22–25)	0 (0–0)	1425 (1279–1570)	9 (6–12)
Chad	2030	23 (22–25)	0 (0–1)	1464 (1292–1635)	9 (5–13)
Chad	2031	24 (22–25)	0 (0–1)	1503 (1303–1702)	9 (4–13)
Chad	2032	24 (22–26)	0 (0–1)	1542 (1313–1771)	9 (4–14)
Chad	2033	24 (22–26)	0 (0–1)	1581 (1321–1840)	9 (3–14)
Chad	2034	25 (23–27)	0 (0–1)	1620 (1329–1911)	9 (2–15)
Chad	2035	25 (23–27)	0 (0–1)	1659 (1335–1983)	8 (1–16)
Chad	2036	25 (23–27)	0 (0–1)	1698 (1340–2056)	8 (1–16)
Chile	2022	213 (204–223)	1 (1–1)	2681 (2541–2821)	21 (20–23)
Chile	2023	213 (200–227)	1 (1–1)	2658 (2456–2860)	20 (18–22)
Chile	2024	213 (197–230)	1 (1–1)	2635 (2349–2921)	19 (16–22)
Chile	2025	213 (194–232)	1 (1–1)	2612 (2226–2998)	17 (14–21)
Chile	2026	213 (192–235)	1 (0–1)	2589 (2091–3087)	16 (12–20)
Chile	2027	213 (190–237)	1 (0–1)	2566 (1945–3187)	14 (10–18)
Chile	2028	213 (188–238)	0 (0–1)	2543 (1788–3298)	13 (9–17)
Chile	2029	213 (186–240)	0 (0–1)	2520 (1623–3417)	12 (7–16)
Chile	2030	213 (185–242)	0 (0–1)	2497 (1450–3545)	10 (5–15)
Chile	2031	213 (183–243)	0 (0–0)	2474 (1268–3680)	9 (3–14)
Chile	2032	213 (181–245)	0 (0–0)	2451 (1079–3823)	7 (2–13)
Chile	2033	213 (180–246)	0 (0–0)	2428 (883–3973)	6 (0–12)
Chile	2034	213 (179–248)	0 (-0–0)	2405 (680–4130)	4 (-1–10)
Chile	2035	213 (177–249)	0 (-0–0)	2382 (471–4294)	3 (-3–9)
Chile	2036	213 (176–250)	0 (-0–0)	2359 (255–4464)	2 (-5–8)
China	2022	[illegible]6100 (64720–67480	3 (3–3)	[illegible]79463 (3221302–33376[illegible]	69 (66–71)
China	2023	[illegible]6558 (63625–69491	3 (3–3)	[illegible]09811 (3182901–34367[illegible]	66 (60–72)
China	2024	[illegible]7016 (63104–70929	3 (2–3)	[illegible]40160 (3170367–35099[illegible]	63 (55–71)
China	2025	[illegible]7474 (62783–72166	3 (2–3)	[illegible]70508 (3166663–35743[illegible]	60 (51–69)

China	2026	7932 (62574–73291	3 (2–3)	00857 (3167884–36338	57 (46–68)
China	2027	8391 (62440–74341	2 (2–3)	31205 (3172362–36900	54 (42–66)
China	2028	8849 (62359–75338	2 (2–3)	61554 (3179201–37439	51 (38–64)
China	2029	9307 (62320–76293	2 (2–3)	91902 (3187852–37959	49 (35–63)
China	2030	9765 (62314–77216	2 (2–3)	22251 (3197952–38465	46 (31–61)
China	2031	0223 (62336–78111	2 (1–3)	52599 (3209243–38959	43 (27–59)
China	2032	0681 (62380–78983	2 (1–3)	82948 (3221538–39443	40 (23–57)
China	2033	1139 (62444–79835	2 (1–3)	13297 (3234693–39919	37 (20–55)
China	2034	1598 (62525–80671	2 (1–2)	43645 (3248596–40386	34 (16–52)
China	2035	2056 (62621–81491	2 (1–2)	73994 (3263156–40848	31 (13–50)
China	2036	2514 (62730–82298	2 (1–2)	04342 (3278302–41303	29 (9–48)
Colombia	2022	259 (243–276)	0 (0–1)	2473 (2227–2720)	12 (10–14)
Colombia	2023	259 (236–282)	0 (0–1)	2435 (2086–2784)	12 (9–14)
Colombia	2024	259 (231–288)	0 (0–1)	2397 (1970–2824)	11 (8–15)
Colombia	2025	259 (226–292)	0 (0–1)	2359 (1866–2852)	11 (7–16)
Colombia	2026	259 (222–296)	0 (0–1)	2321 (1769–2872)	11 (5–16)
Colombia	2027	259 (219–300)	0 (0–1)	2282 (1678–2886)	11 (4–17)
Colombia	2028	259 (216–303)	0 (0–1)	2244 (1592–2896)	10 (3–18)
Colombia	2029	259 (213–306)	0 (0–1)	2206 (1509–2903)	10 (1–19)
Colombia	2030	259 (210–309)	0 (-0–1)	2168 (1428–2907)	10 (-0–20)
Colombia	2031	259 (207–311)	0 (-0–1)	2130 (1350–2909)	10 (-2–21)
Colombia	2032	259 (205–314)	0 (-0–1)	2091 (1274–2909)	9 (-3–22)
Colombia	2033	259 (202–316)	0 (-0–1)	2053 (1199–2907)	9 (-5–23)
Colombia	2034	259 (200–319)	0 (-0–1)	2015 (1126–2904)	9 (-6–24)
Colombia	2035	259 (198–321)	0 (-0–1)	1977 (1054–2899)	9 (-8–26)
Colombia	2036	259 (196–323)	0 (-0–1)	1939 (984–2893)	8 (-10–27)
Comoros	2022	1 (1–1)	0 (0–0)	366 (352–380)	7 (6–7)
Comoros	2023	1 (1–1)	0 (0–0)	370 (350–390)	7 (6–7)
Comoros	2024	1 (1–1)	0 (0–0)	374 (349–398)	7 (6–7)
Comoros	2025	1 (1–2)	0 (0–0)	378 (350–406)	7 (5–8)
Comoros	2026	1 (1–2)	0 (0–0)	382 (350–414)	6 (5–8)
Comoros	2027	1 (1–2)	0 (0–0)	386 (351–421)	6 (5–8)
Comoros	2028	2 (1–2)	0 (0–0)	390 (353–428)	6 (4–8)

Comoros	2029	2 (1–2)	0 (0–0)	394 (354–434)	6 (4–9)
Comoros	2030	2 (1–2)	0 (0–0)	398 (356–441)	6 (4–9)
Comoros	2031	2 (1–2)	0 (0–0)	402 (358–447)	6 (3–9)
Comoros	2032	2 (1–2)	0 (0–0)	406 (359–454)	6 (3–10)
Comoros	2033	2 (1–2)	0 (0–0)	411 (361–460)	6 (2–10)
Comoros	2034	2 (1–2)	0 (0–0)	415 (364–466)	6 (2–10)
Comoros	2035	2 (1–2)	0 (0–0)	419 (366–472)	6 (2–11)
Comoros	2036	2 (1–2)	0 (0–0)	423 (368–478)	6 (1–11)
Congo	2022	8 (8–8)	0 (0–0)	1156 (1136–1176)	8 (7–8)
Congo	2023	8 (8–9)	0 (0–0)	1194 (1155–1234)	8 (7–8)
Congo	2024	9 (8–9)	0 (0–0)	1233 (1170–1297)	8 (7–8)
Congo	2025	9 (8–10)	0 (0–0)	1272 (1181–1363)	8 (6–9)
Congo	2026	9 (8–10)	0 (0–0)	1311 (1189–1433)	7 (6–9)
Congo	2027	9 (8–11)	0 (0–0)	1350 (1195–1505)	7 (5–10)
Congo	2028	9 (8–11)	0 (0–0)	1389 (1198–1580)	7 (5–10)
Congo	2029	10 (7–12)	0 (0–0)	1428 (1198–1657)	7 (4–10)
Congo	2030	10 (7–12)	0 (0–0)	1466 (1197–1736)	7 (4–11)
Congo	2031	10 (7–13)	0 (0–0)	1505 (1193–1818)	7 (3–11)
Congo	2032	10 (7–13)	0 (0–0)	1544 (1187–1902)	7 (2–12)
Congo	2033	10 (6–14)	0 (0–0)	1583 (1179–1987)	7 (2–12)
Congo	2034	10 (6–15)	0 (-0–1)	1622 (1169–2075)	7 (1–13)
Congo	2035	11 (6–15)	0 (-0–1)	1661 (1158–2164)	7 (0–14)
Congo	2036	11 (5–16)	0 (-0–1)	1700 (1144–2255)	7 (-1–14)
Cook Islands	2022	0 (0–0)	0 (0–0)	4 (4–4)	10 (10–10)
Cook Islands	2023	0 (0–0)	0 (0–0)	4 (4–4)	10 (9–11)
Cook Islands	2024	0 (0–0)	0 (0–0)	4 (4–4)	10 (8–11)
Cook Islands	2025	0 (0–0)	0 (0–0)	4 (4–4)	9 (8–11)
Cook Islands	2026	0 (0–0)	0 (0–0)	4 (4–4)	9 (7–12)
Cook Islands	2027	0 (0–0)	0 (0–0)	4 (4–4)	9 (6–12)
Cook Islands	2028	0 (0–0)	0 (0–0)	4 (4–5)	9 (5–13)
Cook Islands	2029	0 (0–0)	0 (0–1)	4 (4–5)	9 (4–14)
Cook Islands	2030	0 (0–0)	0 (0–1)	4 (4–5)	8 (2–14)
Cook Islands	2031	0 (0–0)	0 (0–1)	4 (4–5)	8 (1–15)

Cook Islands	2032	0 (0–0)	0 (-0–1)	4 (4–5)	8 (0–16)
Cook Islands	2033	0 (0–0)	0 (-0–1)	4 (4–5)	8 (-1–17)
Cook Islands	2034	0 (0–0)	0 (-0–1)	4 (4–5)	8 (-3–18)
Cook Islands	2035	0 (0–0)	0 (-0–1)	4 (4–5)	7 (-4–19)
Cook Islands	2036	0 (0–0)	0 (-0–1)	4 (4–5)	7 (-5–20)
Costa Rica	2022	54 (50–59)	1 (1–1)	401 (366–436)	22 (18–26)
Costa Rica	2023	54 (48–61)	1 (1–1)	405 (365–445)	21 (16–26)
Costa Rica	2024	54 (47–62)	1 (0–1)	403 (355–451)	21 (14–27)
Costa Rica	2025	54 (45–63)	1 (0–1)	404 (351–457)	20 (12–28)
Costa Rica	2026	54 (44–64)	1 (0–1)	404 (345–462)	19 (9–29)
Costa Rica	2027	54 (43–65)	1 (0–1)	404 (341–467)	19 (7–30)
Costa Rica	2028	54 (43–66)	0 (0–1)	404 (336–472)	18 (4–32)
Costa Rica	2029	54 (42–67)	0 (-0–1)	404 (332–476)	17 (2–33)
Costa Rica	2030	54 (41–68)	0 (-0–1)	404 (328–480)	17 (-1–34)
Costa Rica	2031	54 (40–68)	0 (-0–1)	404 (324–484)	16 (-4–36)
Costa Rica	2032	54 (40–69)	0 (-0–1)	404 (320–487)	15 (-7–38)
Costa Rica	2033	54 (39–70)	0 (-1–1)	404 (317–491)	15 (-10–39)
Costa Rica	2034	54 (38–70)	-0 (-1–1)	404 (314–494)	14 (-13–41)
Costa Rica	2035	54 (38–71)	-0 (-1–0)	404 (311–497)	13 (-16–43)
Costa Rica	2036	54 (37–72)	-0 (-1–0)	404 (307–500)	13 (-19–45)
Croatia	2022	10 (10–10)	0 (0–0)	554 (545–563)	2 (2–2)
Croatia	2023	10 (10–10)	0 (0–0)	562 (542–582)	2 (2–2)
Croatia	2024	10 (9–11)	0 (0–0)	570 (536–603)	2 (2–2)
Croatia	2025	10 (9–11)	0 (0–0)	578 (529–627)	2 (1–2)
Croatia	2026	10 (8–11)	0 (0–0)	586 (519–652)	2 (1–3)
Croatia	2027	10 (8–12)	0 (0–0)	594 (508–679)	2 (1–3)
Croatia	2028	10 (8–12)	0 (0–0)	601 (496–707)	2 (1–3)
Croatia	2029	10 (7–13)	0 (0–0)	609 (482–737)	2 (0–3)
Croatia	2030	10 (6–13)	0 (0–0)	617 (467–768)	2 (-0–3)
Croatia	2031	10 (6–14)	0 (0–0)	625 (450–800)	1 (-1–3)
Croatia	2032	10 (5–14)	0 (-0–0)	633 (432–834)	1 (-1–4)
Croatia	2033	10 (5–15)	0 (-0–0)	641 (413–868)	1 (-1–4)
Croatia	2034	10 (4–16)	0 (-0–0)	649 (393–904)	1 (-2–4)

Croatia	2035	10 (3–16)	0 (-0–0)	657 (372–941)	1 (-2–4)
Croatia	2036	10 (3–17)	0 (-0–0)	664 (350–979)	1 (-3–5)
Cuba	2022	111 (99–123)	1 (1–1)	2548 (2268–2828)	26 (21–30)
Cuba	2023	107 (90–124)	1 (1–1)	2548 (2153–2944)	24 (18–31)
Cuba	2024	103 (82–124)	1 (1–2)	2548 (2064–3033)	23 (14–32)
Cuba	2025	98 (74–123)	1 (0–2)	2548 (1989–3108)	22 (11–33)
Cuba	2026	94 (67–121)	1 (0–2)	2548 (1922–3174)	20 (7–34)
Cuba	2027	90 (60–120)	1 (0–2)	2548 (1863–3234)	19 (3–35)
Cuba	2028	86 (54–118)	1 (-0–2)	2548 (1808–3289)	18 (-0–36)
Cuba	2029	82 (47–116)	1 (-0–2)	2548 (1757–3340)	17 (-4–37)
Cuba	2030	78 (41–114)	1 (-0–2)	2548 (1709–3388)	15 (-8–39)
Cuba	2031	73 (35–112)	1 (-1–2)	2548 (1663–3434)	14 (-12–40)
Cuba	2032	69 (29–109)	1 (-1–2)	2548 (1620–3477)	13 (-16–41)
Cuba	2033	65 (23–107)	1 (-1–2)	2548 (1579–3518)	12 (-20–43)
Cuba	2034	61 (17–105)	1 (-1–2)	2548 (1539–3558)	10 (-24–44)
Cuba	2035	57 (11–102)	1 (-1–2)	2548 (1501–3596)	9 (-28–46)
Cuba	2036	52 (6–99)	0 (-2–3)	2548 (1464–3632)	8 (-32–48)
Cyprus	2022	102 (97–107)	1 (0–1)	9200 (8820–9579)	12 (11–13)
Cyprus	2023	102 (95–109)	0 (0–1)	9366 (8830–9903)	12 (11–13)
Cyprus	2024	102 (94–111)	0 (0–1)	9533 (8876–10190)	12 (10–13)
Cyprus	2025	102 (92–112)	0 (0–1)	9700 (8941–10459)	11 (10–13)
Cyprus	2026	102 (91–113)	0 (0–1)	9866 (9018–10715)	11 (9–13)
Cyprus	2027	102 (90–114)	0 (0–1)	10033 (9103–10963)	11 (8–13)
Cyprus	2028	102 (89–115)	0 (0–1)	10200 (9196–11204)	10 (8–13)
Cyprus	2029	102 (88–116)	0 (0–1)	10366 (9293–11440)	10 (8–12)
Cyprus	2030	102 (87–117)	0 (0–0)	10533 (9395–11672)	10 (7–12)
Cyprus	2031	102 (87–118)	0 (0–0)	10700 (9500–11900)	9 (7–12)
Cyprus	2032	102 (86–119)	0 (0–0)	10866 (9608–12125)	9 (6–12)
Cyprus	2033	102 (85–120)	0 (0–0)	11033 (9718–12348)	9 (6–12)
Cyprus	2034	102 (84–120)	0 (0–0)	11200 (9831–12568)	8 (5–11)
Cyprus	2035	102 (84–121)	0 (0–0)	11366 (9946–12786)	8 (5–11)
Cyprus	2036	102 (83–122)	0 (0–0)	11533 (10063–13003)	8 (4–11)
Czechia	2022	15 (14–15)	1 (1–1)	280 (269–291)	14 (13–15)

Czechia	2023	15 (14–16)	1 (1–1)	283 (264–302)	13 (12–14)
Czechia	2024	15 (14–16)	1 (1–1)	287 (261–313)	13 (11–14)
Czechia	2025	15 (14–16)	1 (0–1)	291 (259–324)	12 (10–14)
Czechia	2026	15 (14–16)	1 (0–1)	296 (258–334)	12 (10–14)
Czechia	2027	15 (14–17)	1 (0–1)	301 (258–344)	11 (9–14)
Czechia	2028	15 (14–17)	0 (0–1)	306 (259–353)	11 (8–13)
Czechia	2029	15 (14–17)	0 (0–1)	311 (260–362)	10 (7–13)
Czechia	2030	16 (14–17)	0 (0–1)	316 (261–371)	10 (7–13)
Czechia	2031	16 (14–17)	0 (0–1)	321 (262–379)	9 (6–12)
Czechia	2032	16 (14–18)	0 (0–1)	326 (264–388)	9 (5–12)
Czechia	2033	16 (14–18)	0 (0–1)	331 (266–396)	8 (4–11)
Czechia	2034	16 (14–18)	0 (0–0)	336 (268–404)	7 (4–11)
Czechia	2035	16 (14–18)	0 (0–0)	341 (270–412)	7 (3–11)
Czechia	2036	16 (14–18)	0 (0–0)	346 (272–420)	6 (2–10)
C么te d'Ivoire	2022	126 (117–134)	1 (1–1)	6255 (5979–6531)	13 (12–15)
C么te d'Ivoire	2023	124 (111–138)	1 (0–1)	6301 (5911–6692)	13 (11–15)
C么te d'Ivoire	2024	123 (104–142)	1 (0–1)	6348 (5870–6826)	12 (9–15)
C么te d'Ivoire	2025	121 (97–146)	1 (0–1)	6394 (5842–6946)	12 (8–16)
C么te d'Ivoire	2026	120 (90–150)	0 (0–1)	6441 (5823–7058)	11 (6–16)
C么te d'Ivoire	2027	119 (82–155)	0 (0–1)	6487 (5811–7163)	11 (5–17)
C么te d'Ivoire	2028	117 (74–160)	0 (0–1)	6533 (5803–7263)	11 (3–18)
C么te d'Ivoire	2029	116 (66–165)	0 (0–1)	6580 (5799–7360)	10 (2–18)
C么te d'Ivoire	2030	114 (58–171)	0 (0–1)	6626 (5798–7454)	10 (0–19)
C么te d'Ivoire	2031	113 (49–177)	0 (-0–1)	6672 (5800–7545)	9 (-1–20)
C么te d'Ivoire	2032	111 (40–183)	0 (-0–1)	6719 (5803–7634)	9 (-3–20)
C么te d'Ivoire	2033	110 (30–189)	0 (-0–1)	6765 (5809–7721)	8 (-5–21)
C么te d'Ivoire	2034	108 (21–196)	0 (-0–1)	6812 (5816–7807)	8 (-7–22)
C么te d'Ivoire	2035	107 (11–203)	0 (-0–1)	6858 (5825–7891)	7 (-8–23)
C么te d'Ivoire	2036	106 (1–210)	0 (-0–1)	6904 (5835–7973)	7 (-10–24)
mocratic People's Republic of Ko	2022	733 (729–737)	2 (2–2)	30763 (30591–30935)	60 (60–60)
mocratic People's Republic of Ko	2023	739 (729–749)	2 (2–2)	31165 (30780–31549)	59 (58–60)
mocratic People's Republic of Ko	2024	745 (726–763)	2 (2–2)	31567 (30923–32210)	58 (57–60)
mocratic People's Republic of Ko	2025	750 (722–779)	2 (2–2)	31968 (31026–32911)	58 (55–60)

mocratic People's Republic of Ko	2026	756 (717–795)	2 (2–2)	32370 (31094–33646)	57 (53–61)
mocratic People's Republic of Ko	2027	762 (710–813)	2 (2–2)	32772 (31131–34413)	56 (51–61)
mocratic People's Republic of Ko	2028	767 (702–833)	2 (2–2)	33174 (31138–35210)	55 (49–61)
mocratic People's Republic of Ko	2029	773 (693–853)	2 (2–2)	33576 (31119–36033)	54 (47–62)
mocratic People's Republic of Ko	2030	779 (684–874)	2 (2–2)	33978 (31073–36882)	54 (44–63)
mocratic People's Republic of Ko	2031	784 (673–896)	2 (1–2)	34380 (31004–37755)	53 (42–64)
mocratic People's Republic of Ko	2032	790 (661–919)	2 (1–2)	34781 (30912–38651)	52 (39–64)
mocratic People's Republic of Ko	2033	796 (649–942)	2 (1–2)	35183 (30797–39569)	51 (37–65)
mocratic People's Republic of Ko	2034	801 (636–967)	2 (1–2)	35585 (30662–40509)	50 (34–66)
mocratic People's Republic of Ko	2035	807 (622–992)	2 (1–2)	35987 (30506–41468)	50 (32–68)
mocratic People's Republic of Ko	2036	813 (607–1018)	2 (1–2)	36389 (30331–42447)	49 (29–69)
Democratic Republic of the Congo	2022	80 (78–81)	0 (0–0)	8190 (8042–8337)	5 (5–6)
Democratic Republic of the Congo	2023	81 (79–84)	0 (0–0)	8422 (8164–8681)	5 (5–6)
Democratic Republic of the Congo	2024	83 (79–87)	0 (0–0)	8655 (8276–9033)	5 (5–6)
Democratic Republic of the Congo	2025	85 (80–90)	0 (0–0)	8887 (8378–9397)	5 (5–6)
Democratic Republic of the Congo	2026	86 (80–93)	0 (0–0)	9120 (8469–9771)	5 (5–6)
Democratic Republic of the Congo	2027	88 (80–96)	0 (0–0)	9353 (8550–10156)	5 (4–6)
Democratic Republic of the Congo	2028	90 (80–99)	0 (0–0)	9585 (8621–10550)	5 (4–6)
Democratic Republic of the Congo	2029	91 (80–103)	0 (0–0)	9818 (8683–10953)	5 (4–6)
Democratic Republic of the Congo	2030	93 (80–106)	0 (0–0)	10051 (8736–11365)	5 (4–6)
Democratic Republic of the Congo	2031	95 (80–110)	0 (0–0)	10283 (8781–11785)	5 (3–6)
Democratic Republic of the Congo	2032	96 (80–113)	0 (0–0)	10516 (8818–12214)	5 (3–7)
Democratic Republic of the Congo	2033	98 (79–117)	0 (0–0)	10749 (8847–12650)	5 (3–7)
Democratic Republic of the Congo	2034	100 (79–121)	0 (0–0)	10981 (8869–13094)	5 (3–7)
Democratic Republic of the Congo	2035	102 (78–125)	0 (0–0)	11214 (8883–13545)	5 (2–7)
Democratic Republic of the Congo	2036	103 (78–128)	0 (0–0)	11446 (8890–14003)	5 (2–7)
Denmark	2022	82 (76–87)	1 (1–1)	5301 (5067–5536)	13 (12–14)
Denmark	2023	82 (72–91)	1 (1–1)	5269 (4906–5633)	13 (11–15)
Denmark	2024	82 (68–96)	1 (0–1)	5253 (4863–5643)	12 (9–16)
Denmark	2025	82 (63–101)	1 (0–1)	5245 (4848–5641)	12 (8–16)
Denmark	2026	82 (58–106)	1 (0–1)	5240 (4842–5639)	12 (7–17)
Denmark	2027	82 (53–111)	1 (0–1)	5238 (4839–5637)	11 (5–18)
Denmark	2028	82 (48–117)	1 (0–1)	5237 (4838–5636)	11 (4–19)

Denmark	2029	82 (42–123)	1 (0–1)	5237 (4838–5636)	11 (2–19)
Denmark	2030	83 (36–129)	1 (0–1)	5236 (4837–5635)	11 (1–20)
Denmark	2031	83 (29–136)	1 (-0–1)	5236 (4837–5635)	10 (-1–21)
Denmark	2032	83 (23–143)	0 (-0–1)	5236 (4837–5635)	10 (-3–23)
Denmark	2033	83 (16–150)	0 (-0–1)	5236 (4837–5635)	10 (-5–24)
Denmark	2034	83 (9–157)	0 (-0–1)	5236 (4837–5635)	9 (-6–25)
Denmark	2035	83 (1–165)	0 (-0–1)	5236 (4837–5635)	9 (-8–26)
Denmark	2036	83 (-6–173)	0 (-0–1)	5236 (4837–5635)	9 (-10–27)
Djibouti	2022	3 (3–3)	1 (0–1)	463 (457–469)	12 (12–12)
Djibouti	2023	3 (3–3)	0 (0–1)	476 (468–483)	12 (11–12)
Djibouti	2024	3 (3–3)	0 (0–0)	488 (478–498)	11 (11–12)
Djibouti	2025	3 (3–3)	0 (0–0)	500 (488–512)	11 (10–12)
Djibouti	2026	3 (3–3)	0 (0–0)	512 (497–527)	11 (10–12)
Djibouti	2027	3 (3–4)	0 (0–0)	525 (507–542)	10 (9–12)
Djibouti	2028	3 (3–4)	0 (0–0)	537 (516–558)	10 (9–11)
Djibouti	2029	4 (3–4)	0 (0–0)	549 (525–573)	10 (8–11)
Djibouti	2030	4 (3–4)	0 (0–0)	561 (534–589)	10 (8–11)
Djibouti	2031	4 (3–4)	0 (0–0)	574 (543–604)	9 (7–11)
Djibouti	2032	4 (3–4)	0 (0–0)	586 (552–620)	9 (7–11)
Djibouti	2033	4 (3–4)	0 (0–0)	598 (561–636)	9 (6–11)
Djibouti	2034	4 (3–4)	0 (0–0)	611 (569–652)	8 (5–11)
Djibouti	2035	4 (4–4)	0 (0–0)	623 (577–668)	8 (5–12)
Djibouti	2036	4 (4–4)	0 (0–0)	635 (586–685)	8 (4–12)
Dominica	2022	1 (1–1)	1 (1–1)	16 (16–16)	19 (19–19)
Dominica	2023	1 (1–1)	1 (1–1)	16 (15–16)	19 (18–20)
Dominica	2024	1 (1–1)	1 (1–1)	16 (15–17)	19 (17–20)
Dominica	2025	1 (1–1)	1 (1–1)	16 (15–17)	18 (17–20)
Dominica	2026	1 (1–1)	1 (1–1)	16 (15–18)	18 (16–21)
Dominica	2027	1 (1–1)	1 (1–1)	16 (15–18)	18 (15–21)
Dominica	2028	1 (1–1)	1 (1–1)	17 (14–19)	18 (15–22)
Dominica	2029	1 (1–1)	1 (1–1)	17 (14–19)	18 (14–22)
Dominica	2030	1 (1–1)	1 (1–1)	17 (14–20)	18 (13–23)
Dominica	2031	1 (1–1)	1 (1–1)	17 (13–21)	18 (12–23)

Dominica	2032	1 (1–1)	1 (0–1)	17 (13–21)	17 (11–24)
Dominica	2033	1 (1–1)	1 (0–1)	17 (13–22)	17 (10–25)
Dominica	2034	1 (1–1)	1 (0–1)	17 (12–23)	17 (9–25)
Dominica	2035	1 (1–1)	1 (0–1)	18 (12–23)	17 (8–26)
Dominica	2036	1 (1–1)	1 (0–1)	18 (11–24)	17 (7–27)
Dominican Republic	2022	61 (57–64)	1 (1–1)	1633 (1550–1717)	13 (12–14)
Dominican Republic	2023	62 (56–67)	1 (0–1)	1666 (1548–1784)	13 (11–14)
Dominican Republic	2024	62 (56–69)	1 (0–1)	1698 (1553–1842)	13 (10–15)
Dominican Republic	2025	63 (56–71)	1 (0–1)	1730 (1563–1897)	12 (10–15)
Dominican Republic	2026	64 (56–73)	1 (0–1)	1763 (1576–1949)	12 (9–16)
Dominican Republic	2027	65 (56–74)	1 (0–1)	1795 (1590–1999)	12 (9–16)
Dominican Republic	2028	66 (57–76)	1 (0–1)	1827 (1606–2048)	12 (8–17)
Dominican Republic	2029	67 (57–78)	1 (0–1)	1859 (1623–2095)	12 (8–17)
Dominican Republic	2030	68 (57–79)	1 (0–1)	1892 (1641–2142)	12 (8–17)
Dominican Republic	2031	69 (58–81)	1 (0–1)	1924 (1660–2188)	12 (7–18)
Dominican Republic	2032	70 (58–82)	1 (0–1)	1956 (1680–2233)	12 (7–18)
Dominican Republic	2033	71 (58–84)	1 (0–1)	1989 (1700–2278)	12 (7–18)
Dominican Republic	2034	72 (59–85)	1 (0–1)	2021 (1720–2322)	12 (6–19)
Dominican Republic	2035	73 (59–87)	1 (0–1)	2053 (1741–2365)	12 (6–19)
Dominican Republic	2036	74 (60–88)	1 (0–1)	2086 (1762–2409)	12 (6–19)
Ecuador	2022	117 (108–126)	1 (1–1)	583 (525–641)	16 (14–19)
Ecuador	2023	116 (101–130)	1 (0–1)	583 (501–666)	15 (12–19)
Ecuador	2024	114 (94–134)	1 (0–1)	583 (482–684)	15 (10–19)
Ecuador	2025	113 (87–138)	1 (0–1)	583 (467–700)	14 (8–19)
Ecuador	2026	111 (80–142)	1 (0–1)	583 (453–714)	13 (7–19)
Ecuador	2027	110 (73–146)	1 (0–1)	583 (440–726)	12 (5–18)
Ecuador	2028	108 (66–151)	0 (0–1)	583 (429–737)	11 (4–18)
Ecuador	2029	107 (58–155)	0 (0–1)	583 (418–748)	10 (3–17)
Ecuador	2030	105 (50–160)	0 (0–1)	583 (408–758)	9 (1–17)
Ecuador	2031	104 (42–166)	0 (-0–1)	583 (399–768)	8 (-0–16)
Ecuador	2032	102 (33–171)	0 (-0–1)	583 (390–777)	7 (-1–16)
Ecuador	2033	101 (25–177)	0 (-0–1)	583 (381–785)	6 (-3–15)
Ecuador	2034	99 (16–183)	0 (-0–1)	583 (373–793)	5 (-4–15)

Ecuador	2035	98 (7–189)	0 (-0–1)	583 (365–801)	5 (-5–14)
Ecuador	2036	96 (-3–195)	0 (-0–1)	583 (357–809)	4 (-6–14)
Egypt	2022	433 (418–448)	1 (1–1)	5985 (5782–6188)	17 (16–18)
Egypt	2023	441 (406–475)	1 (1–1)	6086 (5799–6373)	17 (15–19)
Egypt	2024	450 (400–499)	1 (1–1)	6187 (5836–6538)	17 (14–20)
Egypt	2025	459 (398–521)	1 (1–1)	6288 (5882–6693)	17 (13–21)
Egypt	2026	469 (398–540)	1 (1–1)	6389 (5936–6842)	17 (12–22)
Egypt	2027	479 (399–559)	1 (0–1)	6490 (5993–6986)	17 (11–23)
Egypt	2028	489 (400–577)	1 (0–1)	6591 (6054–7127)	17 (10–24)
Egypt	2029	498 (403–594)	1 (0–1)	6692 (6118–7265)	17 (9–25)
Egypt	2030	508 (406–610)	1 (0–1)	6793 (6185–7401)	17 (8–26)
Egypt	2031	518 (409–626)	1 (0–1)	6894 (6253–7535)	17 (7–27)
Egypt	2032	527 (413–642)	1 (0–1)	6995 (6322–7667)	17 (6–27)
Egypt	2033	537 (417–657)	1 (0–1)	7096 (6393–7798)	17 (5–28)
Egypt	2034	547 (421–672)	1 (0–1)	7197 (6466–7928)	17 (4–29)
Egypt	2035	557 (426–687)	1 (0–1)	7298 (6539–8056)	17 (4–30)
Egypt	2036	566 (430–702)	1 (0–1)	7399 (6613–8184)	17 (3–30)
El Salvador	2022	37 (34–40)	1 (1–1)	349 (322–376)	16 (14–18)
El Salvador	2023	38 (34–42)	1 (1–1)	354 (317–392)	16 (14–19)
El Salvador	2024	39 (33–44)	1 (0–1)	360 (314–407)	16 (13–20)
El Salvador	2025	39 (33–45)	1 (0–1)	366 (312–420)	16 (13–20)
El Salvador	2026	40 (33–46)	1 (0–1)	372 (312–432)	16 (12–20)
El Salvador	2027	40 (33–48)	1 (0–1)	378 (312–443)	16 (12–21)
El Salvador	2028	41 (33–49)	1 (0–1)	383 (312–454)	16 (11–21)
El Salvador	2029	42 (33–50)	1 (0–1)	389 (313–465)	16 (11–22)
El Salvador	2030	42 (33–51)	1 (0–1)	395 (314–475)	16 (11–22)
El Salvador	2031	43 (33–52)	1 (0–1)	401 (316–485)	16 (10–22)
El Salvador	2032	43 (33–53)	1 (0–1)	406 (317–495)	16 (10–22)
El Salvador	2033	44 (34–54)	1 (0–1)	412 (319–505)	16 (10–23)
El Salvador	2034	45 (34–55)	1 (0–1)	418 (321–515)	16 (10–23)
El Salvador	2035	45 (34–56)	1 (0–1)	424 (323–524)	16 (9–23)
El Salvador	2036	46 (34–57)	1 (0–1)	429 (325–533)	16 (9–24)
Equatorial Guinea	2022	1 (1–1)	0 (0–0)	170 (166–174)	5 (5–6)

Equatorial Guinea	2023	1 (1–1)	0 (0–0)	175 (166–185)	5 (4–6)
Equatorial Guinea	2024	1 (1–1)	0 (0–0)	180 (164–196)	5 (4–7)
Equatorial Guinea	2025	1 (1–1)	0 (0–0)	185 (162–209)	5 (3–8)
Equatorial Guinea	2026	1 (1–1)	0 (0–0)	191 (159–223)	5 (2–8)
Equatorial Guinea	2027	1 (1–2)	0 (0–0)	196 (155–237)	5 (1–9)
Equatorial Guinea	2028	1 (1–2)	0 (0–0)	201 (150–252)	5 (-0–10)
Equatorial Guinea	2029	1 (1–2)	0 (-0–0)	206 (144–267)	5 (-1–11)
Equatorial Guinea	2030	1 (1–2)	0 (-0–0)	211 (138–284)	5 (-3–12)
Equatorial Guinea	2031	1 (0–2)	0 (-0–0)	216 (131–301)	5 (-4–13)
Equatorial Guinea	2032	1 (0–2)	0 (-0–0)	221 (124–318)	5 (-5–14)
Equatorial Guinea	2033	1 (0–2)	0 (-0–1)	226 (116–336)	5 (-7–16)
Equatorial Guinea	2034	1 (0–2)	0 (-0–1)	231 (108–355)	5 (-8–17)
Equatorial Guinea	2035	1 (0–3)	0 (-0–1)	236 (99–374)	4 (-9–18)
Equatorial Guinea	2036	1 (-0–3)	0 (-0–1)	242 (90–393)	4 (-11–20)
Eritrea	2022	6 (6–6)	0 (0–0)	1066 (1026–1106)	5 (5–5)
Eritrea	2023	6 (5–6)	0 (0–0)	1044 (963–1124)	5 (4–5)
Eritrea	2024	6 (5–6)	0 (0–0)	1017 (909–1124)	5 (4–5)
Eritrea	2025	6 (5–6)	0 (0–0)	999 (879–1119)	5 (4–5)
Eritrea	2026	6 (5–6)	0 (0–0)	993 (868–1117)	4 (3–5)
Eritrea	2027	6 (5–7)	0 (0–0)	992 (865–1119)	4 (3–5)
Eritrea	2028	6 (5–7)	0 (0–0)	990 (861–1119)	4 (3–5)
Eritrea	2029	6 (5–7)	0 (0–0)	985 (853–1117)	4 (3–5)
Eritrea	2030	6 (5–7)	0 (0–0)	978 (843–1113)	4 (2–5)
Eritrea	2031	6 (5–7)	0 (0–0)	972 (835–1109)	4 (2–5)
Eritrea	2032	6 (5–7)	0 (0–0)	967 (828–1106)	3 (2–5)
Eritrea	2033	6 (5–7)	0 (0–0)	964 (824–1103)	3 (1–5)
Eritrea	2034	6 (5–7)	0 (0–0)	961 (820–1102)	3 (1–5)
Eritrea	2035	6 (5–7)	0 (0–0)	958 (817–1100)	3 (1–5)
Eritrea	2036	6 (5–7)	0 (0–0)	956 (814–1098)	3 (1–5)
Estonia	2022	22 (19–26)	1 (1–1)	636 (560–712)	22 (18–26)
Estonia	2023	21 (17–26)	1 (1–1)	636 (528–743)	20 (14–26)
Estonia	2024	20 (15–26)	1 (0–1)	636 (504–767)	19 (11–26)
Estonia	2025	19 (13–26)	1 (0–1)	636 (484–788)	17 (8–26)

Estonia	2026	18 (11–26)	1 (0–1)	636 (466–806)	15 (6–25)
Estonia	2027	17 (10–25)	1 (0–1)	636 (450–822)	14 (3–24)
Estonia	2028	16 (8–25)	1 (0–1)	636 (435–837)	12 (1–23)
Estonia	2029	15 (6–24)	0 (0–1)	636 (421–851)	10 (-2–23)
Estonia	2030	14 (5–24)	0 (-0–1)	636 (408–864)	9 (-4–22)
Estonia	2031	13 (3–24)	0 (-0–1)	636 (395–876)	7 (-7–21)
Estonia	2032	12 (2–23)	0 (-0–1)	636 (384–888)	5 (-9–20)
Estonia	2033	11 (0–23)	0 (-0–1)	636 (373–899)	4 (-11–19)
Estonia	2034	10 (-1–22)	0 (-0–1)	636 (362–910)	2 (-13–18)
Estonia	2035	9 (-2–21)	0 (-0–1)	636 (351–920)	0 (-16–17)
Estonia	2036	8 (-4–21)	0 (-0–1)	636 (341–930)	-1 (-18–15)
Eswatini	2022	1 (1–1)	0 (0–0)	275 (265–285)	7 (6–7)
Eswatini	2023	1 (1–1)	0 (0–0)	265 (240–291)	6 (5–7)
Eswatini	2024	1 (1–1)	0 (0–0)	255 (209–301)	6 (5–7)
Eswatini	2025	1 (1–1)	0 (0–0)	245 (174–316)	6 (4–7)
Eswatini	2026	1 (1–2)	0 (0–0)	235 (136–334)	5 (3–7)
Eswatini	2027	1 (0–2)	0 (0–0)	225 (95–355)	5 (2–8)
Eswatini	2028	1 (0–2)	0 (0–0)	215 (50–379)	4 (1–8)
Eswatini	2029	1 (-0–2)	0 (-0–0)	204 (4–405)	4 (-0–9)
Eswatini	2030	1 (-0–2)	0 (-0–0)	194 (-45–434)	4 (-1–9)
Eswatini	2031	1 (-1–2)	0 (-0–0)	184 (-97–465)	3 (-3–10)
Eswatini	2032	1 (-1–2)	0 (-0–0)	174 (-150–498)	3 (-4–10)
Eswatini	2033	1 (-1–2)	0 (-0–0)	164 (-206–534)	3 (-5–11)
Eswatini	2034	0 (-1–2)	0 (-0–0)	154 (-263–571)	2 (-6–11)
Eswatini	2035	0 (-2–2)	0 (-0–0)	143 (-323–610)	2 (-8–12)
Eswatini	2036	0 (-2–3)	0 (-0–0)	133 (-384–651)	2 (-9–13)
Ethiopia	2022	41 (40–41)	0 (0–0)	3011 (2981–3041)	2 (2–2)
Ethiopia	2023	41 (40–43)	0 (0–0)	3090 (3023–3157)	2 (2–3)
Ethiopia	2024	41 (39–44)	0 (0–0)	3169 (3056–3281)	2 (2–3)
Ethiopia	2025	42 (38–45)	0 (0–0)	3247 (3083–3412)	2 (2–3)
Ethiopia	2026	42 (37–47)	0 (0–0)	3326 (3104–3549)	2 (2–3)
Ethiopia	2027	42 (36–49)	0 (0–0)	3405 (3119–3691)	2 (2–3)
Ethiopia	2028	43 (35–50)	0 (0–0)	3484 (3129–3839)	2 (1–3)

Ethiopia	2029	43 (34–52)	0 (0–0)	3563 (3134–3991)	2 (1–3)
Ethiopia	2030	43 (32–54)	0 (0–0)	3641 (3135–4148)	2 (1–3)
Ethiopia	2031	44 (31–56)	0 (0–0)	3720 (3132–4309)	2 (1–4)
Ethiopia	2032	44 (29–58)	0 (0–0)	3799 (3124–4474)	2 (0–4)
Ethiopia	2033	44 (28–61)	0 (0–0)	3878 (3113–4642)	2 (0–4)
Ethiopia	2034	45 (26–63)	0 (-0–0)	3956 (3098–4815)	2 (-0–4)
Ethiopia	2035	45 (24–65)	0 (-0–0)	4035 (3080–4991)	2 (-0–4)
Ethiopia	2036	45 (22–68)	0 (-0–0)	4114 (3058–5170)	2 (-1–5)
Fiji	2022	3 (3–4)	0 (0–0)	113 (108–117)	11 (11–12)
Fiji	2023	3 (3–4)	0 (0–0)	114 (108–121)	11 (10–13)
Fiji	2024	3 (3–4)	0 (0–0)	116 (108–124)	11 (9–13)
Fiji	2025	3 (3–4)	0 (0–0)	118 (109–126)	11 (9–13)
Fiji	2026	3 (3–4)	0 (0–0)	119 (109–129)	11 (8–14)
Fiji	2027	3 (3–4)	0 (0–1)	121 (110–132)	11 (8–14)
Fiji	2028	3 (3–4)	0 (0–1)	122 (110–134)	11 (7–14)
Fiji	2029	3 (3–4)	0 (0–1)	124 (111–136)	10 (6–14)
Fiji	2030	3 (3–4)	0 (0–1)	125 (112–139)	10 (6–15)
Fiji	2031	3 (3–4)	0 (0–1)	127 (113–141)	10 (5–15)
Fiji	2032	3 (3–4)	0 (0–1)	128 (114–143)	10 (5–16)
Fiji	2033	3 (3–4)	0 (0–1)	130 (115–145)	10 (4–16)
Fiji	2034	3 (3–4)	0 (0–1)	132 (116–148)	10 (3–16)
Fiji	2035	3 (3–4)	0 (0–1)	133 (117–150)	10 (2–17)
Fiji	2036	3 (3–4)	0 (0–1)	135 (118–152)	9 (2–17)
Finland	2022	33 (32–35)	0 (0–0)	1945 (1849–2042)	6 (6–7)
Finland	2023	33 (30–36)	0 (0–0)	1945 (1837–2053)	6 (5–7)
Finland	2024	32 (28–37)	0 (0–0)	1945 (1815–2075)	6 (4–8)
Finland	2025	32 (26–38)	0 (0–0)	1945 (1802–2089)	6 (3–8)
Finland	2026	31 (23–39)	0 (0–0)	1945 (1787–2103)	6 (2–9)
Finland	2027	31 (21–40)	0 (0–0)	1945 (1775–2115)	5 (1–9)
Finland	2028	30 (18–42)	0 (0–0)	1945 (1763–2127)	5 (0–10)
Finland	2029	29 (16–43)	0 (0–0)	1945 (1752–2138)	5 (-1–11)
Finland	2030	29 (13–45)	0 (-0–0)	1945 (1741–2149)	5 (-2–12)
Finland	2031	28 (10–46)	0 (-0–0)	1945 (1731–2159)	5 (-3–13)

Finland	2032	28 (7–48)	0 (-0–0)	1945 (1722–2168)	4 (-5–14)
Finland	2033	27 (4–50)	0 (-0–0)	1945 (1713–2177)	4 (-6–15)
Finland	2034	26 (1–52)	0 (-0–1)	1945 (1704–2186)	4 (-8–16)
Finland	2035	26 (-2–54)	0 (-0–1)	1945 (1696–2195)	4 (-9–17)
Finland	2036	25 (-5–56)	0 (-0–1)	1945 (1687–2203)	4 (-11–18)
France	2022	542 (528–556)	0 (0–0)	33479 (31248–35709)	9 (9–10)
France	2023	516 (485–548)	0 (0–0)	32331 (29177–35486)	9 (8–9)
France	2024	491 (438–544)	0 (0–0)	31184 (27320–35047)	8 (7–9)
France	2025	466 (388–543)	0 (0–0)	30037 (25576–34498)	8 (6–9)
France	2026	440 (335–545)	0 (0–0)	28889 (23902–33877)	7 (4–9)
France	2027	415 (280–550)	0 (0–0)	27742 (22278–33206)	6 (3–10)
France	2028	390 (222–557)	0 (0–0)	26595 (20693–32496)	6 (2–10)
France	2029	364 (162–566)	0 (0–0)	25448 (19139–31757)	5 (0–10)
France	2030	339 (100–578)	0 (-0–0)	24300 (17609–30992)	5 (-1–10)
France	2031	313 (36–591)	0 (-0–0)	23153 (16099–30207)	4 (-3–11)
France	2032	288 (-30–606)	0 (-0–0)	22006 (14608–29404)	3 (-4–11)
France	2033	263 (-98–623)	0 (-0–0)	20858 (13132–28585)	3 (-6–12)
France	2034	237 (-168–642)	0 (-0–0)	19711 (11669–27754)	2 (-8–12)
France	2035	212 (-239–663)	0 (-0–0)	18564 (10218–26910)	2 (-9–13)
France	2036	187 (-312–685)	0 (-0–1)	17417 (8778–26056)	1 (-11–13)
Gabon	2022	3 (3–3)	0 (0–0)	379 (373–385)	6 (6–7)
Gabon	2023	3 (3–3)	0 (0–0)	382 (368–395)	6 (6–7)
Gabon	2024	3 (3–3)	0 (0–0)	384 (362–407)	6 (6–6)
Gabon	2025	3 (3–3)	0 (0–0)	387 (354–420)	6 (6–6)
Gabon	2026	3 (3–3)	0 (0–0)	390 (345–435)	6 (6–6)
Gabon	2027	3 (3–3)	0 (0–0)	393 (336–451)	6 (5–6)
Gabon	2028	3 (3–3)	0 (0–0)	396 (325–467)	6 (5–6)
Gabon	2029	3 (3–3)	0 (0–0)	399 (313–485)	6 (5–6)
Gabon	2030	3 (3–3)	0 (0–0)	402 (300–504)	6 (5–6)
Gabon	2031	3 (3–3)	0 (0–0)	405 (286–523)	5 (5–6)
Gabon	2032	3 (3–3)	0 (0–0)	408 (272–543)	5 (5–6)
Gabon	2033	3 (3–3)	0 (0–0)	410 (256–564)	5 (5–6)
Gabon	2034	3 (3–3)	0 (0–0)	413 (241–586)	5 (5–6)

Gabon	2035	3 (3–3)	0 (0–0)	416 (224–609)	5 (4–6)
Gabon	2036	3 (3–3)	0 (0–0)	419 (206–632)	5 (4–6)
Gambia	2022	1 (1–1)	0 (0–0)	70 (67–74)	3 (3–3)
Gambia	2023	1 (1–1)	0 (0–0)	72 (67–76)	3 (2–3)
Gambia	2024	1 (1–1)	0 (0–0)	73 (68–79)	3 (2–3)
Gambia	2025	1 (1–1)	0 (0–0)	75 (68–81)	3 (2–3)
Gambia	2026	1 (1–1)	0 (0–0)	76 (69–83)	3 (2–3)
Gambia	2027	1 (1–1)	0 (0–0)	77 (70–85)	2 (2–3)
Gambia	2028	1 (1–1)	0 (0–0)	79 (71–87)	2 (2–3)
Gambia	2029	1 (1–1)	0 (0–0)	80 (71–89)	2 (2–3)
Gambia	2030	1 (1–1)	0 (0–0)	82 (72–91)	2 (1–3)
Gambia	2031	1 (1–1)	0 (0–0)	83 (73–93)	2 (1–3)
Gambia	2032	1 (1–1)	0 (0–0)	84 (74–95)	2 (1–3)
Gambia	2033	1 (1–1)	0 (0–0)	86 (75–96)	2 (1–3)
Gambia	2034	1 (1–1)	0 (0–0)	87 (76–98)	2 (1–3)
Gambia	2035	1 (1–1)	0 (0–0)	88 (77–100)	2 (1–3)
Gambia	2036	1 (1–1)	0 (0–0)	90 (78–102)	2 (1–3)
Georgia	2022	91 (78–105)	2 (1–2)	662 (542–783)	39 (33–45)
Georgia	2023	93 (71–116)	2 (1–2)	651 (430–872)	41 (31–50)
Georgia	2024	98 (69–126)	2 (1–2)	645 (334–956)	43 (31–56)
Georgia	2025	100 (70–130)	2 (1–2)	642 (250–1034)	45 (32–57)
Georgia	2026	102 (71–132)	2 (1–2)	640 (176–1104)	45 (32–57)
Georgia	2027	102 (72–133)	2 (1–2)	639 (110–1168)	45 (32–57)
Georgia	2028	103 (72–133)	2 (1–2)	638 (51–1226)	45 (32–57)
Georgia	2029	103 (72–134)	2 (1–3)	638 (-4–1280)	45 (32–57)
Georgia	2030	103 (73–134)	2 (0–3)	638 (-54–1330)	45 (32–57)
Georgia	2031	103 (73–134)	2 (0–3)	638 (-101–1377)	45 (32–57)
Georgia	2032	103 (73–134)	2 (0–3)	638 (-145–1421)	45 (32–57)
Georgia	2033	103 (73–134)	2 (0–3)	638 (-187–1463)	45 (32–57)
Georgia	2034	103 (73–134)	2 (0–3)	638 (-227–1503)	45 (32–57)
Georgia	2035	103 (73–134)	2 (0–3)	638 (-266–1541)	45 (32–57)
Georgia	2036	103 (73–134)	2 (0–3)	638 (-302–1578)	45 (32–57)
Germany	2022	1186 (1128–1244)	1 (1–1)	61206 (56998–65415)	14 (13–15)

Germany	2023	1161 (1067–1254)	1 (1–1)	61206 (55254–67159)	13 (12–15)
Germany	2024	1135 (1007–1264)	1 (0–1)	61206 (53916–68497)	13 (11–15)
Germany	2025	1110 (945–1275)	1 (0–1)	61206 (52789–69624)	12 (10–15)
Germany	2026	1085 (881–1288)	1 (0–1)	61206 (51795–70618)	12 (8–15)
Germany	2027	1059 (815–1303)	0 (0–1)	61206 (50897–71516)	11 (7–15)
Germany	2028	1034 (748–1320)	0 (0–1)	61206 (50071–72342)	11 (6–16)
Germany	2029	1009 (678–1339)	0 (0–1)	61206 (49302–73111)	10 (5–16)
Germany	2030	984 (607–1360)	0 (0–1)	61206 (48580–73833)	10 (3–16)
Germany	2031	958 (534–1383)	0 (0–1)	61206 (47897–74516)	9 (2–17)
Germany	2032	933 (459–1407)	0 (-0–1)	61206 (47247–75166)	9 (0–17)
Germany	2033	908 (382–1433)	0 (-0–1)	61206 (46626–75787)	8 (-1–17)
Germany	2034	882 (304–1461)	0 (-0–1)	61206 (46031–76382)	8 (-3–18)
Germany	2035	857 (224–1490)	0 (-0–1)	61206 (45458–76955)	7 (-4–18)
Germany	2036	832 (143–1521)	0 (-0–1)	61206 (44905–77508)	7 (-6–19)
Ghana	2022	21 (21–21)	0 (0–0)	1202 (1191–1213)	3 (3–3)
Ghana	2023	21 (20–22)	0 (0–0)	1217 (1193–1242)	3 (3–3)
Ghana	2024	21 (20–22)	0 (0–0)	1233 (1192–1273)	3 (3–3)
Ghana	2025	22 (20–23)	0 (0–0)	1248 (1189–1307)	3 (3–3)
Ghana	2026	22 (20–24)	0 (0–0)	1263 (1183–1343)	3 (2–3)
Ghana	2027	22 (20–24)	0 (0–0)	1279 (1175–1382)	3 (2–3)
Ghana	2028	22 (20–25)	0 (0–0)	1294 (1166–1422)	3 (2–3)
Ghana	2029	23 (20–25)	0 (0–0)	1309 (1155–1463)	3 (2–3)
Ghana	2030	23 (20–26)	0 (0–0)	1324 (1142–1507)	3 (2–3)
Ghana	2031	23 (20–26)	0 (0–0)	1340 (1128–1552)	3 (2–3)
Ghana	2032	23 (20–27)	0 (0–0)	1355 (1112–1598)	2 (2–3)
Ghana	2033	24 (20–27)	0 (0–0)	1370 (1095–1646)	2 (2–3)
Ghana	2034	24 (20–27)	0 (0–0)	1385 (1076–1695)	2 (1–3)
Ghana	2035	24 (20–28)	0 (0–0)	1401 (1056–1745)	2 (1–3)
Ghana	2036	24 (20–28)	0 (0–0)	1416 (1035–1797)	2 (1–3)
Greece	2022	312 (294–331)	1 (1–1)	4264 (4062–4465)	27 (25–29)
Greece	2023	308 (282–333)	1 (1–1)	4248 (3991–4505)	26 (23–29)
Greece	2024	303 (271–335)	1 (1–1)	4235 (3949–4522)	25 (21–29)
Greece	2025	298 (262–335)	1 (1–1)	4225 (3922–4529)	25 (20–30)

Greece	2026	293 (253–334)	1 (1–1)	4218 (3903–4532)	24 (18–30)
Greece	2027	289 (244–333)	1 (1–1)	4211 (3891–4532)	23 (16–30)
Greece	2028	284 (236–332)	1 (1–1)	4206 (3882–4531)	23 (14–31)
Greece	2029	279 (227–331)	1 (1–1)	4202 (3875–4529)	22 (12–32)
Greece	2030	274 (220–329)	1 (0–1)	4199 (3871–4528)	21 (10–32)
Greece	2031	270 (212–327)	1 (0–1)	4197 (3867–4526)	21 (8–33)
Greece	2032	265 (204–326)	1 (0–1)	4195 (3865–4525)	20 (6–33)
Greece	2033	260 (197–324)	1 (0–1)	4193 (3863–4524)	19 (4–34)
Greece	2034	255 (190–321)	0 (0–1)	4192 (3861–4523)	18 (2–35)
Greece	2035	251 (182–319)	0 (0–1)	4191 (3860–4522)	18 (-0–36)
Greece	2036	246 (175–317)	0 (0–1)	4190 (3859–4521)	17 (-2–37)
Greenland	2022	1 (1–1)	1 (1–1)	92 (89–95)	26 (25–28)
Greenland	2023	1 (1–1)	1 (1–1)	91 (88–95)	25 (22–27)
Greenland	2024	1 (1–1)	1 (1–1)	90 (86–94)	23 (19–27)
Greenland	2025	1 (1–1)	1 (1–1)	89 (84–94)	21 (16–26)
Greenland	2026	1 (1–1)	1 (1–1)	88 (82–94)	19 (13–26)
Greenland	2027	1 (1–1)	1 (0–1)	87 (80–95)	18 (10–25)
Greenland	2028	1 (1–1)	1 (0–1)	87 (78–95)	16 (6–25)
Greenland	2029	1 (1–1)	1 (0–1)	86 (76–95)	14 (3–25)
Greenland	2030	1 (1–1)	1 (0–1)	85 (74–96)	12 (-1–25)
Greenland	2031	1 (1–1)	0 (-0–1)	84 (71–96)	11 (-4–25)
Greenland	2032	1 (1–1)	0 (-0–1)	83 (69–97)	9 (-8–25)
Greenland	2033	1 (1–1)	0 (-0–1)	82 (66–98)	7 (-12–25)
Greenland	2034	1 (1–1)	0 (-1–1)	81 (64–99)	5 (-15–26)
Greenland	2035	1 (0–1)	0 (-1–1)	80 (61–99)	3 (-19–26)
Greenland	2036	1 (0–1)	0 (-1–1)	79 (58–100)	2 (-23–27)
Grenada	2022	0 (0–0)	0 (0–0)	24 (20–27)	8 (7–8)
Grenada	2023	0 (0–0)	0 (0–0)	24 (19–29)	7 (6–8)
Grenada	2024	0 (0–0)	0 (0–0)	24 (18–30)	7 (5–8)
Grenada	2025	0 (0–0)	0 (0–0)	24 (17–31)	7 (5–8)
Grenada	2026	0 (0–0)	0 (0–0)	24 (16–32)	6 (4–8)
Grenada	2027	0 (0–0)	0 (0–0)	24 (15–32)	6 (4–8)
Grenada	2028	0 (0–0)	0 (0–0)	24 (15–33)	6 (3–8)

Grenada	2029	0 (0–0)	0 (0–0)	24 (14–34)	5 (3–8)
Grenada	2030	0 (0–0)	0 (0–0)	24 (13–34)	5 (3–8)
Grenada	2031	0 (0–0)	0 (0–0)	24 (13–35)	5 (2–7)
Grenada	2032	0 (0–0)	0 (-0–0)	24 (12–35)	5 (2–7)
Grenada	2033	0 (0–0)	0 (-0–0)	24 (12–36)	4 (1–7)
Grenada	2034	0 (0–0)	0 (-0–0)	24 (11–36)	4 (1–7)
Grenada	2035	0 (0–0)	0 (-0–0)	24 (11–37)	4 (0–7)
Grenada	2036	0 (0–0)	0 (-0–0)	24 (10–37)	3 (0–7)
Guam	2022	1 (1–1)	0 (0–0)	29 (27–31)	10 (9–10)
Guam	2023	1 (1–1)	0 (0–0)	29 (26–32)	10 (9–11)
Guam	2024	1 (1–1)	0 (0–0)	30 (26–33)	10 (8–11)
Guam	2025	1 (1–1)	0 (0–0)	30 (26–34)	10 (8–11)
Guam	2026	1 (1–1)	0 (0–0)	31 (26–35)	10 (8–12)
Guam	2027	1 (1–1)	0 (0–0)	31 (26–36)	10 (7–12)
Guam	2028	1 (1–1)	0 (0–0)	32 (26–37)	10 (7–12)
Guam	2029	1 (1–1)	0 (0–0)	32 (27–38)	10 (7–12)
Guam	2030	1 (1–1)	0 (0–0)	33 (27–39)	10 (7–13)
Guam	2031	1 (1–1)	0 (0–0)	33 (27–39)	10 (7–13)
Guam	2032	1 (1–1)	0 (0–0)	34 (27–40)	10 (6–13)
Guam	2033	1 (1–1)	0 (0–0)	34 (27–41)	10 (6–13)
Guam	2034	1 (1–1)	0 (0–0)	35 (27–42)	10 (6–13)
Guam	2035	1 (1–1)	0 (0–0)	35 (28–43)	10 (6–13)
Guam	2036	1 (1–1)	0 (0–0)	36 (28–43)	10 (6–14)
Guatemala	2022	81 (71–90)	1 (1–1)	487 (443–530)	17 (13–22)
Guatemala	2023	80 (66–95)	1 (0–1)	494 (433–556)	17 (9–24)
Guatemala	2024	80 (60–99)	1 (0–1)	501 (426–577)	16 (6–26)
Guatemala	2025	79 (55–104)	1 (0–1)	509 (422–596)	15 (3–27)
Guatemala	2026	79 (49–109)	1 (-0–1)	516 (418–613)	14 (-1–29)
Guatemala	2027	79 (44–114)	1 (-0–1)	523 (416–630)	13 (-4–31)
Guatemala	2028	78 (37–119)	0 (-0–1)	530 (415–645)	12 (-8–33)
Guatemala	2029	78 (31–124)	0 (-1–1)	538 (414–661)	12 (-12–35)
Guatemala	2030	77 (25–130)	0 (-1–2)	545 (414–675)	11 (-16–37)
Guatemala	2031	77 (18–135)	0 (-1–2)	552 (414–690)	10 (-20–39)

Guatemala	2032	76 (11–141)	0 (-1–2)	559 (415–704)	9 (-24–42)
Guatemala	2033	76 (4–148)	0 (-1–2)	566 (416–717)	8 (-28–44)
Guatemala	2034	76 (-3–154)	0 (-1–2)	574 (417–731)	7 (-32–46)
Guatemala	2035	75 (-10–161)	0 (-2–2)	581 (418–744)	6 (-36–49)
Guatemala	2036	75 (-18–167)	0 (-2–2)	588 (420–757)	6 (-41–52)
Guinea	2022	16 (16–16)	0 (0–0)	254 (251–257)	7 (7–7)
Guinea	2023	15 (15–16)	0 (0–0)	254 (247–261)	7 (7–7)
Guinea	2024	15 (14–16)	0 (0–0)	254 (242–265)	7 (6–7)
Guinea	2025	15 (14–16)	0 (0–0)	254 (236–271)	7 (6–7)
Guinea	2026	15 (13–16)	0 (0–0)	253 (230–277)	6 (5–7)
Guinea	2027	14 (12–16)	0 (0–0)	253 (224–283)	6 (5–7)
Guinea	2028	14 (12–17)	0 (0–0)	253 (216–290)	6 (4–8)
Guinea	2029	14 (11–17)	0 (0–0)	253 (209–298)	6 (4–8)
Guinea	2030	13 (10–17)	0 (0–0)	253 (200–306)	6 (3–8)
Guinea	2031	13 (9–17)	0 (0–0)	253 (192–315)	5 (3–8)
Guinea	2032	13 (8–18)	0 (0–0)	253 (183–324)	5 (2–8)
Guinea	2033	13 (7–18)	0 (0–0)	253 (173–333)	5 (2–8)
Guinea	2034	12 (6–18)	0 (0–0)	253 (163–343)	5 (1–9)
Guinea	2035	12 (5–19)	0 (0–0)	253 (153–353)	5 (0–9)
Guinea	2036	12 (4–19)	0 (0–0)	253 (142–363)	4 (-0–9)
Guinea-Bissau	2022	2 (2–2)	0 (0–0)	162 (160–163)	8 (8–8)
Guinea-Bissau	2023	2 (2–2)	0 (0–0)	164 (160–168)	8 (8–8)
Guinea-Bissau	2024	2 (2–2)	0 (0–0)	167 (160–173)	8 (7–8)
Guinea-Bissau	2025	2 (2–2)	0 (0–0)	169 (158–179)	7 (7–8)
Guinea-Bissau	2026	2 (2–2)	0 (0–0)	171 (156–186)	7 (6–8)
Guinea-Bissau	2027	2 (2–3)	0 (0–0)	173 (154–193)	7 (6–8)
Guinea-Bissau	2028	2 (2–3)	0 (0–0)	175 (151–200)	7 (5–8)
Guinea-Bissau	2029	2 (2–3)	0 (0–0)	178 (147–208)	7 (5–9)
Guinea-Bissau	2030	2 (2–3)	0 (0–0)	180 (143–216)	7 (4–9)
Guinea-Bissau	2031	2 (1–3)	0 (0–0)	182 (139–225)	6 (4–9)
Guinea-Bissau	2032	2 (1–3)	0 (0–0)	184 (135–234)	6 (3–9)
Guinea-Bissau	2033	2 (1–3)	0 (0–0)	187 (130–243)	6 (3–9)
Guinea-Bissau	2034	2 (1–3)	0 (0–0)	189 (125–252)	6 (2–10)

Guinea-Bissau	2035	2 (1–3)	0 (0–0)	191 (120–262)	6 (2–10)
Guinea-Bissau	2036	2 (1–3)	0 (0–0)	193 (114–272)	6 (1–10)
Guyana	2022	2 (2–2)	0 (0–0)	63 (59–67)	9 (7–10)
Guyana	2023	2 (2–3)	0 (0–0)	64 (58–70)	8 (6–11)
Guyana	2024	2 (2–3)	0 (0–0)	65 (58–72)	8 (5–11)
Guyana	2025	2 (2–3)	0 (0–0)	66 (58–73)	8 (4–11)
Guyana	2026	2 (2–3)	0 (0–0)	66 (57–75)	7 (3–11)
Guyana	2027	2 (2–3)	0 (0–0)	67 (57–77)	7 (3–11)
Guyana	2028	2 (2–3)	0 (0–0)	68 (57–78)	7 (2–12)
Guyana	2029	2 (2–3)	0 (0–0)	69 (57–80)	6 (1–12)
Guyana	2030	2 (2–3)	0 (0–0)	69 (57–81)	6 (1–12)
Guyana	2031	2 (2–3)	0 (-0–0)	70 (58–83)	6 (0–12)
Guyana	2032	2 (2–3)	0 (-0–0)	71 (58–84)	6 (-0–12)
Guyana	2033	2 (2–3)	0 (-0–0)	72 (58–85)	5 (-1–11)
Guyana	2034	2 (2–3)	0 (-0–0)	72 (58–87)	5 (-1–11)
Guyana	2035	2 (2–3)	0 (-0–0)	73 (58–88)	5 (-2–11)
Guyana	2036	2 (2–3)	0 (-0–0)	74 (58–89)	4 (-3–11)
Haiti	2022	35 (34–35)	0 (0–0)	701 (688–714)	12 (12–13)
Haiti	2023	35 (34–36)	0 (0–1)	712 (692–732)	12 (11–13)
Haiti	2024	35 (33–37)	0 (0–1)	725 (692–758)	12 (11–13)
Haiti	2025	35 (33–37)	0 (0–1)	736 (691–781)	12 (10–13)
Haiti	2026	35 (32–37)	0 (0–1)	749 (689–809)	12 (9–14)
Haiti	2027	35 (32–38)	0 (0–1)	760 (685–836)	11 (9–14)
Haiti	2028	35 (31–38)	0 (0–1)	773 (680–865)	11 (8–15)
Haiti	2029	35 (31–38)	0 (0–1)	784 (674–895)	11 (7–15)
Haiti	2030	34 (30–38)	0 (0–1)	796 (667–926)	11 (6–16)
Haiti	2031	34 (30–38)	0 (0–1)	808 (659–958)	11 (5–16)
Haiti	2032	34 (29–38)	0 (0–1)	820 (650–991)	10 (4–17)
Haiti	2033	34 (29–38)	0 (0–1)	832 (640–1025)	10 (3–17)
Haiti	2034	33 (28–38)	0 (0–1)	844 (629–1060)	10 (2–18)
Haiti	2035	33 (28–38)	0 (0–1)	856 (617–1095)	10 (1–19)
Haiti	2036	33 (28–38)	0 (0–1)	868 (605–1131)	10 (0–19)
High SDI	2022	6447 (16206–16689	1 (1–1)	763234 (751709–774759	16 (15–16)

High SDI	2023	6640 (16099–17180	1 (1–1)	763047 (744053–782040	15 (14–16)
High SDI	2024	6832 (15927–17737	1 (1–1)	762859 (736226–789492	15 (14–17)
High SDI	2025	7024 (15700–18349	1 (1–1)	762672 (727973–797370	15 (13–17)
High SDI	2026	7217 (15423–19010	1 (1–1)	762484 (719237–805731	15 (12–18)
High SDI	2027	7409 (15102–19716	1 (1–1)	762297 (710011–814583	15 (11–19)
High SDI	2028	7601 (14740–20462	1 (0–1)	762109 (700303–823916	14 (9–19)
High SDI	2029	7793 (14340–21247	1 (0–1)	761922 (690127–833716	14 (8–20)
High SDI	2030	7986 (13903–22068	1 (0–1)	761734 (679499–843969	14 (7–21)
High SDI	2031	8178 (13433–22923	1 (0–1)	761547 (668434–854659	14 (6–22)
High SDI	2032	8370 (12931–23810	1 (0–1)	761359 (656946–865773	14 (4–23)
High SDI	2033	8563 (12398–24727	1 (0–1)	761172 (645048–877295	13 (3–24)
High SDI	2034	8755 (11835–25675	1 (0–1)	760984 (632754–889214	13 (1–25)
High SDI	2035	8947 (11243–26651	1 (0–1)	760797 (620075–901518	13 (-0–26)
High SDI	2036	9139 (10624–27654	1 (0–1)	760609 (607022–914196	13 (-2–27)
High-middle SDI	2022	9095 (38307–39882	2 (2–2)	84131 (1753763–181449	45 (43–46)
High-middle SDI	2023	9095 (37505–40684	2 (2–2)	00369 (1734344–186639	43 (40–46)
High-middle SDI	2024	9095 (36989–41200	2 (2–2)	16607 (1728310–190490	41 (37–45)
High-middle SDI	2025	9095 (36577–41613	2 (1–2)	32845 (1726858–193883	39 (34–44)
High-middle SDI	2026	9095 (36223–41967	2 (1–2)	49083 (1727962–197020	37 (32–43)
High-middle SDI	2027	9095 (35908–42281	2 (1–2)	65321 (1730757–199988	35 (29–42)
High-middle SDI	2028	9095 (35622–42568	2 (1–2)	81559 (1734779–202834	34 (27–40)
High-middle SDI	2029	9095 (35357–42832	1 (1–2)	97797 (1739741–205585	32 (24–39)
High-middle SDI	2030	9095 (35110–43079	1 (1–2)	14035 (1745457–208261	30 (22–38)
High-middle SDI	2031	9095 (34878–43312	1 (1–2)	30273 (1751791–210875	28 (19–36)
High-middle SDI	2032	9095 (34657–43532	1 (1–2)	46511 (1758647–213437	26 (17–35)
High-middle SDI	2033	9095 (34448–43742	1 (1–2)	62749 (1765950–215954	24 (15–33)
High-middle SDI	2034	9095 (34247–43943	1 (1–2)	78987 (1773641–218433	22 (13–32)
High-middle SDI	2035	9095 (34054–44136	1 (1–1)	95225 (1781674–220877	20 (10–30)
High-middle SDI	2036	9095 (33868–44321	1 (1–1)	11463 (1790011–223291	18 (8–29)
Honduras	2022	61 (59–64)	1 (1–1)	493 (476–510)	23 (22–24)
Honduras	2023	62 (58–66)	1 (1–1)	500 (467–532)	24 (22–25)
Honduras	2024	63 (58–69)	1 (1–1)	506 (456–557)	24 (22–26)
Honduras	2025	64 (57–71)	1 (1–1)	513 (442–583)	24 (22–27)

Honduras	2026	65 (57–74)	1 (1–1)	520 (427–612)	25 (22–27)
Honduras	2027	67 (57–76)	1 (1–1)	526 (409–643)	25 (22–27)
Honduras	2028	68 (57–78)	1 (1–1)	533 (390–676)	25 (23–27)
Honduras	2029	69 (58–81)	1 (1–1)	540 (369–710)	25 (23–27)
Honduras	2030	70 (58–83)	1 (1–1)	546 (347–746)	25 (23–27)
Honduras	2031	72 (58–85)	1 (1–1)	553 (323–783)	25 (22–27)
Honduras	2032	73 (59–87)	1 (1–1)	560 (297–822)	25 (22–27)
Honduras	2033	74 (59–89)	1 (1–1)	566 (271–862)	25 (22–27)
Honduras	2034	75 (60–91)	1 (1–1)	573 (243–903)	25 (22–27)
Honduras	2035	76 (60–93)	1 (1–1)	580 (213–946)	25 (22–27)
Honduras	2036	78 (61–94)	1 (1–1)	586 (183–990)	25 (22–27)
Hungary	2022	138 (125–151)	1 (1–1)	6199 (5619–6779)	18 (16–20)
Hungary	2023	129 (111–148)	1 (1–1)	5990 (5052–6928)	16 (13–19)
Hungary	2024	121 (98–144)	1 (0–1)	5781 (4485–7077)	15 (11–18)
Hungary	2025	112 (86–139)	1 (0–1)	5572 (3904–7241)	13 (9–17)
Hungary	2026	104 (74–133)	0 (0–1)	5363 (3303–7423)	11 (6–16)
Hungary	2027	95 (63–127)	0 (0–1)	5154 (2683–7625)	10 (4–15)
Hungary	2028	87 (52–122)	0 (0–1)	4945 (2043–7847)	8 (2–14)
Hungary	2029	78 (41–115)	0 (0–0)	4736 (1384–8089)	7 (0–13)
Hungary	2030	70 (30–109)	0 (-0–0)	4527 (705–8350)	5 (-2–12)
Hungary	2031	61 (19–103)	0 (-0–0)	4318 (8–8629)	3 (-4–10)
Hungary	2032	52 (9–96)	0 (-0–0)	4109 (-707–8926)	2 (-6–9)
Hungary	2033	44 (-2–90)	-0 (-0–0)	3901 (-1440–9241)	0 (-8–8)
Hungary	2034	35 (-12–83)	-0 (-0–0)	3692 (-2190–9573)	-2 (-10–6)
Hungary	2035	27 (-22–76)	-0 (-0–0)	3483 (-2956–9921)	-3 (-11–5)
Hungary	2036	18 (-33–69)	-0 (-1–0)	3274 (-3739–10286)	-5 (-13–4)
Iceland	2022	3 (3–3)	0 (0–1)	206 (196–215)	10 (8–11)
Iceland	2023	3 (2–3)	0 (0–1)	205 (189–222)	9 (7–12)
Iceland	2024	3 (2–3)	0 (0–1)	205 (183–227)	9 (5–12)
Iceland	2025	3 (2–3)	0 (0–0)	205 (178–232)	8 (3–13)
Iceland	2026	2 (2–3)	0 (0–0)	205 (174–236)	7 (2–13)
Iceland	2027	2 (2–3)	0 (0–1)	205 (170–240)	7 (-0–13)
Iceland	2028	2 (1–3)	0 (0–1)	205 (167–243)	6 (-2–14)

Iceland	2029	2 (1–3)	0 (-0–1)	205 (163–246)	5 (-4–15)
Iceland	2030	2 (1–3)	0 (-0–1)	205 (161–249)	5 (-6–15)
Iceland	2031	2 (1–2)	0 (-0–1)	205 (158–252)	4 (-8–16)
Iceland	2032	2 (1–2)	0 (-0–1)	205 (155–254)	4 (-10–17)
Iceland	2033	1 (1–2)	0 (-0–1)	205 (153–257)	3 (-12–17)
Iceland	2034	1 (0–2)	0 (-0–1)	205 (150–259)	2 (-14–18)
Iceland	2035	1 (0–2)	0 (-1–1)	205 (148–261)	2 (-16–19)
Iceland	2036	1 (0–2)	0 (-1–1)	205 (146–263)	1 (-18–20)
India	2022	4172 (4030–4314)	0 (0–0)	60241 (353454–367027	8 (8–9)
India	2023	4204 (3968–4441)	0 (0–0)	65103 (353742–376464	8 (7–9)
India	2024	4237 (3934–4540)	0 (0–0)	70047 (354972–385122	8 (7–9)
India	2025	4269 (3912–4626)	0 (0–0)	75018 (356827–393209	7 (6–9)
India	2026	4301 (3897–4705)	0 (0–0)	79998 (359106–400891	7 (6–9)
India	2027	4333 (3887–4779)	0 (0–0)	84982 (361686–408278	7 (5–8)
India	2028	4366 (3881–4850)	0 (0–0)	89967 (364488–415446	7 (5–8)
India	2029	4398 (3878–4918)	0 (0–0)	94952 (367462–422443	6 (5–8)
India	2030	4430 (3877–4983)	0 (0–0)	99938 (370573–429302	6 (4–8)
India	2031	4462 (3877–5047)	0 (0–0)	04923 (373796–436050	6 (4–8)
India	2032	4495 (3880–5109)	0 (0–0)	09908 (377114–442702	5 (3–7)
India	2033	4527 (3884–5170)	0 (0–0)	14894 (380513–449274	5 (3–7)
India	2034	4559 (3889–5229)	0 (0–0)	19879 (383982–455776	5 (3–7)
India	2035	4591 (3895–5288)	0 (0–0)	24865 (387512–462217	4 (2–7)
India	2036	4623 (3902–5345)	0 (0–0)	29850 (391097–468603	4 (2–7)
Indonesia	2022	1527 (1517–1537)	1 (1–1)	28015 (27700–28330)	16 (16–16)
Indonesia	2023	1551 (1528–1574)	1 (1–1)	28871 (28311–29430)	16 (15–16)
Indonesia	2024	1574 (1538–1611)	1 (1–1)	29726 (28900–30553)	15 (15–16)
Indonesia	2025	1598 (1548–1647)	1 (1–1)	30582 (29462–31701)	15 (14–16)
Indonesia	2026	1621 (1561–1681)	1 (1–1)	31437 (30000–32875)	15 (13–17)
Indonesia	2027	1645 (1576–1715)	1 (1–1)	32293 (30513–34073)	15 (13–17)
Indonesia	2028	1669 (1592–1747)	1 (1–1)	33149 (31005–35293)	15 (12–17)
Indonesia	2029	1694 (1609–1778)	1 (0–1)	34004 (31475–36534)	14 (12–17)
Indonesia	2030	1718 (1627–1809)	1 (0–1)	34860 (31925–37795)	14 (11–18)
Indonesia	2031	1743 (1646–1840)	1 (0–1)	35716 (32356–39075)	14 (10–18)

Indonesia	2032	1767 (1665–1869)	1 (0–1)	36571 (32768–40374)	14 (9–19)
Indonesia	2033	1791 (1684–1899)	1 (0–1)	37427 (33162–41691)	14 (8–19)
Indonesia	2034	1816 (1703–1929)	1 (0–1)	38282 (33540–43025)	14 (8–20)
Indonesia	2035	1840 (1723–1958)	1 (0–1)	39138 (33901–44376)	13 (7–20)
Indonesia	2036	1865 (1743–1987)	1 (0–1)	39994 (34245–45742)	13 (6–21)
Iran (Islamic Republic of)	2022	718 (695–741)	1 (1–1)	17950 (17328–18573)	20 (19–21)
Iran (Islamic Republic of)	2023	727 (689–764)	1 (1–1)	18245 (17365–19126)	20 (18–21)
Iran (Islamic Republic of)	2024	735 (686–785)	1 (1–1)	18540 (17461–19619)	19 (16–21)
Iran (Islamic Republic of)	2025	744 (685–803)	1 (1–1)	18835 (17590–20080)	18 (14–21)
Iran (Islamic Republic of)	2026	753 (685–820)	1 (1–1)	19130 (17737–20522)	17 (12–21)
Iran (Islamic Republic of)	2027	762 (687–837)	1 (0–1)	19425 (17899–20950)	16 (10–21)
Iran (Islamic Republic of)	2028	770 (689–852)	1 (0–1)	19720 (18072–21367)	15 (8–22)
Iran (Islamic Republic of)	2029	779 (691–867)	1 (0–1)	20015 (18253–21776)	14 (5–22)
Iran (Islamic Republic of)	2030	788 (694–882)	1 (0–1)	20310 (18441–22178)	13 (3–23)
Iran (Islamic Republic of)	2031	797 (697–897)	1 (0–1)	20604 (18635–22574)	12 (0–23)
Iran (Islamic Republic of)	2032	806 (701–911)	0 (-0–1)	20899 (18834–22965)	11 (-2–24)
Iran (Islamic Republic of)	2033	814 (704–924)	0 (-0–1)	21194 (19037–23351)	10 (-5–25)
Iran (Islamic Republic of)	2034	823 (708–938)	0 (-0–1)	21489 (19244–23734)	9 (-8–25)
Iran (Islamic Republic of)	2035	832 (713–951)	0 (-0–1)	21784 (19454–24114)	8 (-11–26)
Iran (Islamic Republic of)	2036	841 (717–965)	0 (-1–1)	22079 (19667–24491)	7 (-13–27)
Iraq	2022	149 (144–154)	1 (1–1)	2910 (2840–2980)	15 (14–15)
Iraq	2023	154 (146–163)	1 (1–1)	3036 (2917–3156)	14 (13–15)
Iraq	2024	160 (148–171)	1 (1–1)	3200 (3008–3392)	14 (13–16)
Iraq	2025	165 (150–180)	1 (1–1)	3341 (3072–3610)	14 (12–16)
Iraq	2026	170 (151–189)	1 (1–1)	3496 (3138–3853)	13 (11–16)
Iraq	2027	176 (153–199)	1 (1–1)	3642 (3190–4093)	13 (11–16)
Iraq	2028	181 (154–208)	1 (0–1)	3793 (3239–4347)	13 (10–16)
Iraq	2029	186 (154–218)	1 (0–1)	3941 (3279–4604)	13 (9–16)
Iraq	2030	191 (155–228)	1 (0–1)	4092 (3314–4870)	12 (9–16)
Iraq	2031	197 (156–238)	1 (0–1)	4241 (3342–5140)	12 (8–16)
Iraq	2032	202 (156–248)	1 (0–1)	4390 (3364–5416)	12 (8–15)
Iraq	2033	207 (156–258)	1 (0–1)	4540 (3381–5698)	11 (7–15)
Iraq	2034	212 (156–269)	1 (0–1)	4689 (3393–5985)	11 (7–15)

Iraq	2035	218 (156–280)	1 (0–1)	4839 (3400–6277)	11 (6–15)
Iraq	2036	223 (155–291)	1 (0–1)	4988 (3402–6574)	10 (6–15)
Ireland	2022	35 (32–38)	0 (0–0)	3057 (2914–3199)	8 (7–9)
Ireland	2023	33 (29–37)	0 (0–0)	3037 (2836–3238)	7 (5–9)
Ireland	2024	31 (26–36)	0 (0–0)	3017 (2771–3263)	7 (4–9)
Ireland	2025	29 (23–34)	0 (0–0)	2997 (2713–3282)	6 (2–9)
Ireland	2026	26 (20–33)	0 (0–0)	2977 (2659–3296)	5 (1–9)
Ireland	2027	24 (17–31)	0 (-0–0)	2958 (2609–3306)	4 (-1–9)
Ireland	2028	22 (15–30)	0 (-0–0)	2938 (2561–3314)	3 (-3–10)
Ireland	2029	20 (12–28)	0 (-0–0)	2918 (2516–3320)	3 (-5–10)
Ireland	2030	18 (9–27)	0 (-0–0)	2898 (2471–3325)	2 (-6–10)
Ireland	2031	16 (7–25)	0 (-0–0)	2878 (2428–3328)	1 (-8–10)
Ireland	2032	14 (4–23)	0 (-0–0)	2859 (2387–3330)	0 (-10–10)
Ireland	2033	12 (2–22)	-0 (-1–0)	2839 (2346–3332)	-1 (-12–11)
Ireland	2034	10 (-1–20)	-0 (-1–0)	2819 (2306–3332)	-1 (-14–11)
Ireland	2035	7 (-3–18)	-0 (-1–0)	2799 (2267–3332)	-2 (-16–11)
Ireland	2036	5 (-6–17)	-0 (-1–0)	2779 (2228–3330)	-3 (-18–12)
Israel	2022	59 (55–63)	0 (0–0)	1239 (1172–1306)	10 (8–11)
Israel	2023	59 (54–64)	0 (0–0)	1239 (1122–1355)	9 (7–10)
Israel	2024	59 (52–65)	0 (0–0)	1239 (1088–1389)	8 (6–10)
Israel	2025	59 (51–66)	0 (0–0)	1239 (1060–1417)	8 (5–10)
Israel	2026	59 (51–67)	0 (0–0)	1239 (1037–1441)	7 (5–9)
Israel	2027	59 (50–68)	0 (0–0)	1239 (1015–1462)	6 (4–9)
Israel	2028	59 (49–69)	0 (0–0)	1239 (996–1482)	6 (3–9)
Israel	2029	59 (48–70)	0 (0–0)	1239 (978–1500)	5 (2–8)
Israel	2030	59 (48–70)	0 (-0–0)	1239 (961–1517)	4 (1–8)
Israel	2031	59 (47–71)	0 (-0–0)	1239 (945–1533)	4 (0–7)
Israel	2032	59 (46–71)	0 (-0–0)	1239 (930–1548)	3 (-0–7)
Israel	2033	59 (46–72)	0 (-0–0)	1239 (915–1562)	3 (-1–6)
Israel	2034	59 (45–72)	0 (-0–0)	1239 (902–1576)	2 (-2–6)
Israel	2035	59 (45–73)	0 (-0–0)	1239 (888–1589)	1 (-3–5)
Israel	2036	59 (44–73)	0 (-0–0)	1239 (876–1602)	1 (-4–5)
Italy	2022	1037 (1003–1072)	1 (1–1)	16467 (15734–17201)	14 (13–15)

Italy	2023	1020 (975–1065)	1 (1–1)	15974 (14937–17012)	14 (12–16)
Italy	2024	1002 (940–1064)	1 (1–1)	15481 (14210–16752)	13 (11–16)
Italy	2025	985 (901–1068)	1 (0–1)	14988 (13521–16455)	13 (9–16)
Italy	2026	967 (858–1076)	1 (0–1)	14495 (12854–16135)	12 (8–17)
Italy	2027	950 (812–1087)	1 (0–1)	14002 (12205–15799)	12 (6–17)
Italy	2028	932 (764–1100)	1 (0–1)	13509 (11568–15450)	11 (5–18)
Italy	2029	915 (713–1116)	1 (0–1)	13016 (10941–15091)	11 (3–19)
Italy	2030	897 (660–1134)	0 (0–1)	12523 (10322–14723)	10 (1–19)
Italy	2031	880 (605–1154)	0 (0–1)	12029 (9710–14349)	10 (-1–20)
Italy	2032	862 (548–1176)	0 (-0–1)	11536 (9103–13969)	9 (-2–21)
Italy	2033	844 (490–1199)	0 (-0–1)	11043 (8502–13585)	9 (-4–22)
Italy	2034	827 (430–1224)	0 (-0–1)	10550 (7905–13195)	8 (-6–23)
Italy	2035	809 (368–1251)	0 (-0–1)	10057 (7312–12802)	8 (-8–24)
Italy	2036	792 (304–1280)	0 (-0–1)	9564 (6723–12405)	7 (-10–25)
Jamaica	2022	17 (13–21)	1 (0–1)	506 (387–625)	12 (8–17)
Jamaica	2023	17 (13–21)	1 (0–1)	513 (384–641)	12 (7–17)
Jamaica	2024	17 (11–22)	0 (0–1)	509 (352–666)	11 (6–17)
Jamaica	2025	17 (11–22)	0 (0–1)	511 (341–681)	11 (5–17)
Jamaica	2026	17 (10–23)	0 (0–1)	510 (322–697)	11 (4–18)
Jamaica	2027	17 (10–23)	0 (0–1)	510 (310–711)	10 (3–18)
Jamaica	2028	17 (10–24)	0 (0–1)	510 (295–724)	10 (2–18)
Jamaica	2029	17 (9–24)	0 (0–1)	510 (284–737)	9 (1–18)
Jamaica	2030	17 (9–25)	0 (0–1)	510 (271–749)	9 (-0–18)
Jamaica	2031	17 (8–25)	0 (-0–1)	510 (260–760)	8 (-1–18)
Jamaica	2032	17 (8–25)	0 (-0–1)	510 (249–771)	8 (-2–18)
Jamaica	2033	17 (8–26)	0 (-0–1)	510 (239–781)	7 (-3–18)
Jamaica	2034	17 (7–26)	0 (-0–1)	510 (229–791)	7 (-4–17)
Jamaica	2035	17 (7–26)	0 (-0–1)	510 (220–801)	7 (-4–17)
Jamaica	2036	17 (7–27)	0 (-0–1)	510 (210–810)	6 (-5–17)
Japan	2022	5435 (5295–5575)	1 (1–1)	08686 (105502–111870	26 (25–28)
Japan	2023	5325 (5075–5576)	1 (1–1)	08601 (102800–114403	26 (23–29)
Japan	2024	5215 (4890–5541)	1 (1–2)	108516 (99792–117240)	25 (21–30)
Japan	2025	5106 (4720–5492)	1 (1–2)	108431 (96471–120391)	25 (19–31)

Japan	2026	4996 (4558–5434)	1 (1–2)	108347 (92855–123839)	24 (17–32)
Japan	2027	4886 (4401–5371)	1 (1–2)	108262 (88960–127563)	24 (14–33)
Japan	2028	4776 (4249–5304)	1 (1–2)	108177 (84806–131548)	23 (12–35)
Japan	2029	4667 (4100–5233)	1 (0–2)	108092 (80405–135779)	23 (9–37)
Japan	2030	4557 (3953–5160)	1 (0–2)	108007 (75772–140242)	22 (6–39)
Japan	2031	4447 (3809–5085)	1 (0–3)	107922 (70916–144928)	22 (3–40)
Japan	2032	4337 (3666–5008)	1 (-0–3)	107837 (65848–149827)	21 (-0–43)
Japan	2033	4227 (3525–4930)	1 (-0–3)	107752 (60576–154929)	21 (-3–45)
Japan	2034	4118 (3385–4850)	1 (-1–3)	107668 (55107–160228)	20 (-7–47)
Japan	2035	4008 (3246–4769)	1 (-1–3)	107583 (49449–165716)	20 (-10–49)
Japan	2036	3898 (3109–4687)	1 (-1–4)	107498 (43607–171388)	19 (-14–52)
Jordan	2022	39 (38–40)	1 (1–1)	767 (759–776)	12 (12–12)
Jordan	2023	41 (40–42)	1 (0–1)	819 (796–841)	12 (11–13)
Jordan	2024	43 (41–45)	1 (0–1)	870 (831–910)	11 (10–13)
Jordan	2025	45 (42–48)	0 (0–1)	922 (863–981)	11 (9–12)
Jordan	2026	47 (43–51)	0 (0–1)	973 (892–1055)	10 (8–12)
Jordan	2027	49 (44–54)	0 (0–1)	1025 (919–1131)	10 (7–12)
Jordan	2028	51 (44–57)	0 (0–1)	1076 (944–1209)	9 (6–12)
Jordan	2029	53 (45–60)	0 (0–1)	1128 (967–1289)	9 (6–11)
Jordan	2030	54 (45–63)	0 (0–0)	1179 (988–1370)	8 (5–11)
Jordan	2031	56 (46–67)	0 (0–0)	1231 (1008–1454)	8 (4–11)
Jordan	2032	58 (46–70)	0 (0–0)	1282 (1026–1538)	7 (4–10)
Jordan	2033	60 (47–74)	0 (0–0)	1334 (1043–1625)	7 (3–10)
Jordan	2034	62 (47–77)	0 (0–0)	1385 (1058–1713)	6 (2–10)
Jordan	2035	64 (47–81)	0 (0–0)	1437 (1071–1802)	5 (2–9)
Jordan	2036	66 (47–85)	0 (0–0)	1488 (1084–1893)	5 (1–9)
Kazakhstan	2022	156 (140–171)	1 (1–1)	5350 (4498–6202)	21 (18–25)
Kazakhstan	2023	152 (126–178)	1 (1–1)	5134 (3831–6438)	19 (14–24)
Kazakhstan	2024	148 (112–184)	1 (0–1)	4919 (3200–6637)	17 (11–23)
Kazakhstan	2025	144 (97–191)	1 (0–1)	4703 (2575–6831)	15 (8–22)
Kazakhstan	2026	140 (82–199)	0 (0–1)	4488 (1947–7029)	13 (5–21)
Kazakhstan	2027	136 (66–207)	0 (0–1)	4272 (1310–7235)	11 (2–20)
Kazakhstan	2028	133 (49–216)	0 (0–1)	4057 (662–7451)	9 (-1–18)

Kazakhstan	2029	129 (32–226)	0 (-0–1)	3841 (3–7679)	7 (-4–17)
Kazakhstan	2030	125 (14–236)	0 (-0–1)	3626 (-668–7919)	4 (-6–15)
Kazakhstan	2031	121 (-4–246)	0 (-0–0)	3410 (-1352–8172)	2 (-9–13)
Kazakhstan	2032	117 (-23–258)	0 (-0–0)	3195 (-2048–8438)	0 (-12–12)
Kazakhstan	2033	113 (-43–269)	-0 (-0–0)	2979 (-2757–8716)	-2 (-14–10)
Kazakhstan	2034	109 (-63–282)	-0 (-1–0)	2764 (-3479–9006)	-4 (-17–9)
Kazakhstan	2035	106 (-83–294)	-0 (-1–0)	2548 (-4213–9309)	-6 (-20–7)
Kazakhstan	2036	102 (-104–308)	-0 (-1–0)	2333 (-4959–9624)	-8 (-22–5)
Kenya	2022	61 (60–62)	0 (0–0)	8408 (8317–8499)	7 (6–7)
Kenya	2023	61 (59–63)	0 (0–0)	8578 (8398–8758)	6 (6–7)
Kenya	2024	61 (57–65)	0 (0–0)	8750 (8480–9020)	6 (6–7)
Kenya	2025	61 (56–67)	0 (0–0)	8922 (8565–9279)	6 (5–7)
Kenya	2026	62 (54–69)	0 (0–0)	9095 (8655–9534)	6 (5–7)
Kenya	2027	62 (53–71)	0 (0–0)	9267 (8750–9785)	6 (4–7)
Kenya	2028	62 (51–74)	0 (0–0)	9441 (8850–10032)	6 (4–7)
Kenya	2029	63 (48–77)	0 (0–0)	9614 (8954–10274)	6 (3–8)
Kenya	2030	63 (46–79)	0 (0–0)	9788 (9063–10512)	5 (3–8)
Kenya	2031	63 (44–82)	0 (0–0)	9961 (9176–10746)	5 (2–8)
Kenya	2032	63 (41–86)	0 (0–0)	10135 (9292–10977)	5 (2–8)
Kenya	2033	64 (38–89)	0 (0–0)	10309 (9411–11206)	5 (1–9)
Kenya	2034	64 (36–92)	0 (0–0)	10482 (9533–11431)	5 (0–9)
Kenya	2035	64 (33–96)	0 (0–0)	10656 (9657–11655)	5 (-0–9)
Kenya	2036	64 (30–99)	0 (-0–0)	10830 (9784–11876)	4 (-1–10)
Kiribati	2022	2 (2–2)	2 (2–3)	48 (47–48)	69 (68–70)
Kiribati	2023	2 (2–2)	2 (2–3)	48 (48–49)	68 (66–70)
Kiribati	2024	2 (2–2)	2 (2–3)	49 (48–50)	68 (64–71)
Kiribati	2025	2 (2–2)	2 (2–3)	50 (49–51)	67 (62–71)
Kiribati	2026	2 (2–2)	2 (2–3)	51 (49–52)	66 (60–72)
Kiribati	2027	2 (2–2)	2 (2–3)	51 (50–53)	65 (57–73)
Kiribati	2028	2 (2–2)	2 (2–3)	52 (50–54)	64 (54–74)
Kiribati	2029	2 (2–2)	2 (2–3)	53 (51–55)	64 (52–76)
Kiribati	2030	2 (2–2)	2 (2–3)	53 (51–55)	63 (49–77)
Kiribati	2031	2 (2–2)	2 (2–3)	54 (52–56)	62 (46–79)

Kiribati	2032	2 (2–2)	2 (2–3)	55 (52–57)	61 (42–80)
Kiribati	2033	2 (2–2)	2 (1–3)	55 (53–58)	60 (39–82)
Kiribati	2034	2 (2–2)	2 (1–3)	56 (54–59)	60 (36–84)
Kiribati	2035	2 (2–2)	2 (1–3)	57 (54–60)	59 (32–86)
Kiribati	2036	2 (2–2)	2 (1–3)	58 (55–60)	58 (28–88)
Kuwait	2022	7 (6–8)	0 (0–0)	212 (174–251)	6 (4–8)
Kuwait	2023	7 (6–9)	0 (0–0)	212 (159–266)	6 (3–9)
Kuwait	2024	8 (7–9)	0 (0–0)	212 (146–279)	6 (3–10)
Kuwait	2025	8 (7–9)	0 (0–0)	212 (136–289)	6 (2–10)
Kuwait	2026	8 (7–9)	0 (0–1)	212 (127–298)	6 (2–11)
Kuwait	2027	8 (7–10)	0 (0–1)	212 (119–306)	6 (1–11)
Kuwait	2028	8 (7–10)	0 (0–1)	212 (112–313)	6 (1–11)
Kuwait	2029	8 (7–10)	0 (0–1)	212 (105–320)	6 (0–12)
Kuwait	2030	9 (7–10)	0 (0–1)	212 (98–327)	6 (-0–12)
Kuwait	2031	9 (7–10)	0 (0–1)	212 (92–333)	6 (-0–13)
Kuwait	2032	9 (7–11)	0 (-0–1)	212 (86–339)	6 (-1–13)
Kuwait	2033	9 (7–11)	0 (-0–1)	212 (80–345)	6 (-1–13)
Kuwait	2034	9 (7–11)	0 (-0–1)	212 (75–350)	6 (-1–13)
Kuwait	2035	9 (7–11)	0 (-0–1)	212 (70–355)	6 (-2–14)
Kuwait	2036	10 (8–12)	0 (-0–1)	212 (65–360)	6 (-2–14)
Kyrgyzstan	2022	103 (96–109)	2 (2–2)	1534 (1368–1700)	54 (49–60)
Kyrgyzstan	2023	103 (91–115)	2 (2–2)	1547 (1332–1761)	53 (43–62)
Kyrgyzstan	2024	103 (86–120)	2 (1–2)	1558 (1317–1799)	51 (38–64)
Kyrgyzstan	2025	102 (83–122)	2 (1–3)	1569 (1289–1849)	49 (34–64)
Kyrgyzstan	2026	102 (81–123)	2 (1–3)	1581 (1251–1910)	47 (30–65)
Kyrgyzstan	2027	102 (80–123)	2 (1–3)	1592 (1204–1980)	45 (26–65)
Kyrgyzstan	2028	102 (80–123)	2 (1–3)	1604 (1149–2059)	44 (22–65)
Kyrgyzstan	2029	101 (79–123)	2 (1–3)	1615 (1087–2143)	42 (19–65)
Kyrgyzstan	2030	101 (79–123)	2 (1–3)	1626 (1018–2234)	40 (15–64)
Kyrgyzstan	2031	101 (79–123)	2 (0–3)	1638 (945–2330)	38 (12–64)
Kyrgyzstan	2032	101 (79–123)	2 (0–3)	1649 (867–2432)	36 (9–64)
Kyrgyzstan	2033	101 (79–123)	1 (0–3)	1660 (784–2537)	34 (6–63)
Kyrgyzstan	2034	101 (79–123)	1 (0–3)	1672 (696–2647)	33 (3–63)

Kyrgyzstan	2035	101 (79–123)	1 (0–3)	1683 (605–2761)	31 (-0–62)
Kyrgyzstan	2036	101 (79–123)	1 (-0–3)	1695 (510–2879)	29 (-3–61)
Lao People's Democratic Republic	2022	41 (40–41)	1 (1–1)	1134 (1122–1147)	22 (21–22)
Lao People's Democratic Republic	2023	41 (40–42)	1 (1–1)	1154 (1131–1178)	21 (21–22)
Lao People's Democratic Republic	2024	42 (40–43)	1 (1–1)	1174 (1137–1212)	21 (20–22)
Lao People's Democratic Republic	2025	42 (40–44)	1 (1–1)	1194 (1141–1248)	20 (19–22)
Lao People's Democratic Republic	2026	43 (40–45)	1 (1–1)	1214 (1143–1286)	20 (18–22)
Lao People's Democratic Republic	2027	43 (40–46)	1 (1–1)	1234 (1143–1325)	19 (17–22)
Lao People's Democratic Republic	2028	44 (40–47)	1 (1–1)	1254 (1142–1366)	19 (16–22)
Lao People's Democratic Republic	2029	44 (39–49)	1 (1–1)	1274 (1139–1409)	19 (14–23)
Lao People's Democratic Republic	2030	44 (39–50)	1 (1–1)	1294 (1136–1452)	18 (13–23)
Lao People's Democratic Republic	2031	45 (39–51)	1 (1–1)	1314 (1131–1497)	18 (12–23)
Lao People's Democratic Republic	2032	45 (38–52)	1 (1–1)	1334 (1124–1544)	17 (11–24)
Lao People's Democratic Republic	2033	46 (38–54)	1 (0–1)	1354 (1117–1591)	17 (9–24)
Lao People's Democratic Republic	2034	46 (37–55)	1 (0–1)	1374 (1109–1639)	16 (8–24)
Lao People's Democratic Republic	2035	47 (37–57)	1 (0–1)	1394 (1099–1689)	16 (7–25)
Lao People's Democratic Republic	2036	47 (36–58)	1 (0–1)	1414 (1089–1739)	15 (5–25)
Latvia	2022	33 (29–38)	1 (1–1)	1056 (926–1186)	26 (22–30)
Latvia	2023	31 (21–42)	1 (1–1)	1056 (872–1240)	24 (17–31)
Latvia	2024	30 (16–43)	1 (1–1)	1056 (831–1282)	23 (15–31)
Latvia	2025	28 (11–44)	1 (1–1)	1056 (796–1317)	21 (13–30)
Latvia	2026	26 (7–45)	1 (0–1)	1056 (765–1347)	20 (10–29)
Latvia	2027	24 (3–45)	1 (0–1)	1056 (737–1375)	18 (8–28)
Latvia	2028	22 (-0–45)	1 (0–1)	1056 (712–1401)	17 (6–28)
Latvia	2029	21 (-4–45)	1 (0–1)	1056 (688–1424)	15 (4–27)
Latvia	2030	19 (-7–45)	1 (0–1)	1056 (665–1447)	14 (2–26)
Latvia	2031	17 (-11–45)	0 (0–1)	1056 (644–1468)	12 (-0–25)
Latvia	2032	15 (-14–44)	0 (-0–1)	1056 (624–1488)	11 (-2–24)
Latvia	2033	13 (-17–44)	0 (-0–1)	1056 (605–1507)	9 (-4–23)
Latvia	2034	12 (-20–44)	0 (-0–1)	1056 (587–1526)	8 (-6–22)
Latvia	2035	10 (-23–43)	0 (-0–1)	1056 (569–1543)	6 (-8–21)
Latvia	2036	8 (-26–42)	0 (-0–1)	1056 (552–1560)	5 (-10–20)
Lebanon	2022	54 (53–56)	1 (1–1)	648 (633–662)	18 (18–19)

Lebanon	2023	54 (52–57)	1 (1–1)	657 (628–686)	18 (16–20)
Lebanon	2024	54 (50–59)	1 (1–1)	666 (624–708)	17 (15–20)
Lebanon	2025	54 (48–60)	1 (1–1)	675 (621–730)	17 (14–20)
Lebanon	2026	54 (47–62)	1 (1–1)	684 (618–751)	16 (13–19)
Lebanon	2027	54 (45–63)	1 (1–1)	693 (616–771)	16 (13–19)
Lebanon	2028	54 (43–65)	1 (1–1)	702 (615–790)	15 (12–19)
Lebanon	2029	54 (42–66)	1 (0–1)	711 (614–808)	15 (11–19)
Lebanon	2030	54 (40–68)	1 (0–1)	720 (614–826)	14 (10–19)
Lebanon	2031	54 (39–69)	1 (0–1)	729 (615–843)	14 (9–18)
Lebanon	2032	54 (37–71)	1 (0–1)	737 (616–859)	13 (9–18)
Lebanon	2033	54 (36–72)	1 (0–1)	746 (617–875)	13 (8–18)
Lebanon	2034	54 (35–74)	1 (0–1)	755 (619–891)	12 (7–17)
Lebanon	2035	54 (33–75)	1 (0–1)	764 (621–907)	12 (7–17)
Lebanon	2036	54 (32–76)	1 (0–1)	773 (623–922)	11 (6–17)
Lesotho	2022	10 (10–10)	1 (1–1)	1451 (1408–1494)	26 (25–26)
Lesotho	2023	9 (9–10)	1 (1–1)	1389 (1277–1501)	25 (23–27)
Lesotho	2024	9 (7–10)	1 (1–1)	1327 (1128–1525)	24 (21–27)
Lesotho	2025	8 (6–10)	1 (1–1)	1264 (965–1564)	24 (20–28)
Lesotho	2026	8 (5–11)	1 (1–1)	1202 (789–1615)	24 (18–29)
Lesotho	2027	7 (3–11)	1 (1–1)	1140 (602–1678)	24 (17–30)
Lesotho	2028	6 (1–11)	1 (1–1)	1078 (404–1752)	23 (16–31)
Lesotho	2029	6 (-0–12)	1 (0–1)	1016 (197–1835)	23 (15–32)
Lesotho	2030	5 (-2–12)	1 (0–1)	954 (-20–1927)	23 (14–32)
Lesotho	2031	5 (-4–13)	1 (0–1)	891 (-245–2028)	23 (14–33)
Lesotho	2032	4 (-6–13)	1 (0–1)	829 (-478–2137)	23 (13–34)
Lesotho	2033	3 (-8–14)	1 (0–1)	767 (-719–2253)	23 (12–35)
Lesotho	2034	3 (-10–15)	1 (0–1)	705 (-967–2377)	23 (12–35)
Lesotho	2035	2 (-12–16)	1 (0–1)	643 (-1223–2509)	23 (11–36)
Lesotho	2036	1 (-14–17)	1 (0–1)	581 (-1486–2647)	23 (11–36)
Liberia	2022	5 (5–5)	0 (0–0)	361 (356–366)	6 (5–6)
Liberia	2023	5 (5–5)	0 (0–0)	378 (367–390)	6 (5–6)
Liberia	2024	5 (5–5)	0 (0–0)	396 (376–416)	6 (5–6)
Liberia	2025	5 (5–6)	0 (0–0)	413 (384–442)	6 (5–6)

Liberia	2026	5 (5–6)	0 (0–0)	431 (392–470)	6 (5–7)
Liberia	2027	5 (4–6)	0 (0–0)	448 (398–499)	6 (5–7)
Liberia	2028	5 (4–7)	0 (0–0)	466 (403–528)	6 (4–7)
Liberia	2029	6 (4–7)	0 (0–0)	483 (408–559)	6 (4–7)
Liberia	2030	6 (4–7)	0 (0–0)	501 (411–590)	6 (4–7)
Liberia	2031	6 (4–8)	0 (0–0)	518 (414–622)	6 (4–7)
Liberia	2032	6 (4–8)	0 (0–0)	536 (417–655)	6 (4–7)
Liberia	2033	6 (4–8)	0 (0–0)	553 (418–688)	6 (4–7)
Liberia	2034	6 (4–9)	0 (0–0)	571 (419–722)	6 (4–7)
Liberia	2035	6 (3–9)	0 (0–0)	588 (420–757)	6 (4–8)
Liberia	2036	7 (3–10)	0 (0–0)	606 (419–792)	6 (4–8)
Libya	2022	31 (30–32)	1 (1–1)	1126 (1101–1150)	14 (13–15)
Libya	2023	31 (29–34)	1 (1–1)	1155 (1101–1209)	14 (13–16)
Libya	2024	32 (27–36)	1 (1–1)	1184 (1093–1275)	14 (12–16)
Libya	2025	32 (26–38)	1 (1–1)	1214 (1080–1347)	14 (11–17)
Libya	2026	33 (24–41)	1 (1–1)	1243 (1062–1423)	14 (10–18)
Libya	2027	33 (22–44)	1 (1–1)	1272 (1040–1504)	14 (10–18)
Libya	2028	33 (20–47)	1 (1–1)	1301 (1014–1589)	14 (9–19)
Libya	2029	34 (17–50)	1 (1–1)	1331 (983–1678)	14 (9–19)
Libya	2030	34 (15–54)	1 (1–1)	1360 (949–1771)	14 (9–19)
Libya	2031	35 (12–57)	1 (1–1)	1389 (912–1867)	14 (8–20)
Libya	2032	35 (9–61)	1 (1–1)	1419 (871–1966)	14 (8–20)
Libya	2033	35 (6–65)	1 (1–1)	1448 (828–2068)	14 (7–21)
Libya	2034	36 (3–69)	1 (1–1)	1477 (781–2174)	14 (7–21)
Libya	2035	36 (-0–73)	1 (1–1)	1507 (731–2282)	14 (7–21)
Libya	2036	37 (-4–77)	1 (1–1)	1536 (679–2393)	14 (6–22)
Lithuania	2022	50 (45–55)	1 (1–1)	1780 (1616–1943)	24 (21–27)
Lithuania	2023	48 (41–55)	1 (1–1)	1780 (1548–2011)	22 (18–27)
Lithuania	2024	46 (37–55)	1 (1–1)	1780 (1496–2063)	21 (16–26)
Lithuania	2025	44 (34–54)	1 (1–1)	1780 (1452–2107)	20 (14–26)
Lithuania	2026	42 (31–54)	1 (0–1)	1780 (1413–2146)	19 (12–25)
Lithuania	2027	41 (28–53)	1 (0–1)	1780 (1378–2181)	17 (10–25)
Lithuania	2028	39 (25–52)	1 (0–1)	1780 (1346–2213)	16 (8–24)

Lithuania	2029	37 (23–51)	1 (0–1)	1780 (1316–2243)	15 (6–23)
Lithuania	2030	35 (20–50)	1 (0–1)	1780 (1288–2271)	13 (5–22)
Lithuania	2031	33 (17–49)	0 (0–1)	1780 (1262–2297)	12 (3–21)
Lithuania	2032	31 (15–48)	0 (0–1)	1780 (1236–2323)	11 (1–21)
Lithuania	2033	30 (12–47)	0 (0–1)	1780 (1212–2347)	10 (-1–20)
Lithuania	2034	28 (9–46)	0 (-0–1)	1780 (1189–2370)	8 (-2–19)
Lithuania	2035	26 (7–45)	0 (-0–1)	1780 (1167–2392)	7 (-4–18)
Lithuania	2036	24 (4–44)	0 (-0–1)	1780 (1145–2414)	6 (-6–17)
Low SDI	2022	1724 (1696–1751)	0 (0–0)	28726 (127427–130025	8 (8–9)
Low SDI	2023	1737 (1698–1776)	0 (0–0)	31008 (128104–133912	8 (8–9)
Low SDI	2024	1751 (1703–1799)	0 (0–0)	33289 (128430–138149	8 (8–8)
Low SDI	2025	1764 (1709–1820)	0 (0–0)	35571 (128458–142684	8 (7–8)
Low SDI	2026	1778 (1716–1840)	0 (0–0)	37853 (128222–147484	8 (7–8)
Low SDI	2027	1792 (1724–1859)	0 (0–0)	40135 (127746–152523	7 (7–8)
Low SDI	2028	1805 (1732–1878)	0 (0–0)	42416 (127051–157782	7 (7–8)
Low SDI	2029	1819 (1741–1897)	0 (0–0)	44698 (126150–163246	7 (6–8)
Low SDI	2030	1833 (1750–1915)	0 (0–0)	46980 (125056–168903	7 (6–8)
Low SDI	2031	1846 (1759–1933)	0 (0–0)	49261 (123780–174743	7 (6–7)
Low SDI	2032	1860 (1768–1951)	0 (0–0)	51543 (122331–180755	6 (6–7)
Low SDI	2033	1874 (1778–1969)	0 (0–0)	53825 (120716–186934	6 (5–7)
Low SDI	2034	1887 (1788–1987)	0 (0–0)	56107 (118942–193271	6 (5–7)
Low SDI	2035	1901 (1798–2004)	0 (0–0)	58388 (117015–199762	6 (5–7)
Low SDI	2036	1914 (1808–2021)	0 (0–0)	60670 (114940–206400	6 (5–6)
Low-middle SDI	2022	7816 (7704–7928)	1 (1–1)	48877 (443874–453879	13 (13–14)
Low-middle SDI	2023	7876 (7683–8070)	1 (1–1)	54317 (445751–462883	13 (12–13)
Low-middle SDI	2024	7937 (7687–8186)	1 (0–1)	59865 (448336–471393	13 (12–13)
Low-middle SDI	2025	7997 (7702–8292)	1 (0–1)	65454 (451415–479493	12 (11–13)
Low-middle SDI	2026	8057 (7723–8391)	0 (0–1)	71060 (454837–487283	12 (11–13)
Low-middle SDI	2027	8117 (7747–8487)	0 (0–1)	76672 (458507–494837	11 (10–12)
Low-middle SDI	2028	8177 (7775–8579)	0 (0–0)	82287 (462360–502214	11 (10–12)
Low-middle SDI	2029	8238 (7806–8669)	0 (0–0)	87902 (466355–509450	11 (10–12)
Low-middle SDI	2030	8298 (7838–8757)	0 (0–0)	93518 (470463–516573	10 (9–11)
Low-middle SDI	2031	8358 (7872–8844)	0 (0–0)	99134 (474664–523605	10 (9–11)

Low-middle SDI	2032	8418 (7907–8929)	0 (0–0)	504751 (478942–530559	9 (8–11)
Low-middle SDI	2033	8478 (7944–9013)	0 (0–0)	510367 (483286–537448	9 (8–10)
Low-middle SDI	2034	8538 (7981–9096)	0 (0–0)	515983 (487687–544279	9 (7–10)
Low-middle SDI	2035	8599 (8019–9178)	0 (0–0)	521599 (492139–551060	8 (7–10)
Low-middle SDI	2036	8659 (8059–9259)	0 (0–0)	527216 (496634–557797	8 (6–10)
Luxembourg	2022	5 (5–5)	0 (0–0)	304 (287–321)	9 (8–10)
Luxembourg	2023	5 (4–5)	0 (0–0)	305 (286–323)	8 (7–10)
Luxembourg	2024	5 (4–5)	0 (0–0)	305 (286–324)	8 (6–10)
Luxembourg	2025	5 (4–5)	0 (0–0)	305 (286–324)	7 (5–10)
Luxembourg	2026	4 (4–5)	0 (0–0)	305 (286–324)	7 (4–10)
Luxembourg	2027	4 (3–5)	0 (0–0)	305 (286–324)	6 (3–10)
Luxembourg	2028	4 (3–5)	0 (0–0)	305 (286–324)	6 (2–10)
Luxembourg	2029	4 (3–5)	0 (0–0)	305 (286–324)	5 (0–10)
Luxembourg	2030	4 (3–5)	0 (-0–0)	305 (286–324)	5 (-1–10)
Luxembourg	2031	4 (2–5)	0 (-0–0)	305 (286–324)	4 (-2–10)
Luxembourg	2032	4 (2–5)	0 (-0–0)	305 (286–324)	4 (-3–11)
Luxembourg	2033	4 (2–5)	0 (-0–0)	305 (286–324)	3 (-4–11)
Luxembourg	2034	4 (2–6)	0 (-0–0)	305 (286–324)	3 (-6–11)
Luxembourg	2035	4 (2–6)	0 (-0–0)	305 (286–324)	2 (-7–11)
Luxembourg	2036	3 (1–6)	0 (-0–1)	305 (286–324)	2 (-8–12)
Madagascar	2022	15 (15–16)	0 (0–0)	7535 (7359–7711)	3 (3–4)
Madagascar	2023	15 (15–16)	0 (0–0)	7675 (7393–7956)	3 (3–4)
Madagascar	2024	16 (14–17)	0 (0–0)	7815 (7429–8200)	3 (3–4)
Madagascar	2025	16 (14–17)	0 (0–0)	7955 (7462–8447)	3 (3–4)
Madagascar	2026	16 (13–18)	0 (0–0)	8095 (7490–8699)	3 (3–4)
Madagascar	2027	16 (13–19)	0 (0–0)	8235 (7513–8956)	3 (2–4)
Madagascar	2028	16 (12–20)	0 (0–0)	8375 (7530–9219)	3 (2–4)
Madagascar	2029	16 (12–20)	0 (0–0)	8514 (7542–9487)	3 (2–4)
Madagascar	2030	16 (11–21)	0 (0–0)	8654 (7549–9760)	3 (1–4)
Madagascar	2031	16 (11–22)	0 (0–0)	8794 (7551–10038)	3 (1–4)
Madagascar	2032	16 (10–23)	0 (0–0)	8934 (7548–10321)	3 (1–4)
Madagascar	2033	17 (9–24)	0 (0–0)	9074 (7540–10608)	3 (1–5)
Madagascar	2034	17 (8–25)	0 (0–0)	9214 (7528–10901)	2 (0–5)

Madagascar	2035	17 (8–26)	0 (0–0)	9354 (7511–11198)	2 (0–5)
Madagascar	2036	17 (7–27)	0 (-0–0)	9494 (7490–11499)	2 (-0–5)
Malawi	2022	13 (12–13)	0 (0–0)	9111 (8974–9247)	4 (4–4)
Malawi	2023	12 (12–13)	0 (0–0)	9233 (8923–9543)	4 (4–4)
Malawi	2024	12 (12–13)	0 (0–0)	9375 (8885–9865)	4 (4–4)
Malawi	2025	12 (11–13)	0 (0–0)	9533 (8876–10189)	4 (3–4)
Malawi	2026	12 (11–14)	0 (0–0)	9700 (8897–10503)	4 (3–5)
Malawi	2027	12 (10–14)	0 (0–0)	9872 (8942–10801)	4 (3–5)
Malawi	2028	12 (10–14)	0 (0–0)	10045 (9005–11086)	4 (2–5)
Malawi	2029	12 (9–15)	0 (0–0)	10219 (9080–11358)	4 (2–5)
Malawi	2030	12 (9–15)	0 (0–0)	10392 (9164–11621)	3 (2–5)
Malawi	2031	12 (8–16)	0 (0–0)	10565 (9254–11876)	3 (1–5)
Malawi	2032	12 (8–16)	0 (0–0)	10738 (9349–12126)	3 (1–5)
Malawi	2033	12 (7–17)	0 (0–0)	10910 (9448–12371)	3 (1–6)
Malawi	2034	12 (6–17)	0 (0–0)	11082 (9551–12613)	3 (0–6)
Malawi	2035	12 (6–18)	0 (-0–0)	11254 (9657–12851)	3 (-0–6)
Malawi	2036	12 (5–18)	0 (-0–0)	11426 (9765–13087)	3 (-1–6)
Malaysia	2022	118 (110–126)	0 (0–0)	5508 (5235–5781)	10 (9–11)
Malaysia	2023	119 (111–128)	0 (0–1)	5617 (5231–6003)	10 (8–11)
Malaysia	2024	121 (111–130)	0 (0–1)	5727 (5254–6199)	9 (7–12)
Malaysia	2025	122 (112–132)	0 (0–1)	5836 (5290–6382)	9 (7–12)
Malaysia	2026	123 (113–134)	0 (0–1)	5946 (5335–6556)	9 (6–11)
Malaysia	2027	125 (113–136)	0 (0–1)	6055 (5386–6724)	9 (6–11)
Malaysia	2028	126 (114–138)	0 (0–1)	6164 (5442–6887)	8 (5–11)
Malaysia	2029	128 (115–140)	0 (0–1)	6274 (5502–7046)	8 (5–11)
Malaysia	2030	129 (116–142)	0 (0–1)	6383 (5564–7202)	8 (4–11)
Malaysia	2031	130 (117–144)	0 (0–1)	6493 (5630–7356)	7 (4–11)
Malaysia	2032	132 (118–146)	0 (0–0)	6602 (5697–7507)	7 (3–11)
Malaysia	2033	133 (118–148)	0 (0–0)	6712 (5766–7657)	7 (3–11)
Malaysia	2034	135 (119–150)	0 (0–0)	6821 (5837–7805)	6 (2–11)
Malaysia	2035	136 (120–152)	0 (0–0)	6930 (5909–7952)	6 (2–10)
Malaysia	2036	137 (121–153)	0 (0–0)	7040 (5983–8097)	6 (1–10)
Maldives	2022	1 (1–1)	0 (0–0)	31 (24–37)	7 (7–8)

Maldives	2023	1 (1–1)	0 (0–0)	31 (24–37)	7 (6–9)
Maldives	2024	1 (1–1)	0 (0–0)	31 (24–37)	7 (5–10)
Maldives	2025	1 (1–1)	0 (0–0)	31 (24–37)	7 (4–11)
Maldives	2026	1 (1–1)	0 (0–1)	31 (24–37)	7 (3–11)
Maldives	2027	1 (1–1)	0 (0–1)	31 (24–37)	7 (2–12)
Maldives	2028	1 (1–1)	0 (0–1)	31 (24–37)	7 (1–14)
Maldives	2029	1 (1–1)	0 (0–1)	31 (24–37)	7 (-0–15)
Maldives	2030	1 (1–1)	0 (0–1)	31 (24–37)	7 (-1–16)
Maldives	2031	1 (1–1)	0 (-0–1)	31 (24–37)	7 (-3–17)
Maldives	2032	1 (1–1)	0 (-0–1)	31 (24–37)	7 (-4–19)
Maldives	2033	1 (1–1)	0 (-0–1)	31 (24–37)	7 (-6–20)
Maldives	2034	1 (1–1)	0 (-0–1)	31 (24–37)	7 (-7–21)
Maldives	2035	1 (1–1)	0 (-0–1)	31 (24–37)	7 (-9–23)
Maldives	2036	1 (1–1)	0 (-0–1)	31 (24–37)	7 (-10–24)
Mali	2022	45 (45–46)	1 (1–1)	811 (804–819)	13 (13–13)
Mali	2023	45 (44–46)	1 (1–1)	824 (808–840)	13 (12–13)
Mali	2024	45 (43–47)	1 (1–1)	837 (810–864)	13 (12–13)
Mali	2025	45 (42–48)	1 (0–1)	849 (810–889)	13 (12–14)
Mali	2026	45 (41–49)	1 (0–1)	862 (808–916)	13 (11–14)
Mali	2027	45 (39–50)	1 (0–1)	875 (805–944)	12 (11–14)
Mali	2028	44 (38–51)	1 (0–1)	887 (801–973)	12 (11–14)
Mali	2029	44 (36–52)	1 (0–1)	900 (796–1003)	12 (10–14)
Mali	2030	44 (35–53)	1 (0–1)	912 (790–1035)	12 (10–15)
Mali	2031	44 (33–55)	1 (0–1)	925 (783–1067)	12 (10–15)
Mali	2032	44 (31–56)	1 (0–1)	938 (775–1101)	12 (9–15)
Mali	2033	43 (29–58)	1 (0–1)	950 (765–1135)	12 (9–15)
Mali	2034	43 (27–59)	1 (0–1)	963 (755–1170)	12 (9–15)
Mali	2035	43 (25–61)	1 (0–1)	975 (744–1206)	12 (9–16)
Mali	2036	43 (23–63)	1 (0–1)	988 (733–1243)	12 (8–16)
Malta	2022	5 (5–6)	0 (0–1)	176 (164–188)	11 (9–13)
Malta	2023	5 (4–6)	0 (0–1)	176 (160–193)	11 (7–16)
Malta	2024	5 (4–7)	0 (0–1)	176 (156–196)	12 (4–20)
Malta	2025	5 (3–7)	0 (0–1)	176 (153–200)	12 (0–24)

Malta	2026	5 (3–7)	0 (0–1)	176 (150–202)	12 (-4–28)
Malta	2027	5 (3–7)	0 (-0–1)	176 (147–205)	12 (-8–33)
Malta	2028	5 (3–8)	0 (-0–1)	176 (145–207)	13 (-13–38)
Malta	2029	5 (3–8)	0 (-0–1)	176 (143–209)	13 (-18–44)
Malta	2030	5 (2–8)	0 (-0–1)	176 (141–211)	13 (-23–49)
Malta	2031	5 (2–8)	0 (-0–1)	176 (139–213)	13 (-29–56)
Malta	2032	5 (2–8)	0 (-1–1)	176 (137–215)	14 (-35–62)
Malta	2033	5 (2–8)	0 (-1–1)	176 (136–217)	14 (-41–69)
Malta	2034	5 (2–8)	0 (-1–1)	176 (134–218)	14 (-48–76)
Malta	2035	5 (2–9)	0 (-1–1)	176 (132–220)	14 (-54–83)
Malta	2036	5 (1–9)	0 (-1–1)	176 (131–222)	14 (-61–90)
Marshall Islands	2022	0 (0–0)	1 (1–1)	6 (6–6)	28 (27–29)
Marshall Islands	2023	0 (0–0)	1 (1–1)	6 (6–7)	28 (27–29)
Marshall Islands	2024	0 (0–0)	1 (1–1)	7 (6–7)	28 (26–29)
Marshall Islands	2025	0 (0–0)	1 (1–1)	7 (6–7)	27 (26–29)
Marshall Islands	2026	0 (0–0)	1 (1–1)	7 (6–7)	27 (25–29)
Marshall Islands	2027	0 (0–0)	1 (1–1)	7 (6–7)	27 (25–29)
Marshall Islands	2028	0 (0–0)	1 (1–1)	7 (6–8)	26 (24–29)
Marshall Islands	2029	0 (0–0)	1 (1–1)	7 (6–8)	26 (24–28)
Marshall Islands	2030	0 (0–0)	1 (1–1)	7 (6–8)	26 (23–28)
Marshall Islands	2031	0 (0–0)	1 (1–1)	7 (6–8)	25 (23–28)
Marshall Islands	2032	0 (0–0)	1 (1–1)	7 (6–8)	25 (22–28)
Marshall Islands	2033	0 (0–1)	1 (1–1)	8 (6–9)	25 (22–28)
Marshall Islands	2034	0 (0–1)	1 (1–1)	8 (6–9)	24 (21–27)
Marshall Islands	2035	0 (0–1)	1 (1–1)	8 (6–9)	24 (21–27)
Marshall Islands	2036	0 (0–1)	1 (1–1)	8 (6–9)	24 (20–27)
Mauritania	2022	5 (5–5)	0 (0–0)	410 (401–418)	6 (6–6)
Mauritania	2023	5 (5–5)	0 (0–0)	432 (416–448)	6 (5–6)
Mauritania	2024	5 (5–6)	0 (0–0)	454 (428–479)	6 (5–7)
Mauritania	2025	5 (5–6)	0 (0–0)	476 (440–511)	6 (5–7)
Mauritania	2026	6 (5–6)	0 (0–0)	497 (450–544)	6 (5–7)
Mauritania	2027	6 (5–7)	0 (0–0)	519 (460–579)	6 (4–8)
Mauritania	2028	6 (5–7)	0 (0–0)	541 (468–614)	6 (4–8)

Mauritania	2029	6 (5–7)	0 (0–0)	563 (475–650)	6 (4–9)
Mauritania	2030	6 (5–8)	0 (0–0)	584 (482–687)	6 (4–9)
Mauritania	2031	6 (5–8)	0 (0–0)	606 (487–725)	6 (3–9)
Mauritania	2032	7 (4–9)	0 (0–0)	628 (492–764)	6 (3–10)
Mauritania	2033	7 (4–9)	0 (0–0)	650 (496–803)	6 (2–11)
Mauritania	2034	7 (4–10)	0 (0–0)	672 (500–843)	7 (2–11)
Mauritania	2035	7 (4–10)	0 (0–0)	693 (503–884)	7 (2–12)
Mauritania	2036	7 (4–10)	0 (0–0)	715 (505–925)	7 (1–12)
Mauritius	2022	15 (13–16)	1 (1–1)	518 (478–558)	20 (17–23)
Mauritius	2023	15 (13–17)	1 (1–1)	528 (455–602)	20 (14–26)
Mauritius	2024	15 (12–18)	1 (1–1)	538 (442–635)	20 (13–27)
Mauritius	2025	15 (11–19)	1 (1–1)	549 (434–663)	20 (11–29)
Mauritius	2026	15 (11–19)	1 (0–1)	559 (429–689)	20 (10–30)
Mauritius	2027	15 (10–20)	1 (0–1)	569 (425–713)	20 (9–31)
Mauritius	2028	15 (10–20)	1 (0–1)	579 (422–736)	20 (8–32)
Mauritius	2029	15 (9–20)	1 (0–1)	589 (421–758)	20 (7–33)
Mauritius	2030	15 (9–21)	1 (0–1)	600 (420–779)	20 (7–34)
Mauritius	2031	15 (9–21)	1 (0–1)	610 (420–800)	20 (6–34)
Mauritius	2032	15 (8–22)	1 (0–1)	620 (420–820)	20 (5–35)
Mauritius	2033	15 (8–22)	1 (0–1)	630 (421–839)	20 (4–36)
Mauritius	2034	15 (8–22)	1 (0–1)	640 (422–858)	20 (4–36)
Mauritius	2035	15 (7–22)	1 (0–1)	650 (424–877)	20 (3–37)
Mauritius	2036	15 (7–23)	1 (0–1)	661 (426–896)	20 (2–38)
Mexico	2022	472 (457–487)	0 (0–0)	6177 (5917–6436)	9 (8–9)
Mexico	2023	471 (444–498)	0 (0–0)	6177 (5810–6543)	9 (8–9)
Mexico	2024	471 (438–505)	0 (0–0)	6177 (5727–6626)	8 (7–10)
Mexico	2025	471 (432–511)	0 (0–0)	6177 (5658–6695)	8 (6–10)
Mexico	2026	471 (427–516)	0 (0–0)	6177 (5597–6756)	8 (6–10)
Mexico	2027	471 (422–520)	0 (0–0)	6177 (5541–6812)	8 (5–10)
Mexico	2028	471 (418–525)	0 (0–0)	6177 (5490–6863)	7 (4–11)
Mexico	2029	471 (414–528)	0 (0–0)	6177 (5443–6910)	7 (4–11)
Mexico	2030	471 (411–532)	0 (0–1)	6177 (5399–6954)	7 (3–11)
Mexico	2031	471 (407–535)	0 (0–1)	6177 (5357–6996)	7 (2–12)

Mexico	2032	471 (404–539)	0 (0–1)	6177 (5316–7037)	7 (1–12)
Mexico	2033	471 (401–542)	0 (-0–1)	6177 (5278–7075)	6 (0–12)
Mexico	2034	471 (398–545)	0 (-0–1)	6177 (5242–7111)	6 (-0–13)
Mexico	2035	471 (395–547)	0 (-0–1)	6177 (5206–7147)	6 (-1–13)
Mexico	2036	471 (392–550)	0 (-0–1)	6177 (5172–7181)	6 (-2–14)
Micronesia (Federated States of)	2022	1 (1–1)	1 (1–1)	19 (19–19)	42 (41–42)
Micronesia (Federated States of)	2023	1 (1–1)	1 (1–1)	19 (19–20)	41 (41–42)
Micronesia (Federated States of)	2024	1 (1–1)	1 (1–1)	20 (19–20)	41 (40–42)
Micronesia (Federated States of)	2025	1 (1–1)	1 (1–1)	20 (20–21)	41 (39–42)
Micronesia (Federated States of)	2026	1 (1–1)	1 (1–1)	20 (20–21)	40 (39–42)
Micronesia (Federated States of)	2027	1 (1–1)	1 (1–1)	21 (20–22)	40 (38–43)
Micronesia (Federated States of)	2028	1 (1–1)	1 (1–1)	21 (20–22)	40 (37–43)
Micronesia (Federated States of)	2029	1 (1–1)	1 (1–1)	21 (20–23)	40 (36–43)
Micronesia (Federated States of)	2030	1 (1–1)	1 (1–1)	22 (20–23)	39 (35–44)
Micronesia (Federated States of)	2031	1 (1–1)	1 (1–2)	22 (21–24)	39 (34–44)
Micronesia (Federated States of)	2032	1 (1–1)	1 (1–2)	22 (21–24)	39 (33–44)
Micronesia (Federated States of)	2033	1 (1–2)	1 (1–2)	23 (21–25)	38 (32–45)
Micronesia (Federated States of)	2034	1 (1–2)	1 (1–2)	23 (21–25)	38 (31–45)
Micronesia (Federated States of)	2035	1 (1–2)	1 (1–2)	23 (21–26)	38 (30–46)
Micronesia (Federated States of)	2036	1 (1–2)	1 (1–2)	24 (21–26)	37 (28–46)
Middle SDI	2022	·3345 (42560–44130	2 (2–2)	60923 (2026837–20950(	36 (35–37)
Middle SDI	2023	·3584 (41965–45203	2 (1–2)	77156 (2006029–214828	34 (32–37)
Middle SDI	2024	·3824 (41673–45974	1 (1–2)	93390 (1998752–21880:	33 (29–36)
Middle SDI	2025	·4063 (41488–46638	1 (1–2)	09623 (1996250–22229(	31 (27–35)
Middle SDI	2026	·4302 (41364–47241	1 (1–2)	25857 (1996432–225528	29 (25–34)
Middle SDI	2027	·4541 (41280–47803	1 (1–1)	42090 (1998396–228578	28 (22–33)
Middle SDI	2028	·4781 (41225–48337	1 (1–1)	58324 (2001654–23149(	26 (20–32)
Middle SDI	2029	·5020 (41193–48847	1 (1–1)	74557 (2005908–23432(	24 (18–31)
Middle SDI	2030	·5259 (41178–49340	1 (1–1)	90791 (2010958–23706:	23 (16–29)
Middle SDI	2031	·5499 (41179–49818	1 (1–1)	07024 (2016664–239738	21 (14–28)
Middle SDI	2032	·5738 (41192–50283	1 (1–1)	23258 (2022922–24235(	19 (12–27)
Middle SDI	2033	·5977 (41216–50738	1 (1–1)	39491 (2029654–24493:	18 (10–25)
Middle SDI	2034	·6216 (41249–51183	1 (1–1)	55725 (2036798–24746:	16 (8–24)

Middle SDI	2035	·6456 (41291–51621	1 (0–1)	71958 (2044304–24996	14 (6–23)
Middle SDI	2036	·6695 (41339–52050	1 (0–1)	88192 (2052133–25242	12 (4–21)
Monaco	2022	1 (1–1)	1 (1–1)	38 (38–39)	17 (17–17)
Monaco	2023	1 (1–1)	1 (1–1)	39 (38–40)	17 (16–17)
Monaco	2024	1 (1–1)	1 (1–1)	39 (37–41)	17 (16–18)
Monaco	2025	1 (1–1)	1 (1–1)	40 (37–42)	16 (15–18)
Monaco	2026	1 (1–1)	1 (1–1)	40 (36–43)	16 (14–18)
Monaco	2027	1 (1–1)	1 (1–1)	40 (36–45)	16 (14–19)
Monaco	2028	1 (1–1)	1 (1–1)	41 (35–46)	16 (13–19)
Monaco	2029	1 (1–1)	1 (1–1)	41 (34–48)	16 (12–20)
Monaco	2030	1 (1–1)	1 (1–1)	41 (33–50)	16 (11–20)
Monaco	2031	1 (1–1)	1 (0–1)	42 (32–51)	16 (11–21)
Monaco	2032	1 (1–1)	1 (0–1)	42 (31–53)	15 (10–21)
Monaco	2033	1 (1–1)	1 (0–1)	42 (29–55)	15 (9–22)
Monaco	2034	1 (1–1)	1 (0–1)	43 (28–57)	15 (8–23)
Monaco	2035	1 (1–1)	1 (0–1)	43 (27–59)	15 (7–23)
Monaco	2036	1 (1–1)	1 (0–1)	43 (25–61)	15 (6–24)
Mongolia	2022	84 (81–87)	3 (3–4)	2260 (2188–2332)	92 (87–97)
Mongolia	2023	85 (80–90)	3 (3–4)	2294 (2192–2396)	91 (84–99)
Mongolia	2024	86 (80–93)	3 (3–4)	2327 (2203–2452)	91 (81–100)
Mongolia	2025	88 (79–96)	3 (3–4)	2361 (2217–2505)	90 (79–100)
Mongolia	2026	89 (79–100)	3 (3–4)	2395 (2233–2556)	89 (77–101)
Mongolia	2027	91 (78–103)	3 (3–4)	2428 (2252–2605)	88 (75–101)
Mongolia	2028	92 (77–107)	3 (3–4)	2462 (2271–2652)	87 (73–102)
Mongolia	2029	93 (76–110)	3 (3–4)	2495 (2291–2699)	87 (72–102)
Mongolia	2030	95 (76–114)	3 (3–4)	2529 (2313–2745)	86 (70–102)
Mongolia	2031	96 (75–118)	3 (3–4)	2562 (2334–2790)	85 (68–102)
Mongolia	2032	98 (73–122)	3 (2–4)	2596 (2357–2835)	84 (67–102)
Mongolia	2033	99 (72–126)	3 (2–4)	2629 (2380–2879)	84 (65–102)
Mongolia	2034	100 (71–130)	3 (2–4)	2663 (2403–2923)	83 (64–102)
Mongolia	2035	102 (70–134)	3 (2–4)	2697 (2427–2966)	82 (62–102)
Mongolia	2036	103 (68–138)	3 (2–4)	2730 (2451–3009)	81 (61–102)
Montenegro	2022	11 (10–12)	1 (1–1)	279 (263–294)	24 (22–26)

Montenegro	2023	11 (10–12)	1 (1–1)	279 (247–310)	21 (16–25)
Montenegro	2024	11 (9–12)	1 (1–1)	279 (237–320)	17 (10–25)
Montenegro	2025	10 (8–13)	1 (1–1)	279 (229–328)	14 (4–25)
Montenegro	2026	10 (7–13)	1 (1–1)	279 (222–335)	11 (-3–25)
Montenegro	2027	10 (6–13)	1 (0–1)	279 (215–342)	8 (-11–26)
Montenegro	2028	9 (5–13)	1 (0–1)	279 (210–347)	5 (-18–27)
Montenegro	2029	9 (4–14)	1 (0–1)	279 (205–352)	1 (-26–29)
Montenegro	2030	8 (3–14)	1 (0–1)	279 (200–357)	-2 (-34–31)
Montenegro	2031	8 (2–15)	1 (-0–1)	279 (195–362)	-5 (-43–33)
Montenegro	2032	8 (0–15)	1 (-0–1)	279 (191–366)	-8 (-52–35)
Montenegro	2033	7 (-1–16)	1 (-0–1)	279 (187–370)	-11 (-61–38)
Montenegro	2034	7 (-2–16)	1 (-0–2)	279 (183–374)	-15 (-70–41)
Montenegro	2035	7 (-3–17)	1 (-1–2)	279 (179–378)	-18 (-79–44)
Montenegro	2036	6 (-4–17)	0 (-1–2)	279 (175–382)	-21 (-89–47)
Morocco	2022	40 (39–41)	0 (0–0)	1334 (1317–1351)	3 (3–3)
Morocco	2023	40 (39–41)	0 (0–0)	1359 (1321–1397)	3 (3–3)
Morocco	2024	41 (39–42)	0 (0–0)	1384 (1320–1448)	3 (2–3)
Morocco	2025	41 (38–43)	0 (0–0)	1409 (1316–1503)	3 (2–3)
Morocco	2026	41 (38–45)	0 (0–0)	1435 (1308–1561)	3 (2–3)
Morocco	2027	41 (37–46)	0 (0–0)	1460 (1297–1623)	3 (2–3)
Morocco	2028	42 (36–47)	0 (0–0)	1485 (1283–1687)	2 (2–3)
Morocco	2029	42 (35–48)	0 (0–0)	1510 (1267–1754)	2 (2–3)
Morocco	2030	42 (34–50)	0 (0–0)	1536 (1247–1824)	2 (1–3)
Morocco	2031	42 (33–51)	0 (0–0)	1561 (1226–1896)	2 (1–3)
Morocco	2032	43 (32–53)	0 (0–0)	1586 (1202–1970)	2 (1–4)
Morocco	2033	43 (31–54)	0 (0–0)	1611 (1176–2047)	2 (1–4)
Morocco	2034	43 (30–56)	0 (0–0)	1637 (1148–2125)	2 (1–4)
Morocco	2035	43 (29–57)	0 (0–0)	1662 (1118–2206)	2 (0–4)
Morocco	2036	44 (28–59)	0 (0–0)	1687 (1086–2288)	2 (0–4)
Mozambique	2022	30 (29–30)	0 (0–0)	3989 (3945–4034)	7 (7–7)
Mozambique	2023	29 (28–30)	0 (0–0)	3973 (3876–4070)	7 (6–7)
Mozambique	2024	29 (27–30)	0 (0–0)	3958 (3798–4117)	6 (6–7)
Mozambique	2025	28 (26–30)	0 (0–0)	3943 (3714–4172)	6 (5–7)

Mozambique	2026	28 (25–31)	0 (0–0)	3929 (3625–4233)	6 (5–7)
Mozambique	2027	27 (24–31)	0 (0–0)	3916 (3533–4300)	5 (4–6)
Mozambique	2028	27 (23–31)	0 (0–0)	3904 (3437–4371)	5 (4–6)
Mozambique	2029	27 (21–32)	0 (0–0)	3892 (3339–4445)	5 (3–6)
Mozambique	2030	26 (20–32)	0 (0–0)	3881 (3239–4523)	5 (3–6)
Mozambique	2031	26 (19–33)	0 (0–0)	3871 (3138–4603)	4 (2–6)
Mozambique	2032	26 (18–33)	0 (0–0)	3861 (3036–4685)	4 (1–7)
Mozambique	2033	26 (17–34)	0 (0–0)	3851 (2933–4769)	4 (1–7)
Mozambique	2034	25 (16–34)	0 (0–0)	3842 (2830–4854)	3 (0–7)
Mozambique	2035	25 (15–35)	0 (0–0)	3834 (2727–4941)	3 (-1–7)
Mozambique	2036	25 (14–35)	0 (0–0)	3826 (2623–5028)	3 (-1–7)
Myanmar	2022	269 (264–273)	1 (1–1)	10363 (10234–10492)	13 (13–13)
Myanmar	2023	270 (260–279)	1 (1–1)	10496 (10248–10744)	13 (12–14)
Myanmar	2024	271 (256–287)	1 (0–1)	10631 (10234–11029)	13 (11–14)
Myanmar	2025	272 (250–295)	1 (0–1)	10766 (10199–11333)	12 (11–14)
Myanmar	2026	274 (243–305)	1 (0–1)	10901 (10144–11657)	12 (10–14)
Myanmar	2027	275 (235–315)	1 (0–1)	11035 (10073–11998)	12 (9–15)
Myanmar	2028	276 (227–326)	0 (0–1)	11170 (9986–12354)	12 (8–15)
Myanmar	2029	278 (218–337)	0 (0–1)	11305 (9884–12725)	11 (7–16)
Myanmar	2030	279 (209–349)	0 (0–1)	11439 (9768–13110)	11 (6–16)
Myanmar	2031	280 (198–362)	0 (0–1)	11574 (9639–13508)	11 (4–17)
Myanmar	2032	282 (188–375)	0 (0–1)	11708 (9498–13919)	10 (3–18)
Myanmar	2033	283 (177–389)	0 (0–1)	11843 (9344–14342)	10 (2–18)
Myanmar	2034	284 (165–403)	0 (0–1)	11978 (9179–14776)	10 (1–19)
Myanmar	2035	286 (153–418)	0 (0–1)	12112 (9003–15221)	10 (-1–20)
Myanmar	2036	287 (140–434)	0 (-0–1)	12247 (8817–15677)	9 (-2–21)
Namibia	2022	2 (2–2)	0 (0–0)	142 (139–146)	3 (3–4)
Namibia	2023	2 (2–2)	0 (0–0)	142 (135–150)	3 (3–4)
Namibia	2024	2 (2–2)	0 (0–0)	143 (131–155)	3 (3–4)
Namibia	2025	2 (2–2)	0 (0–0)	143 (126–160)	3 (2–4)
Namibia	2026	2 (2–2)	0 (0–0)	143 (122–165)	3 (2–4)
Namibia	2027	2 (1–2)	0 (0–0)	143 (117–170)	3 (2–4)
Namibia	2028	2 (1–2)	0 (0–0)	143 (112–175)	3 (1–5)

Namibia	2029	2 (1–2)	0 (0–0)	144 (108–180)	3 (1–5)
Namibia	2030	2 (1–2)	0 (0–0)	144 (103–184)	3 (0–5)
Namibia	2031	2 (1–2)	0 (-0–0)	144 (98–189)	3 (-0–6)
Namibia	2032	2 (1–2)	0 (-0–0)	144 (94–194)	3 (-1–6)
Namibia	2033	2 (1–2)	0 (-0–0)	144 (90–198)	2 (-1–6)
Namibia	2034	2 (1–2)	0 (-0–0)	144 (85–203)	2 (-2–7)
Namibia	2035	2 (1–2)	0 (-0–0)	144 (81–207)	2 (-2–7)
Namibia	2036	2 (1–2)	0 (-0–0)	144 (77–211)	2 (-3–7)
Nauru	2022	0 (0–0)	1 (1–2)	1 (1–2)	44 (44–45)
Nauru	2023	0 (0–0)	1 (1–2)	2 (1–2)	44 (42–45)
Nauru	2024	0 (0–0)	1 (1–2)	2 (1–2)	43 (40–45)
Nauru	2025	0 (0–0)	1 (1–2)	2 (1–2)	42 (38–46)
Nauru	2026	0 (0–0)	1 (1–2)	2 (1–2)	41 (36–46)
Nauru	2027	0 (0–0)	1 (1–2)	2 (1–2)	40 (34–47)
Nauru	2028	0 (0–0)	1 (1–2)	2 (1–2)	40 (32–48)
Nauru	2029	0 (0–0)	1 (1–2)	2 (1–2)	39 (29–49)
Nauru	2030	0 (0–0)	1 (1–2)	2 (1–2)	38 (27–50)
Nauru	2031	0 (0–0)	1 (1–2)	2 (1–2)	37 (24–51)
Nauru	2032	0 (0–0)	1 (1–2)	2 (1–2)	37 (22–52)
Nauru	2033	0 (0–0)	1 (1–2)	2 (1–2)	36 (19–53)
Nauru	2034	0 (0–0)	1 (1–2)	2 (1–2)	35 (16–54)
Nauru	2035	0 (0–0)	1 (0–2)	2 (1–2)	34 (13–56)
Nauru	2036	0 (0–0)	1 (0–2)	2 (1–2)	34 (10–57)
Nepal	2022	109 (107–110)	0 (0–1)	10147 (10038–10255)	11 (11–11)
Nepal	2023	109 (106–112)	0 (0–1)	10245 (10023–10468)	11 (10–11)
Nepal	2024	110 (105–114)	0 (0–1)	10363 (9967–10758)	11 (10–11)
Nepal	2025	110 (104–117)	0 (0–1)	10458 (9866–11051)	10 (9–12)
Nepal	2026	111 (102–119)	0 (0–1)	10566 (9738–11394)	10 (8–12)
Nepal	2027	111 (100–122)	0 (0–1)	10662 (9576–11748)	10 (8–12)
Nepal	2028	112 (98–126)	0 (0–1)	10766 (9393–12138)	10 (7–13)
Nepal	2029	113 (96–129)	0 (0–1)	10863 (9183–12543)	10 (6–13)
Nepal	2030	113 (94–132)	0 (0–1)	10965 (8955–12974)	10 (6–13)
Nepal	2031	114 (92–136)	0 (0–1)	11063 (8705–13421)	9 (5–14)

Nepal	2032	114 (89–139)	0 (0–1)	11163 (8437–13890)	9 (4–14)
Nepal	2033	115 (86–143)	0 (0–1)	11262 (8151–14374)	9 (3–15)
Nepal	2034	115 (84–147)	0 (0–1)	11362 (7848–14877)	9 (3–15)
Nepal	2035	116 (81–151)	0 (0–1)	11462 (7529–15395)	9 (2–16)
Nepal	2036	117 (78–156)	0 (0–1)	11562 (7194–15929)	9 (1–16)
Netherlands	2022	188 (169–206)	0 (0–1)	17256 (16593–17919)	10 (8–11)
Netherlands	2023	183 (155–211)	0 (0–1)	17101 (16040–18162)	9 (7–12)
Netherlands	2024	178 (140–216)	0 (0–1)	16945 (15492–18398)	9 (5–13)
Netherlands	2025	173 (126–221)	0 (0–1)	16789 (14931–18648)	8 (4–13)
Netherlands	2026	169 (111–226)	0 (0–1)	16634 (14353–18915)	8 (2–14)
Netherlands	2027	164 (96–232)	0 (0–1)	16478 (13755–19202)	7 (0–15)
Netherlands	2028	159 (80–238)	0 (-0–1)	16323 (13137–19509)	7 (-2–16)
Netherlands	2029	154 (65–244)	0 (-0–1)	16167 (12499–19835)	6 (-4–17)
Netherlands	2030	150 (48–251)	0 (-0–1)	16011 (11842–20181)	6 (-6–18)
Netherlands	2031	145 (32–258)	0 (-0–1)	15856 (11165–20546)	5 (-8–19)
Netherlands	2032	140 (15–265)	0 (-0–1)	15700 (10470–20930)	5 (-10–20)
Netherlands	2033	135 (-3–273)	0 (-1–1)	15545 (9758–21332)	5 (-12–21)
Netherlands	2034	131 (-20–281)	0 (-1–1)	15389 (9027–21751)	4 (-14–23)
Netherlands	2035	126 (-38–290)	0 (-1–1)	15233 (8279–22188)	4 (-17–24)
Netherlands	2036	121 (-57–299)	0 (-1–1)	15078 (7515–22641)	3 (-19–25)
New Zealand	2022	31 (29–33)	0 (0–0)	1459 (1324–1595)	9 (8–9)
New Zealand	2023	31 (28–35)	0 (0–0)	1459 (1268–1651)	8 (6–10)
New Zealand	2024	32 (27–37)	0 (0–0)	1459 (1225–1694)	8 (6–11)
New Zealand	2025	32 (26–38)	0 (0–0)	1459 (1188–1730)	8 (5–11)
New Zealand	2026	32 (25–40)	0 (0–1)	1459 (1156–1762)	8 (4–12)
New Zealand	2027	33 (24–41)	0 (0–1)	1459 (1127–1791)	8 (2–13)
New Zealand	2028	33 (23–43)	0 (0–1)	1459 (1101–1818)	8 (1–14)
New Zealand	2029	33 (22–45)	0 (0–1)	1459 (1076–1842)	8 (-0–15)
New Zealand	2030	34 (20–47)	0 (-0–1)	1459 (1053–1866)	7 (-1–16)
New Zealand	2031	34 (19–49)	0 (-0–1)	1459 (1031–1888)	7 (-3–18)
New Zealand	2032	34 (18–51)	0 (-0–1)	1459 (1010–1908)	7 (-4–19)
New Zealand	2033	35 (16–53)	0 (-0–1)	1459 (990–1928)	7 (-6–20)
New Zealand	2034	35 (15–55)	0 (-0–1)	1459 (971–1948)	7 (-8–22)

New Zealand	2035	35 (14–57)	0 (-0–1)	1459 (952–1966)	7 (-9–23)
New Zealand	2036	36 (12–59)	0 (-0–1)	1459 (934–1984)	7 (-11–25)
Nicaragua	2022	23 (22–25)	0 (0–1)	187 (176–198)	12 (10–13)
Nicaragua	2023	24 (22–25)	0 (0–1)	190 (178–202)	11 (10–13)
Nicaragua	2024	24 (22–26)	0 (0–1)	193 (180–207)	11 (9–13)
Nicaragua	2025	24 (22–26)	0 (0–1)	197 (183–211)	11 (9–13)
Nicaragua	2026	25 (22–27)	0 (0–1)	200 (185–215)	11 (9–12)
Nicaragua	2027	25 (23–27)	0 (0–1)	204 (188–219)	10 (8–12)
Nicaragua	2028	25 (23–28)	0 (0–0)	207 (190–223)	10 (8–12)
Nicaragua	2029	26 (23–28)	0 (0–0)	210 (193–228)	10 (8–12)
Nicaragua	2030	26 (23–29)	0 (0–0)	214 (195–232)	10 (7–12)
Nicaragua	2031	26 (23–29)	0 (0–0)	217 (198–236)	9 (7–12)
Nicaragua	2032	27 (24–30)	0 (0–0)	220 (201–240)	9 (7–12)
Nicaragua	2033	27 (24–30)	0 (0–0)	224 (203–244)	9 (6–11)
Nicaragua	2034	28 (24–31)	0 (0–0)	227 (206–248)	9 (6–11)
Nicaragua	2035	28 (24–31)	0 (0–0)	230 (209–252)	8 (6–11)
Nicaragua	2036	28 (25–32)	0 (0–0)	234 (212–256)	8 (5–11)
Niger	2022	13 (13–13)	0 (0–0)	910 (896–923)	4 (4–4)
Niger	2023	13 (13–14)	0 (0–0)	931 (908–953)	4 (4–4)
Niger	2024	13 (13–14)	0 (0–0)	954 (918–991)	4 (4–4)
Niger	2025	14 (13–14)	0 (0–0)	976 (925–1027)	4 (4–4)
Niger	2026	14 (13–15)	0 (0–0)	999 (932–1067)	4 (3–4)
Niger	2027	14 (13–15)	0 (0–0)	1022 (936–1107)	4 (3–4)
Niger	2028	14 (13–16)	0 (0–0)	1044 (940–1149)	4 (3–4)
Niger	2029	14 (13–16)	0 (0–0)	1067 (942–1192)	4 (3–4)
Niger	2030	15 (12–17)	0 (0–0)	1089 (943–1236)	4 (3–4)
Niger	2031	15 (12–17)	0 (0–0)	1112 (942–1282)	3 (3–4)
Niger	2032	15 (12–18)	0 (0–0)	1135 (941–1328)	3 (3–4)
Niger	2033	15 (12–19)	0 (0–0)	1157 (939–1376)	3 (3–4)
Niger	2034	15 (12–19)	0 (0–0)	1180 (935–1424)	3 (3–4)
Niger	2035	16 (12–20)	0 (0–0)	1202 (931–1474)	3 (3–4)
Niger	2036	16 (11–20)	0 (0–0)	1225 (926–1524)	3 (3–4)
Nigeria	2022	25 (25–25)	0 (0–0)	6014 (5941–6087)	1 (1–1)

Nigeria	2023	25 (24–26)	0 (0–0)	6217 (6054–6380)	1 (1–1)
Nigeria	2024	25 (24–27)	0 (0–0)	6419 (6147–6692)	1 (1–1)
Nigeria	2025	25 (23–28)	0 (0–0)	6622 (6223–7021)	1 (1–1)
Nigeria	2026	25 (22–28)	0 (0–0)	6825 (6285–7365)	1 (0–1)
Nigeria	2027	25 (21–29)	0 (0–0)	7027 (6333–7722)	1 (0–1)
Nigeria	2028	26 (21–30)	0 (0–0)	7230 (6368–8091)	1 (0–1)
Nigeria	2029	26 (20–32)	0 (0–0)	7432 (6393–8472)	1 (0–1)
Nigeria	2030	26 (19–33)	0 (0–0)	7635 (6406–8864)	1 (0–1)
Nigeria	2031	26 (17–34)	0 (0–0)	7838 (6409–9266)	1 (0–1)
Nigeria	2032	26 (16–35)	0 (0–0)	8040 (6403–9678)	0 (0–1)
Nigeria	2033	26 (15–37)	0 (0–0)	8243 (6387–10099)	0 (0–1)
Nigeria	2034	26 (14–38)	0 (-0–0)	8446 (6362–10529)	0 (-0–1)
Nigeria	2035	26 (13–39)	0 (-0–0)	8648 (6329–10968)	0 (-0–1)
Nigeria	2036	26 (11–41)	0 (-0–0)	8851 (6287–11415)	0 (-0–1)
Niue	2022	0 (0–0)	1 (1–1)	0 (0–0)	18 (17–18)
Niue	2023	0 (0–0)	1 (1–1)	0 (0–0)	18 (17–18)
Niue	2024	0 (0–0)	1 (1–1)	0 (0–0)	18 (17–19)
Niue	2025	0 (0–0)	1 (1–1)	0 (0–0)	18 (16–19)
Niue	2026	0 (0–0)	1 (1–1)	0 (0–0)	18 (16–19)
Niue	2027	0 (0–0)	1 (1–1)	0 (0–0)	18 (15–20)
Niue	2028	0 (0–0)	1 (1–1)	0 (0–0)	18 (15–20)
Niue	2029	0 (0–0)	1 (1–1)	0 (0–0)	18 (14–21)
Niue	2030	0 (0–0)	1 (1–1)	0 (0–0)	18 (14–21)
Niue	2031	0 (0–0)	1 (1–1)	0 (0–0)	17 (14–21)
Niue	2032	0 (0–0)	1 (1–1)	0 (0–0)	17 (13–22)
Niue	2033	0 (0–0)	1 (1–1)	0 (0–0)	17 (13–22)
Niue	2034	0 (0–0)	1 (0–1)	0 (0–0)	17 (12–23)
Niue	2035	0 (0–0)	1 (0–1)	0 (0–0)	17 (12–23)
Niue	2036	0 (0–0)	1 (0–1)	0 (0–0)	17 (11–23)
North Macedonia	2022	63 (59–66)	2 (2–2)	585 (558–613)	44 (40–48)
North Macedonia	2023	62 (56–67)	2 (2–2)	570 (530–610)	42 (35–50)
North Macedonia	2024	59 (52–66)	2 (1–2)	554 (506–603)	41 (31–51)
North Macedonia	2025	58 (48–67)	2 (1–2)	542 (478–607)	40 (28–52)

North Macedonia	2026	57 (45–68)	2 (1–2)	530 (443–617)	38 (25–52)
North Macedonia	2027	55 (40–70)	1 (1–2)	516 (407–624)	37 (22–52)
North Macedonia	2028	53 (35–70)	1 (0–2)	502 (372–631)	36 (19–53)
North Macedonia	2029	51 (31–72)	1 (-0–3)	489 (335–642)	35 (17–52)
North Macedonia	2030	50 (26–74)	1 (-0–3)	476 (295–656)	33 (14–52)
North Macedonia	2031	48 (21–76)	1 (-1–3)	462 (254–670)	32 (12–52)
North Macedonia	2032	46 (16–77)	1 (-1–3)	448 (212–684)	31 (9–52)
North Macedonia	2033	45 (11–79)	1 (-1–3)	435 (169–700)	29 (7–52)
North Macedonia	2034	44 (5–82)	1 (-2–3)	421 (125–718)	28 (5–51)
North Macedonia	2035	42 (-0–84)	1 (-2–4)	408 (79–737)	27 (3–51)
North Macedonia	2036	40 (-6–86)	1 (-2–4)	394 (32–756)	25 (0–50)
Northern Mariana Islands	2022	0 (0–0)	1 (1–1)	9 (9–10)	20 (19–21)
Northern Mariana Islands	2023	0 (0–0)	1 (1–1)	9 (8–10)	20 (19–22)
Northern Mariana Islands	2024	0 (0–0)	1 (1–1)	10 (8–11)	20 (18–22)
Northern Mariana Islands	2025	0 (0–0)	1 (1–1)	10 (8–11)	20 (16–23)
Northern Mariana Islands	2026	0 (0–0)	1 (0–1)	10 (8–12)	19 (15–23)
Northern Mariana Islands	2027	0 (0–0)	1 (0–1)	10 (8–12)	19 (14–24)
Northern Mariana Islands	2028	0 (0–1)	1 (0–1)	11 (8–13)	19 (13–25)
Northern Mariana Islands	2029	0 (0–1)	1 (0–1)	11 (8–13)	19 (11–26)
Northern Mariana Islands	2030	0 (0–1)	1 (0–1)	11 (8–14)	18 (10–27)
Northern Mariana Islands	2031	0 (0–1)	1 (0–1)	11 (9–14)	18 (8–28)
Northern Mariana Islands	2032	0 (0–1)	1 (-0–1)	11 (9–14)	18 (7–29)
Northern Mariana Islands	2033	0 (0–1)	1 (-0–1)	12 (9–15)	18 (5–30)
Northern Mariana Islands	2034	0 (0–1)	1 (-0–1)	12 (9–15)	17 (3–32)
Northern Mariana Islands	2035	0 (0–1)	1 (-0–1)	12 (9–15)	17 (1–33)
Northern Mariana Islands	2036	0 (0–1)	1 (-0–2)	12 (9–16)	17 (-0–34)
Norway	2022	23 (21–25)	0 (0–0)	1652 (1550–1755)	5 (4–5)
Norway	2023	23 (20–27)	0 (0–0)	1641 (1503–1779)	5 (4–5)
Norway	2024	23 (18–29)	0 (0–0)	1631 (1470–1792)	4 (3–5)
Norway	2025	23 (16–31)	0 (0–0)	1622 (1444–1800)	4 (3–6)
Norway	2026	23 (13–34)	0 (0–0)	1614 (1423–1804)	4 (2–6)
Norway	2027	23 (11–36)	0 (0–0)	1607 (1406–1807)	4 (1–7)
Norway	2028	23 (8–39)	0 (0–0)	1600 (1392–1808)	4 (0–7)

Norway	2029	24 (5–42)	0 (-0–0)	1594 (1381–1807)	4 (-1–8)
Norway	2030	24 (2–45)	0 (-0–0)	1589 (1371–1807)	3 (-1–8)
Norway	2031	24 (-2–49)	0 (-0–0)	1584 (1362–1806)	3 (-2–9)
Norway	2032	24 (-5–52)	0 (-0–0)	1579 (1355–1804)	3 (-4–10)
Norway	2033	24 (-9–56)	0 (-0–0)	1575 (1348–1803)	3 (-5–10)
Norway	2034	24 (-13–60)	0 (-0–0)	1572 (1343–1801)	3 (-6–11)
Norway	2035	24 (-17–64)	0 (-0–1)	1569 (1338–1799)	3 (-7–12)
Norway	2036	24 (-21–68)	0 (-0–1)	1566 (1334–1798)	2 (-8–13)
Oman	2022	4 (4–4)	0 (0–0)	154 (148–160)	5 (5–5)
Oman	2023	4 (4–5)	0 (0–0)	158 (148–167)	5 (4–5)
Oman	2024	4 (3–5)	0 (0–0)	161 (148–174)	4 (3–5)
Oman	2025	4 (3–5)	0 (0–0)	165 (148–181)	4 (2–6)
Oman	2026	4 (3–5)	0 (-0–0)	168 (148–188)	3 (1–6)
Oman	2027	4 (2–6)	0 (-0–0)	171 (148–195)	3 (-1–6)
Oman	2028	4 (2–6)	0 (-0–0)	175 (147–203)	2 (-2–6)
Oman	2029	4 (1–7)	0 (-0–0)	178 (146–210)	2 (-3–7)
Oman	2030	4 (1–7)	-0 (-0–0)	182 (146–218)	1 (-5–7)
Oman	2031	4 (0–7)	-0 (-0–0)	185 (145–225)	1 (-6–8)
Oman	2032	4 (-1–8)	-0 (-0–0)	188 (143–233)	1 (-7–9)
Oman	2033	4 (-1–9)	-0 (-0–0)	192 (142–242)	0 (-9–9)
Oman	2034	4 (-2–9)	-0 (-1–0)	195 (141–250)	-0 (-11–10)
Oman	2035	4 (-2–10)	-0 (-1–0)	199 (139–258)	-1 (-12–10)
Oman	2036	4 (-3–10)	-0 (-1–0)	202 (137–267)	-1 (-14–11)
Pakistan	2022	370 (363–378)	0 (0–0)	64057 (63343–64771)	8 (7–8)
Pakistan	2023	372 (359–384)	0 (0–0)	65071 (63475–66668)	7 (7–8)
Pakistan	2024	374 (356–391)	0 (0–0)	66086 (63414–68758)	7 (6–8)
Pakistan	2025	376 (354–398)	0 (0–0)	67100 (63189–71012)	7 (5–8)
Pakistan	2026	379 (352–405)	0 (0–0)	68115 (62819–73411)	7 (5–9)
Pakistan	2027	381 (351–412)	0 (0–0)	69130 (62317–75942)	6 (4–9)
Pakistan	2028	384 (350–418)	0 (0–0)	70144 (61695–78593)	6 (3–9)
Pakistan	2029	386 (350–423)	0 (0–0)	71159 (60959–81358)	6 (2–10)
Pakistan	2030	389 (350–427)	0 (0–0)	72173 (60118–84229)	6 (1–10)
Pakistan	2031	391 (351–430)	0 (0–0)	73188 (59176–87199)	5 (0–11)

Pakistan	2032	392 (352–433)	0 (-0–0)	74202 (58139–90265)	5 (-1–11)
Pakistan	2033	393 (353–434)	0 (-0–0)	75217 (57011–93423)	5 (-2–12)
Pakistan	2034	394 (353–435)	0 (-0–0)	76231 (55795–96667)	5 (-3–12)
Pakistan	2035	394 (353–435)	0 (-0–1)	77246 (54495–99996)	5 (-4–13)
Pakistan	2036	394 (353–435)	0 (-0–1)	78260 (53114–103406)	4 (-5–13)
Palau	2022	0 (0–0)	1 (1–1)	5 (5–5)	26 (25–27)
Palau	2023	0 (0–0)	1 (1–1)	5 (5–5)	26 (25–27)
Palau	2024	0 (0–0)	1 (1–1)	5 (5–6)	25 (24–27)
Palau	2025	0 (0–0)	1 (1–1)	5 (5–6)	25 (23–27)
Palau	2026	0 (0–0)	1 (1–1)	6 (5–6)	25 (22–27)
Palau	2027	0 (0–0)	1 (1–1)	6 (5–6)	24 (21–27)
Palau	2028	0 (0–0)	1 (1–1)	6 (5–6)	24 (21–27)
Palau	2029	0 (0–0)	1 (1–1)	6 (5–6)	23 (20–27)
Palau	2030	0 (0–0)	1 (1–1)	6 (5–7)	23 (19–26)
Palau	2031	0 (0–0)	1 (1–1)	6 (5–7)	22 (18–26)
Palau	2032	0 (0–0)	1 (1–1)	6 (5–7)	22 (18–26)
Palau	2033	0 (0–0)	1 (1–1)	6 (5–7)	21 (17–26)
Palau	2034	0 (0–0)	1 (1–1)	6 (5–7)	21 (16–26)
Palau	2035	0 (0–0)	1 (1–1)	6 (5–7)	20 (15–25)
Palau	2036	0 (0–0)	1 (1–1)	6 (6–7)	20 (15–25)
Palestine	2022	16 (16–17)	1 (1–1)	208 (202–214)	15 (14–16)
Palestine	2023	17 (15–18)	1 (1–1)	214 (202–225)	15 (14–16)
Palestine	2024	17 (15–18)	1 (1–1)	219 (202–235)	15 (12–17)
Palestine	2025	17 (15–19)	1 (1–1)	223 (202–243)	14 (11–17)
Palestine	2026	17 (14–19)	1 (0–1)	227 (202–251)	14 (10–17)
Palestine	2027	17 (14–20)	1 (0–1)	230 (202–258)	13 (9–17)
Palestine	2028	17 (14–20)	1 (0–1)	234 (203–264)	13 (8–17)
Palestine	2029	17 (13–21)	1 (0–1)	237 (203–271)	12 (8–17)
Palestine	2030	17 (13–21)	1 (0–1)	240 (204–277)	12 (7–17)
Palestine	2031	17 (13–21)	1 (0–1)	244 (205–283)	11 (6–16)
Palestine	2032	17 (13–21)	1 (0–1)	247 (206–288)	11 (5–16)
Palestine	2033	17 (12–22)	0 (0–1)	250 (207–294)	10 (4–16)
Palestine	2034	17 (12–22)	0 (0–1)	254 (208–299)	10 (3–16)

Palestine	2035	17 (12–22)	0 (0–1)	257 (209–305)	9 (2–16)
Palestine	2036	17 (12–22)	0 (0–1)	260 (211–310)	8 (2–15)
Panama	2022	18 (17–19)	0 (0–0)	200 (189–211)	9 (7–10)
Panama	2023	18 (17–20)	0 (0–0)	202 (186–217)	8 (6–10)
Panama	2024	19 (17–21)	0 (0–0)	203 (184–222)	8 (6–11)
Panama	2025	19 (17–21)	0 (0–0)	205 (183–227)	8 (5–11)
Panama	2026	19 (17–21)	0 (0–0)	206 (182–231)	7 (4–11)
Panama	2027	19 (16–22)	0 (0–0)	208 (181–235)	7 (4–10)
Panama	2028	19 (16–22)	0 (0–0)	210 (181–238)	7 (3–10)
Panama	2029	19 (16–23)	0 (0–0)	211 (181–242)	6 (2–10)
Panama	2030	20 (16–23)	0 (0–0)	213 (180–245)	6 (2–10)
Panama	2031	20 (16–23)	0 (0–0)	214 (180–249)	6 (1–10)
Panama	2032	20 (16–24)	0 (0–0)	216 (180–252)	5 (1–10)
Panama	2033	20 (16–24)	0 (0–0)	218 (180–255)	5 (-0–10)
Panama	2034	20 (16–24)	0 (-0–0)	219 (180–258)	4 (-1–10)
Panama	2035	20 (16–25)	0 (-0–0)	221 (180–262)	4 (-1–9)
Panama	2036	21 (16–25)	0 (-0–0)	222 (180–265)	4 (-2–9)
Papua New Guinea	2022	40 (39–40)	1 (1–1)	501 (491–511)	20 (19–20)
Papua New Guinea	2023	41 (39–42)	1 (1–1)	517 (500–534)	20 (19–20)
Papua New Guinea	2024	42 (40–44)	1 (1–1)	534 (509–558)	19 (18–21)
Papua New Guinea	2025	43 (40–46)	1 (1–1)	550 (516–583)	19 (18–21)
Papua New Guinea	2026	44 (41–47)	1 (1–1)	566 (523–609)	19 (17–21)
Papua New Guinea	2027	45 (41–49)	1 (1–1)	583 (530–635)	19 (17–22)
Papua New Guinea	2028	46 (42–51)	1 (1–1)	599 (535–662)	19 (16–22)
Papua New Guinea	2029	48 (42–53)	1 (1–1)	615 (541–690)	19 (16–22)
Papua New Guinea	2030	49 (42–55)	1 (1–1)	631 (545–718)	19 (15–23)
Papua New Guinea	2031	50 (42–58)	1 (0–1)	648 (549–747)	19 (14–23)
Papua New Guinea	2032	51 (43–60)	1 (0–1)	664 (553–776)	19 (14–23)
Papua New Guinea	2033	52 (43–62)	1 (0–1)	680 (555–805)	18 (13–24)
Papua New Guinea	2034	53 (43–64)	1 (0–1)	697 (558–836)	18 (13–24)
Papua New Guinea	2035	55 (43–66)	1 (0–1)	713 (560–866)	18 (12–25)
Papua New Guinea	2036	56 (43–69)	1 (0–1)	729 (561–897)	18 (11–25)
Paraguay	2022	46 (43–48)	1 (1–1)	1969 (1901–2037)	17 (16–19)

Paraguay	2023	46 (41–50)	1 (1–1)	2006 (1910–2102)	17 (14–20)
Paraguay	2024	46 (40–51)	1 (1–1)	2043 (1926–2161)	17 (13–20)
Paraguay	2025	46 (38–53)	1 (1–1)	2080 (1945–2216)	16 (11–21)
Paraguay	2026	46 (37–54)	1 (0–1)	2117 (1966–2269)	16 (10–21)
Paraguay	2027	46 (36–55)	1 (0–1)	2154 (1988–2321)	15 (9–21)
Paraguay	2028	46 (35–56)	1 (0–1)	2191 (2012–2371)	15 (8–21)
Paraguay	2029	46 (34–57)	1 (0–1)	2228 (2036–2420)	14 (7–21)
Paraguay	2030	46 (34–57)	1 (0–1)	2265 (2062–2469)	14 (6–21)
Paraguay	2031	46 (33–58)	1 (0–1)	2302 (2088–2517)	13 (5–21)
Paraguay	2032	46 (32–59)	1 (0–1)	2339 (2114–2564)	13 (4–21)
Paraguay	2033	46 (32–59)	1 (0–1)	2376 (2141–2611)	12 (3–21)
Paraguay	2034	46 (31–60)	1 (0–1)	2413 (2169–2658)	12 (3–21)
Paraguay	2035	46 (30–61)	0 (0–1)	2450 (2196–2704)	11 (2–21)
Paraguay	2036	46 (30–61)	0 (0–1)	2487 (2225–2750)	11 (1–21)
Peru	2022	215 (199–231)	1 (1–1)	997 (925–1069)	15 (13–17)
Peru	2023	218 (196–241)	1 (1–1)	994 (869–1119)	15 (12–18)
Peru	2024	221 (194–249)	1 (1–1)	993 (822–1164)	15 (12–18)
Peru	2025	225 (193–257)	1 (0–1)	992 (783–1202)	15 (11–19)
Peru	2026	228 (192–264)	1 (0–1)	992 (749–1236)	15 (11–19)
Peru	2027	231 (192–270)	1 (0–1)	992 (718–1266)	15 (10–20)
Peru	2028	235 (192–277)	1 (0–1)	992 (691–1293)	15 (10–20)
Peru	2029	238 (193–283)	1 (0–1)	992 (666–1318)	15 (10–20)
Peru	2030	241 (193–289)	1 (0–1)	992 (642–1342)	15 (9–21)
Peru	2031	244 (194–295)	1 (0–1)	992 (620–1364)	15 (9–21)
Peru	2032	248 (195–300)	1 (0–1)	992 (600–1385)	15 (9–21)
Peru	2033	251 (196–306)	1 (0–1)	992 (580–1404)	15 (8–22)
Peru	2034	254 (197–312)	1 (0–1)	992 (561–1423)	15 (8–22)
Peru	2035	257 (198–317)	1 (0–1)	992 (543–1441)	15 (8–22)
Peru	2036	261 (199–322)	1 (0–1)	992 (526–1458)	15 (8–22)
Philippines	2022	326 (318–333)	0 (0–0)	10463 (10270–10656)	10 (9–10)
Philippines	2023	328 (320–336)	0 (0–0)	10642 (10369–10914)	10 (9–10)
Philippines	2024	331 (321–341)	0 (0–0)	10820 (10486–11155)	9 (8–10)
Philippines	2025	334 (321–346)	0 (0–0)	10999 (10613–11385)	9 (8–10)

Philippines	2026	336 (320–353)	0 (0–0)	11178 (10746–11609)	9 (7–10)
Philippines	2027	339 (318–360)	0 (0–0)	11356 (10884–11829)	8 (7–10)
Philippines	2028	342 (316–367)	0 (0–0)	11535 (11025–12046)	8 (6–10)
Philippines	2029	345 (314–375)	0 (0–0)	11714 (11168–12260)	8 (6–10)
Philippines	2030	347 (311–383)	0 (0–0)	11893 (11314–12471)	8 (5–10)
Philippines	2031	350 (308–392)	0 (0–0)	12071 (11461–12682)	7 (4–10)
Philippines	2032	353 (304–401)	0 (0–0)	12250 (11610–12890)	7 (4–10)
Philippines	2033	355 (301–410)	0 (0–0)	12429 (11760–13097)	7 (3–10)
Philippines	2034	358 (296–420)	0 (0–0)	12607 (11912–13303)	6 (3–10)
Philippines	2035	361 (292–430)	0 (0–0)	12786 (12064–13508)	6 (2–10)
Philippines	2036	364 (287–440)	0 (0–0)	12965 (12218–13712)	6 (1–11)
Poland	2022	625 (587–662)	1 (1–1)	20720 (19698–21742)	20 (18–23)
Poland	2023	602 (550–655)	1 (1–1)	20829 (19467–22191)	19 (16–22)
Poland	2024	580 (516–644)	1 (1–1)	20925 (19350–22501)	17 (13–21)
Poland	2025	558 (483–632)	1 (0–1)	21010 (19287–22734)	15 (11–19)
Poland	2026	535 (452–618)	1 (0–1)	21085 (19255–22916)	13 (9–18)
Poland	2027	513 (422–604)	0 (0–1)	21151 (19242–23060)	11 (6–17)
Poland	2028	491 (392–589)	0 (0–1)	21209 (19241–23177)	10 (4–15)
Poland	2029	468 (363–573)	0 (0–1)	21260 (19248–23273)	8 (2–14)
Poland	2030	446 (334–557)	0 (0–1)	21305 (19259–23352)	6 (-0–12)
Poland	2031	423 (306–541)	0 (-0–0)	21345 (19273–23417)	4 (-2–11)
Poland	2032	401 (278–524)	0 (-0–0)	21380 (19288–23472)	2 (-4–9)
Poland	2033	379 (250–508)	0 (-0–0)	21411 (19304–23518)	1 (-7–8)
Poland	2034	356 (222–490)	0 (-0–0)	21438 (19319–23557)	-1 (-9–6)
Poland	2035	334 (195–473)	-0 (-0–0)	21462 (19334–23590)	-3 (-11–5)
Poland	2036	312 (168–456)	-0 (-0–0)	21483 (19348–23618)	-5 (-13–3)
Portugal	2022	156 (146–166)	1 (1–1)	5086 (4769–5402)	17 (15–19)
Portugal	2023	149 (136–163)	1 (1–1)	5086 (4638–5533)	16 (13–18)
Portugal	2024	143 (126–160)	1 (0–1)	5086 (4538–5634)	14 (11–17)
Portugal	2025	136 (117–156)	1 (0–1)	5086 (4453–5718)	13 (9–16)
Portugal	2026	130 (108–152)	1 (0–1)	5086 (4378–5793)	11 (7–15)
Portugal	2027	123 (99–148)	0 (0–1)	5086 (4311–5861)	10 (5–14)
Portugal	2028	117 (91–143)	0 (0–1)	5086 (4249–5923)	8 (3–13)

Portugal	2029	110 (82–138)	0 (0–1)	5086 (4191–5980)	7 (2–12)
Portugal	2030	104 (74–133)	0 (-0–1)	5086 (4137–6035)	5 (-0–11)
Portugal	2031	97 (66–128)	0 (-0–1)	5086 (4085–6086)	4 (-2–10)
Portugal	2032	91 (58–123)	0 (-0–1)	5086 (4037–6135)	2 (-4–8)
Portugal	2033	84 (50–118)	0 (-0–1)	5086 (3990–6182)	1 (-5–7)
Portugal	2034	78 (42–113)	0 (-0–1)	5086 (3945–6226)	-1 (-7–6)
Portugal	2035	71 (34–108)	0 (-0–1)	5086 (3902–6269)	-2 (-9–5)
Portugal	2036	64 (26–103)	0 (-1–1)	5086 (3861–6311)	-4 (-11–3)
Puerto Rico	2022	17 (15–19)	0 (0–0)	779 (662–896)	5 (4–6)
Puerto Rico	2023	17 (14–19)	0 (0–0)	760 (618–902)	5 (4–6)
Puerto Rico	2024	16 (14–19)	0 (0–0)	741 (579–904)	4 (3–6)
Puerto Rico	2025	16 (13–19)	0 (0–0)	723 (541–904)	4 (3–5)
Puerto Rico	2026	15 (12–18)	0 (0–0)	704 (506–902)	4 (2–5)
Puerto Rico	2027	15 (11–18)	0 (0–0)	685 (472–899)	3 (1–5)
Puerto Rico	2028	14 (11–18)	0 (0–0)	667 (439–895)	3 (1–5)
Puerto Rico	2029	14 (10–18)	0 (0–0)	648 (406–890)	2 (0–4)
Puerto Rico	2030	14 (9–18)	0 (0–0)	629 (375–884)	2 (-0–4)
Puerto Rico	2031	13 (9–17)	0 (0–0)	611 (344–877)	2 (-1–4)
Puerto Rico	2032	13 (8–17)	0 (-0–0)	592 (314–870)	1 (-1–3)
Puerto Rico	2033	12 (8–17)	0 (-0–0)	573 (284–863)	1 (-2–3)
Puerto Rico	2034	12 (7–17)	0 (-0–0)	555 (254–855)	0 (-2–3)
Puerto Rico	2035	11 (6–17)	0 (-0–1)	536 (225–847)	-0 (-3–2)
Puerto Rico	2036	11 (6–16)	0 (-0–1)	517 (196–838)	-1 (-3–2)
Qatar	2022	3 (3–3)	0 (0–0)	148 (143–153)	7 (4–10)
Qatar	2023	3 (3–3)	0 (-0–1)	151 (143–160)	7 (1–13)
Qatar	2024	3 (3–3)	0 (-0–1)	155 (143–166)	7 (-1–15)
Qatar	2025	3 (3–3)	0 (-0–1)	158 (145–172)	7 (-3–17)
Qatar	2026	3 (3–3)	0 (-0–1)	162 (146–177)	7 (-4–18)
Qatar	2027	3 (3–4)	0 (-0–1)	165 (148–182)	7 (-5–20)
Qatar	2028	3 (3–4)	0 (-0–1)	169 (150–187)	7 (-7–21)
Qatar	2029	3 (3–4)	0 (-0–1)	172 (152–192)	7 (-8–22)
Qatar	2030	3 (3–4)	0 (-1–1)	175 (154–197)	7 (-9–23)
Qatar	2031	3 (3–4)	0 (-1–1)	179 (156–201)	7 (-10–24)

Qatar	2032	3 (3–4)	0 (-1–1)	182 (158–206)	7 (-10–25)
Qatar	2033	3 (3–4)	0 (-1–1)	186 (161–211)	7 (-11–26)
Qatar	2034	3 (3–4)	0 (-1–1)	189 (163–215)	7 (-12–26)
Qatar	2035	3 (3–4)	0 (-1–1)	193 (166–220)	7 (-13–27)
Qatar	2036	3 (3–4)	0 (-1–1)	196 (168–224)	7 (-14–28)
Republic of Korea	2022	1701 (1643–1759)	2 (2–2)	23803 (23065–24542)	34 (30–38)
Republic of Korea	2023	1735 (1630–1841)	2 (1–2)	24208 (22892–25523)	33 (26–40)
Republic of Korea	2024	1769 (1610–1929)	2 (1–2)	24566 (22646–26487)	32 (22–43)
Republic of Korea	2025	1803 (1585–2022)	2 (1–2)	24884 (22336–27433)	31 (17–46)
Republic of Korea	2026	1837 (1554–2121)	2 (1–2)	25167 (21976–28357)	31 (12–49)
Republic of Korea	2027	1871 (1518–2225)	2 (1–3)	25417 (21577–29258)	30 (7–52)
Republic of Korea	2028	1905 (1477–2333)	2 (0–3)	25640 (21148–30131)	29 (1–56)
Republic of Korea	2029	1939 (1432–2446)	2 (0–3)	25837 (20697–30978)	28 (-4–60)
Republic of Korea	2030	1973 (1382–2564)	2 (-0–3)	26013 (20228–31797)	27 (-11–65)
Republic of Korea	2031	2007 (1329–2685)	1 (-0–3)	26168 (19747–32589)	26 (-17–69)
Republic of Korea	2032	2041 (1271–2810)	1 (-1–4)	26306 (19257–33355)	25 (-24–74)
Republic of Korea	2033	2075 (1210–2940)	1 (-1–4)	26429 (18763–34094)	25 (-30–79)
Republic of Korea	2034	2109 (1145–3072)	1 (-1–4)	26537 (18266–34809)	24 (-38–85)
Republic of Korea	2035	2143 (1077–3209)	1 (-2–4)	26634 (17768–35499)	23 (-45–90)
Republic of Korea	2036	2177 (1005–3348)	1 (-2–5)	26719 (17272–36167)	22 (-52–96)
Republic of Moldova	2022	73 (64–83)	1 (1–1)	1652 (1485–1818)	32 (26–37)
Republic of Moldova	2023	73 (57–90)	1 (1–1)	1652 (1416–1887)	30 (21–40)
Republic of Moldova	2024	73 (53–94)	1 (1–1)	1652 (1363–1940)	29 (17–41)
Republic of Moldova	2025	73 (49–98)	1 (1–2)	1652 (1318–1985)	28 (13–42)
Republic of Moldova	2026	73 (46–101)	1 (0–2)	1652 (1279–2024)	26 (10–43)
Republic of Moldova	2027	73 (43–104)	1 (0–2)	1652 (1243–2060)	25 (7–43)
Republic of Moldova	2028	73 (40–107)	1 (0–2)	1652 (1211–2092)	24 (4–43)
Republic of Moldova	2029	73 (38–109)	1 (0–2)	1652 (1180–2123)	23 (2–43)
Republic of Moldova	2030	73 (35–111)	1 (0–2)	1652 (1152–2151)	21 (-1–43)
Republic of Moldova	2031	73 (33–114)	1 (-0–2)	1652 (1125–2178)	20 (-4–43)
Republic of Moldova	2032	73 (31–116)	1 (-0–2)	1652 (1099–2204)	19 (-6–43)
Republic of Moldova	2033	73 (29–118)	1 (-0–2)	1652 (1074–2229)	17 (-9–43)
Republic of Moldova	2034	73 (27–120)	1 (-0–2)	1652 (1051–2252)	16 (-11–43)

Republic of Moldova	2035	73 (25–121)	1 (-0–2)	1652 (1028–2275)	15 (-13–43)
Republic of Moldova	2036	73 (24–123)	1 (-0–2)	1652 (1006–2297)	13 (-16–42)
Romania	2022	351 (325–378)	1 (1–1)	8741 (8155–9327)	27 (24–29)
Romania	2023	347 (310–385)	1 (1–1)	8746 (7838–9653)	26 (23–29)
Romania	2024	343 (298–389)	1 (1–1)	8750 (7540–9961)	25 (22–29)
Romania	2025	340 (287–392)	1 (1–1)	8755 (7242–10268)	25 (20–29)
Romania	2026	336 (276–395)	1 (1–1)	8760 (6937–10583)	24 (19–29)
Romania	2027	332 (267–397)	1 (1–1)	8765 (6623–10906)	24 (18–29)
Romania	2028	328 (258–398)	1 (1–1)	8769 (6299–11239)	23 (17–29)
Romania	2029	324 (249–399)	1 (1–1)	8774 (5964–11584)	22 (16–29)
Romania	2030	320 (241–399)	1 (1–1)	8779 (5618–11939)	22 (15–28)
Romania	2031	316 (232–400)	1 (1–1)	8783 (5262–12305)	21 (14–28)
Romania	2032	312 (224–400)	1 (0–1)	8788 (4894–12683)	20 (13–28)
Romania	2033	308 (217–400)	1 (0–1)	8793 (4515–13070)	20 (12–27)
Romania	2034	304 (209–400)	1 (0–1)	8798 (4126–13469)	19 (11–27)
Romania	2035	301 (202–400)	1 (0–1)	8802 (3727–13878)	18 (10–27)
Romania	2036	297 (194–399)	1 (0–1)	8807 (3317–14297)	18 (9–27)
Russian Federation	2022	3126 (2804–3447)	1 (1–1)	85156 (78062–92250)	34 (29–39)
Russian Federation	2023	3038 (2500–3575)	1 (1–1)	85156 (75123–95188)	32 (24–40)
Russian Federation	2024	2950 (2261–3639)	1 (1–1)	85156 (72869–97443)	30 (20–41)
Russian Federation	2025	2862 (2049–3675)	1 (1–1)	85156 (70968–99344)	28 (16–41)
Russian Federation	2026	2774 (1854–3694)	1 (1–1)	85156 (69293–101018)	27 (13–41)
Russian Federation	2027	2686 (1670–3702)	1 (0–1)	85156 (67779–102532)	25 (9–40)
Russian Federation	2028	2598 (1494–3702)	1 (0–1)	85156 (66387–103925)	23 (6–40)
Russian Federation	2029	2510 (1325–3695)	1 (0–1)	85156 (65091–105220)	21 (3–39)
Russian Federation	2030	2422 (1161–3683)	1 (0–1)	85156 (63874–106438)	19 (-0–38)
Russian Federation	2031	2334 (1001–3666)	1 (0–1)	85156 (62723–107589)	17 (-3–37)
Russian Federation	2032	2246 (846–3646)	1 (-0–1)	85156 (61628–108684)	15 (-6–37)
Russian Federation	2033	2158 (693–3623)	1 (-0–1)	85156 (60582–109730)	13 (-9–36)
Russian Federation	2034	2070 (543–3597)	1 (-0–1)	85156 (59578–110733)	12 (-12–35)
Russian Federation	2035	1982 (395–3569)	0 (-0–1)	85156 (58613–111699)	10 (-14–34)
Russian Federation	2036	1894 (250–3539)	0 (-0–1)	85156 (57681–112630)	8 (-17–33)
Rwanda	2022	30 (28–31)	1 (1–1)	4294 (4055–4534)	12 (11–12)

Rwanda	2023	29 (26–32)	1 (0–1)	4177 (3616–4737)	12 (10–13)
Rwanda	2024	28 (23–33)	1 (0–1)	4030 (3254–4807)	12 (9–14)
Rwanda	2025	27 (22–33)	1 (0–1)	3894 (2983–4804)	12 (8–15)
Rwanda	2026	27 (20–33)	1 (0–1)	3779 (2791–4767)	12 (7–16)
Rwanda	2027	26 (20–33)	1 (0–1)	3690 (2659–4721)	12 (6–17)
Rwanda	2028	26 (19–33)	1 (0–1)	3623 (2568–4677)	12 (5–18)
Rwanda	2029	26 (19–32)	1 (0–1)	3573 (2506–4640)	12 (4–19)
Rwanda	2030	25 (18–32)	1 (0–1)	3537 (2464–4610)	12 (3–20)
Rwanda	2031	25 (18–32)	1 (0–1)	3512 (2436–4588)	12 (2–21)
Rwanda	2032	25 (18–32)	1 (0–1)	3494 (2416–4571)	12 (1–22)
Rwanda	2033	25 (18–32)	1 (0–1)	3481 (2403–4560)	12 (-0–23)
Rwanda	2034	25 (18–32)	1 (0–1)	3473 (2394–4551)	12 (-1–24)
Rwanda	2035	25 (17–32)	1 (0–1)	3467 (2388–4545)	12 (-2–25)
Rwanda	2036	25 (17–32)	1 (0–1)	3462 (2383–4541)	12 (-2–26)
Saint Kitts and Nevis	2022	0 (0–0)	0 (0–0)	10 (10–11)	7 (6–8)
Saint Kitts and Nevis	2023	0 (0–0)	0 (0–0)	11 (10–11)	7 (5–8)
Saint Kitts and Nevis	2024	0 (0–0)	0 (0–0)	11 (10–12)	6 (5–8)
Saint Kitts and Nevis	2025	0 (0–0)	0 (0–0)	11 (10–12)	6 (4–8)
Saint Kitts and Nevis	2026	0 (0–0)	0 (0–0)	12 (10–13)	6 (4–8)
Saint Kitts and Nevis	2027	0 (0–0)	0 (0–0)	12 (10–14)	5 (3–7)
Saint Kitts and Nevis	2028	0 (0–0)	0 (0–0)	12 (10–14)	5 (3–7)
Saint Kitts and Nevis	2029	0 (0–0)	0 (0–0)	12 (10–15)	5 (2–7)
Saint Kitts and Nevis	2030	0 (0–0)	0 (0–0)	13 (10–15)	4 (2–7)
Saint Kitts and Nevis	2031	0 (0–0)	0 (0–0)	13 (10–16)	4 (1–7)
Saint Kitts and Nevis	2032	0 (0–0)	0 (0–0)	13 (10–17)	4 (1–7)
Saint Kitts and Nevis	2033	0 (0–0)	0 (0–0)	14 (10–17)	3 (1–6)
Saint Kitts and Nevis	2034	0 (0–0)	0 (0–0)	14 (9–18)	3 (0–6)
Saint Kitts and Nevis	2035	0 (0–0)	0 (0–0)	14 (9–19)	3 (-0–6)
Saint Kitts and Nevis	2036	0 (0–0)	0 (-0–0)	14 (9–20)	3 (-1–6)
Saint Lucia	2022	1 (1–1)	1 (0–1)	53 (50–55)	12 (11–13)
Saint Lucia	2023	1 (1–1)	0 (0–1)	54 (50–57)	11 (10–13)
Saint Lucia	2024	1 (1–1)	0 (0–1)	54 (50–59)	11 (9–12)
Saint Lucia	2025	1 (1–1)	0 (0–1)	55 (50–60)	10 (8–12)

Saint Lucia	2026	1 (1–1)	0 (0–1)	56 (51–61)	9 (7–12)
Saint Lucia	2027	1 (1–1)	0 (0–1)	57 (51–63)	9 (6–11)
Saint Lucia	2028	1 (1–1)	0 (0–1)	58 (51–64)	8 (5–11)
Saint Lucia	2029	1 (1–1)	0 (0–1)	58 (52–65)	8 (5–11)
Saint Lucia	2030	1 (1–1)	0 (0–1)	59 (52–66)	7 (4–10)
Saint Lucia	2031	1 (1–1)	0 (-0–1)	60 (52–68)	6 (3–10)
Saint Lucia	2032	1 (1–1)	0 (-0–1)	61 (53–69)	6 (2–9)
Saint Lucia	2033	1 (1–1)	0 (-0–1)	62 (53–70)	5 (2–9)
Saint Lucia	2034	1 (1–1)	0 (-0–1)	62 (54–71)	5 (1–8)
Saint Lucia	2035	1 (1–1)	0 (-0–1)	63 (54–72)	4 (0–8)
Saint Lucia	2036	1 (1–1)	0 (-0–1)	64 (55–73)	3 (-1–7)
Saint Vincent and the Grenadines	2022	1 (1–1)	0 (0–0)	16 (15–17)	11 (10–12)
Saint Vincent and the Grenadines	2023	1 (1–1)	0 (0–0)	16 (15–18)	11 (9–12)
Saint Vincent and the Grenadines	2024	1 (1–1)	0 (0–0)	17 (15–18)	10 (9–12)
Saint Vincent and the Grenadines	2025	1 (1–1)	0 (0–0)	17 (14–19)	10 (8–12)
Saint Vincent and the Grenadines	2026	1 (1–1)	0 (0–0)	17 (14–20)	10 (8–12)
Saint Vincent and the Grenadines	2027	1 (1–1)	0 (0–0)	17 (13–22)	9 (7–12)
Saint Vincent and the Grenadines	2028	1 (1–1)	0 (0–0)	18 (13–23)	9 (7–12)
Saint Vincent and the Grenadines	2029	1 (1–1)	0 (0–0)	18 (12–24)	9 (6–12)
Saint Vincent and the Grenadines	2030	1 (1–1)	0 (0–0)	18 (12–25)	9 (6–12)
Saint Vincent and the Grenadines	2031	1 (1–1)	0 (0–0)	19 (11–26)	8 (5–11)
Saint Vincent and the Grenadines	2032	1 (1–1)	0 (0–0)	19 (10–28)	8 (5–11)
Saint Vincent and the Grenadines	2033	1 (1–1)	0 (0–0)	19 (9–29)	8 (4–11)
Saint Vincent and the Grenadines	2034	1 (1–1)	0 (0–0)	19 (9–30)	7 (4–11)
Saint Vincent and the Grenadines	2035	1 (1–1)	0 (0–0)	20 (8–32)	7 (3–11)
Saint Vincent and the Grenadines	2036	1 (0–1)	0 (0–0)	20 (7–33)	7 (3–11)
Samoa	2022	1 (1–1)	1 (1–1)	13 (12–13)	22 (22–23)
Samoa	2023	1 (1–1)	1 (1–1)	13 (13–13)	22 (22–23)
Samoa	2024	1 (1–1)	1 (1–1)	13 (13–13)	22 (21–23)
Samoa	2025	1 (1–1)	1 (1–1)	13 (13–14)	22 (21–23)
Samoa	2026	1 (1–1)	1 (1–1)	13 (13–14)	22 (20–24)
Samoa	2027	1 (1–1)	1 (1–1)	14 (13–15)	22 (20–24)
Samoa	2028	1 (1–1)	1 (1–1)	14 (13–15)	22 (19–24)

Samoa	2029	1 (1–1)	1 (1–1)	14 (13–16)	22 (18–25)
Samoa	2030	1 (1–1)	1 (1–1)	14 (13–16)	21 (18–25)
Samoa	2031	1 (1–1)	1 (1–1)	15 (13–17)	21 (17–26)
Samoa	2032	1 (1–1)	1 (1–1)	15 (13–17)	21 (16–26)
Samoa	2033	1 (1–1)	1 (1–1)	15 (13–18)	21 (15–27)
Samoa	2034	1 (1–1)	1 (1–1)	15 (13–18)	21 (14–27)
Samoa	2035	1 (1–1)	1 (1–1)	16 (12–19)	21 (14–28)
Samoa	2036	1 (1–1)	1 (1–1)	16 (12–19)	21 (13–29)
San Marino	2022	1 (1–1)	1 (1–1)	5 (4–6)	19 (15–23)
San Marino	2023	1 (1–1)	1 (1–1)	5 (4–7)	17 (12–22)
San Marino	2024	1 (0–1)	1 (0–1)	5 (4–7)	16 (9–22)
San Marino	2025	1 (0–1)	1 (0–1)	5 (3–7)	14 (6–21)
San Marino	2026	1 (0–1)	1 (0–1)	5 (3–8)	12 (4–20)
San Marino	2027	1 (0–1)	0 (0–1)	5 (3–8)	10 (1–19)
San Marino	2028	1 (0–1)	0 (-0–1)	5 (3–8)	8 (-1–18)
San Marino	2029	1 (0–1)	0 (-0–1)	5 (3–8)	7 (-4–17)
San Marino	2030	1 (0–1)	0 (-0–1)	5 (2–8)	5 (-6–16)
San Marino	2031	1 (0–1)	0 (-0–1)	5 (2–8)	3 (-9–14)
San Marino	2032	1 (0–1)	0 (-1–1)	5 (2–9)	1 (-11–13)
San Marino	2033	1 (0–1)	-0 (-1–1)	5 (2–9)	-1 (-13–12)
San Marino	2034	1 (0–1)	-0 (-1–1)	5 (2–9)	-2 (-16–11)
San Marino	2035	1 (0–1)	-0 (-1–1)	5 (2–9)	-4 (-18–9)
San Marino	2036	1 (0–1)	-0 (-1–0)	5 (2–9)	-6 (-20–8)
Sao Tome and Principe	2022	0 (0–0)	0 (0–0)	14 (14–14)	7 (7–8)
Sao Tome and Principe	2023	0 (0–0)	0 (0–0)	15 (14–15)	7 (7–8)
Sao Tome and Principe	2024	0 (0–0)	0 (0–0)	15 (15–16)	7 (6–8)
Sao Tome and Principe	2025	0 (0–0)	0 (0–0)	16 (15–17)	7 (6–8)
Sao Tome and Principe	2026	0 (0–0)	0 (0–0)	17 (15–18)	7 (5–8)
Sao Tome and Principe	2027	0 (0–0)	0 (0–0)	17 (16–19)	7 (5–8)
Sao Tome and Principe	2028	0 (0–0)	0 (0–0)	18 (16–20)	6 (4–9)
Sao Tome and Principe	2029	0 (0–0)	0 (0–0)	18 (16–21)	6 (3–9)
Sao Tome and Principe	2030	0 (0–0)	0 (0–0)	19 (16–22)	6 (3–9)
Sao Tome and Principe	2031	0 (0–0)	0 (0–0)	19 (16–23)	6 (2–10)

Sao Tome and Principe	2032	0 (0–0)	0 (0–0)	20 (16–24)	6 (1–10)
Sao Tome and Principe	2033	0 (0–0)	0 (0–0)	21 (17–25)	6 (1–11)
Sao Tome and Principe	2034	0 (0–0)	0 (-0–0)	21 (17–26)	6 (0–11)
Sao Tome and Principe	2035	0 (0–0)	0 (-0–0)	22 (17–27)	5 (-1–11)
Sao Tome and Principe	2036	0 (0–0)	0 (-0–0)	22 (17–28)	5 (-2–12)
Saudi Arabia	2022	44 (44–45)	0 (0–0)	2407 (2374–2440)	6 (5–6)
Saudi Arabia	2023	45 (44–47)	0 (0–0)	2500 (2426–2573)	5 (5–6)
Saudi Arabia	2024	46 (45–48)	0 (0–0)	2592 (2470–2715)	5 (5–6)
Saudi Arabia	2025	48 (45–50)	0 (0–0)	2684 (2505–2864)	5 (4–6)
Saudi Arabia	2026	49 (45–52)	0 (0–0)	2777 (2534–3020)	5 (4–6)
Saudi Arabia	2027	50 (45–55)	0 (0–0)	2869 (2557–3182)	5 (3–6)
Saudi Arabia	2028	51 (45–57)	0 (0–0)	2962 (2574–3349)	5 (3–6)
Saudi Arabia	2029	52 (45–59)	0 (0–0)	3054 (2587–3522)	4 (3–6)
Saudi Arabia	2030	53 (44–62)	0 (0–0)	3147 (2594–3699)	4 (2–6)
Saudi Arabia	2031	54 (44–64)	0 (0–0)	3239 (2597–3882)	4 (1–7)
Saudi Arabia	2032	55 (44–67)	0 (0–0)	3332 (2595–4068)	4 (1–7)
Saudi Arabia	2033	56 (43–69)	0 (0–0)	3424 (2589–4259)	4 (0–7)
Saudi Arabia	2034	57 (43–72)	0 (0–0)	3516 (2580–4453)	4 (-0–7)
Saudi Arabia	2035	58 (42–75)	0 (-0–0)	3609 (2566–4652)	3 (-1–7)
Saudi Arabia	2036	59 (41–77)	0 (-0–0)	3701 (2549–4854)	3 (-1–8)
Senegal	2022	19 (18–20)	0 (0–0)	1315 (1273–1357)	6 (6–7)
Senegal	2023	19 (18–20)	0 (0–0)	1360 (1292–1427)	6 (5–7)
Senegal	2024	19 (18–21)	0 (0–0)	1404 (1312–1497)	6 (5–7)
Senegal	2025	19 (18–21)	0 (0–0)	1449 (1331–1567)	6 (5–7)
Senegal	2026	20 (18–21)	0 (0–0)	1494 (1348–1639)	6 (4–8)
Senegal	2027	20 (18–22)	0 (0–0)	1538 (1365–1712)	6 (4–8)
Senegal	2028	20 (18–22)	0 (0–0)	1583 (1380–1786)	6 (4–8)
Senegal	2029	20 (18–22)	0 (0–0)	1628 (1394–1862)	6 (3–8)
Senegal	2030	20 (18–23)	0 (0–0)	1672 (1406–1939)	6 (3–8)
Senegal	2031	20 (18–23)	0 (0–0)	1717 (1417–2017)	5 (2–8)
Senegal	2032	20 (18–23)	0 (0–0)	1762 (1427–2096)	5 (2–9)
Senegal	2033	21 (18–23)	0 (0–0)	1806 (1436–2177)	5 (2–9)
Senegal	2034	21 (18–24)	0 (0–0)	1851 (1444–2258)	5 (1–9)

Senegal	2035	21 (18–24)	0 (0–0)	1896 (1451–2341)	5 (1–9)
Senegal	2036	21 (18–24)	0 (0–0)	1941 (1456–2425)	5 (0–9)
Serbia	2022	142 (129–155)	1 (1–1)	3595 (3334–3857)	22 (19–24)
Serbia	2023	139 (118–160)	1 (1–1)	3554 (3155–3953)	21 (17–25)
Serbia	2024	135 (106–164)	1 (1–1)	3512 (2988–4037)	20 (15–25)
Serbia	2025	132 (95–170)	1 (1–1)	3471 (2823–4118)	19 (13–26)
Serbia	2026	129 (83–175)	1 (0–1)	3429 (2658–4201)	19 (11–26)
Serbia	2027	126 (71–181)	1 (0–1)	3388 (2490–4285)	18 (9–27)
Serbia	2028	123 (58–187)	1 (0–1)	3346 (2320–4372)	17 (6–28)
Serbia	2029	119 (44–194)	1 (0–1)	3304 (2146–4463)	16 (4–29)
Serbia	2030	116 (31–202)	1 (0–1)	3263 (1969–4556)	16 (2–29)
Serbia	2031	113 (17–209)	1 (0–1)	3221 (1788–4654)	15 (-1–30)
Serbia	2032	110 (2–217)	1 (-0–1)	3180 (1604–4755)	14 (-3–31)
Serbia	2033	106 (-13–226)	1 (-0–1)	3138 (1417–4859)	13 (-5–32)
Serbia	2034	103 (-28–235)	1 (-0–1)	3096 (1225–4968)	13 (-8–33)
Serbia	2035	100 (-44–244)	0 (-0–1)	3055 (1030–5079)	12 (-11–34)
Serbia	2036	97 (-60–253)	0 (-0–1)	3013 (832–5195)	11 (-13–35)
Seychelles	2022	1 (1–1)	1 (1–1)	56 (53–58)	14 (12–15)
Seychelles	2023	1 (1–1)	1 (0–1)	56 (51–61)	13 (11–15)
Seychelles	2024	1 (1–1)	1 (0–1)	57 (51–63)	13 (10–15)
Seychelles	2025	1 (1–1)	1 (0–1)	58 (50–65)	12 (9–15)
Seychelles	2026	1 (1–1)	0 (0–1)	58 (50–67)	12 (8–16)
Seychelles	2027	1 (1–1)	0 (0–1)	59 (50–69)	11 (7–15)
Seychelles	2028	1 (1–1)	0 (0–1)	60 (49–71)	11 (6–15)
Seychelles	2029	1 (1–1)	0 (0–1)	61 (49–72)	10 (5–15)
Seychelles	2030	1 (1–1)	0 (0–1)	61 (49–74)	10 (4–15)
Seychelles	2031	1 (1–1)	0 (0–1)	62 (49–75)	9 (3–15)
Seychelles	2032	1 (1–1)	0 (0–1)	63 (49–76)	8 (2–15)
Seychelles	2033	1 (1–1)	0 (0–1)	64 (50–78)	8 (1–15)
Seychelles	2034	1 (1–1)	0 (0–1)	64 (50–79)	7 (1–14)
Seychelles	2035	1 (1–1)	0 (0–1)	65 (50–80)	7 (-0–14)
Seychelles	2036	1 (1–1)	0 (0–1)	66 (50–82)	6 (-1–14)
Sierra Leone	2022	12 (12–12)	0 (0–0)	719 (709–728)	8 (8–8)

Sierra Leone	2023	12 (12–13)	0 (0–0)	742 (721–763)	8 (7–9)
Sierra Leone	2024	12 (11–13)	0 (0–0)	766 (731–801)	8 (7–9)
Sierra Leone	2025	12 (11–14)	0 (0–0)	789 (738–840)	8 (7–9)
Sierra Leone	2026	13 (11–14)	0 (0–0)	812 (743–882)	8 (6–9)
Sierra Leone	2027	13 (11–15)	0 (0–0)	836 (746–925)	8 (6–10)
Sierra Leone	2028	13 (10–15)	0 (0–0)	859 (748–970)	7 (5–10)
Sierra Leone	2029	13 (10–16)	0 (0–0)	883 (749–1017)	7 (5–10)
Sierra Leone	2030	13 (10–17)	0 (0–0)	906 (748–1065)	7 (4–11)
Sierra Leone	2031	13 (9–17)	0 (0–0)	930 (746–1114)	7 (4–11)
Sierra Leone	2032	13 (9–18)	0 (0–0)	953 (742–1164)	7 (3–11)
Sierra Leone	2033	14 (8–19)	0 (0–0)	976 (737–1216)	7 (3–12)
Sierra Leone	2034	14 (8–20)	0 (0–0)	1000 (731–1268)	7 (2–12)
Sierra Leone	2035	14 (7–21)	0 (0–0)	1023 (725–1322)	7 (2–12)
Sierra Leone	2036	14 (7–21)	0 (0–0)	1047 (716–1377)	7 (1–13)
Singapore	2022	19 (17–20)	0 (0–0)	826 (696–956)	4 (3–5)
Singapore	2023	18 (15–21)	0 (0–0)	823 (670–977)	4 (2–6)
Singapore	2024	18 (13–22)	0 (0–0)	822 (660–983)	4 (1–7)
Singapore	2025	17 (11–24)	0 (-0–0)	821 (656–985)	3 (-1–8)
Singapore	2026	17 (9–25)	0 (-0–0)	820 (654–985)	3 (-3–9)
Singapore	2027	17 (6–27)	0 (-0–0)	819 (653–985)	2 (-5–10)
Singapore	2028	16 (3–29)	0 (-0–0)	819 (653–985)	2 (-7–11)
Singapore	2029	16 (0–31)	0 (-0–1)	819 (653–985)	2 (-9–12)
Singapore	2030	15 (-3–34)	0 (-0–1)	819 (652–985)	1 (-11–14)
Singapore	2031	15 (-6–36)	0 (-1–1)	819 (652–985)	1 (-14–15)
Singapore	2032	15 (-10–39)	0 (-1–1)	819 (652–985)	1 (-16–17)
Singapore	2033	14 (-13–42)	0 (-1–1)	819 (652–985)	0 (-19–19)
Singapore	2034	14 (-17–45)	0 (-1–1)	819 (652–985)	-0 (-21–21)
Singapore	2035	13 (-21–48)	-0 (-1–1)	819 (652–985)	-1 (-24–23)
Singapore	2036	13 (-25–51)	-0 (-1–1)	819 (652–985)	-1 (-27–25)
Slovakia	2022	69 (65–73)	1 (1–1)	2814 (2683–2944)	18 (16–19)
Slovakia	2023	69 (63–75)	1 (1–1)	2836 (2680–2991)	17 (15–20)
Slovakia	2024	69 (60–77)	1 (1–1)	2850 (2686–3014)	17 (13–21)
Slovakia	2025	69 (58–79)	1 (0–1)	2859 (2691–3026)	16 (12–21)

Slovakia	2026	68 (55–82)	1 (0–1)	2864 (2695–3033)	16 (10–22)
Slovakia	2027	68 (53–84)	1 (0–1)	2868 (2698–3037)	16 (8–23)
Slovakia	2028	68 (50–86)	1 (0–1)	2870 (2700–3040)	15 (7–24)
Slovakia	2029	68 (47–89)	1 (0–1)	2872 (2702–3042)	15 (5–25)
Slovakia	2030	68 (44–92)	1 (0–1)	2873 (2703–3043)	15 (3–26)
Slovakia	2031	67 (40–95)	1 (0–1)	2873 (2703–3043)	14 (1–27)
Slovakia	2032	67 (37–97)	1 (0–1)	2874 (2704–3044)	14 (-1–28)
Slovakia	2033	67 (34–100)	1 (-0–1)	2874 (2704–3044)	13 (-3–30)
Slovakia	2034	67 (30–104)	1 (-0–1)	2874 (2704–3044)	13 (-5–31)
Slovakia	2035	67 (27–107)	1 (-0–1)	2874 (2704–3044)	13 (-7–32)
Slovakia	2036	67 (23–110)	0 (-0–1)	2874 (2704–3044)	12 (-9–34)
Slovenia	2022	32 (29–35)	1 (1–1)	985 (893–1078)	16 (14–19)
Slovenia	2023	31 (27–35)	1 (1–1)	985 (855–1116)	15 (11–18)
Slovenia	2024	30 (25–35)	1 (0–1)	985 (825–1145)	13 (9–18)
Slovenia	2025	29 (24–35)	1 (0–1)	985 (800–1170)	12 (7–17)
Slovenia	2026	29 (22–35)	0 (0–1)	985 (779–1192)	11 (5–16)
Slovenia	2027	28 (21–35)	0 (0–1)	985 (759–1212)	9 (3–15)
Slovenia	2028	27 (20–34)	0 (0–1)	985 (741–1230)	8 (1–14)
Slovenia	2029	26 (18–34)	0 (0–0)	985 (724–1247)	7 (-0–14)
Slovenia	2030	25 (17–34)	0 (0–0)	985 (708–1263)	5 (-2–13)
Slovenia	2031	25 (16–33)	0 (0–0)	985 (693–1278)	4 (-4–12)
Slovenia	2032	24 (15–33)	0 (-0–0)	985 (679–1292)	2 (-6–11)
Slovenia	2033	23 (13–33)	0 (-0–0)	985 (665–1306)	1 (-8–10)
Slovenia	2034	22 (12–32)	0 (-0–0)	985 (652–1319)	-0 (-9–8)
Slovenia	2035	21 (11–32)	0 (-0–0)	985 (639–1331)	-2 (-11–7)
Slovenia	2036	21 (10–31)	-0 (-0–0)	985 (627–1343)	-3 (-13–6)
Solomon Islands	2022	6 (6–7)	2 (2–2)	80 (79–82)	48 (47–50)
Solomon Islands	2023	7 (6–7)	2 (2–2)	82 (80–85)	48 (45–51)
Solomon Islands	2024	7 (6–7)	2 (2–2)	84 (81–88)	47 (43–51)
Solomon Islands	2025	7 (6–7)	2 (1–2)	86 (82–91)	47 (42–52)
Solomon Islands	2026	7 (6–7)	2 (1–2)	88 (83–94)	46 (40–53)
Solomon Islands	2027	7 (6–8)	2 (1–2)	90 (84–97)	46 (38–54)
Solomon Islands	2028	7 (6–8)	2 (1–2)	92 (85–100)	46 (36–55)

Solomon Islands	2029	7 (6–8)	2 (1–2)	94 (85–104)	45 (34–56)
Solomon Islands	2030	7 (6–9)	2 (1–2)	96 (86–107)	45 (32–57)
Solomon Islands	2031	8 (6–9)	2 (1–2)	98 (87–110)	44 (30–58)
Solomon Islands	2032	8 (6–9)	1 (1–2)	100 (88–113)	44 (28–60)
Solomon Islands	2033	8 (6–9)	1 (1–2)	103 (88–117)	43 (25–61)
Solomon Islands	2034	8 (6–10)	1 (1–2)	105 (89–120)	43 (23–63)
Solomon Islands	2035	8 (6–10)	1 (1–2)	107 (90–124)	42 (21–64)
Solomon Islands	2036	8 (6–10)	1 (1–2)	109 (90–127)	42 (18–66)
Somalia	2022	24 (24–25)	0 (0–0)	2854 (2823–2885)	10 (10–10)
Somalia	2023	25 (24–25)	0 (0–0)	2872 (2795–2949)	10 (10–10)
Somalia	2024	25 (24–26)	0 (0–0)	2893 (2768–3017)	10 (10–10)
Somalia	2025	25 (24–26)	0 (0–0)	2916 (2746–3085)	10 (9–10)
Somalia	2026	26 (24–27)	0 (0–0)	2940 (2728–3152)	9 (9–10)
Somalia	2027	26 (25–27)	0 (0–0)	2966 (2714–3217)	9 (9–10)
Somalia	2028	26 (25–28)	0 (0–0)	2992 (2703–3280)	9 (8–10)
Somalia	2029	27 (25–28)	0 (0–0)	3018 (2695–3341)	9 (8–10)
Somalia	2030	27 (25–29)	0 (0–0)	3045 (2690–3399)	9 (8–10)
Somalia	2031	27 (25–29)	0 (0–0)	3071 (2687–3456)	9 (8–10)
Somalia	2032	28 (26–29)	0 (0–0)	3098 (2686–3511)	8 (7–9)
Somalia	2033	28 (26–30)	0 (0–0)	3125 (2686–3564)	8 (7–9)
Somalia	2034	28 (26–30)	0 (0–0)	3153 (2688–3617)	8 (7–9)
Somalia	2035	28 (26–31)	0 (0–0)	3180 (2692–3668)	8 (7–9)
Somalia	2036	29 (26–31)	0 (0–0)	3207 (2696–3718)	8 (6–9)
South Africa	2022	153 (143–164)	0 (0–0)	26901 (24741–29062)	9 (8–10)
South Africa	2023	155 (137–172)	0 (0–0)	27003 (23215–30790)	8 (7–10)
South Africa	2024	156 (135–177)	0 (0–0)	27077 (21999–32154)	8 (6–10)
South Africa	2025	157 (135–179)	0 (0–0)	27131 (21084–33179)	8 (5–10)
South Africa	2026	157 (134–180)	0 (0–0)	27173 (20416–33929)	7 (5–10)
South Africa	2027	158 (134–181)	0 (0–0)	27204 (19941–34467)	7 (4–10)
South Africa	2028	158 (134–182)	0 (0–0)	27229 (19609–34849)	7 (4–10)
South Africa	2029	158 (134–182)	0 (0–0)	27248 (19379–35117)	6 (3–10)
South Africa	2030	159 (135–182)	0 (0–0)	27263 (19222–35305)	6 (3–9)
South Africa	2031	159 (135–183)	0 (0–0)	27275 (19116–35435)	6 (2–9)

South Africa	2032	159 (135–183)	0 (0–0)	27285 (19044–35526)	5 (2–9)
South Africa	2033	159 (135–183)	0 (-0–0)	27293 (18997–35588)	5 (1–9)
South Africa	2034	159 (135–183)	0 (-0–0)	27299 (18966–35632)	5 (1–9)
South Africa	2035	159 (135–183)	0 (-0–0)	27304 (18946–35662)	4 (0–9)
South Africa	2036	159 (135–183)	0 (-0–0)	27308 (18933–35684)	4 (-0–8)
South Sudan	2022	13 (13–13)	0 (0–0)	2043 (2006–2080)	9 (8–9)
South Sudan	2023	13 (13–14)	0 (0–0)	2082 (2012–2152)	9 (8–9)
South Sudan	2024	13 (12–14)	0 (0–0)	2118 (2012–2224)	9 (8–9)
South Sudan	2025	14 (12–15)	0 (0–0)	2151 (2006–2296)	9 (8–10)
South Sudan	2026	14 (12–15)	0 (0–0)	2181 (1995–2367)	9 (7–10)
South Sudan	2027	14 (12–16)	0 (0–0)	2208 (1980–2436)	9 (7–10)
South Sudan	2028	14 (11–17)	0 (0–0)	2233 (1962–2503)	9 (7–10)
South Sudan	2029	14 (11–18)	0 (0–0)	2254 (1941–2568)	9 (6–11)
South Sudan	2030	14 (10–18)	0 (0–0)	2274 (1918–2630)	9 (6–11)
South Sudan	2031	14 (10–19)	0 (0–0)	2290 (1892–2688)	9 (6–11)
South Sudan	2032	15 (9–20)	0 (0–0)	2304 (1865–2744)	9 (5–12)
South Sudan	2033	15 (9–21)	0 (0–0)	2316 (1837–2796)	9 (5–12)
South Sudan	2034	15 (8–22)	0 (0–0)	2326 (1807–2845)	8 (4–13)
South Sudan	2035	15 (7–23)	0 (0–0)	2334 (1777–2891)	8 (4–13)
South Sudan	2036	15 (7–24)	0 (0–0)	2340 (1746–2934)	8 (3–14)
Spain	2022	639 (617–661)	1 (1–1)	20838 (19902–21774)	15 (14–15)
Spain	2023	614 (582–645)	1 (1–1)	20529 (19205–21852)	14 (12–15)
Spain	2024	589 (550–627)	1 (0–1)	20219 (18598–21840)	13 (11–15)
Spain	2025	564 (519–608)	1 (0–1)	19910 (18038–21782)	12 (9–15)
Spain	2026	539 (489–588)	0 (0–1)	19600 (17507–21693)	11 (7–15)
Spain	2027	514 (459–568)	0 (0–1)	19291 (16998–21583)	10 (6–15)
Spain	2028	489 (430–547)	0 (0–1)	18981 (16505–21458)	10 (4–15)
Spain	2029	464 (401–526)	0 (0–1)	18672 (16024–21319)	9 (2–16)
Spain	2030	438 (372–505)	0 (0–1)	18362 (15554–21170)	8 (0–16)
Spain	2031	413 (343–483)	0 (-0–1)	18053 (15093–21013)	7 (-2–16)
Spain	2032	388 (315–462)	0 (-0–1)	17743 (14639–20848)	6 (-4–16)
Spain	2033	363 (287–440)	0 (-0–1)	17434 (14191–20676)	6 (-6–17)
Spain	2034	338 (259–418)	0 (-0–1)	17124 (13749–20499)	5 (-8–17)

Spain	2035	313 (231–396)	0 (-0–1)	16815 (13312–20317)	4 (-10–18)
Spain	2036	288 (203–374)	0 (-1–1)	16505 (12880–20130)	3 (-12–18)
Sri Lanka	2022	49 (44–53)	0 (0–0)	8106 (7603–8609)	4 (3–5)
Sri Lanka	2023	49 (42–55)	0 (0–0)	8196 (7485–8908)	4 (2–5)
Sri Lanka	2024	49 (41–57)	0 (0–0)	8286 (7414–9157)	3 (2–5)
Sri Lanka	2025	49 (39–58)	0 (0–0)	8376 (7369–9382)	3 (1–5)
Sri Lanka	2026	49 (38–59)	0 (0–0)	8466 (7341–9591)	3 (1–4)
Sri Lanka	2027	49 (37–60)	0 (0–0)	8555 (7323–9788)	2 (0–4)
Sri Lanka	2028	49 (36–61)	0 (-0–0)	8645 (7314–9976)	2 (-0–4)
Sri Lanka	2029	49 (35–62)	0 (-0–0)	8735 (7312–10158)	1 (-1–4)
Sri Lanka	2030	49 (35–63)	0 (-0–0)	8825 (7315–10334)	1 (-1–4)
Sri Lanka	2031	49 (34–64)	0 (-0–0)	8915 (7324–10506)	1 (-2–3)
Sri Lanka	2032	49 (33–64)	0 (-0–0)	9005 (7336–10673)	0 (-3–3)
Sri Lanka	2033	49 (32–65)	-0 (-0–0)	9094 (7351–10837)	-0 (-3–3)
Sri Lanka	2034	49 (32–66)	-0 (-0–0)	9184 (7370–10998)	-0 (-4–3)
Sri Lanka	2035	49 (31–66)	-0 (-0–0)	9274 (7391–11156)	-1 (-4–2)
Sri Lanka	2036	49 (30–67)	-0 (-0–0)	9364 (7415–11312)	-1 (-4–2)
Sudan	2022	207 (205–208)	1 (1–1)	6495 (6458–6533)	26 (25–26)
Sudan	2023	208 (205–212)	1 (1–1)	6634 (6537–6731)	25 (25–26)
Sudan	2024	210 (204–216)	1 (1–1)	6773 (6602–6944)	25 (24–26)
Sudan	2025	212 (203–221)	1 (1–1)	6912 (6654–7170)	25 (23–26)
Sudan	2026	214 (202–225)	1 (1–1)	7051 (6695–7406)	25 (23–27)
Sudan	2027	215 (201–230)	1 (1–1)	7190 (6727–7653)	24 (22–27)
Sudan	2028	217 (199–235)	1 (1–1)	7329 (6750–7908)	24 (21–27)
Sudan	2029	218 (197–240)	1 (1–1)	7468 (6764–8172)	24 (20–28)
Sudan	2030	220 (195–245)	1 (1–1)	7607 (6770–8443)	24 (19–28)
Sudan	2031	221 (193–250)	1 (1–1)	7746 (6769–8722)	23 (18–29)
Sudan	2032	223 (190–255)	1 (1–1)	7884 (6761–9007)	23 (17–29)
Sudan	2033	224 (188–260)	1 (1–1)	8023 (6747–9300)	23 (16–30)
Sudan	2034	225 (185–265)	1 (1–1)	8162 (6726–9599)	23 (15–30)
Sudan	2035	226 (182–270)	1 (1–1)	8301 (6698–9904)	22 (14–31)
Sudan	2036	227 (179–276)	1 (1–1)	8440 (6665–10215)	22 (13–31)
Suriname	2022	3 (3–3)	0 (0–1)	74 (70–78)	12 (10–13)

Suriname	2023	3 (2–3)	0 (0–1)	76 (67–84)	11 (8–15)
Suriname	2024	3 (2–4)	0 (0–1)	80 (68–91)	11 (7–15)
Suriname	2025	3 (2–4)	0 (0–1)	82 (70–95)	11 (6–16)
Suriname	2026	3 (2–4)	0 (0–1)	83 (70–97)	11 (5–16)
Suriname	2027	3 (2–4)	0 (0–1)	84 (69–98)	10 (5–16)
Suriname	2028	3 (2–4)	0 (0–1)	85 (69–100)	10 (4–15)
Suriname	2029	3 (2–4)	0 (0–1)	86 (70–102)	9 (3–15)
Suriname	2030	3 (2–4)	0 (0–1)	87 (70–105)	9 (2–15)
Suriname	2031	3 (2–4)	0 (0–1)	89 (71–107)	8 (1–15)
Suriname	2032	3 (2–4)	0 (0–1)	90 (71–109)	8 (1–15)
Suriname	2033	3 (2–4)	0 (-0–1)	91 (71–111)	8 (0–15)
Suriname	2034	3 (2–4)	0 (-0–1)	92 (72–113)	7 (-1–15)
Suriname	2035	3 (2–4)	0 (-0–1)	94 (72–115)	7 (-1–15)
Suriname	2036	3 (2–4)	0 (-0–1)	95 (73–117)	6 (-2–15)
Sweden	2022	82 (75–88)	0 (0–0)	4532 (4288–4776)	7 (6–7)
Sweden	2023	81 (70–91)	0 (0–0)	4532 (4109–4956)	6 (5–8)
Sweden	2024	80 (65–94)	0 (0–0)	4532 (3985–5079)	6 (4–8)
Sweden	2025	78 (59–98)	0 (0–0)	4532 (3885–5179)	6 (4–8)
Sweden	2026	77 (53–102)	0 (0–0)	4532 (3799–5266)	5 (3–8)
Sweden	2027	76 (47–106)	0 (0–0)	4532 (3721–5343)	5 (2–9)
Sweden	2028	75 (41–110)	0 (0–0)	4532 (3650–5414)	5 (1–9)
Sweden	2029	74 (34–114)	0 (0–0)	4532 (3585–5480)	4 (-0–9)
Sweden	2030	73 (27–119)	0 (-0–0)	4532 (3524–5541)	4 (-1–10)
Sweden	2031	72 (20–124)	0 (-0–0)	4532 (3466–5598)	4 (-2–10)
Sweden	2032	71 (12–130)	0 (-0–1)	4532 (3411–5653)	4 (-3–10)
Sweden	2033	70 (5–135)	0 (-0–1)	4532 (3359–5705)	3 (-4–11)
Sweden	2034	69 (-3–141)	0 (-0–1)	4532 (3309–5755)	3 (-5–11)
Sweden	2035	68 (-11–147)	0 (-0–1)	4532 (3261–5803)	3 (-7–12)
Sweden	2036	67 (-19–153)	0 (-0–1)	4532 (3215–5849)	2 (-8–12)
Switzerland	2022	85 (80–89)	0 (0–0)	4369 (4081–4657)	9 (8–10)
Switzerland	2023	82 (75–90)	0 (0–0)	4369 (3962–4777)	8 (7–10)
Switzerland	2024	80 (68–92)	0 (0–0)	4369 (3871–4868)	8 (6–10)
Switzerland	2025	78 (62–94)	0 (0–0)	4369 (3793–4945)	8 (4–11)

Switzerland	2026	76 (55–97)	0 (0–1)	4369 (3725–5013)	7 (3–11)
Switzerland	2027	74 (47–100)	0 (0–1)	4369 (3664–5075)	7 (2–12)
Switzerland	2028	72 (39–104)	0 (0–1)	4369 (3607–5131)	6 (0–13)
Switzerland	2029	69 (31–107)	0 (-0–1)	4369 (3555–5184)	6 (-1–13)
Switzerland	2030	67 (23–112)	0 (-0–1)	4369 (3505–5233)	6 (-3–14)
Switzerland	2031	65 (14–116)	0 (-0–1)	4369 (3459–5280)	5 (-4–15)
Switzerland	2032	63 (5–121)	0 (-0–1)	4369 (3414–5324)	5 (-6–16)
Switzerland	2033	61 (-5–126)	0 (-0–1)	4369 (3372–5367)	5 (-8–17)
Switzerland	2034	59 (-14–131)	0 (-0–1)	4369 (3331–5408)	4 (-10–18)
Switzerland	2035	56 (-24–137)	0 (-0–1)	4369 (3292–5447)	4 (-12–19)
Switzerland	2036	54 (-35–143)	0 (-1–1)	4369 (3254–5485)	3 (-13–20)
Syrian Arab Republic	2022	67 (64–69)	1 (1–1)	957 (929–984)	12 (11–13)
Syrian Arab Republic	2023	67 (64–71)	1 (0–1)	974 (928–1019)	12 (10–13)
Syrian Arab Republic	2024	68 (64–72)	1 (0–1)	991 (931–1051)	11 (10–13)
Syrian Arab Republic	2025	69 (64–74)	1 (0–1)	1008 (936–1080)	11 (9–13)
Syrian Arab Republic	2026	69 (64–75)	1 (0–1)	1025 (943–1108)	11 (8–13)
Syrian Arab Republic	2027	70 (64–76)	0 (0–1)	1042 (950–1134)	10 (8–13)
Syrian Arab Republic	2028	71 (64–77)	0 (0–1)	1059 (959–1160)	10 (7–12)
Syrian Arab Republic	2029	71 (64–78)	0 (0–1)	1076 (968–1185)	10 (7–12)
Syrian Arab Republic	2030	72 (65–79)	0 (0–1)	1093 (978–1209)	9 (6–12)
Syrian Arab Republic	2031	72 (65–80)	0 (0–1)	1111 (988–1233)	9 (6–12)
Syrian Arab Republic	2032	73 (65–81)	0 (0–1)	1128 (999–1257)	9 (5–12)
Syrian Arab Republic	2033	74 (65–82)	0 (0–1)	1145 (1010–1280)	8 (5–11)
Syrian Arab Republic	2034	74 (66–83)	0 (0–1)	1162 (1021–1303)	8 (4–11)
Syrian Arab Republic	2035	75 (66–84)	0 (0–1)	1179 (1032–1326)	8 (4–11)
Syrian Arab Republic	2036	75 (66–85)	0 (0–1)	1196 (1044–1348)	7 (4–11)
Taiwan (Province of China)	2022	447 (412–482)	1 (1–1)	37547 (35596–39498)	19 (15–23)
Taiwan (Province of China)	2023	447 (398–497)	1 (1–1)	37293 (34121–40465)	18 (12–23)
Taiwan (Province of China)	2024	447 (387–508)	1 (1–1)	37040 (32638–41441)	16 (9–23)
Taiwan (Province of China)	2025	447 (377–517)	1 (0–1)	36786 (31099–42473)	14 (6–22)
Taiwan (Province of China)	2026	447 (369–526)	1 (0–1)	36533 (29492–43573)	13 (4–22)
Taiwan (Province of China)	2027	447 (362–533)	1 (0–1)	36279 (27813–44745)	11 (1–21)
Taiwan (Province of China)	2028	447 (355–540)	1 (0–1)	36026 (26064–45988)	9 (-1–20)

Taiwan (Province of China)	2029	447 (348–546)	0 (-0–1)	35772 (24244–47300)	8 (-3–19)
Taiwan (Province of China)	2030	447 (342–552)	0 (-0–1)	35519 (22357–48680)	6 (-6–18)
Taiwan (Province of China)	2031	447 (337–558)	0 (-0–1)	35265 (20404–50126)	4 (-8–17)
Taiwan (Province of China)	2032	447 (331–563)	0 (-0–1)	35011 (18387–51636)	3 (-10–16)
Taiwan (Province of China)	2033	447 (326–569)	0 (-0–1)	34758 (16308–53207)	1 (-13–15)
Taiwan (Province of China)	2034	447 (321–574)	0 (-1–1)	34504 (14170–54839)	-0 (-15–14)
Taiwan (Province of China)	2035	447 (316–578)	0 (-1–1)	34251 (11973–56529)	-2 (-17–13)
Taiwan (Province of China)	2036	447 (312–583)	0 (-1–1)	33997 (9718–58276)	-4 (-19–12)
Tajikistan	2022	65 (60–70)	1 (1–1)	2741 (2575–2906)	26 (21–31)
Tajikistan	2023	65 (57–74)	1 (1–1)	2798 (2515–3082)	23 (17–30)
Tajikistan	2024	66 (53–78)	1 (1–1)	2856 (2446–3266)	21 (13–29)
Tajikistan	2025	66 (50–83)	1 (0–1)	2914 (2367–3461)	18 (9–27)
Tajikistan	2026	67 (46–87)	1 (0–1)	2972 (2278–3665)	16 (6–26)
Tajikistan	2027	67 (42–92)	1 (0–1)	3029 (2179–3880)	13 (2–24)
Tajikistan	2028	68 (38–97)	1 (0–1)	3087 (2070–4104)	11 (-1–23)
Tajikistan	2029	68 (33–103)	0 (-0–1)	3145 (1952–4337)	8 (-5–21)
Tajikistan	2030	68 (28–108)	0 (-0–1)	3203 (1826–4579)	6 (-8–19)
Tajikistan	2031	69 (23–114)	0 (-0–1)	3260 (1691–4830)	3 (-11–17)
Tajikistan	2032	69 (18–120)	0 (-0–1)	3318 (1548–5088)	0 (-15–16)
Tajikistan	2033	70 (13–127)	0 (-1–1)	3376 (1397–5355)	-2 (-18–14)
Tajikistan	2034	70 (7–133)	-0 (-1–1)	3433 (1238–5629)	-5 (-21–12)
Tajikistan	2035	71 (1–140)	-0 (-1–1)	3491 (1073–5910)	-7 (-24–10)
Tajikistan	2036	71 (-5–147)	-0 (-1–0)	3549 (900–6198)	-10 (-28–8)
Thailand	2022	660 (632–687)	1 (1–1)	41494 (40205–42783)	16 (14–17)
Thailand	2023	665 (626–704)	1 (1–1)	42155 (40332–43978)	15 (13–17)
Thailand	2024	671 (623–718)	1 (0–1)	42816 (40583–45048)	15 (12–18)
Thailand	2025	676 (621–731)	1 (0–1)	43477 (40899–46055)	15 (11–18)
Thailand	2026	681 (620–743)	1 (0–1)	44138 (41256–47020)	15 (10–19)
Thailand	2027	687 (619–754)	1 (0–1)	44799 (41642–47956)	14 (9–19)
Thailand	2028	692 (619–765)	1 (0–1)	45460 (42050–48871)	14 (8–20)
Thailand	2029	697 (620–775)	1 (0–1)	46121 (42475–49767)	14 (7–20)
Thailand	2030	703 (620–785)	1 (0–1)	46782 (42915–50649)	14 (6–21)
Thailand	2031	708 (621–795)	1 (0–1)	47443 (43367–51520)	13 (5–21)

Thailand	2032	713 (622–805)	1 (0–1)	48104 (43829–52380)	13 (4–22)
Thailand	2033	719 (623–814)	1 (0–1)	48765 (44300–53231)	13 (3–23)
Thailand	2034	724 (625–823)	1 (0–1)	49427 (44779–54074)	13 (2–23)
Thailand	2035	729 (627–832)	1 (0–1)	50088 (45265–54911)	12 (1–24)
Thailand	2036	735 (628–841)	0 (-0–1)	50749 (45756–55741)	12 (-0–25)
Timor-Leste	2022	5 (5–5)	1 (1–1)	125 (123–128)	15 (15–15)
Timor-Leste	2023	5 (5–5)	1 (1–1)	128 (124–132)	15 (14–16)
Timor-Leste	2024	5 (5–5)	1 (1–1)	130 (123–136)	15 (14–16)
Timor-Leste	2025	5 (5–6)	1 (1–1)	132 (123–141)	15 (13–17)
Timor-Leste	2026	5 (5–6)	1 (1–1)	134 (122–147)	15 (12–17)
Timor-Leste	2027	5 (5–6)	1 (0–1)	136 (121–152)	15 (11–18)
Timor-Leste	2028	6 (5–6)	1 (0–1)	139 (119–158)	15 (11–19)
Timor-Leste	2029	6 (5–6)	1 (0–1)	141 (118–164)	14 (10–19)
Timor-Leste	2030	6 (5–7)	1 (0–1)	143 (116–170)	14 (9–20)
Timor-Leste	2031	6 (5–7)	1 (0–1)	145 (114–177)	14 (8–21)
Timor-Leste	2032	6 (4–7)	1 (0–1)	147 (112–183)	14 (7–22)
Timor-Leste	2033	6 (4–7)	1 (0–1)	150 (109–190)	14 (6–23)
Timor-Leste	2034	6 (4–8)	1 (0–1)	152 (106–197)	14 (5–23)
Timor-Leste	2035	6 (4–8)	1 (0–1)	154 (104–204)	14 (4–24)
Timor-Leste	2036	6 (4–8)	1 (0–1)	156 (101–212)	14 (3–25)
Togo	2022	17 (17–17)	0 (0–0)	1302 (1289–1315)	11 (11–11)
Togo	2023	17 (17–18)	0 (0–0)	1357 (1328–1386)	11 (10–11)
Togo	2024	18 (17–18)	0 (0–0)	1412 (1363–1461)	11 (10–11)
Togo	2025	18 (17–19)	0 (0–0)	1467 (1395–1539)	10 (10–11)
Togo	2026	18 (17–20)	0 (0–0)	1522 (1425–1619)	10 (9–11)
Togo	2027	19 (17–21)	0 (0–0)	1577 (1452–1702)	10 (9–11)
Togo	2028	19 (17–22)	0 (0–0)	1632 (1477–1787)	10 (9–11)
Togo	2029	20 (16–23)	0 (0–0)	1687 (1500–1875)	10 (8–11)
Togo	2030	20 (16–24)	0 (0–0)	1742 (1521–1964)	10 (8–11)
Togo	2031	20 (16–25)	0 (0–0)	1797 (1540–2055)	9 (8–11)
Togo	2032	21 (16–26)	0 (0–0)	1852 (1557–2147)	9 (7–11)
Togo	2033	21 (16–27)	0 (0–0)	1907 (1573–2242)	9 (7–11)
Togo	2034	22 (15–28)	0 (0–0)	1962 (1587–2338)	9 (7–11)

Togo	2035	22 (15–29)	0 (0–0)	2017 (1599–2435)	9 (7–11)
Togo	2036	23 (15–30)	0 (0–0)	2072 (1610–2534)	8 (6–10)
Tokelau	2022	0 (0–0)	1 (1–1)	0 (0–0)	17 (17–17)
Tokelau	2023	0 (0–0)	1 (1–1)	0 (0–0)	17 (16–17)
Tokelau	2024	0 (0–0)	1 (1–1)	0 (0–0)	16 (16–17)
Tokelau	2025	0 (0–0)	1 (1–1)	0 (0–0)	16 (15–17)
Tokelau	2026	0 (0–0)	1 (0–1)	0 (0–0)	16 (14–17)
Tokelau	2027	0 (0–0)	1 (0–1)	0 (0–0)	16 (14–18)
Tokelau	2028	0 (0–0)	1 (0–1)	0 (0–0)	15 (13–18)
Tokelau	2029	0 (0–0)	1 (0–1)	0 (0–0)	15 (12–18)
Tokelau	2030	0 (0–0)	1 (0–1)	0 (0–0)	15 (11–18)
Tokelau	2031	0 (0–0)	0 (0–1)	0 (0–0)	14 (10–19)
Tokelau	2032	0 (0–0)	0 (0–1)	0 (0–0)	14 (9–19)
Tokelau	2033	0 (0–0)	0 (0–1)	0 (0–0)	14 (9–19)
Tokelau	2034	0 (0–0)	0 (0–1)	0 (0–0)	14 (8–20)
Tokelau	2035	0 (0–0)	0 (0–1)	0 (0–0)	13 (7–20)
Tokelau	2036	0 (0–0)	0 (0–1)	0 (0–0)	13 (6–20)
Tonga	2022	1 (1–1)	2 (2–2)	14 (14–15)	39 (37–41)
Tonga	2023	1 (1–1)	2 (1–2)	14 (13–16)	38 (34–42)
Tonga	2024	1 (1–1)	2 (1–2)	14 (13–16)	38 (33–43)
Tonga	2025	1 (1–1)	2 (1–2)	14 (12–16)	37 (31–43)
Tonga	2026	1 (1–2)	2 (1–2)	14 (12–17)	36 (29–43)
Tonga	2027	1 (1–2)	1 (1–2)	14 (12–17)	36 (28–43)
Tonga	2028	1 (1–2)	1 (1–2)	14 (12–17)	35 (27–43)
Tonga	2029	1 (1–2)	1 (1–2)	14 (12–17)	34 (25–43)
Tonga	2030	1 (1–2)	1 (1–2)	14 (11–17)	34 (24–43)
Tonga	2031	1 (1–2)	1 (1–2)	14 (11–18)	33 (23–43)
Tonga	2032	1 (1–2)	1 (1–2)	14 (11–18)	32 (22–43)
Tonga	2033	1 (1–2)	1 (1–2)	14 (11–18)	32 (21–43)
Tonga	2034	1 (1–2)	1 (1–2)	14 (11–18)	31 (19–43)
Tonga	2035	1 (1–2)	1 (1–2)	14 (11–18)	30 (18–42)
Tonga	2036	1 (1–2)	1 (1–2)	14 (10–18)	30 (17–42)
Trinidad and Tobago	2022	5 (5–6)	0 (0–0)	214 (201–228)	7 (6–8)

Trinidad and Tobago	2023	5 (5–6)	0 (0–0)	217 (198–236)	7 (5–8)
Trinidad and Tobago	2024	5 (5–6)	0 (0–0)	219 (196–243)	6 (5–8)
Trinidad and Tobago	2025	5 (5–6)	0 (0–0)	222 (195–249)	6 (4–8)
Trinidad and Tobago	2026	5 (5–6)	0 (0–0)	224 (194–254)	5 (3–8)
Trinidad and Tobago	2027	5 (5–6)	0 (0–0)	227 (194–260)	5 (3–7)
Trinidad and Tobago	2028	5 (5–6)	0 (0–0)	229 (194–265)	5 (2–7)
Trinidad and Tobago	2029	5 (4–6)	0 (0–0)	232 (194–270)	4 (2–7)
Trinidad and Tobago	2030	5 (4–7)	0 (0–0)	234 (194–275)	4 (1–7)
Trinidad and Tobago	2031	5 (4–7)	0 (0–0)	237 (194–280)	4 (1–6)
Trinidad and Tobago	2032	5 (4–7)	0 (-0–0)	239 (195–284)	3 (0–6)
Trinidad and Tobago	2033	5 (4–7)	0 (-0–0)	242 (195–289)	3 (-0–6)
Trinidad and Tobago	2034	5 (4–7)	0 (-0–0)	245 (196–293)	3 (-1–6)
Trinidad and Tobago	2035	5 (4–7)	0 (-0–0)	247 (197–298)	2 (-1–5)
Trinidad and Tobago	2036	5 (4–7)	0 (-0–0)	250 (197–302)	2 (-2–5)
Tunisia	2022	70 (69–71)	1 (1–1)	914 (904–923)	11 (11–12)
Tunisia	2023	71 (69–72)	1 (0–1)	937 (920–953)	11 (10–12)
Tunisia	2024	71 (68–74)	1 (0–1)	958 (932–984)	11 (10–12)
Tunisia	2025	72 (68–76)	0 (0–1)	980 (944–1017)	10 (10–11)
Tunisia	2026	72 (67–77)	0 (0–1)	1002 (953–1051)	10 (9–11)
Tunisia	2027	73 (66–79)	0 (0–1)	1024 (963–1086)	10 (9–11)
Tunisia	2028	73 (66–81)	0 (0–1)	1046 (971–1122)	10 (8–11)
Tunisia	2029	74 (65–83)	0 (0–1)	1068 (978–1159)	9 (8–11)
Tunisia	2030	74 (64–85)	0 (0–1)	1090 (984–1197)	9 (8–10)
Tunisia	2031	75 (62–88)	0 (0–1)	1112 (989–1235)	9 (7–10)
Tunisia	2032	76 (61–90)	0 (0–1)	1134 (994–1274)	8 (7–10)
Tunisia	2033	76 (60–92)	0 (0–1)	1156 (998–1315)	8 (7–10)
Tunisia	2034	77 (59–95)	0 (0–1)	1178 (1001–1355)	8 (6–9)
Tunisia	2035	77 (57–97)	0 (-0–1)	1200 (1004–1397)	8 (6–9)
Tunisia	2036	78 (56–100)	0 (-0–1)	1222 (1006–1439)	7 (6–9)
Turkey	2022	1290 (1252–1328)	1 (1–1)	13930 (13618–14242)	32 (30–34)
Turkey	2023	1285 (1210–1359)	1 (1–1)	14029 (13383–14674)	31 (27–35)
Turkey	2024	1281 (1170–1392)	1 (1–2)	14108 (13104–15113)	30 (23–36)
Turkey	2025	1279 (1132–1425)	1 (1–2)	14173 (12800–15546)	29 (19–38)

Turkey	2026	1277 (1098–1457)	1 (1–2)	14225 (12484–15966)	28 (15–41)
Turkey	2027	1276 (1065–1487)	1 (0–2)	14268 (12165–16371)	27 (10–43)
Turkey	2028	1275 (1035–1515)	1 (0–2)	14302 (11846–16758)	26 (5–46)
Turkey	2029	1275 (1007–1542)	1 (-0–2)	14330 (11532–17128)	25 (-0–49)
Turkey	2030	1274 (982–1567)	1 (-0–2)	14353 (11225–17480)	24 (-6–53)
Turkey	2031	1274 (957–1591)	1 (-0–2)	14371 (10925–17816)	23 (-11–56)
Turkey	2032	1274 (934–1613)	1 (-1–2)	14386 (10634–18137)	21 (-17–60)
Turkey	2033	1274 (913–1635)	1 (-1–3)	14398 (10352–18443)	20 (-24–64)
Turkey	2034	1274 (892–1655)	1 (-1–3)	14408 (10079–18736)	19 (-30–69)
Turkey	2035	1273 (873–1674)	1 (-2–3)	14415 (9814–19017)	18 (-37–73)
Turkey	2036	1273 (854–1693)	1 (-2–3)	14422 (9558–19286)	17 (-44–78)
Turkmenistan	2022	33 (29–36)	1 (1–1)	2433 (2073–2792)	20 (15–24)
Turkmenistan	2023	32 (27–37)	1 (0–1)	2477 (1906–3047)	18 (12–25)
Turkmenistan	2024	31 (25–37)	1 (0–1)	2521 (1745–3296)	16 (8–24)
Turkmenistan	2025	31 (23–38)	1 (0–1)	2565 (1579–3550)	15 (5–24)
Turkmenistan	2026	30 (22–38)	1 (0–1)	2609 (1405–3812)	13 (2–23)
Turkmenistan	2027	29 (21–38)	0 (-0–1)	2653 (1222–4083)	11 (-0–22)
Turkmenistan	2028	28 (19–38)	0 (-0–1)	2697 (1030–4363)	9 (-3–21)
Turkmenistan	2029	28 (18–38)	0 (-0–1)	2741 (828–4654)	7 (-6–21)
Turkmenistan	2030	27 (17–38)	0 (-0–1)	2785 (616–4953)	6 (-8–20)
Turkmenistan	2031	26 (15–38)	0 (-0–1)	2829 (395–5262)	4 (-11–19)
Turkmenistan	2032	26 (14–37)	0 (-1–1)	2873 (165–5580)	2 (-13–17)
Turkmenistan	2033	25 (13–37)	0 (-1–1)	2917 (-74–5907)	0 (-16–16)
Turkmenistan	2034	24 (12–37)	0 (-1–1)	2961 (-321–6242)	-1 (-18–15)
Turkmenistan	2035	24 (11–37)	-0 (-1–1)	3005 (-577–6586)	-3 (-20–14)
Turkmenistan	2036	23 (9–37)	-0 (-1–1)	3049 (-841–6939)	-5 (-23–13)
Tuvalu	2022	0 (0–0)	1 (1–1)	2 (2–2)	29 (29–30)
Tuvalu	2023	0 (0–0)	1 (1–1)	2 (2–2)	29 (28–30)
Tuvalu	2024	0 (0–0)	1 (1–1)	2 (2–2)	29 (27–30)
Tuvalu	2025	0 (0–0)	1 (1–1)	2 (2–2)	28 (26–30)
Tuvalu	2026	0 (0–0)	1 (1–1)	2 (2–2)	28 (25–31)
Tuvalu	2027	0 (0–0)	1 (1–1)	2 (2–2)	28 (24–31)
Tuvalu	2028	0 (0–0)	1 (1–1)	2 (2–2)	27 (23–32)

Tuvalu	2029	0 (0–0)	1 (1–1)	2 (2–2)	27 (21–32)
Tuvalu	2030	0 (0–0)	1 (1–1)	2 (2–2)	26 (20–33)
Tuvalu	2031	0 (0–0)	1 (1–1)	2 (2–2)	26 (19–33)
Tuvalu	2032	0 (0–0)	1 (1–1)	2 (2–2)	26 (17–34)
Tuvalu	2033	0 (0–0)	1 (1–1)	2 (2–2)	25 (16–35)
Tuvalu	2034	0 (0–0)	1 (1–1)	2 (2–2)	25 (14–36)
Tuvalu	2035	0 (0–0)	1 (0–1)	2 (2–2)	25 (13–37)
Tuvalu	2036	0 (0–0)	1 (0–1)	2 (2–2)	24 (11–37)
Uganda	2022	24 (24–25)	0 (0–0)	5957 (5858–6055)	4 (4–4)
Uganda	2023	25 (23–26)	0 (0–0)	6109 (5926–6292)	4 (4–5)
Uganda	2024	25 (23–26)	0 (0–0)	6267 (5974–6559)	4 (3–5)
Uganda	2025	25 (22–27)	0 (0–0)	6422 (6007–6837)	4 (3–5)
Uganda	2026	25 (21–28)	0 (0–0)	6579 (6026–7131)	4 (3–5)
Uganda	2027	25 (21–28)	0 (0–0)	6735 (6033–7437)	4 (2–6)
Uganda	2028	24 (20–29)	0 (0–0)	6891 (6028–7754)	4 (2–6)
Uganda	2029	24 (19–29)	0 (0–0)	7047 (6013–8081)	4 (1–6)
Uganda	2030	24 (19–29)	0 (0–0)	7203 (5988–8419)	4 (0–7)
Uganda	2031	24 (18–29)	0 (-0–0)	7360 (5954–8766)	4 (-0–7)
Uganda	2032	24 (18–29)	0 (-0–0)	7516 (5910–9122)	3 (-1–8)
Uganda	2033	23 (18–29)	0 (-0–0)	7672 (5857–9487)	3 (-1–8)
Uganda	2034	23 (17–29)	0 (-0–0)	7828 (5797–9860)	3 (-2–9)
Uganda	2035	23 (17–29)	0 (-0–0)	7985 (5728–10241)	3 (-3–9)
Uganda	2036	23 (17–29)	0 (-0–0)	8141 (5652–10630)	3 (-3–10)
Ukraine	2022	722 (611–834)	1 (1–1)	19150 (17184–21115)	27 (23–32)
Ukraine	2023	672 (479–865)	1 (1–1)	18773 (15994–21553)	25 (17–33)
Ukraine	2024	622 (373–870)	1 (0–1)	18397 (14993–21801)	23 (13–33)
Ukraine	2025	571 (277–866)	1 (0–1)	18021 (14090–21952)	21 (9–32)
Ukraine	2026	521 (188–855)	1 (0–1)	17645 (13250–22039)	19 (6–32)
Ukraine	2027	471 (102–839)	1 (0–1)	17268 (12454–22083)	17 (2–31)
Ukraine	2028	420 (20–821)	1 (-0–1)	16892 (11692–22092)	15 (-1–30)
Ukraine	2029	370 (-60–800)	0 (-0–1)	16516 (10957–22075)	13 (-4–29)
Ukraine	2030	320 (-138–778)	0 (-0–1)	16140 (10244–22036)	10 (-7–28)
Ukraine	2031	269 (-215–754)	0 (-0–1)	15763 (9548–21979)	8 (-10–27)

Ukraine	2032	219 (-290–728)	0 (-0–1)	15387 (8869–21906)	6 (-14–26)
Ukraine	2033	169 (-364–702)	0 (-1–1)	15011 (8203–21819)	4 (-17–25)
Ukraine	2034	118 (-437–674)	0 (-1–1)	14635 (7548–21721)	2 (-19–24)
Ukraine	2035	68 (-509–645)	0 (-1–1)	14259 (6905–21612)	0 (-22–23)
Ukraine	2036	18 (-581–616)	-0 (-1–1)	13882 (6270–21494)	-2 (-25–21)
United Arab Emirates	2022	10 (9–11)	0 (0–1)	384 (355–413)	8 (6–10)
United Arab Emirates	2023	11 (10–12)	0 (0–1)	394 (363–425)	7 (4–11)
United Arab Emirates	2024	11 (10–12)	0 (0–1)	404 (372–437)	7 (3–11)
United Arab Emirates	2025	11 (10–12)	0 (0–1)	414 (380–448)	6 (1–11)
United Arab Emirates	2026	11 (10–12)	0 (0–1)	424 (388–460)	6 (0–11)
United Arab Emirates	2027	11 (10–13)	0 (0–1)	434 (397–471)	5 (-1–12)
United Arab Emirates	2028	12 (10–13)	0 (0–1)	444 (405–483)	5 (-2–12)
United Arab Emirates	2029	12 (10–13)	0 (-0–1)	454 (414–494)	4 (-3–12)
United Arab Emirates	2030	12 (10–13)	0 (-0–1)	464 (422–505)	4 (-4–11)
United Arab Emirates	2031	12 (11–14)	0 (-0–1)	474 (431–516)	3 (-5–11)
United Arab Emirates	2032	12 (11–14)	0 (-0–1)	484 (440–527)	3 (-6–11)
United Arab Emirates	2033	13 (11–14)	0 (-0–1)	494 (449–539)	2 (-7–11)
United Arab Emirates	2034	13 (11–14)	0 (-0–1)	503 (457–550)	2 (-8–11)
United Arab Emirates	2035	13 (11–15)	0 (-0–1)	513 (466–561)	1 (-9–11)
United Arab Emirates	2036	13 (11–15)	0 (-0–1)	523 (475–572)	1 (-9–11)
United Kingdom	2022	704 (674–734)	0 (0–1)	70137 (67732–72541)	10 (9–10)
United Kingdom	2023	687 (634–739)	0 (0–1)	69257 (65453–73061)	9 (8–10)
United Kingdom	2024	670 (593–747)	0 (0–1)	68377 (63215–73540)	9 (7–10)
United Kingdom	2025	653 (549–756)	0 (0–1)	67498 (60946–74049)	8 (6–11)
United Kingdom	2026	635 (503–768)	0 (0–1)	66618 (58627–74609)	8 (5–11)
United Kingdom	2027	618 (455–781)	0 (0–1)	65738 (56248–75228)	7 (4–11)
United Kingdom	2028	601 (405–797)	0 (0–1)	64859 (53808–75909)	7 (3–11)
United Kingdom	2029	584 (354–815)	0 (0–1)	63979 (51305–76653)	7 (2–12)
United Kingdom	2030	567 (300–834)	0 (0–1)	63099 (48741–77458)	6 (0–12)
United Kingdom	2031	550 (245–855)	0 (-0–1)	62220 (46116–78323)	6 (-1–13)
United Kingdom	2032	533 (188–878)	0 (-0–1)	61340 (43432–79248)	5 (-2–13)
United Kingdom	2033	516 (130–902)	0 (-0–1)	60460 (40690–80230)	5 (-4–13)
United Kingdom	2034	499 (70–927)	0 (-0–1)	59581 (37891–81270)	5 (-5–14)

United Kingdom	2035	481 (8–955)	0 (-0–1)	58701 (35037–82364)	4 (-6–15)
United Kingdom	2036	464 (-55–983)	0 (-0–1)	57821 (32129–83513)	4 (-8–15)
United Republic of Tanzania	2022	74 (74–75)	0 (0–0)	10475 (10383–10567)	7 (7–8)
United Republic of Tanzania	2023	75 (73–76)	0 (0–0)	10553 (10354–10751)	7 (7–8)
United Republic of Tanzania	2024	75 (72–77)	0 (0–0)	10542 (10226–10858)	7 (7–8)
United Republic of Tanzania	2025	75 (71–78)	0 (0–0)	10446 (10012–10880)	7 (6–8)
United Republic of Tanzania	2026	74 (70–78)	0 (0–0)	10273 (9730–10817)	7 (6–8)
United Republic of Tanzania	2027	74 (69–78)	0 (0–0)	10037 (9398–10675)	7 (6–8)
United Republic of Tanzania	2028	73 (68–78)	0 (0–0)	9754 (9040–10468)	7 (5–8)
United Republic of Tanzania	2029	72 (67–78)	0 (0–0)	9445 (8678–10213)	7 (5–9)
United Republic of Tanzania	2030	71 (66–77)	0 (0–0)	9132 (8332–9933)	7 (4–9)
United Republic of Tanzania	2031	71 (65–77)	0 (0–0)	8837 (8021–9652)	7 (4–9)
United Republic of Tanzania	2032	70 (64–76)	0 (0–0)	8578 (7759–9397)	7 (4–9)
United Republic of Tanzania	2033	70 (64–76)	0 (0–0)	8373 (7554–9193)	6 (3–10)
United Republic of Tanzania	2034	69 (63–75)	0 (0–0)	8236 (7412–9059)	6 (3–10)
United Republic of Tanzania	2035	69 (63–75)	0 (0–0)	8173 (7334–9012)	6 (2–10)
United Republic of Tanzania	2036	69 (63–75)	0 (0–0)	8187 (7320–9054)	6 (2–11)
United States of America	2022	2035 (1980–2091)	0 (0–0)	234834 (230592–239077	8 (7–8)
United States of America	2023	2006 (1928–2084)	0 (0–0)	236397 (228519–244275	8 (7–8)
United States of America	2024	1977 (1881–2073)	0 (0–0)	237960 (227292–248628	7 (6–8)
United States of America	2025	1947 (1837–2058)	0 (0–0)	239522 (226339–252706	7 (6–8)
United States of America	2026	1918 (1794–2042)	0 (0–0)	241085 (225506–256664	7 (5–8)
United States of America	2027	1889 (1753–2024)	0 (0–0)	242648 (224729–260566	6 (5–8)
United States of America	2028	1859 (1713–2006)	0 (0–0)	244210 (223973–264447	6 (4–8)
United States of America	2029	1830 (1673–1987)	0 (0–0)	245773 (223218–268327	6 (4–8)
United States of America	2030	1801 (1634–1967)	0 (0–0)	247336 (222452–272219	6 (3–8)
United States of America	2031	1771 (1596–1946)	0 (0–0)	248898 (221666–276130	5 (3–8)
United States of America	2032	1742 (1558–1926)	0 (0–0)	250461 (220855–280067	5 (2–8)
United States of America	2033	1713 (1521–1905)	0 (0–0)	252024 (220014–284033	5 (2–8)
United States of America	2034	1683 (1484–1883)	0 (0–0)	253586 (219142–288031	5 (1–8)
United States of America	2035	1654 (1447–1861)	0 (0–0)	255149 (218236–292062	4 (0–8)
United States of America	2036	1625 (1410–1839)	0 (0–0)	256712 (217294–296129	4 (-0–8)
United States Virgin Islands	2022	1 (0–1)	0 (0–0)	19 (18–21)	6 (6–7)

United States Virgin Islands	2023	1 (0–1)	0 (0–0)	19 (16–21)	6 (5–7)
United States Virgin Islands	2024	1 (0–1)	0 (0–0)	18 (15–22)	6 (4–7)
United States Virgin Islands	2025	1 (0–1)	0 (0–0)	17 (13–22)	5 (4–7)
United States Virgin Islands	2026	1 (0–1)	0 (0–0)	17 (11–22)	5 (3–7)
United States Virgin Islands	2027	1 (0–1)	0 (0–0)	16 (10–22)	5 (2–7)
United States Virgin Islands	2028	1 (0–1)	0 (0–0)	15 (8–23)	4 (2–7)
United States Virgin Islands	2029	1 (0–1)	0 (0–0)	15 (6–23)	4 (1–6)
United States Virgin Islands	2030	1 (0–1)	0 (0–0)	14 (4–24)	3 (1–6)
United States Virgin Islands	2031	1 (0–1)	0 (0–0)	13 (2–24)	3 (0–6)
United States Virgin Islands	2032	1 (0–1)	0 (-0–0)	13 (0–25)	3 (-0–6)
United States Virgin Islands	2033	1 (0–1)	0 (-0–0)	12 (-2–26)	2 (-1–6)
United States Virgin Islands	2034	1 (0–1)	0 (-0–0)	11 (-4–26)	2 (-1–5)
United States Virgin Islands	2035	1 (0–1)	0 (-0–0)	11 (-6–27)	2 (-2–5)
United States Virgin Islands	2036	1 (0–1)	0 (-0–0)	10 (-8–28)	1 (-2–5)
Uruguay	2022	53 (50–57)	1 (1–1)	2142 (1938–2346)	25 (23–27)
Uruguay	2023	53 (48–58)	1 (1–1)	2142 (1854–2431)	24 (22–27)
Uruguay	2024	53 (47–60)	1 (1–1)	2142 (1789–2495)	24 (21–27)
Uruguay	2025	53 (46–61)	1 (1–1)	2142 (1735–2550)	23 (20–27)
Uruguay	2026	53 (45–61)	1 (1–1)	2142 (1686–2598)	23 (19–27)
Uruguay	2027	53 (45–62)	1 (1–1)	2142 (1643–2642)	22 (18–27)
Uruguay	2028	53 (44–63)	1 (1–1)	2142 (1603–2682)	22 (17–26)
Uruguay	2029	53 (43–64)	1 (1–1)	2142 (1566–2719)	21 (16–26)
Uruguay	2030	53 (43–64)	1 (1–1)	2142 (1531–2754)	21 (15–26)
Uruguay	2031	53 (42–65)	1 (1–1)	2142 (1498–2787)	20 (14–26)
Uruguay	2032	53 (41–65)	1 (1–1)	2142 (1466–2818)	19 (13–25)
Uruguay	2033	53 (41–66)	1 (0–1)	2142 (1436–2848)	19 (13–25)
Uruguay	2034	53 (40–66)	1 (0–1)	2142 (1407–2877)	18 (12–25)
Uruguay	2035	53 (40–67)	1 (0–1)	2142 (1380–2905)	18 (11–25)
Uruguay	2036	53 (39–67)	1 (0–1)	2142 (1353–2932)	17 (10–24)
Uzbekistan	2022	122 (114–130)	0 (0–0)	4859 (4108–5611)	12 (10–13)
Uzbekistan	2023	123 (109–136)	0 (0–0)	4859 (3479–6240)	11 (9–14)
Uzbekistan	2024	123 (105–141)	0 (0–1)	4859 (3058–6661)	11 (8–14)
Uzbekistan	2025	123 (101–145)	0 (0–1)	4859 (2718–7001)	10 (7–14)

Uzbekistan	2026	123 (98–148)	0 (0–1)	4859 (2425–7294)	10 (6–14)
Uzbekistan	2027	123 (95–151)	0 (0–1)	4859 (2164–7555)	10 (5–14)
Uzbekistan	2028	123 (92–154)	0 (0–1)	4859 (1926–7793)	9 (4–14)
Uzbekistan	2029	123 (89–156)	0 (0–1)	4859 (1705–8014)	9 (3–14)
Uzbekistan	2030	123 (87–159)	0 (0–1)	4859 (1500–8219)	8 (2–14)
Uzbekistan	2031	123 (85–161)	0 (0–1)	4859 (1306–8413)	8 (2–14)
Uzbekistan	2032	123 (83–163)	0 (0–0)	4859 (1122–8597)	7 (1–14)
Uzbekistan	2033	123 (81–165)	0 (0–0)	4859 (947–8772)	7 (0–14)
Uzbekistan	2034	123 (79–167)	0 (0–0)	4859 (779–8940)	6 (-1–13)
Uzbekistan	2035	123 (77–169)	0 (-0–0)	4859 (618–9101)	6 (-1–13)
Uzbekistan	2036	123 (75–170)	0 (-0–0)	4859 (463–9256)	5 (-2–13)
Vanuatu	2022	1 (1–1)	1 (1–1)	21 (21–21)	22 (21–23)
Vanuatu	2023	1 (1–2)	1 (1–1)	21 (21–22)	21 (20–23)
Vanuatu	2024	2 (1–2)	1 (1–1)	22 (21–22)	21 (19–23)
Vanuatu	2025	2 (1–2)	1 (1–1)	22 (21–23)	20 (18–22)
Vanuatu	2026	2 (1–2)	1 (1–1)	22 (22–23)	20 (17–22)
Vanuatu	2027	2 (1–2)	1 (1–1)	23 (22–24)	19 (16–22)
Vanuatu	2028	2 (1–2)	1 (1–1)	23 (22–24)	19 (15–22)
Vanuatu	2029	2 (2–2)	1 (1–1)	23 (22–25)	18 (15–22)
Vanuatu	2030	2 (2–2)	1 (1–1)	24 (23–25)	18 (14–21)
Vanuatu	2031	2 (2–2)	1 (0–1)	24 (23–25)	17 (13–21)
Vanuatu	2032	2 (2–2)	1 (0–1)	24 (23–26)	17 (13–21)
Vanuatu	2033	2 (2–2)	1 (0–1)	25 (24–26)	16 (12–20)
Vanuatu	2034	2 (2–2)	1 (0–1)	25 (24–26)	16 (11–20)
Vanuatu	2035	2 (2–2)	1 (0–1)	26 (24–27)	15 (10–20)
Vanuatu	2036	2 (2–2)	1 (0–1)	26 (24–27)	15 (10–19)
Venezuela (Bolivarian Republic o	2022	156 (147–165)	1 (0–1)	2182 (2054–2311)	13 (12–14)
Venezuela (Bolivarian Republic o	2023	154 (142–166)	0 (0–1)	2203 (2071–2336)	13 (11–15)
Venezuela (Bolivarian Republic o	2024	153 (139–166)	0 (0–1)	2224 (2087–2361)	13 (10–16)
Venezuela (Bolivarian Republic o	2025	152 (137–166)	0 (0–1)	2245 (2104–2387)	13 (9–16)
Venezuela (Bolivarian Republic o	2026	151 (135–166)	0 (0–1)	2266 (2121–2412)	12 (8–17)
Venezuela (Bolivarian Republic o	2027	150 (134–166)	0 (0–1)	2287 (2138–2437)	12 (7–18)
Venezuela (Bolivarian Republic o	2028	149 (133–165)	0 (0–1)	2308 (2155–2461)	12 (6–18)

/enezuela (Bolivarian Republic o	2029	148 (132–165)	0 (0–1)	2329 (2172–2486)	12 (5–19)
/enezuela (Bolivarian Republic o	2030	148 (131–165)	0 (0–0)	2350 (2190–2511)	12 (4–20)
/enezuela (Bolivarian Republic o	2031	147 (130–164)	0 (0–0)	2371 (2207–2535)	12 (3–21)
/enezuela (Bolivarian Republic o	2032	147 (130–164)	0 (-0–0)	2392 (2224–2560)	12 (1–22)
/enezuela (Bolivarian Republic o	2033	147 (130–164)	0 (-0–0)	2413 (2242–2584)	11 (0–23)
/enezuela (Bolivarian Republic o	2034	146 (129–164)	0 (-0–0)	2434 (2259–2609)	11 (-1–24)
/enezuela (Bolivarian Republic o	2035	146 (129–163)	0 (-0–0)	2455 (2277–2633)	11 (-2–25)
/enezuela (Bolivarian Republic o	2036	146 (129–163)	0 (-0–0)	2476 (2295–2657)	11 (-3–26)
Viet Nam	2022	841 (826–856)	1 (1–1)	26921 (26663–27179)	21 (20–22)
Viet Nam	2023	847 (816–879)	1 (1–1)	27588 (27084–28092)	21 (19–22)
Viet Nam	2024	844 (792–896)	1 (1–1)	28255 (27465–29044)	20 (18–23)
Viet Nam	2025	834 (761–907)	1 (1–1)	28922 (27810–30033)	20 (17–23)
Viet Nam	2026	827 (734–920)	1 (1–1)	29588 (28121–31056)	20 (16–24)
Viet Nam	2027	820 (714–926)	1 (1–1)	30255 (28401–32109)	20 (14–25)
Viet Nam	2028	814 (697–930)	1 (0–1)	30922 (28653–33191)	19 (13–26)
Viet Nam	2029	808 (684–932)	1 (0–1)	31589 (28878–34299)	19 (12–27)
Viet Nam	2030	803 (672–934)	1 (0–1)	32255 (29078–35433)	19 (10–28)
Viet Nam	2031	798 (663–934)	1 (0–1)	32922 (29254–36591)	19 (9–29)
Viet Nam	2032	794 (654–933)	1 (0–1)	33589 (29407–37771)	19 (8–30)
Viet Nam	2033	790 (647–933)	1 (0–1)	34256 (29538–38973)	19 (6–31)
Viet Nam	2034	786 (641–932)	1 (0–1)	34923 (29649–40197)	18 (5–32)
Viet Nam	2035	783 (635–931)	1 (0–1)	35589 (29738–41440)	18 (3–33)
Viet Nam	2036	780 (631–929)	1 (0–1)	36256 (29809–42703)	18 (2–34)
Yemen	2022	294 (289–299)	2 (2–2)	6071 (5966–6176)	50 (49–52)
Yemen	2023	302 (293–310)	2 (2–2)	6186 (5986–6387)	50 (48–52)
Yemen	2024	310 (297–322)	2 (2–2)	6294 (6002–6585)	49 (47–52)
Yemen	2025	318 (301–335)	2 (2–2)	6397 (6022–6772)	48 (45–52)
Yemen	2026	326 (305–347)	2 (2–2)	6497 (6045–6949)	48 (44–52)
Yemen	2027	334 (308–360)	2 (2–2)	6595 (6073–7118)	47 (43–51)
Yemen	2028	342 (311–374)	2 (2–2)	6693 (6106–7280)	46 (42–51)
Yemen	2029	350 (313–387)	2 (2–2)	6790 (6142–7437)	46 (41–51)
Yemen	2030	358 (315–401)	2 (2–2)	6886 (6183–7589)	45 (40–50)
Yemen	2031	366 (317–415)	2 (2–2)	6982 (6227–7737)	44 (39–50)

Yemen	2032	374 (319–430)	2 (2–2)	7078 (6274–7882)	44 (38–50)
Yemen	2033	382 (320–445)	2 (2–2)	7174 (6323–8024)	43 (37–49)
Yemen	2034	390 (321–460)	2 (2–2)	7269 (6375–8164)	42 (36–49)
Yemen	2035	399 (322–475)	2 (2–2)	7365 (6429–8302)	42 (35–48)
Yemen	2036	407 (323–490)	2 (2–2)	7461 (6484–8438)	41 (34–48)
Zambia	2022	18 (17–18)	0 (0–0)	3555 (3507–3603)	6 (6–7)
Zambia	2023	17 (17–18)	0 (0–0)	3590 (3484–3696)	6 (6–7)
Zambia	2024	17 (16–19)	0 (0–0)	3623 (3448–3798)	6 (5–7)
Zambia	2025	17 (16–19)	0 (0–0)	3655 (3403–3907)	6 (5–7)
Zambia	2026	17 (15–19)	0 (0–0)	3686 (3349–4022)	6 (4–7)
Zambia	2027	17 (14–20)	0 (0–0)	3715 (3289–4141)	5 (4–7)
Zambia	2028	17 (13–21)	0 (0–0)	3743 (3222–4264)	5 (3–8)
Zambia	2029	17 (13–21)	0 (0–0)	3770 (3151–4389)	5 (2–8)
Zambia	2030	17 (12–22)	0 (0–0)	3796 (3074–4517)	5 (2–8)
Zambia	2031	17 (11–23)	0 (0–0)	3821 (2994–4647)	5 (1–8)
Zambia	2032	17 (10–23)	0 (-0–0)	3844 (2911–4778)	4 (0–9)
Zambia	2033	16 (9–24)	0 (-0–0)	3867 (2824–4911)	4 (-1–9)
Zambia	2034	16 (8–25)	0 (-0–0)	3889 (2734–5044)	4 (-1–10)
Zambia	2035	16 (7–26)	0 (-0–0)	3910 (2643–5178)	4 (-2–10)
Zambia	2036	16 (5–27)	0 (-0–0)	3930 (2549–5312)	4 (-3–10)
Zimbabwe	2022	53 (51–54)	1 (1–1)	7320 (7147–7492)	20 (19–21)
Zimbabwe	2023	52 (49–55)	1 (1–1)	7338 (6979–7698)	20 (19–21)
Zimbabwe	2024	52 (47–56)	1 (1–1)	7354 (6790–7917)	20 (19–22)
Zimbabwe	2025	51 (44–58)	1 (1–1)	7366 (6591–8142)	21 (18–23)
Zimbabwe	2026	50 (41–60)	1 (1–1)	7377 (6388–8366)	21 (18–24)
Zimbabwe	2027	50 (38–62)	1 (1–1)	7386 (6185–8587)	22 (19–25)
Zimbabwe	2028	49 (34–64)	1 (1–1)	7393 (5984–8802)	22 (19–26)
Zimbabwe	2029	49 (30–67)	1 (1–1)	7399 (5787–9011)	23 (19–27)
Zimbabwe	2030	48 (26–70)	1 (1–1)	7404 (5595–9214)	23 (20–27)
Zimbabwe	2031	48 (22–73)	1 (1–1)	7408 (5408–9409)	24 (20–28)
Zimbabwe	2032	47 (18–76)	1 (1–1)	7412 (5226–9597)	24 (20–29)
Zimbabwe	2033	46 (14–79)	1 (1–1)	7415 (5050–9779)	25 (21–29)
Zimbabwe	2034	46 (9–82)	1 (1–1)	7417 (4880–9954)	25 (21–29)

Zimbabwe	2035	45 (4–86)	1 (1–1)	7419 (4715–10123)	25 (21–29)
Zimbabwe	2036	45 (-0–90)	1 (1–1)	7420 (4556–10285)	25 (21–29)

Supplement table.5 Disease burden prediction for liver cancer death and disability-adjusted life years numbers and age-standardized rate to 2036 in different countries.

Country	Year	Death		DALY	
		(N)	ASR(per100,000)	(N)	ASR(per100,000)
Afghanistan	2022	26 (26–26)	0 (0–0)	859 (848–871)	7 (7–7)
Afghanistan	2023	27 (26–28)	0 (0–0)	901 (876–926)	7 (7–7)
Afghanistan	2024	28 (27–30)	0 (0–0)	943 (901–985)	7 (7–7)
Afghanistan	2025	29 (28–31)	0 (0–0)	985 (924–1047)	7 (7–7)
Afghanistan	2026	30 (28–33)	0 (0–0)	1027 (944–1110)	7 (7–7)
Afghanistan	2027	32 (28–35)	0 (0–0)	1069 (962–1176)	7 (7–7)
Afghanistan	2028	33 (29–37)	0 (0–0)	1111 (979–1244)	7 (7–7)
Afghanistan	2029	34 (29–39)	0 (0–0)	1153 (993–1313)	7 (7–7)
Afghanistan	2030	35 (29–41)	0 (0–0)	1195 (1006–1384)	7 (7–8)
Afghanistan	2031	36 (29–43)	0 (0–0)	1237 (1017–1457)	7 (7–8)
Afghanistan	2032	37 (29–45)	0 (0–0)	1279 (1027–1531)	7 (7–8)
Afghanistan	2033	38 (29–47)	0 (0–0)	1321 (1035–1606)	7 (7–8)
Afghanistan	2034	39 (29–49)	0 (0–0)	1363 (1042–1683)	7 (7–8)
Afghanistan	2035	40 (29–51)	0 (0–0)	1405 (1048–1761)	7 (7–8)
Afghanistan	2036	41 (29–54)	0 (0–0)	1447 (1052–1841)	7 (7–8)
Albania	2022	53 (51–55)	1 (1–1)	1220 (1172–1269)	28 (26–30)
Albania	2023	54 (50–58)	1 (1–1)	1243 (1135–1352)	28 (25–32)
Albania	2024	55 (48–63)	1 (1–1)	1266 (1084–1448)	28 (24–33)
Albania	2025	56 (46–67)	1 (1–1)	1289 (1023–1555)	29 (23–35)
Albania	2026	58 (43–72)	1 (1–2)	1312 (952–1672)	29 (22–36)
Albania	2027	59 (40–78)	1 (1–2)	1335 (872–1798)	29 (21–38)
Albania	2028	60 (37–84)	1 (1–2)	1358 (784–1932)	30 (20–40)
Albania	2029	61 (33–90)	1 (1–2)	1381 (688–2074)	30 (18–42)
Albania	2030	63 (29–96)	1 (1–2)	1404 (585–2223)	30 (17–43)
Albania	2031	64 (25–103)	1 (1–2)	1427 (475–2379)	31 (16–45)
Albania	2032	65 (21–110)	1 (1–2)	1450 (358–2542)	31 (15–47)
Albania	2033	66 (16–117)	1 (1–2)	1473 (236–2711)	31 (14–49)
Albania	2034	68 (11–124)	1 (1–2)	1496 (107–2885)	32 (12–51)
Albania	2035	69 (6–132)	1 (0–2)	1519 (-27–3065)	32 (11–53)
Albania	2036	70 (1–140)	1 (0–2)	1542 (-167–3251)	32 (9–55)
Algeria	2022	62 (62–63)	0 (0–0)	1685 (1671–1699)	4 (4–4)

Algeria	2023	65 (64–66)	0 (0–0)	1749 (1714–1783)	4 (4–4)
Algeria	2024	67 (65–69)	0 (0–0)	1812 (1752–1873)	4 (4–5)
Algeria	2025	70 (67–73)	0 (0–0)	1876 (1786–1967)	4 (4–5)
Algeria	2026	72 (69–76)	0 (0–0)	1940 (1815–2064)	4 (4–5)
Algeria	2027	75 (70–80)	0 (0–0)	2003 (1842–2165)	5 (4–5)
Algeria	2028	78 (71–84)	0 (0–0)	2067 (1865–2269)	5 (4–5)
Algeria	2029	80 (73–88)	0 (0–0)	2131 (1886–2376)	5 (4–5)
Algeria	2030	83 (74–91)	0 (0–0)	2195 (1903–2486)	5 (4–6)
Algeria	2031	85 (75–95)	0 (0–0)	2258 (1919–2598)	5 (4–6)
Algeria	2032	88 (76–99)	0 (0–0)	2322 (1931–2713)	5 (3–6)
Algeria	2033	90 (77–104)	0 (0–0)	2386 (1942–2830)	5 (3–6)
Algeria	2034	93 (78–108)	0 (0–0)	2449 (1950–2949)	5 (3–7)
Algeria	2035	95 (78–112)	0 (0–0)	2513 (1956–3070)	5 (3–7)
Algeria	2036	98 (79–116)	0 (0–0)	2577 (1960–3193)	5 (3–7)
American Samoa	2022	0 (0–0)	1 (1–1)	11 (11–11)	20 (19–21)
American Samoa	2023	0 (0–0)	1 (1–1)	11 (11–12)	20 (18–22)
American Samoa	2024	0 (0–0)	1 (1–1)	11 (11–12)	20 (17–22)
American Samoa	2025	0 (0–0)	1 (1–1)	12 (11–13)	20 (17–23)
American Samoa	2026	0 (0–0)	1 (1–1)	12 (11–13)	20 (16–23)
American Samoa	2027	0 (0–0)	1 (1–1)	12 (11–14)	20 (16–24)
American Samoa	2028	0 (0–0)	1 (1–1)	12 (11–14)	20 (16–24)
American Samoa	2029	0 (0–0)	1 (1–1)	13 (11–14)	20 (15–25)
American Samoa	2030	0 (0–0)	1 (0–1)	13 (11–15)	20 (15–25)
American Samoa	2031	0 (0–0)	1 (0–1)	13 (11–15)	20 (15–25)
American Samoa	2032	0 (0–0)	1 (0–1)	13 (11–15)	20 (14–25)
American Samoa	2033	0 (0–0)	1 (0–1)	14 (11–16)	20 (14–26)
American Samoa	2034	0 (0–1)	1 (0–1)	14 (12–16)	20 (14–26)
American Samoa	2035	0 (0–1)	1 (0–1)	14 (12–16)	20 (14–26)
American Samoa	2036	0 (0–1)	1 (0–1)	14 (12–17)	20 (13–26)
Andorra	2022	1 (1–2)	1 (1–1)	38 (36–40)	25 (24–26)
Andorra	2023	2 (1–2)	1 (1–1)	39 (36–41)	24 (23–26)
Andorra	2024	2 (1–2)	1 (1–1)	39 (36–42)	24 (22–26)
Andorra	2025	2 (1–2)	1 (1–1)	40 (36–43)	24 (21–27)

Andorra	2026	2 (1–2)	1 (1–1)	40 (36–44)	24 (20–27)
Andorra	2027	2 (1–2)	1 (1–1)	41 (36–45)	23 (20–27)
Andorra	2028	2 (1–2)	1 (1–1)	41 (37–46)	23 (19–27)
Andorra	2029	2 (1–2)	1 (1–1)	42 (37–47)	23 (19–27)
Andorra	2030	2 (1–2)	1 (1–1)	43 (37–48)	23 (18–27)
Andorra	2031	2 (1–2)	1 (1–1)	43 (37–49)	22 (18–27)
Andorra	2032	2 (2–2)	1 (1–1)	44 (38–50)	22 (17–27)
Andorra	2033	2 (2–2)	1 (1–1)	44 (38–50)	22 (17–27)
Andorra	2034	2 (2–2)	1 (1–1)	45 (38–51)	21 (16–27)
Andorra	2035	2 (2–2)	1 (1–1)	45 (39–52)	21 (16–27)
Andorra	2036	2 (2–2)	1 (1–1)	46 (39–53)	21 (15–27)
Angola	2022	80 (79–82)	1 (1–1)	2554 (2500–2608)	17 (16–18)
Angola	2023	84 (81–87)	1 (1–1)	2666 (2565–2767)	17 (16–18)
Angola	2024	88 (83–92)	1 (1–1)	2777 (2623–2931)	17 (15–19)
Angola	2025	91 (85–98)	1 (1–1)	2889 (2676–3102)	18 (15–20)
Angola	2026	95 (86–103)	1 (1–1)	3000 (2722–3278)	18 (14–21)
Angola	2027	98 (88–109)	1 (1–1)	3112 (2764–3460)	18 (14–22)
Angola	2028	102 (89–115)	1 (0–1)	3223 (2800–3646)	18 (13–23)
Angola	2029	106 (90–121)	1 (0–1)	3335 (2832–3837)	18 (13–24)
Angola	2030	109 (91–127)	1 (0–1)	3446 (2859–4033)	18 (12–25)
Angola	2031	113 (92–134)	1 (0–1)	3557 (2882–4233)	19 (11–26)
Angola	2032	117 (93–140)	1 (0–1)	3669 (2901–4436)	19 (10–27)
Angola	2033	120 (93–147)	1 (0–1)	3780 (2917–4644)	19 (9–29)
Angola	2034	124 (94–154)	1 (0–1)	3892 (2928–4855)	19 (8–30)
Angola	2035	127 (94–161)	1 (0–1)	4003 (2937–5070)	19 (7–31)
Angola	2036	131 (94–167)	1 (0–1)	4115 (2941–5288)	20 (6–33)
Antigua and Barbuda	2022	0 (0–0)	0 (0–0)	6 (5–6)	5 (4–5)
Antigua and Barbuda	2023	0 (0–0)	0 (0–0)	6 (5–7)	5 (4–6)
Antigua and Barbuda	2024	0 (0–0)	0 (0–0)	6 (5–7)	5 (4–6)
Antigua and Barbuda	2025	0 (0–0)	0 (0–0)	7 (5–8)	5 (4–6)
Antigua and Barbuda	2026	0 (0–0)	0 (0–0)	7 (5–8)	5 (4–6)
Antigua and Barbuda	2027	0 (0–0)	0 (0–0)	7 (5–9)	5 (4–6)
Antigua and Barbuda	2028	0 (0–0)	0 (0–0)	7 (5–9)	5 (3–6)

Antigua and Barbuda	2029	0 (0–0)	0 (0–0)	8 (5–10)	5 (3–6)
Antigua and Barbuda	2030	0 (0–0)	0 (0–0)	8 (5–10)	5 (3–7)
Antigua and Barbuda	2031	0 (0–0)	0 (0–0)	8 (5–11)	5 (3–7)
Antigua and Barbuda	2032	0 (0–0)	0 (0–0)	8 (5–12)	5 (3–7)
Antigua and Barbuda	2033	0 (0–1)	0 (0–0)	9 (5–12)	5 (3–7)
Antigua and Barbuda	2034	0 (0–1)	0 (0–0)	9 (5–13)	5 (3–7)
Antigua and Barbuda	2035	0 (0–1)	0 (0–0)	9 (5–13)	5 (3–7)
Antigua and Barbuda	2036	0 (0–1)	0 (0–0)	9 (5–14)	5 (3–7)
Argentina	2022	72 (68–76)	0 (0–0)	1915 (1805–2026)	4 (3–4)
Argentina	2023	73 (66–80)	0 (0–0)	1952 (1760–2145)	4 (3–4)
Argentina	2024	75 (65–84)	0 (0–0)	1990 (1729–2251)	4 (3–4)
Argentina	2025	76 (65–88)	0 (0–0)	2028 (1708–2347)	4 (3–4)
Argentina	2026	78 (64–91)	0 (0–0)	2065 (1694–2437)	4 (3–4)
Argentina	2027	79 (64–94)	0 (0–0)	2103 (1687–2520)	4 (2–5)
Argentina	2028	80 (64–97)	0 (0–0)	2142 (1683–2600)	4 (2–5)
Argentina	2029	82 (64–100)	0 (0–0)	2180 (1683–2676)	4 (2–5)
Argentina	2030	83 (64–103)	0 (0–0)	2218 (1686–2749)	4 (2–5)
Argentina	2031	85 (65–105)	0 (0–0)	2256 (1691–2820)	4 (2–5)
Argentina	2032	86 (65–108)	0 (0–0)	2294 (1698–2890)	4 (2–5)
Argentina	2033	88 (65–110)	0 (0–0)	2332 (1706–2958)	4 (2–5)
Argentina	2034	89 (66–113)	0 (0–0)	2370 (1716–3024)	4 (2–5)
Argentina	2035	91 (66–115)	0 (0–0)	2408 (1726–3089)	4 (2–5)
Argentina	2036	92 (67–118)	0 (0–0)	2446 (1738–3153)	4 (2–5)
Armenia	2022	34 (30–39)	1 (1–1)	895 (771–1018)	21 (18–24)
Armenia	2023	34 (28–41)	1 (1–1)	895 (720–1069)	22 (17–27)
Armenia	2024	34 (26–43)	1 (1–1)	895 (681–1109)	22 (17–28)
Armenia	2025	34 (25–44)	1 (1–1)	895 (648–1142)	23 (16–29)
Armenia	2026	34 (24–45)	1 (1–1)	895 (619–1171)	23 (16–30)
Armenia	2027	34 (23–46)	1 (1–1)	895 (592–1197)	23 (16–30)
Armenia	2028	34 (22–47)	1 (1–1)	895 (568–1221)	23 (16–30)
Armenia	2029	34 (21–48)	1 (1–1)	895 (546–1244)	23 (16–30)
Armenia	2030	34 (20–49)	1 (1–1)	895 (524–1265)	24 (16–31)
Armenia	2031	34 (19–50)	1 (1–1)	895 (504–1285)	24 (17–31)

Armenia	2032	34 (18–51)	1 (1–1)	895 (485–1304)	24 (17–31)
Armenia	2033	34 (18–51)	1 (1–1)	895 (467–1322)	24 (17–31)
Armenia	2034	34 (17–52)	1 (1–1)	895 (450–1340)	24 (17–31)
Armenia	2035	34 (16–53)	1 (1–1)	895 (433–1357)	24 (17–31)
Armenia	2036	34 (16–53)	1 (1–1)	895 (417–1373)	24 (17–31)
Australia	2022	168 (165–172)	0 (0–0)	4281 (4177–4385)	11 (11–11)
Australia	2023	173 (167–179)	0 (0–0)	4392 (4230–4553)	11 (11–12)
Australia	2024	178 (170–186)	0 (0–0)	4502 (4287–4717)	11 (11–12)
Australia	2025	183 (172–193)	0 (0–0)	4612 (4343–4881)	11 (11–12)
Australia	2026	188 (175–201)	0 (0–0)	4723 (4398–5047)	12 (11–12)
Australia	2027	193 (178–208)	0 (0–0)	4833 (4452–5213)	12 (11–12)
Australia	2028	198 (180–215)	0 (0–0)	4943 (4504–5382)	12 (11–13)
Australia	2029	202 (182–223)	0 (0–0)	5054 (4554–5553)	12 (11–13)
Australia	2030	207 (184–230)	0 (0–0)	5164 (4602–5726)	12 (11–13)
Australia	2031	212 (187–238)	0 (0–0)	5274 (4648–5900)	12 (11–13)
Australia	2032	217 (189–245)	0 (0–0)	5385 (4692–6077)	12 (11–14)
Australia	2033	222 (190–253)	0 (0–0)	5495 (4735–6255)	13 (11–14)
Australia	2034	227 (192–261)	0 (0–1)	5605 (4775–6435)	13 (12–14)
Australia	2035	232 (194–269)	0 (0–1)	5716 (4814–6617)	13 (12–14)
Australia	2036	236 (196–277)	0 (0–1)	5826 (4850–6801)	13 (12–14)
Austria	2022	94 (90–97)	1 (1–1)	2223 (2132–2315)	14 (13–15)
Austria	2023	96 (91–101)	1 (0–1)	2244 (2105–2383)	14 (13–15)
Austria	2024	98 (91–104)	1 (0–1)	2264 (2081–2448)	14 (12–15)
Austria	2025	99 (92–106)	1 (0–1)	2285 (2059–2511)	14 (12–16)
Austria	2026	101 (93–109)	1 (0–1)	2306 (2036–2575)	14 (11–16)
Austria	2027	103 (94–112)	1 (0–1)	2326 (2012–2640)	13 (11–16)
Austria	2028	105 (95–114)	1 (0–1)	2347 (1987–2706)	13 (10–17)
Austria	2029	107 (97–117)	1 (0–1)	2367 (1961–2773)	13 (10–17)
Austria	2030	108 (98–119)	1 (0–1)	2388 (1934–2841)	13 (9–17)
Austria	2031	110 (99–122)	1 (0–1)	2408 (1906–2911)	13 (9–18)
Austria	2032	112 (100–124)	1 (0–1)	2429 (1876–2982)	13 (8–18)
Austria	2033	114 (102–126)	1 (0–1)	2449 (1845–3054)	13 (7–19)
Austria	2034	116 (103–129)	1 (0–1)	2470 (1813–3127)	13 (7–19)

Austria	2035	118 (104–131)	1 (0–1)	2490 (1779–3202)	13 (6–20)
Austria	2036	119 (106–133)	1 (0–1)	2511 (1745–3277)	13 (6–20)
Azerbaijan	2022	99 (97–101)	1 (1–1)	2876 (2815–2937)	24 (23–24)
Azerbaijan	2023	102 (98–107)	1 (1–1)	2989 (2852–3125)	24 (22–25)
Azerbaijan	2024	106 (99–113)	1 (1–1)	3101 (2873–3329)	24 (21–26)
Azerbaijan	2025	109 (99–120)	1 (1–1)	3214 (2880–3548)	24 (21–27)
Azerbaijan	2026	113 (99–127)	1 (1–1)	3326 (2874–3779)	24 (20–27)
Azerbaijan	2027	116 (98–135)	1 (1–1)	3439 (2857–4021)	24 (19–28)
Azerbaijan	2028	120 (97–143)	1 (1–1)	3552 (2830–4273)	24 (19–29)
Azerbaijan	2029	123 (96–151)	1 (1–1)	3664 (2793–4535)	24 (18–29)
Azerbaijan	2030	127 (95–160)	1 (1–1)	3777 (2747–4806)	24 (18–30)
Azerbaijan	2031	131 (93–168)	1 (1–1)	3889 (2693–5086)	24 (17–30)
Azerbaijan	2032	134 (91–177)	1 (1–1)	4002 (2630–5374)	24 (17–31)
Azerbaijan	2033	138 (89–187)	1 (1–1)	4115 (2560–5669)	24 (16–31)
Azerbaijan	2034	141 (86–196)	1 (1–1)	4227 (2482–5972)	24 (16–32)
Azerbaijan	2035	145 (83–206)	1 (1–1)	4340 (2397–6282)	24 (16–32)
Azerbaijan	2036	148 (81–216)	1 (1–1)	4452 (2305–6600)	24 (15–33)
Bahamas	2022	1 (1–1)	0 (0–0)	26 (25–27)	6 (5–6)
Bahamas	2023	1 (1–1)	0 (0–0)	27 (25–29)	6 (5–6)
Bahamas	2024	1 (1–1)	0 (0–0)	27 (25–30)	6 (5–7)
Bahamas	2025	1 (1–1)	0 (0–0)	28 (25–31)	6 (5–7)
Bahamas	2026	1 (1–1)	0 (0–0)	29 (25–33)	6 (5–7)
Bahamas	2027	1 (1–1)	0 (0–0)	30 (25–34)	6 (5–7)
Bahamas	2028	1 (1–1)	0 (0–0)	30 (25–36)	6 (4–7)
Bahamas	2029	1 (1–1)	0 (0–0)	31 (25–38)	6 (4–7)
Bahamas	2030	1 (1–1)	0 (0–0)	32 (25–39)	6 (4–7)
Bahamas	2031	1 (1–1)	0 (0–0)	33 (25–41)	6 (4–7)
Bahamas	2032	1 (1–2)	0 (0–0)	33 (24–42)	6 (4–7)
Bahamas	2033	1 (1–2)	0 (0–0)	34 (24–44)	6 (4–7)
Bahamas	2034	1 (1–2)	0 (0–0)	35 (24–46)	6 (4–7)
Bahamas	2035	1 (1–2)	0 (0–0)	36 (24–48)	6 (4–7)
Bahamas	2036	1 (1–2)	0 (0–0)	36 (24–49)	6 (4–8)
Bahrain	2022	4 (4–4)	0 (0–0)	118 (115–121)	10 (10–11)

Bahrain	2023	5 (5–5)	0 (0–1)	139 (131–147)	11 (10–13)
Bahrain	2024	6 (5–6)	1 (0–1)	162 (146–178)	12 (9–14)
Bahrain	2025	6 (5–7)	1 (0–1)	186 (160–211)	13 (9–16)
Bahrain	2026	7 (6–8)	1 (0–1)	210 (173–247)	13 (9–18)
Bahrain	2027	8 (6–10)	1 (0–1)	235 (185–285)	14 (8–20)
Bahrain	2028	9 (7–11)	1 (0–1)	260 (195–325)	15 (7–23)
Bahrain	2029	10 (7–12)	1 (0–1)	285 (204–366)	16 (6–25)
Bahrain	2030	11 (7–14)	1 (0–1)	310 (211–409)	16 (5–28)
Bahrain	2031	11 (7–15)	1 (0–1)	335 (218–453)	17 (4–30)
Bahrain	2032	12 (8–17)	1 (0–1)	361 (223–498)	18 (3–33)
Bahrain	2033	13 (8–18)	1 (-0–2)	386 (228–544)	19 (2–36)
Bahrain	2034	14 (8–20)	1 (-0–2)	411 (231–592)	19 (1–38)
Bahrain	2035	15 (8–21)	1 (-0–2)	437 (233–640)	20 (-1–41)
Bahrain	2036	15 (8–23)	1 (-0–2)	462 (235–689)	21 (-2–44)
Bangladesh	2022	459 (450–469)	0 (0–0)	12040 (11843–12238)	8 (8–8)
Bangladesh	2023	478 (457–499)	0 (0–0)	12474 (12032–12916)	8 (7–9)
Bangladesh	2024	497 (461–532)	0 (0–0)	12908 (12168–13647)	8 (7–9)
Bangladesh	2025	515 (463–567)	0 (0–0)	13342 (12259–14424)	8 (7–9)
Bangladesh	2026	534 (464–605)	0 (0–0)	13775 (12310–15241)	8 (7–9)
Bangladesh	2027	553 (462–643)	0 (0–0)	14209 (12324–16095)	8 (7–9)
Bangladesh	2028	572 (459–684)	0 (0–0)	14643 (12304–16982)	8 (7–9)
Bangladesh	2029	590 (454–726)	0 (0–0)	15077 (12254–17900)	8 (7–9)
Bangladesh	2030	609 (448–769)	0 (0–0)	15511 (12174–18848)	8 (7–9)
Bangladesh	2031	628 (441–814)	0 (0–0)	15945 (12066–19823)	8 (7–10)
Bangladesh	2032	646 (433–860)	0 (0–0)	16378 (11932–20825)	8 (6–10)
Bangladesh	2033	665 (423–907)	0 (0–0)	16812 (11773–21852)	8 (6–10)
Bangladesh	2034	684 (412–956)	0 (0–0)	17246 (11589–22903)	8 (6–10)
Bangladesh	2035	703 (400–1005)	0 (0–0)	17680 (11382–23977)	8 (6–10)
Bangladesh	2036	721 (387–1056)	0 (0–0)	18114 (11153–25074)	8 (6–10)
Barbados	2022	1 (1–1)	0 (0–0)	14 (14–15)	3 (3–3)
Barbados	2023	1 (1–1)	0 (0–0)	15 (14–16)	3 (3–3)
Barbados	2024	1 (1–1)	0 (0–0)	15 (13–16)	3 (2–3)
Barbados	2025	1 (1–1)	0 (0–0)	15 (13–16)	3 (2–3)

Barbados	2026	1 (1–1)	0 (0–0)	15 (13–17)	3 (2–3)
Barbados	2027	1 (1–1)	0 (0–0)	15 (14–17)	3 (2–3)
Barbados	2028	1 (1–1)	0 (0–0)	16 (14–17)	3 (2–3)
Barbados	2029	1 (1–1)	0 (0–0)	16 (14–18)	3 (2–3)
Barbados	2030	1 (1–1)	0 (0–0)	16 (14–18)	3 (2–3)
Barbados	2031	1 (1–1)	0 (0–0)	16 (14–18)	3 (2–4)
Barbados	2032	1 (1–1)	0 (0–0)	16 (14–19)	3 (2–4)
Barbados	2033	1 (1–1)	0 (0–0)	16 (14–19)	3 (2–4)
Barbados	2034	1 (1–1)	0 (0–0)	17 (14–19)	3 (2–4)
Barbados	2035	1 (1–1)	0 (0–0)	17 (14–20)	3 (2–4)
Barbados	2036	1 (1–1)	0 (0–0)	17 (14–20)	3 (2–4)
Belarus	2022	61 (57–65)	0 (0–0)	1660 (1543–1776)	10 (10–11)
Belarus	2023	61 (56–67)	0 (0–0)	1660 (1495–1824)	10 (9–11)
Belarus	2024	62 (55–69)	0 (0–0)	1660 (1458–1861)	10 (9–11)
Belarus	2025	63 (55–70)	0 (0–0)	1660 (1427–1893)	10 (9–11)
Belarus	2026	63 (55–72)	0 (0–0)	1660 (1400–1920)	10 (9–11)
Belarus	2027	64 (55–74)	0 (0–0)	1660 (1375–1945)	10 (9–11)
Belarus	2028	65 (54–75)	0 (0–0)	1660 (1352–1968)	10 (9–11)
Belarus	2029	65 (54–76)	0 (0–0)	1660 (1331–1989)	10 (9–11)
Belarus	2030	66 (54–78)	0 (0–0)	1660 (1311–2009)	10 (9–11)
Belarus	2031	67 (54–79)	0 (0–0)	1660 (1292–2028)	10 (9–11)
Belarus	2032	67 (54–80)	0 (0–0)	1660 (1274–2046)	10 (9–11)
Belarus	2033	68 (54–81)	0 (0–0)	1660 (1257–2063)	10 (9–11)
Belarus	2034	69 (55–82)	0 (0–0)	1660 (1240–2080)	10 (9–11)
Belarus	2035	69 (55–84)	0 (0–0)	1660 (1224–2095)	10 (9–11)
Belarus	2036	70 (55–85)	0 (0–0)	1660 (1209–2111)	10 (9–11)
Belgium	2022	90 (85–95)	0 (0–0)	2057 (1946–2168)	10 (9–11)
Belgium	2023	90 (81–100)	0 (0–0)	2057 (1821–2293)	10 (9–11)
Belgium	2024	90 (77–104)	0 (0–0)	2057 (1743–2372)	10 (8–12)
Belgium	2025	90 (73–107)	0 (0–0)	2057 (1680–2434)	10 (8–12)
Belgium	2026	90 (70–111)	0 (0–0)	2057 (1627–2488)	10 (8–12)
Belgium	2027	90 (67–114)	0 (0–0)	2057 (1579–2535)	10 (7–13)
Belgium	2028	90 (65–116)	0 (0–0)	2057 (1536–2579)	10 (7–13)

Belgium	2029	91 (62–119)	0 (0–0)	2057 (1496–2619)	10 (7–13)
Belgium	2030	91 (60–121)	0 (0–0)	2057 (1458–2656)	10 (7–13)
Belgium	2031	91 (58–123)	0 (0–0)	2057 (1423–2691)	10 (7–14)
Belgium	2032	91 (56–125)	0 (0–0)	2057 (1390–2724)	10 (6–14)
Belgium	2033	91 (54–127)	0 (0–0)	2057 (1358–2756)	10 (6–14)
Belgium	2034	91 (52–129)	0 (0–0)	2057 (1328–2786)	10 (6–14)
Belgium	2035	91 (51–130)	0 (0–0)	2057 (1299–2816)	10 (6–14)
Belgium	2036	91 (49–132)	0 (0–0)	2057 (1271–2844)	10 (6–14)
Belize	2022	1 (1–1)	0 (0–0)	15 (15–16)	5 (4–5)
Belize	2023	1 (1–1)	0 (0–0)	16 (15–17)	5 (4–5)
Belize	2024	1 (1–1)	0 (0–0)	16 (15–18)	5 (4–6)
Belize	2025	1 (1–1)	0 (0–0)	17 (15–19)	5 (4–6)
Belize	2026	1 (1–1)	0 (0–0)	17 (15–19)	5 (3–6)
Belize	2027	1 (1–1)	0 (0–0)	17 (15–20)	5 (3–6)
Belize	2028	1 (1–1)	0 (0–0)	18 (15–21)	5 (3–6)
Belize	2029	1 (1–1)	0 (0–0)	18 (15–21)	5 (3–6)
Belize	2030	1 (1–1)	0 (0–0)	19 (15–22)	5 (3–6)
Belize	2031	1 (1–1)	0 (0–0)	19 (15–22)	5 (3–6)
Belize	2032	1 (1–1)	0 (0–0)	19 (15–23)	5 (3–6)
Belize	2033	1 (1–1)	0 (0–0)	20 (16–24)	5 (3–6)
Belize	2034	1 (1–1)	0 (0–0)	20 (16–24)	5 (3–6)
Belize	2035	1 (1–1)	0 (0–0)	20 (16–25)	5 (3–6)
Belize	2036	1 (1–1)	0 (0–0)	21 (16–25)	5 (3–6)
Benin	2022	20 (19–20)	0 (0–0)	594 (571–616)	10 (9–10)
Benin	2023	20 (18–21)	0 (0–0)	604 (553–655)	9 (8–11)
Benin	2024	20 (18–23)	0 (0–0)	614 (530–699)	9 (7–12)
Benin	2025	20 (17–24)	0 (0–0)	625 (501–749)	9 (6–12)
Benin	2026	21 (16–26)	0 (0–0)	635 (467–803)	9 (4–13)
Benin	2027	21 (14–28)	0 (0–1)	646 (429–862)	9 (3–15)
Benin	2028	21 (13–30)	0 (0–1)	656 (388–924)	9 (1–16)
Benin	2029	22 (12–32)	0 (0–1)	667 (343–990)	8 (-0–17)
Benin	2030	22 (10–34)	0 (-0–1)	677 (294–1060)	8 (-2–19)
Benin	2031	22 (9–36)	0 (-0–1)	687 (243–1132)	8 (-4–20)

Benin	2032	23 (7–38)	0 (-0–1)	698 (188–1208)	8 (-6–22)
Benin	2033	23 (5–41)	0 (-0–1)	708 (130–1286)	8 (-8–23)
Benin	2034	23 (3–43)	0 (-0–1)	719 (70–1368)	7 (-10–25)
Benin	2035	24 (1–46)	0 (-0–1)	729 (7–1452)	7 (-12–27)
Benin	2036	24 (-1–48)	0 (-0–1)	740 (-59–1538)	7 (-15–29)
Bermuda	2022	0 (0–0)	0 (0–0)	4 (4–5)	3 (3–4)
Bermuda	2023	0 (0–0)	0 (0–0)	4 (4–5)	3 (3–4)
Bermuda	2024	0 (0–0)	0 (0–0)	4 (4–5)	3 (3–4)
Bermuda	2025	0 (0–0)	0 (0–0)	4 (4–5)	3 (2–4)
Bermuda	2026	0 (0–0)	0 (0–0)	4 (4–5)	3 (2–5)
Bermuda	2027	0 (0–0)	0 (0–0)	4 (4–5)	3 (2–5)
Bermuda	2028	0 (0–0)	0 (0–0)	4 (3–5)	3 (2–5)
Bermuda	2029	0 (0–0)	0 (0–0)	4 (3–5)	3 (1–5)
Bermuda	2030	0 (0–0)	0 (0–0)	4 (3–5)	3 (1–6)
Bermuda	2031	0 (0–0)	0 (0–0)	4 (3–5)	3 (1–6)
Bermuda	2032	0 (0–0)	0 (0–0)	4 (3–5)	3 (0–6)
Bermuda	2033	0 (0–0)	0 (0–0)	4 (3–5)	3 (0–6)
Bermuda	2034	0 (0–0)	0 (-0–0)	4 (3–5)	3 (-0–7)
Bermuda	2035	0 (0–0)	0 (-0–0)	4 (3–5)	3 (-0–7)
Bermuda	2036	0 (0–0)	0 (-0–0)	4 (3–5)	3 (-1–7)
Bhutan	2022	2 (2–2)	0 (0–0)	43 (42–43)	7 (7–7)
Bhutan	2023	2 (2–2)	0 (0–0)	44 (43–45)	7 (7–7)
Bhutan	2024	2 (2–2)	0 (0–0)	45 (44–46)	7 (6–7)
Bhutan	2025	2 (2–2)	0 (0–0)	46 (45–48)	7 (6–7)
Bhutan	2026	2 (2–2)	0 (0–0)	47 (46–49)	7 (6–7)
Bhutan	2027	2 (2–2)	0 (0–0)	48 (46–51)	7 (6–7)
Bhutan	2028	2 (2–2)	0 (0–0)	50 (47–52)	7 (6–7)
Bhutan	2029	2 (2–2)	0 (0–0)	51 (48–54)	7 (6–7)
Bhutan	2030	2 (2–2)	0 (0–0)	52 (48–55)	7 (6–7)
Bhutan	2031	2 (2–2)	0 (0–0)	53 (49–57)	7 (6–7)
Bhutan	2032	2 (2–2)	0 (0–0)	54 (50–58)	7 (6–7)
Bhutan	2033	2 (2–2)	0 (0–0)	55 (50–60)	7 (6–7)
Bhutan	2034	2 (2–2)	0 (0–0)	56 (51–61)	7 (6–7)

Bhutan	2035	2 (2–2)	0 (0–0)	57 (51–63)	7 (6–7)
Bhutan	2036	2 (2–2)	0 (0–0)	58 (52–65)	7 (6–7)
Bolivia (Plurinational State of)	2022	20 (20–20)	0 (0–0)	500 (492–509)	5 (5–5)
Bolivia (Plurinational State of)	2023	21 (20–21)	0 (0–0)	517 (502–533)	5 (5–5)
Bolivia (Plurinational State of)	2024	21 (21–22)	0 (0–0)	531 (513–550)	5 (5–5)
Bolivia (Plurinational State of)	2025	22 (21–22)	0 (0–0)	545 (522–569)	5 (5–6)
Bolivia (Plurinational State of)	2026	22 (21–23)	0 (0–0)	559 (530–588)	5 (5–6)
Bolivia (Plurinational State of)	2027	23 (22–24)	0 (0–0)	573 (537–609)	5 (5–6)
Bolivia (Plurinational State of)	2028	23 (22–25)	0 (0–0)	587 (544–630)	5 (5–6)
Bolivia (Plurinational State of)	2029	24 (22–26)	0 (0–0)	601 (550–652)	5 (5–6)
Bolivia (Plurinational State of)	2030	24 (23–26)	0 (0–0)	614 (555–674)	5 (4–6)
Bolivia (Plurinational State of)	2031	25 (23–27)	0 (0–0)	628 (560–697)	5 (4–6)
Bolivia (Plurinational State of)	2032	26 (23–28)	0 (0–0)	642 (564–720)	5 (4–6)
Bolivia (Plurinational State of)	2033	26 (23–29)	0 (0–0)	656 (568–744)	5 (4–6)
Bolivia (Plurinational State of)	2034	27 (23–30)	0 (0–0)	670 (572–768)	5 (4–6)
Bolivia (Plurinational State of)	2035	27 (24–31)	0 (0–0)	684 (575–793)	5 (4–6)
Bolivia (Plurinational State of)	2036	28 (24–32)	0 (0–0)	698 (578–818)	5 (4–6)
Bosnia and Herzegovina	2022	49 (47–51)	1 (1–1)	1185 (1122–1247)	20 (18–21)
Bosnia and Herzegovina	2023	49 (46–52)	1 (1–1)	1185 (1096–1273)	20 (18–21)
Bosnia and Herzegovina	2024	50 (46–54)	1 (1–1)	1185 (1077–1292)	20 (18–21)
Bosnia and Herzegovina	2025	50 (46–55)	1 (1–1)	1185 (1060–1309)	20 (17–22)
Bosnia and Herzegovina	2026	51 (46–56)	1 (1–1)	1185 (1045–1324)	20 (17–22)
Bosnia and Herzegovina	2027	51 (46–57)	1 (1–1)	1185 (1032–1337)	20 (17–22)
Bosnia and Herzegovina	2028	52 (46–57)	1 (1–1)	1185 (1020–1349)	20 (17–22)
Bosnia and Herzegovina	2029	52 (46–58)	1 (1–1)	1185 (1008–1361)	20 (16–23)
Bosnia and Herzegovina	2030	53 (46–59)	1 (1–1)	1185 (998–1371)	20 (16–23)
Bosnia and Herzegovina	2031	53 (46–60)	1 (1–1)	1185 (988–1381)	20 (16–23)
Bosnia and Herzegovina	2032	54 (46–61)	1 (1–1)	1185 (978–1391)	20 (16–23)
Bosnia and Herzegovina	2033	54 (46–62)	1 (1–1)	1185 (969–1400)	20 (16–23)
Bosnia and Herzegovina	2034	55 (47–62)	1 (1–1)	1185 (960–1409)	20 (16–23)
Bosnia and Herzegovina	2035	55 (47–63)	1 (1–1)	1185 (952–1418)	20 (16–24)
Bosnia and Herzegovina	2036	55 (47–64)	1 (1–1)	1185 (943–1426)	20 (15–24)
Botswana	2022	9 (8–9)	1 (1–1)	269 (261–277)	15 (14–16)

Botswana	2023	9 (8–10)	1 (1–1)	278 (260–296)	15 (13–17)
Botswana	2024	9 (8–10)	1 (0–1)	287 (257–317)	15 (12–19)
Botswana	2025	10 (8–11)	1 (0–1)	296 (252–339)	15 (10–21)
Botswana	2026	10 (8–12)	1 (0–1)	305 (246–364)	16 (9–23)
Botswana	2027	10 (8–12)	1 (0–1)	314 (238–389)	16 (6–25)
Botswana	2028	10 (8–13)	1 (0–1)	323 (229–416)	16 (4–28)
Botswana	2029	11 (7–14)	1 (0–1)	331 (218–445)	16 (2–31)
Botswana	2030	11 (7–15)	1 (0–1)	340 (206–474)	16 (-1–34)
Botswana	2031	11 (7–16)	1 (-0–1)	349 (194–505)	16 (-4–37)
Botswana	2032	12 (6–17)	1 (-0–1)	358 (180–537)	17 (-7–40)
Botswana	2033	12 (6–18)	1 (-0–2)	367 (165–570)	17 (-10–43)
Botswana	2034	12 (5–19)	1 (-0–2)	376 (149–603)	17 (-13–47)
Botswana	2035	12 (5–20)	1 (-0–2)	385 (132–638)	17 (-16–51)
Botswana	2036	13 (4–21)	1 (-1–2)	394 (114–673)	17 (-20–55)
Brazil	2022	476 (460–492)	0 (0–0)	12174 (11731–12616)	5 (4–5)
Brazil	2023	484 (462–507)	0 (0–0)	12358 (11732–12983)	5 (4–5)
Brazil	2024	492 (465–520)	0 (0–0)	12542 (11776–13308)	4 (4–5)
Brazil	2025	500 (469–532)	0 (0–0)	12726 (11842–13611)	4 (4–5)
Brazil	2026	508 (473–544)	0 (0–0)	12910 (11922–13899)	4 (4–5)
Brazil	2027	517 (477–556)	0 (0–0)	13095 (12011–14178)	4 (4–5)
Brazil	2028	525 (482–567)	0 (0–0)	13279 (12109–14449)	4 (4–5)
Brazil	2029	533 (488–578)	0 (0–0)	13463 (12212–14714)	4 (3–5)
Brazil	2030	541 (493–589)	0 (0–0)	13647 (12320–14974)	4 (3–5)
Brazil	2031	549 (498–599)	0 (0–0)	13831 (12433–15230)	4 (3–5)
Brazil	2032	557 (504–610)	0 (0–0)	14016 (12549–15482)	4 (3–5)
Brazil	2033	565 (510–620)	0 (0–0)	14200 (12668–15732)	4 (3–5)
Brazil	2034	573 (516–631)	0 (0–0)	14384 (12790–15979)	4 (3–5)
Brazil	2035	581 (521–641)	0 (0–0)	14568 (12914–16223)	4 (3–5)
Brazil	2036	589 (527–651)	0 (0–0)	14752 (13040–16465)	4 (3–5)
Brunei Darussalam	2022	3 (3–3)	1 (1–1)	100 (98–102)	23 (22–24)
Brunei Darussalam	2023	4 (3–4)	1 (1–1)	106 (102–111)	24 (21–26)
Brunei Darussalam	2024	4 (3–4)	1 (1–1)	112 (104–120)	24 (20–29)
Brunei Darussalam	2025	4 (4–4)	1 (1–1)	118 (107–129)	25 (19–32)

Brunei Darussalam	2026	4 (4–5)	1 (1–1)	124 (109–139)	26 (17–35)
Brunei Darussalam	2027	4 (4–5)	1 (1–1)	130 (110–149)	26 (15–38)
Brunei Darussalam	2028	5 (4–5)	1 (1–1)	136 (111–160)	27 (12–42)
Brunei Darussalam	2029	5 (4–6)	1 (0–1)	141 (112–171)	28 (10–45)
Brunei Darussalam	2030	5 (4–6)	1 (0–1)	147 (113–182)	28 (7–49)
Brunei Darussalam	2031	5 (4–7)	1 (0–1)	153 (113–193)	29 (4–54)
Brunei Darussalam	2032	5 (4–7)	1 (0–1)	159 (113–205)	30 (1–58)
Brunei Darussalam	2033	6 (4–8)	1 (0–1)	165 (113–217)	30 (-2–62)
Brunei Darussalam	2034	6 (4–8)	1 (0–2)	171 (112–230)	31 (-5–67)
Brunei Darussalam	2035	6 (4–8)	1 (0–2)	177 (112–242)	32 (-9–72)
Brunei Darussalam	2036	6 (4–9)	1 (0–2)	183 (111–255)	32 (-12–77)
Bulgaria	2022	50 (41–59)	0 (0–0)	1418 (1163–1672)	12 (10–14)
Bulgaria	2023	50 (33–67)	0 (0–1)	1411 (933–1889)	12 (8–16)
Bulgaria	2024	50 (26–74)	0 (0–1)	1408 (721–2094)	12 (7–18)
Bulgaria	2025	50 (19–80)	0 (0–1)	1405 (530–2281)	12 (5–19)
Bulgaria	2026	50 (13–86)	0 (0–1)	1404 (357–2451)	12 (4–20)
Bulgaria	2027	50 (8–92)	0 (0–1)	1403 (200–2607)	12 (3–22)
Bulgaria	2028	50 (3–96)	0 (0–1)	1403 (57–2749)	12 (2–23)
Bulgaria	2029	50 (-1–101)	0 (0–1)	1403 (-74–2880)	12 (1–24)
Bulgaria	2030	50 (-6–105)	0 (-0–1)	1403 (-196–3002)	12 (-0–25)
Bulgaria	2031	50 (-10–109)	0 (-0–1)	1403 (-311–3116)	12 (-1–25)
Bulgaria	2032	50 (-13–113)	0 (-0–1)	1402 (-418–3223)	12 (-2–26)
Bulgaria	2033	50 (-17–116)	0 (-0–1)	1402 (-520–3324)	12 (-3–27)
Bulgaria	2034	50 (-20–120)	0 (-0–1)	1402 (-616–3421)	12 (-4–28)
Bulgaria	2035	50 (-23–123)	0 (-0–1)	1402 (-709–3513)	12 (-4–28)
Bulgaria	2036	50 (-26–126)	0 (-0–1)	1402 (-797–3602)	12 (-5–29)
Burkina Faso	2022	69 (68–71)	1 (1–1)	2287 (2242–2331)	20 (19–21)
Burkina Faso	2023	69 (67–72)	1 (1–1)	2286 (2194–2378)	20 (18–21)
Burkina Faso	2024	69 (65–73)	1 (1–1)	2269 (2129–2409)	20 (17–22)
Burkina Faso	2025	68 (63–73)	1 (1–1)	2239 (2055–2423)	19 (16–23)
Burkina Faso	2026	67 (61–73)	1 (1–1)	2200 (1979–2421)	19 (15–24)
Burkina Faso	2027	66 (59–73)	1 (0–1)	2155 (1906–2405)	19 (14–25)
Burkina Faso	2028	65 (58–72)	1 (0–1)	2110 (1841–2379)	19 (12–26)

Burkina Faso	2029	64 (56–71)	1 (0–1)	2067 (1786–2348)	19 (11–27)
Burkina Faso	2030	63 (55–70)	1 (0–1)	2030 (1744–2317)	19 (10–28)
Burkina Faso	2031	62 (55–70)	1 (0–1)	2001 (1713–2289)	19 (9–29)
Burkina Faso	2032	62 (54–70)	1 (0–1)	1980 (1692–2268)	19 (7–30)
Burkina Faso	2033	62 (54–70)	1 (0–1)	1969 (1681–2258)	19 (6–31)
Burkina Faso	2034	62 (54–70)	1 (0–1)	1967 (1677–2258)	19 (5–32)
Burkina Faso	2035	63 (55–70)	1 (0–1)	1974 (1679–2268)	18 (4–33)
Burkina Faso	2036	63 (55–71)	1 (0–1)	1987 (1687–2286)	18 (2–34)
Burundi	2022	5 (5–5)	0 (0–0)	155 (152–158)	3 (3–3)
Burundi	2023	5 (5–5)	0 (0–0)	160 (154–167)	3 (2–3)
Burundi	2024	5 (5–6)	0 (0–0)	166 (155–176)	3 (2–3)
Burundi	2025	6 (5–6)	0 (0–0)	171 (155–186)	3 (2–3)
Burundi	2026	6 (5–6)	0 (0–0)	176 (155–197)	3 (2–3)
Burundi	2027	6 (5–7)	0 (0–0)	181 (154–208)	3 (2–3)
Burundi	2028	6 (5–7)	0 (0–0)	187 (153–220)	3 (2–4)
Burundi	2029	6 (5–8)	0 (0–0)	192 (152–233)	3 (1–4)
Burundi	2030	6 (5–8)	0 (0–0)	197 (150–245)	3 (1–4)
Burundi	2031	7 (5–8)	0 (0–0)	203 (147–258)	3 (1–4)
Burundi	2032	7 (5–9)	0 (0–0)	208 (144–272)	3 (1–5)
Burundi	2033	7 (5–9)	0 (0–0)	213 (141–286)	3 (1–5)
Burundi	2034	7 (5–10)	0 (0–0)	219 (138–300)	3 (0–5)
Burundi	2035	7 (5–10)	0 (0–0)	224 (134–314)	3 (0–5)
Burundi	2036	8 (4–11)	0 (0–0)	229 (129–329)	3 (-0–6)
Cabo Verde	2022	2 (2–2)	0 (0–0)	64 (62–66)	12 (12–13)
Cabo Verde	2023	2 (2–2)	0 (0–0)	66 (63–69)	12 (11–13)
Cabo Verde	2024	2 (2–3)	0 (0–0)	68 (64–73)	12 (11–14)
Cabo Verde	2025	2 (2–3)	0 (0–1)	70 (65–76)	12 (11–14)
Cabo Verde	2026	2 (2–3)	0 (0–1)	73 (66–80)	12 (11–14)
Cabo Verde	2027	3 (2–3)	0 (0–1)	75 (66–83)	12 (10–15)
Cabo Verde	2028	3 (2–3)	0 (0–1)	77 (67–87)	12 (10–15)
Cabo Verde	2029	3 (2–3)	0 (0–1)	79 (67–91)	12 (10–15)
Cabo Verde	2030	3 (2–3)	0 (0–1)	82 (68–95)	12 (10–15)
Cabo Verde	2031	3 (2–4)	0 (0–1)	84 (68–99)	12 (10–15)

Cabo Verde	2032	3 (2–4)	0 (0–1)	86 (69–103)	12 (9–15)
Cabo Verde	2033	3 (2–4)	0 (0–1)	88 (69–107)	12 (9–16)
Cabo Verde	2034	3 (2–4)	0 (0–1)	90 (69–112)	12 (9–16)
Cabo Verde	2035	3 (2–4)	0 (0–1)	93 (69–116)	12 (9–16)
Cabo Verde	2036	3 (2–4)	0 (0–1)	95 (69–120)	12 (9–16)
Cambodia	2022	99 (99–100)	1 (1–1)	2875 (2851–2900)	20 (20–21)
Cambodia	2023	103 (101–105)	1 (1–1)	2974 (2921–3026)	21 (20–21)
Cambodia	2024	106 (103–109)	1 (1–1)	3069 (2984–3154)	21 (19–22)
Cambodia	2025	109 (104–113)	1 (1–1)	3161 (3040–3282)	21 (19–23)
Cambodia	2026	112 (106–118)	1 (1–1)	3250 (3091–3409)	21 (19–23)
Cambodia	2027	114 (107–122)	1 (1–1)	3336 (3139–3534)	21 (18–24)
Cambodia	2028	117 (108–126)	1 (1–1)	3420 (3182–3659)	21 (17–25)
Cambodia	2029	119 (109–130)	1 (1–1)	3502 (3223–3782)	21 (17–26)
Cambodia	2030	122 (110–133)	1 (1–1)	3582 (3261–3903)	21 (16–26)
Cambodia	2031	124 (111–137)	1 (1–1)	3660 (3298–4022)	21 (16–27)
Cambodia	2032	127 (112–141)	1 (1–1)	3736 (3332–4140)	22 (15–28)
Cambodia	2033	129 (113–144)	1 (0–1)	3811 (3366–4256)	22 (14–29)
Cambodia	2034	131 (114–148)	1 (0–1)	3884 (3398–4371)	22 (14–29)
Cambodia	2035	133 (115–151)	1 (0–1)	3956 (3429–4484)	22 (13–30)
Cambodia	2036	135 (116–154)	1 (0–1)	4027 (3459–4595)	22 (13–31)
Cameroon	2022	69 (67–70)	0 (0–0)	2330 (2284–2376)	14 (13–15)
Cameroon	2023	69 (66–72)	0 (0–1)	2357 (2260–2454)	14 (13–15)
Cameroon	2024	69 (65–74)	0 (0–1)	2368 (2216–2521)	14 (12–16)
Cameroon	2025	69 (63–75)	0 (0–1)	2366 (2157–2574)	14 (11–17)
Cameroon	2026	69 (61–76)	0 (0–1)	2349 (2089–2609)	13 (9–17)
Cameroon	2027	68 (59–76)	0 (0–1)	2321 (2016–2626)	13 (8–18)
Cameroon	2028	66 (57–76)	0 (0–1)	2283 (1942–2624)	13 (7–19)
Cameroon	2029	65 (55–75)	0 (0–1)	2239 (1871–2607)	13 (6–20)
Cameroon	2030	64 (53–74)	0 (0–1)	2192 (1805–2578)	13 (5–21)
Cameroon	2031	62 (51–73)	0 (0–1)	2143 (1747–2540)	13 (4–22)
Cameroon	2032	61 (50–72)	0 (0–1)	2098 (1697–2498)	13 (3–23)
Cameroon	2033	60 (49–71)	0 (0–1)	2057 (1655–2458)	13 (1–24)
Cameroon	2034	59 (48–70)	0 (0–1)	2023 (1621–2424)	13 (0–25)

Cameroon	2035	58 (47–70)	0 (0–1)	1997 (1594–2400)	13 (-1–26)
Cameroon	2036	58 (47–69)	0 (-0–1)	1981 (1573–2389)	13 (-2–27)
Canada	2022	327 (320–335)	0 (0–0)	7521 (7333–7708)	11 (11–11)
Canada	2023	336 (324–349)	0 (0–0)	7695 (7388–8001)	11 (11–12)
Canada	2024	346 (328–363)	0 (0–0)	7869 (7442–8296)	11 (11–12)
Canada	2025	355 (331–378)	0 (0–0)	8043 (7490–8596)	11 (11–12)
Canada	2026	364 (334–393)	0 (0–1)	8217 (7531–8903)	12 (11–12)
Canada	2027	373 (337–409)	0 (0–1)	8391 (7564–9218)	12 (11–13)
Canada	2028	382 (339–424)	0 (0–1)	8565 (7591–9540)	12 (11–13)
Canada	2029	391 (341–441)	0 (0–1)	8739 (7610–9869)	12 (11–13)
Canada	2030	400 (343–457)	0 (0–1)	8913 (7623–10204)	12 (11–13)
Canada	2031	409 (344–474)	1 (0–1)	9088 (7629–10546)	12 (11–13)
Canada	2032	418 (345–491)	1 (0–1)	9262 (7628–10895)	12 (11–14)
Canada	2033	427 (346–508)	1 (0–1)	9436 (7622–11250)	12 (11–14)
Canada	2034	436 (346–526)	1 (0–1)	9610 (7609–11611)	13 (11–14)
Canada	2035	445 (346–544)	1 (0–1)	9784 (7591–11977)	13 (11–14)
Canada	2036	454 (346–562)	1 (0–1)	9958 (7566–12350)	13 (11–14)
Central African Republic	2022	9 (9–9)	0 (0–0)	290 (280–300)	10 (9–11)
Central African Republic	2023	9 (8–10)	0 (0–0)	292 (271–314)	10 (9–11)
Central African Republic	2024	9 (8–10)	0 (0–0)	295 (260–329)	10 (8–12)
Central African Republic	2025	9 (8–11)	0 (0–0)	297 (248–345)	10 (7–13)
Central African Republic	2026	9 (7–11)	0 (0–1)	298 (235–362)	10 (6–14)
Central African Republic	2027	9 (7–12)	0 (0–1)	300 (221–380)	10 (5–15)
Central African Republic	2028	9 (6–12)	0 (0–1)	301 (206–397)	10 (4–16)
Central African Republic	2029	9 (6–13)	0 (0–1)	303 (191–415)	10 (2–17)
Central African Republic	2030	9 (5–13)	0 (0–1)	304 (176–432)	10 (1–18)
Central African Republic	2031	9 (5–14)	0 (-0–1)	305 (161–450)	9 (-0–19)
Central African Republic	2032	9 (4–15)	0 (-0–1)	306 (145–467)	9 (-1–20)
Central African Republic	2033	9 (4–15)	0 (-0–1)	307 (130–484)	9 (-3–21)
Central African Republic	2034	10 (3–16)	0 (-0–1)	308 (114–501)	9 (-4–23)
Central African Republic	2035	10 (3–16)	1 (-0–1)	309 (99–518)	9 (-5–24)
Central African Republic	2036	10 (2–17)	1 (-0–1)	309 (84–535)	9 (-6–25)
Chad	2022	38 (37–40)	1 (1–1)	1182 (1138–1226)	17 (16–18)

Chad	2023	39 (36–42)	1 (1–1)	1195 (1108–1281)	17 (15–19)
Chad	2024	39 (35–43)	1 (1–1)	1200 (1073–1327)	17 (13–21)
Chad	2025	39 (34–44)	1 (0–1)	1198 (1037–1359)	17 (12–22)
Chad	2026	39 (33–45)	1 (0–1)	1191 (1004–1379)	17 (10–24)
Chad	2027	39 (33–45)	1 (0–1)	1181 (975–1387)	17 (8–26)
Chad	2028	39 (32–45)	1 (0–1)	1169 (951–1387)	17 (6–29)
Chad	2029	38 (32–45)	1 (0–1)	1157 (932–1381)	17 (3–31)
Chad	2030	38 (31–44)	1 (0–1)	1145 (918–1372)	17 (1–33)
Chad	2031	37 (31–44)	1 (0–1)	1135 (907–1363)	17 (-2–36)
Chad	2032	37 (31–44)	1 (-0–1)	1126 (898–1354)	17 (-5–39)
Chad	2033	37 (30–44)	1 (-0–2)	1120 (892–1349)	17 (-8–42)
Chad	2034	37 (30–44)	1 (-0–2)	1117 (888–1345)	17 (-11–45)
Chad	2035	37 (30–44)	1 (-0–2)	1115 (885–1345)	17 (-14–48)
Chad	2036	37 (30–44)	1 (-0–2)	1115 (884–1346)	17 (-17–51)
Chile	2022	77 (74–80)	0 (0–0)	1925 (1844–2007)	7 (7–8)
Chile	2023	79 (74–84)	0 (0–0)	1999 (1857–2140)	8 (7–8)
Chile	2024	82 (75–88)	0 (0–0)	2065 (1897–2233)	8 (7–8)
Chile	2025	84 (75–94)	0 (0–0)	2118 (1905–2332)	8 (7–9)
Chile	2026	86 (74–99)	0 (0–0)	2184 (1919–2449)	8 (7–9)
Chile	2027	89 (74–104)	0 (0–0)	2248 (1939–2557)	8 (7–9)
Chile	2028	92 (74–110)	0 (0–0)	2307 (1946–2668)	8 (7–9)
Chile	2029	94 (73–116)	0 (0–0)	2370 (1954–2786)	8 (7–9)
Chile	2030	97 (72–122)	0 (0–0)	2433 (1963–2903)	8 (7–10)
Chile	2031	99 (71–128)	0 (0–0)	2494 (1965–3022)	8 (7–10)
Chile	2032	102 (69–134)	0 (0–0)	2556 (1967–3146)	8 (7–10)
Chile	2033	104 (68–141)	0 (0–0)	2619 (1967–3270)	8 (7–10)
Chile	2034	107 (66–147)	0 (0–0)	2680 (1965–3396)	8 (7–10)
Chile	2035	109 (64–154)	0 (0–0)	2742 (1960–3524)	9 (7–10)
Chile	2036	112 (62–161)	0 (0–0)	2804 (1954–3654)	9 (7–10)
China	2022	3926 (23386–24465	1 (1–1)	717134 (699887–734382	33 (32–34)
China	2023	4337 (23131–25544	1 (1–1)	729753 (689980–769525	33 (30–35)
China	2024	4636 (22783–26490	1 (1–1)	737045 (675178–798912	32 (29–36)
China	2025	4935 (22608–27263	1 (1–1)	744336 (666406–822267	32 (27–36)

China	2026	5234 (22515–27954	1 (1–1)	751628 (660421–842836	31 (26–37)
China	2027	5533 (22471–28596	1 (1–1)	758920 (656136–861704	31 (25–37)
China	2028	5832 (22462–29203	1 (1–1)	766212 (653030–879394	30 (24–37)
China	2029	6131 (22479–29784	1 (1–1)	773503 (650801–896205	30 (22–37)
China	2030	6430 (22516–30344	1 (1–1)	780795 (649260–912330	29 (21–37)
China	2031	6729 (22570–30889	1 (1–1)	788087 (648276–927898	29 (20–37)
China	2032	7028 (22637–31419	1 (1–1)	795379 (647755–943002	28 (19–37)
China	2033	7327 (22716–31939	1 (1–1)	302670 (647627–957714	28 (18–37)
China	2034	7626 (22805–32448	1 (1–1)	309962 (647838–972086	27 (18–37)
China	2035	7925 (22903–32948	1 (1–1)	317254 (648346–986161	27 (17–37)
China	2036	8225 (23009–33440	1 (1–1)	324546 (649116–999975	26 (16–36)
Colombia	2022	64 (59–70)	0 (0–0)	1503 (1347–1659)	3 (2–3)
Colombia	2023	65 (57–73)	0 (0–0)	1503 (1283–1724)	3 (2–3)
Colombia	2024	66 (56–76)	0 (0–0)	1503 (1233–1773)	2 (1–4)
Colombia	2025	67 (55–79)	0 (0–0)	1503 (1192–1815)	2 (1–4)
Colombia	2026	68 (55–81)	0 (0–0)	1503 (1155–1852)	2 (1–4)
Colombia	2027	69 (55–83)	0 (0–0)	1503 (1122–1885)	2 (1–4)
Colombia	2028	70 (54–85)	0 (0–0)	1503 (1091–1915)	2 (0–4)
Colombia	2029	70 (54–87)	0 (0–0)	1503 (1063–1944)	2 (0–4)
Colombia	2030	71 (54–89)	0 (0–0)	1503 (1036–1971)	2 (0–4)
Colombia	2031	72 (54–90)	0 (0–0)	1503 (1011–1996)	2 (-0–4)
Colombia	2032	73 (54–92)	0 (0–0)	1503 (986–2020)	2 (-0–4)
Colombia	2033	74 (54–94)	0 (0–0)	1503 (963–2043)	2 (-1–4)
Colombia	2034	75 (54–96)	0 (0–0)	1503 (941–2065)	2 (-1–4)
Colombia	2035	76 (54–97)	0 (0–0)	1503 (920–2086)	1 (-1–4)
Colombia	2036	76 (54–99)	0 (0–0)	1503 (900–2107)	1 (-1–4)
Comoros	2022	1 (1–1)	0 (0–0)	31 (30–33)	6 (5–6)
Comoros	2023	1 (1–1)	0 (0–0)	32 (30–34)	6 (5–6)
Comoros	2024	1 (1–1)	0 (0–0)	32 (30–35)	6 (5–6)
Comoros	2025	1 (1–1)	0 (0–0)	32 (30–35)	5 (5–6)
Comoros	2026	1 (1–1)	0 (0–0)	33 (30–36)	5 (4–6)
Comoros	2027	1 (1–1)	0 (0–0)	33 (30–37)	5 (4–7)
Comoros	2028	1 (1–1)	0 (0–0)	34 (30–37)	5 (4–7)

Comoros	2029	1 (1–1)	0 (0–0)	34 (30–38)	5 (4–7)
Comoros	2030	1 (1–1)	0 (0–0)	34 (30–38)	5 (3–7)
Comoros	2031	1 (1–1)	0 (0–0)	35 (30–39)	5 (3–7)
Comoros	2032	1 (1–1)	0 (0–0)	35 (30–40)	5 (3–6)
Comoros	2033	1 (1–2)	0 (0–0)	35 (31–40)	5 (3–6)
Comoros	2034	1 (1–2)	0 (0–0)	36 (31–41)	5 (3–6)
Comoros	2035	1 (1–2)	0 (0–0)	36 (31–41)	4 (2–6)
Comoros	2036	1 (1–2)	0 (0–0)	36 (31–42)	4 (2–6)
Congo	2022	11 (11–11)	0 (0–0)	335 (327–343)	10 (10–10)
Congo	2023	11 (11–12)	0 (0–0)	349 (334–364)	10 (9–11)
Congo	2024	12 (11–12)	0 (0–0)	363 (339–387)	10 (9–12)
Congo	2025	12 (11–13)	0 (0–0)	377 (343–411)	10 (8–12)
Congo	2026	13 (11–14)	0 (0–0)	391 (345–437)	10 (8–13)
Congo	2027	13 (11–15)	0 (0–0)	405 (347–463)	10 (7–14)
Congo	2028	14 (11–16)	0 (0–1)	419 (348–490)	11 (7–14)
Congo	2029	14 (12–16)	0 (0–1)	433 (348–519)	11 (6–15)
Congo	2030	14 (12–17)	0 (0–1)	447 (347–548)	11 (5–16)
Congo	2031	15 (12–18)	0 (0–1)	461 (345–578)	11 (4–17)
Congo	2032	15 (11–19)	0 (0–1)	475 (342–608)	11 (4–18)
Congo	2033	16 (11–20)	0 (0–1)	489 (339–640)	11 (3–19)
Congo	2034	16 (11–21)	0 (0–1)	503 (335–672)	11 (2–20)
Congo	2035	17 (11–22)	0 (0–1)	517 (330–705)	11 (1–21)
Congo	2036	17 (11–23)	0 (0–1)	532 (325–738)	11 (0–23)
Cook Islands	2022	0 (0–0)	1 (1–1)	8 (8–8)	32 (32–33)
Cook Islands	2023	0 (0–0)	1 (1–1)	8 (8–9)	33 (32–34)
Cook Islands	2024	0 (0–0)	1 (1–1)	8 (8–9)	33 (32–34)
Cook Islands	2025	0 (0–0)	1 (1–1)	8 (8–9)	33 (32–34)
Cook Islands	2026	0 (0–0)	1 (1–1)	9 (8–9)	33 (31–34)
Cook Islands	2027	0 (0–0)	1 (1–1)	9 (8–9)	32 (31–33)
Cook Islands	2028	0 (0–0)	1 (1–1)	9 (8–10)	32 (30–33)
Cook Islands	2029	0 (0–0)	1 (1–1)	9 (8–10)	32 (30–33)
Cook Islands	2030	0 (0–0)	1 (1–1)	9 (8–10)	32 (30–33)
Cook Islands	2031	0 (0–0)	1 (1–1)	9 (8–10)	32 (30–33)

Cook Islands	2032	0 (0–0)	1 (1–1)	9 (8–10)	32 (31–34)
Cook Islands	2033	0 (0–0)	1 (1–1)	9 (8–10)	33 (31–34)
Cook Islands	2034	0 (0–0)	1 (1–1)	9 (8–11)	33 (31–34)
Cook Islands	2035	0 (0–0)	1 (1–1)	10 (8–11)	33 (31–34)
Cook Islands	2036	0 (0–0)	1 (1–1)	10 (8–11)	32 (31–34)
Costa Rica	2022	27 (25–28)	0 (0–1)	619 (579–660)	11 (10–12)
Costa Rica	2023	27 (25–30)	0 (0–1)	632 (574–689)	11 (9–13)
Costa Rica	2024	28 (25–31)	0 (0–1)	644 (574–714)	11 (9–13)
Costa Rica	2025	28 (25–32)	0 (0–1)	657 (576–738)	11 (8–13)
Costa Rica	2026	29 (25–33)	0 (0–1)	669 (579–760)	11 (8–14)
Costa Rica	2027	29 (25–34)	0 (0–1)	682 (583–781)	11 (8–14)
Costa Rica	2028	30 (25–34)	0 (0–1)	694 (587–801)	11 (8–14)
Costa Rica	2029	31 (26–35)	0 (0–1)	707 (593–821)	11 (7–14)
Costa Rica	2030	31 (26–36)	0 (0–1)	719 (598–841)	11 (7–15)
Costa Rica	2031	32 (26–37)	0 (0–1)	732 (604–860)	11 (7–15)
Costa Rica	2032	32 (27–38)	0 (0–1)	745 (610–879)	11 (7–15)
Costa Rica	2033	33 (27–39)	0 (0–1)	757 (617–897)	11 (7–15)
Costa Rica	2034	33 (27–39)	0 (0–1)	770 (624–915)	11 (6–15)
Costa Rica	2035	34 (28–40)	0 (0–1)	782 (631–934)	11 (6–16)
Costa Rica	2036	34 (28–41)	0 (0–1)	795 (638–951)	11 (6–16)
Croatia	2022	23 (23–24)	0 (0–0)	772 (751–794)	5 (5–5)
Croatia	2023	24 (23–25)	0 (0–0)	784 (736–832)	5 (5–6)
Croatia	2024	24 (22–26)	0 (0–0)	795 (715–876)	5 (4–6)
Croatia	2025	25 (22–28)	0 (0–0)	807 (689–925)	5 (3–7)
Croatia	2026	25 (22–29)	0 (0–0)	818 (658–978)	5 (3–7)
Croatia	2027	26 (22–30)	0 (0–0)	830 (624–1035)	5 (2–8)
Croatia	2028	26 (22–30)	0 (0–0)	841 (586–1096)	5 (1–8)
Croatia	2029	26 (22–31)	0 (0–0)	853 (545–1160)	5 (-0–9)
Croatia	2030	26 (22–31)	0 (-0–0)	864 (500–1228)	4 (-1–10)
Croatia	2031	26 (22–31)	0 (-0–0)	875 (453–1298)	4 (-2–11)
Croatia	2032	26 (21–31)	0 (-0–0)	887 (402–1372)	4 (-3–12)
Croatia	2033	26 (21–31)	0 (-0–0)	898 (349–1448)	4 (-4–13)
Croatia	2034	26 (21–30)	0 (-0–0)	910 (293–1527)	4 (-5–13)

Croatia	2035	25 (21–30)	0 (-0–0)	921 (235–1608)	4 (-7–15)
Croatia	2036	25 (20–30)	0 (-0–1)	933 (174–1692)	4 (-8–16)
Cuba	2022	34 (30–38)	0 (0–0)	788 (690–887)	10 (8–11)
Cuba	2023	34 (27–42)	0 (0–0)	793 (614–972)	10 (7–12)
Cuba	2024	34 (24–44)	0 (0–1)	793 (545–1040)	10 (6–14)
Cuba	2025	34 (22–46)	0 (0–1)	788 (501–1076)	10 (5–15)
Cuba	2026	34 (20–47)	0 (0–1)	785 (476–1093)	10 (4–15)
Cuba	2027	34 (20–47)	0 (0–1)	783 (462–1104)	10 (3–16)
Cuba	2028	34 (19–48)	0 (0–1)	784 (451–1116)	10 (3–17)
Cuba	2029	34 (19–49)	0 (0–1)	786 (439–1133)	10 (2–17)
Cuba	2030	34 (18–50)	0 (0–1)	787 (422–1153)	10 (2–18)
Cuba	2031	34 (17–50)	0 (0–1)	788 (402–1173)	10 (1–18)
Cuba	2032	34 (16–51)	0 (0–1)	787 (383–1191)	10 (1–19)
Cuba	2033	34 (16–52)	0 (0–1)	786 (367–1206)	10 (0–19)
Cuba	2034	34 (15–53)	0 (0–1)	786 (352–1219)	10 (-0–19)
Cuba	2035	34 (14–53)	0 (-0–1)	786 (339–1232)	10 (-1–20)
Cuba	2036	34 (14–54)	0 (-0–1)	786 (327–1245)	10 (-1–20)
Cyprus	2022	31 (29–33)	0 (0–0)	817 (748–886)	4 (4–5)
Cyprus	2023	30 (27–34)	0 (0–0)	811 (717–904)	4 (3–5)
Cyprus	2024	30 (25–35)	0 (0–0)	804 (695–913)	4 (3–5)
Cyprus	2025	29 (23–35)	0 (0–0)	799 (678–919)	4 (3–5)
Cyprus	2026	28 (22–35)	0 (0–0)	794 (664–923)	4 (3–5)
Cyprus	2027	28 (21–34)	0 (0–0)	789 (653–925)	4 (2–5)
Cyprus	2028	27 (21–34)	0 (0–0)	785 (643–926)	4 (2–5)
Cyprus	2029	27 (20–34)	0 (0–0)	781 (635–927)	4 (2–5)
Cyprus	2030	27 (20–34)	0 (0–0)	777 (628–926)	4 (2–5)
Cyprus	2031	27 (20–34)	0 (0–0)	774 (623–926)	3 (2–5)
Cyprus	2032	27 (20–34)	0 (0–0)	772 (618–925)	3 (2–5)
Cyprus	2033	27 (20–34)	0 (0–0)	769 (613–925)	3 (1–5)
Cyprus	2034	27 (20–34)	0 (0–0)	767 (609–924)	3 (1–5)
Cyprus	2035	27 (20–34)	0 (0–0)	764 (606–923)	3 (1–5)
Cyprus	2036	27 (20–34)	0 (0–0)	763 (603–922)	3 (1–5)
Czechia	2022	8 (8–8)	0 (0–0)	186 (183–190)	9 (9–9)

Czechia	2023	8 (8–8)	0 (0–0)	189 (184–194)	9 (9–9)
Czechia	2024	8 (8–9)	0 (0–0)	193 (186–199)	9 (8–10)
Czechia	2025	8 (8–9)	0 (0–0)	196 (189–203)	9 (8–10)
Czechia	2026	9 (8–9)	0 (0–0)	199 (191–207)	9 (8–10)
Czechia	2027	9 (8–9)	0 (0–0)	202 (193–211)	9 (8–10)
Czechia	2028	9 (8–9)	0 (0–0)	205 (196–215)	9 (8–10)
Czechia	2029	9 (9–10)	0 (0–0)	209 (199–219)	9 (8–10)
Czechia	2030	9 (9–10)	0 (0–0)	212 (201–223)	9 (8–10)
Czechia	2031	9 (9–10)	0 (0–0)	215 (204–226)	9 (8–10)
Czechia	2032	9 (9–10)	0 (0–0)	218 (206–230)	9 (8–10)
Czechia	2033	10 (9–10)	0 (0–0)	221 (209–234)	9 (8–10)
Czechia	2034	10 (9–10)	0 (0–0)	225 (212–237)	9 (7–11)
Czechia	2035	10 (9–11)	0 (0–0)	228 (214–241)	9 (7–11)
Czechia	2036	10 (9–11)	0 (0–0)	231 (217–245)	9 (7–11)
C厶te d'Ivoire	2022	64 (57–71)	0 (0–0)	1484 (1313–1656)	7 (6–8)
C厶te d'Ivoire	2023	64 (54–74)	0 (0–0)	1456 (1213–1699)	7 (6–8)
C厶te d'Ivoire	2024	64 (52–76)	0 (0–0)	1427 (1130–1725)	7 (5–8)
C厶te d'Ivoire	2025	64 (50–78)	0 (0–0)	1399 (1055–1742)	6 (4–8)
C厶te d'Ivoire	2026	64 (49–80)	0 (0–0)	1370 (986–1754)	6 (4–8)
C厶te d'Ivoire	2027	64 (47–81)	0 (0–0)	1341 (921–1762)	6 (3–8)
C厶te d'Ivoire	2028	64 (46–82)	0 (0–0)	1313 (858–1767)	5 (3–8)
C厶te d'Ivoire	2029	64 (45–84)	0 (0–0)	1284 (799–1770)	5 (2–8)
C厶te d'Ivoire	2030	64 (44–85)	0 (0–0)	1256 (740–1771)	5 (2–8)
C厶te d'Ivoire	2031	64 (42–86)	0 (0–0)	1227 (684–1770)	4 (1–8)
C厶te d'Ivoire	2032	64 (41–87)	0 (0–0)	1198 (629–1768)	4 (1–8)
C厶te d'Ivoire	2033	64 (40–88)	0 (0–0)	1170 (575–1765)	4 (0–7)
C厶te d'Ivoire	2034	64 (39–89)	0 (0–0)	1141 (522–1760)	3 (-0–7)
C厶te d'Ivoire	2035	64 (39–90)	0 (0–0)	1113 (470–1755)	3 (-1–7)
C厶te d'Ivoire	2036	64 (38–91)	0 (-0–0)	1084 (419–1749)	3 (-1–7)
Democratic People's Republic of Kore	2022	258 (254–262)	1 (1–1)	8709 (8573–8846)	24 (24–25)
Democratic People's Republic of Kore	2023	263 (254–272)	1 (1–1)	8880 (8574–9185)	24 (23–26)
Democratic People's Republic of Kore	2024	269 (254–284)	1 (1–1)	9051 (8540–9562)	25 (23–27)
Democratic People's Republic of Kore	2025	274 (252–296)	1 (1–1)	9221 (8473–9969)	25 (22–28)

)emocratic People's Republic of Kore	2026	280 (250–309)	1 (1–1)	9392 (8379–10405)	25 (21–29)
)emocratic People's Republic of Kore	2027	285 (246–323)	1 (1–1)	9563 (8260–10866)	25 (19–31)
)emocratic People's Republic of Kore	2028	290 (243–338)	1 (1–1)	9734 (8118–11350)	25 (18–33)
)emocratic People's Republic of Kore	2029	296 (238–353)	1 (0–1)	9904 (7954–11855)	26 (17–35)
)emocratic People's Republic of Kore	2030	301 (233–369)	1 (0–1)	10075 (7769–12381)	26 (15–36)
)emocratic People's Republic of Kore	2031	306 (227–386)	1 (0–1)	10246 (7566–12926)	26 (13–39)
)emocratic People's Republic of Kore	2032	312 (221–403)	1 (0–1)	10417 (7344–13489)	26 (11–41)
)emocratic People's Republic of Kore	2033	317 (214–420)	1 (0–1)	10587 (7105–14069)	26 (10–43)
)emocratic People's Republic of Kore	2034	323 (207–438)	1 (0–1)	10758 (6849–14667)	26 (8–45)
)emocratic People's Republic of Kore	2035	328 (200–456)	1 (0–1)	10929 (6578–15280)	27 (5–48)
)emocratic People's Republic of Kore	2036	333 (191–475)	1 (0–1)	11100 (6290–15909)	27 (3–50)
Democratic Republic of the Congo	2022	48 (47–49)	0 (0–0)	1475 (1443–1507)	3 (3–3)
Democratic Republic of the Congo	2023	49 (47–51)	0 (0–0)	1525 (1466–1585)	3 (3–4)
Democratic Republic of the Congo	2024	51 (48–54)	0 (0–0)	1576 (1485–1667)	3 (3–4)
Democratic Republic of the Congo	2025	53 (49–57)	0 (0–0)	1626 (1500–1753)	3 (3–4)
Democratic Republic of the Congo	2026	54 (49–60)	0 (0–0)	1677 (1512–1841)	3 (3–4)
Democratic Republic of the Congo	2027	56 (49–63)	0 (0–0)	1727 (1521–1934)	3 (3–4)
Democratic Republic of the Congo	2028	58 (49–66)	0 (0–0)	1778 (1527–2029)	3 (3–4)
Democratic Republic of the Congo	2029	59 (49–69)	0 (0–0)	1829 (1531–2126)	3 (2–4)
Democratic Republic of the Congo	2030	61 (49–73)	0 (0–0)	1879 (1531–2227)	3 (2–5)
Democratic Republic of the Congo	2031	63 (49–76)	0 (0–0)	1930 (1530–2330)	3 (2–5)
Democratic Republic of the Congo	2032	64 (49–80)	0 (0–0)	1980 (1526–2435)	3 (2–5)
Democratic Republic of the Congo	2033	66 (49–83)	0 (0–0)	2031 (1519–2543)	3 (2–5)
Democratic Republic of the Congo	2034	68 (48–87)	0 (0–0)	2081 (1510–2652)	3 (2–5)
Democratic Republic of the Congo	2035	69 (48–91)	0 (0–0)	2132 (1500–2764)	3 (1–5)
Democratic Republic of the Congo	2036	71 (47–94)	0 (0–0)	2183 (1487–2878)	3 (1–6)
Denmark	2022	47 (44–50)	0 (0–0)	982 (919–1046)	9 (8–9)
Denmark	2023	47 (43–52)	0 (0–0)	996 (891–1100)	9 (7–10)
Denmark	2024	48 (43–53)	0 (0–0)	1010 (873–1147)	9 (7–10)
Denmark	2025	49 (43–55)	0 (0–0)	1025 (861–1190)	9 (7–11)
Denmark	2026	50 (43–57)	0 (0–0)	1040 (852–1228)	9 (7–11)
Denmark	2027	51 (43–58)	0 (0–0)	1055 (846–1264)	9 (7–11)
Denmark	2028	51 (44–59)	0 (0–1)	1070 (842–1298)	9 (7–11)

Denmark	2029	52 (44–61)	0 (0–1)	1085 (839–1331)	9 (7–11)
Denmark	2030	53 (44–62)	0 (0–1)	1100 (838–1363)	9 (7–11)
Denmark	2031	54 (44–63)	0 (0–1)	1115 (838–1393)	9 (7–11)
Denmark	2032	55 (45–65)	0 (0–1)	1131 (838–1423)	9 (7–11)
Denmark	2033	55 (45–66)	0 (0–1)	1146 (839–1452)	9 (7–11)
Denmark	2034	56 (46–67)	0 (0–1)	1161 (841–1480)	9 (7–11)
Denmark	2035	57 (46–68)	0 (0–1)	1176 (843–1508)	9 (7–11)
Denmark	2036	58 (46–69)	0 (0–1)	1191 (846–1536)	9 (7–11)
Djibouti	2022	2 (2–2)	0 (0–0)	73 (72–74)	9 (9–10)
Djibouti	2023	3 (2–3)	0 (0–0)	76 (74–77)	9 (9–10)
Djibouti	2024	3 (3–3)	0 (0–0)	78 (76–80)	9 (9–10)
Djibouti	2025	3 (3–3)	0 (0–0)	80 (77–83)	9 (8–10)
Djibouti	2026	3 (3–3)	0 (0–0)	82 (79–85)	9 (8–10)
Djibouti	2027	3 (3–3)	0 (0–0)	84 (80–88)	9 (8–10)
Djibouti	2028	3 (3–3)	0 (0–0)	86 (82–91)	9 (7–10)
Djibouti	2029	3 (3–3)	0 (0–0)	88 (83–94)	9 (7–11)
Djibouti	2030	3 (3–3)	0 (0–0)	91 (85–97)	9 (7–11)
Djibouti	2031	3 (3–4)	0 (0–0)	93 (86–100)	9 (6–11)
Djibouti	2032	3 (3–4)	0 (0–0)	95 (87–102)	9 (6–11)
Djibouti	2033	3 (3–4)	0 (0–0)	97 (89–105)	9 (6–12)
Djibouti	2034	4 (3–4)	0 (0–0)	99 (90–108)	9 (5–12)
Djibouti	2035	4 (3–4)	0 (0–1)	101 (91–111)	8 (5–12)
Djibouti	2036	4 (3–4)	0 (0–1)	103 (93–114)	8 (4–12)
Dominica	2022	0 (0–0)	0 (0–0)	6 (5–6)	6 (6–7)
Dominica	2023	0 (0–0)	0 (0–0)	6 (5–6)	6 (6–7)
Dominica	2024	0 (0–0)	0 (0–0)	6 (5–6)	6 (5–7)
Dominica	2025	0 (0–0)	0 (0–0)	6 (5–7)	6 (5–7)
Dominica	2026	0 (0–0)	0 (0–0)	6 (5–7)	6 (5–8)
Dominica	2027	0 (0–0)	0 (0–0)	6 (5–7)	6 (5–8)
Dominica	2028	0 (0–0)	0 (0–0)	6 (5–7)	6 (5–8)
Dominica	2029	0 (0–0)	0 (0–0)	6 (5–7)	6 (5–8)
Dominica	2030	0 (0–0)	0 (0–0)	6 (5–7)	6 (5–8)
Dominica	2031	0 (0–0)	0 (0–0)	6 (5–8)	6 (4–8)

Dominica	2032	0 (0–0)	0 (0–0)	6 (5–8)	6 (4–8)
Dominica	2033	0 (0–0)	0 (0–0)	7 (5–8)	6 (4–8)
Dominica	2034	0 (0–0)	0 (0–0)	7 (5–8)	6 (4–9)
Dominica	2035	0 (0–0)	0 (0–0)	7 (5–8)	6 (4–9)
Dominica	2036	0 (0–0)	0 (0–0)	7 (5–8)	6 (4–9)
Dominican Republic	2022	19 (19–20)	0 (0–0)	490 (476–504)	5 (5–5)
Dominican Republic	2023	20 (19–21)	0 (0–0)	497 (472–522)	5 (4–5)
Dominican Republic	2024	20 (19–21)	0 (0–0)	506 (471–541)	5 (4–5)
Dominican Republic	2025	20 (19–22)	0 (0–0)	516 (472–559)	5 (4–5)
Dominican Republic	2026	21 (19–23)	0 (0–0)	526 (475–576)	5 (4–5)
Dominican Republic	2027	21 (19–23)	0 (0–0)	536 (479–593)	5 (4–5)
Dominican Republic	2028	22 (19–24)	0 (0–0)	546 (483–609)	5 (4–5)
Dominican Republic	2029	22 (20–25)	0 (0–0)	556 (488–625)	5 (4–6)
Dominican Republic	2030	23 (20–25)	0 (0–0)	567 (493–640)	5 (4–6)
Dominican Republic	2031	23 (20–26)	0 (0–0)	577 (499–655)	5 (4–6)
Dominican Republic	2032	23 (21–26)	0 (0–0)	587 (504–670)	5 (3–6)
Dominican Republic	2033	24 (21–27)	0 (0–0)	598 (510–685)	5 (3–6)
Dominican Republic	2034	24 (21–27)	0 (0–0)	608 (517–699)	5 (3–6)
Dominican Republic	2035	25 (21–28)	0 (0–0)	618 (523–714)	5 (3–6)
Dominican Republic	2036	25 (22–28)	0 (0–0)	629 (530–728)	5 (3–6)
Ecuador	2022	28 (25–31)	0 (0–0)	650 (572–729)	4 (3–5)
Ecuador	2023	28 (23–32)	0 (0–0)	650 (539–762)	4 (3–5)
Ecuador	2024	28 (22–33)	0 (0–0)	650 (514–787)	4 (2–6)
Ecuador	2025	28 (22–34)	0 (0–0)	650 (493–808)	4 (2–6)
Ecuador	2026	28 (21–35)	0 (0–0)	650 (474–827)	4 (2–6)
Ecuador	2027	28 (20–36)	0 (0–0)	650 (457–844)	4 (1–6)
Ecuador	2028	28 (20–36)	0 (0–0)	650 (442–859)	4 (1–7)
Ecuador	2029	28 (19–37)	0 (0–0)	650 (427–873)	4 (1–7)
Ecuador	2030	28 (18–37)	0 (0–0)	650 (414–887)	4 (1–7)
Ecuador	2031	28 (18–38)	0 (0–0)	650 (401–900)	4 (1–7)
Ecuador	2032	28 (17–38)	0 (0–0)	650 (389–912)	4 (1–7)
Ecuador	2033	28 (17–39)	0 (0–0)	650 (377–924)	4 (0–7)
Ecuador	2034	28 (16–39)	0 (0–0)	650 (366–935)	4 (0–8)

Ecuador	2035	28 (16–40)	0 (0–0)	650 (355–945)	4 (0–8)
Ecuador	2036	28 (16–40)	0 (0–0)	650 (345–956)	4 (0–8)
Egypt	2022	1409 (1360–1458)	2 (2–2)	40250 (38821–41680)	55 (52–58)
Egypt	2023	1448 (1370–1526)	2 (2–2)	41364 (39050–43679)	55 (49–61)
Egypt	2024	1481 (1395–1567)	2 (2–2)	42301 (39699–44902)	55 (47–62)
Egypt	2025	1514 (1420–1608)	2 (2–3)	43237 (40376–46098)	55 (46–64)
Egypt	2026	1547 (1446–1648)	2 (2–3)	44173 (41075–47271)	55 (45–65)
Egypt	2027	1580 (1473–1688)	2 (2–3)	45109 (41790–48428)	55 (43–66)
Egypt	2028	1613 (1499–1727)	2 (2–3)	46045 (42520–49570)	55 (42–67)
Egypt	2029	1646 (1526–1766)	2 (2–3)	46981 (43261–50702)	55 (41–68)
Egypt	2030	1679 (1554–1805)	2 (2–3)	47918 (44012–51823)	55 (41–69)
Egypt	2031	1712 (1581–1843)	2 (2–3)	48854 (44771–52937)	55 (40–70)
Egypt	2032	1745 (1609–1881)	2 (2–3)	49790 (45537–54042)	55 (39–71)
Egypt	2033	1778 (1637–1920)	2 (2–3)	50726 (46310–55142)	55 (38–71)
Egypt	2034	1811 (1666–1957)	2 (1–3)	51662 (47089–56235)	55 (38–72)
Egypt	2035	1845 (1694–1995)	2 (1–3)	52598 (47873–57324)	55 (37–73)
Egypt	2036	1878 (1722–2033)	2 (1–3)	53535 (48662–58407)	55 (36–73)
El Salvador	2022	5 (5–6)	0 (0–0)	140 (132–149)	2 (2–2)
El Salvador	2023	5 (5–6)	0 (0–0)	142 (130–155)	2 (2–3)
El Salvador	2024	5 (5–6)	0 (0–0)	144 (129–159)	2 (2–3)
El Salvador	2025	6 (5–6)	0 (0–0)	146 (129–164)	2 (2–3)
El Salvador	2026	6 (5–6)	0 (0–0)	148 (129–168)	2 (2–3)
El Salvador	2027	6 (5–6)	0 (0–0)	150 (129–172)	2 (2–3)
El Salvador	2028	6 (5–7)	0 (0–0)	152 (129–175)	2 (2–3)
El Salvador	2029	6 (5–7)	0 (0–0)	154 (130–179)	2 (2–3)
El Salvador	2030	6 (5–7)	0 (0–0)	157 (130–183)	2 (2–3)
El Salvador	2031	6 (5–7)	0 (0–0)	159 (131–186)	2 (2–3)
El Salvador	2032	6 (5–7)	0 (0–0)	161 (132–189)	2 (2–3)
El Salvador	2033	6 (5–7)	0 (0–0)	163 (132–193)	2 (2–3)
El Salvador	2034	6 (5–7)	0 (0–0)	165 (133–196)	2 (2–3)
El Salvador	2035	6 (5–7)	0 (0–0)	167 (134–199)	2 (2–3)
El Salvador	2036	6 (5–8)	0 (0–0)	169 (135–202)	2 (2–3)
Equatorial Guinea	2022	2 (2–2)	0 (0–0)	53 (52–54)	8 (8–9)

Equatorial Guinea	2023	2 (2–2)	0 (0–0)	56 (54–58)	9 (8–9)
Equatorial Guinea	2024	2 (2–2)	0 (0–0)	59 (56–62)	9 (8–10)
Equatorial Guinea	2025	2 (2–2)	0 (0–0)	62 (58–66)	9 (8–10)
Equatorial Guinea	2026	2 (2–2)	0 (0–0)	65 (59–71)	9 (8–11)
Equatorial Guinea	2027	2 (2–2)	0 (0–0)	68 (60–75)	9 (8–11)
Equatorial Guinea	2028	2 (2–3)	0 (0–0)	71 (62–80)	10 (7–12)
Equatorial Guinea	2029	2 (2–3)	0 (0–0)	74 (63–85)	10 (7–13)
Equatorial Guinea	2030	2 (2–3)	0 (0–0)	77 (64–90)	10 (7–13)
Equatorial Guinea	2031	3 (2–3)	0 (0–1)	80 (64–95)	10 (7–14)
Equatorial Guinea	2032	3 (2–3)	0 (0–1)	83 (65–100)	10 (6–15)
Equatorial Guinea	2033	3 (2–3)	0 (0–1)	86 (66–106)	11 (6–15)
Equatorial Guinea	2034	3 (2–3)	0 (0–1)	89 (66–111)	11 (6–16)
Equatorial Guinea	2035	3 (2–4)	0 (0–1)	92 (67–116)	11 (5–17)
Equatorial Guinea	2036	3 (2–4)	0 (0–1)	95 (67–122)	11 (5–18)
Eritrea	2022	2 (2–3)	0 (0–0)	88 (85–90)	2 (2–2)
Eritrea	2023	2 (2–3)	0 (0–0)	89 (84–95)	2 (2–2)
Eritrea	2024	3 (2–3)	0 (0–0)	91 (83–99)	2 (2–3)
Eritrea	2025	3 (2–3)	0 (0–0)	92 (81–103)	2 (2–3)
Eritrea	2026	3 (2–3)	0 (0–0)	93 (79–107)	2 (1–3)
Eritrea	2027	3 (2–3)	0 (0–0)	93 (77–110)	2 (1–3)
Eritrea	2028	3 (2–3)	0 (0–0)	94 (75–113)	2 (1–3)
Eritrea	2029	3 (2–3)	0 (0–0)	94 (73–116)	2 (1–3)
Eritrea	2030	3 (2–3)	0 (0–0)	95 (71–119)	2 (1–4)
Eritrea	2031	3 (2–3)	0 (0–0)	95 (69–121)	2 (1–4)
Eritrea	2032	3 (2–3)	0 (0–0)	95 (67–124)	2 (1–4)
Eritrea	2033	3 (2–4)	0 (0–0)	95 (65–126)	2 (0–4)
Eritrea	2034	3 (2–4)	0 (0–0)	95 (63–128)	2 (0–4)
Eritrea	2035	3 (2–4)	0 (0–0)	96 (61–130)	2 (0–4)
Eritrea	2036	3 (2–4)	0 (0–0)	96 (59–132)	2 (0–4)
Estonia	2022	10 (9–11)	0 (0–0)	254 (228–280)	12 (10–13)
Estonia	2023	10 (9–12)	0 (0–0)	251 (220–282)	12 (10–13)
Estonia	2024	10 (9–12)	0 (0–0)	249 (217–281)	12 (10–13)
Estonia	2025	10 (8–12)	0 (0–0)	248 (215–281)	12 (10–13)

Estonia	2026	10 (8–13)	0 (0–0)	247 (215–280)	12 (10–13)
Estonia	2027	10 (8–13)	0 (0–0)	247 (214–280)	12 (10–13)
Estonia	2028	10 (8–13)	0 (0–0)	247 (214–280)	12 (10–13)
Estonia	2029	10 (7–13)	0 (0–0)	247 (214–279)	12 (10–13)
Estonia	2030	10 (7–13)	0 (0–0)	246 (214–279)	12 (10–13)
Estonia	2031	10 (7–13)	0 (0–0)	246 (214–279)	12 (10–13)
Estonia	2032	10 (7–14)	0 (0–0)	246 (214–279)	12 (10–13)
Estonia	2033	10 (7–14)	0 (0–0)	246 (213–279)	12 (10–13)
Estonia	2034	10 (7–14)	0 (0–0)	246 (213–279)	12 (10–13)
Estonia	2035	10 (7–14)	0 (0–0)	246 (213–279)	12 (10–13)
Estonia	2036	10 (6–14)	0 (0–0)	246 (213–279)	12 (10–13)
Eswatini	2022	5 (5–6)	1 (1–1)	172 (164–179)	26 (25–28)
Eswatini	2023	5 (5–6)	1 (1–1)	172 (151–192)	26 (22–30)
Eswatini	2024	5 (4–7)	1 (1–1)	172 (132–212)	27 (19–34)
Eswatini	2025	5 (4–7)	1 (1–1)	172 (108–236)	27 (14–39)
Eswatini	2026	5 (3–8)	1 (0–2)	172 (80–265)	27 (9–45)
Eswatini	2027	5 (2–9)	1 (0–2)	173 (47–299)	28 (3–52)
Eswatini	2028	5 (1–10)	1 (0–2)	173 (11–336)	28 (-3–60)
Eswatini	2029	5 (-1–12)	1 (-0–3)	174 (-29–377)	29 (-11–68)
Eswatini	2030	5 (-2–13)	1 (-0–3)	174 (-73–421)	29 (-19–77)
Eswatini	2031	5 (-3–14)	1 (-1–3)	175 (-119–468)	29 (-27–86)
Eswatini	2032	5 (-5–16)	1 (-1–4)	175 (-168–518)	30 (-36–96)
Eswatini	2033	5 (-6–17)	1 (-1–4)	175 (-219–570)	30 (-46–106)
Eswatini	2034	5 (-8–19)	1 (-2–4)	176 (-273–625)	31 (-56–117)
Eswatini	2035	5 (-10–21)	1 (-2–5)	176 (-330–682)	31 (-66–129)
Eswatini	2036	5 (-12–22)	1 (-2–5)	177 (-389–742)	32 (-77–140)
Ethiopia	2022	18 (18–18)	0 (0–0)	386 (293–479)	1 (1–1)
Ethiopia	2023	19 (18–19)	0 (0–0)	386 (293–479)	1 (1–1)
Ethiopia	2024	19 (18–20)	0 (0–0)	386 (293–479)	1 (1–1)
Ethiopia	2025	20 (19–21)	0 (0–0)	386 (293–479)	1 (1–1)
Ethiopia	2026	20 (19–22)	0 (0–0)	386 (293–479)	1 (1–1)
Ethiopia	2027	21 (19–23)	0 (0–0)	386 (293–479)	1 (1–1)
Ethiopia	2028	22 (19–24)	0 (0–0)	386 (293–479)	1 (1–1)

Ethiopia	2029	22 (19–25)	0 (0–0)	386 (293–479)	1 (1–1)
Ethiopia	2030	23 (19–26)	0 (0–0)	386 (293–479)	1 (1–1)
Ethiopia	2031	23 (20–27)	0 (0–0)	386 (293–479)	1 (1–2)
Ethiopia	2032	24 (20–29)	0 (0–0)	386 (293–479)	1 (1–2)
Ethiopia	2033	25 (20–30)	0 (0–0)	386 (293–479)	1 (1–2)
Ethiopia	2034	25 (20–31)	0 (0–0)	386 (293–479)	1 (1–2)
Ethiopia	2035	26 (20–32)	0 (0–0)	386 (293–479)	1 (1–2)
Ethiopia	2036	26 (19–33)	0 (0–0)	386 (293–479)	1 (1–2)
Fiji	2022	4 (4–4)	0 (0–0)	121 (116–126)	13 (13–14)
Fiji	2023	4 (4–4)	0 (0–1)	123 (114–132)	14 (12–15)
Fiji	2024	4 (4–4)	0 (0–1)	125 (114–136)	14 (12–15)
Fiji	2025	4 (4–5)	0 (0–1)	126 (113–139)	14 (12–15)
Fiji	2026	4 (4–5)	0 (0–1)	128 (113–143)	14 (12–16)
Fiji	2027	4 (4–5)	0 (0–1)	130 (113–146)	14 (12–16)
Fiji	2028	4 (4–5)	0 (0–1)	131 (114–149)	14 (12–16)
Fiji	2029	4 (4–5)	0 (0–1)	133 (114–152)	14 (12–16)
Fiji	2030	4 (4–5)	0 (0–1)	135 (114–155)	14 (12–16)
Fiji	2031	5 (4–5)	0 (0–1)	136 (115–158)	14 (12–16)
Fiji	2032	5 (4–5)	0 (0–1)	138 (115–161)	14 (12–16)
Fiji	2033	5 (4–5)	0 (0–1)	140 (116–163)	14 (12–16)
Fiji	2034	5 (4–5)	0 (0–1)	141 (117–166)	14 (12–16)
Fiji	2035	5 (4–6)	0 (0–1)	143 (117–169)	14 (12–16)
Fiji	2036	5 (4–6)	0 (0–1)	145 (118–171)	14 (12–16)
Finland	2022	45 (43–46)	0 (0–0)	987 (949–1026)	9 (8–9)
Finland	2023	46 (43–48)	0 (0–0)	1002 (933–1070)	9 (8–10)
Finland	2024	46 (43–50)	0 (0–0)	1016 (927–1105)	9 (8–10)
Finland	2025	47 (43–52)	0 (0–0)	1030 (924–1136)	9 (8–10)
Finland	2026	48 (43–53)	0 (0–0)	1045 (924–1165)	9 (8–10)
Finland	2027	49 (43–54)	0 (0–0)	1059 (926–1192)	9 (8–10)
Finland	2028	49 (44–55)	0 (0–0)	1073 (929–1218)	9 (8–10)
Finland	2029	50 (44–57)	0 (0–0)	1088 (932–1243)	9 (8–10)
Finland	2030	51 (44–58)	0 (0–0)	1102 (936–1268)	9 (7–10)
Finland	2031	52 (45–59)	0 (0–0)	1117 (941–1292)	9 (7–10)

Finland	2032	53 (45–60)	0 (0–0)	1131 (947–1315)	9 (7–10)
Finland	2033	53 (45–61)	0 (0–0)	1145 (952–1338)	9 (7–10)
Finland	2034	54 (46–62)	0 (0–0)	1160 (959–1361)	9 (7–11)
Finland	2035	55 (46–64)	0 (0–0)	1174 (965–1383)	9 (7–11)
Finland	2036	56 (47–65)	0 (0–0)	1188 (972–1405)	9 (7–11)
France	2022	894 (861–926)	1 (1–1)	20737 (19951–21524)	18 (17–19)
France	2023	898 (841–954)	1 (1–1)	20735 (19366–22105)	18 (17–20)
France	2024	901 (819–984)	1 (1–1)	20734 (18735–22732)	19 (17–20)
France	2025	905 (795–1016)	1 (1–1)	20732 (18048–23415)	19 (17–21)
France	2026	909 (768–1050)	1 (1–1)	20730 (17307–24153)	20 (18–22)
France	2027	913 (739–1087)	1 (1–1)	20728 (16513–24943)	20 (18–23)
France	2028	917 (708–1125)	1 (1–1)	20726 (15668–25784)	21 (18–23)
France	2029	920 (675–1166)	1 (1–1)	20724 (14777–26672)	21 (19–23)
France	2030	924 (640–1208)	1 (1–1)	20722 (13839–27605)	21 (19–24)
France	2031	928 (604–1252)	1 (1–1)	20720 (12859–28582)	21 (19–24)
France	2032	932 (565–1298)	1 (1–1)	20718 (11836–29600)	21 (19–24)
France	2033	935 (525–1346)	1 (1–1)	20716 (10774–30659)	21 (19–23)
France	2034	939 (484–1395)	1 (1–1)	20715 (9673–31756)	21 (18–23)
France	2035	943 (441–1445)	1 (1–1)	20713 (8534–32891)	20 (18–23)
France	2036	947 (396–1498)	1 (1–1)	20711 (7359–34062)	20 (17–23)
Gabon	2022	6 (6–6)	1 (1–1)	185 (182–188)	15 (14–15)
Gabon	2023	6 (6–6)	1 (1–1)	189 (184–195)	15 (14–15)
Gabon	2024	6 (6–7)	1 (1–1)	193 (185–201)	15 (14–16)
Gabon	2025	7 (6–7)	1 (1–1)	197 (185–208)	15 (14–16)
Gabon	2026	7 (6–7)	1 (0–1)	201 (186–216)	15 (14–16)
Gabon	2027	7 (6–8)	1 (0–1)	205 (186–223)	15 (13–16)
Gabon	2028	7 (6–8)	1 (0–1)	209 (186–231)	15 (13–16)
Gabon	2029	7 (6–8)	1 (0–1)	212 (185–240)	15 (13–17)
Gabon	2030	7 (6–9)	1 (0–1)	216 (185–248)	15 (13–17)
Gabon	2031	7 (6–9)	1 (0–1)	220 (184–257)	15 (13–17)
Gabon	2032	7 (5–10)	1 (0–1)	224 (183–266)	15 (13–17)
Gabon	2033	8 (5–10)	1 (0–1)	228 (181–275)	15 (13–17)
Gabon	2034	8 (5–10)	1 (0–1)	232 (180–284)	15 (12–17)

Gabon	2035	8 (5–11)	1 (0–1)	236 (178–293)	15 (12–17)
Gabon	2036	8 (5–11)	1 (0–1)	240 (176–303)	15 (12–17)
Gambia	2022	23 (22–24)	2 (2–2)	769 (728–811)	63 (58–68)
Gambia	2023	23 (22–25)	2 (2–2)	782 (723–841)	62 (55–69)
Gambia	2024	24 (22–26)	2 (2–2)	794 (723–866)	61 (53–70)
Gambia	2025	24 (22–26)	2 (2–2)	807 (724–890)	61 (51–71)
Gambia	2026	24 (22–27)	2 (2–2)	820 (727–912)	60 (49–71)
Gambia	2027	25 (22–28)	2 (2–2)	832 (731–934)	59 (47–71)
Gambia	2028	25 (22–28)	2 (2–2)	845 (735–955)	59 (45–72)
Gambia	2029	26 (22–29)	2 (1–2)	857 (740–975)	58 (44–72)
Gambia	2030	26 (23–29)	2 (1–2)	870 (746–994)	57 (42–72)
Gambia	2031	26 (23–30)	2 (1–2)	883 (751–1014)	56 (41–72)
Gambia	2032	27 (23–30)	2 (1–2)	895 (758–1033)	56 (39–72)
Gambia	2033	27 (23–31)	2 (1–2)	908 (764–1052)	55 (38–72)
Gambia	2034	27 (23–32)	2 (1–2)	920 (771–1070)	54 (36–72)
Gambia	2035	28 (24–32)	2 (1–2)	933 (778–1088)	54 (35–72)
Gambia	2036	28 (24–33)	2 (1–2)	946 (785–1106)	53 (34–72)
Georgia	2022	32 (26–38)	1 (0–1)	911 (738–1083)	16 (13–19)
Georgia	2023	31 (22–40)	1 (0–1)	911 (667–1154)	16 (11–20)
Georgia	2024	30 (19–42)	1 (0–1)	911 (612–1209)	15 (10–21)
Georgia	2025	30 (17–43)	1 (0–1)	911 (566–1255)	15 (9–21)
Georgia	2026	30 (15–44)	0 (0–1)	911 (525–1296)	15 (8–21)
Georgia	2027	29 (14–44)	0 (0–1)	911 (488–1333)	15 (8–21)
Georgia	2028	29 (14–44)	0 (0–1)	911 (454–1367)	14 (7–21)
Georgia	2029	29 (13–44)	0 (0–1)	911 (423–1398)	14 (7–21)
Georgia	2030	28 (13–44)	0 (0–1)	911 (393–1428)	14 (7–21)
Georgia	2031	28 (13–44)	0 (0–1)	911 (365–1456)	14 (7–21)
Georgia	2032	28 (12–44)	0 (0–1)	911 (339–1482)	14 (7–21)
Georgia	2033	28 (12–44)	0 (0–1)	911 (313–1508)	14 (7–21)
Georgia	2034	28 (12–44)	0 (0–1)	911 (289–1532)	14 (6–21)
Georgia	2035	28 (12–44)	0 (0–1)	911 (265–1556)	14 (6–21)
Georgia	2036	28 (12–44)	0 (0–1)	911 (243–1578)	14 (6–21)
Germany	2022	852 (825–879)	0 (0–0)	19045 (18428–19661)	11 (11–12)

Germany	2023	863 (815–910)	0 (0–0)	19312 (18166–20459)	11 (10–12)
Germany	2024	874 (809–940)	0 (0–0)	19580 (18081–21079)	11 (10–12)
Germany	2025	886 (806–967)	0 (0–1)	19848 (18064–21631)	11 (10–13)
Germany	2026	899 (805–993)	0 (0–1)	20115 (18087–22144)	11 (10–13)
Germany	2027	912 (806–1017)	0 (0–1)	20383 (18136–22630)	11 (10–13)
Germany	2028	924 (807–1041)	0 (0–1)	20651 (18205–23096)	11 (9–13)
Germany	2029	937 (810–1063)	0 (0–1)	20918 (18289–23548)	11 (9–13)
Germany	2030	949 (814–1085)	0 (0–1)	21186 (18384–23987)	11 (9–13)
Germany	2031	962 (818–1107)	0 (0–1)	21454 (18490–24417)	11 (9–13)
Germany	2032	975 (822–1127)	0 (0–1)	21721 (18604–24838)	11 (9–14)
Germany	2033	987 (827–1148)	0 (0–1)	21989 (18726–25252)	11 (9–14)
Germany	2034	1000 (832–1168)	0 (0–1)	22257 (18853–25660)	11 (9–14)
Germany	2035	1013 (838–1188)	0 (0–1)	22524 (18986–26062)	11 (9–14)
Germany	2036	1025 (844–1207)	0 (0–1)	22792 (19125–26459)	11 (8–14)
Ghana	2022	53 (52–54)	0 (0–0)	1490 (1453–1528)	8 (8–8)
Ghana	2023	54 (51–56)	0 (0–0)	1511 (1437–1585)	8 (7–9)
Ghana	2024	54 (51–58)	0 (0–0)	1532 (1421–1642)	8 (7–9)
Ghana	2025	55 (50–60)	0 (0–0)	1552 (1407–1697)	8 (6–9)
Ghana	2026	56 (50–62)	0 (0–0)	1572 (1395–1750)	8 (6–10)
Ghana	2027	57 (50–64)	0 (0–0)	1592 (1384–1800)	8 (6–10)
Ghana	2028	58 (50–65)	0 (0–0)	1613 (1376–1849)	8 (5–10)
Ghana	2029	58 (50–67)	0 (0–0)	1633 (1370–1896)	8 (5–10)
Ghana	2030	59 (50–68)	0 (0–0)	1653 (1365–1941)	8 (5–11)
Ghana	2031	60 (50–70)	0 (0–0)	1673 (1361–1984)	8 (5–11)
Ghana	2032	61 (50–71)	0 (0–0)	1693 (1359–2027)	8 (4–11)
Ghana	2033	61 (50–73)	0 (0–0)	1713 (1359–2068)	8 (4–12)
Ghana	2034	62 (50–74)	0 (0–0)	1733 (1359–2108)	8 (4–12)
Ghana	2035	63 (50–76)	0 (0–0)	1753 (1360–2147)	8 (3–12)
Ghana	2036	64 (50–77)	0 (0–1)	1774 (1362–2185)	8 (3–12)
Greece	2022	126 (118–135)	1 (1–1)	2914 (2699–3129)	15 (14–17)
Greece	2023	129 (113–145)	1 (1–1)	2973 (2583–3363)	16 (13–18)
Greece	2024	132 (111–153)	1 (0–1)	3032 (2524–3540)	16 (13–19)
Greece	2025	134 (110–159)	1 (0–1)	3092 (2488–3695)	16 (13–20)

Greece	2026	137 (109–165)	1 (0–1)	3151 (2466–3836)	16 (13–20)
Greece	2027	140 (108–171)	1 (0–1)	3210 (2452–3969)	17 (12–21)
Greece	2028	143 (108–177)	1 (0–1)	3269 (2444–4095)	17 (12–22)
Greece	2029	145 (109–182)	1 (0–1)	3329 (2442–4216)	17 (12–22)
Greece	2030	148 (109–187)	1 (0–1)	3388 (2443–4333)	17 (12–23)
Greece	2031	151 (109–192)	1 (0–1)	3447 (2448–4446)	18 (12–23)
Greece	2032	153 (110–197)	1 (0–1)	3506 (2456–4557)	18 (12–24)
Greece	2033	156 (110–201)	1 (0–1)	3566 (2466–4666)	18 (12–24)
Greece	2034	159 (111–206)	1 (0–1)	3625 (2478–4772)	19 (12–25)
Greece	2035	161 (112–211)	1 (0–1)	3684 (2492–4877)	19 (12–25)
Greece	2036	164 (113–215)	1 (0–1)	3744 (2508–4980)	19 (12–26)
Greenland	2022	1 (1–1)	1 (1–1)	18 (18–19)	23 (22–24)
Greenland	2023	1 (1–1)	1 (1–1)	19 (18–19)	23 (21–24)
Greenland	2024	1 (1–1)	1 (1–1)	19 (18–20)	23 (21–25)
Greenland	2025	1 (1–1)	1 (1–1)	19 (18–20)	23 (21–25)
Greenland	2026	1 (1–1)	1 (1–1)	19 (18–20)	23 (20–25)
Greenland	2027	1 (1–1)	1 (1–1)	20 (18–21)	23 (20–25)
Greenland	2028	1 (1–1)	1 (1–1)	20 (18–21)	23 (20–25)
Greenland	2029	1 (1–1)	1 (1–1)	20 (19–21)	23 (20–26)
Greenland	2030	1 (1–1)	1 (1–1)	20 (19–22)	23 (20–26)
Greenland	2031	1 (1–1)	1 (1–1)	20 (19–22)	23 (19–26)
Greenland	2032	1 (1–1)	1 (1–1)	21 (19–22)	23 (19–26)
Greenland	2033	1 (1–1)	1 (1–1)	21 (19–23)	23 (19–26)
Greenland	2034	1 (1–1)	1 (1–1)	21 (19–23)	23 (19–26)
Greenland	2035	1 (1–1)	1 (1–1)	21 (20–23)	23 (19–27)
Greenland	2036	1 (1–1)	1 (1–1)	22 (20–24)	23 (19–27)
Grenada	2022	0 (0–0)	0 (0–0)	8 (8–9)	7 (6–7)
Grenada	2023	0 (0–0)	0 (0–0)	8 (7–9)	7 (6–8)
Grenada	2024	0 (0–0)	0 (0–0)	9 (7–10)	7 (6–8)
Grenada	2025	0 (0–0)	0 (0–0)	9 (7–10)	7 (5–8)
Grenada	2026	0 (0–0)	0 (0–0)	9 (7–10)	7 (5–8)
Grenada	2027	0 (0–1)	0 (0–0)	9 (7–11)	7 (5–8)
Grenada	2028	0 (0–1)	0 (0–0)	9 (7–11)	7 (5–8)

Grenada	2029	0 (0–1)	0 (0–0)	9 (7–11)	7 (5–8)
Grenada	2030	0 (0–1)	0 (0–0)	10 (7–12)	7 (5–9)
Grenada	2031	0 (0–1)	0 (0–0)	10 (8–12)	7 (5–9)
Grenada	2032	0 (0–1)	0 (0–0)	10 (8–12)	7 (4–9)
Grenada	2033	0 (0–1)	0 (0–0)	10 (8–13)	7 (4–9)
Grenada	2034	1 (0–1)	0 (0–0)	10 (8–13)	7 (4–9)
Grenada	2035	1 (0–1)	0 (0–0)	10 (8–13)	7 (4–9)
Grenada	2036	1 (0–1)	0 (0–0)	11 (8–13)	7 (4–9)
Guam	2022	1 (1–1)	1 (1–1)	40 (39–41)	19 (18–20)
Guam	2023	1 (1–1)	1 (1–1)	41 (39–43)	19 (18–20)
Guam	2024	1 (1–1)	1 (1–1)	42 (39–44)	20 (18–21)
Guam	2025	1 (1–2)	1 (1–1)	43 (39–46)	20 (18–22)
Guam	2026	1 (1–2)	1 (1–1)	44 (39–48)	20 (18–23)
Guam	2027	1 (1–2)	1 (1–1)	45 (40–50)	20 (18–23)
Guam	2028	1 (1–2)	1 (1–1)	45 (40–51)	21 (17–24)
Guam	2029	2 (1–2)	1 (1–1)	46 (40–53)	21 (17–24)
Guam	2030	2 (1–2)	1 (1–1)	47 (41–54)	21 (17–25)
Guam	2031	2 (1–2)	1 (1–1)	48 (41–55)	22 (17–26)
Guam	2032	2 (1–2)	1 (1–1)	49 (42–57)	22 (18–26)
Guam	2033	2 (1–2)	1 (1–1)	50 (42–58)	22 (18–27)
Guam	2034	2 (1–2)	1 (1–1)	51 (43–60)	22 (18–27)
Guam	2035	2 (1–2)	1 (1–1)	52 (43–61)	23 (18–28)
Guam	2036	2 (1–2)	1 (1–1)	53 (44–62)	23 (18–28)
Guatemala	2022	31 (26–35)	0 (0–0)	756 (640–871)	7 (4–9)
Guatemala	2023	31 (22–39)	0 (0–0)	756 (538–974)	7 (2–11)
Guatemala	2024	31 (20–42)	0 (0–1)	756 (470–1041)	7 (1–12)
Guatemala	2025	31 (17–44)	0 (0–1)	756 (416–1096)	7 (-0–13)
Guatemala	2026	31 (16–46)	0 (-0–1)	756 (369–1143)	7 (-1–14)
Guatemala	2027	31 (14–47)	0 (-0–1)	756 (327–1184)	7 (-2–15)
Guatemala	2028	31 (12–49)	0 (-0–1)	756 (289–1223)	7 (-3–16)
Guatemala	2029	31 (11–50)	0 (-0–1)	756 (254–1258)	7 (-3–17)
Guatemala	2030	31 (10–52)	0 (-0–1)	756 (221–1291)	7 (-4–17)
Guatemala	2031	31 (9–53)	0 (-0–1)	756 (190–1322)	7 (-4–18)

Guatemala	2032	31 (7–54)	0 (-0–1)	756 (160–1351)	7 (-5–18)
Guatemala	2033	31 (6–55)	0 (-0–1)	756 (132–1379)	7 (-6–19)
Guatemala	2034	31 (5–56)	0 (-0–1)	756 (106–1406)	7 (-6–19)
Guatemala	2035	31 (4–57)	0 (-0–1)	756 (80–1431)	7 (-7–20)
Guatemala	2036	31 (3–58)	0 (-0–1)	756 (55–1456)	7 (-7–20)
Guinea	2022	54 (52–56)	1 (1–1)	1675 (1606–1744)	25 (24–27)
Guinea	2023	54 (49–59)	1 (1–1)	1679 (1524–1833)	25 (22–28)
Guinea	2024	54 (46–62)	1 (1–1)	1682 (1424–1941)	25 (20–29)
Guinea	2025	54 (42–66)	1 (1–1)	1686 (1307–2065)	24 (17–31)
Guinea	2026	54 (38–70)	1 (1–1)	1690 (1177–2203)	24 (15–33)
Guinea	2027	54 (33–75)	1 (0–1)	1694 (1034–2354)	23 (11–35)
Guinea	2028	54 (28–80)	1 (0–1)	1698 (879–2516)	23 (8–38)
Guinea	2029	54 (22–85)	1 (0–1)	1701 (713–2690)	22 (5–40)
Guinea	2030	54 (17–91)	1 (0–2)	1705 (537–2873)	22 (1–43)
Guinea	2031	54 (11–97)	1 (-0–2)	1709 (351–3067)	22 (-3–46)
Guinea	2032	54 (4–103)	1 (-0–2)	1713 (156–3269)	21 (-7–49)
Guinea	2033	54 (-2–110)	1 (-0–2)	1716 (-48–3481)	21 (-11–52)
Guinea	2034	54 (-9–116)	1 (-0–2)	1720 (-260–3701)	20 (-15–56)
Guinea	2035	54 (-16–123)	1 (-1–2)	1724 (-481–3929)	20 (-20–60)
Guinea	2036	54 (-24–131)	1 (-1–2)	1728 (-709–4165)	19 (-24–63)
Guinea-Bissau	2022	6 (6–6)	1 (1–1)	201 (194–209)	20 (19–21)
Guinea-Bissau	2023	6 (5–6)	1 (1–1)	201 (187–215)	20 (18–22)
Guinea-Bissau	2024	6 (5–6)	1 (1–1)	201 (183–220)	19 (17–22)
Guinea-Bissau	2025	6 (5–6)	1 (1–1)	201 (180–223)	19 (16–21)
Guinea-Bissau	2026	6 (5–7)	1 (1–1)	201 (177–226)	18 (15–21)
Guinea-Bissau	2027	6 (5–7)	1 (0–1)	201 (174–229)	18 (14–21)
Guinea-Bissau	2028	6 (5–7)	1 (0–1)	201 (172–231)	17 (13–21)
Guinea-Bissau	2029	6 (5–7)	1 (0–1)	201 (169–234)	16 (12–21)
Guinea-Bissau	2030	6 (5–7)	1 (0–1)	201 (167–236)	16 (11–20)
Guinea-Bissau	2031	6 (5–7)	1 (0–1)	201 (165–238)	15 (11–20)
Guinea-Bissau	2032	6 (5–7)	0 (0–1)	201 (163–239)	15 (10–20)
Guinea-Bissau	2033	6 (5–7)	0 (0–1)	201 (162–241)	14 (9–19)
Guinea-Bissau	2034	6 (5–7)	0 (0–1)	201 (160–243)	14 (8–19)

Guinea-Bissau	2035	6 (5–7)	0 (0–1)	201 (158–245)	13 (8–19)
Guinea-Bissau	2036	6 (5–7)	0 (0–1)	201 (157–246)	13 (7–18)
Guyana	2022	1 (1–1)	0 (0–0)	31 (28–34)	4 (4–5)
Guyana	2023	1 (1–1)	0 (0–0)	31 (27–35)	4 (4–5)
Guyana	2024	1 (1–1)	0 (0–0)	31 (26–36)	4 (3–5)
Guyana	2025	1 (1–1)	0 (0–0)	31 (25–36)	4 (3–5)
Guyana	2026	1 (1–1)	0 (0–0)	31 (25–37)	4 (3–6)
Guyana	2027	1 (1–1)	0 (0–0)	31 (24–38)	4 (3–6)
Guyana	2028	1 (1–1)	0 (0–0)	31 (24–38)	4 (3–6)
Guyana	2029	1 (1–1)	0 (0–0)	31 (23–39)	4 (3–6)
Guyana	2030	1 (1–1)	0 (0–0)	31 (23–39)	4 (3–6)
Guyana	2031	1 (1–1)	0 (0–0)	31 (22–40)	4 (3–6)
Guyana	2032	1 (1–1)	0 (0–0)	31 (22–40)	4 (3–6)
Guyana	2033	1 (1–1)	0 (0–0)	31 (21–40)	4 (2–6)
Guyana	2034	1 (1–1)	0 (0–0)	31 (21–41)	4 (2–6)
Guyana	2035	1 (1–1)	0 (0–0)	31 (21–41)	4 (2–6)
Guyana	2036	1 (1–1)	0 (0–0)	31 (20–41)	4 (2–6)
Haiti	2022	5 (5–5)	0 (0–0)	130 (128–132)	2 (2–2)
Haiti	2023	5 (5–5)	0 (0–0)	134 (130–137)	2 (2–2)
Haiti	2024	5 (5–5)	0 (0–0)	138 (132–143)	2 (2–2)
Haiti	2025	5 (5–5)	0 (0–0)	141 (134–149)	2 (2–2)
Haiti	2026	5 (5–6)	0 (0–0)	145 (135–156)	2 (2–2)
Haiti	2027	5 (5–6)	0 (0–0)	149 (136–162)	2 (1–2)
Haiti	2028	6 (5–6)	0 (0–0)	153 (137–169)	2 (1–2)
Haiti	2029	6 (5–6)	0 (0–0)	157 (138–176)	2 (1–2)
Haiti	2030	6 (5–7)	0 (0–0)	161 (139–183)	2 (1–2)
Haiti	2031	6 (5–7)	0 (0–0)	165 (139–190)	2 (1–2)
Haiti	2032	6 (5–7)	0 (0–0)	169 (140–198)	2 (1–2)
Haiti	2033	6 (5–7)	0 (0–0)	173 (140–206)	2 (1–2)
Haiti	2034	6 (5–8)	0 (0–0)	177 (140–213)	2 (1–2)
Haiti	2035	7 (5–8)	0 (0–0)	181 (140–221)	2 (1–3)
Haiti	2036	7 (5–8)	0 (0–0)	184 (139–229)	2 (1–3)
High SDI	2022	1752 (11436–12068	1 (1–1)	268466 (260343–276589)	14 (13–15)

High SDI	2023	1842 (11190–12494	1 (1–1)	267987 (252199–283776	14 (12–15)
High SDI	2024	1932 (11009–12854	1 (0–1)	267541 (247320–287762	14 (12–15)
High SDI	2025	2022 (10843–13200	1 (0–1)	267124 (243717–290532	13 (11–15)
High SDI	2026	2111 (10678–13545	1 (0–1)	266736 (240872–292600	13 (11–15)
High SDI	2027	2201 (10510–13893	1 (0–1)	266373 (238546–294200	13 (10–15)
High SDI	2028	2291 (10337–14246	1 (0–1)	266034 (236604–295465	13 (10–15)
High SDI	2029	2381 (10157–14605	1 (0–1)	265718 (234960–296477	12 (9–15)
High SDI	2030	12471 (9970–14971)	1 (0–1)	265424 (233554–297294	12 (9–15)
High SDI	2031	12561 (9776–15345)	0 (0–1)	265149 (232341–297956	12 (8–15)
High SDI	2032	12651 (9575–15726)	0 (0–1)	264892 (231290–298494	11 (8–15)
High SDI	2033	12741 (9366–16115)	0 (0–1)	264652 (230373–298932	11 (7–15)
High SDI	2034	12830 (9149–16511)	0 (0–1)	264429 (229570–299287	11 (7–15)
High SDI	2035	12920 (8925–16915)	0 (0–1)	264220 (228865–299575	11 (6–15)
High SDI	2036	13010 (8694–17326)	0 (0–1)	264025 (228244–299807	10 (6–15)
High-middle SDI	2022	4190 (13891–14490	1 (1–1)	410350 (400813–419887	21 (20–22)
High-middle SDI	2023	4462 (13821–15102	1 (1–1)	417211 (394797–439626	21 (20–23)
High-middle SDI	2024	4697 (13784–15611	1 (1–1)	421407 (387063–455751	21 (19–24)
High-middle SDI	2025	4874 (13786–15962	1 (1–1)	425603 (382515–468691	21 (18–24)
High-middle SDI	2026	5016 (13821–16212	1 (1–1)	429798 (379463–480134	21 (18–25)
High-middle SDI	2027	5159 (13886–16432	1 (1–1)	433994 (377330–490658	21 (17–25)
High-middle SDI	2028	5318 (13971–16665	1 (1–1)	438190 (375837–500543	21 (17–25)
High-middle SDI	2029	5493 (14068–16918	1 (1–1)	442385 (374821–509950	21 (17–26)
High-middle SDI	2030	5672 (14165–17180	1 (1–1)	446581 (374179–518983	21 (16–26)
High-middle SDI	2031	5848 (14260–17437	1 (1–1)	450777 (373840–527713	21 (16–26)
High-middle SDI	2032	6019 (14356–17683	1 (1–1)	454972 (373755–536190	21 (16–27)
High-middle SDI	2033	6187 (14454–17919	1 (1–1)	459168 (373884–544452	21 (15–27)
High-middle SDI	2034	6354 (14556–18152	1 (1–1)	463364 (374198–552529	21 (15–27)
High-middle SDI	2035	6523 (14662–18384	1 (1–1)	467559 (374675–560444	21 (15–27)
High-middle SDI	2036	6694 (14771–18616	1 (1–1)	471755 (375294–568215	21 (15–28)
Honduras	2022	15 (15–16)	0 (0–0)	384 (376–393)	6 (6–6)
Honduras	2023	16 (15–16)	0 (0–0)	396 (377–415)	6 (5–6)
Honduras	2024	16 (15–17)	0 (0–0)	408 (376–440)	6 (5–6)
Honduras	2025	16 (14–19)	0 (0–0)	420 (373–467)	6 (5–6)

Honduras	2026	17 (14–20)	0 (0–0)	432 (369–495)	6 (5–7)
Honduras	2027	17 (14–21)	0 (0–0)	444 (363–525)	6 (5–7)
Honduras	2028	18 (13–22)	0 (0–0)	456 (355–557)	6 (5–7)
Honduras	2029	18 (13–24)	0 (0–0)	468 (346–590)	6 (5–7)
Honduras	2030	19 (12–25)	0 (0–0)	480 (336–624)	6 (5–7)
Honduras	2031	19 (12–27)	0 (0–0)	492 (324–659)	6 (5–7)
Honduras	2032	19 (11–28)	0 (0–0)	504 (312–695)	6 (5–7)
Honduras	2033	20 (10–30)	0 (0–0)	516 (298–733)	6 (5–8)
Honduras	2034	20 (9–31)	0 (0–0)	528 (284–772)	7 (5–8)
Honduras	2035	21 (9–33)	0 (0–0)	540 (268–811)	7 (5–8)
Honduras	2036	21 (8–35)	0 (0–0)	551 (251–852)	7 (5–8)
Hungary	2022	34 (30–38)	0 (0–0)	890 (775–1004)	5 (4–6)
Hungary	2023	34 (27–41)	0 (0–0)	886 (688–1083)	5 (4–7)
Hungary	2024	34 (25–43)	0 (0–0)	884 (617–1151)	5 (3–7)
Hungary	2025	34 (24–45)	0 (0–0)	883 (558–1209)	5 (3–7)
Hungary	2026	34 (22–47)	0 (0–0)	883 (506–1260)	5 (3–8)
Hungary	2027	34 (21–48)	0 (0–0)	883 (460–1306)	5 (2–8)
Hungary	2028	34 (20–49)	0 (0–0)	883 (419–1347)	5 (2–8)
Hungary	2029	34 (19–50)	0 (0–0)	883 (381–1385)	5 (2–9)
Hungary	2030	34 (18–51)	0 (0–0)	883 (345–1421)	5 (1–9)
Hungary	2031	34 (17–52)	0 (0–0)	883 (312–1454)	5 (1–9)
Hungary	2032	34 (16–53)	0 (0–0)	883 (281–1485)	5 (1–9)
Hungary	2033	34 (15–54)	0 (0–0)	883 (251–1515)	5 (1–10)
Hungary	2034	34 (14–55)	0 (0–0)	883 (222–1544)	5 (0–10)
Hungary	2035	34 (13–56)	0 (0–0)	883 (195–1571)	5 (0–10)
Hungary	2036	34 (12–56)	0 (0–0)	883 (169–1597)	5 (0–10)
Iceland	2022	2 (2–2)	0 (0–0)	53 (51–55)	9 (9–9)
Iceland	2023	2 (2–3)	0 (0–0)	55 (52–59)	9 (8–10)
Iceland	2024	3 (2–3)	0 (0–0)	58 (53–62)	9 (8–10)
Iceland	2025	3 (2–3)	0 (0–0)	60 (53–66)	9 (8–10)
Iceland	2026	3 (2–3)	0 (0–1)	62 (54–70)	9 (7–10)
Iceland	2027	3 (2–3)	0 (0–1)	64 (54–74)	9 (7–10)
Iceland	2028	3 (2–4)	0 (0–1)	67 (55–79)	8 (7–10)

Iceland	2029	3 (2–4)	0 (0–1)	69 (55–83)	8 (7–10)
Iceland	2030	3 (2–4)	0 (0–1)	71 (55–87)	8 (7–10)
Iceland	2031	3 (2–4)	0 (0–1)	74 (55–92)	8 (7–10)
Iceland	2032	3 (2–4)	0 (0–1)	76 (55–97)	8 (7–10)
Iceland	2033	3 (2–5)	1 (0–1)	78 (55–101)	8 (7–10)
Iceland	2034	4 (2–5)	1 (0–1)	80 (55–106)	8 (7–10)
Iceland	2035	4 (2–5)	1 (0–1)	83 (54–111)	8 (6–10)
Iceland	2036	4 (2–5)	1 (0–1)	85 (54–116)	8 (6–10)
India	2022	2595 (2541–2649)	0 (0–0)	70241 (68864–71618)	5 (5–6)
India	2023	2659 (2569–2749)	0 (0–0)	71561 (69047–74076)	5 (5–6)
India	2024	2723 (2596–2850)	0 (0–0)	72954 (69368–76540)	5 (5–6)
India	2025	2787 (2620–2954)	0 (0–0)	74377 (69769–78986)	5 (5–6)
India	2026	2851 (2641–3061)	0 (0–0)	75813 (70212–81414)	5 (5–6)
India	2027	2915 (2660–3170)	0 (0–0)	77255 (70676–83833)	5 (5–6)
India	2028	2979 (2676–3282)	0 (0–0)	78698 (71145–86252)	5 (5–6)
India	2029	3043 (2690–3396)	0 (0–0)	80143 (71611–88675)	5 (5–6)
India	2030	3107 (2701–3513)	0 (0–0)	81588 (72068–91108)	5 (5–6)
India	2031	3171 (2710–3632)	0 (0–0)	83033 (72513–93553)	5 (5–6)
India	2032	3235 (2717–3753)	0 (0–0)	84478 (72944–96012)	5 (5–6)
India	2033	3299 (2722–3876)	0 (0–0)	85924 (73359–98488)	5 (5–6)
India	2034	3363 (2725–4001)	0 (0–0)	87369 (73756–100981)	5 (5–6)
India	2035	3427 (2725–4128)	0 (0–0)	88814 (74136–103492)	5 (5–6)
India	2036	3491 (2724–4257)	0 (0–0)	90260 (74497–106022)	5 (5–6)
Indonesia	2022	1206 (1198–1214)	0 (0–0)	36672 (36431–36912)	13 (13–13)
Indonesia	2023	1247 (1229–1264)	0 (0–0)	37855 (37318–38393)	13 (12–13)
Indonesia	2024	1287 (1258–1316)	0 (0–0)	39039 (38139–39938)	13 (12–13)
Indonesia	2025	1328 (1285–1370)	0 (0–0)	40222 (38906–41539)	13 (12–14)
Indonesia	2026	1368 (1311–1426)	0 (0–1)	41406 (39623–43188)	13 (12–14)
Indonesia	2027	1409 (1335–1483)	0 (0–1)	42589 (40296–44882)	13 (12–14)
Indonesia	2028	1450 (1358–1542)	0 (0–1)	43773 (40929–46617)	13 (11–15)
Indonesia	2029	1490 (1379–1601)	0 (0–1)	44956 (41523–48389)	13 (11–15)
Indonesia	2030	1531 (1399–1662)	0 (0–1)	46140 (42082–50197)	13 (11–15)
Indonesia	2031	1571 (1419–1724)	0 (0–1)	47323 (42607–52039)	13 (10–16)

Indonesia	2032	1612 (1437–1787)	0 (0–1)	48507 (43100–53913)	13 (10–16)
Indonesia	2033	1652 (1454–1851)	0 (0–1)	49690 (43562–55818)	13 (10–17)
Indonesia	2034	1693 (1470–1916)	0 (0–1)	50873 (43995–57752)	13 (9–17)
Indonesia	2035	1734 (1486–1982)	0 (0–1)	52057 (44400–59714)	13 (9–18)
Indonesia	2036	1774 (1500–2048)	0 (0–1)	53240 (44777–61704)	13 (9–18)
Iran (Islamic Republic of)	2022	149 (144–155)	0 (0–0)	4136 (3989–4282)	5 (4–5)
Iran (Islamic Republic of)	2023	153 (141–165)	0 (0–0)	4255 (3928–4583)	5 (4–5)
Iran (Islamic Republic of)	2024	157 (137–177)	0 (0–0)	4375 (3827–4923)	5 (4–6)
Iran (Islamic Republic of)	2025	161 (132–191)	0 (0–0)	4494 (3692–5296)	5 (4–6)
Iran (Islamic Republic of)	2026	165 (125–205)	0 (0–0)	4614 (3527–5700)	5 (3–6)
Iran (Islamic Republic of)	2027	169 (118–220)	0 (0–0)	4733 (3336–6130)	5 (3–6)
Iran (Islamic Republic of)	2028	173 (110–237)	0 (0–0)	4852 (3120–6585)	5 (3–7)
Iran (Islamic Republic of)	2029	177 (101–254)	0 (0–0)	4972 (2880–7064)	5 (3–7)
Iran (Islamic Republic of)	2030	181 (91–272)	0 (0–0)	5091 (2619–7564)	5 (2–7)
Iran (Islamic Republic of)	2031	185 (80–290)	0 (0–0)	5211 (2337–8084)	5 (2–7)
Iran (Islamic Republic of)	2032	189 (69–310)	0 (0–0)	5330 (2036–8625)	5 (2–7)
Iran (Islamic Republic of)	2033	193 (56–330)	0 (0–0)	5450 (1716–9183)	5 (2–8)
Iran (Islamic Republic of)	2034	197 (44–351)	0 (0–0)	5569 (1378–9760)	5 (2–8)
Iran (Islamic Republic of)	2035	201 (30–372)	0 (0–0)	5689 (1023–10354)	5 (2–8)
Iran (Islamic Republic of)	2036	205 (16–394)	0 (0–0)	5808 (651–10965)	5 (1–8)
Iraq	2022	128 (125–132)	1 (1–1)	3445 (3354–3536)	13 (13–13)
Iraq	2023	134 (127–141)	1 (1–1)	3569 (3366–3773)	13 (12–14)
Iraq	2024	140 (129–151)	1 (0–1)	3694 (3353–4034)	13 (12–14)
Iraq	2025	146 (131–161)	1 (0–1)	3818 (3319–4317)	13 (11–14)
Iraq	2026	152 (132–172)	1 (0–1)	3942 (3267–4617)	13 (11–14)
Iraq	2027	158 (133–183)	1 (0–1)	4066 (3197–4935)	13 (11–14)
Iraq	2028	164 (134–195)	1 (0–1)	4190 (3113–5268)	13 (11–15)
Iraq	2029	170 (134–206)	1 (0–1)	4315 (3014–5615)	12 (10–15)
Iraq	2030	176 (133–219)	0 (0–1)	4439 (2901–5976)	12 (10–15)
Iraq	2031	182 (133–231)	0 (0–1)	4563 (2776–6350)	12 (10–14)
Iraq	2032	188 (132–244)	0 (0–1)	4687 (2639–6736)	12 (10–14)
Iraq	2033	194 (131–257)	0 (0–1)	4811 (2490–7133)	12 (10–14)
Iraq	2034	200 (130–270)	0 (0–1)	4936 (2329–7542)	12 (10–14)

Iraq	2035	206 (128–284)	0 (0–1)	5060 (2158–7961)	12 (10–14)
Iraq	2036	212 (126–298)	0 (0–1)	5184 (1977–8391)	12 (10–14)
Ireland	2022	26 (25–27)	0 (0–0)	572 (550–593)	7 (7–8)
Ireland	2023	26 (25–28)	0 (0–0)	581 (551–611)	7 (7–8)
Ireland	2024	27 (25–28)	0 (0–0)	591 (554–628)	7 (7–8)
Ireland	2025	27 (25–29)	0 (0–0)	601 (559–644)	7 (7–8)
Ireland	2026	28 (25–30)	0 (0–0)	611 (564–659)	7 (6–8)
Ireland	2027	28 (25–31)	0 (0–0)	621 (569–673)	7 (6–8)
Ireland	2028	28 (26–31)	0 (0–0)	631 (575–687)	7 (6–8)
Ireland	2029	29 (26–32)	0 (0–0)	641 (581–701)	7 (6–8)
Ireland	2030	29 (26–33)	0 (0–0)	651 (587–714)	7 (6–9)
Ireland	2031	30 (27–33)	0 (0–0)	660 (593–728)	7 (6–9)
Ireland	2032	30 (27–34)	0 (0–0)	670 (600–741)	7 (6–9)
Ireland	2033	31 (27–34)	0 (0–0)	680 (607–754)	7 (6–9)
Ireland	2034	31 (27–35)	0 (0–0)	690 (613–767)	7 (6–9)
Ireland	2035	32 (28–36)	0 (0–0)	700 (620–779)	7 (6–9)
Ireland	2036	32 (28–36)	0 (0–0)	710 (627–792)	7 (6–9)
Israel	2022	26 (25–27)	0 (0–0)	620 (592–649)	5 (5–6)
Israel	2023	27 (25–28)	0 (0–0)	631 (591–671)	5 (5–6)
Israel	2024	27 (26–28)	0 (0–0)	641 (592–690)	5 (5–6)
Israel	2025	27 (26–29)	0 (0–0)	651 (595–708)	5 (4–6)
Israel	2026	28 (26–29)	0 (0–0)	661 (598–725)	5 (4–6)
Israel	2027	28 (27–30)	0 (0–0)	672 (603–741)	5 (4–6)
Israel	2028	29 (27–31)	0 (0–0)	682 (607–757)	5 (4–6)
Israel	2029	29 (27–31)	0 (0–0)	692 (612–772)	5 (4–7)
Israel	2030	30 (28–32)	0 (0–0)	703 (618–787)	5 (4–7)
Israel	2031	30 (28–32)	0 (0–0)	713 (623–802)	5 (4–7)
Israel	2032	30 (28–33)	0 (0–0)	723 (629–817)	5 (4–7)
Israel	2033	31 (29–33)	0 (0–0)	733 (635–831)	5 (4–7)
Israel	2034	31 (29–34)	0 (0–0)	744 (642–845)	5 (4–7)
Israel	2035	32 (29–34)	0 (0–0)	754 (648–860)	5 (4–7)
Israel	2036	32 (30–35)	0 (0–0)	764 (655–874)	5 (4–7)
Italy	2022	596 (504–688)	0 (0–1)	13329 (11203–15455)	10 (8–13)

Italy	2023	596 (466–726)	0 (0–1)	13329 (10322–16336)	10 (7–13)
Italy	2024	596 (436–756)	0 (0–1)	13329 (9646–17011)	10 (6–13)
Italy	2025	596 (412–780)	0 (0–1)	13329 (9077–17581)	9 (5–13)
Italy	2026	596 (390–802)	0 (0–1)	13329 (8575–18083)	9 (4–13)
Italy	2027	596 (370–822)	0 (0–1)	13329 (8121–18537)	9 (3–14)
Italy	2028	596 (352–840)	0 (0–1)	13329 (7704–18954)	8 (3–14)
Italy	2029	596 (335–857)	0 (0–1)	13329 (7315–19342)	8 (2–14)
Italy	2030	596 (320–873)	0 (0–1)	13329 (6950–19707)	7 (1–13)
Italy	2031	596 (305–887)	0 (0–1)	13329 (6605–20052)	7 (0–13)
Italy	2032	596 (290–902)	0 (0–1)	13329 (6277–20380)	7 (-0–13)
Italy	2033	596 (277–915)	0 (-0–1)	13329 (5964–20694)	6 (-1–13)
Italy	2034	596 (264–928)	0 (-0–1)	13329 (5663–20995)	6 (-2–13)
Italy	2035	596 (251–941)	0 (-0–1)	13329 (5374–21284)	5 (-2–13)
Italy	2036	596 (239–953)	0 (-0–1)	13329 (5094–21563)	5 (-3–13)
Jamaica	2022	4 (3–5)	0 (0–0)	97 (72–121)	3 (2–4)
Jamaica	2023	4 (3–5)	0 (0–0)	97 (70–124)	3 (2–4)
Jamaica	2024	4 (3–5)	0 (0–0)	97 (64–130)	3 (2–4)
Jamaica	2025	4 (2–5)	0 (0–0)	97 (61–133)	3 (2–4)
Jamaica	2026	4 (2–5)	0 (0–0)	97 (57–137)	3 (2–4)
Jamaica	2027	4 (2–5)	0 (0–0)	97 (54–140)	3 (2–4)
Jamaica	2028	4 (2–5)	0 (0–0)	97 (51–143)	3 (2–4)
Jamaica	2029	4 (2–6)	0 (0–0)	97 (48–146)	3 (2–4)
Jamaica	2030	4 (2–6)	0 (0–0)	97 (46–148)	3 (2–4)
Jamaica	2031	4 (2–6)	0 (0–0)	97 (43–151)	3 (2–4)
Jamaica	2032	4 (2–6)	0 (0–0)	97 (41–153)	3 (2–4)
Jamaica	2033	4 (2–6)	0 (0–0)	97 (38–155)	3 (2–4)
Jamaica	2034	4 (2–6)	0 (0–0)	97 (36–158)	3 (2–4)
Jamaica	2035	4 (1–6)	0 (0–0)	97 (34–160)	3 (2–4)
Jamaica	2036	4 (1–6)	0 (0–0)	97 (32–162)	3 (2–4)
Japan	2022	2550 (2244–2857)	1 (1–1)	46786 (38893–54679)	15 (12–19)
Japan	2023	2575 (2000–3149)	1 (0–1)	46700 (31920–61479)	15 (8–23)
Japan	2024	2600 (1721–3479)	1 (0–1)	46613 (24012–69214)	15 (5–26)
Japan	2025	2625 (1406–3844)	1 (0–1)	46526 (15193–77859)	15 (2–29)

Japan	2026	2650 (1057–4242)	1 (0–1)	46439 (5528–87349)	15 (-1–32)
Japan	2027	2674 (679–4670)	1 (-0–1)	46352 (-4921–97625)	15 (-4–34)
Japan	2028	2699 (271–5127)	1 (-0–1)	46265 (-16103–108633)	15 (-6–37)
Japan	2029	2724 (-163–5611)	1 (-0–2)	46178 (-27976–120332)	15 (-8–39)
Japan	2030	2749 (-623–6121)	1 (-0–2)	46091 (-40501–132684)	15 (-10–41)
Japan	2031	2774 (-1107–6655)	1 (-0–2)	46004 (-53648–145656)	15 (-12–43)
Japan	2032	2799 (-1614–7211)	1 (-0–2)	45917 (-67387–159222)	15 (-14–45)
Japan	2033	2824 (-2143–7790)	1 (-0–2)	45830 (-81696–173357)	15 (-16–46)
Japan	2034	2848 (-2694–8391)	1 (-0–2)	45744 (-96551–188039)	15 (-17–48)
Japan	2035	2873 (-3265–9012)	1 (-1–2)	45657 (-111935–203248)	15 (-19–49)
Japan	2036	2898 (-3856–9652)	1 (-1–2)	45570 (-127829–218968)	15 (-20–51)
Jordan	2022	15 (15–15)	0 (0–0)	427 (422–431)	5 (4–5)
Jordan	2023	16 (15–16)	0 (0–0)	460 (446–474)	4 (4–5)
Jordan	2024	17 (16–18)	0 (0–0)	494 (468–519)	4 (4–5)
Jordan	2025	18 (17–20)	0 (0–0)	527 (488–566)	4 (4–5)
Jordan	2026	19 (17–21)	0 (0–0)	560 (506–615)	4 (4–5)
Jordan	2027	20 (18–23)	0 (0–0)	594 (523–665)	4 (4–5)
Jordan	2028	22 (18–25)	0 (0–0)	627 (538–716)	4 (4–5)
Jordan	2029	23 (19–27)	0 (0–0)	661 (552–769)	4 (3–5)
Jordan	2030	24 (19–29)	0 (0–0)	694 (565–824)	4 (3–5)
Jordan	2031	25 (19–31)	0 (0–0)	728 (576–879)	4 (3–5)
Jordan	2032	26 (20–33)	0 (0–0)	761 (587–936)	4 (3–5)
Jordan	2033	27 (20–35)	0 (0–0)	795 (596–993)	4 (3–5)
Jordan	2034	28 (20–37)	0 (0–0)	828 (604–1052)	4 (3–5)
Jordan	2035	30 (20–39)	0 (0–0)	861 (611–1112)	4 (3–5)
Jordan	2036	31 (20–41)	0 (0–0)	895 (618–1172)	4 (3–4)
Kazakhstan	2022	71 (60–83)	0 (0–0)	2147 (1784–2510)	10 (8–13)
Kazakhstan	2023	69 (53–85)	0 (0–0)	2061 (1548–2575)	10 (6–13)
Kazakhstan	2024	66 (47–86)	0 (0–0)	1976 (1347–2604)	9 (4–13)
Kazakhstan	2025	64 (41–86)	0 (0–0)	1890 (1164–2616)	8 (3–13)
Kazakhstan	2026	61 (36–87)	0 (0–0)	1805 (993–2616)	7 (2–13)
Kazakhstan	2027	59 (31–86)	0 (0–0)	1719 (830–2608)	7 (0–13)
Kazakhstan	2028	56 (26–86)	0 (0–0)	1633 (673–2594)	6 (-1–13)

Kazakhstan	2029	54 (22–86)	0 (-0–0)	1548 (521–2575)	5 (-2–12)
Kazakhstan	2030	51 (17–85)	0 (-0–0)	1462 (373–2552)	4 (-3–12)
Kazakhstan	2031	49 (13–85)	0 (-0–0)	1377 (229–2525)	4 (-5–12)
Kazakhstan	2032	46 (9–84)	0 (-0–0)	1291 (87–2496)	3 (-6–11)
Kazakhstan	2033	44 (4–83)	0 (-0–0)	1206 (-52–2464)	2 (-7–11)
Kazakhstan	2034	41 (0–82)	0 (-0–0)	1120 (-189–2430)	1 (-8–10)
Kazakhstan	2035	39 (-4–81)	0 (-0–0)	1035 (-324–2393)	0 (-9–10)
Kazakhstan	2036	36 (-8–80)	0 (-0–0)	949 (-457–2356)	-0 (-10–10)
Kenya	2022	48 (48–49)	0 (0–0)	1445 (1427–1463)	5 (5–5)
Kenya	2023	50 (49–51)	0 (0–0)	1485 (1449–1521)	5 (5–6)
Kenya	2024	51 (49–53)	0 (0–0)	1523 (1470–1576)	5 (5–6)
Kenya	2025	52 (50–55)	0 (0–0)	1560 (1491–1629)	5 (5–6)
Kenya	2026	54 (51–56)	0 (0–0)	1596 (1512–1680)	5 (5–6)
Kenya	2027	55 (51–58)	0 (0–0)	1632 (1534–1730)	5 (4–6)
Kenya	2028	56 (52–60)	0 (0–0)	1667 (1557–1778)	5 (4–6)
Kenya	2029	57 (53–61)	0 (0–0)	1702 (1580–1825)	5 (4–7)
Kenya	2030	58 (54–63)	0 (0–0)	1737 (1603–1872)	5 (4–7)
Kenya	2031	59 (55–64)	0 (0–0)	1772 (1628–1917)	5 (4–7)
Kenya	2032	61 (55–66)	0 (0–0)	1807 (1652–1962)	5 (4–7)
Kenya	2033	62 (56–67)	0 (0–0)	1842 (1678–2006)	5 (3–7)
Kenya	2034	63 (57–69)	0 (0–0)	1877 (1704–2050)	5 (3–7)
Kenya	2035	64 (58–70)	0 (0–0)	1911 (1730–2093)	5 (3–7)
Kenya	2036	65 (59–71)	0 (0–0)	1946 (1756–2136)	5 (3–7)
Kiribati	2022	1 (1–1)	1 (1–1)	24 (23–24)	26 (25–27)
Kiribati	2023	1 (1–1)	1 (1–1)	24 (23–26)	26 (23–28)
Kiribati	2024	1 (1–1)	1 (1–1)	25 (22–27)	26 (21–30)
Kiribati	2025	1 (1–1)	1 (1–1)	25 (22–29)	26 (19–32)
Kiribati	2026	1 (1–1)	1 (1–1)	26 (21–30)	26 (17–35)
Kiribati	2027	1 (1–1)	1 (0–1)	26 (20–32)	26 (14–38)
Kiribati	2028	1 (1–1)	1 (0–1)	27 (19–34)	26 (11–40)
Kiribati	2029	1 (1–1)	1 (0–1)	27 (18–36)	25 (7–44)
Kiribati	2030	1 (1–1)	1 (0–2)	28 (17–38)	25 (4–47)
Kiribati	2031	1 (1–1)	1 (-0–2)	28 (16–40)	25 (0–51)

Kiribati	2032	1 (0–1)	1 (-0–2)	28 (15–42)	25 (-4–54)
Kiribati	2033	1 (0–1)	1 (-0–2)	29 (13–45)	25 (-8–58)
Kiribati	2034	1 (0–1)	1 (-0–2)	29 (12–47)	25 (-12–62)
Kiribati	2035	1 (0–2)	1 (-1–2)	30 (10–49)	25 (-17–67)
Kiribati	2036	1 (0–2)	1 (-1–2)	30 (9–52)	25 (-21–71)
Kuwait	2022	3 (2–3)	0 (0–0)	74 (53–94)	2 (-0–4)
Kuwait	2023	2 (2–3)	0 (-0–0)	72 (44–99)	2 (-1–4)
Kuwait	2024	2 (1–3)	0 (-0–0)	70 (39–101)	1 (-2–5)
Kuwait	2025	2 (1–3)	0 (-0–0)	69 (35–102)	1 (-3–5)
Kuwait	2026	2 (1–3)	0 (-0–0)	67 (32–103)	1 (-4–5)
Kuwait	2027	2 (1–3)	0 (-0–0)	66 (29–103)	0 (-4–5)
Kuwait	2028	2 (1–3)	0 (-0–0)	65 (28–103)	0 (-5–5)
Kuwait	2029	2 (1–3)	0 (-0–0)	65 (26–103)	-0 (-6–5)
Kuwait	2030	2 (1–3)	0 (-0–0)	64 (25–103)	-1 (-6–5)
Kuwait	2031	2 (1–3)	0 (-0–0)	63 (24–103)	-1 (-7–5)
Kuwait	2032	2 (1–3)	0 (-0–0)	63 (23–103)	-1 (-8–5)
Kuwait	2033	2 (1–3)	0 (-0–0)	63 (23–102)	-1 (-8–5)
Kuwait	2034	2 (1–3)	0 (-0–0)	62 (22–102)	-2 (-9–5)
Kuwait	2035	2 (1–3)	0 (-0–0)	62 (22–102)	-2 (-9–5)
Kuwait	2036	2 (1–3)	0 (-0–0)	62 (22–102)	-2 (-10–5)
Kyrgyzstan	2022	21 (18–23)	0 (0–0)	614 (543–684)	11 (9–13)
Kyrgyzstan	2023	20 (16–25)	0 (0–1)	604 (463–746)	11 (7–16)
Kyrgyzstan	2024	20 (14–26)	0 (0–1)	595 (414–777)	12 (6–18)
Kyrgyzstan	2025	20 (13–26)	0 (0–1)	587 (377–797)	12 (4–20)
Kyrgyzstan	2026	19 (12–27)	0 (0–1)	580 (348–812)	12 (3–21)
Kyrgyzstan	2027	19 (11–27)	0 (0–1)	573 (324–822)	12 (2–23)
Kyrgyzstan	2028	19 (10–27)	0 (0–1)	566 (303–829)	13 (0–25)
Kyrgyzstan	2029	19 (10–27)	0 (0–1)	560 (286–835)	13 (-1–27)
Kyrgyzstan	2030	18 (9–27)	0 (-0–1)	555 (271–839)	13 (-2–29)
Kyrgyzstan	2031	18 (9–27)	0 (-0–1)	549 (258–841)	13 (-4–31)
Kyrgyzstan	2032	18 (9–27)	0 (-0–1)	545 (246–843)	14 (-5–33)
Kyrgyzstan	2033	18 (8–27)	1 (-0–1)	540 (236–844)	14 (-7–35)
Kyrgyzstan	2034	18 (8–27)	1 (-0–1)	536 (227–845)	14 (-8–37)

Kyrgyzstan	2035	18 (8–27)	1 (-0–1)	532 (219–845)	14 (-10–39)
Kyrgyzstan	2036	17 (7–27)	1 (-0–1)	529 (212–845)	15 (-11–41)
Lao People's Democratic Republic	2022	47 (47–48)	1 (1–1)	1420 (1404–1437)	26 (25–26)
Lao People's Democratic Republic	2023	49 (47–50)	1 (1–1)	1460 (1424–1497)	26 (25–27)
Lao People's Democratic Republic	2024	50 (48–52)	1 (1–1)	1500 (1439–1561)	26 (24–28)
Lao People's Democratic Republic	2025	51 (48–54)	1 (1–1)	1540 (1450–1629)	26 (23–28)
Lao People's Democratic Republic	2026	53 (49–56)	1 (1–1)	1580 (1459–1701)	26 (23–29)
Lao People's Democratic Republic	2027	54 (49–59)	1 (1–1)	1619 (1464–1775)	26 (22–30)
Lao People's Democratic Republic	2028	55 (49–61)	1 (1–1)	1659 (1466–1853)	26 (21–30)
Lao People's Democratic Republic	2029	57 (49–64)	1 (1–1)	1699 (1466–1932)	26 (20–31)
Lao People's Democratic Republic	2030	58 (49–67)	1 (1–1)	1739 (1463–2015)	26 (20–32)
Lao People's Democratic Republic	2031	59 (49–70)	1 (1–1)	1779 (1458–2099)	26 (19–33)
Lao People's Democratic Republic	2032	61 (49–72)	1 (1–1)	1819 (1451–2186)	26 (18–33)
Lao People's Democratic Republic	2033	62 (49–75)	1 (1–1)	1858 (1442–2275)	26 (17–34)
Lao People's Democratic Republic	2034	63 (49–78)	1 (1–1)	1898 (1431–2366)	26 (16–35)
Lao People's Democratic Republic	2035	65 (48–81)	1 (1–1)	1938 (1418–2458)	26 (16–36)
Lao People's Democratic Republic	2036	66 (48–85)	1 (1–1)	1978 (1403–2553)	26 (15–36)
Latvia	2022	13 (11–14)	0 (0–0)	342 (299–385)	10 (9–12)
Latvia	2023	12 (10–15)	0 (0–0)	345 (282–408)	10 (8–12)
Latvia	2024	12 (10–14)	0 (0–0)	346 (282–411)	10 (8–12)
Latvia	2025	12 (10–14)	0 (0–0)	347 (282–411)	10 (8–12)
Latvia	2026	12 (10–15)	0 (0–0)	347 (282–411)	10 (8–12)
Latvia	2027	12 (10–15)	0 (0–0)	347 (282–411)	10 (8–12)
Latvia	2028	12 (10–15)	0 (0–0)	347 (282–411)	10 (8–12)
Latvia	2029	12 (10–15)	0 (0–0)	347 (282–411)	10 (8–12)
Latvia	2030	12 (10–15)	0 (0–0)	347 (282–411)	10 (8–12)
Latvia	2031	12 (10–15)	0 (0–0)	347 (282–411)	10 (8–12)
Latvia	2032	12 (10–15)	0 (0–0)	347 (282–411)	10 (8–12)
Latvia	2033	12 (10–15)	0 (0–0)	347 (282–411)	10 (8–12)
Latvia	2034	12 (10–15)	0 (0–0)	347 (282–411)	10 (8–12)
Latvia	2035	12 (10–15)	0 (0–0)	347 (282–411)	10 (8–12)
Latvia	2036	12 (10–15)	0 (0–0)	347 (282–411)	10 (8–12)
Lebanon	2022	18 (18–19)	0 (0–0)	491 (483–500)	8 (8–9)

Lebanon	2023	19 (18–19)	0 (0–0)	499 (484–514)	8 (8–9)
Lebanon	2024	19 (18–20)	0 (0–0)	506 (487–526)	8 (8–9)
Lebanon	2025	19 (18–20)	0 (0–0)	514 (490–537)	8 (8–9)
Lebanon	2026	20 (19–21)	0 (0–0)	521 (495–548)	8 (7–9)
Lebanon	2027	20 (19–21)	0 (0–0)	529 (499–558)	8 (7–9)
Lebanon	2028	20 (19–22)	0 (0–0)	537 (504–569)	8 (7–9)
Lebanon	2029	21 (19–22)	0 (0–0)	544 (510–579)	8 (7–9)
Lebanon	2030	21 (19–23)	0 (0–0)	552 (515–588)	8 (7–9)
Lebanon	2031	21 (20–23)	0 (0–0)	559 (520–598)	8 (7–9)
Lebanon	2032	22 (20–23)	0 (0–0)	567 (526–608)	8 (7–9)
Lebanon	2033	22 (20–24)	0 (0–0)	574 (532–617)	8 (6–9)
Lebanon	2034	22 (20–24)	0 (0–0)	582 (537–626)	8 (6–9)
Lebanon	2035	22 (20–25)	0 (0–0)	589 (543–636)	8 (6–9)
Lebanon	2036	23 (21–25)	0 (0–0)	597 (549–645)	8 (6–9)
Lesotho	2022	19 (19–20)	2 (2–2)	643 (628–658)	51 (50–52)
Lesotho	2023	19 (18–20)	2 (2–2)	644 (607–681)	51 (48–54)
Lesotho	2024	19 (17–21)	2 (2–2)	645 (579–712)	51 (46–56)
Lesotho	2025	19 (16–22)	2 (1–2)	646 (545–748)	52 (44–59)
Lesotho	2026	19 (15–23)	2 (1–2)	648 (507–788)	52 (41–62)
Lesotho	2027	19 (13–25)	2 (1–2)	649 (464–833)	52 (39–66)
Lesotho	2028	19 (12–26)	2 (1–2)	650 (417–882)	52 (35–69)
Lesotho	2029	19 (10–27)	2 (1–3)	651 (367–934)	53 (32–73)
Lesotho	2030	19 (9–29)	2 (1–3)	652 (314–990)	53 (29–77)
Lesotho	2031	19 (7–31)	2 (1–3)	653 (257–1048)	53 (25–82)
Lesotho	2032	19 (5–33)	2 (1–3)	654 (198–1110)	54 (21–86)
Lesotho	2033	19 (3–34)	2 (1–3)	655 (136–1174)	54 (17–91)
Lesotho	2034	19 (1–36)	2 (0–3)	656 (71–1242)	54 (12–96)
Lesotho	2035	18 (-2–38)	2 (0–4)	657 (3–1311)	54 (8–101)
Lesotho	2036	18 (-4–41)	2 (0–4)	658 (-67–1384)	55 (3–106)
Liberia	2022	19 (19–20)	1 (1–1)	640 (619–661)	22 (21–24)
Liberia	2023	19 (18–21)	1 (1–1)	662 (614–710)	22 (20–25)
Liberia	2024	20 (18–21)	1 (1–1)	684 (604–764)	22 (18–26)
Liberia	2025	20 (17–22)	1 (1–1)	706 (589–823)	22 (17–28)

Liberia	2026	20 (17–23)	1 (1–1)	728 (570–886)	22 (16–29)
Liberia	2027	20 (17–24)	1 (1–1)	750 (546–954)	22 (14–30)
Liberia	2028	20 (16–24)	1 (0–1)	772 (520–1025)	22 (13–31)
Liberia	2029	20 (16–25)	1 (0–1)	794 (489–1099)	22 (12–32)
Liberia	2030	20 (15–25)	1 (0–1)	816 (456–1177)	22 (11–33)
Liberia	2031	20 (15–26)	1 (0–1)	838 (419–1257)	22 (10–34)
Liberia	2032	20 (14–26)	1 (0–1)	860 (380–1341)	22 (9–35)
Liberia	2033	20 (14–27)	1 (0–1)	882 (338–1427)	22 (8–36)
Liberia	2034	20 (14–27)	1 (0–1)	905 (293–1516)	22 (7–37)
Liberia	2035	20 (13–28)	1 (0–1)	927 (246–1607)	22 (7–38)
Liberia	2036	20 (13–28)	1 (0–1)	949 (197–1701)	22 (6–39)
Libya	2022	34 (33–34)	1 (1–1)	1063 (1049–1077)	16 (16–16)
Libya	2023	35 (33–36)	1 (1–1)	1089 (1058–1120)	16 (15–17)
Libya	2024	35 (33–37)	1 (1–1)	1114 (1062–1166)	16 (15–17)
Libya	2025	36 (33–39)	1 (1–1)	1139 (1063–1215)	16 (14–17)
Libya	2026	37 (33–41)	1 (0–1)	1164 (1061–1267)	16 (14–18)
Libya	2027	38 (33–43)	1 (0–1)	1189 (1057–1322)	16 (13–18)
Libya	2028	39 (32–45)	1 (0–1)	1215 (1051–1379)	16 (13–18)
Libya	2029	39 (32–47)	1 (0–1)	1240 (1042–1438)	15 (13–18)
Libya	2030	40 (31–49)	1 (0–1)	1265 (1031–1499)	15 (12–19)
Libya	2031	41 (31–51)	1 (0–1)	1290 (1018–1562)	15 (12–19)
Libya	2032	42 (30–54)	1 (0–1)	1316 (1004–1627)	15 (12–19)
Libya	2033	43 (29–56)	1 (0–1)	1341 (988–1694)	15 (11–20)
Libya	2034	43 (28–58)	1 (0–1)	1366 (969–1763)	15 (11–20)
Libya	2035	44 (27–61)	1 (0–1)	1391 (950–1833)	15 (11–20)
Libya	2036	45 (26–64)	1 (0–1)	1417 (929–1905)	15 (11–20)
Lithuania	2022	20 (19–22)	0 (0–0)	532 (487–577)	11 (10–12)
Lithuania	2023	20 (18–23)	0 (0–0)	539 (475–603)	11 (10–12)
Lithuania	2024	21 (18–23)	0 (0–0)	547 (469–625)	11 (10–13)
Lithuania	2025	21 (18–24)	0 (0–0)	555 (464–645)	11 (9–13)
Lithuania	2026	21 (18–25)	0 (0–0)	562 (461–663)	12 (9–14)
Lithuania	2027	22 (18–26)	0 (0–0)	570 (459–680)	12 (9–14)
Lithuania	2028	22 (18–26)	0 (0–0)	577 (458–697)	12 (9–14)

Lithuania	2029	22 (18–27)	0 (0–1)	585 (457–713)	12 (9–15)
Lithuania	2030	23 (18–27)	0 (0–1)	593 (457–728)	12 (9–15)
Lithuania	2031	23 (18–28)	0 (0–1)	600 (457–743)	12 (9–15)
Lithuania	2032	23 (18–28)	0 (0–1)	608 (458–758)	12 (9–16)
Lithuania	2033	24 (18–29)	0 (0–1)	616 (459–772)	13 (9–16)
Lithuania	2034	24 (19–30)	0 (0–1)	623 (460–786)	13 (9–16)
Lithuania	2035	24 (19–30)	0 (0–1)	631 (462–800)	13 (9–16)
Lithuania	2036	25 (19–31)	0 (0–1)	638 (463–813)	13 (9–17)
Low SDI	2022	1412 (1397–1426)	0 (0–0)	41781 (41351–42210)	7 (7–7)
Low SDI	2023	1434 (1406–1462)	0 (0–0)	42487 (41638–43337)	7 (7–7)
Low SDI	2024	1455 (1414–1496)	0 (0–0)	43140 (41868–44412)	7 (7–7)
Low SDI	2025	1475 (1421–1529)	0 (0–0)	43755 (42075–45435)	7 (6–8)
Low SDI	2026	1494 (1428–1560)	0 (0–0)	44343 (42276–46410)	7 (6–8)
Low SDI	2027	1513 (1436–1590)	0 (0–0)	44912 (42481–47343)	7 (6–8)
Low SDI	2028	1532 (1444–1619)	0 (0–0)	45468 (42696–48240)	7 (6–8)
Low SDI	2029	1550 (1452–1647)	0 (0–0)	46014 (42923–49106)	7 (5–8)
Low SDI	2030	1568 (1462–1674)	0 (0–0)	46554 (43162–49946)	7 (5–9)
Low SDI	2031	1586 (1471–1701)	0 (0–0)	47089 (43414–50764)	7 (5–9)
Low SDI	2032	1604 (1481–1727)	0 (0–0)	47621 (43679–51564)	7 (4–9)
Low SDI	2033	1622 (1491–1753)	0 (0–0)	48151 (43954–52347)	7 (4–10)
Low SDI	2034	1640 (1502–1778)	0 (0–0)	48678 (44241–53116)	7 (3–10)
Low SDI	2035	1658 (1513–1802)	0 (0–0)	49205 (44537–53873)	7 (3–10)
Low SDI	2036	1675 (1524–1827)	0 (0–0)	49731 (44843–54619)	7 (3–11)
Low-middle SDI	2022	5672 (5603–5741)	0 (0–0)	161665 (159653–163676	10 (10–10)
Low-middle SDI	2023	5797 (5662–5932)	0 (0–0)	164783 (160903–168664	10 (10–10)
Low-middle SDI	2024	5922 (5733–6111)	0 (0–0)	167902 (162575–173228	10 (10–11)
Low-middle SDI	2025	6047 (5807–6288)	0 (0–0)	171020 (164371–177669	10 (10–11)
Low-middle SDI	2026	6173 (5881–6465)	0 (0–0)	174139 (166215–182063	10 (10–11)
Low-middle SDI	2027	6298 (5954–6642)	0 (0–0)	177257 (168074–186440	10 (10–11)
Low-middle SDI	2028	6423 (6026–6820)	0 (0–0)	180376 (169933–190818	10 (10–11)
Low-middle SDI	2029	6548 (6096–7000)	0 (0–0)	183494 (171783–195205	10 (10–11)
Low-middle SDI	2030	6673 (6165–7181)	0 (0–0)	186612 (173617–199608	10 (10–11)
Low-middle SDI	2031	6799 (6233–7364)	0 (0–0)	189731 (175433–204029	10 (9–11)

Low-middle SDI	2032	6924 (6299–7548)	0 (0–0)	192849 (177227–208471)	10 (9–11)
Low-middle SDI	2033	7049 (6364–7734)	0 (0–0)	195968 (178999–212936)	10 (9–11)
Low-middle SDI	2034	7174 (6427–7921)	0 (0–0)	199086 (180748–217424)	10 (9–11)
Low-middle SDI	2035	7299 (6488–8110)	0 (0–0)	202205 (182473–221937)	10 (9–11)
Low-middle SDI	2036	7424 (6548–8300)	0 (0–0)	205323 (184172–226473)	10 (9–11)
Luxembourg	2022	5 (4–5)	0 (0–0)	106 (101–110)	10 (10–11)
Luxembourg	2023	5 (4–5)	0 (0–0)	107 (99–115)	11 (10–12)
Luxembourg	2024	5 (4–5)	0 (0–0)	109 (99–119)	11 (10–12)
Luxembourg	2025	5 (4–5)	0 (0–1)	110 (98–122)	11 (10–13)
Luxembourg	2026	5 (4–5)	0 (0–1)	112 (98–125)	11 (10–13)
Luxembourg	2027	5 (4–6)	0 (0–1)	113 (99–128)	11 (10–13)
Luxembourg	2028	5 (4–6)	0 (0–1)	115 (99–131)	11 (10–13)
Luxembourg	2029	5 (4–6)	0 (0–1)	116 (99–133)	11 (10–13)
Luxembourg	2030	5 (4–6)	0 (0–1)	118 (99–136)	11 (10–13)
Luxembourg	2031	5 (4–6)	0 (0–1)	119 (100–138)	11 (10–13)
Luxembourg	2032	5 (4–6)	0 (0–1)	121 (100–141)	11 (10–13)
Luxembourg	2033	5 (4–6)	0 (0–1)	122 (101–143)	11 (10–13)
Luxembourg	2034	5 (4–6)	0 (0–1)	124 (102–146)	11 (10–13)
Luxembourg	2035	5 (4–6)	0 (0–1)	125 (102–148)	11 (10–13)
Luxembourg	2036	6 (5–7)	0 (0–1)	127 (103–150)	11 (10–13)
Madagascar	2022	8 (6–9)	0 (0–0)	283 (275–290)	2 (2–2)
Madagascar	2023	8 (6–9)	0 (0–0)	284 (273–296)	2 (2–2)
Madagascar	2024	8 (6–9)	0 (0–0)	283 (264–302)	2 (2–2)
Madagascar	2025	8 (6–9)	0 (0–0)	281 (257–306)	2 (2–2)
Madagascar	2026	8 (6–9)	0 (0–0)	279 (250–309)	2 (2–2)
Madagascar	2027	8 (6–9)	0 (0–0)	278 (245–310)	2 (2–3)
Madagascar	2028	8 (6–9)	0 (0–0)	276 (241–311)	2 (1–3)
Madagascar	2029	8 (6–9)	0 (0–0)	275 (237–312)	2 (1–3)
Madagascar	2030	8 (6–9)	0 (0–0)	273 (234–312)	2 (1–3)
Madagascar	2031	8 (6–9)	0 (0–0)	272 (232–312)	2 (1–3)
Madagascar	2032	8 (6–9)	0 (0–0)	271 (229–312)	2 (1–3)
Madagascar	2033	8 (6–9)	0 (0–0)	270 (227–312)	2 (1–3)
Madagascar	2034	8 (6–9)	0 (0–0)	269 (225–312)	2 (1–4)

Madagascar	2035	8 (6–9)	0 (0–0)	268 (223–312)	2 (0–4)
Madagascar	2036	8 (6–9)	0 (0–0)	267 (222–311)	2 (0–4)
Malawi	2022	30 (29–31)	0 (0–0)	885 (864–906)	10 (10–11)
Malawi	2023	31 (29–32)	0 (0–0)	902 (855–949)	10 (10–11)
Malawi	2024	31 (29–34)	0 (0–0)	920 (841–998)	11 (9–12)
Malawi	2025	32 (28–36)	0 (0–0)	937 (822–1052)	11 (9–12)
Malawi	2026	32 (27–38)	0 (0–0)	955 (799–1110)	11 (9–13)
Malawi	2027	33 (26–40)	0 (0–1)	972 (772–1172)	11 (8–14)
Malawi	2028	33 (25–42)	0 (0–1)	990 (742–1238)	11 (8–14)
Malawi	2029	34 (24–44)	0 (0–1)	1007 (708–1306)	11 (8–15)
Malawi	2030	35 (23–47)	0 (0–1)	1025 (671–1378)	12 (8–15)
Malawi	2031	35 (21–49)	0 (0–1)	1042 (631–1453)	12 (8–16)
Malawi	2032	36 (20–52)	0 (0–1)	1060 (589–1531)	12 (8–16)
Malawi	2033	36 (18–54)	0 (0–1)	1077 (543–1611)	12 (8–16)
Malawi	2034	37 (17–57)	0 (0–1)	1095 (495–1694)	12 (8–17)
Malawi	2035	37 (15–60)	0 (0–1)	1112 (445–1780)	12 (8–17)
Malawi	2036	38 (13–63)	0 (0–1)	1130 (392–1867)	12 (8–17)
Malaysia	2022	169 (163–175)	1 (1–1)	4904 (4716–5092)	16 (14–17)
Malaysia	2023	172 (164–181)	1 (1–1)	5011 (4746–5277)	16 (14–17)
Malaysia	2024	176 (166–187)	1 (0–1)	5119 (4794–5444)	16 (14–18)
Malaysia	2025	180 (168–192)	1 (0–1)	5226 (4851–5602)	16 (14–18)
Malaysia	2026	184 (170–197)	1 (0–1)	5334 (4914–5753)	16 (13–18)
Malaysia	2027	187 (172–202)	1 (0–1)	5441 (4981–5901)	16 (13–18)
Malaysia	2028	191 (175–207)	1 (0–1)	5548 (5052–6045)	16 (13–18)
Malaysia	2029	195 (178–212)	1 (0–1)	5656 (5125–6187)	16 (13–19)
Malaysia	2030	198 (180–217)	1 (0–1)	5763 (5200–6326)	16 (13–19)
Malaysia	2031	202 (183–221)	1 (0–1)	5871 (5277–6464)	16 (13–19)
Malaysia	2032	206 (186–226)	1 (0–1)	5978 (5355–6601)	16 (13–19)
Malaysia	2033	210 (189–231)	1 (0–1)	6085 (5435–6736)	16 (13–19)
Malaysia	2034	213 (191–235)	1 (0–1)	6193 (5516–6870)	16 (13–19)
Malaysia	2035	217 (194–240)	1 (0–1)	6300 (5598–7003)	16 (13–19)
Malaysia	2036	221 (197–244)	1 (0–1)	6408 (5680–7135)	16 (13–19)
Maldives	2022	3 (2–3)	1 (1–1)	66 (64–67)	17 (17–18)

Maldives	2023	3 (3–3)	1 (1–1)	70 (68–73)	18 (17–19)
Maldives	2024	3 (3–3)	1 (1–1)	75 (71–79)	18 (16–20)
Maldives	2025	3 (3–3)	1 (1–1)	80 (75–86)	18 (16–21)
Maldives	2026	3 (3–4)	1 (1–1)	85 (77–93)	19 (15–23)
Maldives	2027	3 (3–4)	1 (1–1)	90 (80–100)	19 (14–24)
Maldives	2028	4 (3–4)	1 (0–1)	95 (83–108)	19 (13–26)
Maldives	2029	4 (3–4)	1 (0–1)	100 (85–115)	20 (12–27)
Maldives	2030	4 (3–5)	1 (0–1)	105 (87–123)	20 (11–29)
Maldives	2031	4 (3–5)	1 (0–2)	110 (89–131)	20 (10–31)
Maldives	2032	4 (3–5)	1 (0–2)	115 (91–139)	21 (9–33)
Maldives	2033	4 (3–6)	1 (0–2)	120 (93–147)	21 (7–35)
Maldives	2034	5 (3–6)	1 (0–2)	125 (94–155)	21 (6–37)
Maldives	2035	5 (3–6)	1 (-0–2)	130 (96–163)	22 (5–39)
Maldives	2036	5 (3–7)	1 (-0–2)	135 (97–172)	22 (3–41)
Mali	2022	148 (146–150)	2 (2–2)	4407 (4344–4471)	43 (42–44)
Mali	2023	152 (147–157)	2 (2–2)	4553 (4411–4694)	44 (42–45)
Mali	2024	157 (149–164)	2 (2–2)	4698 (4461–4935)	44 (41–46)
Mali	2025	161 (150–172)	2 (2–2)	4844 (4496–5191)	44 (41–47)
Mali	2026	165 (150–181)	2 (2–2)	4989 (4519–5459)	44 (40–48)
Mali	2027	170 (150–189)	2 (2–2)	5135 (4530–5739)	44 (39–49)
Mali	2028	174 (150–199)	2 (2–2)	5280 (4530–6030)	44 (39–50)
Mali	2029	179 (149–208)	2 (1–2)	5425 (4520–6331)	44 (38–51)
Mali	2030	183 (148–218)	2 (1–2)	5571 (4501–6641)	44 (37–51)
Mali	2031	187 (147–228)	2 (1–2)	5716 (4473–6960)	44 (37–52)
Mali	2032	192 (145–238)	2 (1–2)	5862 (4436–7287)	44 (36–53)
Mali	2033	196 (144–249)	2 (1–2)	6007 (4392–7623)	44 (36–53)
Mali	2034	201 (141–260)	2 (1–2)	6153 (4339–7966)	44 (35–54)
Mali	2035	205 (139–271)	2 (1–2)	6298 (4279–8317)	44 (35–54)
Mali	2036	209 (137–282)	2 (1–2)	6444 (4212–8675)	44 (34–55)
Malta	2022	3 (2–3)	0 (0–0)	59 (57–61)	7 (6–7)
Malta	2023	3 (2–3)	0 (0–0)	60 (57–63)	7 (6–7)
Malta	2024	3 (2–3)	0 (0–0)	61 (57–65)	7 (6–7)
Malta	2025	3 (2–3)	0 (0–0)	62 (58–66)	7 (6–7)

Malta	2026	3 (2–3)	0 (0–0)	63 (58–68)	7 (6–7)
Malta	2027	3 (2–3)	0 (0–0)	64 (59–69)	7 (6–8)
Malta	2028	3 (2–3)	0 (0–0)	65 (60–71)	7 (6–8)
Malta	2029	3 (2–3)	0 (0–0)	66 (60–72)	7 (6–8)
Malta	2030	3 (2–4)	0 (0–0)	67 (61–74)	7 (6–8)
Malta	2031	3 (2–4)	0 (0–0)	68 (62–75)	7 (6–8)
Malta	2032	3 (2–4)	0 (0–0)	70 (63–77)	7 (6–8)
Malta	2033	3 (2–4)	0 (0–0)	71 (63–78)	7 (6–8)
Malta	2034	3 (2–4)	0 (0–0)	72 (64–79)	7 (6–8)
Malta	2035	3 (2–4)	0 (0–0)	73 (65–81)	7 (5–8)
Malta	2036	3 (2–4)	0 (0–0)	74 (66–82)	7 (5–8)
Marshall Islands	2022	0 (0–0)	0 (0–0)	4 (4–4)	9 (9–9)
Marshall Islands	2023	0 (0–0)	0 (0–0)	4 (4–4)	9 (9–10)
Marshall Islands	2024	0 (0–0)	0 (0–0)	4 (4–5)	9 (8–10)
Marshall Islands	2025	0 (0–0)	0 (0–0)	5 (4–5)	9 (8–10)
Marshall Islands	2026	0 (0–0)	0 (0–0)	5 (4–5)	9 (8–10)
Marshall Islands	2027	0 (0–0)	0 (0–0)	5 (4–5)	9 (8–11)
Marshall Islands	2028	0 (0–0)	0 (0–0)	5 (4–5)	9 (8–11)
Marshall Islands	2029	0 (0–0)	0 (0–0)	5 (5–5)	9 (7–11)
Marshall Islands	2030	0 (0–0)	0 (0–0)	5 (5–5)	9 (7–11)
Marshall Islands	2031	0 (0–0)	0 (0–0)	5 (5–5)	9 (7–11)
Marshall Islands	2032	0 (0–0)	0 (0–0)	5 (5–6)	9 (7–12)
Marshall Islands	2033	0 (0–0)	0 (0–0)	5 (5–6)	9 (7–12)
Marshall Islands	2034	0 (0–0)	0 (0–0)	5 (5–6)	9 (7–12)
Marshall Islands	2035	0 (0–0)	0 (0–0)	5 (5–6)	9 (7–12)
Marshall Islands	2036	0 (0–0)	0 (0–0)	6 (5–6)	9 (6–12)
Mauritania	2022	24 (23–25)	1 (1–1)	739 (713–765)	29 (28–31)
Mauritania	2023	25 (23–26)	1 (1–1)	762 (716–808)	30 (27–32)
Mauritania	2024	26 (23–28)	1 (1–1)	786 (717–854)	30 (25–34)
Mauritania	2025	26 (24–29)	1 (1–1)	809 (716–902)	30 (24–36)
Mauritania	2026	27 (23–31)	1 (1–1)	832 (712–952)	30 (23–38)
Mauritania	2027	28 (23–33)	1 (1–1)	856 (707–1005)	31 (21–40)
Mauritania	2028	29 (23–35)	1 (1–2)	879 (700–1059)	31 (19–43)

Mauritania	2029	30 (23–36)	1 (1–2)	903 (691–1115)	31 (17–45)
Mauritania	2030	31 (23–38)	1 (1–2)	926 (680–1172)	32 (15–48)
Mauritania	2031	31 (22–40)	1 (1–2)	949 (667–1231)	32 (13–50)
Mauritania	2032	32 (22–42)	1 (0–2)	973 (654–1292)	32 (11–53)
Mauritania	2033	33 (22–44)	1 (0–2)	996 (638–1354)	32 (9–56)
Mauritania	2034	34 (21–47)	1 (0–2)	1020 (621–1418)	33 (6–59)
Mauritania	2035	35 (21–49)	1 (0–2)	1043 (603–1483)	33 (4–62)
Mauritania	2036	36 (20–51)	1 (0–2)	1067 (583–1550)	33 (1–65)
Mauritius	2022	1 (0–2)	0 (-0–0)	38 (11–65)	2 (-1–5)
Mauritius	2023	1 (0–3)	0 (-0–0)	38 (-1–76)	3 (-1–7)
Mauritius	2024	1 (-0–3)	0 (-0–0)	38 (-9–85)	3 (-1–7)
Mauritius	2025	1 (-0–3)	0 (-0–0)	38 (-17–92)	3 (-1–7)
Mauritius	2026	1 (-1–3)	0 (-0–0)	38 (-23–98)	3 (-1–7)
Mauritius	2027	1 (-1–4)	0 (-0–0)	38 (-29–104)	3 (-1–7)
Mauritius	2028	1 (-1–4)	0 (-0–0)	38 (-34–109)	3 (-1–7)
Mauritius	2029	1 (-1–4)	0 (-0–0)	38 (-39–114)	3 (-1–7)
Mauritius	2030	1 (-1–4)	0 (-0–0)	38 (-44–119)	3 (-1–7)
Mauritius	2031	1 (-2–4)	0 (-0–0)	38 (-48–123)	3 (-1–7)
Mauritius	2032	1 (-2–4)	0 (-0–0)	38 (-52–127)	3 (-1–7)
Mauritius	2033	1 (-2–5)	0 (-0–0)	38 (-56–131)	3 (-1–7)
Mauritius	2034	1 (-2–5)	0 (-0–0)	38 (-60–135)	3 (-1–7)
Mauritius	2035	1 (-2–5)	0 (-0–0)	38 (-64–139)	3 (-1–7)
Mauritius	2036	1 (-2–5)	0 (-0–0)	38 (-67–142)	3 (-1–7)
Mexico	2022	256 (245–266)	0 (0–0)	6094 (5837–6352)	5 (4–5)
Mexico	2023	261 (246–275)	0 (0–0)	6215 (5851–6579)	5 (4–5)
Mexico	2024	266 (248–284)	0 (0–0)	6336 (5890–6782)	5 (4–5)
Mexico	2025	271 (251–292)	0 (0–0)	6456 (5941–6971)	5 (4–5)
Mexico	2026	277 (254–299)	0 (0–0)	6577 (6001–7153)	5 (4–6)
Mexico	2027	282 (257–307)	0 (0–0)	6698 (6067–7328)	5 (4–6)
Mexico	2028	287 (260–314)	0 (0–0)	6818 (6137–7500)	5 (4–6)
Mexico	2029	292 (263–321)	0 (0–0)	6939 (6210–7667)	5 (3–6)
Mexico	2030	297 (267–328)	0 (0–0)	7059 (6287–7832)	5 (3–6)
Mexico	2031	303 (270–335)	0 (0–0)	7180 (6366–7994)	5 (3–6)

Mexico	2032	308 (274–342)	0 (0–0)	7301 (6447–8155)	5 (3–6)
Mexico	2033	313 (278–348)	0 (0–0)	7421 (6529–8313)	5 (3–6)
Mexico	2034	318 (282–355)	0 (0–0)	7542 (6614–8471)	5 (3–6)
Mexico	2035	324 (285–362)	0 (0–0)	7663 (6699–8626)	5 (3–6)
Mexico	2036	329 (289–368)	0 (0–0)	7783 (6786–8781)	5 (3–6)
Micronesia (Federated States of)	2022	1 (1–1)	1 (1–1)	19 (19–20)	21 (20–21)
Micronesia (Federated States of)	2023	1 (1–1)	1 (1–1)	20 (19–20)	21 (20–22)
Micronesia (Federated States of)	2024	1 (1–1)	1 (1–1)	20 (19–21)	21 (19–23)
Micronesia (Federated States of)	2025	1 (1–1)	1 (1–1)	20 (19–22)	21 (18–24)
Micronesia (Federated States of)	2026	1 (1–1)	1 (1–1)	21 (19–22)	21 (17–25)
Micronesia (Federated States of)	2027	1 (1–1)	1 (1–1)	21 (19–23)	21 (16–26)
Micronesia (Federated States of)	2028	1 (1–1)	1 (0–1)	21 (19–23)	21 (15–28)
Micronesia (Federated States of)	2029	1 (1–1)	1 (0–1)	21 (19–24)	22 (14–29)
Micronesia (Federated States of)	2030	1 (1–1)	1 (0–1)	22 (19–24)	22 (13–31)
Micronesia (Federated States of)	2031	1 (1–1)	1 (0–1)	22 (19–25)	22 (11–32)
Micronesia (Federated States of)	2032	1 (1–1)	1 (0–1)	22 (19–25)	22 (10–34)
Micronesia (Federated States of)	2033	1 (1–1)	1 (0–1)	22 (19–25)	22 (8–35)
Micronesia (Federated States of)	2034	1 (1–1)	1 (0–1)	23 (20–26)	22 (7–37)
Micronesia (Federated States of)	2035	1 (1–1)	1 (0–1)	23 (20–26)	22 (5–39)
Micronesia (Federated States of)	2036	1 (1–1)	1 (0–1)	23 (20–27)	22 (4–41)
Middle SDI	2022	0719 (20382–21055	1 (1–1)	15002 (604429–625575	21 (20–21)
Middle SDI	2023	1007 (20268–21746	1 (1–1)	23002 (599501–646503	21 (19–22)
Middle SDI	2024	1319 (20194–22445	1 (1–1)	32079 (596252–667906	20 (19–22)
Middle SDI	2025	1632 (20178–23086	1 (1–1)	40984 (594978–686991	20 (18–22)
Middle SDI	2026	1937 (20215–23659	1 (1–1)	49293 (595308–703279	20 (17–22)
Middle SDI	2027	2235 (20292–24178	1 (1–1)	57083 (596759–717406	19 (17–22)
Middle SDI	2028	2528 (20396–24661	1 (1–1)	64597 (598968–730227	19 (16–22)
Middle SDI	2029	2819 (20518–25121	1 (1–1)	72048 (601701–742396	19 (16–22)
Middle SDI	2030	3111 (20654–25567	1 (1–1)	79545 (604806–754284	19 (15–22)
Middle SDI	2031	3403 (20800–26005	1 (1–1)	87114 (608189–766038	18 (15–22)
Middle SDI	2032	3695 (20954–26436	1 (1–1)	94734 (611788–777680	18 (14–22)
Middle SDI	2033	3987 (21114–26861	1 (1–1)	702376 (615565–789188	18 (14–22)
Middle SDI	2034	4280 (21280–27280	1 (0–1)	710021 (619496–800545	17 (13–21)

Middle SDI	2035	4573 (21451–27695	1 (0–1)	717657 (623567–811747	17 (13–21)
Middle SDI	2036	4866 (21626–28105	1 (0–1)	725286 (627765–822807	17 (12–21)
Monaco	2022	1 (1–1)	1 (1–1)	18 (18–18)	22 (22–22)
Monaco	2023	1 (1–1)	1 (1–1)	18 (17–19)	22 (21–23)
Monaco	2024	1 (1–1)	1 (1–1)	18 (17–20)	22 (20–24)
Monaco	2025	1 (1–1)	1 (1–1)	18 (16–20)	22 (19–26)
Monaco	2026	1 (1–1)	1 (1–1)	19 (16–21)	22 (18–27)
Monaco	2027	1 (1–1)	1 (1–1)	19 (15–22)	23 (16–29)
Monaco	2028	1 (1–1)	1 (1–1)	19 (14–23)	23 (15–31)
Monaco	2029	1 (1–1)	1 (0–1)	19 (14–24)	23 (13–33)
Monaco	2030	1 (1–1)	1 (0–1)	19 (13–26)	23 (11–35)
Monaco	2031	1 (0–1)	1 (0–1)	19 (12–27)	23 (9–37)
Monaco	2032	1 (0–1)	1 (0–2)	19 (11–28)	23 (7–40)
Monaco	2033	1 (0–1)	1 (0–2)	20 (10–29)	23 (4–42)
Monaco	2034	1 (0–2)	1 (0–2)	20 (9–31)	24 (2–45)
Monaco	2035	1 (0–2)	1 (-0–2)	20 (8–32)	24 (-0–48)
Monaco	2036	1 (0–2)	1 (-0–2)	20 (6–34)	24 (-3–50)
Mongolia	2022	198 (193–203)	8 (7–8)	6122 (5971–6274)	215 (204–225)
Mongolia	2023	202 (192–212)	8 (7–8)	6251 (5954–6549)	215 (195–234)
Mongolia	2024	206 (193–219)	8 (7–9)	6380 (5989–6772)	215 (189–240)
Mongolia	2025	210 (195–226)	8 (7–9)	6509 (6042–6977)	215 (185–245)
Mongolia	2026	215 (197–232)	8 (6–9)	6638 (6106–7171)	215 (180–249)
Mongolia	2027	219 (199–238)	8 (6–9)	6767 (6176–7359)	215 (177–253)
Mongolia	2028	223 (201–244)	8 (6–9)	6897 (6253–7540)	215 (173–256)
Mongolia	2029	227 (204–250)	8 (6–9)	7026 (6333–7718)	215 (170–259)
Mongolia	2030	231 (206–255)	8 (6–9)	7155 (6416–7893)	215 (167–262)
Mongolia	2031	235 (209–261)	8 (6–10)	7284 (6502–8065)	215 (164–265)
Mongolia	2032	239 (212–266)	8 (6–10)	7413 (6591–8234)	215 (162–268)
Mongolia	2033	243 (215–272)	8 (6–10)	7542 (6681–8402)	215 (159–270)
Mongolia	2034	247 (218–277)	8 (6–10)	7671 (6773–8568)	215 (157–273)
Mongolia	2035	251 (220–282)	8 (5–10)	7800 (6866–8733)	215 (155–275)
Mongolia	2036	255 (223–288)	8 (5–10)	7929 (6961–8896)	215 (153–277)
Montenegro	2022	8 (8–9)	1 (1–1)	217 (209–225)	22 (21–23)

Montenegro	2023	8 (8–9)	1 (1–1)	215 (202–228)	21 (20–23)
Montenegro	2024	8 (7–9)	1 (1–1)	213 (195–232)	21 (19–23)
Montenegro	2025	8 (7–9)	1 (1–1)	212 (188–235)	21 (18–23)
Montenegro	2026	8 (7–9)	1 (1–1)	210 (181–239)	20 (17–23)
Montenegro	2027	8 (7–9)	1 (1–1)	208 (173–243)	20 (16–24)
Montenegro	2028	8 (6–10)	1 (1–1)	206 (166–247)	20 (15–24)
Montenegro	2029	8 (6–10)	1 (1–1)	205 (158–252)	19 (14–24)
Montenegro	2030	8 (6–10)	1 (0–1)	203 (149–257)	19 (13–25)
Montenegro	2031	8 (5–10)	1 (0–1)	201 (141–262)	18 (12–25)
Montenegro	2032	8 (5–10)	1 (0–1)	199 (132–267)	18 (11–25)
Montenegro	2033	8 (5–11)	1 (0–1)	198 (123–273)	18 (10–26)
Montenegro	2034	8 (4–11)	1 (0–1)	196 (114–278)	17 (8–26)
Montenegro	2035	8 (4–11)	1 (0–1)	194 (104–284)	17 (7–27)
Montenegro	2036	8 (4–11)	1 (0–1)	193 (94–291)	17 (6–27)
Morocco	2022	14 (14–14)	0 (0–0)	411 (406–416)	1 (1–1)
Morocco	2023	14 (14–15)	0 (0–0)	427 (416–438)	1 (1–1)
Morocco	2024	15 (14–16)	0 (0–0)	442 (424–461)	1 (1–1)
Morocco	2025	16 (15–17)	0 (0–0)	458 (431–485)	1 (1–1)
Morocco	2026	16 (15–18)	0 (0–0)	474 (437–510)	1 (1–1)
Morocco	2027	17 (15–18)	0 (0–0)	490 (443–537)	1 (1–1)
Morocco	2028	17 (15–19)	0 (0–0)	506 (447–564)	1 (1–1)
Morocco	2029	18 (15–20)	0 (0–0)	521 (451–592)	1 (1–1)
Morocco	2030	18 (15–22)	0 (0–0)	537 (454–620)	1 (1–2)
Morocco	2031	19 (15–23)	0 (0–0)	553 (457–650)	1 (1–2)
Morocco	2032	19 (15–24)	0 (0–0)	569 (458–680)	1 (1–2)
Morocco	2033	20 (15–25)	0 (0–0)	585 (459–710)	1 (1–2)
Morocco	2034	21 (15–26)	0 (0–0)	601 (460–741)	1 (1–2)
Morocco	2035	21 (15–27)	0 (0–0)	616 (460–773)	1 (1–2)
Morocco	2036	22 (15–28)	0 (0–0)	632 (459–805)	1 (0–2)
Mozambique	2022	146 (144–148)	1 (1–1)	4220 (4162–4278)	34 (33–34)
Mozambique	2023	146 (141–151)	1 (1–1)	4225 (4095–4355)	33 (31–35)
Mozambique	2024	146 (138–154)	1 (1–1)	4230 (4013–4447)	32 (30–35)
Mozambique	2025	146 (134–158)	1 (1–1)	4234 (3917–4552)	32 (28–36)

Mozambique	2026	146 (130–162)	1 (1–1)	4239 (3809–4669)	31 (26–36)
Mozambique	2027	146 (126–166)	1 (1–2)	4244 (3691–4797)	30 (24–37)
Mozambique	2028	146 (121–171)	1 (1–2)	4248 (3563–4934)	30 (21–38)
Mozambique	2029	146 (116–177)	1 (1–2)	4253 (3425–5081)	29 (19–39)
Mozambique	2030	146 (110–182)	1 (1–2)	4258 (3279–5236)	29 (16–41)
Mozambique	2031	146 (104–188)	1 (1–2)	4263 (3125–5400)	28 (14–42)
Mozambique	2032	146 (98–194)	1 (0–2)	4267 (2964–5571)	27 (11–43)
Mozambique	2033	146 (92–201)	1 (0–2)	4272 (2794–5749)	27 (8–45)
Mozambique	2034	146 (85–207)	1 (0–2)	4277 (2618–5935)	26 (5–46)
Mozambique	2035	146 (78–214)	1 (0–2)	4281 (2435–6128)	25 (2–48)
Mozambique	2036	146 (71–221)	1 (-0–2)	4286 (2245–6327)	25 (-1–50)
Myanmar	2022	147 (133–162)	0 (0–0)	4174 (3814–4535)	9 (9–9)
Myanmar	2023	147 (133–162)	0 (0–0)	4174 (3814–4535)	9 (9–9)
Myanmar	2024	147 (133–162)	0 (0–0)	4174 (3814–4535)	9 (8–10)
Myanmar	2025	147 (133–162)	0 (0–0)	4174 (3814–4535)	9 (8–10)
Myanmar	2026	147 (133–162)	0 (0–0)	4174 (3814–4535)	9 (8–10)
Myanmar	2027	147 (133–162)	0 (0–0)	4174 (3814–4535)	9 (8–11)
Myanmar	2028	147 (133–162)	0 (0–0)	4174 (3814–4535)	9 (7–11)
Myanmar	2029	147 (133–162)	0 (0–0)	4174 (3814–4535)	9 (7–12)
Myanmar	2030	147 (133–162)	0 (0–0)	4174 (3814–4535)	9 (6–12)
Myanmar	2031	147 (133–162)	0 (0–0)	4174 (3814–4535)	9 (6–13)
Myanmar	2032	147 (133–162)	0 (0–0)	4174 (3814–4535)	9 (6–13)
Myanmar	2033	147 (133–162)	0 (0–1)	4174 (3814–4535)	9 (5–14)
Myanmar	2034	147 (133–162)	0 (0–1)	4174 (3814–4535)	9 (5–14)
Myanmar	2035	147 (133–162)	0 (0–1)	4174 (3814–4535)	9 (4–15)
Myanmar	2036	147 (133–162)	0 (0–1)	4174 (3814–4535)	9 (4–15)
Namibia	2022	3 (3–3)	0 (0–0)	85 (83–88)	6 (6–6)
Namibia	2023	3 (3–3)	0 (0–0)	86 (81–92)	6 (5–6)
Namibia	2024	3 (3–4)	0 (0–0)	87 (78–97)	6 (5–7)
Namibia	2025	3 (3–4)	0 (0–0)	88 (74–102)	6 (5–7)
Namibia	2026	3 (3–4)	0 (0–0)	89 (70–108)	6 (5–8)
Namibia	2027	3 (2–4)	0 (0–0)	90 (66–115)	7 (5–8)
Namibia	2028	3 (2–4)	0 (0–0)	91 (61–122)	7 (5–8)

Namibia	2029	3 (2–5)	0 (0–0)	92 (56–129)	7 (5–9)
Namibia	2030	3 (2–5)	0 (0–0)	93 (50–136)	7 (5–9)
Namibia	2031	3 (1–5)	0 (0–0)	94 (44–145)	7 (5–9)
Namibia	2032	3 (1–6)	0 (0–0)	95 (38–153)	7 (5–9)
Namibia	2033	3 (1–6)	0 (0–0)	96 (31–162)	7 (5–9)
Namibia	2034	3 (1–6)	0 (0–0)	97 (24–171)	7 (5–9)
Namibia	2035	3 (0–7)	0 (0–0)	98 (17–180)	7 (5–9)
Namibia	2036	3 (-0–7)	0 (0–0)	99 (9–189)	7 (5–9)
Nauru	2022	0 (0–0)	1 (1–1)	2 (2–2)	23 (22–24)
Nauru	2023	0 (0–0)	1 (1–1)	2 (2–2)	23 (21–24)
Nauru	2024	0 (0–0)	1 (1–1)	2 (2–2)	23 (20–25)
Nauru	2025	0 (0–0)	1 (1–1)	2 (1–2)	23 (19–26)
Nauru	2026	0 (0–0)	1 (1–1)	2 (1–2)	23 (19–27)
Nauru	2027	0 (0–0)	1 (0–1)	2 (1–2)	23 (18–28)
Nauru	2028	0 (0–0)	1 (0–1)	2 (1–2)	23 (17–29)
Nauru	2029	0 (0–0)	1 (0–1)	2 (1–2)	23 (16–29)
Nauru	2030	0 (0–0)	1 (0–1)	2 (1–2)	23 (16–30)
Nauru	2031	0 (0–0)	1 (0–1)	2 (1–2)	23 (15–30)
Nauru	2032	0 (0–0)	1 (0–1)	2 (1–2)	23 (15–31)
Nauru	2033	0 (0–0)	1 (0–1)	2 (1–2)	23 (15–31)
Nauru	2034	0 (0–0)	1 (0–1)	2 (1–2)	23 (14–32)
Nauru	2035	0 (0–0)	1 (0–1)	2 (1–2)	23 (14–32)
Nauru	2036	0 (0–0)	1 (0–1)	2 (1–2)	23 (13–32)
Nepal	2022	84 (83–84)	0 (0–0)	2117 (2101–2133)	9 (8–9)
Nepal	2023	86 (85–87)	0 (0–0)	2180 (2145–2215)	9 (8–9)
Nepal	2024	89 (87–91)	0 (0–0)	2243 (2184–2302)	9 (8–9)
Nepal	2025	91 (88–95)	0 (0–0)	2306 (2220–2392)	9 (8–9)
Nepal	2026	94 (90–98)	0 (0–0)	2369 (2252–2486)	9 (8–10)
Nepal	2027	97 (91–102)	0 (0–0)	2432 (2282–2582)	9 (8–10)
Nepal	2028	99 (92–106)	0 (0–0)	2495 (2309–2681)	9 (8–10)
Nepal	2029	102 (93–110)	0 (0–0)	2558 (2333–2783)	9 (8–11)
Nepal	2030	104 (94–114)	0 (0–0)	2621 (2355–2887)	9 (8–11)
Nepal	2031	107 (95–118)	0 (0–0)	2684 (2375–2993)	9 (8–11)

Nepal	2032	110 (96–123)	0 (0–1)	2747 (2393–3101)	10 (7–12)
Nepal	2033	112 (97–127)	0 (0–1)	2810 (2409–3211)	10 (7–12)
Nepal	2034	115 (98–131)	0 (0–1)	2873 (2423–3323)	10 (7–13)
Nepal	2035	117 (99–136)	0 (0–1)	2936 (2435–3437)	10 (7–13)
Nepal	2036	120 (99–140)	0 (0–1)	2999 (2445–3553)	10 (6–14)
Netherlands	2022	116 (112–120)	0 (0–0)	2632 (2560–2704)	8 (7–8)
Netherlands	2023	118 (112–125)	0 (0–0)	2751 (2632–2870)	8 (7–8)
Netherlands	2024	121 (111–130)	0 (0–0)	2823 (2663–2982)	8 (7–9)
Netherlands	2025	123 (111–135)	0 (0–0)	2835 (2648–3023)	8 (6–9)
Netherlands	2026	126 (112–139)	0 (0–0)	2819 (2616–3022)	8 (6–9)
Netherlands	2027	128 (112–144)	0 (0–0)	2824 (2612–3036)	8 (6–9)
Netherlands	2028	130 (113–148)	0 (0–0)	2883 (2664–3103)	8 (6–9)
Netherlands	2029	133 (114–151)	0 (0–0)	2989 (2759–3220)	8 (6–10)
Netherlands	2030	135 (115–155)	0 (0–0)	3101 (2852–3349)	8 (6–10)
Netherlands	2031	137 (116–159)	0 (0–0)	3174 (2905–3442)	8 (5–10)
Netherlands	2032	140 (117–163)	0 (0–0)	3194 (2909–3480)	8 (5–10)
Netherlands	2033	142 (118–166)	0 (0–0)	3187 (2891–3484)	8 (5–10)
Netherlands	2034	145 (119–170)	0 (0–0)	3196 (2893–3499)	8 (5–10)
Netherlands	2035	147 (121–173)	0 (0–0)	3251 (2942–3560)	8 (5–10)
Netherlands	2036	149 (122–176)	0 (0–0)	3348 (3031–3665)	8 (5–10)
New Zealand	2022	38 (36–39)	0 (0–0)	951 (912–990)	12 (12–13)
New Zealand	2023	38 (36–41)	0 (0–0)	971 (924–1018)	13 (12–13)
New Zealand	2024	39 (37–42)	0 (0–1)	991 (937–1045)	13 (12–14)
New Zealand	2025	40 (37–43)	0 (0–1)	1011 (952–1071)	13 (12–14)
New Zealand	2026	41 (37–44)	0 (0–1)	1032 (966–1097)	13 (12–14)
New Zealand	2027	42 (38–45)	0 (0–1)	1052 (981–1122)	13 (12–14)
New Zealand	2028	43 (38–47)	0 (0–1)	1072 (997–1147)	13 (12–15)
New Zealand	2029	43 (39–48)	0 (0–1)	1092 (1012–1172)	13 (12–15)
New Zealand	2030	44 (40–49)	0 (0–1)	1112 (1028–1196)	13 (12–15)
New Zealand	2031	45 (40–50)	1 (0–1)	1132 (1044–1220)	14 (12–15)
New Zealand	2032	46 (41–51)	1 (0–1)	1152 (1060–1244)	14 (12–16)
New Zealand	2033	47 (41–52)	1 (0–1)	1173 (1077–1268)	14 (12–16)
New Zealand	2034	47 (42–53)	1 (0–1)	1193 (1094–1292)	14 (12–16)

New Zealand	2035	48 (42–54)	1 (0–1)	1213 (1110–1315)	14 (12–16)
New Zealand	2036	49 (43–55)	1 (0–1)	1233 (1127–1339)	14 (12–16)
Nicaragua	2022	10 (9–10)	0 (0–0)	261 (249–273)	5 (5–5)
Nicaragua	2023	10 (9–11)	0 (0–0)	267 (250–284)	5 (4–6)
Nicaragua	2024	10 (9–11)	0 (0–0)	274 (249–299)	5 (4–6)
Nicaragua	2025	11 (9–12)	0 (0–0)	280 (247–313)	5 (4–6)
Nicaragua	2026	11 (9–12)	0 (0–0)	287 (245–328)	5 (4–6)
Nicaragua	2027	11 (9–13)	0 (0–0)	293 (242–344)	5 (4–6)
Nicaragua	2028	11 (9–14)	0 (0–0)	300 (239–360)	5 (4–6)
Nicaragua	2029	12 (9–14)	0 (0–0)	306 (235–377)	5 (4–6)
Nicaragua	2030	12 (9–15)	0 (0–0)	312 (231–394)	5 (4–6)
Nicaragua	2031	12 (9–16)	0 (0–0)	319 (226–412)	5 (4–6)
Nicaragua	2032	12 (9–16)	0 (0–0)	325 (220–431)	5 (4–6)
Nicaragua	2033	13 (8–17)	0 (0–0)	332 (214–449)	5 (4–6)
Nicaragua	2034	13 (8–18)	0 (0–0)	338 (208–469)	5 (4–6)
Nicaragua	2035	13 (8–18)	0 (0–0)	345 (201–488)	5 (4–6)
Nicaragua	2036	13 (8–19)	0 (0–0)	351 (194–509)	5 (4–6)
Niger	2022	20 (19–20)	0 (0–0)	603 (579–626)	6 (6–7)
Niger	2023	20 (19–21)	0 (0–0)	603 (569–636)	6 (5–7)
Niger	2024	20 (19–21)	0 (0–0)	603 (561–644)	6 (5–7)
Niger	2025	20 (18–21)	0 (0–0)	603 (555–650)	5 (4–7)
Niger	2026	20 (18–21)	0 (0–0)	603 (549–656)	5 (4–6)
Niger	2027	20 (18–21)	0 (0–0)	603 (544–661)	5 (3–6)
Niger	2028	20 (18–22)	0 (0–0)	603 (540–665)	5 (3–6)
Niger	2029	20 (18–22)	0 (0–0)	603 (535–670)	4 (2–6)
Niger	2030	20 (18–22)	0 (0–0)	603 (531–674)	4 (2–6)
Niger	2031	20 (18–22)	0 (0–0)	603 (527–678)	4 (1–6)
Niger	2032	20 (17–22)	0 (0–0)	603 (524–681)	3 (1–6)
Niger	2033	20 (17–22)	0 (0–0)	603 (520–685)	3 (0–6)
Niger	2034	20 (17–22)	0 (0–0)	603 (517–688)	2 (-0–5)
Niger	2035	20 (17–22)	0 (-0–0)	603 (514–691)	2 (-1–5)
Niger	2036	20 (17–22)	0 (-0–0)	603 (511–695)	2 (-1–5)
Nigeria	2022	65 (64–66)	0 (0–0)	1969 (1929–2009)	2 (2–2)

Nigeria	2023	66 (63–68)	0 (0–0)	1996 (1914–2079)	2 (2–2)
Nigeria	2024	66 (63–70)	0 (0–0)	2014 (1888–2140)	2 (2–2)
Nigeria	2025	67 (62–72)	0 (0–0)	2021 (1854–2187)	2 (1–2)
Nigeria	2026	67 (61–73)	0 (0–0)	2018 (1817–2219)	2 (1–2)
Nigeria	2027	66 (59–73)	0 (0–0)	2007 (1778–2235)	2 (1–2)
Nigeria	2028	66 (58–73)	0 (0–0)	1989 (1741–2237)	2 (1–2)
Nigeria	2029	65 (57–73)	0 (0–0)	1967 (1706–2228)	2 (1–3)
Nigeria	2030	64 (56–73)	0 (0–0)	1943 (1675–2210)	2 (1–3)
Nigeria	2031	64 (56–72)	0 (0–0)	1918 (1648–2188)	2 (1–3)
Nigeria	2032	63 (55–71)	0 (0–0)	1895 (1624–2165)	2 (0–3)
Nigeria	2033	62 (54–70)	0 (0–0)	1874 (1604–2145)	2 (0–3)
Nigeria	2034	62 (54–70)	0 (0–0)	1858 (1587–2130)	2 (-0–3)
Nigeria	2035	61 (53–70)	0 (0–0)	1847 (1573–2121)	2 (-0–3)
Nigeria	2036	61 (53–70)	0 (-0–0)	1840 (1562–2118)	2 (-0–4)
Niue	2022	0 (0–0)	0 (0–0)	0 (0–0)	14 (14–14)
Niue	2023	0 (0–0)	0 (0–1)	0 (0–0)	14 (14–15)
Niue	2024	0 (0–0)	0 (0–1)	0 (0–0)	14 (13–15)
Niue	2025	0 (0–0)	0 (0–1)	0 (0–0)	14 (13–15)
Niue	2026	0 (0–0)	0 (0–1)	0 (0–0)	14 (13–16)
Niue	2027	0 (0–0)	0 (0–1)	0 (0–0)	14 (13–16)
Niue	2028	0 (0–0)	0 (0–1)	0 (0–0)	14 (13–16)
Niue	2029	0 (0–0)	0 (0–1)	0 (0–0)	14 (12–16)
Niue	2030	0 (0–0)	0 (0–1)	0 (0–0)	14 (12–16)
Niue	2031	0 (0–0)	0 (0–1)	0 (0–0)	14 (12–16)
Niue	2032	0 (0–0)	0 (0–1)	0 (0–0)	14 (12–16)
Niue	2033	0 (0–0)	0 (0–1)	0 (0–0)	14 (12–16)
Niue	2034	0 (0–0)	0 (0–1)	0 (0–0)	14 (12–16)
Niue	2035	0 (0–0)	0 (0–1)	0 (0–0)	14 (12–16)
Niue	2036	0 (0–0)	0 (0–1)	0 (0–0)	14 (12–16)
North Macedonia	2022	34 (33–36)	1 (1–1)	910 (876–943)	26 (25–28)
North Macedonia	2023	35 (33–37)	1 (1–1)	917 (863–971)	26 (24–29)
North Macedonia	2024	35 (33–38)	1 (1–1)	924 (856–993)	26 (23–30)
North Macedonia	2025	35 (33–38)	1 (1–1)	931 (851–1012)	26 (22–30)

North Macedonia	2026	36 (32–39)	1 (1–1)	939 (848–1030)	26 (22–31)
North Macedonia	2027	36 (32–40)	1 (1–1)	946 (846–1046)	26 (21–32)
North Macedonia	2028	36 (32–40)	1 (1–1)	953 (844–1062)	26 (20–32)
North Macedonia	2029	37 (32–41)	1 (1–1)	960 (844–1077)	26 (20–33)
North Macedonia	2030	37 (32–42)	1 (1–1)	968 (843–1092)	26 (19–33)
North Macedonia	2031	37 (33–42)	1 (1–1)	975 (844–1106)	26 (19–34)
North Macedonia	2032	38 (33–43)	1 (1–1)	982 (844–1120)	26 (19–34)
North Macedonia	2033	38 (33–44)	1 (1–1)	989 (845–1134)	26 (18–35)
North Macedonia	2034	38 (33–44)	1 (1–1)	997 (846–1147)	26 (18–35)
North Macedonia	2035	39 (33–45)	1 (1–1)	1004 (848–1160)	26 (17–35)
North Macedonia	2036	39 (33–45)	1 (1–1)	1011 (849–1173)	26 (17–36)
Northern Mariana Islands	2022	0 (0–0)	1 (1–1)	13 (12–14)	20 (19–21)
Northern Mariana Islands	2023	0 (0–0)	1 (1–1)	13 (12–14)	20 (18–22)
Northern Mariana Islands	2024	0 (0–0)	1 (1–1)	14 (12–15)	20 (18–22)
Northern Mariana Islands	2025	0 (0–1)	1 (1–1)	14 (12–15)	20 (18–22)
Northern Mariana Islands	2026	0 (0–1)	1 (1–1)	14 (12–16)	20 (17–23)
Northern Mariana Islands	2027	0 (0–1)	1 (1–1)	14 (13–16)	20 (17–23)
Northern Mariana Islands	2028	0 (0–1)	1 (1–1)	15 (13–17)	20 (17–23)
Northern Mariana Islands	2029	0 (0–1)	1 (1–1)	15 (13–17)	20 (17–23)
Northern Mariana Islands	2030	1 (0–1)	1 (1–1)	15 (13–18)	20 (17–24)
Northern Mariana Islands	2031	1 (0–1)	1 (1–1)	16 (13–18)	20 (16–24)
Northern Mariana Islands	2032	1 (0–1)	1 (1–1)	16 (13–18)	20 (16–24)
Northern Mariana Islands	2033	1 (0–1)	1 (1–1)	16 (13–19)	20 (16–24)
Northern Mariana Islands	2034	1 (0–1)	1 (1–1)	16 (14–19)	20 (16–24)
Northern Mariana Islands	2035	1 (0–1)	1 (1–1)	17 (14–20)	20 (16–24)
Northern Mariana Islands	2036	1 (0–1)	1 (1–1)	17 (14–20)	20 (16–25)
Norway	2022	24 (22–26)	0 (0–0)	599 (557–641)	7 (6–7)
Norway	2023	24 (21–27)	0 (0–0)	601 (528–674)	7 (6–7)
Norway	2024	24 (20–28)	0 (0–0)	602 (503–700)	6 (5–7)
Norway	2025	24 (19–29)	0 (0–0)	602 (482–723)	6 (5–7)
Norway	2026	24 (18–30)	0 (0–0)	602 (463–742)	6 (5–7)
Norway	2027	24 (18–31)	0 (0–0)	603 (446–759)	6 (5–7)
Norway	2028	24 (17–32)	0 (0–0)	603 (430–775)	6 (5–7)

Norway	2029	24 (16–32)	0 (0–0)	603 (416–789)	6 (5–7)
Norway	2030	24 (16–33)	0 (0–0)	603 (403–803)	6 (5–7)
Norway	2031	24 (15–34)	0 (0–0)	603 (390–815)	6 (5–7)
Norway	2032	24 (15–34)	0 (0–0)	603 (378–827)	6 (5–7)
Norway	2033	24 (14–35)	0 (0–0)	603 (367–838)	6 (5–7)
Norway	2034	24 (14–35)	0 (0–0)	603 (357–849)	6 (5–7)
Norway	2035	24 (13–35)	0 (0–0)	603 (346–859)	6 (5–7)
Norway	2036	24 (13–36)	0 (0–0)	603 (336–869)	6 (5–7)
Oman	2022	5 (5–5)	0 (0–0)	157 (152–161)	6 (5–6)
Oman	2023	5 (4–5)	0 (0–0)	158 (146–170)	6 (5–6)
Oman	2024	5 (4–6)	0 (0–0)	160 (139–181)	6 (5–7)
Oman	2025	5 (4–6)	0 (0–0)	163 (134–193)	6 (5–7)
Oman	2026	5 (4–6)	0 (0–0)	167 (131–202)	6 (5–7)
Oman	2027	5 (4–6)	0 (0–0)	170 (129–211)	6 (5–7)
Oman	2028	5 (4–7)	0 (0–0)	173 (127–219)	6 (5–8)
Oman	2029	5 (4–7)	0 (0–0)	176 (126–226)	6 (5–8)
Oman	2030	5 (4–7)	0 (0–0)	180 (126–234)	6 (5–8)
Oman	2031	6 (4–7)	0 (0–0)	183 (125–241)	6 (5–8)
Oman	2032	6 (4–7)	0 (0–0)	186 (125–247)	6 (5–8)
Oman	2033	6 (4–8)	0 (0–0)	190 (125–254)	6 (5–8)
Oman	2034	6 (4–8)	0 (0–0)	193 (125–260)	6 (5–8)
Oman	2035	6 (4–8)	0 (0–0)	196 (126–267)	6 (5–8)
Oman	2036	6 (4–8)	0 (0–0)	199 (126–273)	6 (5–8)
Pakistan	2022	367 (362–372)	0 (0–0)	10819 (10662–10977)	8 (7–8)
Pakistan	2023	372 (362–382)	0 (0–0)	10988 (10684–11292)	8 (7–8)
Pakistan	2024	378 (365–391)	0 (0–0)	11156 (10756–11556)	8 (7–8)
Pakistan	2025	383 (367–399)	0 (0–0)	11324 (10847–11801)	8 (7–9)
Pakistan	2026	388 (371–406)	0 (0–0)	11492 (10949–12035)	8 (6–9)
Pakistan	2027	394 (374–414)	0 (0–0)	11660 (11058–12263)	8 (6–10)
Pakistan	2028	399 (378–421)	0 (0–0)	11829 (11173–12485)	8 (5–10)
Pakistan	2029	405 (382–428)	0 (0–0)	11997 (11291–12702)	8 (5–11)
Pakistan	2030	410 (385–435)	0 (0–0)	12165 (11413–12917)	8 (4–11)
Pakistan	2031	415 (389–441)	0 (0–0)	12333 (11538–13129)	8 (4–12)

Pakistan	2032	421 (393–448)	0 (0–0)	12501 (11665–13338)	8 (3–12)
Pakistan	2033	426 (398–455)	0 (0–0)	12670 (11793–13546)	8 (2–13)
Pakistan	2034	432 (402–462)	0 (0–1)	12838 (11924–13752)	8 (2–14)
Pakistan	2035	437 (406–468)	0 (0–1)	13006 (12056–13956)	8 (1–14)
Pakistan	2036	442 (410–475)	0 (0–1)	13174 (12189–14160)	8 (0–15)
Palau	2022	0 (0–0)	1 (1–1)	7 (7–7)	26 (25–26)
Palau	2023	0 (0–0)	1 (1–1)	7 (7–8)	26 (24–28)
Palau	2024	0 (0–0)	1 (1–1)	7 (7–8)	26 (23–29)
Palau	2025	0 (0–0)	1 (1–1)	7 (6–8)	26 (22–31)
Palau	2026	0 (0–0)	1 (1–1)	8 (6–9)	26 (20–33)
Palau	2027	0 (0–0)	1 (1–1)	8 (6–9)	27 (19–35)
Palau	2028	0 (0–0)	1 (1–1)	8 (6–9)	27 (17–37)
Palau	2029	0 (0–0)	1 (0–1)	8 (6–10)	27 (15–39)
Palau	2030	0 (0–0)	1 (0–1)	8 (6–10)	27 (13–42)
Palau	2031	0 (0–0)	1 (0–1)	8 (6–10)	28 (11–44)
Palau	2032	0 (0–0)	1 (0–2)	8 (6–10)	28 (9–47)
Palau	2033	0 (0–0)	1 (0–2)	8 (6–11)	28 (7–50)
Palau	2034	0 (0–0)	1 (0–2)	9 (6–11)	28 (4–53)
Palau	2035	0 (0–0)	1 (0–2)	9 (6–11)	29 (2–56)
Palau	2036	0 (0–0)	1 (-0–2)	9 (6–11)	29 (-1–59)
Palestine	2022	14 (13–14)	1 (1–1)	386 (377–395)	13 (13–14)
Palestine	2023	14 (13–15)	1 (0–1)	401 (381–421)	13 (12–14)
Palestine	2024	14 (13–15)	1 (0–1)	416 (383–450)	13 (12–15)
Palestine	2025	15 (13–16)	1 (0–1)	432 (383–480)	13 (11–15)
Palestine	2026	15 (14–16)	1 (0–1)	447 (381–513)	13 (11–15)
Palestine	2027	15 (14–17)	1 (0–1)	462 (377–547)	13 (11–16)
Palestine	2028	15 (14–17)	1 (0–1)	477 (372–583)	13 (10–16)
Palestine	2029	16 (14–18)	1 (0–1)	493 (365–620)	13 (10–16)
Palestine	2030	16 (14–18)	1 (0–1)	508 (357–659)	13 (10–16)
Palestine	2031	16 (14–18)	1 (0–1)	523 (348–698)	13 (10–17)
Palestine	2032	16 (14–19)	1 (0–1)	538 (338–739)	13 (9–17)
Palestine	2033	17 (14–19)	1 (0–1)	554 (326–781)	13 (9–17)
Palestine	2034	17 (14–19)	1 (0–1)	569 (314–824)	13 (9–17)

Palestine	2035	17 (15–20)	1 (0–1)	584 (300–869)	13 (9–18)
Palestine	2036	17 (15–20)	1 (0–1)	600 (285–914)	13 (9–18)
Panama	2022	8 (8–8)	0 (0–0)	180 (173–187)	4 (4–4)
Panama	2023	8 (8–9)	0 (0–0)	187 (172–203)	4 (3–5)
Panama	2024	9 (8–10)	0 (0–0)	195 (172–217)	4 (3–5)
Panama	2025	9 (8–10)	0 (0–0)	202 (173–232)	4 (3–6)
Panama	2026	9 (8–11)	0 (0–0)	210 (173–246)	4 (3–6)
Panama	2027	10 (8–11)	0 (0–0)	217 (173–261)	4 (2–6)
Panama	2028	10 (8–12)	0 (0–0)	225 (173–276)	4 (2–7)
Panama	2029	10 (8–13)	0 (0–0)	232 (172–292)	4 (2–7)
Panama	2030	11 (8–14)	0 (0–0)	239 (171–307)	4 (1–8)
Panama	2031	11 (8–14)	0 (0–0)	247 (170–323)	5 (1–8)
Panama	2032	11 (8–15)	0 (0–0)	254 (169–340)	5 (1–8)
Panama	2033	12 (8–16)	0 (0–0)	262 (167–356)	5 (1–9)
Panama	2034	12 (8–16)	0 (0–0)	269 (165–373)	5 (0–9)
Panama	2035	12 (8–17)	0 (0–0)	277 (163–390)	5 (-0–10)
Panama	2036	13 (8–18)	0 (-0–0)	284 (160–408)	5 (-1–10)
Papua New Guinea	2022	13 (13–13)	0 (0–0)	434 (426–442)	6 (6–6)
Papua New Guinea	2023	13 (13–14)	0 (0–0)	453 (435–470)	6 (6–7)
Papua New Guinea	2024	14 (13–15)	0 (0–0)	471 (442–500)	6 (6–7)
Papua New Guinea	2025	14 (13–16)	0 (0–0)	490 (447–533)	6 (5–7)
Papua New Guinea	2026	15 (13–16)	0 (0–0)	508 (451–566)	6 (5–7)
Papua New Guinea	2027	15 (14–17)	0 (0–0)	527 (453–602)	6 (5–7)
Papua New Guinea	2028	16 (14–18)	0 (0–0)	546 (453–638)	6 (5–7)
Papua New Guinea	2029	17 (14–20)	0 (0–0)	564 (453–676)	6 (4–7)
Papua New Guinea	2030	17 (14–21)	0 (0–0)	583 (451–715)	6 (4–7)
Papua New Guinea	2031	18 (14–22)	0 (0–0)	602 (449–755)	5 (4–7)
Papua New Guinea	2032	18 (14–23)	0 (0–0)	620 (445–796)	5 (4–7)
Papua New Guinea	2033	19 (13–24)	0 (0–0)	639 (440–838)	5 (4–7)
Papua New Guinea	2034	19 (13–25)	0 (0–0)	658 (434–881)	5 (3–7)
Papua New Guinea	2035	20 (13–26)	0 (0–0)	676 (428–925)	5 (3–7)
Papua New Guinea	2036	20 (13–28)	0 (0–0)	695 (420–970)	5 (3–7)
Paraguay	2022	22 (22–23)	0 (0–0)	562 (546–578)	9 (9–10)

Paraguay	2023	23 (22–24)	0 (0–0)	577 (550–603)	9 (8–10)
Paraguay	2024	24 (22–25)	0 (0–0)	592 (555–629)	9 (8–10)
Paraguay	2025	24 (22–26)	0 (0–0)	607 (558–655)	9 (8–10)
Paraguay	2026	25 (22–27)	0 (0–0)	621 (561–682)	9 (8–10)
Paraguay	2027	25 (22–28)	0 (0–0)	636 (563–709)	9 (8–11)
Paraguay	2028	26 (22–29)	0 (0–0)	651 (565–737)	9 (8–11)
Paraguay	2029	26 (22–31)	0 (0–0)	666 (566–766)	9 (7–11)
Paraguay	2030	27 (22–32)	0 (0–0)	681 (566–795)	9 (7–11)
Paraguay	2031	28 (22–33)	0 (0–0)	695 (566–825)	9 (7–11)
Paraguay	2032	28 (22–34)	0 (0–0)	710 (565–855)	9 (7–11)
Paraguay	2033	29 (22–35)	0 (0–0)	725 (564–886)	9 (7–11)
Paraguay	2034	29 (22–37)	0 (0–0)	740 (562–918)	9 (7–11)
Paraguay	2035	30 (22–38)	0 (0–0)	755 (559–950)	9 (7–12)
Paraguay	2036	31 (22–39)	0 (0–0)	769 (556–983)	9 (7–12)
Peru	2022	43 (41–45)	0 (0–0)	1030 (978–1081)	3 (3–3)
Peru	2023	44 (40–48)	0 (0–0)	1051 (961–1142)	3 (2–3)
Peru	2024	45 (40–50)	0 (0–0)	1073 (950–1196)	3 (2–3)
Peru	2025	46 (40–52)	0 (0–0)	1095 (943–1246)	2 (2–3)
Peru	2026	47 (40–54)	0 (0–0)	1116 (941–1292)	2 (2–3)
Peru	2027	48 (40–56)	0 (0–0)	1138 (940–1336)	2 (2–3)
Peru	2028	49 (40–58)	0 (0–0)	1160 (942–1378)	3 (2–3)
Peru	2029	50 (40–59)	0 (0–0)	1182 (946–1418)	3 (2–3)
Peru	2030	50 (40–61)	0 (0–0)	1204 (951–1456)	3 (2–3)
Peru	2031	51 (40–63)	0 (0–0)	1225 (957–1494)	3 (2–3)
Peru	2032	52 (41–64)	0 (0–0)	1247 (963–1531)	3 (2–3)
Peru	2033	53 (41–66)	0 (0–0)	1269 (971–1567)	3 (2–3)
Peru	2034	54 (41–67)	0 (0–0)	1291 (979–1602)	3 (2–3)
Peru	2035	55 (42–69)	0 (0–0)	1312 (988–1637)	3 (2–3)
Peru	2036	56 (42–70)	0 (0–0)	1334 (997–1671)	3 (2–3)
Philippines	2022	681 (666–697)	1 (1–1)	21022 (20508–21536)	22 (21–23)
Philippines	2023	691 (665–717)	1 (1–1)	21289 (20395–22183)	21 (20–23)
Philippines	2024	700 (667–734)	1 (1–1)	21551 (20336–22767)	21 (19–23)
Philippines	2025	710 (670–750)	1 (1–1)	21812 (20321–23302)	21 (18–23)

Philippines	2026	719 (675–764)	1 (1–1)	22071 (20341–23802)	20 (17–23)
Philippines	2027	729 (679–779)	1 (1–1)	22331 (20386–24275)	20 (16–23)
Philippines	2028	739 (685–793)	1 (1–1)	22590 (20452–24728)	19 (15–23)
Philippines	2029	748 (690–806)	1 (0–1)	22849 (20533–25165)	19 (15–23)
Philippines	2030	758 (696–819)	1 (0–1)	23108 (20626–25589)	19 (14–23)
Philippines	2031	767 (702–832)	1 (0–1)	23367 (20730–26004)	18 (13–23)
Philippines	2032	777 (708–845)	1 (0–1)	23626 (20843–26409)	18 (13–23)
Philippines	2033	786 (715–858)	1 (0–1)	23885 (20963–26808)	17 (12–23)
Philippines	2034	796 (721–871)	1 (0–1)	24144 (21089–27200)	17 (11–23)
Philippines	2035	805 (728–883)	1 (0–1)	24403 (21221–27586)	17 (11–22)
Philippines	2036	815 (734–895)	1 (0–1)	24662 (21357–27968)	16 (10–22)
Poland	2022	144 (135–153)	0 (0–0)	3719 (3468–3971)	6 (5–6)
Poland	2023	148 (129–167)	0 (0–0)	3810 (3285–4334)	6 (5–7)
Poland	2024	151 (126–177)	0 (0–0)	3900 (3202–4598)	6 (5–7)
Poland	2025	155 (125–185)	0 (0–0)	3990 (3154–4826)	6 (5–7)
Poland	2026	159 (124–193)	0 (0–0)	4081 (3126–5035)	6 (5–8)
Poland	2027	162 (124–201)	0 (0–0)	4171 (3111–5230)	6 (5–8)
Poland	2028	166 (124–208)	0 (0–0)	4261 (3106–5417)	6 (4–8)
Poland	2029	169 (124–215)	0 (0–0)	4352 (3108–5595)	6 (4–8)
Poland	2030	173 (125–221)	0 (0–0)	4442 (3116–5768)	7 (4–9)
Poland	2031	177 (126–228)	0 (0–0)	4532 (3128–5936)	7 (4–9)
Poland	2032	180 (126–234)	0 (0–0)	4623 (3145–6100)	7 (4–9)
Poland	2033	184 (127–240)	0 (0–0)	4713 (3166–6260)	7 (4–9)
Poland	2034	187 (129–246)	0 (0–0)	4803 (3189–6418)	7 (4–10)
Poland	2035	191 (130–252)	0 (0–0)	4894 (3215–6572)	7 (4–10)
Poland	2036	195 (131–258)	0 (0–0)	4984 (3243–6725)	7 (4–10)
Portugal	2022	99 (94–103)	0 (0–0)	2605 (2483–2727)	14 (13–14)
Portugal	2023	101 (94–107)	0 (0–1)	2663 (2491–2835)	14 (13–15)
Portugal	2024	103 (95–111)	0 (0–1)	2721 (2511–2932)	14 (13–15)
Portugal	2025	105 (96–114)	0 (0–1)	2779 (2536–3022)	14 (13–16)
Portugal	2026	107 (97–117)	1 (0–1)	2837 (2565–3109)	15 (13–16)
Portugal	2027	110 (99–120)	1 (0–1)	2895 (2597–3193)	15 (13–17)
Portugal	2028	112 (100–124)	1 (0–1)	2953 (2632–3275)	15 (13–17)

Portugal	2029	114 (102–127)	1 (0–1)	3011 (2667–3355)	15 (13–17)
Portugal	2030	116 (103–130)	1 (0–1)	3069 (2705–3434)	16 (13–18)
Portugal	2031	118 (104–133)	1 (0–1)	3127 (2743–3512)	16 (13–18)
Portugal	2032	121 (106–135)	1 (0–1)	3185 (2782–3588)	16 (13–19)
Portugal	2033	123 (108–138)	1 (0–1)	3243 (2822–3664)	16 (14–19)
Portugal	2034	125 (109–141)	1 (0–1)	3301 (2863–3740)	17 (14–19)
Portugal	2035	127 (111–144)	1 (0–1)	3359 (2905–3814)	17 (14–20)
Portugal	2036	130 (112–147)	1 (0–1)	3417 (2947–3888)	17 (14–20)
Puerto Rico	2022	17 (16–19)	0 (0–0)	405 (374–437)	7 (6–7)
Puerto Rico	2023	18 (16–19)	0 (0–0)	411 (366–455)	7 (6–8)
Puerto Rico	2024	18 (16–20)	0 (0–0)	416 (362–471)	7 (6–8)
Puerto Rico	2025	18 (16–21)	0 (0–0)	422 (359–485)	7 (5–8)
Puerto Rico	2026	18 (16–21)	0 (0–0)	427 (357–498)	7 (5–8)
Puerto Rico	2027	19 (16–22)	0 (0–0)	433 (356–510)	7 (5–8)
Puerto Rico	2028	19 (16–22)	0 (0–0)	438 (355–521)	7 (5–8)
Puerto Rico	2029	19 (16–23)	0 (0–0)	444 (355–533)	7 (5–8)
Puerto Rico	2030	20 (16–23)	0 (0–0)	449 (355–544)	7 (5–9)
Puerto Rico	2031	20 (16–24)	0 (0–0)	455 (355–554)	7 (5–9)
Puerto Rico	2032	20 (16–24)	0 (0–0)	460 (356–565)	7 (5–9)
Puerto Rico	2033	20 (16–25)	0 (0–0)	466 (357–575)	7 (4–9)
Puerto Rico	2034	21 (16–25)	0 (0–0)	471 (358–585)	7 (4–9)
Puerto Rico	2035	21 (16–26)	0 (0–0)	477 (359–594)	7 (4–9)
Puerto Rico	2036	21 (16–26)	0 (0–0)	482 (360–604)	7 (4–9)
Qatar	2022	9 (9–9)	1 (1–1)	285 (277–292)	23 (21–25)
Qatar	2023	10 (9–10)	1 (1–1)	305 (288–321)	23 (19–27)
Qatar	2024	11 (10–11)	1 (1–1)	324 (297–351)	23 (18–28)
Qatar	2025	11 (10–13)	1 (1–1)	344 (305–384)	23 (17–29)
Qatar	2026	12 (10–14)	1 (1–1)	364 (310–418)	23 (16–29)
Qatar	2027	13 (11–15)	1 (1–1)	384 (315–453)	23 (16–30)
Qatar	2028	14 (11–16)	1 (1–1)	404 (318–489)	23 (15–31)
Qatar	2029	14 (11–18)	1 (1–1)	424 (320–527)	23 (15–31)
Qatar	2030	15 (11–19)	1 (1–1)	443 (321–566)	23 (14–32)
Qatar	2031	16 (11–21)	1 (1–1)	463 (321–605)	23 (13–32)

Qatar	2032	17 (11–22)	1 (1–1)	483 (320–646)	23 (13–33)
Qatar	2033	17 (11–23)	1 (1–1)	503 (318–688)	23 (13–33)
Qatar	2034	18 (11–25)	1 (1–1)	523 (316–730)	23 (12–34)
Qatar	2035	19 (11–26)	1 (1–1)	543 (312–773)	23 (12–34)
Qatar	2036	20 (11–28)	1 (1–1)	562 (307–818)	23 (11–35)
Republic of Korea	2022	1628 (1587–1669)	2 (2–2)	41420 (40204–42635)	45 (43–48)
Republic of Korea	2023	1628 (1553–1703)	2 (2–2)	40975 (38793–43157)	46 (41–50)
Republic of Korea	2024	1628 (1530–1726)	2 (1–2)	40530 (37693–43366)	46 (39–53)
Republic of Korea	2025	1628 (1512–1744)	2 (1–2)	40084 (36719–43450)	46 (36–57)
Republic of Korea	2026	1628 (1496–1760)	2 (1–2)	39639 (35817–43462)	47 (34–60)
Republic of Korea	2027	1628 (1482–1774)	2 (1–2)	39194 (34964–43424)	47 (31–64)
Republic of Korea	2028	1628 (1470–1787)	2 (1–2)	38749 (34147–43351)	47 (27–68)
Republic of Korea	2029	1628 (1458–1798)	2 (1–3)	38304 (33358–43250)	48 (24–72)
Republic of Korea	2030	1628 (1447–1810)	2 (1–3)	37859 (32592–43126)	48 (20–76)
Republic of Korea	2031	1628 (1436–1820)	2 (1–3)	37414 (31844–42984)	49 (17–81)
Republic of Korea	2032	1628 (1426–1830)	2 (1–3)	36969 (31111–42826)	49 (13–85)
Republic of Korea	2033	1628 (1417–1839)	2 (0–3)	36524 (30392–42655)	49 (8–90)
Republic of Korea	2034	1628 (1408–1848)	2 (0–3)	36079 (29685–42472)	50 (4–95)
Republic of Korea	2035	1628 (1399–1857)	2 (0–4)	35634 (28988–42279)	50 (-1–101)
Republic of Korea	2036	1628 (1391–1865)	2 (0–4)	35189 (28301–42077)	50 (-5–106)
Republic of Moldova	2022	17 (13–22)	0 (0–0)	501 (362–640)	9 (6–12)
Republic of Moldova	2023	17 (10–25)	0 (0–0)	505 (287–722)	9 (5–14)
Republic of Moldova	2024	17 (9–26)	0 (0–0)	506 (250–763)	10 (4–15)
Republic of Moldova	2025	17 (8–26)	0 (0–1)	507 (235–779)	10 (4–15)
Republic of Moldova	2026	17 (8–26)	0 (0–1)	507 (231–782)	10 (4–16)
Republic of Moldova	2027	17 (8–26)	0 (0–1)	506 (230–782)	10 (4–16)
Republic of Moldova	2028	17 (8–26)	0 (0–1)	506 (230–782)	10 (4–16)
Republic of Moldova	2029	17 (8–26)	0 (0–1)	505 (229–782)	10 (4–16)
Republic of Moldova	2030	17 (8–26)	0 (0–1)	505 (229–782)	10 (4–16)
Republic of Moldova	2031	17 (8–26)	0 (0–1)	505 (229–782)	10 (4–16)
Republic of Moldova	2032	17 (8–26)	0 (0–1)	505 (229–782)	10 (4–16)
Republic of Moldova	2033	17 (8–26)	0 (0–1)	505 (229–782)	10 (4–16)
Republic of Moldova	2034	17 (8–26)	0 (0–1)	505 (229–782)	10 (4–16)

Republic of Moldova	2035	17 (8–26)	0 (0–1)	505 (229–782)	10 (4–16)
Republic of Moldova	2036	17 (8–26)	0 (0–1)	505 (229–782)	10 (4–16)
Romania	2022	121 (114–128)	0 (0–0)	3242 (3030–3453)	10 (9–11)
Romania	2023	123 (113–133)	0 (0–0)	3302 (3003–3601)	10 (9–11)
Romania	2024	126 (113–138)	0 (0–0)	3362 (2996–3729)	10 (9–12)
Romania	2025	128 (113–142)	0 (0–0)	3423 (2999–3846)	11 (9–12)
Romania	2026	130 (114–146)	0 (0–0)	3483 (3010–3956)	11 (9–12)
Romania	2027	133 (115–150)	0 (0–0)	3543 (3025–4062)	11 (9–13)
Romania	2028	135 (116–154)	0 (0–0)	3604 (3044–4164)	11 (9–13)
Romania	2029	137 (117–158)	0 (0–0)	3664 (3065–4263)	11 (9–13)
Romania	2030	140 (118–161)	0 (0–0)	3725 (3089–4360)	11 (9–13)
Romania	2031	142 (119–165)	0 (0–0)	3785 (3115–4454)	12 (9–14)
Romania	2032	144 (120–168)	0 (0–0)	3845 (3143–4547)	12 (9–14)
Romania	2033	147 (122–172)	0 (0–0)	3906 (3172–4639)	12 (10–14)
Romania	2034	149 (123–175)	0 (0–1)	3966 (3203–4729)	12 (10–15)
Romania	2035	151 (124–179)	0 (0–1)	4026 (3234–4819)	12 (10–15)
Romania	2036	154 (126–182)	0 (0–1)	4087 (3267–4907)	12 (10–15)
Russian Federation	2022	714 (658–769)	0 (0–0)	20023 (18221–21826)	9 (8–10)
Russian Federation	2023	725 (647–804)	0 (0–0)	20034 (16999–23069)	9 (7–10)
Russian Federation	2024	737 (641–833)	0 (0–0)	20038 (15997–24078)	9 (7–10)
Russian Federation	2025	749 (638–860)	0 (0–0)	20039 (15152–24926)	9 (7–11)
Russian Federation	2026	760 (637–884)	0 (0–0)	20039 (14419–25660)	9 (7–11)
Russian Federation	2027	772 (636–908)	0 (0–0)	20040 (13767–26313)	9 (6–11)
Russian Federation	2028	784 (637–930)	0 (0–0)	20040 (13174–26905)	9 (6–11)
Russian Federation	2029	795 (639–952)	0 (0–0)	20040 (12628–27451)	9 (6–11)
Russian Federation	2030	807 (641–973)	0 (0–0)	20040 (12120–27960)	9 (6–12)
Russian Federation	2031	819 (644–994)	0 (0–0)	20040 (11642–28437)	9 (6–12)
Russian Federation	2032	830 (647–1014)	0 (0–0)	20040 (11190–28889)	9 (6–12)
Russian Federation	2033	842 (650–1034)	0 (0–0)	20040 (10760–29319)	9 (6–12)
Russian Federation	2034	854 (654–1053)	0 (0–0)	20040 (10349–29730)	9 (5–12)
Russian Federation	2035	865 (658–1073)	0 (0–0)	20040 (9955–30124)	9 (5–12)
Russian Federation	2036	877 (663–1092)	0 (0–0)	20040 (9576–30504)	9 (5–12)
Rwanda	2022	26 (25–26)	0 (0–0)	676 (656–695)	10 (9–11)

Rwanda	2023	26 (25–28)	0 (0–0)	678 (628–728)	10 (7–12)
Rwanda	2024	27 (25–30)	0 (0–1)	664 (574–754)	10 (6–13)
Rwanda	2025	28 (24–32)	0 (0–1)	649 (527–771)	10 (6–14)
Rwanda	2026	30 (25–34)	0 (0–1)	644 (504–785)	10 (5–14)
Rwanda	2027	31 (25–36)	0 (0–1)	651 (501–801)	10 (5–15)
Rwanda	2028	32 (25–38)	0 (0–1)	661 (505–816)	10 (4–15)
Rwanda	2029	33 (25–41)	0 (0–1)	665 (503–828)	10 (4–16)
Rwanda	2030	34 (25–43)	0 (0–1)	663 (490–836)	10 (4–16)
Rwanda	2031	35 (24–45)	0 (0–1)	657 (472–843)	10 (3–16)
Rwanda	2032	36 (24–48)	0 (0–1)	653 (457–850)	10 (3–17)
Rwanda	2033	37 (24–50)	0 (0–1)	654 (448–859)	10 (3–17)
Rwanda	2034	38 (24–53)	0 (0–1)	657 (445–869)	10 (2–17)
Rwanda	2035	39 (23–55)	0 (0–1)	660 (441–878)	10 (2–17)
Rwanda	2036	40 (23–58)	0 (0–1)	660 (435–886)	10 (2–18)
Saint Kitts and Nevis	2022	0 (0–0)	0 (0–0)	4 (4–4)	5 (4–6)
Saint Kitts and Nevis	2023	0 (0–0)	0 (0–0)	4 (4–5)	5 (4–6)
Saint Kitts and Nevis	2024	0 (0–0)	0 (0–0)	5 (4–5)	5 (4–6)
Saint Kitts and Nevis	2025	0 (0–0)	0 (0–0)	5 (4–6)	5 (4–6)
Saint Kitts and Nevis	2026	0 (0–0)	0 (0–0)	5 (4–6)	5 (4–6)
Saint Kitts and Nevis	2027	0 (0–0)	0 (0–0)	5 (4–7)	5 (4–6)
Saint Kitts and Nevis	2028	0 (0–0)	0 (0–0)	6 (4–7)	5 (4–7)
Saint Kitts and Nevis	2029	0 (0–0)	0 (0–0)	6 (4–8)	5 (3–7)
Saint Kitts and Nevis	2030	0 (0–0)	0 (0–0)	6 (4–8)	5 (3–7)
Saint Kitts and Nevis	2031	0 (0–0)	0 (0–0)	6 (4–9)	5 (3–7)
Saint Kitts and Nevis	2032	0 (0–0)	0 (0–0)	7 (4–9)	5 (3–7)
Saint Kitts and Nevis	2033	0 (0–0)	0 (0–0)	7 (4–10)	5 (3–7)
Saint Kitts and Nevis	2034	0 (0–0)	0 (0–0)	7 (4–10)	5 (3–7)
Saint Kitts and Nevis	2035	0 (0–0)	0 (0–0)	7 (4–11)	5 (3–7)
Saint Kitts and Nevis	2036	0 (0–0)	0 (0–0)	8 (3–12)	5 (3–7)
Saint Lucia	2022	0 (0–0)	0 (0–0)	8 (7–8)	3 (3–3)
Saint Lucia	2023	0 (0–0)	0 (0–0)	8 (7–9)	3 (3–3)
Saint Lucia	2024	0 (0–0)	0 (0–0)	8 (7–9)	3 (2–4)
Saint Lucia	2025	0 (0–0)	0 (0–0)	8 (7–9)	3 (2–4)

Saint Lucia	2026	0 (0–0)	0 (0–0)	8 (7–9)	3 (2–4)
Saint Lucia	2027	0 (0–0)	0 (0–0)	8 (7–10)	3 (2–4)
Saint Lucia	2028	0 (0–0)	0 (0–0)	9 (7–10)	3 (2–4)
Saint Lucia	2029	0 (0–0)	0 (0–0)	9 (7–10)	3 (2–4)
Saint Lucia	2030	0 (0–0)	0 (0–0)	9 (8–10)	3 (2–4)
Saint Lucia	2031	0 (0–1)	0 (0–0)	9 (8–10)	3 (2–4)
Saint Lucia	2032	0 (0–1)	0 (0–0)	9 (8–10)	3 (1–4)
Saint Lucia	2033	0 (0–1)	0 (0–0)	9 (8–11)	2 (1–4)
Saint Lucia	2034	0 (0–1)	0 (0–0)	9 (8–11)	2 (1–4)
Saint Lucia	2035	0 (0–1)	0 (0–0)	9 (8–11)	2 (1–4)
Saint Lucia	2036	0 (0–1)	0 (0–0)	9 (8–11)	2 (1–4)
Saint Vincent and the Grenadines	2022	0 (0–0)	0 (0–0)	8 (8–9)	6 (5–6)
Saint Vincent and the Grenadines	2023	0 (0–0)	0 (0–0)	9 (8–9)	6 (5–6)
Saint Vincent and the Grenadines	2024	0 (0–0)	0 (0–0)	9 (8–10)	6 (5–7)
Saint Vincent and the Grenadines	2025	0 (0–0)	0 (0–0)	9 (8–10)	6 (5–7)
Saint Vincent and the Grenadines	2026	0 (0–0)	0 (0–0)	9 (8–10)	6 (4–7)
Saint Vincent and the Grenadines	2027	0 (0–0)	0 (0–0)	9 (8–10)	6 (4–7)
Saint Vincent and the Grenadines	2028	0 (0–0)	0 (0–0)	9 (8–11)	6 (4–7)
Saint Vincent and the Grenadines	2029	0 (0–1)	0 (0–0)	9 (8–11)	6 (4–7)
Saint Vincent and the Grenadines	2030	0 (0–1)	0 (0–0)	9 (8–11)	6 (4–7)
Saint Vincent and the Grenadines	2031	0 (0–1)	0 (0–0)	9 (8–11)	6 (4–7)
Saint Vincent and the Grenadines	2032	0 (0–1)	0 (0–0)	10 (8–11)	6 (4–8)
Saint Vincent and the Grenadines	2033	0 (0–1)	0 (0–0)	10 (8–12)	6 (4–8)
Saint Vincent and the Grenadines	2034	0 (0–1)	0 (0–0)	10 (8–12)	6 (4–8)
Saint Vincent and the Grenadines	2035	0 (0–1)	0 (0–0)	10 (8–12)	6 (4–8)
Saint Vincent and the Grenadines	2036	0 (0–1)	0 (0–0)	10 (8–12)	6 (3–8)
Samoa	2022	1 (1–1)	0 (0–1)	23 (22–24)	14 (14–15)
Samoa	2023	1 (1–1)	0 (0–1)	23 (22–25)	14 (13–15)
Samoa	2024	1 (1–1)	0 (0–1)	24 (22–26)	14 (13–16)
Samoa	2025	1 (1–1)	0 (0–1)	24 (21–27)	14 (12–17)
Samoa	2026	1 (1–1)	0 (0–1)	24 (21–28)	14 (12–17)
Samoa	2027	1 (1–1)	0 (0–1)	24 (20–29)	14 (11–18)
Samoa	2028	1 (1–1)	0 (0–1)	24 (19–29)	14 (11–18)

Samoa	2029	1 (1–1)	0 (0–1)	25 (19–30)	14 (10–19)
Samoa	2030	1 (1–1)	0 (0–1)	25 (18–31)	14 (10–19)
Samoa	2031	1 (1–1)	0 (0–1)	25 (18–32)	14 (9–20)
Samoa	2032	1 (1–1)	0 (0–1)	25 (17–32)	15 (9–20)
Samoa	2033	1 (1–1)	0 (0–1)	25 (17–33)	15 (8–21)
Samoa	2034	1 (1–1)	0 (0–1)	25 (16–33)	15 (8–21)
Samoa	2035	1 (1–1)	0 (0–1)	25 (16–34)	15 (7–22)
Samoa	2036	1 (1–1)	0 (0–1)	25 (15–34)	15 (7–22)
San Marino	2022	0 (0–0)	0 (0–0)	4 (4–4)	6 (6–6)
San Marino	2023	0 (0–0)	0 (0–0)	4 (4–4)	6 (6–6)
San Marino	2024	0 (0–0)	0 (0–0)	4 (4–4)	6 (5–6)
San Marino	2025	0 (0–0)	0 (0–0)	4 (4–4)	6 (5–6)
San Marino	2026	0 (0–0)	0 (0–0)	4 (4–4)	6 (5–6)
San Marino	2027	0 (0–0)	0 (0–0)	4 (4–4)	6 (5–6)
San Marino	2028	0 (0–0)	0 (0–0)	4 (4–4)	6 (5–6)
San Marino	2029	0 (0–0)	0 (0–0)	4 (4–4)	6 (5–6)
San Marino	2030	0 (0–0)	0 (0–0)	4 (4–4)	6 (5–6)
San Marino	2031	0 (0–0)	0 (0–0)	4 (4–4)	6 (5–6)
San Marino	2032	0 (0–0)	0 (0–0)	4 (4–4)	6 (5–6)
San Marino	2033	0 (0–0)	0 (0–0)	4 (4–5)	6 (5–6)
San Marino	2034	0 (0–0)	0 (0–0)	4 (4–5)	6 (5–6)
San Marino	2035	0 (0–0)	0 (0–0)	4 (4–5)	6 (5–6)
San Marino	2036	0 (0–0)	0 (0–0)	4 (4–5)	6 (5–6)
Sao Tome and Principe	2022	0 (0–0)	0 (0–0)	4 (3–4)	3 (2–3)
Sao Tome and Principe	2023	0 (0–0)	0 (0–0)	4 (3–4)	3 (2–3)
Sao Tome and Principe	2024	0 (0–0)	0 (0–0)	4 (3–4)	2 (2–3)
Sao Tome and Principe	2025	0 (0–0)	0 (0–0)	4 (3–4)	2 (2–3)
Sao Tome and Principe	2026	0 (0–0)	0 (0–0)	4 (3–4)	2 (1–3)
Sao Tome and Principe	2027	0 (0–0)	0 (0–0)	4 (3–4)	2 (1–3)
Sao Tome and Principe	2028	0 (0–0)	0 (0–0)	4 (3–5)	2 (1–3)
Sao Tome and Principe	2029	0 (0–0)	0 (0–0)	4 (3–5)	2 (1–3)
Sao Tome and Principe	2030	0 (0–0)	0 (0–0)	4 (3–5)	2 (1–4)
Sao Tome and Principe	2031	0 (0–0)	0 (0–0)	4 (3–5)	2 (0–4)

Sao Tome and Principe	2032	0 (0–0)	0 (-0–0)	4 (3–5)	2 (-0–4)
Sao Tome and Principe	2033	0 (0–0)	0 (-0–0)	4 (3–5)	2 (-0–4)
Sao Tome and Principe	2034	0 (0–0)	0 (-0–0)	4 (3–5)	2 (-1–4)
Sao Tome and Principe	2035	0 (0–0)	0 (-0–0)	4 (3–5)	2 (-1–4)
Sao Tome and Principe	2036	0 (0–0)	0 (-0–0)	4 (3–5)	2 (-1–4)
Saudi Arabia	2022	72 (71–73)	0 (0–0)	2218 (2183–2253)	9 (9–9)
Saudi Arabia	2023	75 (72–78)	0 (0–0)	2320 (2232–2409)	9 (8–9)
Saudi Arabia	2024	78 (74–83)	0 (0–0)	2422 (2264–2580)	9 (8–10)
Saudi Arabia	2025	82 (75–89)	0 (0–0)	2524 (2283–2765)	9 (7–10)
Saudi Arabia	2026	85 (75–95)	0 (0–0)	2626 (2291–2962)	9 (7–11)
Saudi Arabia	2027	89 (76–101)	0 (0–0)	2728 (2288–3169)	9 (6–11)
Saudi Arabia	2028	92 (76–108)	0 (0–0)	2831 (2276–3385)	9 (6–11)
Saudi Arabia	2029	95 (76–115)	0 (0–0)	2933 (2256–3610)	9 (6–12)
Saudi Arabia	2030	99 (75–122)	0 (0–0)	3035 (2227–3842)	9 (6–12)
Saudi Arabia	2031	102 (75–129)	0 (0–0)	3137 (2192–4082)	9 (5–12)
Saudi Arabia	2032	105 (74–137)	0 (0–0)	3239 (2149–4329)	9 (5–13)
Saudi Arabia	2033	109 (73–144)	0 (0–0)	3341 (2099–4583)	9 (5–13)
Saudi Arabia	2034	112 (72–152)	0 (0–0)	3443 (2043–4843)	9 (5–13)
Saudi Arabia	2035	115 (70–160)	0 (0–0)	3545 (1981–5110)	9 (5–13)
Saudi Arabia	2036	119 (69–169)	0 (0–0)	3648 (1913–5382)	9 (5–13)
Senegal	2022	32 (29–34)	0 (0–0)	1037 (953–1121)	11 (9–12)
Senegal	2023	32 (28–35)	0 (0–0)	1037 (918–1155)	10 (8–12)
Senegal	2024	32 (28–36)	0 (0–0)	1037 (892–1182)	9 (7–12)
Senegal	2025	32 (27–36)	0 (0–0)	1037 (869–1204)	9 (6–12)
Senegal	2026	32 (27–37)	0 (0–0)	1037 (849–1224)	8 (5–12)
Senegal	2027	32 (26–38)	0 (0–0)	1037 (831–1242)	7 (4–11)
Senegal	2028	32 (26–38)	0 (0–0)	1037 (815–1259)	7 (3–11)
Senegal	2029	32 (25–38)	0 (0–0)	1037 (800–1274)	6 (2–11)
Senegal	2030	32 (25–39)	0 (0–0)	1037 (785–1288)	6 (1–10)
Senegal	2031	32 (24–39)	0 (0–0)	1037 (772–1302)	5 (0–10)
Senegal	2032	32 (24–40)	0 (0–0)	1037 (759–1315)	4 (-1–9)
Senegal	2033	32 (24–40)	0 (-0–0)	1037 (746–1327)	4 (-2–9)
Senegal	2034	32 (23–40)	0 (-0–0)	1037 (735–1339)	3 (-3–9)

Senegal	2035	32 (23–41)	0 (-0–0)	1037 (723–1350)	2 (-3–8)
Senegal	2036	32 (23–41)	0 (-0–0)	1037 (712–1361)	2 (-4–8)
Serbia	2022	100 (95–105)	1 (1–1)	2544 (2412–2677)	17 (16–18)
Serbia	2023	100 (91–109)	1 (1–1)	2546 (2322–2771)	17 (15–18)
Serbia	2024	100 (88–111)	1 (1–1)	2548 (2253–2843)	17 (14–19)
Serbia	2025	99 (85–114)	1 (1–1)	2549 (2202–2897)	16 (14–19)
Serbia	2026	99 (83–115)	1 (0–1)	2551 (2165–2937)	16 (13–20)
Serbia	2027	99 (81–116)	1 (0–1)	2552 (2137–2966)	16 (12–21)
Serbia	2028	98 (79–117)	1 (0–1)	2553 (2117–2988)	16 (11–21)
Serbia	2029	98 (78–118)	1 (0–1)	2554 (2103–3005)	16 (10–22)
Serbia	2030	98 (77–118)	1 (0–1)	2554 (2092–3017)	16 (10–23)
Serbia	2031	97 (76–118)	1 (0–1)	2555 (2084–3026)	16 (9–24)
Serbia	2032	97 (75–119)	1 (0–1)	2556 (2078–3034)	16 (8–25)
Serbia	2033	97 (75–119)	1 (0–1)	2556 (2073–3039)	16 (7–25)
Serbia	2034	97 (74–119)	1 (0–1)	2557 (2070–3043)	16 (6–26)
Serbia	2035	96 (74–119)	1 (0–1)	2557 (2068–3046)	16 (5–27)
Serbia	2036	96 (74–119)	1 (0–1)	2557 (2066–3049)	16 (4–28)
Seychelles	2022	1 (1–1)	1 (1–1)	19 (19–20)	15 (14–15)
Seychelles	2023	1 (1–1)	1 (0–1)	20 (19–21)	15 (13–16)
Seychelles	2024	1 (1–1)	1 (0–1)	21 (19–22)	15 (13–17)
Seychelles	2025	1 (1–1)	1 (0–1)	21 (19–24)	15 (12–18)
Seychelles	2026	1 (1–1)	1 (0–1)	22 (19–25)	15 (11–19)
Seychelles	2027	1 (1–1)	1 (0–1)	23 (19–27)	15 (10–21)
Seychelles	2028	1 (1–1)	1 (0–1)	24 (19–28)	15 (8–22)
Seychelles	2029	1 (1–1)	1 (0–1)	24 (18–30)	15 (7–24)
Seychelles	2030	1 (1–1)	1 (0–1)	25 (18–32)	16 (6–25)
Seychelles	2031	1 (0–1)	1 (0–1)	26 (18–34)	16 (4–27)
Seychelles	2032	1 (0–1)	1 (0–1)	26 (17–36)	16 (3–29)
Seychelles	2033	1 (0–1)	1 (-0–1)	27 (17–37)	16 (1–31)
Seychelles	2034	1 (0–1)	1 (-0–1)	28 (16–39)	16 (-1–33)
Seychelles	2035	1 (0–1)	1 (-0–1)	29 (16–41)	16 (-2–35)
Seychelles	2036	1 (0–1)	1 (-0–1)	29 (15–44)	16 (-4–37)
Sierra Leone	2022	19 (19–20)	0 (0–0)	614 (595–632)	13 (12–14)

Sierra Leone	2023	19 (18–20)	0 (0–0)	617 (584–649)	13 (11–14)
Sierra Leone	2024	19 (17–20)	0 (0–0)	611 (562–660)	12 (10–14)
Sierra Leone	2025	18 (16–20)	0 (0–0)	596 (530–662)	11 (8–13)
Sierra Leone	2026	18 (15–20)	0 (0–0)	573 (491–655)	9 (6–13)
Sierra Leone	2027	17 (14–19)	0 (0–0)	544 (448–641)	8 (3–12)
Sierra Leone	2028	16 (12–19)	0 (0–0)	514 (407–621)	6 (1–11)
Sierra Leone	2029	15 (11–18)	0 (-0–0)	487 (373–601)	4 (-1–10)
Sierra Leone	2030	14 (10–18)	0 (-0–0)	466 (348–584)	3 (-2–9)
Sierra Leone	2031	14 (10–17)	0 (-0–0)	453 (332–573)	2 (-4–8)
Sierra Leone	2032	13 (10–17)	0 (-0–0)	447 (326–569)	1 (-5–7)
Sierra Leone	2033	13 (10–17)	0 (-0–0)	448 (325–571)	0 (-6–6)
Sierra Leone	2034	13 (10–17)	-0 (-0–0)	451 (327–576)	-0 (-6–6)
Sierra Leone	2035	13 (10–17)	-0 (-0–0)	453 (327–580)	-1 (-7–5)
Sierra Leone	2036	13 (9–17)	-0 (-0–0)	451 (321–581)	-2 (-8–4)
Singapore	2022	33 (31–35)	0 (0–0)	827 (768–885)	9 (7–10)
Singapore	2023	33 (30–36)	0 (0–0)	836 (753–918)	8 (6–10)
Singapore	2024	34 (30–37)	0 (0–0)	845 (744–946)	8 (6–10)
Singapore	2025	34 (30–38)	0 (0–0)	854 (738–970)	8 (5–10)
Singapore	2026	34 (30–39)	0 (0–0)	863 (733–993)	7 (4–10)
Singapore	2027	35 (30–40)	0 (0–0)	872 (730–1015)	7 (4–10)
Singapore	2028	35 (30–41)	0 (0–0)	882 (728–1035)	6 (3–10)
Singapore	2029	36 (30–42)	0 (0–0)	891 (726–1055)	6 (2–10)
Singapore	2030	36 (30–43)	0 (0–0)	900 (725–1074)	6 (2–9)
Singapore	2031	37 (30–43)	0 (0–0)	909 (725–1093)	5 (1–9)
Singapore	2032	37 (30–44)	0 (0–0)	918 (725–1111)	5 (0–9)
Singapore	2033	38 (31–45)	0 (0–0)	927 (726–1129)	4 (-0–9)
Singapore	2034	38 (31–46)	0 (0–0)	936 (727–1146)	4 (-1–9)
Singapore	2035	39 (31–46)	0 (0–0)	946 (728–1163)	4 (-1–8)
Singapore	2036	39 (31–47)	0 (-0–0)	955 (729–1180)	3 (-2–8)
Slovakia	2022	41 (39–42)	0 (0–0)	1062 (1017–1107)	11 (11–12)
Slovakia	2023	40 (38–43)	0 (0–0)	1062 (998–1126)	11 (10–12)
Slovakia	2024	40 (38–43)	0 (0–0)	1062 (984–1140)	11 (10–12)
Slovakia	2025	40 (37–43)	0 (0–0)	1062 (972–1152)	11 (9–12)

Slovakia	2026	40 (37–43)	0 (0–0)	1062 (961–1163)	10 (9–12)
Slovakia	2027	40 (36–43)	0 (0–0)	1062 (951–1172)	10 (9–12)
Slovakia	2028	40 (36–43)	0 (0–0)	1062 (943–1181)	10 (8–12)
Slovakia	2029	40 (36–43)	0 (0–0)	1062 (934–1189)	10 (8–11)
Slovakia	2030	39 (36–43)	0 (0–0)	1062 (927–1197)	9 (8–11)
Slovakia	2031	39 (35–43)	0 (0–0)	1062 (919–1204)	9 (7–11)
Slovakia	2032	39 (35–43)	0 (0–0)	1062 (912–1211)	9 (7–11)
Slovakia	2033	39 (35–43)	0 (0–0)	1062 (906–1218)	9 (7–11)
Slovakia	2034	39 (35–43)	0 (0–0)	1062 (899–1224)	9 (6–11)
Slovakia	2035	39 (35–43)	0 (0–0)	1062 (893–1230)	8 (6–11)
Slovakia	2036	39 (35–43)	0 (0–0)	1062 (887–1236)	8 (6–11)
Slovenia	2022	19 (18–21)	0 (0–0)	454 (417–492)	11 (10–13)
Slovenia	2023	20 (18–22)	0 (0–0)	454 (401–508)	11 (10–13)
Slovenia	2024	20 (18–22)	0 (0–0)	454 (389–520)	11 (9–13)
Slovenia	2025	20 (18–22)	0 (0–0)	454 (379–530)	11 (9–14)
Slovenia	2026	21 (18–23)	0 (0–0)	454 (370–539)	11 (9–14)
Slovenia	2027	21 (19–23)	0 (0–0)	454 (362–547)	11 (8–14)
Slovenia	2028	21 (19–24)	0 (0–0)	454 (355–554)	11 (8–14)
Slovenia	2029	21 (19–24)	0 (0–0)	454 (348–561)	11 (8–15)
Slovenia	2030	22 (19–24)	0 (0–0)	454 (341–568)	11 (8–15)
Slovenia	2031	22 (19–25)	0 (0–0)	454 (335–574)	11 (8–15)
Slovenia	2032	22 (19–25)	0 (0–0)	454 (329–579)	11 (7–15)
Slovenia	2033	22 (20–25)	0 (0–0)	454 (324–585)	11 (7–15)
Slovenia	2034	23 (20–26)	0 (0–0)	454 (319–590)	11 (7–16)
Slovenia	2035	23 (20–26)	0 (0–0)	454 (313–595)	11 (7–16)
Slovenia	2036	23 (20–26)	0 (0–0)	454 (309–600)	11 (7–16)
Solomon Islands	2022	3 (3–3)	1 (1–1)	90 (87–92)	20 (19–21)
Solomon Islands	2023	3 (3–3)	1 (1–1)	91 (87–95)	19 (18–21)
Solomon Islands	2024	3 (3–3)	1 (1–1)	93 (87–98)	19 (17–21)
Solomon Islands	2025	3 (3–3)	1 (1–1)	94 (88–100)	19 (16–21)
Solomon Islands	2026	3 (3–3)	1 (0–1)	95 (88–102)	19 (16–22)
Solomon Islands	2027	3 (3–3)	1 (0–1)	97 (89–105)	18 (15–22)
Solomon Islands	2028	3 (3–3)	1 (0–1)	98 (89–107)	18 (15–22)

Solomon Islands	2029	3 (3–3)	1 (0–1)	99 (90–109)	18 (14–22)
Solomon Islands	2030	3 (3–3)	1 (0–1)	101 (91–111)	18 (14–22)
Solomon Islands	2031	3 (3–3)	1 (0–1)	102 (92–113)	18 (13–22)
Solomon Islands	2032	3 (3–3)	1 (0–1)	104 (93–115)	17 (13–22)
Solomon Islands	2033	3 (3–3)	1 (0–1)	105 (93–117)	17 (12–22)
Solomon Islands	2034	3 (3–3)	1 (0–1)	106 (94–118)	17 (12–22)
Solomon Islands	2035	3 (3–3)	1 (0–1)	108 (95–120)	17 (11–22)
Solomon Islands	2036	3 (3–4)	1 (0–1)	109 (96–122)	16 (11–22)
Somalia	2022	25 (24–25)	0 (0–0)	799 (788–809)	10 (10–10)
Somalia	2023	25 (25–26)	0 (0–0)	817 (796–838)	10 (10–11)
Somalia	2024	26 (25–27)	0 (0–0)	834 (802–866)	10 (10–11)
Somalia	2025	26 (25–27)	0 (0–0)	849 (806–892)	10 (9–11)
Somalia	2026	26 (25–28)	0 (0–0)	864 (810–917)	10 (9–12)
Somalia	2027	27 (25–28)	0 (0–0)	877 (814–941)	10 (9–12)
Somalia	2028	27 (25–29)	0 (0–0)	891 (818–964)	10 (9–12)
Somalia	2029	28 (26–30)	0 (0–0)	903 (821–986)	10 (8–13)
Somalia	2030	28 (26–30)	0 (0–0)	916 (825–1007)	10 (8–13)
Somalia	2031	28 (26–31)	0 (0–0)	928 (829–1027)	11 (8–13)
Somalia	2032	29 (26–31)	0 (0–1)	940 (834–1047)	11 (8–13)
Somalia	2033	29 (26–32)	0 (0–1)	952 (838–1066)	11 (7–14)
Somalia	2034	29 (26–32)	0 (0–1)	964 (843–1085)	11 (7–14)
Somalia	2035	30 (27–33)	0 (0–1)	976 (848–1104)	11 (7–14)
Somalia	2036	30 (27–33)	0 (0–1)	988 (853–1122)	11 (7–15)
South Africa	2022	237 (225–249)	0 (0–1)	7558 (7154–7962)	14 (13–15)
South Africa	2023	245 (218–272)	0 (0–1)	7811 (7037–8585)	14 (12–17)
South Africa	2024	252 (207–298)	0 (0–1)	8063 (6863–9264)	14 (11–18)
South Africa	2025	260 (193–326)	1 (0–1)	8316 (6637–9995)	15 (9–20)
South Africa	2026	267 (177–357)	1 (0–1)	8569 (6363–10775)	15 (8–22)
South Africa	2027	274 (159–390)	1 (0–1)	8822 (6044–11599)	15 (6–24)
South Africa	2028	282 (138–425)	1 (0–1)	9074 (5683–12465)	15 (4–26)
South Africa	2029	289 (116–462)	1 (-0–1)	9327 (5284–13370)	15 (2–29)
South Africa	2030	297 (92–501)	1 (-0–1)	9580 (4848–14311)	16 (0–31)
South Africa	2031	304 (66–542)	1 (-0–1)	9832 (4377–15288)	16 (-2–34)

South Africa	2032	311 (38–584)	1 (-0–1)	10085 (3873–16297)	16 (-4–36)
South Africa	2033	319 (9–628)	1 (-0–2)	10338 (3336–17339)	16 (-7–39)
South Africa	2034	326 (-21–673)	1 (-1–2)	10590 (2769–18412)	16 (-9–42)
South Africa	2035	334 (-53–720)	1 (-1–2)	10843 (2173–19514)	17 (-12–45)
South Africa	2036	341 (-86–768)	1 (-1–2)	11096 (1547–20644)	17 (-15–48)
South Sudan	2022	13 (13–13)	0 (0–0)	391 (381–402)	8 (8–9)
South Sudan	2023	13 (13–14)	0 (0–0)	402 (381–422)	8 (8–9)
South Sudan	2024	14 (13–15)	0 (0–0)	412 (383–441)	8 (8–9)
South Sudan	2025	14 (13–15)	0 (0–0)	422 (384–461)	8 (8–9)
South Sudan	2026	14 (13–16)	0 (0–0)	433 (385–481)	8 (8–9)
South Sudan	2027	15 (13–17)	0 (0–0)	443 (385–501)	8 (8–9)
South Sudan	2028	15 (13–17)	0 (0–0)	454 (385–522)	8 (7–9)
South Sudan	2029	15 (13–18)	0 (0–0)	464 (385–543)	8 (7–9)
South Sudan	2030	16 (13–19)	0 (0–0)	474 (384–565)	8 (7–10)
South Sudan	2031	16 (12–19)	0 (0–0)	485 (383–587)	8 (7–10)
South Sudan	2032	16 (12–20)	0 (0–0)	495 (381–609)	8 (7–10)
South Sudan	2033	17 (12–21)	0 (0–0)	506 (379–632)	8 (7–10)
South Sudan	2034	17 (12–22)	0 (0–0)	516 (377–655)	8 (7–10)
South Sudan	2035	17 (12–22)	0 (0–0)	526 (374–679)	8 (7–10)
South Sudan	2036	18 (12–23)	0 (0–0)	537 (370–703)	8 (7–10)
Spain	2022	520 (502–538)	1 (1–1)	12586 (12106–13066)	15 (14–16)
Spain	2023	519 (487–551)	1 (1–1)	12528 (11739–13316)	15 (14–17)
Spain	2024	518 (472–565)	1 (1–1)	12469 (11366–13572)	15 (13–17)
Spain	2025	517 (454–579)	1 (0–1)	12411 (10977–13845)	15 (13–18)
Spain	2026	516 (436–596)	1 (0–1)	12353 (10568–14138)	15 (13–18)
Spain	2027	515 (417–613)	1 (0–1)	12294 (10139–14450)	15 (13–18)
Spain	2028	514 (396–632)	1 (0–1)	12236 (9691–14781)	15 (13–18)
Spain	2029	513 (374–651)	1 (0–1)	12178 (9224–15132)	16 (12–19)
Spain	2030	512 (351–672)	1 (0–1)	12120 (8739–15501)	16 (12–19)
Spain	2031	511 (328–694)	1 (0–1)	12061 (8235–15887)	16 (12–19)
Spain	2032	509 (303–716)	1 (0–1)	12003 (7715–16291)	16 (12–19)
Spain	2033	508 (277–740)	1 (0–1)	11945 (7178–16712)	16 (12–19)
Spain	2034	507 (250–764)	1 (0–1)	11887 (6625–17148)	16 (12–19)

Spain	2035	506 (223–790)	1 (0–1)	11828 (6056–17600)	16 (12–19)
Spain	2036	505 (194–816)	1 (0–1)	11770 (5472–18068)	16 (12–19)
Sri Lanka	2022	35 (33–37)	0 (0–0)	896 (842–950)	3 (3–4)
Sri Lanka	2023	35 (32–38)	0 (0–0)	896 (824–968)	3 (2–4)
Sri Lanka	2024	35 (31–39)	0 (0–0)	887 (797–977)	3 (2–4)
Sri Lanka	2025	35 (30–40)	0 (0–0)	868 (758–977)	3 (2–4)
Sri Lanka	2026	35 (30–40)	0 (0–0)	854 (736–973)	3 (2–4)
Sri Lanka	2027	35 (29–41)	0 (0–0)	837 (714–961)	3 (2–4)
Sri Lanka	2028	35 (29–41)	0 (0–0)	822 (697–948)	3 (1–4)
Sri Lanka	2029	35 (29–42)	0 (0–0)	813 (687–938)	2 (1–4)
Sri Lanka	2030	35 (28–42)	0 (0–0)	807 (680–933)	2 (1–4)
Sri Lanka	2031	35 (28–42)	0 (0–0)	806 (677–935)	2 (1–4)
Sri Lanka	2032	35 (27–43)	0 (0–0)	810 (676–945)	2 (1–3)
Sri Lanka	2033	35 (27–43)	0 (0–0)	818 (677–958)	2 (1–3)
Sri Lanka	2034	35 (27–43)	0 (0–0)	828 (683–974)	2 (1–3)
Sri Lanka	2035	35 (26–44)	0 (0–0)	840 (690–990)	2 (0–3)
Sri Lanka	2036	35 (26–44)	0 (0–0)	851 (700–1003)	2 (0–3)
Sudan	2022	42 (41–42)	0 (0–0)	1200 (1186–1214)	5 (5–5)
Sudan	2023	43 (42–45)	0 (0–0)	1244 (1213–1275)	5 (5–6)
Sudan	2024	45 (43–47)	0 (0–0)	1288 (1236–1339)	5 (5–6)
Sudan	2025	46 (44–49)	0 (0–0)	1331 (1256–1407)	6 (5–6)
Sudan	2026	48 (44–52)	0 (0–0)	1375 (1272–1478)	6 (5–6)
Sudan	2027	50 (45–54)	0 (0–0)	1419 (1287–1551)	6 (5–6)
Sudan	2028	51 (45–57)	0 (0–0)	1463 (1299–1626)	6 (5–7)
Sudan	2029	53 (46–59)	0 (0–0)	1506 (1309–1704)	6 (5–7)
Sudan	2030	54 (46–62)	0 (0–0)	1550 (1316–1784)	6 (5–7)
Sudan	2031	56 (46–65)	0 (0–0)	1594 (1322–1865)	6 (4–8)
Sudan	2032	57 (46–68)	0 (0–0)	1638 (1326–1949)	6 (4–8)
Sudan	2033	59 (46–71)	0 (0–0)	1681 (1329–2034)	6 (4–8)
Sudan	2034	60 (46–74)	0 (0–0)	1725 (1329–2121)	6 (4–8)
Sudan	2035	62 (46–77)	0 (0–0)	1769 (1328–2210)	6 (4–9)
Sudan	2036	63 (46–80)	0 (0–0)	1813 (1325–2300)	6 (3–9)
Suriname	2022	2 (2–2)	0 (0–0)	50 (48–51)	7 (7–8)

Suriname	2023	2 (2–2)	0 (0–0)	51 (48–54)	7 (6–8)
Suriname	2024	2 (2–2)	0 (0–0)	52 (48–56)	7 (6–8)
Suriname	2025	2 (2–2)	0 (0–0)	53 (48–58)	7 (6–8)
Suriname	2026	2 (2–2)	0 (0–0)	54 (48–59)	7 (6–8)
Suriname	2027	2 (2–2)	0 (0–0)	55 (49–61)	7 (6–8)
Suriname	2028	2 (2–2)	0 (0–0)	56 (49–62)	7 (6–8)
Suriname	2029	2 (2–2)	0 (0–0)	57 (50–64)	7 (6–8)
Suriname	2030	2 (2–2)	0 (0–0)	58 (50–65)	7 (6–8)
Suriname	2031	2 (2–2)	0 (0–0)	59 (51–67)	7 (6–8)
Suriname	2032	2 (2–2)	0 (0–0)	60 (51–68)	7 (6–8)
Suriname	2033	2 (2–2)	0 (0–0)	61 (52–70)	7 (6–8)
Suriname	2034	2 (2–3)	0 (0–0)	62 (53–71)	7 (6–8)
Suriname	2035	2 (2–3)	0 (0–0)	63 (53–72)	7 (6–8)
Suriname	2036	2 (2–3)	0 (0–0)	64 (54–74)	7 (6–8)
Sweden	2022	63 (57–70)	0 (0–0)	1366 (1214–1518)	7 (6–8)
Sweden	2023	63 (51–76)	0 (0–0)	1366 (1089–1643)	7 (5–9)
Sweden	2024	64 (46–81)	0 (0–0)	1366 (1005–1727)	7 (5–9)
Sweden	2025	64 (42–85)	0 (0–0)	1366 (938–1794)	7 (4–9)
Sweden	2026	64 (38–89)	0 (0–0)	1366 (879–1853)	7 (4–9)
Sweden	2027	64 (35–93)	0 (0–0)	1366 (827–1905)	7 (4–9)
Sweden	2028	64 (31–96)	0 (0–0)	1366 (780–1952)	7 (4–9)
Sweden	2029	64 (29–99)	0 (0–0)	1366 (736–1996)	7 (4–9)
Sweden	2030	64 (26–101)	0 (0–0)	1366 (695–2037)	7 (4–9)
Sweden	2031	64 (23–104)	0 (0–0)	1366 (656–2076)	7 (4–9)
Sweden	2032	64 (21–106)	0 (0–0)	1366 (620–2112)	7 (4–9)
Sweden	2033	64 (19–109)	0 (0–0)	1366 (585–2147)	7 (4–9)
Sweden	2034	64 (16–111)	0 (0–0)	1366 (551–2181)	7 (4–9)
Sweden	2035	64 (14–113)	0 (0–0)	1366 (519–2213)	7 (4–9)
Sweden	2036	64 (12–115)	0 (0–0)	1366 (488–2244)	7 (4–9)
Switzerland	2022	79 (76–83)	0 (0–0)	1710 (1620–1799)	10 (10–11)
Switzerland	2023	80 (75–85)	0 (0–0)	1710 (1583–1836)	10 (9–11)
Switzerland	2024	81 (75–87)	0 (0–0)	1710 (1555–1864)	10 (9–11)
Switzerland	2025	81 (74–88)	0 (0–0)	1710 (1531–1888)	10 (8–11)

Switzerland	2026	82 (74–90)	0 (0–1)	1710 (1510–1909)	10 (8–11)
Switzerland	2027	83 (74–91)	0 (0–1)	1710 (1491–1928)	10 (8–11)
Switzerland	2028	84 (74–93)	0 (0–1)	1710 (1473–1946)	10 (8–12)
Switzerland	2029	84 (74–94)	0 (0–1)	1710 (1457–1962)	9 (7–12)
Switzerland	2030	85 (75–96)	0 (0–1)	1710 (1442–1977)	9 (7–12)
Switzerland	2031	86 (75–97)	0 (0–1)	1710 (1427–1992)	9 (7–12)
Switzerland	2032	87 (75–98)	0 (0–1)	1710 (1413–2006)	9 (7–12)
Switzerland	2033	87 (75–99)	0 (0–1)	1710 (1400–2019)	9 (6–12)
Switzerland	2034	88 (75–101)	0 (0–1)	1710 (1388–2031)	9 (6–11)
Switzerland	2035	89 (76–102)	0 (0–1)	1710 (1376–2043)	9 (6–11)
Switzerland	2036	90 (76–103)	0 (0–1)	1710 (1364–2055)	9 (6–11)
Syrian Arab Republic	2022	63 (61–66)	0 (0–0)	1764 (1705–1824)	12 (11–12)
Syrian Arab Republic	2023	64 (60–69)	0 (0–1)	1789 (1670–1908)	12 (10–13)
Syrian Arab Republic	2024	65 (59–72)	0 (0–1)	1807 (1628–1987)	12 (10–14)
Syrian Arab Republic	2025	66 (57–74)	0 (0–1)	1821 (1582–2059)	12 (9–14)
Syrian Arab Republic	2026	66 (55–77)	0 (0–1)	1830 (1536–2125)	12 (8–15)
Syrian Arab Republic	2027	66 (54–79)	0 (0–1)	1837 (1489–2185)	12 (8–16)
Syrian Arab Republic	2028	67 (52–81)	0 (0–1)	1842 (1444–2241)	12 (7–16)
Syrian Arab Republic	2029	67 (50–83)	0 (0–1)	1846 (1400–2292)	12 (7–17)
Syrian Arab Republic	2030	67 (49–85)	0 (0–1)	1849 (1357–2340)	12 (6–17)
Syrian Arab Republic	2031	67 (47–86)	0 (0–1)	1851 (1317–2384)	12 (6–18)
Syrian Arab Republic	2032	67 (46–88)	0 (0–1)	1852 (1278–2426)	12 (5–18)
Syrian Arab Republic	2033	67 (45–89)	0 (0–1)	1853 (1241–2465)	12 (5–18)
Syrian Arab Republic	2034	67 (43–91)	0 (0–1)	1854 (1206–2502)	12 (5–19)
Syrian Arab Republic	2035	67 (42–92)	0 (0–1)	1854 (1172–2537)	12 (4–19)
Syrian Arab Republic	2036	67 (41–93)	0 (0–1)	1855 (1139–2570)	12 (4–19)
Taiwan (Province of China)	2022	388 (325–451)	1 (1–1)	10716 (8870–12563)	28 (21–35)
Taiwan (Province of China)	2023	388 (299–477)	1 (1–1)	10716 (8105–13328)	29 (20–38)
Taiwan (Province of China)	2024	388 (279–497)	1 (1–1)	10716 (7518–13915)	29 (19–39)
Taiwan (Province of China)	2025	388 (262–514)	1 (1–1)	10716 (7023–14409)	30 (19–41)
Taiwan (Province of China)	2026	388 (247–529)	1 (1–1)	10716 (6587–14845)	30 (19–42)
Taiwan (Province of China)	2027	388 (233–542)	1 (1–1)	10716 (6193–15239)	31 (19–42)
Taiwan (Province of China)	2028	388 (221–554)	1 (1–1)	10716 (5831–15602)	31 (19–43)

Taiwan (Province of China)	2029	388 (210–566)	1 (1–1)	10716 (5493–15939)	31 (19–43)
Taiwan (Province of China)	2030	388 (199–577)	1 (1–1)	10716 (5177–16256)	32 (20–44)
Taiwan (Province of China)	2031	388 (189–587)	1 (1–1)	10716 (4877–16556)	32 (20–44)
Taiwan (Province of China)	2032	388 (179–597)	1 (1–2)	10716 (4592–16841)	32 (20–44)
Taiwan (Province of China)	2033	388 (169–606)	1 (1–2)	10716 (4320–17113)	32 (20–44)
Taiwan (Province of China)	2034	388 (161–615)	1 (1–2)	10716 (4058–17374)	32 (20–45)
Taiwan (Province of China)	2035	388 (152–623)	1 (1–2)	10716 (3807–17625)	32 (20–45)
Taiwan (Province of China)	2036	388 (144–632)	1 (1–2)	10716 (3565–17868)	32 (20–45)
Tajikistan	2022	14 (13–14)	0 (0–0)	419 (403–434)	6 (5–6)
Tajikistan	2023	14 (13–15)	0 (0–0)	429 (401–457)	6 (5–7)
Tajikistan	2024	14 (13–16)	0 (0–0)	441 (396–486)	6 (4–7)
Tajikistan	2025	14 (12–17)	0 (0–0)	452 (388–515)	5 (4–7)
Tajikistan	2026	15 (12–18)	0 (0–0)	463 (379–547)	5 (4–7)
Tajikistan	2027	15 (11–19)	0 (0–0)	474 (367–581)	5 (3–7)
Tajikistan	2028	15 (11–20)	0 (0–0)	485 (354–616)	5 (3–7)
Tajikistan	2029	15 (10–21)	0 (0–0)	496 (339–653)	5 (3–7)
Tajikistan	2030	16 (9–22)	0 (0–0)	507 (323–692)	5 (2–7)
Tajikistan	2031	16 (8–23)	0 (0–0)	518 (305–732)	5 (2–7)
Tajikistan	2032	16 (8–24)	0 (0–0)	530 (286–773)	4 (2–7)
Tajikistan	2033	16 (7–26)	0 (0–0)	541 (266–816)	4 (2–7)
Tajikistan	2034	17 (6–27)	0 (0–0)	552 (244–860)	4 (1–7)
Tajikistan	2035	17 (5–29)	0 (0–0)	563 (221–905)	4 (1–7)
Tajikistan	2036	17 (4–30)	0 (0–0)	574 (197–951)	4 (1–7)
Thailand	2022	1475 (1408–1542)	1 (1–1)	40843 (38692–42995)	36 (33–40)
Thailand	2023	1494 (1400–1589)	1 (1–1)	41289 (38246–44332)	35 (30–41)
Thailand	2024	1513 (1397–1629)	1 (1–1)	41735 (38008–45462)	35 (28–41)
Thailand	2025	1532 (1398–1666)	1 (1–1)	42181 (37878–46484)	34 (26–41)
Thailand	2026	1551 (1402–1701)	1 (1–1)	42627 (37815–47438)	33 (24–41)
Thailand	2027	1570 (1406–1735)	1 (1–1)	43073 (37802–48343)	32 (23–41)
Thailand	2028	1589 (1412–1767)	1 (1–1)	43519 (37826–49211)	31 (21–41)
Thailand	2029	1609 (1419–1798)	1 (1–1)	43964 (37878–50050)	30 (19–41)
Thailand	2030	1628 (1426–1829)	1 (1–1)	44410 (37955–50865)	29 (18–40)
Thailand	2031	1647 (1435–1859)	1 (1–1)	44856 (38052–51660)	28 (16–40)

Thailand	2032	1666 (1443–1888)	1 (1–1)	45302 (38165–52438)	27 (15–40)
Thailand	2033	1685 (1452–1917)	1 (0–1)	45748 (38294–53202)	26 (13–39)
Thailand	2034	1704 (1462–1945)	1 (0–1)	46194 (38436–53952)	25 (12–39)
Thailand	2035	1723 (1472–1974)	1 (0–1)	46639 (38588–54690)	25 (11–39)
Thailand	2036	1742 (1482–2001)	1 (0–1)	47085 (38752–55419)	24 (9–38)
Timor-Leste	2022	3 (3–3)	0 (0–0)	73 (72–75)	8 (8–8)
Timor-Leste	2023	3 (3–3)	0 (0–0)	76 (73–78)	8 (8–8)
Timor-Leste	2024	3 (3–3)	0 (0–0)	78 (74–82)	8 (8–9)
Timor-Leste	2025	3 (3–3)	0 (0–0)	80 (75–86)	8 (7–9)
Timor-Leste	2026	3 (3–3)	0 (0–0)	83 (75–90)	8 (7–9)
Timor-Leste	2027	3 (3–3)	0 (0–0)	85 (75–94)	8 (7–10)
Timor-Leste	2028	3 (3–3)	0 (0–0)	87 (76–99)	8 (7–10)
Timor-Leste	2029	3 (3–3)	0 (0–0)	90 (76–103)	9 (7–11)
Timor-Leste	2030	3 (3–4)	0 (0–0)	92 (76–108)	9 (6–11)
Timor-Leste	2031	3 (3–4)	0 (0–0)	94 (75–113)	9 (6–12)
Timor-Leste	2032	3 (3–4)	0 (0–0)	97 (75–118)	9 (6–12)
Timor-Leste	2033	3 (3–4)	0 (0–0)	99 (74–123)	9 (5–13)
Timor-Leste	2034	3 (3–4)	0 (0–0)	101 (74–129)	9 (5–13)
Timor-Leste	2035	4 (3–4)	0 (0–0)	104 (73–134)	9 (5–14)
Timor-Leste	2036	4 (3–5)	0 (0–0)	106 (72–139)	9 (4–14)
Togo	2022	15 (15–15)	0 (0–0)	500 (488–512)	10 (10–10)
Togo	2023	15 (15–16)	0 (0–0)	511 (488–534)	10 (9–11)
Togo	2024	16 (15–17)	0 (0–0)	521 (487–555)	10 (9–11)
Togo	2025	16 (15–17)	0 (0–0)	530 (485–575)	10 (8–12)
Togo	2026	16 (15–18)	0 (0–0)	539 (484–593)	10 (7–12)
Togo	2027	17 (15–18)	0 (0–0)	547 (484–610)	10 (7–13)
Togo	2028	17 (15–19)	0 (0–0)	555 (483–627)	10 (6–13)
Togo	2029	17 (15–19)	0 (0–0)	563 (484–643)	10 (6–14)
Togo	2030	17 (15–20)	0 (0–0)	571 (484–658)	10 (5–15)
Togo	2031	18 (15–20)	0 (0–1)	579 (485–672)	10 (5–15)
Togo	2032	18 (15–21)	0 (0–1)	587 (487–686)	10 (4–16)
Togo	2033	18 (15–21)	0 (0–1)	594 (488–700)	10 (3–16)
Togo	2034	18 (15–21)	0 (0–1)	602 (490–714)	10 (3–17)

Togo	2035	18 (15–22)	0 (0–1)	610 (493–727)	10 (2–17)
Togo	2036	19 (15–22)	0 (0–1)	618 (495–740)	10 (2–18)
Tokelau	2022	0 (0–0)	1 (1–1)	0 (0–0)	15 (15–15)
Tokelau	2023	0 (0–0)	1 (0–1)	0 (0–0)	15 (14–16)
Tokelau	2024	0 (0–0)	1 (0–1)	0 (0–0)	15 (14–17)
Tokelau	2025	0 (0–0)	1 (0–1)	0 (0–0)	16 (14–17)
Tokelau	2026	0 (0–0)	1 (0–1)	0 (0–0)	16 (14–18)
Tokelau	2027	0 (0–0)	1 (0–1)	0 (0–0)	16 (14–18)
Tokelau	2028	0 (0–0)	1 (0–1)	0 (0–0)	16 (14–18)
Tokelau	2029	0 (0–0)	1 (0–1)	0 (0–0)	16 (14–19)
Tokelau	2030	0 (0–0)	1 (0–1)	0 (0–0)	16 (13–19)
Tokelau	2031	0 (0–0)	1 (0–1)	0 (0–0)	16 (13–19)
Tokelau	2032	0 (0–0)	1 (0–1)	0 (0–0)	17 (13–20)
Tokelau	2033	0 (0–0)	1 (0–1)	0 (0–0)	17 (13–20)
Tokelau	2034	0 (0–0)	1 (0–1)	0 (0–1)	17 (13–20)
Tokelau	2035	0 (0–0)	1 (0–1)	0 (0–1)	17 (13–21)
Tokelau	2036	0 (0–0)	1 (0–1)	0 (0–1)	17 (13–21)
Tonga	2022	2 (2–2)	3 (3–3)	69 (66–73)	81 (76–86)
Tonga	2023	2 (2–3)	3 (2–3)	69 (62–75)	79 (70–88)
Tonga	2024	2 (2–3)	3 (2–3)	69 (59–78)	78 (64–91)
Tonga	2025	2 (2–3)	3 (2–3)	69 (58–79)	77 (60–94)
Tonga	2026	2 (2–3)	3 (2–3)	68 (56–81)	76 (54–98)
Tonga	2027	2 (2–3)	3 (2–4)	68 (56–81)	75 (47–102)
Tonga	2028	2 (2–3)	3 (1–4)	68 (55–82)	73 (40–106)
Tonga	2029	2 (2–3)	3 (1–4)	68 (54–82)	72 (33–111)
Tonga	2030	2 (2–3)	2 (1–4)	68 (54–82)	71 (26–116)
Tonga	2031	2 (2–3)	2 (1–4)	68 (54–83)	70 (19–121)
Tonga	2032	2 (2–3)	2 (0–5)	68 (53–83)	69 (11–127)
Tonga	2033	2 (2–3)	2 (-0–5)	68 (53–83)	68 (2–133)
Tonga	2034	2 (2–3)	2 (-0–5)	68 (53–83)	67 (-6–139)
Tonga	2035	2 (2–3)	2 (-1–5)	68 (53–83)	65 (-15–146)
Tonga	2036	2 (2–3)	2 (-1–5)	68 (53–83)	64 (-24–152)
Trinidad and Tobago	2022	3 (3–4)	0 (0–0)	91 (84–98)	5 (4–5)

Trinidad and Tobago	2023	3 (3–4)	0 (0–0)	92 (83–102)	5 (4–5)
Trinidad and Tobago	2024	3 (3–4)	0 (0–0)	94 (82–106)	5 (4–5)
Trinidad and Tobago	2025	4 (3–4)	0 (0–0)	95 (82–109)	5 (3–6)
Trinidad and Tobago	2026	4 (3–4)	0 (0–0)	97 (81–112)	5 (3–6)
Trinidad and Tobago	2027	4 (3–4)	0 (0–0)	98 (81–115)	5 (3–6)
Trinidad and Tobago	2028	4 (3–4)	0 (0–0)	99 (81–117)	5 (3–6)
Trinidad and Tobago	2029	4 (3–4)	0 (0–0)	101 (81–120)	5 (3–6)
Trinidad and Tobago	2030	4 (3–5)	0 (0–0)	102 (81–123)	5 (3–6)
Trinidad and Tobago	2031	4 (3–5)	0 (0–0)	103 (82–125)	5 (3–6)
Trinidad and Tobago	2032	4 (3–5)	0 (0–0)	105 (82–127)	5 (3–6)
Trinidad and Tobago	2033	4 (3–5)	0 (0–0)	106 (82–130)	5 (3–6)
Trinidad and Tobago	2034	4 (3–5)	0 (0–0)	107 (83–132)	5 (3–7)
Trinidad and Tobago	2035	4 (3–5)	0 (0–0)	109 (83–134)	5 (3–7)
Trinidad and Tobago	2036	4 (3–5)	0 (0–0)	110 (84–137)	5 (2–7)
Tunisia	2022	32 (32–32)	0 (0–0)	844 (836–852)	6 (6–6)
Tunisia	2023	33 (32–34)	0 (0–0)	870 (854–886)	6 (6–6)
Tunisia	2024	34 (33–35)	0 (0–0)	897 (871–922)	6 (6–6)
Tunisia	2025	35 (34–36)	0 (0–0)	923 (886–960)	6 (6–6)
Tunisia	2026	36 (34–38)	0 (0–0)	949 (900–998)	6 (6–6)
Tunisia	2027	37 (35–39)	0 (0–0)	975 (913–1038)	6 (6–6)
Tunisia	2028	38 (35–41)	0 (0–0)	1002 (925–1078)	6 (6–7)
Tunisia	2029	39 (36–42)	0 (0–0)	1028 (936–1120)	6 (6–7)
Tunisia	2030	40 (36–44)	0 (0–0)	1054 (946–1163)	6 (6–7)
Tunisia	2031	41 (36–45)	0 (0–0)	1080 (955–1206)	6 (6–7)
Tunisia	2032	42 (37–47)	0 (0–0)	1107 (963–1250)	6 (6–7)
Tunisia	2033	43 (37–49)	0 (0–0)	1133 (971–1295)	6 (6–7)
Tunisia	2034	44 (37–50)	0 (0–0)	1159 (978–1341)	6 (6–7)
Tunisia	2035	45 (38–52)	0 (0–0)	1185 (984–1387)	6 (6–7)
Tunisia	2036	46 (38–54)	0 (0–0)	1212 (989–1434)	6 (6–7)
Turkey	2022	318 (312–324)	0 (0–0)	8650 (8486–8815)	9 (8–9)
Turkey	2023	325 (313–338)	0 (0–0)	8811 (8444–9178)	9 (8–9)
Turkey	2024	333 (314–352)	0 (0–0)	8972 (8357–9586)	9 (8–9)
Turkey	2025	340 (313–368)	0 (0–0)	9132 (8233–10031)	8 (7–10)

Turkey	2026	348 (312–384)	0 (0–0)	9293 (8076–10510)	8 (7–10)
Turkey	2027	355 (310–401)	0 (0–0)	9454 (7888–11019)	8 (6–11)
Turkey	2028	363 (307–418)	0 (0–0)	9614 (7672–11556)	8 (6–11)
Turkey	2029	370 (304–436)	0 (0–0)	9775 (7430–12119)	8 (5–12)
Turkey	2030	378 (300–455)	0 (0–0)	9936 (7164–12707)	8 (4–12)
Turkey	2031	385 (296–475)	0 (0–0)	10096 (6875–13317)	8 (4–13)
Turkey	2032	393 (291–494)	0 (0–1)	10257 (6565–13949)	8 (3–13)
Turkey	2033	400 (285–515)	0 (0–1)	10417 (6233–14602)	8 (2–14)
Turkey	2034	407 (279–536)	0 (0–1)	10578 (5881–15276)	8 (2–15)
Turkey	2035	415 (272–557)	0 (0–1)	10739 (5509–15968)	8 (1–15)
Turkey	2036	422 (265–579)	0 (-0–1)	10899 (5119–16679)	8 (0–16)
Turkmenistan	2022	15 (13–16)	0 (0–0)	473 (428–518)	10 (8–12)
Turkmenistan	2023	15 (13–17)	0 (0–0)	473 (409–537)	10 (7–12)
Turkmenistan	2024	15 (12–17)	0 (0–0)	473 (395–551)	10 (7–13)
Turkmenistan	2025	15 (12–18)	0 (0–0)	473 (382–563)	10 (6–14)
Turkmenistan	2026	15 (12–18)	0 (0–0)	473 (372–574)	10 (6–14)
Turkmenistan	2027	15 (11–18)	0 (0–0)	473 (362–583)	10 (6–14)
Turkmenistan	2028	15 (11–19)	0 (0–0)	473 (353–592)	10 (5–15)
Turkmenistan	2029	15 (11–19)	0 (0–1)	473 (345–600)	10 (5–15)
Turkmenistan	2030	15 (11–19)	0 (0–1)	473 (337–608)	10 (5–15)
Turkmenistan	2031	15 (10–19)	0 (0–1)	473 (330–616)	10 (4–16)
Turkmenistan	2032	15 (10–20)	0 (0–1)	473 (323–623)	10 (4–16)
Turkmenistan	2033	15 (10–20)	0 (0–1)	473 (316–629)	10 (4–16)
Turkmenistan	2034	15 (10–20)	0 (0–1)	473 (310–636)	10 (4–16)
Turkmenistan	2035	15 (9–20)	0 (0–1)	473 (304–642)	10 (3–17)
Turkmenistan	2036	15 (9–21)	0 (0–1)	473 (298–648)	10 (3–17)
Tuvalu	2022	0 (0–0)	1 (1–1)	2 (2–2)	16 (16–16)
Tuvalu	2023	0 (0–0)	1 (1–1)	2 (2–2)	16 (15–17)
Tuvalu	2024	0 (0–0)	1 (0–1)	2 (2–2)	16 (15–17)
Tuvalu	2025	0 (0–0)	1 (0–1)	2 (2–2)	16 (14–18)
Tuvalu	2026	0 (0–0)	1 (0–1)	2 (2–2)	16 (14–18)
Tuvalu	2027	0 (0–0)	1 (0–1)	2 (2–2)	16 (14–18)
Tuvalu	2028	0 (0–0)	1 (0–1)	2 (2–2)	16 (14–18)

Tuvalu	2029	0 (0–0)	1 (0–1)	2 (2–2)	16 (14–18)
Tuvalu	2030	0 (0–0)	1 (0–1)	2 (2–2)	16 (13–19)
Tuvalu	2031	0 (0–0)	1 (0–1)	2 (2–2)	16 (13–19)
Tuvalu	2032	0 (0–0)	1 (0–1)	2 (2–2)	16 (13–19)
Tuvalu	2033	0 (0–0)	1 (0–1)	2 (2–2)	16 (13–19)
Tuvalu	2034	0 (0–0)	1 (0–1)	2 (2–2)	16 (13–19)
Tuvalu	2035	0 (0–0)	1 (0–1)	2 (2–2)	16 (13–19)
Tuvalu	2036	0 (0–0)	1 (0–1)	2 (2–2)	16 (13–19)
Uganda	2022	47 (46–48)	0 (0–0)	1405 (1373–1436)	8 (8–8)
Uganda	2023	48 (46–50)	0 (0–0)	1451 (1389–1513)	8 (7–9)
Uganda	2024	50 (46–53)	0 (0–0)	1498 (1398–1597)	8 (7–9)
Uganda	2025	51 (46–56)	0 (0–0)	1544 (1402–1686)	8 (6–10)
Uganda	2026	53 (45–60)	0 (0–0)	1590 (1401–1780)	8 (6–10)
Uganda	2027	54 (45–63)	0 (0–0)	1637 (1395–1878)	8 (5–11)
Uganda	2028	56 (44–67)	0 (0–0)	1683 (1386–1980)	8 (5–12)
Uganda	2029	57 (43–71)	0 (0–0)	1729 (1373–2086)	8 (4–13)
Uganda	2030	59 (42–75)	0 (0–0)	1776 (1356–2195)	8 (3–14)
Uganda	2031	60 (41–79)	0 (0–1)	1822 (1336–2308)	8 (2–14)
Uganda	2032	62 (39–84)	0 (0–1)	1868 (1313–2424)	8 (1–15)
Uganda	2033	63 (38–88)	0 (0–1)	1915 (1286–2543)	8 (1–16)
Uganda	2034	64 (36–93)	0 (0–1)	1961 (1257–2665)	9 (-0–17)
Uganda	2035	66 (34–98)	0 (0–1)	2007 (1225–2789)	9 (-1–18)
Uganda	2036	67 (33–102)	0 (-0–1)	2054 (1191–2916)	9 (-2–20)
Ukraine	2022	110 (79–141)	0 (0–0)	3218 (2298–4139)	5 (3–6)
Ukraine	2023	110 (67–153)	0 (0–0)	3233 (1938–4528)	5 (3–7)
Ukraine	2024	110 (48–172)	0 (0–0)	3235 (1378–5091)	5 (2–7)
Ukraine	2025	110 (30–190)	0 (0–0)	3242 (822–5662)	5 (1–8)
Ukraine	2026	110 (9–211)	0 (0–0)	3247 (192–6302)	5 (0–9)
Ukraine	2027	110 (-13–233)	0 (-0–0)	3253 (-474–6981)	5 (-1–10)
Ukraine	2028	111 (-36–257)	0 (-0–0)	3259 (-1189–7707)	5 (-2–11)
Ukraine	2029	111 (-61–282)	0 (-0–0)	3265 (-1942–8472)	5 (-3–12)
Ukraine	2030	111 (-87–308)	0 (-0–0)	3271 (-2735–9276)	5 (-4–14)
Ukraine	2031	111 (-114–336)	0 (-0–0)	3276 (-3565–10117)	5 (-6–15)

Ukraine	2032	111 (-142–364)	0 (-0–1)	3282 (-4430–10994)	5 (-7–16)
Ukraine	2033	111 (-172–394)	0 (-0–1)	3288 (-5329–11905)	5 (-8–18)
Ukraine	2034	111 (-202–425)	0 (-0–1)	3294 (-6261–12849)	5 (-10–19)
Ukraine	2035	111 (-234–456)	0 (-0–1)	3299 (-7225–13824)	5 (-11–21)
Ukraine	2036	111 (-267–489)	0 (-0–1)	3305 (-8220–14830)	5 (-13–22)
United Arab Emirates	2022	37 (36–38)	1 (1–1)	1232 (1204–1259)	19 (18–20)
United Arab Emirates	2023	40 (38–43)	1 (1–1)	1333 (1250–1416)	19 (17–21)
United Arab Emirates	2024	44 (40–48)	1 (1–1)	1435 (1281–1589)	20 (16–23)
United Arab Emirates	2025	47 (41–54)	1 (1–1)	1536 (1299–1773)	20 (15–25)
United Arab Emirates	2026	51 (42–60)	1 (0–1)	1638 (1307–1969)	20 (13–27)
United Arab Emirates	2027	54 (42–67)	1 (0–1)	1739 (1305–2174)	21 (12–30)
United Arab Emirates	2028	58 (43–73)	1 (0–1)	1841 (1294–2388)	21 (10–32)
United Arab Emirates	2029	62 (43–80)	1 (0–1)	1943 (1274–2611)	21 (8–35)
United Arab Emirates	2030	65 (43–87)	1 (0–2)	2044 (1247–2841)	22 (6–37)
United Arab Emirates	2031	69 (43–94)	1 (-0–2)	2146 (1213–3078)	22 (4–40)
United Arab Emirates	2032	72 (42–102)	1 (-0–2)	2247 (1172–3323)	22 (1–43)
United Arab Emirates	2033	76 (42–110)	1 (-0–2)	2349 (1124–3574)	23 (-1–47)
United Arab Emirates	2034	79 (41–117)	1 (-0–2)	2450 (1069–3831)	23 (-4–50)
United Arab Emirates	2035	83 (40–125)	1 (-1–2)	2552 (1009–4095)	23 (-6–53)
United Arab Emirates	2036	86 (39–134)	1 (-1–2)	2654 (943–4364)	24 (-9–57)
United Kingdom	2022	802 (785–819)	1 (1–1)	16845 (16499–17192)	14 (14–15)
United Kingdom	2023	821 (786–856)	1 (1–1)	17048 (16317–17779)	14 (14–15)
United Kingdom	2024	840 (788–892)	1 (1–1)	17349 (16276–18423)	15 (14–16)
United Kingdom	2025	859 (790–928)	1 (1–1)	17564 (16022–19107)	15 (14–16)
United Kingdom	2026	878 (791–964)	1 (1–1)	17805 (15758–19853)	15 (14–17)
United Kingdom	2027	897 (791–1002)	1 (1–1)	18072 (15498–20645)	15 (14–17)
United Kingdom	2028	915 (791–1040)	1 (1–1)	18298 (15131–21466)	15 (14–17)
United Kingdom	2029	934 (789–1080)	1 (1–1)	18549 (14761–22336)	16 (14–18)
United Kingdom	2030	953 (786–1120)	1 (1–1)	18799 (14358–23240)	16 (14–18)
United Kingdom	2031	972 (783–1161)	1 (1–1)	19036 (13898–24173)	16 (14–18)
United Kingdom	2032	991 (779–1203)	1 (1–1)	19286 (13426–25145)	16 (14–19)
United Kingdom	2033	1010 (774–1246)	1 (1–1)	19531 (12916–26146)	17 (14–19)
United Kingdom	2034	1029 (768–1290)	1 (1–1)	19773 (12371–27175)	17 (14–19)

United Kingdom	2035	1048 (761–1334)	1 (1–1)	20021 (11806–28235)	17 (14–20)
United Kingdom	2036	1067 (754–1379)	1 (1–1)	20265 (11207–29322)	17 (14–20)
United Republic of Tanzania	2022	102 (101–103)	0 (0–0)	2996 (2962–3030)	10 (10–10)
United Republic of Tanzania	2023	105 (102–108)	0 (0–0)	3100 (3012–3187)	10 (10–11)
United Republic of Tanzania	2024	108 (103–113)	0 (0–0)	3198 (3042–3355)	10 (9–11)
United Republic of Tanzania	2025	111 (103–119)	0 (0–0)	3292 (3055–3528)	10 (9–12)
United Republic of Tanzania	2026	114 (103–124)	0 (0–0)	3380 (3054–3706)	10 (9–12)
United Republic of Tanzania	2027	116 (102–130)	0 (0–0)	3463 (3041–3884)	11 (8–13)
United Republic of Tanzania	2028	119 (101–136)	0 (0–1)	3541 (3018–4064)	11 (8–14)
United Republic of Tanzania	2029	121 (100–142)	0 (0–1)	3614 (2985–4243)	11 (7–14)
United Republic of Tanzania	2030	123 (99–147)	0 (0–1)	3683 (2946–4420)	11 (7–15)
United Republic of Tanzania	2031	125 (97–153)	0 (0–1)	3748 (2899–4596)	11 (6–15)
United Republic of Tanzania	2032	127 (95–159)	0 (0–1)	3809 (2847–4770)	11 (6–16)
United Republic of Tanzania	2033	129 (93–164)	0 (0–1)	3866 (2790–4942)	11 (5–17)
United Republic of Tanzania	2034	130 (91–170)	0 (0–1)	3920 (2729–5112)	11 (5–17)
United Republic of Tanzania	2035	132 (89–175)	0 (0–1)	3971 (2663–5279)	11 (4–18)
United Republic of Tanzania	2036	133 (86–180)	0 (0–1)	4019 (2595–5443)	11 (4–18)
United States of America	2022	2674 (2633–2714)	0 (0–0)	65090 (63971–66210)	12 (11–12)
United States of America	2023	2733 (2656–2809)	0 (0–0)	66482 (64459–68506)	12 (11–12)
United States of America	2024	2792 (2692–2892)	0 (0–0)	67874 (65240–70507)	12 (11–12)
United States of America	2025	2851 (2732–2970)	0 (0–0)	69265 (66138–72392)	12 (11–13)
United States of America	2026	2910 (2774–3045)	0 (0–1)	70657 (67104–74209)	12 (10–13)
United States of America	2027	2968 (2818–3119)	0 (0–1)	72048 (68116–75980)	12 (10–13)
United States of America	2028	3027 (2864–3191)	0 (0–1)	73440 (69162–77718)	11 (10–13)
United States of America	2029	3086 (2910–3262)	0 (0–1)	74831 (70233–79429)	11 (10–13)
United States of America	2030	3145 (2958–3332)	0 (0–1)	76223 (71325–81120)	11 (9–14)
United States of America	2031	3204 (3006–3402)	0 (0–1)	77614 (72435–82794)	11 (9–14)
United States of America	2032	3263 (3055–3471)	0 (0–1)	79006 (73559–84453)	11 (9–14)
United States of America	2033	3322 (3104–3540)	0 (0–1)	80398 (74696–86099)	11 (8–14)
United States of America	2034	3381 (3153–3608)	0 (0–1)	81789 (75843–87735)	11 (8–15)
United States of America	2035	3440 (3203–3676)	0 (0–1)	83181 (77001–89361)	11 (8–15)
United States of America	2036	3499 (3253–3744)	0 (0–1)	84572 (78166–90978)	11 (7–15)
United States Virgin Islands	2022	0 (0–0)	0 (0–0)	8 (8–8)	5 (4–5)

United States Virgin Islands	2023	0 (0–0)	0 (0–0)	8 (7–9)	5 (4–5)
United States Virgin Islands	2024	0 (0–0)	0 (0–0)	8 (7–9)	5 (4–5)
United States Virgin Islands	2025	0 (0–0)	0 (0–0)	8 (7–10)	5 (4–5)
United States Virgin Islands	2026	0 (0–0)	0 (0–0)	9 (7–10)	5 (4–5)
United States Virgin Islands	2027	0 (0–0)	0 (0–0)	9 (7–10)	5 (4–5)
United States Virgin Islands	2028	0 (0–0)	0 (0–0)	9 (7–10)	5 (4–5)
United States Virgin Islands	2029	0 (0–0)	0 (0–0)	9 (7–11)	5 (4–6)
United States Virgin Islands	2030	0 (0–0)	0 (0–0)	9 (7–11)	5 (3–6)
United States Virgin Islands	2031	0 (0–0)	0 (0–0)	9 (7–11)	5 (3–6)
United States Virgin Islands	2032	0 (0–0)	0 (0–0)	10 (7–12)	5 (3–6)
United States Virgin Islands	2033	0 (0–0)	0 (0–0)	10 (7–12)	5 (3–6)
United States Virgin Islands	2034	0 (0–0)	0 (0–0)	10 (8–12)	5 (3–6)
United States Virgin Islands	2035	0 (0–1)	0 (0–0)	10 (8–12)	5 (3–6)
United States Virgin Islands	2036	0 (0–1)	0 (0–0)	10 (8–13)	5 (3–6)
Uruguay	2022	16 (16–17)	0 (0–0)	410 (387–433)	8 (8–9)
Uruguay	2023	17 (16–18)	0 (0–0)	419 (386–451)	8 (8–9)
Uruguay	2024	17 (16–19)	0 (0–0)	427 (387–467)	9 (8–10)
Uruguay	2025	17 (16–19)	0 (0–0)	436 (390–482)	9 (8–10)
Uruguay	2026	18 (16–20)	0 (0–0)	444 (393–495)	9 (8–10)
Uruguay	2027	18 (16–20)	0 (0–0)	452 (396–509)	9 (8–10)
Uruguay	2028	18 (16–21)	0 (0–0)	461 (400–522)	9 (8–11)
Uruguay	2029	19 (16–21)	0 (0–0)	469 (404–534)	9 (8–11)
Uruguay	2030	19 (17–22)	0 (0–0)	478 (409–547)	10 (8–11)
Uruguay	2031	20 (17–22)	0 (0–0)	486 (413–559)	10 (8–11)
Uruguay	2032	20 (17–23)	0 (0–0)	494 (418–571)	10 (8–12)
Uruguay	2033	20 (17–23)	0 (0–0)	503 (423–582)	10 (8–12)
Uruguay	2034	21 (17–24)	0 (0–0)	511 (428–594)	10 (8–12)
Uruguay	2035	21 (18–24)	0 (0–0)	520 (434–606)	10 (8–12)
Uruguay	2036	21 (18–25)	0 (0–0)	528 (439–617)	10 (8–13)
Uzbekistan	2022	89 (86–91)	0 (0–0)	2758 (2683–2834)	9 (8–9)
Uzbekistan	2023	91 (88–94)	0 (0–0)	2824 (2717–2932)	8 (8–9)
Uzbekistan	2024	93 (89–97)	0 (0–0)	2890 (2758–3021)	8 (7–9)
Uzbekistan	2025	95 (91–100)	0 (0–0)	2956 (2804–3108)	8 (7–10)

Uzbekistan	2026	97 (92–102)	0 (0–0)	3021 (2851–3191)	8 (7–10)
Uzbekistan	2027	99 (94–105)	0 (0–0)	3087 (2901–3273)	8 (6–10)
Uzbekistan	2028	101 (96–107)	0 (0–0)	3153 (2952–3354)	8 (6–10)
Uzbekistan	2029	104 (97–110)	0 (0–0)	3218 (3004–3433)	8 (6–10)
Uzbekistan	2030	106 (99–112)	0 (0–0)	3284 (3056–3512)	8 (5–11)
Uzbekistan	2031	108 (101–115)	0 (0–0)	3350 (3110–3590)	8 (5–11)
Uzbekistan	2032	110 (103–117)	0 (0–0)	3415 (3164–3667)	8 (4–11)
Uzbekistan	2033	112 (104–120)	0 (0–0)	3481 (3218–3744)	8 (4–11)
Uzbekistan	2034	114 (106–122)	0 (0–0)	3547 (3273–3821)	8 (3–12)
Uzbekistan	2035	116 (108–125)	0 (0–0)	3613 (3328–3897)	7 (3–12)
Uzbekistan	2036	118 (110–127)	0 (0–0)	3678 (3384–3972)	7 (2–12)
Vanuatu	2022	1 (1–1)	0 (0–0)	21 (20–21)	10 (10–10)
Vanuatu	2023	1 (1–1)	0 (0–0)	21 (20–22)	10 (9–11)
Vanuatu	2024	1 (1–1)	0 (0–0)	21 (20–22)	10 (9–11)
Vanuatu	2025	1 (1–1)	0 (0–0)	22 (20–23)	10 (9–11)
Vanuatu	2026	1 (1–1)	0 (0–0)	22 (20–24)	10 (8–12)
Vanuatu	2027	1 (1–1)	0 (0–0)	22 (20–24)	10 (8–12)
Vanuatu	2028	1 (1–1)	0 (0–0)	23 (21–25)	10 (8–12)
Vanuatu	2029	1 (1–1)	0 (0–0)	23 (21–25)	10 (8–12)
Vanuatu	2030	1 (1–1)	0 (0–0)	23 (21–26)	10 (7–12)
Vanuatu	2031	1 (1–1)	0 (0–0)	24 (21–26)	10 (7–13)
Vanuatu	2032	1 (1–1)	0 (0–0)	24 (21–27)	10 (7–13)
Vanuatu	2033	1 (1–1)	0 (0–0)	24 (21–27)	10 (7–13)
Vanuatu	2034	1 (1–1)	0 (0–0)	25 (22–28)	10 (7–13)
Vanuatu	2035	1 (1–1)	0 (0–0)	25 (22–28)	10 (7–13)
Vanuatu	2036	1 (1–1)	0 (0–0)	25 (22–29)	10 (7–13)
Venezuela (Bolivarian Republic of)	2022	54 (47–62)	0 (0–0)	1388 (1192–1585)	5 (3–6)
Venezuela (Bolivarian Republic of)	2023	53 (40–66)	0 (0–0)	1354 (1004–1703)	5 (1–8)
Venezuela (Bolivarian Republic of)	2024	51 (33–69)	0 (0–0)	1295 (826–1763)	5 (0–9)
Venezuela (Bolivarian Republic of)	2025	48 (27–69)	0 (-0–0)	1228 (676–1780)	5 (-1–10)
Venezuela (Bolivarian Republic of)	2026	45 (22–69)	0 (-0–0)	1163 (558–1769)	5 (-2–11)
Venezuela (Bolivarian Republic of)	2027	43 (18–67)	0 (-0–0)	1108 (472–1744)	5 (-3–12)
Venezuela (Bolivarian Republic of)	2028	41 (15–66)	0 (-0–1)	1063 (412–1715)	5 (-4–13)

Venezuela (Bolivarian Republic of)	2029	39 (14–65)	0 (-0–1)	1030 (371–1689)	5 (-5–14)
Venezuela (Bolivarian Republic of)	2030	38 (13–64)	0 (-0–1)	1007 (346–1669)	5 (-6–15)
Venezuela (Bolivarian Republic of)	2031	38 (12–64)	0 (-0–1)	993 (330–1655)	5 (-6–16)
Venezuela (Bolivarian Republic of)	2032	37 (11–63)	0 (-0–1)	985 (322–1647)	5 (-7–16)
Venezuela (Bolivarian Republic of)	2033	37 (11–63)	0 (-0–1)	981 (319–1644)	5 (-8–17)
Venezuela (Bolivarian Republic of)	2034	37 (11–63)	0 (-0–1)	981 (319–1643)	5 (-8–18)
Venezuela (Bolivarian Republic of)	2035	37 (11–63)	0 (-0–1)	983 (320–1645)	5 (-9–18)
Venezuela (Bolivarian Republic of)	2036	37 (11–63)	0 (-0–1)	985 (323–1648)	5 (-9–19)
Viet Nam	2022	2109 (2078–2141)	2 (2–2)	65314 (64382–66245)	55 (54–57)
Viet Nam	2023	2172 (2117–2226)	2 (2–2)	67174 (65504–68844)	55 (53–57)
Viet Nam	2024	2234 (2154–2313)	2 (2–2)	69034 (66548–71521)	54 (52–57)
Viet Nam	2025	2296 (2188–2403)	2 (2–2)	70894 (67509–74280)	54 (51–57)
Viet Nam	2026	2358 (2221–2495)	2 (2–2)	72755 (68392–77118)	54 (50–57)
Viet Nam	2027	2420 (2251–2588)	2 (2–2)	74615 (69199–80031)	53 (50–57)
Viet Nam	2028	2482 (2279–2684)	2 (2–2)	76475 (69937–83014)	53 (49–57)
Viet Nam	2029	2544 (2305–2782)	2 (2–2)	78336 (70608–86063)	52 (48–56)
Viet Nam	2030	2606 (2330–2882)	2 (2–2)	80196 (71216–89176)	52 (47–56)
Viet Nam	2031	2668 (2352–2983)	2 (2–2)	82056 (71764–92349)	51 (47–56)
Viet Nam	2032	2730 (2373–3086)	2 (2–2)	83916 (72253–95579)	51 (46–56)
Viet Nam	2033	2792 (2393–3191)	2 (2–2)	85777 (72688–98865)	50 (45–55)
Viet Nam	2034	2854 (2410–3297)	2 (1–2)	87637 (73069–102205)	50 (45–55)
Viet Nam	2035	2916 (2427–3405)	2 (1–2)	89497 (73399–105596)	50 (44–55)
Viet Nam	2036	2978 (2442–3514)	2 (1–2)	91358 (73679–109036)	49 (43–55)
Yemen	2022	29 (29–29)	0 (0–0)	859 (845–873)	5 (5–5)
Yemen	2023	30 (29–31)	0 (0–0)	893 (867–920)	5 (5–5)
Yemen	2024	31 (30–33)	0 (0–0)	928 (887–969)	5 (5–6)
Yemen	2025	32 (31–34)	0 (0–0)	962 (904–1019)	5 (5–6)
Yemen	2026	34 (31–36)	0 (0–0)	996 (921–1072)	5 (5–6)
Yemen	2027	35 (32–38)	0 (0–0)	1030 (935–1125)	5 (5–6)
Yemen	2028	36 (32–39)	0 (0–0)	1065 (949–1181)	5 (5–6)
Yemen	2029	37 (33–41)	0 (0–0)	1099 (961–1237)	6 (5–6)
Yemen	2030	38 (33–43)	0 (0–0)	1133 (971–1295)	6 (4–7)
Yemen	2031	39 (33–45)	0 (0–0)	1167 (981–1354)	6 (4–7)

Yemen	2032	40 (34–47)	0 (0–0)	1202 (989–1414)	6 (4–7)
Yemen	2033	42 (34–49)	0 (0–0)	1236 (997–1475)	6 (4–7)
Yemen	2034	43 (34–51)	0 (0–0)	1270 (1003–1537)	6 (4–8)
Yemen	2035	44 (35–53)	0 (0–0)	1304 (1008–1601)	6 (4–8)
Yemen	2036	45 (35–55)	0 (0–0)	1339 (1013–1665)	6 (4–8)
Zambia	2022	17 (16–18)	0 (0–0)	475 (451–499)	6 (6–7)
Zambia	2023	18 (15–20)	0 (0–0)	494 (427–560)	6 (5–8)
Zambia	2024	18 (15–22)	0 (0–0)	513 (393–632)	7 (4–9)
Zambia	2025	19 (14–24)	0 (0–0)	532 (349–714)	7 (3–11)
Zambia	2026	19 (12–26)	0 (0–0)	550 (298–803)	7 (2–12)
Zambia	2027	20 (11–29)	0 (0–1)	569 (239–899)	7 (0–14)
Zambia	2028	21 (9–32)	0 (0–1)	588 (174–1003)	7 (-2–16)
Zambia	2029	21 (8–35)	0 (-0–1)	607 (102–1112)	7 (-3–18)
Zambia	2030	22 (6–38)	0 (-0–1)	626 (25–1227)	8 (-5–20)
Zambia	2031	22 (4–41)	0 (-0–1)	645 (-58–1347)	8 (-7–23)
Zambia	2032	23 (2–44)	0 (-0–1)	664 (-146–1473)	8 (-9–25)
Zambia	2033	24 (-1–48)	0 (-0–1)	682 (-238–1603)	8 (-12–28)
Zambia	2034	24 (-3–51)	0 (-0–1)	701 (-336–1738)	8 (-14–30)
Zambia	2035	25 (-5–55)	0 (-1–1)	720 (-437–1878)	8 (-16–33)
Zambia	2036	25 (-8–59)	0 (-1–1)	739 (-544–2022)	9 (-19–36)
Zimbabwe	2022	72 (69–75)	1 (1–1)	2184 (2093–2275)	27 (25–29)
Zimbabwe	2023	73 (66–79)	1 (1–1)	2210 (2015–2406)	27 (23–31)
Zimbabwe	2024	74 (63–84)	1 (1–1)	2236 (1923–2548)	27 (21–33)
Zimbabwe	2025	74 (60–88)	1 (1–1)	2260 (1826–2694)	27 (18–36)
Zimbabwe	2026	75 (57–93)	1 (1–2)	2281 (1727–2835)	27 (15–39)
Zimbabwe	2027	76 (54–98)	1 (1–2)	2299 (1631–2968)	27 (11–43)
Zimbabwe	2028	77 (52–102)	1 (0–2)	2314 (1542–3086)	27 (7–46)
Zimbabwe	2029	77 (49–105)	1 (0–2)	2325 (1461–3188)	27 (3–50)
Zimbabwe	2030	78 (47–108)	1 (0–2)	2332 (1392–3272)	27 (-1–55)
Zimbabwe	2031	78 (46–111)	1 (-0–2)	2335 (1333–3336)	27 (-6–59)
Zimbabwe	2032	78 (44–112)	1 (-0–2)	2334 (1286–3382)	27 (-11–64)
Zimbabwe	2033	79 (44–114)	1 (-0–3)	2330 (1249–3410)	27 (-16–69)
Zimbabwe	2034	79 (43–114)	1 (-1–3)	2322 (1221–3423)	26 (-21–74)

Zimbabwe	2035	78 (42–114)	1 (-1–3)	2312 (1200–3424)	26 (-27–79)
Zimbabwe	2036	78 (42–114)	1 (-1–3)	2300 (1183–3416)	26 (-32–85)

Supplement table.6 Disease burden prediction for pancreatic cancer death and disability-adjusted life years numbers and age-standardized rate to 2036 in different countries.

Country	Year	Death		DALY	
		(N)	ASR(per100,000)	(N)	ASR(per100,000)
Afghanistan	2022	16 (16–16)	0 (0–0)	528 (524–531)	4 (4–4)
Afghanistan	2023	17 (16–17)	0 (0–0)	550 (541–559)	4 (4–4)
Afghanistan	2024	17 (17–18)	0 (0–0)	572 (557–588)	4 (4–4)
Afghanistan	2025	18 (17–18)	0 (0–0)	595 (571–618)	4 (4–5)
Afghanistan	2026	18 (17–19)	0 (0–0)	617 (584–649)	4 (4–5)
Afghanistan	2027	19 (17–20)	0 (0–0)	639 (596–682)	4 (4–5)
Afghanistan	2028	19 (17–21)	0 (0–0)	661 (608–715)	4 (4–5)
Afghanistan	2029	20 (17–22)	0 (0–0)	683 (619–748)	5 (4–5)
Afghanistan	2030	20 (17–22)	0 (0–0)	706 (629–783)	5 (4–5)
Afghanistan	2031	20 (18–23)	0 (0–0)	728 (638–818)	5 (4–5)
Afghanistan	2032	21 (18–24)	0 (0–0)	750 (647–854)	5 (4–5)
Afghanistan	2033	21 (18–25)	0 (0–0)	772 (655–890)	5 (4–5)
Afghanistan	2034	22 (18–26)	0 (0–0)	795 (662–927)	5 (4–5)
Afghanistan	2035	22 (18–27)	0 (0–0)	817 (669–965)	5 (4–6)
Afghanistan	2036	23 (17–28)	0 (0–0)	839 (675–1003)	5 (4–6)
Albania	2022	52 (51–54)	1 (1–1)	1181 (1139–1222)	27 (26–28)
Albania	2023	53 (50–56)	1 (1–1)	1200 (1125–1274)	27 (25–29)
Albania	2024	54 (50–58)	1 (1–1)	1221 (1117–1324)	27 (24–30)
Albania	2025	55 (50–60)	1 (1–1)	1242 (1114–1371)	27 (23–31)
Albania	2026	56 (50–63)	1 (1–1)	1265 (1114–1416)	27 (22–31)
Albania	2027	57 (50–65)	1 (1–1)	1287 (1116–1458)	27 (22–32)
Albania	2028	58 (50–66)	1 (1–1)	1310 (1121–1499)	27 (21–33)
Albania	2029	59 (50–68)	1 (1–1)	1333 (1127–1538)	27 (21–33)
Albania	2030	60 (51–70)	1 (1–1)	1355 (1134–1576)	27 (20–34)
Albania	2031	61 (51–72)	1 (1–1)	1378 (1142–1614)	27 (20–34)
Albania	2032	63 (52–74)	1 (1–1)	1401 (1151–1650)	27 (19–34)
Albania	2033	64 (52–75)	1 (1–2)	1423 (1161–1686)	27 (19–35)
Albania	2034	65 (52–77)	1 (1–2)	1446 (1171–1721)	27 (19–35)
Albania	2035	66 (53–78)	1 (1–2)	1469 (1182–1755)	27 (18–36)
Albania	2036	67 (54–80)	1 (1–2)	1491 (1194–1789)	27 (18–36)
Algeria	2022	86 (85–87)	0 (0–0)	2092 (2077–2108)	6 (6–6)

Algeria	2023	88 (87–90)	0 (0–0)	2162 (2128–2197)	6 (6–6)
Algeria	2024	91 (88–93)	0 (0–0)	2232 (2175–2290)	6 (5–6)
Algeria	2025	93 (90–97)	0 (0–0)	2302 (2218–2387)	6 (5–6)
Algeria	2026	96 (91–101)	0 (0–0)	2372 (2258–2487)	6 (5–6)
Algeria	2027	98 (92–105)	0 (0–0)	2442 (2295–2590)	6 (5–6)
Algeria	2028	101 (93–109)	0 (0–0)	2512 (2330–2695)	6 (5–6)
Algeria	2029	103 (94–113)	0 (0–0)	2582 (2362–2803)	6 (5–7)
Algeria	2030	106 (94–117)	0 (0–0)	2652 (2392–2913)	6 (5–7)
Algeria	2031	108 (95–122)	0 (0–0)	2722 (2419–3025)	6 (5–7)
Algeria	2032	111 (95–126)	0 (0–0)	2792 (2445–3140)	6 (4–7)
Algeria	2033	113 (96–131)	0 (0–0)	2862 (2469–3256)	6 (4–7)
Algeria	2034	116 (96–135)	0 (0–0)	2932 (2490–3374)	6 (4–7)
Algeria	2035	118 (97–140)	0 (0–0)	3002 (2510–3494)	6 (4–8)
Algeria	2036	121 (97–145)	0 (0–0)	3072 (2529–3616)	6 (4–8)
American Samoa	2022	0 (0–0)	1 (1–1)	12 (11–13)	22 (21–23)
American Samoa	2023	0 (0–0)	1 (1–1)	12 (11–13)	22 (21–24)
American Samoa	2024	0 (0–0)	1 (1–1)	12 (12–13)	23 (20–25)
American Samoa	2025	0 (0–0)	1 (1–1)	13 (12–14)	23 (20–25)
American Samoa	2026	0 (0–0)	1 (1–1)	13 (12–14)	23 (20–26)
American Samoa	2027	0 (0–0)	1 (1–1)	13 (12–15)	23 (20–26)
American Samoa	2028	0 (0–1)	1 (1–1)	13 (12–15)	23 (20–26)
American Samoa	2029	0 (0–1)	1 (1–1)	14 (12–15)	23 (20–27)
American Samoa	2030	0 (0–1)	1 (1–1)	14 (12–16)	24 (20–27)
American Samoa	2031	0 (0–1)	1 (1–1)	14 (12–16)	24 (20–27)
American Samoa	2032	0 (0–1)	1 (1–1)	14 (13–16)	24 (20–28)
American Samoa	2033	1 (0–1)	1 (1–1)	15 (13–16)	24 (20–28)
American Samoa	2034	1 (0–1)	1 (1–1)	15 (13–17)	24 (20–29)
American Samoa	2035	1 (0–1)	1 (1–1)	15 (13–17)	24 (20–29)
American Samoa	2036	1 (0–1)	1 (1–1)	15 (13–17)	25 (20–29)
Andorra	2022	2 (2–3)	2 (1–2)	60 (55–66)	39 (35–42)
Andorra	2023	2 (2–3)	2 (1–2)	60 (52–68)	38 (33–43)
Andorra	2024	2 (2–3)	1 (1–2)	60 (50–70)	37 (31–43)
Andorra	2025	2 (2–3)	1 (1–2)	60 (49–71)	36 (29–43)

Andorra	2026	2 (2–3)	1 (1–2)	60 (48–73)	35 (27–43)
Andorra	2027	2 (2–3)	1 (1–2)	60 (46–74)	34 (25–43)
Andorra	2028	2 (2–3)	1 (1–2)	60 (45–75)	33 (24–43)
Andorra	2029	2 (2–3)	1 (1–2)	60 (44–76)	32 (22–42)
Andorra	2030	2 (2–3)	1 (1–2)	60 (43–77)	31 (20–42)
Andorra	2031	2 (2–3)	1 (1–2)	60 (42–78)	30 (19–42)
Andorra	2032	2 (2–3)	1 (1–2)	60 (42–79)	29 (17–41)
Andorra	2033	2 (2–3)	1 (1–2)	60 (41–80)	28 (16–41)
Andorra	2034	2 (2–3)	1 (1–2)	60 (40–80)	28 (15–41)
Andorra	2035	2 (2–3)	1 (1–2)	60 (39–81)	27 (13–40)
Andorra	2036	2 (2–3)	1 (0–2)	60 (38–82)	26 (12–40)
Angola	2022	32 (31–32)	0 (0–0)	974 (961–987)	7 (6–7)
Angola	2023	33 (32–34)	0 (0–0)	1017 (988–1045)	7 (6–7)
Angola	2024	34 (33–36)	0 (0–0)	1059 (1012–1107)	7 (6–7)
Angola	2025	35 (33–38)	0 (0–0)	1102 (1032–1172)	7 (6–7)
Angola	2026	37 (34–40)	0 (0–0)	1145 (1050–1239)	7 (6–7)
Angola	2027	38 (34–42)	0 (0–0)	1187 (1066–1309)	7 (6–7)
Angola	2028	39 (35–44)	0 (0–0)	1230 (1079–1381)	6 (6–7)
Angola	2029	41 (35–46)	0 (0–0)	1273 (1090–1455)	6 (5–7)
Angola	2030	42 (35–49)	0 (0–0)	1315 (1100–1531)	6 (5–7)
Angola	2031	43 (36–51)	0 (0–0)	1358 (1107–1608)	6 (5–7)
Angola	2032	45 (36–54)	0 (0–0)	1400 (1113–1687)	6 (5–7)
Angola	2033	46 (36–56)	0 (0–0)	1443 (1118–1768)	6 (5–7)
Angola	2034	47 (36–59)	0 (0–0)	1486 (1121–1851)	6 (5–7)
Angola	2035	49 (36–61)	0 (0–0)	1528 (1122–1935)	6 (5–7)
Angola	2036	50 (36–64)	0 (0–0)	1571 (1122–2020)	6 (5–7)
Antigua and Barbuda	2022	1 (0–1)	0 (0–1)	13 (12–14)	11 (10–12)
Antigua and Barbuda	2023	1 (0–1)	0 (0–1)	13 (12–14)	11 (10–13)
Antigua and Barbuda	2024	1 (0–1)	0 (0–1)	13 (12–15)	11 (9–14)
Antigua and Barbuda	2025	1 (0–1)	0 (0–1)	14 (12–15)	11 (9–14)
Antigua and Barbuda	2026	1 (0–1)	0 (0–1)	14 (12–15)	11 (8–15)
Antigua and Barbuda	2027	1 (0–1)	0 (0–1)	14 (12–16)	11 (8–15)
Antigua and Barbuda	2028	1 (0–1)	0 (0–1)	14 (13–16)	11 (8–15)

Antigua and Barbuda	2029	1 (0–1)	0 (0–1)	15 (13–17)	11 (7–15)
Antigua and Barbuda	2030	1 (0–1)	0 (0–1)	15 (13–17)	11 (7–16)
Antigua and Barbuda	2031	1 (0–1)	0 (0–1)	15 (13–17)	11 (7–16)
Antigua and Barbuda	2032	1 (1–1)	0 (0–1)	15 (13–18)	11 (7–16)
Antigua and Barbuda	2033	1 (1–1)	0 (0–1)	16 (13–18)	11 (6–16)
Antigua and Barbuda	2034	1 (1–1)	0 (0–1)	16 (13–18)	11 (6–17)
Antigua and Barbuda	2035	1 (1–1)	0 (0–1)	16 (14–19)	11 (6–17)
Antigua and Barbuda	2036	1 (1–1)	0 (0–1)	16 (14–19)	11 (6–17)
Argentina	2022	703 (679–726)	1 (1–1)	18345 (17698–18993)	34 (32–35)
Argentina	2023	682 (636–728)	1 (1–1)	17990 (16802–19177)	32 (29–34)
Argentina	2024	669 (601–737)	1 (1–1)	17798 (16122–19475)	30 (26–34)
Argentina	2025	660 (572–748)	1 (1–1)	17695 (15583–19807)	28 (22–34)
Argentina	2026	654 (547–761)	1 (0–1)	17639 (15139–20140)	26 (19–34)
Argentina	2027	651 (526–775)	1 (0–1)	17609 (14759–20460)	24 (15–34)
Argentina	2028	648 (508–789)	1 (-0–1)	17593 (14424–20762)	23 (11–34)
Argentina	2029	647 (492–802)	0 (-0–1)	17585 (14123–21046)	21 (7–34)
Argentina	2030	646 (477–815)	0 (-1–1)	17580 (13847–21313)	19 (3–35)
Argentina	2031	645 (463–827)	0 (-1–1)	17577 (13591–21564)	17 (-1–35)
Argentina	2032	645 (450–839)	0 (-1–1)	17576 (13350–21802)	15 (-5–36)
Argentina	2033	644 (438–850)	0 (-1–1)	17575 (13123–22028)	14 (-9–37)
Argentina	2034	644 (427–861)	-0 (-2–1)	17575 (12907–22243)	12 (-14–37)
Argentina	2035	644 (417–872)	-0 (-2–2)	17575 (12701–22449)	10 (-18–38)
Argentina	2036	644 (406–882)	-0 (-2–2)	17575 (12503–22646)	8 (-23–39)
Armenia	2022	85 (81–90)	2 (2–2)	2171 (2045–2297)	49 (46–53)
Armenia	2023	87 (80–93)	2 (2–2)	2205 (2026–2383)	49 (45–54)
Armenia	2024	88 (80–96)	2 (2–2)	2238 (2019–2457)	49 (43–55)
Armenia	2025	90 (81–99)	2 (2–2)	2272 (2019–2524)	49 (43–56)
Armenia	2026	91 (81–102)	2 (2–2)	2305 (2023–2588)	49 (42–57)
Armenia	2027	93 (82–104)	2 (2–2)	2339 (2030–2648)	49 (41–58)
Armenia	2028	94 (82–106)	2 (2–2)	2373 (2038–2707)	49 (40–59)
Armenia	2029	96 (83–109)	2 (2–2)	2406 (2049–2764)	49 (40–59)
Armenia	2030	97 (84–111)	2 (2–2)	2440 (2061–2819)	49 (39–60)
Armenia	2031	99 (84–113)	2 (2–2)	2474 (2074–2873)	49 (38–60)

Armenia	2032	100 (85–116)	2 (2–3)	2507 (2088–2926)	49 (38–61)
Armenia	2033	102 (86–118)	2 (2–3)	2541 (2103–2978)	49 (37–61)
Armenia	2034	103 (87–120)	2 (2–3)	2574 (2119–3030)	49 (37–62)
Armenia	2035	105 (88–122)	2 (2–3)	2608 (2135–3081)	49 (36–62)
Armenia	2036	106 (89–124)	2 (2–3)	2642 (2152–3131)	49 (36–63)
Australia	2022	309 (299–319)	1 (1–1)	7146 (6895–7398)	17 (16–18)
Australia	2023	312 (298–327)	1 (1–1)	7202 (6846–7557)	17 (16–18)
Australia	2024	315 (297–333)	1 (1–1)	7257 (6822–7692)	17 (15–18)
Australia	2025	318 (297–338)	1 (1–1)	7312 (6809–7814)	16 (15–18)
Australia	2026	321 (298–344)	1 (1–1)	7367 (6805–7929)	16 (14–18)
Australia	2027	324 (299–349)	1 (1–1)	7422 (6807–8038)	16 (14–17)
Australia	2028	327 (299–354)	1 (1–1)	7477 (6813–8142)	15 (13–17)
Australia	2029	330 (300–359)	1 (1–1)	7533 (6822–8243)	15 (13–17)
Australia	2030	332 (302–363)	1 (0–1)	7588 (6834–8341)	15 (12–17)
Australia	2031	335 (303–368)	1 (0–1)	7643 (6848–8437)	14 (12–17)
Australia	2032	338 (304–372)	1 (0–1)	7698 (6865–8531)	14 (11–17)
Australia	2033	341 (306–377)	1 (0–1)	7753 (6883–8623)	14 (11–16)
Australia	2034	344 (307–381)	1 (0–1)	7808 (6903–8714)	13 (10–16)
Australia	2035	347 (309–386)	1 (0–1)	7863 (6923–8803)	13 (10–16)
Australia	2036	350 (310–390)	1 (0–1)	7919 (6946–8892)	13 (9–16)
Austria	2022	241 (233–250)	1 (1–1)	5668 (5471–5866)	35 (33–36)
Austria	2023	244 (228–259)	1 (1–1)	5723 (5332–6114)	34 (31–37)
Austria	2024	246 (226–267)	1 (1–1)	5778 (5261–6295)	33 (29–38)
Austria	2025	249 (225–273)	1 (1–1)	5833 (5215–6450)	33 (27–39)
Austria	2026	252 (224–279)	1 (1–2)	5887 (5184–6591)	32 (25–39)
Austria	2027	254 (224–285)	1 (1–2)	5942 (5162–6722)	31 (23–40)
Austria	2028	257 (224–290)	1 (1–2)	5997 (5146–6847)	31 (21–40)
Austria	2029	259 (224–295)	1 (1–2)	6051 (5136–6967)	30 (19–41)
Austria	2030	262 (224–300)	1 (1–2)	6106 (5131–7082)	29 (17–41)
Austria	2031	265 (225–305)	1 (1–2)	6161 (5129–7193)	29 (16–42)
Austria	2032	267 (225–309)	1 (1–2)	6216 (5130–7302)	28 (14–43)
Austria	2033	270 (226–314)	1 (0–2)	6270 (5133–7408)	27 (12–43)
Austria	2034	273 (227–318)	1 (0–2)	6325 (5139–7512)	27 (9–44)

Austria	2035	275 (227–323)	1 (0–2)	6380 (5146–7613)	26 (7–45)
Austria	2036	278 (228–327)	1 (0–2)	6435 (5156–7714)	25 (5–46)
Azerbaijan	2022	75 (73–78)	1 (1–1)	2184 (2118–2249)	19 (18–20)
Azerbaijan	2023	75 (71–80)	1 (1–1)	2231 (2111–2350)	19 (17–20)
Azerbaijan	2024	74 (67–82)	1 (1–1)	2222 (2020–2423)	18 (16–21)
Azerbaijan	2025	74 (64–85)	1 (1–1)	2292 (2019–2565)	18 (14–22)
Azerbaijan	2026	74 (60–87)	1 (0–1)	2312 (1961–2664)	18 (13–23)
Azerbaijan	2027	73 (57–90)	1 (0–1)	2391 (1976–2807)	18 (12–23)
Azerbaijan	2028	73 (53–93)	1 (0–1)	2424 (1946–2901)	18 (11–24)
Azerbaijan	2029	73 (50–96)	1 (0–1)	2499 (1971–3027)	18 (10–25)
Azerbaijan	2030	73 (46–99)	1 (0–1)	2533 (1959–3108)	18 (10–26)
Azerbaijan	2031	72 (43–102)	1 (0–1)	2601 (1987–3214)	18 (9–26)
Azerbaijan	2032	72 (40–105)	1 (0–1)	2634 (1985–3284)	18 (8–27)
Azerbaijan	2033	72 (36–108)	1 (0–1)	2695 (2014–3376)	18 (7–28)
Azerbaijan	2034	72 (33–111)	1 (0–1)	2729 (2017–3441)	18 (7–28)
Azerbaijan	2035	72 (31–113)	1 (0–1)	2785 (2045–3525)	17 (6–29)
Azerbaijan	2036	72 (28–116)	1 (0–1)	2820 (2053–3588)	17 (5–30)
Bahamas	2022	1 (1–1)	0 (0–0)	40 (39–41)	9 (9–9)
Bahamas	2023	1 (1–2)	0 (0–0)	41 (39–43)	9 (8–10)
Bahamas	2024	1 (1–2)	0 (0–0)	42 (39–45)	9 (8–10)
Bahamas	2025	2 (1–2)	0 (0–0)	43 (39–46)	9 (8–10)
Bahamas	2026	2 (1–2)	0 (0–0)	43 (39–48)	9 (8–10)
Bahamas	2027	2 (1–2)	0 (0–0)	44 (39–49)	9 (8–10)
Bahamas	2028	2 (1–2)	0 (0–0)	45 (39–50)	9 (8–10)
Bahamas	2029	2 (1–2)	0 (0–0)	46 (40–52)	9 (8–10)
Bahamas	2030	2 (1–2)	0 (0–0)	46 (40–53)	9 (8–10)
Bahamas	2031	2 (1–2)	0 (0–0)	47 (40–54)	9 (8–10)
Bahamas	2032	2 (1–2)	0 (0–0)	48 (41–55)	9 (8–10)
Bahamas	2033	2 (1–2)	0 (0–0)	49 (41–56)	9 (8–10)
Bahamas	2034	2 (1–2)	0 (0–0)	50 (41–58)	9 (8–10)
Bahamas	2035	2 (1–2)	0 (0–0)	50 (42–59)	9 (8–10)
Bahamas	2036	2 (2–2)	0 (0–0)	51 (42–60)	9 (8–10)
Bahrain	2022	7 (7–7)	1 (1–1)	206 (204–208)	19 (18–19)

Bahrain	2023	8 (8–8)	1 (1–1)	219 (213–225)	19 (18–20)
Bahrain	2024	8 (8–9)	1 (1–1)	232 (221–243)	19 (17–21)
Bahrain	2025	9 (8–9)	1 (1–1)	245 (228–262)	19 (17–21)
Bahrain	2026	9 (8–10)	1 (1–1)	258 (234–282)	19 (16–22)
Bahrain	2027	10 (9–11)	1 (1–1)	271 (240–303)	19 (16–22)
Bahrain	2028	10 (9–12)	1 (1–1)	285 (245–324)	19 (16–22)
Bahrain	2029	11 (9–12)	1 (1–1)	298 (249–346)	19 (15–23)
Bahrain	2030	11 (9–13)	1 (1–1)	311 (253–369)	19 (15–23)
Bahrain	2031	12 (9–14)	1 (1–1)	324 (256–392)	19 (15–23)
Bahrain	2032	12 (9–15)	1 (1–1)	337 (259–416)	19 (14–24)
Bahrain	2033	13 (9–16)	1 (1–1)	350 (261–440)	19 (14–24)
Bahrain	2034	13 (9–17)	1 (1–1)	364 (263–464)	19 (14–24)
Bahrain	2035	14 (9–18)	1 (1–1)	377 (264–489)	19 (14–24)
Bahrain	2036	14 (10–19)	1 (1–1)	390 (265–515)	19 (14–24)
Bangladesh	2022	314 (308–321)	0 (0–0)	7852 (7733–7971)	5 (5–5)
Bangladesh	2023	326 (312–341)	0 (0–0)	8141 (7875–8407)	5 (5–6)
Bangladesh	2024	339 (314–363)	0 (0–0)	8430 (7984–8876)	5 (5–6)
Bangladesh	2025	351 (316–386)	0 (0–0)	8719 (8067–9372)	5 (5–6)
Bangladesh	2026	363 (315–411)	0 (0–0)	9008 (8125–9892)	5 (5–6)
Bangladesh	2027	375 (314–437)	0 (0–0)	9297 (8161–10434)	5 (5–6)
Bangladesh	2028	387 (311–464)	0 (0–0)	9587 (8177–10996)	5 (5–6)
Bangladesh	2029	400 (308–492)	0 (0–0)	9876 (8174–11577)	5 (5–6)
Bangladesh	2030	412 (303–521)	0 (0–0)	10165 (8154–12176)	5 (5–6)
Bangladesh	2031	424 (298–551)	0 (0–0)	10454 (8117–12791)	5 (4–6)
Bangladesh	2032	436 (291–581)	0 (0–0)	10743 (8064–13422)	5 (4–6)
Bangladesh	2033	449 (284–613)	0 (0–0)	11032 (7995–14069)	5 (4–6)
Bangladesh	2034	461 (276–645)	0 (0–0)	11321 (7913–14730)	5 (4–6)
Bangladesh	2035	473 (268–678)	0 (0–0)	11611 (7816–15405)	5 (4–6)
Bangladesh	2036	485 (258–712)	0 (0–0)	11900 (7705–16094)	5 (4–6)
Barbados	2022	2 (2–2)	0 (0–0)	51 (49–54)	10 (9–11)
Barbados	2023	2 (2–2)	0 (0–0)	52 (49–55)	10 (9–11)
Barbados	2024	2 (2–3)	0 (0–0)	52 (48–56)	10 (9–11)
Barbados	2025	2 (2–3)	0 (0–0)	53 (48–58)	10 (9–11)

Barbados	2026	2 (2–3)	0 (0–0)	54 (48–59)	10 (9–12)
Barbados	2027	2 (2–3)	0 (0–1)	54 (49–60)	10 (9–12)
Barbados	2028	2 (2–3)	0 (0–1)	55 (49–61)	10 (8–12)
Barbados	2029	2 (2–3)	0 (0–1)	55 (49–62)	10 (8–12)
Barbados	2030	2 (2–3)	0 (0–1)	56 (49–63)	10 (8–12)
Barbados	2031	2 (2–3)	0 (0–1)	56 (49–64)	10 (8–12)
Barbados	2032	3 (2–3)	0 (0–1)	57 (49–65)	10 (8–12)
Barbados	2033	3 (2–3)	0 (0–1)	58 (50–65)	10 (8–12)
Barbados	2034	3 (2–3)	0 (0–1)	58 (50–66)	10 (8–12)
Barbados	2035	3 (2–3)	0 (0–1)	59 (50–67)	10 (8–13)
Barbados	2036	3 (2–3)	0 (0–1)	59 (50–68)	10 (8–13)
Belarus	2022	211 (201–222)	1 (1–1)	5894 (5585–6203)	38 (36–40)
Belarus	2023	214 (198–229)	1 (1–1)	5959 (5521–6396)	38 (35–41)
Belarus	2024	216 (197–235)	1 (1–1)	6023 (5487–6558)	38 (34–41)
Belarus	2025	219 (197–240)	1 (1–1)	6087 (5469–6706)	38 (33–42)
Belarus	2026	221 (196–245)	1 (1–1)	6152 (5461–6843)	38 (33–42)
Belarus	2027	223 (196–250)	1 (1–1)	6216 (5459–6974)	38 (32–43)
Belarus	2028	226 (197–255)	1 (1–2)	6281 (5463–7099)	38 (32–43)
Belarus	2029	228 (197–259)	1 (1–2)	6345 (5471–7220)	38 (32–44)
Belarus	2030	230 (197–263)	1 (1–2)	6410 (5482–7337)	38 (31–44)
Belarus	2031	233 (198–267)	1 (1–2)	6474 (5496–7452)	38 (31–44)
Belarus	2032	235 (199–271)	1 (1–2)	6539 (5513–7564)	38 (31–45)
Belarus	2033	237 (199–275)	1 (1–2)	6603 (5532–7674)	38 (30–45)
Belarus	2034	240 (200–279)	1 (1–2)	6667 (5553–7782)	38 (30–45)
Belarus	2035	242 (201–283)	1 (1–2)	6732 (5575–7889)	38 (30–46)
Belarus	2036	244 (202–287)	1 (1–2)	6796 (5599–7994)	38 (29–46)
Belgium	2022	281 (266–297)	1 (1–1)	6314 (5933–6695)	31 (28–33)
Belgium	2023	281 (259–304)	1 (1–1)	6314 (5775–6852)	30 (27–33)
Belgium	2024	281 (254–309)	1 (1–1)	6314 (5654–6973)	30 (26–34)
Belgium	2025	281 (250–313)	1 (1–1)	6314 (5552–7076)	30 (25–34)
Belgium	2026	281 (246–316)	1 (1–1)	6314 (5463–7165)	29 (24–34)
Belgium	2027	281 (243–320)	1 (1–1)	6314 (5381–7247)	29 (24–34)
Belgium	2028	281 (240–323)	1 (1–1)	6314 (5307–7321)	29 (23–35)

Belgium	2029	281 (237–326)	1 (1–1)	6314 (5237–7391)	28 (22–35)
Belgium	2030	281 (234–328)	1 (1–1)	6314 (5172–7456)	28 (22–35)
Belgium	2031	281 (232–331)	1 (1–1)	6314 (5110–7518)	28 (21–35)
Belgium	2032	281 (229–333)	1 (1–1)	6314 (5051–7577)	28 (20–35)
Belgium	2033	281 (227–336)	1 (1–1)	6314 (4995–7633)	27 (20–35)
Belgium	2034	281 (225–338)	1 (1–1)	6314 (4941–7687)	27 (19–35)
Belgium	2035	281 (223–340)	1 (1–1)	6314 (4889–7739)	27 (18–35)
Belgium	2036	281 (221–342)	1 (1–1)	6314 (4839–7789)	26 (18–35)
Belize	2022	1 (1–1)	0 (0–0)	39 (38–40)	12 (11–13)
Belize	2023	1 (1–2)	0 (0–1)	40 (38–42)	12 (9–14)
Belize	2024	1 (1–2)	0 (0–1)	41 (38–44)	12 (8–15)
Belize	2025	2 (1–2)	0 (0–1)	42 (38–46)	12 (6–17)
Belize	2026	2 (1–2)	0 (0–1)	43 (39–47)	12 (4–19)
Belize	2027	2 (1–2)	0 (0–1)	44 (39–49)	12 (2–21)
Belize	2028	2 (1–2)	0 (0–1)	45 (40–50)	12 (-0–24)
Belize	2029	2 (1–2)	0 (0–1)	46 (40–52)	12 (-3–26)
Belize	2030	2 (1–2)	0 (0–1)	47 (41–53)	12 (-6–29)
Belize	2031	2 (1–2)	0 (0–1)	48 (41–54)	12 (-8–32)
Belize	2032	2 (1–2)	0 (0–1)	49 (42–56)	12 (-11–35)
Belize	2033	2 (2–2)	0 (0–1)	50 (43–57)	12 (-14–38)
Belize	2034	2 (2–2)	0 (0–1)	51 (43–58)	12 (-17–41)
Belize	2035	2 (2–2)	0 (0–1)	52 (44–59)	12 (-21–44)
Belize	2036	2 (2–2)	0 (0–1)	53 (45–61)	12 (-24–47)
Benin	2022	5 (5–5)	0 (0–0)	150 (147–153)	3 (3–3)
Benin	2023	5 (5–6)	0 (0–0)	153 (148–158)	3 (2–3)
Benin	2024	6 (5–6)	0 (0–0)	156 (150–162)	3 (2–3)
Benin	2025	6 (5–6)	0 (0–0)	159 (151–167)	3 (2–3)
Benin	2026	6 (5–6)	0 (0–0)	162 (153–171)	3 (2–3)
Benin	2027	6 (6–6)	0 (0–0)	165 (155–174)	3 (2–3)
Benin	2028	6 (6–6)	0 (0–0)	168 (157–178)	3 (2–3)
Benin	2029	6 (6–6)	0 (0–0)	171 (159–182)	3 (2–4)
Benin	2030	6 (6–7)	0 (0–0)	174 (162–186)	3 (1–4)
Benin	2031	6 (6–7)	0 (0–0)	176 (164–189)	3 (1–4)

Benin	2032	6 (6–7)	0 (0–0)	179 (166–193)	3 (1–4)
Benin	2033	6 (6–7)	0 (0–0)	182 (168–196)	3 (1–4)
Benin	2034	7 (6–7)	0 (0–0)	185 (171–200)	3 (1–5)
Benin	2035	7 (6–7)	0 (0–0)	188 (173–203)	3 (0–5)
Benin	2036	7 (6–7)	0 (0–0)	191 (175–207)	2 (0–5)
Bermuda	2022	1 (1–1)	1 (1–1)	26 (25–27)	20 (19–22)
Bermuda	2023	1 (1–1)	1 (1–1)	26 (24–28)	20 (18–22)
Bermuda	2024	1 (1–1)	1 (1–1)	27 (24–29)	20 (18–23)
Bermuda	2025	1 (1–1)	1 (1–1)	27 (24–30)	20 (18–23)
Bermuda	2026	1 (1–1)	1 (1–1)	27 (24–30)	20 (17–24)
Bermuda	2027	1 (1–1)	1 (1–1)	28 (24–31)	20 (17–24)
Bermuda	2028	1 (1–1)	1 (1–1)	28 (24–31)	20 (17–24)
Bermuda	2029	1 (1–1)	1 (1–1)	28 (24–32)	20 (16–24)
Bermuda	2030	1 (1–1)	1 (1–1)	28 (24–32)	20 (16–25)
Bermuda	2031	1 (1–1)	1 (1–1)	29 (25–33)	20 (16–25)
Bermuda	2032	1 (1–1)	1 (1–1)	29 (25–33)	20 (16–25)
Bermuda	2033	1 (1–2)	1 (1–1)	29 (25–34)	20 (16–25)
Bermuda	2034	1 (1–2)	1 (1–1)	30 (25–34)	20 (15–26)
Bermuda	2035	1 (1–2)	1 (1–1)	30 (25–35)	20 (15–26)
Bermuda	2036	1 (1–2)	1 (1–1)	30 (25–35)	20 (15–26)
Bhutan	2022	1 (1–1)	0 (0–0)	17 (17–17)	3 (3–3)
Bhutan	2023	1 (1–1)	0 (0–0)	17 (17–17)	3 (3–3)
Bhutan	2024	1 (1–1)	0 (0–0)	18 (17–18)	3 (3–3)
Bhutan	2025	1 (1–1)	0 (0–0)	18 (18–19)	3 (3–3)
Bhutan	2026	1 (1–1)	0 (0–0)	19 (18–19)	3 (3–3)
Bhutan	2027	1 (1–1)	0 (0–0)	19 (18–20)	3 (3–3)
Bhutan	2028	1 (1–1)	0 (0–0)	19 (18–21)	3 (3–3)
Bhutan	2029	1 (1–1)	0 (0–0)	20 (18–21)	3 (3–3)
Bhutan	2030	1 (1–1)	0 (0–0)	20 (19–22)	3 (3–3)
Bhutan	2031	1 (1–1)	0 (0–0)	21 (19–23)	3 (3–3)
Bhutan	2032	1 (1–1)	0 (0–0)	21 (19–24)	3 (3–3)
Bhutan	2033	1 (1–1)	0 (0–0)	22 (19–24)	3 (2–3)
Bhutan	2034	1 (1–1)	0 (0–0)	22 (19–25)	3 (2–3)

Bhutan	2035	1 (1–1)	0 (0–0)	23 (19–26)	3 (2–3)
Bhutan	2036	1 (1–1)	0 (0–0)	23 (19–27)	3 (2–3)
Bolivia (Plurinational State of)	2022	47 (47–48)	1 (1–1)	1231 (1215–1248)	13 (13–13)
Bolivia (Plurinational State of)	2023	47 (46–49)	1 (0–1)	1241 (1204–1277)	13 (12–13)
Bolivia (Plurinational State of)	2024	48 (45–50)	1 (0–1)	1250 (1188–1311)	13 (12–13)
Bolivia (Plurinational State of)	2025	48 (44–51)	1 (0–1)	1259 (1169–1349)	13 (12–13)
Bolivia (Plurinational State of)	2026	48 (43–53)	0 (0–1)	1268 (1146–1390)	12 (11–14)
Bolivia (Plurinational State of)	2027	48 (41–54)	0 (0–1)	1278 (1121–1434)	12 (11–14)
Bolivia (Plurinational State of)	2028	48 (40–56)	0 (0–1)	1287 (1092–1481)	12 (10–14)
Bolivia (Plurinational State of)	2029	48 (38–58)	0 (0–1)	1296 (1061–1531)	12 (10–15)
Bolivia (Plurinational State of)	2030	48 (36–59)	0 (0–1)	1305 (1028–1583)	12 (10–15)
Bolivia (Plurinational State of)	2031	48 (35–61)	0 (0–1)	1314 (992–1637)	12 (9–15)
Bolivia (Plurinational State of)	2032	48 (33–63)	0 (0–1)	1324 (954–1693)	12 (9–16)
Bolivia (Plurinational State of)	2033	48 (31–65)	0 (0–1)	1333 (914–1752)	12 (8–16)
Bolivia (Plurinational State of)	2034	48 (29–68)	0 (0–1)	1342 (872–1812)	12 (8–16)
Bolivia (Plurinational State of)	2035	48 (26–70)	0 (0–1)	1351 (828–1875)	12 (7–17)
Bolivia (Plurinational State of)	2036	48 (24–72)	0 (0–1)	1361 (782–1939)	12 (7–17)
Bosnia and Herzegovina	2022	100 (95–106)	2 (2–2)	2404 (2262–2545)	41 (38–43)
Bosnia and Herzegovina	2023	98 (90–107)	2 (1–2)	2244 (2000–2488)	40 (36–44)
Bosnia and Herzegovina	2024	96 (84–108)	2 (1–2)	2114 (1723–2506)	39 (33–44)
Bosnia and Herzegovina	2025	94 (78–110)	1 (1–2)	1967 (1418–2516)	38 (31–44)
Bosnia and Herzegovina	2026	92 (72–112)	1 (1–2)	1830 (1101–2560)	37 (28–45)
Bosnia and Herzegovina	2027	90 (65–114)	1 (1–2)	1687 (765–2610)	35 (25–46)
Bosnia and Herzegovina	2028	88 (59–116)	1 (1–2)	1548 (416–2680)	34 (23–46)
Bosnia and Herzegovina	2029	85 (52–119)	1 (1–2)	1406 (52–2761)	33 (20–47)
Bosnia and Herzegovina	2030	83 (45–121)	1 (1–2)	1266 (-324–2857)	32 (17–48)
Bosnia and Herzegovina	2031	81 (38–124)	1 (1–2)	1125 (-713–2964)	31 (14–49)
Bosnia and Herzegovina	2032	79 (30–127)	1 (1–2)	985 (-1113–3083)	30 (10–50)
Bosnia and Herzegovina	2033	77 (23–131)	1 (0–2)	844 (-1525–3213)	29 (7–51)
Bosnia and Herzegovina	2034	75 (15–134)	1 (0–2)	703 (-1947–3354)	28 (4–52)
Bosnia and Herzegovina	2035	72 (7–138)	1 (0–2)	562 (-2380–3505)	27 (1–53)
Bosnia and Herzegovina	2036	70 (-1–142)	1 (0–2)	422 (-2822–3666)	26 (-3–55)
Botswana	2022	9 (9–9)	1 (1–1)	256 (252–261)	16 (15–16)

Botswana	2023	9 (9–10)	1 (1–1)	261 (252–271)	16 (15–16)
Botswana	2024	9 (9–10)	1 (1–1)	266 (252–281)	16 (15–17)
Botswana	2025	10 (9–10)	1 (1–1)	271 (252–290)	16 (14–17)
Botswana	2026	10 (9–10)	1 (1–1)	276 (254–299)	16 (14–18)
Botswana	2027	10 (9–11)	1 (1–1)	282 (256–307)	16 (14–18)
Botswana	2028	10 (9–11)	1 (1–1)	287 (258–316)	16 (14–18)
Botswana	2029	10 (9–11)	1 (1–1)	292 (261–323)	16 (14–18)
Botswana	2030	10 (9–12)	1 (1–1)	297 (264–331)	16 (14–19)
Botswana	2031	11 (10–12)	1 (1–1)	303 (267–338)	16 (14–19)
Botswana	2032	11 (10–12)	1 (1–1)	308 (271–346)	16 (14–19)
Botswana	2033	11 (10–12)	1 (1–1)	313 (274–353)	16 (14–18)
Botswana	2034	11 (10–12)	1 (1–1)	319 (277–360)	16 (14–18)
Botswana	2035	11 (10–13)	1 (1–1)	324 (281–367)	16 (14–18)
Botswana	2036	12 (10–13)	1 (1–1)	329 (285–374)	16 (14–18)
Brazil	2022	1907 (1875–1940)	1 (1–1)	48494 (47706–49282)	18 (18–19)
Brazil	2023	1950 (1884–2016)	1 (1–1)	49563 (48022–51104)	18 (17–19)
Brazil	2024	1993 (1900–2086)	1 (1–1)	50632 (48458–52807)	18 (17–19)
Brazil	2025	2036 (1916–2155)	1 (1–1)	51701 (48913–54489)	17 (16–19)
Brazil	2026	2079 (1933–2224)	1 (1–1)	52770 (49365–56176)	17 (16–19)
Brazil	2027	2121 (1949–2294)	1 (1–1)	53839 (49804–57875)	17 (15–19)
Brazil	2028	2164 (1965–2364)	1 (1–1)	54908 (50225–59592)	17 (15–18)
Brazil	2029	2207 (1979–2435)	1 (1–1)	55977 (50627–61328)	16 (14–18)
Brazil	2030	2250 (1993–2506)	1 (1–1)	57047 (51009–63084)	16 (14–18)
Brazil	2031	2293 (2007–2579)	1 (1–1)	58116 (51371–64861)	16 (13–18)
Brazil	2032	2336 (2019–2652)	1 (1–1)	59185 (51711–66658)	15 (13–18)
Brazil	2033	2378 (2030–2726)	1 (1–1)	60254 (52032–68476)	15 (13–18)
Brazil	2034	2421 (2041–2801)	1 (1–1)	61323 (52331–70314)	15 (12–17)
Brazil	2035	2464 (2051–2877)	1 (0–1)	62392 (52611–72173)	15 (12–17)
Brazil	2036	2507 (2060–2953)	1 (0–1)	63461 (52871–74051)	14 (12–17)
Brunei Darussalam	2022	2 (2–2)	1 (1–1)	61 (60–62)	15 (14–15)
Brunei Darussalam	2023	2 (2–2)	1 (1–1)	62 (60–64)	15 (13–16)
Brunei Darussalam	2024	2 (2–2)	1 (0–1)	63 (60–66)	14 (12–16)
Brunei Darussalam	2025	2 (2–3)	1 (0–1)	64 (59–68)	14 (11–17)

Brunei Darussalam	2026	2 (2–3)	1 (0–1)	64 (58–71)	14 (10–17)
Brunei Darussalam	2027	2 (2–3)	1 (0–1)	65 (57–73)	13 (8–18)
Brunei Darussalam	2028	2 (2–3)	1 (0–1)	66 (56–76)	13 (7–19)
Brunei Darussalam	2029	3 (2–3)	1 (0–1)	67 (55–79)	13 (5–20)
Brunei Darussalam	2030	3 (2–3)	1 (0–1)	67 (53–82)	12 (4–21)
Brunei Darussalam	2031	3 (2–3)	1 (-0–1)	68 (52–85)	12 (2–22)
Brunei Darussalam	2032	3 (2–4)	1 (-0–1)	69 (50–88)	12 (0–23)
Brunei Darussalam	2033	3 (2–4)	0 (-0–1)	70 (48–91)	11 (-2–24)
Brunei Darussalam	2034	3 (2–4)	0 (-0–1)	70 (46–94)	11 (-4–25)
Brunei Darussalam	2035	3 (1–4)	0 (-0–1)	71 (44–98)	10 (-6–27)
Brunei Darussalam	2036	3 (1–4)	0 (-1–1)	72 (42–102)	10 (-8–28)
Bulgaria	2022	215 (204–227)	2 (2–2)	5939 (5602–6276)	50 (47–52)
Bulgaria	2023	215 (195–235)	2 (2–2)	5939 (5358–6520)	50 (45–54)
Bulgaria	2024	215 (189–241)	2 (1–2)	5939 (5190–6688)	50 (43–56)
Bulgaria	2025	215 (185–246)	2 (1–2)	5939 (5053–6825)	50 (42–57)
Bulgaria	2026	215 (181–250)	2 (1–2)	5939 (4935–6943)	50 (41–58)
Bulgaria	2027	215 (177–254)	2 (1–2)	5939 (4829–7049)	50 (40–59)
Bulgaria	2028	215 (174–257)	2 (1–2)	5939 (4732–7146)	50 (40–60)
Bulgaria	2029	215 (171–260)	2 (1–2)	5939 (4643–7235)	50 (39–60)
Bulgaria	2030	215 (168–263)	2 (1–2)	5939 (4559–7319)	50 (38–61)
Bulgaria	2031	215 (165–266)	2 (1–2)	5939 (4480–7398)	50 (38–62)
Bulgaria	2032	215 (162–268)	2 (1–2)	5939 (4405–7473)	50 (37–62)
Bulgaria	2033	215 (160–271)	2 (1–2)	5939 (4334–7544)	50 (36–63)
Bulgaria	2034	215 (158–273)	2 (1–2)	5939 (4266–7612)	50 (36–63)
Bulgaria	2035	215 (155–275)	2 (1–2)	5939 (4200–7678)	50 (35–64)
Bulgaria	2036	215 (153–278)	2 (1–2)	5939 (4137–7741)	50 (35–65)
Burkina Faso	2022	9 (8–9)	0 (0–0)	258 (255–261)	2 (2–2)
Burkina Faso	2023	9 (9–9)	0 (0–0)	266 (259–273)	2 (2–2)
Burkina Faso	2024	9 (9–9)	0 (0–0)	274 (262–286)	2 (2–3)
Burkina Faso	2025	9 (9–10)	0 (0–0)	282 (264–300)	2 (2–3)
Burkina Faso	2026	10 (9–10)	0 (0–0)	290 (265–315)	2 (2–3)
Burkina Faso	2027	10 (9–11)	0 (0–0)	298 (266–330)	3 (2–3)
Burkina Faso	2028	10 (9–11)	0 (0–0)	306 (267–345)	3 (2–3)

Burkina Faso	2029	10 (9–12)	0 (0–0)	314 (267–361)	3 (2–3)
Burkina Faso	2030	11 (9–12)	0 (0–0)	322 (266–378)	3 (2–3)
Burkina Faso	2031	11 (9–13)	0 (0–0)	330 (265–395)	3 (2–3)
Burkina Faso	2032	11 (9–13)	0 (0–0)	338 (264–413)	3 (2–3)
Burkina Faso	2033	11 (9–14)	0 (0–0)	346 (262–431)	3 (2–3)
Burkina Faso	2034	11 (9–14)	0 (0–0)	354 (260–449)	3 (2–3)
Burkina Faso	2035	12 (8–15)	0 (0–0)	362 (257–468)	3 (2–3)
Burkina Faso	2036	12 (8–15)	0 (0–0)	370 (254–487)	3 (2–3)
Burundi	2022	5 (5–6)	0 (0–0)	114 (71–157)	3 (3–3)
Burundi	2023	6 (5–6)	0 (0–0)	114 (71–157)	3 (3–3)
Burundi	2024	6 (5–6)	0 (0–0)	114 (71–157)	3 (3–3)
Burundi	2025	6 (5–6)	0 (0–0)	114 (71–157)	3 (3–3)
Burundi	2026	6 (5–6)	0 (0–0)	114 (71–157)	3 (2–3)
Burundi	2027	6 (5–6)	0 (0–0)	114 (71–157)	3 (2–3)
Burundi	2028	5 (5–6)	0 (0–0)	114 (71–157)	3 (2–3)
Burundi	2029	5 (5–6)	0 (0–0)	114 (71–157)	3 (2–4)
Burundi	2030	5 (4–6)	0 (0–0)	114 (71–157)	3 (2–4)
Burundi	2031	5 (4–6)	0 (0–0)	114 (71–157)	3 (2–4)
Burundi	2032	5 (4–6)	0 (0–0)	114 (71–157)	3 (1–4)
Burundi	2033	5 (4–6)	0 (0–0)	114 (71–157)	3 (1–4)
Burundi	2034	4 (3–6)	0 (0–0)	114 (71–157)	3 (1–4)
Burundi	2035	4 (3–5)	0 (0–0)	114 (71–157)	3 (1–5)
Burundi	2036	4 (3–5)	0 (0–0)	114 (71–157)	3 (1–5)
Cabo Verde	2022	2 (1–2)	0 (0–0)	44 (42–46)	9 (8–9)
Cabo Verde	2023	2 (1–2)	0 (0–0)	45 (42–49)	9 (8–10)
Cabo Verde	2024	2 (1–2)	0 (0–0)	47 (42–51)	9 (7–11)
Cabo Verde	2025	2 (1–2)	0 (0–0)	48 (42–54)	9 (6–11)
Cabo Verde	2026	2 (1–2)	0 (0–0)	49 (42–56)	9 (6–12)
Cabo Verde	2027	2 (1–2)	0 (0–0)	50 (42–58)	9 (5–12)
Cabo Verde	2028	2 (1–2)	0 (0–1)	52 (43–61)	9 (5–13)
Cabo Verde	2029	2 (1–2)	0 (0–1)	53 (43–63)	9 (4–13)
Cabo Verde	2030	2 (2–2)	0 (0–1)	54 (44–65)	9 (4–14)
Cabo Verde	2031	2 (2–2)	0 (0–1)	55 (44–67)	9 (4–14)

Cabo Verde	2032	2 (2–2)	0 (0–1)	57 (45–69)	9 (3–14)
Cabo Verde	2033	2 (2–3)	0 (0–1)	58 (46–70)	9 (3–15)
Cabo Verde	2034	2 (2–3)	0 (0–1)	59 (46–72)	9 (3–15)
Cabo Verde	2035	2 (2–3)	0 (0–1)	61 (47–74)	9 (2–15)
Cabo Verde	2036	2 (2–3)	0 (0–1)	62 (48–76)	9 (2–16)
Cambodia	2022	75 (75–75)	1 (1–1)	2055 (2045–2064)	15 (15–15)
Cambodia	2023	78 (77–79)	1 (1–1)	2132 (2111–2154)	15 (15–15)
Cambodia	2024	81 (79–82)	1 (1–1)	2210 (2174–2246)	15 (15–15)
Cambodia	2025	84 (82–85)	1 (1–1)	2287 (2235–2340)	15 (15–16)
Cambodia	2026	86 (84–89)	1 (1–1)	2365 (2293–2436)	15 (15–16)
Cambodia	2027	89 (86–92)	1 (1–1)	2442 (2350–2534)	15 (15–16)
Cambodia	2028	92 (88–96)	1 (1–1)	2520 (2406–2633)	15 (15–16)
Cambodia	2029	95 (90–100)	1 (1–1)	2597 (2460–2734)	15 (15–16)
Cambodia	2030	98 (92–104)	1 (1–1)	2675 (2512–2837)	16 (15–16)
Cambodia	2031	101 (94–107)	1 (1–1)	2752 (2564–2941)	16 (15–17)
Cambodia	2032	103 (96–111)	1 (1–1)	2830 (2613–3046)	16 (15–17)
Cambodia	2033	106 (98–115)	1 (1–1)	2907 (2662–3152)	16 (15–17)
Cambodia	2034	109 (99–119)	1 (1–1)	2985 (2709–3260)	16 (15–17)
Cambodia	2035	112 (101–123)	1 (1–1)	3062 (2756–3368)	16 (15–17)
Cambodia	2036	115 (103–127)	1 (1–1)	3139 (2801–3478)	16 (15–17)
Cameroon	2022	25 (25–25)	0 (0–0)	775 (770–780)	5 (5–5)
Cameroon	2023	25 (25–26)	0 (0–0)	798 (788–808)	5 (5–5)
Cameroon	2024	26 (26–27)	0 (0–0)	822 (806–838)	5 (5–5)
Cameroon	2025	27 (26–28)	0 (0–0)	845 (822–869)	5 (5–5)
Cameroon	2026	27 (26–29)	0 (0–0)	869 (838–900)	5 (4–5)
Cameroon	2027	28 (27–30)	0 (0–0)	893 (853–932)	5 (4–6)
Cameroon	2028	29 (27–30)	0 (0–0)	916 (867–965)	5 (4–6)
Cameroon	2029	29 (27–32)	0 (0–0)	940 (881–999)	5 (4–6)
Cameroon	2030	30 (27–33)	0 (0–0)	964 (894–1033)	5 (4–6)
Cameroon	2031	31 (28–34)	0 (0–0)	987 (907–1067)	5 (4–6)
Cameroon	2032	31 (28–35)	0 (0–0)	1011 (919–1103)	5 (3–6)
Cameroon	2033	32 (28–36)	0 (0–0)	1034 (931–1138)	5 (3–6)
Cameroon	2034	33 (28–37)	0 (0–0)	1058 (942–1174)	5 (3–7)

Cameroon	2035	33 (28–38)	0 (0–0)	1082 (953–1211)	5 (3–7)
Cameroon	2036	34 (29–39)	0 (0–0)	1105 (963–1248)	5 (2–7)
Canada	2022	745 (717–773)	1 (1–1)	16099 (15490–16707)	23 (22–24)
Canada	2023	751 (711–791)	1 (1–1)	16184 (15323–17044)	22 (21–24)
Canada	2024	757 (708–806)	1 (1–1)	16269 (15215–17322)	22 (20–24)
Canada	2025	763 (706–819)	1 (1–1)	16354 (15138–17571)	21 (19–23)
Canada	2026	769 (705–832)	1 (1–1)	16439 (15079–17799)	21 (18–23)
Canada	2027	774 (705–843)	1 (1–1)	16524 (15035–18014)	20 (17–23)
Canada	2028	780 (706–855)	1 (1–1)	16610 (15000–18219)	19 (17–22)
Canada	2029	786 (706–866)	1 (1–1)	16695 (14974–18415)	19 (16–22)
Canada	2030	792 (707–877)	1 (1–1)	16780 (14955–18605)	18 (15–22)
Canada	2031	798 (709–887)	1 (1–1)	16865 (14942–18789)	18 (14–21)
Canada	2032	804 (710–897)	1 (1–1)	16950 (14933–18968)	17 (13–21)
Canada	2033	810 (712–907)	1 (1–1)	17036 (14929–19143)	17 (13–20)
Canada	2034	816 (714–917)	1 (1–1)	17121 (14928–19314)	16 (12–20)
Canada	2035	821 (716–927)	1 (1–1)	17206 (14930–19482)	15 (11–19)
Canada	2036	827 (718–936)	1 (0–1)	17291 (14935–19647)	15 (11–19)
Central African Republic	2022	3 (3–3)	0 (0–0)	111 (109–112)	4 (4–4)
Central African Republic	2023	3 (3–4)	0 (0–0)	113 (109–116)	4 (4–4)
Central African Republic	2024	4 (3–4)	0 (0–0)	114 (109–120)	4 (4–4)
Central African Republic	2025	4 (3–4)	0 (0–0)	116 (108–124)	4 (4–4)
Central African Republic	2026	4 (3–4)	0 (0–0)	118 (106–129)	4 (3–4)
Central African Republic	2027	4 (3–4)	0 (0–0)	119 (105–134)	4 (3–4)
Central African Republic	2028	4 (3–4)	0 (0–0)	121 (103–139)	4 (3–4)
Central African Republic	2029	4 (3–4)	0 (0–0)	123 (101–145)	4 (3–4)
Central African Republic	2030	4 (3–5)	0 (0–0)	124 (98–150)	4 (3–5)
Central African Republic	2031	4 (3–5)	0 (0–0)	126 (96–156)	4 (3–5)
Central African Republic	2032	4 (3–5)	0 (0–0)	128 (93–162)	4 (3–5)
Central African Republic	2033	4 (3–5)	0 (0–0)	129 (90–168)	4 (2–5)
Central African Republic	2034	4 (3–5)	0 (0–0)	131 (87–175)	4 (2–5)
Central African Republic	2035	4 (3–5)	0 (0–0)	133 (84–182)	4 (2–5)
Central African Republic	2036	4 (3–6)	0 (0–0)	134 (80–188)	4 (2–5)
Chad	2022	7 (7–7)	0 (0–0)	198 (196–200)	3 (3–3)

Chad	2023	7 (7–7)	0 (0–0)	204 (200–209)	3 (3–3)
Chad	2024	7 (7–8)	0 (0–0)	210 (203–218)	3 (3–3)
Chad	2025	8 (7–8)	0 (0–0)	217 (206–227)	3 (3–3)
Chad	2026	8 (7–8)	0 (0–0)	223 (209–237)	3 (3–3)
Chad	2027	8 (7–9)	0 (0–0)	229 (211–247)	3 (3–4)
Chad	2028	8 (7–9)	0 (0–0)	235 (213–258)	3 (3–4)
Chad	2029	8 (7–9)	0 (0–0)	241 (214–268)	3 (3–4)
Chad	2030	8 (7–10)	0 (0–0)	248 (216–280)	3 (2–4)
Chad	2031	9 (7–10)	0 (0–0)	254 (217–291)	3 (2–4)
Chad	2032	9 (7–10)	0 (0–0)	260 (217–303)	3 (2–4)
Chad	2033	9 (7–11)	0 (0–0)	266 (218–314)	3 (2–4)
Chad	2034	9 (7–11)	0 (0–0)	272 (218–326)	3 (2–5)
Chad	2035	9 (7–11)	0 (0–0)	279 (218–339)	3 (2–5)
Chad	2036	10 (7–12)	0 (0–0)	285 (218–351)	3 (2–5)
Chile	2022	177 (170–184)	1 (1–1)	4870 (4666–5075)	19 (18–21)
Chile	2023	180 (171–190)	1 (1–1)	4957 (4668–5246)	19 (17–21)
Chile	2024	184 (172–195)	1 (1–1)	5044 (4689–5398)	19 (17–22)
Chile	2025	187 (173–201)	1 (1–1)	5130 (4721–5539)	20 (17–22)
Chile	2026	190 (175–205)	1 (1–1)	5217 (4760–5674)	20 (17–22)
Chile	2027	193 (176–210)	1 (1–1)	5304 (4803–5805)	20 (17–23)
Chile	2028	196 (178–215)	1 (1–1)	5390 (4849–5931)	20 (17–23)
Chile	2029	200 (180–219)	1 (1–1)	5477 (4899–6055)	20 (17–23)
Chile	2030	203 (182–224)	1 (1–1)	5564 (4950–6177)	20 (17–23)
Chile	2031	206 (184–228)	1 (1–1)	5650 (5004–6297)	20 (17–23)
Chile	2032	209 (186–232)	1 (1–1)	5737 (5059–6415)	20 (17–23)
Chile	2033	213 (189–236)	1 (1–1)	5824 (5116–6532)	20 (17–23)
Chile	2034	216 (191–241)	1 (1–1)	5911 (5173–6648)	20 (17–23)
Chile	2035	219 (193–245)	1 (1–1)	5997 (5232–6762)	20 (17–24)
Chile	2036	222 (196–249)	1 (1–1)	6084 (5292–6876)	20 (17–24)
China	2022	4095 (23884–2430[illegible]	1 (1–1)	[illegible]16259 (610628–621890	28 (27–28)
China	2023	4897 (24338–2545[illegible]	1 (1–1)	[illegible]31362 (617127–645598	28 (27–28)
China	2024	5699 (24773–2662[illegible]	1 (1–1)	[illegible]46466 (621493–671439	28 (27–29)
China	2025	[illegible]501 (25167–2783[illegible]	1 (1–1)	[illegible]61569 (624079–699060	28 (26–29)

China	2026	7303 (25521–29084	1 (1–1)	576673 (625106–728240	28 (26–29)
China	2027	3105 (25838–3037	1 (1–1)	591777 (624730–758824	28 (26–29)
China	2028	3906 (26118–31694	1 (1–1)	706880 (623066–790694	28 (26–29)
China	2029	9708 (26366–3305	1 (1–1)	721984 (620207–823760	28 (26–30)
China	2030	0510 (26581–3444	1 (1–1)	737087 (616227–857947	28 (26–30)
China	2031	1312 (26766–3585	1 (1–1)	752191 (611189–893193	28 (25–30)
China	2032	2114 (26922–3730	1 (1–1)	767294 (605144–929444	28 (25–30)
China	2033	2916 (27051–3878	1 (1–1)	782398 (598139–966657	28 (25–30)
China	2034	3718 (27153–4028	1 (1–1)	97501 (590214–100478	28 (25–30)
China	2035	4520 (27229–4181	1 (1–1)	12605 (581404–104380	28 (25–30)
China	2036	5322 (27281–43364	1 (1–1)	27708 (571740–108367	28 (25–30)
Colombia	2022	172 (164–179)	0 (0–0)	4445 (4232–4658)	8 (7–9)
Colombia	2023	174 (163–184)	0 (0–0)	4489 (4188–4790)	8 (7–9)
Colombia	2024	176 (163–189)	0 (0–0)	4534 (4165–4902)	8 (7–9)
Colombia	2025	178 (163–193)	0 (0–0)	4578 (4152–5004)	8 (6–9)
Colombia	2026	180 (163–197)	0 (0–0)	4623 (4146–5099)	8 (6–10)
Colombia	2027	182 (164–200)	0 (0–0)	4667 (4145–5189)	8 (6–10)
Colombia	2028	184 (164–204)	0 (0–0)	4711 (4148–5275)	8 (6–10)
Colombia	2029	186 (165–207)	0 (0–0)	4756 (4154–5358)	8 (5–10)
Colombia	2030	188 (166–211)	0 (0–0)	4800 (4162–5439)	8 (5–11)
Colombia	2031	190 (166–214)	0 (0–0)	4845 (4171–5518)	8 (5–11)
Colombia	2032	192 (167–217)	0 (0–0)	4889 (4183–5595)	8 (4–12)
Colombia	2033	194 (168–220)	0 (0–0)	4934 (4196–5671)	8 (4–12)
Colombia	2034	196 (169–224)	0 (0–0)	4978 (4210–5746)	8 (3–12)
Colombia	2035	199 (170–227)	0 (0–1)	5022 (4226–5819)	8 (3–13)
Colombia	2036	201 (172–230)	0 (0–1)	5067 (4242–5891)	8 (3–13)
Comoros	2022	1 (1–1)	0 (0–0)	30 (29–31)	6 (5–6)
Comoros	2023	1 (1–1)	0 (0–0)	31 (30–33)	6 (5–6)
Comoros	2024	1 (1–1)	0 (0–0)	32 (30–34)	6 (5–6)
Comoros	2025	1 (1–1)	0 (0–0)	33 (30–36)	6 (5–6)
Comoros	2026	1 (1–1)	0 (0–0)	34 (31–38)	6 (5–7)
Comoros	2027	1 (1–1)	0 (0–0)	35 (31–40)	6 (5–7)
Comoros	2028	1 (1–2)	0 (0–0)	36 (31–42)	6 (5–7)

Comoros	2029	1 (1–2)	0 (0–0)	38 (31–44)	6 (5–7)
Comoros	2030	1 (1–2)	0 (0–0)	39 (32–46)	6 (5–7)
Comoros	2031	2 (1–2)	0 (0–0)	40 (32–48)	6 (4–7)
Comoros	2032	2 (1–2)	0 (0–0)	41 (32–50)	6 (4–7)
Comoros	2033	2 (1–2)	0 (0–0)	42 (32–52)	6 (4–8)
Comoros	2034	2 (1–2)	0 (0–0)	43 (32–54)	6 (4–8)
Comoros	2035	2 (1–2)	0 (0–0)	44 (32–56)	6 (4–8)
Comoros	2036	2 (1–2)	0 (0–0)	45 (32–58)	6 (4–8)
Congo	2022	8 (8–8)	0 (0–0)	248 (244–251)	8 (8–8)
Congo	2023	9 (8–9)	0 (0–0)	258 (250–265)	8 (7–8)
Congo	2024	9 (9–9)	0 (0–0)	267 (255–279)	8 (7–8)
Congo	2025	9 (9–10)	0 (0–0)	277 (259–295)	8 (7–9)
Congo	2026	10 (9–10)	0 (0–0)	287 (263–311)	8 (7–9)
Congo	2027	10 (9–11)	0 (0–0)	297 (266–328)	8 (7–9)
Congo	2028	10 (9–11)	0 (0–0)	306 (268–345)	8 (7–9)
Congo	2029	10 (9–12)	0 (0–0)	316 (270–362)	8 (6–10)
Congo	2030	11 (9–12)	0 (0–0)	326 (271–381)	8 (6–10)
Congo	2031	11 (9–13)	0 (0–0)	336 (272–399)	8 (6–11)
Congo	2032	11 (9–13)	0 (0–0)	346 (273–418)	8 (6–11)
Congo	2033	12 (9–14)	0 (0–0)	355 (273–438)	8 (5–11)
Congo	2034	12 (9–15)	0 (0–0)	365 (273–458)	8 (5–12)
Congo	2035	12 (9–15)	0 (0–0)	375 (272–478)	9 (5–12)
Congo	2036	13 (9–16)	0 (0–0)	385 (271–499)	9 (5–13)
Cook Islands	2022	0 (0–0)	1 (1–1)	4 (4–4)	15 (14–15)
Cook Islands	2023	0 (0–0)	1 (1–1)	4 (4–4)	15 (14–16)
Cook Islands	2024	0 (0–0)	1 (1–1)	4 (4–4)	15 (14–16)
Cook Islands	2025	0 (0–0)	1 (0–1)	4 (4–4)	15 (13–17)
Cook Islands	2026	0 (0–0)	1 (0–1)	4 (4–5)	15 (13–17)
Cook Islands	2027	0 (0–0)	1 (0–1)	4 (4–5)	15 (13–17)
Cook Islands	2028	0 (0–0)	1 (0–1)	4 (4–5)	15 (12–17)
Cook Islands	2029	0 (0–0)	1 (0–1)	4 (4–5)	15 (12–18)
Cook Islands	2030	0 (0–0)	1 (0–1)	4 (4–5)	15 (12–18)
Cook Islands	2031	0 (0–0)	1 (0–1)	4 (4–5)	15 (12–18)

Cook Islands	2032	0 (0–0)	1 (0–1)	5 (4–5)	15 (12–18)
Cook Islands	2033	0 (0–0)	1 (0–1)	5 (4–5)	15 (11–18)
Cook Islands	2034	0 (0–0)	1 (0–1)	5 (4–5)	15 (11–18)
Cook Islands	2035	0 (0–0)	1 (0–1)	5 (4–5)	15 (11–19)
Cook Islands	2036	0 (0–0)	1 (0–1)	5 (4–6)	15 (11–19)
Costa Rica	2022	30 (28–32)	1 (0–1)	720 (671–768)	13 (11–14)
Costa Rica	2023	30 (27–33)	1 (0–1)	735 (666–803)	13 (10–15)
Costa Rica	2024	31 (27–34)	1 (0–1)	749 (665–834)	13 (10–15)
Costa Rica	2025	31 (28–35)	1 (0–1)	764 (667–861)	13 (10–16)
Costa Rica	2026	32 (28–37)	1 (0–1)	779 (671–888)	13 (9–16)
Costa Rica	2027	33 (28–38)	1 (0–1)	794 (675–913)	13 (9–17)
Costa Rica	2028	33 (28–39)	1 (0–1)	809 (681–938)	13 (9–17)
Costa Rica	2029	34 (28–40)	1 (0–1)	824 (687–961)	13 (8–17)
Costa Rica	2030	35 (29–41)	1 (0–1)	839 (693–985)	13 (8–17)
Costa Rica	2031	35 (29–41)	1 (0–1)	854 (700–1007)	13 (8–18)
Costa Rica	2032	36 (29–42)	1 (0–1)	869 (708–1030)	13 (7–18)
Costa Rica	2033	36 (30–43)	1 (0–1)	884 (716–1052)	13 (7–18)
Costa Rica	2034	37 (30–44)	1 (0–1)	899 (724–1074)	13 (7–18)
Costa Rica	2035	38 (30–45)	1 (0–1)	913 (732–1095)	13 (7–19)
Costa Rica	2036	38 (31–46)	1 (0–1)	928 (740–1116)	13 (7–19)
Croatia	2022	11 (11–12)	0 (0–0)	350 (345–355)	3 (3–3)
Croatia	2023	12 (11–12)	0 (0–0)	359 (349–369)	3 (2–3)
Croatia	2024	12 (11–12)	0 (0–0)	368 (352–383)	3 (2–3)
Croatia	2025	12 (12–13)	0 (0–0)	376 (355–397)	3 (2–3)
Croatia	2026	13 (12–13)	0 (0–0)	384 (359–410)	3 (2–3)
Croatia	2027	13 (12–14)	0 (0–0)	393 (362–423)	3 (2–3)
Croatia	2028	13 (12–14)	0 (0–0)	401 (366–435)	3 (2–3)
Croatia	2029	13 (12–15)	0 (0–0)	409 (370–448)	3 (2–3)
Croatia	2030	14 (12–15)	0 (0–0)	417 (374–459)	3 (2–3)
Croatia	2031	14 (12–15)	0 (0–0)	424 (378–471)	3 (2–3)
Croatia	2032	14 (12–16)	0 (0–0)	432 (382–483)	3 (2–3)
Croatia	2033	14 (13–16)	0 (0–0)	440 (387–494)	3 (2–3)
Croatia	2034	15 (13–16)	0 (0–0)	448 (391–505)	3 (2–3)

Croatia	2035	15 (13–17)	0 (0–0)	456 (396–516)	3 (2–3)
Croatia	2036	15 (13–17)	0 (0–0)	464 (401–527)	3 (2–3)
Cuba	2022	141 (132–150)	2 (1–2)	3142 (2911–3372)	40 (36–43)
Cuba	2023	141 (128–154)	2 (1–2)	3142 (2816–3468)	40 (37–44)
Cuba	2024	141 (125–157)	2 (1–2)	3142 (2742–3541)	41 (37–45)
Cuba	2025	141 (123–159)	2 (1–2)	3142 (2680–3603)	41 (37–45)
Cuba	2026	141 (121–161)	2 (1–2)	3142 (2626–3658)	41 (37–46)
Cuba	2027	141 (119–163)	2 (1–2)	3142 (2577–3707)	42 (37–46)
Cuba	2028	141 (117–165)	2 (1–2)	3142 (2532–3752)	42 (37–46)
Cuba	2029	141 (116–166)	2 (1–2)	3142 (2489–3794)	42 (38–46)
Cuba	2030	141 (114–168)	2 (1–2)	3142 (2450–3834)	42 (38–46)
Cuba	2031	141 (113–169)	2 (1–2)	3142 (2412–3871)	42 (38–46)
Cuba	2032	141 (111–171)	2 (1–2)	3142 (2377–3907)	42 (38–46)
Cuba	2033	141 (110–172)	2 (1–2)	3142 (2343–3941)	42 (38–46)
Cuba	2034	141 (109–173)	2 (1–2)	3142 (2310–3973)	42 (38–46)
Cuba	2035	141 (107–175)	2 (1–2)	3142 (2279–4005)	42 (38–46)
Cuba	2036	141 (106–176)	2 (1–2)	3142 (2249–4035)	42 (38–46)
Cyprus	2022	152 (144–161)	1 (1–1)	3980 (3769–4191)	20 (19–22)
Cyprus	2023	152 (138–167)	1 (1–1)	4022 (3723–4320)	20 (18–23)
Cyprus	2024	152 (133–172)	1 (1–1)	4063 (3697–4429)	20 (17–24)
Cyprus	2025	152 (129–176)	1 (1–1)	4105 (3682–4527)	20 (16–25)
Cyprus	2026	152 (125–180)	1 (1–1)	4146 (3674–4618)	20 (15–26)
Cyprus	2027	152 (122–183)	1 (1–1)	4188 (3670–4705)	20 (15–26)
Cyprus	2028	152 (119–186)	1 (0–1)	4229 (3670–4788)	20 (14–27)
Cyprus	2029	152 (116–189)	1 (0–1)	4271 (3673–4868)	20 (14–27)
Cyprus	2030	152 (113–191)	1 (0–1)	4312 (3678–4946)	20 (13–28)
Cyprus	2031	152 (111–194)	1 (0–1)	4354 (3686–5021)	20 (13–28)
Cyprus	2032	152 (109–196)	1 (0–1)	4395 (3695–5095)	20 (12–29)
Cyprus	2033	152 (107–198)	1 (0–1)	4436 (3705–5168)	20 (12–29)
Cyprus	2034	152 (105–200)	1 (0–1)	4478 (3717–5239)	20 (11–29)
Cyprus	2035	152 (103–202)	1 (0–1)	4519 (3729–5310)	20 (11–30)
Cyprus	2036	152 (101–204)	1 (0–1)	4561 (3743–5379)	20 (11–30)
Czechia	2022	22 (21–23)	1 (1–1)	503 (486–520)	24 (23–25)

Czechia	2023	22 (20–23)	1 (1–1)	492 (461–523)	23 (21–25)
Czechia	2024	21 (19–23)	1 (1–1)	472 (420–524)	21 (18–24)
Czechia	2025	21 (18–24)	1 (1–1)	463 (390–536)	20 (16–24)
Czechia	2026	21 (17–24)	1 (1–1)	449 (353–546)	18 (13–24)
Czechia	2027	20 (16–25)	1 (0–1)	443 (324–562)	17 (10–24)
Czechia	2028	20 (15–25)	1 (0–1)	434 (291–576)	15 (7–24)
Czechia	2029	20 (14–26)	1 (0–1)	429 (264–594)	13 (3–24)
Czechia	2030	20 (13–26)	1 (0–1)	423 (236–611)	12 (-0–24)
Czechia	2031	20 (12–27)	0 (-0–1)	420 (212–629)	10 (-4–24)
Czechia	2032	20 (11–28)	0 (-0–1)	416 (187–646)	9 (-7–25)
Czechia	2033	19 (10–28)	0 (-0–1)	414 (165–664)	7 (-11–25)
Czechia	2034	19 (10–29)	0 (-1–1)	412 (143–681)	6 (-14–26)
Czechia	2035	19 (9–30)	0 (-1–1)	411 (123–698)	4 (-18–26)
Czechia	2036	19 (8–30)	0 (-1–1)	409 (103–714)	2 (-22–27)
C么te d'Ivoire	2022	381 (371–392)	2 (2–2)	9021 (8702–9340)	46 (44–48)
C么te d'Ivoire	2023	384 (368–399)	2 (2–2)	9021 (8570–9472)	45 (43–47)
C么te d'Ivoire	2024	386 (367–404)	2 (2–2)	9021 (8469–9573)	45 (42–47)
C么te d'Ivoire	2025	388 (366–409)	2 (2–2)	9021 (8383–9658)	44 (42–47)
C么te d'Ivoire	2026	390 (366–414)	2 (2–2)	9021 (8308–9734)	44 (41–46)
C么te d'Ivoire	2027	392 (365–419)	2 (2–2)	9021 (8240–9802)	43 (41–46)
C么te d'Ivoire	2028	394 (365–423)	2 (2–2)	9021 (8178–9864)	43 (40–46)
C么te d'Ivoire	2029	396 (365–427)	2 (2–2)	9021 (8119–9923)	42 (39–45)
C么te d'Ivoire	2030	398 (366–431)	2 (2–2)	9021 (8065–9977)	42 (39–45)
C么te d'Ivoire	2031	400 (366–435)	2 (2–2)	9021 (8013–10029)	41 (38–45)
C么te d'Ivoire	2032	402 (366–439)	2 (2–2)	9021 (7964–10078)	41 (38–44)
C么te d'Ivoire	2033	405 (367–442)	2 (1–2)	9021 (7917–10125)	40 (37–44)
C么te d'Ivoire	2034	407 (368–446)	2 (1–2)	9021 (7872–10170)	40 (36–44)
C么te d'Ivoire	2035	409 (368–449)	2 (1–2)	9021 (7828–10214)	40 (36–43)
C么te d'Ivoire	2036	411 (369–453)	2 (1–2)	9021 (7786–10256)	39 (35–43)
Democratic People's Republic of Korea	2022	195 (194–196)	1 (1–1)	5967 (5952–5982)	17 (17–17)
Democratic People's Republic of Korea	2023	198 (197–200)	1 (1–1)	6055 (6023–6086)	17 (16–17)
Democratic People's Republic of Korea	2024	202 (199–204)	1 (1–1)	6134 (6078–6190)	17 (16–17)
Democratic People's Republic of Korea	2025	205 (202–208)	1 (1–1)	6210 (6126–6293)	17 (16–17)

Democratic People's Republic of Korea	2026	208 (204–212)	1 (1–1)	6287 (6176–6397)	17 (16–17)
Democratic People's Republic of Korea	2027	211 (206–216)	1 (1–1)	6368 (6233–6503)	17 (16–17)
Democratic People's Republic of Korea	2028	214 (208–219)	1 (1–1)	6455 (6302–6608)	17 (16–17)
Democratic People's Republic of Korea	2029	217 (211–223)	1 (1–1)	6547 (6380–6714)	16 (15–17)
Democratic People's Republic of Korea	2030	219 (213–226)	1 (0–1)	6641 (6464–6819)	16 (15–18)
Democratic People's Republic of Korea	2031	222 (216–229)	1 (0–1)	6737 (6552–6921)	16 (15–18)
Democratic People's Republic of Korea	2032	225 (218–232)	1 (0–1)	6831 (6640–7022)	16 (15–18)
Democratic People's Republic of Korea	2033	228 (221–235)	1 (0–1)	6923 (6726–7120)	16 (15–18)
Democratic People's Republic of Korea	2034	231 (224–238)	1 (0–1)	7012 (6809–7216)	16 (14–18)
Democratic People's Republic of Korea	2035	234 (227–242)	1 (0–1)	7100 (6890–7310)	16 (14–19)
Democratic People's Republic of Korea	2036	238 (230–245)	1 (0–1)	7187 (6969–7404)	16 (14–19)
Democratic Republic of the Congo	2022	46 (46–47)	0 (0–0)	1427 (1408–1445)	3 (3–3)
Democratic Republic of the Congo	2023	48 (47–49)	0 (0–0)	1481 (1444–1518)	3 (3–3)
Democratic Republic of the Congo	2024	50 (47–52)	0 (0–0)	1536 (1476–1596)	3 (3–3)
Democratic Republic of the Congo	2025	51 (48–55)	0 (0–0)	1591 (1505–1677)	3 (3–4)
Democratic Republic of the Congo	2026	53 (48–58)	0 (0–0)	1646 (1532–1760)	3 (3–4)
Democratic Republic of the Congo	2027	55 (49–61)	0 (0–0)	1701 (1555–1847)	3 (3–4)
Democratic Republic of the Congo	2028	56 (49–64)	0 (0–0)	1756 (1577–1935)	3 (3–4)
Democratic Republic of the Congo	2029	58 (49–67)	0 (0–0)	1811 (1596–2026)	3 (3–4)
Democratic Republic of the Congo	2030	59 (49–70)	0 (0–0)	1866 (1612–2119)	3 (3–4)
Democratic Republic of the Congo	2031	61 (49–73)	0 (0–0)	1921 (1627–2214)	3 (2–4)
Democratic Republic of the Congo	2032	63 (49–77)	0 (0–0)	1976 (1640–2311)	4 (2–5)
Democratic Republic of the Congo	2033	64 (48–80)	0 (0–0)	2031 (1651–2410)	4 (2–5)
Democratic Republic of the Congo	2034	66 (48–84)	0 (0–0)	2086 (1661–2510)	4 (2–5)
Democratic Republic of the Congo	2035	68 (48–88)	0 (0–0)	2140 (1668–2613)	4 (2–5)
Democratic Republic of the Congo	2036	69 (47–91)	0 (0–0)	2195 (1674–2716)	4 (2–5)
Denmark	2022	226 (211–241)	2 (2–2)	4543 (4230–4856)	40 (36–44)
Denmark	2023	226 (199–252)	2 (2–2)	4514 (3974–5055)	40 (33–47)
Denmark	2024	226 (192–260)	2 (1–2)	4488 (3784–5192)	40 (31–49)
Denmark	2025	226 (185–267)	2 (1–2)	4467 (3661–5274)	40 (29–51)
Denmark	2026	226 (180–272)	2 (1–2)	4452 (3589–5315)	40 (28–53)
Denmark	2027	226 (175–277)	2 (1–2)	4442 (3551–5332)	40 (26–54)
Denmark	2028	226 (170–281)	2 (1–2)	4436 (3535–5337)	40 (25–55)

Denmark	2029	226 (166–286)	2 (1–3)	4433 (3529–5337)	40 (24–56)
Denmark	2030	226 (162–289)	2 (1–3)	4432 (3527–5337)	40 (23–57)
Denmark	2031	226 (159–293)	2 (1–3)	4433 (3528–5337)	40 (22–58)
Denmark	2032	226 (155–296)	2 (1–3)	4434 (3529–5339)	40 (21–59)
Denmark	2033	226 (152–300)	2 (1–3)	4435 (3530–5340)	40 (20–60)
Denmark	2034	226 (149–303)	2 (1–3)	4437 (3531–5342)	40 (19–61)
Denmark	2035	226 (146–306)	2 (1–3)	4438 (3532–5343)	40 (18–62)
Denmark	2036	226 (143–309)	2 (1–3)	4438 (3533–5344)	40 (17–63)
Djibouti	2022	2 (2–2)	0 (0–0)	70 (69–71)	9 (9–10)
Djibouti	2023	3 (2–3)	0 (0–0)	73 (71–74)	9 (9–10)
Djibouti	2024	3 (3–3)	0 (0–0)	75 (74–77)	9 (9–10)
Djibouti	2025	3 (3–3)	0 (0–0)	78 (76–80)	9 (9–10)
Djibouti	2026	3 (3–3)	0 (0–0)	81 (78–84)	9 (9–10)
Djibouti	2027	3 (3–3)	0 (0–0)	83 (80–87)	9 (9–10)
Djibouti	2028	3 (3–3)	0 (0–0)	86 (81–91)	9 (8–10)
Djibouti	2029	3 (3–3)	0 (0–0)	89 (83–95)	9 (8–10)
Djibouti	2030	3 (3–3)	0 (0–0)	92 (85–98)	9 (8–11)
Djibouti	2031	3 (3–4)	0 (0–0)	94 (87–102)	9 (8–11)
Djibouti	2032	3 (3–4)	0 (0–0)	97 (88–106)	9 (8–11)
Djibouti	2033	3 (3–4)	0 (0–0)	100 (90–110)	9 (8–11)
Djibouti	2034	4 (3–4)	0 (0–0)	103 (92–114)	9 (7–11)
Djibouti	2035	4 (3–4)	0 (0–0)	105 (93–117)	9 (7–12)
Djibouti	2036	4 (3–4)	0 (0–0)	108 (95–121)	9 (7–12)
Dominica	2022	1 (1–1)	1 (1–1)	15 (15–15)	17 (16–17)
Dominica	2023	1 (1–1)	1 (1–1)	15 (15–15)	17 (16–17)
Dominica	2024	1 (1–1)	1 (1–1)	15 (15–16)	17 (16–17)
Dominica	2025	1 (1–1)	1 (1–1)	15 (15–16)	17 (16–18)
Dominica	2026	1 (1–1)	1 (1–1)	16 (15–16)	17 (16–18)
Dominica	2027	1 (1–1)	1 (1–1)	16 (15–17)	17 (16–18)
Dominica	2028	1 (1–1)	1 (1–1)	16 (15–17)	17 (16–18)
Dominica	2029	1 (1–1)	1 (1–1)	16 (15–18)	17 (16–19)
Dominica	2030	1 (1–1)	1 (1–1)	17 (15–18)	17 (16–19)
Dominica	2031	1 (1–1)	1 (1–1)	17 (15–19)	18 (16–19)

Dominica	2032	1 (1–1)	1 (1–1)	17 (15–19)	18 (16–19)
Dominica	2033	1 (1–1)	1 (1–1)	17 (15–20)	18 (16–20)
Dominica	2034	1 (1–1)	1 (1–1)	17 (14–20)	18 (16–20)
Dominica	2035	1 (1–1)	1 (1–1)	18 (14–21)	18 (16–20)
Dominica	2036	1 (1–1)	1 (1–1)	18 (14–22)	18 (16–20)
Dominican Republic	2022	80 (77–84)	1 (1–1)	1939 (1868–2009)	19 (18–20)
Dominican Republic	2023	82 (78–87)	1 (1–1)	1984 (1885–2084)	19 (18–20)
Dominican Republic	2024	84 (79–90)	1 (1–1)	2030 (1908–2152)	19 (18–21)
Dominican Republic	2025	86 (80–92)	1 (1–1)	2076 (1935–2217)	20 (18–21)
Dominican Republic	2026	88 (81–95)	1 (1–1)	2122 (1964–2280)	20 (18–22)
Dominican Republic	2027	90 (82–98)	1 (1–1)	2168 (1995–2340)	20 (18–22)
Dominican Republic	2028	92 (83–100)	1 (1–1)	2213 (2027–2400)	20 (18–22)
Dominican Republic	2029	94 (85–103)	1 (1–1)	2259 (2060–2459)	20 (18–23)
Dominican Republic	2030	96 (86–105)	1 (1–1)	2305 (2094–2517)	21 (18–23)
Dominican Republic	2031	98 (88–108)	1 (1–1)	2351 (2128–2574)	21 (18–24)
Dominican Republic	2032	100 (89–110)	1 (1–1)	2397 (2163–2631)	21 (18–24)
Dominican Republic	2033	101 (90–113)	1 (1–1)	2443 (2198–2687)	21 (18–24)
Dominican Republic	2034	103 (92–115)	1 (1–1)	2488 (2234–2743)	21 (18–24)
Dominican Republic	2035	105 (93–117)	1 (1–1)	2534 (2270–2798)	22 (18–25)
Dominican Republic	2036	107 (95–120)	1 (1–1)	2580 (2307–2853)	22 (18–25)
Ecuador	2022	59 (55–62)	0 (0–0)	1440 (1343–1538)	9 (8–10)
Ecuador	2023	60 (54–66)	0 (0–0)	1467 (1305–1629)	9 (7–11)
Ecuador	2024	61 (53–69)	0 (0–0)	1496 (1282–1710)	9 (7–12)
Ecuador	2025	62 (52–72)	0 (0–0)	1524 (1267–1782)	10 (7–12)
Ecuador	2026	63 (52–74)	0 (0–1)	1553 (1258–1848)	10 (7–12)
Ecuador	2027	64 (52–77)	0 (0–1)	1582 (1254–1911)	10 (7–12)
Ecuador	2028	66 (52–79)	0 (0–1)	1611 (1253–1970)	10 (7–13)
Ecuador	2029	67 (52–82)	0 (0–1)	1641 (1254–2028)	10 (7–13)
Ecuador	2030	68 (52–84)	0 (0–1)	1670 (1256–2083)	10 (7–13)
Ecuador	2031	69 (52–86)	0 (0–1)	1699 (1261–2136)	10 (7–13)
Ecuador	2032	70 (53–88)	0 (0–1)	1728 (1267–2189)	10 (7–13)
Ecuador	2033	72 (53–90)	0 (0–1)	1757 (1274–2240)	10 (7–13)
Ecuador	2034	73 (54–92)	0 (0–1)	1786 (1281–2290)	10 (7–13)

Ecuador	2035	74 (54–94)	0 (0–1)	1815 (1290–2340)	10 (7–13)
Ecuador	2036	75 (54–96)	0 (0–1)	1844 (1300–2388)	10 (7–13)
Egypt	2022	571 (558–584)	1 (1–1)	16394 (16005–16783)	23 (22–23)
Egypt	2023	589 (562–616)	1 (1–1)	16895 (16115–17676)	23 (22–24)
Egypt	2024	606 (570–642)	1 (1–1)	17370 (16326–18413)	23 (22–25)
Egypt	2025	622 (576–669)	1 (1–1)	17844 (16506–19182)	24 (22–26)
Egypt	2026	639 (581–698)	1 (1–1)	18318 (16659–19977)	24 (22–27)
Egypt	2027	656 (585–727)	1 (1–1)	18793 (16787–20798)	25 (22–28)
Egypt	2028	673 (588–757)	1 (1–1)	19267 (16893–21641)	25 (22–28)
Egypt	2029	689 (590–788)	1 (1–1)	19741 (16977–22506)	25 (22–29)
Egypt	2030	706 (592–820)	1 (1–1)	20215 (17041–23390)	26 (22–30)
Egypt	2031	723 (593–852)	1 (1–1)	20690 (17086–24293)	26 (23–30)
Egypt	2032	739 (593–885)	1 (1–1)	21164 (17114–25215)	27 (23–31)
Egypt	2033	756 (593–919)	1 (1–1)	21638 (17123–26153)	27 (23–31)
Egypt	2034	773 (592–954)	1 (1–1)	22113 (17117–27109)	28 (23–32)
Egypt	2035	789 (590–989)	1 (1–1)	22587 (17094–28080)	28 (23–33)
Egypt	2036	806 (588–1024)	1 (1–1)	23061 (17055–29068)	28 (24–33)
El Salvador	2022	17 (16–18)	0 (0–0)	474 (450–497)	8 (7–8)
El Salvador	2023	17 (16–19)	0 (0–0)	484 (452–517)	8 (7–9)
El Salvador	2024	18 (17–19)	0 (0–0)	495 (455–536)	8 (7–9)
El Salvador	2025	18 (17–20)	0 (0–0)	506 (460–553)	8 (7–9)
El Salvador	2026	19 (17–20)	0 (0–0)	517 (466–569)	8 (7–9)
El Salvador	2027	19 (17–21)	0 (0–0)	528 (472–585)	8 (7–10)
El Salvador	2028	20 (17–22)	0 (0–0)	539 (478–601)	9 (7–10)
El Salvador	2029	20 (18–22)	0 (0–0)	550 (485–616)	9 (7–10)
El Salvador	2030	20 (18–23)	0 (0–0)	561 (492–631)	9 (7–10)
El Salvador	2031	21 (18–23)	0 (0–0)	572 (499–646)	9 (7–11)
El Salvador	2032	21 (18–24)	0 (0–0)	583 (506–660)	9 (7–11)
El Salvador	2033	22 (19–24)	0 (0–0)	594 (514–675)	9 (7–11)
El Salvador	2034	22 (19–25)	0 (0–0)	605 (522–689)	9 (7–11)
El Salvador	2035	22 (19–25)	0 (0–0)	616 (529–703)	9 (8–11)
El Salvador	2036	23 (20–26)	0 (0–0)	627 (537–717)	10 (8–12)
Equatorial Guinea	2022	2 (2–2)	0 (0–0)	54 (54–55)	9 (9–9)

Equatorial Guinea	2023	2 (2–2)	0 (0–0)	56 (54–58)	9 (8–9)
Equatorial Guinea	2024	2 (2–2)	0 (0–0)	58 (55–62)	9 (8–10)
Equatorial Guinea	2025	2 (2–2)	0 (0–0)	61 (56–65)	9 (8–10)
Equatorial Guinea	2026	2 (2–2)	0 (0–0)	63 (56–69)	9 (7–10)
Equatorial Guinea	2027	2 (2–2)	0 (0–0)	65 (56–73)	9 (7–11)
Equatorial Guinea	2028	2 (2–2)	0 (0–0)	67 (56–77)	9 (7–11)
Equatorial Guinea	2029	2 (2–3)	0 (0–0)	69 (56–81)	9 (6–12)
Equatorial Guinea	2030	2 (2–3)	0 (0–0)	71 (56–86)	9 (6–12)
Equatorial Guinea	2031	2 (2–3)	0 (0–0)	73 (56–90)	9 (5–13)
Equatorial Guinea	2032	2 (2–3)	0 (0–0)	75 (55–95)	9 (4–13)
Equatorial Guinea	2033	2 (2–3)	0 (0–0)	77 (55–99)	9 (4–14)
Equatorial Guinea	2034	2 (2–3)	0 (0–1)	79 (54–104)	9 (3–15)
Equatorial Guinea	2035	2 (2–3)	0 (0–1)	81 (53–109)	9 (3–15)
Equatorial Guinea	2036	3 (2–4)	0 (0–1)	83 (52–114)	9 (2–16)
Eritrea	2022	3 (3–3)	0 (0–0)	87 (85–89)	2 (2–2)
Eritrea	2023	3 (3–3)	0 (0–0)	90 (86–95)	2 (2–2)
Eritrea	2024	3 (3–3)	0 (0–0)	93 (86–100)	2 (2–3)
Eritrea	2025	3 (3–3)	0 (0–0)	97 (88–106)	2 (2–3)
Eritrea	2026	3 (3–3)	0 (0–0)	100 (90–111)	2 (2–3)
Eritrea	2027	3 (3–3)	0 (0–0)	104 (91–116)	2 (2–3)
Eritrea	2028	3 (3–4)	0 (0–0)	107 (92–122)	2 (2–3)
Eritrea	2029	3 (3–4)	0 (0–0)	110 (92–128)	2 (2–3)
Eritrea	2030	3 (3–4)	0 (0–0)	114 (92–135)	2 (2–3)
Eritrea	2031	3 (3–4)	0 (0–0)	117 (93–141)	2 (2–3)
Eritrea	2032	4 (3–4)	0 (0–0)	121 (94–147)	2 (2–3)
Eritrea	2033	4 (3–4)	0 (0–0)	124 (94–154)	2 (2–3)
Eritrea	2034	4 (3–5)	0 (0–0)	127 (94–161)	2 (2–3)
Eritrea	2035	4 (3–5)	0 (0–0)	131 (94–167)	2 (1–3)
Eritrea	2036	4 (3–5)	0 (0–0)	134 (93–174)	2 (1–3)
Estonia	2022	35 (31–38)	1 (1–2)	875 (780–971)	38 (33–43)
Estonia	2023	35 (30–40)	1 (1–2)	884 (770–998)	38 (31–45)
Estonia	2024	35 (29–41)	1 (1–2)	890 (769–1011)	38 (29–46)
Estonia	2025	35 (28–42)	1 (1–2)	894 (770–1017)	38 (28–48)

Estonia	2026	35 (27–43)	1 (1–2)	896 (772–1021)	38 (26–49)
Estonia	2027	35 (26–43)	1 (1–2)	898 (773–1023)	38 (25–50)
Estonia	2028	35 (26–44)	1 (1–2)	899 (773–1024)	38 (24–51)
Estonia	2029	35 (25–45)	2 (1–2)	899 (774–1025)	38 (24–52)
Estonia	2030	35 (25–45)	2 (1–2)	900 (774–1025)	38 (23–53)
Estonia	2031	35 (24–46)	2 (1–2)	900 (775–1025)	38 (22–54)
Estonia	2032	35 (23–46)	2 (1–2)	900 (775–1025)	38 (21–54)
Estonia	2033	35 (23–47)	2 (1–2)	900 (775–1026)	38 (20–55)
Estonia	2034	35 (22–47)	2 (1–2)	900 (775–1026)	38 (20–56)
Estonia	2035	35 (22–48)	2 (1–2)	900 (775–1026)	38 (19–56)
Estonia	2036	35 (22–48)	2 (1–2)	900 (775–1026)	38 (18–57)
Eswatini	2022	3 (3–3)	0 (0–0)	74 (72–75)	12 (12–12)
Eswatini	2023	2 (2–3)	0 (0–0)	72 (68–76)	12 (11–12)
Eswatini	2024	2 (2–3)	0 (0–0)	71 (64–78)	12 (10–13)
Eswatini	2025	2 (2–3)	0 (0–0)	69 (58–80)	12 (10–13)
Eswatini	2026	2 (2–3)	0 (0–1)	67 (52–83)	12 (9–14)
Eswatini	2027	2 (1–3)	0 (0–1)	65 (45–86)	12 (9–14)
Eswatini	2028	2 (1–3)	0 (0–1)	64 (38–90)	12 (9–15)
Eswatini	2029	2 (1–3)	0 (0–1)	62 (30–94)	12 (9–15)
Eswatini	2030	2 (0–3)	0 (0–1)	60 (22–99)	12 (8–16)
Eswatini	2031	1 (-0–3)	0 (0–1)	59 (13–104)	12 (8–16)
Eswatini	2032	1 (-1–3)	0 (0–1)	57 (4–109)	12 (8–16)
Eswatini	2033	1 (-1–4)	0 (0–1)	55 (-5–115)	13 (9–17)
Eswatini	2034	1 (-1–4)	0 (0–1)	53 (-14–121)	13 (9–17)
Eswatini	2035	1 (-2–4)	0 (0–1)	52 (-24–127)	13 (9–17)
Eswatini	2036	1 (-2–4)	0 (0–1)	50 (-34–134)	13 (9–17)
Ethiopia	2022	16 (16–16)	0 (0–0)	443 (439–447)	1 (1–1)
Ethiopia	2023	17 (16–17)	0 (0–0)	459 (450–468)	1 (1–1)
Ethiopia	2024	17 (16–18)	0 (0–0)	475 (460–490)	1 (1–1)
Ethiopia	2025	17 (17–18)	0 (0–0)	491 (469–513)	1 (1–1)
Ethiopia	2026	18 (17–19)	0 (0–0)	507 (478–536)	1 (1–1)
Ethiopia	2027	18 (17–20)	0 (0–0)	523 (485–561)	1 (1–1)
Ethiopia	2028	19 (17–21)	0 (0–0)	539 (492–586)	1 (1–1)

Ethiopia	2029	19 (17–22)	0 (0–0)	555 (498–612)	1 (1–1)
Ethiopia	2030	20 (17–22)	0 (0–0)	571 (504–638)	1 (1–1)
Ethiopia	2031	20 (17–23)	0 (0–0)	587 (509–665)	1 (1–1)
Ethiopia	2032	21 (17–24)	0 (0–0)	603 (514–692)	1 (1–1)
Ethiopia	2033	21 (17–25)	0 (0–0)	619 (518–720)	1 (1–1)
Ethiopia	2034	22 (17–26)	0 (0–0)	635 (522–749)	1 (1–1)
Ethiopia	2035	22 (17–27)	0 (0–0)	651 (525–777)	1 (1–1)
Ethiopia	2036	22 (17–28)	0 (0–0)	667 (528–807)	1 (1–1)
Fiji	2022	4 (3–4)	0 (0–0)	101 (99–104)	11 (11–12)
Fiji	2023	4 (3–4)	0 (0–0)	104 (100–108)	11 (11–12)
Fiji	2024	4 (4–4)	0 (0–0)	106 (101–111)	12 (11–12)
Fiji	2025	4 (4–4)	0 (0–0)	108 (102–115)	12 (11–13)
Fiji	2026	4 (4–4)	0 (0–0)	111 (103–118)	12 (10–13)
Fiji	2027	4 (4–4)	0 (0–0)	113 (104–122)	12 (10–13)
Fiji	2028	4 (4–4)	0 (0–0)	116 (105–126)	12 (10–13)
Fiji	2029	4 (4–5)	0 (0–0)	118 (106–130)	12 (10–14)
Fiji	2030	4 (4–5)	0 (0–0)	120 (107–133)	12 (10–14)
Fiji	2031	4 (4–5)	0 (0–0)	123 (108–137)	12 (10–14)
Fiji	2032	4 (4–5)	0 (0–0)	125 (109–141)	12 (10–14)
Fiji	2033	5 (4–5)	0 (0–0)	127 (110–145)	12 (10–15)
Fiji	2034	5 (4–5)	0 (0–0)	130 (111–149)	12 (10–15)
Fiji	2035	5 (4–5)	0 (0–0)	132 (111–153)	12 (9–15)
Fiji	2036	5 (4–6)	0 (0–0)	135 (112–157)	12 (9–15)
Finland	2022	138 (133–143)	1 (1–1)	3138 (3011–3266)	29 (28–31)
Finland	2023	140 (133–146)	1 (1–1)	3161 (2981–3342)	29 (27–31)
Finland	2024	141 (133–149)	1 (1–1)	3184 (2963–3405)	29 (26–31)
Finland	2025	142 (133–152)	1 (1–1)	3207 (2952–3462)	28 (25–31)
Finland	2026	144 (133–155)	1 (1–1)	3230 (2945–3516)	28 (25–32)
Finland	2027	145 (134–157)	1 (1–1)	3253 (2941–3566)	28 (24–32)
Finland	2028	147 (134–159)	1 (1–1)	3276 (2939–3614)	28 (24–32)
Finland	2029	148 (135–162)	1 (1–1)	3299 (2939–3660)	28 (23–32)
Finland	2030	150 (135–164)	1 (1–1)	3322 (2940–3705)	27 (23–32)
Finland	2031	151 (136–166)	1 (1–1)	3345 (2942–3749)	27 (23–32)

Finland	2032	152 (137–168)	1 (1–1)	3368 (2945–3792)	27 (22–32)
Finland	2033	154 (137–170)	1 (1–1)	3391 (2950–3833)	27 (22–32)
Finland	2034	155 (138–172)	1 (1–1)	3415 (2955–3874)	27 (21–32)
Finland	2035	157 (139–175)	1 (1–1)	3438 (2960–3915)	26 (21–32)
Finland	2036	158 (140–177)	1 (1–1)	3461 (2967–3955)	26 (20–32)
France	2022	1676 (1646–1705)	1 (1–1)	38827 (38060–39594)	33 (32–34)
France	2023	1634 (1571–1697)	1 (1–1)	37790 (36155–39424)	32 (31–34)
France	2024	1596 (1494–1697)	1 (1–1)	36875 (34260–39489)	32 (29–34)
France	2025	1562 (1418–1705)	1 (1–1)	36068 (32404–39732)	31 (28–34)
France	2026	1531 (1343–1718)	1 (1–1)	35356 (30601–40112)	30 (26–35)
France	2027	1503 (1270–1736)	1 (1–1)	34729 (28859–40598)	30 (25–35)
France	2028	1478 (1199–1757)	1 (1–1)	34175 (27182–41168)	30 (24–36)
France	2029	1455 (1130–1781)	1 (1–1)	33687 (25569–41804)	30 (23–36)
France	2030	1435 (1062–1807)	1 (1–1)	33256 (24022–42489)	29 (22–37)
France	2031	1416 (997–1836)	1 (1–1)	32876 (22537–43214)	29 (21–37)
France	2032	1400 (934–1866)	1 (1–1)	32540 (21113–43968)	29 (20–38)
France	2033	1385 (873–1897)	1 (1–1)	32245 (19747–44743)	29 (20–38)
France	2034	1371 (813–1929)	1 (1–1)	31984 (18435–45533)	29 (19–39)
France	2035	1359 (756–1962)	1 (1–1)	31754 (17175–46332)	29 (18–40)
France	2036	1348 (701–1996)	1 (1–2)	31551 (15964–47137)	29 (17–40)
Gabon	2022	4 (4–4)	0 (0–0)	114 (113–116)	9 (9–10)
Gabon	2023	4 (4–4)	0 (0–0)	116 (114–119)	9 (9–10)
Gabon	2024	4 (4–4)	0 (0–0)	118 (114–123)	10 (9–10)
Gabon	2025	4 (4–4)	0 (0–0)	120 (114–127)	10 (9–10)
Gabon	2026	4 (4–4)	0 (0–0)	122 (113–131)	10 (9–10)
Gabon	2027	4 (4–5)	0 (0–0)	124 (113–136)	10 (9–10)
Gabon	2028	4 (4–5)	0 (0–0)	126 (112–141)	10 (9–10)
Gabon	2029	4 (4–5)	0 (0–0)	128 (111–146)	10 (10–11)
Gabon	2030	4 (4–5)	0 (0–0)	130 (110–151)	10 (10–11)
Gabon	2031	4 (4–5)	0 (0–0)	132 (108–156)	10 (10–11)
Gabon	2032	5 (4–5)	0 (0–0)	134 (107–162)	10 (10–11)
Gabon	2033	5 (4–6)	0 (0–0)	136 (105–167)	10 (10–11)
Gabon	2034	5 (4–6)	0 (0–0)	138 (103–173)	11 (10–11)

Gabon	2035	5 (3–6)	0 (0–0)	140 (101–179)	11 (10–11)
Gabon	2036	5 (3–6)	0 (0–0)	142 (99–185)	11 (10–12)
Gambia	2022	1 (1–1)	0 (0–0)	42 (40–44)	4 (4–4)
Gambia	2023	1 (1–2)	0 (0–0)	43 (40–45)	4 (4–4)
Gambia	2024	1 (1–2)	0 (0–0)	44 (40–47)	4 (3–4)
Gambia	2025	2 (1–2)	0 (0–0)	44 (41–48)	4 (3–4)
Gambia	2026	2 (1–2)	0 (0–0)	45 (41–49)	4 (3–4)
Gambia	2027	2 (1–2)	0 (0–0)	46 (42–51)	4 (3–4)
Gambia	2028	2 (1–2)	0 (0–0)	47 (42–52)	4 (3–4)
Gambia	2029	2 (1–2)	0 (0–0)	48 (43–53)	4 (3–4)
Gambia	2030	2 (1–2)	0 (0–0)	49 (43–54)	4 (3–5)
Gambia	2031	2 (2–2)	0 (0–0)	49 (44–55)	4 (3–5)
Gambia	2032	2 (2–2)	0 (0–0)	50 (44–57)	4 (3–5)
Gambia	2033	2 (2–2)	0 (0–0)	51 (45–58)	4 (3–5)
Gambia	2034	2 (2–2)	0 (0–0)	52 (45–59)	4 (3–5)
Gambia	2035	2 (2–2)	0 (0–0)	53 (46–60)	4 (3–5)
Gambia	2036	2 (2–2)	0 (0–0)	54 (46–61)	4 (3–5)
Georgia	2022	68 (62–74)	1 (1–1)	1829 (1660–1999)	33 (30–36)
Georgia	2023	69 (60–78)	1 (1–1)	1865 (1625–2105)	34 (30–38)
Georgia	2024	71 (60–81)	1 (1–1)	1900 (1607–2194)	35 (30–40)
Georgia	2025	72 (60–84)	1 (1–1)	1936 (1597–2275)	36 (30–42)
Georgia	2026	73 (60–87)	1 (1–2)	1972 (1592–2351)	36 (30–43)
Georgia	2027	75 (60–90)	1 (1–2)	2007 (1592–2422)	37 (30–44)
Georgia	2028	76 (60–93)	1 (1–2)	2043 (1594–2491)	38 (30–46)
Georgia	2029	78 (60–95)	1 (1–2)	2078 (1599–2558)	38 (30–47)
Georgia	2030	79 (60–98)	1 (1–2)	2114 (1605–2622)	39 (30–48)
Georgia	2031	80 (61–100)	1 (1–2)	2149 (1613–2685)	40 (30–50)
Georgia	2032	82 (61–102)	1 (1–2)	2185 (1622–2747)	41 (31–51)
Georgia	2033	83 (62–105)	1 (1–2)	2220 (1633–2808)	41 (31–52)
Georgia	2034	85 (62–107)	1 (1–2)	2256 (1645–2867)	42 (31–53)
Georgia	2035	86 (63–109)	2 (1–2)	2291 (1657–2926)	43 (31–54)
Georgia	2036	87 (63–111)	2 (1–2)	2327 (1670–2984)	44 (32–55)
Germany	2022	2793 (2724–2863)	1 (1–2)	62433 (60812–64053)	37 (36–38)

Germany	2023	2823 (2724–2921)	1 (1–2)	62989 (60698–65281)	37 (36–39)
Germany	2024	2852 (2731–2972)	1 (1–2)	63546 (60740–66353)	37 (35–39)
Germany	2025	2881 (2741–3020)	1 (1–2)	64103 (60863–67344)	37 (35–39)
Germany	2026	2910 (2754–3066)	1 (1–2)	64660 (61037–68283)	37 (35–40)
Germany	2027	2939 (2768–3110)	1 (1–2)	65217 (61248–69186)	37 (35–40)
Germany	2028	2968 (2783–3152)	1 (1–2)	65774 (61487–70061)	37 (34–40)
Germany	2029	2997 (2799–3194)	1 (1–2)	66331 (61748–70913)	37 (34–40)
Germany	2030	3026 (2817–3235)	1 (1–2)	66887 (62027–71748)	37 (34–40)
Germany	2031	3055 (2834–3276)	1 (1–2)	67444 (62321–72568)	37 (34–41)
Germany	2032	3084 (2853–3316)	1 (1–2)	68001 (62627–73375)	37 (34–41)
Germany	2033	3113 (2871–3355)	1 (1–2)	68558 (62945–74171)	37 (34–41)
Germany	2034	3142 (2891–3394)	1 (1–2)	69115 (63273–74957)	37 (33–41)
Germany	2035	3171 (2910–3432)	1 (1–2)	69672 (63609–75734)	37 (33–41)
Germany	2036	3200 (2930–3471)	1 (1–2)	70229 (63953–76504)	37 (33–41)
Ghana	2022	29 (28–29)	0 (0–0)	788 (781–794)	4 (4–4)
Ghana	2023	29 (28–30)	0 (0–0)	808 (792–823)	4 (4–4)
Ghana	2024	30 (29–31)	0 (0–0)	828 (802–854)	4 (4–4)
Ghana	2025	30 (28–32)	0 (0–0)	848 (811–886)	4 (4–5)
Ghana	2026	31 (28–33)	0 (0–0)	868 (818–919)	4 (4–5)
Ghana	2027	31 (28–34)	0 (0–0)	889 (823–954)	4 (4–5)
Ghana	2028	32 (28–35)	0 (0–0)	909 (828–990)	4 (4–5)
Ghana	2029	32 (27–37)	0 (0–0)	929 (831–1027)	4 (4–5)
Ghana	2030	32 (27–38)	0 (0–0)	949 (834–1065)	4 (4–5)
Ghana	2031	33 (26–40)	0 (0–0)	970 (835–1104)	4 (3–5)
Ghana	2032	33 (26–41)	0 (0–0)	990 (836–1144)	4 (3–5)
Ghana	2033	34 (25–43)	0 (0–0)	1010 (835–1185)	4 (3–5)
Ghana	2034	34 (25–44)	0 (0–0)	1030 (834–1226)	4 (3–5)
Ghana	2035	35 (24–46)	0 (0–0)	1051 (832–1269)	4 (3–5)
Ghana	2036	35 (23–47)	0 (0–0)	1071 (829–1312)	4 (3–5)
Greece	2022	454 (437–470)	2 (2–2)	9889 (9531–10248)	50 (48–52)
Greece	2023	458 (428–489)	2 (2–2)	10060 (9446–10675)	50 (47–53)
Greece	2024	463 (424–503)	2 (2–2)	10148 (9426–10870)	50 (46–54)
Greece	2025	468 (422–515)	2 (2–2)	10236 (9420–11051)	50 (46–54)

Greece	2026	473 (420–526)	2 (2–2)	10323 (9424–11222)	50 (45–55)
Greece	2027	478 (420–537)	2 (2–2)	10411 (9435–11387)	50 (45–55)
Greece	2028	483 (419–547)	2 (2–2)	10498 (9452–11545)	50 (45–56)
Greece	2029	488 (420–557)	2 (2–2)	10586 (9473–11700)	50 (44–56)
Greece	2030	493 (420–566)	2 (2–2)	10674 (9498–11850)	50 (44–56)
Greece	2031	498 (421–575)	2 (2–2)	10761 (9526–11997)	50 (43–57)
Greece	2032	503 (422–584)	2 (2–2)	10849 (9557–12142)	50 (43–57)
Greece	2033	508 (423–593)	2 (2–2)	10937 (9590–12284)	50 (43–57)
Greece	2034	513 (424–602)	2 (2–2)	11024 (9625–12424)	50 (43–58)
Greece	2035	518 (426–610)	2 (2–2)	11112 (9662–12562)	50 (42–58)
Greece	2036	523 (427–618)	2 (2–2)	11200 (9701–12698)	50 (42–58)
Greenland	2022	2 (2–2)	3 (3–3)	71 (69–73)	81 (78–85)
Greenland	2023	2 (2–3)	3 (3–3)	69 (66–71)	79 (74–85)
Greenland	2024	2 (2–3)	3 (3–3)	69 (65–72)	77 (71–84)
Greenland	2025	2 (2–3)	3 (3–3)	69 (64–73)	76 (67–84)
Greenland	2026	2 (2–3)	3 (3–3)	68 (62–74)	74 (64–84)
Greenland	2027	2 (2–3)	3 (3–3)	68 (61–76)	72 (60–83)
Greenland	2028	3 (2–3)	3 (2–3)	68 (59–77)	70 (57–83)
Greenland	2029	3 (2–3)	3 (2–3)	68 (57–79)	68 (53–83)
Greenland	2030	3 (2–3)	3 (2–3)	68 (56–81)	66 (49–82)
Greenland	2031	3 (2–3)	3 (2–3)	68 (54–82)	64 (46–82)
Greenland	2032	3 (2–3)	3 (2–3)	68 (52–84)	62 (42–82)
Greenland	2033	3 (2–3)	3 (2–3)	68 (49–86)	60 (38–82)
Greenland	2034	3 (2–3)	3 (2–3)	68 (47–88)	58 (35–82)
Greenland	2035	3 (2–3)	3 (2–3)	68 (45–90)	56 (31–82)
Greenland	2036	3 (2–3)	2 (2–3)	68 (43–93)	54 (27–82)
Grenada	2022	1 (1–1)	1 (1–1)	25 (24–26)	20 (19–21)
Grenada	2023	1 (1–1)	1 (1–1)	25 (24–27)	20 (18–21)
Grenada	2024	1 (1–1)	1 (1–1)	26 (24–28)	20 (18–22)
Grenada	2025	1 (1–1)	1 (1–1)	26 (24–28)	20 (18–22)
Grenada	2026	1 (1–1)	1 (1–1)	27 (24–29)	20 (17–22)
Grenada	2027	1 (1–1)	1 (1–1)	27 (25–30)	20 (17–22)
Grenada	2028	1 (1–1)	1 (1–1)	28 (25–30)	20 (17–22)

Grenada	2029	1 (1–1)	1 (1–1)	28 (25–31)	20 (17–23)
Grenada	2030	1 (1–1)	1 (1–1)	29 (26–32)	20 (17–23)
Grenada	2031	1 (1–1)	1 (1–1)	29 (26–32)	20 (16–23)
Grenada	2032	1 (1–1)	1 (0–1)	29 (26–33)	20 (16–23)
Grenada	2033	1 (1–1)	1 (0–1)	30 (26–34)	20 (16–23)
Grenada	2034	1 (1–1)	1 (0–1)	30 (27–34)	20 (16–23)
Grenada	2035	1 (1–1)	1 (0–1)	31 (27–35)	20 (16–24)
Grenada	2036	1 (1–1)	1 (0–1)	31 (27–35)	20 (16–24)
Guam	2022	1 (1–1)	1 (0–1)	34 (33–36)	16 (15–17)
Guam	2023	1 (1–1)	1 (0–1)	35 (32–38)	16 (14–18)
Guam	2024	1 (1–1)	1 (0–1)	36 (32–40)	16 (14–18)
Guam	2025	1 (1–1)	1 (0–1)	36 (32–41)	16 (14–19)
Guam	2026	1 (1–1)	1 (0–1)	37 (32–42)	16 (13–19)
Guam	2027	1 (1–1)	1 (0–1)	38 (32–44)	16 (13–19)
Guam	2028	1 (1–1)	1 (0–1)	39 (33–45)	16 (13–19)
Guam	2029	1 (1–1)	1 (0–1)	39 (33–46)	16 (12–20)
Guam	2030	1 (1–2)	1 (0–1)	40 (33–47)	16 (12–20)
Guam	2031	1 (1–2)	1 (0–1)	41 (34–48)	16 (12–20)
Guam	2032	1 (1–2)	1 (0–1)	42 (34–49)	16 (12–20)
Guam	2033	1 (1–2)	1 (0–1)	42 (34–50)	16 (11–21)
Guam	2034	1 (1–2)	1 (0–1)	43 (35–51)	16 (11–21)
Guam	2035	1 (1–2)	1 (0–1)	44 (35–53)	16 (11–21)
Guam	2036	1 (1–2)	1 (0–1)	45 (35–54)	16 (11–21)
Guatemala	2022	25 (23–27)	0 (0–0)	652 (597–707)	6 (5–7)
Guatemala	2023	26 (23–29)	0 (0–0)	668 (591–745)	6 (4–7)
Guatemala	2024	27 (23–30)	0 (0–0)	684 (589–778)	6 (4–7)
Guatemala	2025	27 (23–31)	0 (0–0)	699 (590–808)	6 (4–8)
Guatemala	2026	28 (23–33)	0 (0–0)	715 (593–837)	6 (3–8)
Guatemala	2027	28 (23–34)	0 (0–0)	731 (597–864)	6 (3–8)
Guatemala	2028	29 (23–35)	0 (0–0)	747 (602–891)	6 (3–8)
Guatemala	2029	30 (24–36)	0 (0–0)	762 (608–917)	6 (3–8)
Guatemala	2030	30 (24–37)	0 (0–0)	778 (615–942)	6 (3–9)
Guatemala	2031	31 (24–38)	0 (0–0)	794 (622–966)	6 (2–9)

Guatemala	2032	32 (24–39)	0 (0–0)	810 (629–991)	6 (2–9)
Guatemala	2033	32 (25–40)	0 (0–0)	826 (637–1014)	6 (2–9)
Guatemala	2034	33 (25–41)	0 (0–0)	841 (645–1038)	6 (2–9)
Guatemala	2035	33 (25–41)	0 (0–0)	857 (653–1061)	6 (2–9)
Guatemala	2036	34 (26–42)	0 (0–0)	873 (662–1084)	6 (2–9)
Guinea	2022	7 (7–7)	0 (0–0)	202 (201–204)	3 (3–3)
Guinea	2023	7 (7–7)	0 (0–0)	206 (203–209)	3 (3–3)
Guinea	2024	7 (7–8)	0 (0–0)	210 (206–214)	3 (3–3)
Guinea	2025	7 (7–8)	0 (0–0)	214 (208–219)	3 (3–4)
Guinea	2026	7 (7–8)	0 (0–0)	218 (211–224)	3 (3–4)
Guinea	2027	8 (7–8)	0 (0–0)	222 (214–229)	3 (3–4)
Guinea	2028	8 (7–8)	0 (0–0)	226 (217–234)	3 (3–4)
Guinea	2029	8 (7–9)	0 (0–0)	230 (221–238)	3 (3–4)
Guinea	2030	8 (7–9)	0 (0–0)	234 (224–243)	3 (3–4)
Guinea	2031	8 (6–9)	0 (0–0)	237 (227–248)	3 (2–4)
Guinea	2032	8 (6–9)	0 (0–0)	241 (231–252)	3 (2–4)
Guinea	2033	8 (6–10)	0 (0–0)	245 (234–257)	3 (2–4)
Guinea	2034	8 (6–10)	0 (0–0)	249 (237–261)	3 (2–4)
Guinea	2035	8 (6–10)	0 (0–0)	253 (241–266)	3 (2–5)
Guinea	2036	8 (6–10)	0 (0–0)	257 (244–270)	3 (2–5)
Guinea-Bissau	2022	1 (1–1)	0 (0–0)	30 (30–31)	3 (3–3)
Guinea-Bissau	2023	1 (1–1)	0 (0–0)	31 (31–32)	3 (3–3)
Guinea-Bissau	2024	1 (1–1)	0 (0–0)	32 (31–33)	3 (3–3)
Guinea-Bissau	2025	1 (1–1)	0 (0–0)	32 (31–34)	3 (3–4)
Guinea-Bissau	2026	1 (1–1)	0 (0–0)	33 (31–35)	3 (3–4)
Guinea-Bissau	2027	1 (1–1)	0 (0–0)	34 (31–37)	3 (3–4)
Guinea-Bissau	2028	1 (1–1)	0 (0–0)	34 (31–38)	3 (3–4)
Guinea-Bissau	2029	1 (1–1)	0 (0–0)	35 (30–39)	3 (3–4)
Guinea-Bissau	2030	1 (1–1)	0 (0–0)	36 (30–41)	3 (3–4)
Guinea-Bissau	2031	1 (1–1)	0 (0–0)	36 (30–42)	3 (3–4)
Guinea-Bissau	2032	1 (1–1)	0 (0–0)	37 (30–44)	3 (3–4)
Guinea-Bissau	2033	1 (1–1)	0 (0–0)	37 (29–46)	3 (3–4)
Guinea-Bissau	2034	1 (1–1)	0 (0–0)	38 (29–47)	3 (2–4)

Guinea-Bissau	2035	1 (1–2)	0 (0–0)	39 (28–49)	3 (2–4)
Guinea-Bissau	2036	1 (1–2)	0 (0–0)	39 (28–51)	3 (2–4)
Guyana	2022	3 (3–3)	0 (0–0)	82 (77–87)	11 (10–12)
Guyana	2023	3 (3–3)	0 (0–0)	84 (77–91)	11 (10–13)
Guyana	2024	3 (3–3)	0 (0–0)	85 (77–94)	11 (10–13)
Guyana	2025	3 (3–3)	0 (0–0)	87 (77–97)	11 (9–13)
Guyana	2026	3 (3–3)	0 (0–0)	88 (77–100)	11 (9–14)
Guyana	2027	3 (3–3)	0 (0–0)	90 (77–102)	11 (9–14)
Guyana	2028	3 (3–3)	0 (0–0)	91 (78–105)	11 (9–14)
Guyana	2029	3 (3–4)	0 (0–1)	93 (78–107)	11 (8–14)
Guyana	2030	3 (3–4)	0 (0–1)	94 (79–109)	11 (8–14)
Guyana	2031	3 (3–4)	0 (0–1)	96 (80–112)	11 (8–14)
Guyana	2032	3 (3–4)	0 (0–1)	97 (80–114)	11 (8–15)
Guyana	2033	3 (3–4)	0 (0–1)	99 (81–116)	11 (8–15)
Guyana	2034	3 (3–4)	0 (0–1)	100 (82–118)	11 (8–15)
Guyana	2035	3 (3–4)	0 (0–1)	101 (83–120)	11 (8–15)
Guyana	2036	3 (3–4)	0 (0–1)	103 (83–122)	11 (7–15)
Haiti	2022	16 (15–16)	0 (0–0)	455 (448–462)	6 (5–6)
Haiti	2023	16 (15–16)	0 (0–0)	464 (452–476)	5 (5–6)
Haiti	2024	16 (15–17)	0 (0–0)	473 (454–493)	5 (5–6)
Haiti	2025	16 (15–17)	0 (0–0)	482 (455–509)	5 (5–6)
Haiti	2026	17 (15–18)	0 (0–0)	491 (455–527)	5 (5–6)
Haiti	2027	17 (15–18)	0 (0–0)	500 (455–546)	5 (5–6)
Haiti	2028	17 (15–19)	0 (0–0)	510 (454–565)	5 (5–6)
Haiti	2029	17 (15–20)	0 (0–0)	519 (452–585)	5 (5–6)
Haiti	2030	18 (15–20)	0 (0–0)	528 (450–606)	5 (4–6)
Haiti	2031	18 (15–21)	0 (0–0)	537 (447–627)	5 (4–6)
Haiti	2032	18 (15–22)	0 (0–0)	546 (443–649)	5 (4–6)
Haiti	2033	18 (15–22)	0 (0–0)	555 (439–671)	5 (4–6)
Haiti	2034	19 (14–23)	0 (0–0)	564 (435–694)	5 (4–7)
Haiti	2035	19 (14–24)	0 (0–0)	573 (430–717)	5 (4–7)
Haiti	2036	19 (14–24)	0 (0–0)	582 (424–741)	5 (3–7)
High SDI	2022	[illegible]950 (26671–2722[illegible]	1 (1–1)	[illegible]00858 (593987–607729[illegible]	31 (30–31)

High SDI	2023	7243 (26767–2771	1 (1–1)	605936 (594379–617493	31 (30–31)
High SDI	2024	7533 (26894–2817	1 (1–1)	611077 (595698–626456	30 (29–31)
High SDI	2025	7823 (27045–2860	1 (1–1)	616239 (597647–634831	30 (29–31)
High SDI	2026	8111 (27214–2900	1 (1–1)	621409 (600031–642787	30 (29–31)
High SDI	2027	8400 (27396–2940	1 (1–1)	626582 (602724–650440	29 (28–31)
High SDI	2028	8689 (27588–2978	1 (1–1)	631756 (605648–657864	29 (28–30)
High SDI	2029	8977 (27788–3016	1 (1–1)	636930 (608749–665111	29 (27–30)
High SDI	2030	9266 (27994–3053	1 (1–1)	642104 (611992–672217	28 (27–30)
High SDI	2031	9554 (28204–3090	1 (1–1)	647279 (615352–679206	28 (27–30)
High SDI	2032	9843 (28419–3126	1 (1–1)	652453 (618809–686098	28 (26–30)
High SDI	2033	0132 (28638–3162	1 (1–1)	657628 (622350–692906	28 (26–29)
High SDI	2034	0420 (28860–3198	1 (1–1)	662802 (625963–699641	27 (25–29)
High SDI	2035	0709 (29084–3233	1 (1–1)	667976 (629639–706313	27 (25–29)
High SDI	2036	0997 (29311–3268	1 (1–1)	673151 (633372–712929	27 (25–29)
High-middle SDI	2022	4732 (24432–2503	1 (1–1)	638545 (630635–646456	32 (31–32)
High-middle SDI	2023	5140 (24598–2568	1 (1–1)	646933 (633241–660624	32 (31–33)
High-middle SDI	2024	5557 (24790–2632	1 (1–1)	655494 (636940–674048	32 (30–33)
High-middle SDI	2025	5977 (24998–2695	1 (1–1)	664128 (641428–686827	32 (30–33)
High-middle SDI	2026	6399 (25213–2758	1 (1–1)	672791 (646479–699103	32 (30–34)
High-middle SDI	2027	6821 (25433–2821	1 (1–1)	681466 (651938–710994	32 (30–34)
High-middle SDI	2028	7244 (25653–2883	1 (1–1)	690147 (657703–722590	32 (29–34)
High-middle SDI	2029	7666 (25873–2946	1 (1–1)	698829 (663706–733952	32 (29–34)
High-middle SDI	2030	8089 (26091–3008	1 (1–1)	707512 (669897–745127	32 (29–34)
High-middle SDI	2031	8512 (26306–3071	1 (1–1)	716196 (676243–756148	32 (29–35)
High-middle SDI	2032	8935 (26518–3135	1 (1–1)	724880 (682719–767041	32 (29–35)
High-middle SDI	2033	9357 (26727–3198	1 (1–1)	733564 (689304–777823	32 (28–35)
High-middle SDI	2034	9780 (26933–3262	1 (1–1)	742247 (695984–788511	32 (28–35)
High-middle SDI	2035	0203 (27135–3327	1 (1–1)	750931 (702748–799115	32 (28–35)
High-middle SDI	2036	0626 (27333–3391	1 (1–1)	759615 (709585–809646	32 (28–35)
Honduras	2022	30 (30–31)	0 (0–0)	807 (791–822)	12 (12–12)
Honduras	2023	31 (30–32)	0 (0–1)	828 (798–859)	12 (11–13)
Honduras	2024	32 (30–34)	0 (0–1)	850 (802–898)	12 (11–13)
Honduras	2025	32 (30–35)	0 (0–1)	871 (804–939)	12 (11–13)

Honduras	2026	33 (29–37)	0 (0–1)	892 (803–982)	12 (11–13)
Honduras	2027	34 (29–39)	0 (0–1)	914 (801–1026)	12 (11–14)
Honduras	2028	35 (28–41)	1 (0–1)	935 (798–1073)	13 (11–14)
Honduras	2029	35 (28–42)	1 (0–1)	957 (792–1121)	13 (11–14)
Honduras	2030	36 (27–44)	1 (0–1)	978 (786–1171)	13 (11–14)
Honduras	2031	37 (27–46)	1 (0–1)	1000 (777–1222)	13 (11–15)
Honduras	2032	37 (26–48)	1 (0–1)	1021 (768–1275)	13 (11–15)
Honduras	2033	38 (25–51)	1 (0–1)	1042 (757–1328)	13 (11–15)
Honduras	2034	39 (25–53)	1 (0–1)	1064 (744–1384)	13 (11–15)
Honduras	2035	39 (24–55)	1 (0–1)	1085 (731–1440)	13 (11–16)
Honduras	2036	40 (23–57)	1 (0–1)	1107 (716–1497)	14 (11–16)
Hungary	2022	286 (275–298)	2 (2–2)	7404 (7065–7744)	43 (41–46)
Hungary	2023	282 (263–300)	2 (2–2)	7251 (6710–7792)	42 (39–46)
Hungary	2024	277 (251–302)	2 (2–2)	7098 (6359–7836)	41 (37–46)
Hungary	2025	272 (239–305)	2 (2–2)	6944 (6002–7886)	41 (35–46)
Hungary	2026	267 (227–307)	2 (2–2)	6791 (5637–7945)	40 (33–47)
Hungary	2027	262 (214–310)	2 (2–2)	6637 (5262–8013)	39 (30–47)
Hungary	2028	257 (201–313)	2 (2–2)	6484 (4877–8091)	38 (28–47)
Hungary	2029	252 (187–317)	2 (2–2)	6331 (4483–8179)	37 (26–47)
Hungary	2030	247 (173–321)	2 (2–2)	6177 (4079–8276)	36 (24–48)
Hungary	2031	242 (159–325)	2 (2–2)	6024 (3665–8382)	35 (21–48)
Hungary	2032	237 (145–330)	2 (2–2)	5870 (3243–8498)	34 (19–49)
Hungary	2033	232 (130–335)	2 (2–2)	5717 (2811–8623)	33 (16–49)
Hungary	2034	228 (115–340)	2 (2–2)	5564 (2371–8756)	32 (14–50)
Hungary	2035	223 (99–346)	2 (2–2)	5410 (1923–8898)	31 (11–50)
Hungary	2036	218 (84–352)	2 (2–2)	5257 (1466–9048)	30 (9–51)
Iceland	2022	7 (6–7)	1 (1–1)	148 (142–154)	26 (25–28)
Iceland	2023	7 (6–7)	1 (1–1)	149 (141–157)	26 (24–28)
Iceland	2024	7 (6–7)	1 (1–1)	150 (140–160)	26 (23–28)
Iceland	2025	7 (6–7)	1 (1–1)	151 (139–163)	25 (22–28)
Iceland	2026	7 (6–8)	1 (1–1)	152 (139–165)	25 (21–28)
Iceland	2027	7 (6–8)	1 (1–1)	153 (139–168)	24 (21–28)
Iceland	2028	7 (6–8)	1 (1–1)	154 (139–170)	24 (20–27)

Iceland	2029	7 (6–8)	1 (1–1)	155 (139–172)	23 (19–27)
Iceland	2030	7 (6–8)	1 (1–1)	156 (139–174)	23 (18–27)
Iceland	2031	7 (6–8)	1 (1–1)	157 (139–176)	22 (18–27)
Iceland	2032	7 (6–8)	1 (1–1)	158 (139–178)	22 (17–27)
Iceland	2033	7 (6–8)	1 (1–1)	159 (139–180)	21 (16–26)
Iceland	2034	7 (6–8)	1 (1–1)	161 (139–182)	21 (16–26)
Iceland	2035	7 (6–8)	1 (1–1)	162 (140–183)	20 (15–26)
Iceland	2036	7 (6–9)	1 (1–1)	163 (140–185)	20 (14–25)
India	2022	1946 (1912–1981)	0 (0–0)	50481 (49608–51354)	4 (4–4)
India	2023	1992 (1915–2069)	0 (0–0)	51628 (49676–53580)	4 (4–4)
India	2024	2037 (1908–2166)	0 (0–0)	52775 (49508–56042)	4 (4–4)
India	2025	2082 (1893–2271)	0 (0–0)	53922 (49140–58705)	4 (4–4)
India	2026	2127 (1872–2383)	0 (0–0)	55070 (48595–61545)	4 (4–4)
India	2027	2172 (1844–2501)	0 (0–0)	56217 (47888–64546)	4 (3–5)
India	2028	2218 (1810–2625)	0 (0–0)	57364 (47033–67695)	4 (3–5)
India	2029	2263 (1771–2755)	0 (0–0)	58511 (46041–70981)	4 (3–5)
India	2030	2308 (1727–2889)	0 (0–0)	59658 (44919–74398)	4 (3–5)
India	2031	2353 (1678–3029)	0 (0–0)	60806 (43674–77937)	4 (3–5)
India	2032	2398 (1624–3173)	0 (0–0)	61953 (42313–81593)	4 (3–5)
India	2033	2444 (1566–3322)	0 (0–0)	63100 (40840–85360)	4 (3–5)
India	2034	2489 (1503–3474)	0 (0–0)	64247 (39261–89234)	4 (3–6)
India	2035	2534 (1437–3631)	0 (0–0)	65394 (37579–93210)	4 (2–6)
India	2036	2579 (1367–3792)	0 (0–0)	66542 (35797–97286)	4 (2–6)
Indonesia	2022	1443 (1435–1450)	1 (1–1)	41308 (41110–41506)	15 (15–15)
Indonesia	2023	1494 (1477–1510)	1 (1–1)	42697 (42254–43140)	15 (15–15)
Indonesia	2024	1545 (1517–1572)	1 (1–1)	44086 (43345–44828)	15 (14–15)
Indonesia	2025	1596 (1555–1636)	1 (1–1)	45475 (44390–46560)	15 (14–16)
Indonesia	2026	1647 (1592–1702)	1 (1–1)	46864 (45395–48334)	15 (14–16)
Indonesia	2027	1698 (1627–1768)	1 (1–1)	48253 (46363–50143)	15 (14–16)
Indonesia	2028	1749 (1661–1836)	1 (1–1)	49642 (47297–51987)	15 (14–16)
Indonesia	2029	1799 (1694–1905)	1 (1–1)	51031 (48201–53861)	15 (14–17)
Indonesia	2030	1850 (1725–1975)	1 (1–1)	52420 (49075–55765)	15 (13–17)
Indonesia	2031	1901 (1756–2047)	1 (1–1)	53809 (49921–57697)	15 (13–17)

Indonesia	2032	1952 (1786–2119)	1 (1–1)	55198 (50740–59655)	15 (13–18)
Indonesia	2033	2003 (1814–2192)	1 (0–1)	56587 (51535–61639)	15 (13–18)
Indonesia	2034	2054 (1842–2266)	1 (0–1)	57976 (52305–63647)	15 (12–19)
Indonesia	2035	2105 (1869–2341)	1 (0–1)	59365 (53052–65678)	16 (12–19)
Indonesia	2036	2156 (1895–2417)	1 (0–1)	60754 (53776–67731)	16 (12–19)
Iran (Islamic Republic of)	2022	299 (288–310)	0 (0–0)	8605 (8285–8926)	10 (10–10)
Iran (Islamic Republic of)	2023	303 (284–321)	0 (0–0)	8816 (8281–9351)	10 (9–11)
Iran (Islamic Republic of)	2024	301 (271–331)	0 (0–0)	9026 (8268–9784)	10 (9–11)
Iran (Islamic Republic of)	2025	303 (261–344)	0 (0–0)	9236 (8241–10231)	10 (9–12)
Iran (Islamic Republic of)	2026	302 (246–358)	0 (0–0)	9447 (8199–10694)	11 (9–12)
Iran (Islamic Republic of)	2027	303 (233–373)	0 (0–0)	9657 (8141–11173)	11 (9–12)
Iran (Islamic Republic of)	2028	303 (217–389)	0 (0–0)	9867 (8068–11667)	11 (9–12)
Iran (Islamic Republic of)	2029	304 (201–407)	0 (0–0)	10078 (7981–12175)	11 (9–13)
Iran (Islamic Republic of)	2030	304 (183–425)	0 (0–1)	10288 (7879–12697)	11 (9–13)
Iran (Islamic Republic of)	2031	305 (165–444)	0 (0–1)	10498 (7764–13233)	11 (9–13)
Iran (Islamic Republic of)	2032	305 (145–464)	0 (0–1)	10709 (7636–13781)	11 (9–13)
Iran (Islamic Republic of)	2033	305 (125–485)	0 (0–1)	10919 (7496–14343)	11 (9–14)
Iran (Islamic Republic of)	2034	306 (104–507)	0 (0–1)	11129 (7343–14916)	12 (9–14)
Iran (Islamic Republic of)	2035	306 (83–530)	0 (0–1)	11340 (7178–15501)	12 (9–14)
Iran (Islamic Republic of)	2036	307 (60–553)	0 (0–1)	11550 (7002–16098)	12 (9–14)
Iraq	2022	199 (194–204)	1 (1–1)	5399 (5296–5502)	21 (20–21)
Iraq	2023	208 (200–217)	1 (1–1)	5585 (5354–5816)	21 (20–22)
Iraq	2024	218 (205–231)	1 (1–1)	5770 (5384–6157)	21 (20–23)
Iraq	2025	227 (210–245)	1 (1–1)	5956 (5390–6522)	22 (20–24)
Iraq	2026	237 (214–260)	1 (1–1)	6141 (5375–6908)	22 (20–24)
Iraq	2027	247 (218–275)	1 (1–1)	6327 (5341–7313)	23 (20–25)
Iraq	2028	256 (221–291)	1 (1–1)	6513 (5290–7735)	23 (20–26)
Iraq	2029	266 (224–307)	1 (1–1)	6698 (5222–8174)	23 (19–27)
Iraq	2030	275 (227–323)	1 (1–1)	6884 (5139–8628)	24 (19–28)
Iraq	2031	285 (230–340)	1 (1–1)	7069 (5042–9097)	24 (19–30)
Iraq	2032	294 (232–357)	1 (1–1)	7255 (4931–9579)	25 (19–31)
Iraq	2033	304 (233–374)	1 (1–1)	7441 (4806–10075)	25 (18–32)
Iraq	2034	313 (235–392)	1 (1–1)	7626 (4669–10583)	25 (18–33)

Iraq	2035	323 (236–409)	1 (1–1)	7812 (4520–11104)	26 (18–34)
Iraq	2036	332 (237–428)	1 (1–2)	7997 (4359–11636)	26 (17–35)
Ireland	2022	82 (79–86)	1 (1–1)	1805 (1722–1888)	23 (21–24)
Ireland	2023	84 (79–89)	1 (1–1)	1856 (1733–1978)	22 (19–24)
Ireland	2024	86 (81–92)	1 (1–1)	1906 (1769–2043)	21 (18–24)
Ireland	2025	88 (82–93)	1 (1–1)	1938 (1799–2077)	20 (17–23)
Ireland	2026	88 (83–94)	1 (1–1)	1950 (1810–2089)	19 (16–23)
Ireland	2027	88 (83–94)	1 (1–1)	1947 (1808–2087)	18 (14–22)
Ireland	2028	88 (82–94)	1 (1–1)	1940 (1799–2080)	17 (13–22)
Ireland	2029	88 (82–94)	1 (0–1)	1932 (1792–2073)	16 (12–21)
Ireland	2030	87 (82–93)	1 (0–1)	1928 (1787–2069)	16 (11–20)
Ireland	2031	87 (82–93)	1 (0–1)	1926 (1785–2067)	15 (10–20)
Ireland	2032	87 (82–93)	1 (0–1)	1927 (1786–2067)	14 (9–19)
Ireland	2033	87 (82–93)	1 (0–1)	1928 (1787–2069)	13 (7–19)
Ireland	2034	87 (82–93)	1 (0–1)	1929 (1788–2070)	12 (6–18)
Ireland	2035	88 (82–93)	0 (0–1)	1930 (1789–2070)	11 (5–17)
Ireland	2036	88 (82–93)	0 (0–1)	1930 (1789–2071)	10 (4–17)
Israel	2022	139 (133–144)	1 (1–1)	3164 (3015–3312)	27 (25–29)
Israel	2023	141 (133–149)	1 (1–1)	3208 (2998–3417)	26 (23–29)
Israel	2024	143 (133–153)	1 (1–1)	3252 (2995–3508)	26 (22–29)
Israel	2025	145 (134–156)	1 (1–1)	3296 (2999–3592)	26 (22–30)
Israel	2026	147 (134–160)	1 (1–1)	3339 (3008–3671)	25 (21–30)
Israel	2027	149 (135–163)	1 (1–1)	3383 (3020–3747)	25 (20–30)
Israel	2028	151 (136–166)	1 (1–1)	3427 (3035–3820)	25 (19–30)
Israel	2029	153 (137–169)	1 (1–1)	3471 (3052–3891)	24 (19–30)
Israel	2030	155 (138–172)	1 (1–1)	3515 (3070–3960)	24 (18–30)
Israel	2031	157 (139–175)	1 (1–1)	3559 (3090–4028)	24 (17–30)
Israel	2032	159 (140–178)	1 (1–1)	3603 (3111–4095)	23 (17–30)
Israel	2033	161 (142–181)	1 (1–1)	3647 (3133–4161)	23 (16–30)
Israel	2034	163 (143–184)	1 (1–1)	3691 (3156–4226)	23 (15–30)
Israel	2035	165 (144–186)	1 (1–1)	3735 (3180–4290)	22 (15–30)
Israel	2036	167 (145–189)	1 (1–1)	3779 (3205–4354)	22 (14–30)
Italy	2022	1654 (1616–1692)	1 (1–1)	35769 (34699–36839)	28 (27–30)

Italy	2023	1661 (1593–1728)	1 (1–1)	35769 (34256–37282)	28 (26–30)
Italy	2024	1667 (1579–1755)	1 (1–1)	35769 (33916–37622)	28 (26–30)
Italy	2025	1674 (1569–1778)	1 (1–1)	35769 (33629–37909)	27 (25–30)
Italy	2026	1680 (1562–1799)	1 (1–1)	35769 (33376–38162)	27 (24–29)
Italy	2027	1687 (1556–1818)	1 (1–1)	35769 (33148–38390)	26 (24–29)
Italy	2028	1694 (1551–1837)	1 (1–1)	35769 (32938–38600)	26 (23–29)
Italy	2029	1700 (1547–1854)	1 (1–1)	35769 (32743–38795)	25 (22–29)
Italy	2030	1707 (1543–1870)	1 (1–1)	35769 (32559–38979)	25 (22–29)
Italy	2031	1713 (1541–1886)	1 (1–1)	35769 (32385–39153)	25 (21–28)
Italy	2032	1720 (1538–1902)	1 (1–1)	35769 (32220–39318)	24 (20–28)
Italy	2033	1727 (1536–1917)	1 (1–1)	35769 (32062–39476)	24 (20–28)
Italy	2034	1733 (1535–1932)	1 (1–1)	35769 (31911–39627)	23 (19–27)
Italy	2035	1740 (1534–1946)	1 (1–1)	35769 (31765–39773)	23 (19–27)
Italy	2036	1746 (1533–1960)	1 (1–1)	35769 (31625–39913)	22 (18–27)
Jamaica	2022	13 (11–15)	0 (0–0)	327 (276–377)	11 (9–13)
Jamaica	2023	13 (11–15)	0 (0–0)	335 (281–389)	11 (9–13)
Jamaica	2024	13 (11–16)	0 (0–1)	338 (272–405)	11 (9–13)
Jamaica	2025	13 (11–16)	0 (0–1)	345 (273–416)	11 (9–13)
Jamaica	2026	14 (11–16)	0 (0–1)	349 (270–428)	11 (9–13)
Jamaica	2027	14 (11–17)	0 (0–1)	355 (270–439)	11 (9–13)
Jamaica	2028	14 (11–17)	0 (0–1)	360 (269–450)	11 (9–13)
Jamaica	2029	14 (11–18)	0 (0–1)	365 (270–460)	11 (9–13)
Jamaica	2030	14 (11–18)	0 (0–1)	370 (269–470)	11 (9–13)
Jamaica	2031	15 (11–18)	0 (0–1)	375 (270–480)	11 (9–13)
Jamaica	2032	15 (11–19)	0 (0–1)	380 (271–490)	11 (9–13)
Jamaica	2033	15 (11–19)	0 (0–1)	385 (271–499)	11 (9–13)
Jamaica	2034	15 (11–19)	0 (0–1)	391 (272–509)	11 (9–13)
Jamaica	2035	15 (11–20)	0 (0–1)	396 (274–518)	11 (9–13)
Jamaica	2036	16 (11–20)	0 (0–1)	401 (275–527)	11 (9–13)
Japan	2022	4414 (4341–4487)	1 (1–1)	86897 (85020–88774)	30 (29–31)
Japan	2023	4459 (4356–4563)	1 (1–1)	86918 (84048–89789)	30 (29–31)
Japan	2024	4505 (4378–4631)	1 (1–1)	86939 (83155–90724)	29 (28–30)
Japan	2025	4550 (4404–4696)	1 (1–1)	86960 (82276–91645)	29 (27–30)

Japan	2026	4596 (4432–4759)	1 (1–1)	86982 (81389–92574)	28 (27–30)
Japan	2027	4641 (4462–4820)	1 (1–1)	87003 (80484–93522)	28 (26–30)
Japan	2028	4686 (4493–4880)	1 (1–1)	87024 (79555–94492)	28 (26–30)
Japan	2029	4732 (4525–4939)	1 (1–1)	87045 (78602–95488)	27 (25–29)
Japan	2030	4777 (4558–4996)	1 (1–1)	87066 (77621–96511)	27 (25–29)
Japan	2031	4823 (4591–5054)	1 (1–1)	87087 (76613–97561)	26 (24–29)
Japan	2032	4868 (4625–5110)	1 (1–1)	87108 (75577–98639)	26 (24–28)
Japan	2033	4913 (4660–5167)	1 (1–1)	87129 (74514–99745)	26 (23–28)
Japan	2034	4959 (4695–5222)	1 (1–1)	87151 (73424–100877)	25 (23–28)
Japan	2035	5004 (4730–5278)	1 (1–1)	87172 (72306–102037)	25 (22–28)
Japan	2036	5049 (4766–5333)	1 (1–1)	87193 (71162–103223)	24 (22–27)
Jordan	2022	49 (48–50)	1 (1–1)	1406 (1390–1423)	15 (15–16)
Jordan	2023	52 (51–53)	1 (1–1)	1496 (1455–1537)	15 (15–16)
Jordan	2024	55 (53–58)	1 (1–1)	1586 (1513–1659)	15 (14–17)
Jordan	2025	58 (55–62)	1 (1–1)	1675 (1565–1786)	15 (14–17)
Jordan	2026	61 (57–66)	1 (1–1)	1765 (1612–1919)	15 (13–18)
Jordan	2027	64 (59–70)	1 (1–1)	1855 (1655–2056)	15 (13–18)
Jordan	2028	68 (60–75)	1 (1–1)	1945 (1693–2197)	15 (12–18)
Jordan	2029	71 (62–79)	1 (0–1)	2035 (1728–2341)	15 (12–19)
Jordan	2030	74 (63–84)	1 (0–1)	2124 (1759–2490)	15 (12–19)
Jordan	2031	77 (65–89)	1 (0–1)	2214 (1787–2641)	15 (12–19)
Jordan	2032	80 (66–94)	1 (0–1)	2304 (1812–2796)	15 (11–20)
Jordan	2033	83 (67–99)	1 (0–1)	2394 (1834–2954)	15 (11–20)
Jordan	2034	86 (68–104)	1 (0–1)	2483 (1853–3114)	15 (11–20)
Jordan	2035	89 (69–109)	1 (0–1)	2573 (1869–3278)	15 (11–20)
Jordan	2036	92 (70–114)	1 (0–1)	2663 (1882–3444)	15 (10–21)
Kazakhstan	2022	133 (125–141)	1 (1–1)	4111 (3843–4379)	20 (18–21)
Kazakhstan	2023	135 (123–146)	1 (1–1)	4155 (3776–4534)	20 (17–23)
Kazakhstan	2024	136 (122–150)	1 (1–1)	4199 (3735–4663)	20 (16–25)
Kazakhstan	2025	138 (121–154)	1 (1–1)	4243 (3708–4779)	20 (15–26)
Kazakhstan	2026	139 (121–157)	1 (1–1)	4287 (3688–4886)	21 (15–26)
Kazakhstan	2027	141 (121–161)	1 (0–1)	4331 (3675–4987)	21 (15–27)
Kazakhstan	2028	142 (121–164)	1 (0–1)	4375 (3667–5083)	21 (15–28)

Kazakhstan	2029	144 (121–167)	1 (0–1)	4419 (3662–5176)	21 (15–28)
Kazakhstan	2030	145 (121–170)	1 (0–1)	4463 (3660–5266)	21 (15–28)
Kazakhstan	2031	147 (121–173)	1 (0–1)	4507 (3660–5354)	22 (15–29)
Kazakhstan	2032	148 (121–175)	1 (0–1)	4551 (3663–5439)	22 (15–29)
Kazakhstan	2033	150 (122–178)	1 (0–1)	4595 (3667–5522)	22 (15–29)
Kazakhstan	2034	151 (122–181)	1 (0–1)	4639 (3674–5604)	22 (15–29)
Kazakhstan	2035	153 (122–183)	1 (0–1)	4683 (3681–5685)	22 (15–29)
Kazakhstan	2036	154 (123–186)	1 (0–1)	4727 (3690–5764)	22 (15–29)
Kenya	2022	46 (45–46)	0 (0–0)	1290 (1277–1302)	5 (5–5)
Kenya	2023	47 (46–48)	0 (0–0)	1332 (1304–1361)	5 (5–5)
Kenya	2024	48 (46–50)	0 (0–0)	1375 (1328–1423)	5 (5–5)
Kenya	2025	50 (47–52)	0 (0–0)	1418 (1349–1487)	5 (5–5)
Kenya	2026	51 (47–55)	0 (0–0)	1461 (1367–1555)	5 (5–5)
Kenya	2027	53 (48–57)	0 (0–0)	1504 (1383–1625)	5 (5–6)
Kenya	2028	54 (48–60)	0 (0–0)	1547 (1397–1696)	5 (5–6)
Kenya	2029	55 (48–62)	0 (0–0)	1589 (1409–1770)	5 (5–6)
Kenya	2030	57 (48–65)	0 (0–0)	1632 (1418–1846)	5 (5–6)
Kenya	2031	58 (48–68)	0 (0–0)	1675 (1427–1924)	5 (5–6)
Kenya	2032	60 (48–71)	0 (0–0)	1718 (1433–2003)	5 (5–6)
Kenya	2033	61 (48–74)	0 (0–0)	1761 (1438–2084)	5 (5–6)
Kenya	2034	62 (48–77)	0 (0–0)	1804 (1441–2166)	6 (5–6)
Kenya	2035	64 (48–80)	0 (0–0)	1847 (1443–2250)	6 (5–6)
Kenya	2036	65 (48–83)	0 (0–0)	1889 (1443–2335)	6 (5–6)
Kiribati	2022	0 (0–0)	0 (0–0)	5 (5–5)	6 (6–6)
Kiribati	2023	0 (0–0)	0 (0–0)	5 (5–6)	6 (6–7)
Kiribati	2024	0 (0–0)	0 (0–0)	6 (5–6)	6 (6–7)
Kiribati	2025	0 (0–0)	0 (0–0)	6 (5–6)	6 (6–7)
Kiribati	2026	0 (0–0)	0 (0–0)	6 (6–6)	6 (6–7)
Kiribati	2027	0 (0–0)	0 (0–0)	6 (6–6)	6 (5–7)
Kiribati	2028	0 (0–0)	0 (0–0)	6 (6–6)	6 (5–8)
Kiribati	2029	0 (0–0)	0 (0–0)	6 (6–6)	6 (5–8)
Kiribati	2030	0 (0–0)	0 (0–0)	6 (6–7)	6 (5–8)
Kiribati	2031	0 (0–0)	0 (0–0)	6 (6–7)	6 (4–8)

Kiribati	2032	0 (0–0)	0 (0–0)	6 (6–7)	6 (4–9)
Kiribati	2033	0 (0–0)	0 (0–0)	6 (6–7)	6 (4–9)
Kiribati	2034	0 (0–0)	0 (0–0)	7 (6–7)	6 (3–9)
Kiribati	2035	0 (0–0)	0 (0–0)	7 (6–7)	6 (3–10)
Kiribati	2036	0 (0–0)	0 (0–0)	7 (6–7)	6 (3–10)
Kuwait	2022	17 (15–19)	1 (0–1)	496 (448–545)	14 (10–17)
Kuwait	2023	18 (15–20)	1 (0–1)	509 (441–578)	14 (10–17)
Kuwait	2024	18 (15–21)	1 (0–1)	522 (438–606)	14 (10–17)
Kuwait	2025	18 (15–22)	1 (0–1)	535 (438–632)	14 (10–18)
Kuwait	2026	19 (15–23)	1 (0–1)	548 (440–657)	14 (9–18)
Kuwait	2027	19 (15–24)	1 (0–1)	561 (443–680)	13 (9–18)
Kuwait	2028	20 (15–24)	1 (0–1)	574 (446–702)	13 (9–18)
Kuwait	2029	20 (15–25)	1 (0–1)	587 (450–724)	13 (9–18)
Kuwait	2030	21 (16–26)	1 (0–1)	600 (455–746)	13 (9–18)
Kuwait	2031	21 (16–27)	1 (0–1)	613 (460–767)	13 (9–18)
Kuwait	2032	22 (16–27)	1 (0–1)	626 (466–787)	13 (9–18)
Kuwait	2033	22 (16–28)	1 (0–1)	640 (472–807)	13 (9–18)
Kuwait	2034	23 (16–29)	1 (0–1)	653 (478–827)	13 (9–18)
Kuwait	2035	23 (17–29)	1 (0–1)	666 (484–847)	13 (9–18)
Kuwait	2036	23 (17–30)	1 (0–1)	679 (491–866)	13 (9–18)
Kyrgyzstan	2022	39 (36–42)	1 (1–1)	1196 (1110–1283)	21 (18–23)
Kyrgyzstan	2023	39 (34–44)	1 (1–1)	1217 (1059–1374)	21 (16–25)
Kyrgyzstan	2024	40 (33–47)	1 (1–1)	1237 (1031–1442)	21 (15–27)
Kyrgyzstan	2025	41 (33–49)	1 (0–1)	1257 (1012–1501)	21 (14–28)
Kyrgyzstan	2026	41 (32–50)	1 (0–1)	1277 (999–1555)	21 (13–29)
Kyrgyzstan	2027	42 (32–52)	1 (0–1)	1297 (990–1605)	21 (12–29)
Kyrgyzstan	2028	42 (31–53)	1 (0–1)	1317 (983–1652)	21 (11–30)
Kyrgyzstan	2029	43 (31–55)	1 (0–1)	1337 (978–1697)	21 (11–31)
Kyrgyzstan	2030	44 (31–56)	1 (0–1)	1358 (975–1741)	21 (10–32)
Kyrgyzstan	2031	44 (31–58)	1 (0–1)	1378 (973–1783)	21 (9–32)
Kyrgyzstan	2032	45 (31–59)	1 (0–1)	1398 (972–1824)	21 (9–33)
Kyrgyzstan	2033	46 (31–60)	1 (0–1)	1418 (972–1864)	21 (8–33)
Kyrgyzstan	2034	46 (31–61)	1 (0–1)	1438 (973–1903)	21 (8–34)

Kyrgyzstan	2035	47 (31–63)	1 (0–1)	1458 (975–1942)	21 (7–34)
Kyrgyzstan	2036	48 (31–64)	1 (0–1)	1478 (977–1980)	21 (7–35)
Lao People's Democratic Republic	2022	24 (24–25)	1 (1–1)	693 (690–697)	13 (13–13)
Lao People's Democratic Republic	2023	25 (25–26)	1 (1–1)	715 (707–723)	13 (13–13)
Lao People's Democratic Republic	2024	26 (25–26)	1 (1–1)	737 (723–750)	13 (13–14)
Lao People's Democratic Republic	2025	27 (26–27)	1 (1–1)	759 (739–778)	13 (13–14)
Lao People's Democratic Republic	2026	27 (27–28)	1 (1–1)	781 (754–807)	13 (13–14)
Lao People's Democratic Republic	2027	28 (27–29)	1 (1–1)	803 (768–837)	13 (13–14)
Lao People's Democratic Republic	2028	29 (27–31)	1 (1–1)	824 (782–867)	14 (13–14)
Lao People's Democratic Republic	2029	30 (28–32)	1 (1–1)	846 (795–898)	14 (13–14)
Lao People's Democratic Republic	2030	31 (28–33)	1 (0–1)	868 (807–929)	14 (13–15)
Lao People's Democratic Republic	2031	31 (29–34)	1 (0–1)	890 (819–960)	14 (13–15)
Lao People's Democratic Republic	2032	32 (29–35)	1 (0–1)	912 (831–993)	14 (12–15)
Lao People's Democratic Republic	2033	33 (30–36)	1 (0–1)	934 (842–1025)	14 (12–15)
Lao People's Democratic Republic	2034	34 (30–37)	1 (0–1)	956 (853–1058)	14 (12–15)
Lao People's Democratic Republic	2035	34 (30–38)	1 (0–1)	977 (863–1092)	14 (12–16)
Lao People's Democratic Republic	2036	35 (31–40)	1 (0–1)	999 (873–1126)	14 (12–16)
Latvia	2022	55 (51–59)	2 (1–2)	1510 (1381–1640)	46 (42–50)
Latvia	2023	56 (50–62)	2 (1–2)	1551 (1360–1742)	46 (41–52)
Latvia	2024	57 (50–63)	2 (1–2)	1589 (1384–1794)	47 (41–53)
Latvia	2025	57 (50–63)	2 (1–2)	1589 (1384–1794)	47 (41–53)
Latvia	2026	57 (50–63)	2 (1–2)	1589 (1384–1794)	47 (41–53)
Latvia	2027	57 (50–63)	2 (1–2)	1589 (1384–1794)	47 (41–53)
Latvia	2028	57 (50–63)	2 (1–2)	1589 (1384–1794)	47 (41–53)
Latvia	2029	57 (50–63)	2 (1–2)	1589 (1384–1794)	47 (41–53)
Latvia	2030	57 (50–63)	2 (1–2)	1589 (1384–1794)	47 (41–53)
Latvia	2031	57 (50–63)	2 (1–2)	1589 (1384–1794)	47 (41–53)
Latvia	2032	57 (50–63)	2 (1–2)	1589 (1384–1794)	47 (41–53)
Latvia	2033	57 (50–63)	2 (1–2)	1589 (1384–1794)	47 (41–53)
Latvia	2034	57 (50–63)	2 (1–2)	1589 (1384–1794)	47 (41–53)
Latvia	2035	57 (50–63)	2 (1–2)	1589 (1384–1794)	47 (41–53)
Latvia	2036	57 (50–63)	2 (1–2)	1589 (1384–1794)	47 (41–53)
Lebanon	2022	56 (55–57)	1 (1–1)	1294 (1273–1315)	22 (21–22)

Lebanon	2023	56 (54–58)	1 (1–1)	1313 (1269–1357)	22 (21–23)
Lebanon	2024	56 (53–59)	1 (1–1)	1333 (1266–1401)	22 (20–23)
Lebanon	2025	56 (52–61)	1 (1–1)	1354 (1263–1446)	22 (19–24)
Lebanon	2026	57 (50–63)	1 (1–1)	1376 (1261–1491)	22 (19–24)
Lebanon	2027	57 (49–64)	1 (1–1)	1399 (1261–1537)	22 (18–25)
Lebanon	2028	57 (48–66)	1 (1–1)	1422 (1262–1582)	22 (17–26)
Lebanon	2029	57 (46–68)	1 (1–1)	1446 (1264–1627)	21 (17–26)
Lebanon	2030	57 (44–70)	1 (1–1)	1469 (1267–1671)	21 (16–27)
Lebanon	2031	57 (43–72)	1 (1–1)	1493 (1272–1715)	21 (16–27)
Lebanon	2032	57 (41–74)	1 (1–1)	1518 (1277–1758)	21 (15–28)
Lebanon	2033	58 (40–76)	1 (1–1)	1542 (1284–1800)	21 (15–28)
Lebanon	2034	58 (38–77)	1 (1–1)	1566 (1291–1842)	21 (14–29)
Lebanon	2035	58 (36–79)	1 (1–1)	1591 (1299–1883)	21 (14–29)
Lebanon	2036	58 (35–81)	1 (1–1)	1615 (1308–1923)	21 (13–29)
Lesotho	2022	8 (8–9)	1 (1–1)	248 (244–253)	21 (21–21)
Lesotho	2023	8 (8–9)	1 (1–1)	249 (238–261)	21 (20–22)
Lesotho	2024	9 (8–9)	1 (1–1)	255 (236–273)	22 (20–23)
Lesotho	2025	9 (8–10)	1 (1–1)	260 (237–283)	22 (20–24)
Lesotho	2026	9 (8–10)	1 (1–1)	265 (238–292)	22 (20–24)
Lesotho	2027	9 (8–10)	1 (1–1)	270 (240–301)	23 (20–25)
Lesotho	2028	9 (8–10)	1 (1–1)	276 (242–309)	23 (20–26)
Lesotho	2029	9 (8–11)	1 (1–1)	281 (245–317)	24 (21–26)
Lesotho	2030	10 (8–11)	1 (1–1)	286 (247–325)	24 (21–27)
Lesotho	2031	10 (8–11)	1 (1–1)	291 (250–333)	24 (21–28)
Lesotho	2032	10 (8–11)	1 (1–1)	297 (253–340)	25 (21–28)
Lesotho	2033	10 (8–12)	1 (1–1)	302 (256–348)	25 (21–29)
Lesotho	2034	10 (8–12)	1 (1–1)	307 (259–355)	25 (22–29)
Lesotho	2035	10 (9–12)	1 (1–1)	312 (262–362)	26 (22–30)
Lesotho	2036	11 (9–12)	1 (1–1)	318 (266–369)	26 (22–30)
Liberia	2022	3 (3–3)	0 (0–0)	91 (90–93)	3 (3–4)
Liberia	2023	3 (3–3)	0 (0–0)	95 (92–98)	3 (3–4)
Liberia	2024	3 (3–3)	0 (0–0)	99 (93–104)	4 (3–4)
Liberia	2025	3 (3–3)	0 (0–0)	103 (95–111)	4 (3–4)

Liberia	2026	3 (3–4)	0 (0–0)	106 (96–117)	4 (3–4)
Liberia	2027	3 (3–4)	0 (0–0)	110 (96–124)	4 (3–4)
Liberia	2028	4 (3–4)	0 (0–0)	114 (97–131)	4 (3–4)
Liberia	2029	4 (3–4)	0 (0–0)	118 (97–139)	4 (3–4)
Liberia	2030	4 (3–5)	0 (0–0)	122 (97–146)	4 (3–4)
Liberia	2031	4 (3–5)	0 (0–0)	125 (97–154)	4 (3–4)
Liberia	2032	4 (3–5)	0 (0–0)	129 (97–162)	4 (3–4)
Liberia	2033	4 (3–5)	0 (0–0)	133 (96–170)	4 (3–4)
Liberia	2034	4 (3–6)	0 (0–0)	137 (95–178)	4 (3–4)
Liberia	2035	4 (3–6)	0 (0–0)	141 (94–187)	4 (3–4)
Liberia	2036	4 (3–6)	0 (0–0)	145 (93–196)	4 (3–4)
Libya	2022	60 (59–61)	1 (1–1)	1752 (1728–1776)	28 (27–29)
Libya	2023	61 (59–64)	1 (1–1)	1802 (1748–1856)	28 (26–29)
Libya	2024	63 (59–67)	1 (1–1)	1852 (1762–1942)	28 (25–30)
Libya	2025	64 (58–70)	1 (1–1)	1902 (1771–2034)	28 (25–31)
Libya	2026	66 (58–74)	1 (1–1)	1952 (1774–2130)	28 (24–31)
Libya	2027	67 (57–78)	1 (1–1)	2002 (1773–2231)	28 (23–32)
Libya	2028	69 (56–81)	1 (1–1)	2053 (1769–2337)	28 (23–32)
Libya	2029	70 (55–86)	1 (1–1)	2103 (1760–2446)	28 (22–33)
Libya	2030	72 (54–90)	1 (1–1)	2153 (1748–2558)	28 (22–33)
Libya	2031	73 (52–94)	1 (1–1)	2203 (1732–2674)	28 (21–34)
Libya	2032	75 (50–99)	1 (1–1)	2253 (1713–2793)	28 (21–34)
Libya	2033	76 (49–104)	1 (1–1)	2303 (1691–2915)	27 (20–35)
Libya	2034	78 (47–108)	1 (1–1)	2353 (1666–3040)	27 (20–35)
Libya	2035	79 (45–113)	1 (1–1)	2403 (1639–3168)	27 (20–35)
Libya	2036	81 (43–118)	1 (1–1)	2454 (1608–3299)	27 (19–36)
Lithuania	2022	73 (68–78)	1 (1–1)	1917 (1760–2074)	39 (36–42)
Lithuania	2023	73 (66–80)	1 (1–1)	1917 (1695–2140)	39 (35–43)
Lithuania	2024	73 (64–82)	1 (1–2)	1917 (1645–2190)	39 (34–44)
Lithuania	2025	73 (63–83)	1 (1–2)	1917 (1603–2232)	39 (34–44)
Lithuania	2026	73 (62–84)	1 (1–2)	1917 (1566–2269)	39 (35–44)
Lithuania	2027	73 (61–85)	1 (1–2)	1917 (1532–2302)	39 (35–44)
Lithuania	2028	73 (60–86)	1 (1–2)	1917 (1501–2333)	39 (35–44)

Lithuania	2029	73 (59–87)	1 (1–2)	1917 (1473–2362)	39 (35–44)
Lithuania	2030	73 (58–88)	1 (1–2)	1917 (1446–2389)	39 (35–44)
Lithuania	2031	73 (57–89)	1 (1–2)	1917 (1420–2414)	39 (35–44)
Lithuania	2032	73 (57–90)	1 (1–2)	1917 (1396–2439)	39 (35–44)
Lithuania	2033	73 (56–90)	1 (1–2)	1917 (1373–2462)	39 (35–44)
Lithuania	2034	73 (55–91)	1 (1–2)	1917 (1351–2484)	39 (35–44)
Lithuania	2035	73 (54–92)	1 (1–2)	1917 (1329–2505)	39 (35–44)
Lithuania	2036	73 (54–92)	1 (1–2)	1917 (1309–2526)	39 (35–44)
Low SDI	2022	660 (654–666)	0 (0–0)	18209 (18061–18358)	3 (3–3)
Low SDI	2023	675 (662–689)	0 (0–0)	18726 (18393–19058)	3 (3–3)
Low SDI	2024	691 (669–713)	0 (0–0)	19242 (18685–19798)	3 (3–3)
Low SDI	2025	707 (675–739)	0 (0–0)	19758 (18943–20572)	3 (3–3)
Low SDI	2026	723 (679–767)	0 (0–0)	20274 (19171–21377)	3 (3–4)
Low SDI	2027	739 (682–795)	0 (0–0)	20790 (19371–22209)	3 (3–4)
Low SDI	2028	755 (685–824)	0 (0–0)	21306 (19547–23066)	3 (3–4)
Low SDI	2029	770 (686–855)	0 (0–0)	21822 (19698–23946)	3 (3–4)
Low SDI	2030	786 (687–886)	0 (0–0)	22338 (19828–24849)	3 (3–4)
Low SDI	2031	802 (686–918)	0 (0–0)	22855 (19936–25773)	3 (3–4)
Low SDI	2032	818 (685–950)	0 (0–0)	23371 (20025–26716)	3 (3–4)
Low SDI	2033	834 (683–984)	0 (0–0)	23887 (20095–27678)	3 (3–4)
Low SDI	2034	849 (681–1018)	0 (0–0)	24403 (20147–28659)	3 (3–4)
Low SDI	2035	865 (677–1053)	0 (0–0)	24919 (20181–29657)	3 (3–4)
Low SDI	2036	881 (673–1089)	0 (0–0)	25435 (20198–30672)	3 (3–4)
Low-middle SDI	2022	4172 (4144–4200)	0 (0–0)	12896 (112098–113695	7 (7–7)
Low-middle SDI	2023	4259 (4196–4321)	0 (0–0)	15460 (113674–117246	7 (7–7)
Low-middle SDI	2024	4345 (4240–4450)	0 (0–0)	18023 (115035–121011	7 (7–7)
Low-middle SDI	2025	4432 (4278–4585)	0 (0–0)	20586 (116212–124961	7 (7–8)
Low-middle SDI	2026	4518 (4310–4726)	0 (0–0)	23149 (117226–129072	7 (7–8)
Low-middle SDI	2027	4605 (4337–4873)	0 (0–0)	25713 (118094–133331	7 (7–8)
Low-middle SDI	2028	4691 (4359–5024)	0 (0–0)	28276 (118826–137726	7 (7–8)
Low-middle SDI	2029	4778 (4377–5179)	0 (0–0)	30839 (119432–142246	8 (7–8)
Low-middle SDI	2030	4865 (4391–5339)	0 (0–0)	33402 (119920–146885	8 (7–8)
Low-middle SDI	2031	4951 (4400–5502)	0 (0–0)	35966 (120295–151636	8 (7–8)

Low-middle SDI	2032	5038 (4406–5669)	0 (0–0)	[illegible]38529 (120564–156494	8 (7–8)
Low-middle SDI	2033	5124 (4409–5840)	0 (0–0)	[illegible]41092 (120730–161454	8 (7–8)
Low-middle SDI	2034	5211 (4407–6014)	0 (0–0)	[illegible]43655 (120799–166511	8 (7–8)
Low-middle SDI	2035	5298 (4403–6192)	0 (0–0)	[illegible]46219 (120774–171663	8 (7–8)
Low-middle SDI	2036	5384 (4396–6373)	0 (0–0)	[illegible]48782 (120658–176905	8 (7–8)
Luxembourg	2022	13 (12–13)	1 (1–1)	294 (279–308)	28 (26–30)
Luxembourg	2023	13 (12–14)	1 (1–1)	296 (275–316)	28 (25–31)
Luxembourg	2024	13 (12–14)	1 (1–1)	298 (273–323)	27 (24–31)
Luxembourg	2025	13 (12–14)	1 (1–1)	300 (271–329)	27 (23–31)
Luxembourg	2026	13 (12–15)	1 (1–1)	302 (270–334)	26 (22–31)
Luxembourg	2027	13 (12–15)	1 (1–1)	304 (269–339)	26 (21–31)
Luxembourg	2028	13 (12–15)	1 (1–1)	306 (268–344)	25 (20–31)
Luxembourg	2029	14 (12–15)	1 (1–1)	308 (267–349)	25 (19–31)
Luxembourg	2030	14 (12–16)	1 (0–1)	310 (267–354)	24 (19–30)
Luxembourg	2031	14 (12–16)	1 (0–1)	312 (267–358)	24 (18–30)
Luxembourg	2032	14 (12–16)	1 (0–1)	314 (266–363)	24 (17–30)
Luxembourg	2033	14 (12–16)	1 (0–1)	317 (266–367)	23 (16–30)
Luxembourg	2034	14 (12–16)	1 (0–1)	319 (266–371)	23 (15–30)
Luxembourg	2035	14 (12–17)	1 (0–1)	321 (267–375)	22 (15–30)
Luxembourg	2036	14 (12–17)	1 (0–1)	323 (267–379)	22 (14–29)
Madagascar	2022	9 (9–9)	0 (0–0)	269 (263–275)	2 (2–2)
Madagascar	2023	9 (9–10)	0 (0–0)	277 (266–287)	2 (2–2)
Madagascar	2024	9 (9–10)	0 (0–0)	284 (269–300)	2 (2–2)
Madagascar	2025	10 (9–10)	0 (0–0)	292 (271–313)	2 (2–2)
Madagascar	2026	10 (9–11)	0 (0–0)	300 (273–327)	2 (2–2)
Madagascar	2027	10 (9–11)	0 (0–0)	308 (275–341)	2 (2–2)
Madagascar	2028	10 (9–12)	0 (0–0)	315 (276–355)	2 (2–2)
Madagascar	2029	11 (9–12)	0 (0–0)	323 (276–370)	2 (1–2)
Madagascar	2030	11 (9–13)	0 (0–0)	331 (277–385)	2 (1–3)
Madagascar	2031	11 (9–13)	0 (0–0)	338 (276–400)	2 (1–3)
Madagascar	2032	12 (9–14)	0 (0–0)	346 (276–416)	2 (1–3)
Madagascar	2033	12 (9–15)	0 (0–0)	354 (275–432)	2 (1–3)
Madagascar	2034	12 (9–15)	0 (0–0)	362 (274–449)	2 (1–3)

Madagascar	2035	12 (9–16)	0 (0–0)	369 (273–466)	2 (1–3)
Madagascar	2036	13 (9–16)	0 (0–0)	377 (271–483)	2 (1–3)
Malawi	2022	6 (6–6)	0 (0–0)	175 (173–176)	2 (2–2)
Malawi	2023	6 (6–7)	0 (0–0)	179 (174–183)	2 (2–2)
Malawi	2024	7 (6–7)	0 (0–0)	182 (176–189)	2 (2–2)
Malawi	2025	7 (6–7)	0 (0–0)	186 (176–196)	2 (2–2)
Malawi	2026	7 (6–7)	0 (0–0)	190 (177–203)	2 (2–2)
Malawi	2027	7 (6–8)	0 (0–0)	194 (177–211)	2 (2–2)
Malawi	2028	7 (6–8)	0 (0–0)	198 (176–219)	2 (2–3)
Malawi	2029	7 (6–8)	0 (0–0)	201 (176–227)	2 (2–3)
Malawi	2030	7 (6–8)	0 (0–0)	205 (175–236)	2 (2–3)
Malawi	2031	7 (6–9)	0 (0–0)	209 (174–245)	2 (1–3)
Malawi	2032	7 (6–9)	0 (0–0)	213 (172–254)	2 (1–3)
Malawi	2033	7 (6–9)	0 (0–0)	217 (171–263)	2 (1–3)
Malawi	2034	8 (6–10)	0 (0–0)	220 (169–272)	2 (1–3)
Malawi	2035	8 (5–10)	0 (0–0)	224 (167–282)	2 (1–3)
Malawi	2036	8 (5–10)	0 (0–0)	228 (164–292)	2 (1–3)
Malaysia	2022	100 (95–105)	0 (0–0)	2719 (2613–2825)	9 (8–10)
Malaysia	2023	104 (99–110)	0 (0–0)	2781 (2660–2903)	9 (8–10)
Malaysia	2024	106 (99–112)	0 (0–0)	2844 (2710–2979)	9 (8–10)
Malaysia	2025	108 (101–116)	0 (0–0)	2907 (2760–3054)	9 (8–10)
Malaysia	2026	110 (103–118)	0 (0–0)	2970 (2812–3128)	9 (8–10)
Malaysia	2027	113 (105–121)	0 (0–0)	3033 (2864–3201)	9 (7–11)
Malaysia	2028	115 (106–124)	0 (0–0)	3096 (2917–3274)	9 (7–11)
Malaysia	2029	118 (108–127)	0 (0–0)	3159 (2971–3347)	9 (7–11)
Malaysia	2030	120 (110–130)	0 (0–0)	3222 (3025–3418)	9 (7–11)
Malaysia	2031	122 (112–133)	0 (0–0)	3285 (3079–3490)	9 (7–11)
Malaysia	2032	125 (114–136)	0 (0–0)	3347 (3134–3561)	9 (7–11)
Malaysia	2033	127 (116–138)	0 (0–0)	3410 (3189–3632)	9 (7–11)
Malaysia	2034	129 (117–141)	0 (0–0)	3473 (3244–3702)	9 (7–11)
Malaysia	2035	132 (119–144)	0 (0–0)	3536 (3300–3772)	9 (7–11)
Malaysia	2036	134 (121–147)	0 (0–0)	3599 (3356–3842)	9 (7–11)
Maldives	2022	1 (1–1)	0 (0–0)	27 (26–28)	7 (7–8)

Maldives	2023	1 (1–1)	0 (0–0)	28 (27–29)	7 (6–8)
Maldives	2024	1 (1–1)	0 (0–0)	29 (28–31)	7 (6–8)
Maldives	2025	1 (1–1)	0 (0–0)	31 (29–33)	7 (6–8)
Maldives	2026	1 (1–1)	0 (0–0)	32 (29–34)	7 (6–8)
Maldives	2027	1 (1–1)	0 (0–0)	33 (30–36)	7 (6–8)
Maldives	2028	1 (1–1)	0 (0–0)	34 (30–38)	7 (5–8)
Maldives	2029	1 (1–2)	0 (0–0)	36 (31–40)	7 (5–8)
Maldives	2030	1 (1–2)	0 (0–0)	37 (31–43)	6 (5–8)
Maldives	2031	1 (1–2)	0 (0–0)	38 (32–45)	6 (5–8)
Maldives	2032	1 (1–2)	0 (0–0)	39 (32–47)	6 (5–8)
Maldives	2033	2 (1–2)	0 (0–0)	41 (32–49)	6 (5–8)
Maldives	2034	2 (1–2)	0 (0–0)	42 (32–51)	6 (5–8)
Maldives	2035	2 (1–2)	0 (0–0)	43 (32–54)	6 (4–8)
Maldives	2036	2 (1–2)	0 (0–0)	44 (33–56)	6 (4–8)
Mali	2022	20 (20–20)	0 (0–0)	536 (532–539)	6 (6–6)
Mali	2023	20 (20–21)	0 (0–0)	549 (541–557)	6 (6–6)
Mali	2024	21 (20–21)	0 (0–0)	563 (550–576)	6 (6–6)
Mali	2025	21 (20–22)	0 (0–0)	576 (557–595)	6 (5–6)
Mali	2026	21 (20–22)	0 (0–0)	590 (564–616)	6 (5–6)
Mali	2027	22 (20–23)	0 (0–0)	604 (570–637)	6 (5–6)
Mali	2028	22 (20–24)	0 (0–0)	617 (576–658)	6 (5–6)
Mali	2029	22 (20–25)	0 (0–0)	631 (581–680)	6 (5–7)
Mali	2030	23 (20–25)	0 (0–0)	644 (585–703)	6 (5–7)
Mali	2031	23 (20–26)	0 (0–0)	658 (590–726)	6 (5–7)
Mali	2032	23 (19–27)	0 (0–0)	671 (593–750)	6 (5–7)
Mali	2033	24 (19–28)	0 (0–0)	685 (596–774)	6 (4–7)
Mali	2034	24 (19–29)	0 (0–0)	698 (599–798)	6 (4–7)
Mali	2035	24 (19–29)	0 (0–0)	712 (601–823)	6 (4–8)
Mali	2036	24 (19–30)	0 (0–0)	726 (603–848)	6 (4–8)
Malta	2022	10 (10–11)	1 (1–1)	250 (236–264)	31 (29–33)
Malta	2023	10 (10–11)	1 (1–1)	253 (233–273)	31 (28–34)
Malta	2024	11 (10–12)	1 (1–1)	255 (231–280)	31 (27–34)
Malta	2025	11 (10–12)	1 (1–1)	258 (230–286)	31 (27–35)

Malta	2026	11 (10–12)	1 (1–1)	261 (230–292)	31 (26–35)
Malta	2027	11 (10–12)	1 (1–1)	264 (229–298)	31 (26–36)
Malta	2028	11 (10–13)	1 (1–1)	266 (229–303)	31 (26–36)
Malta	2029	11 (10–13)	1 (1–1)	269 (229–309)	31 (25–36)
Malta	2030	11 (10–13)	1 (1–1)	272 (230–314)	31 (25–37)
Malta	2031	11 (10–13)	1 (1–1)	274 (230–319)	31 (25–37)
Malta	2032	12 (10–13)	1 (1–1)	277 (231–324)	31 (24–37)
Malta	2033	12 (10–14)	1 (1–1)	280 (231–328)	31 (24–38)
Malta	2034	12 (10–14)	1 (1–1)	283 (232–333)	31 (24–38)
Malta	2035	12 (10–14)	1 (1–1)	285 (233–338)	31 (23–38)
Malta	2036	12 (10–14)	1 (1–1)	288 (234–342)	31 (23–39)
Marshall Islands	2022	0 (0–0)	0 (0–0)	5 (5–5)	12 (11–12)
Marshall Islands	2023	0 (0–0)	0 (0–0)	5 (5–5)	12 (11–12)
Marshall Islands	2024	0 (0–0)	0 (0–0)	5 (5–5)	12 (11–12)
Marshall Islands	2025	0 (0–0)	0 (0–0)	5 (5–6)	12 (11–12)
Marshall Islands	2026	0 (0–0)	0 (0–0)	6 (5–6)	12 (11–13)
Marshall Islands	2027	0 (0–0)	0 (0–0)	6 (5–6)	12 (11–13)
Marshall Islands	2028	0 (0–0)	0 (0–0)	6 (6–6)	12 (11–13)
Marshall Islands	2029	0 (0–0)	0 (0–0)	6 (6–6)	12 (12–13)
Marshall Islands	2030	0 (0–0)	0 (0–0)	6 (6–7)	13 (12–13)
Marshall Islands	2031	0 (0–0)	0 (0–0)	6 (6–7)	13 (12–14)
Marshall Islands	2032	0 (0–0)	0 (0–1)	6 (6–7)	13 (12–14)
Marshall Islands	2033	0 (0–0)	0 (0–1)	6 (6–7)	13 (12–14)
Marshall Islands	2034	0 (0–0)	0 (0–1)	7 (6–7)	13 (12–14)
Marshall Islands	2035	0 (0–0)	0 (0–1)	7 (6–8)	13 (12–14)
Marshall Islands	2036	0 (0–0)	0 (0–1)	7 (6–8)	13 (12–14)
Mauritania	2022	4 (4–4)	0 (0–0)	124 (121–127)	5 (5–5)
Mauritania	2023	5 (4–5)	0 (0–0)	130 (126–135)	5 (5–5)
Mauritania	2024	5 (5–5)	0 (0–0)	137 (130–144)	5 (5–6)
Mauritania	2025	5 (5–5)	0 (0–0)	143 (133–153)	5 (5–6)
Mauritania	2026	5 (5–6)	0 (0–0)	149 (136–162)	6 (5–6)
Mauritania	2027	5 (5–6)	0 (0–0)	155 (139–172)	6 (5–6)
Mauritania	2028	6 (5–6)	0 (0–0)	161 (141–182)	6 (5–7)

Mauritania	2029	6 (5–7)	0 (0–0)	168 (143–192)	6 (5–7)
Mauritania	2030	6 (5–7)	0 (0–0)	174 (145–202)	6 (5–7)
Mauritania	2031	6 (5–7)	0 (0–0)	180 (147–213)	6 (5–7)
Mauritania	2032	6 (5–8)	0 (0–0)	186 (149–224)	6 (5–7)
Mauritania	2033	7 (5–8)	0 (0–0)	192 (150–235)	6 (5–8)
Mauritania	2034	7 (5–8)	0 (0–0)	199 (151–246)	7 (5–8)
Mauritania	2035	7 (5–9)	0 (0–0)	205 (152–257)	7 (5–8)
Mauritania	2036	7 (5–9)	0 (0–0)	211 (153–269)	7 (5–9)
Mauritius	2022	14 (13–15)	1 (1–1)	376 (353–399)	19 (17–21)
Mauritius	2023	15 (13–16)	1 (1–1)	390 (353–427)	19 (16–21)
Mauritius	2024	15 (13–17)	1 (1–1)	404 (354–455)	18 (15–21)
Mauritius	2025	16 (14–18)	1 (1–1)	418 (354–482)	18 (15–22)
Mauritius	2026	16 (14–19)	1 (1–1)	432 (353–511)	18 (14–22)
Mauritius	2027	17 (14–20)	1 (1–1)	446 (352–540)	18 (14–22)
Mauritius	2028	18 (14–22)	1 (1–1)	460 (351–569)	18 (14–22)
Mauritius	2029	18 (14–23)	1 (1–1)	474 (348–599)	18 (14–22)
Mauritius	2030	19 (14–24)	1 (1–1)	488 (346–630)	18 (13–22)
Mauritius	2031	19 (14–25)	1 (1–1)	502 (342–662)	17 (13–22)
Mauritius	2032	20 (14–26)	1 (0–1)	516 (338–694)	17 (13–22)
Mauritius	2033	21 (13–28)	1 (0–1)	530 (333–726)	17 (13–21)
Mauritius	2034	21 (13–29)	1 (0–1)	544 (328–760)	17 (13–21)
Mauritius	2035	22 (13–30)	1 (0–1)	558 (322–793)	17 (13–21)
Mauritius	2036	22 (13–31)	1 (0–1)	572 (316–828)	17 (13–21)
Mexico	2022	563 (549–578)	0 (0–0)	14124 (13769–14479)	10 (10–11)
Mexico	2023	570 (542–597)	0 (0–0)	14279 (13598–14961)	10 (9–11)
Mexico	2024	576 (540–613)	0 (0–0)	14435 (13539–15330)	10 (9–11)
Mexico	2025	583 (539–626)	0 (0–0)	14590 (13522–15658)	9 (8–11)
Mexico	2026	589 (540–639)	0 (0–0)	14745 (13529–15961)	9 (8–11)
Mexico	2027	596 (541–651)	0 (0–0)	14900 (13552–16248)	9 (7–11)
Mexico	2028	602 (543–662)	0 (0–0)	15055 (13587–16523)	9 (7–10)
Mexico	2029	609 (545–673)	0 (0–0)	15211 (13632–16790)	8 (6–10)
Mexico	2030	615 (547–684)	0 (0–0)	15366 (13683–17049)	8 (6–10)
Mexico	2031	622 (549–694)	0 (0–0)	15521 (13741–17301)	8 (5–10)

Mexico	2032	628 (552–705)	0 (0–0)	15676 (13803–17549)	7 (5–10)
Mexico	2033	635 (555–715)	0 (0–0)	15832 (13870–17793)	7 (4–10)
Mexico	2034	642 (558–725)	0 (0–0)	15987 (13941–18032)	7 (4–9)
Mexico	2035	648 (561–735)	0 (0–0)	16142 (14015–18269)	6 (3–9)
Mexico	2036	655 (565–744)	0 (0–0)	16297 (14093–18502)	6 (3–9)
Micronesia (Federated States of)	2022	1 (1–1)	1 (1–1)	19 (19–19)	22 (21–22)
Micronesia (Federated States of)	2023	1 (1–1)	1 (1–1)	20 (20–20)	22 (21–22)
Micronesia (Federated States of)	2024	1 (1–1)	1 (1–1)	20 (20–20)	22 (21–22)
Micronesia (Federated States of)	2025	1 (1–1)	1 (1–1)	21 (20–21)	22 (21–22)
Micronesia (Federated States of)	2026	1 (1–1)	1 (1–1)	21 (20–22)	22 (21–23)
Micronesia (Federated States of)	2027	1 (1–1)	1 (1–1)	21 (21–22)	22 (21–23)
Micronesia (Federated States of)	2028	1 (1–1)	1 (1–1)	22 (21–23)	22 (21–23)
Micronesia (Federated States of)	2029	1 (1–1)	1 (1–1)	22 (21–23)	22 (20–24)
Micronesia (Federated States of)	2030	1 (1–1)	1 (1–1)	22 (21–24)	22 (20–24)
Micronesia (Federated States of)	2031	1 (1–1)	1 (1–1)	23 (21–24)	22 (20–24)
Micronesia (Federated States of)	2032	1 (1–1)	1 (1–1)	23 (21–25)	22 (20–25)
Micronesia (Federated States of)	2033	1 (1–1)	1 (1–1)	24 (22–26)	22 (20–25)
Micronesia (Federated States of)	2034	1 (1–1)	1 (1–1)	24 (22–26)	22 (19–26)
Micronesia (Federated States of)	2035	1 (1–1)	1 (1–1)	24 (22–27)	23 (19–26)
Micronesia (Federated States of)	2036	1 (1–1)	1 (1–1)	25 (22–28)	23 (19–26)
Middle SDI	2022	5831 (16697–1696	1 (1–1)	44017 (440701–447334	15 (15–15)
Middle SDI	2023	7296 (16998–1759	1 (1–1)	55059 (447643–462474	15 (15–16)
Middle SDI	2024	7761 (17262–1826	1 (1–1)	66100 (453690–478509	15 (15–16)
Middle SDI	2025	8227 (17496–1895	1 (1–1)	77141 (458975–495306	15 (14–16)
Middle SDI	2026	8692 (17702–1968	1 (1–1)	88182 (463586–512778	15 (14–17)
Middle SDI	2027	9157 (17884–2043	1 (1–1)	99223 (467585–530860	15 (13–17)
Middle SDI	2028	9622 (18043–2120	1 (1–1)	10264 (471022–549506	15 (13–17)
Middle SDI	2029	0088 (18182–2199	1 (1–1)	21305 (473936–568674	15 (12–18)
Middle SDI	2030	0553 (18300–2280	1 (1–1)	32346 (476357–588335	15 (12–18)
Middle SDI	2031	1018 (18400–2363	1 (1–1)	43387 (478312–608462	15 (11–19)
Middle SDI	2032	1484 (18482–2448	1 (1–1)	54428 (479825–629032	15 (11–19)
Middle SDI	2033	1949 (18546–2535	1 (1–1)	65469 (480914–650024	15 (10–20)
Middle SDI	2034	2414 (18595–2623	1 (1–1)	76511 (481598–671423	15 (10–20)

Middle SDI	2035	2879 (18628–2713	1 (1–1)	587552 (481890–693213	15 (9–21)
Middle SDI	2036	3345 (18645–2804	1 (1–1)	598593 (481806–715379	15 (8–22)
Monaco	2022	2 (2–2)	2 (2–2)	38 (38–39)	46 (46–47)
Monaco	2023	2 (2–2)	2 (2–2)	39 (38–39)	46 (45–47)
Monaco	2024	2 (2–2)	2 (2–2)	39 (38–39)	46 (45–47)
Monaco	2025	2 (2–2)	2 (2–2)	39 (38–39)	46 (44–47)
Monaco	2026	2 (2–2)	2 (2–2)	39 (38–40)	45 (44–47)
Monaco	2027	2 (2–2)	2 (2–2)	39 (38–40)	45 (43–47)
Monaco	2028	2 (2–2)	2 (2–2)	39 (38–40)	45 (43–47)
Monaco	2029	2 (2–2)	2 (2–2)	40 (38–41)	45 (42–47)
Monaco	2030	2 (2–2)	2 (2–2)	40 (38–41)	44 (41–47)
Monaco	2031	2 (2–2)	2 (2–2)	40 (39–41)	44 (41–47)
Monaco	2032	2 (2–2)	2 (2–2)	40 (39–42)	44 (40–48)
Monaco	2033	2 (2–2)	2 (2–2)	40 (39–42)	44 (39–48)
Monaco	2034	2 (2–2)	2 (1–2)	40 (39–42)	43 (39–48)
Monaco	2035	2 (2–2)	2 (1–2)	41 (39–43)	43 (38–48)
Monaco	2036	2 (2–2)	2 (1–2)	41 (39–43)	43 (37–48)
Mongolia	2022	31 (30–32)	1 (1–1)	959 (920–999)	34 (32–35)
Mongolia	2023	31 (29–34)	1 (1–1)	955 (866–1044)	33 (30–36)
Mongolia	2024	31 (27–35)	1 (1–1)	950 (802–1099)	32 (27–37)
Mongolia	2025	31 (25–38)	1 (1–1)	946 (728–1163)	31 (25–38)
Mongolia	2026	31 (23–40)	1 (1–1)	941 (646–1236)	31 (22–39)
Mongolia	2027	32 (21–42)	1 (1–1)	936 (557–1315)	30 (20–41)
Mongolia	2028	32 (18–45)	1 (1–1)	932 (462–1402)	30 (18–42)
Mongolia	2029	32 (16–48)	1 (1–2)	927 (360–1494)	30 (16–44)
Mongolia	2030	32 (13–51)	1 (1–2)	923 (252–1593)	29 (14–45)
Mongolia	2031	32 (10–54)	1 (0–2)	918 (139–1697)	29 (12–47)
Mongolia	2032	32 (7–57)	1 (0–2)	913 (20–1807)	29 (10–48)
Mongolia	2033	32 (4–61)	1 (0–2)	909 (-104–1921)	29 (8–50)
Mongolia	2034	32 (0–64)	1 (0–2)	904 (-233–2041)	29 (6–51)
Mongolia	2035	32 (-3–68)	1 (0–2)	899 (-366–2165)	29 (5–52)
Mongolia	2036	33 (-7–72)	1 (0–2)	895 (-504–2294)	29 (3–54)
Montenegro	2022	19 (18–20)	2 (2–2)	457 (434–480)	46 (44–48)

Montenegro	2023	19 (18–21)	2 (2–2)	409 (357–460)	41 (36–46)
Montenegro	2024	19 (17–21)	2 (2–2)	360 (274–447)	36 (27–44)
Montenegro	2025	19 (17–22)	2 (2–2)	312 (186–438)	31 (18–43)
Montenegro	2026	19 (16–22)	2 (2–2)	264 (93–434)	26 (9–43)
Montenegro	2027	19 (16–23)	2 (2–2)	215 (-4–435)	21 (-1–43)
Montenegro	2028	19 (15–23)	2 (2–2)	167 (-105–440)	16 (-11–43)
Montenegro	2029	19 (15–24)	2 (2–2)	119 (-210–448)	11 (-22–43)
Montenegro	2030	19 (14–24)	2 (2–2)	70 (-318–459)	6 (-33–44)
Montenegro	2031	19 (13–25)	2 (2–2)	22 (-430–474)	1 (-44–45)
Montenegro	2032	19 (13–25)	2 (2–2)	-26 (-544–492)	-5 (-56–47)
Montenegro	2033	19 (12–26)	2 (2–2)	-75 (-662–513)	-10 (-68–49)
Montenegro	2034	19 (11–26)	2 (2–2)	-123 (-782–536)	-15 (-80–51)
Montenegro	2035	19 (11–27)	2 (2–2)	-171 (-905–562)	-20 (-92–53)
Montenegro	2036	19 (10–27)	2 (2–2)	-220 (-1031–591)	-25 (-105–56)
Morocco	2022	58 (57–58)	0 (0–0)	1612 (1598–1626)	4 (4–4)
Morocco	2023	59 (58–61)	0 (0–0)	1658 (1626–1690)	4 (4–4)
Morocco	2024	61 (59–63)	0 (0–0)	1704 (1650–1757)	4 (4–4)
Morocco	2025	63 (60–66)	0 (0–0)	1750 (1672–1828)	4 (4–5)
Morocco	2026	65 (61–68)	0 (0–0)	1796 (1690–1902)	4 (4–5)
Morocco	2027	66 (61–71)	0 (0–0)	1842 (1706–1978)	4 (4–5)
Morocco	2028	68 (62–74)	0 (0–0)	1888 (1719–2057)	4 (4–5)
Morocco	2029	70 (62–77)	0 (0–0)	1934 (1730–2138)	4 (4–5)
Morocco	2030	71 (62–80)	0 (0–0)	1980 (1739–2221)	4 (4–5)
Morocco	2031	73 (63–83)	0 (0–0)	2026 (1745–2306)	4 (3–5)
Morocco	2032	75 (63–86)	0 (0–0)	2072 (1750–2393)	4 (3–5)
Morocco	2033	76 (63–89)	0 (0–0)	2118 (1753–2482)	4 (3–6)
Morocco	2034	78 (63–93)	0 (0–0)	2164 (1755–2572)	4 (3–6)
Morocco	2035	80 (63–96)	0 (0–0)	2209 (1754–2665)	5 (3–6)
Morocco	2036	81 (63–99)	0 (0–0)	2255 (1752–2758)	5 (3–6)
Mozambique	2022	6 (6–6)	0 (0–0)	175 (174–176)	1 (1–1)
Mozambique	2023	6 (6–7)	0 (0–0)	176 (173–180)	1 (1–2)
Mozambique	2024	6 (6–7)	0 (0–0)	178 (172–183)	1 (1–2)
Mozambique	2025	6 (6–7)	0 (0–0)	179 (171–187)	1 (1–2)

Mozambique	2026	7 (6–7)	0 (0–0)	181 (170–191)	1 (1–2)
Mozambique	2027	7 (6–7)	0 (0–0)	182 (168–196)	1 (1–2)
Mozambique	2028	7 (6–7)	0 (0–0)	183 (166–200)	1 (1–2)
Mozambique	2029	7 (6–7)	0 (0–0)	185 (164–205)	1 (1–2)
Mozambique	2030	7 (5–8)	0 (0–0)	186 (162–210)	1 (1–2)
Mozambique	2031	7 (5–8)	0 (0–0)	187 (159–216)	1 (1–2)
Mozambique	2032	7 (5–8)	0 (0–0)	189 (157–221)	1 (1–2)
Mozambique	2033	7 (5–8)	0 (0–0)	190 (154–227)	1 (1–2)
Mozambique	2034	7 (5–9)	0 (0–0)	192 (151–233)	1 (1–2)
Mozambique	2035	7 (5–9)	0 (0–0)	193 (147–239)	1 (1–2)
Mozambique	2036	7 (4–9)	0 (0–0)	194 (144–245)	1 (1–2)
Myanmar	2022	224 (222–226)	0 (0–0)	5822 (5777–5868)	11 (11–11)
Myanmar	2023	230 (226–233)	0 (0–0)	5971 (5886–6057)	11 (11–11)
Myanmar	2024	236 (230–241)	0 (0–0)	6122 (5985–6259)	11 (11–11)
Myanmar	2025	242 (234–250)	0 (0–0)	6272 (6077–6466)	11 (11–11)
Myanmar	2026	247 (237–258)	0 (0–0)	6422 (6162–6681)	11 (10–12)
Myanmar	2027	253 (240–267)	0 (0–0)	6572 (6242–6901)	11 (10–12)
Myanmar	2028	259 (242–276)	0 (0–0)	6722 (6317–7127)	11 (10–12)
Myanmar	2029	265 (245–285)	0 (0–0)	6872 (6386–7357)	11 (10–12)
Myanmar	2030	271 (247–294)	0 (0–1)	7022 (6451–7592)	11 (10–12)
Myanmar	2031	277 (249–304)	0 (0–1)	7172 (6511–7832)	11 (10–12)
Myanmar	2032	282 (251–314)	0 (0–1)	7322 (6567–8076)	11 (10–12)
Myanmar	2033	288 (253–323)	0 (0–1)	7471 (6619–8324)	11 (9–13)
Myanmar	2034	294 (255–334)	0 (0–1)	7621 (6667–8576)	11 (9–13)
Myanmar	2035	300 (256–344)	0 (0–1)	7771 (6711–8832)	11 (9–13)
Myanmar	2036	306 (257–354)	0 (0–1)	7921 (6751–9092)	11 (9–13)
Namibia	2022	2 (2–3)	0 (0–0)	64 (63–66)	4 (4–5)
Namibia	2023	2 (2–3)	0 (0–0)	65 (63–68)	4 (4–5)
Namibia	2024	3 (2–3)	0 (0–0)	66 (63–70)	4 (4–5)
Namibia	2025	3 (2–3)	0 (0–0)	67 (62–72)	4 (4–5)
Namibia	2026	3 (2–3)	0 (0–0)	68 (62–74)	5 (4–5)
Namibia	2027	3 (2–3)	0 (0–0)	69 (61–77)	5 (4–5)
Namibia	2028	3 (2–3)	0 (0–0)	70 (61–79)	5 (4–5)

Namibia	2029	3 (2–3)	0 (0–0)	71 (61–81)	5 (4–5)
Namibia	2030	3 (2–3)	0 (0–0)	72 (61–84)	5 (4–5)
Namibia	2031	3 (2–3)	0 (0–0)	73 (60–86)	5 (4–5)
Namibia	2032	3 (2–3)	0 (0–0)	74 (60–88)	5 (4–5)
Namibia	2033	3 (2–3)	0 (0–0)	75 (60–90)	5 (4–5)
Namibia	2034	3 (2–3)	0 (0–0)	76 (60–92)	5 (4–5)
Namibia	2035	3 (2–4)	0 (0–0)	77 (60–94)	5 (4–5)
Namibia	2036	3 (2–4)	0 (0–0)	78 (61–96)	5 (4–5)
Nauru	2022	0 (0–0)	1 (1–1)	2 (2–2)	25 (25–25)
Nauru	2023	0 (0–0)	1 (1–1)	2 (2–2)	25 (25–26)
Nauru	2024	0 (0–0)	1 (1–1)	2 (2–2)	25 (24–26)
Nauru	2025	0 (0–0)	1 (1–1)	2 (2–2)	25 (24–26)
Nauru	2026	0 (0–0)	1 (1–1)	2 (2–2)	25 (24–27)
Nauru	2027	0 (0–0)	1 (1–1)	2 (2–2)	25 (24–27)
Nauru	2028	0 (0–0)	1 (1–1)	2 (2–2)	25 (23–27)
Nauru	2029	0 (0–0)	1 (1–1)	2 (2–2)	25 (23–27)
Nauru	2030	0 (0–0)	1 (1–1)	2 (2–2)	25 (23–28)
Nauru	2031	0 (0–0)	1 (1–1)	2 (2–2)	25 (23–28)
Nauru	2032	0 (0–0)	1 (1–1)	2 (2–2)	25 (23–28)
Nauru	2033	0 (0–0)	1 (1–1)	2 (2–2)	25 (22–28)
Nauru	2034	0 (0–0)	1 (1–1)	2 (2–2)	25 (22–28)
Nauru	2035	0 (0–0)	1 (1–1)	2 (2–2)	25 (22–28)
Nauru	2036	0 (0–0)	1 (1–1)	2 (2–2)	25 (22–29)
Nepal	2022	53 (52–53)	0 (0–0)	1259 (1248–1270)	5 (5–5)
Nepal	2023	54 (53–55)	0 (0–0)	1293 (1269–1317)	5 (5–5)
Nepal	2024	56 (54–57)	0 (0–0)	1327 (1287–1366)	5 (5–6)
Nepal	2025	57 (55–59)	0 (0–0)	1361 (1303–1419)	5 (5–6)
Nepal	2026	58 (55–62)	0 (0–0)	1395 (1317–1473)	5 (5–6)
Nepal	2027	60 (56–64)	0 (0–0)	1429 (1328–1529)	5 (5–6)
Nepal	2028	61 (56–66)	0 (0–0)	1463 (1338–1587)	6 (5–6)
Nepal	2029	63 (56–69)	0 (0–0)	1497 (1346–1647)	6 (5–6)
Nepal	2030	64 (57–71)	0 (0–0)	1530 (1352–1709)	6 (5–7)
Nepal	2031	65 (57–74)	0 (0–0)	1564 (1357–1771)	6 (5–7)

Nepal	2032	67 (57–76)	0 (0–0)	1598 (1361–1836)	6 (5–7)
Nepal	2033	68 (57–79)	0 (0–0)	1632 (1363–1901)	6 (4–7)
Nepal	2034	69 (57–82)	0 (0–0)	1666 (1364–1968)	6 (4–7)
Nepal	2035	71 (57–84)	0 (0–0)	1700 (1364–2036)	6 (4–8)
Nepal	2036	72 (57–87)	0 (0–0)	1734 (1362–2106)	6 (4–8)
Netherlands	2022	497 (482–512)	1 (1–1)	10645 (10264–11027)	31 (30–32)
Netherlands	2023	495 (471–519)	1 (1–1)	10645 (10106–11185)	30 (28–32)
Netherlands	2024	494 (461–527)	1 (1–1)	10645 (9985–11306)	30 (27–33)
Netherlands	2025	493 (451–534)	1 (1–1)	10645 (9882–11408)	29 (25–33)
Netherlands	2026	492 (441–542)	1 (1–1)	10645 (9792–11498)	28 (24–33)
Netherlands	2027	491 (432–550)	1 (1–1)	10645 (9711–11580)	27 (22–33)
Netherlands	2028	490 (422–557)	1 (1–1)	10645 (9636–11655)	27 (21–33)
Netherlands	2029	489 (413–565)	1 (1–1)	10645 (9566–11724)	26 (19–33)
Netherlands	2030	489 (405–572)	1 (1–1)	10645 (9501–11790)	25 (18–33)
Netherlands	2031	488 (396–580)	1 (1–1)	10645 (9439–11852)	25 (17–33)
Netherlands	2032	488 (388–587)	1 (1–1)	10645 (9380–11911)	24 (16–33)
Netherlands	2033	487 (380–594)	1 (1–1)	10645 (9324–11967)	24 (15–33)
Netherlands	2034	487 (373–601)	1 (1–1)	10645 (9270–12021)	23 (13–32)
Netherlands	2035	487 (365–608)	1 (1–1)	10645 (9218–12073)	22 (12–32)
Netherlands	2036	486 (358–615)	1 (1–1)	10645 (9168–12123)	22 (11–32)
New Zealand	2022	81 (78–84)	1 (1–1)	1820 (1749–1891)	22 (21–24)
New Zealand	2023	82 (78–87)	1 (1–1)	1854 (1746–1963)	22 (20–24)
New Zealand	2024	84 (78–90)	1 (1–1)	1889 (1744–2033)	22 (19–25)
New Zealand	2025	86 (78–93)	1 (1–1)	1923 (1743–2103)	22 (19–25)
New Zealand	2026	87 (78–96)	1 (1–1)	1957 (1742–2173)	22 (18–26)
New Zealand	2027	89 (78–100)	1 (1–1)	1992 (1739–2244)	22 (17–26)
New Zealand	2028	91 (78–103)	1 (1–1)	2026 (1736–2316)	22 (17–27)
New Zealand	2029	92 (78–106)	1 (1–1)	2060 (1731–2389)	22 (16–27)
New Zealand	2030	94 (78–110)	1 (1–1)	2095 (1725–2464)	22 (15–28)
New Zealand	2031	96 (78–113)	1 (1–1)	2129 (1718–2540)	21 (15–28)
New Zealand	2032	97 (78–117)	1 (1–1)	2163 (1710–2616)	21 (14–29)
New Zealand	2033	99 (78–121)	1 (1–1)	2198 (1701–2694)	21 (13–29)
New Zealand	2034	101 (77–124)	1 (1–1)	2232 (1690–2774)	21 (13–30)

New Zealand	2035	102 (77–128)	1 (1–1)	2266 (1679–2854)	21 (12–30)
New Zealand	2036	104 (77–132)	1 (1–1)	2301 (1666–2935)	21 (11–31)
Nicaragua	2022	12 (12–13)	0 (0–0)	338 (324–352)	6 (6–7)
Nicaragua	2023	13 (12–13)	0 (0–0)	346 (329–363)	6 (6–7)
Nicaragua	2024	13 (12–14)	0 (0–0)	354 (335–373)	6 (5–7)
Nicaragua	2025	13 (13–14)	0 (0–0)	362 (341–384)	6 (5–7)
Nicaragua	2026	14 (13–15)	0 (0–0)	371 (347–394)	6 (5–7)
Nicaragua	2027	14 (13–15)	0 (0–0)	379 (354–404)	6 (4–7)
Nicaragua	2028	14 (13–15)	0 (0–0)	387 (360–413)	6 (4–7)
Nicaragua	2029	15 (14–16)	0 (0–0)	395 (367–423)	6 (4–7)
Nicaragua	2030	15 (14–16)	0 (0–0)	403 (374–433)	5 (3–7)
Nicaragua	2031	15 (14–16)	0 (0–0)	411 (380–442)	5 (3–8)
Nicaragua	2032	16 (14–17)	0 (0–0)	420 (387–452)	5 (3–8)
Nicaragua	2033	16 (15–17)	0 (0–0)	428 (394–461)	5 (2–8)
Nicaragua	2034	16 (15–17)	0 (0–0)	436 (401–471)	5 (2–8)
Nicaragua	2035	16 (15–18)	0 (0–0)	444 (408–480)	5 (2–8)
Nicaragua	2036	17 (15–18)	0 (0–0)	452 (415–490)	5 (1–8)
Niger	2022	4 (4–4)	0 (0–0)	109 (108–111)	1 (1–1)
Niger	2023	4 (4–4)	0 (0–0)	112 (110–115)	1 (1–1)
Niger	2024	4 (4–4)	0 (0–0)	116 (112–120)	1 (1–1)
Niger	2025	4 (4–5)	0 (0–0)	119 (113–124)	1 (1–1)
Niger	2026	4 (4–5)	0 (0–0)	122 (115–129)	1 (1–1)
Niger	2027	5 (4–5)	0 (0–0)	125 (116–134)	1 (1–1)
Niger	2028	5 (4–5)	0 (0–0)	128 (117–140)	1 (1–1)
Niger	2029	5 (4–5)	0 (0–0)	132 (118–145)	1 (1–1)
Niger	2030	5 (4–5)	0 (0–0)	135 (119–150)	1 (1–1)
Niger	2031	5 (4–6)	0 (0–0)	138 (120–156)	1 (1–1)
Niger	2032	5 (4–6)	0 (0–0)	141 (120–162)	1 (1–1)
Niger	2033	5 (4–6)	0 (0–0)	144 (121–168)	1 (1–1)
Niger	2034	5 (4–6)	0 (0–0)	147 (121–174)	1 (1–1)
Niger	2035	5 (4–6)	0 (0–0)	151 (122–180)	1 (1–1)
Niger	2036	6 (4–7)	0 (0–0)	154 (122–186)	1 (1–1)
Nigeria	2022	17 (17–17)	0 (0–0)	488 (482–493)	0 (0–0)

Nigeria	2023	18 (17–18)	0 (0–0)	500 (491–509)	0 (0–0)
Nigeria	2024	18 (18–19)	0 (0–0)	512 (499–525)	0 (0–0)
Nigeria	2025	19 (18–20)	0 (0–0)	524 (507–542)	0 (0–1)
Nigeria	2026	19 (18–21)	0 (0–0)	536 (514–559)	0 (0–1)
Nigeria	2027	20 (18–21)	0 (0–0)	549 (521–576)	0 (0–1)
Nigeria	2028	20 (18–22)	0 (0–0)	561 (528–593)	0 (0–1)
Nigeria	2029	21 (18–23)	0 (0–0)	573 (535–611)	0 (0–1)
Nigeria	2030	21 (18–24)	0 (0–0)	585 (541–629)	0 (0–1)
Nigeria	2031	22 (18–25)	0 (0–0)	597 (548–647)	0 (0–1)
Nigeria	2032	22 (18–26)	0 (0–0)	609 (554–665)	0 (0–1)
Nigeria	2033	23 (18–27)	0 (0–0)	622 (559–684)	0 (0–1)
Nigeria	2034	23 (18–28)	0 (0–0)	634 (565–703)	0 (0–1)
Nigeria	2035	24 (18–29)	0 (0–0)	646 (570–722)	0 (0–1)
Nigeria	2036	24 (18–30)	0 (0–0)	658 (575–741)	0 (0–1)
Niue	2022	0 (0–0)	1 (1–1)	0 (0–0)	16 (16–16)
Niue	2023	0 (0–0)	1 (1–1)	0 (0–0)	16 (16–16)
Niue	2024	0 (0–0)	1 (1–1)	0 (0–0)	16 (16–17)
Niue	2025	0 (0–0)	1 (1–1)	0 (0–0)	16 (16–17)
Niue	2026	0 (0–0)	1 (1–1)	0 (0–0)	16 (16–17)
Niue	2027	0 (0–0)	1 (1–1)	0 (0–0)	17 (15–18)
Niue	2028	0 (0–0)	1 (1–1)	0 (0–0)	17 (15–18)
Niue	2029	0 (0–0)	1 (1–1)	0 (0–0)	17 (15–19)
Niue	2030	0 (0–0)	1 (1–1)	0 (0–0)	17 (15–19)
Niue	2031	0 (0–0)	1 (1–1)	0 (0–0)	17 (15–20)
Niue	2032	0 (0–0)	1 (1–1)	0 (0–0)	17 (14–20)
Niue	2033	0 (0–0)	1 (1–1)	0 (0–0)	17 (14–21)
Niue	2034	0 (0–0)	1 (1–1)	0 (0–0)	18 (14–21)
Niue	2035	0 (0–0)	1 (1–1)	0 (0–1)	18 (14–22)
Niue	2036	0 (0–0)	1 (1–1)	0 (0–1)	18 (13–22)
North Macedonia	2022	55 (53–57)	2 (2–2)	1447 (1394–1500)	42 (40–44)
North Macedonia	2023	55 (52–58)	2 (1–2)	1442 (1359–1526)	41 (38–44)
North Macedonia	2024	55 (51–59)	2 (1–2)	1438 (1326–1551)	40 (36–43)
North Macedonia	2025	55 (49–60)	2 (1–2)	1434 (1292–1576)	39 (35–43)

North Macedonia	2026	55 (48–61)	1 (1–2)	1430 (1257–1602)	38 (33–43)
North Macedonia	2027	54 (47–62)	1 (1–2)	1426 (1221–1630)	37 (31–43)
North Macedonia	2028	54 (45–63)	1 (1–2)	1421 (1184–1659)	36 (29–43)
North Macedonia	2029	54 (44–64)	1 (1–2)	1417 (1145–1689)	35 (27–44)
North Macedonia	2030	54 (42–65)	1 (1–2)	1413 (1105–1720)	34 (25–44)
North Macedonia	2031	54 (40–67)	1 (1–2)	1409 (1064–1753)	33 (23–44)
North Macedonia	2032	53 (39–68)	1 (1–2)	1404 (1022–1787)	33 (21–44)
North Macedonia	2033	53 (37–69)	1 (1–2)	1400 (979–1822)	32 (19–45)
North Macedonia	2034	53 (35–71)	1 (0–2)	1396 (934–1858)	31 (16–45)
North Macedonia	2035	53 (33–72)	1 (0–2)	1392 (888–1895)	30 (14–46)
North Macedonia	2036	53 (32–74)	1 (0–2)	1387 (841–1934)	29 (12–46)
Northern Mariana Islands	2022	1 (0–1)	1 (1–1)	15 (15–16)	23 (21–24)
Northern Mariana Islands	2023	1 (0–1)	1 (1–1)	15 (14–16)	22 (20–25)
Northern Mariana Islands	2024	1 (0–1)	1 (1–1)	15 (14–17)	22 (19–25)
Northern Mariana Islands	2025	1 (0–1)	1 (1–1)	16 (14–17)	22 (17–26)
Northern Mariana Islands	2026	1 (1–1)	1 (1–1)	16 (14–18)	22 (16–27)
Northern Mariana Islands	2027	1 (1–1)	1 (1–1)	16 (14–19)	22 (16–28)
Northern Mariana Islands	2028	1 (1–1)	1 (1–1)	17 (14–19)	22 (15–28)
Northern Mariana Islands	2029	1 (1–1)	1 (1–1)	17 (14–20)	22 (14–29)
Northern Mariana Islands	2030	1 (1–1)	1 (0–1)	17 (15–20)	22 (13–30)
Northern Mariana Islands	2031	1 (1–1)	1 (0–1)	18 (15–21)	22 (13–30)
Northern Mariana Islands	2032	1 (1–1)	1 (0–1)	18 (15–21)	22 (12–31)
Northern Mariana Islands	2033	1 (1–1)	1 (0–1)	18 (15–22)	22 (12–31)
Northern Mariana Islands	2034	1 (1–1)	1 (0–1)	19 (15–22)	22 (11–32)
Northern Mariana Islands	2035	1 (1–1)	1 (0–1)	19 (16–23)	22 (11–32)
Northern Mariana Islands	2036	1 (1–1)	1 (0–1)	19 (16–23)	22 (10–33)
Norway	2022	92 (88–96)	1 (1–1)	2030 (1951–2110)	22 (21–23)
Norway	2023	92 (86–99)	1 (1–1)	2013 (1901–2125)	21 (20–23)
Norway	2024	93 (84–102)	1 (1–1)	1996 (1859–2134)	21 (19–23)
Norway	2025	93 (82–104)	1 (1–1)	1979 (1821–2138)	20 (18–22)
Norway	2026	93 (80–106)	1 (1–1)	1962 (1785–2139)	19 (17–22)
Norway	2027	93 (78–107)	1 (1–1)	1945 (1751–2139)	19 (16–22)
Norway	2028	93 (77–109)	1 (1–1)	1928 (1719–2138)	18 (15–21)

Norway	2029	93 (75–110)	1 (1–1)	1911 (1687–2135)	18 (14–21)
Norway	2030	93 (74–111)	1 (1–1)	1894 (1657–2132)	17 (13–21)
Norway	2031	93 (73–113)	1 (0–1)	1877 (1627–2128)	16 (12–20)
Norway	2032	93 (72–114)	1 (0–1)	1860 (1598–2123)	16 (12–20)
Norway	2033	93 (71–115)	1 (0–1)	1843 (1569–2117)	15 (11–19)
Norway	2034	93 (69–116)	1 (0–1)	1826 (1541–2112)	14 (10–19)
Norway	2035	93 (69–117)	1 (0–1)	1809 (1513–2105)	14 (9–18)
Norway	2036	93 (68–118)	1 (0–1)	1792 (1485–2099)	13 (8–18)
Oman	2022	3 (3–3)	0 (0–0)	89 (85–93)	4 (4–4)
Oman	2023	3 (3–3)	0 (0–0)	92 (86–97)	4 (3–4)
Oman	2024	3 (3–3)	0 (0–0)	89 (79–99)	4 (3–4)
Oman	2025	3 (3–4)	0 (0–0)	90 (76–104)	4 (3–4)
Oman	2026	3 (2–4)	0 (0–0)	88 (70–107)	4 (3–4)
Oman	2027	3 (2–4)	0 (0–0)	89 (65–112)	4 (3–5)
Oman	2028	3 (2–4)	0 (0–0)	88 (58–117)	4 (3–5)
Oman	2029	3 (2–4)	0 (0–0)	88 (53–122)	4 (2–5)
Oman	2030	3 (2–4)	0 (0–0)	87 (45–128)	4 (2–5)
Oman	2031	3 (2–5)	0 (0–0)	87 (39–134)	4 (2–5)
Oman	2032	3 (1–5)	0 (0–0)	86 (31–140)	4 (2–5)
Oman	2033	3 (1–5)	0 (0–0)	86 (24–147)	4 (2–5)
Oman	2034	3 (1–5)	0 (0–0)	85 (16–154)	4 (2–5)
Oman	2035	3 (1–6)	0 (0–0)	85 (7–162)	4 (2–5)
Oman	2036	3 (1–6)	0 (0–0)	84 (-1–169)	4 (2–5)
Pakistan	2022	221 (219–223)	0 (0–0)	6072 (6024–6121)	5 (4–5)
Pakistan	2023	225 (221–229)	0 (0–0)	6205 (6105–6305)	5 (4–5)
Pakistan	2024	229 (224–235)	0 (0–0)	6334 (6178–6490)	4 (4–5)
Pakistan	2025	233 (226–241)	0 (0–0)	6460 (6246–6674)	4 (4–5)
Pakistan	2026	238 (228–248)	0 (0–0)	6584 (6312–6855)	4 (4–5)
Pakistan	2027	242 (230–254)	0 (0–0)	6706 (6377–7034)	4 (4–5)
Pakistan	2028	246 (232–260)	0 (0–0)	6826 (6443–7209)	4 (4–5)
Pakistan	2029	250 (234–266)	0 (0–0)	6945 (6508–7382)	4 (3–5)
Pakistan	2030	254 (236–272)	0 (0–0)	7063 (6574–7552)	4 (3–5)
Pakistan	2031	258 (238–278)	0 (0–0)	7180 (6641–7719)	4 (3–6)

Pakistan	2032	262 (240–284)	0 (0–0)	7297 (6710–7883)	4 (3–6)
Pakistan	2033	266 (242–290)	0 (0–0)	7413 (6780–8045)	4 (3–6)
Pakistan	2034	270 (244–295)	0 (0–0)	7528 (6851–8205)	4 (2–6)
Pakistan	2035	274 (247–301)	0 (0–0)	7643 (6923–8363)	4 (2–6)
Pakistan	2036	278 (249–306)	0 (0–0)	7758 (6997–8519)	4 (2–6)
Palau	2022	0 (0–0)	1 (1–1)	3 (3–3)	13 (13–13)
Palau	2023	0 (0–0)	1 (1–1)	3 (3–4)	13 (13–13)
Palau	2024	0 (0–0)	1 (1–1)	4 (3–4)	13 (12–13)
Palau	2025	0 (0–0)	1 (1–1)	4 (3–4)	13 (12–13)
Palau	2026	0 (0–0)	1 (1–1)	4 (4–4)	13 (12–14)
Palau	2027	0 (0–0)	1 (1–1)	4 (4–4)	13 (12–14)
Palau	2028	0 (0–0)	1 (1–1)	4 (4–4)	13 (12–14)
Palau	2029	0 (0–0)	1 (1–1)	4 (4–4)	13 (11–14)
Palau	2030	0 (0–0)	1 (1–1)	4 (4–4)	13 (11–14)
Palau	2031	0 (0–0)	1 (1–1)	4 (4–5)	13 (11–14)
Palau	2032	0 (0–0)	1 (1–1)	4 (4–5)	13 (11–14)
Palau	2033	0 (0–0)	1 (1–1)	4 (4–5)	13 (10–15)
Palau	2034	0 (0–0)	1 (1–1)	4 (4–5)	13 (10–15)
Palau	2035	0 (0–0)	1 (1–1)	4 (4–5)	12 (10–15)
Palau	2036	0 (0–0)	1 (1–1)	4 (4–5)	12 (10–15)
Palestine	2022	21 (20–21)	1 (1–1)	580 (567–593)	20 (20–21)
Palestine	2023	22 (21–23)	1 (1–1)	606 (585–628)	20 (19–21)
Palestine	2024	23 (22–24)	1 (1–1)	633 (602–664)	20 (19–21)
Palestine	2025	24 (22–26)	1 (1–1)	660 (619–701)	20 (19–22)
Palestine	2026	25 (23–27)	1 (1–1)	687 (635–738)	20 (18–22)
Palestine	2027	26 (23–28)	1 (1–1)	713 (650–777)	20 (18–22)
Palestine	2028	27 (24–30)	1 (1–1)	740 (665–815)	20 (18–22)
Palestine	2029	28 (24–31)	1 (1–1)	767 (679–855)	20 (18–22)
Palestine	2030	29 (25–33)	1 (1–1)	793 (692–895)	20 (18–22)
Palestine	2031	30 (25–34)	1 (1–1)	820 (704–936)	20 (18–23)
Palestine	2032	31 (25–36)	1 (1–1)	847 (717–977)	20 (18–23)
Palestine	2033	32 (26–38)	1 (1–1)	874 (728–1019)	20 (17–23)
Palestine	2034	33 (26–39)	1 (1–1)	900 (739–1061)	20 (17–23)

Palestine	2035	34 (26–41)	1 (1–1)	927 (750–1104)	20 (17–23)
Palestine	2036	35 (27–43)	1 (1–1)	954 (759–1148)	20 (17–23)
Panama	2022	12 (11–12)	0 (0–0)	279 (264–294)	6 (5–7)
Panama	2023	12 (11–13)	0 (0–0)	285 (264–306)	6 (5–7)
Panama	2024	12 (11–14)	0 (0–0)	291 (266–317)	6 (5–8)
Panama	2025	13 (11–14)	0 (0–0)	297 (268–327)	6 (5–8)
Panama	2026	13 (11–15)	0 (0–0)	303 (270–336)	6 (5–8)
Panama	2027	13 (11–15)	0 (0–0)	309 (273–346)	6 (5–8)
Panama	2028	13 (11–15)	0 (0–0)	315 (276–355)	6 (5–8)
Panama	2029	14 (11–16)	0 (0–0)	322 (280–363)	6 (5–8)
Panama	2030	14 (12–16)	0 (0–0)	328 (283–372)	6 (5–8)
Panama	2031	14 (12–17)	0 (0–0)	334 (287–380)	7 (5–8)
Panama	2032	14 (12–17)	0 (0–0)	340 (291–389)	7 (5–8)
Panama	2033	15 (12–17)	0 (0–0)	346 (294–397)	7 (5–8)
Panama	2034	15 (12–18)	0 (0–0)	352 (298–405)	7 (5–8)
Panama	2035	15 (12–18)	0 (0–0)	358 (302–413)	7 (5–8)
Panama	2036	15 (12–19)	0 (0–0)	364 (307–421)	7 (5–8)
Papua New Guinea	2022	12 (11–12)	0 (0–0)	371 (365–378)	6 (6–6)
Papua New Guinea	2023	12 (12–12)	0 (0–0)	386 (375–398)	6 (5–6)
Papua New Guinea	2024	12 (12–13)	0 (0–0)	401 (383–418)	6 (5–6)
Papua New Guinea	2025	13 (12–14)	0 (0–0)	415 (392–439)	6 (5–6)
Papua New Guinea	2026	13 (12–15)	0 (0–0)	430 (399–460)	6 (5–6)
Papua New Guinea	2027	14 (12–15)	0 (0–0)	444 (406–482)	6 (5–6)
Papua New Guinea	2028	14 (12–16)	0 (0–0)	459 (413–505)	6 (5–6)
Papua New Guinea	2029	14 (12–17)	0 (0–0)	474 (419–528)	6 (5–6)
Papua New Guinea	2030	15 (12–18)	0 (0–0)	488 (425–552)	6 (5–6)
Papua New Guinea	2031	15 (11–19)	0 (0–0)	503 (430–576)	5 (5–6)
Papua New Guinea	2032	15 (11–20)	0 (0–0)	517 (435–600)	5 (5–6)
Papua New Guinea	2033	16 (11–20)	0 (0–0)	532 (439–625)	5 (5–6)
Papua New Guinea	2034	16 (11–21)	0 (0–0)	547 (443–650)	5 (5–6)
Papua New Guinea	2035	16 (11–22)	0 (0–0)	561 (447–676)	5 (5–6)
Papua New Guinea	2036	17 (10–23)	0 (0–0)	576 (450–702)	5 (5–6)
Paraguay	2022	51 (49–52)	1 (1–1)	1203 (1171–1235)	20 (19–21)

Paraguay	2023	52 (50–54)	1 (1–1)	1232 (1187–1277)	20 (18–21)
Paraguay	2024	53 (51–55)	1 (1–1)	1261 (1206–1317)	20 (18–22)
Paraguay	2025	54 (51–57)	1 (1–1)	1290 (1226–1354)	20 (17–22)
Paraguay	2026	55 (52–59)	1 (1–1)	1320 (1248–1391)	19 (16–23)
Paraguay	2027	57 (53–60)	1 (1–1)	1349 (1271–1427)	19 (16–23)
Paraguay	2028	58 (54–62)	1 (1–1)	1378 (1294–1463)	19 (15–24)
Paraguay	2029	59 (55–63)	1 (1–1)	1407 (1317–1498)	19 (14–24)
Paraguay	2030	60 (56–65)	1 (0–1)	1437 (1341–1533)	19 (13–25)
Paraguay	2031	62 (57–66)	1 (0–1)	1466 (1365–1567)	19 (12–26)
Paraguay	2032	63 (58–68)	1 (0–1)	1495 (1389–1601)	19 (11–26)
Paraguay	2033	64 (59–69)	1 (0–1)	1524 (1414–1635)	19 (10–27)
Paraguay	2034	65 (60–70)	1 (0–1)	1554 (1438–1669)	19 (9–28)
Paraguay	2035	67 (61–72)	1 (0–1)	1583 (1463–1702)	19 (8–29)
Paraguay	2036	68 (62–73)	1 (0–1)	1612 (1488–1736)	19 (7–30)
Peru	2022	114 (108–120)	0 (0–0)	2800 (2656–2944)	8 (7–9)
Peru	2023	116 (106–126)	0 (0–0)	2847 (2597–3097)	8 (7–9)
Peru	2024	118 (104–132)	0 (0–0)	2899 (2559–3238)	8 (7–9)
Peru	2025	120 (103–137)	0 (0–0)	2953 (2537–3369)	8 (7–9)
Peru	2026	123 (103–142)	0 (0–0)	3008 (2526–3490)	8 (7–9)
Peru	2027	125 (103–147)	0 (0–0)	3063 (2522–3605)	8 (7–9)
Peru	2028	127 (103–152)	0 (0–0)	3119 (2523–3714)	8 (7–9)
Peru	2029	130 (103–156)	0 (0–0)	3174 (2529–3819)	8 (7–9)
Peru	2030	132 (104–160)	0 (0–0)	3230 (2539–3921)	8 (7–9)
Peru	2031	134 (104–164)	0 (0–0)	3286 (2552–4020)	8 (7–9)
Peru	2032	137 (105–168)	0 (0–0)	3341 (2566–4116)	8 (7–9)
Peru	2033	139 (106–172)	0 (0–0)	3397 (2583–4210)	8 (7–9)
Peru	2034	141 (107–176)	0 (0–0)	3452 (2602–4303)	8 (7–9)
Peru	2035	144 (107–180)	0 (0–0)	3508 (2622–4394)	8 (7–9)
Peru	2036	146 (108–183)	0 (0–0)	3564 (2644–4483)	8 (7–9)
Philippines	2022	457 (451–463)	1 (1–1)	13057 (12853–13260)	14 (14–14)
Philippines	2023	470 (462–477)	1 (1–1)	13326 (13038–13614)	14 (14–15)
Philippines	2024	482 (472–493)	1 (1–1)	13596 (13243–13949)	14 (14–15)
Philippines	2025	495 (480–510)	1 (1–1)	13865 (13458–14273)	14 (14–15)

Philippines	2026	507 (488–527)	1 (1–1)	14135 (13680–14590)	14 (14–15)
Philippines	2027	520 (494–546)	1 (1–1)	14405 (13906–14904)	14 (14–15)
Philippines	2028	533 (501–564)	1 (1–1)	14674 (14136–15213)	14 (14–15)
Philippines	2029	545 (507–584)	1 (1–1)	14944 (14368–15520)	15 (14–15)
Philippines	2030	558 (512–604)	1 (0–1)	15214 (14603–15825)	15 (14–16)
Philippines	2031	571 (517–624)	1 (0–1)	15483 (14839–16127)	15 (14–16)
Philippines	2032	583 (521–645)	1 (0–1)	15753 (15077–16428)	15 (14–16)
Philippines	2033	596 (525–666)	1 (1–1)	16022 (15317–16728)	15 (14–16)
Philippines	2034	608 (529–688)	1 (0–1)	16292 (15558–17026)	15 (14–16)
Philippines	2035	621 (533–709)	1 (0–1)	16562 (15800–17324)	15 (14–16)
Philippines	2036	634 (536–732)	1 (0–1)	16831 (16043–17620)	15 (14–16)
Poland	2022	986 (957–1015)	1 (1–1)	24583 (23696–25470)	37 (35–38)
Poland	2023	992 (952–1033)	1 (1–1)	24298 (22852–25744)	36 (33–38)
Poland	2024	998 (948–1048)	1 (1–1)	24013 (22002–26024)	35 (32–38)
Poland	2025	1004 (947–1062)	1 (1–1)	23728 (21124–26331)	34 (30–38)
Poland	2026	1010 (946–1074)	1 (1–1)	23442 (20214–26671)	33 (28–38)
Poland	2027	1016 (946–1087)	1 (1–1)	23157 (19270–27044)	32 (26–39)
Poland	2028	1022 (946–1098)	1 (1–1)	22872 (18293–27451)	32 (24–39)
Poland	2029	1028 (947–1110)	1 (1–1)	22587 (17283–27890)	31 (23–39)
Poland	2030	1034 (948–1120)	1 (1–1)	22302 (16242–28361)	30 (21–39)
Poland	2031	1040 (950–1131)	1 (1–1)	22016 (15169–28863)	29 (19–39)
Poland	2032	1046 (951–1142)	1 (1–1)	21731 (14067–29395)	28 (17–39)
Poland	2033	1052 (953–1152)	1 (1–1)	21446 (12936–29956)	27 (15–40)
Poland	2034	1058 (955–1162)	1 (1–2)	21161 (11778–30544)	26 (13–40)
Poland	2035	1064 (957–1172)	1 (1–2)	20876 (10591–31160)	26 (11–40)
Poland	2036	1071 (959–1182)	1 (0–2)	20590 (9379–31802)	25 (9–41)
Portugal	2022	162 (155–169)	1 (1–1)	4166 (3961–4371)	22 (20–23)
Portugal	2023	163 (153–174)	1 (1–1)	4195 (3905–4486)	22 (20–23)
Portugal	2024	165 (152–177)	1 (1–1)	4225 (3870–4580)	22 (20–24)
Portugal	2025	166 (151–180)	1 (1–1)	4255 (3844–4665)	22 (20–24)
Portugal	2026	167 (151–183)	1 (1–1)	4284 (3826–4743)	22 (20–24)
Portugal	2027	168 (151–186)	1 (1–1)	4314 (3812–4816)	22 (20–24)
Portugal	2028	170 (150–189)	1 (1–1)	4344 (3801–4886)	22 (21–24)

Portugal	2029	171 (150–191)	1 (1–1)	4373 (3793–4954)	22 (21–24)
Portugal	2030	172 (150–194)	1 (1–1)	4403 (3788–5018)	22 (21–24)
Portugal	2031	173 (150–196)	1 (1–1)	4433 (3784–5081)	22 (21–24)
Portugal	2032	175 (150–199)	1 (1–1)	4462 (3782–5143)	22 (21–24)
Portugal	2033	176 (151–201)	1 (1–1)	4492 (3781–5202)	22 (21–24)
Portugal	2034	177 (151–203)	1 (1–1)	4522 (3782–5261)	22 (21–24)
Portugal	2035	178 (151–205)	1 (1–1)	4551 (3784–5319)	22 (21–24)
Portugal	2036	179 (151–207)	1 (1–1)	4581 (3786–5375)	22 (21–24)
Puerto Rico	2022	42 (39–44)	1 (1–1)	957 (894–1021)	16 (14–17)
Puerto Rico	2023	43 (39–46)	1 (1–1)	975 (885–1065)	16 (14–18)
Puerto Rico	2024	43 (39–48)	1 (1–1)	993 (882–1103)	16 (13–18)
Puerto Rico	2025	44 (39–49)	1 (1–1)	1010 (883–1137)	16 (13–18)
Puerto Rico	2026	45 (39–51)	1 (0–1)	1028 (885–1170)	16 (13–19)
Puerto Rico	2027	46 (40–52)	1 (0–1)	1045 (889–1201)	16 (12–19)
Puerto Rico	2028	47 (40–53)	1 (0–1)	1063 (895–1231)	16 (12–19)
Puerto Rico	2029	47 (40–55)	1 (0–1)	1081 (901–1260)	16 (12–19)
Puerto Rico	2030	48 (41–56)	1 (0–1)	1098 (907–1289)	16 (12–20)
Puerto Rico	2031	49 (41–57)	1 (0–1)	1116 (914–1317)	16 (11–20)
Puerto Rico	2032	50 (41–58)	1 (0–1)	1133 (922–1344)	16 (11–20)
Puerto Rico	2033	51 (42–59)	1 (0–1)	1151 (930–1371)	16 (11–20)
Puerto Rico	2034	51 (42–60)	1 (0–1)	1168 (939–1398)	16 (11–21)
Puerto Rico	2035	52 (43–62)	1 (0–1)	1186 (948–1424)	16 (11–21)
Puerto Rico	2036	53 (43–63)	1 (0–1)	1204 (957–1450)	16 (11–21)
Qatar	2022	5 (5–5)	1 (0–1)	170 (163–176)	13 (11–14)
Qatar	2023	5 (5–6)	1 (0–1)	175 (165–185)	13 (11–15)
Qatar	2024	5 (5–6)	1 (0–1)	170 (154–187)	13 (10–15)
Qatar	2025	6 (5–6)	1 (0–1)	174 (152–196)	13 (10–15)
Qatar	2026	6 (5–7)	1 (0–1)	170 (142–199)	13 (9–16)
Qatar	2027	6 (5–7)	1 (0–1)	173 (138–208)	13 (9–16)
Qatar	2028	6 (5–7)	1 (0–1)	170 (129–212)	13 (9–16)
Qatar	2029	6 (4–7)	1 (0–1)	172 (123–221)	13 (8–17)
Qatar	2030	6 (4–8)	1 (0–1)	170 (115–226)	13 (8–17)
Qatar	2031	6 (4–8)	1 (0–1)	172 (109–234)	13 (8–17)

Qatar	2032	6 (4–9)	1 (0–1)	170 (100–241)	13 (8–17)
Qatar	2033	6 (4–9)	1 (0–1)	171 (93–249)	13 (8–18)
Qatar	2034	6 (4–9)	1 (0–1)	170 (85–255)	13 (7–18)
Qatar	2035	7 (3–10)	1 (0–1)	171 (78–263)	13 (7–18)
Qatar	2036	7 (3–10)	1 (0–1)	170 (70–270)	13 (7–18)
Republic of Korea	2022	1058 (1039–1076)	1 (1–1)	23083 (22584–23582)	24 (23–25)
Republic of Korea	2023	1082 (1056–1108)	1 (1–1)	23490 (22606–24374)	24 (22–26)
Republic of Korea	2024	1106 (1069–1144)	1 (1–1)	23838 (22621–25055)	24 (21–26)
Republic of Korea	2025	1131 (1078–1183)	1 (1–1)	24158 (22653–25663)	24 (20–27)
Republic of Korea	2026	1155 (1086–1224)	1 (1–1)	24465 (22707–26224)	24 (19–28)
Republic of Korea	2027	1180 (1092–1267)	1 (1–1)	24767 (22783–26751)	24 (18–29)
Republic of Korea	2028	1204 (1097–1312)	1 (1–1)	25065 (22877–27254)	24 (17–30)
Republic of Korea	2029	1228 (1099–1358)	1 (1–1)	25363 (22986–27740)	24 (16–31)
Republic of Korea	2030	1253 (1101–1405)	1 (1–1)	25660 (23108–28211)	24 (15–32)
Republic of Korea	2031	1277 (1101–1453)	1 (1–1)	25956 (23241–28671)	23 (14–33)
Republic of Korea	2032	1302 (1100–1503)	1 (1–1)	26253 (23383–29122)	23 (13–34)
Republic of Korea	2033	1326 (1098–1554)	1 (1–1)	26549 (23533–29565)	23 (12–35)
Republic of Korea	2034	1351 (1095–1606)	1 (1–2)	26845 (23690–30001)	23 (11–36)
Republic of Korea	2035	1375 (1091–1659)	1 (1–2)	27142 (23853–30431)	23 (10–37)
Republic of Korea	2036	1399 (1086–1713)	1 (1–2)	27438 (24020–30856)	23 (9–38)
Republic of Moldova	2022	74 (69–80)	1 (1–1)	2117 (1946–2288)	37 (33–40)
Republic of Moldova	2023	75 (67–83)	1 (1–1)	2117 (1876–2359)	37 (32–41)
Republic of Moldova	2024	76 (67–85)	1 (1–1)	2117 (1821–2413)	37 (31–42)
Republic of Moldova	2025	77 (66–87)	1 (1–1)	2117 (1776–2459)	37 (30–43)
Republic of Moldova	2026	77 (65–89)	1 (1–1)	2117 (1735–2499)	37 (29–44)
Republic of Moldova	2027	78 (65–91)	1 (1–1)	2117 (1699–2535)	37 (28–45)
Republic of Moldova	2028	79 (65–93)	1 (1–2)	2117 (1665–2569)	37 (28–45)
Republic of Moldova	2029	80 (65–95)	1 (1–2)	2117 (1634–2600)	37 (27–46)
Republic of Moldova	2030	80 (64–97)	1 (1–2)	2117 (1605–2629)	37 (27–47)
Republic of Moldova	2031	81 (64–98)	1 (1–2)	2117 (1577–2657)	37 (26–47)
Republic of Moldova	2032	82 (64–100)	1 (1–2)	2117 (1551–2684)	37 (25–48)
Republic of Moldova	2033	83 (64–101)	1 (1–2)	2117 (1526–2709)	37 (25–48)
Republic of Moldova	2034	84 (64–103)	1 (1–2)	2117 (1501–2733)	37 (25–49)

Republic of Moldova	2035	84 (64–104)	1 (1–2)	2117 (1478–2756)	37 (24–49)
Republic of Moldova	2036	85 (65–106)	1 (1–2)	2117 (1456–2779)	37 (24–50)
Romania	2022	508 (482–533)	1 (1–2)	13776 (13001–14551)	43 (40–46)
Romania	2023	513 (471–555)	1 (1–2)	13782 (12482–15082)	43 (39–47)
Romania	2024	519 (464–574)	1 (1–2)	13784 (12058–15510)	43 (38–47)
Romania	2025	525 (459–591)	1 (1–2)	13785 (11700–15869)	43 (38–48)
Romania	2026	530 (455–606)	1 (1–2)	13785 (11390–16180)	43 (37–49)
Romania	2027	536 (452–620)	1 (1–2)	13785 (11114–16456)	43 (37–49)
Romania	2028	542 (451–634)	1 (1–2)	13785 (10863–16707)	43 (36–50)
Romania	2029	548 (449–646)	1 (1–2)	13785 (10632–16938)	43 (36–50)
Romania	2030	554 (449–659)	1 (1–2)	13785 (10417–17153)	43 (35–51)
Romania	2031	559 (448–671)	1 (1–2)	13785 (10214–17356)	43 (35–51)
Romania	2032	565 (448–683)	1 (1–2)	13785 (10023–17547)	43 (34–52)
Romania	2033	571 (448–694)	1 (1–2)	13785 (9840–17730)	43 (34–52)
Romania	2034	577 (449–705)	1 (1–2)	13785 (9666–17904)	43 (34–52)
Romania	2035	583 (449–716)	1 (1–2)	13785 (9499–18071)	43 (33–53)
Romania	2036	588 (450–727)	1 (1–2)	13785 (9338–18232)	43 (33–53)
Russian Federation	2022	3088 (2905–3271)	1 (1–1)	84767 (79674–89860)	38 (35–41)
Russian Federation	2023	3081 (2775–3386)	1 (1–1)	81875 (73783–89967)	38 (34–43)
Russian Federation	2024	3078 (2673–3483)	1 (1–1)	80108 (70635–89580)	38 (33–44)
Russian Federation	2025	3077 (2588–3566)	1 (1–1)	79538 (69722–89354)	38 (32–44)
Russian Federation	2026	3077 (2516–3638)	1 (1–1)	79821 (69995–89646)	38 (32–45)
Russian Federation	2027	3077 (2451–3702)	1 (1–1)	80487 (70620–90354)	38 (31–46)
Russian Federation	2028	3077 (2393–3761)	1 (1–1)	81153 (71193–91112)	38 (31–46)
Russian Federation	2029	3077 (2339–3815)	1 (1–1)	81602 (71574–91631)	38 (30–47)
Russian Federation	2030	3077 (2289–3865)	1 (1–1)	81787 (71735–91839)	38 (30–47)
Russian Federation	2031	3077 (2241–3912)	1 (1–1)	81764 (71710–91817)	38 (29–48)
Russian Federation	2032	3077 (2197–3957)	1 (1–1)	81632 (71577–91687)	38 (29–48)
Russian Federation	2033	3077 (2154–3999)	1 (1–1)	81483 (71424–91541)	38 (28–49)
Russian Federation	2034	3077 (2114–4040)	1 (1–1)	81371 (71309–91434)	38 (28–49)
Russian Federation	2035	3077 (2075–4079)	1 (1–1)	81317 (71254–91381)	38 (27–49)
Russian Federation	2036	3077 (2037–4116)	1 (1–1)	81313 (71249–91377)	38 (27–50)
Rwanda	2022	25 (24–26)	0 (0–0)	631 (608–654)	10 (9–10)

Rwanda	2023	26 (24–28)	0 (0–0)	658 (599–716)	9 (9–10)
Rwanda	2024	27 (24–30)	0 (0–0)	688 (596–781)	9 (9–10)
Rwanda	2025	28 (24–32)	0 (0–0)	718 (595–840)	9 (8–11)
Rwanda	2026	29 (24–34)	0 (0–1)	745 (590–899)	9 (8–11)
Rwanda	2027	30 (24–37)	0 (0–1)	771 (578–965)	9 (8–11)
Rwanda	2028	31 (23–39)	0 (0–1)	799 (562–1037)	9 (7–12)
Rwanda	2029	32 (23–42)	0 (0–1)	828 (546–1111)	9 (7–12)
Rwanda	2030	34 (23–45)	0 (0–1)	857 (529–1185)	9 (7–12)
Rwanda	2031	35 (22–47)	0 (0–1)	884 (508–1260)	9 (7–12)
Rwanda	2032	36 (21–50)	0 (0–1)	912 (484–1340)	9 (7–12)
Rwanda	2033	37 (21–53)	0 (0–1)	940 (458–1422)	9 (6–13)
Rwanda	2034	38 (20–56)	0 (0–1)	968 (431–1506)	9 (6–13)
Rwanda	2035	39 (19–59)	1 (0–1)	996 (402–1591)	9 (6–13)
Rwanda	2036	40 (18–62)	1 (0–1)	1024 (370–1678)	9 (6–13)
Saint Kitts and Nevis	2022	0 (0–0)	0 (0–0)	8 (8–8)	10 (10–11)
Saint Kitts and Nevis	2023	0 (0–0)	0 (0–0)	8 (8–9)	10 (10–11)
Saint Kitts and Nevis	2024	0 (0–0)	0 (0–0)	8 (8–9)	10 (10–11)
Saint Kitts and Nevis	2025	0 (0–0)	0 (0–0)	8 (8–9)	11 (10–12)
Saint Kitts and Nevis	2026	0 (0–0)	0 (0–0)	9 (8–9)	11 (10–12)
Saint Kitts and Nevis	2027	0 (0–0)	0 (0–0)	9 (8–10)	11 (10–12)
Saint Kitts and Nevis	2028	0 (0–0)	0 (0–0)	9 (8–10)	11 (10–12)
Saint Kitts and Nevis	2029	0 (0–0)	0 (0–0)	9 (8–10)	11 (10–12)
Saint Kitts and Nevis	2030	0 (0–0)	0 (0–1)	9 (8–11)	11 (10–12)
Saint Kitts and Nevis	2031	0 (0–0)	0 (0–1)	10 (8–11)	11 (10–12)
Saint Kitts and Nevis	2032	0 (0–0)	0 (0–1)	10 (8–11)	11 (10–12)
Saint Kitts and Nevis	2033	0 (0–0)	0 (0–1)	10 (8–12)	11 (10–12)
Saint Kitts and Nevis	2034	0 (0–0)	0 (0–1)	10 (8–12)	11 (10–12)
Saint Kitts and Nevis	2035	0 (0–0)	0 (0–1)	10 (8–12)	11 (10–12)
Saint Kitts and Nevis	2036	0 (0–0)	0 (0–1)	10 (8–13)	11 (10–12)
Saint Lucia	2022	1 (1–1)	1 (0–1)	33 (32–34)	13 (12–14)
Saint Lucia	2023	1 (1–1)	1 (0–1)	33 (32–35)	13 (12–14)
Saint Lucia	2024	1 (1–1)	1 (0–1)	34 (32–36)	13 (11–15)
Saint Lucia	2025	1 (1–1)	1 (0–1)	35 (32–37)	13 (11–15)

Saint Lucia	2026	1 (1–1)	1 (0–1)	35 (33–38)	13 (11–15)
Saint Lucia	2027	1 (1–2)	1 (0–1)	36 (33–39)	13 (10–16)
Saint Lucia	2028	1 (1–2)	1 (0–1)	37 (33–40)	13 (10–16)
Saint Lucia	2029	1 (1–2)	1 (0–1)	37 (34–41)	13 (10–16)
Saint Lucia	2030	1 (1–2)	1 (0–1)	38 (34–42)	13 (9–16)
Saint Lucia	2031	1 (1–2)	1 (0–1)	39 (35–43)	13 (9–17)
Saint Lucia	2032	2 (1–2)	1 (0–1)	39 (35–44)	13 (9–17)
Saint Lucia	2033	2 (1–2)	1 (0–1)	40 (35–45)	13 (9–17)
Saint Lucia	2034	2 (1–2)	1 (0–1)	41 (36–45)	13 (9–17)
Saint Lucia	2035	2 (1–2)	1 (0–1)	41 (36–46)	13 (8–17)
Saint Lucia	2036	2 (1–2)	1 (0–1)	42 (37–47)	13 (8–18)
Saint Vincent and the Grenadines	2022	1 (1–1)	1 (0–1)	20 (20–21)	13 (13–14)
Saint Vincent and the Grenadines	2023	1 (1–1)	1 (0–1)	21 (20–22)	13 (13–14)
Saint Vincent and the Grenadines	2024	1 (1–1)	1 (0–1)	21 (19–23)	13 (13–14)
Saint Vincent and the Grenadines	2025	1 (1–1)	1 (0–1)	22 (19–24)	13 (12–15)
Saint Vincent and the Grenadines	2026	1 (1–1)	1 (0–1)	22 (19–25)	13 (12–15)
Saint Vincent and the Grenadines	2027	1 (1–1)	1 (0–1)	22 (19–26)	13 (12–15)
Saint Vincent and the Grenadines	2028	1 (1–1)	1 (0–1)	23 (18–28)	13 (12–15)
Saint Vincent and the Grenadines	2029	1 (1–1)	1 (0–1)	23 (18–29)	13 (12–15)
Saint Vincent and the Grenadines	2030	1 (1–1)	1 (0–1)	24 (17–30)	13 (12–15)
Saint Vincent and the Grenadines	2031	1 (1–1)	1 (0–1)	24 (17–32)	13 (12–15)
Saint Vincent and the Grenadines	2032	1 (1–1)	1 (0–1)	25 (16–33)	13 (12–15)
Saint Vincent and the Grenadines	2033	1 (1–1)	1 (0–1)	25 (16–35)	13 (12–15)
Saint Vincent and the Grenadines	2034	1 (1–1)	1 (0–1)	26 (15–36)	13 (11–15)
Saint Vincent and the Grenadines	2035	1 (1–1)	1 (0–1)	26 (14–38)	13 (11–16)
Saint Vincent and the Grenadines	2036	1 (1–1)	1 (0–1)	27 (14–40)	13 (11–16)
Samoa	2022	1 (1–1)	1 (1–1)	22 (22–22)	14 (14–14)
Samoa	2023	1 (1–1)	1 (1–1)	22 (22–23)	14 (13–14)
Samoa	2024	1 (1–1)	1 (0–1)	23 (22–23)	14 (13–14)
Samoa	2025	1 (1–1)	1 (0–1)	23 (22–24)	14 (13–15)
Samoa	2026	1 (1–1)	1 (0–1)	23 (22–25)	14 (13–15)
Samoa	2027	1 (1–1)	1 (0–1)	24 (22–25)	14 (13–15)
Samoa	2028	1 (1–1)	1 (0–1)	24 (23–26)	14 (13–15)

Samoa	2029	1 (1–1)	1 (0–1)	25 (23–27)	14 (12–15)
Samoa	2030	1 (1–1)	1 (0–1)	25 (23–27)	14 (12–16)
Samoa	2031	1 (1–1)	1 (0–1)	25 (23–28)	14 (12–16)
Samoa	2032	1 (1–1)	1 (0–1)	26 (23–29)	14 (12–16)
Samoa	2033	1 (1–1)	1 (0–1)	26 (23–29)	14 (12–16)
Samoa	2034	1 (1–1)	1 (0–1)	27 (23–30)	14 (12–16)
Samoa	2035	1 (1–1)	1 (0–1)	27 (23–30)	14 (11–17)
Samoa	2036	1 (1–1)	1 (0–1)	27 (23–31)	14 (11–17)
San Marino	2022	1 (0–1)	1 (1–1)	12 (10–13)	17 (15–20)
San Marino	2023	1 (0–1)	1 (1–1)	12 (9–14)	17 (13–21)
San Marino	2024	1 (0–1)	1 (0–1)	12 (8–15)	16 (11–21)
San Marino	2025	1 (0–1)	1 (0–1)	12 (8–15)	16 (10–21)
San Marino	2026	1 (0–1)	1 (0–1)	12 (7–16)	15 (9–21)
San Marino	2027	1 (0–1)	1 (0–1)	12 (7–16)	14 (7–21)
San Marino	2028	1 (0–1)	1 (0–1)	12 (7–17)	14 (6–21)
San Marino	2029	1 (0–1)	1 (0–1)	12 (6–17)	13 (5–21)
San Marino	2030	1 (0–1)	1 (0–1)	12 (6–17)	13 (4–21)
San Marino	2031	1 (0–1)	0 (0–1)	12 (6–18)	12 (3–21)
San Marino	2032	1 (0–1)	0 (0–1)	12 (5–18)	11 (2–21)
San Marino	2033	1 (0–1)	0 (-0–1)	12 (5–18)	11 (1–21)
San Marino	2034	1 (0–1)	0 (-0–1)	12 (5–18)	10 (-0–21)
San Marino	2035	1 (0–1)	0 (-0–1)	12 (5–19)	10 (-1–20)
San Marino	2036	1 (0–1)	0 (-0–1)	12 (4–19)	9 (-2–20)
Sao Tome and Principe	2022	0 (0–0)	0 (0–0)	2 (2–2)	1 (1–1)
Sao Tome and Principe	2023	0 (0–0)	0 (0–0)	2 (2–2)	1 (1–1)
Sao Tome and Principe	2024	0 (0–0)	0 (0–0)	2 (2–2)	1 (1–1)
Sao Tome and Principe	2025	0 (0–0)	0 (0–0)	2 (2–2)	1 (1–1)
Sao Tome and Principe	2026	0 (0–0)	0 (0–0)	2 (2–2)	1 (1–1)
Sao Tome and Principe	2027	0 (0–0)	0 (0–0)	2 (2–2)	1 (1–1)
Sao Tome and Principe	2028	0 (0–0)	0 (0–0)	2 (2–2)	1 (1–1)
Sao Tome and Principe	2029	0 (0–0)	0 (0–0)	2 (2–2)	1 (1–1)
Sao Tome and Principe	2030	0 (0–0)	0 (0–0)	2 (2–2)	1 (1–1)
Sao Tome and Principe	2031	0 (0–0)	0 (0–0)	2 (2–2)	1 (1–1)

Sao Tome and Principe	2032	0 (0–0)	0 (0–0)	2 (2–2)	1 (1–1)
Sao Tome and Principe	2033	0 (0–0)	0 (0–0)	2 (2–3)	1 (1–1)
Sao Tome and Principe	2034	0 (0–0)	0 (0–0)	2 (2–3)	1 (1–2)
Sao Tome and Principe	2035	0 (0–0)	0 (0–0)	2 (2–3)	1 (1–2)
Sao Tome and Principe	2036	0 (0–0)	0 (0–0)	2 (2–3)	1 (1–2)
Saudi Arabia	2022	60 (60–61)	0 (0–0)	2028 (2013–2042)	7 (7–7)
Saudi Arabia	2023	63 (62–64)	0 (0–0)	2115 (2083–2148)	7 (7–7)
Saudi Arabia	2024	66 (64–67)	0 (0–0)	2203 (2149–2257)	7 (7–7)
Saudi Arabia	2025	68 (66–71)	0 (0–0)	2291 (2212–2370)	7 (6–7)
Saudi Arabia	2026	71 (68–74)	0 (0–0)	2378 (2271–2486)	7 (6–7)
Saudi Arabia	2027	74 (70–78)	0 (0–0)	2466 (2328–2604)	7 (6–8)
Saudi Arabia	2028	76 (71–81)	0 (0–0)	2554 (2383–2725)	7 (6–8)
Saudi Arabia	2029	79 (73–85)	0 (0–0)	2642 (2435–2848)	7 (5–8)
Saudi Arabia	2030	82 (74–89)	0 (0–0)	2729 (2485–2973)	6 (5–8)
Saudi Arabia	2031	84 (76–93)	0 (0–0)	2817 (2534–3101)	6 (5–8)
Saudi Arabia	2032	87 (77–96)	0 (0–0)	2905 (2580–3230)	6 (4–8)
Saudi Arabia	2033	89 (79–100)	0 (0–0)	2992 (2624–3361)	6 (4–9)
Saudi Arabia	2034	92 (80–104)	0 (0–0)	3080 (2667–3494)	6 (4–9)
Saudi Arabia	2035	95 (81–108)	0 (0–0)	3168 (2708–3628)	6 (3–9)
Saudi Arabia	2036	97 (82–112)	0 (0–0)	3256 (2747–3764)	6 (3–9)
Senegal	2022	11 (11–11)	0 (0–0)	327 (315–339)	4 (3–4)
Senegal	2023	11 (11–12)	0 (0–0)	334 (317–351)	4 (3–4)
Senegal	2024	12 (11–12)	0 (0–0)	340 (319–361)	4 (3–4)
Senegal	2025	12 (11–13)	0 (0–0)	346 (322–370)	4 (3–4)
Senegal	2026	12 (11–13)	0 (0–0)	353 (326–379)	4 (3–4)
Senegal	2027	12 (12–13)	0 (0–0)	359 (330–388)	4 (3–4)
Senegal	2028	13 (12–14)	0 (0–0)	365 (334–397)	4 (3–4)
Senegal	2029	13 (12–14)	0 (0–0)	372 (338–406)	4 (3–4)
Senegal	2030	13 (12–15)	0 (0–0)	378 (342–414)	4 (3–4)
Senegal	2031	14 (12–15)	0 (0–0)	384 (347–422)	4 (3–4)
Senegal	2032	14 (12–16)	0 (0–0)	391 (351–430)	4 (3–4)
Senegal	2033	14 (12–16)	0 (0–0)	397 (356–439)	4 (3–4)
Senegal	2034	15 (13–17)	0 (0–0)	403 (360–447)	4 (3–4)

Senegal	2035	15 (13–17)	0 (0–0)	410 (365–455)	4 (3–4)
Senegal	2036	15 (13–18)	0 (0–0)	416 (370–462)	4 (3–4)
Serbia	2022	246 (235–257)	2 (1–2)	6252 (5945–6559)	42 (39–44)
Serbia	2023	246 (230–263)	2 (1–2)	6239 (5772–6705)	42 (39–44)
Serbia	2024	247 (225–268)	2 (1–2)	6226 (5614–6837)	42 (38–45)
Serbia	2025	247 (220–274)	2 (1–2)	6213 (5460–6966)	42 (37–46)
Serbia	2026	247 (216–279)	2 (1–2)	6200 (5305–7095)	42 (37–46)
Serbia	2027	248 (211–284)	2 (1–2)	6187 (5148–7225)	42 (37–47)
Serbia	2028	248 (206–290)	2 (1–2)	6174 (4988–7359)	42 (36–47)
Serbia	2029	248 (201–296)	2 (1–2)	6161 (4825–7496)	42 (36–47)
Serbia	2030	249 (196–302)	2 (1–2)	6148 (4658–7637)	42 (35–48)
Serbia	2031	249 (191–307)	2 (1–2)	6134 (4487–7782)	42 (35–48)
Serbia	2032	250 (185–314)	2 (1–2)	6121 (4312–7931)	42 (35–48)
Serbia	2033	250 (180–320)	2 (1–2)	6108 (4133–8083)	42 (34–49)
Serbia	2034	250 (174–326)	2 (1–2)	6095 (3951–8240)	42 (34–49)
Serbia	2035	251 (169–333)	2 (1–2)	6082 (3764–8400)	42 (34–49)
Serbia	2036	251 (163–339)	2 (1–2)	6069 (3574–8565)	42 (34–50)
Seychelles	2022	1 (1–1)	1 (1–1)	27 (26–29)	22 (21–24)
Seychelles	2023	1 (1–1)	1 (1–1)	27 (24–29)	23 (21–25)
Seychelles	2024	1 (1–1)	1 (1–1)	27 (23–30)	24 (21–26)
Seychelles	2025	1 (1–1)	1 (1–1)	26 (22–31)	24 (22–26)
Seychelles	2026	1 (1–1)	1 (1–1)	26 (20–32)	25 (22–27)
Seychelles	2027	1 (1–1)	1 (1–1)	26 (19–33)	25 (22–27)
Seychelles	2028	1 (1–1)	1 (1–1)	26 (18–34)	25 (22–27)
Seychelles	2029	1 (1–1)	1 (1–1)	26 (17–35)	24 (22–27)
Seychelles	2030	1 (1–1)	1 (1–1)	26 (17–35)	24 (22–27)
Seychelles	2031	1 (1–1)	1 (1–1)	26 (16–36)	24 (22–26)
Seychelles	2032	1 (1–1)	1 (1–1)	26 (15–37)	24 (22–26)
Seychelles	2033	1 (1–1)	1 (1–1)	26 (14–37)	24 (22–26)
Seychelles	2034	1 (1–1)	1 (1–1)	26 (14–38)	24 (22–26)
Seychelles	2035	1 (1–1)	1 (1–1)	26 (13–39)	24 (22–26)
Seychelles	2036	1 (1–1)	1 (1–1)	26 (13–39)	24 (22–27)
Sierra Leone	2022	6 (6–6)	0 (0–0)	168 (166–171)	4 (4–4)

Sierra Leone	2023	6 (6–6)	0 (0–0)	174 (170–178)	4 (4–4)
Sierra Leone	2024	6 (6–6)	0 (0–0)	180 (173–186)	4 (4–4)
Sierra Leone	2025	6 (6–6)	0 (0–0)	185 (176–194)	4 (4–4)
Sierra Leone	2026	6 (6–7)	0 (0–0)	191 (179–203)	4 (4–4)
Sierra Leone	2027	7 (6–7)	0 (0–0)	196 (182–211)	4 (4–4)
Sierra Leone	2028	7 (6–7)	0 (0–0)	202 (184–220)	4 (4–4)
Sierra Leone	2029	7 (6–8)	0 (0–0)	208 (186–229)	4 (3–4)
Sierra Leone	2030	7 (6–8)	0 (0–0)	213 (188–238)	4 (3–4)
Sierra Leone	2031	7 (6–8)	0 (0–0)	219 (190–248)	4 (3–4)
Sierra Leone	2032	7 (6–8)	0 (0–0)	224 (191–258)	4 (3–4)
Sierra Leone	2033	8 (6–9)	0 (0–0)	230 (193–267)	4 (3–4)
Sierra Leone	2034	8 (6–9)	0 (0–0)	236 (194–277)	4 (3–4)
Sierra Leone	2035	8 (7–9)	0 (0–0)	241 (195–288)	4 (3–4)
Sierra Leone	2036	8 (7–10)	0 (0–0)	247 (196–298)	4 (3–4)
Singapore	2022	35 (32–38)	0 (0–0)	861 (787–936)	10 (8–11)
Singapore	2023	36 (33–39)	0 (0–0)	876 (788–964)	10 (8–11)
Singapore	2024	37 (33–40)	0 (0–0)	891 (792–991)	9 (8–11)
Singapore	2025	37 (33–41)	0 (0–0)	906 (796–1016)	9 (8–11)
Singapore	2026	38 (33–42)	0 (0–0)	921 (801–1041)	9 (7–11)
Singapore	2027	39 (34–43)	0 (0–0)	936 (807–1065)	9 (7–10)
Singapore	2028	39 (34–44)	0 (0–0)	951 (814–1088)	9 (7–10)
Singapore	2029	40 (35–45)	0 (0–0)	966 (821–1110)	8 (7–10)
Singapore	2030	41 (35–46)	0 (0–0)	980 (828–1133)	8 (6–10)
Singapore	2031	41 (35–47)	0 (0–0)	995 (836–1155)	8 (6–10)
Singapore	2032	42 (36–48)	0 (0–0)	1010 (844–1176)	8 (6–10)
Singapore	2033	43 (36–49)	0 (0–0)	1025 (852–1198)	8 (6–9)
Singapore	2034	43 (37–50)	0 (0–0)	1040 (861–1219)	7 (6–9)
Singapore	2035	44 (37–51)	0 (0–0)	1055 (870–1240)	7 (5–9)
Singapore	2036	45 (37–52)	0 (0–0)	1070 (879–1261)	7 (5–9)
Slovakia	2022	132 (128–136)	1 (1–1)	3474 (3351–3596)	38 (36–39)
Slovakia	2023	133 (128–139)	1 (1–1)	3499 (3325–3672)	37 (35–40)
Slovakia	2024	134 (127–141)	1 (1–1)	3523 (3311–3735)	37 (34–40)
Slovakia	2025	136 (128–144)	1 (1–1)	3548 (3303–3793)	37 (34–40)

Slovakia	2026	137 (128–146)	1 (1–2)	3573 (3299–3846)	37 (33–40)
Slovakia	2027	138 (128–148)	1 (1–2)	3597 (3297–3897)	36 (32–40)
Slovakia	2028	139 (129–150)	1 (1–2)	3622 (3298–3946)	36 (32–41)
Slovakia	2029	140 (129–152)	1 (1–2)	3647 (3300–3993)	36 (31–41)
Slovakia	2030	142 (129–154)	1 (1–2)	3671 (3304–4038)	36 (31–41)
Slovakia	2031	143 (130–156)	1 (1–2)	3696 (3309–4083)	35 (30–41)
Slovakia	2032	144 (131–157)	1 (1–2)	3721 (3314–4127)	35 (30–41)
Slovakia	2033	145 (131–159)	1 (1–2)	3745 (3321–4169)	35 (29–41)
Slovakia	2034	146 (132–161)	1 (1–2)	3770 (3328–4211)	35 (29–41)
Slovakia	2035	148 (132–163)	1 (1–2)	3794 (3336–4253)	34 (28–41)
Slovakia	2036	149 (133–164)	1 (1–2)	3819 (3345–4293)	34 (28–41)
Slovenia	2022	45 (42–47)	1 (1–1)	1096 (1026–1165)	28 (26–30)
Slovenia	2023	45 (42–49)	1 (1–1)	1105 (1007–1203)	28 (26–31)
Slovenia	2024	46 (42–50)	1 (1–1)	1115 (995–1235)	29 (25–32)
Slovenia	2025	46 (42–51)	1 (1–1)	1124 (986–1263)	29 (25–33)
Slovenia	2026	47 (42–52)	1 (1–1)	1134 (979–1289)	29 (25–33)
Slovenia	2027	47 (42–53)	1 (1–1)	1144 (974–1313)	30 (25–34)
Slovenia	2028	48 (42–54)	1 (1–1)	1153 (970–1337)	30 (25–34)
Slovenia	2029	49 (42–55)	1 (1–1)	1163 (967–1359)	30 (25–34)
Slovenia	2030	49 (42–56)	1 (1–1)	1173 (965–1380)	30 (26–35)
Slovenia	2031	50 (42–57)	1 (1–1)	1182 (963–1401)	30 (26–35)
Slovenia	2032	50 (42–58)	1 (1–1)	1192 (962–1422)	30 (26–35)
Slovenia	2033	51 (42–59)	1 (1–1)	1202 (962–1441)	31 (26–35)
Slovenia	2034	51 (42–60)	1 (1–1)	1211 (962–1461)	31 (26–35)
Slovenia	2035	52 (43–61)	1 (1–1)	1221 (962–1480)	31 (26–36)
Slovenia	2036	52 (43–62)	1 (1–1)	1231 (963–1499)	31 (26–36)
Solomon Islands	2022	2 (2–2)	1 (0–1)	59 (58–60)	14 (14–14)
Solomon Islands	2023	2 (2–2)	1 (0–1)	60 (58–62)	14 (14–15)
Solomon Islands	2024	2 (2–2)	1 (0–1)	62 (59–65)	14 (14–15)
Solomon Islands	2025	2 (2–2)	1 (0–1)	64 (60–68)	15 (14–15)
Solomon Islands	2026	2 (2–2)	1 (0–1)	66 (60–71)	15 (14–15)
Solomon Islands	2027	2 (2–2)	1 (0–1)	67 (60–75)	15 (14–16)
Solomon Islands	2028	2 (2–2)	1 (0–1)	69 (60–78)	15 (14–16)

Solomon Islands	2029	2 (2–3)	1 (0–1)	71 (60–82)	15 (14–16)
Solomon Islands	2030	2 (2–3)	1 (0–1)	73 (60–85)	15 (14–16)
Solomon Islands	2031	2 (2–3)	1 (1–1)	74 (60–89)	15 (14–16)
Solomon Islands	2032	2 (2–3)	1 (1–1)	76 (60–93)	15 (14–17)
Solomon Islands	2033	3 (2–3)	1 (1–1)	78 (59–97)	16 (14–17)
Solomon Islands	2034	3 (2–3)	1 (1–1)	80 (59–101)	16 (14–17)
Solomon Islands	2035	3 (2–3)	1 (1–1)	81 (58–105)	16 (14–17)
Solomon Islands	2036	3 (2–3)	1 (1–1)	83 (58–109)	16 (15–17)
Somalia	2022	6 (6–6)	0 (0–0)	189 (187–191)	3 (3–3)
Somalia	2023	6 (6–6)	0 (0–0)	191 (187–195)	3 (2–3)
Somalia	2024	6 (6–6)	0 (0–0)	194 (188–200)	3 (2–3)
Somalia	2025	6 (6–7)	0 (0–0)	196 (189–203)	2 (2–3)
Somalia	2026	6 (6–7)	0 (0–0)	199 (191–207)	2 (2–3)
Somalia	2027	7 (6–7)	0 (0–0)	201 (192–210)	2 (2–3)
Somalia	2028	7 (6–7)	0 (0–0)	204 (194–214)	2 (2–3)
Somalia	2029	7 (6–7)	0 (0–0)	206 (195–217)	2 (2–3)
Somalia	2030	7 (6–7)	0 (0–0)	209 (197–220)	2 (2–3)
Somalia	2031	7 (6–7)	0 (0–0)	211 (199–223)	2 (2–3)
Somalia	2032	7 (6–7)	0 (0–0)	213 (200–226)	2 (2–3)
Somalia	2033	7 (7–7)	0 (0–0)	216 (202–230)	2 (2–3)
Somalia	2034	7 (7–8)	0 (0–0)	218 (204–233)	2 (1–3)
Somalia	2035	7 (7–8)	0 (0–0)	221 (206–236)	2 (1–3)
Somalia	2036	7 (7–8)	0 (0–0)	223 (208–239)	2 (1–3)
South Africa	2022	302 (293–312)	1 (1–1)	8861 (8574–9149)	17 (16–18)
South Africa	2023	307 (293–321)	1 (1–1)	9004 (8598–9410)	17 (16–19)
South Africa	2024	312 (295–329)	1 (1–1)	9147 (8649–9644)	17 (15–19)
South Africa	2025	317 (298–336)	1 (1–1)	9289 (8715–9864)	17 (15–19)
South Africa	2026	322 (300–344)	1 (1–1)	9432 (8790–10074)	17 (15–19)
South Africa	2027	327 (303–351)	1 (1–1)	9575 (8871–10278)	17 (15–20)
South Africa	2028	332 (306–357)	1 (1–1)	9717 (8957–10477)	17 (15–20)
South Africa	2029	337 (309–364)	1 (1–1)	9860 (9047–10673)	17 (14–20)
South Africa	2030	342 (312–371)	1 (1–1)	10003 (9141–10865)	17 (14–20)
South Africa	2031	346 (316–377)	1 (1–1)	10145 (9237–11054)	17 (14–20)

South Africa	2032	351 (319–384)	1 (1–1)	10288 (9335–11241)	17 (14–20)
South Africa	2033	356 (323–390)	1 (0–1)	10431 (9435–11426)	17 (14–21)
South Africa	2034	361 (326–396)	1 (0–1)	10573 (9537–11609)	17 (14–21)
South Africa	2035	366 (330–402)	1 (0–1)	10716 (9641–11791)	17 (13–21)
South Africa	2036	371 (333–409)	1 (0–1)	10859 (9746–11971)	17 (13–21)
South Sudan	2022	7 (7–7)	0 (0–0)	209 (206–212)	5 (5–5)
South Sudan	2023	7 (7–7)	0 (0–0)	214 (207–220)	5 (5–5)
South Sudan	2024	7 (7–8)	0 (0–0)	219 (208–229)	5 (4–5)
South Sudan	2025	7 (7–8)	0 (0–0)	223 (209–237)	5 (4–5)
South Sudan	2026	8 (7–8)	0 (0–0)	228 (209–247)	5 (4–5)
South Sudan	2027	8 (7–9)	0 (0–0)	233 (209–256)	5 (4–5)
South Sudan	2028	8 (7–9)	0 (0–0)	237 (209–266)	5 (4–5)
South Sudan	2029	8 (6–9)	0 (0–0)	242 (208–277)	5 (4–6)
South Sudan	2030	8 (6–10)	0 (0–0)	247 (206–287)	5 (4–6)
South Sudan	2031	8 (6–10)	0 (0–0)	252 (205–298)	5 (3–6)
South Sudan	2032	8 (6–11)	0 (0–0)	256 (203–310)	5 (3–6)
South Sudan	2033	8 (6–11)	0 (0–0)	261 (201–321)	5 (3–6)
South Sudan	2034	8 (5–11)	0 (0–0)	266 (199–333)	5 (3–6)
South Sudan	2035	9 (5–12)	0 (0–0)	271 (196–345)	5 (3–7)
South Sudan	2036	9 (5–12)	0 (0–0)	275 (193–357)	5 (3–7)
Spain	2022	887 (857–917)	1 (1–1)	21343 (20433–22252)	25 (23–26)
Spain	2023	883 (834–932)	1 (1–1)	21406 (20029–22783)	24 (22–27)
Spain	2024	874 (802–945)	1 (1–1)	20995 (18957–23033)	23 (19–26)
Spain	2025	869 (776–963)	1 (1–1)	20970 (18362–23578)	22 (17–27)
Spain	2026	864 (749–980)	1 (1–1)	20763 (17550–23976)	21 (14–27)
Spain	2027	861 (725–998)	1 (1–1)	20722 (16952–24492)	20 (12–28)
Spain	2028	859 (702–1015)	1 (1–1)	20614 (16294–24934)	19 (9–28)
Spain	2029	857 (681–1032)	1 (1–1)	20579 (15742–25416)	18 (6–29)
Spain	2030	855 (660–1049)	1 (1–1)	20521 (15185–25857)	16 (3–30)
Spain	2031	854 (641–1066)	1 (1–1)	20495 (14687–26304)	15 (-0–31)
Spain	2032	853 (623–1082)	1 (1–1)	20463 (14201–26725)	14 (-4–32)
Spain	2033	852 (606–1097)	1 (1–1)	20446 (13751–27141)	13 (-7–34)
Spain	2034	851 (590–1112)	1 (1–1)	20428 (13318–27538)	12 (-11–35)

Spain	2035	851 (574–1127)	1 (1–1)	20417 (12910–27924)	11 (-14–36)
Spain	2036	850 (560–1141)	1 (1–1)	20406 (12517–28295)	10 (-18–38)
Sri Lanka	2022	40 (38–42)	0 (0–0)	1022 (969–1075)	4 (3–4)
Sri Lanka	2023	40 (38–43)	0 (0–0)	1036 (961–1111)	3 (3–4)
Sri Lanka	2024	41 (37–44)	0 (0–0)	1049 (957–1141)	3 (3–4)
Sri Lanka	2025	42 (38–46)	0 (0–0)	1062 (956–1168)	3 (3–4)
Sri Lanka	2026	42 (38–47)	0 (0–0)	1075 (957–1194)	3 (3–4)
Sri Lanka	2027	43 (38–48)	0 (0–0)	1089 (959–1218)	3 (3–4)
Sri Lanka	2028	43 (38–49)	0 (0–0)	1102 (962–1242)	3 (2–4)
Sri Lanka	2029	44 (38–49)	0 (0–0)	1115 (965–1265)	3 (2–4)
Sri Lanka	2030	44 (38–50)	0 (0–0)	1128 (969–1287)	3 (2–4)
Sri Lanka	2031	45 (39–51)	0 (0–0)	1141 (974–1309)	3 (2–4)
Sri Lanka	2032	45 (39–52)	0 (0–0)	1155 (979–1330)	3 (2–4)
Sri Lanka	2033	46 (39–53)	0 (0–0)	1168 (984–1351)	3 (2–4)
Sri Lanka	2034	47 (39–54)	0 (0–0)	1181 (990–1372)	3 (2–4)
Sri Lanka	2035	47 (40–55)	0 (0–0)	1194 (996–1392)	3 (2–4)
Sri Lanka	2036	48 (40–55)	0 (0–0)	1208 (1002–1413)	3 (2–4)
Sudan	2022	46 (46–46)	0 (0–0)	1301 (1294–1309)	6 (6–6)
Sudan	2023	48 (47–48)	0 (0–0)	1354 (1338–1370)	6 (6–6)
Sudan	2024	50 (49–51)	0 (0–0)	1407 (1380–1434)	6 (6–6)
Sudan	2025	51 (50–53)	0 (0–0)	1460 (1421–1500)	6 (6–6)
Sudan	2026	53 (51–55)	0 (0–0)	1513 (1459–1567)	6 (6–7)
Sudan	2027	55 (52–57)	0 (0–0)	1566 (1497–1635)	6 (6–7)
Sudan	2028	56 (53–59)	0 (0–0)	1619 (1533–1705)	6 (6–7)
Sudan	2029	58 (54–62)	0 (0–0)	1672 (1568–1775)	7 (6–7)
Sudan	2030	60 (55–64)	0 (0–0)	1725 (1603–1847)	7 (6–7)
Sudan	2031	61 (56–67)	0 (0–0)	1778 (1636–1920)	7 (6–7)
Sudan	2032	63 (57–69)	0 (0–0)	1831 (1668–1993)	7 (6–8)
Sudan	2033	65 (58–72)	0 (0–0)	1884 (1699–2068)	7 (6–8)
Sudan	2034	67 (59–74)	0 (0–0)	1937 (1729–2144)	7 (6–8)
Sudan	2035	68 (60–77)	0 (0–0)	1989 (1759–2220)	7 (6–8)
Sudan	2036	70 (61–79)	0 (0–0)	2042 (1787–2297)	7 (6–9)
Suriname	2022	5 (4–5)	1 (1–1)	131 (125–136)	19 (18–20)

Suriname	2023	5 (4–5)	1 (1–1)	133 (121–145)	20 (17–22)
Suriname	2024	5 (4–5)	1 (1–1)	135 (119–152)	21 (18–24)
Suriname	2025	5 (4–6)	1 (1–1)	138 (118–158)	21 (18–24)
Suriname	2026	5 (4–6)	1 (1–1)	140 (117–163)	21 (18–25)
Suriname	2027	5 (4–6)	1 (1–1)	143 (117–168)	21 (18–25)
Suriname	2028	5 (4–6)	1 (1–1)	145 (117–173)	22 (18–25)
Suriname	2029	5 (4–6)	1 (1–1)	147 (118–177)	22 (18–25)
Suriname	2030	5 (4–6)	1 (1–1)	150 (118–182)	22 (18–25)
Suriname	2031	5 (4–7)	1 (1–1)	152 (119–186)	22 (18–25)
Suriname	2032	6 (4–7)	1 (1–1)	155 (119–190)	22 (18–25)
Suriname	2033	6 (4–7)	1 (1–1)	157 (120–194)	22 (18–25)
Suriname	2034	6 (5–7)	1 (1–1)	160 (121–198)	22 (18–25)
Suriname	2035	6 (5–7)	1 (1–1)	162 (122–202)	22 (18–25)
Suriname	2036	6 (5–7)	1 (1–1)	164 (123–206)	22 (18–25)
Sweden	2022	275 (258–291)	1 (1–1)	5487 (5164–5810)	28 (26–30)
Sweden	2023	268 (240–296)	1 (1–1)	5405 (4884–5926)	27 (24–31)
Sweden	2024	262 (226–299)	1 (1–1)	5358 (4711–6004)	27 (23–31)
Sweden	2025	258 (216–300)	1 (1–2)	5332 (4613–6051)	27 (22–32)
Sweden	2026	255 (209–300)	1 (1–2)	5319 (4560–6077)	26 (21–32)
Sweden	2027	252 (205–300)	1 (1–2)	5313 (4534–6091)	26 (20–32)
Sweden	2028	251 (202–300)	1 (1–2)	5310 (4522–6098)	26 (19–32)
Sweden	2029	250 (200–300)	1 (1–2)	5310 (4518–6102)	25 (18–32)
Sweden	2030	249 (200–299)	1 (1–2)	5310 (4516–6105)	25 (17–32)
Sweden	2031	249 (199–299)	1 (1–2)	5311 (4516–6106)	24 (16–33)
Sweden	2032	249 (199–299)	1 (1–2)	5312 (4517–6107)	24 (15–33)
Sweden	2033	249 (199–299)	1 (1–2)	5313 (4517–6108)	24 (15–33)
Sweden	2034	249 (199–299)	1 (1–2)	5313 (4518–6108)	23 (14–33)
Sweden	2035	249 (199–299)	1 (1–2)	5314 (4518–6109)	23 (13–33)
Sweden	2036	249 (199–299)	1 (1–2)	5314 (4519–6109)	22 (12–32)
Switzerland	2022	227 (216–238)	1 (1–1)	4728 (4468–4988)	29 (26–31)
Switzerland	2023	231 (211–251)	1 (1–1)	4795 (4331–5258)	29 (25–33)
Switzerland	2024	234 (208–260)	1 (1–1)	4861 (4259–5463)	29 (25–34)
Switzerland	2025	238 (207–269)	1 (1–1)	4928 (4214–5641)	30 (25–34)

Switzerland	2026	242 (207–276)	1 (1–2)	4994 (4184–5805)	30 (25–34)
Switzerland	2027	245 (207–284)	1 (1–2)	5061 (4165–5957)	30 (25–35)
Switzerland	2028	249 (207–291)	1 (1–2)	5128 (4153–6103)	30 (25–35)
Switzerland	2029	252 (207–297)	1 (1–2)	5194 (4147–6242)	30 (25–35)
Switzerland	2030	256 (208–304)	1 (1–2)	5261 (4145–6377)	30 (25–35)
Switzerland	2031	260 (209–310)	1 (1–2)	5328 (4148–6507)	30 (25–35)
Switzerland	2032	263 (210–317)	1 (1–2)	5394 (4153–6635)	30 (25–35)
Switzerland	2033	267 (211–323)	1 (1–2)	5461 (4162–6759)	30 (25–35)
Switzerland	2034	271 (212–329)	1 (1–2)	5527 (4173–6881)	30 (25–35)
Switzerland	2035	274 (214–335)	1 (1–2)	5594 (4187–7001)	30 (25–35)
Switzerland	2036	278 (215–340)	1 (1–2)	5661 (4202–7119)	30 (25–35)
Syrian Arab Republic	2022	72 (71–74)	1 (1–1)	2012 (1962–2062)	13 (13–14)
Syrian Arab Republic	2023	74 (71–77)	1 (0–1)	2056 (1964–2148)	13 (13–14)
Syrian Arab Republic	2024	76 (71–80)	1 (0–1)	2099 (1970–2229)	14 (13–15)
Syrian Arab Republic	2025	77 (72–83)	1 (0–1)	2143 (1980–2305)	14 (12–15)
Syrian Arab Republic	2026	79 (72–85)	1 (0–1)	2186 (1994–2378)	14 (12–15)
Syrian Arab Republic	2027	80 (73–88)	1 (0–1)	2229 (2011–2448)	14 (12–15)
Syrian Arab Republic	2028	82 (73–90)	1 (0–1)	2273 (2030–2515)	14 (12–15)
Syrian Arab Republic	2029	83 (74–93)	1 (0–1)	2316 (2052–2580)	14 (12–16)
Syrian Arab Republic	2030	85 (75–95)	1 (0–1)	2359 (2075–2644)	14 (12–16)
Syrian Arab Republic	2031	87 (76–97)	1 (0–1)	2403 (2099–2707)	14 (12–16)
Syrian Arab Republic	2032	88 (77–99)	1 (0–1)	2446 (2124–2768)	14 (12–16)
Syrian Arab Republic	2033	90 (78–102)	1 (0–1)	2489 (2150–2829)	14 (12–16)
Syrian Arab Republic	2034	91 (79–104)	1 (0–1)	2533 (2177–2888)	14 (12–16)
Syrian Arab Republic	2035	93 (80–106)	1 (0–1)	2576 (2205–2947)	14 (12–17)
Syrian Arab Republic	2036	95 (81–108)	1 (0–1)	2619 (2233–3005)	15 (12–17)
Taiwan (Province of China)	2022	426 (409–443)	1 (1–1)	10761 (10294–11228)	26 (24–28)
Taiwan (Province of China)	2023	435 (411–459)	1 (1–1)	10980 (10320–11641)	26 (23–28)
Taiwan (Province of China)	2024	444 (415–474)	1 (1–1)	11200 (10391–12009)	25 (23–28)
Taiwan (Province of China)	2025	454 (420–488)	1 (1–1)	11420 (10486–12353)	25 (22–28)
Taiwan (Province of China)	2026	463 (425–501)	1 (1–1)	11639 (10595–12683)	25 (22–28)
Taiwan (Province of China)	2027	472 (430–514)	1 (1–1)	11859 (10715–13003)	25 (22–28)
Taiwan (Province of China)	2028	481 (436–526)	1 (1–1)	12078 (10843–13314)	25 (22–28)

Taiwan (Province of China)	2029	491 (442–539)	1 (1–1)	12298 (10977–13619)	25 (22–28)
Taiwan (Province of China)	2030	500 (449–551)	1 (1–1)	12517 (11117–13918)	25 (22–28)
Taiwan (Province of China)	2031	509 (455–563)	1 (1–1)	12737 (11260–14214)	25 (22–28)
Taiwan (Province of China)	2032	518 (462–575)	1 (1–1)	12957 (11408–14505)	25 (22–28)
Taiwan (Province of China)	2033	528 (469–587)	1 (1–1)	13176 (11559–14794)	25 (22–28)
Taiwan (Province of China)	2034	537 (476–598)	1 (1–1)	13396 (11712–15080)	25 (22–28)
Taiwan (Province of China)	2035	546 (482–610)	1 (1–1)	13615 (11868–15363)	25 (22–28)
Taiwan (Province of China)	2036	555 (489–621)	1 (1–1)	13835 (12026–15644)	25 (22–28)
Tajikistan	2022	7 (7–7)	0 (0–0)	215 (208–223)	3 (3–3)
Tajikistan	2023	7 (7–8)	0 (0–0)	222 (208–237)	3 (3–3)
Tajikistan	2024	8 (7–8)	0 (0–0)	229 (206–252)	3 (2–3)
Tajikistan	2025	8 (7–9)	0 (0–0)	236 (203–269)	3 (2–4)
Tajikistan	2026	8 (6–10)	0 (0–0)	243 (199–287)	3 (2–4)
Tajikistan	2027	8 (6–10)	0 (0–0)	249 (194–305)	3 (2–4)
Tajikistan	2028	8 (6–11)	0 (0–0)	256 (188–325)	3 (2–4)
Tajikistan	2029	9 (6–11)	0 (0–0)	263 (181–345)	2 (1–4)
Tajikistan	2030	9 (6–12)	0 (0–0)	270 (173–367)	2 (1–4)
Tajikistan	2031	9 (5–13)	0 (0–0)	277 (165–389)	2 (1–4)
Tajikistan	2032	9 (5–14)	0 (0–0)	283 (156–411)	2 (1–4)
Tajikistan	2033	9 (5–14)	0 (0–0)	290 (146–435)	2 (1–4)
Tajikistan	2034	10 (4–15)	0 (0–0)	297 (135–459)	2 (1–4)
Tajikistan	2035	10 (4–16)	0 (0–0)	304 (124–483)	2 (0–4)
Tajikistan	2036	10 (3–17)	0 (0–0)	311 (113–509)	2 (0–3)
Thailand	2022	793 (776–810)	1 (1–1)	20353 (19902–20805)	18 (18–19)
Thailand	2023	823 (794–852)	1 (1–1)	20760 (20122–21399)	18 (17–19)
Thailand	2024	853 (811–894)	1 (1–1)	21168 (20386–21949)	18 (17–20)
Thailand	2025	882 (828–937)	1 (1–1)	21575 (20672–22477)	18 (17–20)
Thailand	2026	912 (844–981)	1 (1–1)	21982 (20973–22991)	18 (17–20)
Thailand	2027	942 (858–1026)	1 (1–1)	22389 (21283–23494)	18 (17–20)
Thailand	2028	972 (872–1071)	1 (1–1)	22796 (21602–23990)	18 (16–20)
Thailand	2029	1002 (885–1118)	1 (1–1)	23203 (21927–24480)	18 (16–20)
Thailand	2030	1031 (898–1165)	1 (1–1)	23610 (22256–24964)	18 (16–20)
Thailand	2031	1061 (909–1213)	1 (1–1)	24017 (22590–25445)	18 (16–21)

Thailand	2032	1091 (920–1262)	1 (1–1)	24425 (22928–25921)	18 (16–21)
Thailand	2033	1121 (930–1312)	1 (1–1)	24832 (23268–26395)	18 (16–21)
Thailand	2034	1151 (939–1362)	1 (1–1)	25239 (23612–26866)	18 (16–21)
Thailand	2035	1181 (948–1413)	1 (1–1)	25646 (23957–27335)	18 (16–21)
Thailand	2036	1210 (956–1465)	1 (1–1)	26053 (24305–27801)	18 (16–21)
Timor-Leste	2022	3 (3–3)	0 (0–0)	71 (70–72)	8 (8–8)
Timor-Leste	2023	3 (3–3)	0 (0–0)	73 (71–75)	8 (8–8)
Timor-Leste	2024	3 (3–3)	0 (0–0)	74 (72–77)	8 (7–8)
Timor-Leste	2025	3 (3–3)	0 (0–0)	76 (72–80)	8 (7–9)
Timor-Leste	2026	3 (2–3)	0 (0–0)	78 (72–83)	8 (7–9)
Timor-Leste	2027	3 (2–3)	0 (0–0)	79 (73–86)	8 (7–9)
Timor-Leste	2028	3 (2–3)	0 (0–0)	81 (73–89)	8 (7–9)
Timor-Leste	2029	3 (2–3)	0 (0–0)	83 (73–93)	8 (6–10)
Timor-Leste	2030	3 (2–4)	0 (0–0)	84 (73–96)	8 (6–10)
Timor-Leste	2031	3 (2–4)	0 (0–0)	86 (72–100)	8 (6–10)
Timor-Leste	2032	3 (2–4)	0 (0–0)	88 (72–103)	8 (6–11)
Timor-Leste	2033	3 (2–4)	0 (0–0)	89 (72–107)	8 (5–11)
Timor-Leste	2034	3 (2–4)	0 (0–0)	91 (71–111)	8 (5–11)
Timor-Leste	2035	3 (2–5)	0 (0–0)	93 (71–114)	8 (5–12)
Timor-Leste	2036	3 (2–5)	0 (0–0)	94 (70–118)	8 (4–12)
Togo	2022	8 (7–8)	0 (0–0)	229 (226–231)	5 (5–5)
Togo	2023	8 (8–8)	0 (0–0)	239 (234–244)	5 (5–5)
Togo	2024	8 (8–8)	0 (0–0)	248 (240–257)	5 (5–5)
Togo	2025	9 (8–9)	0 (0–0)	258 (246–271)	5 (5–5)
Togo	2026	9 (8–9)	0 (0–0)	268 (252–285)	5 (5–5)
Togo	2027	9 (9–10)	0 (0–0)	278 (257–300)	5 (5–5)
Togo	2028	10 (9–10)	0 (0–0)	288 (262–315)	5 (5–5)
Togo	2029	10 (9–11)	0 (0–0)	298 (266–330)	5 (5–5)
Togo	2030	10 (9–11)	0 (0–0)	308 (270–346)	5 (5–6)
Togo	2031	10 (9–12)	0 (0–0)	318 (274–362)	5 (5–6)
Togo	2032	11 (9–12)	0 (0–0)	328 (277–379)	5 (5–6)
Togo	2033	11 (9–13)	0 (0–0)	338 (280–395)	5 (5–6)
Togo	2034	11 (10–13)	0 (0–0)	348 (283–412)	5 (5–6)

Togo	2035	12 (10–14)	0 (0–0)	358 (286–430)	5 (5–6)
Togo	2036	12 (10–15)	0 (0–0)	368 (288–447)	5 (5–6)
Tokelau	2022	0 (0–0)	0 (0–0)	0 (0–0)	13 (12–13)
Tokelau	2023	0 (0–0)	0 (0–0)	0 (0–0)	13 (12–13)
Tokelau	2024	0 (0–0)	0 (0–0)	0 (0–0)	13 (12–13)
Tokelau	2025	0 (0–0)	0 (0–0)	0 (0–0)	13 (12–13)
Tokelau	2026	0 (0–0)	0 (0–0)	0 (0–0)	13 (13–13)
Tokelau	2027	0 (0–0)	0 (0–0)	0 (0–0)	13 (13–13)
Tokelau	2028	0 (0–0)	0 (0–1)	0 (0–0)	13 (13–14)
Tokelau	2029	0 (0–0)	0 (0–1)	0 (0–0)	13 (13–14)
Tokelau	2030	0 (0–0)	0 (0–1)	0 (0–0)	14 (13–14)
Tokelau	2031	0 (0–0)	0 (0–1)	0 (0–0)	14 (13–14)
Tokelau	2032	0 (0–0)	0 (0–1)	0 (0–0)	14 (13–14)
Tokelau	2033	0 (0–0)	0 (0–1)	0 (0–0)	14 (13–14)
Tokelau	2034	0 (0–0)	0 (0–1)	0 (0–0)	14 (13–14)
Tokelau	2035	0 (0–0)	1 (0–1)	0 (0–0)	14 (13–14)
Tokelau	2036	0 (0–0)	1 (0–1)	0 (0–0)	14 (14–14)
Tonga	2022	1 (1–1)	1 (1–1)	23 (22–24)	28 (27–29)
Tonga	2023	1 (1–1)	1 (1–1)	23 (22–25)	28 (26–29)
Tonga	2024	1 (1–1)	1 (1–1)	24 (22–25)	27 (25–30)
Tonga	2025	1 (1–1)	1 (1–1)	24 (22–26)	27 (25–30)
Tonga	2026	1 (1–1)	1 (1–1)	24 (22–26)	27 (24–30)
Tonga	2027	1 (1–1)	1 (1–1)	24 (22–27)	27 (24–30)
Tonga	2028	1 (1–1)	1 (1–1)	25 (22–27)	27 (24–30)
Tonga	2029	1 (1–1)	1 (1–1)	25 (22–28)	27 (24–31)
Tonga	2030	1 (1–1)	1 (1–1)	25 (22–28)	27 (24–31)
Tonga	2031	1 (1–1)	1 (1–1)	26 (22–29)	27 (23–31)
Tonga	2032	1 (1–1)	1 (1–1)	26 (22–29)	27 (23–31)
Tonga	2033	1 (1–1)	1 (1–1)	26 (23–30)	27 (23–31)
Tonga	2034	1 (1–1)	1 (1–1)	27 (23–30)	27 (23–31)
Tonga	2035	1 (1–1)	1 (1–1)	27 (23–31)	27 (23–31)
Tonga	2036	1 (1–1)	1 (1–1)	27 (23–31)	27 (23–31)
Trinidad and Tobago	2022	11 (11–12)	1 (1–1)	299 (286–312)	15 (14–16)

Trinidad and Tobago	2023	11 (11–12)	1 (1–1)	304 (286–322)	15 (14–16)
Trinidad and Tobago	2024	12 (11–12)	1 (0–1)	309 (286–331)	15 (13–17)
Trinidad and Tobago	2025	12 (11–13)	1 (0–1)	314 (288–340)	15 (13–17)
Trinidad and Tobago	2026	12 (11–13)	1 (0–1)	319 (290–348)	15 (13–17)
Trinidad and Tobago	2027	12 (11–13)	1 (0–1)	324 (292–355)	15 (13–17)
Trinidad and Tobago	2028	12 (11–13)	1 (0–1)	328 (294–363)	15 (12–18)
Trinidad and Tobago	2029	12 (11–14)	1 (0–1)	333 (297–370)	15 (12–18)
Trinidad and Tobago	2030	13 (11–14)	1 (0–1)	338 (299–377)	15 (12–18)
Trinidad and Tobago	2031	13 (11–14)	1 (0–1)	343 (302–384)	15 (12–18)
Trinidad and Tobago	2032	13 (12–15)	1 (0–1)	348 (305–391)	15 (12–18)
Trinidad and Tobago	2033	13 (12–15)	1 (0–1)	353 (308–398)	15 (12–18)
Trinidad and Tobago	2034	13 (12–15)	1 (0–1)	358 (311–405)	15 (11–19)
Trinidad and Tobago	2035	14 (12–15)	1 (0–1)	363 (314–411)	15 (11–19)
Trinidad and Tobago	2036	14 (12–16)	1 (0–1)	368 (317–418)	15 (11–19)
Tunisia	2022	68 (68–69)	1 (0–1)	1692 (1678–1707)	12 (12–12)
Tunisia	2023	70 (69–71)	0 (0–1)	1741 (1717–1766)	12 (12–12)
Tunisia	2024	72 (70–73)	0 (0–1)	1786 (1746–1826)	12 (11–12)
Tunisia	2025	73 (71–76)	0 (0–1)	1834 (1778–1889)	12 (11–13)
Tunisia	2026	75 (72–78)	0 (0–1)	1879 (1805–1953)	12 (11–13)
Tunisia	2027	77 (73–81)	0 (0–1)	1926 (1833–2020)	12 (11–13)
Tunisia	2028	79 (74–84)	0 (0–1)	1972 (1858–2087)	12 (10–13)
Tunisia	2029	81 (75–86)	0 (0–1)	2019 (1882–2156)	12 (10–14)
Tunisia	2030	82 (76–89)	0 (0–1)	2065 (1904–2226)	12 (10–14)
Tunisia	2031	84 (76–92)	0 (0–1)	2112 (1925–2298)	12 (10–14)
Tunisia	2032	86 (77–95)	0 (0–1)	2158 (1946–2371)	12 (9–15)
Tunisia	2033	88 (78–98)	0 (0–1)	2205 (1965–2445)	12 (9–15)
Tunisia	2034	89 (78–101)	0 (0–1)	2251 (1982–2519)	12 (9–15)
Tunisia	2035	91 (79–104)	0 (0–1)	2297 (1999–2595)	12 (8–16)
Tunisia	2036	93 (79–107)	0 (0–1)	2344 (2015–2672)	12 (8–16)
Turkey	2022	1201 (1182–1219)	1 (1–1)	32346 (31884–32807)	32 (31–33)
Turkey	2023	1224 (1182–1267)	1 (1–1)	33024 (31991–34057)	32 (30–33)
Turkey	2024	1248 (1177–1319)	1 (1–1)	33703 (31974–35431)	32 (29–34)
Turkey	2025	1272 (1168–1376)	1 (1–1)	34381 (31851–36911)	32 (29–35)

Turkey	2026	1296 (1155–1436)	1 (1–1)	35060 (31634–38485)	32 (28–36)
Turkey	2027	1319 (1139–1500)	1 (1–1)	35738 (31332–40144)	32 (27–37)
Turkey	2028	1343 (1119–1567)	1 (1–1)	36417 (30952–41881)	32 (26–38)
Turkey	2029	1367 (1097–1637)	1 (1–1)	37095 (30498–43692)	32 (25–38)
Turkey	2030	1391 (1071–1710)	1 (1–1)	37774 (29977–45571)	32 (24–39)
Turkey	2031	1415 (1043–1786)	1 (1–2)	38452 (29390–47515)	32 (23–40)
Turkey	2032	1438 (1013–1864)	1 (1–2)	39131 (28741–49520)	31 (22–41)
Turkey	2033	1462 (980–1945)	1 (1–2)	39809 (28034–51584)	31 (21–42)
Turkey	2034	1486 (944–2027)	1 (1–2)	40488 (27270–53705)	31 (21–42)
Turkey	2035	1510 (907–2113)	1 (1–2)	41166 (26452–55881)	31 (20–43)
Turkey	2036	1533 (867–2200)	1 (1–2)	41845 (25581–58109)	31 (19–44)
Turkmenistan	2022	12 (11–13)	0 (0–0)	370 (341–399)	8 (7–9)
Turkmenistan	2023	12 (11–14)	0 (0–0)	381 (330–432)	8 (6–10)
Turkmenistan	2024	13 (10–15)	0 (0–0)	392 (326–458)	8 (6–11)
Turkmenistan	2025	13 (10–15)	0 (0–0)	403 (324–481)	9 (6–11)
Turkmenistan	2026	13 (10–16)	0 (0–0)	414 (325–503)	9 (6–12)
Turkmenistan	2027	14 (11–17)	0 (0–0)	425 (326–523)	9 (6–12)
Turkmenistan	2028	14 (11–17)	0 (0–0)	436 (329–543)	9 (5–13)
Turkmenistan	2029	14 (11–18)	0 (0–0)	447 (332–562)	9 (5–13)
Turkmenistan	2030	15 (11–18)	0 (0–0)	458 (335–580)	10 (5–14)
Turkmenistan	2031	15 (11–19)	0 (0–0)	469 (339–598)	10 (5–14)
Turkmenistan	2032	15 (11–19)	0 (0–1)	480 (343–616)	10 (5–15)
Turkmenistan	2033	16 (11–20)	0 (0–1)	491 (348–633)	10 (5–15)
Turkmenistan	2034	16 (11–21)	0 (0–1)	502 (353–650)	11 (5–16)
Turkmenistan	2035	16 (12–21)	0 (0–1)	513 (358–667)	11 (5–16)
Turkmenistan	2036	17 (12–22)	0 (0–1)	524 (363–684)	11 (5–17)
Tuvalu	2022	0 (0–0)	1 (1–1)	2 (2–2)	15 (15–15)
Tuvalu	2023	0 (0–0)	1 (1–1)	2 (2–2)	15 (15–15)
Tuvalu	2024	0 (0–0)	1 (1–1)	2 (2–2)	15 (15–15)
Tuvalu	2025	0 (0–0)	1 (1–1)	2 (2–2)	15 (15–16)
Tuvalu	2026	0 (0–0)	1 (1–1)	2 (2–2)	15 (15–16)
Tuvalu	2027	0 (0–0)	1 (1–1)	2 (2–2)	15 (15–16)
Tuvalu	2028	0 (0–0)	1 (1–1)	2 (2–2)	15 (15–16)

Tuvalu	2029	0 (0–0)	1 (1–1)	2 (2–2)	16 (15–16)
Tuvalu	2030	0 (0–0)	1 (1–1)	2 (2–2)	16 (15–17)
Tuvalu	2031	0 (0–0)	1 (1–1)	2 (2–2)	16 (14–17)
Tuvalu	2032	0 (0–0)	1 (1–1)	2 (2–2)	16 (14–17)
Tuvalu	2033	0 (0–0)	1 (1–1)	2 (2–2)	16 (14–18)
Tuvalu	2034	0 (0–0)	1 (0–1)	2 (2–2)	16 (14–18)
Tuvalu	2035	0 (0–0)	1 (0–1)	2 (2–2)	16 (14–18)
Tuvalu	2036	0 (0–0)	1 (0–1)	2 (2–2)	16 (14–18)
Uganda	2022	35 (35–36)	0 (0–0)	996 (982–1009)	6 (6–6)
Uganda	2023	36 (35–37)	0 (0–0)	1024 (995–1053)	6 (6–6)
Uganda	2024	37 (35–39)	0 (0–0)	1051 (1006–1096)	6 (6–7)
Uganda	2025	38 (36–40)	0 (0–0)	1077 (1016–1139)	6 (6–7)
Uganda	2026	39 (36–41)	0 (0–0)	1103 (1025–1182)	6 (6–7)
Uganda	2027	40 (36–43)	0 (0–0)	1128 (1033–1224)	6 (6–7)
Uganda	2028	40 (36–44)	0 (0–0)	1153 (1042–1265)	6 (6–7)
Uganda	2029	41 (37–45)	0 (0–0)	1178 (1050–1305)	6 (6–7)
Uganda	2030	42 (37–47)	0 (0–0)	1202 (1059–1344)	6 (5–7)
Uganda	2031	43 (37–48)	0 (0–0)	1225 (1068–1383)	6 (5–7)
Uganda	2032	43 (38–49)	0 (0–0)	1249 (1077–1421)	6 (5–7)
Uganda	2033	44 (38–51)	0 (0–0)	1272 (1087–1458)	6 (5–8)
Uganda	2034	45 (38–52)	0 (0–0)	1296 (1097–1494)	6 (5–8)
Uganda	2035	46 (39–53)	0 (0–0)	1319 (1107–1530)	6 (5–8)
Uganda	2036	47 (39–54)	0 (0–0)	1342 (1118–1566)	6 (5–7)
Ukraine	2022	750 (693–808)	1 (1–1)	22599 (20828–24369)	32 (30–35)
Ukraine	2023	757 (664–850)	1 (1–1)	22833 (19999–25667)	33 (28–37)
Ukraine	2024	761 (657–866)	1 (1–1)	22968 (19861–26076)	33 (28–38)
Ukraine	2025	765 (655–874)	1 (1–1)	23046 (19853–26239)	33 (28–38)
Ukraine	2026	767 (655–878)	1 (1–1)	23091 (19870–26312)	33 (28–38)
Ukraine	2027	768 (655–880)	1 (1–1)	23117 (19886–26348)	33 (28–38)
Ukraine	2028	769 (656–882)	1 (1–1)	23132 (19898–26365)	33 (28–39)
Ukraine	2029	769 (656–882)	1 (1–1)	23140 (19906–26375)	33 (28–39)
Ukraine	2030	770 (657–883)	1 (1–1)	23145 (19910–26380)	33 (28–39)
Ukraine	2031	770 (657–883)	1 (1–1)	23148 (19913–26383)	33 (28–39)

Ukraine	2032	770 (657–883)	1 (1–1)	23150 (19915–26385)	33 (28–39)
Ukraine	2033	770 (657–883)	1 (1–1)	23151 (19916–26386)	33 (28–39)
Ukraine	2034	770 (657–884)	1 (1–1)	23151 (19916–26387)	33 (28–39)
Ukraine	2035	770 (657–884)	1 (1–1)	23152 (19916–26387)	33 (28–39)
Ukraine	2036	770 (657–884)	1 (1–1)	23152 (19917–26387)	33 (28–39)
United Arab Emirates	2022	31 (28–34)	1 (1–1)	1101 (1002–1201)	17 (14–20)
United Arab Emirates	2023	32 (29–35)	1 (1–1)	1132 (1019–1245)	15 (10–21)
United Arab Emirates	2024	33 (29–37)	1 (1–1)	1163 (1037–1288)	14 (7–21)
United Arab Emirates	2025	34 (29–38)	1 (1–1)	1193 (1057–1330)	12 (4–21)
United Arab Emirates	2026	35 (30–39)	1 (0–1)	1224 (1077–1371)	11 (0–22)
United Arab Emirates	2027	35 (30–41)	1 (0–1)	1255 (1098–1411)	9 (-3–22)
United Arab Emirates	2028	36 (31–42)	1 (0–1)	1285 (1119–1451)	8 (-7–23)
United Arab Emirates	2029	37 (31–43)	1 (0–1)	1316 (1141–1491)	6 (-10–23)
United Arab Emirates	2030	38 (32–44)	1 (0–1)	1347 (1164–1529)	5 (-14–24)
United Arab Emirates	2031	39 (32–46)	1 (0–1)	1377 (1187–1568)	3 (-18–24)
United Arab Emirates	2032	40 (33–47)	1 (0–1)	1408 (1210–1606)	2 (-22–25)
United Arab Emirates	2033	41 (33–48)	1 (0–1)	1439 (1233–1644)	0 (-25–26)
United Arab Emirates	2034	41 (34–49)	1 (0–1)	1469 (1257–1682)	-1 (-29–27)
United Arab Emirates	2035	42 (34–50)	1 (0–1)	1500 (1280–1719)	-3 (-33–28)
United Arab Emirates	2036	43 (35–51)	1 (0–1)	1530 (1304–1757)	-4 (-38–29)
United Kingdom	2022	1641 (1594–1689)	1 (1–1)	33498 (32423–34574)	27 (26–28)
United Kingdom	2023	1642 (1577–1707)	1 (1–1)	33342 (31821–34863)	27 (25–28)
United Kingdom	2024	1643 (1566–1720)	1 (1–1)	33185 (31322–35048)	26 (24–28)
United Kingdom	2025	1644 (1558–1729)	1 (1–1)	33028 (30877–35179)	26 (24–28)
United Kingdom	2026	1644 (1551–1737)	1 (1–1)	32871 (30466–35277)	25 (23–27)
United Kingdom	2027	1645 (1546–1743)	1 (1–1)	32715 (30080–35349)	25 (22–27)
United Kingdom	2028	1646 (1542–1749)	1 (1–1)	32558 (29712–35404)	24 (21–27)
United Kingdom	2029	1646 (1539–1753)	1 (1–1)	32401 (29359–35444)	23 (20–26)
United Kingdom	2030	1647 (1537–1757)	1 (1–1)	32245 (29018–35471)	23 (20–26)
United Kingdom	2031	1647 (1534–1760)	1 (1–1)	32088 (28687–35489)	22 (19–26)
United Kingdom	2032	1648 (1533–1763)	1 (1–1)	31931 (28364–35499)	22 (18–25)
United Kingdom	2033	1648 (1531–1765)	1 (1–1)	31774 (28048–35500)	21 (18–25)
United Kingdom	2034	1648 (1530–1767)	1 (1–1)	31618 (27740–35496)	21 (17–24)

United Kingdom	2035	1649 (1529–1769)	1 (1–1)	31461 (27436–35486)	20 (16–24)
United Kingdom	2036	1649 (1528–1770)	1 (1–1)	31304 (27138–35470)	20 (15–24)
United Republic of Tanzania	2022	62 (62–63)	0 (0–0)	1741 (1728–1753)	6 (6–6)
United Republic of Tanzania	2023	64 (63–65)	0 (0–0)	1799 (1767–1831)	6 (6–6)
United Republic of Tanzania	2024	65 (63–68)	0 (0–0)	1857 (1798–1916)	6 (6–6)
United Republic of Tanzania	2025	67 (64–70)	0 (0–0)	1914 (1823–2006)	6 (6–7)
United Republic of Tanzania	2026	69 (64–73)	0 (0–0)	1972 (1844–2100)	6 (6–7)
United Republic of Tanzania	2027	70 (64–76)	0 (0–0)	2029 (1860–2199)	6 (6–7)
United Republic of Tanzania	2028	72 (64–80)	0 (0–0)	2087 (1872–2302)	6 (6–7)
United Republic of Tanzania	2029	73 (63–83)	0 (0–0)	2144 (1880–2409)	6 (5–7)
United Republic of Tanzania	2030	75 (63–87)	0 (0–0)	2202 (1885–2518)	6 (5–7)
United Republic of Tanzania	2031	76 (63–90)	0 (0–0)	2259 (1888–2631)	6 (5–7)
United Republic of Tanzania	2032	78 (62–94)	0 (0–0)	2317 (1887–2747)	6 (5–8)
United Republic of Tanzania	2033	80 (61–98)	0 (0–0)	2374 (1883–2865)	6 (5–8)
United Republic of Tanzania	2034	81 (61–102)	0 (0–0)	2432 (1877–2986)	6 (5–8)
United Republic of Tanzania	2035	83 (60–106)	0 (0–0)	2489 (1869–3110)	6 (5–8)
United Republic of Tanzania	2036	84 (59–110)	0 (0–0)	2547 (1857–3236)	6 (5–8)
United States of America	2022	8006 (7908–8103)	1 (1–1)	182561 (180137–184986	32 (31–33)
United States of America	2023	8108 (7955–8262)	1 (1–1)	184445 (180445–188444	32 (31–33)
United States of America	2024	8211 (8004–8418)	1 (1–1)	186296 (181035–191557	31 (30–32)
United States of America	2025	8314 (8053–8575)	1 (1–1)	188137 (181823–194452	31 (30–32)
United States of America	2026	8417 (8100–8734)	1 (1–1)	189975 (182749–197202	31 (29–32)
United States of America	2027	8520 (8145–8894)	1 (1–1)	191813 (183773–199852	31 (29–32)
United States of America	2028	8623 (8188–9057)	1 (1–1)	193649 (184872–202427	30 (29–32)
United States of America	2029	8726 (8229–9222)	1 (1–1)	195486 (186027–204945	30 (28–32)
United States of America	2030	8828 (8268–9389)	1 (1–1)	197323 (187229–207417	30 (28–32)
United States of America	2031	8931 (8304–9559)	1 (1–1)	199160 (188467–209852	29 (27–31)
United States of America	2032	9034 (8338–9730)	1 (1–1)	200996 (189738–212255	29 (27–31)
United States of America	2033	9137 (8370–9904)	1 (1–1)	202833 (191036–214630	29 (27–31)
United States of America	2034	9240 (8400–10080	1 (1–1)	204670 (192357–216983	29 (26–31)
United States of America	2035	9343 (8428–10258	1 (1–1)	206507 (193699–219314	28 (26–31)
United States of America	2036	9446 (8454–10437	1 (1–1)	208343 (195060–221627	28 (26–30)
United States Virgin Islands	2022	1 (1–1)	0 (0–0)	15 (13–16)	9 (9–10)

United States Virgin Islands	2023	1 (0–1)	0 (0–0)	14 (11–17)	9 (8–11)
United States Virgin Islands	2024	1 (0–1)	0 (0–0)	13 (9–17)	10 (7–12)
United States Virgin Islands	2025	1 (0–1)	0 (0–1)	12 (6–18)	10 (7–13)
United States Virgin Islands	2026	0 (0–1)	0 (0–1)	11 (4–18)	10 (7–13)
United States Virgin Islands	2027	0 (0–1)	0 (0–1)	10 (1–19)	10 (7–14)
United States Virgin Islands	2028	0 (-0–1)	0 (0–1)	9 (-2–20)	11 (7–14)
United States Virgin Islands	2029	0 (-0–1)	0 (0–1)	8 (-5–21)	11 (7–15)
United States Virgin Islands	2030	0 (-0–1)	0 (0–1)	7 (-8–23)	11 (7–15)
United States Virgin Islands	2031	0 (-1–1)	0 (0–1)	6 (-12–24)	11 (7–15)
United States Virgin Islands	2032	0 (-1–1)	0 (0–1)	5 (-15–26)	11 (7–15)
United States Virgin Islands	2033	0 (-1–1)	0 (0–1)	4 (-18–27)	11 (7–15)
United States Virgin Islands	2034	0 (-1–1)	0 (0–1)	3 (-22–29)	11 (7–15)
United States Virgin Islands	2035	0 (-1–1)	0 (0–1)	2 (-26–31)	11 (7–16)
United States Virgin Islands	2036	0 (-1–2)	0 (0–1)	1 (-29–32)	11 (7–16)
Uruguay	2022	94 (90–97)	2 (2–2)	2328 (2236–2419)	48 (46–50)
Uruguay	2023	95 (90–99)	2 (2–2)	2360 (2249–2472)	48 (45–51)
Uruguay	2024	96 (91–101)	2 (2–2)	2380 (2247–2512)	48 (44–52)
Uruguay	2025	97 (91–102)	2 (2–2)	2403 (2253–2552)	48 (44–52)
Uruguay	2026	98 (92–104)	2 (2–2)	2425 (2260–2590)	48 (43–53)
Uruguay	2027	99 (92–105)	2 (2–2)	2447 (2268–2627)	48 (43–53)
Uruguay	2028	100 (93–107)	2 (2–2)	2470 (2277–2662)	48 (42–54)
Uruguay	2029	101 (93–108)	2 (2–2)	2492 (2288–2697)	48 (42–54)
Uruguay	2030	102 (94–110)	2 (2–2)	2515 (2298–2731)	48 (41–55)
Uruguay	2031	103 (95–111)	2 (2–2)	2537 (2310–2764)	48 (41–55)
Uruguay	2032	104 (95–112)	2 (2–2)	2559 (2322–2797)	48 (41–55)
Uruguay	2033	105 (96–114)	2 (2–2)	2582 (2334–2829)	48 (40–56)
Uruguay	2034	106 (97–115)	2 (2–2)	2604 (2347–2861)	48 (40–56)
Uruguay	2035	107 (97–116)	2 (1–2)	2626 (2360–2893)	48 (40–56)
Uruguay	2036	108 (98–118)	2 (1–2)	2649 (2373–2925)	48 (40–56)
Uzbekistan	2022	65 (63–68)	0 (0–0)	2038 (1957–2119)	6 (6–7)
Uzbekistan	2023	67 (63–72)	0 (0–0)	2104 (1964–2243)	7 (6–7)
Uzbekistan	2024	70 (63–76)	0 (0–0)	2169 (1967–2372)	7 (6–7)
Uzbekistan	2025	72 (63–80)	0 (0–0)	2235 (1965–2506)	7 (6–7)

Uzbekistan	2026	74 (63–85)	0 (0–0)	2301 (1957–2646)	7 (6–8)
Uzbekistan	2027	76 (63–90)	0 (0–0)	2367 (1944–2790)	7 (6–8)
Uzbekistan	2028	78 (62–95)	0 (0–0)	2433 (1927–2939)	7 (6–8)
Uzbekistan	2029	81 (61–100)	0 (0–0)	2499 (1904–3093)	7 (6–8)
Uzbekistan	2030	83 (60–105)	0 (0–0)	2565 (1878–3252)	7 (6–8)
Uzbekistan	2031	85 (59–110)	0 (0–0)	2631 (1847–3414)	7 (6–8)
Uzbekistan	2032	87 (58–116)	0 (0–0)	2697 (1812–3581)	8 (6–9)
Uzbekistan	2033	89 (57–122)	0 (0–0)	2762 (1773–3752)	8 (7–9)
Uzbekistan	2034	92 (56–127)	0 (0–0)	2828 (1730–3926)	8 (7–9)
Uzbekistan	2035	94 (54–133)	0 (0–0)	2894 (1684–4105)	8 (7–9)
Uzbekistan	2036	96 (52–139)	0 (0–0)	2960 (1634–4286)	8 (7–9)
Vanuatu	2022	1 (1–1)	0 (0–0)	16 (16–16)	8 (8–8)
Vanuatu	2023	1 (1–1)	0 (0–0)	16 (16–17)	8 (8–8)
Vanuatu	2024	1 (1–1)	0 (0–0)	17 (16–17)	8 (7–8)
Vanuatu	2025	1 (1–1)	0 (0–0)	17 (17–18)	8 (7–8)
Vanuatu	2026	1 (1–1)	0 (0–0)	18 (17–18)	8 (7–8)
Vanuatu	2027	1 (1–1)	0 (0–0)	18 (17–19)	8 (7–9)
Vanuatu	2028	1 (1–1)	0 (0–0)	19 (18–20)	8 (7–9)
Vanuatu	2029	1 (1–1)	0 (0–0)	19 (18–20)	8 (7–9)
Vanuatu	2030	1 (1–1)	0 (0–0)	19 (18–21)	8 (7–9)
Vanuatu	2031	1 (1–1)	0 (0–0)	20 (18–21)	8 (6–9)
Vanuatu	2032	1 (1–1)	0 (0–0)	20 (19–22)	8 (6–9)
Vanuatu	2033	1 (1–1)	0 (0–0)	21 (19–23)	8 (6–9)
Vanuatu	2034	1 (1–1)	0 (0–0)	21 (19–23)	8 (6–9)
Vanuatu	2035	1 (1–1)	0 (0–0)	22 (19–24)	8 (6–9)
Vanuatu	2036	1 (1–1)	0 (0–0)	22 (20–25)	7 (5–10)
Venezuela (Bolivarian Republic of)	2022	140 (130–150)	0 (0–1)	3659 (3381–3938)	11 (9–13)
Venezuela (Bolivarian Republic of)	2023	144 (127–161)	0 (0–1)	3757 (3287–4227)	11 (8–15)
Venezuela (Bolivarian Republic of)	2024	148 (125–170)	0 (0–1)	3855 (3251–4458)	11 (6–16)
Venezuela (Bolivarian Republic of)	2025	151 (125–177)	0 (0–1)	3952 (3240–4664)	11 (6–17)
Venezuela (Bolivarian Republic of)	2026	155 (126–185)	0 (0–1)	4050 (3244–4856)	11 (5–17)
Venezuela (Bolivarian Republic of)	2027	159 (126–192)	0 (0–1)	4148 (3257–5038)	11 (5–17)
Venezuela (Bolivarian Republic of)	2028	163 (127–198)	0 (0–1)	4245 (3278–5213)	11 (5–18)

Venezuela (Bolivarian Republic of)	2029	167 (128–205)	0 (0–1)	4343 (3304–5382)	11 (4–18)
Venezuela (Bolivarian Republic of)	2030	170 (130–211)	0 (0–1)	4441 (3335–5546)	11 (4–18)
Venezuela (Bolivarian Republic of)	2031	174 (131–217)	0 (0–1)	4539 (3370–5707)	11 (4–18)
Venezuela (Bolivarian Republic of)	2032	178 (133–223)	0 (0–1)	4636 (3408–5864)	11 (4–18)
Venezuela (Bolivarian Republic of)	2033	182 (135–229)	0 (0–1)	4734 (3449–6019)	11 (4–18)
Venezuela (Bolivarian Republic of)	2034	185 (136–235)	0 (0–1)	4832 (3492–6171)	11 (4–18)
Venezuela (Bolivarian Republic of)	2035	189 (138–240)	0 (0–1)	4929 (3538–6321)	11 (4–18)
Venezuela (Bolivarian Republic of)	2036	193 (140–246)	0 (0–1)	5027 (3585–6470)	11 (4–18)
Viet Nam	2022	397 (395–400)	0 (0–0)	11136 (11070–11202)	10 (10–10)
Viet Nam	2023	410 (404–416)	0 (0–0)	11473 (11325–11620)	10 (10–10)
Viet Nam	2024	423 (413–433)	0 (0–0)	11810 (11563–12056)	10 (10–10)
Viet Nam	2025	436 (422–451)	0 (0–0)	12146 (11785–12508)	10 (10–10)
Viet Nam	2026	449 (430–469)	0 (0–0)	12483 (11994–12972)	10 (10–11)
Viet Nam	2027	462 (437–487)	0 (0–0)	12820 (12191–13449)	10 (10–11)
Viet Nam	2028	475 (444–506)	0 (0–0)	13157 (12377–13938)	10 (10–11)
Viet Nam	2029	488 (450–526)	0 (0–0)	13494 (12552–14436)	10 (10–11)
Viet Nam	2030	501 (456–545)	0 (0–0)	13831 (12718–14944)	10 (10–11)
Viet Nam	2031	514 (462–565)	0 (0–0)	14168 (12874–15462)	11 (10–11)
Viet Nam	2032	527 (468–586)	0 (0–0)	14505 (13022–15988)	11 (10–11)
Viet Nam	2033	540 (473–607)	0 (0–0)	14842 (13161–16523)	11 (10–11)
Viet Nam	2034	553 (477–628)	0 (0–0)	15179 (13292–17066)	11 (10–12)
Viet Nam	2035	566 (482–649)	0 (0–0)	15516 (13415–17617)	11 (10–12)
Viet Nam	2036	579 (486–671)	0 (0–0)	15853 (13531–18175)	11 (10–12)
Yemen	2022	40 (39–41)	0 (0–0)	1163 (1144–1182)	7 (7–7)
Yemen	2023	41 (40–43)	0 (0–0)	1204 (1168–1241)	7 (7–7)
Yemen	2024	43 (41–44)	0 (0–0)	1246 (1192–1299)	7 (7–8)
Yemen	2025	44 (42–46)	0 (0–0)	1287 (1216–1358)	7 (7–8)
Yemen	2026	45 (43–48)	0 (0–0)	1328 (1239–1418)	7 (7–8)
Yemen	2027	47 (43–50)	0 (0–0)	1370 (1261–1478)	7 (7–8)
Yemen	2028	48 (44–52)	0 (0–0)	1411 (1282–1540)	7 (7–8)
Yemen	2029	49 (45–54)	0 (0–0)	1452 (1302–1602)	7 (7–8)
Yemen	2030	51 (45–56)	0 (0–0)	1493 (1321–1666)	8 (7–8)
Yemen	2031	52 (46–58)	0 (0–0)	1535 (1339–1730)	8 (7–8)

Yemen	2032	53 (46–61)	0 (0–0)	1576 (1356–1796)	8 (7–8)
Yemen	2033	55 (47–63)	0 (0–0)	1617 (1373–1862)	8 (7–9)
Yemen	2034	56 (47–65)	0 (0–0)	1659 (1388–1929)	8 (7–9)
Yemen	2035	57 (48–67)	0 (0–0)	1700 (1403–1997)	8 (7–9)
Yemen	2036	59 (48–69)	0 (0–0)	1741 (1417–2066)	8 (7–9)
Zambia	2022	18 (18–18)	0 (0–0)	503 (497–508)	7 (7–7)
Zambia	2023	18 (18–19)	0 (0–0)	515 (503–526)	7 (6–7)
Zambia	2024	19 (18–19)	0 (0–0)	527 (508–546)	7 (6–7)
Zambia	2025	19 (18–20)	0 (0–0)	539 (511–567)	6 (6–7)
Zambia	2026	19 (18–21)	0 (0–0)	551 (513–589)	6 (6–7)
Zambia	2027	19 (17–21)	0 (0–0)	563 (514–612)	6 (5–7)
Zambia	2028	20 (17–22)	0 (0–0)	575 (515–636)	6 (5–8)
Zambia	2029	20 (17–23)	0 (0–0)	588 (514–661)	6 (5–8)
Zambia	2030	20 (17–24)	0 (0–0)	600 (513–687)	6 (4–8)
Zambia	2031	21 (16–25)	0 (0–0)	612 (511–713)	6 (4–8)
Zambia	2032	21 (16–26)	0 (0–0)	624 (508–740)	6 (4–9)
Zambia	2033	21 (16–27)	0 (0–0)	636 (505–768)	6 (3–9)
Zambia	2034	21 (15–27)	0 (0–0)	648 (501–796)	6 (3–9)
Zambia	2035	22 (15–28)	0 (0–0)	661 (497–825)	6 (3–9)
Zambia	2036	22 (14–29)	0 (0–0)	673 (491–854)	6 (2–10)
Zimbabwe	2022	55 (54–56)	1 (1–1)	1646 (1610–1682)	21 (20–22)
Zimbabwe	2023	54 (51–57)	1 (1–1)	1662 (1585–1739)	21 (19–22)
Zimbabwe	2024	53 (48–58)	1 (1–1)	1676 (1552–1800)	21 (18–23)
Zimbabwe	2025	53 (45–61)	1 (1–1)	1689 (1514–1863)	20 (17–24)
Zimbabwe	2026	53 (43–63)	1 (1–1)	1700 (1473–1927)	20 (15–25)
Zimbabwe	2027	52 (40–65)	1 (1–1)	1710 (1429–1992)	20 (14–26)
Zimbabwe	2028	52 (38–66)	1 (1–1)	1719 (1383–2056)	20 (12–28)
Zimbabwe	2029	52 (36–68)	1 (1–1)	1727 (1335–2120)	20 (10–29)
Zimbabwe	2030	52 (35–70)	1 (1–1)	1735 (1287–2182)	19 (8–31)
Zimbabwe	2031	52 (33–71)	1 (1–1)	1741 (1238–2244)	19 (6–32)
Zimbabwe	2032	52 (31–73)	1 (1–1)	1747 (1190–2304)	19 (4–34)
Zimbabwe	2033	52 (30–74)	1 (1–1)	1752 (1141–2363)	19 (2–36)
Zimbabwe	2034	52 (29–76)	1 (1–1)	1757 (1092–2421)	19 (-1–38)

Zimbabwe	2035	52 (27–77)	1 (1–1)	1761 (1044–2477)	18 (-3–40)
Zimbabwe	2036	52 (26–78)	1 (1–1)	1764 (996–2533)	18 (-5–42)

Supplement table.7 Disease burden prediction for colon and rectum cancer death and disability-adjusted life years numbers and age-standardized rate to 2036 in different countries.

Country	Year	Death		DALY	
		(N)	ASR(per100,000)	(N)	ASR(per100,000)
Afghanistan	2022	20 (20–20)	0 (0–0)	683 (677–689)	5 (5–5)
Afghanistan	2023	21 (20–21)	0 (0–0)	703 (691–716)	5 (5–5)
Afghanistan	2024	21 (20–22)	0 (0–0)	723 (702–744)	5 (5–5)
Afghanistan	2025	21 (20–22)	0 (0–0)	744 (713–774)	5 (5–5)
Afghanistan	2026	22 (20–23)	0 (0–0)	764 (722–806)	5 (5–5)
Afghanistan	2027	22 (20–24)	0 (0–0)	784 (730–838)	5 (5–6)
Afghanistan	2028	22 (20–24)	0 (0–0)	804 (738–871)	5 (4–6)
Afghanistan	2029	23 (20–25)	0 (0–0)	825 (744–905)	5 (4–6)
Afghanistan	2030	23 (20–26)	0 (0–0)	845 (750–940)	5 (4–6)
Afghanistan	2031	23 (20–27)	0 (0–0)	865 (755–975)	5 (4–6)
Afghanistan	2032	24 (20–28)	0 (0–0)	885 (759–1012)	5 (4–6)
Afghanistan	2033	24 (20–28)	0 (0–0)	905 (762–1049)	5 (4–6)
Afghanistan	2034	25 (20–29)	0 (0–0)	926 (765–1087)	5 (3–6)
Afghanistan	2035	25 (20–30)	0 (0–0)	946 (767–1125)	5 (3–6)
Afghanistan	2036	25 (19–31)	0 (0–0)	966 (768–1164)	5 (3–7)
Albania	2022	21 (20–21)	0 (0–0)	447 (430–464)	10 (10–11)
Albania	2023	21 (20–22)	0 (0–1)	454 (423–484)	10 (9–11)
Albania	2024	21 (19–23)	0 (0–1)	461 (419–503)	10 (9–11)
Albania	2025	22 (19–24)	0 (0–1)	468 (416–521)	10 (9–12)
Albania	2026	22 (19–25)	0 (0–1)	476 (414–537)	10 (8–12)
Albania	2027	22 (19–25)	0 (0–1)	483 (413–553)	10 (8–12)
Albania	2028	23 (19–26)	0 (0–1)	491 (413–568)	10 (8–12)
Albania	2029	23 (19–27)	0 (0–1)	498 (414–582)	10 (8–13)
Albania	2030	23 (19–27)	0 (0–1)	506 (415–596)	10 (7–13)
Albania	2031	24 (19–28)	0 (0–1)	513 (417–610)	10 (7–13)
Albania	2032	24 (19–29)	0 (0–1)	521 (419–623)	10 (7–13)
Albania	2033	24 (19–29)	0 (0–1)	529 (421–636)	10 (7–13)
Albania	2034	25 (20–30)	0 (0–1)	536 (424–649)	10 (7–13)
Albania	2035	25 (20–31)	0 (0–1)	544 (427–661)	10 (7–14)
Albania	2036	26 (20–31)	0 (0–1)	551 (430–673)	10 (7–14)
Algeria	2022	61 (60–62)	0 (0–0)	1543 (1529–1558)	4 (4–4)

Algeria	2023	62 (61–64)	0 (0–0)	1583 (1551–1615)	4 (4–4)
Algeria	2024	64 (61–66)	0 (0–0)	1623 (1570–1676)	4 (4–4)
Algeria	2025	65 (62–68)	0 (0–0)	1662 (1585–1740)	4 (4–4)
Algeria	2026	66 (62–71)	0 (0–0)	1702 (1597–1807)	4 (4–4)
Algeria	2027	67 (62–73)	0 (0–0)	1742 (1607–1877)	4 (3–5)
Algeria	2028	69 (61–76)	0 (0–0)	1782 (1614–1949)	4 (3–5)
Algeria	2029	70 (61–78)	0 (0–0)	1821 (1619–2024)	4 (3–5)
Algeria	2030	71 (61–81)	0 (0–0)	1861 (1622–2100)	4 (3–5)
Algeria	2031	72 (61–84)	0 (0–0)	1901 (1622–2179)	4 (3–5)
Algeria	2032	74 (60–87)	0 (0–0)	1940 (1621–2260)	4 (3–5)
Algeria	2033	75 (60–90)	0 (0–0)	1980 (1618–2342)	4 (2–5)
Algeria	2034	76 (59–93)	0 (0–0)	2020 (1614–2426)	4 (2–5)
Algeria	2035	77 (58–97)	0 (0–0)	2060 (1608–2511)	4 (2–6)
Algeria	2036	79 (58–100)	0 (0–0)	2099 (1600–2599)	4 (2–6)
American Samoa	2022	0 (0–0)	1 (1–1)	10 (10–10)	18 (17–19)
American Samoa	2023	0 (0–0)	1 (1–1)	10 (10–10)	18 (17–19)
American Samoa	2024	0 (0–0)	1 (1–1)	10 (10–11)	18 (17–19)
American Samoa	2025	0 (0–0)	1 (1–1)	10 (10–11)	18 (16–19)
American Samoa	2026	0 (0–0)	1 (1–1)	10 (10–11)	18 (16–19)
American Samoa	2027	0 (0–0)	1 (1–1)	11 (10–11)	18 (16–20)
American Samoa	2028	0 (0–0)	1 (1–1)	11 (10–11)	18 (16–20)
American Samoa	2029	0 (0–0)	1 (1–1)	11 (10–12)	18 (16–20)
American Samoa	2030	0 (0–0)	1 (1–1)	11 (10–12)	18 (16–20)
American Samoa	2031	0 (0–0)	1 (1–1)	11 (11–12)	18 (16–20)
American Samoa	2032	0 (0–0)	1 (1–1)	11 (11–12)	18 (15–20)
American Samoa	2033	0 (0–0)	1 (1–1)	12 (11–12)	18 (15–20)
American Samoa	2034	0 (0–0)	1 (1–1)	12 (11–12)	18 (15–20)
American Samoa	2035	0 (0–0)	1 (1–1)	12 (11–13)	18 (15–20)
American Samoa	2036	0 (0–0)	1 (1–1)	12 (11–13)	18 (15–21)
Andorra	2022	1 (1–1)	1 (1–1)	31 (28–34)	20 (18–22)
Andorra	2023	1 (1–1)	1 (1–1)	31 (26–35)	19 (16–22)
Andorra	2024	1 (1–1)	1 (1–1)	31 (25–36)	18 (15–22)
Andorra	2025	1 (1–1)	1 (1–1)	31 (25–37)	18 (14–22)

Andorra	2026	1 (1–2)	1 (0–1)	31 (24–37)	17 (13–22)
Andorra	2027	1 (1–2)	1 (0–1)	31 (23–38)	16 (12–21)
Andorra	2028	1 (1–2)	1 (0–1)	31 (23–39)	16 (11–21)
Andorra	2029	1 (1–2)	1 (0–1)	31 (22–39)	15 (10–21)
Andorra	2030	1 (1–2)	1 (0–1)	31 (22–40)	15 (9–21)
Andorra	2031	1 (1–2)	1 (0–1)	31 (21–40)	14 (8–20)
Andorra	2032	1 (1–2)	1 (0–1)	31 (21–41)	13 (7–20)
Andorra	2033	1 (1–2)	1 (0–1)	31 (20–41)	13 (6–20)
Andorra	2034	1 (1–2)	0 (0–1)	31 (20–41)	12 (5–19)
Andorra	2035	1 (1–2)	0 (0–1)	31 (19–42)	12 (4–19)
Andorra	2036	1 (1–2)	0 (0–1)	31 (19–42)	11 (3–19)
Angola	2022	28 (27–28)	0 (0–0)	850 (838–863)	6 (6–6)
Angola	2023	28 (28–29)	0 (0–0)	880 (852–908)	6 (6–6)
Angola	2024	29 (28–31)	0 (0–0)	910 (863–957)	6 (5–6)
Angola	2025	30 (28–32)	0 (0–0)	940 (871–1008)	6 (5–6)
Angola	2026	31 (28–34)	0 (0–0)	969 (877–1062)	6 (5–7)
Angola	2027	32 (28–36)	0 (0–0)	999 (880–1118)	6 (5–7)
Angola	2028	33 (28–37)	0 (0–0)	1029 (881–1176)	6 (5–7)
Angola	2029	34 (28–39)	0 (0–0)	1058 (880–1237)	6 (5–7)
Angola	2030	35 (28–41)	0 (0–0)	1088 (877–1299)	6 (5–7)
Angola	2031	36 (28–43)	0 (0–0)	1118 (873–1363)	6 (5–7)
Angola	2032	36 (28–45)	0 (0–0)	1147 (866–1428)	6 (5–7)
Angola	2033	37 (27–47)	0 (0–0)	1177 (859–1495)	6 (5–7)
Angola	2034	38 (27–49)	0 (0–0)	1207 (849–1564)	6 (5–7)
Angola	2035	39 (27–51)	0 (0–0)	1236 (838–1634)	6 (5–7)
Angola	2036	40 (26–54)	0 (0–0)	1266 (826–1706)	6 (5–7)
Antigua and Barbuda	2022	0 (0–0)	0 (0–0)	10 (9–10)	9 (8–9)
Antigua and Barbuda	2023	0 (0–0)	0 (0–0)	10 (9–11)	9 (8–9)
Antigua and Barbuda	2024	0 (0–0)	0 (0–0)	10 (9–11)	9 (7–10)
Antigua and Barbuda	2025	0 (0–0)	0 (0–0)	10 (9–11)	9 (7–10)
Antigua and Barbuda	2026	0 (0–0)	0 (0–0)	10 (9–11)	9 (7–10)
Antigua and Barbuda	2027	0 (0–0)	0 (0–0)	11 (9–12)	9 (7–10)
Antigua and Barbuda	2028	0 (0–0)	0 (0–0)	11 (10–12)	9 (7–10)

Antigua and Barbuda	2029	0 (0–0)	0 (0–0)	11 (10–12)	9 (7–10)
Antigua and Barbuda	2030	0 (0–1)	0 (0–0)	11 (10–13)	9 (7–11)
Antigua and Barbuda	2031	0 (0–1)	0 (0–0)	11 (10–13)	9 (6–11)
Antigua and Barbuda	2032	0 (0–1)	0 (0–0)	12 (10–13)	9 (6–11)
Antigua and Barbuda	2033	0 (0–1)	0 (0–0)	12 (10–13)	9 (6–11)
Antigua and Barbuda	2034	0 (0–1)	0 (0–0)	12 (10–14)	9 (6–11)
Antigua and Barbuda	2035	0 (0–1)	0 (0–0)	12 (10–14)	9 (6–11)
Antigua and Barbuda	2036	0 (0–1)	0 (0–0)	12 (10–14)	9 (6–11)
Argentina	2022	480 (462–497)	1 (1–1)	12749 (12298–13200)	23 (22–24)
Argentina	2023	461 (430–492)	1 (1–1)	12291 (11488–13093)	22 (20–24)
Argentina	2024	442 (396–488)	1 (1–1)	11833 (10644–13022)	21 (18–23)
Argentina	2025	423 (360–486)	1 (1–1)	11374 (9761–12988)	20 (16–23)
Argentina	2026	404 (323–486)	1 (0–1)	10916 (8842–12990)	18 (14–23)
Argentina	2027	385 (284–487)	1 (0–1)	10458 (7888–13028)	17 (12–23)
Argentina	2028	367 (244–489)	1 (0–1)	9999 (6901–13098)	16 (9–22)
Argentina	2029	348 (203–492)	1 (0–1)	9541 (5884–13198)	15 (7–22)
Argentina	2030	329 (161–497)	0 (0–1)	9083 (4837–13329)	13 (5–22)
Argentina	2031	310 (117–503)	0 (0–1)	8625 (3762–13487)	12 (2–22)
Argentina	2032	291 (72–510)	0 (-0–1)	8166 (2660–13673)	11 (-0–22)
Argentina	2033	272 (27–518)	0 (-0–1)	7708 (1532–13884)	10 (-3–22)
Argentina	2034	253 (-20–527)	0 (-0–1)	7250 (380–14120)	8 (-6–23)
Argentina	2035	235 (-68–537)	0 (-0–1)	6792 (-797–14380)	7 (-8–23)
Argentina	2036	216 (-116–548)	0 (-1–1)	6333 (-1997–14664)	6 (-11–23)
Armenia	2022	30 (28–32)	1 (1–1)	757 (706–807)	17 (16–19)
Armenia	2023	31 (28–33)	1 (1–1)	757 (685–829)	17 (16–19)
Armenia	2024	31 (28–34)	1 (1–1)	757 (669–845)	17 (15–20)
Armenia	2025	31 (27–35)	1 (1–1)	757 (655–858)	17 (15–20)
Armenia	2026	32 (27–36)	1 (1–1)	757 (643–870)	17 (15–20)
Armenia	2027	32 (27–36)	1 (1–1)	757 (632–881)	17 (15–20)
Armenia	2028	32 (27–37)	1 (1–1)	757 (622–891)	17 (15–20)
Armenia	2029	32 (27–38)	1 (1–1)	757 (613–900)	17 (14–21)
Armenia	2030	33 (27–38)	1 (1–1)	757 (604–909)	17 (14–21)
Armenia	2031	33 (27–39)	1 (1–1)	757 (596–917)	17 (14–21)

Armenia	2032	33 (27–40)	1 (1–1)	757 (588–925)	17 (14–21)
Armenia	2033	34 (27–40)	1 (0–1)	757 (581–933)	17 (14–21)
Armenia	2034	34 (27–41)	1 (0–1)	757 (574–940)	17 (14–21)
Armenia	2035	34 (27–41)	1 (0–1)	757 (567–947)	17 (14–21)
Armenia	2036	35 (28–42)	1 (0–1)	757 (560–953)	17 (14–21)
Australia	2022	175 (168–182)	0 (0–0)	4469 (4288–4651)	11 (11–12)
Australia	2023	173 (164–183)	0 (0–0)	4425 (4168–4682)	11 (10–12)
Australia	2024	172 (160–184)	0 (0–0)	4382 (4067–4696)	11 (9–12)
Australia	2025	171 (157–184)	0 (0–0)	4338 (3974–4701)	11 (9–12)
Australia	2026	169 (154–185)	0 (0–0)	4294 (3888–4700)	10 (8–13)
Australia	2027	168 (151–185)	0 (0–0)	4250 (3805–4695)	10 (7–13)
Australia	2028	167 (148–185)	0 (0–0)	4206 (3726–4687)	10 (7–13)
Australia	2029	165 (146–185)	0 (0–0)	4163 (3649–4676)	9 (6–13)
Australia	2030	164 (143–185)	0 (0–0)	4119 (3574–4664)	9 (5–13)
Australia	2031	163 (141–184)	0 (0–0)	4075 (3500–4650)	9 (4–14)
Australia	2032	161 (138–184)	0 (0–0)	4031 (3428–4634)	9 (3–14)
Australia	2033	160 (136–184)	0 (0–1)	3987 (3358–4617)	8 (3–14)
Australia	2034	159 (134–183)	0 (0–1)	3943 (3288–4599)	8 (2–15)
Australia	2035	157 (131–183)	0 (0–1)	3900 (3220–4579)	8 (1–15)
Australia	2036	156 (129–183)	0 (-0–1)	3856 (3152–4560)	8 (-0–15)
Austria	2022	87 (84–90)	0 (0–1)	2141 (2055–2227)	14 (13–14)
Austria	2023	84 (79–89)	0 (0–1)	2071 (1931–2211)	13 (12–14)
Austria	2024	81 (74–88)	0 (0–1)	2000 (1806–2194)	13 (11–14)
Austria	2025	78 (69–88)	0 (0–0)	1930 (1678–2181)	12 (11–13)
Austria	2026	76 (64–87)	0 (0–0)	1859 (1548–2170)	12 (10–13)
Austria	2027	73 (58–87)	0 (0–0)	1788 (1414–2162)	11 (9–13)
Austria	2028	70 (53–87)	0 (0–0)	1718 (1278–2158)	11 (9–13)
Austria	2029	67 (48–87)	0 (0–0)	1647 (1138–2157)	10 (8–12)
Austria	2030	64 (42–87)	0 (0–0)	1577 (995–2158)	10 (7–12)
Austria	2031	61 (36–87)	0 (0–0)	1506 (849–2163)	9 (7–11)
Austria	2032	59 (30–87)	0 (0–0)	1436 (701–2170)	9 (6–11)
Austria	2033	56 (24–87)	0 (0–0)	1365 (550–2180)	8 (6–11)
Austria	2034	53 (18–88)	0 (0–0)	1294 (396–2193)	8 (5–10)

Austria	2035	50 (12–88)	0 (0–0)	1224 (239–2208)	7 (4–10)
Austria	2036	47 (5–89)	0 (0–0)	1153 (80–2226)	7 (4–9)
Azerbaijan	2022	35 (34–37)	0 (0–0)	1055 (1019–1090)	9 (9–10)
Azerbaijan	2023	35 (33–38)	0 (0–0)	1071 (1006–1136)	9 (9–10)
Azerbaijan	2024	35 (31–39)	0 (0–0)	1047 (934–1160)	10 (9–10)
Azerbaijan	2025	35 (30–40)	0 (0–0)	1065 (910–1221)	10 (9–11)
Azerbaijan	2026	35 (28–41)	0 (0–0)	1049 (845–1252)	11 (9–12)
Azerbaijan	2027	35 (27–43)	0 (0–0)	1066 (821–1311)	11 (10–12)
Azerbaijan	2028	35 (26–44)	0 (0–0)	1053 (767–1340)	11 (10–13)
Azerbaijan	2029	35 (24–45)	0 (0–0)	1069 (747–1390)	12 (10–13)
Azerbaijan	2030	35 (23–46)	0 (0–0)	1058 (703–1413)	12 (11–13)
Azerbaijan	2031	35 (22–47)	0 (0–0)	1070 (687–1453)	12 (11–14)
Azerbaijan	2032	35 (21–48)	0 (0–0)	1061 (651–1470)	12 (10–13)
Azerbaijan	2033	35 (20–49)	0 (0–0)	1071 (638–1503)	12 (10–13)
Azerbaijan	2034	35 (19–50)	0 (0–0)	1062 (607–1517)	11 (10–13)
Azerbaijan	2035	35 (18–51)	0 (0–0)	1070 (595–1545)	11 (9–13)
Azerbaijan	2036	35 (18–52)	0 (0–0)	1063 (568–1557)	11 (9–12)
Bahamas	2022	2 (2–2)	0 (0–0)	55 (53–57)	12 (12–13)
Bahamas	2023	2 (2–2)	0 (0–1)	56 (54–58)	12 (12–13)
Bahamas	2024	2 (2–2)	0 (0–1)	57 (55–60)	12 (11–13)
Bahamas	2025	2 (2–2)	0 (0–1)	58 (55–61)	12 (11–14)
Bahamas	2026	2 (2–2)	0 (0–1)	59 (56–63)	12 (11–14)
Bahamas	2027	2 (2–2)	0 (0–1)	61 (57–64)	12 (11–14)
Bahamas	2028	2 (2–2)	0 (0–1)	62 (58–66)	12 (11–14)
Bahamas	2029	2 (2–3)	0 (0–1)	63 (58–67)	12 (11–14)
Bahamas	2030	2 (2–3)	0 (0–1)	64 (59–68)	12 (11–14)
Bahamas	2031	2 (2–3)	0 (0–1)	65 (60–70)	12 (11–14)
Bahamas	2032	2 (2–3)	0 (0–1)	66 (61–71)	12 (11–14)
Bahamas	2033	2 (2–3)	0 (0–1)	67 (62–72)	12 (10–14)
Bahamas	2034	2 (2–3)	0 (0–1)	68 (63–74)	12 (10–14)
Bahamas	2035	2 (2–3)	0 (0–1)	69 (64–75)	12 (10–14)
Bahamas	2036	3 (2–3)	0 (0–1)	70 (65–76)	12 (10–15)
Bahrain	2022	3 (3–3)	0 (0–0)	97 (96–99)	9 (9–9)

Bahrain	2023	3 (3–4)	0 (0–0)	103 (101–106)	9 (8–10)
Bahrain	2024	4 (4–4)	0 (0–0)	109 (105–114)	9 (8–10)
Bahrain	2025	4 (4–4)	0 (0–0)	115 (109–122)	9 (8–10)
Bahrain	2026	4 (4–4)	0 (0–0)	121 (112–130)	9 (7–11)
Bahrain	2027	4 (4–5)	0 (0–0)	127 (116–138)	9 (7–11)
Bahrain	2028	5 (4–5)	0 (0–0)	133 (119–147)	9 (7–11)
Bahrain	2029	5 (4–5)	0 (0–1)	139 (122–156)	9 (7–11)
Bahrain	2030	5 (4–6)	0 (0–1)	145 (124–165)	9 (6–12)
Bahrain	2031	5 (4–6)	0 (0–1)	151 (127–174)	9 (6–12)
Bahrain	2032	5 (4–6)	0 (0–1)	157 (130–184)	9 (6–12)
Bahrain	2033	6 (4–7)	0 (0–1)	163 (132–193)	9 (6–12)
Bahrain	2034	6 (5–7)	0 (0–1)	169 (134–203)	9 (6–12)
Bahrain	2035	6 (5–8)	0 (0–1)	174 (136–213)	9 (6–13)
Bahrain	2036	6 (5–8)	0 (0–1)	180 (138–223)	9 (5–13)
Bangladesh	2022	303 (295–312)	0 (0–0)	7604 (7442–7765)	5 (5–5)
Bangladesh	2023	312 (293–332)	0 (0–0)	7811 (7450–8173)	5 (5–6)
Bangladesh	2024	321 (289–353)	0 (0–0)	8018 (7414–8623)	5 (4–6)
Bangladesh	2025	330 (284–377)	0 (0–0)	8226 (7341–9111)	5 (4–6)
Bangladesh	2026	339 (276–403)	0 (0–0)	8433 (7235–9632)	5 (4–6)
Bangladesh	2027	348 (267–430)	0 (0–0)	8641 (7099–10182)	5 (4–6)
Bangladesh	2028	357 (256–459)	0 (0–0)	8848 (6935–10760)	5 (4–6)
Bangladesh	2029	366 (244–489)	0 (0–0)	9055 (6747–11364)	4 (3–6)
Bangladesh	2030	375 (231–520)	0 (0–0)	9263 (6534–11991)	4 (3–6)
Bangladesh	2031	384 (216–552)	0 (0–0)	9470 (6299–12641)	4 (3–6)
Bangladesh	2032	393 (201–586)	0 (0–0)	9677 (6042–13313)	4 (3–5)
Bangladesh	2033	402 (184–621)	0 (0–0)	9885 (5764–14005)	4 (3–5)
Bangladesh	2034	411 (166–656)	0 (0–0)	10092 (5467–14717)	4 (3–5)
Bangladesh	2035	420 (147–693)	0 (0–0)	10299 (5150–15448)	4 (2–5)
Bangladesh	2036	429 (128–731)	0 (0–0)	10507 (4815–16198)	4 (2–5)
Barbados	2022	2 (2–2)	0 (0–0)	49 (47–52)	10 (9–10)
Barbados	2023	2 (2–2)	0 (0–0)	50 (46–54)	10 (9–11)
Barbados	2024	2 (2–2)	0 (0–0)	51 (46–55)	10 (9–11)
Barbados	2025	2 (2–3)	0 (0–0)	51 (46–56)	10 (9–11)

Barbados	2026	2 (2–3)	0 (0–0)	52 (46–58)	10 (9–11)
Barbados	2027	2 (2–3)	0 (0–0)	53 (46–59)	10 (9–11)
Barbados	2028	2 (2–3)	0 (0–0)	53 (46–60)	10 (9–11)
Barbados	2029	2 (2–3)	0 (0–0)	54 (47–61)	10 (9–12)
Barbados	2030	2 (2–3)	0 (0–0)	55 (47–63)	10 (9–12)
Barbados	2031	2 (2–3)	0 (0–0)	55 (47–64)	10 (9–12)
Barbados	2032	2 (2–3)	0 (0–0)	56 (48–65)	10 (9–12)
Barbados	2033	3 (2–3)	0 (0–0)	57 (48–66)	10 (9–12)
Barbados	2034	3 (2–3)	0 (0–0)	58 (48–67)	10 (9–12)
Barbados	2035	3 (2–3)	0 (0–0)	58 (49–68)	10 (9–12)
Barbados	2036	3 (2–3)	0 (0–0)	59 (49–69)	10 (9–12)
Belarus	2022	147 (134–159)	1 (1–1)	3867 (3507–4226)	25 (22–27)
Belarus	2023	145 (127–162)	1 (1–1)	3812 (3351–4274)	25 (21–28)
Belarus	2024	143 (123–162)	1 (1–1)	3769 (3252–4285)	25 (20–29)
Belarus	2025	141 (120–162)	1 (1–1)	3734 (3185–4282)	25 (20–30)
Belarus	2026	140 (118–162)	1 (1–1)	3706 (3137–4274)	25 (19–31)
Belarus	2027	139 (116–162)	1 (1–1)	3683 (3102–4264)	25 (19–31)
Belarus	2028	138 (114–162)	1 (1–1)	3665 (3076–4254)	25 (18–32)
Belarus	2029	137 (113–161)	1 (1–1)	3650 (3057–4244)	25 (18–32)
Belarus	2030	137 (112–161)	1 (1–1)	3639 (3042–4236)	25 (17–33)
Belarus	2031	136 (111–161)	1 (1–1)	3630 (3031–4229)	25 (17–33)
Belarus	2032	136 (111–160)	1 (1–1)	3622 (3022–4222)	25 (16–33)
Belarus	2033	135 (110–160)	1 (1–1)	3616 (3015–4217)	25 (16–34)
Belarus	2034	135 (110–160)	1 (1–1)	3611 (3010–4213)	25 (16–34)
Belarus	2035	134 (109–159)	1 (1–1)	3607 (3005–4209)	25 (15–34)
Belarus	2036	134 (109–159)	1 (1–1)	3604 (3002–4207)	25 (15–35)
Belgium	2022	129 (123–135)	1 (1–1)	2981 (2813–3148)	14 (13–15)
Belgium	2023	127 (118–136)	1 (0–1)	2921 (2684–3158)	14 (12–15)
Belgium	2024	124 (114–135)	0 (0–1)	2862 (2572–3152)	13 (11–15)
Belgium	2025	122 (110–134)	0 (0–1)	2802 (2467–3137)	12 (11–14)
Belgium	2026	120 (106–133)	0 (0–1)	2743 (2368–3118)	12 (10–14)
Belgium	2027	117 (102–132)	0 (0–1)	2684 (2273–3094)	11 (9–14)
Belgium	2028	115 (99–131)	0 (0–0)	2624 (2181–3067)	11 (8–13)

Belgium	2029	112 (95–130)	0 (0–0)	2565 (2091–3039)	10 (7–13)
Belgium	2030	110 (92–128)	0 (0–0)	2505 (2003–3008)	9 (7–12)
Belgium	2031	107 (88–127)	0 (0–0)	2446 (1916–2976)	9 (6–12)
Belgium	2032	105 (85–125)	0 (0–0)	2387 (1831–2942)	8 (5–11)
Belgium	2033	102 (81–124)	0 (0–0)	2327 (1747–2908)	8 (4–11)
Belgium	2034	100 (78–122)	0 (0–0)	2268 (1664–2872)	7 (4–10)
Belgium	2035	98 (75–120)	0 (0–0)	2209 (1582–2835)	6 (3–10)
Belgium	2036	95 (72–119)	0 (0–0)	2149 (1500–2798)	6 (2–10)
Belize	2022	1 (1–1)	0 (0–0)	24 (23–25)	7 (7–8)
Belize	2023	1 (1–1)	0 (0–0)	25 (23–26)	7 (6–8)
Belize	2024	1 (1–1)	0 (0–0)	25 (23–27)	7 (6–9)
Belize	2025	1 (1–1)	0 (0–0)	26 (24–28)	7 (6–9)
Belize	2026	1 (1–1)	0 (0–0)	26 (24–29)	7 (5–9)
Belize	2027	1 (1–1)	0 (0–0)	27 (24–30)	7 (5–10)
Belize	2028	1 (1–1)	0 (0–0)	28 (25–31)	7 (5–10)
Belize	2029	1 (1–1)	0 (0–0)	28 (25–31)	7 (4–10)
Belize	2030	1 (1–1)	0 (0–0)	29 (25–32)	7 (4–10)
Belize	2031	1 (1–1)	0 (0–0)	29 (26–33)	7 (4–11)
Belize	2032	1 (1–1)	0 (0–0)	30 (26–34)	7 (4–11)
Belize	2033	1 (1–1)	0 (0–0)	30 (26–35)	7 (4–11)
Belize	2034	1 (1–1)	0 (0–0)	31 (27–35)	7 (3–11)
Belize	2035	1 (1–1)	0 (0–0)	32 (27–36)	7 (3–11)
Belize	2036	1 (1–1)	0 (0–0)	32 (28–37)	7 (3–12)
Benin	2022	3 (3–3)	0 (0–0)	78 (76–79)	1 (1–1)
Benin	2023	3 (3–3)	0 (0–0)	79 (77–81)	1 (1–1)
Benin	2024	3 (3–3)	0 (0–0)	80 (77–82)	1 (1–1)
Benin	2025	3 (3–3)	0 (0–0)	81 (78–84)	1 (1–1)
Benin	2026	3 (3–3)	0 (0–0)	82 (78–85)	1 (1–1)
Benin	2027	3 (3–3)	0 (0–0)	83 (79–87)	1 (1–1)
Benin	2028	3 (3–3)	0 (0–0)	84 (80–88)	1 (1–1)
Benin	2029	3 (3–3)	0 (0–0)	85 (80–90)	1 (1–1)
Benin	2030	3 (3–3)	0 (0–0)	86 (81–91)	1 (1–1)
Benin	2031	3 (3–3)	0 (0–0)	87 (82–92)	1 (1–1)

Benin	2032	3 (3–3)	0 (0–0)	88 (83–93)	1 (1–1)
Benin	2033	3 (3–4)	0 (0–0)	89 (83–95)	1 (1–1)
Benin	2034	3 (3–4)	0 (0–0)	90 (84–96)	1 (1–1)
Benin	2035	3 (3–4)	0 (0–0)	91 (85–97)	1 (1–1)
Benin	2036	3 (3–4)	0 (0–0)	92 (86–99)	1 (1–1)
Bermuda	2022	1 (1–1)	1 (1–1)	19 (18–20)	15 (14–16)
Bermuda	2023	1 (1–1)	1 (1–1)	20 (19–22)	15 (14–17)
Bermuda	2024	1 (1–1)	1 (1–1)	20 (18–22)	15 (13–17)
Bermuda	2025	1 (1–1)	1 (0–1)	21 (18–24)	15 (12–18)
Bermuda	2026	1 (1–1)	1 (0–1)	21 (17–25)	15 (12–19)
Bermuda	2027	1 (1–1)	1 (0–1)	22 (16–27)	15 (11–19)
Bermuda	2028	1 (1–1)	1 (0–1)	22 (15–28)	15 (10–20)
Bermuda	2029	1 (1–1)	1 (0–1)	22 (15–30)	15 (9–21)
Bermuda	2030	1 (1–1)	1 (0–1)	22 (14–31)	15 (8–21)
Bermuda	2031	1 (1–1)	1 (0–1)	23 (13–33)	15 (8–22)
Bermuda	2032	1 (1–2)	1 (0–1)	23 (11–35)	15 (7–23)
Bermuda	2033	1 (1–2)	1 (0–1)	24 (10–37)	15 (6–24)
Bermuda	2034	1 (1–2)	1 (0–1)	24 (9–39)	15 (5–25)
Bermuda	2035	1 (0–2)	1 (0–1)	24 (8–41)	15 (4–25)
Bermuda	2036	1 (0–2)	1 (0–1)	25 (7–43)	15 (3–26)
Bhutan	2022	1 (1–1)	0 (0–0)	16 (16–16)	3 (2–3)
Bhutan	2023	1 (1–1)	0 (0–0)	16 (16–17)	3 (2–3)
Bhutan	2024	1 (1–1)	0 (0–0)	17 (16–17)	3 (2–3)
Bhutan	2025	1 (1–1)	0 (0–0)	17 (17–18)	3 (2–3)
Bhutan	2026	1 (1–1)	0 (0–0)	17 (17–18)	3 (2–3)
Bhutan	2027	1 (1–1)	0 (0–0)	18 (17–19)	2 (2–3)
Bhutan	2028	1 (1–1)	0 (0–0)	18 (17–19)	2 (2–3)
Bhutan	2029	1 (1–1)	0 (0–0)	19 (18–20)	2 (2–3)
Bhutan	2030	1 (1–1)	0 (0–0)	19 (18–20)	2 (2–3)
Bhutan	2031	1 (1–1)	0 (0–0)	19 (18–21)	2 (2–3)
Bhutan	2032	1 (1–1)	0 (0–0)	20 (18–21)	2 (2–3)
Bhutan	2033	1 (1–1)	0 (0–0)	20 (18–22)	2 (2–3)
Bhutan	2034	1 (1–1)	0 (0–0)	20 (18–22)	2 (2–3)

Bhutan	2035	1 (1–1)	0 (0–0)	21 (19–23)	2 (2–3)
Bhutan	2036	1 (1–1)	0 (0–0)	21 (19–24)	2 (2–3)
Bolivia (Plurinational State of)	2022	30 (30–30)	0 (0–0)	779 (771–787)	8 (8–8)
Bolivia (Plurinational State of)	2023	29 (28–30)	0 (0–0)	781 (764–798)	8 (8–8)
Bolivia (Plurinational State of)	2024	28 (26–30)	0 (0–0)	783 (754–812)	8 (8–8)
Bolivia (Plurinational State of)	2025	27 (24–29)	0 (0–0)	785 (742–827)	8 (7–8)
Bolivia (Plurinational State of)	2026	25 (21–29)	0 (0–0)	786 (729–844)	8 (7–8)
Bolivia (Plurinational State of)	2027	24 (18–29)	0 (0–0)	788 (714–862)	8 (7–9)
Bolivia (Plurinational State of)	2028	22 (15–29)	0 (0–0)	790 (698–882)	8 (7–9)
Bolivia (Plurinational State of)	2029	20 (11–29)	0 (0–0)	792 (681–903)	8 (7–9)
Bolivia (Plurinational State of)	2030	18 (7–29)	0 (0–0)	794 (663–925)	8 (7–9)
Bolivia (Plurinational State of)	2031	16 (3–30)	0 (0–0)	795 (643–948)	8 (7–9)
Bolivia (Plurinational State of)	2032	14 (-1–30)	0 (0–0)	797 (623–972)	8 (7–9)
Bolivia (Plurinational State of)	2033	12 (-6–31)	0 (0–0)	799 (602–997)	8 (7–9)
Bolivia (Plurinational State of)	2034	10 (-11–32)	0 (0–0)	801 (579–1023)	8 (7–9)
Bolivia (Plurinational State of)	2035	8 (-16–33)	0 (0–0)	803 (556–1050)	8 (7–9)
Bolivia (Plurinational State of)	2036	6 (-21–34)	0 (0–0)	805 (532–1078)	8 (7–9)
Bosnia and Herzegovina	2022	68 (64–72)	1 (1–1)	1628 (1518–1737)	27 (25–29)
Bosnia and Herzegovina	2023	63 (56–71)	1 (1–1)	1516 (1328–1704)	25 (22–28)
Bosnia and Herzegovina	2024	60 (49–72)	1 (1–1)	1427 (1126–1728)	23 (18–29)
Bosnia and Herzegovina	2025	56 (40–72)	1 (1–1)	1325 (903–1746)	22 (14–29)
Bosnia and Herzegovina	2026	53 (31–74)	1 (0–1)	1230 (670–1790)	20 (10–30)
Bosnia and Herzegovina	2027	49 (22–76)	1 (0–1)	1131 (423–1839)	18 (6–30)
Bosnia and Herzegovina	2028	45 (12–79)	1 (0–1)	1035 (166–1903)	17 (2–31)
Bosnia and Herzegovina	2029	42 (2–81)	1 (-0–1)	937 (-103–1976)	15 (-3–33)
Bosnia and Herzegovina	2030	38 (-9–85)	1 (-0–1)	840 (-380–2060)	13 (-8–34)
Bosnia and Herzegovina	2031	34 (-20–88)	1 (-0–1)	742 (-668–2152)	11 (-13–36)
Bosnia and Herzegovina	2032	31 (-31–92)	0 (-1–1)	645 (-964–2254)	10 (-18–37)
Bosnia and Herzegovina	2033	27 (-43–97)	0 (-1–2)	547 (-1269–2364)	8 (-23–39)
Bosnia and Herzegovina	2034	23 (-55–101)	0 (-1–2)	450 (-1583–2482)	6 (-28–41)
Bosnia and Herzegovina	2035	20 (-67–106)	0 (-1–2)	352 (-1904–2609)	5 (-34–43)
Bosnia and Herzegovina	2036	16 (-80–111)	0 (-1–2)	255 (-2233–2743)	3 (-40–46)
Botswana	2022	6 (6–6)	0 (0–0)	156 (153–159)	10 (9–10)

Botswana	2023	6 (6–6)	0 (0–0)	159 (153–165)	10 (9–10)
Botswana	2024	6 (6–6)	0 (0–0)	162 (154–171)	9 (8–11)
Botswana	2025	6 (6–6)	0 (0–0)	166 (155–177)	9 (8–11)
Botswana	2026	6 (6–6)	0 (0–0)	169 (155–182)	9 (7–12)
Botswana	2027	6 (6–7)	0 (0–0)	172 (156–187)	9 (6–12)
Botswana	2028	6 (6–7)	0 (0–1)	175 (157–192)	9 (6–13)
Botswana	2029	6 (6–7)	0 (0–1)	178 (158–197)	9 (5–14)
Botswana	2030	6 (6–7)	0 (0–1)	181 (160–202)	9 (4–14)
Botswana	2031	7 (6–7)	0 (0–1)	184 (161–206)	9 (3–15)
Botswana	2032	7 (6–7)	0 (0–1)	187 (162–211)	9 (2–16)
Botswana	2033	7 (6–8)	0 (0–1)	190 (164–215)	9 (1–17)
Botswana	2034	7 (6–8)	0 (0–1)	192 (165–220)	9 (0–18)
Botswana	2035	7 (6–8)	0 (-0–1)	195 (167–224)	9 (-1–19)
Botswana	2036	7 (6–8)	0 (-0–1)	198 (169–228)	9 (-2–20)
Brazil	2022	1229 (1207–1250)	0 (0–0)	32764 (32208–33319)	12 (12–13)
Brazil	2023	1250 (1220–1281)	0 (0–0)	33308 (32392–34223)	12 (12–13)
Brazil	2024	1272 (1235–1309)	0 (0–0)	33852 (32682–35021)	12 (11–13)
Brazil	2025	1294 (1250–1337)	0 (0–0)	34395 (33018–35773)	12 (11–13)
Brazil	2026	1315 (1267–1363)	0 (0–0)	34939 (33382–36497)	12 (11–13)
Brazil	2027	1337 (1284–1390)	0 (0–0)	35483 (33764–37202)	12 (11–13)
Brazil	2028	1358 (1301–1415)	0 (0–0)	36027 (34160–37894)	12 (10–13)
Brazil	2029	1380 (1319–1441)	0 (0–0)	36571 (34567–38574)	12 (10–13)
Brazil	2030	1401 (1337–1466)	0 (0–0)	37115 (34983–39246)	12 (10–13)
Brazil	2031	1423 (1355–1491)	0 (0–0)	37658 (35406–39911)	11 (10–13)
Brazil	2032	1445 (1373–1516)	0 (0–0)	38202 (35835–40569)	11 (10–13)
Brazil	2033	1466 (1391–1541)	0 (0–0)	38746 (36270–41222)	11 (9–13)
Brazil	2034	1488 (1410–1565)	0 (0–0)	39290 (36709–41871)	11 (9–13)
Brazil	2035	1509 (1428–1590)	0 (0–0)	39834 (37152–42515)	11 (9–13)
Brazil	2036	1531 (1447–1614)	0 (0–0)	40378 (37599–43156)	11 (9–13)
Brunei Darussalam	2022	2 (2–3)	1 (1–1)	75 (74–76)	18 (17–19)
Brunei Darussalam	2023	3 (2–3)	1 (1–1)	76 (73–78)	18 (16–19)
Brunei Darussalam	2024	3 (2–3)	1 (1–1)	76 (72–80)	17 (15–20)
Brunei Darussalam	2025	3 (2–3)	1 (0–1)	76 (70–83)	17 (13–21)

Brunei Darussalam	2026	3 (2–3)	1 (0–1)	77 (68–85)	17 (12–21)
Brunei Darussalam	2027	3 (2–3)	1 (0–1)	77 (66–88)	16 (10–22)
Brunei Darussalam	2028	3 (2–3)	1 (0–1)	78 (64–91)	16 (8–23)
Brunei Darussalam	2029	3 (2–3)	1 (0–1)	78 (62–94)	16 (7–25)
Brunei Darussalam	2030	3 (2–4)	1 (-0–1)	78 (59–98)	15 (5–26)
Brunei Darussalam	2031	3 (2–4)	1 (-0–1)	79 (57–101)	15 (3–27)
Brunei Darussalam	2032	3 (2–4)	1 (-0–1)	79 (54–105)	15 (1–29)
Brunei Darussalam	2033	3 (2–4)	1 (-0–1)	80 (51–108)	14 (-1–30)
Brunei Darussalam	2034	3 (2–4)	1 (-1–2)	80 (48–112)	14 (-4–32)
Brunei Darussalam	2035	3 (2–5)	1 (-1–2)	80 (44–117)	14 (-6–33)
Brunei Darussalam	2036	3 (1–5)	0 (-1–2)	81 (41–121)	13 (-8–35)
Bulgaria	2022	166 (158–175)	1 (1–1)	4538 (4283–4793)	37 (35–39)
Bulgaria	2023	166 (154–179)	1 (1–1)	4538 (4177–4898)	37 (34–40)
Bulgaria	2024	166 (151–182)	1 (1–1)	4538 (4096–4979)	37 (33–41)
Bulgaria	2025	166 (149–184)	1 (1–1)	4538 (4028–5048)	37 (33–41)
Bulgaria	2026	166 (147–186)	1 (1–1)	4538 (3968–5108)	37 (32–42)
Bulgaria	2027	166 (145–188)	1 (1–1)	4538 (3914–5162)	37 (32–42)
Bulgaria	2028	166 (143–190)	1 (1–1)	4538 (3864–5212)	37 (31–43)
Bulgaria	2029	166 (141–192)	1 (1–1)	4538 (3817–5259)	37 (31–43)
Bulgaria	2030	166 (140–193)	1 (1–1)	4538 (3773–5302)	37 (31–43)
Bulgaria	2031	166 (138–195)	1 (1–1)	4538 (3732–5344)	37 (30–44)
Bulgaria	2032	166 (137–196)	1 (1–1)	4538 (3693–5383)	37 (30–44)
Bulgaria	2033	166 (136–197)	1 (1–1)	4538 (3655–5421)	37 (30–44)
Bulgaria	2034	166 (134–199)	1 (1–2)	4538 (3619–5457)	37 (29–45)
Bulgaria	2035	166 (133–200)	1 (1–2)	4538 (3584–5491)	37 (29–45)
Bulgaria	2036	166 (132–201)	1 (1–2)	4538 (3551–5525)	37 (29–45)
Burkina Faso	2022	7 (6–7)	0 (0–0)	185 (183–188)	2 (2–2)
Burkina Faso	2023	7 (7–7)	0 (0–0)	189 (184–195)	2 (2–2)
Burkina Faso	2024	7 (7–7)	0 (0–0)	194 (184–203)	2 (2–2)
Burkina Faso	2025	7 (6–7)	0 (0–0)	198 (184–211)	2 (2–2)
Burkina Faso	2026	7 (6–8)	0 (0–0)	202 (183–220)	2 (2–2)
Burkina Faso	2027	7 (6–8)	0 (0–0)	206 (182–230)	2 (2–2)
Burkina Faso	2028	7 (6–8)	0 (0–0)	210 (180–240)	2 (2–2)

Burkina Faso	2029	7 (6–9)	0 (0–0)	214 (178–250)	2 (2–2)
Burkina Faso	2030	8 (6–9)	0 (0–0)	218 (175–260)	2 (2–2)
Burkina Faso	2031	8 (6–9)	0 (0–0)	222 (172–271)	2 (2–2)
Burkina Faso	2032	8 (6–10)	0 (0–0)	226 (169–283)	2 (2–2)
Burkina Faso	2033	8 (6–10)	0 (0–0)	230 (166–294)	2 (2–2)
Burkina Faso	2034	8 (6–10)	0 (0–0)	234 (162–306)	2 (2–2)
Burkina Faso	2035	8 (5–11)	0 (0–0)	238 (158–318)	2 (2–2)
Burkina Faso	2036	8 (5–11)	0 (0–0)	242 (153–331)	2 (2–2)
Burundi	2022	8 (8–8)	0 (0–0)	234 (230–237)	4 (4–4)
Burundi	2023	8 (8–9)	0 (0–0)	240 (233–248)	4 (4–4)
Burundi	2024	9 (8–9)	0 (0–0)	247 (235–260)	4 (4–5)
Burundi	2025	9 (8–9)	0 (0–0)	254 (236–272)	4 (4–5)
Burundi	2026	9 (8–10)	0 (0–0)	261 (237–285)	4 (3–5)
Burundi	2027	9 (8–10)	0 (0–0)	268 (236–299)	4 (3–5)
Burundi	2028	9 (8–11)	0 (0–0)	275 (236–314)	4 (3–5)
Burundi	2029	10 (8–11)	0 (0–0)	281 (234–328)	4 (3–5)
Burundi	2030	10 (8–12)	0 (0–0)	288 (233–344)	4 (3–6)
Burundi	2031	10 (8–12)	0 (0–0)	295 (230–360)	4 (2–6)
Burundi	2032	10 (8–13)	0 (0–0)	302 (228–376)	4 (2–6)
Burundi	2033	11 (8–13)	0 (0–0)	309 (225–393)	4 (2–6)
Burundi	2034	11 (8–14)	0 (0–0)	316 (221–410)	4 (1–7)
Burundi	2035	11 (7–14)	0 (0–0)	322 (218–427)	4 (1–7)
Burundi	2036	11 (7–15)	0 (0–0)	329 (213–445)	4 (1–7)
Cabo Verde	2022	0 (0–1)	0 (0–0)	13 (12–14)	3 (3–3)
Cabo Verde	2023	1 (0–1)	0 (0–0)	13 (12–15)	3 (3–3)
Cabo Verde	2024	1 (0–1)	0 (0–0)	14 (12–15)	3 (2–3)
Cabo Verde	2025	1 (0–1)	0 (0–0)	14 (12–16)	3 (2–3)
Cabo Verde	2026	1 (0–1)	0 (0–0)	14 (12–16)	3 (2–4)
Cabo Verde	2027	1 (0–1)	0 (0–0)	15 (12–17)	3 (2–4)
Cabo Verde	2028	1 (0–1)	0 (0–0)	15 (13–17)	3 (2–4)
Cabo Verde	2029	1 (0–1)	0 (0–0)	15 (13–18)	3 (2–4)
Cabo Verde	2030	1 (0–1)	0 (0–0)	16 (13–18)	3 (2–4)
Cabo Verde	2031	1 (0–1)	0 (0–0)	16 (13–19)	3 (2–4)

Cabo Verde	2032	1 (0–1)	0 (0–0)	16 (13–19)	3 (2–4)
Cabo Verde	2033	1 (0–1)	0 (0–0)	17 (13–20)	3 (2–4)
Cabo Verde	2034	1 (0–1)	0 (0–0)	17 (14–20)	3 (2–4)
Cabo Verde	2035	1 (0–1)	0 (0–0)	17 (14–21)	3 (2–4)
Cabo Verde	2036	1 (0–1)	0 (0–0)	17 (14–21)	3 (2–4)
Cambodia	2022	104 (104–105)	1 (1–1)	2850 (2836–2865)	21 (21–21)
Cambodia	2023	108 (107–109)	1 (1–1)	2947 (2914–2980)	21 (21–21)
Cambodia	2024	112 (110–113)	1 (1–1)	3044 (2989–3098)	21 (20–22)
Cambodia	2025	115 (112–118)	1 (1–1)	3140 (3060–3220)	21 (20–22)
Cambodia	2026	119 (115–122)	1 (1–1)	3237 (3129–3346)	21 (20–22)
Cambodia	2027	122 (117–127)	1 (1–1)	3334 (3195–3473)	21 (20–22)
Cambodia	2028	126 (120–132)	1 (1–1)	3431 (3258–3604)	21 (20–22)
Cambodia	2029	129 (122–137)	1 (1–1)	3527 (3319–3736)	21 (19–23)
Cambodia	2030	133 (124–141)	1 (1–1)	3624 (3377–3871)	21 (19–23)
Cambodia	2031	136 (127–146)	1 (1–1)	3721 (3434–4008)	21 (19–23)
Cambodia	2032	140 (129–151)	1 (1–1)	3818 (3489–4147)	21 (19–23)
Cambodia	2033	144 (131–156)	1 (1–1)	3914 (3542–4287)	21 (19–23)
Cambodia	2034	147 (133–162)	1 (1–1)	4011 (3593–4430)	21 (19–24)
Cambodia	2035	151 (135–167)	1 (1–1)	4108 (3642–4574)	21 (18–24)
Cambodia	2036	154 (136–172)	1 (1–1)	4205 (3690–4719)	21 (18–24)
Cameroon	2022	13 (13–13)	0 (0–0)	389 (386–392)	3 (3–3)
Cameroon	2023	13 (13–13)	0 (0–0)	397 (391–402)	3 (3–3)
Cameroon	2024	14 (13–14)	0 (0–0)	404 (396–412)	3 (2–3)
Cameroon	2025	14 (13–14)	0 (0–0)	411 (402–421)	3 (2–3)
Cameroon	2026	14 (14–14)	0 (0–0)	419 (407–430)	2 (2–3)
Cameroon	2027	14 (14–15)	0 (0–0)	426 (413–439)	2 (2–3)
Cameroon	2028	14 (14–15)	0 (0–0)	433 (419–448)	2 (2–3)
Cameroon	2029	15 (14–15)	0 (0–0)	441 (425–457)	2 (2–3)
Cameroon	2030	15 (14–16)	0 (0–0)	448 (431–465)	2 (2–3)
Cameroon	2031	15 (15–16)	0 (0–0)	456 (437–474)	2 (2–3)
Cameroon	2032	15 (15–16)	0 (0–0)	463 (444–482)	2 (2–3)
Cameroon	2033	16 (15–17)	0 (0–0)	470 (450–490)	2 (1–3)
Cameroon	2034	16 (15–17)	0 (0–0)	478 (456–499)	2 (1–3)

Cameroon	2035	16 (15–17)	0 (0–0)	485 (463–507)	2 (1–3)
Cameroon	2036	16 (16–17)	0 (0–0)	492 (469–515)	2 (1–3)
Canada	2022	400 (386–414)	1 (1–1)	9619 (9296–9941)	14 (14–15)
Canada	2023	400 (380–420)	1 (0–1)	9699 (9309–10089)	14 (13–15)
Canada	2024	400 (376–425)	1 (0–1)	9754 (9337–10170)	13 (12–14)
Canada	2025	400 (372–429)	0 (0–1)	9791 (9362–10219)	13 (11–14)
Canada	2026	400 (368–432)	0 (0–1)	9816 (9382–10250)	12 (11–14)
Canada	2027	400 (365–435)	0 (0–1)	9832 (9396–10269)	12 (10–13)
Canada	2028	400 (363–438)	0 (0–0)	9844 (9406–10282)	11 (9–13)
Canada	2029	400 (360–440)	0 (0–0)	9852 (9414–10290)	11 (9–12)
Canada	2030	400 (358–443)	0 (0–0)	9857 (9419–10295)	10 (8–12)
Canada	2031	400 (355–445)	0 (0–0)	9861 (9422–10299)	10 (8–12)
Canada	2032	400 (353–447)	0 (0–0)	9863 (9425–10301)	9 (7–11)
Canada	2033	400 (351–449)	0 (0–0)	9865 (9426–10303)	9 (6–11)
Canada	2034	400 (349–451)	0 (0–0)	9866 (9427–10304)	8 (6–10)
Canada	2035	400 (347–453)	0 (0–0)	9867 (9428–10305)	7 (5–10)
Canada	2036	400 (345–455)	0 (0–0)	9867 (9429–10306)	7 (4–9)
Central African Republic	2022	4 (4–4)	0 (0–0)	123 (121–125)	4 (4–4)
Central African Republic	2023	4 (4–4)	0 (0–0)	124 (120–128)	4 (4–5)
Central African Republic	2024	4 (4–4)	0 (0–0)	125 (118–132)	4 (4–5)
Central African Republic	2025	4 (4–4)	0 (0–0)	126 (116–136)	4 (4–5)
Central African Republic	2026	4 (4–4)	0 (0–0)	127 (113–141)	4 (4–5)
Central African Republic	2027	4 (3–4)	0 (0–0)	128 (110–146)	4 (4–5)
Central African Republic	2028	4 (3–5)	0 (0–0)	129 (107–151)	4 (3–5)
Central African Republic	2029	4 (3–5)	0 (0–0)	130 (103–156)	4 (3–5)
Central African Republic	2030	4 (3–5)	0 (0–0)	131 (99–162)	4 (3–5)
Central African Republic	2031	4 (3–5)	0 (0–0)	132 (95–168)	4 (3–5)
Central African Republic	2032	4 (3–5)	0 (0–0)	133 (91–175)	4 (3–5)
Central African Republic	2033	4 (3–5)	0 (0–0)	134 (86–181)	4 (3–5)
Central African Republic	2034	4 (3–6)	0 (0–0)	135 (82–188)	4 (2–5)
Central African Republic	2035	4 (2–6)	0 (0–0)	136 (77–195)	4 (2–5)
Central African Republic	2036	4 (2–6)	0 (0–0)	137 (71–203)	4 (2–5)
Chad	2022	6 (6–6)	0 (0–0)	160 (158–162)	3 (3–3)

Chad	2023	6 (6–6)	0 (0–0)	164 (160–168)	3 (2–3)
Chad	2024	6 (6–6)	0 (0–0)	168 (161–174)	3 (2–3)
Chad	2025	6 (6–7)	0 (0–0)	172 (162–181)	3 (2–3)
Chad	2026	6 (6–7)	0 (0–0)	175 (163–188)	3 (2–3)
Chad	2027	6 (6–7)	0 (0–0)	179 (163–195)	3 (2–3)
Chad	2028	7 (6–7)	0 (0–0)	183 (163–203)	3 (2–3)
Chad	2029	7 (6–8)	0 (0–0)	187 (163–211)	3 (2–3)
Chad	2030	7 (6–8)	0 (0–0)	191 (162–219)	3 (2–3)
Chad	2031	7 (6–8)	0 (0–0)	194 (162–227)	3 (2–3)
Chad	2032	7 (6–8)	0 (0–0)	198 (161–236)	3 (2–4)
Chad	2033	7 (6–9)	0 (0–0)	202 (160–245)	3 (1–4)
Chad	2034	7 (6–9)	0 (0–0)	206 (158–254)	3 (1–4)
Chad	2035	7 (5–9)	0 (0–0)	210 (157–263)	3 (1–4)
Chad	2036	7 (5–10)	0 (0–0)	214 (155–272)	3 (1–4)
Chile	2022	123 (119–127)	0 (0–1)	3435 (3307–3564)	14 (13–14)
Chile	2023	125 (119–131)	0 (0–1)	3494 (3313–3675)	14 (13–15)
Chile	2024	127 (120–135)	0 (0–1)	3553 (3331–3775)	14 (13–15)
Chile	2025	130 (121–138)	1 (0–1)	3611 (3355–3868)	14 (13–15)
Chile	2026	132 (123–141)	1 (0–1)	3670 (3384–3956)	14 (13–15)
Chile	2027	134 (124–144)	1 (0–1)	3729 (3415–4042)	14 (13–15)
Chile	2028	136 (125–147)	1 (0–1)	3787 (3448–4126)	14 (13–15)
Chile	2029	138 (127–150)	1 (0–1)	3846 (3484–4208)	14 (13–15)
Chile	2030	141 (128–153)	1 (0–1)	3905 (3520–4289)	14 (13–15)
Chile	2031	143 (130–156)	1 (0–1)	3963 (3558–4368)	14 (13–15)
Chile	2032	145 (131–159)	1 (0–1)	4022 (3597–4447)	14 (13–15)
Chile	2033	147 (133–162)	1 (0–1)	4080 (3637–4524)	14 (13–15)
Chile	2034	149 (134–164)	1 (0–1)	4139 (3677–4601)	14 (13–15)
Chile	2035	152 (136–167)	1 (0–1)	4198 (3718–4677)	14 (13–15)
Chile	2036	154 (138–170)	1 (0–1)	4256 (3760–4753)	14 (13–15)
China	2022	[illegible]771 (17541–1800[illegible]	1 (1–1)	[illegible]70202 (464699–47570[illegible]	21 (21–22)
China	2023	[illegible]265 (17751–1877[illegible]	1 (1–1)	[illegible]81154 (468850–49345[illegible]	21 (20–22)
China	2024	[illegible]758 (17899–1961[illegible]	1 (1–1)	[illegible]92106 (471517–51269[illegible]	21 (20–23)
China	2025	[illegible]252 (17994–2051[illegible]	1 (1–1)	[illegible]03058 (472920–53319[illegible]	21 (19–24)

China	2026	746 (18042–2145	1 (1–1)	14011 (473203–55481	21 (18–25)
China	2027	240 (18048–2243	1 (1–1)	24963 (472472–57745	21 (17–26)
China	2028	734 (18015–2345	1 (1–1)	35915 (470808–60102	21 (16–27)
China	2029	228 (17946–2451	1 (1–1)	46867 (468275–62545	21 (15–28)
China	2030	722 (17842–2560	1 (1–1)	57819 (464926–65071	21 (14–29)
China	2031	215 (17706–2672	1 (1–1)	68771 (460804–67673	21 (13–30)
China	2032	709 (17540–2787	1 (1–1)	79724 (455947–70350	21 (12–31)
China	2033	203 (17344–2906	1 (1–1)	90676 (450388–73096	21 (10–32)
China	2034	697 (17121–3027	1 (1–1)	01628 (444155–75910	21 (9–34)
China	2035	191 (16870–3151	1 (1–1)	12580 (437274–78788	21 (8–35)
China	2036	685 (16593–3277	1 (1–1)	23532 (429768–81729	21 (6–37)
Colombia	2022	133 (127–139)	0 (0–0)	3554 (3388–3721)	6 (6–7)
Colombia	2023	136 (127–144)	0 (0–0)	3617 (3382–3852)	6 (6–7)
Colombia	2024	138 (128–148)	0 (0–0)	3680 (3392–3968)	6 (6–7)
Colombia	2025	141 (129–152)	0 (0–0)	3743 (3410–4076)	6 (5–7)
Colombia	2026	143 (130–156)	0 (0–0)	3806 (3434–4178)	6 (5–7)
Colombia	2027	146 (131–160)	0 (0–0)	3869 (3461–4276)	6 (5–7)
Colombia	2028	148 (133–163)	0 (0–0)	3931 (3491–4371)	6 (5–7)
Colombia	2029	150 (134–167)	0 (0–0)	3994 (3524–4465)	6 (5–8)
Colombia	2030	153 (136–170)	0 (0–0)	4057 (3558–4556)	6 (5–8)
Colombia	2031	155 (137–174)	0 (0–0)	4120 (3594–4646)	6 (5–8)
Colombia	2032	158 (139–177)	0 (0–0)	4183 (3631–4734)	6 (5–8)
Colombia	2033	160 (140–180)	0 (0–0)	4246 (3670–4822)	6 (5–8)
Colombia	2034	163 (142–184)	0 (0–0)	4309 (3709–4908)	6 (5–8)
Colombia	2035	165 (144–187)	0 (0–0)	4371 (3749–4994)	6 (5–8)
Colombia	2036	168 (146–190)	0 (0–0)	4434 (3790–5078)	6 (5–8)
Comoros	2022	1 (1–1)	0 (0–0)	34 (33–35)	7 (6–7)
Comoros	2023	1 (1–1)	0 (0–0)	35 (33–37)	7 (6–7)
Comoros	2024	1 (1–1)	0 (0–0)	36 (34–38)	7 (6–7)
Comoros	2025	1 (1–2)	0 (0–0)	37 (34–40)	7 (6–7)
Comoros	2026	2 (1–2)	0 (0–0)	38 (35–42)	7 (6–7)
Comoros	2027	2 (1–2)	0 (0–0)	39 (35–44)	7 (6–7)
Comoros	2028	2 (1–2)	0 (0–0)	40 (35–46)	7 (6–7)

Comoros	2029	2 (1–2)	0 (0–0)	42 (36–47)	7 (6–7)
Comoros	2030	2 (1–2)	0 (0–0)	43 (36–49)	7 (6–7)
Comoros	2031	2 (1–2)	0 (0–0)	44 (36–51)	7 (6–7)
Comoros	2032	2 (1–2)	0 (0–0)	45 (36–53)	7 (6–7)
Comoros	2033	2 (2–2)	0 (0–0)	46 (37–55)	7 (6–7)
Comoros	2034	2 (2–2)	0 (0–0)	47 (37–57)	7 (6–8)
Comoros	2035	2 (2–2)	0 (0–0)	48 (37–59)	7 (6–8)
Comoros	2036	2 (2–2)	0 (0–0)	49 (37–61)	7 (6–8)
Congo	2022	6 (6–6)	0 (0–0)	186 (183–189)	6 (6–6)
Congo	2023	6 (6–7)	0 (0–0)	192 (186–198)	6 (6–6)
Congo	2024	7 (6–7)	0 (0–0)	198 (187–208)	6 (5–6)
Congo	2025	7 (6–7)	0 (0–0)	204 (189–219)	6 (5–6)
Congo	2026	7 (6–8)	0 (0–0)	210 (189–231)	6 (5–6)
Congo	2027	7 (6–8)	0 (0–0)	216 (189–242)	6 (5–6)
Congo	2028	7 (6–8)	0 (0–0)	222 (189–255)	6 (5–6)
Congo	2029	8 (6–9)	0 (0–0)	228 (188–268)	6 (5–6)
Congo	2030	8 (6–9)	0 (0–0)	234 (186–281)	6 (5–6)
Congo	2031	8 (6–9)	0 (0–0)	239 (184–294)	6 (5–6)
Congo	2032	8 (6–10)	0 (0–0)	245 (182–308)	6 (5–6)
Congo	2033	8 (6–10)	0 (0–0)	251 (180–323)	6 (5–6)
Congo	2034	8 (6–11)	0 (0–0)	257 (177–337)	6 (5–6)
Congo	2035	9 (6–11)	0 (0–0)	263 (174–352)	6 (5–6)
Congo	2036	9 (6–12)	0 (0–0)	269 (170–368)	6 (5–6)
Cook Islands	2022	0 (0–0)	0 (0–0)	1 (1–1)	5 (5–5)
Cook Islands	2023	0 (0–0)	0 (0–0)	1 (1–1)	5 (5–6)
Cook Islands	2024	0 (0–0)	0 (0–0)	1 (1–1)	5 (5–6)
Cook Islands	2025	0 (0–0)	0 (0–0)	1 (1–2)	5 (4–6)
Cook Islands	2026	0 (0–0)	0 (0–0)	1 (1–2)	5 (4–6)
Cook Islands	2027	0 (0–0)	0 (0–0)	1 (1–2)	5 (4–6)
Cook Islands	2028	0 (0–0)	0 (0–0)	1 (1–2)	5 (4–6)
Cook Islands	2029	0 (0–0)	0 (0–0)	1 (1–2)	5 (4–6)
Cook Islands	2030	0 (0–0)	0 (0–0)	1 (1–2)	5 (4–6)
Cook Islands	2031	0 (0–0)	0 (0–0)	1 (1–2)	4 (3–6)

Cook Islands	2032	0 (0–0)	0 (0–0)	1 (1–2)	4 (3–6)
Cook Islands	2033	0 (0–0)	0 (0–0)	1 (1–2)	4 (3–6)
Cook Islands	2034	0 (0–0)	0 (0–0)	1 (1–2)	4 (3–6)
Cook Islands	2035	0 (0–0)	0 (0–0)	1 (1–2)	4 (3–6)
Cook Islands	2036	0 (0–0)	0 (0–0)	1 (1–2)	4 (3–5)
Costa Rica	2022	28 (26–30)	1 (0–1)	717 (660–774)	13 (11–14)
Costa Rica	2023	29 (26–31)	1 (0–1)	734 (670–799)	13 (11–14)
Costa Rica	2024	29 (27–32)	1 (0–1)	752 (680–823)	13 (11–15)
Costa Rica	2025	30 (27–33)	1 (0–1)	769 (691–847)	13 (11–15)
Costa Rica	2026	31 (28–34)	1 (0–1)	786 (703–870)	13 (11–15)
Costa Rica	2027	32 (28–35)	1 (0–1)	804 (715–893)	13 (10–15)
Costa Rica	2028	32 (29–36)	1 (0–1)	821 (727–915)	13 (10–15)
Costa Rica	2029	33 (29–37)	1 (0–1)	838 (740–937)	13 (10–15)
Costa Rica	2030	34 (30–38)	1 (0–1)	856 (752–959)	13 (10–15)
Costa Rica	2031	34 (30–38)	1 (0–1)	873 (765–981)	13 (10–15)
Costa Rica	2032	35 (31–39)	1 (0–1)	890 (778–1003)	13 (10–15)
Costa Rica	2033	36 (31–40)	1 (0–1)	908 (792–1024)	13 (10–16)
Costa Rica	2034	36 (32–41)	1 (0–1)	925 (805–1045)	13 (10–16)
Costa Rica	2035	37 (32–42)	1 (0–1)	943 (818–1067)	13 (10–16)
Costa Rica	2036	38 (33–43)	1 (0–1)	960 (832–1088)	13 (10–16)
Croatia	2022	7 (7–7)	0 (0–0)	217 (213–221)	2 (2–2)
Croatia	2023	7 (7–7)	0 (0–0)	221 (213–229)	2 (1–2)
Croatia	2024	7 (7–8)	0 (0–0)	225 (212–239)	2 (1–2)
Croatia	2025	7 (7–8)	0 (0–0)	230 (209–250)	1 (1–2)
Croatia	2026	8 (7–8)	0 (0–0)	234 (206–262)	1 (1–2)
Croatia	2027	8 (7–9)	0 (0–0)	238 (203–274)	1 (1–2)
Croatia	2028	8 (7–9)	0 (0–0)	243 (198–287)	1 (1–2)
Croatia	2029	8 (6–10)	0 (0–0)	247 (194–300)	1 (1–2)
Croatia	2030	8 (6–10)	0 (0–0)	251 (188–314)	1 (0–2)
Croatia	2031	8 (6–10)	0 (0–0)	255 (182–328)	1 (0–2)
Croatia	2032	8 (6–11)	0 (0–0)	260 (176–343)	1 (0–2)
Croatia	2033	9 (6–11)	0 (0–0)	264 (169–359)	1 (-0–3)
Croatia	2034	9 (6–12)	0 (0–0)	268 (162–375)	1 (-0–3)

Croatia	2035	9 (5–12)	0 (0–0)	272 (154–391)	1 (-1–3)
Croatia	2036	9 (5–13)	0 (-0–0)	277 (146–408)	1 (-1–3)
Cuba	2022	127 (119–136)	1 (1–2)	2813 (2598–3027)	35 (32–38)
Cuba	2023	129 (116–141)	1 (1–2)	2813 (2510–3115)	36 (32–39)
Cuba	2024	130 (115–145)	1 (1–2)	2813 (2442–3183)	36 (32–40)
Cuba	2025	131 (114–148)	1 (1–2)	2813 (2384–3241)	36 (33–40)
Cuba	2026	132 (113–152)	1 (1–2)	2813 (2334–3291)	37 (33–40)
Cuba	2027	134 (113–155)	1 (1–2)	2813 (2288–3337)	37 (33–41)
Cuba	2028	135 (112–158)	1 (1–2)	2813 (2246–3379)	37 (33–41)
Cuba	2029	136 (112–161)	1 (1–2)	2813 (2207–3418)	37 (33–41)
Cuba	2030	138 (112–163)	1 (1–2)	2813 (2170–3455)	37 (33–41)
Cuba	2031	139 (111–166)	1 (1–2)	2813 (2135–3490)	37 (33–41)
Cuba	2032	140 (111–169)	1 (1–2)	2813 (2102–3523)	37 (33–41)
Cuba	2033	141 (111–171)	1 (1–2)	2813 (2071–3554)	37 (33–41)
Cuba	2034	143 (111–174)	1 (1–2)	2813 (2040–3585)	37 (33–41)
Cuba	2035	144 (112–176)	1 (1–2)	2813 (2011–3614)	37 (33–41)
Cuba	2036	145 (112–179)	1 (1–2)	2813 (1983–3642)	37 (33–41)
Cyprus	2022	141 (136–145)	1 (1–1)	3544 (3424–3665)	18 (18–19)
Cyprus	2023	143 (136–149)	1 (1–1)	3588 (3418–3758)	18 (17–19)
Cyprus	2024	144 (137–152)	1 (1–1)	3631 (3423–3839)	18 (17–20)
Cyprus	2025	146 (137–155)	1 (1–1)	3674 (3434–3915)	18 (17–20)
Cyprus	2026	148 (138–158)	1 (1–1)	3718 (3449–3987)	18 (17–20)
Cyprus	2027	150 (139–161)	1 (1–1)	3761 (3466–4056)	18 (16–20)
Cyprus	2028	152 (140–164)	1 (1–1)	3804 (3486–4123)	18 (16–20)
Cyprus	2029	154 (141–166)	1 (1–1)	3848 (3507–4188)	18 (16–20)
Cyprus	2030	155 (142–169)	1 (1–1)	3891 (3530–4252)	18 (16–21)
Cyprus	2031	157 (143–172)	1 (1–1)	3934 (3554–4315)	18 (16–21)
Cyprus	2032	159 (144–174)	1 (1–1)	3977 (3578–4377)	18 (16–21)
Cyprus	2033	161 (145–177)	1 (1–1)	4021 (3604–4438)	18 (16–21)
Cyprus	2034	163 (146–179)	1 (1–1)	4064 (3630–4498)	18 (15–21)
Cyprus	2035	165 (148–182)	1 (1–1)	4107 (3657–4558)	18 (15–21)
Cyprus	2036	166 (149–184)	1 (1–1)	4151 (3685–4617)	18 (15–21)
Czechia	2022	12 (11–12)	1 (1–1)	280 (272–288)	13 (13–14)

Czechia	2023	12 (11–13)	1 (1–1)	287 (272–301)	13 (12–13)
Czechia	2024	12 (11–13)	1 (0–1)	291 (270–311)	12 (11–13)
Czechia	2025	12 (12–13)	1 (0–1)	294 (270–319)	11 (10–13)
Czechia	2026	12 (12–13)	1 (0–1)	298 (271–325)	11 (9–13)
Czechia	2027	13 (12–14)	0 (0–1)	302 (272–332)	10 (8–13)
Czechia	2028	13 (12–14)	0 (0–1)	305 (273–338)	10 (7–13)
Czechia	2029	13 (12–14)	0 (0–1)	309 (274–344)	9 (6–13)
Czechia	2030	13 (12–14)	0 (0–1)	313 (276–350)	8 (4–12)
Czechia	2031	13 (12–15)	0 (0–1)	316 (277–355)	8 (3–12)
Czechia	2032	13 (12–15)	0 (0–1)	320 (279–361)	7 (2–12)
Czechia	2033	14 (12–15)	0 (0–1)	324 (281–367)	7 (1–12)
Czechia	2034	14 (12–15)	0 (0–1)	328 (283–372)	6 (-0–12)
Czechia	2035	14 (12–15)	0 (0–0)	331 (285–378)	6 (-1–12)
Czechia	2036	14 (13–16)	0 (0–0)	335 (287–383)	5 (-3–13)
C么te d'Ivoire	2022	217 (208–225)	1 (1–1)	5175 (4944–5406)	26 (24–27)
C么te d'Ivoire	2023	214 (202–226)	1 (1–1)	5100 (4774–5427)	25 (23–27)
C么te d'Ivoire	2024	212 (197–227)	1 (1–1)	5025 (4625–5425)	24 (21–27)
C么te d'Ivoire	2025	209 (192–227)	1 (1–1)	4951 (4489–5413)	23 (20–26)
C么te d'Ivoire	2026	207 (188–226)	1 (1–1)	4876 (4359–5392)	22 (18–26)
C么te d'Ivoire	2027	205 (183–226)	1 (1–1)	4801 (4235–5367)	21 (17–25)
C么te d'Ivoire	2028	202 (179–225)	1 (1–1)	4726 (4115–5337)	20 (16–24)
C么te d'Ivoire	2029	200 (175–224)	1 (1–1)	4651 (3998–5305)	19 (15–24)
C么te d'Ivoire	2030	198 (172–224)	1 (1–1)	4577 (3884–5269)	18 (14–23)
C么te d'Ivoire	2031	195 (168–223)	1 (0–1)	4502 (3772–5232)	17 (12–22)
C么te d'Ivoire	2032	193 (164–222)	1 (0–1)	4427 (3661–5193)	16 (11–22)
C么te d'Ivoire	2033	190 (160–220)	1 (0–1)	4352 (3552–5152)	16 (10–21)
C么te d'Ivoire	2034	188 (157–219)	1 (0–1)	4277 (3445–5110)	15 (9–20)
C么te d'Ivoire	2035	186 (153–218)	1 (0–1)	4203 (3338–5067)	14 (8–20)
C么te d'Ivoire	2036	183 (150–217)	1 (0–1)	4128 (3233–5022)	13 (7–19)
Democratic People's Republic of Korea	2022	160 (159–161)	0 (0–0)	4970 (4953–4986)	14 (14–14)
Democratic People's Republic of Korea	2023	162 (161–164)	0 (0–0)	5028 (4987–5069)	14 (14–14)
Democratic People's Republic of Korea	2024	164 (161–167)	0 (0–0)	5085 (5013–5158)	14 (14–14)
Democratic People's Republic of Korea	2025	166 (162–170)	0 (0–0)	5143 (5033–5254)	14 (13–14)

Democratic People's Republic of Korea	2026	168 (163–174)	0 (0–0)	5201 (5048–5354)	14 (13–14)
Democratic People's Republic of Korea	2027	170 (163–178)	0 (0–0)	5259 (5058–5459)	14 (13–14)
Democratic People's Republic of Korea	2028	172 (163–181)	0 (0–0)	5316 (5065–5568)	13 (13–14)
Democratic People's Republic of Korea	2029	174 (163–186)	0 (0–0)	5374 (5068–5680)	13 (12–14)
Democratic People's Republic of Korea	2030	176 (163–190)	0 (0–0)	5432 (5067–5796)	13 (12–14)
Democratic People's Republic of Korea	2031	178 (163–194)	0 (0–0)	5489 (5063–5916)	13 (12–14)
Democratic People's Republic of Korea	2032	180 (163–198)	0 (0–0)	5547 (5056–6038)	13 (12–15)
Democratic People's Republic of Korea	2033	182 (162–203)	0 (0–0)	5605 (5046–6164)	13 (11–15)
Democratic People's Republic of Korea	2034	185 (162–208)	0 (0–0)	5663 (5033–6292)	13 (11–15)
Democratic People's Republic of Korea	2035	187 (161–212)	0 (0–0)	5720 (5017–6423)	13 (11–15)
Democratic People's Republic of Korea	2036	189 (160–217)	0 (0–0)	5778 (4999–6557)	13 (11–15)
Democratic Republic of the Congo	2022	37 (37–38)	0 (0–0)	1130 (1114–1145)	3 (3–3)
Democratic Republic of the Congo	2023	39 (38–40)	0 (0–0)	1168 (1137–1198)	3 (2–3)
Democratic Republic of the Congo	2024	40 (38–41)	0 (0–0)	1206 (1158–1253)	3 (2–3)
Democratic Republic of the Congo	2025	41 (39–43)	0 (0–0)	1244 (1177–1310)	3 (2–3)
Democratic Republic of the Congo	2026	42 (39–45)	0 (0–0)	1282 (1194–1370)	3 (2–3)
Democratic Republic of the Congo	2027	43 (40–47)	0 (0–0)	1320 (1209–1431)	3 (2–3)
Democratic Republic of the Congo	2028	45 (40–49)	0 (0–0)	1358 (1223–1493)	3 (2–3)
Democratic Republic of the Congo	2029	46 (40–52)	0 (0–0)	1396 (1235–1557)	3 (2–3)
Democratic Republic of the Congo	2030	47 (40–54)	0 (0–0)	1434 (1245–1623)	3 (2–3)
Democratic Republic of the Congo	2031	48 (41–56)	0 (0–0)	1472 (1254–1690)	3 (2–3)
Democratic Republic of the Congo	2032	50 (41–58)	0 (0–0)	1510 (1262–1759)	3 (2–4)
Democratic Republic of the Congo	2033	51 (41–61)	0 (0–0)	1548 (1268–1828)	3 (2–4)
Democratic Republic of the Congo	2034	52 (41–63)	0 (0–0)	1586 (1273–1899)	3 (2–4)
Democratic Republic of the Congo	2035	53 (41–66)	0 (0–0)	1624 (1277–1972)	3 (2–4)
Democratic Republic of the Congo	2036	54 (41–68)	0 (0–0)	1662 (1280–2045)	3 (1–4)
Denmark	2022	132 (123–142)	1 (1–1)	2713 (2518–2908)	23 (21–26)
Denmark	2023	132 (115–150)	1 (1–1)	2709 (2358–3061)	22 (18–27)
Denmark	2024	133 (109–157)	1 (1–1)	2708 (2219–3197)	22 (16–28)
Denmark	2025	133 (103–163)	1 (1–1)	2707 (2097–3317)	21 (13–28)
Denmark	2026	133 (97–168)	1 (1–2)	2706 (1990–3423)	20 (11–29)
Denmark	2027	133 (92–173)	1 (0–2)	2706 (1894–3518)	19 (9–29)
Denmark	2028	133 (88–178)	1 (0–2)	2706 (1807–3605)	18 (8–29)

Denmark	2029	133 (84–182)	1 (0–2)	2706 (1727–3685)	17 (6–29)
Denmark	2030	133 (80–185)	1 (0–2)	2706 (1653–3758)	17 (4–29)
Denmark	2031	133 (76–189)	1 (0–2)	2706 (1584–3828)	16 (2–29)
Denmark	2032	133 (73–192)	1 (0–2)	2706 (1519–3893)	15 (1–29)
Denmark	2033	133 (70–196)	1 (0–2)	2706 (1457–3955)	14 (-1–29)
Denmark	2034	133 (67–199)	1 (0–2)	2706 (1398–4014)	13 (-2–29)
Denmark	2035	133 (64–201)	1 (0–2)	2706 (1342–4070)	12 (-4–29)
Denmark	2036	133 (61–204)	1 (0–2)	2706 (1288–4124)	12 (-5–29)
Djibouti	2022	3 (3–3)	1 (1–1)	95 (94–96)	13 (13–14)
Djibouti	2023	3 (3–4)	1 (1–1)	98 (96–100)	13 (13–14)
Djibouti	2024	4 (3–4)	1 (1–1)	102 (98–105)	14 (13–14)
Djibouti	2025	4 (4–4)	1 (1–1)	105 (100–109)	14 (13–15)
Djibouti	2026	4 (4–4)	1 (1–1)	108 (102–114)	14 (13–15)
Djibouti	2027	4 (4–4)	1 (1–1)	111 (104–119)	14 (13–15)
Djibouti	2028	4 (4–4)	1 (1–1)	115 (105–124)	14 (13–15)
Djibouti	2029	4 (4–4)	1 (0–1)	118 (107–129)	14 (13–16)
Djibouti	2030	4 (4–5)	1 (0–1)	121 (108–134)	14 (13–16)
Djibouti	2031	4 (4–5)	1 (0–1)	125 (109–140)	14 (13–16)
Djibouti	2032	5 (4–5)	1 (0–1)	128 (111–145)	14 (13–16)
Djibouti	2033	5 (4–5)	1 (0–1)	131 (112–151)	14 (13–16)
Djibouti	2034	5 (4–5)	1 (0–1)	134 (113–156)	15 (13–17)
Djibouti	2035	5 (4–6)	1 (0–1)	138 (113–162)	15 (13–17)
Djibouti	2036	5 (4–6)	1 (0–1)	141 (114–168)	15 (13–17)
Dominica	2022	0 (0–0)	0 (0–0)	8 (8–8)	10 (9–10)
Dominica	2023	0 (0–0)	0 (0–0)	8 (8–9)	10 (9–10)
Dominica	2024	0 (0–0)	0 (0–0)	9 (8–9)	10 (9–10)
Dominica	2025	0 (0–0)	0 (0–0)	9 (8–9)	10 (9–10)
Dominica	2026	0 (0–0)	0 (0–0)	9 (8–9)	10 (9–10)
Dominica	2027	0 (0–0)	0 (0–0)	9 (8–10)	10 (9–10)
Dominica	2028	0 (0–0)	0 (0–0)	9 (8–10)	10 (9–10)
Dominica	2029	0 (0–0)	0 (0–0)	9 (8–10)	10 (9–10)
Dominica	2030	0 (0–0)	0 (0–0)	9 (8–10)	10 (8–11)
Dominica	2031	0 (0–0)	0 (0–0)	9 (8–11)	9 (8–11)

Dominica	2032	0 (0–0)	0 (0–0)	9 (8–11)	9 (8–11)
Dominica	2033	0 (0–0)	0 (0–0)	9 (7–11)	9 (8–11)
Dominica	2034	0 (0–0)	0 (0–0)	10 (7–12)	9 (8–11)
Dominica	2035	0 (0–0)	0 (0–0)	10 (7–12)	9 (8–11)
Dominica	2036	0 (0–0)	0 (0–0)	10 (7–13)	9 (8–11)
Dominican Republic	2022	41 (39–43)	0 (0–0)	980 (941–1019)	10 (9–10)
Dominican Republic	2023	42 (39–45)	0 (0–0)	1001 (946–1056)	10 (9–10)
Dominican Republic	2024	43 (40–46)	0 (0–0)	1022 (954–1089)	10 (9–10)
Dominican Republic	2025	44 (40–47)	0 (0–0)	1042 (964–1120)	10 (8–11)
Dominican Republic	2026	45 (41–49)	0 (0–0)	1063 (976–1150)	10 (8–11)
Dominican Republic	2027	46 (41–50)	0 (0–0)	1083 (988–1179)	10 (8–11)
Dominican Republic	2028	46 (42–51)	0 (0–0)	1104 (1001–1207)	10 (8–11)
Dominican Republic	2029	47 (42–53)	0 (0–1)	1125 (1014–1235)	10 (8–11)
Dominican Republic	2030	48 (43–54)	0 (0–1)	1145 (1028–1262)	10 (8–11)
Dominican Republic	2031	49 (43–55)	0 (0–1)	1166 (1043–1289)	10 (8–11)
Dominican Republic	2032	50 (44–56)	0 (0–1)	1187 (1057–1316)	10 (8–11)
Dominican Republic	2033	51 (44–57)	0 (0–1)	1207 (1072–1342)	10 (8–11)
Dominican Republic	2034	52 (45–58)	0 (0–1)	1228 (1087–1369)	10 (8–11)
Dominican Republic	2035	53 (46–60)	0 (0–1)	1249 (1103–1394)	10 (8–12)
Dominican Republic	2036	53 (46–61)	0 (0–1)	1269 (1118–1420)	10 (7–12)
Ecuador	2022	40 (38–42)	0 (0–0)	973 (935–1010)	6 (5–6)
Ecuador	2023	41 (38–44)	0 (0–0)	990 (925–1055)	6 (5–6)
Ecuador	2024	42 (38–45)	0 (0–0)	1009 (921–1098)	6 (5–7)
Ecuador	2025	43 (38–47)	0 (0–0)	1029 (921–1138)	6 (5–7)
Ecuador	2026	43 (39–48)	0 (0–0)	1050 (924–1176)	6 (5–7)
Ecuador	2027	44 (39–50)	0 (0–0)	1070 (929–1211)	6 (5–7)
Ecuador	2028	45 (39–51)	0 (0–0)	1091 (935–1246)	6 (5–7)
Ecuador	2029	46 (40–52)	0 (0–0)	1111 (943–1279)	6 (5–7)
Ecuador	2030	47 (40–53)	0 (0–0)	1132 (951–1312)	6 (5–7)
Ecuador	2031	48 (41–55)	0 (0–0)	1152 (961–1344)	6 (4–7)
Ecuador	2032	49 (41–56)	0 (0–0)	1173 (970–1375)	6 (4–7)
Ecuador	2033	49 (42–57)	0 (0–0)	1193 (981–1405)	6 (4–7)
Ecuador	2034	50 (42–58)	0 (0–0)	1214 (992–1436)	6 (4–7)

Ecuador	2035	51 (43–59)	0 (0–0)	1234 (1003–1465)	6 (4–7)
Ecuador	2036	52 (43–61)	0 (0–0)	1255 (1015–1495)	6 (4–7)
Egypt	2022	244 (238–251)	0 (0–0)	7389 (7199–7578)	10 (10–10)
Egypt	2023	250 (239–261)	0 (0–0)	7551 (7206–7896)	10 (9–11)
Egypt	2024	256 (241–271)	0 (0–0)	7713 (7264–8163)	10 (9–11)
Egypt	2025	262 (242–282)	0 (0–0)	7875 (7341–8410)	10 (9–11)
Egypt	2026	267 (242–293)	0 (0–0)	8038 (7431–8645)	10 (8–11)
Egypt	2027	273 (243–304)	0 (0–0)	8200 (7528–8872)	10 (8–12)
Egypt	2028	279 (243–316)	0 (0–0)	8362 (7631–9094)	10 (8–12)
Egypt	2029	285 (242–327)	0 (0–0)	8525 (7739–9311)	10 (8–12)
Egypt	2030	291 (242–340)	0 (0–0)	8687 (7850–9524)	10 (8–12)
Egypt	2031	296 (241–352)	0 (0–0)	8849 (7964–9735)	10 (8–12)
Egypt	2032	302 (240–365)	0 (0–1)	9012 (8080–9943)	10 (7–12)
Egypt	2033	308 (238–378)	0 (0–1)	9174 (8199–10149)	10 (7–13)
Egypt	2034	314 (237–391)	0 (0–1)	9336 (8320–10353)	10 (7–13)
Egypt	2035	320 (235–404)	0 (0–1)	9498 (8442–10555)	10 (7–13)
Egypt	2036	326 (233–418)	0 (0–1)	9661 (8566–10756)	10 (7–13)
El Salvador	2022	11 (10–11)	0 (0–0)	314 (295–333)	5 (5–6)
El Salvador	2023	11 (10–12)	0 (0–0)	321 (294–348)	5 (5–6)
El Salvador	2024	11 (10–12)	0 (0–0)	328 (295–361)	5 (5–6)
El Salvador	2025	12 (10–13)	0 (0–0)	335 (297–373)	5 (5–6)
El Salvador	2026	12 (10–13)	0 (0–0)	342 (300–385)	5 (5–6)
El Salvador	2027	12 (11–14)	0 (0–0)	349 (303–396)	6 (5–7)
El Salvador	2028	12 (11–14)	0 (0–0)	356 (306–407)	6 (5–7)
El Salvador	2029	13 (11–14)	0 (0–0)	363 (310–417)	6 (5–7)
El Salvador	2030	13 (11–15)	0 (0–0)	370 (313–428)	6 (5–7)
El Salvador	2031	13 (11–15)	0 (0–0)	378 (317–438)	6 (5–7)
El Salvador	2032	13 (11–15)	0 (0–0)	385 (321–448)	6 (5–7)
El Salvador	2033	13 (11–16)	0 (0–0)	392 (326–457)	6 (5–7)
El Salvador	2034	14 (12–16)	0 (0–0)	399 (330–467)	6 (5–7)
El Salvador	2035	14 (12–16)	0 (0–0)	406 (334–477)	6 (5–8)
El Salvador	2036	14 (12–17)	0 (0–0)	413 (339–486)	6 (5–8)
Equatorial Guinea	2022	1 (1–1)	0 (0–0)	37 (36–37)	6 (6–6)

Equatorial Guinea	2023	1 (1–1)	0 (0–0)	38 (36–39)	6 (6–6)
Equatorial Guinea	2024	1 (1–1)	0 (0–0)	39 (36–41)	6 (5–7)
Equatorial Guinea	2025	1 (1–1)	0 (0–0)	40 (36–44)	6 (5–7)
Equatorial Guinea	2026	1 (1–1)	0 (0–0)	41 (35–46)	6 (5–7)
Equatorial Guinea	2027	1 (1–2)	0 (0–0)	42 (35–49)	6 (4–7)
Equatorial Guinea	2028	1 (1–2)	0 (0–0)	43 (34–51)	6 (4–8)
Equatorial Guinea	2029	1 (1–2)	0 (0–0)	44 (34–54)	6 (3–8)
Equatorial Guinea	2030	1 (1–2)	0 (0–0)	45 (33–57)	6 (3–8)
Equatorial Guinea	2031	1 (1–2)	0 (0–0)	46 (32–60)	6 (2–9)
Equatorial Guinea	2032	1 (1–2)	0 (0–0)	47 (31–63)	5 (2–9)
Equatorial Guinea	2033	1 (1–2)	0 (0–0)	48 (30–66)	5 (1–10)
Equatorial Guinea	2034	1 (1–2)	0 (0–0)	49 (28–69)	5 (1–10)
Equatorial Guinea	2035	2 (1–2)	0 (0–0)	50 (27–73)	5 (0–11)
Equatorial Guinea	2036	2 (1–2)	0 (-0–0)	51 (26–76)	5 (-1–11)
Eritrea	2022	4 (4–4)	0 (0–0)	143 (139–146)	4 (4–4)
Eritrea	2023	4 (4–5)	0 (0–0)	148 (140–156)	4 (4–4)
Eritrea	2024	4 (4–5)	0 (0–0)	154 (140–167)	4 (3–4)
Eritrea	2025	5 (4–5)	0 (0–0)	159 (139–179)	4 (3–4)
Eritrea	2026	5 (4–5)	0 (0–0)	164 (137–192)	4 (3–4)
Eritrea	2027	5 (4–6)	0 (0–0)	170 (135–205)	4 (3–4)
Eritrea	2028	5 (4–6)	0 (0–0)	175 (132–219)	4 (3–4)
Eritrea	2029	5 (4–7)	0 (0–0)	181 (128–234)	4 (3–4)
Eritrea	2030	5 (4–7)	0 (0–0)	186 (124–249)	4 (3–4)
Eritrea	2031	5 (4–7)	0 (0–0)	192 (119–264)	4 (3–4)
Eritrea	2032	6 (3–8)	0 (0–0)	197 (114–281)	4 (3–4)
Eritrea	2033	6 (3–8)	0 (0–0)	203 (108–297)	4 (3–4)
Eritrea	2034	6 (3–9)	0 (0–0)	208 (102–314)	4 (3–4)
Eritrea	2035	6 (3–9)	0 (0–0)	214 (96–332)	4 (3–4)
Eritrea	2036	6 (3–10)	0 (0–0)	219 (89–350)	4 (3–4)
Estonia	2022	19 (18–21)	1 (1–1)	467 (427–508)	20 (18–22)
Estonia	2023	19 (18–21)	1 (1–1)	467 (410–525)	20 (17–23)
Estonia	2024	19 (17–21)	1 (1–1)	467 (397–538)	20 (16–23)
Estonia	2025	19 (17–21)	1 (1–1)	467 (386–549)	20 (16–24)

Estonia	2026	19 (17–21)	1 (1–1)	467 (376–559)	20 (15–24)
Estonia	2027	19 (17–21)	1 (1–1)	467 (367–568)	20 (15–25)
Estonia	2028	19 (17–21)	1 (1–1)	467 (359–576)	20 (14–25)
Estonia	2029	19 (17–21)	1 (1–1)	467 (352–583)	20 (14–26)
Estonia	2030	19 (17–21)	1 (1–1)	467 (345–590)	20 (14–26)
Estonia	2031	19 (17–21)	1 (1–1)	467 (338–597)	20 (13–26)
Estonia	2032	19 (17–21)	1 (1–1)	467 (332–603)	20 (13–27)
Estonia	2033	19 (17–21)	1 (1–1)	467 (326–609)	20 (13–27)
Estonia	2034	19 (17–21)	1 (1–1)	467 (320–615)	20 (12–27)
Estonia	2035	19 (17–21)	1 (1–1)	467 (314–621)	20 (12–27)
Estonia	2036	19 (17–21)	1 (0–1)	467 (309–626)	20 (12–28)
Eswatini	2022	1 (1–1)	0 (0–0)	42 (41–42)	7 (7–7)
Eswatini	2023	1 (1–1)	0 (0–0)	40 (38–43)	7 (6–7)
Eswatini	2024	1 (1–1)	0 (0–0)	39 (35–43)	6 (6–7)
Eswatini	2025	1 (1–1)	0 (0–0)	38 (31–44)	6 (5–7)
Eswatini	2026	1 (1–1)	0 (0–0)	36 (27–46)	6 (5–8)
Eswatini	2027	1 (1–1)	0 (0–0)	35 (23–47)	7 (5–8)
Eswatini	2028	1 (0–2)	0 (0–0)	34 (19–49)	7 (5–9)
Eswatini	2029	1 (0–2)	0 (0–0)	33 (14–51)	7 (5–9)
Eswatini	2030	1 (-0–2)	0 (0–0)	31 (9–53)	7 (5–9)
Eswatini	2031	1 (-0–2)	0 (0–0)	30 (4–56)	7 (5–10)
Eswatini	2032	1 (-1–2)	0 (0–0)	29 (-1–59)	7 (5–10)
Eswatini	2033	1 (-1–2)	0 (0–0)	27 (-7–61)	8 (5–10)
Eswatini	2034	0 (-1–2)	0 (0–0)	26 (-12–64)	8 (5–10)
Eswatini	2035	0 (-1–2)	0 (0–0)	25 (-18–68)	8 (5–10)
Eswatini	2036	0 (-2–2)	0 (0–0)	24 (-24–71)	8 (5–11)
Ethiopia	2022	73 (72–74)	0 (0–0)	1866 (1842–1889)	4 (4–4)
Ethiopia	2023	73 (71–76)	0 (0–0)	1892 (1852–1932)	4 (4–4)
Ethiopia	2024	74 (70–78)	0 (0–0)	1908 (1842–1975)	4 (4–4)
Ethiopia	2025	75 (69–81)	0 (0–0)	1914 (1816–2013)	4 (4–5)
Ethiopia	2026	75 (67–84)	0 (0–0)	1910 (1777–2043)	4 (3–5)
Ethiopia	2027	76 (65–87)	0 (0–0)	1896 (1727–2065)	4 (3–5)
Ethiopia	2028	77 (63–90)	0 (0–0)	1872 (1668–2077)	4 (3–5)

Ethiopia	2029	77 (60–94)	0 (0–0)	1841 (1602–2080)	4 (3–5)
Ethiopia	2030	78 (58–98)	0 (0–0)	1802 (1531–2073)	4 (3–5)
Ethiopia	2031	78 (55–102)	0 (0–0)	1757 (1457–2058)	4 (2–6)
Ethiopia	2032	79 (52–106)	0 (0–0)	1708 (1381–2034)	4 (2–6)
Ethiopia	2033	79 (49–110)	0 (0–0)	1655 (1306–2004)	4 (2–6)
Ethiopia	2034	80 (45–115)	0 (0–0)	1600 (1232–1968)	4 (2–6)
Ethiopia	2035	81 (42–119)	0 (0–0)	1545 (1162–1927)	4 (1–7)
Ethiopia	2036	81 (38–124)	0 (0–0)	1490 (1097–1884)	4 (1–7)
Fiji	2022	3 (3–3)	0 (0–0)	84 (82–87)	10 (9–10)
Fiji	2023	3 (3–3)	0 (0–0)	86 (82–89)	10 (9–10)
Fiji	2024	3 (3–3)	0 (0–0)	87 (82–92)	10 (9–11)
Fiji	2025	3 (3–3)	0 (0–0)	89 (82–95)	10 (8–11)
Fiji	2026	3 (3–3)	0 (0–0)	90 (82–98)	10 (8–11)
Fiji	2027	3 (3–4)	0 (0–0)	91 (82–101)	10 (8–11)
Fiji	2028	3 (3–4)	0 (0–0)	93 (82–104)	10 (8–12)
Fiji	2029	3 (3–4)	0 (0–0)	94 (81–107)	10 (7–12)
Fiji	2030	3 (3–4)	0 (0–0)	96 (81–111)	10 (7–12)
Fiji	2031	3 (3–4)	0 (0–0)	97 (81–114)	10 (7–13)
Fiji	2032	3 (3–4)	0 (0–0)	99 (80–117)	10 (7–13)
Fiji	2033	4 (3–4)	0 (0–0)	100 (80–121)	10 (6–13)
Fiji	2034	4 (3–4)	0 (0–1)	102 (79–124)	10 (6–14)
Fiji	2035	4 (3–4)	0 (0–1)	103 (78–128)	10 (6–14)
Fiji	2036	4 (3–5)	0 (0–1)	105 (78–131)	10 (5–14)
Finland	2022	46 (44–47)	0 (0–0)	1086 (1058–1115)	10 (10–10)
Finland	2023	46 (44–47)	0 (0–0)	1096 (1055–1138)	10 (9–10)
Finland	2024	46 (43–48)	0 (0–0)	1104 (1057–1151)	10 (9–10)
Finland	2025	46 (43–48)	0 (0–0)	1109 (1060–1158)	9 (9–10)
Finland	2026	46 (42–49)	0 (0–0)	1111 (1062–1161)	9 (8–10)
Finland	2027	46 (42–49)	0 (0–0)	1113 (1063–1162)	9 (8–10)
Finland	2028	46 (42–49)	0 (0–0)	1113 (1064–1162)	9 (8–10)
Finland	2029	46 (41–50)	0 (0–0)	1113 (1064–1162)	9 (8–10)
Finland	2030	46 (41–50)	0 (0–0)	1113 (1063–1162)	8 (7–10)
Finland	2031	46 (41–50)	0 (0–0)	1113 (1063–1162)	8 (7–9)

Finland	2032	46 (41–51)	0 (0–0)	1113 (1063–1162)	8 (7–9)
Finland	2033	46 (40–51)	0 (0–0)	1112 (1063–1162)	8 (7–9)
Finland	2034	46 (40–51)	0 (0–0)	1112 (1063–1162)	8 (6–9)
Finland	2035	46 (40–51)	0 (0–0)	1112 (1063–1162)	8 (6–9)
Finland	2036	46 (40–51)	0 (0–0)	1112 (1063–1162)	7 (6–9)
France	2022	879 (860–898)	1 (1–1)	20709 (20278–21141)	17 (17–18)
France	2023	875 (843–906)	1 (1–1)	20966 (20296–21637)	17 (16–17)
France	2024	873 (830–916)	1 (1–1)	21469 (20654–22284)	16 (15–17)
France	2025	872 (820–925)	1 (1–1)	22074 (21203–22945)	16 (14–17)
France	2026	872 (812–932)	1 (1–1)	22628 (21753–23504)	15 (14–17)
France	2027	872 (804–940)	1 (0–1)	23001 (22116–23887)	15 (13–17)
France	2028	872 (798–946)	1 (0–1)	23119 (22183–24054)	15 (13–17)
France	2029	872 (792–952)	1 (0–1)	22974 (21962–23985)	14 (12–16)
France	2030	872 (786–958)	1 (0–1)	22623 (21547–23700)	14 (12–16)
France	2031	872 (781–963)	0 (0–1)	22170 (21061–23278)	13 (11–16)
France	2032	872 (776–968)	0 (0–1)	21731 (20617–22844)	13 (11–16)
France	2033	872 (771–973)	0 (0–1)	21412 (20296–22527)	13 (10–15)
France	2034	872 (766–977)	0 (0–1)	21279 (20144–22413)	12 (10–15)
France	2035	872 (762–982)	0 (0–1)	21348 (20178–22519)	12 (9–15)
France	2036	872 (758–986)	0 (0–0)	21587 (20382–22792)	12 (9–14)
Gabon	2022	3 (3–3)	0 (0–0)	90 (89–92)	8 (7–8)
Gabon	2023	3 (3–3)	0 (0–0)	91 (89–94)	8 (7–8)
Gabon	2024	3 (3–3)	0 (0–0)	92 (88–97)	8 (7–8)
Gabon	2025	3 (3–3)	0 (0–0)	93 (87–99)	8 (7–8)
Gabon	2026	3 (3–3)	0 (0–0)	94 (85–103)	8 (7–8)
Gabon	2027	3 (3–4)	0 (0–0)	95 (84–106)	8 (7–8)
Gabon	2028	3 (3–4)	0 (0–0)	96 (82–110)	8 (7–8)
Gabon	2029	3 (3–4)	0 (0–0)	97 (80–113)	8 (7–8)
Gabon	2030	3 (3–4)	0 (0–0)	98 (78–117)	8 (7–8)
Gabon	2031	3 (3–4)	0 (0–0)	98 (75–121)	8 (7–8)
Gabon	2032	3 (3–4)	0 (0–0)	99 (73–126)	8 (7–8)
Gabon	2033	3 (2–4)	0 (0–0)	100 (70–130)	8 (7–8)
Gabon	2034	3 (2–4)	0 (0–0)	101 (68–134)	8 (7–8)

Gabon	2035	3 (2–5)	0 (0–0)	102 (65–139)	8 (7–8)
Gabon	2036	3 (2–5)	0 (0–0)	103 (62–144)	8 (7–8)
Gambia	2022	0 (0–1)	0 (0–0)	14 (14–15)	1 (1–1)
Gambia	2023	1 (0–1)	0 (0–0)	15 (14–16)	1 (1–1)
Gambia	2024	1 (0–1)	0 (0–0)	15 (14–16)	1 (1–1)
Gambia	2025	1 (0–1)	0 (0–0)	15 (14–16)	1 (1–1)
Gambia	2026	1 (0–1)	0 (0–0)	15 (14–17)	1 (1–1)
Gambia	2027	1 (0–1)	0 (0–0)	15 (14–17)	1 (1–1)
Gambia	2028	1 (0–1)	0 (0–0)	16 (14–18)	1 (1–1)
Gambia	2029	1 (0–1)	0 (0–0)	16 (14–18)	1 (1–1)
Gambia	2030	1 (0–1)	0 (0–0)	16 (14–18)	1 (1–1)
Gambia	2031	1 (0–1)	0 (0–0)	16 (14–19)	1 (1–1)
Gambia	2032	1 (1–1)	0 (0–0)	17 (14–19)	1 (1–1)
Gambia	2033	1 (1–1)	0 (0–0)	17 (14–19)	1 (1–1)
Gambia	2034	1 (1–1)	0 (0–0)	17 (14–20)	1 (1–1)
Gambia	2035	1 (1–1)	0 (0–0)	17 (15–20)	1 (1–1)
Gambia	2036	1 (1–1)	0 (0–0)	17 (15–20)	1 (1–1)
Georgia	2022	43 (39–48)	1 (1–1)	1153 (1024–1281)	21 (19–23)
Georgia	2023	43 (37–50)	1 (1–1)	1153 (970–1335)	21 (18–24)
Georgia	2024	43 (35–52)	1 (1–1)	1153 (929–1376)	21 (17–25)
Georgia	2025	43 (34–53)	1 (1–1)	1153 (895–1410)	21 (16–25)
Georgia	2026	43 (33–54)	1 (1–1)	1153 (865–1441)	21 (16–26)
Georgia	2027	43 (32–55)	1 (1–1)	1153 (837–1468)	21 (15–26)
Georgia	2028	43 (31–56)	1 (1–1)	1153 (812–1493)	21 (15–27)
Georgia	2029	43 (30–57)	1 (1–1)	1153 (788–1517)	21 (14–27)
Georgia	2030	43 (29–58)	1 (1–1)	1153 (766–1539)	21 (14–28)
Georgia	2031	43 (29–58)	1 (0–1)	1153 (745–1560)	21 (14–28)
Georgia	2032	43 (28–59)	1 (0–1)	1153 (725–1580)	21 (13–28)
Georgia	2033	43 (27–60)	1 (0–1)	1153 (706–1599)	21 (13–29)
Georgia	2034	43 (26–60)	1 (0–1)	1153 (688–1617)	21 (13–29)
Georgia	2035	43 (26–61)	1 (0–1)	1153 (671–1635)	21 (12–29)
Georgia	2036	43 (25–62)	1 (0–1)	1153 (654–1651)	21 (12–30)
Germany	2022	1199 (1154–1244)	1 (1–1)	28059 (26967–29150)	17 (16–18)

Germany	2023	1184 (1120–1249)	1 (1–1)	27678 (26134–29221)	16 (15–18)
Germany	2024	1170 (1091–1248)	1 (1–1)	27297 (25406–29187)	16 (14–17)
Germany	2025	1155 (1064–1246)	1 (1–1)	26915 (24732–29099)	15 (14–17)
Germany	2026	1140 (1039–1242)	1 (0–1)	26534 (24094–28975)	15 (13–17)
Germany	2027	1126 (1015–1237)	1 (0–1)	26153 (23480–28827)	14 (12–16)
Germany	2028	1111 (991–1231)	1 (0–1)	25772 (22884–28660)	14 (12–16)
Germany	2029	1096 (968–1224)	0 (0–1)	25391 (22304–28478)	13 (11–16)
Germany	2030	1081 (945–1217)	0 (0–1)	25010 (21735–28285)	13 (10–15)
Germany	2031	1067 (923–1210)	0 (0–1)	24629 (21177–28081)	12 (10–15)
Germany	2032	1052 (902–1202)	0 (0–1)	24248 (20628–27868)	12 (9–14)
Germany	2033	1037 (880–1194)	0 (0–1)	23867 (20086–27648)	11 (8–14)
Germany	2034	1023 (859–1186)	0 (0–0)	23486 (19550–27421)	11 (8–14)
Germany	2035	1008 (838–1177)	0 (0–0)	23105 (19020–27189)	10 (7–13)
Germany	2036	993 (818–1169)	0 (0–0)	22723 (18496–26951)	10 (6–13)
Ghana	2022	13 (13–13)	0 (0–0)	342 (340–345)	2 (2–2)
Ghana	2023	13 (13–14)	0 (0–0)	347 (341–352)	2 (2–2)
Ghana	2024	14 (13–14)	0 (0–0)	351 (342–361)	2 (2–2)
Ghana	2025	14 (13–14)	0 (0–0)	356 (342–370)	2 (2–2)
Ghana	2026	14 (13–15)	0 (0–0)	360 (342–379)	2 (2–2)
Ghana	2027	14 (13–15)	0 (0–0)	365 (341–389)	2 (2–2)
Ghana	2028	14 (13–15)	0 (0–0)	369 (340–399)	2 (2–2)
Ghana	2029	14 (13–16)	0 (0–0)	374 (338–410)	2 (2–2)
Ghana	2030	14 (12–16)	0 (0–0)	378 (336–421)	2 (2–2)
Ghana	2031	14 (12–16)	0 (0–0)	383 (333–432)	2 (2–2)
Ghana	2032	14 (12–17)	0 (0–0)	387 (331–444)	2 (1–2)
Ghana	2033	15 (12–17)	0 (0–0)	392 (328–456)	2 (1–2)
Ghana	2034	15 (12–18)	0 (0–0)	396 (324–468)	2 (1–2)
Ghana	2035	15 (11–18)	0 (0–0)	401 (321–481)	2 (1–2)
Ghana	2036	15 (11–19)	0 (0–0)	405 (317–494)	2 (1–2)
Greece	2022	203 (193–213)	1 (1–1)	4336 (4133–4539)	21 (20–22)
Greece	2023	205 (191–219)	1 (1–1)	4370 (4083–4657)	21 (20–22)
Greece	2024	207 (190–225)	1 (1–1)	4404 (4052–4756)	21 (19–23)
Greece	2025	209 (189–229)	1 (1–1)	4438 (4032–4844)	21 (19–23)

Greece	2026	211 (189–234)	1 (1–1)	4472 (4018–4926)	21 (19–23)
Greece	2027	214 (189–238)	1 (1–1)	4506 (4009–5004)	21 (18–24)
Greece	2028	216 (189–242)	1 (1–1)	4540 (4003–5078)	21 (18–24)
Greece	2029	218 (189–246)	1 (1–1)	4574 (4000–5149)	21 (18–24)
Greece	2030	220 (190–250)	1 (1–1)	4608 (3999–5217)	21 (18–24)
Greece	2031	222 (190–253)	1 (1–1)	4642 (4000–5284)	21 (18–24)
Greece	2032	224 (191–257)	1 (1–1)	4676 (4003–5350)	21 (18–24)
Greece	2033	226 (191–261)	1 (1–1)	4710 (4007–5414)	21 (17–25)
Greece	2034	228 (192–264)	1 (1–1)	4744 (4012–5477)	21 (17–25)
Greece	2035	230 (193–267)	1 (1–1)	4778 (4018–5538)	21 (17–25)
Greece	2036	232 (193–271)	1 (1–1)	4812 (4026–5599)	21 (17–25)
Greenland	2022	1 (1–1)	2 (1–2)	35 (34–36)	41 (39–43)
Greenland	2023	1 (1–1)	1 (1–2)	34 (33–35)	40 (38–42)
Greenland	2024	1 (1–1)	1 (1–2)	34 (32–36)	39 (36–41)
Greenland	2025	1 (1–1)	1 (1–2)	34 (32–36)	37 (34–41)
Greenland	2026	1 (1–1)	1 (1–1)	34 (31–36)	36 (33–40)
Greenland	2027	1 (1–1)	1 (1–1)	34 (30–37)	35 (31–39)
Greenland	2028	1 (1–1)	1 (1–1)	33 (30–37)	34 (29–38)
Greenland	2029	1 (1–1)	1 (1–1)	33 (29–38)	32 (28–37)
Greenland	2030	1 (1–1)	1 (1–1)	33 (28–38)	31 (26–36)
Greenland	2031	1 (1–1)	1 (1–1)	33 (27–39)	30 (25–35)
Greenland	2032	1 (1–1)	1 (1–1)	33 (27–39)	29 (23–34)
Greenland	2033	1 (1–1)	1 (1–1)	33 (26–40)	28 (22–33)
Greenland	2034	1 (1–1)	1 (1–1)	33 (25–40)	26 (21–32)
Greenland	2035	1 (1–1)	1 (1–1)	32 (24–41)	25 (19–31)
Greenland	2036	1 (1–1)	1 (1–1)	32 (23–42)	24 (18–30)
Grenada	2022	1 (0–1)	0 (0–0)	13 (13–14)	11 (10–12)
Grenada	2023	1 (0–1)	0 (0–0)	14 (13–14)	11 (10–12)
Grenada	2024	1 (0–1)	0 (0–0)	14 (13–15)	11 (10–12)
Grenada	2025	1 (0–1)	0 (0–1)	14 (13–15)	11 (10–12)
Grenada	2026	1 (0–1)	0 (0–1)	14 (13–16)	11 (9–12)
Grenada	2027	1 (0–1)	0 (0–1)	15 (13–16)	11 (9–13)
Grenada	2028	1 (1–1)	0 (0–1)	15 (14–16)	11 (9–13)

Grenada	2029	1 (1–1)	0 (0–1)	15 (14–16)	11 (9–13)
Grenada	2030	1 (1–1)	0 (0–1)	15 (14–17)	11 (9–13)
Grenada	2031	1 (1–1)	0 (0–1)	16 (14–17)	11 (9–13)
Grenada	2032	1 (1–1)	0 (0–1)	16 (14–17)	11 (9–13)
Grenada	2033	1 (1–1)	0 (0–1)	16 (14–18)	11 (9–13)
Grenada	2034	1 (1–1)	0 (0–1)	16 (14–18)	11 (9–13)
Grenada	2035	1 (1–1)	0 (0–1)	16 (15–18)	11 (8–13)
Grenada	2036	1 (1–1)	0 (0–1)	17 (15–19)	11 (8–14)
Guam	2022	1 (1–1)	0 (0–0)	29 (28–30)	14 (13–15)
Guam	2023	1 (1–1)	0 (0–0)	29 (27–32)	14 (12–15)
Guam	2024	1 (1–1)	0 (0–0)	30 (27–33)	14 (12–15)
Guam	2025	1 (1–1)	0 (0–0)	30 (26–34)	14 (12–16)
Guam	2026	1 (1–1)	0 (0–0)	31 (26–35)	14 (12–16)
Guam	2027	1 (1–1)	0 (0–0)	31 (26–36)	14 (12–16)
Guam	2028	1 (1–1)	0 (0–1)	31 (26–37)	14 (12–16)
Guam	2029	1 (1–1)	0 (0–1)	32 (26–38)	14 (12–16)
Guam	2030	1 (1–1)	0 (0–1)	32 (26–39)	14 (12–16)
Guam	2031	1 (1–1)	0 (0–1)	33 (26–39)	14 (12–17)
Guam	2032	1 (1–1)	0 (0–1)	33 (26–40)	14 (12–17)
Guam	2033	1 (1–1)	0 (0–1)	34 (26–41)	14 (12–17)
Guam	2034	1 (1–1)	0 (0–1)	34 (26–42)	14 (12–17)
Guam	2035	1 (1–1)	0 (0–1)	35 (27–43)	14 (12–17)
Guam	2036	1 (1–1)	0 (0–1)	35 (27–43)	14 (12–17)
Guatemala	2022	17 (16–18)	0 (0–0)	458 (434–482)	4 (3–4)
Guatemala	2023	18 (16–19)	0 (0–0)	469 (435–503)	4 (3–5)
Guatemala	2024	18 (16–20)	0 (0–0)	480 (438–521)	4 (3–5)
Guatemala	2025	18 (17–20)	0 (0–0)	491 (443–539)	4 (3–5)
Guatemala	2026	19 (17–21)	0 (0–0)	502 (449–556)	4 (3–5)
Guatemala	2027	19 (17–22)	0 (0–0)	513 (455–572)	4 (3–5)
Guatemala	2028	20 (17–22)	0 (0–0)	524 (461–588)	4 (3–5)
Guatemala	2029	20 (18–23)	0 (0–0)	536 (468–603)	4 (3–5)
Guatemala	2030	21 (18–23)	0 (0–0)	547 (475–618)	4 (3–5)
Guatemala	2031	21 (18–24)	0 (0–0)	558 (482–633)	4 (2–5)

Guatemala	2032	21 (18–25)	0 (0–0)	569 (489–648)	4 (2–5)
Guatemala	2033	22 (19–25)	0 (0–0)	580 (497–663)	4 (2–5)
Guatemala	2034	22 (19–26)	0 (0–0)	591 (505–677)	4 (2–5)
Guatemala	2035	23 (19–26)	0 (0–0)	602 (513–692)	4 (2–6)
Guatemala	2036	23 (20–27)	0 (0–0)	613 (521–706)	4 (2–6)
Guinea	2022	5 (5–5)	0 (0–0)	138 (137–140)	2 (2–2)
Guinea	2023	5 (5–5)	0 (0–0)	139 (136–142)	2 (2–2)
Guinea	2024	5 (5–5)	0 (0–0)	139 (134–145)	2 (2–2)
Guinea	2025	5 (5–5)	0 (0–0)	140 (132–148)	2 (2–2)
Guinea	2026	5 (5–5)	0 (0–0)	141 (130–151)	2 (2–2)
Guinea	2027	5 (4–5)	0 (0–0)	141 (127–155)	2 (2–2)
Guinea	2028	5 (4–6)	0 (0–0)	142 (124–159)	2 (2–3)
Guinea	2029	5 (4–6)	0 (0–0)	142 (121–163)	2 (2–3)
Guinea	2030	5 (4–6)	0 (0–0)	143 (118–168)	2 (2–3)
Guinea	2031	5 (4–6)	0 (0–0)	143 (115–172)	2 (1–3)
Guinea	2032	5 (4–6)	0 (0–0)	144 (111–177)	2 (1–3)
Guinea	2033	5 (4–6)	0 (0–0)	145 (107–182)	2 (1–3)
Guinea	2034	5 (3–6)	0 (0–0)	145 (104–187)	2 (1–3)
Guinea	2035	5 (3–6)	0 (0–0)	146 (99–192)	2 (1–3)
Guinea	2036	5 (3–7)	0 (0–0)	146 (95–198)	2 (1–3)
Guinea-Bissau	2022	1 (1–1)	0 (0–0)	22 (22–22)	3 (3–3)
Guinea-Bissau	2023	1 (1–1)	0 (0–0)	22 (22–23)	3 (2–3)
Guinea-Bissau	2024	1 (1–1)	0 (0–0)	23 (22–23)	2 (2–3)
Guinea-Bissau	2025	1 (1–1)	0 (0–0)	23 (22–24)	2 (2–3)
Guinea-Bissau	2026	1 (1–1)	0 (0–0)	23 (22–25)	2 (2–3)
Guinea-Bissau	2027	1 (1–1)	0 (0–0)	23 (21–26)	2 (2–3)
Guinea-Bissau	2028	1 (1–1)	0 (0–0)	24 (21–26)	2 (2–3)
Guinea-Bissau	2029	1 (1–1)	0 (0–0)	24 (21–27)	2 (2–3)
Guinea-Bissau	2030	1 (1–1)	0 (0–0)	24 (21–28)	2 (2–3)
Guinea-Bissau	2031	1 (1–1)	0 (0–0)	25 (20–29)	2 (2–3)
Guinea-Bissau	2032	1 (0–1)	0 (0–0)	25 (20–30)	2 (2–3)
Guinea-Bissau	2033	1 (0–1)	0 (0–0)	25 (20–31)	2 (1–3)
Guinea-Bissau	2034	1 (0–1)	0 (0–0)	25 (19–32)	2 (1–3)

Guinea-Bissau	2035	1 (0–1)	0 (0–0)	26 (19–33)	2 (1–3)
Guinea-Bissau	2036	1 (0–1)	0 (0–0)	26 (18–34)	2 (1–3)
Guyana	2022	2 (2–3)	0 (0–0)	71 (66–76)	10 (9–11)
Guyana	2023	2 (2–3)	0 (0–0)	72 (65–80)	10 (8–12)
Guyana	2024	3 (2–3)	0 (0–0)	73 (64–83)	10 (8–12)
Guyana	2025	3 (2–3)	0 (0–0)	74 (63–85)	10 (8–12)
Guyana	2026	3 (2–3)	0 (0–0)	75 (63–87)	10 (7–13)
Guyana	2027	3 (2–3)	0 (0–0)	76 (63–90)	10 (7–13)
Guyana	2028	3 (2–3)	0 (0–0)	77 (63–92)	10 (7–13)
Guyana	2029	3 (2–3)	0 (0–0)	78 (63–94)	10 (7–13)
Guyana	2030	3 (2–3)	0 (0–1)	80 (63–96)	10 (6–14)
Guyana	2031	3 (2–3)	0 (0–1)	81 (64–98)	10 (6–14)
Guyana	2032	3 (2–3)	0 (0–1)	82 (64–100)	10 (6–14)
Guyana	2033	3 (2–3)	0 (0–1)	83 (64–101)	10 (6–14)
Guyana	2034	3 (2–4)	0 (0–1)	84 (64–103)	10 (6–14)
Guyana	2035	3 (2–4)	0 (0–1)	85 (65–105)	10 (6–14)
Guyana	2036	3 (2–4)	0 (0–1)	86 (65–107)	10 (5–15)
Haiti	2022	18 (17–18)	0 (0–0)	494 (487–502)	6 (6–6)
Haiti	2023	18 (17–18)	0 (0–0)	503 (491–516)	6 (6–6)
Haiti	2024	18 (17–19)	0 (0–0)	513 (493–532)	6 (6–6)
Haiti	2025	18 (17–19)	0 (0–0)	521 (494–549)	6 (6–7)
Haiti	2026	19 (17–20)	0 (0–0)	531 (494–567)	6 (5–7)
Haiti	2027	19 (17–20)	0 (0–0)	540 (494–586)	6 (5–7)
Haiti	2028	19 (17–21)	0 (0–0)	549 (492–605)	6 (5–7)
Haiti	2029	19 (17–22)	0 (0–0)	558 (490–625)	6 (5–7)
Haiti	2030	20 (17–22)	0 (0–0)	567 (488–646)	6 (4–7)
Haiti	2031	20 (17–23)	0 (0–0)	576 (484–668)	6 (4–7)
Haiti	2032	20 (16–24)	0 (0–0)	585 (481–690)	6 (4–8)
Haiti	2033	20 (16–24)	0 (0–0)	594 (476–712)	6 (3–8)
Haiti	2034	21 (16–25)	0 (0–0)	603 (471–735)	6 (3–8)
Haiti	2035	21 (16–26)	0 (0–0)	612 (466–759)	6 (3–8)
Haiti	2036	21 (15–27)	0 (0–0)	621 (460–783)	5 (2–8)
High SDI	2022	[illegible]220 (13084–1335[illegible]	1 (1–1)	[illegible]14872 (311301–31844[illegible]	17 (16–17)

High SDI	2023	220 (13028–1341	1 (1–1)	14239 (309188–31929	16 (16–17)
High SDI	2024	220 (12984–1345	1 (1–1)	13605 (307419–31979	16 (15–16)
High SDI	2025	220 (12948–1349	1 (1–1)	12972 (305829–32011	15 (15–16)
High SDI	2026	220 (12916–1352	1 (1–1)	12338 (304352–32032	15 (14–16)
High SDI	2027	220 (12887–1355	1 (1–1)	11704 (302956–32045	15 (13–16)
High SDI	2028	220 (12860–1357	1 (0–1)	11071 (301621–32052	14 (13–15)
High SDI	2029	220 (12836–1360	1 (0–1)	10437 (300335–32053	14 (12–15)
High SDI	2030	220 (12812–1362	1 (0–1)	09803 (299089–32051	13 (12–15)
High SDI	2031	220 (12790–1364	0 (0–1)	09170 (297876–32046	13 (11–14)
High SDI	2032	220 (12769–1367	0 (0–1)	08536 (296691–32038	12 (11–14)
High SDI	2033	220 (12749–1369	0 (0–1)	07902 (295531–32027	12 (10–14)
High SDI	2034	220 (12730–1370	0 (0–1)	07269 (294392–32014	11 (10–13)
High SDI	2035	220 (12712–1372	0 (0–1)	06635 (293272–31999	11 (9–13)
High SDI	2036	220 (12694–1374	0 (0–0)	06002 (292169–31983	11 (9–13)
High-middle SDI	2022	921 (15717–1612	1 (1–1)	17670 (412007–42333	21 (20–21)
High-middle SDI	2023	122 (15835–1640	1 (1–1)	22320 (414310–43033	21 (20–21)
High-middle SDI	2024	323 (15971–1667	1 (1–1)	26969 (417159–43677	21 (20–21)
High-middle SDI	2025	525 (16118–1693	1 (1–1)	31618 (420291–44294	20 (19–21)
High-middle SDI	2026	726 (16271–1718	1 (1–1)	36267 (423603–44893	20 (19–21)
High-middle SDI	2027	927 (16429–1742	1 (1–1)	40917 (427043–45479	20 (19–21)
High-middle SDI	2028	128 (16591–1766	1 (1–1)	45566 (430581–46055	20 (18–21)
High-middle SDI	2029	330 (16755–1790	1 (1–1)	50215 (434196–46623	20 (18–21)
High-middle SDI	2030	531 (16921–1814	1 (1–1)	54864 (437873–47185	20 (18–21)
High-middle SDI	2031	732 (17090–1837	1 (1–1)	59514 (441603–47742	19 (18–21)
High-middle SDI	2032	933 (17259–1860	1 (1–1)	64163 (445378–48294	19 (17–21)
High-middle SDI	2033	135 (17431–1883	1 (1–1)	68812 (449192–48843	19 (17–21)
High-middle SDI	2034	336 (17603–1906	1 (1–1)	73461 (453040–49388	19 (17–21)
High-middle SDI	2035	537 (17777–1929	1 (1–1)	78111 (456919–49930	19 (17–21)
High-middle SDI	2036	739 (17952–1952	1 (1–1)	82760 (460824–50469	19 (16–21)
Honduras	2022	11 (11–12)	0 (0–0)	310 (303–317)	5 (4–5)
Honduras	2023	12 (11–12)	0 (0–0)	317 (303–330)	4 (4–5)
Honduras	2024	12 (11–13)	0 (0–0)	323 (302–344)	4 (4–5)
Honduras	2025	12 (11–13)	0 (0–0)	330 (300–359)	4 (4–5)

Honduras	2026	12 (10–14)	0 (0–0)	336 (297–375)	4 (4–5)
Honduras	2027	12 (10–15)	0 (0–0)	342 (293–392)	4 (4–5)
Honduras	2028	12 (10–15)	0 (0–0)	349 (289–409)	4 (4–5)
Honduras	2029	13 (9–16)	0 (0–0)	355 (284–427)	4 (4–5)
Honduras	2030	13 (9–17)	0 (0–0)	362 (278–446)	4 (4–5)
Honduras	2031	13 (9–17)	0 (0–0)	368 (271–465)	4 (4–5)
Honduras	2032	13 (8–18)	0 (0–0)	375 (264–485)	4 (4–5)
Honduras	2033	13 (8–19)	0 (0–0)	381 (256–506)	4 (4–5)
Honduras	2034	14 (7–20)	0 (0–0)	388 (248–527)	4 (3–5)
Honduras	2035	14 (7–21)	0 (0–0)	394 (239–549)	4 (3–6)
Honduras	2036	14 (6–22)	0 (0–0)	400 (230–571)	4 (3–6)
Hungary	2022	226 (214–237)	1 (1–1)	5949 (5622–6276)	34 (32–35)
Hungary	2023	226 (210–242)	1 (1–1)	5963 (5509–6418)	33 (31–35)
Hungary	2024	227 (207–246)	1 (1–1)	5977 (5429–6526)	32 (30–34)
Hungary	2025	227 (205–249)	1 (1–1)	5991 (5367–6614)	32 (29–34)
Hungary	2026	227 (203–252)	1 (1–1)	6004 (5318–6690)	31 (28–34)
Hungary	2027	228 (202–254)	1 (1–1)	6016 (5276–6756)	30 (26–34)
Hungary	2028	228 (200–256)	1 (1–1)	6028 (5241–6816)	30 (25–35)
Hungary	2029	228 (199–258)	1 (1–1)	6040 (5211–6869)	29 (23–35)
Hungary	2030	229 (198–259)	1 (1–1)	6052 (5185–6918)	28 (21–35)
Hungary	2031	229 (197–261)	1 (1–1)	6063 (5163–6962)	28 (20–36)
Hungary	2032	229 (196–262)	1 (1–1)	6073 (5143–7003)	27 (18–37)
Hungary	2033	230 (196–263)	1 (0–1)	6083 (5126–7041)	27 (16–37)
Hungary	2034	230 (195–264)	1 (0–1)	6093 (5111–7076)	26 (14–38)
Hungary	2035	230 (195–265)	1 (0–1)	6103 (5098–7108)	25 (12–39)
Hungary	2036	230 (194–266)	1 (0–1)	6112 (5086–7139)	25 (10–40)
Iceland	2022	2 (2–2)	0 (0–0)	54 (52–57)	10 (9–10)
Iceland	2023	2 (2–3)	0 (0–0)	54 (51–58)	9 (8–10)
Iceland	2024	2 (2–3)	0 (0–0)	54 (50–59)	9 (7–10)
Iceland	2025	2 (2–3)	0 (0–0)	54 (50–59)	8 (7–10)
Iceland	2026	2 (2–3)	0 (0–0)	54 (49–60)	8 (6–10)
Iceland	2027	2 (2–3)	0 (0–0)	54 (49–60)	8 (6–10)
Iceland	2028	2 (2–3)	0 (0–0)	54 (48–61)	7 (5–10)

Iceland	2029	2 (2–3)	0 (0–0)	54 (48–61)	7 (5–9)
Iceland	2030	2 (2–3)	0 (0–0)	54 (47–62)	7 (4–9)
Iceland	2031	2 (2–3)	0 (0–0)	54 (47–62)	6 (4–9)
Iceland	2032	2 (2–3)	0 (0–0)	54 (47–62)	6 (3–9)
Iceland	2033	2 (2–3)	0 (0–0)	54 (46–63)	5 (3–8)
Iceland	2034	2 (2–3)	0 (0–0)	54 (46–63)	5 (2–8)
Iceland	2035	2 (2–3)	0 (0–0)	54 (46–63)	5 (2–8)
Iceland	2036	2 (2–3)	0 (0–0)	54 (45–64)	4 (1–8)
India	2022	1655 (1618–1691)	0 (0–0)	44205 (43337–45073)	3 (3–4)
India	2023	1689 (1632–1746)	0 (0–0)	45006 (43424–46587)	3 (3–4)
India	2024	1724 (1648–1800)	0 (0–0)	45807 (43639–47974)	3 (3–4)
India	2025	1759 (1663–1854)	0 (0–0)	46607 (43888–49327)	3 (3–3)
India	2026	1793 (1677–1909)	0 (0–0)	47408 (44146–50670)	3 (3–3)
India	2027	1828 (1691–1965)	0 (0–0)	48209 (44402–52016)	3 (3–3)
India	2028	1863 (1704–2021)	0 (0–0)	49010 (44651–53369)	3 (3–3)
India	2029	1897 (1717–2078)	0 (0–0)	49811 (44891–54731)	3 (3–3)
India	2030	1932 (1728–2136)	0 (0–0)	50612 (45118–56105)	3 (3–3)
India	2031	1967 (1739–2194)	0 (0–0)	51412 (45334–57491)	3 (3–3)
India	2032	2001 (1749–2253)	0 (0–0)	52213 (45536–58891)	3 (3–3)
India	2033	2036 (1758–2313)	0 (0–0)	53014 (45725–60303)	3 (3–3)
India	2034	2071 (1767–2374)	0 (0–0)	53815 (45901–61729)	3 (3–3)
India	2035	2105 (1775–2436)	0 (0–0)	54616 (46063–63169)	3 (2–3)
India	2036	2140 (1782–2498)	0 (0–0)	55417 (46211–64622)	3 (2–3)
Indonesia	2022	1547 (1537–1557)	1 (1–1)	43797 (43530–44064)	16 (16–16)
Indonesia	2023	1595 (1573–1617)	1 (1–1)	45075 (44477–45673)	16 (16–16)
Indonesia	2024	1643 (1606–1680)	1 (1–1)	46353 (45352–47353)	16 (15–17)
Indonesia	2025	1691 (1637–1745)	1 (1–1)	47630 (46166–49095)	16 (15–17)
Indonesia	2026	1740 (1666–1813)	1 (1–1)	48908 (46925–50891)	16 (15–17)
Indonesia	2027	1788 (1694–1882)	1 (1–1)	50186 (47636–52737)	16 (14–18)
Indonesia	2028	1836 (1719–1953)	1 (1–1)	51464 (48300–54628)	16 (14–18)
Indonesia	2029	1884 (1743–2025)	1 (1–1)	52742 (48923–56561)	16 (14–18)
Indonesia	2030	1932 (1766–2099)	1 (1–1)	54020 (49506–58533)	16 (13–19)
Indonesia	2031	1981 (1787–2174)	1 (1–1)	55297 (50051–60544)	16 (13–19)

Indonesia	2032	2029 (1807–2251)	1 (0–1)	56575 (50561–62590)	16 (12–20)
Indonesia	2033	2077 (1825–2328)	1 (0–1)	57853 (51036–64670)	16 (12–20)
Indonesia	2034	2125 (1843–2407)	1 (0–1)	59131 (51479–66783)	16 (11–21)
Indonesia	2035	2173 (1859–2488)	1 (0–1)	60409 (51891–68927)	16 (11–21)
Indonesia	2036	2222 (1874–2569)	1 (0–1)	61687 (52271–71102)	16 (10–22)
Iran (Islamic Republic of)	2022	169 (163–175)	0 (0–0)	4915 (4734–5097)	6 (5–6)
Iran (Islamic Republic of)	2023	170 (160–180)	0 (0–0)	4976 (4688–5265)	6 (5–6)
Iran (Islamic Republic of)	2024	166 (151–182)	0 (0–0)	4852 (4380–5323)	5 (5–6)
Iran (Islamic Republic of)	2025	166 (144–187)	0 (0–0)	4869 (4224–5515)	5 (5–6)
Iran (Islamic Republic of)	2026	163 (134–192)	0 (0–0)	4778 (3917–5640)	5 (4–6)
Iran (Islamic Republic of)	2027	162 (126–198)	0 (0–0)	4770 (3689–5851)	5 (4–6)
Iran (Islamic Republic of)	2028	160 (115–204)	0 (0–0)	4699 (3371–6026)	5 (4–6)
Iran (Islamic Republic of)	2029	158 (105–211)	0 (0–0)	4676 (3093–6259)	5 (4–7)
Iran (Islamic Republic of)	2030	156 (94–218)	0 (0–0)	4615 (2757–6474)	5 (4–7)
Iran (Islamic Republic of)	2031	155 (83–226)	0 (0–0)	4584 (2439–6729)	5 (3–7)
Iran (Islamic Republic of)	2032	153 (71–235)	0 (0–0)	4530 (2083–6977)	5 (3–7)
Iran (Islamic Republic of)	2033	151 (59–243)	0 (0–0)	4493 (1733–7254)	5 (3–7)
Iran (Islamic Republic of)	2034	149 (46–253)	0 (0–0)	4444 (1357–7531)	5 (3–7)
Iran (Islamic Republic of)	2035	147 (33–262)	0 (0–0)	4404 (979–7829)	5 (3–7)
Iran (Islamic Republic of)	2036	146 (19–272)	0 (0–0)	4357 (581–8132)	5 (3–7)
Iraq	2022	81 (79–83)	0 (0–0)	2282 (2243–2320)	9 (8–9)
Iraq	2023	84 (81–88)	0 (0–0)	2336 (2250–2423)	9 (8–9)
Iraq	2024	88 (83–92)	0 (0–0)	2391 (2246–2535)	9 (8–9)
Iraq	2025	91 (85–97)	0 (0–0)	2445 (2233–2657)	9 (8–9)
Iraq	2026	95 (87–103)	0 (0–0)	2500 (2213–2787)	9 (8–10)
Iraq	2027	98 (88–108)	0 (0–0)	2554 (2185–2923)	9 (8–10)
Iraq	2028	101 (90–113)	0 (0–0)	2609 (2151–3066)	9 (8–10)
Iraq	2029	105 (91–119)	0 (0–0)	2663 (2111–3216)	9 (8–11)
Iraq	2030	108 (92–124)	0 (0–0)	2718 (2065–3371)	9 (8–11)
Iraq	2031	112 (93–130)	0 (0–0)	2772 (2013–3531)	9 (8–11)
Iraq	2032	115 (94–136)	0 (0–0)	2827 (1957–3697)	10 (8–11)
Iraq	2033	118 (95–142)	0 (0–0)	2881 (1895–3867)	10 (8–12)
Iraq	2034	122 (96–148)	0 (0–0)	2936 (1829–4043)	10 (7–12)

Iraq	2035	125 (97–154)	0 (0–0)	2990 (1758–4222)	10 (7–12)
Iraq	2036	129 (98–160)	0 (0–0)	3045 (1683–4407)	10 (7–13)
Ireland	2022	47 (45–49)	1 (1–1)	1075 (1027–1124)	13 (13–14)
Ireland	2023	46 (43–49)	1 (0–1)	1058 (989–1126)	13 (11–14)
Ireland	2024	45 (42–48)	0 (0–1)	1040 (956–1124)	12 (10–13)
Ireland	2025	44 (40–48)	0 (0–1)	1022 (926–1119)	11 (9–13)
Ireland	2026	43 (39–48)	0 (0–0)	1005 (897–1113)	10 (8–12)
Ireland	2027	43 (38–48)	0 (0–0)	987 (869–1105)	9 (7–11)
Ireland	2028	42 (37–47)	0 (0–0)	969 (842–1097)	8 (6–11)
Ireland	2029	41 (36–47)	0 (0–0)	951 (815–1088)	7 (5–10)
Ireland	2030	40 (34–46)	0 (0–0)	934 (789–1079)	7 (4–9)
Ireland	2031	40 (33–46)	0 (0–0)	916 (763–1069)	6 (3–9)
Ireland	2032	39 (32–46)	0 (0–0)	898 (738–1058)	5 (2–8)
Ireland	2033	38 (31–45)	0 (0–0)	881 (714–1048)	4 (1–7)
Ireland	2034	37 (30–45)	0 (-0–0)	863 (689–1037)	3 (-0–6)
Ireland	2035	37 (29–44)	0 (-0–0)	845 (665–1026)	2 (-1–6)
Ireland	2036	36 (28–44)	0 (-0–0)	828 (641–1014)	1 (-2–5)
Israel	2022	57 (55–59)	0 (0–0)	1312 (1251–1373)	11 (10–12)
Israel	2023	57 (53–60)	0 (0–0)	1293 (1208–1379)	10 (9–12)
Israel	2024	57 (52–61)	0 (0–0)	1290 (1172–1408)	10 (8–12)
Israel	2025	57 (51–62)	0 (0–0)	1285 (1130–1441)	9 (6–12)
Israel	2026	57 (50–64)	0 (0–1)	1284 (1098–1471)	9 (5–13)
Israel	2027	57 (49–65)	0 (0–1)	1289 (1072–1506)	8 (3–13)
Israel	2028	57 (48–66)	0 (0–1)	1295 (1050–1540)	8 (2–14)
Israel	2029	57 (47–68)	0 (0–1)	1304 (1036–1572)	7 (-0–14)
Israel	2030	58 (47–69)	0 (-0–1)	1316 (1029–1604)	7 (-2–15)
Israel	2031	58 (47–70)	0 (-0–1)	1329 (1026–1632)	6 (-4–16)
Israel	2032	59 (47–71)	0 (-0–1)	1344 (1030–1658)	5 (-6–17)
Israel	2033	59 (47–71)	0 (-0–1)	1359 (1037–1681)	5 (-8–17)
Israel	2034	59 (47–72)	0 (-0–1)	1374 (1047–1700)	4 (-10–18)
Israel	2035	60 (47–72)	0 (-0–1)	1388 (1059–1717)	4 (-12–19)
Israel	2036	60 (48–73)	0 (-1–1)	1402 (1072–1732)	3 (-14–20)
Italy	2022	811 (790–831)	1 (1–1)	18208 (17611–18805)	14 (14–15)

Italy	2023	800 (767–834)	1 (1–1)	17897 (16969–18826)	14 (13–15)
Italy	2024	790 (744–836)	1 (0–1)	17587 (16345–18829)	14 (13–15)
Italy	2025	780 (721–839)	1 (0–1)	17276 (15718–18834)	13 (12–14)
Italy	2026	770 (697–842)	0 (0–1)	16965 (15085–18846)	13 (11–14)
Italy	2027	759 (673–846)	0 (0–1)	16655 (14440–18869)	12 (11–14)
Italy	2028	749 (648–851)	0 (0–1)	16344 (13785–18903)	12 (10–13)
Italy	2029	739 (622–856)	0 (0–0)	16034 (13118–18949)	11 (10–13)
Italy	2030	729 (596–862)	0 (0–0)	15723 (12438–19007)	11 (9–13)
Italy	2031	718 (569–868)	0 (0–0)	15412 (11747–19077)	10 (8–12)
Italy	2032	708 (541–875)	0 (0–0)	15102 (11044–19159)	10 (8–12)
Italy	2033	698 (513–883)	0 (0–0)	14791 (10330–19252)	9 (7–12)
Italy	2034	688 (484–891)	0 (0–0)	14480 (9604–19357)	9 (7–11)
Italy	2035	677 (455–900)	0 (0–0)	14170 (8866–19473)	9 (6–11)
Italy	2036	667 (425–909)	0 (0–0)	13859 (8118–19600)	8 (6–10)
Jamaica	2022	15 (12–17)	0 (0–1)	376 (314–439)	12 (9–14)
Jamaica	2023	15 (13–17)	0 (0–1)	386 (320–452)	12 (10–15)
Jamaica	2024	15 (12–18)	0 (0–1)	391 (309–473)	12 (9–15)
Jamaica	2025	16 (12–19)	0 (0–1)	399 (311–487)	12 (9–15)
Jamaica	2026	16 (12–19)	0 (0–1)	405 (308–502)	12 (9–15)
Jamaica	2027	16 (12–20)	0 (0–1)	412 (309–516)	12 (9–15)
Jamaica	2028	16 (12–20)	0 (0–1)	419 (308–530)	12 (9–15)
Jamaica	2029	17 (12–21)	0 (0–1)	426 (309–543)	12 (9–15)
Jamaica	2030	17 (12–21)	0 (0–1)	433 (310–556)	12 (9–15)
Jamaica	2031	17 (12–22)	0 (0–1)	440 (311–568)	12 (9–15)
Jamaica	2032	17 (12–22)	0 (0–1)	446 (312–580)	12 (9–15)
Jamaica	2033	18 (13–23)	0 (0–1)	453 (314–593)	12 (9–15)
Jamaica	2034	18 (13–23)	0 (0–1)	460 (316–604)	12 (9–15)
Jamaica	2035	18 (13–23)	0 (0–1)	467 (318–616)	12 (9–15)
Jamaica	2036	18 (13–24)	0 (0–1)	474 (320–628)	12 (9–15)
Japan	2022	2145 (2094–2196)	1 (1–1)	46056 (44763–47350)	16 (16–17)
Japan	2023	2156 (2064–2248)	1 (1–1)	46420 (43528–49312)	16 (15–17)
Japan	2024	2157 (2033–2282)	1 (1–1)	46784 (41945–51623)	15 (14–17)
Japan	2025	2152 (2005–2300)	1 (1–1)	47148 (40064–54232)	15 (13–17)

Japan	2026	2144 (1982–2307)	1 (0–1)	47512 (37920–57103)	15 (13–16)
Japan	2027	2135 (1965–2306)	1 (0–1)	47876 (35538–60213)	14 (12–16)
Japan	2028	2127 (1952–2302)	1 (0–1)	48240 (32937–63542)	14 (12–16)
Japan	2029	2119 (1942–2297)	1 (0–1)	48604 (30131–67076)	13 (11–16)
Japan	2030	2113 (1935–2291)	0 (0–1)	48967 (27134–70801)	13 (10–15)
Japan	2031	2109 (1931–2287)	0 (0–1)	49331 (23955–74708)	12 (10–15)
Japan	2032	2106 (1927–2284)	0 (0–1)	49695 (20603–78788)	12 (9–15)
Japan	2033	2104 (1926–2282)	0 (0–1)	50059 (17086–83032)	12 (9–14)
Japan	2034	2103 (1925–2281)	0 (0–1)	50423 (13411–87435)	11 (8–14)
Japan	2035	2103 (1924–2281)	0 (0–1)	50787 (9583–91991)	11 (8–14)
Japan	2036	2103 (1924–2281)	0 (0–1)	51151 (5609–96693)	10 (7–14)
Jordan	2022	39 (39–40)	1 (0–1)	1193 (1181–1206)	13 (13–13)
Jordan	2023	42 (41–43)	0 (0–1)	1267 (1235–1299)	13 (12–13)
Jordan	2024	44 (42–46)	0 (0–1)	1341 (1283–1399)	13 (12–14)
Jordan	2025	47 (44–50)	0 (0–1)	1415 (1326–1504)	12 (11–14)
Jordan	2026	49 (45–53)	0 (0–1)	1489 (1365–1614)	12 (11–14)
Jordan	2027	52 (46–57)	0 (0–1)	1563 (1399–1727)	12 (10–14)
Jordan	2028	54 (47–60)	0 (0–1)	1638 (1431–1844)	12 (10–14)
Jordan	2029	56 (48–64)	0 (0–1)	1712 (1459–1965)	12 (10–14)
Jordan	2030	59 (49–68)	0 (0–1)	1786 (1484–2088)	12 (10–14)
Jordan	2031	61 (50–72)	0 (0–1)	1860 (1506–2214)	12 (9–14)
Jordan	2032	64 (51–76)	0 (0–1)	1934 (1525–2343)	11 (9–14)
Jordan	2033	66 (52–80)	0 (0–1)	2008 (1542–2474)	11 (9–14)
Jordan	2034	69 (52–85)	0 (0–1)	2082 (1556–2608)	11 (9–14)
Jordan	2035	71 (53–89)	0 (0–1)	2156 (1568–2745)	11 (8–14)
Jordan	2036	73 (53–93)	0 (0–1)	2230 (1578–2883)	11 (8–14)
Kazakhstan	2022	68 (63–72)	0 (0–0)	2096 (1949–2244)	10 (9–11)
Kazakhstan	2023	69 (63–74)	0 (0–0)	2121 (1932–2310)	10 (8–12)
Kazakhstan	2024	69 (63–74)	0 (0–0)	2121 (1932–2310)	10 (7–12)
Kazakhstan	2025	69 (63–74)	0 (0–0)	2121 (1932–2310)	9 (7–12)
Kazakhstan	2026	69 (63–74)	0 (0–0)	2121 (1932–2310)	9 (6–12)
Kazakhstan	2027	69 (63–74)	0 (0–0)	2121 (1932–2310)	9 (6–12)
Kazakhstan	2028	69 (63–74)	0 (0–0)	2121 (1932–2310)	9 (5–12)

Kazakhstan	2029	69 (63–74)	0 (0–0)	2121 (1932–2310)	8 (5–12)
Kazakhstan	2030	69 (63–74)	0 (0–0)	2121 (1932–2310)	8 (5–12)
Kazakhstan	2031	69 (63–74)	0 (0–0)	2121 (1932–2310)	8 (4–12)
Kazakhstan	2032	69 (63–74)	0 (0–0)	2121 (1932–2310)	8 (4–12)
Kazakhstan	2033	69 (63–74)	0 (0–0)	2121 (1932–2310)	8 (3–12)
Kazakhstan	2034	69 (63–74)	0 (0–0)	2121 (1932–2310)	7 (3–12)
Kazakhstan	2035	69 (63–74)	0 (0–0)	2121 (1932–2310)	7 (2–12)
Kazakhstan	2036	69 (63–74)	0 (0–0)	2121 (1932–2310)	7 (2–12)
Kenya	2022	32 (32–33)	0 (0–0)	921 (911–930)	4 (3–4)
Kenya	2023	33 (32–34)	0 (0–0)	941 (920–963)	4 (3–4)
Kenya	2024	34 (32–35)	0 (0–0)	962 (926–999)	4 (3–4)
Kenya	2025	34 (32–36)	0 (0–0)	983 (930–1036)	3 (3–4)
Kenya	2026	35 (32–38)	0 (0–0)	1004 (932–1076)	3 (3–4)
Kenya	2027	35 (32–39)	0 (0–0)	1024 (932–1117)	3 (3–4)
Kenya	2028	36 (31–41)	0 (0–0)	1045 (930–1160)	3 (3–4)
Kenya	2029	37 (31–42)	0 (0–0)	1066 (927–1205)	3 (2–4)
Kenya	2030	37 (31–44)	0 (0–0)	1087 (923–1251)	3 (2–5)
Kenya	2031	38 (30–46)	0 (0–0)	1107 (917–1298)	3 (2–5)
Kenya	2032	39 (30–48)	0 (0–0)	1128 (910–1347)	3 (2–5)
Kenya	2033	39 (29–49)	0 (0–0)	1149 (901–1397)	3 (1–5)
Kenya	2034	40 (28–51)	0 (0–0)	1170 (892–1448)	3 (1–5)
Kenya	2035	40 (28–53)	0 (0–0)	1190 (881–1500)	3 (1–6)
Kenya	2036	41 (27–55)	0 (0–0)	1211 (869–1553)	3 (1–6)
Kiribati	2022	0 (0–0)	1 (1–1)	15 (15–15)	17 (17–18)
Kiribati	2023	0 (0–1)	1 (1–1)	15 (15–16)	17 (17–18)
Kiribati	2024	1 (0–1)	1 (1–1)	16 (15–16)	17 (16–18)
Kiribati	2025	1 (0–1)	1 (1–1)	16 (15–16)	17 (16–18)
Kiribati	2026	1 (1–1)	1 (1–1)	16 (16–16)	17 (15–19)
Kiribati	2027	1 (1–1)	1 (1–1)	16 (16–17)	17 (15–19)
Kiribati	2028	1 (1–1)	1 (1–1)	17 (16–17)	17 (14–20)
Kiribati	2029	1 (1–1)	1 (1–1)	17 (16–17)	17 (14–20)
Kiribati	2030	1 (1–1)	1 (0–1)	17 (16–18)	17 (13–21)
Kiribati	2031	1 (1–1)	1 (0–1)	17 (17–18)	17 (12–21)

Kiribati	2032	1 (1–1)	1 (0–1)	17 (17–18)	17 (12–22)
Kiribati	2033	1 (1–1)	1 (0–1)	18 (17–18)	17 (11–22)
Kiribati	2034	1 (1–1)	1 (0–1)	18 (17–19)	17 (10–23)
Kiribati	2035	1 (1–1)	1 (0–1)	18 (17–19)	16 (9–23)
Kiribati	2036	1 (1–1)	1 (0–1)	18 (18–19)	16 (9–24)
Kuwait	2022	10 (9–11)	0 (0–0)	304 (271–338)	8 (6–10)
Kuwait	2023	10 (9–12)	0 (0–0)	313 (266–359)	8 (5–11)
Kuwait	2024	10 (8–12)	0 (0–0)	321 (263–378)	8 (5–11)
Kuwait	2025	11 (8–13)	0 (0–0)	329 (263–395)	8 (4–12)
Kuwait	2026	11 (8–13)	0 (0–1)	337 (263–411)	8 (4–12)
Kuwait	2027	11 (8–14)	0 (0–1)	345 (264–426)	8 (3–13)
Kuwait	2028	11 (9–14)	0 (0–1)	353 (266–440)	8 (3–13)
Kuwait	2029	12 (9–15)	0 (0–1)	361 (268–455)	8 (3–13)
Kuwait	2030	12 (9–15)	0 (0–1)	369 (270–468)	8 (2–14)
Kuwait	2031	12 (9–16)	0 (0–1)	377 (273–482)	8 (2–14)
Kuwait	2032	12 (9–16)	0 (0–1)	385 (276–495)	8 (2–14)
Kuwait	2033	13 (9–16)	0 (0–1)	393 (279–508)	8 (1–15)
Kuwait	2034	13 (9–17)	0 (0–1)	401 (282–521)	8 (1–15)
Kuwait	2035	13 (9–17)	0 (0–1)	410 (286–533)	8 (1–15)
Kuwait	2036	13 (9–18)	0 (0–1)	418 (290–546)	8 (1–15)
Kyrgyzstan	2022	16 (15–17)	0 (0–0)	516 (493–538)	9 (8–10)
Kyrgyzstan	2023	17 (15–18)	0 (0–0)	528 (491–564)	9 (8–10)
Kyrgyzstan	2024	17 (15–19)	0 (0–0)	539 (488–590)	9 (8–10)
Kyrgyzstan	2025	17 (15–19)	0 (0–0)	551 (485–617)	9 (7–10)
Kyrgyzstan	2026	17 (15–20)	0 (0–0)	563 (481–645)	9 (7–10)
Kyrgyzstan	2027	18 (15–21)	0 (0–0)	575 (476–673)	8 (7–10)
Kyrgyzstan	2028	18 (14–22)	0 (0–0)	586 (470–703)	8 (7–10)
Kyrgyzstan	2029	18 (14–23)	0 (0–0)	598 (464–733)	8 (7–10)
Kyrgyzstan	2030	19 (14–24)	0 (0–0)	610 (456–764)	8 (6–10)
Kyrgyzstan	2031	19 (14–25)	0 (0–0)	622 (448–795)	8 (6–10)
Kyrgyzstan	2032	19 (13–25)	0 (0–0)	633 (439–828)	8 (6–10)
Kyrgyzstan	2033	20 (13–26)	0 (0–0)	645 (430–861)	8 (6–10)
Kyrgyzstan	2034	20 (13–27)	0 (0–0)	657 (419–895)	8 (6–10)

Kyrgyzstan	2035	20 (12–28)	0 (0–0)	669 (408–929)	8 (5–10)
Kyrgyzstan	2036	21 (12–30)	0 (0–0)	681 (397–964)	7 (5–10)
Lao People's Democratic Republic	2022	35 (35–35)	1 (1–1)	994 (988–1000)	19 (19–19)
Lao People's Democratic Republic	2023	36 (36–36)	1 (1–1)	1022 (1010–1033)	19 (19–19)
Lao People's Democratic Republic	2024	37 (36–38)	1 (1–1)	1049 (1031–1067)	19 (19–19)
Lao People's Democratic Republic	2025	38 (37–39)	1 (1–1)	1077 (1051–1103)	19 (19–19)
Lao People's Democratic Republic	2026	39 (37–40)	1 (1–1)	1105 (1070–1139)	19 (19–19)
Lao People's Democratic Republic	2027	40 (38–42)	1 (1–1)	1132 (1088–1176)	19 (19–20)
Lao People's Democratic Republic	2028	41 (38–43)	1 (1–1)	1160 (1106–1214)	19 (19–20)
Lao People's Democratic Republic	2029	42 (39–44)	1 (1–1)	1188 (1123–1252)	19 (19–20)
Lao People's Democratic Republic	2030	42 (39–46)	1 (1–1)	1215 (1139–1292)	19 (19–20)
Lao People's Democratic Republic	2031	43 (39–47)	1 (1–1)	1243 (1155–1331)	19 (19–20)
Lao People's Democratic Republic	2032	44 (40–49)	1 (1–1)	1271 (1170–1372)	19 (19–20)
Lao People's Democratic Republic	2033	45 (40–50)	1 (1–1)	1298 (1184–1413)	19 (19–20)
Lao People's Democratic Republic	2034	46 (40–52)	1 (1–1)	1326 (1198–1454)	19 (19–20)
Lao People's Democratic Republic	2035	47 (41–53)	1 (1–1)	1354 (1212–1496)	19 (19–20)
Lao People's Democratic Republic	2036	48 (41–55)	1 (1–1)	1381 (1224–1538)	19 (19–20)
Latvia	2022	29 (27–31)	1 (1–1)	718 (657–780)	21 (20–23)
Latvia	2023	29 (27–32)	1 (1–1)	718 (631–805)	21 (19–24)
Latvia	2024	30 (27–32)	1 (1–1)	718 (612–825)	21 (18–24)
Latvia	2025	30 (27–33)	1 (1–1)	718 (595–841)	21 (18–25)
Latvia	2026	30 (27–33)	1 (1–1)	718 (581–856)	21 (17–25)
Latvia	2027	30 (27–33)	1 (1–1)	718 (568–869)	21 (17–26)
Latvia	2028	30 (27–34)	1 (1–1)	718 (556–881)	21 (17–26)
Latvia	2029	30 (27–34)	1 (1–1)	718 (544–892)	21 (16–26)
Latvia	2030	31 (27–34)	1 (1–1)	718 (534–903)	21 (16–27)
Latvia	2031	31 (27–34)	1 (1–1)	718 (524–913)	21 (16–27)
Latvia	2032	31 (27–34)	1 (1–1)	718 (514–922)	21 (15–27)
Latvia	2033	31 (27–34)	1 (1–1)	718 (505–931)	21 (15–27)
Latvia	2034	31 (27–34)	1 (1–1)	718 (497–940)	21 (15–28)
Latvia	2035	31 (27–34)	1 (1–1)	718 (488–948)	21 (15–28)
Latvia	2036	31 (27–34)	1 (1–1)	718 (480–956)	21 (14–28)
Lebanon	2022	43 (42–44)	1 (1–1)	983 (963–1004)	17 (16–17)

Lebanon	2023	43 (41–45)	1 (1–1)	992 (948–1036)	17 (16–18)
Lebanon	2024	43 (40–46)	1 (1–1)	1000 (930–1070)	17 (16–19)
Lebanon	2025	43 (39–47)	1 (1–1)	1006 (908–1104)	17 (15–19)
Lebanon	2026	43 (38–49)	1 (1–1)	1012 (885–1139)	18 (15–20)
Lebanon	2027	43 (37–50)	1 (1–1)	1018 (861–1174)	18 (16–21)
Lebanon	2028	43 (35–51)	1 (1–1)	1022 (836–1209)	18 (16–21)
Lebanon	2029	43 (34–53)	1 (0–1)	1026 (810–1243)	19 (16–21)
Lebanon	2030	44 (33–54)	1 (0–1)	1030 (784–1276)	19 (16–22)
Lebanon	2031	44 (31–56)	1 (0–1)	1033 (758–1308)	19 (16–22)
Lebanon	2032	44 (30–57)	1 (0–1)	1036 (732–1340)	19 (16–22)
Lebanon	2033	44 (29–59)	1 (0–1)	1038 (706–1370)	19 (16–22)
Lebanon	2034	44 (27–60)	1 (0–1)	1040 (680–1400)	19 (16–22)
Lebanon	2035	44 (26–61)	1 (0–1)	1042 (655–1429)	19 (16–22)
Lebanon	2036	44 (25–63)	1 (0–1)	1044 (630–1458)	19 (16–22)
Lesotho	2022	5 (5–5)	0 (0–0)	154 (151–158)	13 (13–13)
Lesotho	2023	5 (5–5)	0 (0–0)	153 (144–161)	13 (12–13)
Lesotho	2024	5 (4–5)	0 (0–0)	153 (139–167)	13 (11–14)
Lesotho	2025	5 (4–6)	0 (0–0)	155 (136–174)	12 (11–14)
Lesotho	2026	5 (4–6)	0 (0–0)	158 (135–182)	12 (10–14)
Lesotho	2027	5 (3–6)	0 (0–1)	162 (135–190)	12 (9–15)
Lesotho	2028	5 (3–6)	0 (0–1)	166 (135–197)	12 (9–15)
Lesotho	2029	4 (3–6)	0 (0–1)	170 (136–204)	12 (8–15)
Lesotho	2030	4 (3–6)	0 (0–1)	173 (137–209)	12 (8–16)
Lesotho	2031	4 (2–6)	0 (0–1)	177 (139–215)	12 (7–16)
Lesotho	2032	4 (2–7)	0 (0–1)	180 (140–220)	12 (7–17)
Lesotho	2033	4 (2–7)	0 (0–1)	183 (141–225)	12 (6–17)
Lesotho	2034	4 (2–7)	0 (0–1)	186 (142–230)	12 (6–17)
Lesotho	2035	4 (1–7)	0 (0–1)	189 (143–235)	11 (5–18)
Lesotho	2036	4 (1–7)	0 (0–1)	192 (145–240)	11 (5–18)
Liberia	2022	2 (2–2)	0 (0–0)	49 (48–49)	2 (2–2)
Liberia	2023	2 (2–2)	0 (0–0)	50 (49–52)	2 (2–2)
Liberia	2024	2 (2–2)	0 (0–0)	52 (49–55)	2 (2–2)
Liberia	2025	2 (2–2)	0 (0–0)	54 (50–58)	2 (2–2)

Liberia	2026	2 (2–2)	0 (0–0)	56 (51–60)	2 (2–2)
Liberia	2027	2 (2–2)	0 (0–0)	57 (51–64)	2 (2–2)
Liberia	2028	2 (2–2)	0 (0–0)	59 (51–67)	2 (2–2)
Liberia	2029	2 (2–2)	0 (0–0)	61 (51–70)	2 (2–2)
Liberia	2030	2 (2–2)	0 (0–0)	62 (52–73)	2 (2–2)
Liberia	2031	2 (2–2)	0 (0–0)	64 (52–77)	2 (2–2)
Liberia	2032	2 (2–3)	0 (0–0)	66 (52–80)	2 (2–2)
Liberia	2033	2 (2–3)	0 (0–0)	68 (51–84)	2 (2–2)
Liberia	2034	2 (2–3)	0 (0–0)	69 (51–88)	2 (2–2)
Liberia	2035	2 (2–3)	0 (0–0)	71 (51–91)	2 (2–2)
Liberia	2036	2 (2–3)	0 (0–0)	73 (51–95)	2 (2–2)
Libya	2022	28 (28–29)	1 (0–1)	850 (835–866)	13 (13–14)
Libya	2023	29 (28–30)	1 (0–1)	876 (842–910)	13 (12–14)
Libya	2024	30 (27–32)	1 (0–1)	902 (846–959)	13 (12–15)
Libya	2025	31 (27–34)	1 (0–1)	928 (845–1011)	13 (12–15)
Libya	2026	31 (27–36)	1 (0–1)	954 (842–1067)	13 (11–15)
Libya	2027	32 (26–38)	1 (0–1)	980 (835–1125)	13 (11–15)
Libya	2028	33 (25–40)	1 (0–1)	1006 (827–1186)	13 (11–16)
Libya	2029	34 (25–43)	1 (0–1)	1032 (815–1249)	13 (11–16)
Libya	2030	34 (24–45)	1 (0–1)	1058 (802–1314)	13 (11–16)
Libya	2031	35 (23–48)	1 (0–1)	1084 (786–1382)	13 (10–16)
Libya	2032	36 (22–50)	1 (0–1)	1110 (769–1452)	13 (10–16)
Libya	2033	37 (21–53)	1 (0–1)	1136 (749–1523)	13 (10–17)
Libya	2034	38 (19–56)	1 (0–1)	1162 (728–1597)	13 (10–17)
Libya	2035	38 (18–59)	1 (0–1)	1188 (704–1672)	13 (10–17)
Libya	2036	39 (17–62)	1 (0–1)	1214 (679–1749)	13 (10–17)
Lithuania	2022	40 (38–43)	1 (1–1)	980 (915–1045)	20 (18–21)
Lithuania	2023	40 (36–44)	1 (1–1)	980 (888–1072)	20 (18–21)
Lithuania	2024	40 (35–45)	1 (1–1)	980 (867–1093)	20 (18–22)
Lithuania	2025	39 (33–46)	1 (1–1)	980 (850–1110)	20 (18–22)
Lithuania	2026	39 (32–46)	1 (1–1)	980 (835–1125)	20 (18–22)
Lithuania	2027	39 (30–47)	1 (1–1)	980 (821–1139)	20 (18–23)
Lithuania	2028	38 (29–48)	1 (1–1)	980 (808–1152)	20 (18–23)

Lithuania	2029	38 (27–49)	1 (1–1)	980 (796–1164)	20 (18–23)
Lithuania	2030	38 (25–50)	1 (1–1)	980 (785–1175)	20 (18–23)
Lithuania	2031	37 (24–51)	1 (1–1)	980 (774–1185)	21 (18–23)
Lithuania	2032	37 (22–52)	1 (1–1)	980 (764–1195)	21 (18–23)
Lithuania	2033	37 (20–53)	1 (1–1)	980 (755–1205)	21 (18–23)
Lithuania	2034	36 (18–54)	1 (1–1)	980 (746–1214)	21 (18–23)
Lithuania	2035	36 (16–56)	1 (1–1)	980 (737–1223)	21 (18–23)
Lithuania	2036	36 (15–57)	1 (1–1)	980 (728–1232)	21 (18–23)
Low SDI	2022	706 (699–714)	0 (0–0)	19495 (19305–19684)	4 (3–4)
Low SDI	2023	720 (706–735)	0 (0–0)	19893 (19469–20317)	4 (3–4)
Low SDI	2024	734 (713–756)	0 (0–0)	20291 (19583–21000)	3 (3–4)
Low SDI	2025	748 (719–778)	0 (0–0)	20690 (19652–21727)	3 (3–4)
Low SDI	2026	763 (724–802)	0 (0–0)	21088 (19683–22493)	3 (3–4)
Low SDI	2027	777 (728–825)	0 (0–0)	21486 (19679–23293)	3 (3–4)
Low SDI	2028	791 (731–850)	0 (0–0)	21885 (19643–24126)	3 (3–4)
Low SDI	2029	805 (734–875)	0 (0–0)	22283 (19577–24989)	3 (3–4)
Low SDI	2030	819 (736–901)	0 (0–0)	22681 (19483–25879)	3 (3–4)
Low SDI	2031	833 (738–928)	0 (0–0)	23080 (19363–26796)	3 (3–4)
Low SDI	2032	847 (739–955)	0 (0–0)	23478 (19217–27739)	3 (3–4)
Low SDI	2033	861 (739–982)	0 (0–0)	23876 (19047–28706)	3 (3–4)
Low SDI	2034	875 (739–1010)	0 (0–0)	24275 (18854–29696)	3 (3–4)
Low SDI	2035	889 (738–1039)	0 (0–0)	24673 (18638–30708)	3 (3–4)
Low SDI	2036	903 (737–1068)	0 (0–0)	25071 (18401–31742)	3 (2–4)
Low-middle SDI	2022	3531 (3498–3565)	0 (0–0)	97118 (96316–97921)	6 (6–6)
Low-middle SDI	2023	3581 (3506–3657)	0 (0–0)	98645 (96850–100440)	6 (6–6)
Low-middle SDI	2024	3631 (3505–3758)	0 (0–0)	100172 (97169–103175	6 (6–6)
Low-middle SDI	2025	3681 (3497–3866)	0 (0–0)	101699 (97303–106095	6 (6–6)
Low-middle SDI	2026	3731 (3481–3981)	0 (0–0)	103226 (97274–109178	6 (6–6)
Low-middle SDI	2027	3781 (3460–4103)	0 (0–0)	104753 (97097–112409	6 (6–6)
Low-middle SDI	2028	3831 (3432–4230)	0 (0–0)	106280 (96783–115776	6 (6–6)
Low-middle SDI	2029	3881 (3400–4363)	0 (0–0)	107806 (96343–119270	6 (6–6)
Low-middle SDI	2030	3931 (3362–4500)	0 (0–0)	109333 (95784–122882	6 (6–6)
Low-middle SDI	2031	3981 (3320–4643)	0 (0–0)	110860 (95113–126608	6 (6–6)

Low-middle SDI	2032	4031 (3273–4789)	0 (0–0)	112387 (94334–130441	6 (6–6)
Low-middle SDI	2033	4081 (3222–4941)	0 (0–0)	113914 (93452–134376	6 (6–6)
Low-middle SDI	2034	4131 (3166–5096)	0 (0–0)	115441 (92473–138409	6 (6–6)
Low-middle SDI	2035	4181 (3107–5255)	0 (0–0)	116968 (91399–142537	6 (6–6)
Low-middle SDI	2036	4231 (3044–5418)	0 (0–0)	118495 (90233–146756	6 (6–6)
Luxembourg	2022	6 (6–7)	1 (1–1)	150 (142–158)	14 (13–15)
Luxembourg	2023	6 (6–7)	1 (0–1)	149 (139–159)	13 (12–15)
Luxembourg	2024	6 (6–7)	1 (0–1)	148 (136–159)	13 (11–14)
Luxembourg	2025	6 (6–7)	1 (0–1)	146 (134–159)	12 (10–14)
Luxembourg	2026	6 (6–7)	0 (0–1)	145 (132–159)	11 (9–13)
Luxembourg	2027	6 (6–7)	0 (0–1)	144 (129–159)	11 (8–13)
Luxembourg	2028	6 (6–7)	0 (0–1)	143 (127–158)	10 (8–12)
Luxembourg	2029	6 (5–7)	0 (0–1)	141 (125–158)	9 (7–12)
Luxembourg	2030	6 (5–7)	0 (0–1)	140 (123–158)	9 (6–11)
Luxembourg	2031	6 (5–8)	0 (0–1)	139 (121–157)	8 (5–11)
Luxembourg	2032	6 (5–8)	0 (0–0)	138 (119–157)	7 (4–10)
Luxembourg	2033	6 (5–8)	0 (0–0)	137 (117–156)	7 (3–10)
Luxembourg	2034	6 (5–8)	0 (0–0)	135 (115–156)	6 (3–9)
Luxembourg	2035	6 (5–8)	0 (0–0)	134 (113–155)	5 (2–8)
Luxembourg	2036	6 (5–8)	0 (0–0)	133 (111–155)	4 (1–8)
Madagascar	2022	13 (13–13)	0 (0–0)	386 (377–395)	3 (3–3)
Madagascar	2023	13 (13–14)	0 (0–0)	396 (380–411)	3 (3–3)
Madagascar	2024	14 (13–15)	0 (0–0)	405 (383–428)	3 (3–3)
Madagascar	2025	14 (13–15)	0 (0–0)	415 (386–445)	3 (3–3)
Madagascar	2026	14 (13–16)	0 (0–0)	425 (387–463)	3 (2–3)
Madagascar	2027	15 (13–16)	0 (0–0)	435 (388–481)	3 (2–4)
Madagascar	2028	15 (13–17)	0 (0–0)	444 (389–500)	3 (2–4)
Madagascar	2029	15 (13–18)	0 (0–0)	454 (389–519)	3 (2–4)
Madagascar	2030	16 (13–18)	0 (0–0)	464 (388–539)	3 (2–4)
Madagascar	2031	16 (13–19)	0 (0–0)	473 (388–559)	3 (2–4)
Madagascar	2032	16 (13–20)	0 (0–0)	483 (386–580)	3 (2–4)
Madagascar	2033	17 (13–21)	0 (0–0)	493 (384–601)	3 (2–4)
Madagascar	2034	17 (13–21)	0 (0–0)	503 (382–623)	3 (1–4)

Madagascar	2035	17 (12–22)	0 (0–0)	512 (380–645)	3 (1–4)
Madagascar	2036	18 (12–23)	0 (0–0)	522 (377–668)	3 (1–5)
Malawi	2022	10 (10–11)	0 (0–0)	283 (280–286)	4 (3–4)
Malawi	2023	11 (10–11)	0 (0–0)	287 (281–294)	4 (3–4)
Malawi	2024	11 (10–11)	0 (0–0)	292 (282–301)	3 (3–4)
Malawi	2025	11 (10–11)	0 (0–0)	296 (284–309)	3 (3–4)
Malawi	2026	11 (10–12)	0 (0–0)	301 (286–316)	3 (3–4)
Malawi	2027	11 (10–12)	0 (0–0)	306 (288–324)	3 (3–4)
Malawi	2028	11 (11–12)	0 (0–0)	311 (290–331)	3 (2–4)
Malawi	2029	12 (11–12)	0 (0–0)	316 (293–338)	3 (2–4)
Malawi	2030	12 (11–13)	0 (0–0)	321 (296–345)	3 (2–4)
Malawi	2031	12 (11–13)	0 (0–0)	326 (299–352)	3 (2–4)
Malawi	2032	12 (11–13)	0 (0–0)	331 (302–359)	3 (2–5)
Malawi	2033	12 (11–13)	0 (0–0)	336 (305–366)	3 (1–5)
Malawi	2034	12 (11–14)	0 (0–0)	341 (308–373)	3 (1–5)
Malawi	2035	13 (11–14)	0 (0–0)	346 (312–379)	3 (1–5)
Malawi	2036	13 (11–14)	0 (0–0)	351 (315–386)	3 (0–5)
Malaysia	2022	183 (172–194)	1 (1–1)	5062 (4822–5302)	17 (16–18)
Malaysia	2023	191 (179–203)	1 (1–1)	5162 (4823–5501)	17 (15–19)
Malaysia	2024	193 (178–207)	1 (1–1)	5262 (4846–5678)	17 (15–19)
Malaysia	2025	197 (181–213)	1 (1–1)	5362 (4882–5842)	17 (14–19)
Malaysia	2026	201 (183–218)	1 (1–1)	5462 (4926–5999)	17 (14–20)
Malaysia	2027	204 (185–224)	1 (1–1)	5562 (4975–6150)	17 (14–20)
Malaysia	2028	208 (187–229)	1 (1–1)	5662 (5028–6297)	17 (13–20)
Malaysia	2029	212 (190–234)	1 (1–1)	5762 (5084–6441)	17 (13–20)
Malaysia	2030	215 (192–239)	1 (1–1)	5863 (5143–6582)	17 (13–21)
Malaysia	2031	219 (195–243)	1 (0–1)	5963 (5204–6721)	17 (13–21)
Malaysia	2032	223 (198–248)	1 (0–1)	6063 (5267–6858)	17 (13–21)
Malaysia	2033	226 (200–253)	1 (0–1)	6163 (5332–6994)	17 (12–21)
Malaysia	2034	230 (203–258)	1 (0–1)	6263 (5398–7128)	17 (12–21)
Malaysia	2035	234 (206–262)	1 (0–1)	6363 (5465–7261)	17 (12–22)
Malaysia	2036	238 (208–267)	1 (0–1)	6463 (5534–7392)	17 (12–22)
Maldives	2022	1 (1–1)	0 (0–0)	21 (20–22)	6 (5–6)

Maldives	2023	1 (1–1)	0 (0–0)	22 (21–23)	6 (5–6)
Maldives	2024	1 (1–1)	0 (0–0)	23 (22–24)	6 (5–7)
Maldives	2025	1 (1–1)	0 (0–0)	24 (22–26)	6 (4–7)
Maldives	2026	1 (1–1)	0 (0–0)	25 (22–27)	6 (4–7)
Maldives	2027	1 (1–1)	0 (0–0)	26 (23–29)	6 (4–8)
Maldives	2028	1 (1–1)	0 (0–0)	27 (23–30)	6 (3–8)
Maldives	2029	1 (1–1)	0 (0–0)	28 (23–32)	6 (3–9)
Maldives	2030	1 (1–1)	0 (0–0)	28 (23–34)	6 (2–9)
Maldives	2031	1 (1–1)	0 (0–0)	29 (23–35)	6 (2–10)
Maldives	2032	1 (1–1)	0 (0–0)	30 (23–37)	6 (1–10)
Maldives	2033	1 (1–1)	0 (0–0)	31 (23–39)	6 (1–11)
Maldives	2034	1 (1–2)	0 (0–0)	32 (23–41)	6 (0–11)
Maldives	2035	1 (1–2)	0 (0–0)	33 (23–42)	6 (-0–12)
Maldives	2036	1 (1–2)	0 (0–0)	34 (23–44)	6 (-1–12)
Mali	2022	13 (13–14)	0 (0–0)	353 (350–356)	4 (4–4)
Mali	2023	14 (13–14)	0 (0–0)	359 (353–365)	4 (4–4)
Mali	2024	14 (13–14)	0 (0–0)	365 (355–376)	4 (4–4)
Mali	2025	14 (13–14)	0 (0–0)	371 (356–386)	4 (4–4)
Mali	2026	14 (13–15)	0 (0–0)	377 (357–398)	4 (4–4)
Mali	2027	14 (12–15)	0 (0–0)	383 (357–410)	4 (3–4)
Mali	2028	14 (12–16)	0 (0–0)	389 (357–422)	4 (3–4)
Mali	2029	14 (12–16)	0 (0–0)	396 (356–435)	4 (3–4)
Mali	2030	14 (12–16)	0 (0–0)	402 (355–448)	4 (3–4)
Mali	2031	14 (11–17)	0 (0–0)	408 (353–462)	4 (3–4)
Mali	2032	14 (11–17)	0 (0–0)	414 (352–476)	4 (3–4)
Mali	2033	14 (11–18)	0 (0–0)	420 (349–490)	4 (3–4)
Mali	2034	14 (10–18)	0 (0–0)	426 (347–505)	4 (3–5)
Mali	2035	14 (10–19)	0 (0–0)	432 (344–520)	4 (3–5)
Mali	2036	14 (9–20)	0 (0–0)	438 (341–535)	4 (3–5)
Malta	2022	4 (4–4)	0 (0–0)	102 (96–108)	12 (11–13)
Malta	2023	4 (4–5)	0 (0–0)	102 (94–110)	12 (10–13)
Malta	2024	4 (4–5)	0 (0–0)	102 (92–112)	11 (10–13)
Malta	2025	4 (4–5)	0 (0–0)	102 (90–113)	11 (9–13)

Malta	2026	4 (4–5)	0 (0–0)	102 (89–115)	11 (8–13)
Malta	2027	4 (4–5)	0 (0–0)	102 (88–116)	10 (8–13)
Malta	2028	4 (4–5)	0 (0–0)	102 (86–117)	10 (7–13)
Malta	2029	4 (4–5)	0 (0–0)	102 (85–118)	10 (7–13)
Malta	2030	4 (4–5)	0 (0–0)	102 (84–119)	9 (6–13)
Malta	2031	4 (3–5)	0 (0–0)	102 (83–120)	9 (6–12)
Malta	2032	4 (3–5)	0 (0–0)	102 (82–121)	9 (5–12)
Malta	2033	4 (3–5)	0 (0–0)	102 (82–122)	8 (5–12)
Malta	2034	4 (3–5)	0 (0–0)	102 (81–123)	8 (4–12)
Malta	2035	4 (3–5)	0 (0–0)	102 (80–124)	8 (4–12)
Malta	2036	4 (3–5)	0 (0–0)	102 (79–124)	8 (4–12)
Marshall Islands	2022	0 (0–0)	0 (0–0)	5 (5–5)	11 (10–11)
Marshall Islands	2023	0 (0–0)	0 (0–0)	5 (5–5)	11 (10–11)
Marshall Islands	2024	0 (0–0)	0 (0–0)	5 (5–5)	11 (10–11)
Marshall Islands	2025	0 (0–0)	0 (0–0)	5 (5–5)	11 (10–11)
Marshall Islands	2026	0 (0–0)	0 (0–0)	5 (5–5)	11 (10–11)
Marshall Islands	2027	0 (0–0)	0 (0–0)	5 (5–5)	11 (10–11)
Marshall Islands	2028	0 (0–0)	0 (0–0)	5 (5–5)	11 (10–11)
Marshall Islands	2029	0 (0–0)	0 (0–0)	5 (5–6)	11 (10–11)
Marshall Islands	2030	0 (0–0)	0 (0–0)	5 (5–6)	11 (10–11)
Marshall Islands	2031	0 (0–0)	0 (0–0)	5 (5–6)	11 (10–11)
Marshall Islands	2032	0 (0–0)	0 (0–0)	6 (5–6)	11 (10–11)
Marshall Islands	2033	0 (0–0)	0 (0–0)	6 (5–6)	11 (10–11)
Marshall Islands	2034	0 (0–0)	0 (0–0)	6 (5–6)	11 (10–11)
Marshall Islands	2035	0 (0–0)	0 (0–0)	6 (5–6)	11 (10–12)
Marshall Islands	2036	0 (0–0)	0 (0–0)	6 (5–7)	11 (10–12)
Mauritania	2022	2 (2–2)	0 (0–0)	65 (64–67)	3 (3–3)
Mauritania	2023	3 (2–3)	0 (0–0)	68 (66–71)	3 (3–3)
Mauritania	2024	3 (2–3)	0 (0–0)	71 (67–76)	3 (3–3)
Mauritania	2025	3 (2–3)	0 (0–0)	74 (68–80)	3 (3–3)
Mauritania	2026	3 (3–3)	0 (0–0)	77 (69–85)	3 (3–3)
Mauritania	2027	3 (3–3)	0 (0–0)	80 (69–90)	3 (3–4)
Mauritania	2028	3 (3–3)	0 (0–0)	82 (70–95)	3 (3–4)

Mauritania	2029	3 (3–4)	0 (0–0)	85 (70–100)	3 (2–4)
Mauritania	2030	3 (3–4)	0 (0–0)	88 (70–106)	3 (2–4)
Mauritania	2031	3 (3–4)	0 (0–0)	91 (70–111)	3 (2–4)
Mauritania	2032	3 (3–4)	0 (0–0)	94 (70–117)	3 (2–4)
Mauritania	2033	3 (3–4)	0 (0–0)	96 (70–123)	3 (2–5)
Mauritania	2034	4 (3–5)	0 (0–0)	99 (69–129)	3 (2–5)
Mauritania	2035	4 (3–5)	0 (0–0)	102 (69–135)	3 (2–5)
Mauritania	2036	4 (3–5)	0 (0–0)	105 (68–141)	4 (2–5)
Mauritius	2022	13 (12–13)	1 (1–1)	332 (314–350)	17 (16–18)
Mauritius	2023	13 (12–14)	1 (1–1)	347 (319–376)	17 (15–19)
Mauritius	2024	14 (12–15)	1 (1–1)	363 (323–402)	18 (15–20)
Mauritius	2025	15 (13–16)	1 (1–1)	378 (328–428)	18 (15–21)
Mauritius	2026	15 (13–17)	1 (1–1)	393 (331–455)	18 (15–21)
Mauritius	2027	16 (13–19)	1 (1–1)	408 (335–482)	18 (15–22)
Mauritius	2028	16 (13–20)	1 (1–1)	424 (337–510)	18 (15–22)
Mauritius	2029	17 (13–21)	1 (1–1)	439 (340–538)	19 (15–23)
Mauritius	2030	18 (13–22)	1 (1–1)	454 (341–567)	19 (15–23)
Mauritius	2031	18 (14–23)	1 (1–1)	469 (342–596)	19 (15–24)
Mauritius	2032	19 (14–24)	1 (1–1)	485 (343–626)	19 (15–24)
Mauritius	2033	20 (14–26)	1 (1–1)	500 (343–656)	20 (15–25)
Mauritius	2034	20 (14–27)	1 (1–1)	515 (343–687)	20 (15–25)
Mauritius	2035	21 (14–28)	1 (1–1)	530 (342–718)	20 (15–25)
Mauritius	2036	22 (14–29)	1 (1–1)	546 (341–750)	20 (15–26)
Mexico	2022	348 (338–357)	0 (0–0)	9215 (8958–9471)	7 (7–7)
Mexico	2023	357 (343–372)	0 (0–0)	9481 (9078–9884)	7 (6–7)
Mexico	2024	367 (347–386)	0 (0–0)	9747 (9203–10291)	7 (6–7)
Mexico	2025	376 (352–401)	0 (0–0)	10013 (9326–10700)	7 (6–7)
Mexico	2026	386 (356–416)	0 (0–0)	10279 (9444–11114)	7 (6–7)
Mexico	2027	396 (361–430)	0 (0–0)	10545 (9557–11533)	7 (6–7)
Mexico	2028	405 (365–445)	0 (0–0)	10811 (9664–11959)	7 (6–7)
Mexico	2029	415 (369–461)	0 (0–0)	11077 (9764–12390)	7 (6–8)
Mexico	2030	424 (372–476)	0 (0–0)	11343 (9859–12827)	7 (6–8)
Mexico	2031	434 (376–492)	0 (0–0)	11609 (9948–13271)	7 (6–8)

Mexico	2032	443 (379–508)	0 (0–0)	11875 (10031–13720)	7 (6–8)
Mexico	2033	453 (382–524)	0 (0–0)	12141 (10109–14174)	7 (6–8)
Mexico	2034	462 (385–540)	0 (0–0)	12407 (10180–14635)	7 (6–8)
Mexico	2035	472 (388–556)	0 (0–0)	12674 (10247–15101)	7 (6–8)
Mexico	2036	482 (390–573)	0 (0–0)	12940 (10307–15572)	7 (6–8)
Micronesia (Federated States of)	2022	1 (1–1)	1 (1–1)	16 (16–16)	18 (18–18)
Micronesia (Federated States of)	2023	1 (1–1)	1 (1–1)	17 (16–17)	18 (18–18)
Micronesia (Federated States of)	2024	1 (1–1)	1 (1–1)	17 (17–17)	18 (18–18)
Micronesia (Federated States of)	2025	1 (1–1)	1 (1–1)	17 (17–17)	18 (18–18)
Micronesia (Federated States of)	2026	1 (1–1)	1 (1–1)	17 (17–18)	18 (18–19)
Micronesia (Federated States of)	2027	1 (1–1)	1 (1–1)	18 (17–18)	18 (18–19)
Micronesia (Federated States of)	2028	1 (1–1)	1 (1–1)	18 (17–19)	18 (18–19)
Micronesia (Federated States of)	2029	1 (1–1)	1 (1–1)	18 (17–19)	18 (17–19)
Micronesia (Federated States of)	2030	1 (1–1)	1 (1–1)	18 (17–19)	18 (17–19)
Micronesia (Federated States of)	2031	1 (1–1)	1 (1–1)	18 (17–20)	18 (17–19)
Micronesia (Federated States of)	2032	1 (1–1)	1 (1–1)	19 (17–20)	18 (17–19)
Micronesia (Federated States of)	2033	1 (1–1)	1 (1–1)	19 (17–21)	18 (17–19)
Micronesia (Federated States of)	2034	1 (1–1)	1 (1–1)	19 (17–21)	18 (17–19)
Micronesia (Federated States of)	2035	1 (1–1)	1 (1–1)	19 (17–21)	18 (17–19)
Micronesia (Federated States of)	2036	1 (1–1)	1 (1–1)	20 (17–22)	18 (17–19)
Middle SDI	2022	1799 (14640–1495	1 (1–1)	99531 (395536–403525	14 (14–14)
Middle SDI	2023	5158 (14802–1551	1 (1–1)	08103 (399171–417035	14 (13–14)
Middle SDI	2024	5516 (14922–1611	1 (1–1)	16676 (401730–431622	14 (13–14)
Middle SDI	2025	5874 (15004–1674	1 (1–1)	25248 (403369–447127	14 (13–14)
Middle SDI	2026	5232 (15055–1741	1 (0–1)	33821 (404197–463445	13 (13–14)
Middle SDI	2027	5591 (15076–1810	1 (0–1)	42393 (404288–480498	13 (13–14)
Middle SDI	2028	5949 (15070–1882	1 (0–1)	50966 (403702–498229	13 (13–14)
Middle SDI	2029	7307 (15039–1957	1 (0–1)	59538 (402485–516591	13 (12–14)
Middle SDI	2030	7666 (14985–2034	1 (0–1)	68111 (400676–535546	13 (12–14)
Middle SDI	2031	3024 (14908–2114	1 (0–1)	76683 (398305–555061	13 (12–14)
Middle SDI	2032	3382 (14810–2195	1 (0–1)	85256 (395401–575110	13 (12–14)
Middle SDI	2033	3741 (14692–2278	1 (0–1)	93828 (391988–595668	13 (12–14)
Middle SDI	2034	9099 (14554–2364	1 (0–1)	02401 (388085–616716	13 (12–14)

Middle SDI	2035	457 (14398–2451	1 (0–1)	10973 (383712–63823	13 (12–14)
Middle SDI	2036	816 (14223–2540	1 (0–1)	19546 (378885–66020	13 (12–14)
Monaco	2022	1 (1–1)	1 (1–1)	24 (24–24)	29 (29–29)
Monaco	2023	1 (1–1)	1 (1–1)	24 (24–24)	29 (29–30)
Monaco	2024	1 (1–1)	1 (1–1)	24 (24–25)	29 (28–30)
Monaco	2025	1 (1–1)	1 (1–1)	24 (23–25)	29 (28–30)
Monaco	2026	1 (1–1)	1 (1–1)	25 (23–26)	29 (27–31)
Monaco	2027	1 (1–1)	1 (1–1)	25 (23–26)	29 (26–32)
Monaco	2028	1 (1–1)	1 (1–1)	25 (23–27)	29 (26–32)
Monaco	2029	1 (1–1)	1 (1–1)	25 (23–28)	29 (25–33)
Monaco	2030	1 (1–1)	1 (1–1)	25 (22–28)	29 (24–33)
Monaco	2031	1 (1–1)	1 (1–1)	25 (22–29)	29 (24–34)
Monaco	2032	1 (1–1)	1 (1–1)	26 (22–30)	29 (23–35)
Monaco	2033	1 (1–1)	1 (1–1)	26 (21–30)	29 (22–36)
Monaco	2034	1 (1–1)	1 (1–1)	26 (21–31)	29 (21–36)
Monaco	2035	1 (1–1)	1 (1–1)	26 (20–32)	29 (20–37)
Monaco	2036	1 (1–1)	1 (1–1)	26 (20–32)	29 (19–38)
Mongolia	2022	9 (9–10)	0 (0–0)	294 (282–305)	11 (10–11)
Mongolia	2023	10 (9–10)	0 (0–0)	298 (278–317)	11 (10–11)
Mongolia	2024	10 (9–11)	0 (0–0)	301 (273–330)	11 (10–11)
Mongolia	2025	10 (9–11)	0 (0–0)	305 (267–344)	11 (10–11)
Mongolia	2026	10 (9–11)	0 (0–0)	309 (260–358)	11 (9–12)
Mongolia	2027	10 (9–12)	0 (0–0)	313 (253–373)	11 (9–12)
Mongolia	2028	10 (8–12)	0 (0–0)	317 (245–388)	11 (9–12)
Mongolia	2029	11 (8–13)	0 (0–0)	320 (236–405)	11 (9–12)
Mongolia	2030	11 (8–13)	0 (0–0)	324 (227–421)	11 (9–12)
Mongolia	2031	11 (8–14)	0 (0–0)	328 (217–439)	11 (9–12)
Mongolia	2032	11 (8–15)	0 (0–0)	332 (207–457)	11 (9–12)
Mongolia	2033	11 (8–15)	0 (0–0)	336 (196–476)	11 (9–12)
Mongolia	2034	12 (7–16)	0 (0–0)	339 (184–495)	11 (9–12)
Mongolia	2035	12 (7–16)	0 (0–0)	343 (172–514)	11 (9–12)
Mongolia	2036	12 (7–17)	0 (0–0)	347 (160–534)	11 (9–12)
Montenegro	2022	10 (9–10)	1 (1–1)	233 (220–247)	25 (24–26)

Montenegro	2023	10 (9–11)	1 (1–1)	207 (177–238)	25 (23–27)
Montenegro	2024	10 (9–11)	1 (1–1)	182 (131–232)	25 (22–28)
Montenegro	2025	10 (8–11)	1 (1–1)	156 (82–230)	26 (22–29)
Montenegro	2026	10 (8–11)	1 (1–1)	130 (30–230)	26 (22–30)
Montenegro	2027	10 (8–12)	1 (1–1)	104 (-25–233)	26 (22–30)
Montenegro	2028	10 (7–12)	1 (0–1)	78 (-82–238)	26 (22–30)
Montenegro	2029	10 (7–12)	1 (0–1)	52 (-141–245)	26 (22–31)
Montenegro	2030	10 (7–12)	1 (0–1)	26 (-202–254)	26 (22–31)
Montenegro	2031	9 (6–13)	1 (0–1)	0 (-265–266)	27 (22–31)
Montenegro	2032	9 (6–13)	1 (0–1)	-25 (-329–278)	27 (22–31)
Montenegro	2033	9 (5–13)	1 (0–1)	-51 (-396–293)	27 (22–31)
Montenegro	2034	9 (5–14)	1 (0–1)	-77 (-464–309)	27 (22–32)
Montenegro	2035	9 (5–14)	1 (0–1)	-103 (-533–327)	27 (22–32)
Montenegro	2036	9 (4–14)	1 (0–1)	-129 (-605–347)	27 (22–32)
Morocco	2022	62 (61–62)	0 (0–0)	1766 (1749–1782)	5 (5–5)
Morocco	2023	63 (62–64)	0 (0–0)	1804 (1767–1842)	5 (4–5)
Morocco	2024	64 (62–66)	0 (0–0)	1843 (1781–1905)	5 (4–5)
Morocco	2025	65 (62–69)	0 (0–0)	1882 (1790–1973)	5 (4–5)
Morocco	2026	67 (63–71)	0 (0–0)	1920 (1797–2044)	5 (4–5)
Morocco	2027	68 (63–74)	0 (0–0)	1959 (1800–2118)	5 (4–5)
Morocco	2028	69 (63–76)	0 (0–0)	1997 (1800–2195)	5 (4–5)
Morocco	2029	71 (63–79)	0 (0–0)	2036 (1798–2274)	5 (4–5)
Morocco	2030	72 (62–82)	0 (0–0)	2074 (1793–2356)	5 (4–6)
Morocco	2031	73 (62–85)	0 (0–0)	2113 (1786–2440)	5 (4–6)
Morocco	2032	75 (62–88)	0 (0–0)	2152 (1777–2527)	5 (3–6)
Morocco	2033	76 (61–91)	0 (0–0)	2190 (1765–2615)	5 (3–6)
Morocco	2034	77 (61–94)	0 (0–0)	2229 (1752–2706)	5 (3–6)
Morocco	2035	79 (60–97)	0 (0–0)	2267 (1736–2798)	5 (3–6)
Morocco	2036	80 (60–100)	0 (0–0)	2306 (1719–2893)	5 (3–6)
Mozambique	2022	9 (8–9)	0 (0–0)	229 (227–231)	2 (2–2)
Mozambique	2023	9 (8–9)	0 (0–0)	228 (223–233)	2 (2–2)
Mozambique	2024	8 (8–9)	0 (0–0)	228 (219–236)	2 (2–2)
Mozambique	2025	8 (8–9)	0 (0–0)	227 (215–239)	2 (2–2)

Mozambique	2026	8 (8–9)	0 (0–0)	226 (211–242)	2 (2–2)
Mozambique	2027	8 (7–9)	0 (0–0)	226 (206–246)	2 (2–2)
Mozambique	2028	8 (7–9)	0 (0–0)	225 (201–250)	2 (1–2)
Mozambique	2029	8 (7–9)	0 (0–0)	225 (196–254)	2 (1–2)
Mozambique	2030	8 (7–10)	0 (0–0)	224 (190–258)	2 (1–2)
Mozambique	2031	8 (6–10)	0 (0–0)	224 (185–262)	2 (1–2)
Mozambique	2032	8 (6–10)	0 (0–0)	223 (179–267)	2 (1–2)
Mozambique	2033	8 (6–10)	0 (0–0)	223 (174–272)	2 (1–2)
Mozambique	2034	8 (6–10)	0 (0–0)	222 (168–276)	2 (1–2)
Mozambique	2035	8 (5–10)	0 (0–0)	222 (163–281)	2 (1–2)
Mozambique	2036	8 (5–11)	0 (0–0)	221 (157–286)	2 (1–2)
Myanmar	2022	267 (265–270)	1 (1–1)	6943 (6880–7005)	13 (13–13)
Myanmar	2023	271 (266–277)	1 (1–1)	7057 (6930–7185)	13 (13–13)
Myanmar	2024	275 (266–284)	1 (1–1)	7173 (6948–7399)	13 (12–14)
Myanmar	2025	279 (266–292)	1 (1–1)	7286 (6950–7622)	13 (12–14)
Myanmar	2026	282 (265–299)	1 (0–1)	7400 (6934–7867)	13 (12–14)
Myanmar	2027	285 (264–307)	1 (0–1)	7513 (6903–8123)	13 (11–14)
Myanmar	2028	288 (262–314)	1 (0–1)	7627 (6859–8395)	13 (11–15)
Myanmar	2029	291 (260–321)	1 (0–1)	7740 (6802–8677)	13 (10–15)
Myanmar	2030	293 (258–329)	0 (0–1)	7853 (6734–8972)	13 (10–15)
Myanmar	2031	296 (256–336)	0 (0–1)	7966 (6655–9277)	12 (10–15)
Myanmar	2032	298 (253–343)	0 (0–1)	8079 (6567–9592)	12 (9–16)
Myanmar	2033	300 (250–350)	0 (0–1)	8192 (6468–9917)	12 (9–16)
Myanmar	2034	302 (247–356)	0 (0–1)	8306 (6361–10251)	12 (8–16)
Myanmar	2035	304 (244–363)	0 (0–1)	8419 (6244–10593)	12 (8–17)
Myanmar	2036	305 (241–370)	0 (0–1)	8532 (6120–10944)	12 (7–17)
Namibia	2022	3 (3–3)	0 (0–0)	73 (71–75)	5 (5–5)
Namibia	2023	3 (3–3)	0 (0–0)	73 (70–77)	5 (5–5)
Namibia	2024	3 (3–3)	0 (0–0)	73 (68–79)	5 (4–6)
Namibia	2025	3 (3–3)	0 (0–0)	74 (66–81)	5 (4–6)
Namibia	2026	3 (3–3)	0 (0–0)	74 (64–83)	5 (4–6)
Namibia	2027	3 (3–3)	0 (0–0)	74 (62–85)	5 (3–6)
Namibia	2028	3 (3–3)	0 (0–0)	74 (60–88)	5 (3–7)

Namibia	2029	3 (3–4)	0 (0–0)	74 (58–90)	5 (2–7)
Namibia	2030	3 (3–4)	0 (0–0)	74 (56–92)	4 (2–7)
Namibia	2031	3 (3–4)	0 (0–0)	74 (54–94)	4 (1–8)
Namibia	2032	3 (3–4)	0 (0–0)	74 (52–96)	4 (0–8)
Namibia	2033	3 (3–4)	0 (-0–0)	74 (50–98)	4 (-0–8)
Namibia	2034	3 (3–4)	0 (-0–0)	74 (48–100)	4 (-1–9)
Namibia	2035	3 (3–4)	0 (-0–0)	74 (46–102)	4 (-1–9)
Namibia	2036	3 (3–4)	0 (-0–0)	74 (45–104)	4 (-2–10)
Nauru	2022	0 (0–0)	1 (1–1)	2 (2–2)	23 (23–23)
Nauru	2023	0 (0–0)	1 (1–1)	2 (2–2)	23 (23–24)
Nauru	2024	0 (0–0)	1 (1–1)	2 (2–2)	23 (22–24)
Nauru	2025	0 (0–0)	1 (1–1)	2 (2–2)	23 (22–24)
Nauru	2026	0 (0–0)	1 (1–1)	2 (2–2)	23 (22–24)
Nauru	2027	0 (0–0)	1 (1–1)	2 (2–2)	23 (21–24)
Nauru	2028	0 (0–0)	1 (1–1)	2 (2–2)	23 (21–25)
Nauru	2029	0 (0–0)	1 (1–1)	2 (2–2)	23 (21–25)
Nauru	2030	0 (0–0)	1 (1–1)	2 (2–2)	23 (20–25)
Nauru	2031	0 (0–0)	1 (1–1)	2 (1–2)	23 (20–25)
Nauru	2032	0 (0–0)	1 (1–1)	2 (1–2)	23 (19–26)
Nauru	2033	0 (0–0)	1 (1–1)	2 (1–2)	23 (19–26)
Nauru	2034	0 (0–0)	1 (1–1)	2 (1–2)	23 (19–26)
Nauru	2035	0 (0–0)	1 (1–1)	2 (1–2)	23 (18–27)
Nauru	2036	0 (0–0)	1 (1–1)	2 (1–2)	23 (18–27)
Nepal	2022	46 (45–46)	0 (0–0)	1121 (1108–1133)	5 (5–5)
Nepal	2023	46 (45–48)	0 (0–0)	1143 (1118–1167)	5 (4–5)
Nepal	2024	47 (45–49)	0 (0–0)	1165 (1126–1204)	5 (4–5)
Nepal	2025	48 (45–51)	0 (0–0)	1187 (1131–1243)	5 (4–5)
Nepal	2026	49 (45–52)	0 (0–0)	1209 (1134–1284)	5 (4–5)
Nepal	2027	49 (45–54)	0 (0–0)	1231 (1136–1326)	5 (4–5)
Nepal	2028	50 (44–56)	0 (0–0)	1253 (1136–1371)	5 (4–5)
Nepal	2029	51 (44–58)	0 (0–0)	1276 (1135–1416)	5 (4–6)
Nepal	2030	52 (43–60)	0 (0–0)	1298 (1132–1463)	5 (3–6)
Nepal	2031	52 (43–62)	0 (0–0)	1320 (1128–1512)	5 (3–6)

Nepal	2032	53 (42–64)	0 (0–0)	1342 (1123–1561)	5 (3–6)
Nepal	2033	54 (41–66)	0 (0–0)	1364 (1117–1612)	5 (3–6)
Nepal	2034	55 (40–69)	0 (0–0)	1386 (1109–1664)	5 (3–7)
Nepal	2035	55 (40–71)	0 (0–0)	1409 (1100–1717)	5 (2–7)
Nepal	2036	56 (39–73)	0 (0–0)	1431 (1090–1771)	5 (2–7)
Netherlands	2022	378 (354–401)	1 (1–1)	8667 (8100–9234)	26 (24–28)
Netherlands	2023	378 (345–411)	1 (1–1)	8667 (7866–9468)	25 (22–28)
Netherlands	2024	378 (337–418)	1 (1–1)	8667 (7686–9649)	25 (21–28)
Netherlands	2025	378 (331–424)	1 (1–1)	8667 (7534–9800)	24 (20–28)
Netherlands	2026	378 (326–430)	1 (1–1)	8667 (7400–9934)	24 (19–28)
Netherlands	2027	378 (321–435)	1 (1–1)	8667 (7279–10055)	23 (18–28)
Netherlands	2028	378 (316–439)	1 (1–1)	8667 (7168–10166)	23 (17–28)
Netherlands	2029	378 (312–443)	1 (1–1)	8667 (7064–10270)	22 (16–28)
Netherlands	2030	378 (308–447)	1 (1–1)	8667 (6967–10367)	22 (15–28)
Netherlands	2031	378 (304–451)	1 (1–1)	8667 (6875–10459)	21 (14–28)
Netherlands	2032	378 (301–455)	1 (1–1)	8667 (6788–10546)	21 (14–28)
Netherlands	2033	378 (297–458)	1 (1–1)	8667 (6704–10630)	20 (13–27)
Netherlands	2034	378 (294–461)	1 (1–1)	8667 (6624–10710)	20 (12–27)
Netherlands	2035	378 (291–465)	1 (0–1)	8667 (6547–10787)	19 (11–27)
Netherlands	2036	378 (288–468)	1 (0–1)	8667 (6472–10862)	19 (10–27)
New Zealand	2022	68 (65–70)	1 (1–1)	1530 (1476–1584)	18 (18–19)
New Zealand	2023	69 (65–72)	1 (1–1)	1547 (1462–1632)	18 (17–20)
New Zealand	2024	69 (65–74)	1 (1–1)	1564 (1448–1679)	18 (16–21)
New Zealand	2025	70 (64–76)	1 (1–1)	1581 (1434–1727)	18 (14–21)
New Zealand	2026	71 (64–78)	1 (1–1)	1597 (1419–1776)	18 (13–22)
New Zealand	2027	72 (64–81)	1 (1–1)	1614 (1403–1826)	17 (12–23)
New Zealand	2028	73 (63–83)	1 (1–1)	1631 (1385–1877)	17 (10–24)
New Zealand	2029	74 (62–85)	1 (0–1)	1648 (1365–1930)	17 (9–25)
New Zealand	2030	75 (62–88)	1 (0–1)	1664 (1345–1984)	17 (7–26)
New Zealand	2031	76 (61–90)	1 (0–1)	1681 (1323–2039)	17 (6–28)
New Zealand	2032	76 (61–92)	1 (0–1)	1698 (1300–2096)	16 (4–29)
New Zealand	2033	77 (60–95)	1 (0–1)	1715 (1275–2154)	16 (2–30)
New Zealand	2034	78 (59–97)	1 (0–1)	1731 (1249–2213)	16 (-0–32)

New Zealand	2035	79 (58–100)	1 (0–1)	1748 (1222–2274)	16 (-2–33)
New Zealand	2036	80 (57–103)	1 (0–1)	1765 (1194–2336)	15 (-4–35)
Nicaragua	2022	8 (8–8)	0 (0–0)	227 (215–239)	4 (4–5)
Nicaragua	2023	8 (8–9)	0 (0–0)	233 (215–250)	4 (4–5)
Nicaragua	2024	8 (8–9)	0 (0–0)	238 (217–259)	4 (4–5)
Nicaragua	2025	9 (8–9)	0 (0–0)	243 (219–267)	4 (4–5)
Nicaragua	2026	9 (8–10)	0 (0–0)	248 (221–275)	4 (3–5)
Nicaragua	2027	9 (8–10)	0 (0–0)	254 (224–283)	4 (3–5)
Nicaragua	2028	9 (8–10)	0 (0–0)	259 (227–291)	4 (3–5)
Nicaragua	2029	9 (8–10)	0 (0–0)	264 (230–298)	4 (3–5)
Nicaragua	2030	9 (8–11)	0 (0–0)	269 (233–306)	4 (3–5)
Nicaragua	2031	10 (8–11)	0 (0–0)	275 (236–313)	4 (3–5)
Nicaragua	2032	10 (8–11)	0 (0–0)	280 (240–320)	4 (3–5)
Nicaragua	2033	10 (9–11)	0 (0–0)	285 (243–327)	4 (3–5)
Nicaragua	2034	10 (9–12)	0 (0–0)	290 (247–334)	4 (3–6)
Nicaragua	2035	10 (9–12)	0 (0–0)	296 (250–341)	4 (3–6)
Nicaragua	2036	11 (9–12)	0 (0–0)	301 (254–348)	4 (3–6)
Niger	2022	3 (3–3)	0 (0–0)	87 (85–88)	1 (1–1)
Niger	2023	3 (3–4)	0 (0–0)	89 (87–91)	1 (1–1)
Niger	2024	4 (3–4)	0 (0–0)	91 (88–94)	1 (1–1)
Niger	2025	4 (3–4)	0 (0–0)	93 (89–98)	1 (1–1)
Niger	2026	4 (3–4)	0 (0–0)	95 (89–101)	1 (1–1)
Niger	2027	4 (3–4)	0 (0–0)	97 (90–105)	1 (1–1)
Niger	2028	4 (3–4)	0 (0–0)	100 (90–109)	1 (1–1)
Niger	2029	4 (4–4)	0 (0–0)	102 (91–113)	1 (1–1)
Niger	2030	4 (4–4)	0 (0–0)	104 (91–117)	1 (1–1)
Niger	2031	4 (4–5)	0 (0–0)	106 (91–121)	1 (1–1)
Niger	2032	4 (4–5)	0 (0–0)	108 (91–126)	1 (1–1)
Niger	2033	4 (4–5)	0 (0–0)	111 (91–130)	1 (1–1)
Niger	2034	4 (4–5)	0 (0–0)	113 (91–135)	1 (1–1)
Niger	2035	4 (3–5)	0 (0–0)	115 (91–139)	1 (1–1)
Niger	2036	4 (3–5)	0 (0–0)	117 (90–144)	1 (1–1)
Nigeria	2022	37 (36–37)	0 (0–0)	1021 (1010–1032)	1 (1–1)

Nigeria	2023	37 (36–38)	0 (0–0)	1033 (1014–1051)	1 (1–1)
Nigeria	2024	37 (37–38)	0 (0–0)	1044 (1020–1068)	1 (1–1)
Nigeria	2025	38 (37–39)	0 (0–0)	1055 (1026–1085)	1 (1–1)
Nigeria	2026	38 (37–39)	0 (0–0)	1066 (1032–1101)	1 (1–1)
Nigeria	2027	38 (37–40)	0 (0–0)	1078 (1039–1116)	1 (1–1)
Nigeria	2028	39 (37–40)	0 (0–0)	1089 (1047–1131)	1 (1–1)
Nigeria	2029	39 (38–41)	0 (0–0)	1100 (1055–1145)	1 (1–1)
Nigeria	2030	39 (38–41)	0 (0–0)	1111 (1063–1159)	1 (1–1)
Nigeria	2031	40 (38–41)	0 (0–0)	1122 (1071–1173)	1 (1–1)
Nigeria	2032	40 (38–42)	0 (0–0)	1133 (1080–1187)	1 (0–1)
Nigeria	2033	41 (39–42)	0 (0–0)	1145 (1088–1201)	1 (0–1)
Nigeria	2034	41 (39–43)	0 (0–0)	1156 (1097–1215)	1 (0–1)
Nigeria	2035	41 (39–43)	0 (0–0)	1167 (1106–1228)	1 (0–1)
Nigeria	2036	42 (40–44)	0 (0–0)	1178 (1114–1242)	1 (0–1)
Niue	2022	0 (0–0)	0 (0–0)	0 (0–0)	11 (11–11)
Niue	2023	0 (0–0)	0 (0–0)	0 (0–0)	11 (11–11)
Niue	2024	0 (0–0)	0 (0–0)	0 (0–0)	11 (11–11)
Niue	2025	0 (0–0)	0 (0–0)	0 (0–0)	11 (11–12)
Niue	2026	0 (0–0)	0 (0–0)	0 (0–0)	11 (11–12)
Niue	2027	0 (0–0)	0 (0–0)	0 (0–0)	11 (11–12)
Niue	2028	0 (0–0)	0 (0–0)	0 (0–0)	11 (10–12)
Niue	2029	0 (0–0)	0 (0–0)	0 (0–0)	11 (10–12)
Niue	2030	0 (0–0)	0 (0–0)	0 (0–0)	11 (10–12)
Niue	2031	0 (0–0)	0 (0–0)	0 (0–0)	11 (10–12)
Niue	2032	0 (0–0)	0 (0–0)	0 (0–0)	11 (10–12)
Niue	2033	0 (0–0)	0 (0–0)	0 (0–0)	11 (10–12)
Niue	2034	0 (0–0)	0 (0–0)	0 (0–0)	11 (10–12)
Niue	2035	0 (0–0)	0 (0–0)	0 (0–0)	11 (10–12)
Niue	2036	0 (0–0)	0 (0–0)	0 (0–0)	11 (10–12)
North Macedonia	2022	33 (32–35)	1 (1–1)	878 (841–915)	26 (25–28)
North Macedonia	2023	33 (31–35)	1 (1–1)	871 (813–929)	26 (24–29)
North Macedonia	2024	33 (30–36)	1 (1–1)	863 (784–942)	26 (23–30)
North Macedonia	2025	32 (29–36)	1 (1–1)	856 (756–956)	27 (23–30)

North Macedonia	2026	32 (28–37)	1 (1–1)	849 (727–970)	27 (23–31)
North Macedonia	2027	32 (27–37)	1 (1–1)	841 (697–986)	27 (23–31)
North Macedonia	2028	32 (25–38)	1 (1–1)	834 (666–1002)	27 (22–32)
North Macedonia	2029	31 (24–38)	1 (1–1)	826 (634–1019)	27 (22–32)
North Macedonia	2030	31 (23–39)	1 (1–1)	819 (601–1037)	27 (22–32)
North Macedonia	2031	31 (22–40)	1 (1–1)	812 (568–1055)	27 (22–32)
North Macedonia	2032	30 (20–40)	1 (1–1)	804 (533–1075)	27 (22–32)
North Macedonia	2033	30 (19–41)	1 (1–1)	797 (498–1096)	27 (22–33)
North Macedonia	2034	30 (18–42)	1 (1–1)	789 (462–1117)	28 (22–33)
North Macedonia	2035	30 (16–43)	1 (1–1)	782 (425–1139)	28 (22–33)
North Macedonia	2036	29 (15–44)	1 (1–1)	775 (387–1162)	28 (22–33)
Northern Mariana Islands	2022	0 (0–0)	1 (1–1)	11 (10–11)	18 (17–18)
Northern Mariana Islands	2023	0 (0–0)	1 (1–1)	11 (10–11)	18 (17–19)
Northern Mariana Islands	2024	0 (0–0)	1 (1–1)	11 (10–12)	18 (17–19)
Northern Mariana Islands	2025	0 (0–0)	1 (1–1)	11 (10–12)	18 (17–19)
Northern Mariana Islands	2026	0 (0–0)	1 (1–1)	11 (10–12)	18 (17–19)
Northern Mariana Islands	2027	0 (0–0)	1 (1–1)	12 (10–13)	18 (17–20)
Northern Mariana Islands	2028	0 (0–0)	1 (1–1)	12 (11–13)	18 (16–20)
Northern Mariana Islands	2029	0 (0–1)	1 (1–1)	12 (11–13)	18 (16–21)
Northern Mariana Islands	2030	0 (0–1)	1 (1–1)	12 (11–13)	18 (16–21)
Northern Mariana Islands	2031	0 (0–1)	1 (1–1)	12 (11–14)	19 (15–22)
Northern Mariana Islands	2032	0 (0–1)	1 (1–1)	12 (11–14)	19 (15–22)
Northern Mariana Islands	2033	0 (0–1)	1 (1–1)	13 (11–14)	19 (15–23)
Northern Mariana Islands	2034	0 (0–1)	1 (0–1)	13 (11–14)	19 (14–23)
Northern Mariana Islands	2035	0 (0–1)	1 (0–1)	13 (11–15)	19 (14–24)
Northern Mariana Islands	2036	0 (0–1)	1 (0–1)	13 (12–15)	19 (13–24)
Norway	2022	53 (51–56)	1 (0–1)	1211 (1155–1266)	13 (12–14)
Norway	2023	54 (48–59)	1 (0–1)	1196 (1090–1302)	13 (11–14)
Norway	2024	54 (46–62)	0 (0–1)	1176 (1021–1330)	12 (10–15)
Norway	2025	54 (43–66)	0 (0–1)	1152 (952–1352)	12 (9–15)
Norway	2026	55 (41–69)	0 (0–1)	1127 (885–1368)	11 (7–15)
Norway	2027	55 (38–72)	0 (0–1)	1100 (820–1380)	11 (6–15)
Norway	2028	55 (36–75)	0 (0–1)	1072 (757–1388)	10 (5–15)

Norway	2029	55 (33–77)	0 (0–1)	1044 (696–1392)	9 (3–15)
Norway	2030	55 (31–80)	0 (0–1)	1015 (637–1394)	9 (2–15)
Norway	2031	55 (28–82)	0 (0–1)	987 (580–1394)	8 (1–15)
Norway	2032	55 (26–84)	0 (-0–1)	958 (524–1392)	7 (-0–15)
Norway	2033	55 (24–87)	0 (-0–1)	929 (469–1388)	7 (-1–15)
Norway	2034	55 (22–89)	0 (-0–1)	900 (416–1383)	6 (-3–15)
Norway	2035	55 (20–91)	0 (-0–1)	870 (364–1377)	5 (-4–14)
Norway	2036	55 (18–93)	0 (-0–1)	841 (313–1370)	5 (-5–14)
Oman	2022	2 (2–2)	0 (0–0)	49 (47–51)	2 (2–2)
Oman	2023	2 (2–2)	0 (0–0)	50 (47–53)	2 (2–2)
Oman	2024	2 (1–2)	0 (0–0)	48 (42–54)	2 (2–2)
Oman	2025	2 (1–2)	0 (0–0)	48 (40–56)	2 (2–2)
Oman	2026	2 (1–2)	0 (0–0)	47 (36–58)	2 (1–2)
Oman	2027	2 (1–2)	0 (0–0)	47 (33–60)	2 (1–3)
Oman	2028	2 (1–2)	0 (0–0)	45 (28–63)	2 (1–3)
Oman	2029	2 (1–2)	0 (0–0)	45 (25–65)	2 (1–3)
Oman	2030	2 (1–2)	0 (0–0)	44 (20–68)	2 (1–3)
Oman	2031	2 (1–2)	0 (0–0)	43 (16–71)	2 (1–3)
Oman	2032	2 (1–2)	0 (0–0)	43 (11–75)	2 (1–3)
Oman	2033	2 (1–2)	0 (0–0)	42 (6–78)	2 (1–3)
Oman	2034	2 (1–2)	0 (0–0)	41 (1–82)	2 (1–3)
Oman	2035	2 (1–2)	0 (0–0)	41 (-4–85)	2 (1–3)
Oman	2036	2 (1–2)	0 (0–0)	40 (-10–89)	2 (1–3)
Pakistan	2022	257 (254–260)	0 (0–0)	7172 (7093–7251)	5 (5–5)
Pakistan	2023	261 (255–267)	0 (0–0)	7291 (7137–7444)	5 (5–5)
Pakistan	2024	264 (255–273)	0 (0–0)	7411 (7165–7658)	5 (5–6)
Pakistan	2025	267 (254–281)	0 (0–0)	7531 (7179–7883)	5 (5–6)
Pakistan	2026	271 (253–289)	0 (0–0)	7651 (7181–8121)	5 (4–6)
Pakistan	2027	274 (251–297)	0 (0–0)	7771 (7173–8369)	5 (4–6)
Pakistan	2028	278 (249–306)	0 (0–0)	7891 (7155–8627)	5 (4–6)
Pakistan	2029	281 (247–315)	0 (0–0)	8011 (7127–8895)	5 (4–6)
Pakistan	2030	284 (245–324)	0 (0–0)	8131 (7091–9171)	5 (3–6)
Pakistan	2031	288 (242–334)	0 (0–0)	8251 (7047–9455)	5 (3–7)

Pakistan	2032	291 (238–344)	0 (0–0)	8371 (6995–9747)	5 (3–7)
Pakistan	2033	294 (235–354)	0 (0–0)	8491 (6935–10047)	5 (2–7)
Pakistan	2034	298 (231–364)	0 (0–0)	8611 (6868–10354)	5 (2–7)
Pakistan	2035	301 (227–375)	0 (0–0)	8731 (6795–10667)	4 (2–7)
Pakistan	2036	305 (223–386)	0 (0–0)	8851 (6714–10988)	4 (1–8)
Palau	2022	0 (0–0)	0 (0–0)	2 (2–2)	6 (6–6)
Palau	2023	0 (0–0)	0 (0–0)	2 (2–2)	6 (6–6)
Palau	2024	0 (0–0)	0 (0–0)	2 (2–2)	6 (6–6)
Palau	2025	0 (0–0)	0 (0–0)	2 (2–2)	6 (6–6)
Palau	2026	0 (0–0)	0 (0–0)	2 (2–2)	6 (6–6)
Palau	2027	0 (0–0)	0 (0–0)	2 (2–2)	6 (6–6)
Palau	2028	0 (0–0)	0 (0–0)	2 (2–2)	6 (6–6)
Palau	2029	0 (0–0)	0 (0–0)	2 (2–2)	6 (6–6)
Palau	2030	0 (0–0)	0 (0–0)	2 (2–2)	6 (5–6)
Palau	2031	0 (0–0)	0 (0–0)	2 (2–2)	6 (5–6)
Palau	2032	0 (0–0)	0 (0–0)	2 (2–2)	6 (5–6)
Palau	2033	0 (0–0)	0 (0–0)	2 (2–2)	5 (5–6)
Palau	2034	0 (0–0)	0 (0–0)	2 (2–2)	5 (5–6)
Palau	2035	0 (0–0)	0 (0–0)	2 (2–2)	5 (5–6)
Palau	2036	0 (0–0)	0 (0–0)	2 (2–2)	5 (5–6)
Palestine	2022	15 (15–16)	1 (1–1)	443 (433–452)	15 (15–16)
Palestine	2023	16 (15–16)	1 (1–1)	460 (443–476)	15 (14–16)
Palestine	2024	16 (15–17)	1 (1–1)	477 (453–501)	15 (14–16)
Palestine	2025	16 (15–17)	1 (1–1)	494 (463–526)	15 (14–16)
Palestine	2026	17 (15–18)	1 (1–1)	512 (472–551)	15 (13–16)
Palestine	2027	17 (16–18)	1 (1–1)	529 (480–578)	15 (13–16)
Palestine	2028	17 (16–19)	1 (1–1)	546 (488–604)	15 (13–16)
Palestine	2029	18 (16–19)	1 (1–1)	563 (495–632)	15 (13–16)
Palestine	2030	18 (16–20)	1 (1–1)	581 (502–659)	14 (12–16)
Palestine	2031	18 (16–20)	1 (1–1)	598 (508–687)	14 (12–16)
Palestine	2032	18 (16–20)	1 (0–1)	615 (514–716)	14 (12–16)
Palestine	2033	19 (17–21)	1 (0–1)	632 (520–745)	14 (12–16)
Palestine	2034	19 (17–21)	1 (0–1)	650 (525–775)	14 (12–16)

Palestine	2035	19 (17–22)	1 (0–1)	667 (529–805)	14 (11–16)
Palestine	2036	20 (17–22)	1 (0–1)	684 (533–835)	14 (11–16)
Panama	2022	12 (11–12)	0 (0–0)	293 (284–302)	6 (6–7)
Panama	2023	12 (11–12)	0 (0–0)	302 (292–313)	6 (6–7)
Panama	2024	12 (11–13)	0 (0–0)	311 (298–324)	6 (6–7)
Panama	2025	12 (12–13)	0 (0–0)	321 (305–337)	6 (6–7)
Panama	2026	13 (12–14)	0 (0–0)	330 (311–349)	6 (6–7)
Panama	2027	13 (12–14)	0 (0–0)	339 (316–362)	6 (6–7)
Panama	2028	13 (12–15)	0 (0–0)	348 (321–376)	6 (6–7)
Panama	2029	14 (12–16)	0 (0–0)	358 (325–390)	6 (6–7)
Panama	2030	14 (12–16)	0 (0–0)	367 (330–404)	6 (6–7)
Panama	2031	14 (12–17)	0 (0–0)	376 (334–419)	6 (6–7)
Panama	2032	15 (12–17)	0 (0–0)	385 (337–433)	6 (6–7)
Panama	2033	15 (11–18)	0 (0–0)	395 (341–448)	6 (6–7)
Panama	2034	15 (11–19)	0 (0–0)	404 (344–464)	6 (6–7)
Panama	2035	15 (11–19)	0 (0–0)	413 (347–479)	6 (6–7)
Panama	2036	16 (11–20)	0 (0–0)	422 (350–495)	6 (6–7)
Papua New Guinea	2022	6 (6–6)	0 (0–0)	214 (210–219)	3 (3–3)
Papua New Guinea	2023	7 (6–7)	0 (0–0)	222 (215–230)	3 (3–3)
Papua New Guinea	2024	7 (6–7)	0 (0–0)	230 (219–241)	3 (3–3)
Papua New Guinea	2025	7 (7–7)	0 (0–0)	238 (223–253)	3 (3–3)
Papua New Guinea	2026	7 (7–8)	0 (0–0)	245 (226–265)	3 (3–3)
Papua New Guinea	2027	7 (7–8)	0 (0–0)	253 (229–277)	3 (3–3)
Papua New Guinea	2028	8 (7–9)	0 (0–0)	261 (232–289)	3 (3–3)
Papua New Guinea	2029	8 (7–9)	0 (0–0)	268 (235–302)	3 (3–3)
Papua New Guinea	2030	8 (7–9)	0 (0–0)	276 (237–315)	3 (3–3)
Papua New Guinea	2031	8 (7–10)	0 (0–0)	284 (239–328)	3 (2–3)
Papua New Guinea	2032	9 (7–10)	0 (0–0)	292 (241–342)	3 (2–3)
Papua New Guinea	2033	9 (7–11)	0 (0–0)	299 (243–356)	3 (2–3)
Papua New Guinea	2034	9 (7–11)	0 (0–0)	307 (244–370)	3 (2–3)
Papua New Guinea	2035	9 (7–11)	0 (0–0)	315 (245–384)	3 (2–3)
Papua New Guinea	2036	10 (7–12)	0 (0–0)	322 (246–398)	3 (2–3)
Paraguay	2022	33 (32–34)	1 (1–1)	796 (776–815)	13 (12–13)

Paraguay	2023	33 (32–34)	1 (1–1)	804 (777–831)	13 (12–14)
Paraguay	2024	34 (32–35)	1 (0–1)	817 (789–845)	13 (11–14)
Paraguay	2025	35 (33–36)	1 (0–1)	844 (811–876)	13 (11–14)
Paraguay	2026	35 (34–37)	1 (0–1)	858 (822–894)	12 (11–14)
Paraguay	2027	36 (34–38)	1 (0–1)	873 (835–910)	12 (10–15)
Paraguay	2028	37 (35–39)	1 (0–1)	895 (854–935)	12 (10–15)
Paraguay	2029	38 (35–40)	1 (0–1)	911 (868–955)	12 (9–15)
Paraguay	2030	38 (36–40)	1 (0–1)	927 (882–972)	12 (9–15)
Paraguay	2031	39 (37–41)	1 (0–1)	947 (899–994)	12 (9–16)
Paraguay	2032	40 (37–42)	1 (0–1)	964 (914–1014)	12 (8–16)
Paraguay	2033	40 (38–43)	1 (0–1)	980 (929–1032)	12 (8–16)
Paraguay	2034	41 (39–44)	1 (0–1)	999 (946–1052)	12 (7–16)
Paraguay	2035	42 (39–45)	0 (0–1)	1017 (961–1072)	12 (7–17)
Paraguay	2036	43 (40–45)	0 (0–1)	1034 (977–1091)	12 (6–17)
Peru	2022	69 (65–73)	0 (0–0)	1659 (1565–1753)	4 (4–5)
Peru	2023	70 (63–77)	0 (0–0)	1687 (1528–1846)	4 (4–5)
Peru	2024	71 (62–80)	0 (0–0)	1718 (1506–1930)	4 (4–5)
Peru	2025	72 (62–83)	0 (0–0)	1751 (1495–2007)	4 (4–5)
Peru	2026	74 (61–86)	0 (0–0)	1784 (1489–2079)	4 (4–5)
Peru	2027	75 (61–89)	0 (0–0)	1817 (1488–2146)	5 (4–5)
Peru	2028	77 (62–92)	0 (0–0)	1851 (1491–2211)	5 (4–5)
Peru	2029	78 (62–94)	0 (0–0)	1884 (1495–2273)	5 (4–5)
Peru	2030	79 (62–97)	0 (0–0)	1917 (1502–2333)	5 (4–5)
Peru	2031	81 (62–99)	0 (0–0)	1951 (1510–2391)	5 (4–5)
Peru	2032	82 (63–102)	0 (0–0)	1984 (1520–2448)	4 (4–5)
Peru	2033	84 (63–104)	0 (0–0)	2018 (1531–2504)	4 (4–5)
Peru	2034	85 (64–106)	0 (0–0)	2051 (1543–2559)	4 (4–5)
Peru	2035	87 (64–109)	0 (0–0)	2084 (1555–2613)	4 (4–5)
Peru	2036	88 (65–111)	0 (0–0)	2118 (1569–2667)	5 (4–5)
Philippines	2022	606 (599–613)	1 (1–1)	17688 (17445–17931)	19 (19–20)
Philippines	2023	616 (608–624)	1 (1–1)	18044 (17770–18317)	19 (18–20)
Philippines	2024	629 (620–637)	1 (1–1)	18399 (18099–18700)	19 (18–20)
Philippines	2025	642 (633–652)	1 (1–1)	18755 (18429–19080)	19 (18–21)

Philippines	2026	654 (644–664)	1 (1–1)	19110 (18762–19459)	19 (18–21)
Philippines	2027	666 (656–677)	1 (1–1)	19466 (19095–19836)	19 (18–21)
Philippines	2028	679 (668–691)	1 (1–1)	19821 (19431–20212)	19 (18–21)
Philippines	2029	692 (680–704)	1 (1–1)	20177 (19767–20587)	19 (18–21)
Philippines	2030	704 (692–717)	1 (1–1)	20532 (20104–20961)	19 (18–21)
Philippines	2031	717 (704–730)	1 (1–1)	20888 (20441–21334)	19 (18–21)
Philippines	2032	729 (716–743)	1 (1–1)	21243 (20780–21707)	19 (18–21)
Philippines	2033	742 (728–756)	1 (1–1)	21599 (21119–22079)	20 (18–21)
Philippines	2034	754 (740–769)	1 (1–1)	21954 (21458–22450)	20 (18–21)
Philippines	2035	767 (752–782)	1 (1–1)	22310 (21798–22821)	20 (18–21)
Philippines	2036	779 (764–795)	1 (1–1)	22665 (22139–23192)	20 (18–21)
Poland	2022	833 (812–854)	1 (1–1)	20250 (19636–20863)	30 (29–31)
Poland	2023	829 (797–860)	1 (1–1)	19938 (19017–20860)	29 (28–31)
Poland	2024	827 (785–870)	1 (1–1)	19838 (18606–21071)	28 (26–31)
Poland	2025	823 (762–883)	1 (1–1)	19557 (17790–21324)	28 (25–30)
Poland	2026	819 (741–896)	1 (1–1)	19291 (17039–21544)	27 (24–30)
Poland	2027	816 (722–911)	1 (1–1)	19135 (16378–21893)	27 (23–30)
Poland	2028	812 (696–928)	1 (1–1)	18872 (15498–22247)	26 (21–31)
Poland	2029	808 (672–945)	1 (1–1)	18629 (14646–22611)	25 (20–31)
Poland	2030	806 (648–964)	1 (1–1)	18441 (13823–23059)	25 (18–31)
Poland	2031	802 (619–984)	1 (1–1)	18191 (12871–23510)	24 (17–31)
Poland	2032	798 (592–1005)	1 (1–1)	17958 (11929–23986)	23 (15–31)
Poland	2033	795 (563–1027)	1 (1–1)	17752 (10986–24518)	23 (14–32)
Poland	2034	791 (533–1050)	1 (1–1)	17510 (9963–25058)	22 (12–32)
Poland	2035	788 (502–1073)	1 (0–1)	17282 (8938–25626)	21 (10–32)
Poland	2036	784 (471–1098)	1 (0–1)	17066 (7899–26234)	21 (9–33)
Portugal	2022	120 (116–125)	1 (1–1)	3105 (2976–3234)	16 (15–17)
Portugal	2023	118 (111–125)	1 (0–1)	3040 (2831–3250)	15 (14–17)
Portugal	2024	116 (106–126)	1 (0–1)	2976 (2686–3266)	15 (13–17)
Portugal	2025	114 (101–127)	0 (0–1)	2911 (2537–3285)	14 (12–16)
Portugal	2026	112 (96–127)	0 (0–1)	2846 (2384–3308)	14 (11–16)
Portugal	2027	109 (90–129)	0 (0–1)	2782 (2227–3336)	13 (10–16)
Portugal	2028	107 (85–130)	0 (0–1)	2717 (2065–3369)	13 (9–16)

Portugal	2029	105 (79–131)	0 (0–1)	2652 (1899–3406)	12 (8–17)
Portugal	2030	103 (73–133)	0 (0–1)	2588 (1728–3447)	12 (7–17)
Portugal	2031	101 (67–134)	0 (0–1)	2523 (1553–3493)	11 (6–17)
Portugal	2032	99 (61–136)	0 (0–1)	2458 (1374–3543)	11 (5–17)
Portugal	2033	97 (55–138)	0 (0–1)	2394 (1191–3597)	10 (4–17)
Portugal	2034	94 (49–140)	0 (0–1)	2329 (1004–3654)	10 (3–17)
Portugal	2035	92 (42–143)	0 (0–1)	2264 (813–3715)	9 (1–17)
Portugal	2036	90 (35–145)	0 (0–1)	2200 (619–3780)	9 (0–17)
Puerto Rico	2022	30 (28–32)	0 (0–0)	731 (677–785)	13 (12–14)
Puerto Rico	2023	30 (28–32)	0 (0–0)	729 (668–791)	13 (11–14)
Puerto Rico	2024	30 (27–32)	0 (0–1)	724 (649–798)	13 (11–14)
Puerto Rico	2025	29 (27–32)	0 (0–1)	715 (621–809)	13 (11–14)
Puerto Rico	2026	29 (26–33)	0 (0–1)	710 (600–819)	13 (11–14)
Puerto Rico	2027	29 (25–33)	0 (0–1)	703 (575–831)	13 (11–14)
Puerto Rico	2028	29 (24–33)	0 (0–1)	696 (548–844)	13 (11–14)
Puerto Rico	2029	28 (23–34)	0 (0–1)	690 (522–859)	13 (11–14)
Puerto Rico	2030	28 (22–34)	0 (0–1)	684 (494–874)	13 (11–14)
Puerto Rico	2031	28 (21–35)	0 (0–1)	677 (465–890)	13 (11–14)
Puerto Rico	2032	28 (20–36)	0 (0–1)	671 (435–906)	13 (11–14)
Puerto Rico	2033	27 (18–36)	0 (0–1)	664 (404–924)	13 (10–15)
Puerto Rico	2034	27 (17–37)	0 (0–1)	658 (373–942)	13 (10–15)
Puerto Rico	2035	27 (16–38)	0 (0–1)	651 (341–962)	13 (10–15)
Puerto Rico	2036	27 (15–38)	0 (0–1)	645 (308–982)	13 (10–15)
Qatar	2022	2 (2–2)	0 (0–0)	81 (77–85)	6 (5–6)
Qatar	2023	2 (2–3)	0 (0–0)	83 (76–89)	6 (4–7)
Qatar	2024	3 (2–3)	0 (0–0)	79 (69–89)	6 (4–8)
Qatar	2025	3 (2–3)	0 (0–0)	80 (67–93)	6 (4–8)
Qatar	2026	3 (2–3)	0 (0–0)	78 (61–94)	6 (4–9)
Qatar	2027	3 (2–3)	0 (0–0)	78 (57–98)	6 (4–9)
Qatar	2028	3 (2–3)	0 (0–0)	76 (52–101)	6 (4–9)
Qatar	2029	3 (2–3)	0 (0–0)	76 (48–104)	7 (4–9)
Qatar	2030	3 (2–4)	0 (0–0)	75 (43–107)	7 (4–9)
Qatar	2031	3 (2–4)	0 (0–0)	75 (38–111)	7 (4–9)

Qatar	2032	3 (2–4)	0 (0–0)	74 (33–114)	7 (4–10)
Qatar	2033	3 (2–4)	0 (0–0)	73 (29–118)	7 (4–10)
Qatar	2034	3 (2–4)	0 (0–0)	73 (24–121)	7 (4–10)
Qatar	2035	3 (2–4)	0 (0–0)	73 (20–125)	7 (4–10)
Qatar	2036	3 (2–4)	0 (0–0)	72 (16–128)	7 (4–10)
Republic of Korea	2022	533 (525–541)	1 (1–1)	12177 (11930–12423)	13 (12–13)
Republic of Korea	2023	543 (531–554)	1 (1–1)	12333 (11910–12755)	13 (12–14)
Republic of Korea	2024	552 (538–566)	1 (1–1)	12489 (11878–13099)	13 (11–14)
Republic of Korea	2025	562 (546–578)	1 (0–1)	12645 (11832–13457)	12 (11–14)
Republic of Korea	2026	572 (554–590)	1 (0–1)	12801 (11770–13831)	12 (10–15)
Republic of Korea	2027	582 (562–601)	1 (0–1)	12957 (11694–14219)	12 (10–15)
Republic of Korea	2028	591 (570–613)	1 (0–1)	13113 (11604–14621)	12 (9–15)
Republic of Korea	2029	601 (578–624)	1 (0–1)	13269 (11500–15037)	12 (8–16)
Republic of Korea	2030	611 (586–635)	1 (0–1)	13425 (11383–15466)	12 (7–16)
Republic of Korea	2031	620 (595–646)	1 (0–1)	13581 (11254–15907)	12 (7–17)
Republic of Korea	2032	630 (603–657)	1 (0–1)	13737 (11113–16360)	12 (6–17)
Republic of Korea	2033	640 (612–668)	1 (0–1)	13893 (10961–16825)	11 (5–18)
Republic of Korea	2034	650 (620–679)	1 (0–1)	14049 (10797–17301)	11 (4–18)
Republic of Korea	2035	659 (629–689)	0 (0–1)	14205 (10623–17787)	11 (3–19)
Republic of Korea	2036	669 (638–700)	0 (0–1)	14361 (10438–18284)	11 (2–20)
Republic of Moldova	2022	51 (48–54)	1 (1–1)	1424 (1319–1530)	24 (22–26)
Republic of Moldova	2023	51 (45–57)	1 (1–1)	1424 (1233–1616)	24 (20–28)
Republic of Moldova	2024	51 (43–59)	1 (1–1)	1424 (1175–1674)	24 (19–29)
Republic of Moldova	2025	51 (41–61)	1 (1–1)	1424 (1128–1721)	24 (18–30)
Republic of Moldova	2026	51 (40–62)	1 (1–1)	1424 (1088–1761)	24 (18–31)
Republic of Moldova	2027	51 (39–64)	1 (1–1)	1424 (1052–1797)	24 (17–31)
Republic of Moldova	2028	51 (38–65)	1 (1–1)	1424 (1019–1830)	24 (16–32)
Republic of Moldova	2029	51 (37–66)	1 (1–1)	1424 (988–1861)	24 (16–33)
Republic of Moldova	2030	51 (36–67)	1 (1–1)	1424 (960–1889)	24 (15–33)
Republic of Moldova	2031	51 (35–68)	1 (1–1)	1424 (933–1916)	24 (15–34)
Republic of Moldova	2032	51 (34–68)	1 (1–1)	1424 (908–1941)	24 (14–34)
Republic of Moldova	2033	51 (33–69)	1 (0–1)	1424 (883–1966)	24 (14–35)
Republic of Moldova	2034	51 (32–70)	1 (0–1)	1424 (860–1989)	24 (13–35)

Republic of Moldova	2035	51 (31–71)	1 (0–1)	1424 (838–2011)	24 (13–36)
Republic of Moldova	2036	51 (31–71)	1 (0–1)	1424 (817–2032)	24 (12–36)
Romania	2022	372 (350–393)	1 (1–1)	9725 (9118–10331)	30 (28–32)
Romania	2023	376 (340–412)	1 (1–1)	9729 (8658–10800)	30 (26–33)
Romania	2024	381 (333–430)	1 (1–1)	9731 (8259–11203)	30 (25–34)
Romania	2025	387 (327–446)	1 (1–1)	9732 (7915–11550)	30 (24–35)
Romania	2026	392 (324–460)	1 (1–1)	9732 (7612–11853)	30 (23–36)
Romania	2027	397 (321–474)	1 (1–1)	9733 (7341–12124)	30 (22–37)
Romania	2028	403 (319–487)	1 (1–1)	9733 (7096–12369)	30 (22–38)
Romania	2029	408 (317–499)	1 (1–1)	9733 (6871–12594)	30 (21–38)
Romania	2030	413 (316–510)	1 (1–1)	9733 (6662–12803)	30 (20–39)
Romania	2031	419 (316–522)	1 (1–1)	9733 (6466–12999)	30 (20–40)
Romania	2032	424 (316–533)	1 (1–1)	9733 (6281–13184)	30 (19–40)
Romania	2033	430 (316–543)	1 (1–1)	9733 (6106–13360)	30 (19–41)
Romania	2034	435 (316–554)	1 (1–1)	9733 (5938–13527)	30 (18–41)
Romania	2035	440 (316–564)	1 (1–1)	9733 (5778–13688)	30 (18–42)
Romania	2036	446 (317–574)	1 (1–1)	9733 (5624–13842)	30 (17–42)
Russian Federation	2022	1948 (1807–2089)	1 (1–1)	52956 (48883–57028)	23 (21–25)
Russian Federation	2023	1948 (1749–2147)	1 (1–1)	52956 (47196–58715)	23 (20–26)
Russian Federation	2024	1948 (1704–2192)	1 (1–1)	52956 (45901–60010)	24 (20–27)
Russian Federation	2025	1948 (1666–2230)	1 (1–1)	52956 (44810–61101)	24 (20–28)
Russian Federation	2026	1948 (1633–2263)	1 (1–1)	52956 (43849–62063)	24 (20–28)
Russian Federation	2027	1948 (1603–2293)	1 (1–1)	52956 (42979–62932)	24 (20–29)
Russian Federation	2028	1948 (1575–2321)	1 (1–1)	52956 (42180–63731)	25 (20–29)
Russian Federation	2029	1948 (1549–2346)	1 (1–1)	52956 (41436–64475)	25 (20–29)
Russian Federation	2030	1948 (1525–2371)	1 (1–1)	52956 (40737–65174)	25 (20–29)
Russian Federation	2031	1948 (1502–2393)	1 (1–1)	52956 (40076–65835)	25 (20–29)
Russian Federation	2032	1948 (1480–2415)	1 (1–1)	52956 (39448–66464)	25 (20–29)
Russian Federation	2033	1948 (1460–2436)	1 (1–1)	52956 (38847–67064)	25 (20–29)
Russian Federation	2034	1948 (1440–2456)	1 (1–1)	52956 (38271–67640)	25 (20–29)
Russian Federation	2035	1948 (1421–2475)	1 (1–1)	52956 (37717–68195)	25 (20–29)
Russian Federation	2036	1948 (1402–2494)	1 (1–1)	52956 (37182–68730)	25 (20–29)
Rwanda	2022	27 (26–28)	0 (0–0)	680 (655–704)	11 (10–11)

Rwanda	2023	28 (26–30)	0 (0–1)	705 (645–765)	11 (10–11)
Rwanda	2024	29 (26–32)	0 (0–1)	734 (645–823)	11 (10–12)
Rwanda	2025	30 (26–35)	0 (0–1)	763 (653–874)	11 (9–12)
Rwanda	2026	31 (26–37)	0 (0–1)	791 (661–921)	11 (9–13)
Rwanda	2027	32 (25–39)	1 (0–1)	817 (663–972)	11 (9–13)
Rwanda	2028	33 (25–42)	1 (0–1)	844 (662–1027)	11 (8–13)
Rwanda	2029	34 (24–45)	1 (0–1)	872 (661–1083)	11 (8–14)
Rwanda	2030	36 (24–47)	1 (0–1)	900 (660–1140)	11 (8–14)
Rwanda	2031	37 (23–50)	1 (0–1)	927 (658–1197)	11 (7–15)
Rwanda	2032	38 (22–53)	1 (0–1)	954 (654–1255)	11 (7–15)
Rwanda	2033	39 (21–56)	1 (0–1)	982 (649–1315)	11 (7–15)
Rwanda	2034	40 (21–59)	1 (0–1)	1009 (642–1376)	11 (6–16)
Rwanda	2035	41 (20–62)	1 (0–1)	1037 (636–1438)	11 (6–16)
Rwanda	2036	42 (18–66)	1 (0–1)	1064 (627–1501)	11 (6–16)
Saint Kitts and Nevis	2022	0 (0–0)	0 (0–0)	7 (6–7)	8 (8–9)
Saint Kitts and Nevis	2023	0 (0–0)	0 (0–0)	7 (6–7)	8 (8–9)
Saint Kitts and Nevis	2024	0 (0–0)	0 (0–0)	7 (6–7)	8 (8–9)
Saint Kitts and Nevis	2025	0 (0–0)	0 (0–0)	7 (7–8)	8 (8–9)
Saint Kitts and Nevis	2026	0 (0–0)	0 (0–0)	7 (7–8)	8 (8–9)
Saint Kitts and Nevis	2027	0 (0–0)	0 (0–0)	8 (7–9)	8 (8–9)
Saint Kitts and Nevis	2028	0 (0–0)	0 (0–0)	8 (7–9)	8 (8–9)
Saint Kitts and Nevis	2029	0 (0–0)	0 (0–0)	8 (7–9)	8 (8–9)
Saint Kitts and Nevis	2030	0 (0–0)	0 (0–0)	8 (7–10)	8 (8–9)
Saint Kitts and Nevis	2031	0 (0–0)	0 (0–0)	9 (7–10)	8 (8–9)
Saint Kitts and Nevis	2032	0 (0–0)	0 (0–0)	9 (7–11)	8 (8–9)
Saint Kitts and Nevis	2033	0 (0–0)	0 (0–0)	9 (7–11)	8 (8–9)
Saint Kitts and Nevis	2034	0 (0–0)	0 (0–0)	9 (7–12)	8 (8–9)
Saint Kitts and Nevis	2035	0 (0–0)	0 (0–0)	9 (7–12)	8 (8–9)
Saint Kitts and Nevis	2036	0 (0–0)	0 (0–0)	10 (7–13)	8 (8–9)
Saint Lucia	2022	1 (1–1)	0 (0–0)	18 (18–19)	7 (7–8)
Saint Lucia	2023	1 (1–1)	0 (0–0)	19 (18–20)	7 (7–8)
Saint Lucia	2024	1 (1–1)	0 (0–0)	19 (18–21)	7 (7–8)
Saint Lucia	2025	1 (1–1)	0 (0–0)	20 (18–21)	7 (7–8)

Saint Lucia	2026	1 (1–1)	0 (0–0)	20 (18–22)	7 (6–8)
Saint Lucia	2027	1 (1–1)	0 (0–0)	21 (19–23)	7 (6–8)
Saint Lucia	2028	1 (1–1)	0 (0–0)	21 (19–24)	7 (6–8)
Saint Lucia	2029	1 (1–1)	0 (0–0)	22 (19–24)	7 (6–8)
Saint Lucia	2030	1 (1–1)	0 (0–0)	22 (19–25)	7 (6–9)
Saint Lucia	2031	1 (1–1)	0 (0–0)	23 (19–26)	7 (6–9)
Saint Lucia	2032	1 (1–1)	0 (0–0)	23 (19–27)	7 (6–9)
Saint Lucia	2033	1 (1–1)	0 (0–0)	23 (19–28)	7 (6–9)
Saint Lucia	2034	1 (1–1)	0 (0–0)	24 (19–28)	7 (6–9)
Saint Lucia	2035	1 (1–1)	0 (0–0)	24 (20–29)	7 (6–9)
Saint Lucia	2036	1 (1–1)	0 (0–0)	25 (20–30)	7 (6–9)
Saint Vincent and the Grenadines	2022	1 (0–1)	0 (0–0)	13 (13–14)	9 (8–9)
Saint Vincent and the Grenadines	2023	1 (0–1)	0 (0–0)	13 (13–14)	9 (8–9)
Saint Vincent and the Grenadines	2024	1 (0–1)	0 (0–0)	14 (13–15)	9 (8–10)
Saint Vincent and the Grenadines	2025	1 (1–1)	0 (0–0)	14 (13–15)	9 (8–10)
Saint Vincent and the Grenadines	2026	1 (1–1)	0 (0–0)	14 (13–16)	9 (8–10)
Saint Vincent and the Grenadines	2027	1 (1–1)	0 (0–0)	15 (13–16)	9 (8–10)
Saint Vincent and the Grenadines	2028	1 (1–1)	0 (0–0)	15 (13–17)	9 (8–10)
Saint Vincent and the Grenadines	2029	1 (1–1)	0 (0–0)	15 (13–18)	9 (8–10)
Saint Vincent and the Grenadines	2030	1 (1–1)	0 (0–0)	15 (13–18)	9 (7–10)
Saint Vincent and the Grenadines	2031	1 (1–1)	0 (0–0)	16 (13–19)	9 (7–10)
Saint Vincent and the Grenadines	2032	1 (1–1)	0 (0–0)	16 (12–20)	9 (7–10)
Saint Vincent and the Grenadines	2033	1 (1–1)	0 (0–0)	16 (12–20)	9 (7–10)
Saint Vincent and the Grenadines	2034	1 (1–1)	0 (0–0)	17 (12–21)	9 (7–10)
Saint Vincent and the Grenadines	2035	1 (0–1)	0 (0–0)	17 (12–22)	9 (7–11)
Saint Vincent and the Grenadines	2036	1 (0–1)	0 (0–0)	17 (12–22)	9 (7–11)
Samoa	2022	0 (0–0)	0 (0–0)	13 (13–13)	8 (8–9)
Samoa	2023	0 (0–0)	0 (0–0)	14 (13–14)	8 (8–9)
Samoa	2024	0 (0–0)	0 (0–0)	14 (13–14)	8 (8–9)
Samoa	2025	0 (0–1)	0 (0–0)	14 (14–14)	8 (8–9)
Samoa	2026	0 (0–1)	0 (0–0)	14 (14–15)	8 (8–9)
Samoa	2027	0 (0–1)	0 (0–0)	14 (14–15)	8 (8–9)
Samoa	2028	1 (0–1)	0 (0–0)	15 (14–15)	8 (8–9)

Samoa	2029	1 (0–1)	0 (0–0)	15 (14–16)	8 (8–9)
Samoa	2030	1 (0–1)	0 (0–0)	15 (14–16)	8 (7–9)
Samoa	2031	1 (0–1)	0 (0–0)	15 (14–16)	8 (7–9)
Samoa	2032	1 (0–1)	0 (0–0)	15 (14–17)	8 (7–10)
Samoa	2033	1 (0–1)	0 (0–0)	15 (14–17)	8 (7–10)
Samoa	2034	1 (0–1)	0 (0–0)	16 (14–17)	8 (7–10)
Samoa	2035	1 (0–1)	0 (0–0)	16 (14–18)	8 (7–10)
Samoa	2036	1 (0–1)	0 (0–0)	16 (14–18)	8 (7–10)
San Marino	2022	0 (0–0)	0 (0–0)	10 (9–11)	10 (9–12)
San Marino	2023	0 (0–1)	0 (0–0)	10 (8–11)	10 (7–12)
San Marino	2024	0 (0–1)	0 (0–0)	10 (8–11)	9 (6–12)
San Marino	2025	0 (0–1)	0 (0–0)	10 (8–11)	9 (5–12)
San Marino	2026	0 (0–1)	0 (0–0)	10 (8–11)	8 (4–12)
San Marino	2027	0 (0–1)	0 (0–0)	10 (8–11)	7 (3–12)
San Marino	2028	0 (0–1)	0 (0–0)	10 (8–11)	7 (2–11)
San Marino	2029	0 (0–1)	0 (0–0)	10 (8–11)	6 (1–11)
San Marino	2030	0 (0–1)	0 (-0–0)	10 (8–11)	6 (1–11)
San Marino	2031	0 (0–1)	0 (-0–0)	10 (8–11)	5 (-0–11)
San Marino	2032	0 (0–1)	0 (-0–0)	10 (8–11)	5 (-1–10)
San Marino	2033	0 (0–1)	0 (-0–0)	10 (8–11)	4 (-2–10)
San Marino	2034	0 (0–1)	0 (-0–0)	10 (8–11)	3 (-3–10)
San Marino	2035	0 (0–1)	0 (-0–0)	10 (8–11)	3 (-4–9)
San Marino	2036	0 (0–1)	0 (-0–0)	10 (8–11)	2 (-4–9)
Sao Tome and Principe	2022	0 (0–0)	0 (0–0)	6 (6–6)	5 (4–5)
Sao Tome and Principe	2023	0 (0–0)	0 (0–0)	6 (6–6)	5 (4–5)
Sao Tome and Principe	2024	0 (0–0)	0 (0–0)	6 (6–6)	5 (4–5)
Sao Tome and Principe	2025	0 (0–0)	0 (0–0)	6 (6–7)	5 (4–5)
Sao Tome and Principe	2026	0 (0–0)	0 (0–0)	6 (6–7)	5 (4–5)
Sao Tome and Principe	2027	0 (0–0)	0 (0–0)	7 (6–7)	5 (4–5)
Sao Tome and Principe	2028	0 (0–0)	0 (0–0)	7 (6–7)	5 (4–5)
Sao Tome and Principe	2029	0 (0–0)	0 (0–0)	7 (6–8)	5 (4–5)
Sao Tome and Principe	2030	0 (0–0)	0 (0–0)	7 (6–8)	5 (4–5)
Sao Tome and Principe	2031	0 (0–0)	0 (0–0)	7 (6–8)	5 (4–5)

Sao Tome and Principe	2032	0 (0–0)	0 (0–0)	7 (6–9)	5 (4–6)
Sao Tome and Principe	2033	0 (0–0)	0 (0–0)	8 (6–9)	5 (4–6)
Sao Tome and Principe	2034	0 (0–0)	0 (0–0)	8 (6–10)	5 (4–6)
Sao Tome and Principe	2035	0 (0–0)	0 (0–0)	8 (6–10)	5 (4–6)
Sao Tome and Principe	2036	0 (0–0)	0 (0–0)	8 (6–10)	5 (4–6)
Saudi Arabia	2022	47 (47–48)	0 (0–0)	1725 (1711–1740)	5 (5–5)
Saudi Arabia	2023	49 (48–50)	0 (0–0)	1790 (1758–1823)	5 (5–5)
Saudi Arabia	2024	51 (50–52)	0 (0–0)	1855 (1801–1910)	5 (5–6)
Saudi Arabia	2025	53 (51–54)	0 (0–0)	1920 (1840–2000)	5 (5–6)
Saudi Arabia	2026	54 (52–57)	0 (0–0)	1985 (1877–2094)	5 (4–6)
Saudi Arabia	2027	56 (53–59)	0 (0–0)	2050 (1911–2190)	5 (4–6)
Saudi Arabia	2028	58 (54–61)	0 (0–0)	2115 (1943–2288)	5 (4–6)
Saudi Arabia	2029	60 (55–64)	0 (0–0)	2181 (1972–2389)	5 (4–6)
Saudi Arabia	2030	61 (57–66)	0 (0–0)	2246 (1999–2492)	5 (3–6)
Saudi Arabia	2031	63 (57–69)	0 (0–0)	2311 (2024–2597)	5 (3–6)
Saudi Arabia	2032	65 (58–72)	0 (0–0)	2376 (2047–2704)	5 (3–6)
Saudi Arabia	2033	67 (59–74)	0 (0–0)	2441 (2068–2813)	4 (2–7)
Saudi Arabia	2034	69 (60–77)	0 (0–0)	2506 (2088–2923)	4 (2–7)
Saudi Arabia	2035	70 (61–80)	0 (0–0)	2571 (2106–3036)	4 (2–7)
Saudi Arabia	2036	72 (62–82)	0 (0–0)	2636 (2122–3150)	4 (1–7)
Senegal	2022	7 (7–8)	0 (0–0)	215 (207–224)	2 (2–3)
Senegal	2023	8 (7–8)	0 (0–0)	221 (210–231)	2 (2–3)
Senegal	2024	8 (7–8)	0 (0–0)	226 (212–239)	2 (2–3)
Senegal	2025	8 (7–8)	0 (0–0)	231 (215–247)	2 (2–3)
Senegal	2026	8 (7–9)	0 (0–0)	236 (217–255)	2 (2–3)
Senegal	2027	8 (7–9)	0 (0–0)	241 (219–263)	2 (2–3)
Senegal	2028	8 (7–9)	0 (0–0)	246 (221–271)	2 (2–3)
Senegal	2029	8 (7–10)	0 (0–0)	252 (223–280)	2 (2–3)
Senegal	2030	9 (7–10)	0 (0–0)	257 (225–289)	2 (2–3)
Senegal	2031	9 (7–10)	0 (0–0)	262 (226–298)	2 (2–3)
Senegal	2032	9 (7–10)	0 (0–0)	267 (227–307)	2 (2–3)
Senegal	2033	9 (7–11)	0 (0–0)	272 (229–316)	2 (2–3)
Senegal	2034	9 (7–11)	0 (0–0)	277 (230–325)	2 (2–3)

Senegal	2035	9 (8–11)	0 (0–0)	282 (231–334)	2 (2–3)
Senegal	2036	10 (8–12)	0 (0–0)	288 (232–344)	2 (2–3)
Serbia	2022	189 (178–200)	1 (1–1)	4824 (4521–5128)	32 (30–34)
Serbia	2023	188 (172–204)	1 (1–1)	4778 (4314–5242)	32 (29–35)
Serbia	2024	187 (165–208)	1 (1–1)	4732 (4120–5344)	32 (29–36)
Serbia	2025	186 (159–213)	1 (1–1)	4686 (3928–5444)	32 (28–36)
Serbia	2026	184 (152–217)	1 (1–1)	4640 (3734–5546)	32 (28–37)
Serbia	2027	183 (146–221)	1 (1–1)	4594 (3538–5650)	32 (27–37)
Serbia	2028	182 (139–226)	1 (1–1)	4548 (3337–5758)	32 (27–38)
Serbia	2029	181 (132–230)	1 (1–1)	4502 (3133–5871)	32 (26–38)
Serbia	2030	180 (125–235)	1 (1–1)	4455 (2924–5987)	32 (26–38)
Serbia	2031	179 (117–240)	1 (1–1)	4409 (2710–6109)	32 (26–39)
Serbia	2032	178 (110–245)	1 (1–1)	4363 (2492–6234)	32 (25–39)
Serbia	2033	176 (102–251)	1 (1–1)	4317 (2270–6365)	32 (25–39)
Serbia	2034	175 (94–256)	1 (1–1)	4271 (2043–6499)	32 (25–39)
Serbia	2035	174 (86–262)	1 (1–1)	4225 (1811–6638)	32 (25–40)
Serbia	2036	173 (78–268)	1 (1–1)	4179 (1575–6782)	32 (24–40)
Seychelles	2022	1 (1–1)	1 (1–1)	29 (27–30)	22 (21–24)
Seychelles	2023	1 (1–1)	1 (1–1)	29 (27–31)	23 (20–25)
Seychelles	2024	1 (1–1)	1 (1–1)	30 (27–33)	23 (20–26)
Seychelles	2025	1 (1–1)	1 (1–1)	30 (26–34)	24 (20–27)
Seychelles	2026	1 (1–1)	1 (1–1)	31 (26–35)	24 (21–28)
Seychelles	2027	1 (1–1)	1 (1–1)	31 (27–36)	25 (21–29)
Seychelles	2028	1 (1–1)	1 (1–1)	32 (27–37)	25 (21–29)
Seychelles	2029	1 (1–1)	1 (1–1)	32 (27–38)	25 (21–29)
Seychelles	2030	1 (1–1)	1 (1–1)	33 (27–38)	25 (21–29)
Seychelles	2031	1 (1–1)	1 (1–1)	33 (27–39)	25 (21–29)
Seychelles	2032	1 (1–1)	1 (1–1)	34 (27–40)	25 (21–29)
Seychelles	2033	1 (1–1)	1 (1–1)	34 (28–41)	25 (21–29)
Seychelles	2034	1 (1–1)	1 (1–1)	35 (28–42)	25 (21–29)
Seychelles	2035	1 (1–2)	1 (1–1)	35 (28–43)	25 (21–28)
Seychelles	2036	1 (1–2)	1 (1–1)	36 (28–43)	25 (21–28)
Sierra Leone	2022	4 (4–4)	0 (0–0)	106 (104–107)	3 (2–3)

Sierra Leone	2023	4 (4–4)	0 (0–0)	108 (105–112)	3 (2–3)
Sierra Leone	2024	4 (4–4)	0 (0–0)	111 (105–117)	3 (2–3)
Sierra Leone	2025	4 (4–4)	0 (0–0)	114 (105–123)	2 (2–3)
Sierra Leone	2026	4 (4–4)	0 (0–0)	116 (104–128)	2 (2–3)
Sierra Leone	2027	4 (4–5)	0 (0–0)	119 (103–135)	2 (2–3)
Sierra Leone	2028	4 (4–5)	0 (0–0)	122 (102–141)	2 (2–3)
Sierra Leone	2029	4 (4–5)	0 (0–0)	124 (101–148)	2 (2–3)
Sierra Leone	2030	4 (4–5)	0 (0–0)	127 (99–155)	2 (2–3)
Sierra Leone	2031	5 (4–5)	0 (0–0)	130 (97–162)	2 (2–3)
Sierra Leone	2032	5 (3–6)	0 (0–0)	132 (95–169)	2 (2–3)
Sierra Leone	2033	5 (3–6)	0 (0–0)	135 (93–177)	2 (2–3)
Sierra Leone	2034	5 (3–6)	0 (0–0)	137 (91–184)	2 (2–3)
Sierra Leone	2035	5 (3–6)	0 (0–0)	140 (88–192)	2 (2–3)
Sierra Leone	2036	5 (3–7)	0 (0–0)	143 (85–201)	2 (2–3)
Singapore	2022	24 (23–25)	0 (0–0)	624 (588–660)	7 (6–7)
Singapore	2023	24 (21–26)	0 (0–0)	620 (558–681)	6 (5–7)
Singapore	2024	24 (20–27)	0 (0–0)	618 (535–701)	6 (4–7)
Singapore	2025	23 (20–27)	0 (0–0)	617 (516–718)	5 (4–7)
Singapore	2026	23 (19–28)	0 (0–0)	617 (500–734)	5 (3–7)
Singapore	2027	23 (18–28)	0 (0–0)	617 (486–748)	5 (3–7)
Singapore	2028	23 (18–29)	0 (0–0)	617 (473–760)	4 (2–7)
Singapore	2029	23 (17–29)	0 (0–0)	617 (462–772)	4 (1–6)
Singapore	2030	23 (17–30)	0 (0–0)	617 (451–783)	4 (1–6)
Singapore	2031	23 (16–30)	0 (0–0)	617 (441–793)	3 (0–6)
Singapore	2032	23 (16–31)	0 (-0–0)	617 (431–803)	3 (-0–6)
Singapore	2033	23 (16–31)	0 (-0–0)	617 (422–812)	2 (-1–6)
Singapore	2034	23 (15–32)	0 (-0–0)	617 (413–821)	2 (-1–5)
Singapore	2035	23 (15–32)	0 (-0–0)	617 (405–829)	2 (-2–5)
Singapore	2036	23 (15–32)	0 (-0–0)	617 (397–837)	1 (-2–5)
Slovakia	2022	116 (112–119)	1 (1–1)	2970 (2866–3075)	31 (30–33)
Slovakia	2023	117 (112–122)	1 (1–1)	2986 (2839–3134)	31 (29–33)
Slovakia	2024	118 (112–124)	1 (1–1)	3002 (2821–3184)	31 (28–33)
Slovakia	2025	119 (111–126)	1 (1–1)	3019 (2809–3228)	30 (28–33)

Slovakia	2026	119 (112–127)	1 (1–1)	3035 (2801–3268)	30 (27–33)
Slovakia	2027	120 (112–129)	1 (1–1)	3051 (2795–3307)	30 (26–33)
Slovakia	2028	121 (112–130)	1 (1–1)	3067 (2790–3343)	30 (26–33)
Slovakia	2029	122 (112–132)	1 (1–1)	3083 (2787–3379)	29 (25–33)
Slovakia	2030	123 (112–133)	1 (1–1)	3099 (2785–3413)	29 (25–33)
Slovakia	2031	124 (113–135)	1 (1–1)	3115 (2784–3446)	29 (24–33)
Slovakia	2032	125 (113–136)	1 (1–1)	3131 (2784–3478)	28 (23–33)
Slovakia	2033	125 (113–138)	1 (1–1)	3147 (2785–3509)	28 (23–33)
Slovakia	2034	126 (114–139)	1 (1–1)	3163 (2786–3540)	28 (22–33)
Slovakia	2035	127 (114–140)	1 (1–1)	3179 (2788–3571)	27 (22–33)
Slovakia	2036	128 (114–142)	1 (1–1)	3196 (2791–3600)	27 (21–33)
Slovenia	2022	24 (22–26)	1 (0–1)	612 (554–671)	15 (13–17)
Slovenia	2023	24 (20–27)	1 (0–1)	616 (536–696)	14 (11–17)
Slovenia	2024	23 (19–27)	1 (0–1)	620 (524–715)	14 (10–17)
Slovenia	2025	22 (17–28)	0 (0–1)	623 (516–730)	13 (8–17)
Slovenia	2026	22 (15–28)	0 (0–1)	627 (511–743)	12 (7–18)
Slovenia	2027	21 (14–29)	0 (0–1)	630 (506–753)	11 (5–18)
Slovenia	2028	21 (12–29)	0 (0–1)	633 (503–762)	11 (4–18)
Slovenia	2029	20 (10–30)	0 (0–1)	635 (500–770)	10 (2–18)
Slovenia	2030	20 (8–31)	0 (0–1)	638 (498–777)	9 (0–18)
Slovenia	2031	19 (7–31)	0 (-0–1)	640 (497–783)	9 (-2–19)
Slovenia	2032	18 (5–32)	0 (-0–1)	642 (496–789)	8 (-3–19)
Slovenia	2033	18 (3–33)	0 (-0–1)	645 (495–794)	7 (-5–19)
Slovenia	2034	17 (1–34)	0 (-0–1)	647 (495–798)	6 (-7–20)
Slovenia	2035	17 (-1–35)	0 (-0–1)	648 (495–802)	6 (-9–20)
Slovenia	2036	16 (-3–35)	0 (-0–1)	650 (495–806)	5 (-10–20)
Solomon Islands	2022	2 (2–2)	0 (0–0)	57 (56–59)	14 (13–14)
Solomon Islands	2023	2 (2–2)	0 (0–0)	59 (57–61)	13 (13–14)
Solomon Islands	2024	2 (2–2)	0 (0–0)	60 (57–64)	13 (12–14)
Solomon Islands	2025	2 (2–2)	0 (0–1)	62 (57–66)	13 (12–14)
Solomon Islands	2026	2 (2–2)	0 (0–1)	63 (57–69)	13 (12–14)
Solomon Islands	2027	2 (2–2)	0 (0–1)	65 (57–72)	13 (12–14)
Solomon Islands	2028	2 (2–2)	0 (0–1)	66 (57–76)	13 (12–14)

Solomon Islands	2029	2 (2–2)	0 (0–1)	68 (56–79)	13 (12–14)
Solomon Islands	2030	2 (2–2)	0 (0–1)	69 (56–82)	13 (11–14)
Solomon Islands	2031	2 (2–3)	0 (0–1)	71 (55–86)	13 (11–14)
Solomon Islands	2032	2 (2–3)	0 (0–1)	72 (55–90)	13 (11–14)
Solomon Islands	2033	2 (2–3)	0 (0–1)	74 (54–93)	13 (11–14)
Solomon Islands	2034	2 (2–3)	0 (0–1)	75 (53–97)	13 (11–14)
Solomon Islands	2035	2 (2–3)	0 (0–1)	76 (52–101)	13 (11–14)
Solomon Islands	2036	3 (2–3)	0 (0–1)	78 (51–105)	13 (11–14)
Somalia	2022	11 (11–12)	0 (0–0)	355 (351–359)	5 (5–5)
Somalia	2023	11 (11–12)	0 (0–0)	359 (350–368)	5 (5–5)
Somalia	2024	11 (11–12)	0 (0–0)	363 (348–379)	5 (5–5)
Somalia	2025	12 (11–12)	0 (0–0)	367 (345–390)	5 (5–5)
Somalia	2026	12 (11–13)	0 (0–0)	372 (341–402)	5 (5–5)
Somalia	2027	12 (10–13)	0 (0–0)	376 (337–414)	5 (4–5)
Somalia	2028	12 (10–13)	0 (0–0)	380 (332–428)	5 (4–5)
Somalia	2029	12 (10–14)	0 (0–0)	384 (326–442)	5 (4–5)
Somalia	2030	12 (9–14)	0 (0–0)	388 (319–457)	5 (4–5)
Somalia	2031	12 (9–14)	0 (0–0)	392 (312–472)	5 (4–5)
Somalia	2032	12 (9–15)	0 (0–0)	396 (305–488)	5 (4–5)
Somalia	2033	12 (8–15)	0 (0–0)	400 (297–504)	4 (4–5)
Somalia	2034	12 (8–16)	0 (0–0)	404 (288–521)	4 (4–5)
Somalia	2035	12 (7–16)	0 (0–0)	408 (279–538)	4 (4–5)
Somalia	2036	12 (7–17)	0 (0–0)	413 (269–556)	4 (4–5)
South Africa	2022	154 (148–160)	0 (0–0)	4580 (4403–4757)	9 (8–9)
South Africa	2023	157 (148–165)	0 (0–0)	4644 (4394–4895)	9 (8–10)
South Africa	2024	159 (148–169)	0 (0–0)	4709 (4402–5016)	9 (8–10)
South Africa	2025	161 (149–173)	0 (0–0)	4773 (4418–5128)	9 (8–10)
South Africa	2026	163 (150–176)	0 (0–0)	4837 (4441–5234)	9 (7–10)
South Africa	2027	165 (151–180)	0 (0–0)	4902 (4467–5336)	9 (7–10)
South Africa	2028	167 (151–183)	0 (0–0)	4966 (4497–5435)	9 (7–10)
South Africa	2029	170 (153–186)	0 (0–0)	5030 (4529–5532)	9 (7–11)
South Africa	2030	172 (154–190)	0 (0–0)	5095 (4563–5627)	9 (7–11)
South Africa	2031	174 (155–193)	0 (0–0)	5159 (4598–5720)	9 (7–11)

South Africa	2032	176 (156–196)	0 (0–0)	5223 (4635–5811)	9 (7–11)
South Africa	2033	178 (157–199)	0 (0–0)	5288 (4673–5902)	9 (7–11)
South Africa	2034	180 (159–202)	0 (0–0)	5352 (4713–5991)	9 (7–11)
South Africa	2035	182 (160–205)	0 (0–0)	5416 (4753–6080)	9 (7–11)
South Africa	2036	185 (161–208)	0 (0–0)	5481 (4794–6167)	9 (6–11)
South Sudan	2022	10 (10–10)	0 (0–0)	286 (281–290)	7 (6–7)
South Sudan	2023	10 (10–10)	0 (0–0)	292 (283–301)	7 (6–7)
South Sudan	2024	10 (10–11)	0 (0–0)	297 (283–311)	7 (6–7)
South Sudan	2025	10 (9–11)	0 (0–0)	303 (283–323)	7 (6–7)
South Sudan	2026	10 (9–11)	0 (0–0)	309 (283–335)	7 (6–7)
South Sudan	2027	10 (9–12)	0 (0–0)	315 (282–347)	7 (6–7)
South Sudan	2028	10 (9–12)	0 (0–0)	320 (280–360)	7 (5–8)
South Sudan	2029	11 (8–13)	0 (0–0)	326 (278–373)	6 (5–8)
South Sudan	2030	11 (8–13)	0 (0–0)	332 (276–387)	6 (5–8)
South Sudan	2031	11 (8–14)	0 (0–0)	337 (273–402)	6 (5–8)
South Sudan	2032	11 (7–14)	0 (0–0)	343 (270–416)	6 (4–8)
South Sudan	2033	11 (7–15)	0 (0–0)	349 (266–431)	6 (4–9)
South Sudan	2034	11 (7–15)	0 (0–0)	355 (263–447)	6 (4–9)
South Sudan	2035	11 (6–16)	0 (0–0)	360 (258–463)	6 (4–9)
South Sudan	2036	11 (6–17)	0 (0–0)	366 (254–479)	6 (3–9)
Spain	2022	682 (662–702)	1 (1–1)	16213 (15587–16839)	19 (18–20)
Spain	2023	670 (635–704)	1 (1–1)	15885 (14826–16944)	18 (17–19)
Spain	2024	657 (606–708)	1 (1–1)	15557 (14042–17071)	17 (15–19)
Spain	2025	644 (575–712)	1 (1–1)	15228 (13225–17232)	17 (14–19)
Spain	2026	631 (544–719)	1 (1–1)	14900 (12373–17427)	16 (13–19)
Spain	2027	618 (510–726)	1 (0–1)	14572 (11488–17656)	15 (11–19)
Spain	2028	605 (476–735)	1 (0–1)	14244 (10570–17918)	14 (10–19)
Spain	2029	593 (440–745)	1 (0–1)	13915 (9620–18211)	14 (8–19)
Spain	2030	580 (403–756)	1 (0–1)	13587 (8641–18534)	13 (7–19)
Spain	2031	567 (365–769)	0 (0–1)	13259 (7632–18886)	12 (5–19)
Spain	2032	554 (326–782)	0 (0–1)	12931 (6595–19266)	12 (4–20)
Spain	2033	541 (286–797)	0 (0–1)	12603 (5532–19673)	11 (2–20)
Spain	2034	529 (245–812)	0 (0–1)	12274 (4443–20106)	10 (1–20)

Spain	2035	516 (203–829)	0 (0–1)	11946 (3328–20564)	10 (-1–20)
Spain	2036	503 (160–846)	0 (-0–1)	11618 (2190–21046)	9 (-3–20)
Sri Lanka	2022	40 (37–42)	0 (0–0)	998 (940–1057)	3 (3–4)
Sri Lanka	2023	40 (37–43)	0 (0–0)	1010 (927–1092)	3 (3–4)
Sri Lanka	2024	41 (37–45)	0 (0–0)	1021 (920–1122)	3 (3–4)
Sri Lanka	2025	41 (37–46)	0 (0–0)	1032 (916–1149)	3 (3–4)
Sri Lanka	2026	42 (37–47)	0 (0–0)	1044 (913–1174)	3 (2–4)
Sri Lanka	2027	42 (37–48)	0 (0–0)	1055 (912–1198)	3 (2–4)
Sri Lanka	2028	43 (37–49)	0 (0–0)	1066 (912–1220)	3 (2–4)
Sri Lanka	2029	43 (37–49)	0 (0–0)	1078 (913–1242)	3 (2–4)
Sri Lanka	2030	44 (37–50)	0 (0–0)	1089 (914–1264)	3 (2–4)
Sri Lanka	2031	44 (37–51)	0 (0–0)	1100 (916–1284)	3 (2–4)
Sri Lanka	2032	45 (37–52)	0 (0–0)	1112 (918–1305)	3 (2–4)
Sri Lanka	2033	45 (37–53)	0 (0–0)	1123 (921–1325)	3 (2–4)
Sri Lanka	2034	46 (37–54)	0 (0–0)	1134 (924–1344)	3 (1–4)
Sri Lanka	2035	46 (38–54)	0 (0–0)	1145 (928–1363)	3 (1–4)
Sri Lanka	2036	46 (38–55)	0 (0–0)	1157 (931–1382)	3 (1–4)
Sudan	2022	31 (31–31)	0 (0–0)	930 (926–935)	4 (4–4)
Sudan	2023	32 (31–32)	0 (0–0)	955 (945–966)	4 (4–4)
Sudan	2024	32 (32–33)	0 (0–0)	980 (963–998)	4 (4–4)
Sudan	2025	33 (32–34)	0 (0–0)	1006 (980–1031)	4 (4–4)
Sudan	2026	34 (33–35)	0 (0–0)	1031 (996–1066)	4 (4–4)
Sudan	2027	35 (33–36)	0 (0–0)	1056 (1011–1101)	4 (4–4)
Sudan	2028	35 (33–37)	0 (0–0)	1081 (1025–1137)	4 (4–4)
Sudan	2029	36 (34–38)	0 (0–0)	1106 (1039–1173)	4 (4–4)
Sudan	2030	37 (34–39)	0 (0–0)	1131 (1051–1211)	4 (4–4)
Sudan	2031	37 (34–41)	0 (0–0)	1156 (1064–1249)	4 (4–4)
Sudan	2032	38 (35–42)	0 (0–0)	1181 (1075–1287)	4 (4–4)
Sudan	2033	39 (35–43)	0 (0–0)	1206 (1086–1327)	4 (4–4)
Sudan	2034	40 (35–44)	0 (0–0)	1231 (1096–1367)	4 (4–4)
Sudan	2035	40 (35–45)	0 (0–0)	1257 (1106–1407)	4 (4–4)
Sudan	2036	41 (36–47)	0 (0–0)	1282 (1115–1448)	4 (4–4)
Suriname	2022	3 (3–3)	1 (0–1)	92 (88–97)	14 (13–15)

Suriname	2023	3 (3–4)	1 (0–1)	94 (85–104)	15 (13–17)
Suriname	2024	3 (3–4)	1 (0–1)	96 (83–108)	15 (13–18)
Suriname	2025	3 (3–4)	1 (0–1)	97 (82–113)	16 (13–18)
Suriname	2026	4 (3–4)	1 (0–1)	99 (82–116)	16 (13–19)
Suriname	2027	4 (3–4)	1 (0–1)	101 (81–120)	16 (14–19)
Suriname	2028	4 (3–4)	1 (0–1)	102 (81–124)	16 (14–19)
Suriname	2029	4 (3–5)	1 (0–1)	104 (81–127)	16 (14–19)
Suriname	2030	4 (3–5)	1 (0–1)	106 (81–130)	16 (14–19)
Suriname	2031	4 (3–5)	1 (1–1)	107 (82–133)	16 (14–19)
Suriname	2032	4 (3–5)	1 (1–1)	109 (82–136)	16 (14–19)
Suriname	2033	4 (3–5)	1 (1–1)	111 (82–139)	16 (14–19)
Suriname	2034	4 (3–5)	1 (1–1)	112 (83–142)	16 (14–19)
Suriname	2035	4 (3–5)	1 (1–1)	114 (83–145)	16 (14–19)
Suriname	2036	4 (3–5)	1 (1–1)	116 (84–148)	16 (14–19)
Sweden	2022	130 (125–134)	1 (1–1)	2693 (2582–2805)	14 (13–14)
Sweden	2023	129 (120–137)	1 (1–1)	2673 (2475–2872)	13 (12–15)
Sweden	2024	128 (117–140)	1 (0–1)	2664 (2391–2937)	13 (11–15)
Sweden	2025	128 (113–142)	1 (0–1)	2659 (2321–2998)	13 (11–15)
Sweden	2026	128 (110–145)	1 (0–1)	2657 (2262–3053)	12 (10–14)
Sweden	2027	128 (108–147)	1 (0–1)	2656 (2210–3102)	12 (9–14)
Sweden	2028	127 (106–149)	0 (0–1)	2656 (2164–3148)	12 (9–14)
Sweden	2029	127 (104–151)	0 (0–1)	2656 (2121–3190)	11 (8–14)
Sweden	2030	127 (102–153)	0 (0–1)	2656 (2082–3229)	11 (8–14)
Sweden	2031	127 (100–155)	0 (0–1)	2656 (2045–3266)	11 (7–14)
Sweden	2032	127 (99–156)	0 (0–1)	2656 (2010–3301)	10 (7–14)
Sweden	2033	127 (97–158)	0 (0–1)	2656 (1977–3334)	10 (6–13)
Sweden	2034	127 (96–159)	0 (0–1)	2656 (1946–3365)	9 (6–13)
Sweden	2035	127 (94–161)	0 (0–1)	2656 (1916–3395)	9 (5–13)
Sweden	2036	127 (93–162)	0 (0–1)	2656 (1887–3424)	9 (5–13)
Switzerland	2022	90 (86–94)	0 (0–1)	1997 (1902–2092)	11 (11–12)
Switzerland	2023	90 (84–96)	0 (0–1)	2031 (1887–2175)	11 (10–12)
Switzerland	2024	90 (83–97)	0 (0–1)	2054 (1884–2225)	11 (9–12)
Switzerland	2025	90 (82–98)	0 (0–1)	2070 (1887–2253)	11 (9–12)

Switzerland	2026	90 (81–99)	0 (0–1)	2079 (1891–2267)	10 (8–12)
Switzerland	2027	90 (80–100)	0 (0–1)	2085 (1895–2275)	10 (8–12)
Switzerland	2028	90 (80–101)	0 (0–1)	2088 (1897–2279)	10 (8–12)
Switzerland	2029	90 (79–101)	0 (0–0)	2089 (1898–2280)	10 (7–12)
Switzerland	2030	90 (78–102)	0 (0–0)	2090 (1899–2281)	9 (7–12)
Switzerland	2031	90 (77–103)	0 (0–0)	2090 (1899–2282)	9 (6–12)
Switzerland	2032	90 (77–103)	0 (0–0)	2090 (1899–2282)	9 (6–12)
Switzerland	2033	90 (76–104)	0 (0–0)	2090 (1899–2282)	8 (5–11)
Switzerland	2034	90 (76–104)	0 (0–0)	2090 (1899–2282)	8 (5–11)
Switzerland	2035	90 (75–105)	0 (0–0)	2090 (1899–2282)	8 (5–11)
Switzerland	2036	90 (75–105)	0 (0–0)	2090 (1899–2282)	8 (4–11)
Syrian Arab Republic	2022	33 (32–33)	0 (0–0)	935 (907–962)	6 (6–7)
Syrian Arab Republic	2023	33 (31–35)	0 (0–0)	948 (898–998)	6 (6–7)
Syrian Arab Republic	2024	34 (31–36)	0 (0–0)	961 (892–1030)	6 (5–7)
Syrian Arab Republic	2025	34 (31–37)	0 (0–0)	975 (889–1061)	6 (5–7)
Syrian Arab Republic	2026	35 (31–38)	0 (0–0)	988 (887–1089)	6 (5–7)
Syrian Arab Republic	2027	35 (31–39)	0 (0–0)	1002 (887–1116)	6 (5–8)
Syrian Arab Republic	2028	36 (31–40)	0 (0–0)	1015 (889–1142)	6 (5–8)
Syrian Arab Republic	2029	36 (32–41)	0 (0–0)	1029 (891–1166)	6 (5–8)
Syrian Arab Republic	2030	37 (32–41)	0 (0–0)	1042 (894–1190)	6 (4–8)
Syrian Arab Republic	2031	37 (32–42)	0 (0–0)	1056 (898–1213)	6 (4–8)
Syrian Arab Republic	2032	38 (32–43)	0 (0–0)	1069 (902–1236)	6 (4–8)
Syrian Arab Republic	2033	38 (32–44)	0 (0–0)	1083 (907–1258)	6 (4–8)
Syrian Arab Republic	2034	39 (33–45)	0 (0–0)	1096 (913–1280)	6 (4–8)
Syrian Arab Republic	2035	39 (33–45)	0 (0–0)	1110 (918–1301)	6 (4–9)
Syrian Arab Republic	2036	40 (33–46)	0 (0–0)	1123 (924–1322)	6 (4–9)
Taiwan (Province of China)	2022	389 (372–406)	1 (1–1)	9633 (9127–10138)	23 (21–25)
Taiwan (Province of China)	2023	397 (373–421)	1 (1–1)	9806 (9092–10521)	23 (20–26)
Taiwan (Province of China)	2024	405 (376–435)	1 (1–1)	9980 (9105–10856)	23 (20–27)
Taiwan (Province of China)	2025	413 (379–447)	1 (1–1)	10154 (9143–11165)	23 (19–27)
Taiwan (Province of China)	2026	421 (383–459)	1 (1–1)	10327 (9197–11458)	23 (18–28)
Taiwan (Province of China)	2027	429 (387–470)	1 (1–1)	10501 (9263–11739)	23 (18–28)
Taiwan (Province of China)	2028	437 (392–481)	1 (1–1)	10675 (9337–12012)	23 (18–29)

Taiwan (Province of China)	2029	444 (396–492)	1 (1–1)	10849 (9419–12278)	23 (17–29)
Taiwan (Province of China)	2030	452 (401–503)	1 (1–1)	11022 (9506–12539)	23 (17–30)
Taiwan (Province of China)	2031	460 (406–514)	1 (1–1)	11196 (9597–12794)	23 (17–30)
Taiwan (Province of China)	2032	468 (412–524)	1 (1–1)	11370 (9693–13046)	23 (16–30)
Taiwan (Province of China)	2033	476 (417–535)	1 (1–1)	11543 (9792–13294)	23 (16–31)
Taiwan (Province of China)	2034	484 (422–545)	1 (1–1)	11717 (9894–13540)	23 (16–31)
Taiwan (Province of China)	2035	491 (428–555)	1 (1–1)	11891 (9999–13782)	23 (15–31)
Taiwan (Province of China)	2036	499 (434–565)	1 (1–1)	12064 (10106–14022)	23 (15–31)
Tajikistan	2022	7 (7–8)	0 (0–0)	239 (228–249)	3 (3–4)
Tajikistan	2023	8 (7–8)	0 (0–0)	245 (226–264)	3 (3–4)
Tajikistan	2024	8 (7–9)	0 (0–0)	252 (222–282)	3 (2–4)
Tajikistan	2025	8 (7–9)	0 (0–0)	258 (216–301)	3 (2–4)
Tajikistan	2026	8 (6–10)	0 (0–0)	265 (209–321)	3 (1–5)
Tajikistan	2027	8 (6–11)	0 (0–0)	271 (200–343)	3 (1–5)
Tajikistan	2028	9 (6–12)	0 (0–0)	278 (191–365)	3 (0–6)
Tajikistan	2029	9 (5–12)	0 (0–0)	285 (180–389)	3 (-0–6)
Tajikistan	2030	9 (5–13)	0 (0–0)	291 (168–414)	3 (-1–7)
Tajikistan	2031	9 (4–14)	0 (-0–0)	298 (156–440)	3 (-2–7)
Tajikistan	2032	9 (4–15)	0 (-0–0)	304 (142–466)	3 (-2–8)
Tajikistan	2033	10 (3–16)	0 (-0–0)	311 (128–494)	3 (-3–9)
Tajikistan	2034	10 (3–17)	0 (-0–0)	317 (113–522)	3 (-4–9)
Tajikistan	2035	10 (2–18)	0 (-0–0)	324 (97–552)	3 (-4–10)
Tajikistan	2036	10 (2–19)	0 (-0–0)	331 (80–581)	3 (-5–11)
Thailand	2022	866 (845–888)	1 (1–1)	22279 (21692–22866)	20 (19–21)
Thailand	2023	898 (862–935)	1 (1–1)	22919 (22000–23837)	20 (19–21)
Thailand	2024	931 (879–982)	1 (1–1)	23558 (22323–24794)	20 (18–21)
Thailand	2025	963 (895–1031)	1 (1–1)	24198 (22643–25753)	20 (18–22)
Thailand	2026	995 (909–1081)	1 (1–1)	24838 (22953–26722)	20 (18–22)
Thailand	2027	1027 (923–1132)	1 (1–1)	25477 (23252–27703)	20 (18–22)
Thailand	2028	1060 (935–1184)	1 (1–1)	26117 (23538–28696)	20 (18–22)
Thailand	2029	1092 (947–1237)	1 (1–1)	26757 (23812–29702)	20 (18–22)
Thailand	2030	1124 (957–1291)	1 (1–1)	27397 (24073–30720)	20 (17–23)
Thailand	2031	1156 (966–1346)	1 (1–1)	28036 (24321–31752)	20 (17–23)

Thailand	2032	1189 (975–1402)	1 (1–1)	28676 (24556–32796)	20 (17–23)
Thailand	2033	1221 (982–1459)	1 (1–1)	29316 (24779–33852)	20 (17–23)
Thailand	2034	1253 (989–1517)	1 (1–1)	29955 (24990–34920)	20 (17–23)
Thailand	2035	1285 (995–1576)	1 (1–1)	30595 (25190–36001)	20 (17–23)
Thailand	2036	1318 (1000–1635)	1 (1–1)	31235 (25377–37092)	20 (17–23)
Timor-Leste	2022	4 (3–4)	0 (0–0)	94 (93–95)	10 (10–11)
Timor-Leste	2023	4 (3–4)	0 (0–0)	96 (94–98)	10 (10–11)
Timor-Leste	2024	4 (4–4)	0 (0–0)	98 (94–102)	10 (10–11)
Timor-Leste	2025	4 (4–4)	0 (0–0)	100 (95–105)	10 (10–11)
Timor-Leste	2026	4 (4–4)	0 (0–0)	102 (95–109)	10 (9–12)
Timor-Leste	2027	4 (4–4)	0 (0–0)	104 (95–113)	10 (9–12)
Timor-Leste	2028	4 (4–4)	0 (0–0)	106 (95–117)	10 (9–12)
Timor-Leste	2029	4 (4–4)	0 (0–0)	108 (95–122)	10 (8–13)
Timor-Leste	2030	4 (3–5)	0 (0–1)	110 (94–126)	10 (8–13)
Timor-Leste	2031	4 (3–5)	0 (0–1)	112 (94–131)	11 (7–14)
Timor-Leste	2032	4 (3–5)	0 (0–1)	114 (93–135)	11 (7–14)
Timor-Leste	2033	4 (3–5)	0 (0–1)	116 (93–140)	11 (7–14)
Timor-Leste	2034	4 (3–5)	0 (0–1)	118 (92–145)	11 (6–15)
Timor-Leste	2035	4 (3–5)	0 (0–1)	120 (91–150)	11 (6–15)
Timor-Leste	2036	4 (3–6)	0 (0–1)	122 (90–155)	11 (5–16)
Togo	2022	4 (4–4)	0 (0–0)	131 (129–132)	3 (3–3)
Togo	2023	5 (4–5)	0 (0–0)	135 (132–139)	3 (3–3)
Togo	2024	5 (5–5)	0 (0–0)	140 (135–146)	3 (3–3)
Togo	2025	5 (5–5)	0 (0–0)	145 (137–153)	3 (3–3)
Togo	2026	5 (5–5)	0 (0–0)	150 (139–160)	3 (3–3)
Togo	2027	5 (5–6)	0 (0–0)	154 (140–168)	3 (3–3)
Togo	2028	5 (5–6)	0 (0–0)	159 (142–176)	3 (3–3)
Togo	2029	6 (5–6)	0 (0–0)	164 (143–185)	3 (3–3)
Togo	2030	6 (5–6)	0 (0–0)	168 (144–193)	3 (3–3)
Togo	2031	6 (5–7)	0 (0–0)	173 (145–202)	3 (3–3)
Togo	2032	6 (5–7)	0 (0–0)	178 (145–211)	3 (3–3)
Togo	2033	6 (5–7)	0 (0–0)	183 (145–220)	3 (3–3)
Togo	2034	6 (5–8)	0 (0–0)	187 (146–229)	3 (3–3)

Togo	2035	6 (5–8)	0 (0–0)	192 (146–239)	3 (3–3)
Togo	2036	7 (5–8)	0 (0–0)	197 (145–248)	3 (3–3)
Tokelau	2022	0 (0–0)	0 (0–0)	0 (0–0)	10 (9–10)
Tokelau	2023	0 (0–0)	0 (0–0)	0 (0–0)	9 (9–10)
Tokelau	2024	0 (0–0)	0 (0–0)	0 (0–0)	9 (9–10)
Tokelau	2025	0 (0–0)	0 (0–0)	0 (0–0)	9 (9–10)
Tokelau	2026	0 (0–0)	0 (0–0)	0 (0–0)	9 (9–10)
Tokelau	2027	0 (0–0)	0 (0–0)	0 (0–0)	9 (9–10)
Tokelau	2028	0 (0–0)	0 (0–0)	0 (0–0)	9 (9–10)
Tokelau	2029	0 (0–0)	0 (0–0)	0 (0–0)	9 (9–10)
Tokelau	2030	0 (0–0)	0 (0–0)	0 (0–0)	9 (8–10)
Tokelau	2031	0 (0–0)	0 (0–0)	0 (0–0)	9 (8–10)
Tokelau	2032	0 (0–0)	0 (0–0)	0 (0–0)	9 (8–10)
Tokelau	2033	0 (0–0)	0 (0–0)	0 (0–0)	9 (8–10)
Tokelau	2034	0 (0–0)	0 (0–0)	0 (0–0)	9 (8–10)
Tokelau	2035	0 (0–0)	0 (0–0)	0 (0–0)	9 (8–9)
Tokelau	2036	0 (0–0)	0 (0–0)	0 (0–0)	9 (8–9)
Tonga	2022	0 (0–0)	0 (0–0)	6 (6–6)	8 (7–8)
Tonga	2023	0 (0–0)	0 (0–0)	6 (6–7)	8 (7–8)
Tonga	2024	0 (0–0)	0 (0–0)	6 (6–7)	8 (7–8)
Tonga	2025	0 (0–0)	0 (0–0)	6 (6–7)	8 (7–8)
Tonga	2026	0 (0–0)	0 (0–0)	6 (6–7)	8 (7–8)
Tonga	2027	0 (0–0)	0 (0–0)	6 (5–7)	8 (7–9)
Tonga	2028	0 (0–0)	0 (0–0)	6 (5–7)	8 (6–9)
Tonga	2029	0 (0–0)	0 (0–0)	6 (5–7)	8 (6–9)
Tonga	2030	0 (0–0)	0 (0–0)	6 (5–7)	8 (6–9)
Tonga	2031	0 (0–0)	0 (0–0)	6 (5–7)	8 (6–9)
Tonga	2032	0 (0–0)	0 (0–0)	6 (5–7)	8 (6–9)
Tonga	2033	0 (0–0)	0 (0–0)	6 (5–7)	8 (6–9)
Tonga	2034	0 (0–0)	0 (0–0)	6 (5–7)	8 (6–9)
Tonga	2035	0 (0–0)	0 (0–0)	6 (5–7)	8 (6–9)
Tonga	2036	0 (0–0)	0 (0–0)	6 (5–7)	8 (6–9)
Trinidad and Tobago	2022	10 (9–10)	0 (0–1)	268 (252–283)	13 (12–15)

Trinidad and Tobago	2023	10 (9–11)	0 (0–1)	272 (250–294)	13 (12–15)
Trinidad and Tobago	2024	10 (9–11)	0 (0–1)	276 (249–304)	13 (11–15)
Trinidad and Tobago	2025	10 (9–11)	0 (0–1)	281 (249–312)	13 (11–16)
Trinidad and Tobago	2026	11 (9–12)	0 (0–1)	285 (250–321)	13 (11–16)
Trinidad and Tobago	2027	11 (9–12)	0 (0–1)	290 (251–328)	13 (11–16)
Trinidad and Tobago	2028	11 (9–12)	0 (0–1)	294 (252–336)	13 (10–17)
Trinidad and Tobago	2029	11 (9–13)	0 (0–1)	298 (254–343)	13 (10–17)
Trinidad and Tobago	2030	11 (10–13)	0 (0–1)	303 (255–350)	13 (10–17)
Trinidad and Tobago	2031	11 (10–13)	0 (0–1)	307 (257–357)	13 (10–17)
Trinidad and Tobago	2032	12 (10–13)	0 (0–1)	312 (259–364)	13 (9–17)
Trinidad and Tobago	2033	12 (10–14)	0 (0–1)	316 (261–371)	13 (9–18)
Trinidad and Tobago	2034	12 (10–14)	0 (0–1)	320 (263–378)	13 (9–18)
Trinidad and Tobago	2035	12 (10–14)	0 (0–1)	325 (266–384)	13 (9–18)
Trinidad and Tobago	2036	12 (10–14)	0 (0–1)	329 (268–391)	13 (9–18)
Tunisia	2022	50 (50–51)	0 (0–0)	1276 (1264–1288)	9 (9–9)
Tunisia	2023	52 (51–52)	0 (0–0)	1306 (1286–1326)	9 (9–9)
Tunisia	2024	53 (51–54)	0 (0–0)	1330 (1300–1361)	9 (8–9)
Tunisia	2025	54 (52–55)	0 (0–0)	1355 (1317–1393)	9 (8–10)
Tunisia	2026	54 (53–56)	0 (0–0)	1379 (1335–1423)	9 (8–10)
Tunisia	2027	55 (53–58)	0 (0–0)	1404 (1354–1453)	9 (8–10)
Tunisia	2028	56 (54–59)	0 (0–0)	1428 (1374–1483)	9 (7–10)
Tunisia	2029	57 (55–60)	0 (0–0)	1453 (1394–1512)	9 (7–10)
Tunisia	2030	58 (56–61)	0 (0–0)	1477 (1414–1541)	9 (7–10)
Tunisia	2031	59 (57–62)	0 (0–0)	1502 (1435–1569)	9 (6–11)
Tunisia	2032	60 (57–63)	0 (0–0)	1527 (1456–1597)	9 (6–11)
Tunisia	2033	61 (58–64)	0 (0–0)	1551 (1477–1626)	8 (6–11)
Tunisia	2034	62 (59–66)	0 (0–0)	1576 (1498–1653)	8 (5–12)
Tunisia	2035	63 (60–67)	0 (0–1)	1600 (1519–1681)	8 (5–12)
Tunisia	2036	64 (61–68)	0 (0–1)	1625 (1540–1709)	8 (4–12)
Turkey	2022	571 (560–583)	1 (1–1)	16006 (15704–16307)	16 (15–16)
Turkey	2023	577 (551–603)	1 (1–1)	16200 (15527–16874)	16 (15–17)
Turkey	2024	583 (540–626)	1 (0–1)	16395 (15268–17522)	16 (14–17)
Turkey	2025	588 (525–651)	1 (0–1)	16590 (14940–18240)	15 (13–18)

Turkey	2026	594 (508–679)	1 (0–1)	16784 (14550–19019)	15 (12–19)
Turkey	2027	599 (490–709)	1 (0–1)	16979 (14105–19853)	15 (10–20)
Turkey	2028	605 (469–741)	1 (0–1)	17174 (13609–20738)	15 (9–20)
Turkey	2029	610 (446–774)	1 (0–1)	17368 (13065–21671)	15 (8–21)
Turkey	2030	616 (422–810)	1 (0–1)	17563 (12477–22649)	14 (6–22)
Turkey	2031	621 (396–847)	0 (0–1)	17758 (11846–23669)	14 (5–24)
Turkey	2032	627 (368–885)	0 (0–1)	17952 (11176–24729)	14 (3–25)
Turkey	2033	632 (339–925)	0 (0–1)	18147 (10466–25828)	14 (1–26)
Turkey	2034	638 (309–967)	0 (-0–1)	18342 (9720–26964)	13 (-0–27)
Turkey	2035	643 (277–1010)	0 (-0–1)	18537 (8938–28135)	13 (-2–29)
Turkey	2036	649 (244–1054)	0 (-0–1)	18731 (8122–29340)	13 (-4–30)
Turkmenistan	2022	6 (5–6)	0 (0–0)	190 (172–207)	4 (3–5)
Turkmenistan	2023	6 (5–6)	0 (0–0)	190 (165–214)	4 (3–5)
Turkmenistan	2024	6 (5–7)	0 (0–0)	190 (159–220)	4 (3–5)
Turkmenistan	2025	6 (5–7)	0 (0–0)	190 (154–225)	4 (2–5)
Turkmenistan	2026	6 (5–7)	0 (0–0)	190 (150–229)	4 (2–5)
Turkmenistan	2027	6 (5–7)	0 (0–0)	190 (146–233)	3 (2–5)
Turkmenistan	2028	6 (4–7)	0 (0–0)	190 (143–236)	3 (2–5)
Turkmenistan	2029	6 (4–7)	0 (0–0)	190 (140–239)	3 (1–5)
Turkmenistan	2030	6 (4–7)	0 (0–0)	190 (137–242)	3 (1–5)
Turkmenistan	2031	6 (4–7)	0 (0–0)	190 (134–245)	3 (1–5)
Turkmenistan	2032	6 (4–7)	0 (0–0)	190 (131–248)	3 (1–5)
Turkmenistan	2033	6 (4–8)	0 (0–0)	190 (129–250)	3 (1–5)
Turkmenistan	2034	6 (4–8)	0 (0–0)	190 (126–253)	3 (0–5)
Turkmenistan	2035	6 (4–8)	0 (0–0)	190 (124–255)	3 (0–5)
Turkmenistan	2036	6 (4–8)	0 (0–0)	190 (121–258)	2 (-0–5)
Tuvalu	2022	0 (0–0)	0 (0–0)	1 (1–1)	13 (13–13)
Tuvalu	2023	0 (0–0)	0 (0–0)	1 (1–1)	13 (13–13)
Tuvalu	2024	0 (0–0)	0 (0–0)	1 (1–2)	13 (12–13)
Tuvalu	2025	0 (0–0)	0 (0–0)	1 (1–2)	13 (12–13)
Tuvalu	2026	0 (0–0)	0 (0–0)	2 (1–2)	13 (12–13)
Tuvalu	2027	0 (0–0)	0 (0–0)	2 (1–2)	13 (12–13)
Tuvalu	2028	0 (0–0)	0 (0–0)	2 (1–2)	13 (12–13)

Tuvalu	2029	0 (0–0)	0 (0–0)	2 (1–2)	13 (12–13)
Tuvalu	2030	0 (0–0)	0 (0–0)	2 (1–2)	13 (12–13)
Tuvalu	2031	0 (0–0)	0 (0–0)	2 (1–2)	13 (12–13)
Tuvalu	2032	0 (0–0)	0 (0–0)	2 (1–2)	13 (12–13)
Tuvalu	2033	0 (0–0)	0 (0–0)	2 (1–2)	13 (12–13)
Tuvalu	2034	0 (0–0)	0 (0–0)	2 (1–2)	13 (12–13)
Tuvalu	2035	0 (0–0)	0 (0–0)	2 (1–2)	13 (12–14)
Tuvalu	2036	0 (0–0)	0 (0–0)	2 (1–2)	13 (12–14)
Uganda	2022	27 (27–27)	0 (0–0)	767 (754–779)	5 (4–5)
Uganda	2023	27 (27–28)	0 (0–0)	783 (759–808)	5 (4–5)
Uganda	2024	28 (26–29)	0 (0–0)	803 (761–845)	5 (4–5)
Uganda	2025	28 (26–30)	0 (0–0)	819 (757–880)	5 (4–5)
Uganda	2026	29 (26–32)	0 (0–0)	836 (752–919)	4 (3–6)
Uganda	2027	29 (25–33)	0 (0–0)	850 (743–958)	4 (3–6)
Uganda	2028	29 (25–34)	0 (0–0)	865 (732–998)	4 (3–6)
Uganda	2029	30 (24–35)	0 (0–0)	879 (719–1038)	4 (2–6)
Uganda	2030	30 (23–36)	0 (0–0)	892 (705–1079)	4 (2–7)
Uganda	2031	30 (23–38)	0 (0–0)	904 (690–1119)	4 (2–7)
Uganda	2032	30 (22–39)	0 (0–0)	917 (673–1160)	4 (1–7)
Uganda	2033	31 (21–40)	0 (0–0)	928 (655–1201)	4 (1–8)
Uganda	2034	31 (20–41)	0 (0–0)	939 (636–1241)	4 (0–8)
Uganda	2035	31 (19–43)	0 (-0–0)	949 (616–1282)	4 (-0–9)
Uganda	2036	31 (19–44)	0 (-0–0)	959 (595–1322)	4 (-1–9)
Ukraine	2022	507 (455–559)	1 (1–1)	13841 (12345–15336)	19 (17–21)
Ukraine	2023	507 (433–580)	1 (1–1)	13628 (11514–15743)	19 (16–22)
Ukraine	2024	507 (417–597)	1 (1–1)	13416 (10827–16006)	18 (15–22)
Ukraine	2025	507 (403–611)	1 (0–1)	13204 (10214–16195)	18 (14–22)
Ukraine	2026	507 (391–623)	1 (0–1)	12992 (9649–16335)	18 (13–22)
Ukraine	2027	507 (379–634)	1 (0–1)	12780 (9118–16442)	17 (12–23)
Ukraine	2028	507 (369–644)	1 (0–1)	12568 (8612–16524)	17 (11–23)
Ukraine	2029	507 (360–654)	1 (0–1)	12356 (8127–16585)	17 (11–23)
Ukraine	2030	507 (351–662)	1 (0–1)	12144 (7659–16629)	16 (10–23)
Ukraine	2031	507 (342–671)	1 (0–1)	11932 (7204–16660)	16 (9–23)

Ukraine	2032	507 (334–679)	1 (0–1)	11720 (6761–16679)	16 (9–23)
Ukraine	2033	507 (327–687)	1 (0–1)	11508 (6329–16687)	15 (8–23)
Ukraine	2034	507 (319–694)	1 (0–1)	11296 (5905–16686)	15 (8–23)
Ukraine	2035	507 (312–701)	1 (0–1)	11084 (5489–16678)	15 (7–23)
Ukraine	2036	507 (306–708)	1 (0–1)	10872 (5081–16662)	14 (6–23)
United Arab Emirates	2022	10 (9–11)	0 (0–0)	352 (321–383)	5 (4–6)
United Arab Emirates	2023	10 (9–11)	0 (0–0)	361 (325–396)	5 (3–6)
United Arab Emirates	2024	10 (9–11)	0 (0–0)	369 (329–409)	4 (2–6)
United Arab Emirates	2025	11 (9–12)	0 (0–1)	377 (334–421)	3 (0–6)
United Arab Emirates	2026	11 (10–12)	0 (0–1)	386 (339–433)	3 (-1–6)
United Arab Emirates	2027	11 (10–12)	0 (0–1)	394 (344–444)	2 (-2–6)
United Arab Emirates	2028	11 (10–12)	0 (0–1)	403 (349–456)	2 (-3–7)
United Arab Emirates	2029	11 (10–13)	0 (0–1)	411 (355–467)	1 (-5–7)
United Arab Emirates	2030	12 (10–13)	0 (0–1)	419 (361–478)	0 (-6–7)
United Arab Emirates	2031	12 (11–13)	0 (0–1)	428 (366–489)	-0 (-7–7)
United Arab Emirates	2032	12 (11–14)	0 (0–1)	436 (372–500)	-1 (-9–7)
United Arab Emirates	2033	12 (11–14)	0 (0–1)	445 (378–511)	-1 (-10–7)
United Arab Emirates	2034	13 (11–14)	0 (0–1)	453 (384–522)	-2 (-11–7)
United Arab Emirates	2035	13 (11–14)	0 (0–1)	461 (390–532)	-3 (-13–8)
United Arab Emirates	2036	13 (11–15)	0 (0–1)	470 (397–543)	-3 (-14–8)
United Kingdom	2022	953 (923–984)	1 (1–1)	20482 (19733–21232)	17 (16–18)
United Kingdom	2023	947 (896–998)	1 (1–1)	20284 (19046–21522)	17 (15–18)
United Kingdom	2024	941 (869–1013)	1 (1–1)	20086 (18348–21825)	16 (14–18)
United Kingdom	2025	934 (840–1029)	1 (1–1)	19888 (17621–22156)	16 (13–18)
United Kingdom	2026	928 (809–1047)	1 (1–1)	19690 (16861–22519)	15 (12–19)
United Kingdom	2027	922 (778–1066)	1 (0–1)	19492 (16069–22915)	15 (11–19)
United Kingdom	2028	915 (744–1086)	1 (0–1)	19294 (15245–23342)	14 (10–19)
United Kingdom	2029	909 (710–1108)	1 (0–1)	19096 (14391–23801)	14 (8–20)
United Kingdom	2030	903 (674–1132)	1 (0–1)	18898 (13506–24289)	14 (7–20)
United Kingdom	2031	896 (637–1156)	1 (0–1)	18700 (12593–24807)	13 (6–21)
United Kingdom	2032	890 (598–1182)	1 (0–1)	18501 (11651–25352)	13 (4–21)
United Kingdom	2033	884 (559–1209)	1 (0–1)	18303 (10683–25924)	12 (3–22)
United Kingdom	2034	877 (518–1237)	1 (0–1)	18105 (9688–26523)	12 (1–22)

United Kingdom	2035	871 (476–1266)	0 (0–1)	17907 (8668–27147)	11 (-0–23)
United Kingdom	2036	865 (433–1296)	0 (0–1)	17709 (7623–27795)	11 (-2–24)
United Republic of Tanzania	2022	87 (87–88)	0 (0–0)	2450 (2428–2473)	9 (8–9)
United Republic of Tanzania	2023	90 (88–92)	0 (0–0)	2526 (2476–2576)	9 (8–9)
United Republic of Tanzania	2024	92 (89–95)	0 (0–0)	2602 (2519–2686)	9 (8–9)
United Republic of Tanzania	2025	94 (89–99)	0 (0–0)	2678 (2556–2800)	9 (8–9)
United Republic of Tanzania	2026	96 (90–103)	0 (0–0)	2754 (2589–2920)	9 (8–9)
United Republic of Tanzania	2027	98 (90–107)	0 (0–0)	2830 (2617–3043)	9 (8–9)
United Republic of Tanzania	2028	101 (90–111)	0 (0–0)	2906 (2642–3170)	9 (8–10)
United Republic of Tanzania	2029	103 (90–115)	0 (0–0)	2982 (2663–3301)	9 (8–10)
United Republic of Tanzania	2030	105 (90–119)	0 (0–0)	3058 (2681–3435)	9 (8–10)
United Republic of Tanzania	2031	107 (90–124)	0 (0–0)	3134 (2696–3572)	9 (8–10)
United Republic of Tanzania	2032	109 (90–129)	0 (0–0)	3210 (2708–3712)	9 (7–10)
United Republic of Tanzania	2033	111 (90–133)	0 (0–0)	3286 (2716–3855)	9 (7–10)
United Republic of Tanzania	2034	114 (89–138)	0 (0–0)	3361 (2723–4000)	9 (7–10)
United Republic of Tanzania	2035	116 (88–143)	0 (0–0)	3437 (2726–4148)	9 (7–10)
United Republic of Tanzania	2036	118 (88–148)	0 (0–0)	3513 (2727–4299)	9 (7–10)
United States of America	2022	3140 (3078–3203)	1 (1–1)	81429 (79792–83065)	15 (15–16)
United States of America	2023	3140 (3029–3252)	1 (0–1)	81429 (78522–84335)	15 (14–16)
United States of America	2024	3140 (2996–3285)	1 (0–1)	81429 (77658–85199)	14 (13–16)
United States of America	2025	3140 (2969–3312)	0 (0–1)	81429 (76958–85899)	14 (13–15)
United States of America	2026	3140 (2945–3335)	0 (0–1)	81429 (76353–86504)	14 (12–15)
United States of America	2027	3140 (2925–3356)	0 (0–1)	81429 (75813–87044)	13 (11–15)
United States of America	2028	3140 (2906–3375)	0 (0–1)	81429 (75321–87536)	13 (11–15)
United States of America	2029	3140 (2888–3392)	0 (0–0)	81429 (74866–87991)	12 (10–14)
United States of America	2030	3140 (2872–3409)	0 (0–0)	81429 (74440–88417)	12 (10–14)
United States of America	2031	3140 (2856–3424)	0 (0–0)	81429 (74039–88818)	12 (9–14)
United States of America	2032	3140 (2842–3439)	0 (0–0)	81429 (73658–89199)	11 (9–14)
United States of America	2033	3140 (2828–3453)	0 (0–0)	81429 (73295–89562)	11 (8–13)
United States of America	2034	3140 (2814–3466)	0 (0–0)	81429 (72948–89909)	10 (8–13)
United States of America	2035	3140 (2802–3479)	0 (0–0)	81429 (72614–90243)	10 (7–13)
United States of America	2036	3140 (2789–3491)	0 (0–0)	81429 (72293–90564)	9 (7–12)
United States Virgin Islands	2022	1 (1–1)	0 (0–0)	13 (12–14)	8 (7–9)

United States Virgin Islands	2023	1 (0–1)	0 (0–0)	12 (10–14)	7 (6–9)
United States Virgin Islands	2024	1 (0–1)	0 (0–0)	12 (9–14)	7 (5–9)
United States Virgin Islands	2025	1 (0–1)	0 (0–0)	11 (7–15)	6 (4–9)
United States Virgin Islands	2026	0 (0–1)	0 (0–0)	10 (6–15)	6 (3–9)
United States Virgin Islands	2027	0 (0–1)	0 (0–0)	9 (4–15)	5 (2–9)
United States Virgin Islands	2028	0 (0–1)	0 (0–0)	9 (2–15)	5 (1–9)
United States Virgin Islands	2029	0 (0–1)	0 (-0–0)	8 (0–16)	4 (-1–10)
United States Virgin Islands	2030	0 (0–1)	0 (-0–0)	7 (-2–16)	4 (-2–10)
United States Virgin Islands	2031	0 (0–1)	0 (-0–0)	7 (-4–17)	4 (-3–10)
United States Virgin Islands	2032	0 (-0–1)	0 (-0–0)	6 (-6–17)	3 (-4–10)
United States Virgin Islands	2033	0 (-0–1)	0 (-0–0)	5 (-8–18)	3 (-6–11)
United States Virgin Islands	2034	0 (-0–1)	0 (-0–0)	4 (-10–18)	2 (-7–11)
United States Virgin Islands	2035	0 (-0–1)	0 (-0–0)	4 (-12–19)	2 (-9–12)
United States Virgin Islands	2036	0 (-0–1)	0 (-0–1)	3 (-14–20)	1 (-10–12)
Uruguay	2022	68 (65–71)	1 (1–1)	1649 (1576–1722)	33 (32–35)
Uruguay	2023	69 (64–73)	1 (1–1)	1659 (1556–1762)	33 (31–36)
Uruguay	2024	69 (64–75)	1 (1–1)	1669 (1543–1796)	33 (30–36)
Uruguay	2025	70 (64–76)	1 (1–1)	1680 (1533–1826)	33 (30–37)
Uruguay	2026	70 (64–77)	1 (1–1)	1690 (1526–1853)	33 (30–37)
Uruguay	2027	71 (64–78)	1 (1–1)	1700 (1521–1879)	33 (29–37)
Uruguay	2028	72 (64–80)	1 (1–1)	1710 (1517–1904)	33 (29–38)
Uruguay	2029	72 (64–81)	1 (1–1)	1721 (1514–1927)	33 (29–38)
Uruguay	2030	73 (64–82)	1 (1–1)	1731 (1512–1950)	33 (28–38)
Uruguay	2031	73 (64–83)	1 (1–1)	1741 (1510–1972)	33 (28–38)
Uruguay	2032	74 (64–84)	1 (1–1)	1752 (1509–1994)	33 (28–39)
Uruguay	2033	75 (64–85)	1 (1–1)	1762 (1509–2015)	33 (28–39)
Uruguay	2034	75 (64–86)	1 (1–1)	1772 (1509–2036)	33 (27–39)
Uruguay	2035	76 (64–87)	1 (1–1)	1782 (1509–2056)	33 (27–39)
Uruguay	2036	76 (65–88)	1 (1–2)	1793 (1510–2076)	33 (27–40)
Uzbekistan	2022	42 (40–44)	0 (0–0)	1358 (1285–1432)	4 (4–4)
Uzbekistan	2023	43 (39–47)	0 (0–0)	1390 (1254–1525)	4 (4–5)
Uzbekistan	2024	44 (38–50)	0 (0–0)	1421 (1216–1627)	4 (3–5)
Uzbekistan	2025	45 (37–54)	0 (0–0)	1453 (1170–1736)	4 (3–5)

Uzbekistan	2026	46 (36–57)	0 (0–0)	1484 (1117–1852)	4 (3–5)
Uzbekistan	2027	48 (34–61)	0 (0–0)	1516 (1057–1975)	4 (3–5)
Uzbekistan	2028	49 (32–65)	0 (0–0)	1547 (990–2104)	4 (3–5)
Uzbekistan	2029	50 (30–69)	0 (0–0)	1579 (918–2239)	4 (3–5)
Uzbekistan	2030	51 (28–74)	0 (0–0)	1610 (840–2380)	4 (3–5)
Uzbekistan	2031	52 (25–78)	0 (0–0)	1642 (757–2527)	4 (3–5)
Uzbekistan	2032	53 (23–83)	0 (0–0)	1673 (668–2678)	4 (3–5)
Uzbekistan	2033	54 (20–88)	0 (0–0)	1704 (574–2834)	4 (3–5)
Uzbekistan	2034	55 (17–93)	0 (0–0)	1736 (476–2996)	4 (3–5)
Uzbekistan	2035	56 (14–98)	0 (0–0)	1767 (373–3162)	4 (3–5)
Uzbekistan	2036	57 (11–103)	0 (0–0)	1799 (266–3332)	4 (3–5)
Vanuatu	2022	1 (1–1)	0 (0–0)	16 (15–16)	8 (8–8)
Vanuatu	2023	1 (1–1)	0 (0–0)	16 (16–16)	8 (7–8)
Vanuatu	2024	1 (1–1)	0 (0–0)	16 (16–17)	8 (7–8)
Vanuatu	2025	1 (1–1)	0 (0–0)	16 (16–17)	8 (7–8)
Vanuatu	2026	1 (1–1)	0 (0–0)	17 (16–18)	7 (7–8)
Vanuatu	2027	1 (1–1)	0 (0–0)	17 (16–18)	7 (7–8)
Vanuatu	2028	1 (1–1)	0 (0–0)	17 (16–18)	7 (6–8)
Vanuatu	2029	1 (1–1)	0 (0–0)	18 (17–19)	7 (6–8)
Vanuatu	2030	1 (1–1)	0 (0–0)	18 (17–19)	7 (6–8)
Vanuatu	2031	1 (1–1)	0 (0–0)	18 (17–19)	7 (6–8)
Vanuatu	2032	1 (1–1)	0 (0–0)	18 (17–20)	7 (6–8)
Vanuatu	2033	1 (1–1)	0 (0–0)	19 (17–20)	7 (6–8)
Vanuatu	2034	1 (1–1)	0 (0–0)	19 (18–20)	7 (6–8)
Vanuatu	2035	1 (1–1)	0 (0–0)	19 (18–21)	7 (5–8)
Vanuatu	2036	1 (1–1)	0 (0–0)	19 (18–21)	7 (5–8)
Venezuela (Bolivarian Republic of)	2022	94 (90–98)	0 (0–0)	2550 (2433–2668)	8 (7–9)
Venezuela (Bolivarian Republic of)	2023	96 (90–102)	0 (0–0)	2599 (2464–2735)	8 (7–9)
Venezuela (Bolivarian Republic of)	2024	98 (90–105)	0 (0–0)	2649 (2497–2800)	8 (7–9)
Venezuela (Bolivarian Republic of)	2025	99 (91–108)	0 (0–0)	2698 (2532–2863)	8 (7–9)
Venezuela (Bolivarian Republic of)	2026	101 (92–111)	0 (0–0)	2747 (2568–2926)	8 (7–9)
Venezuela (Bolivarian Republic of)	2027	103 (93–113)	0 (0–0)	2796 (2605–2987)	8 (7–9)
Venezuela (Bolivarian Republic of)	2028	105 (94–116)	0 (0–0)	2845 (2643–3048)	8 (6–10)

Venezuela (Bolivarian Republic of)	2029	107 (95–119)	0 (0–0)	2895 (2681–3108)	8 (6–10)
Venezuela (Bolivarian Republic of)	2030	109 (96–121)	0 (0–0)	2944 (2720–3168)	8 (6–10)
Venezuela (Bolivarian Republic of)	2031	110 (97–124)	0 (0–0)	2993 (2759–3227)	8 (6–10)
Venezuela (Bolivarian Republic of)	2032	112 (99–126)	0 (0–0)	3042 (2799–3286)	8 (6–10)
Venezuela (Bolivarian Republic of)	2033	114 (100–129)	0 (0–0)	3091 (2839–3344)	8 (6–10)
Venezuela (Bolivarian Republic of)	2034	116 (101–131)	0 (0–0)	3141 (2879–3402)	8 (6–10)
Venezuela (Bolivarian Republic of)	2035	118 (102–133)	0 (0–0)	3190 (2920–3460)	8 (6–10)
Venezuela (Bolivarian Republic of)	2036	120 (104–136)	0 (0–0)	3239 (2961–3517)	8 (6–10)
Viet Nam	2022	794 (788–800)	1 (1–1)	22518 (22362–22674)	20 (20–20)
Viet Nam	2023	819 (808–831)	1 (1–1)	23093 (22745–23441)	20 (20–21)
Viet Nam	2024	844 (827–862)	1 (1–1)	23668 (23085–24251)	20 (20–21)
Viet Nam	2025	869 (845–894)	1 (1–1)	24243 (23389–25096)	20 (19–21)
Viet Nam	2026	895 (862–927)	1 (1–1)	24817 (23662–25973)	20 (19–21)
Viet Nam	2027	920 (879–960)	1 (1–1)	25392 (23906–26878)	20 (19–21)
Viet Nam	2028	945 (895–994)	1 (1–1)	25967 (24124–27811)	20 (19–22)
Viet Nam	2029	970 (910–1029)	1 (1–1)	26542 (24317–28767)	20 (18–22)
Viet Nam	2030	995 (925–1064)	1 (1–1)	27117 (24486–29747)	20 (18–22)
Viet Nam	2031	1020 (940–1100)	1 (1–1)	27692 (24634–30749)	20 (18–22)
Viet Nam	2032	1045 (954–1136)	1 (1–1)	28266 (24762–31771)	20 (17–23)
Viet Nam	2033	1070 (968–1172)	1 (1–1)	28841 (24869–32813)	20 (17–23)
Viet Nam	2034	1095 (981–1209)	1 (1–1)	29416 (24957–33875)	20 (17–23)
Viet Nam	2035	1120 (993–1246)	1 (1–1)	29991 (25027–34955)	20 (16–24)
Viet Nam	2036	1145 (1006–1284)	1 (1–1)	30566 (25079–36052)	20 (16–24)
Yemen	2022	39 (39–40)	0 (0–0)	1207 (1185–1230)	7 (7–7)
Yemen	2023	40 (39–41)	0 (0–0)	1245 (1207–1282)	7 (7–7)
Yemen	2024	42 (40–43)	0 (0–0)	1282 (1229–1334)	7 (7–7)
Yemen	2025	43 (41–45)	0 (0–0)	1319 (1251–1387)	7 (7–8)
Yemen	2026	44 (41–46)	0 (0–0)	1356 (1272–1441)	7 (7–8)
Yemen	2027	45 (42–48)	0 (0–0)	1394 (1291–1496)	7 (7–8)
Yemen	2028	46 (43–50)	0 (0–0)	1431 (1310–1552)	7 (7–8)
Yemen	2029	47 (43–51)	0 (0–0)	1468 (1328–1609)	7 (7–8)
Yemen	2030	48 (44–53)	0 (0–0)	1506 (1345–1666)	7 (7–8)
Yemen	2031	49 (44–55)	0 (0–0)	1543 (1361–1725)	7 (7–8)

Yemen	2032	51 (45–56)	0 (0–0)	1580 (1376–1784)	7 (7–8)
Yemen	2033	52 (45–58)	0 (0–0)	1617 (1391–1844)	7 (7–8)
Yemen	2034	53 (46–60)	0 (0–0)	1655 (1404–1905)	7 (7–8)
Yemen	2035	54 (46–62)	0 (0–0)	1692 (1417–1967)	7 (7–8)
Yemen	2036	55 (46–64)	0 (0–0)	1729 (1430–2029)	7 (7–8)
Zambia	2022	21 (21–22)	0 (0–0)	611 (604–618)	8 (8–8)
Zambia	2023	22 (21–22)	0 (0–0)	622 (606–638)	8 (8–8)
Zambia	2024	22 (21–23)	0 (0–0)	633 (606–659)	8 (7–8)
Zambia	2025	22 (20–24)	0 (0–0)	644 (605–683)	8 (7–8)
Zambia	2026	22 (20–24)	0 (0–0)	655 (602–708)	8 (6–9)
Zambia	2027	22 (20–25)	0 (0–0)	666 (598–734)	7 (6–9)
Zambia	2028	22 (19–26)	0 (0–0)	677 (593–761)	7 (6–9)
Zambia	2029	23 (18–27)	0 (0–0)	688 (586–790)	7 (5–9)
Zambia	2030	23 (18–28)	0 (0–0)	699 (579–819)	7 (5–9)
Zambia	2031	23 (17–29)	0 (0–0)	710 (570–850)	7 (4–10)
Zambia	2032	23 (17–30)	0 (0–0)	721 (561–881)	7 (3–10)
Zambia	2033	23 (16–31)	0 (0–0)	732 (551–914)	7 (3–10)
Zambia	2034	23 (15–32)	0 (0–0)	743 (539–947)	6 (2–11)
Zambia	2035	24 (14–33)	0 (0–0)	754 (527–981)	6 (2–11)
Zambia	2036	24 (13–34)	0 (-0–0)	765 (514–1016)	6 (1–11)
Zimbabwe	2022	32 (31–33)	0 (0–0)	944 (922–966)	12 (12–13)
Zimbabwe	2023	32 (30–33)	0 (0–0)	947 (900–995)	12 (11–13)
Zimbabwe	2024	32 (29–34)	0 (0–1)	950 (875–1025)	12 (10–13)
Zimbabwe	2025	32 (28–35)	0 (0–1)	952 (848–1057)	11 (9–14)
Zimbabwe	2026	31 (27–36)	0 (0–1)	955 (820–1089)	11 (8–14)
Zimbabwe	2027	31 (26–37)	0 (0–1)	956 (791–1121)	11 (7–15)
Zimbabwe	2028	31 (24–38)	0 (0–1)	958 (763–1153)	11 (6–16)
Zimbabwe	2029	31 (23–39)	0 (0–1)	959 (734–1185)	10 (5–16)
Zimbabwe	2030	31 (22–40)	0 (0–1)	960 (706–1215)	10 (3–17)
Zimbabwe	2031	31 (21–41)	1 (0–1)	961 (678–1245)	10 (2–18)
Zimbabwe	2032	31 (20–42)	1 (0–1)	962 (650–1274)	10 (0–19)
Zimbabwe	2033	31 (19–43)	1 (0–1)	963 (623–1303)	9 (-1–20)
Zimbabwe	2034	31 (18–43)	1 (0–1)	964 (597–1330)	9 (-3–21)

Zimbabwe	2035	31 (17–44)	1 (0–1)	964 (571–1357)	9 (-4–22)
Zimbabwe	2036	31 (16–45)	1 (0–1)	965 (547–1383)	9 (-6–23)

Supplement table.8 Overview of complete GWAS data used in study.

	Phenotype	Number of participants	Ancestry	Consortium /cohort	Author	Year of publication/ updates	PubMed ID
Exposure	Tobacco use	454.787	European	UK Biobank(UKBB)	Joshua D Backman et al.	2021	NA
Outcome	Esophageal cancer	1.277	European	FinnGen	UKA	2024	NA
	Stomach cancer	2.296	European	FinnGen	UKA	2024	NA
	Liver cancer	2.298	European	FinnGen	UKA	2024	NA
	Pancreatic cancer	3.139	European	FinnGen	UKA	2024	NA
	Colon and rectum cancer	7.698	European	FinnGen	UKA	2024	NA

Supplement table.9 Mendelian randomization analysis results of tobacco use and gastrointestinal cancer diseases.

							MR-presso			MR-Egger					Weighted median					IVW					Simple mode					Weighted mode			Cochran’s Q for heterogeneity (IV-Egger intercept for p					
Exposure	resource	outcome	resource	SNP	harmonise	F<10	ysis Casual	SE	pval	OR	OR_95LCI	OR_95UCI	SE	pval	OR	OR_95LCI	OR_95UCI	SE	pval	OR	OR_95LCI	OR_95UCI	SE	pval	OR	OR_95LCI	OR_95UCI	SE	pval	OR	OR_95LCI	OR_95UCI	SE	pval	Q	pval	SE	pval
Tobacco	UKB	Esophageal cancer		6	0	0	0,05	0,07	0,55	0,77	0,32	1,83	0,44	0,58	1,00	0,77	1,29	0,13	0,98	1,05	0,85	1,30	0,11	0,66	0,92	0,63	1,36	0,20	0,70	0,96	0,68	1,35	0,18	0,82	2,33	0,80	0,10	0,50
Tobacco	UKB	Stomach cancer		6	0	0	0,04	0,07	0,56	0,76	0,40	1,47	0,33	0,46	1,11	0,90	1,38	0,11	0,33	1,05	0,89	1,23	0,08	0,58	1,13	0,85	1,51	0,15	0,45	1,14	0,86	1,49	0,14	0,40	3,95	0,56	0,07	0,38
Tobacco	UKB	Liver cancer		6	0	0	0,03	0,07	0,70	0,96	0,50	1,86	0,34	0,92	1,03	0,84	1,27	0,11	0,78	1,03	0,88	1,21	0,08	0,72	1,10	0,80	1,50	0,16	0,59	0,90	0,65	1,26	0,17	0,57	3,81	0,58	0,07	0,85
Tobacco	UKB	Pancreatic cancer		6	0	0	0,10	0,05	0,12	1,22	0,68	2,20	0,30	0,55	1,10	0,91	1,32	0,09	0,32	1,11	0,96	1,27	0,07	0,15	1,11	0,87	1,41	0,12	0,45	1,09	0,86	1,40	0,12	0,50	2,96	0,71	0,06	0,76
Tobacco	UKB	Colon and rectum can		6	0	0	0,08	0,05	0,15	0,86	0,54	1,35	0,23	0,54	1,09	0,94	1,25	0,07	0,26	1,08	0,97	1,22	0,06	0,16	1,08	0,90	1,31	0,10	0,44	1,08	0,92	1,28	0,09	0,40	3,28	0,66	0,05	0,35

Supplementary Material

Supplementary Figures

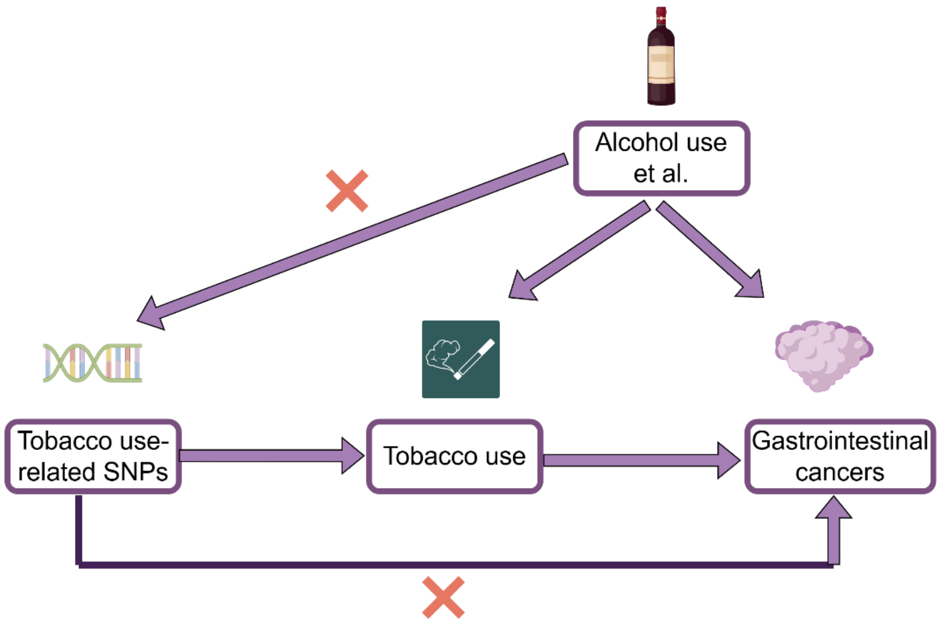

Supplement Figure 1. Study design of the two-sample Mendelian randomization for the effect of genetically predicted tobacco use on gastrointestinal cancers. SNPs, single nucleotide polymorphisms.

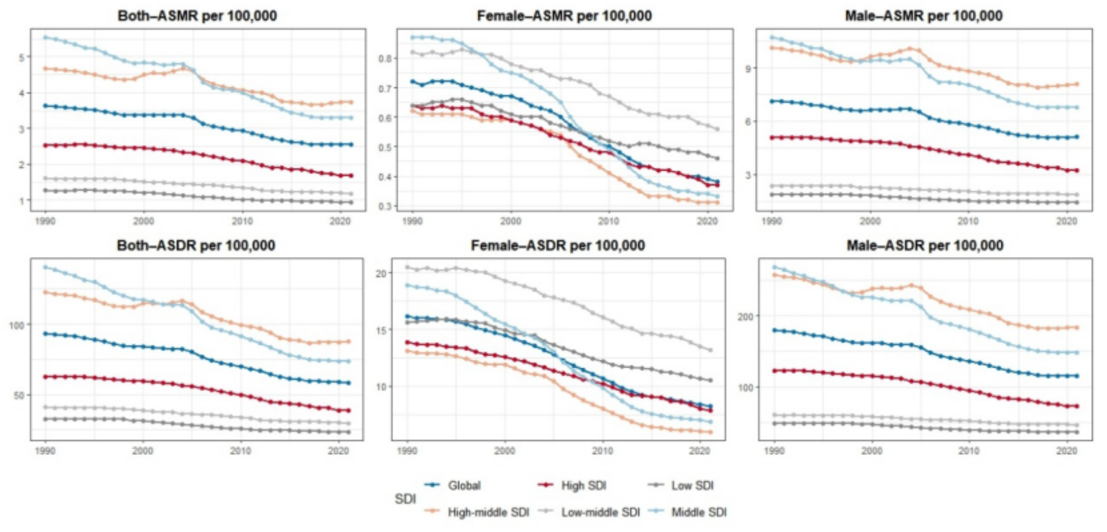

(A)

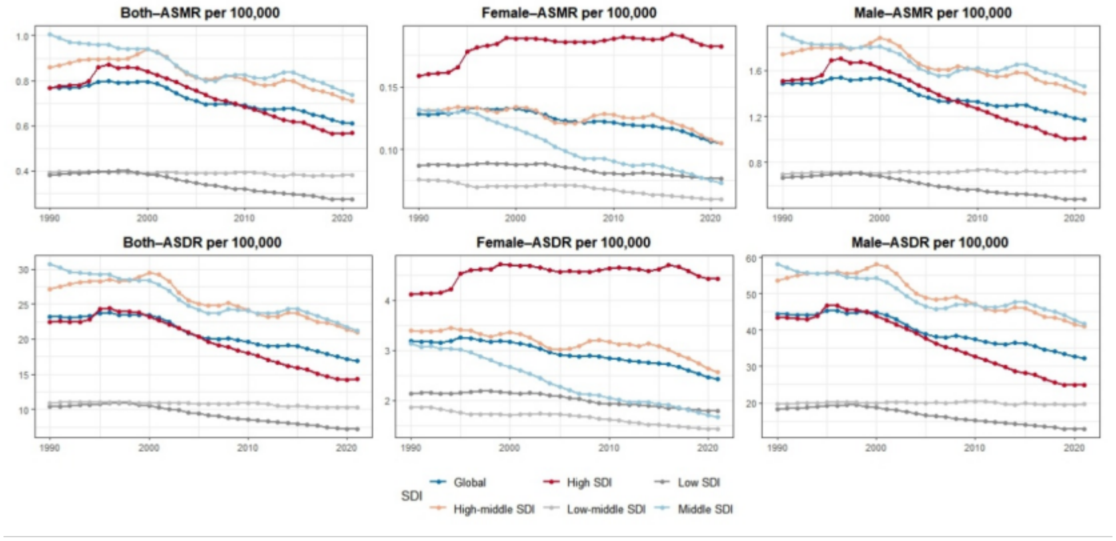
Both–ASMR per 100,000
Female–ASMR per 100,000
Male–ASMR per 100,000
Both–ASDR per 100,000
Female–ASDR per 100,000
Male–ASDR per 100,000
SDI
Global
High SDI
Low SDI
High-middle SDI
Low-middle SDI
Middle SDI

(B)

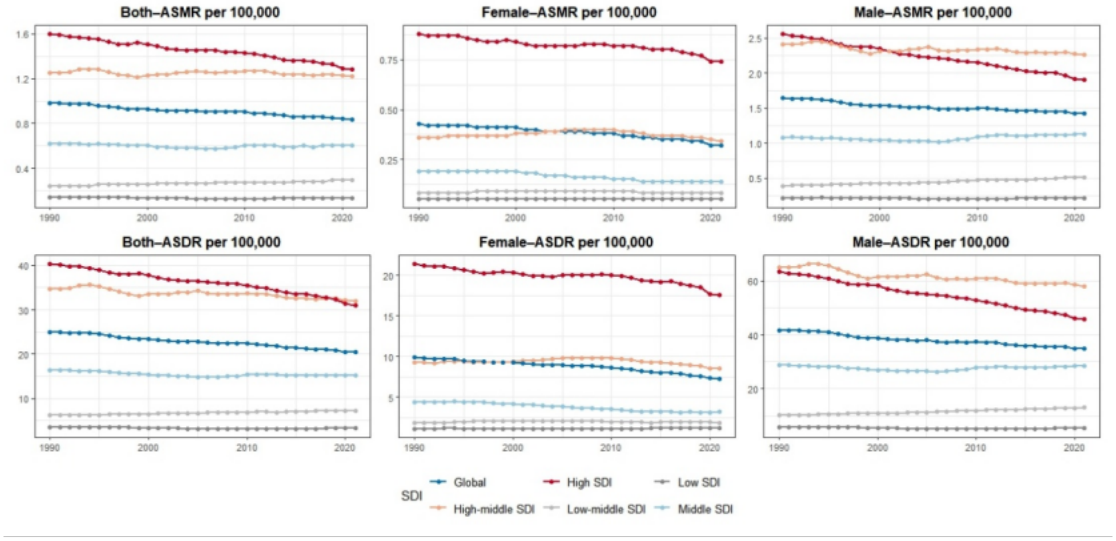
Both–ASMR per 100,000
Female–ASMR per 100,000
Male–ASMR per 100,000
Both–ASDR per 100,000
Female–ASDR per 100,000
Male–ASDR per 100,000
SDI
Global
High SDI
Low SDI
High-middle SDI
Low-middle SDI
Middle SDI

(C)

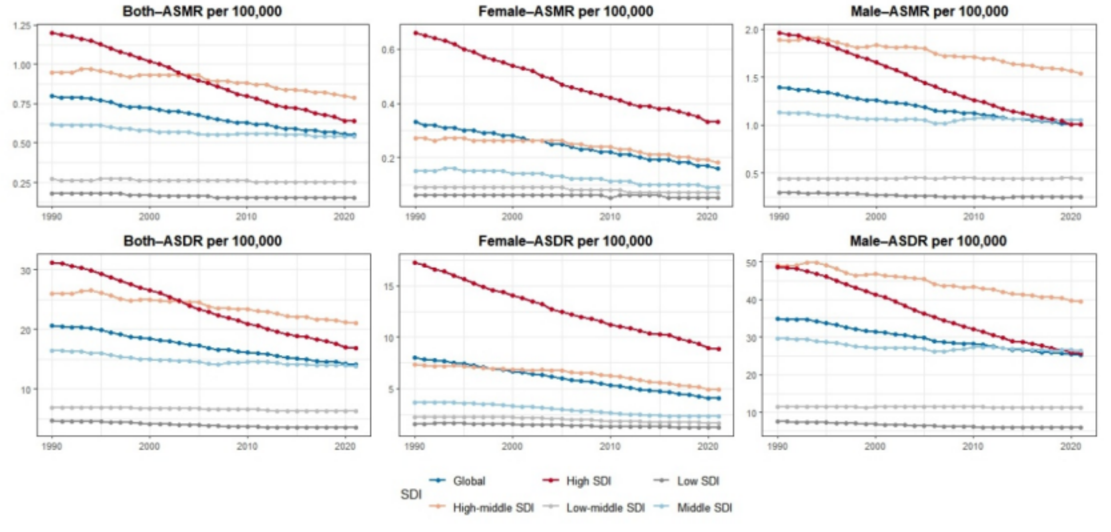

(D)

Supplement Figure 2. Temporal trends of esophageal cancer(A), liver cancer(B), pancreatic cancer(C), and colon and rectum cancer(D) burden attribute to tobacco use in terms of ASMR and ASDR per 100 000 population by sex, 1990–2021 in global, high SDI, high-middle SDI, middle SDI, low-middle SDI and low SDI. ASDR, age-standardised DALYs rate; ASMR, age-standardised mortality rate; DALYs, disability-adjusted life-years; SDI, Socio-Demographic Index.

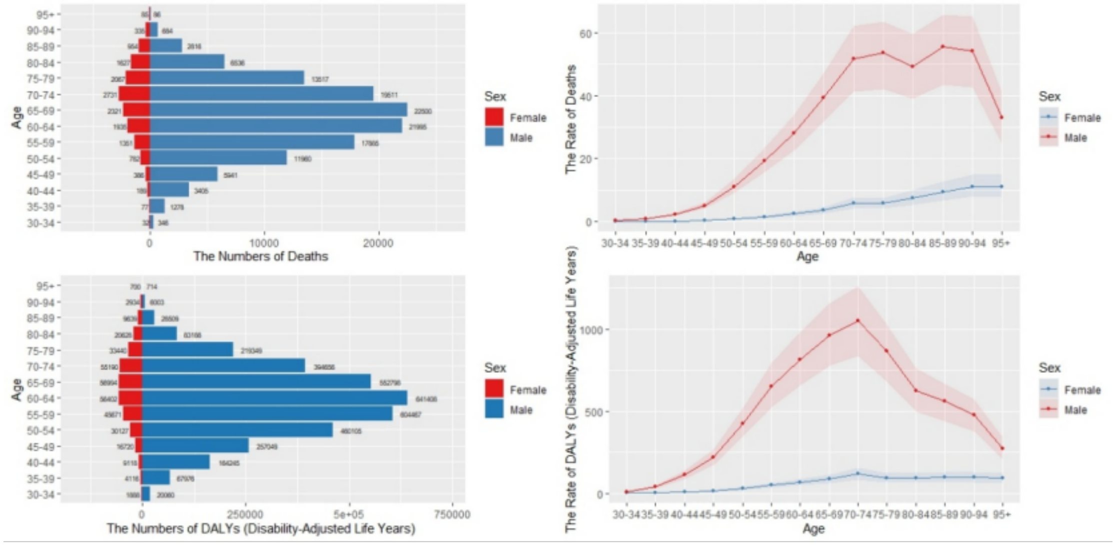

(A)

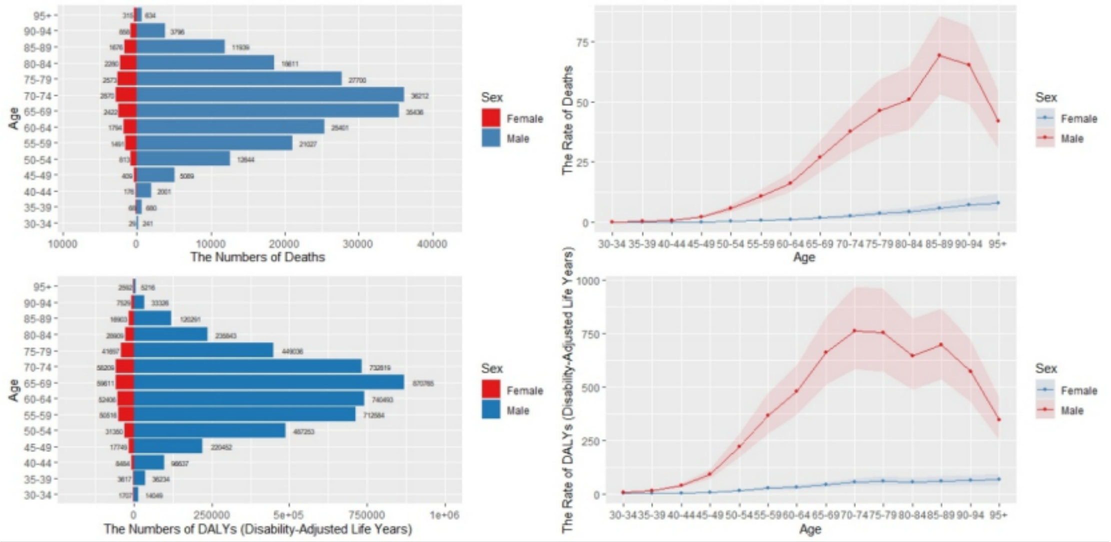

(B)

Supplement Figure 3.1. Global mortality (rate) and disability-adjusted life years (rate) attributable to tobacco use for stomach cancer, by age and sex, in 1990 (A) and 2021 (B).

Shaded areas now include a description indicating that they represent the 95% uncertainty intervals (UIs) around the estimated trends or regression lines.

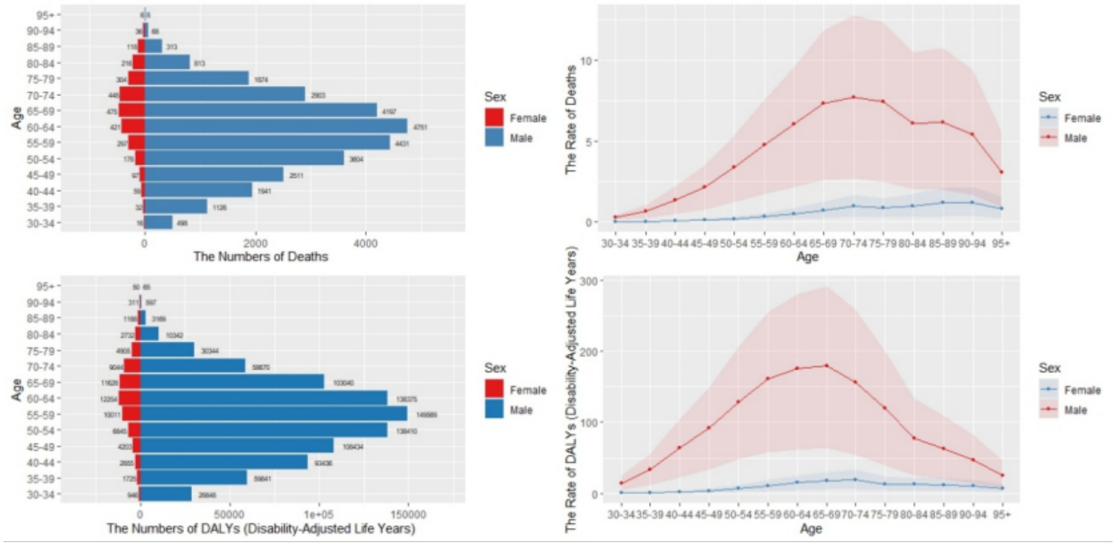

(A)

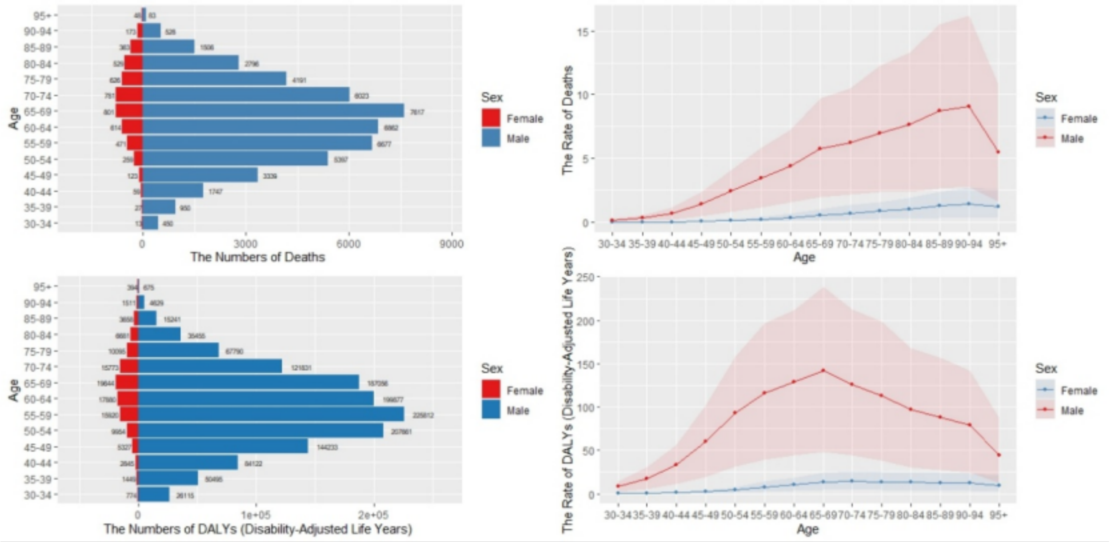

(B)

Supplement Figure 3.2. Global mortality (rate) and disability-adjusted life years (rate) attributable to tobacco use for liver cancer, by age and sex, in 1990 (A) and 2021 (B).

Shaded areas now include a description indicating that they represent the 95% uncertainty intervals (UIs) around the estimated trends or regression lines.

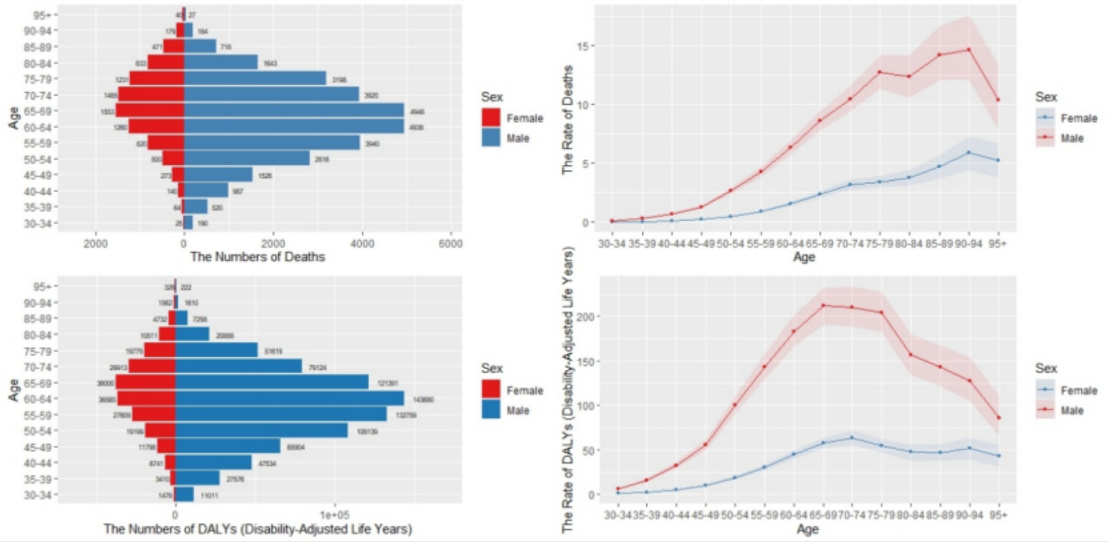

(A)

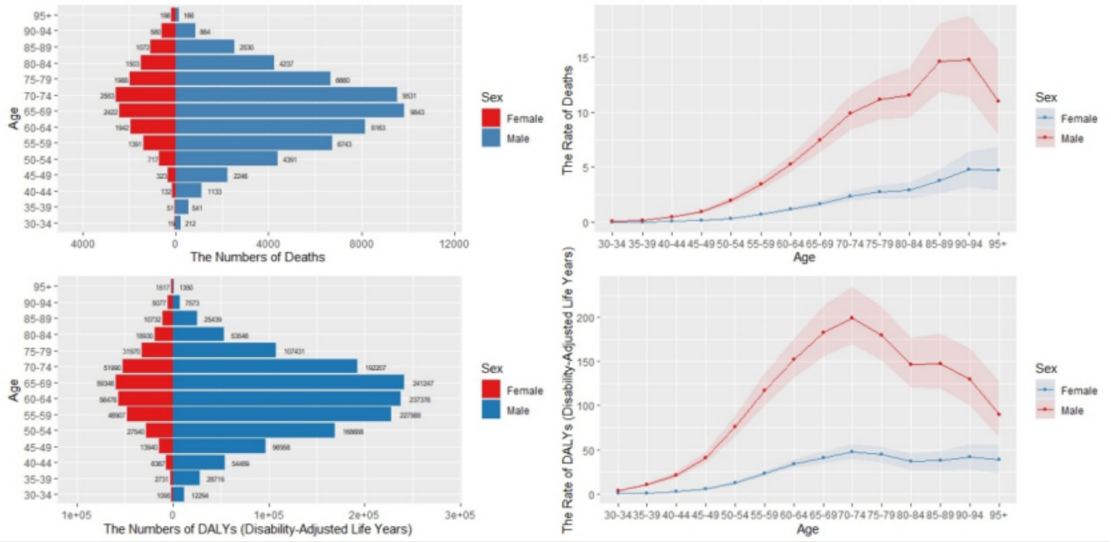

(B)

Supplement Figure 3.3. Global mortality (rate) and disability-adjusted life years (rate) attributable to tobacco use for pancreatic cancer, by age and sex, in 1990 (A) and 2021 (B).

Shaded areas now include a description indicating that they represent the 95% uncertainty intervals (UIs) around the estimated trends or regression lines.

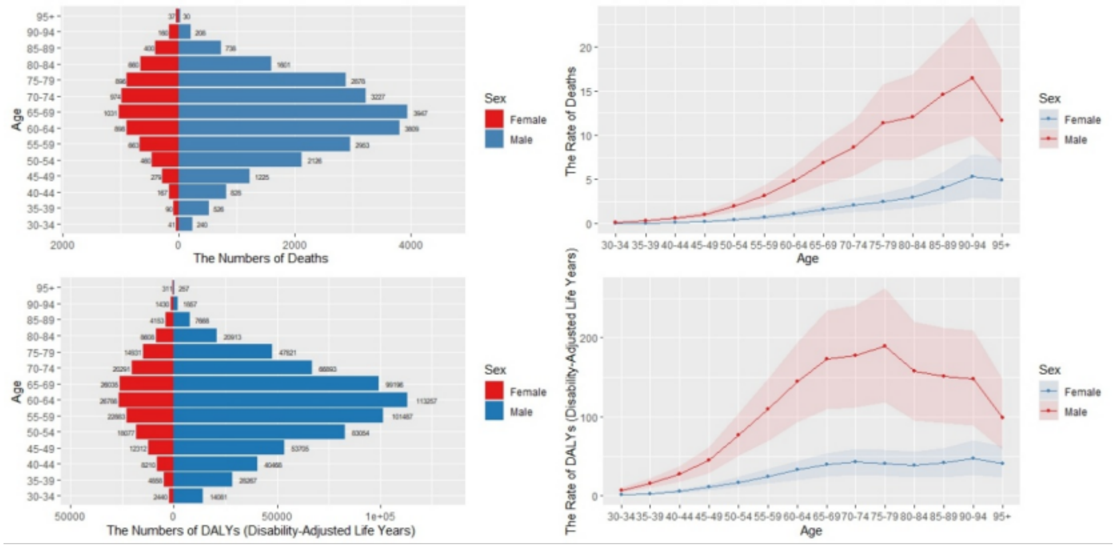

(A)

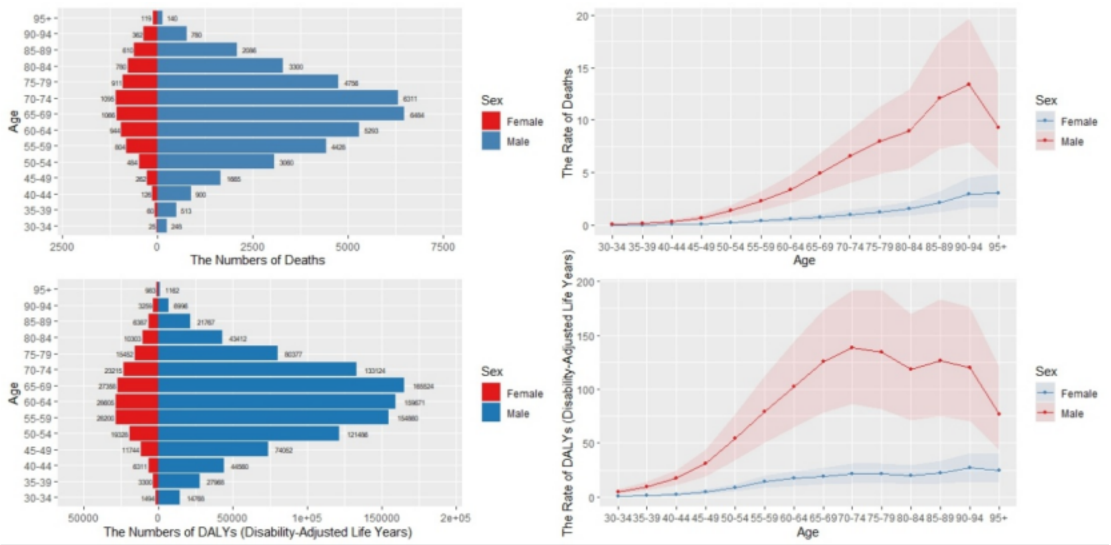

(B)

Supplement Figure 3.4. Global mortality (rate) and disability-adjusted life years (rate) attributable to tobacco use for colon and rectum cancer, by age and sex, in 1990 (A) and 2021 (B).

Shaded areas now include a description indicating that they represent the 95% uncertainty intervals (UIs) around the estimated trends or regression lines.

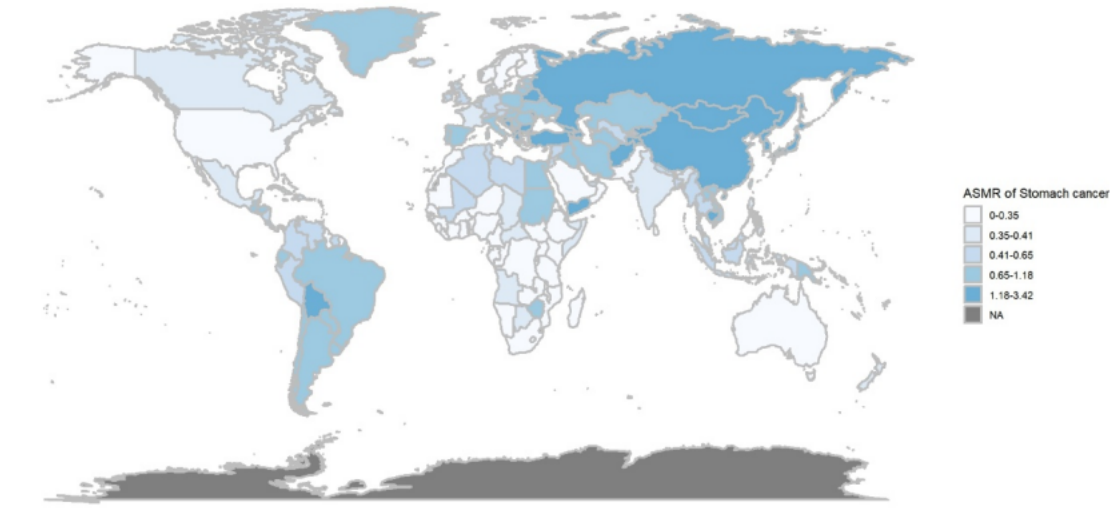

(A)

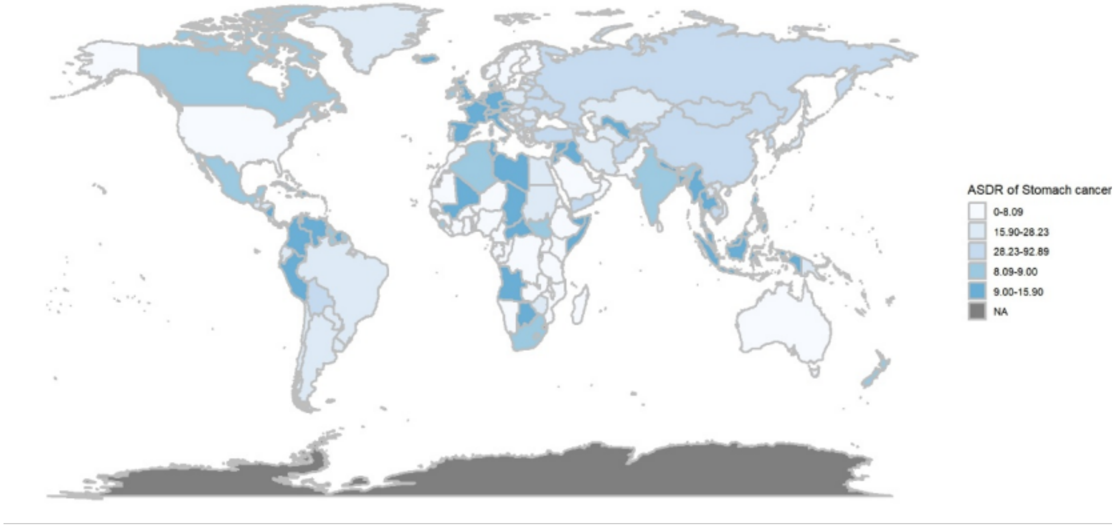

(B)

Supplement Figure 4.1. In the year 2021, global distribution of stomach cancer burden attribute to tobacco use in terms of ASMR per 100 000 (A), ASDR per 100 000 (B). ASMR, age-standardised mortality rate;ASDR, age-standardised DALYs rate; DALYs, disability-adjusted life-years, NA: none.

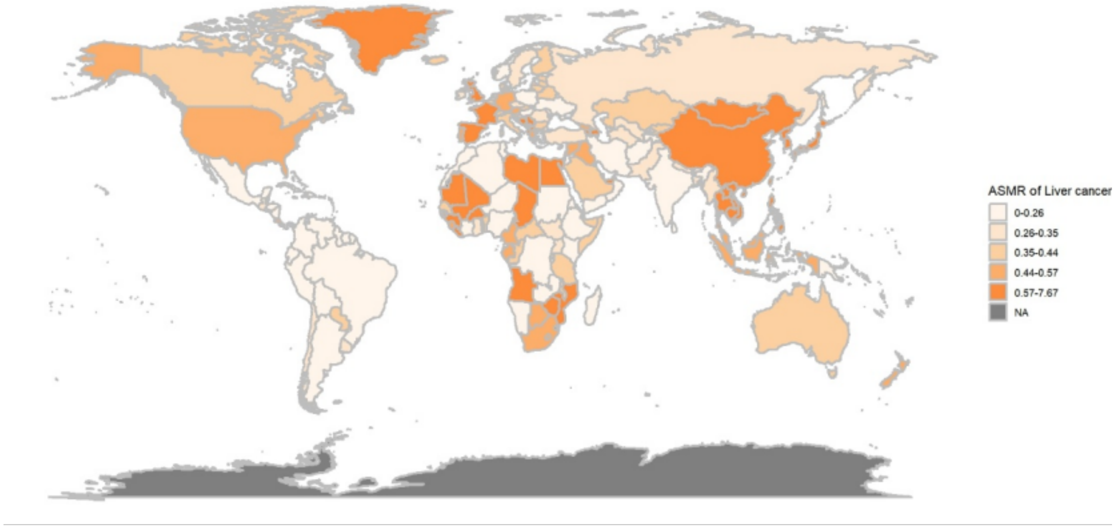

(A)

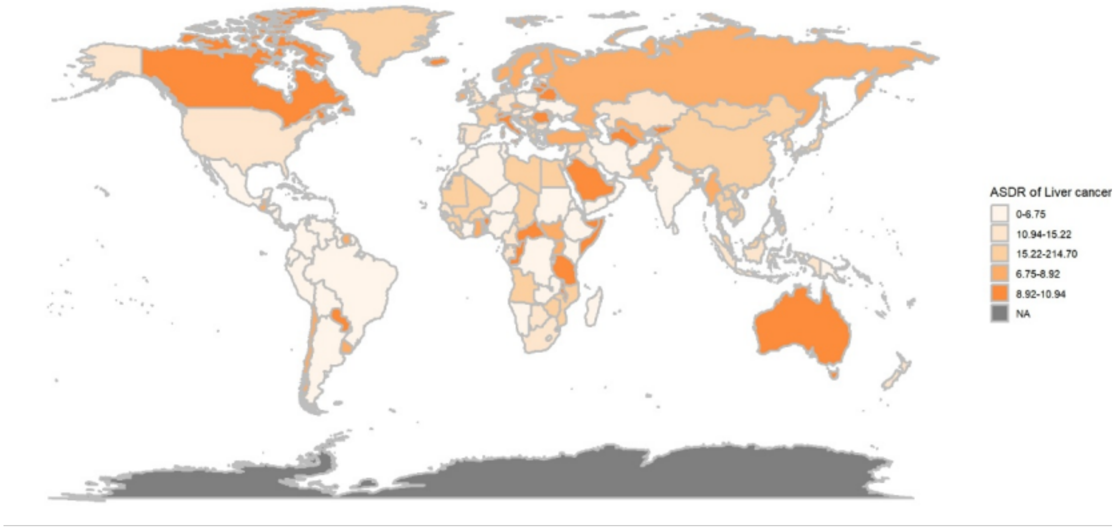

(B)

Supplement Figure 4.2. In the year 2021, global distribution of liver cancer burden attribute to tobacco use in terms of ASMR per 100 000 (A), ASDR per 100 000 (B). ASMR, age-standardised mortality rate;ASDR, age-standardised DALYs rate; DALYs, disability-adjusted life-years, NA: none.

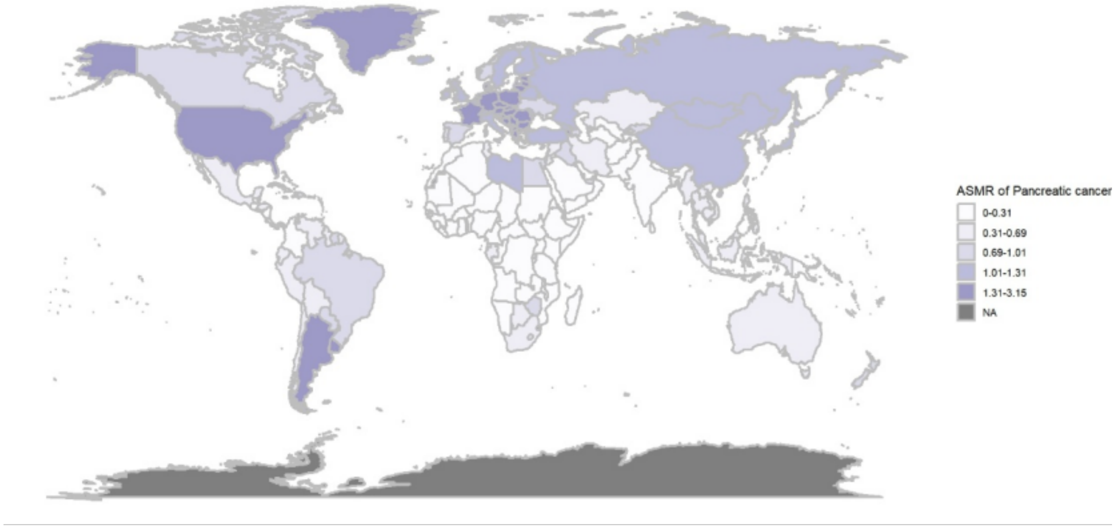

(A)

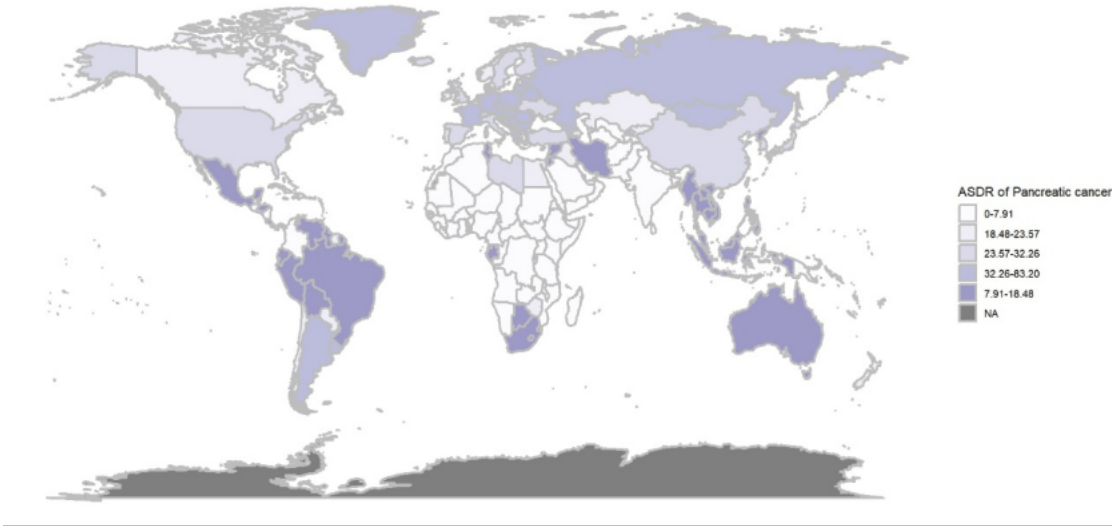

(B)

Supplement Figure 4.3. In the year 2021, global distribution of pancreatic cancer burden attribute to tobacco use in terms of ASMR per 100 000 (A), ASDR per 100 000 (B). ASMR, age-standardised mortality rate;ASDR, age-standardised DALYs rate; DALYs, disability-adjusted life-years, NA: none.

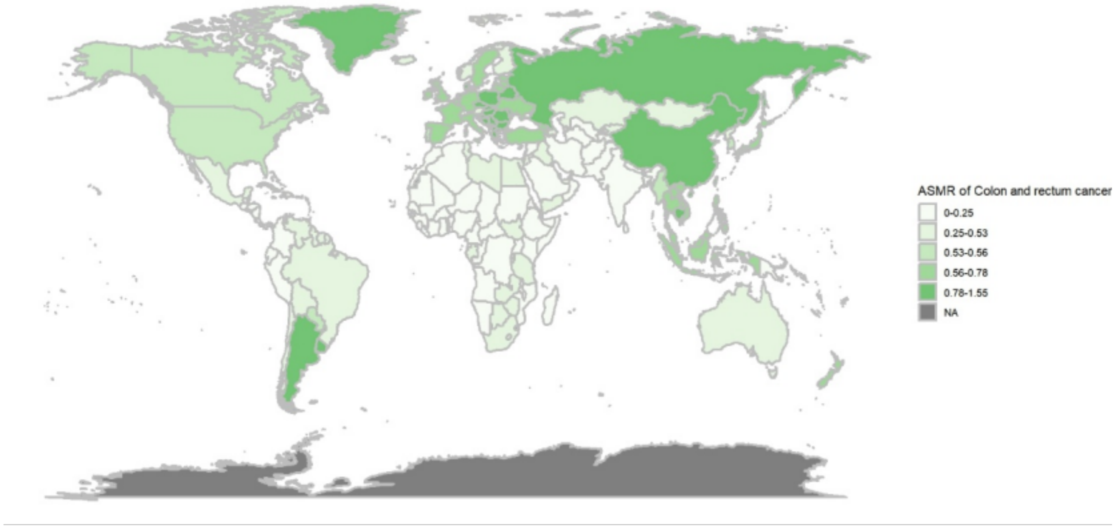

(A)

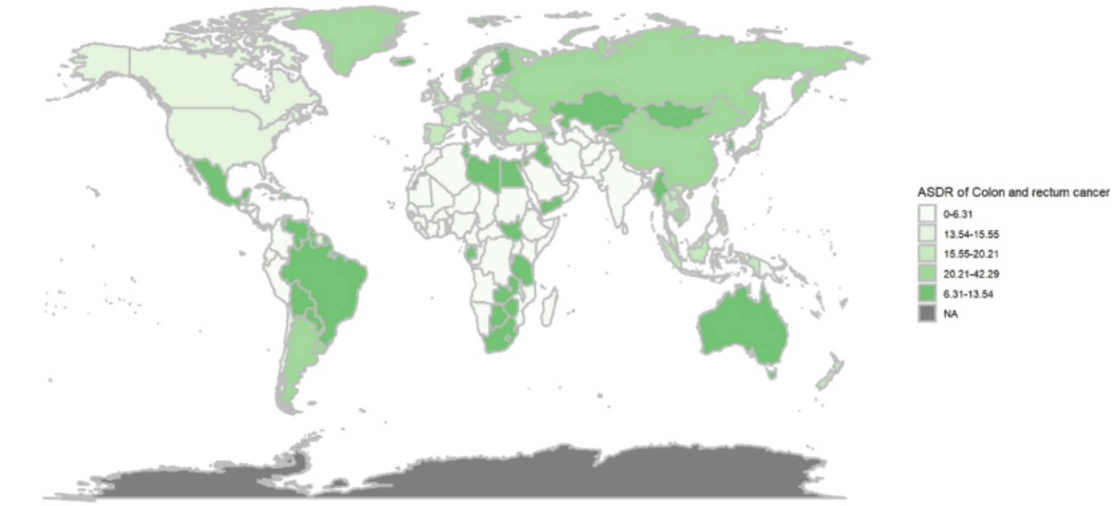

(B)

Supplement Figure 4.4. In the year 2021, global distribution of colon and rectum cancer burden attribute to tobacco use in terms of ASMR per 100 000 (A), ASDR per 100 000 (B). ASMR, age-standardised mortality rate;ASDR, age-standardised DALYs rate; DALYs, disability-adjusted life-years, NA: none.

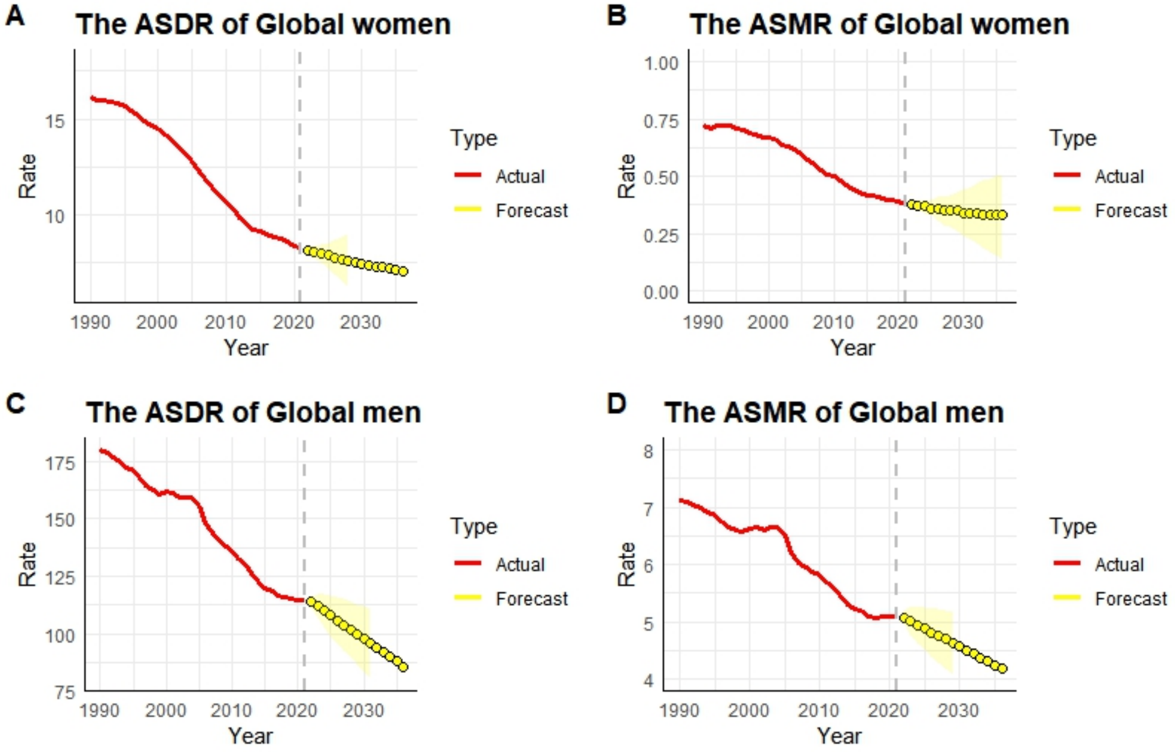

(A)

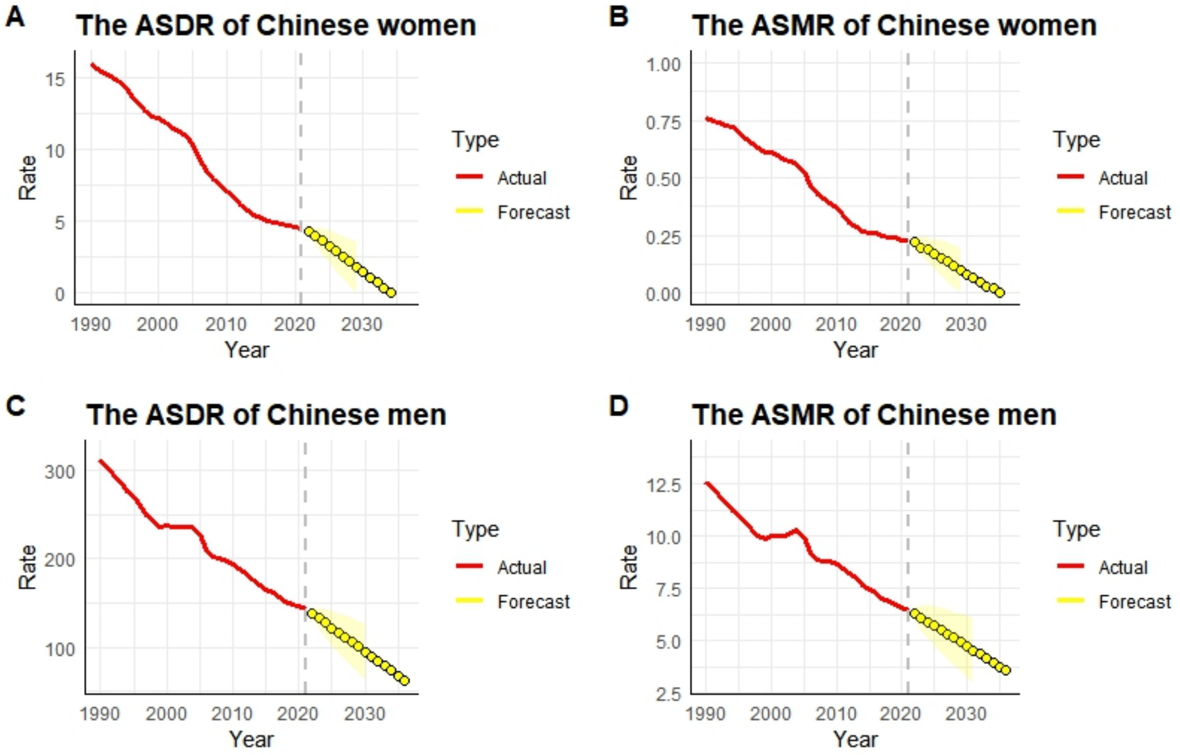

(B)

Supplement Figure 5.1. Trends in ASMR per 100 000 and per 100 000 ASDR attributable to tobacco-related stomach cancer globally (A) and in China (B) from 1990 to 2036, with projections by gender for 2022–2036. ASMR, age-standardised mortality rate;ASDR, age-standardised DALYs rate; DALYs, disability-adjusted life-years.

Shaded areas now include a description indicating that they represent the 95% uncertainty intervals (UIs) around the estimated trends or regression lines.

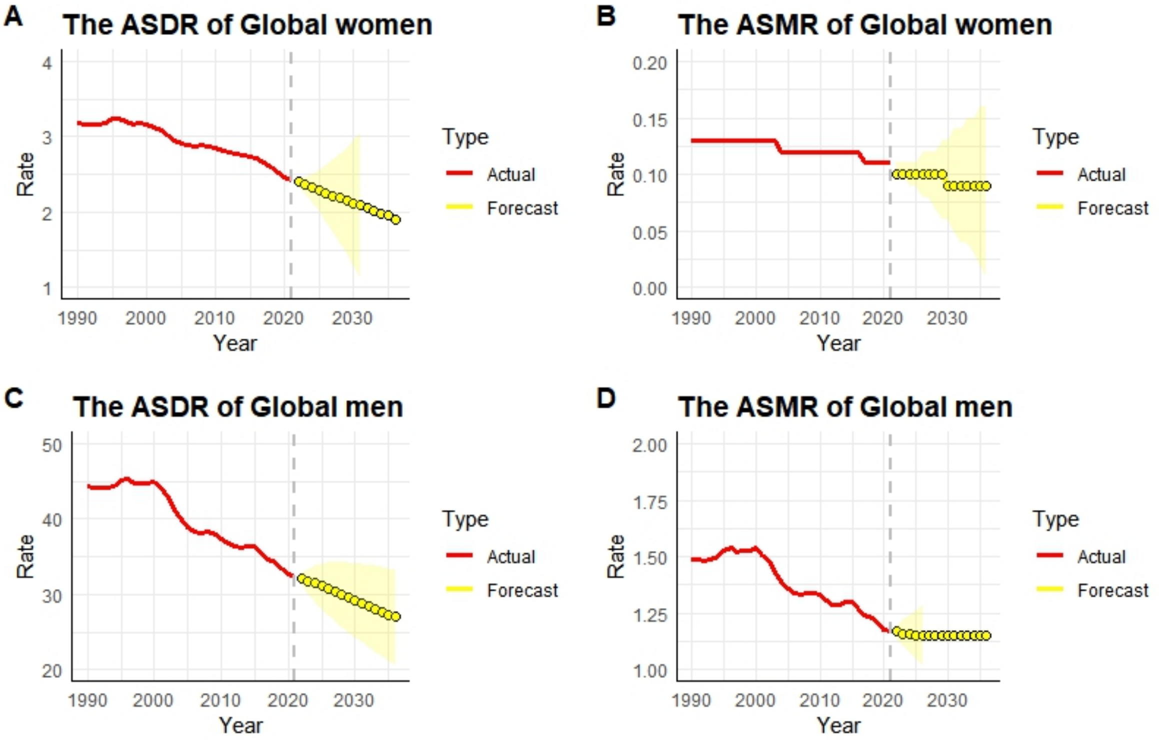
A
The ASDR of Global women
B
The ASMR of Global women
C
The ASDR of Global men
D
The ASMR of Global men
Rate
Year
1990
2000
2010
2020
2030
Type
Actual
Forecast

(A)

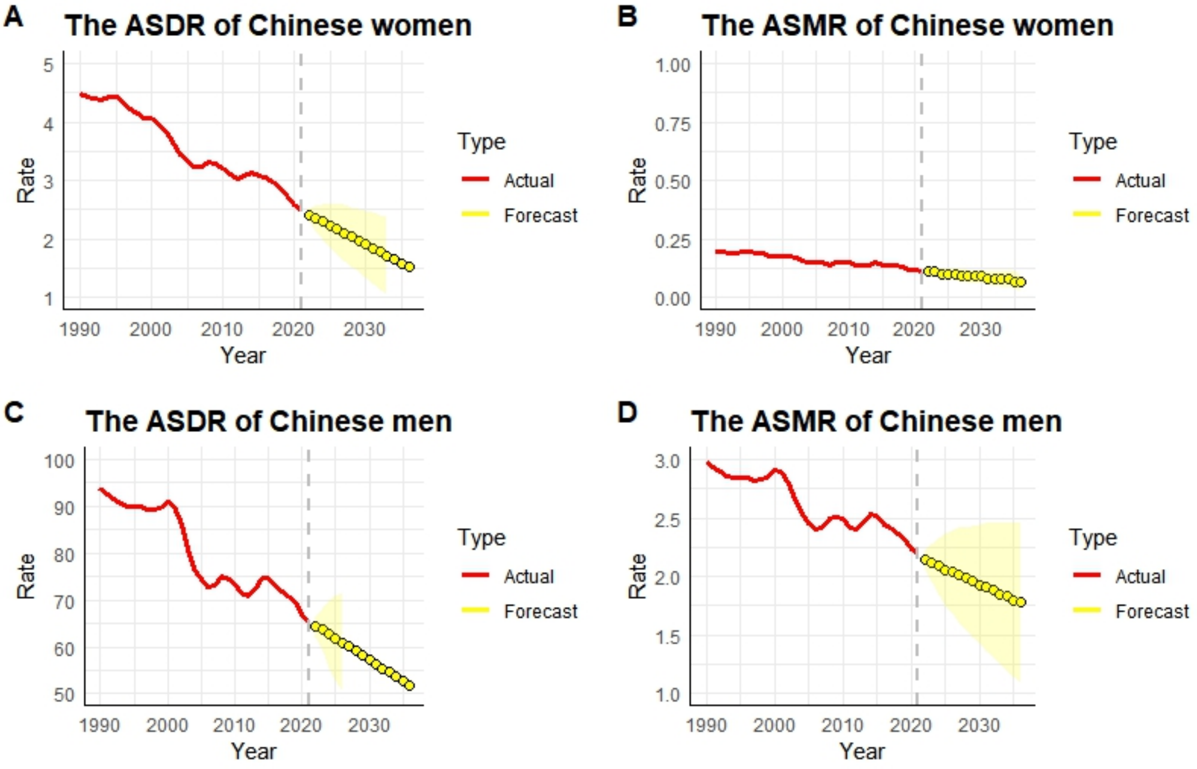
A
The ASDR of Chinese women
B
The ASMR of Chinese women
C
The ASDR of Chinese men
D
The ASMR of Chinese men
Rate
Year
1990
2000
2010
2020
2030
Type
Actual
Forecast

(B)

Supplement Figure 5.2. Trends in ASMR per 100 000 and per 100 000 ASDR attributable to tobacco-related liver cancer globally (A) and in China (B) from 1990 to 2036, with projections by gender for 2022–2036. ASMR, age-standardised mortality rate;ASDR, age-standardised DALYs rate; DALYs, disability-adjusted life-years.

Shaded areas now include a description indicating that they represent the 95% uncertainty intervals (UIs) around the estimated trends or regression lines.

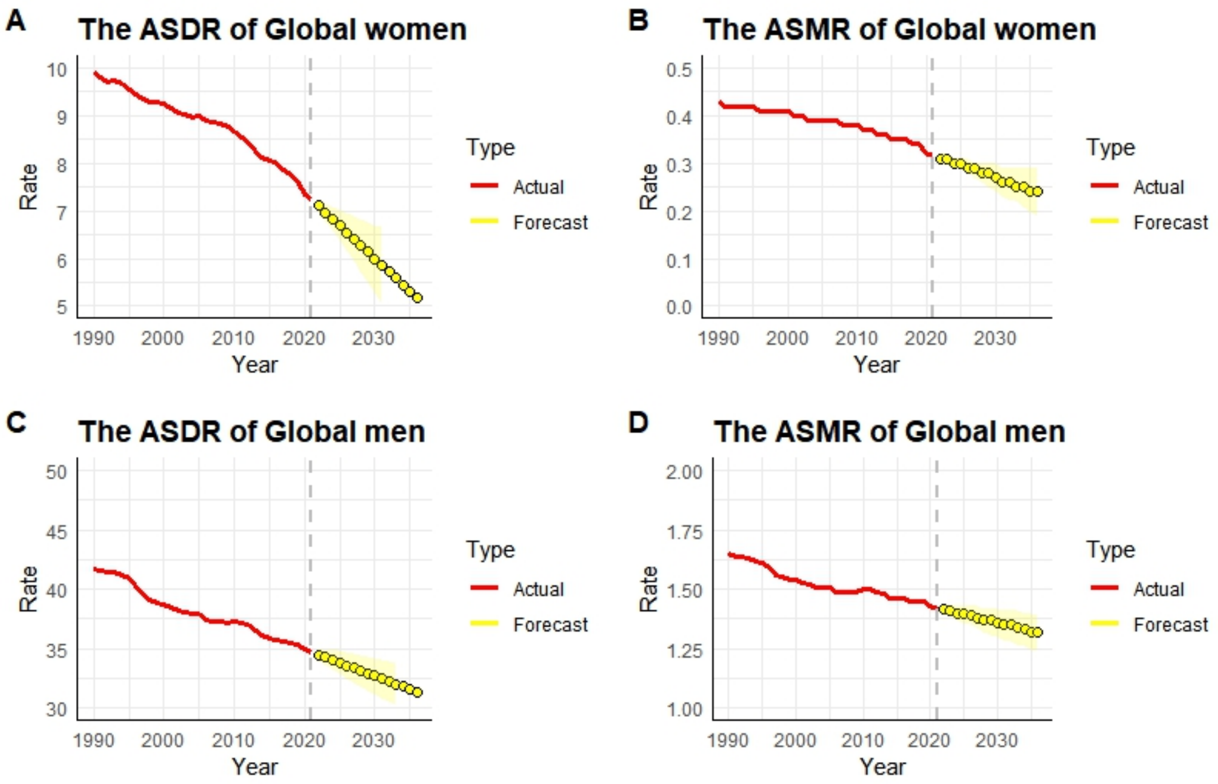

(A)

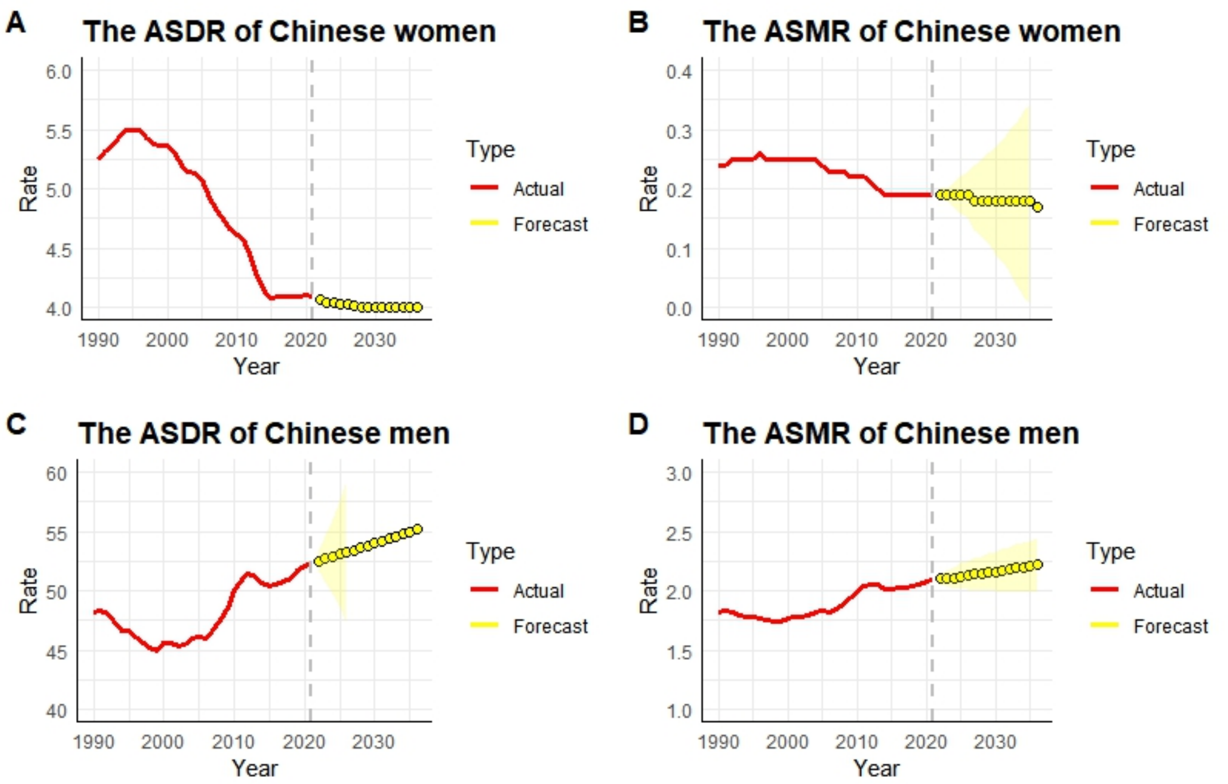

(B)

Supplement Figure 5.3. Trends in ASMR per 100 000 and per 100 000 ASDR attributable to tobacco-related pancreatic cancer globally (A) and in China (B) from 1990 to 2036, with projections by gender for 2022-2036. ASMR, age-standardised mortality rate;ASDR, age-standardised DALYs rate; DALYs, disability-adjusted life-years.

Shaded areas now include a description indicating that they represent the 95% uncertainty intervals (UIs) around the estimated trends or regression lines.

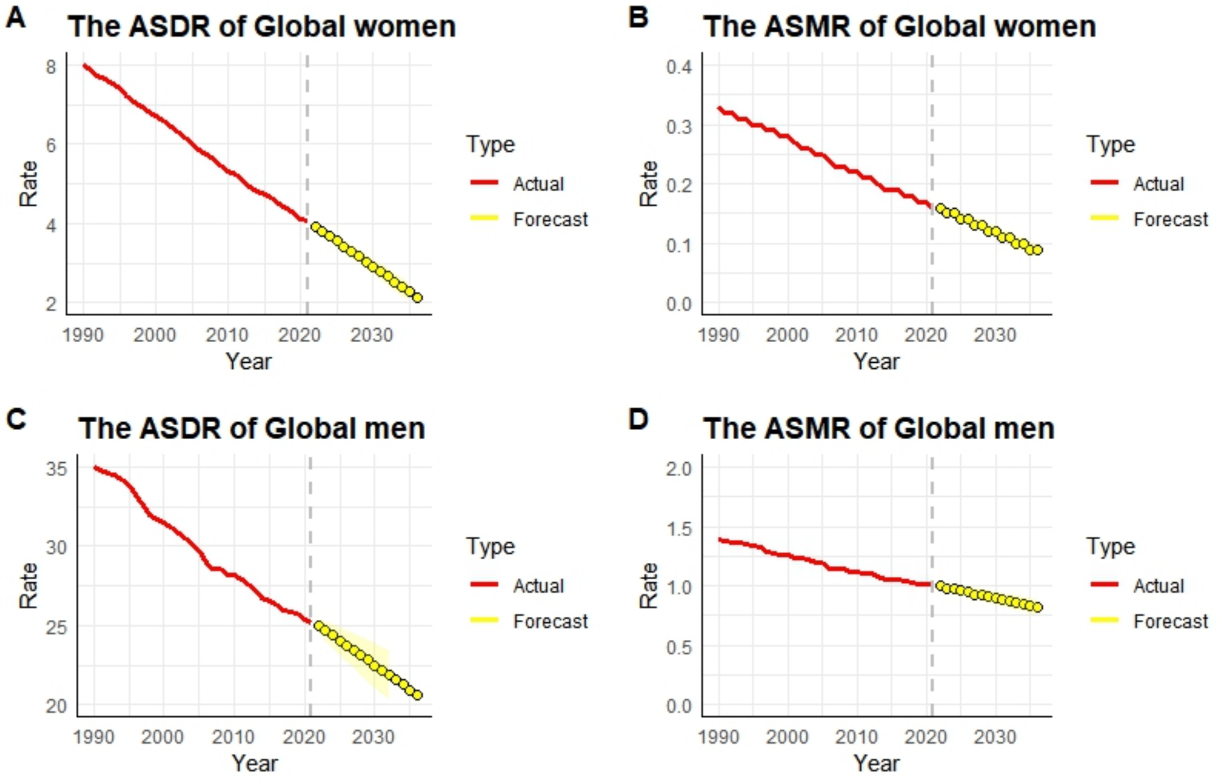
A
The ASDR of Global women
B
The ASMR of Global women
C
The ASDR of Global men
D
The ASMR of Global men
Rate
Year
Type
Actual
Forecast

(A)

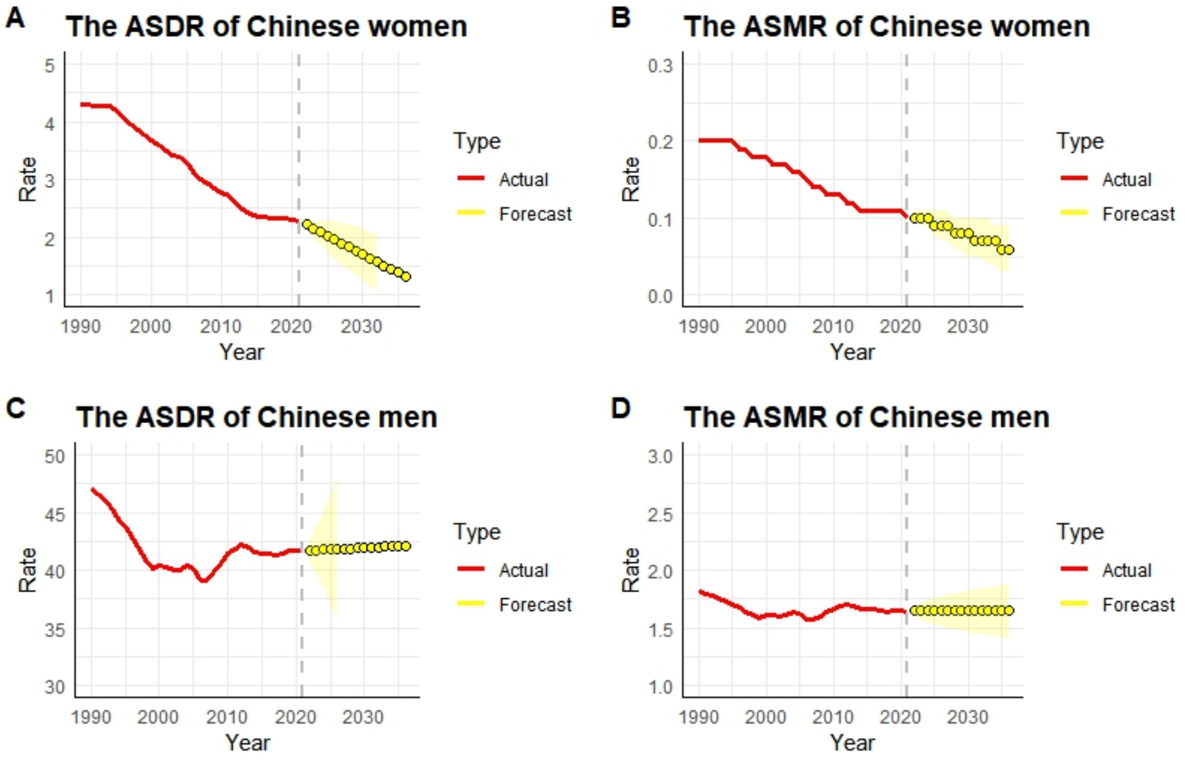
A
The ASDR of Chinese women
B
The ASMR of Chinese women
C
The ASDR of Chinese men
D
The ASMR of Chinese men
Rate
Year
Type
Actual
Forecast

(B)

Supplement Figure 5.4. Trends in ASMR per 100 000 and per 100 000 ASDR attributable to tobacco-related colon and rectum cancer globally (A) and in China (B) from 1990 to 2036, with projections by gender for 2022–2036. ASMR, age-standardised mortality rate;ASDR, age-standardised DALYs rate; DALYs, disability-adjusted life-years.

Shaded areas now include a description indicating that they represent the 95% uncertainty intervals (UIs) around the estimated trends or regression lines.

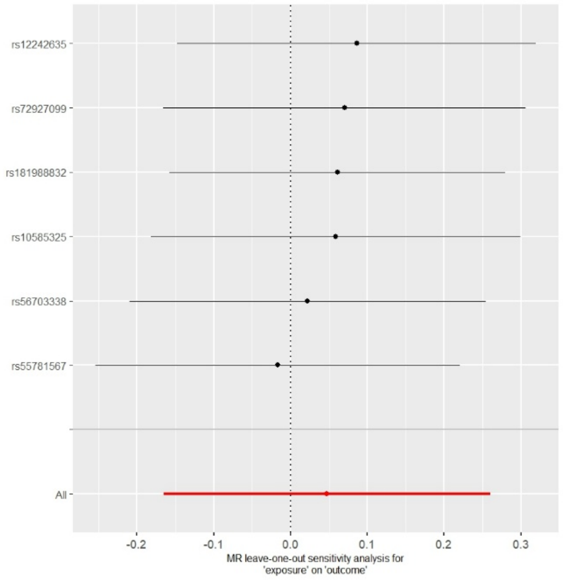

(A)

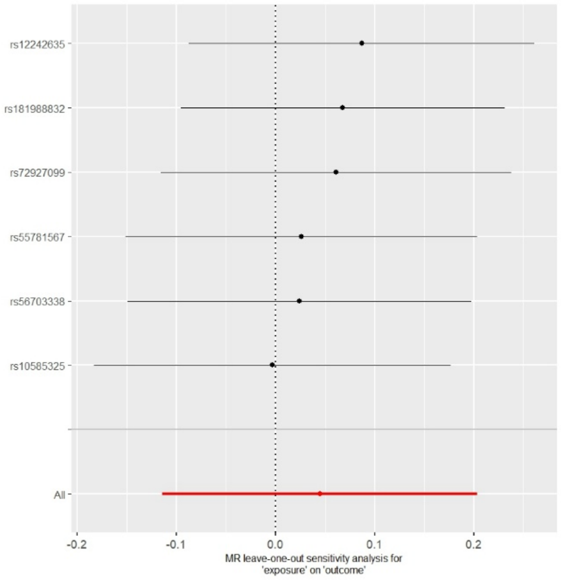

(B)

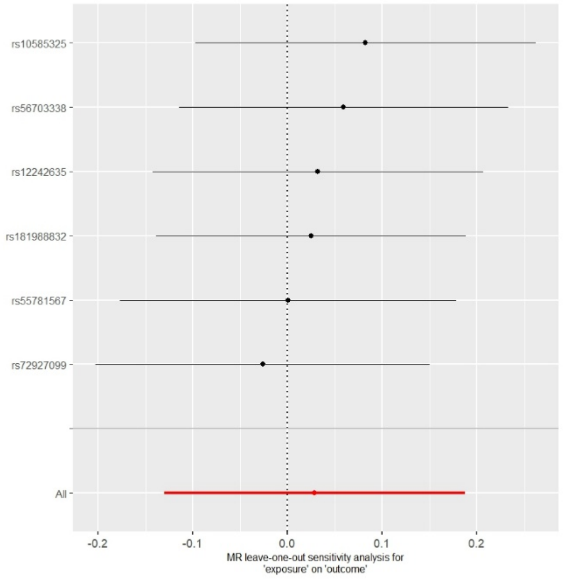

(C)

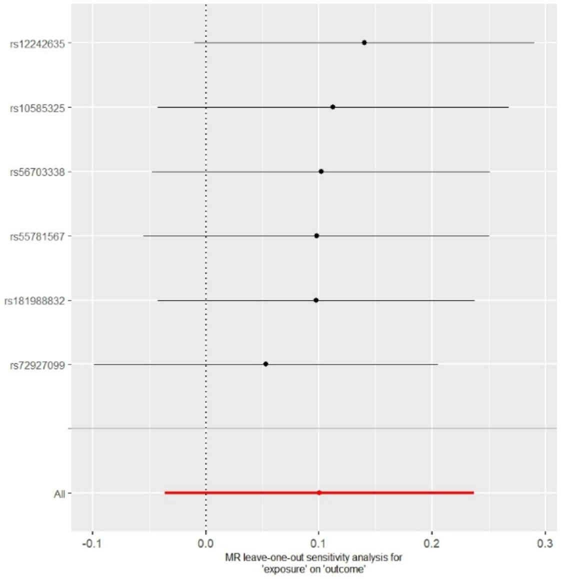

(D)

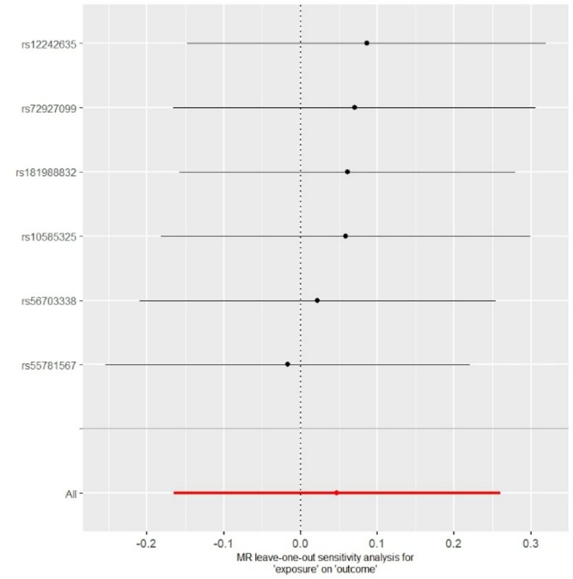

(E)

Supplement Figure 6. Leave-one-out sensitivity analysis for the association between tobacco use and common gastrointestinal cancers. A: esophageal cancer; B: gastric cancer; C: liver cancer; D: pancreatic cancer; E: colon and rectum cancer.

Error bars are clarified as representing the 95% uncertainty intervals of the corresponding point estimates.

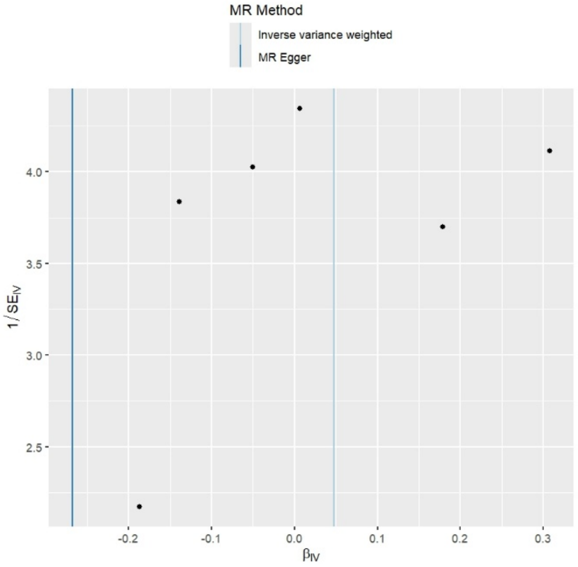

(A)

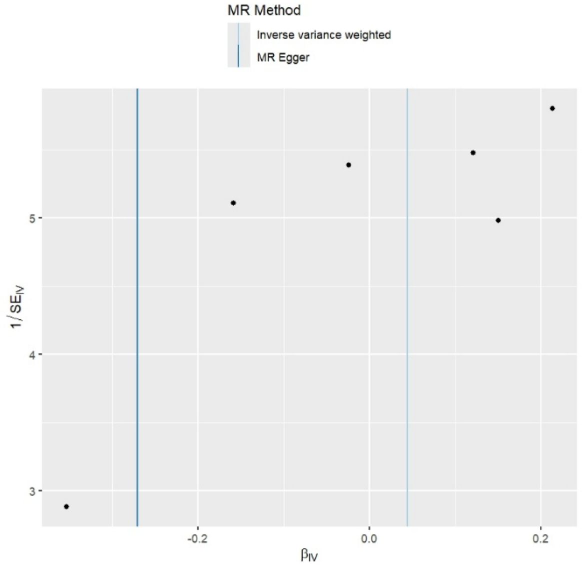

(B)

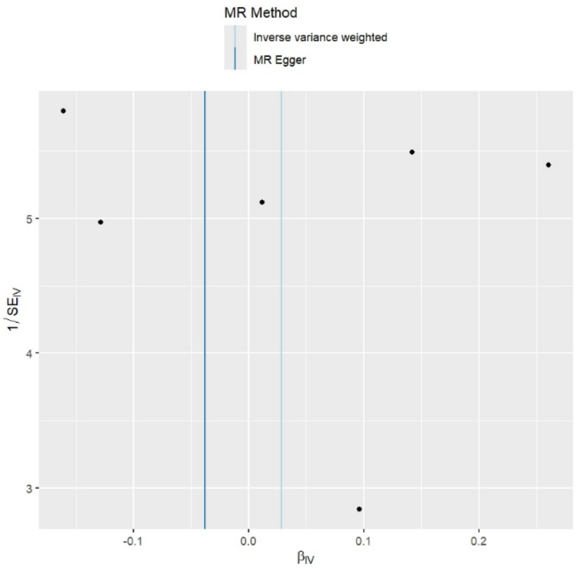

(C)

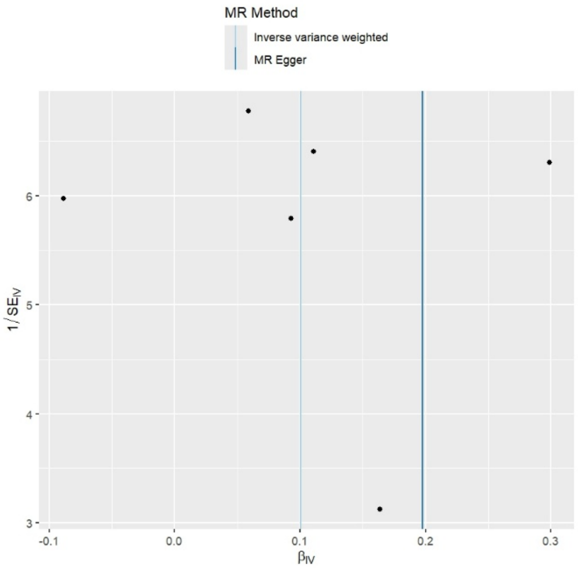

(D)

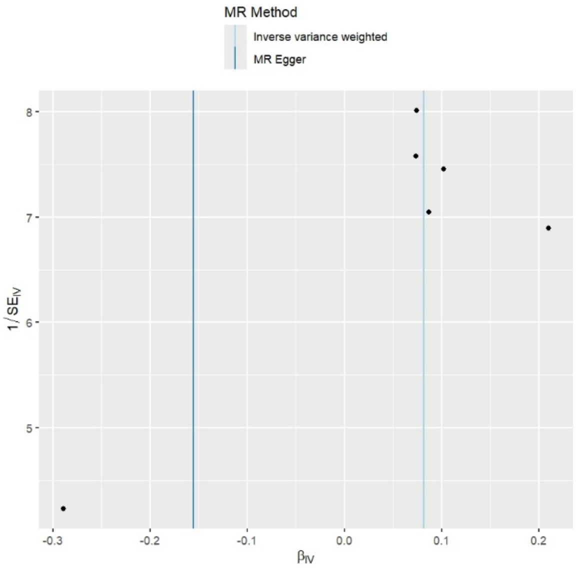

(E)

Supplement Figure 7. Scatter plot of Mendelian randomization estimates for the association between tobacco use and common gastrointestinal cancers. A: esophageal cancer; B: gastric cancer; C: liver cancer; D: pancreatic cancer; E: colon and rectum cancer. Each point represents an individual single nucleotide polymorphism(SNP), plotted according to its association with tobacco use (x-axis) and the corresponding cancer outcome (y-axis).

© **2026 Liu Y. et al.**
